# A Photoactivated Ru (II) Polypyridine Complex Induced Oncotic Necrosis of A549 Cells by Activating Oxidative Phosphorylation and Inhibiting DNA Synthesis as Revealed by Quantitative Proteomics

**DOI:** 10.3390/ijms24097756

**Published:** 2023-04-24

**Authors:** Li Zhu, Hui Liu, Yang Dou, Qun Luo, Liangzhen Gu, Xingkai Liu, Qianxiong Zhou, Juanjuan Han, Fuyi Wang

**Affiliations:** 1 College of Applied Science and Technology, Beijing Key Laboratory of Bioactive Substances and Functional Foods, Beijing Union University, Beijing 100101, China; x694200@163.com (L.Z.);; 2 Beijing National Laboratory for Molecular Sciences, CAS Research/Education Centre for Excellence in Molecular Sciences, National Centre for Mass Spectrometry in Beijing, CAS Key Laboratory of Analytical Chemistry for Living Biosystems, Institute of Chemistry, Chinese Academy of Sciences, Beijing 100190, China; 3 University of Chinese Academy of Sciences, Beijing 100049, China; 4 Laboratory of Photochemical Conversion and Optoelectronic Materials, Technical Institute of Physics and Chemistry, Chinese Academy of Sciences, Beijing 100190, China; 5 College of Traditional Chinese Medicine, Shandong University of Traditional Chinese Medicine, Jinan 250355, China

**Keywords:** DNA damage, oncotic necrosis, photoactivated chemotherapy, quantitative proteomics, reactive oxygen species, ruthenium polypyridine complex

## Abstract

The ruthenium polypyridine complex [Ru(dppa)_2_(pytp)] (PF_6_)_2_ (termed as ZQX-1), where dppa = 4,7-diphenyl-1,10-phenanthroline and pytp = 4′-pyrene-2,2′:6′,2′′-terpyridine, has been shown a high and selective cytotoxicity to hypoxic and cisplatin-resistant cancer cells either under irradiation with blue light or upon two-photon excitation. The IC_50_ values of ZQX-1 towards A549 cancer cells and HEK293 health cells are 0.16 ± 0.09 µM and >100 µM under irradiation at 420 nm, respectively. However, the mechanism of action of ZQX-1 remains unclear. In this work, using the quantitative proteomics method we identified 84 differentially expressed proteins (DEPs) with |fold-change| ≥ 1.2 in A549 cancer cells exposed to ZQX-1 under irradiation at 420 nm. Bioinformatics analysis of the DEPs revealed that photoactivated ZQX-1 generated reactive oxygen species (ROS) to activate oxidative phosphorylation signaling to overproduce ATP; it also released ROS and pyrene derivative to damage DNA and arrest A549 cells at S-phase, which synergistically led to oncotic necrosis and apoptosis of A549 cells to deplete excess ATP, evidenced by the elevated level of PRAP1 and cleaved capase-3. Moreover, the DNA damage inhibited the expression of DNA repair-related proteins, such as RBX1 and GPS1, enhancing photocytotoxicity of ZQX-1, which was reflected in the inhibition of integrin signaling and disruption of ribosome assembly. Importantly, the photoactivated ZQX-1 was shown to activate hypoxia-inducible factor 1A (HIF1A) survival signaling, implying that combining use of ZQX-1 with HIF1A signaling inhibitors may further promote the photocytotoxicity of the prodrug.

## 1. Introduction

Cancer is one of the major diseases endangering human health. Chemotherapy is one of the common methods for cancer treatment. Since Rosenberg discovered the unique cytotoxicity of cisplatin in the late-1960s [1], a number of platinum complexes, including cisplatin, carboplatin and oxaliplatin, have achieved great success in independent or combined use in clinic for treatment of solid tumors [2,3]. However, the severe side effects caused by the poor selectivity of platinum drugs and the innate or/and acquired drug resistance of tumor cells largely limit their further clinical application [4,5,6].

In recent decades, phototherapy, including photodynamic therapy (PDT) and photoactivated chemotherapy (PACT), has become a greatly attractive field to circumvent the side effects of and resistance towards cytotoxic antitumor drugs such as cisplatin. To implement PDT, the photoactivatable precursors, also named prodrugs, undergo decomposition under light irradiation and in the presence of oxygen to produce reactive oxygen species (ROS), such as oxygen radicals and singlet oxygen [7]. The generated ROS are highly active and can induce severe oxidative damage to biological molecules, such as DNA and proteins, leading to cell apoptosis and cell death [7,8,9]. Because tumor cells often grow in a hypoxic microenvironment, the generation of ROS by PDT agents, which relies on the presence of oxygen, is largely limited. To this end, PACT has been developed. PACT prodrugs can be activated under light irradiation to produce cytotoxic species, including ROS, even in the hypoxic microenvironment of cancer cells, exerting anticancer pharmacological functions. As the photo-decomposition of both PDT and PACT prodrugs can be spatiotemporally controlled in a fine manner in the designated location of tumor tissues, the activated drugs show highly selective pharmaco-dynamic activity with fewer side effects.

**Figure 1:**
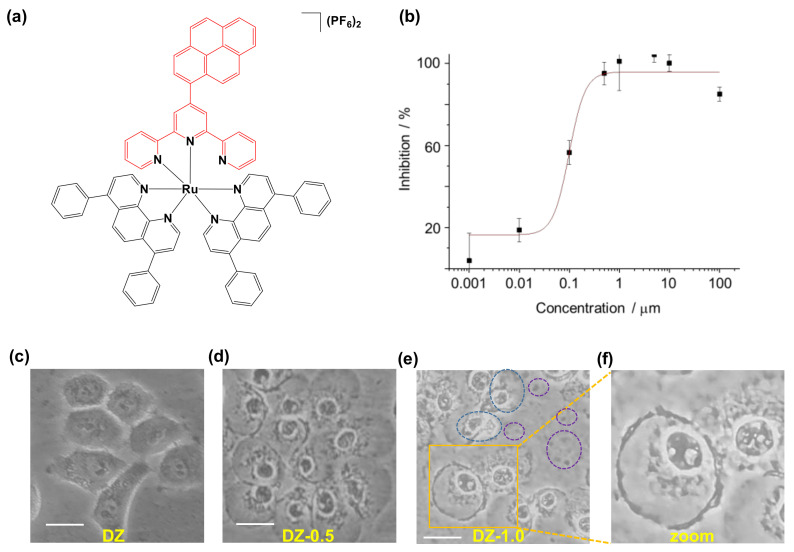
(**a**) Chemical structure of ruthenium polypyridine complex ZQX-1. (**b**) Inhibition of ZQX-1 on growth of A549 cells incubated with various concentrations of ZQX-1 in dark for 4 h, followed by irradiation at 420 nm for 20 min and further incubation in dark for 0.5 h. Inhibition rates (%) are present as mean ± SD, n = 6. (**c**–**f**) Bright-field images of A549 cells incubated with ZQX-1 (0.5 μM) for 4.5 h at 37 °C in dark (DZ) or for 4 h at 37 °C in dark and then irradiated at 420 nm for 20 min, followed by further 0.5 h (DZ-0.5) or 1.0 h (DZ-1.0) of incubation in dark. Blue circle in (**e**) refers to the damaged cells, while purple circle refers to cell debris generated from death cells. Scale bar: 20 μm. (**f**) Enlarged view of image in orange box in (**f**).

Due to their unique photochemical and photophysical properties, transition metal complexes as a class of photoactivatable prodrugs have gained increasing attention for the design and development of both PDT and PACT agents [8,9,10,11,12,13]. In order to conquer the limitation that many metallic prodrugs can only be activated under visible lights with poor ability of tissue penetration, various chemical modifications via the organic ligands coordinating to metal centers, such as Pt, Ir and Ru, have been applied, enabling the prodrugs activatable under near infrared light (NIR) or two-photon excitation [8,14,15,16,17,18]. An excellent example is the ruthenium(II) polypyridine complex [Ru(dppa)_2_(pytp)](PF_6_)_2_ (ZQX-1) (Figure 1a), where dppa = 4,7-diphenyl-1,10-phenanthroline, pytp = 4′-pyrene-2,2′:6′,2′′- terpyridine [9]. In the structure of ZQX-1, the pyrene modified tridentate terpyridine binds to Ru(II) as a bidentate ligand, weakening the ligand field to make the pytpy ligand labile upon irradiation with blue light. On the other hand, the pyrene unit acts as an effective two-photon absorption antenna, allowing ZQX-1 activatable upon two-photon excitation at 740 nm [9]. The photoactivation under both blue and NIR lights triggered not only generation of ROS, but also release of cytotoxic pyrene derivative (pytp) which could also serve as “switch-on” fluorescence reporter for tracking the cellular uptake and visualizing the subcellular distribution of the prodrug [9]. Zhou and co-workers further demonstrated that upon irradiation at 470 nm, ZQX-1 exhibited high cytotoxicity towards human lung cancer cells A549 and cisplatin-resistant A549 cells under both normoxic and hypoxic conditions with IC_50_ values at sub-micromolar level and a phototoxicity index (PI) higher than 70 [9]. However, the molecular mechanism underlying the action of ZQX-1 remains unknown.

To address this issue, in this work, with combining use of bioinformatics analysis, we applied mass spectrometry-based quantitative proteomics to decipher the molecular mechanism of action for the ruthenium polypyridine complex, ZQX-1. Quantitative proteomics based on mass spectrometry is one of the most powerful strategies to study the molecular mechanism of action of drugs [19,20,21,22,23]. The alternation in expression level of proteins in cells exposed to drugs can precisely reflect the pharmacological effects of the drugs. Combined with bioinformatic analysis, quantitatively analyzing of the expression level of the whole proteome in cells or tissues allows the screening out target proteins of the drugs, elucidating the molecular mechanism of action of the drugs [23,24,25,26]. Significantly, quantitatively profiling protein expression in human A549 non-small cell lung cancer cells exposed to ZQX-1 under irradiation at 420 nm, we identified 84 differentially expressed proteins (DEPs) with a fold-change (FC) of ≥1.2 or ≤−1.2 compared those in A549 cells exposed to the same concentration of ZQX-1 without irradiation. The bioinformatic analysis suggested that these DEPs are highly associated with EIF2 signaling, oxidative phosphorylation signaling, hypoxia-inducible factor 1A (HIF1A) survival signaling, tricarboxylic acid cycle (TCA) signaling and integrin signaling pathways. Combined with cellular phenotype assays, we revealed that the photoactivated ZQX-1 induced cell oncotic necrosis through activating oxidative phosphorylation signaling, accompanied by inhibition of integrin signaling and disruption of the assembly of ribosomes via damaging DNA.

## 2. Results and Discussion

### 2.1. Cellular Phenotype Characterization

ZQX-1 was previously reported to be much more cytotoxic towards A549 and human cervical cancer cells (HeLa) under either irradiation at 470 nm or two-photon excitation at 740 nm than in the dark. The photocytotoxicity indexes (PIs) of ZQX-1 towards A549 and HeLa cells under normoxic conditions were 76 and 49, respectively [9]. To evaluate the selectivity of ZQX-1 towards cancer cells and healthy cells, we first measured the half inhibition concentration (IC50) of ZQX-1 towards A549 cancer cells and human embryonic kidney (HEK293T) cells. The results (Figure 1b and Figure S1a in the Supplementary Materials) showed that upon irradiation for 20 min at 420 nm followed by further 0.5 h of incubation in the dark, the IC_50_ value of ZQX-1 towards A549 cells was 0.16 ± 0.09 μM. In contrast, the viability of HEK293T cells treated with ZQX-1 under the same conditions was almost unchanged over the large concentration range from 0.001 to 100 μM (Figure S1a). This indicated that ZQX-1 has little photocytotoxicity towards the healthy cells, being highly selective to cancer cells.

To verify whether the cellular uptake of ZQX-1 dominates its photocytotoxicity as well as its selectivity towards various cells, we applied inductively coupled plasma mass spectrometry (ICP-MS) to determine the concentration of ruthenium inside cells exposed to ZQX-1 in the dark. Notably, in order to precisely determine the cellular concentration of ZQX-1, we used a high dose (5 μM) of the complex to treat the cells in the dark, where ZQX-1 entered inside cells but had little effect on the growth of the cells without irradiation. As shown in Figure S1b, Ru concentrations in A549, HeLa and HEK293T cells were 27.3 ± 4.7, 17.5 ± 5.5 and 13.0 ± 4.8 pg/10^6^ cell, respectively. Indeed, the cellular uptake of ZQX-1 in three cell lines were different from each another. However, only the uptakes of ZQX-1 in A549 and HEK293T cells showed statistically significant difference (*p* < 0.05). Given the significantly different cytotoxicity of ZQX-1 towards A549 (IC_50_ = 0.16 ± 0.09 μM, Figure 1b) and HeLa (IC_50_ = 0.74 ± 0.06 μM, measured under irradiation at 470 nm [9]) and HEK293T (IC_50_ > 100 μM, Figure S1a), the cellular uptake level of ZQX-1 determined its photocytotoxicity, though may not be the only factor.

Next, we investigated the effects of ZQX-1 under irradiation on the morphology of A549 cells. Here, to maintain enough cells for phenotypic characterization, we used 0.5 μM (~3 × IC_50_) ZQX-1 to treat A549 cells. It was clearly shown that the morphology of cells treated with ZQX-1 upon irradiation (Figure 1d) was changed significantly compared with cells without irradiation (Figure 1c). The cells without irradiation showed normal irregular edges and the surrounding area of the cells was clean without optically visible particles (Figure 1c). In contrast, the cells with irradiation were swollen and reshaped into a round shape, accompanied with nucleus shrinking (Figure 1d). With a longer incubation (1.0 h) after irradiation, a lot of “buddings” appeared inside the cells and the cytoplasm condensed around the nucleus. Moreover, large populations of cells in the light-irradiated group suffered from oncotic necrosis, reflected by spilling out of cellular contents and appearance of cell debris generated from death cells [27,28,29] (Figure 1e,f).

**Figure 2:**
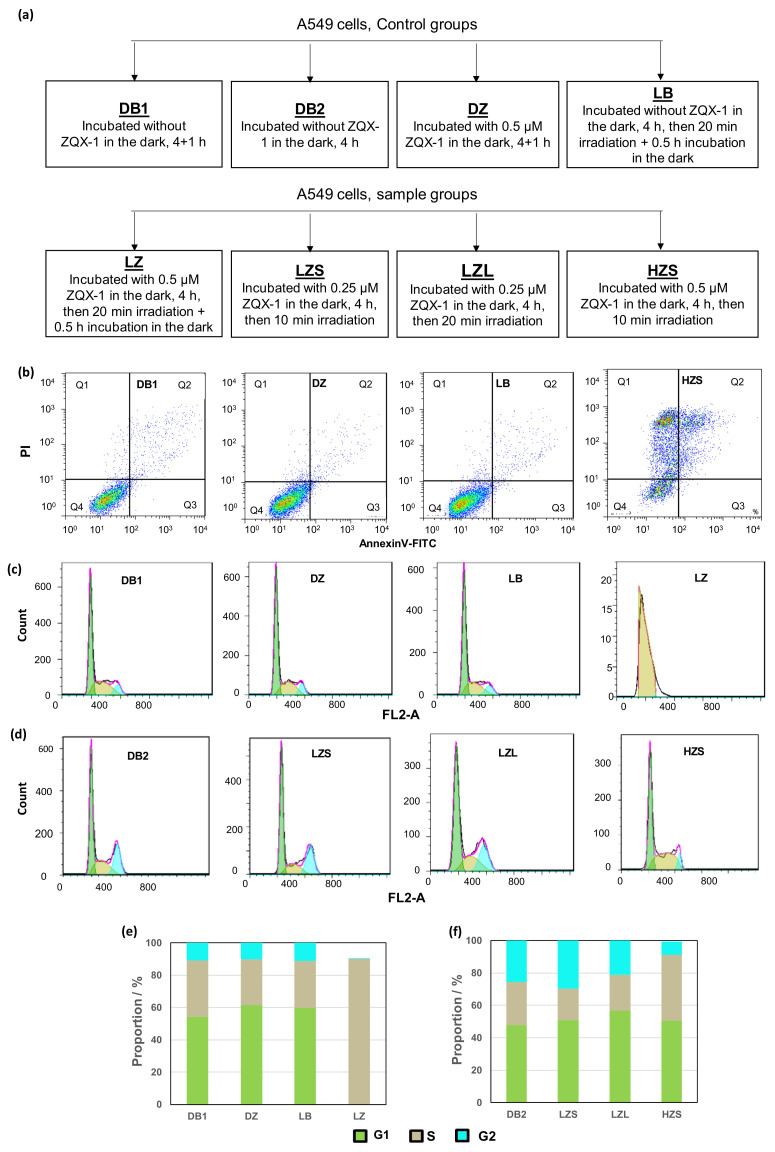
(**a**) Schematic description for designation of control and sample groups in this work. (**b**) Apoptosis assay of A549 cells treated with ZQX-1 under different conditions. (**c**,**d**) Cell cycle profiles and (**e**,**f**) histograms of population distribution in different cell cycles of A549 cells exposed to ZQX-1 under different conditions.

Cell death induced by anticancer drugs mainly occurs in two pathways: apoptosis and necrosis. We then investigated the death pathways of A549 cells exposed to ZQX-1 upon irradiation by performing flow cytometry on A549 cells treated under different conditions. In control groups (Figure 2a), A549 cells were grown without (DB1) or with (DZ) 0.5 µM ZQX-1 in the dark for 5 h. The cells in DB2 and LB groups were grown without ZQX-1 in the dark for 4 h (DB2) and irradiated for 20 min under 420 nm followed by further 0.5 h of incubation in the dark (LB). In the sample groups, A549 cells were cultured with 0.5 µM (LZ and HZS) or 0.25 µM (LZS and LZL) ZQX-1 in the dark for 4 h and irradiated at 420 nm for 20 min (LZ and LZL) or 10 min (LZS and HZS), followed by 0.5 h (LZ) or 0 min (LZS, LZL and HZS) further incubation. The results showed that the majority (>88%) of cells were alive in DB1, DZ and LB groups, indicating again that ZQX-1 in the dark are non-cytotoxic towards A549 cells (Figure 2b). In contrast, upon irradiation for only 10 min at 420 nm, ZQX-1 induced a large proportion of cells to late apoptosis (25.6%) and necrosis (52.3%) (Figure 2b). It was of note that under irradiation at 470 nm, ZQX-1 was previously shown to induce early apoptosis of A549 cells [9]. This inconsistency may be attributed to the difference in wavelengths of lights used to activate ZQX-1 in the previous (470 nm) and present works (420 nm). The lights at different wavelengths provide different energy, perhaps resulting in changes in the rate, yield and production species of the photodecomposition of ZQX-1, which in turn alter the pharmacological effects of the complex.

To find out how ZQX-1 induced rapid death of cancer cells via necrosis and late apoptosis, we characterized the impact of ZQX-1 under irradiation on cell cycle process of A549 cells. We first profiled the cell cycle of four groups of A549 cells treated with ZQX-1 under different conditions. The results indicated that the population distribution of A549 cells in various cell cycle phases in DZ group had no significant difference from that of A549 cells in DB1 group, suggesting that ZQX-1 had no impact on the cell cycle process in the dark (Figure 2c). In contrast, the population distribution of A549 cells in various cell cycle phases in LZ group treated with QZX-1 under irradiation was significantly changed compared to that of cells in LB group without ZQX-1 treatment but with irradiation (Figure 2c). The majority (90.23%) of cells in LZ group were arrested at S-phase; for comparison, the corresponding value in LB group was 29.22% (Figure 2e). Moreover, the cell number collected from LZ group for flow cytometry assay was much less than those collected from other three groups, being only ca. one-twentieth amount of the cells collected from DB1, LB and DZ groups. One of possible reasons is that the photoactivated ZQX-1 had the majority of A549 cells arrested at S-phase, leading to the deaths of a large proportion of cells via oncotic necrosis (Figure 1c–f); thus, much fewer living cells could be collected from the LZ group for flow cytometry analysis.

To further verify the impact of ZQX-1 on the cell cycle process of A549 cells under irradiation, we also characterized the cell cycle profiles of A549 cells treated with different concentrations of ZQX-1 under various times of irradiation. To reduce cell deaths caused by ZQX-1 upon irradiation (*vide supra*), we collected cells immediately after requested times of irradiation for flow cytometry analysis. For LZS and LZL groups, the cells were incubated for 4 h with 0.25 μM of ZQX-1 in the dark and then irradiated for 10 min and 20 min, respectively, at 420 nm, followed by immediate collection of cells for flow cytometer analysis. According to the results shown in Figure 2d,f, the population distribution of A549 cells in LZL group at S phase increased from 19.45% to 22.20%, while at G2 phase it decreased from 29.58% to 21.88%, compared to the cells in LZS group. However, following treatment with 0.5 μM ZQX-1 upon 10 min irradiation (HZS group), the population distribution of A549 cells at S phase increased to 40.71%; this trend was accompanied by the remarkably decrease in distribution of cells at G2 phase to 7.99% (Figure 2f). These results indicate that lower dosage of ZQX-1 upon irradiation had no significant effect on the cell cycle progress of A549 cells, while higher dosage of ZQX-1 even with short time of irradiation could arrest a large proportion of cells at S phase. These results are in consistent with the ICP-MS results (Figure S1b), implying that the concentration of ZQX-1 inside the cells played a crucial role in the photocytotoxicity of ZQX-1, while the irradiation time and the post-irradiation time are also essential factors to maintain high photocytotoxicity of ZQX-1. It is well-known that the main biological process of cells at S phase is DNA synthesis. With regard to this, our results herein demonstrate that it was the inhibition of DNA synthesis, most probably caused by DNA damage by ROS and pyrene derivative generated from photocomposition of ZQX-1 [9], that arrested cells at S phase and prevented cells from achieving mitosis, ultimately leading to cell death (*vide infra*).

**Figure 3:**
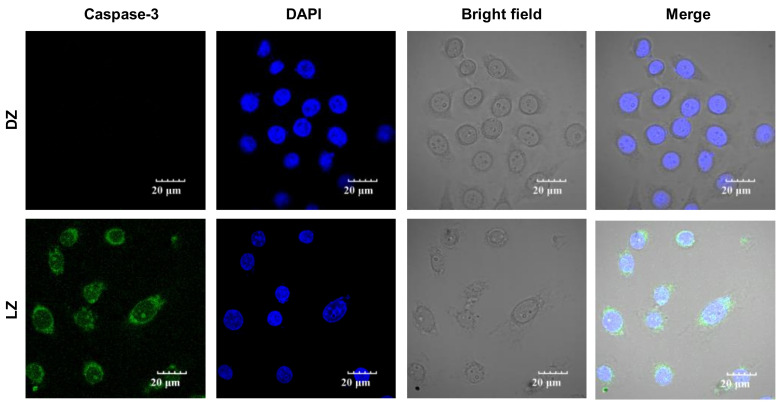
Immunofluorescence confocal images of A549 cells grown under different condition, demonstrating apoptosis and necrosis of A549 cells subject to ZQX-1 under irradiation (LZ group) by elevated level of cleaved caspase-3, compared with control group (DZ). DZ: cells were incubated with 0.5 μm ZQX-1 in the dark for 4.5 h; LZ: cells were incubated with 0.5 μM ZQX-1 in the dark for 4.0 h and refreshed and irradiated at 420 nm for 20 min, followed by further 0.5 h of incubation in the dark.

The elevation of cleaved caspase-3 level by protease is shown to be a marker for apoptosis and cell death [29,30]. To verify the ZQX-1-induced cell death of A549 cells, we performed immunofluorescence imaging of A549 cells treated with 0.5 µM ZQX-1 under irradation at 420 nm. As shown in Figure 3, compared with the cells in Control Group (DZ), the level of cleaved caspase-3 was significantly increased subject to ZQX-1 treatment under irradiation at 420 nm, evidencing the cell death of A549 cells in LZ group.

### 2.2. Quantitative Proteomics Analysis

**Figure 4:**
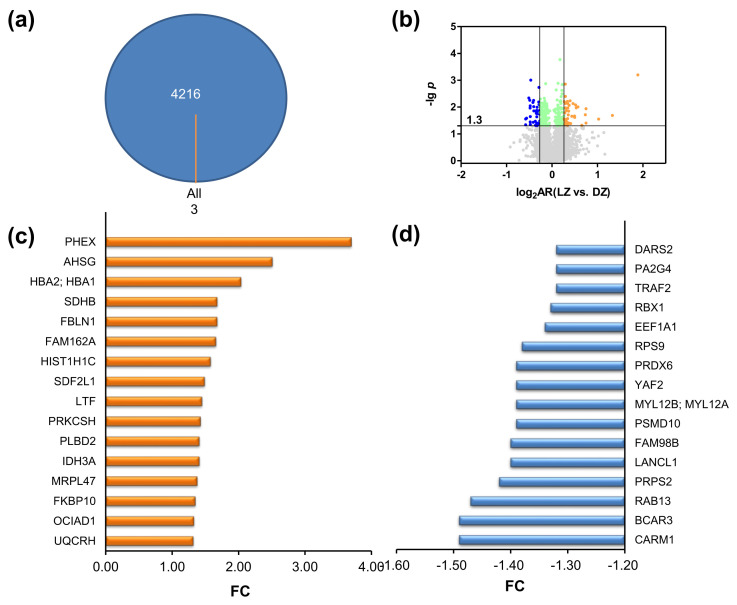
Quantitative proteomics analysis. (**a**) Venn diagram of number of proteins identified in A549 cells treated with 0.5 μM of ZQX-1 with (LZ) or without (DZ) irradiation, showing 4216 proteins commonly expressed in three replicates of each group. DZ: cells were incubated with 0.5 μm ZQX-1 in dark for 4.5 h; LZ: cells were incubated with 0.5 μM ZQX-1 in dark for 4.0 h and refreshed and irradiated at 420 nm for 20 min, followed by further 0.5 h of incubation in dark. (**b**) Volcanic map of proteins identified in both DZ and LZ group with various abundance ratios (ARs) of LZ vs. DZ and *p*-values. Grey points refer to proteins with a *p*-value of > 0.05 (i.e., −lg *p* < 1.3), while orange, green and blue ones to proteins with a *p* ≤ 0.05 (−lg *p* ≥ 1.3 ); orange points also refer to proteins with a log_2_AR (LZ vs. DZ) of ≥ 0.26, green ones to proteins with a log_2_AR of < 0.26 or > 0.26 and blue ones to proteins with log_2_AR of ≤ −0.26. (**c**,**d**) Differentially expressed proteins (DEPs) identified in A549 cells of LZ group with a fold change (FC) of ≥ 1.3 (**c**) or ≤ −1.3 (**d**), compared to those in A549 cells of DZ group.

To explore the molecular mechanism of action of ZQX-1 as an anticancer agent upon irradiation, we applied quantitative proteomics analysis based on mass spectrometry (MS) coupled to tandem–mass–tag (TMT) labeling to profile the protein expression of A549 cells exposed to 0.5 μM of ZQX-1 in the dark followed by irradiation at 420 nm (LZ group). We then compared this approach with that of A549 cells incubated with 0.5 μM of ZQX-1 but without irradiation (DZ group). Based on three independently biological replicates, we totally identified 4219 proteins that were commonly expressed in both groups of cells (Figure 4a, Table S1 in the Supplementary Materials); among them were 247 proteins statistically reliable with a *p*-value of ≤ 0.05 (Figure 4b). Of the 247 proteins, 163 proteins had an abundance ratio (AR) of 0.83–1.20 (green points in Figure 4b), where AR is the ratio of the abundance of a protein expressed in A549 cells in LZ group to that of the protein expressed in DZ group (Figure 4b). This indicates that the expression of a large proportion (~67%) of proteins in A549 was not significantly affected by exposure to ZQX-1 under irradiation. However, among the 247 proteins were 43 proteins (orange points in Figure 4b) upregulated with an AR of ≥ 1.20 and 41 proteins (blue points in Figure 4b) downregulated with an AR of ≤ 0.83.

To straightforwardly indicate the changes in expression of proteins between the cells of LZ and DZ groups, we used fold-change (FC) to represent the change in the expression level of proteins. When AR > 1, FC is equal to AR, indicating the protein was upregulated in LZ group; if AR < 1, FC is equal to the negative reciprocal of AR, indicating the protein was downregulated in LZ group compared to the same protein expressed in DZ group. Accordingly, we found that 17 proteins were significantly upregulated with FC ≥ 1.3 (Figure 4c) and 16 proteins significantly downregulated with FC ≤ −1.3 (Figure 4d) in the A549 cells exposed to ZQX-1 under irradiation. The differentially expressed proteins (DEPs) with 1.3 ≥ |FC| ≥ 1.2 identified in LZ group are shown in Figure S2.

Notably, phosphate-regulating neutral endopeptidase (PHEX), which is encoded by the phosphate-regulating endopeptidase homolog X-linked (PHEX) gene and located at reticulum, Golgi and membrane was upregulated by 3.70-fold, making it the most upregulated protein in A549 cells exposed to ZQX-1 under irradiation. In contrast, the histone-arginine methyltransferase CARM1 was downregulated by 1.49-fold, making it the most downregulated protein in the A549 cells. PHEX belongs to the M13 family of membrane-bound Zn metalloendopeptidases [31]. The inactivating variants in *PHEX* gene were reported to upregulate the expression of fibroblast growth factor 23 (FGF23), causing X-linked hypophosphatemic rickets (XLH) [32]. As an endopeptidase, PHEX cleaves N-linked glycoprotein to release acidic serine- and aspartate-rich motif (ASARM) peptides, which potentially inhibit mineralization, contributing to the development of XLH [33,34]. However, the role of PHEX in the anticancer activity of phototherapeutic agents remains to be explored. In regard to the most downregulated protein CARM1, which catalyzes demethylation of histone H3 at arginine 17, it has been commonly considered that this protein is associated with human carcinogenesis. Several selective inhibitors against CARM have been developed as potential therapeutic agents for various cancers [35]. Our results here suggest that ZQX-1 upon irradiation reduced the expression of CARM in A549 cells, perhaps contributing to its cytotoxicity by disrupting the methylation of H3.

### 2.3. Bioinformatics Analysis

To explore the biological functions of all DEPs identified above, we performed Gene Ontology (GO) analysis. The GO annotations, including cellular components, molecular functions and the involved biological processes of the DEPs with |FC| ≥ 1.2, are shown in Figure S3, while the detailed information is listed in Table S2. These DEPs are mainly located at cytoplasm, mitochondrion, ribosome and different lumens (Figure S3a), while a few are located at Golgi (Table S2). Many of the DEPs are RNA binding proteins or enzymes which possess different catalytic activity, e.g., ribose phosphate diphosphokinase activity and diphosphotransferase activity, nicotinamide N-methyltransferase activity, amine-lyase activity and glucosamine kinase activity (Figure S3b). They involve various biological processes, the top five of which are translational elongation and translation, organonitrogen compound metabolic and peptide biosynthetic processes and positive regulation of proteolysis (Figure S3c).

**Figure 5:**
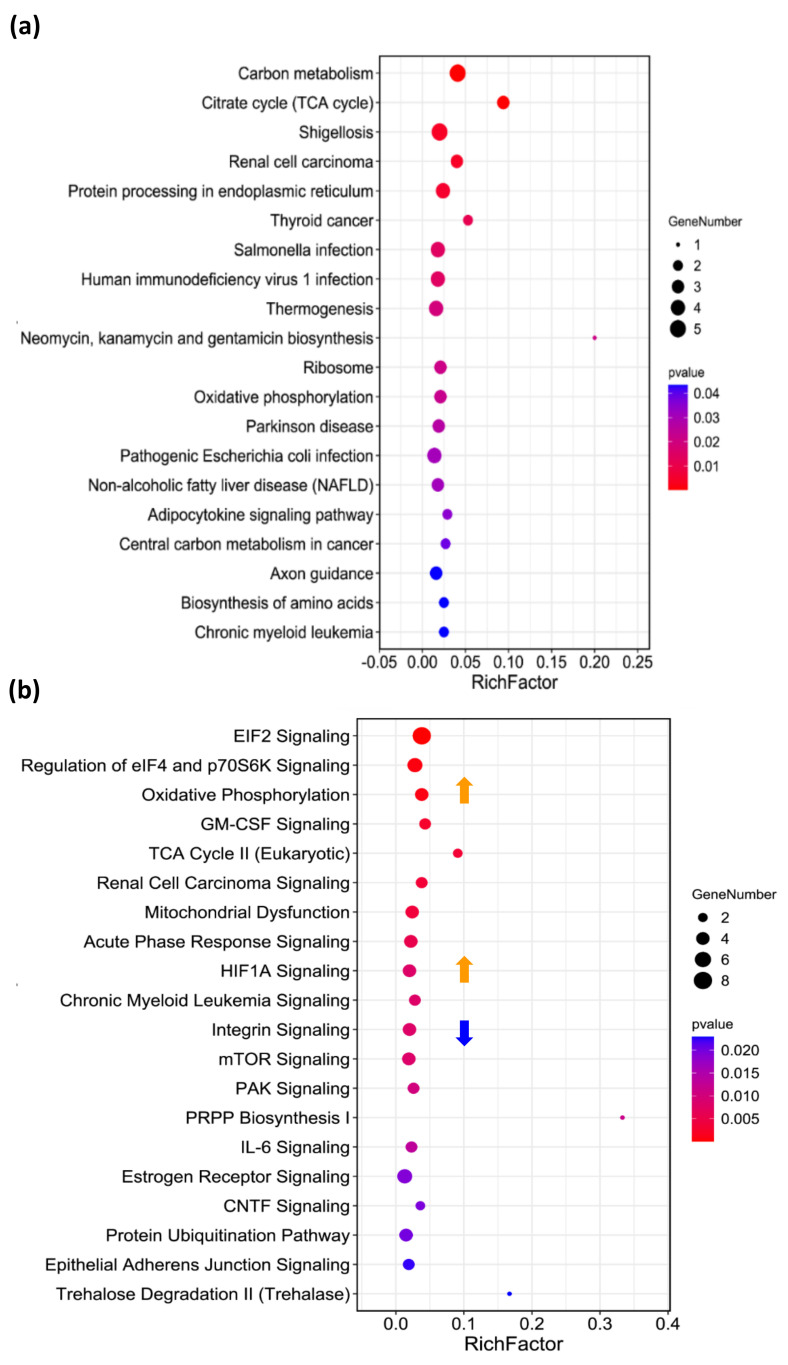
Bioinformatics analysis of differentially expressed proteins (DEPs) with |FC| ≥ 1.2 identified in A549 cells exposed to 0.5 μM of ZQX-1 under irradiation at 420 nm. (**a**) Top 20 associated KEGG pathways. (**b**) Top 20 associated canonical pathways enriched by IPA. Orange arrow refers to activation, while blue one refers to inhibition of a signaling pathway.

Moreover, we performed KEGG (Kyoto Encyclopedia of Genes and Genomes) annotation on the associated signaling pathways of the DEPs. As shown in Figure 5a, the most associated KEGG signaling pathway is carbon metabolism, including carbohydrate metabolism, metabolism of cofactors and vitamins and nucleotide metabolism (Figure S4). Furthermore, the photoactivated ZQX-1 was shown to disrupt genetic information processing, e.g., translation, replication and repair and transcription (Figure S4). These suggest that upon photoactivation, ZQX-1 effectively generated reactive oxygen species (ROS) and released the unstable pyrene derivative [9] to cause DNA lesions, disrupting cellular metabolism and blocking DNA synthesis, which as a consequence arrested cells at S phase (Figure 2c,d) and induced cell necrosis and apoptosis (Figure 2b). Moreover, the DEPs are shown to be highly associated with human cancers, e.g., renal cell carcinoma, thyroid cancer and chronic myeloid leukemia (Figure 5a and Figure S4), supporting the idea that ZQX-1 is an excellent candidate for cancer therapy.

To further decipher the molecular mechanism of action of ZQX-1 inducing rapid necrosis and apoptosis of A549 cells, we enriched the associated canonical pathways of the DEPs with |FC| ≥ 1.2 via Ingenuity Pathway Analysis (IPA). The IPA annotation (Figure 5b) showed that the top 5 most associated canonical pathways of the DEPs are EIF2 signaling (Figure S5), regulation of elF4 and p70S6K signaling (Figure S6), oxidative phosphorylation (Figure S7), granulocyte-macrophage colony-stimulating factor (GM-CSF) signaling (Figure S8) and TCA cycle II pathways (Figure S9). The DEPs were also shown to be highly associated with renal cell carcinoma signaling, mitochondrial dysfunction and acute phase response signaling (Figure 5b); this finding is consistent with the KEGG annotation (Figure 5a). Due to the complexity of the enriched signaling pathways in which both upregulated and downregulated proteins by ZQX-1 are involved (see examples in Figures S5–S9), IPA could not predict whether most of the top 20 pathways were activated or inhibited by treatment of ZQX-1 upon irradiation (Figure S10). However, IPA annotation predicted that the oxidative phosphorylation signaling pathway (Figure S7) and hypoxia-inducible factor 1A (HIF1A) signaling pathway (Figure S11) were activated with a Z-score of +2.6 and +2.0, respectively, while the integrin signaling pathway (Figure S12) was inhibited with a Z-score of −1.7 (Figure S10).

**Figure 6:**
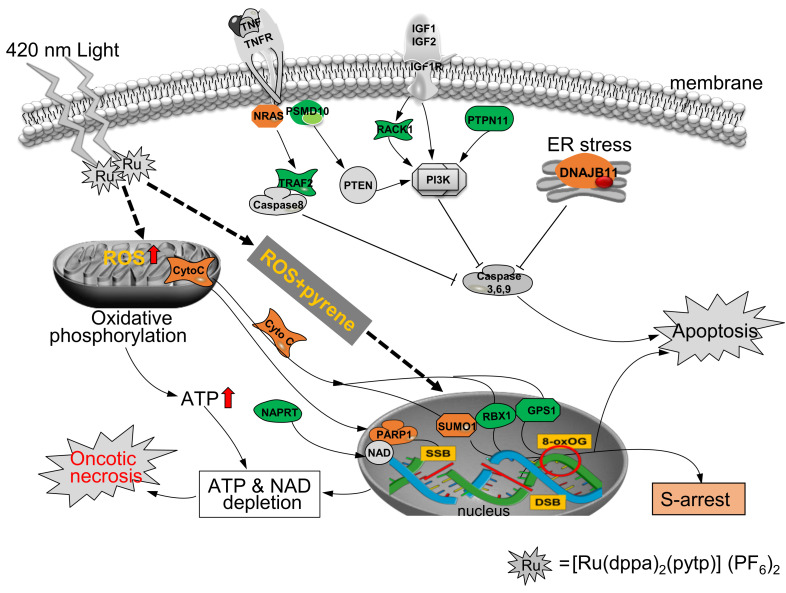
Diagram of molecular mechanism of ZQX-1 inducing oncotic necrosis and apoptosis of A549 cells upon irradiation at 420 nm. The green background behind a protein name refers to downregulation of protein, while orange refers to upregulation.

In the oxidative phosphorylation signaling pathway, four core proteins—ATP synthase subunit e (ATP5I or ATP5ME), cytochrome c oxidase subunit 7A2 (COX7A2), cytochrome b-c1 complex subunit 6 (UQCRH) and succinate dehydrogenase iron-sulfur subunit (SDHB)—were upregulated due to treatment of ZQX-1 under irradiation (Figure S7). SDHB is the iron–sulfur protein (IP) subunit of succinate dehydrogenase (SDH). It is involved in the formation of respiratory complex II of the mitochondrial electron transport chain (METC) (Figure S7) and responsible for transferring electrons from succinate to ubiquitin (coenzyme Q) [36,37]. UQCRH, as a component of ubiquitin–cytochrome C oxidoreductase, is a multi-subunit transmembrane complex that, as part of complex III of METC, drives oxidative phosphorylation [38]. Similarly, COX7A2 is one of components of complex IV of METC involved in oxidative phosphorylation process, of which the related pathways include AMPK enzyme complex pathway, respiratory electron transport, chemosmotic coupling to ATP and uncoupling protein thermogenesis [39]. The ATP5I gene expresses a transport vector in the METC complex V (Figure S7). This mitochondrial ATP synthase catalyzes ATP synthesis by using the electrochemical gradient of protons across cell membranes during oxidative phosphorylation [40]. Collectively, the four components in the regulatory complexes II–V of METC were all upregulated, which, consequently, promoted oxidative phosphorylation signaling to overproduce ATP and release cytochrome c, causing mitochondrial dysfunction and triggering downstream process; this process included oncotic necrosis, which can deplete excess of ATP [41] overproduced by activation of oxidative phosphorylation signaling (Figure 6).

As mentioned earlier, upon irradiation ZQX-1 promoted ROS level and released pyrene derivative [9]. The cytotoxic ROS [42] and pyrene derivative [43,44] can cause DNA lesions, e.g., guanine oxidation, double-strand breaks (DSBs) and single-strand breaks (SSBs) (Figure 6). Without transcriptomic studies, we cannot speculate which of the DEPs we identified resulted from the DNA damages caused by ROS and/or pyrene derivative. However, our proteomics analysis revealed the differentially expression of several proteins responding to or recognizing DNA damage. They are Poly ADP-ribosepolymerase 1 (PARP1, FC = 1.27), GTPase NRas (N-Ras, FC = 1.27), small ubiquitin related modifier (SUMO1, FC = 1.20), E3 ubiquitin-protein ligase (RBX1, FC = −1.33) and COP0 signalosome complex sub-unit 1 (GPS1, FC = −1.26) (Table S3). Among them, both SUMO1 [45,46] and N-Ras [47] are involved in DNA damage response and DNA repair in ATM-dependent manner. PARP1 is also involved in the DNA damage response and checkpoint regulation via binding to zinc finger proteins. It transfers the oxidized ADP unit in nicotinate amine dinucleotide (NAD) to key enzymes, e.g., aprataxin PNK-like factor (APLF) and checkpoint proteins with FHA and RING domains (CHFR) [48,49,50]. The activation or high expression of PARP1 could inhibit caspase-3-like activity [48], which, consequently, switched cells to oncotic necrosis (also termed oncosis) to deplete NAD and ATP overproduced by DNA damage and oxidative stress in cells (Figure 2b and Figure 6). The elevation in expression level of these DNA damage response proteins was evidenced by the occurring of DNA damage in A549 cells exposed to ZQX-1 under irradiation. On the other hand, both the DNA damage recognition proteins—E3 ubiquitin-protein ligase (RBX1) and COP0 signalosome complex subunit 1 (GPS1)—were downregulated by photoactivated ZQX-1, reflecting the impairment of DNA repair capacity in the A549 cells (Figure 6). These collectively confirmed that it was the DNA damage caused by photoactivation of ZQX-1 that inhibited DNA replication and transcription, arresting A549 cells at S phase (Figure 2c–f). Notably, the nicotinate phosphoribosyltransferase (NAPRT), which catalyzes the first step in the biosynthesis of NAD from nicotinic acid [51], was downregulated by 1.26-fold. This indicates that the reduction in NAPRT expression level may also contribute to lower NAD level in A549 cells exposed to ZQX-1 under irradiation (Figure 6).

The cell cycle checkpoint is a negative feedback regulation responding to DNA damage [52]. The proliferation-associated protein 2G4 (PA2G4) and p21-activated protein kinase-interacting protein 1 (PAK1IP1) function to regulate G1/S checkpoint of cell cycle to prevent damaged DNA from entering S phase [53,54]. We identified that both PA2G4 (FC = −1.32) and PAK1IP1 (FC = −1.25) were downregulated by ZQX-1 upon irradiation, which facilitated A549 cells to enter S phase. However, the inhibition on the DNA replication and synthesis described above apparently prevented cells from proceeding to G2 phase, which implied that PA2G4 and PAK1IP1 played important roles in arresting A549 cells at S-phase.

It is well-known that PDT not only induces oxidative stress and anti-tumor immune response, but also triggers the activation of survival pathways, such as hypoxia-inducible factor 1A (HIF1A) survival signaling and unfolded protein response-mediated survival pathway [55]. The activation of HIF1A signaling was considered to contribute to the relatively refractory response to PDT of some tumor types, e.g., perihilar cholangiocarcinoma [55]. Despite ZQX-1 being demonstrated to remarkedly promote ROS level in A549 cells [9], we did not detect increased expression of HIF1A in A549 cells exposed to ZQX-1 upon irradiation. Instead, our proteomic analysis showed the changes in expression level of three proteins—N-Ras (FC = 1.27), hexokinase-1 (HK1, FC = 1.23) and L-lactate dehydrogenase α chain (LDHA, FC = −1.20) (Table S4)—which account for activation of HIF1A survival signaling in A549 cells exposed to ZQX-1 under irradiation (Figures S10 and S11). N-Ras is a member of the Ras sub-family of small GTPases, which regulates the signaling pathways involved in cell proliferation, differentiation and survival [56]. The *RAS* genes, including *H-RAS*, *N-RAS* and *K-RAS*, were shown to be mostly mutated oncogenes in human cancer cells ascribed to oxidative stress and DNA damage [47,57]. Herein, we show that ZQX-1-induced upregulation of N-Ras contributes to activation of the HIF1A survival signaling, though other proteins, such as LDHA, the receptor of activated protein C kinase 1 (RACK1) and RBX1 involved in HIF1A survival signaling, were simultaneously downregulated (Figure S11). It is obvious that the activation of HIF1A signaling did not compete with oncosis and apoptosis induced by ZQX-1 upon irradiation to rescue cells from death. However, these results imply that in combined use of HIF1A signaling inhibitors, ZQX-1 may become a more active PDCT prodrug.

On the other hand, DNA damage and hypoxia caused by ROS can also activate the mitochondrial death pathway of cells. Here, the upregulation of mitochondrial protein FAM162A (FC = 1.66), which is a stress-binding protein associated with cell death [58], coincided with activation of the mitochondrial death pathway. Moreover, we found that the endoplasmic reticulum protein DNA J homolog subfamily B member 11 (DNAJB11) was upregulated by 1.27-fold in the A549 cells exposed to ZQX-1 under irradiation. DNAJB11, also known as ERgj3 or HSP40, is a molecular chaperone associated with the folding, translocation and complexation of newly synthesized proteins in endoplasmic reticulum (ER) [59,60] and is involved in PDT-induced unfolded protein response. Responding to ER-stress and hypoxia induced by chemotherapy including PACT, heat shock proteins, such as DNAJB11 (HSP40) and HSP27, are increasingly expressed, protecting the cells from the photocytotoxicity via enhancing the survival of cells [61] (Figure 6), which is similar to HIF1A survival signaling [55]. Therefore, DNAJB11 inhibitors may enhance the photocytotoxicity of ZQX-1, similar to HIF1A signaling inhibitors.

Unlike the key proteins in the oxidative phosphorylation signaling pathway, which were all upregulated by ZQX-1 upon irradiation, three core proteins involved in integrin signaling pathway, i.e., actin-related protein 2/3 complex subunit 4 (ARPC4), myosin regulatory light chain 12B (MYL12B) and breast cancer anti-estrogen resistance protein 3 (BCAR3), were downregulated by 1.24–1.49-fold under the same conditions. Integrin signaling regulates many cellular behaviors, including motility and growth of cells and cell survival. Despite the N-Ras involved in integrin, signaling pathway was upregulated (Figure S12), while the integrin signaling in A549 cells exposed to ZQX-1 under irradiation was predicted by IPA to be significantly inhibited (Figure S10). One possibility may be that the downregulation of BCAR3, ARPC4 and MYL12B can inhibit cytoskeletal rearrangement and retard cell motility (Figure S12). This finding is consistent with results obtained by cellular phenotype characterization that found that the photoactivated ZQX-1 restrained cell growth and induced oncosis; this finding is reflected by the generation of membrane fragments and cell debris (Figure 1c–f).

Eukaryotic initiation factor 2 (EIF2) signaling is one of two well-known pathways in eukaryotic cells that regulate translation initiation in response to stress (Figure S5). Herein, we found that in the EIF2 signaling pathway, two proteins were upregulated and six proteins downregulated in A549 cells treated with ZQX-1 under irradiation. The downregulated proteins, i.e., EIF3K, PAIP1, RPL32, PRL35A, RPS3 and RPS9, are all components of EIF3 and EIF2 complexes and 60S and 40S ribosomal sub-units. It is well known that ribosomes can read the genetic codes contained in mRNA and translate them into the sequence information of amino acids for protein synthesis, which is crucial for the survival and growth of cells. The EIF3 complex plays a central role in the initiation of mRNA translation by promoting the binding of mRNA to the 40S sub-unit. The K sub-unit of EIF3 can also participate in cell cycle regulation, selectively modulating protein synthesis by regulating the initiation of translation of different types of mRNA [62,63]. Considering the downregulation of EIF3K, PAIP1, RPL32, RPL35A, RPS3 and RPS9 caused by ZQX-1 treatment, it is obvious that this ruthenium complex, upon irradiation, significantly disrupted the assembly of ribosomes and translation process, which inhibited protein synthesis and induced cell death. These results are well consistent with KEGG annotation claiming that the ZQX-1-induced DEPs are highly associated with genetic information processing, e.g., transcription and translation (Figure S4).

As mentioned above, our cellular phenotype characterization demonstrated that late apoptosis induced by the photoactivated ZQX-1 was the death mode of A547 cells second to necrosis (Figure 2b). Apoptosis can generally proceed through three different pathways, namely, the exogenous apoptosis, e.g., Fas receptor (FasR) and CASP8/10-initiated apoptosis, cytochrome C-initiated apoptosis and the endoplasmic reticulum (ER) stress-induced apoptosis [64,65]. Here, we found that, in the A549 cells exposed to ZQX-1 under irradiation, the tyrosine-protein phosphatase non-receptor type 11 (PTPN11) was downregulated by 1.29-fold (Figure S2), which may lead to apoptosis due to inactivation of PI3K survival signaling (Figure 6). Meanwhile, the tumor necrosis factor (TNF) receptor-associated factor 2 (TRAF2) and the receptor of activated protein C kinase 1 (RACK1) were also downregulated by 1.32- and 1.23-fold, respectively. TRAF2 inhibits the death receptor (DR)-mediated apoptosis via mediating degradation of activated caspase-8, leading to negative regulation of TNFR signaling and NF-κB signaling pathway [66,67]. RACK1 as a scaffold protein binds to and stabilizes activated protein kinase C (PKC), increasing PKC-mediated phosphorylation and promoting downstream phosphorylation signaling [68]. Taken together, the downregulation of PTPN11, TRAF2 and RACK1 may synergistically contribute to ZQX-1 induced (endogenous) apoptosis upon irradiation (Figure 6).

The 26S proteasome non-ATPase regulatory sub-unit 10 (PSMD10), also known as Gankyrin, is an ankyrin repeat anti-apoptotic oncoprotein against DNA damaging agents [69,70] and has been shown to contribute to oncogenesis in endometrial and cervical cancer cells [71]. The depletion of PSMD10 was reported to inhibit endometrial cancer cell proliferation via the PTEN/PI3K/AKT signaling pathway [72] (Figure 6). In the ATM signaling pathway, the downregulation of PSMD10 could inhibit the degradation of CDC25A [73,74], thereby increasing the level of CDC25A, inhibiting cyclin dependent kinase 2 (CDK2) and arresting cells at S-phase [74,75]. Here, we found that PSMD10 was downregulated by 1.39-fold by photoactivated ZQX-1, implying that this protein also contributed to ZQX-1-induced cell-cycle arrest and apoptosis (Figure 6).

**Figure 7:**
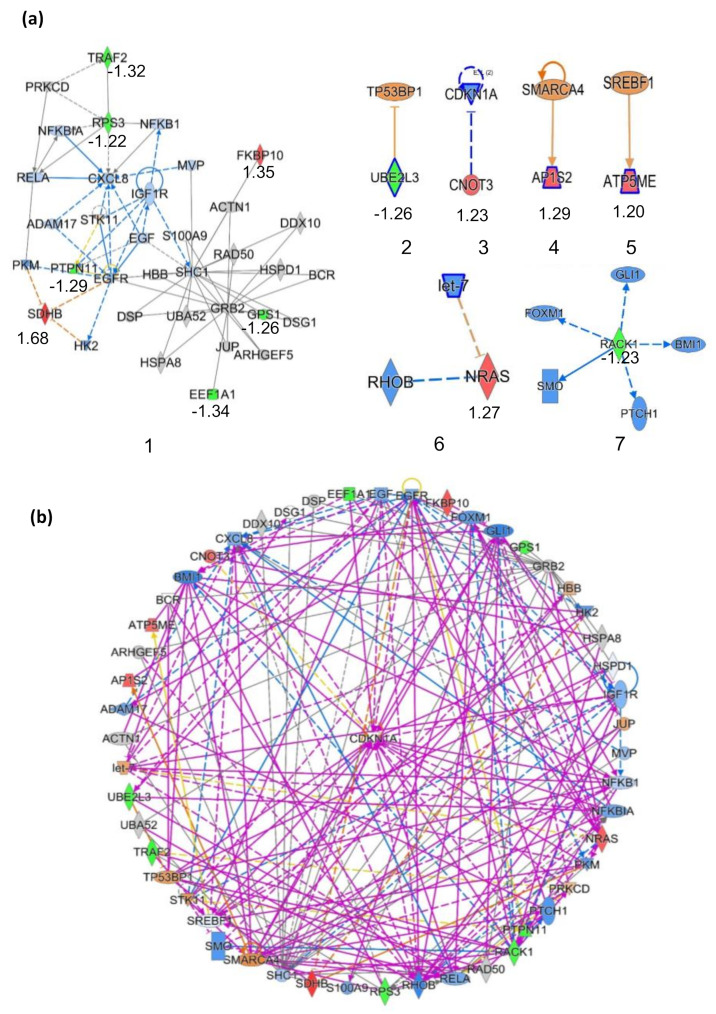
Enrichment of core protein–protein interaction (PPI) networks of DEPs with |FC| ≥ 1.2 identified in A549 cells exposed to ZQX-1 under irradiation. (**a**) The seven PPI networks with which the DEPs are involved; (**b**) PPI network merged from the 7 PPI networks shown in (**a**). The red color refers to upregulation of the genes, while green refers to downregulation with fold-change value underlying the gene name in (**a**). The orange color indicates that gene is predicted by IPA to be upregulated, blue to be downregulated, and grey to have no prediction available. The solid line indicates direct interaction between two proteins, while the dot line indicates indirect interaction.

To further explore the molecular mechanism of action of the photoactivatable ZQX-1, we performed Ingenuity Pathway Analysis (IPA) to enrich the core protein–protein interaction (PPI) networks of DEPs with |FC| ≥ 1.2 identified in A549 cells exposed to ZQX-1 under irradiation. We enriched seven PPI networks (Figure 7) with which the DEPs are involved. As shown in Table S5, these networks are highly associated with cancer, cell cycle, cell death and survival, cellular development and cell growth and proliferation, supporting the ideas that the photoactivated ZQX-1 induces cell death by disrupting cell cycle and cell proliferation.

We merged the seven PPI networks into one network. As shown in Figure 5b, the DEPs involved in this single network interact, directly or indirectly, with each another through the core protein CDKN1A. The cyclin-dependent kinase inhibitor 1 (CDKN1A), also known as CDK-interacting protein 1 (Cip1), is involved in p53/TP53 mediated inhibition of cellular proliferation by preventing phosphorylation of critical cyclin-dependent substrates responding to DNA damage [76,77]. This protein can interact with proliferating cell nuclear antigen, a DNA polymerase accessory factor, playing a regulatory role in DNA replication and DNA damage repair at S-phase [78]. Moreover, CDKN1A can be specifically cleaved by caspase 3-like caspase, playing a crucial role in caspase-induced apoptosis [79]. Collectively, the IPA enrichment suggests that the DEPs identified in A549 cells exposed to ZQX-1 upon irradiation interacted with each another through CDKN1A, triggering p53 mediated inhibition of cellular proliferation by reducing phosphorylation of cyclin-dependent kinases (CDKs) to induce cell apoptosis.

**Figure 8:**
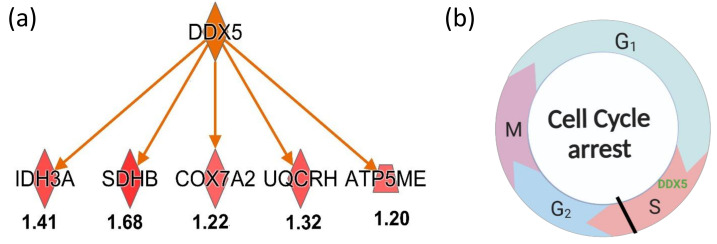
(**a**) Enriched upstream regulators DDX5 in turn regulate five downstream proteins as shown; (**b**) DDX5 promotes cell cycle into S phase and DNA damage induced cell cycle arrest in S phase.

When we used IPA to enrich the upstream regulation proteins of DEPs described above, we found that the most significant upstream regulation protein is DEAD-box helicase 5 (DDX5) (Figure 8a), which is an ATP-dependent RNA helicase. Interestingly, the DDX5 protein in turn regulated five DEPs identified in A548 cells as exposed to ZQX-1 under irradiation (Figure 8a), of which SDHB, COX7A2, UQCRH and ATP5ME are involved in oxidative phosphorylation signaling (Figure S7) and SDHB and IDH3A are involved in TCA cycle signaling pathway (Figure S9). This implies that DDX5 plays a crucial role in the two signaling pathways. Moreover, DDX5 regulates CDKN1A protein, being closely related to cell cycle process. DDX5 can also bind to HNRNPA1, which was downregulated by 1.27-fold and related to EIF2 signaling (Figure S5). These results collectively indicate that DDX5 played an important role in G1/S phase progress [80]. The transition from G1 to S phase was accompanied by an increase in DDX5 level in the cells [81], while S-phase is the only stage in which DNA is synthesized. However, as mention above the DNA synthesis was blocked due to DNA damage by cytotoxic ROS and pyrene derivative released by ZQX-1 upon irradiation [9]; thus, those subjected to exposure to ZQX-1 under irradiation in a large amount of the A549 cells were arrested at S phase, as shown by the cellular phenotype characterization (Figure 2b–f and Figure 8b).

## 3. Materials and Methods

### 3.1. Materials and Reagents

The ruthenium(II) polypyridine complex ZQX-1, [Ru(dppa)_2_(pytpy)](PF_6_)_2_ (Figure 1a) (dppa = 4,7-diphenyl-1,10-phenanthroline, pytp = 4′-pyrene-2,2′:6′,2′′- terpyridine) was synthesized following the procedure reported previously [9]. Human non-small cell lung cancer cells (A549), human cervical cancer cells (HeLa) and human renal epithelial cells (HEK293T) were purchased from the Institute of Basic Medicine at the Chinese Academy of Medical Sciences. Phosphate buffer saline (PBS), cell scrapers, cell petri dishes and centrifugal tubes were provided by Beijing Solaibao Technology Co. Ltd. (Beijing, China). The BCA Kit was purchased from Beyotime Biotechnology Co. Ltd. (Jiangsu, China). SDS-PAGE prefabricated gel, TRIS-MOPS-SDS running buffer powder and 4 × LDS sample buffer were obtained from Beijing Bair Biotechnology Co. Ltd. (Beijing, China), while the Apoptosis Detection Kit was obtained from BD Sciences (San Jose, CA, USA). Urea was purchased from Beijing Inokai Technology Co. Ltd. (Beijing, China), dithiothreitol (DTT), iodoacetamide (IAA) and trypsin from Sigma-Aldrich (St. Louis, MI, USA) and formic acid from Honeywell (Charlotte, NC, USA). Trifluoroacetic acid was purchased from Alfa Aesar (Taiwan), HEPES from Beijing Boaotuoda Technology Co. Ltd. (Beijing, China) and hydroxylamine hydrochloric acid from Adamas Reagent Co. Ltd. (Shanghai, China). Chromatographic-grade water, mass spectrometric-grade acetonitrile and water, nitric acid, TMT labeling reagent and low adsorption tubes were purchased from Thermo Fisher Technology China Co. Ltd. (Waltham, MA, USA). Ultrapure water was produced by Millipore pure water system. The tuning solution for ICP-MS was provided by Agilent Technologies Co. Ltd. (Santa Clara, CA, USA).

### 3.2. Cell Culture

Three cell lines—A549, HeLa and HEK293T—were cultured in Dulbecco’s Modified Eagle’s Medium (DMEM) containing 10% fetal bovine serum (FBA, Gibco, Grand Island, NY, USA) and 1% penicillin–streptomycin (GE Heathcare Life Sciences, Chicago, IL, USA). All cells were cultured in 5% CO_2_ in a 37 °C incubator (SANYO, Osaka, Japan).

### 3.3. Bright-Field Imaging

A549 cells were seed into three petri dishes (10 cm in diameter) and cultured in DMEM at 37 °C until achieving 80% coverage; they were then refreshed with DMEM containing 0.5 μM of ZQX-1 and further cultured for 4.0 h in the dark, followed by washing by PBS 3 times and refreshing with drug-free DMEM. Thereafter, two dishes of cells were irradiated under blue light at 420 nm for 20 min with a power density of 22.5 mW cm^−2^ and further cultured for 0.5 h and 1.0 h, respectively, in the dark. The third dish of cells was not irradiated, instead being further cultured for 1.0 h in the dark. Finally, the three dishes of cells were imaged on an inverted optical microscope (CKX31, OLYMPUS, Tokyo, Japan).

### 3.4. Immunofluorescence Confocal Imaging

A549 cells were cultured with DMEM medium in confocal dishes until dishes were overgrown with cells. After 4 h of culture in the DMEM medium with ZQX-1(0.5 μM) and washed by PBS three times, the A549 cells were divided into two groups: The Light group (LZ) was exposed to 420 nm light (22.5 mW cm^−2^) for 20 min and further incubated for 30 min in the dark; the dark group (DZ) was incubated for further a 1 h in the dark without exposure to light. The two groups of A549 cells were fixed in mixture solution of methanol-acetic acid 3:1 (*V*/*V*) for 10 min at −20 °C and permeated with 0.1% Triton-X100/PBS for 30 min. After 2 h blocking with 5% BSA/PBS, the cells were incubated with 0.2% cleaved caspase-3 primary antibody (Asp175) for 2 h at 37 °C and further incubated with 0.1% Alexa Fluor^®^ 488 secondary antibody (ab150065) for 1 h. Thereafter, the cells were incubated with nuclear staining reagent DAPI (0.1 g/mL) for 15 min, followed by washing with PBS three times. Finally, fluorescence images were recorded by confocal laser scanning microscopy (OLYMPUS, FV3000, Tokyo, Japan).

### 3.5. In Vitro Anti-Proliferative Assay

The A549 or HEK293T cells were transferred into a 96-well plate at a density of 6000 cells/well, cultured in DMEM for 24 h at 37 °C, refreshed with DMEM containing various concentrations of ZQX-1 and cultured for a further 4 h in the dark, followed by washing with PBS three times and refreshed with drug-free DMEM. The 96-well plate was then irradiated under 420 nm for 20 min at a power density of 22.5 mW cm^−2^ and further incubated for 30 min in the dark. Thereafter, 10 μL of CCK-8 reagent (MCE Group, Chiyoda, Japan) was added into each well for 2 h of reaction and the optical absorption density (OD) of each well was measured at 450 nm using a microplate reader (TECAN F50, Männedorf, Switzerland).

### 3.6. Flow Cytometer Assay

For the cell cycle assays, we set up two sets of experiments. In the first set of experiments, a pair of A549 cells samples were cultured in the presence and the absence of ZQX-1 (0.5 μM), respectively, for 4.0 h at 37 °C in the dark and washed three times by PBS, followed by refreshing with drug-free DMEM. Thereafter, the two samples cultured in the dark for further 30 min prior to flow cytometer analysis; the samples were designated as DZ (dark-with ZQX-1) and DB1 (dark-blank 1), respectively. Another pair of A549 samples cultured with and without ZQX-1, respectively, were cultured for 4.0 h at 37 °C in the dark and irradiated with blue light at 420 nm for 20 min, followed by culturing for 30 min in the dark prior to flow cytometer assay. These two samples were named LZ (light with ZQX-1) and LB (light-blank), respectively. In the second set of experiments, the sample preparation was similar to that of the first set of experiments, except for the concentration of ZQX-1 used, irradiation time and incubation time after irradiation. In the second set, the A549 cells cultured without ZQX-1, designated as DB2, was immediately collected after 4.0 h of incubation in the dark for flow cytometer assay, while the A549 cells designated as LZS, LZL and HZS, respectively, were cultured with 0.25 μM (LZS and LZL) or 0.5 μM (HZS) of ZQX-1 in the dark for 4.0 h, refreshed and then irradiated at 420 nm for 10 min (LZS and HZS) or 20 min (LZL). All the ZQX-1 treated samples (LZS, LZL and HZS) were also collected immediately for flow cytometer assay after irradiation on requested times.

For the apoptosis assay, the samples, including DB1, DZ, LB and LZ, were prepared following the similar procedure described above for cell cycle assays, except that the ZQX-1 treated samples, LB and LZ, were collected immediately for flow cytometer assay after 10 min of irradiation at 420 nm. The A549 cells in each sample were detached by trypsin without EDTA after removing suspension, followed by washing with PBS and centrifuging at 1000 rpm for 3 min and then transferred to FACS tubes. After re-suspension in 0.5 mL of 1× Binding Buffer, the cells were incubated with 5 μL Annexin-V conjugate for 5 min, followed by addition of 5 μL propidium iodide (PI) prior to the FACS analysis. The FACS assays were performed on a Calibur flow cytometer (BD, Franklin Lakes, NJ, USA). The data were quantified by FlowJo software ver. 7.6.1, Engine 2.79000, Java Ver.14.1-b02 (Treestar, St. Chico, CA, USA).

### 3.7. Inductively Coupled Plasma Mass Spectrometry (ICP-MS)

For ICP-MS analysis, A549, HeLa and HEK293T cells were cultured in DMEM containing 5.0 μM of ZQX-1 for 4 h in the dark and then washed by PBS three times, detached with trypsin without EDTA and transferred into 1.5 mL centrifuge tube. The cell density was measured with a cell counting apparatus (Luna-II, Gyeonggi-do, Korea) and the concentration of Ru in cells was detected by ICP-MS (Agilent 7700, Agilent Technologies Co. LTD, Santa Clara, CA, USA) after the cells were digested with 1% HNO_3_. The cellular uptake of Ru in the three cell lines was represented as the weight of Ru (pg) per 10^6^ cells. All measurements results were the mean of three independent experiments.

### 3.8. Quantitative Proteomics Analysis

The Quantitative proteomics analysis based on mass spectrometry coupled to tandem–mass–tag (TMT) labeling was performed by following the procedure previously reported by our previous study [23] with minor modifications. In brief, A549 cells cultured in DMEM as described above were divided into DZ (dark-ZQX-1) group and LZ (light-ZQX-1) group and incubated with 0.5 μM of ZQX-1 for 4 h at 37 °C in the dark. After washing by PBS three times and refreshing with DMEM, the DZ cells were incubated further in the dark for 50 min and the LZ cells were irradiated under 420 nm for 20 min at a power density of 22.5 mw/cm^2^, followed by further incubation of 30 min in the dark.

The two groups (DZ and LZ) of cells were individually harvested and lysed on ice and whole cell proteins were extracted by a total protein extraction kit (BestBio, Shanghai, China). A total of 300 μg of proteins extracted from each group was then transferred to 1.5 mL of low protein binding micro-centrifuge tube (Thermo Fisher Scientific, Waltham, MA, USA) and denatured by adding urea to a final concentration of 8 M, followed by incubation for 2 h at 25 °C in a thermomixer (Thermo Fisher Scientific, Waltham, MA, USA). The denatured proteins were then digested by trypsin following the reduction in disulfide bonds by DTT and alkylation of IAA in the dark. The tryptic peptides were then desalted in a C18 cartridge (Waters, Milford, MA, USA). The C18 column was, firstly, activated by 1 mL of acetonitrile (ACN) and 1 mL of 50% (vol/vol) ACN/H_2_O with 0.1% (vol/vol) formic acid (FA) successively, followed by equilibration with 3 mL of 0.1% (vol/vol) TFA in water. The peptides were then loaded onto the C18 column and the desalting was achieved by washing the column with 3 mL of 0.1% (vol/vol) TFA and 1 mL of 1% (vol/vol) FA successively. The peptide residues were sequentially eluted by 1 mL of 50% (vol/vol) ACN and 1 mL of 80% (vol/vol) ACN/H_2_O successively and the eluents were merged, followed by drying in vacuum centrifuge in CentriVap (Thermo Fisher Scientific, Waltham, MA, USA). The three replicates of DZ group were labeled with 127N, 128N and 130N and those of LZ group were labeled with 127C, 128C and 130C of TMT labeling reagent [23]. The labeled peptides derived from DZ and LZ groups were then equivalently mixed and dried by vacuum dryer, before being desalted and dried again as described previously [23].

The labeled peptide mixture was re-dissolved in 100 μL mobile phase A (10% (vol/vol) acetonitrile containing 4.5 mM ammonium formate, pH = 10) for basic reverse-phase chromatography pre-fractionation through HPLC (Agilent Technologies 1260 infinity, Santa Clara, CA, USA) with an Agilent ZORBAX 300 Extend-C18 column. The gradient started with 0% phase B (90% (vol/vol) acetonitrile containing 4.5 mM ammonium formate, pH = 10) until 7 min and continuously increased at 13 min to 16% B, at 73 min 40% B, at 77 min 44% B and at 82 min 60% B; it was then kept 60% B until 96 min, before being increased at 100 min to 90% B. The flow rate was 1 mL/min. The fractions were collected chronologically into twelve tubes from 3 to 96 min, spin-dried and re-dissolved with H_2_O containing 0.1% FA to a 500 μg/μL concentration for mass spectrometry analysis.

The mass spectrometric quantification was performed on an Orbitrap Fusion Lumos mass spectrometer coupled with an EASY-nLC 1200 nano-UPLC system equipped with an Acclaim™ PepMap™ 100 pre-column (20 mm × 75 μm, 3 μm) and Acclaim™ PepMap™ RSLC C18 analytical column (150 mm × 75 μm, 2 μm) (Thermo Fisher Scientific, Waltham, MA, USA). The UPLC mobile phase A was water containing 0.1% FA, while phase B 80% (vol/vol) was acetonitrile/water containing 0.1% FA. The UPLC gradient started with 2% B and increased at 7 min to 7%, then to at 69 min 20%, at 90 min 35% and sharply to 95% within 5 min; it then remained constant for 4 min and, finally, decreased to 2% within 8 min and remained for 3 min. Aliquot (1 μL) for each HPLC fraction described above was loaded to UPLC and the elution from the analytical column was directly infused to the mass spectrometer for MS/MS analysis.

The voltage of electrospray ionization (ESI) was set as 2500 V, while the ion transfer tube temperature was 320 °C. For primary mass spectrometry analysis, Orbitrap detector was operated with a mass resolution of 120,000 and a scanning range from 350 to 1800 *m*/*z.* The Orbitrap was also used to detect MS/MS with a mass resolution of 15,000 and MS^3^ with a resolution of 50,000 and a scanning range of 100–200 *m*/*z*. The HCD fragmentation cell ran with a collision energy of 23% for the second mass spectrometry (MS/MS) analysis and with a collision energy of 60% for the third mass spectrometry (MS^3^) analysis.

The acquired MS/MS Data were searched in Proteome Discoverer 2.3 (Thermo Fisher Scientific, Waltham, MA, USA) database for peptide and protein identification. Sequest HT search engine was used for peptide spectrum matching (PSM). The dynamic modifications were oxidation at methionine, methylation at lysine and arginine, acetylation at lysine and serine, phosphorylation at serine, threonine and tyrosine and TMT labeling at lysine. The static modifications were carbamidomethylation at cysteine and TMT labeling at N-terminus of peptides. The quantitative results were normalized based on the total peptide amount in each sample. Only proteins identified with a false discovery rate (FDR) ≤ 0.01, *p*-value ≤ 0.05 and abundance ratio ≤ 0.833 or ≥ 1.20 were included for bioinformatics analysis.

The entire quantitative proteomics analysis was carried out in three independent biological replicates.

### 3.9. Bioinformatics Analysis

Ingenuity Pathway Analysis (IPA) (QIAGEN Digital Insights) and STRING (Ver 11.5, https://cn.string-db.org, accessed on 5 August 2022) program was used to perform bioinformatics analysis on the differentially expressed proteins (DEPs) identified in A549 cells exposed to ZQX-1 under irradiation at 420 nm.

## 4. Conclusions

The ruthenium polypyridine complex [Ru(dppa)_2_(pytp)]_2_(PF_6_)_2_ (ZQX-1) showed excellent dual phototherapy (PDT and PACT) potency. With light irradiation at 420 nm, the complex causes rapid damage on cell membrane and induces cell oncotic necrosis. Furthermore, the photoactivatable ruthenium complex ZQX-1 showed selective cytotoxicity towards tumor cells over health cells. By means of quantitative proteomics analysis combined with cellular phenotype characterization and bioinformatics analysis, we demonstrated for the first time that, upon irradiation at 420 nm, ZQX-1 promoted ROS level in A549 cells to activate oxidative phosphorylation signaling and released cytotoxic ROS and pyrene derivative to inhibit DNA synthesis by damaging DNA. The dual action synergistically arrested A549 cells at S-phase, inducing cell oncotic necrosis to deplete ATP overproduced by activation of oxidative phosphorylation signaling and NAD responding to DNA damage. Moreover, the photoactivated ZQX-1 downregulated TRAF2, PSMD10, RACK1 and PTPN11, inducing endogenous apoptosis by inhibiting PTEN/PI3K/Akt signaling to further enhance the photocytotoxicity of ZQX-1. Importantly, we revealed that photoactivated ZQX-1 activated HIF1A survival signaling and DNAJB11 mediated survival signaling in A549 cells, though both survival signaling pathways could not compete with oncosis and apoptosis signaling to rescue cancer cells. Nevertheless, these findings imply that combined use with HIF1A signaling and DNAJB11 inhibitors may further promote the photocytotoxicity of ZQX-1. This work provides a theoretical basis for the rational design and development of highly effective phototherapeutic anticancer drugs.

## Data Availability

The data supporting reported results can be found in the Supplementary Materials.

## Supplementary Materials

The supporting information can be downloaded at: https://www.mdpi.com/article/10.3390/ijms24097756/s1.
