# Supplementary material for: A Photoactivated Ru (II) Polypyridine Complex Induced Oncotic Necrosis of A549 Cells by Activating Oxidative Phosphorylation and Inhibiting DNA Synthesis as Revealed by Quantitative Proteomics
Source: Int J Mol Sci. 2023 Apr 24;24(9):7756. doi: 10.3390/ijms24097756 (PMC10178167; doi:10.3390/ijms24097756)
Supplement: Supplementary file 1 [file ijms-24-07756-s001.zip › ijms-2251476-supplementary.pdf]

Supplementary Materials

Figure S1 – S12

Table S1 – S5

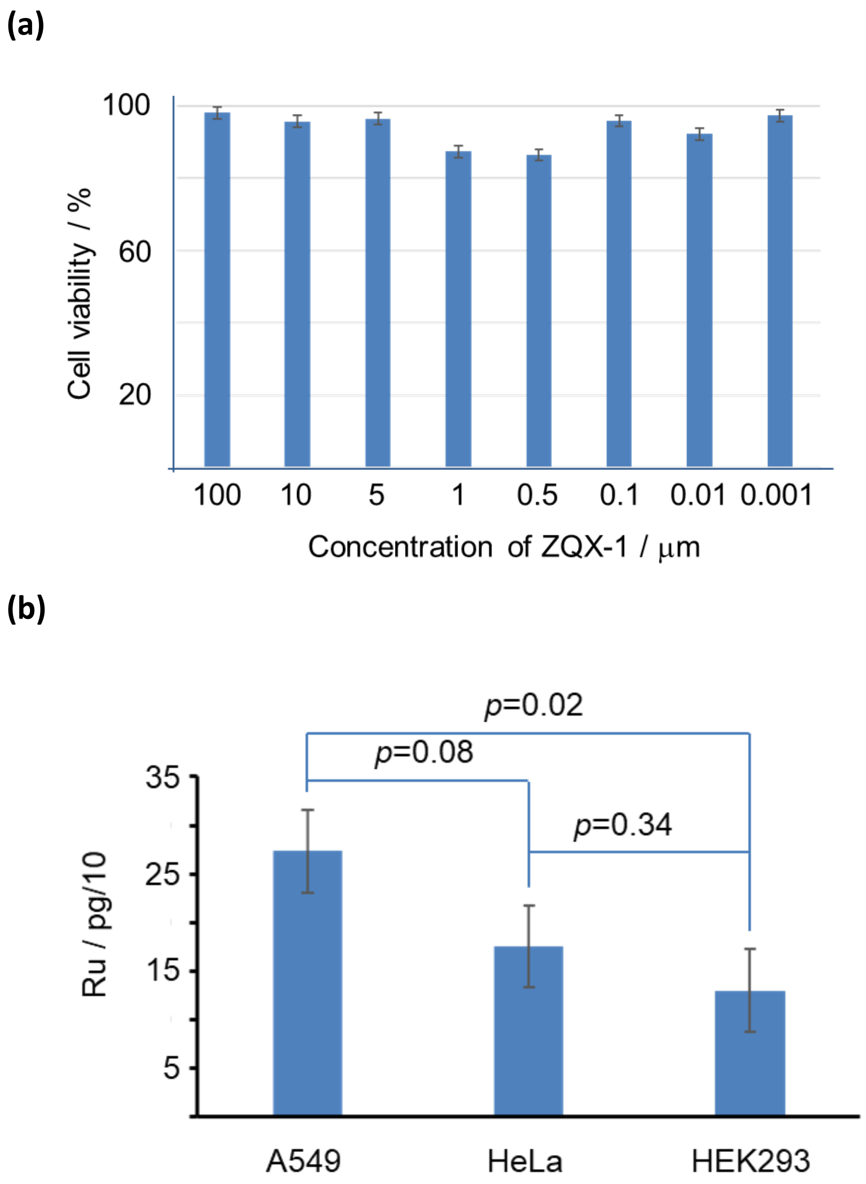

Figure S1. (a) The cell viability of HEK293 incubated with various concentrations of ZQX-1 in the dark for 4 h, followed by irradiation at 420 nm for 20 min and further incubation in the dark for 0.5 h. The cell viability (%) of HEK293T cells are present as mean ± SD, n=6. (b) Ru concentrations determined by ICP-MS in A549, HeLa and HEK293T cells exposed to ZQX-1 (5 µM) for 4 h in the dark. All results are present as mean ± SD, n=3.

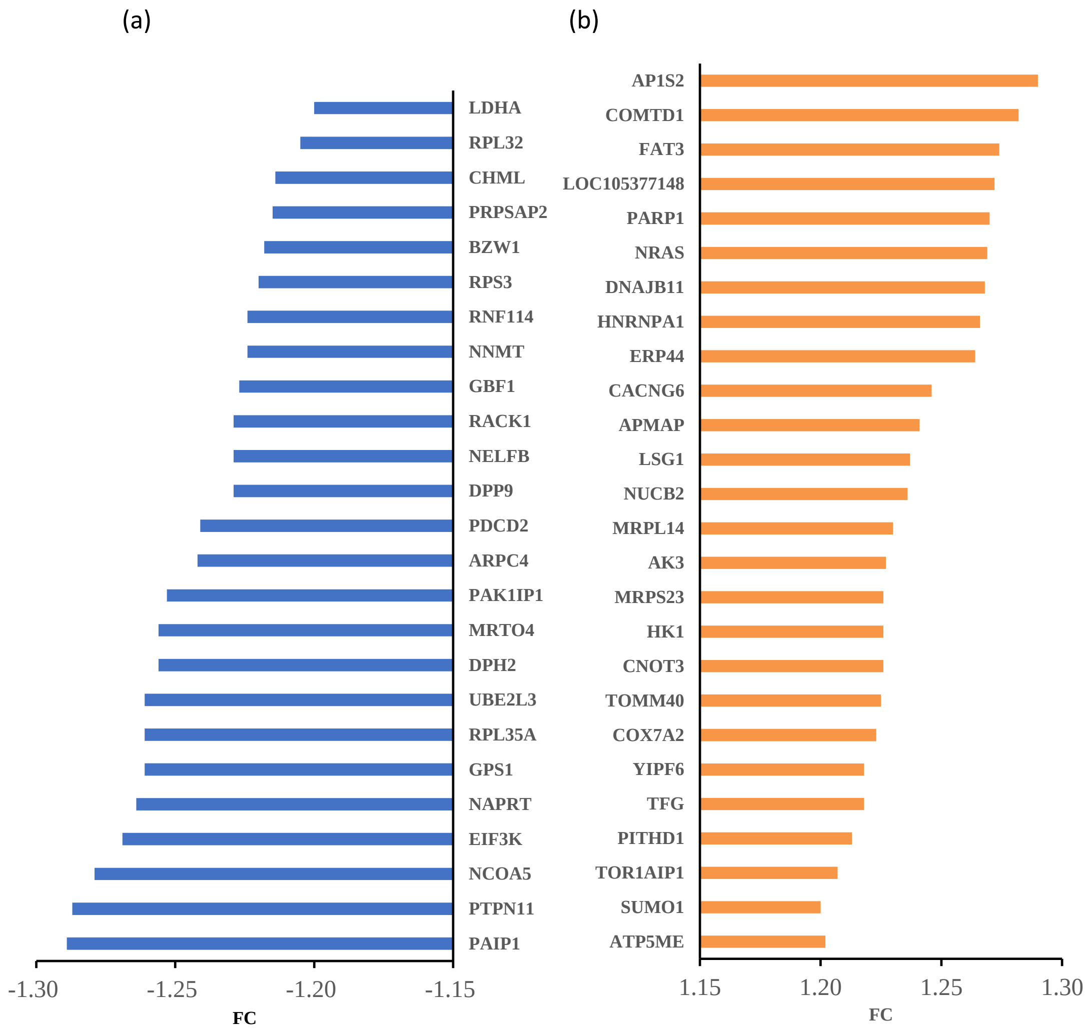

Figure S2. Differentially expressed proteins (DEPs) identified in A549 cells in LZ group with a fold changes (FC) ≤-1.2 and ≥ -1.3 (a) or of ≥ 1.2 and ≤ 1.3 (b), compared to those in A549 cells in LB group.

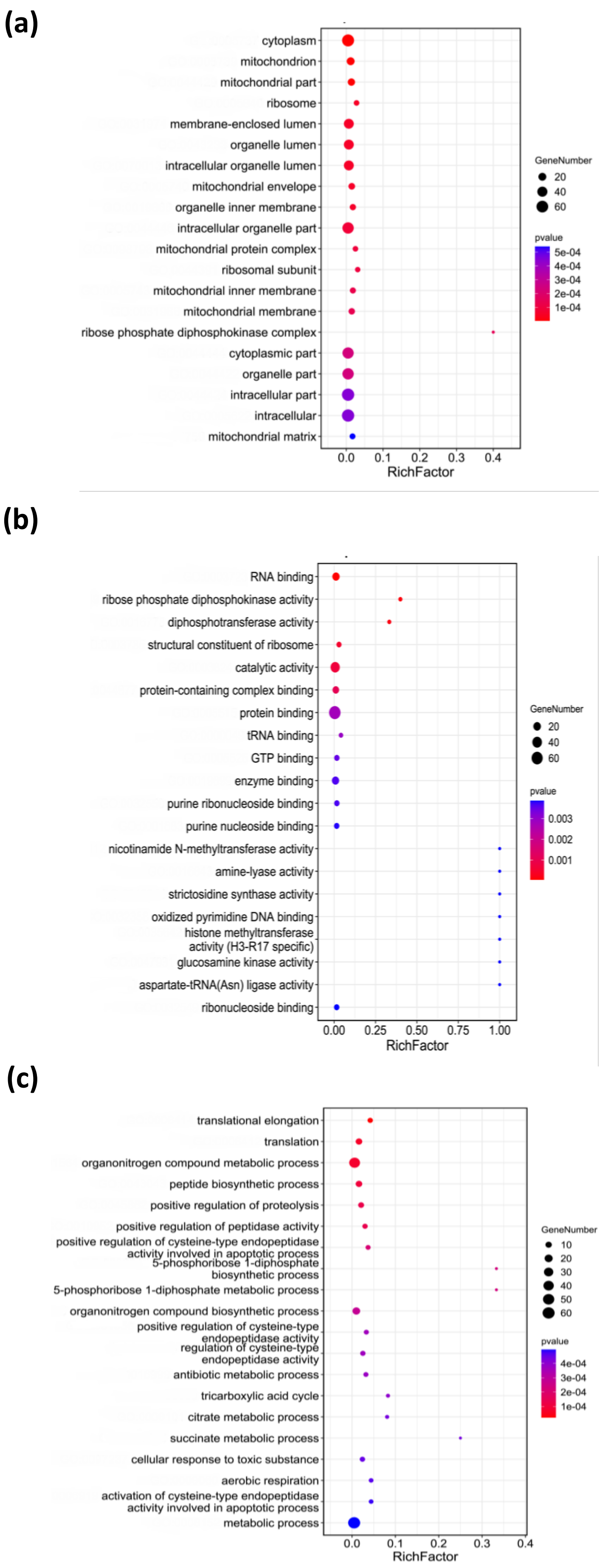

Figure S3. Bioinformatics analysis of the differentially expressed proteins (DEPs) with $|FC| \geq 1.2$ identified in A549 cells exposed to 0.5 μM of ZQX-1 under irradiation at 420 nm. Top 20 GO annotated (a) cellular component, (b) molecular functions and (c) biological process of the DEPs.

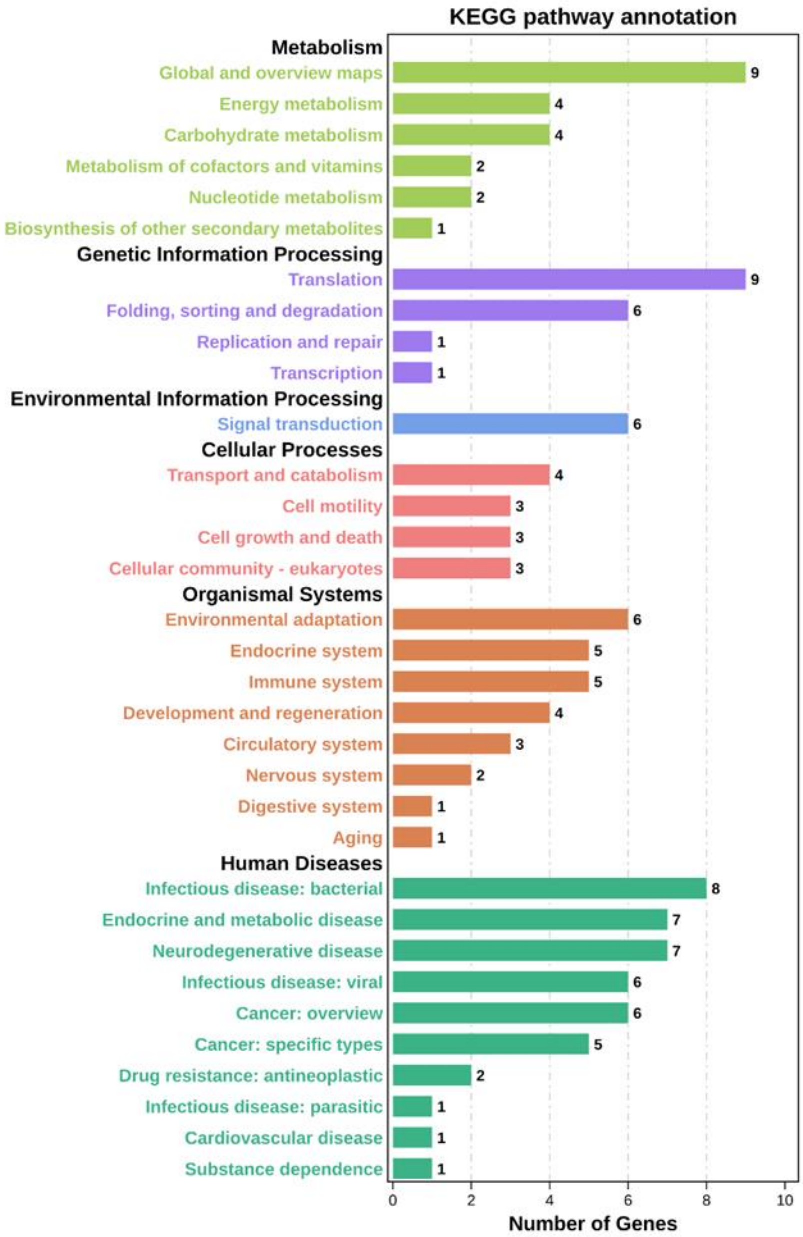

Figure S4. The KEGG pathway annotation of the differentially expressed proteins with |FC| ≥ 1.2 identified in A549 cells treated with 0.5 μM of ZQX-1 under irradiation at 420 nm.

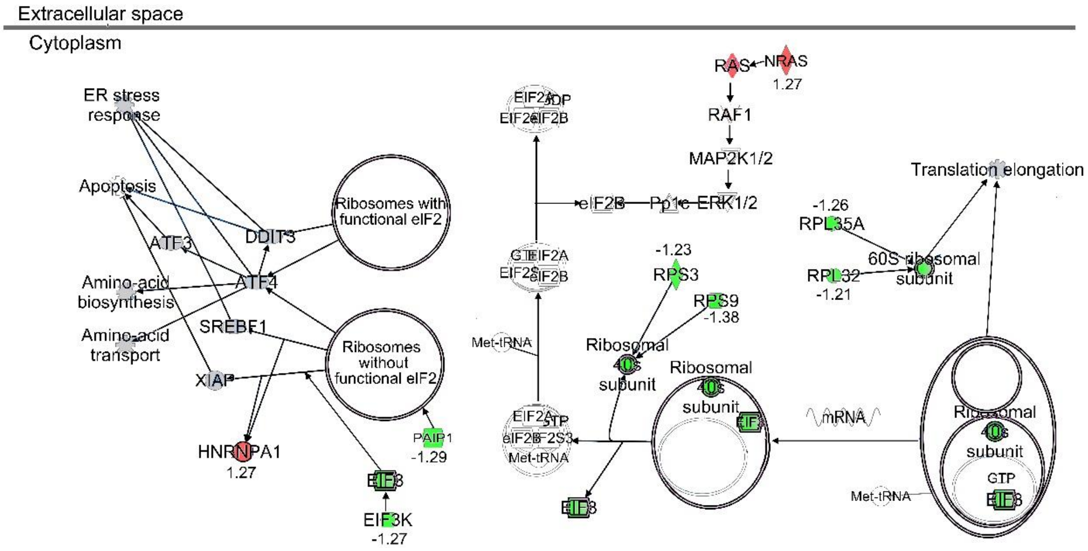

Figure S5. Schematic diagram generated by IPA of EIF2 signaling pathway with which the DEPs with |FC| ≥1.2, identified in A549 cells exposed to 0.5 μM of ZQX-1 under irradiation at 420 nm, are associated. The red color represents the protein (or complex) upregulated, green downregulated.

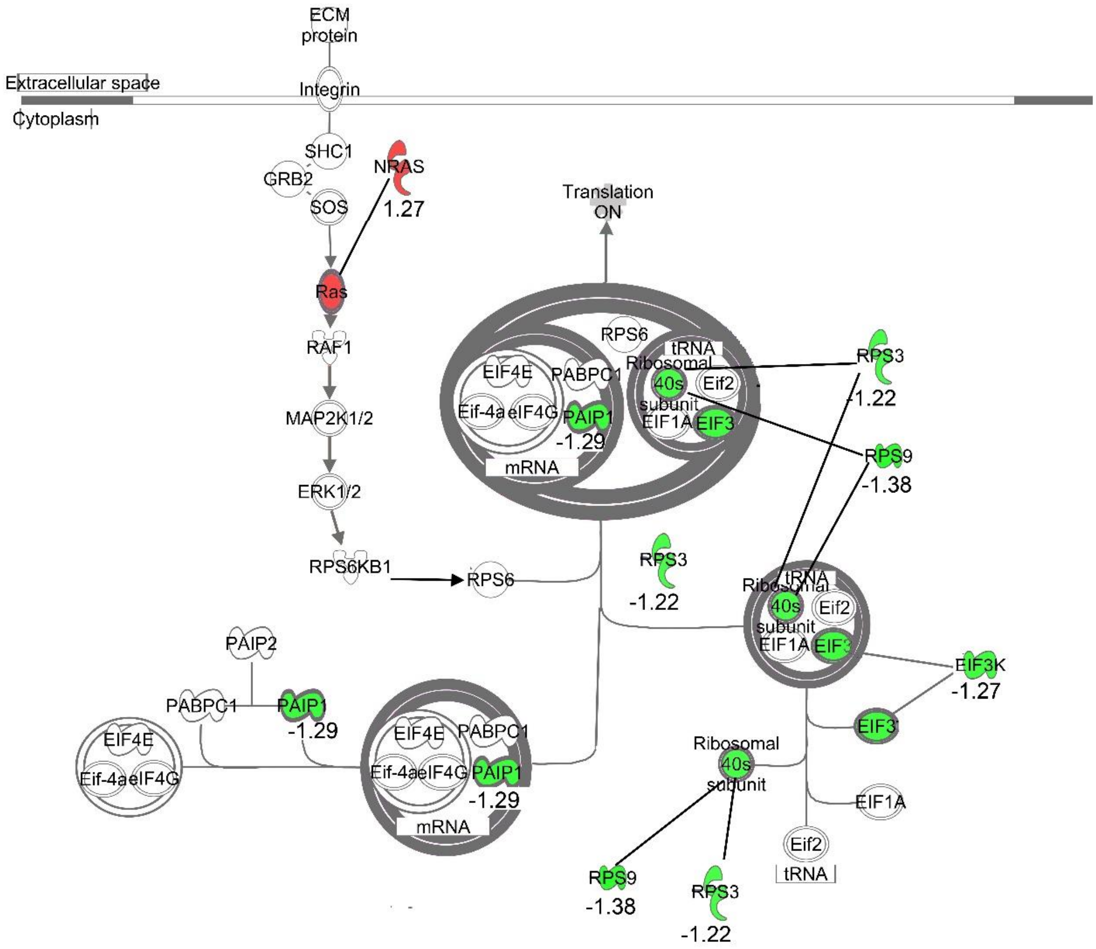

Figure S6. Schematic diagram generated by IPA of elF4 and p70S6K signaling pathway with which the DEPs with |FC| ≥ 1.2, identified in A549 cells exposed to 0.5 μM of ZQX-1 under irradiation at 420 nm, are associated. The red color represents the protein (or complex) upregulated, green downregulated.

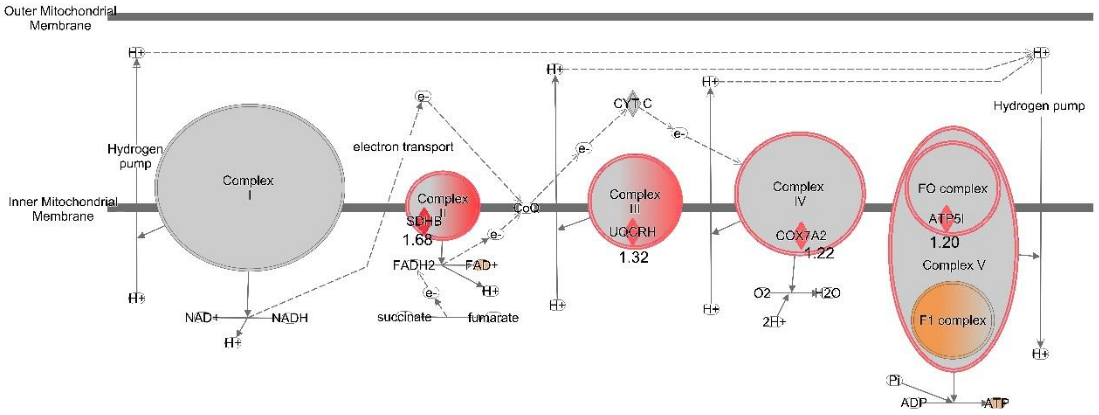

Figure S7. Schematic diagram generated by IPA of oxidative phosphorylation signaling pathway with which the DEPs with |FC| ≥ 1.2, identified in A549 cells exposed to 0.5 μM of ZQX-1 under irradiation at 420 nm, are associated. The red color represents the protein (or complex) upregulated, the orange represents the complex or compound predictively upregulated

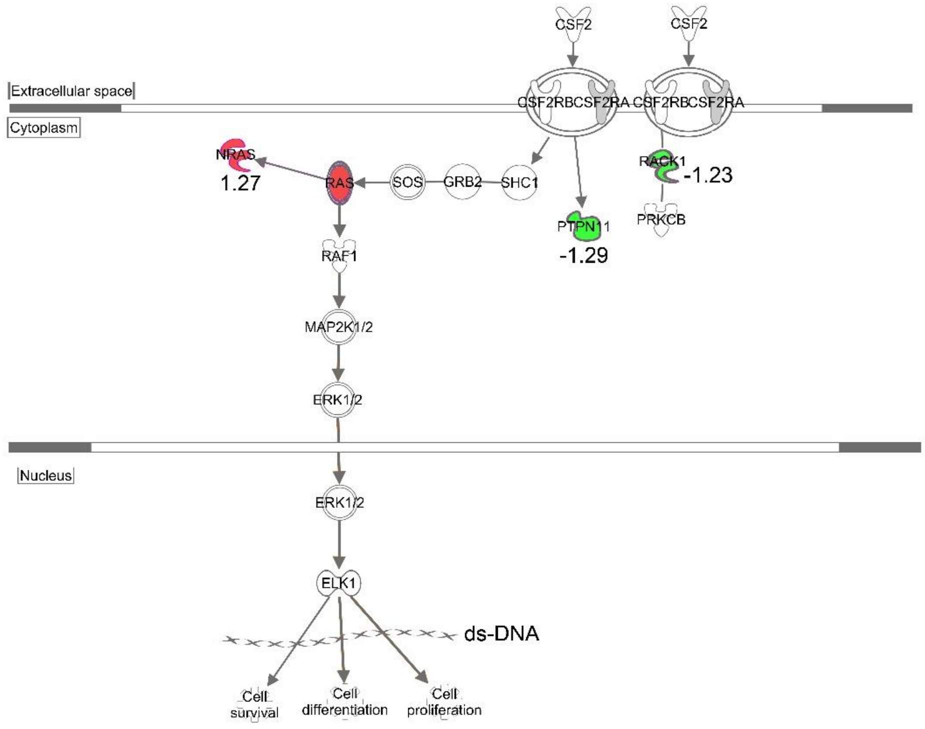

Figure S8. Schematic diagram generated by IPA of granulocyte-macrophage colony-stimulating factor (GM-CSF) signaling pathway with which the DEPs with |FC| ≥ 1.2 identified in A549 cells exposed to 0.5 μM of ZQX-1 under irradiation at 420 nm. The red represents the protein (or complex) upregulated, green downregulated.

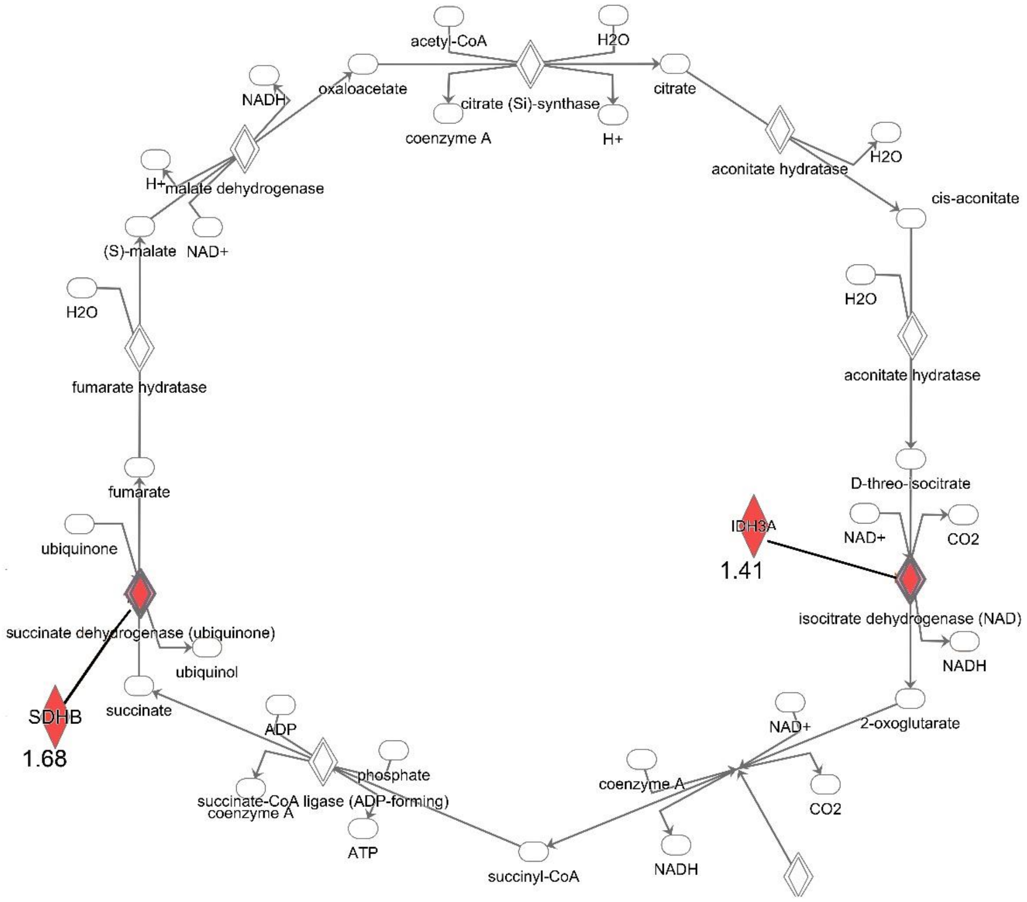

Figure S9. Schematic diagram generated by IPA of TCA cycle II signaling pathway with which the DEPs with |FC|≥1.2, identified in A549 cells exposed to 0.5 μM of ZQX-1 under irradiation at 420 nm, are associated. The red color represents the protein (or complex) upregulated.

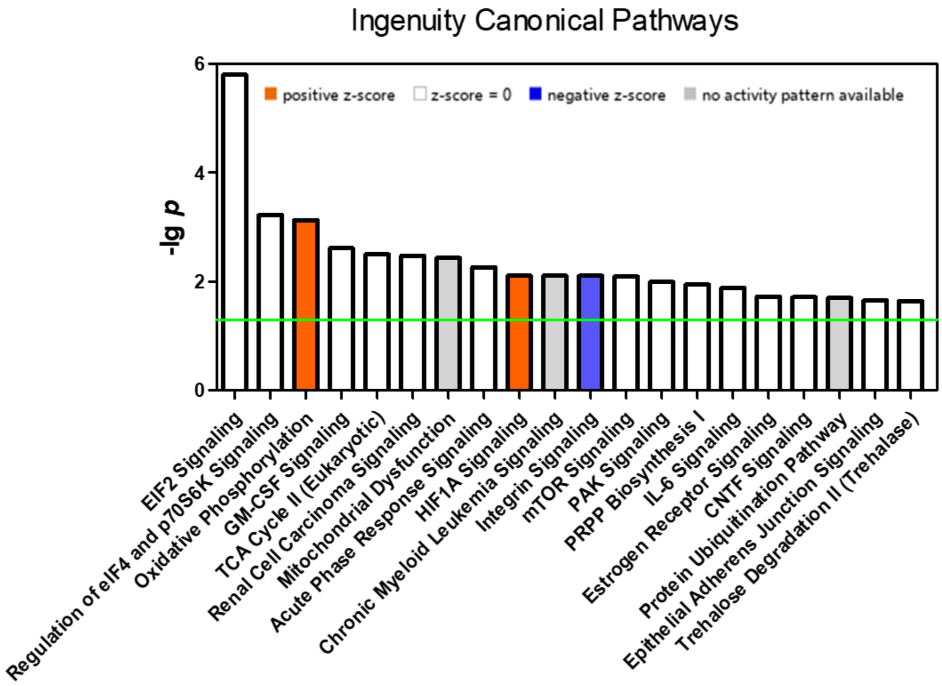

Figure S10. The Top 20 canonical pathways with which the DEPs with |FC| ≥ 1.2, identified in A549 cells exposed to 0.5 μM of ZQX-1 under irradiation at 420 nm, are associated. Green line indicates p ≤ 0.05; Orange represents activation of the pathway, blue inhibition of the pathway.

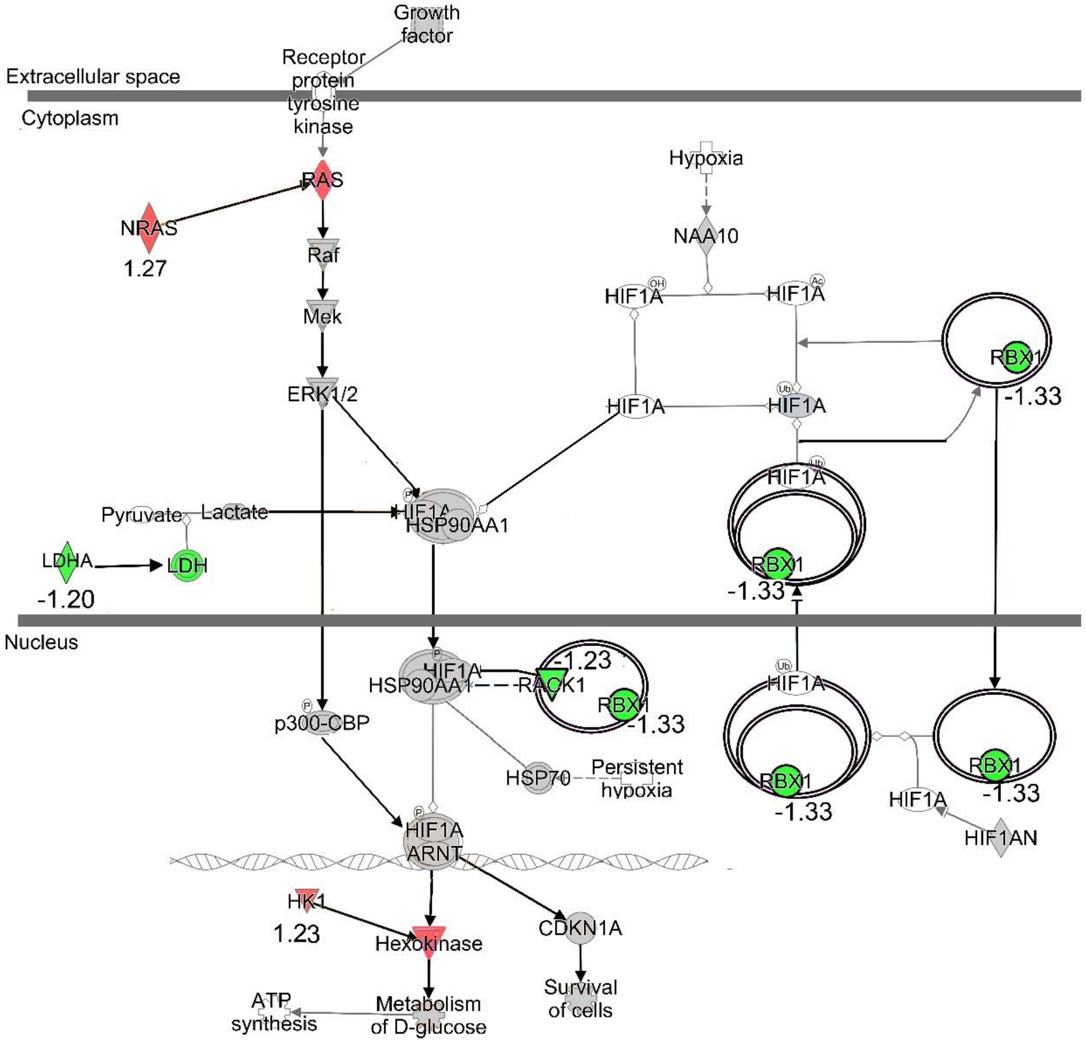

Figure S11. Schematic diagram generated by IPA of HIF1α signaling pathway with which the DEPs with |FC| ≥ 1.2, identified in A549 cells exposed to 0.5 μM of ZQX-1 under irradiation at 420 nm, are associated. The red color represents the protein (or complex) upregulated, green downregulated.

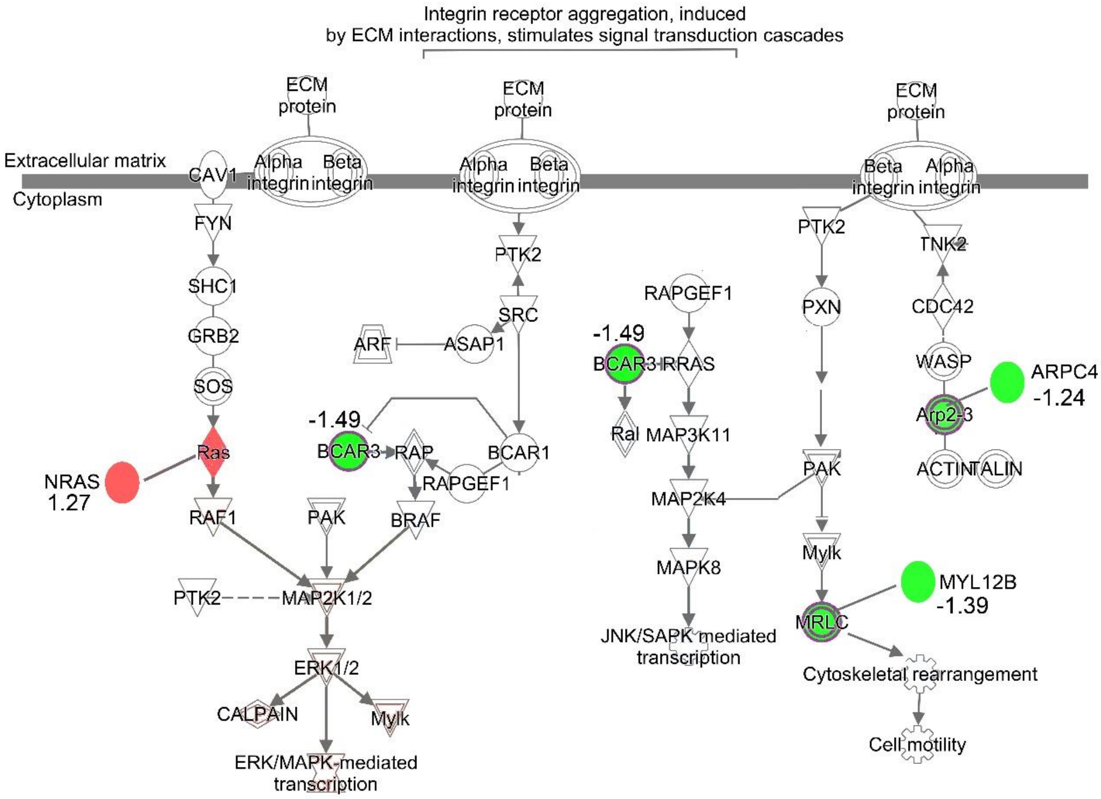

Figure S12. Schematic diagram generated by IPA of integrin signaling with which the DEPs with |FC| ≥ 1.2, identified in A549 cells exposed to 0.5 μM of ZQX-1 under irradiation at 420 nm, are associated. The red color represents the protein (or complex) upregulated, green downregulated.

Table S1. MS/MS raw data for protein identification and quantification in A549 cells.

Checked	FDR	Master	Accession	n	value:	Score	[%]	# Peptides	# PSMs	Peptides	# AAs	MW [kDa]	calc. pI	Sequest	(by Search	Process	Compone	Function	Pfam IDs	Gene ID	Gene ID	Symbol	me	Pathways	ways	Pathways	Pathway	e Ratio:	(Control)	e Ratio	s	s	es	es	Sample:	Sample:	Sample:	Sample:	Sample:	Sample:	Groups	ons
FALSE	High	Master Prc	Q9NYJ1-1	Cytochrom	0	6.264	22	2	3	2	87	10.1	6.04	5.67	2	cell organi:	mitochond		Pf06747	51287	ENSG0000	COA4	11			Mitochonc	2								High	High	High	High	High	High	1	
FALSE	High	Master Prc	O15116	U6 snRNA-	0	7.447	12	1	1	1	133	15.2	5.22	4.47	1	metabolic	cytoplasm;	protein bir	Pf01423	27257	ENSG0000	LSM1	8	RNA degra		mRNA dec	4								High	High	High	High	High	High	1	
FALSE	High	Master Prc	Q9NZN8-1	CCR4-NOT	0.001	4.398	2	1	2	1	540	59.7	7.66	4.84	1	cell differe	cytoplasm;	catalytic a	Pf04153	4848	ENSG0000	CNOT2	12	RNA degra		TP53 regul	10								High	High	High	High	High	High	1	
FALSE	Medium	Master Prc	Q9Y5S2	Serine/thre	0.023	1.533	1	1	1	1	1711	194.2	6.37	1.71	1	cell organi:	cytoplasm;	catalytic a	Pf00038, F	9578	ENSG0000	CDC42BPB	14				0								High	High	High	High	High	High	1	
FALSE	High	Master Prc	Q9UPU5	Ubiquitin c	0	14.633	1	3	4	3	2620	294.2	6.14	3.99	3	metabolic		catalytic a	Pf00443, F	23358	ENSG0000	USP24	1			Ub-specific	4								High	High	High	High	High	High	1	
FALSE	High	Master Prc	Q5HYK3-1	2-methoxy	0.002	3.584	4	1	1	1	327	37.1	6.95	0	1	metabolic	membrane	catalytic a	Pf01170, F	84274	ENSG0000	COQ5	12	Ubiquinon		Ubiquinol	6								High	High	High	High	High	High	1	
FALSE	High	Master Prc	Q9ULV3	Cip1-intera	0.003	2.849	1	1	1	1	898	100	6.11	1.83	1		membrane	metal ion I	Pf12171	25792	ENSG0000	CIZ1	9				0								High	High	High	High	High	High	1	
FALSE	High	Master Prc	P52758	2-iminobut	0.002	3.548	12	1	2	1	137	14.5	8.68	4.15	1	metabolic	cytoplasm;	catalytic a	Pf01042	10247	ENSG0000	HRSP12; R	8				0								High	High	High	High	High	High	1	
FALSE	High	Master Prc	Q8TA86	retinitis pi	0.004	2.771	4	1	1	1	221	26.1	9.79	1.74	1	metabolic	cytosol;nu	metal ion I		6100	ENSG0000	RP9	7	Spliceosom			1								High	High	High	High	High	High	1	
FALSE	Medium	Master Prc	Q9UHN6-1	Cell surface	0.01	2.049	1	1	1	1	1383	154.3	8.15	0	1	developme	membrane	catalytic a	Pf10162	23670	ENSG0000	TMEM2	9				0								High	High	High	High	High	High	1	
FALSE	High	Master Prc	Q13243-1	Serine/argi	0	29.684	24	6	10	5	272	31.2	11.59	20	6	cell organi:	cytosol;nu	protein bir	Pf00076, F	6430	ENSG0000	SRSF5	14	Herpes sim	mRNA Pro	Cleavage o	14								High	High	High	High	High	High	1	
FALSE	High	Master Prc	P02649	Apolipopro	0.004	2.643	3	1	1	1	317	36.1	5.73	1.82	1	cell commu	cytoplasm;	antioxidan	Pf01442	348	ENSG0000	APOE	19	Alzheimer'	Vitamin B1	Post-transl	33								High	High	High	High	High	High	1	
FALSE	High	Master Prc	Q8WVM0	Dimethyla	0	8.421	10	3	3	3	346	39.5	9.26	2.25	3	cell organi:	mitochond	catalytic a	Pf00398	51106	ENSG0000	TFB1M	6		Energy Me	Transcripti	9								High	High	High	High	High	High	1	
FALSE	High	Master Prc	Q9UI09	NADH deh	0	13.186	25	3	3	3	145	17.1	9.63	4.88	3	cell organi:	cytosol;me	catalytic a	Pf05071	55967	ENSG0000	NDUFA12	12	Parkinson's	Electron Tr	Complex I	12								High	High	High	High	High	High	1	
FALSE	High	Master Prc	Q9NUQ7	Ufm1-spec	0.005	2.554	2	1	1	1	469	53.2	7.01	2.96	1	metabolic	cytoplasm;	catalytic a	Pf07910	55325	ENSG0000	UFSP2	4				0								High	High	High	High	High	High	1	
FALSE	Medium	Master Prc	Q9NVV5	Androgen-	0.032	1.394	4	1	1	1	245	28.2	8.25	0	1		membrane	protein bir	Pf04750	51390	ENSG0000	AIG1	6				0								High	High	High	High	High	High	1	[Q75]
FALSE	Medium	Master Prc	Q5T0F9-1	Coiled-coil	0.01	2.056	1	1	1	1	858	94.2	5.26	0	1	metabolic	nucleus	DNA bindi	Pf00168	200014	ENSG0000	CC2D1B	1				0								High	High	High	High	High	High	1	
FALSE	High	Master Prc	P83436	Conserved	0.004	2.786	1	1	2	1	770	86.3	5.47	2.09	1	cell organi:	Golgi;mem	protein bir	Pf10191	91949	ENSG0000	COG7	16			COPI-medi	11								High	High	High	High	High	High	1	
FALSE	High	Master Prc	Q92783	signal tran	0	6.245	4	2	3	2	540	59.1	4.82	2.12	2	cell commu	cytoplasm;	protein bir	Pf00018, F	8027	ENSG0000	STAM	10	Endocytosi	VEGFA-VE(	Cargo reco	23								High	High	High	High	High	High	1	
FALSE	Medium	Master Prc	Q9NXF1	Testis-expr	0.016	1.703	2	1	1	1	929	105.6	9.36	2.09	1	metabolic	cytoplasm;	protein bir	Pf12333	54881	ENSG0000	TEX10	9			Major path	4								High	High	High	High	High	High	1	
FALSE	Low	Master Prc	O00423-1	Echinoderr	0.051	1.18	2	1	1	1	815	89.8	7.06	2.26	1	cell differe	cytoplasm;	metal ion I	Pf00400, F	2009	ENSG0000	EML1	14				0								High	High	High	High	High	High	1	[T285(100
FALSE	High	Master Prc	Q9H1C4	Protein un	0	5.952	3	1	1	1	597	66.6	6.96	1.93	1	defense re	endoplasm	protein bir	Pf05978	81622	ENSG0000	UNC93B1	11			Trafficking	8								High	High	High	High	High	High	1	
FALSE	High	Master Prc	Q9UBB9-1	Tuftelin-in	0	8.964	3	2	2	2	837	96.8	5.67	5.93	2	cell organi:	cytoplasm;	DNA bindi	Pf01585, F	24144	ENSG0000	TFIP11	22			mRNA Spli	4								High	High	High	High	High	High	1	
FALSE	High	Master Prc	Q7Z422	SUZ domai	0	7.333	19	2	2	2	152	17	8.95	4.7	2				Pf12752, F	26099	ENSG0000	SZRD1	1				0								High	High	High	High	High	High	1	
FALSE	High	Master Prc	Q6P1X6	UPF0598 p	0	5.981	7	1	1	1	216	23.9	9.14	3.81	1				Pf14956	414919	ENSG0000	C8orf82	8				0								High	High	High	High	High	High	1	
FALSE	High	Master Prc	Q9Y5J5	Pleckstrin l	0	4.571	8	1	1	1	127	13.9	9.67	1.9	1	cell death;	cytoplasm;		Pf00169	23612	ENSG0000	PHLDA3	1				0								High	High	High	High	High	High	1	
FALSE	Medium	Master Prc	Q9UH17-1	DNA dC->c	0.016	1.708	2	1	1	1	382	45.9	6.06	1.85	1	defense re	nucleus	catalytic a	Pf05240, F	9582	ENSG0000	APOBEC3B	22			Formation	4								High	High	High	High	High	High	1	
FALSE	High	Master Prc	O75976	Carboxype	0	4.865	2	2	2	2	1380	152.8	6.05	2.15	2	metabolic	membrane	catalytic a	Pf00246, F	1362	ENSG0000	CPD	17			Golgi Asso	5								High	High	High	High	High	High	1	
FALSE	Medium	Master Prc	P04156-1	Major prio	0.017	1.679	3	1	3	1	253	27.6	9	0	1	cell organi:	cell surface	metal ion I	Pf00377, F	5621	ENSG0000	PRNP	20	Prion disea	Copper ho	NCAM1 int	6								High	High	High	High	High	High	1	
FALSE	Low	Master Prc	Q5THR3	EF-hand ca	0.071	1.035	1	1	1	1	1501	172.8	8.4	0	1	metabolic	nucleus	metal ion I	Pf08976, F	64800	ENSG0000	EFCAB6	22		Androgen		1								High	High	High	High	High	High	1	[K1083];
FALSE	High	Master Prc	Q5W0V3	Protein FA	0.003	3.448	3	1	1	1	765	86.5	5.29	2.22	1				Pf10257	57700	ENSG0000	FAM160B1	10				0								High	High	High	High	High	High	1	
FALSE	Medium	Master Prc	Q96GX9-1	Methylthic	0.029	1.436	5	1	1	1	242	27.1	7.12	0	1	cell death;	cytoplasm	catalytic a	Pf00596	51074	ENSG0000	APIP	11	Metabolic		Methionin	7								High	High	High	High	High	High	1	
FALSE	High	Master Prc	P31483	nucleolysir	0	9.99	8	3	4	2	386	42.9	7.74	0	3	cell death;	cytoplasm;	protein bir	Pf00076, F	7072	ENSG0000	TIA1	2			FGFR2 alte	5								High	High	High	High	High	High	1	
FALSE	Medium	Master Prc	Q9Y5Q9	General tra	0.02	1.609	1	1	1	1	886	101.2	5.07	0	1	metabolic	membrane	DNA bindi	Pf13414, F	9330	ENSG0000	GTF3C3	2			RNA Polym	6								High	High	High	High	High	High	1	
FALSE	High	Master Prc	Q9Y223-1	bifunctiona	0	22.506	8	4	5	4	722	79.2	6.8	15.12	4	metabolic	cytoplasm;	catalytic a	Pf00370, F	10020	ENSG0000	GNE	9	Metabolic		Sialic acid	12								High	High	High	High	High	High	1	
FALSE	High	Master Prc	P09132-1	Signal reco	0	5.676	8	1	1	1	144	16.1	9.85	3.29	1	cell organi:	cytoplasm;	protein bir	Pf01922	6728	ENSG0000	SRP19	5	Protein ex		SRP-depen	4								High	High	High	High	High	High	1	
FALSE	High	Master Prc	P60983	glia matura	0	9.374	26	2	2	2	142	16.7	5.29	2.9	2	metabolic		enzyme re	Pf00241	2764	ENSG0000	GMFB	14				0								High	High	High	High	High	High	1	
FALSE	High	Master Prc	P14384	Carboxype	0.002	3.886	2	1	1	1	443	50.5	7.36	0	1	metabolic	cell surface	catalytic a	Pf00246, F	1368	ENSG0000	CPM	12			Post-transl	3								High	High	High	High	High	High	1	
FALSE	Low	Master Prc	Q8N4U5	T-complex	0.062	1.107	1	1	1	1	519	58.1	4.96	0	1				Pf05794	255394	ENSG0000	TCP11L2	12				0								High	High	High	High	High	High	1	
FALSE	High	Master Prc	Q5T6F2	Ubiquitin-a	0.002	3.883	1	1	1	1	1119	117	7.34	1.68	1	regulation	cytoplasm;	protein bir	Pf00627, F	55833	ENSG0000	UBAP2	9				0								High	High	High	High	High	High	1	
FALSE	High	Master Prc	Q00613	heat shock	0.003	3.237	2	1	1	1	529	57.2	5.19	2.04	1	cell organi:	chromosor	DNA bindi	Pf00447, F	3297	ENSG0000	HSF1	8	Legionello:		Attenuatio	8								High	High	High	High	High	High	1	
FALSE	High	Master Prc	Q96NA2-1	Rab-intera	0.001	4.502	3	1	1	1	401	44.2	5.59	0	1	cell organi:	cytosol;en	protein bir	Pf09744, F	83547	ENSG0000	RILP	17; CHR_H	Salmonella		MHC class	5								High	High	High	High	High	High	1	
FALSE	Medium	Master Prc	Q13642-1	Isoform 1 c	0.013	1.865	5	1	1	1	280	31.9	8.32	0	1	cell differe	cytoplasm;	metal ion I	Pf00412	2273	ENSG0000	FHL1	X		Notch Sign		1								High	High	High	High	High	High	1	
FALSE	High	Master Prc	P57105	Synaptojar	0.001	4.507	11	1	1	1	145	15.9	6.3	0	1	regulation	membrane	protein bir	Pf00595, F	55333	ENSG0000	SYNJ2BP	14				0								High	High	High	High	High	High	1	
FALSE	High	Master Prc	O15091	Mitochonc	0	6.219	3	1	1	1	583	67.3	8.78	3.52	1	metabolic	mitochond	catalytic a		9692	ENSG0000	KIAA0391	14			tRNA proc	5								High	High	High	High	High	High	1	
FALSE	High	Master Prc	Q3SY69-1	Mitochonc	0	4.659	2	1	1	1	923	101.7	6.52	3.24	1	metabolic	cytoplasm;	catalytic a	Pf00171, F	160428	ENSG0000	ALDH1L2	12	One carbo		Metabolisr	5								High	High	High	High	High	High	1	
FALSE	Medium	Master Prc	O95104	Splicing fac	0.01	2.077	1	1	1	1	1147	125.8	9.55	1.84	1		nucleus	RNA bindi	Pf00076, F	57466	ENSG0000	SCAF4	21				0								High	High	High	High	High	High	1	
FALSE	Low	Master Prc	P50542-1	Peroxisom	0.059	1.127	3	1	1	1	639	70.8	4.54	0	1	cell organi:	cytoplasm;	protein bir	Pf00515, F	5830	ENSG0000	PEX5	12	Peroxisom		E3 ubiquiti	5								High	High	High	High	High	High	1	
FALSE	High	Master Prc	P31751	RAC-beta s	0	4.637	2	1	1	1	481	55.7	6.37	0	1	cell death;	cytoplasm;	catalytic a	Pf00069, F	208	ENSG0000	AKT2	19	T cell recep	AMP-activ	Regulation	170								High	High	High	High	High	High	1	
FALSE	High	Master Prc	Q14103-4	Isoform 4 c	0	140.22	59	21	64	1	287	30.7	8.41	143.38	21	metabolic	cytosol;nu	DNA bindi	Pf00076, F	3184		HNRNPD	4		mRNA Pro	AUF1 (hnR	4								High	High	High	High	High	High	1	[Q249;
FALSE	High	Master Prc	A6NKD9	Coiled-coil	0.004	2.804	3	1	1	1	419	45.2	6.96	2.21	1	developme			Pf10226	317762	ENSG0000	CCDC85C	14				0								High	High	High	High	High	High	1	
FALSE	High	Master Prc	Q92733	proline-ricl	0	5.824	3	1	1	1	491	52.4	5.1	3.38	1	metabolic	nucleus	protein bir	Pf10253	5546	ENSG0000	PRCC	1	Transcripti		mRNA Spli	5								High	High	High	High	High	High	1	
FALSE	High	Master Prc	Q6P1M3-1	Lethal(2) g	0.007	2.249	1	1	1	1	1020	113.4	7.52	0	1	cell divisio	cytoplasm;	enzyme re	Pf08366	3993	ENSG0000	LLGL2	17	Tight junct			2								High	High	High	High	High	High	1	
FALSE	Medium	Master Prc	Q8WYQ3	Coiled-coil	0.022	1.591	6	1	1	1	142	14.1	7.84	0	1	cell organi:	mitochond		Pf06747	400916	ENSG0000	CHCHD10	22; CHR_H			Mitochonc	2								High	High	High	High	High	High	1	
FALSE	High	Master Prc	O75934	Pre-mRNA	0	21.933	31	5	6	5	225	26.1	5.66	10.38	5	cell organi:	nucleus;sp	protein bir	Pf05700	10286	ENSG0000	BCAS2	1	Spliceosom		mRNA Spli	5								High	High	High	High	High	High	1	
FALSE	Low	Master Prc	O14638	Ectonuclec	0.057	1.142	2	1	1	1	875	100.1	6.57	2.52	1	metabolic	extracellul	catalytic a	Pf01033, F	5169	ENSG0000	ENPP3	6	Nicotinate	IL-3 Signali	Vitamin B5	11								High	High	High	High	High	High	1	[K504;
FALSE	Medium	Master Prc	Q96RL6	Sialic acid-	0.013	1.931	2	1	3	1	698	75.7	7.5	0	1		membrane	protein bir	Pf00047, F	114132	ENSG0000	SIGLEC11	19			Immunore	3								High	High	High	High	High	High	1	
FALSE	High	Master Prc	Q96JJ7-1	Protein dis	0	8.58	6	3	4	3	454	51.8	4.91	3.15	3	cellular ho	cell surface	catalytic a	Pf00085, F	54495	ENSG0000	TMX3	18			Platelet de	4								High	High	High	High	High	High	1	
FALSE	High	Master Prc	Q9Y5B8	Nucleoside	0.002	3.652	5	2	2	2	376	42.5	6.47	0	2	metabolic	cytosol	catalytic a	Pf00334	29922	ENSG0000	NME7	1	Pyrimidine		Interconve	6								High	High	High	High	High	High	1	
FALSE	Low	Master Prc	Q13464	Rho-associ	0.054	1.16	1	1	1	1	1354	158.1	5.9	0	1	cell death;	cytoplasm;	catalytic a	Pf00069, F	6093	ENSG0000	ROCK1	18	Chemokine	Androgen	EPHA-med	57								High	High	High	High	High	High	1	
FALSE	High	Master Prc	P42574	Caspase-3	0.004	2.712	4	1	1	1	277	31.6	6.54	0	1	cell death;	cytoplasm;	catalytic a	Pf00656	836	ENSG0000	CASP3	4	Pathways i	miRNA Re	Ligand-ind	83								High	High	High	High	High	High	1	
FALSE	High	Master Prc	Q9BU23-1	Lipase mat	0	4.676	3	1	1	1	707	79.6	10.1	4.3	1	metabolic	endoplasm	protein bir	Pf06762	91289	ENSG0000	LMF2	22			Assembly c	4								High	High	High	High	High	High	1	
FALSE	High	Master Prc	Q9Y4G6	Talin-2 [OS	0	17.894	1	4	8	0	2542	271.4	5.57	15.35	4	cell organi:	cytoplasm;	protein bir	Pf00373, F	83660	ENSG0000	TLN2	15	Platelet ac			4								High	High	High	High	High	High	1	
FALSE	High	Master Prc	Q9NWV8	BRISC and	0	5.856	3	1	3	1	329	36.5	4.64	5.69	1	cell divisio	cytoplasm;	protein bir		29086	ENSG0000	BABAM1	19	Homologo		G2/M DNA	17								High	High	High	High	High	High	1	
FALSE	Medium	Master Prc	Q9H0X4	Protein FA	0.014	1.805	2	1	1	1	552	59.6	6.28	1.63	1		cell surface			83986	ENSG0000	ITFG3; FAN	16				0								High	High	High	High	High	High	1	
FALSE	High	Master Prc	Q8IXQ5	kelch-like p	0.008	2.19	2	1	1	1	586	65.9	6.48	0	1	metabolic	cytoplasm;	catalytic a	Pf00651, F	55975	ENSG0000	KLHL7	7				0								High	High	High	High	High	High	1	
FALSE	High	Master Prc	Q86U28	iron-sulfur	0.002	4.145	8	1	1	1	154	16.5	5.25	1.6	1	cell organi:	mitochond	metal ion I	Pf01521	122961	ENSG0000	ISCA2	14			Mitochonc	2								High	High	High	High	High	High	1	
FALSE	High	Master Prc	P04920-1	Anion exch	0	9.795	3	3	3	3	1241	136.9	6.29	2.57	3	cellular ho	membrane	protein bir	Pf00955, F	6522	ENSG0000	SLC4A2	7	Gastric aci		Bicarbonat	8								High	High	High	High	High	High	1	
FALSE	Medium	Master Prc	Q9BRT3	migration a	0.033	1.383	8	1	1	1	115	12.4	4.37	2.22	1	cell death;	cytoplasm;	protein bir	Pf10262	84299	ENSG0000	MIEN1	17				0								High	High	High	High	High	High	1	
FALSE	High	Master Prc	Q8NGA1	Olfactory r	0.008	2.219	2	1	2	1	313	34.8	8.91	0	1	regulation	membrane	receptor a	Pf00001, F	125963	ENSG0000	OR1M1	19	Olfactory t		Olfactory S	5								High	High	High	High	High	High	1	
FALSE	High	Master Prc	Q460N5-1	Isoform 1 c	0.006	2.378	1	1	1	1	1720	193.6	7.97	0	1	metabolic	cytoplasm;	catalytic a	Pf00644, F	54625		PARP14	3				0								High	High	High	High	High	High	1	
FALSE	High	Master Prc	P10398-1	serine/thre	0	5.353	2	1	1	1	606	67.5	9.01	0	1	cell differe	cytosol;mi	catalytic a	Pf00069, F	369	ENSG0000	ARAF	X	Insulin sigr	Integrin-m	Paradoxica	54								High	High	High	High	High	High	1	
FALSE	High	Master Prc	Q9UHL4	Dipeptidyl	0	9.195	5	2	2	2	492	54.3	6.32	4.12	2	metabolic	cytosol;ext	catalytic a	Pf00561, F	29952	ENSG0000	DPP7	9			Neutrophil	3								High	High	High	High	High	High	1	
FALSE	Medium	Master Prc	P12109	Collagen al	0.015	1.746	1	1	1	1	1028	108.5	5.43	0	1	cell differe	extracellul	protein bir	Pf00092, F	1291	ENSG0000	COL6A1	21	ECM-recep	miRNA tar	ECM prote	21								High	High	High	High	High	High	1	
FALSE	High	Master Prc	P49354	protein far	0.001	4.499	3	1	1	1	379	44.4	5.08	3.89	1	cell organi:	cytoplasm;	catalytic a	Pf01239	2339	ENSG0000	FNTA	8	Terpenoid		Signaling b	10								High	High	High	High	High	High	1	
FALSE	High	Master Prc	Q2NKX8	Dna excisic	0.006	2.479	1	1	1	1	1250	141	5.31	0	1	cell divisio	chromosor	catalytic a	Pf00176, F	54821	ENSG0000	ERCC6L	X			Amplificati	16								High	High	High	High	High	High	1	

FALSE	Low	Master Prc	Q14C87	Transmem	0.054	1.163	1	1	1	1	1099	122.2	5.73	0	1	regulation	membrane	protein bir		121256	ENSG0000	TMEM132	12				0	High	High	High	High	High	High	1	
FALSE	Medium	Master Prc	Q14061	cytochrom	0.012	1.971	11	1	1	1	63	6.9	7.24	0	1	cell organi:	cytoplasm;	metal ion l	Pf05051	10063	ENSG0000	COX17	3	Metabolic	Electron Tı	Mitochonc	6	High	High	High	High	High	High	1	
FALSE	High	Master Prc	P28062-1	Proteasom	0.006	2.381	5	1	2	1	276	30.3	7.43	2.46	1	cell differe	cytoplasm;	catalytic a	Pf00227	5696	ENSG0000	PSMB8	6; CHR_HS	Proteasom	Proteasom	Regulation	152	High	High	High	High	High	High	1	
FALSE	High	Master Prc	Q96P16-1	Regulation	0.003	3.3	5	1	1	1	312	35.7	7.55	3.65	1	metabolic		protein bir	Pf04818	55197	ENSG0000	RPRD1A	18			RNA polym	3	High	High	High	High	High	High	1	
FALSE	High	Master Prc	Q9Y385	ubiquitin-c	0	4.657	3	1	2	1	318	35.2	6.74	0	1	metabolic	cytoplasm;	catalytic a	Pf00179	51465	ENSG0000	UBE2J1	6	Ubiquitin r	Parkin-Ubi	Antigen pr	9	High	High	High	High	High	High	1	
FALSE	High	Master Prc	P30154	Serine/thre	0	31.121	13	8	11	1	601	66.2	4.94	29.02	8	cell death;	membrane	protein bir	Pf13513, P	5519	ENSG0000	PPP2R1B	11	Oocyte me	Focal Adhe	Glycolysis;	125	High	High	High	High	High	High	1	
FALSE	High	Master Prc	Q9UQ13	Leucine-ric	0.002	3.915	2	1	1	1	582	64.8	8.46	3.65	1	regulation	cytoplasm;	enzyme re	Pf12799, P	8036	ENSG0000	SHOC2	10	Ras signali			1	High	High	High	High	High	High	1	
FALSE	High	Master Prc	Q8IUH4	Palmitoylt	0	5.717	3	1	1	1	622	70.8	8.07	4.29	1	regulation	endoplasm	catalytic a	Pf00023, P	54503	ENSG0000	ZDHHC13	11				0	High	High	High	High	High	High	1	
FALSE	Medium	Master Prc	Q8N567	Zinc finger	0.018	1.66	4	1	1	1	271	30.5	9.17	0	1	metabolic	nucleus	metal ion l		84240	ENSG0000	ZCCHC9	5				0	High	High	High	High	High	High	1	
FALSE	High	Master Prc	Q15653-1	NF-kappa-l	0.002	3.901	4	1	1	1	356	37.7	4.84	0	1	metabolic	cytoplasm;	protein bir	Pf00023, P	4793	ENSG0000	NFKBIB	19; CHR_H	NOD-like r	IL-1 signali	IKBKG defi	60	High	High	High	High	High	High	1	
FALSE	High	Master Prc	P07951-2	Isoform 2 c	0	61.438	37	15	32	3	284	33	4.67	51.94	15	cell organi:	cytoskelet	protein bir	Pf00261, P	7169	ENSG0000	TPM2	9		Striated M		1	High	High	High	High	High	High	1	
FALSE	High	Master Prc	Q8N5A5-1	Zinc finger	0	5.705	3	1	1	1	531	57.3	5.43	2.16	1	metabolic	membrane	DNA bindi	Pf00642, P	84619	ENSG0000	ZGPAT	20				0	High	High	High	High	High	High	1	
FALSE	High	Master Prc	Q9UPR0-1	inactive ph	0.003	2.964	2	1	1	1	1127	125.8	6.9	1.66	1	cell differe	cytoplasm	catalytic a	Pf00168, P	23228	ENSG0000	PLCL2	3; CHR_HG				0	High	High	High	High	High	High	1	
FALSE	Low	Master Prc	O76038	secretagog	0.071	1.036	2	1	1	1	276	32	5.41	0	1	cellular ho	cytoplasm;	metal ion l	Pf13405, P	10590	ENSG0000	SCGN	6				0	High	High	High	High	High	High	1	
FALSE	High	Master Prc	Q9Y6D5	Brefeldin A	0	6.813	1	2	2	2	1785	201.9	6.33	4.44	2	cell organi:	cytoplasm;	protein bir	Pf01369, P	10564	ENSG0000	ARFGEF2	20	Endocytosi		Associatio	5	High	High	High	High	High	High	1	
FALSE	High	Master Prc	Q9P0T7	Transmem	0.003	2.946	8	2	3	2	183	20.6	6.65	0	2	transport	endosome		Pf05434	252839	ENSG0000	TMEM9	1				0	High	High	High	High	High	High	1	
FALSE	High	Master Prc	Q92930	Ras-relatec	0	8.444	15	4	5	1	207	23.6	9.07	2.22	4	cell organi:	endosome	catalytic a	Pf00025, P	51762	ENSG0000	RAB8B	15		Ectoderm I	TBC/RABG	9	High	High	High	High	High	High	1	
FALSE	Medium	Master Prc	Q49B96	Cytochrom	0.018	1.653	11	1	1	1	90	10.4	8.72	2.16	1	cell organi:	cytoplasm;		Pf06747	90639	ENSG0000	COX19	7			Mitochonc	2	High	High	High	High	High	High	1	
FALSE	Medium	Master Prc	Q96B54	Zinc finger	0.017	1.668	7	1	1	1	188	20.5	4.17	0	1			metal ion l		126299	ENSG0000	ZNF428	19				0	High	High	High	High	High	High	1	
FALSE	Medium	Master Prc	Q4G0F5	Vacuolar p	0.02	1.608	4	1	1	1	336	39.1	7.36	1.7	1	regulation	cytoplasm;	protein bir	Pf03643	112936	ENSG0000	VPS26B	11	Endocytosi			1	High	High	High	High	High	High	1	
FALSE	High	Master Prc	O95749	geranylger	0	5.45	6	2	2	2	300	34.8	6.14	2.41	2	metabolic	cytoplasm;	catalytic a	Pf00348	9453	ENSG0000	GGPS1	1	Terpenoid		Cholestero	8	High	High	High	High	High	High	1	
FALSE	High	Master Prc	Q9Y597	BTB/POZ d	0	8.731	3	2	2	1	815	88.9	7.03	5.83	2	cell organi:	membrane	protein bir	Pf02214	51133	ENSG0000	KCTD3	1				0	High	High	High	High	High	High	1	
FALSE	High	Master Prc	Q9Y5Z9-1	UbiA preny	0.003	3.444	3	1	1	1	338	36.8	8.15	3.38	1	metabolic	cytoplasm;	antioxidan	Pf01040	29914	ENSG0000	UBIAD1	1			Metabolisr	4	High	High	High	High	High	High	1	
FALSE	High	Master Prc	Q6NYC8	Phostensin	0	12.857	7	3	3	3	613	67.9	5.4	7.98	3		cytoplasm;	protein bir	Pf13914, P	170954	ENSG0000	PPP1R18	6; CHR_HS		TYROBP Ca		1	High	High	High	High	High	High	1	
FALSE	High	Master Prc	Q9BU89	Deoxyhypu	0.009	2.107	4	1	1	1	302	32.9	4.83	2.08	1	metabolic	cytosol	catalytic a	Pf13646	83475	ENSG0000	DOHH	19			Hypusine s	4	High	High	High	High	High	High	1	
FALSE	High	Master Prc	Q99590-1	protein SC/	0	8.106	2	2	2	2	1463	164.6	8.41	3.01	2	cell organi:	nucleus	catalytic a	Pf00097, P	9169	ENSG0000	SCAF11	12		Apoptosis		1	High	High	High	High	High	High	1	
FALSE	High	Master Prc	Q03405	Urokinase	0.002	3.985	4	1	1	1	335	37	6.65	1.81	1	cellular co	cell surface	protein bir	Pf00021	5329	ENSG0000	PLAUR	19	Compleme	Primary Fc	Dissolution	14	High	High	High	High	High	High	1	
FALSE	Medium	Master Prc	Q8TF74	WAS/WAS	0.024	1.509	3	1	1	1	440	46.3	10.93	0	1	regulation	cytoplasm;	protein bir	Pf02205	147179	ENSG0000	WIPF2	17	Endocytosi		Regulation	9	High	High	High	High	High	High	1	[K46]
FALSE	Low	Master Prc	O75884-1	Putative hy	0.06	1.119	12	1	1	1	186	21	6.2	0	1	regulation	cytoplasm;	catalytic a		10741	ENSG0000	RBBP9	20				0	High	High	High	High	High	High	1	
FALSE	Medium	Master Prc	Q16566	calcium/ca	0.049	1.209	1	1	1	1	473	51.9	5.82	0	1	cell differe	cytoplasm;	catalytic a	Pf00069, P	814	ENSG0000	CAMK4	5	Long-term	Energy Me	Signaling b	38	High	High	High	High	High	High	1	[Q398]
FALSE	High	Master Prc	O60346	PH domain	0	7.815	2	1	1	1	1717	184.6	6.28	4.16	1	cell death;	cytoplasm;	catalytic a	Pf00169, P	23239	ENSG0000	PHLPP1	18	PI3K-Akt si	Focal Adhe	Negative r	6	High	High	High	High	High	High	1	
FALSE	Low	Master Prc	Q9NQ03	transcripti	0.06	1.121	6	1	1	1	307	32.6	9.22	0	1	metabolic	nucleus	DNA bindi	Pf00096, P	85508	ENSG0000	SCRT2	20				0	High	High	High	High	High	High	1	[S132(100
FALSE	High	Master Prc	O95167	NADH deh	0.009	2.155	15	2	2	2	84	9.3	8.46	1.62	2	cell organi:	membrane	catalytic a	Pf14987	4696	ENSG0000	NDUFA3	19; CHR_H	Metabolic	Electron Tı	Complex I	13	High	High	High	High	High	High	1	
FALSE	Medium	Master Prc	O94804	Serine/thre	0.018	1.644	1	1	1	1	968	112.1	6.95	1.71	1	metabolic	cytoplasm;	catalytic a	Pf00069, P	6793	ENSG0000	STK10	5			Neutrophil	3	High	High	High	High	High	High	1	
FALSE	High	Master Prc	Q14919-1	Dr1-associ	0.003	3.305	5	1	1	1	205	22.3	5.17	2.35	1	metabolic	nucleus	DNA bindi	Pf00125, P	10589	ENSG0000	DRAP1	11			Signaling b	5	High	High	High	High	High	High	1	
FALSE	High	Master Prc	O15226	NF-kappa-l	0.003	3.379	1	1	1	1	690	77.6	8.79	0	1	metabolic	nucleus	DNA bindi	Pf00035, P	55922	ENSG0000	NKRF	X				0	High	High	High	High	High	High	1	
FALSE	High	Master Prc	Q13459-1	Unconvent	0.001	4.496	1	1	1	1	2157	243.2	8.75	1.94	1	cellular co	cytoplasm;	catalytic a	Pf00063, P	4650		MYO9B	19			SLIT2:ROB(	11	High	High	High	High	High	High	1	
FALSE	High	Master Prc	Q9BTY7	Protein HG	0.001	4.431	5	2	2	2	390	42.1	4.81	1.98	2				Pf04063, P	51236	ENSG0000	FAM203B;	8				0	High	High	High	High	High	High	1	
FALSE	High	Master Prc	O00217	NADH deh	0	6.671	11	2	3	2	210	23.7	6.34	5.79	2	cell organi:	membrane	catalytic a	Pf00037, P	4728	ENSG0000	NDUFS8	11	Alzheimer'	Electron Tı	Complex I	13	High	High	High	High	High	High	1	
FALSE	Medium	Master Prc	Q08426	Peroxisom	0.029	1.439	1	1	1	1	723	79.4	9.14	0	1	metabolic	cytosol;mi	catalytic a	Pf00378, P	1962	ENSG0000	EHHADH	3	Fatty acid	Constitutiv	Beta-oxida	23	High	High	High	High	High	High	1	
FALSE	Low	Master Prc	Q96QE3-1	ATPase far	0.07	1.043	1	1	1	1	1844	207.4	9.19	1.6	1	regulation	nucleus	nucleotide	Pf00004	79915	ENSG0000	ATAD5	17				0	High	High	High	High	High	High	1	[K1735]
FALSE	High	Master Prc	Q8TCC3	39S riboso	0.002	3.553	5	1	1	1	161	18.5	9.99	2.8	1	cell organi:	membrane	structural	Pf00327	51263	ENSG0000	MRPL30	2	Ribosome		Mitochonc	7	High	High	High	High	High	High	1	
FALSE	High	Master Prc	P16930	Fumarylac	0	6.115	6	2	2	2	419	46.3	6.95	3.75	2	metabolic	cytosol	catalytic a	Pf01557, P	2184	ENSG0000	FAH	15	Tyrosine m	Amino Aci	Phenylalar	7	High	High	High	High	High	High	1	
FALSE	Medium	Master Prc	Q5T2E6-1	UPF0668 p	0.022	1.577	1	1	1	1	689	78.7	6.6	1.73	1		membrane	protein bir	Pf08427	79591	ENSG0000	C10orf76	10				0	High	High	High	High	High	High	1	
FALSE	High	Master Prc	Q9NP73-1	Putative bi	0.004	2.693	2	1	1	1	1137	126	6.74	0	1	metabolic	endoplasm	catalytic a	Pf02338, P	79868	ENSG0000	ALG13	X	N-Glycan b		Defective A	10	High	High	High	High	High	High	1	
FALSE	Medium	Master Prc	Q14667-1	Protein KIA	0.044	1.253	0	1	1	1	2235	253.5	7.14	0	1		extracellul		Pf10344, P	9703	ENSG0000	KIAA0100	17				0	High	High	High	High	High	High	1	[K902]
FALSE	High	Master Prc	Q969N2	GPI transa	0.003	3.295	4	2	2	2	578	65.7	8.38	4.1	2	cell death;	cytoplasm;	catalytic a	Pf04113	51604	ENSG0000	PIGT	20	Metabolic		Attachmer	6	High	High	High	High	High	High	1	
FALSE	High	Master Prc	Q96LA8-1	protein arg	0	5.987	4	1	1	1	375	41.9	5.44	0	1	cell growth	cytosol;nu	catalytic a	Pf02353, P	55170	ENSG0000	PRMT6	1			RUNX1 reg	8	High	High	High	High	High	High	1	
FALSE	High	Master Prc	Q9Y394	Dehydroge	0	4.908	6	1	1	1	339	38.3	8.32	3.1	1	metabolic	membrane	catalytic a	Pf00106, P	51635	ENSG0000	DHRS7	14				0	High	High	High	High	High	High	1	
FALSE	High	Master Prc	Q9UK41-1	vacuolar pı	0	10.922	18	2	2	2	221	25.4	5.54	5.94	2	cell commı	cytoplasm;	protein bir	Pf03997	51160	ENSG0000	VPS28	8	Endocytosi		Budding ar	13	High	High	High	High	High	High	1	
FALSE	Medium	Master Prc	Q7Z7N9	Transmem	0.024	1.508	7	1	2	1	219	23.5	7.85	0	1	transport	membrane			374395	ENSG0000	TMEM179	11			Neutrophil	3	High	High	High	High	High	High	1	
FALSE	High	Master Prc	P29992	guanine nu	0	20.721	17	6	7	5	359	42.1	5.69	11.3	6	regulation	cytoplasm;	catalytic a	Pf00025, P	2767	ENSG0000	GNA11	19	Pathways i	Corticotro	Thrombin	33	High	High	High	High	High	High	1	
FALSE	High	Master Prc	Q8NDF8-5	Isoform 5 c	0	4.575	2	1	1	1	698	75.8	9.38	0	1	cell divisio	cytoplasm	catalytic a	Pf01909, P	64282	ENSG0000	PAPD5	16				0	High	High	High	High	High	High	1	
FALSE	High	Master Prc	Q96E39	RNA bindir	0	101.56	43	18	69	1	390	42.1	9.89	135.65	18	metabolic	nucleus	DNA bindi	Pf00076, P	494115	ENSG0000	RBMXL1	1	Spliceosom			1	High	High	High	High	High	High	1	
FALSE	Low	Master Prc	P52848-2	Isoform 2 c	0.05	1.199	4	1	1	1	556	62	6.92	0	1	defense re	membrane	catalytic a	Pf12062	3340		NDST1	5				0	High	High	High	High	High	High	1	
FALSE	High	Master Prc	Q0VDG4-1	secernin-3	0.003	3.079	4	2	3	1	424	48.5	5.55	0	2	metabolic		catalytic a	Pf03577	79634	ENSG0000	SCRN3	2				0	High	High	High	High	High	High	1	
FALSE	High	Master Prc	Q12899	Tripartite r	0.006	2.34	2	1	1	1	539	62.1	5.03	1.91	1	defense re	cytoplasm;	catalytic a	Pf00097, P	7726	ENSG0000	TRIM26	6; CHR_HS			Interferon	4	High	High	High	High	High	High	1	
FALSE	High	Master Prc	Q92614-1	Unconvent	0	6.81	1	1	1	1	2054	233	6.3	4.77	1	cell differe	cell surface	catalytic a	Pf00038, P	399687	ENSG0000	MYO18A	17			Signaling b	6	High	High	High	High	High	High	1	
FALSE	Low	Master Prc	Q9H0U9	Testis-spec	0.071	1.029	2	1	1	1	437	49.2	5.45	0	1	cell organi:	nucleus	protein bir	Pf00956	7259	ENSG0000	TSPYL1	6		Sudden Inf		1	High	High	High	High	High	High	1	
FALSE	Low	Master Prc	Q9NQT5	exosome c	0.062	1.108	3	1	1	1	275	29.6	8.1	1.82	1	metabolic	cytoplasm;	catalytic a		51010	ENSG0000	EXOSC3	9	RNA degra		Major path	15	High	High	High	High	High	High	1	
FALSE	Medium	Master Prc	Q9NPA3	Mid1-inter	0.012	1.952	4	1	1	1	183	20.2	5.5	3.33	1	cell organi:	cytoplasm;	protein bir	Pf07084	58526	ENSG0000	MID1IP1	X			Import of p	4	High	High	High	High	High	High	1	
FALSE	High	Master Prc	O00754	Lysosomal	0	6.338	2	2	2	2	1011	113.7	7.28	2.22	2	metabolic	extracellul	catalytic a	Pf01074, P	4125	ENSG0000	MAN2B1	19	Lysosome;		Neutrophil	8	High	High	High	High	High	High	1	
FALSE	High	Master Prc	Q92544	Transmem	0	8.543	4	2	2	2	642	74.5	6.54	2.8	2	cell organi:	endosome	protein bir	Pf02990	9777	ENSG0000	TM9SF4	20				0	High	High	High	High	High	High	1	
FALSE	High	Master Prc	Q9NRX1	RNA-bindi	0	8.834	10	2	2	2	252	27.9	9.73	3.79	2	metabolic	cytosol;nu	RNA bindi	Pf00013	56902	ENSG0000	PNO1	2			Major path	5	High	High	High	High	High	High	1	
FALSE	Medium	Master Prc	O95295	SNARE-ass	0.014	1.841	7	1	1	1	136	14.9	9.31	0	1	cell commı	cytoplasm;	protein bir	Pf14712	23557	ENSG0000	SNAPIN	1			Golgi Asso	5	High	High	High	High	High	High	1	
FALSE	High	Master Prc	Q9UIG8-1	solute carr	0	5.107	2	1	1	1	710	76.5	6.73	2.01	1	transport	membrane	protein bir	Pf03137, P	28232	ENSG0000	SLCO3A1	15			Transport	4	High	High	High	High	High	High	1	
FALSE	Low	Master Prc	Q9Y3C8	ubiquitin-f	0.065	1.082	4	1	1	1	167	19.4	7.4	0	1	metabolic	cytoplasm	catalytic a	Pf08694	51506	ENSG0000	UFC1	1				0	High	High	High	High	High	High	1	
FALSE	Low	Master Prc	Q14161-1	ARF GTPas	0.05	1.197	1	1	1	1	759	84.5	7.23	0	1	regulation		enzyme re	Pf00023, P	9815	ENSG0000	GIT2	12	Endocytosi	Integrin-m		2	High	High	High	High	High	High	1	
FALSE	High	Master Prc	P02100	Hemoglobi	0.005	2.55	7	1	1	1	147	16.2	8.63	2.06	1	cell organi:	cytosol	metal ion l	Pf00042	3046	ENSG0000	HBE1	11			Factors inv	2	High	High	High	High	High	High	1	
FALSE	High	Master Prc	Q96JB5	CDK5 regu	0	7.444	4	2	2	2	506	56.9	4.75	2.27	2	cell organi:	cytoplasm;	protein bir	Pf05600	80279	ENSG0000	CDK5RAP3	17				0	High	High	High	High	High	High	1	
FALSE	Low	Master Prc	Q8TCT8	Signal pept	0.055	1.152	1	1	1	1	520	58.1	8.32	0	1	metabolic	endosome	catalytic a	Pf02225, P	84888	ENSG0000	SPPL2A	15			Regulation	4	High	High	High	High	High	High	1	
FALSE	Medium	Master Prc	Q6P9B9	Integrator	0.017	1.675	1	1	1	1	1019	107.9	7.05	0	1	metabolic	cytoplasm;	protein bir	Pf14837, P	80789	ENSG0000	INTS5	11			RNA polym	3	High	High	High	High	High	High	1	
FALSE	High	Master Prc	P13284	Gamma-in	0.009	2.114	4	1	2	1	250	27.9	4.88	0	1	metabolic	cytosol;ext	catalytic a	Pf03227	10437	ENSG0000	IFI30	19	Antigen pr		MHC class	7	High	High	High	High	High	High	1	
FALSE	High	Master Prc	Q14331	Protein FR(	0	13.174	13	3	4	3	258	29.2	9.01	4.78	3	metabolic	cytoplasm;	protein bir	Pf06229, P	2483	ENSG0000	FRG1	4; CHR_HG				0	High	High	High	High	High	High	1	
FALSE	High	Master Prc	P46934-4	Isoform 4 c	0	12.745	4	2	3	1	900	104.2	5.97	6.3	2	cell organi:	chromosor	catalytic a	Pf00168, P	4734	ENSG0000	NEDD4	15		Proteasom		2	High	High	High	High	High	High	1	
FALSE	High	Master Prc	P56696	Potassium	0.006	2.512	1	1	1	1	695	77.1	9.54	2.29	1	regulation	membrane	protein bir	Pf00520, P	9132	ENSG0000	KCNQ4	1	Cholinergi		Voltage ga	4	High	High	High	High	High	High	1	[R642]
FALSE	Medium	Master Prc	A4FU01	Myotubula	0.017	1.676	1	1	1	1	709	79.5	7.03	0	1				Pf06602, P	10903	ENSG0000	MTMR11	1				0	High	High	High	High	High	High	1	
FALSE	High	Master Prc	Q9Y5J9	mitochond	0	5.43	13	1	2	1	83	9.3	5.12	1.81	1	transport	membrane	metal ion l	Pf02953	26521	ENSG0000	TIMM8B	11			Mitochonc	2	High	High	High	High	High	High	1	
FALSE	High	Master Prc	Q15006	ER membra	0	7.148	11	2	2	2	297	34.8	6.57	2.03	2	metabolic	cytoplasm;	protein bir	Pf13414, P	9694	ENSG0000	EMC2	8				0	High	High	High	High	High	High	1	

FALSE	High	Master Prc	O15231-1	zinc finger	0	6.379	4	2	2	2	689	73.5	7.01	1.81	2		cytoplasm;	metal ion l	Pf00412	7739	ENSG0000	ZNF185	X				0								High	High	High	High	High	High	1	
FALSE	Medium	Master Prc	Q92615	la-related	0.02	1.603	1	1	1	1	738	80.5	6.92	2.43	1	regulation	cytoplasm;	protein bir	Pf05383, P	23185	ENSG0000	LARP4B	10				0								High	High	High	High	High	High	1	
FALSE	Medium	Master Prc	O95153-1	Peripheral-	0.045	1.237	0	1	1	1	1857	199.9	5.11	0	1	cell organi	cytoplasm;	catalytic ac	Pf00018, P	9256	ENSG0000	BZRAP1; T	17			Norepinep	13								High	High	High	High	High	High	1	
FALSE	High	Master Prc	Q5TDH0	Protein DD	0.007	2.253	2	1	1	1	399	44.5	5.05	0	1	metabolic	cytosol	catalytic ac	Pf00077, P	84301	ENSG0000	DDI2	1				0								High	High	High	High	High	High	1	
FALSE	High	Master Prc	Q7Z7F0-1	Protein BL	0.001	4.239	3	2	3	2	614	64.8	8.73	1.88	2	cell organi	cytoplasm;	protein bir		22889	ENSG0000	KIAA0907;	1				0								High	High	High	High	High	High	1	
FALSE	High	Master Prc	Q01085	Nucleolysi	0	8.372	8	2	3	1	375	41.6	7.74	5.93	2	cell death;	cytoplasm;	DNA bindi	Pf00076, P	7073	ENSG0000	TIAL1	10			FGFR2 alte	5								High	High	High	High	High	High	1	
FALSE	Low	Master Prc	P24311	Cytochrom	0.068	1.054	9	1	1	1	80	9.2	10.27	2.3	1	metabolic	membrane	catalytic ac	Pf05392	1349	ENSG0000	COX7B	X	Non-alcoh	Electron Tr	TP53 Regu	17								High	High	High	High	High	High	1	
FALSE	High	Master Prc	O14641	Segment p	0	10.063	5	3	4	3	736	78.9	6.02	6.49	3	cell organi	cytoplasm;	protein bir	Pf00595, P	1856	ENSG0000	DVL2	17	Notch sign	Neural Cre	WNT medi	38								High	High	High	High	High	High	1	
FALSE	High	Master Prc	P98194-1	Calcium-tr	0	8.677	5	4	4	4	919	100.5	6.74	5.34	4	cell organi	Golgi;mem	catalytic ac	Pf00122, P	27032	ENSG0000	ATP2C1	3			Ion transp	3								High	High	High	High	High	High	1	
FALSE	High	Master Prc	Q9Y666	Solute carr	0.002	3.875	2	2	2	1	1083	119	6.71	1.72	2	cell commu	membrane	catalytic ac	Pf00324, P	10723	ENSG0000	SLC12A7	5	Collecting		Cation-cou	5								High	High	High	High	High	High	1	
FALSE	Low	Master Prc	Q6PI77	Protein BH	0.057	1.134	2	1	1	1	547	60.3	7.58	0	1	regulation	cytoplasm;	protein bir	Pf04826	80823; 10	ENSG0000	BHLHB9; A	X				0								High	High	High	High	High	High	1	[K83]
FALSE	High	Master Prc	Q96EK4	THAP dom	0.002	4.157	3	1	1	1	314	34.4	8.98	2.87	1	metabolic	cytoplasm;	DNA bindi	Pf05485	57215	ENSG0000	THAP11	16				0								High	High	High	High	High	High	1	
FALSE	Medium	Master Prc	Q69YN4	protein vir	0.039	1.295	1	1	1	1	1812	201.9	5.01	0	1	metabolic	cytosol;nu	RNA bindi		25962	ENSG0000	KIAA1429;	8				0								High	High	High	High	High	High	1	
FALSE	High	Master Prc	P17050	alpha-N-ac	0.004	2.643	3	1	1	1	411	46.5	5.19	0	1	metabolic	cytoplasm;	catalytic ac	Pf02065	4668	ENSG0000	NAGA	22	Glycosphir			2								High	High	High	High	High	High	1	
FALSE	Low	Master Prc	P11678	Eosinophil	0.07	1.041	3	1	1	1	715	81	10.29	2.57	1	cellular co	extracellul	antioxidan	Pf03098	8288	ENSG0000	EPX	17	Asthma		Neutrophil	4								High	High	High	High	High	High	1	
FALSE	High	Master Prc	Q6P4I2	WD repeat	0.006	2.496	3	1	1	1	378	41.7	5.78	2.12	1	cell organi	cytoplasm;	protein bir		84942	ENSG0000	WDR73	15				0								High	High	High	High	High	High	1	
FALSE	High	Master Prc	P51580	Thiopurine	0.003	2.959	4	1	1	1	245	28.2	6.23	0	1	metabolic	cytoplasm;	catalytic ac	Pf05724	7172	ENSG0000	TPMT	6	Drug meta	Metapathv	Defective	11								High	High	High	High	High	High	1	
FALSE	Medium	Master Prc	Q8WTW3	conserved	0.02	1.606	1	1	1	1	980	108.9	7.31	0	1	cell organi	Golgi;mem	protein bir	Pf08700	9382	ENSG0000	COG1	17			COPI-medi	11								High	High	High	High	High	High	1	
FALSE	Medium	Master Prc	Q9UK99	F-box only	0.015	1.749	2	1	1	1	471	54.5	5	1.67	1	metabolic	cytosol;nu	catalytic ac	Pf00646, P	26273	ENSG0000	FBXO3	11				0								High	High	High	High	High	High	1	
FALSE	High	Master Prc	P07108	acyl-CoA-b	0.007	2.261	23	1	1	1	87	10	6.57	0	1	metabolic	endoplasm	protein bir	Pf00887	1622	ENSG0000	DBI	2	PPAR signa	Sterol Reg	Mitochonc	8								High	High	High	High	High	High	1	
FALSE	High	Master Prc	P61916	Epididyma	0	8.555	17	2	4	2	151	16.6	7.65	5.02	2	metabolic	endoplasm	protein bir	Pf02221	10577	ENSG0000	NPC2	14	Lysosome	TYROBP Ca	Neutrophil	9								High	High	High	High	High	High	1	
FALSE	High	Master Prc	Q96BD0	solute carr	0	7.578	6	4	5	4	722	77.1	7.83	6.64	4	transport	membrane	protein bir	Pf03137, P	28231	ENSG0000	SLCO4A1	20			Transport	4								High	High	High	High	High	High	1	
FALSE	High	Master Prc	Q02952-1	A-kinase a	0	7.095	2	2	2	2	1782	191.4	4.41	3.35	2	regulation	cytoplasm;	protein bir	Pf03832	9590	ENSG0000	AKAP12	6		G Protein S		1								High	High	High	High	High	High	1	
FALSE	High	Master Prc	Q96FZ7	charged m	0	6.331	6	1	1	1	201	23.5	5.31	2.94	1	cell commu	cytosol;en	protein bir	Pf03357	79643	ENSG0000	CHMP6	17	Endocytosi		Budding ar	12								High	High	High	High	High	High	1	
FALSE	High	Master Prc	O95544	NAD kinas	0	11.729	7	2	2	2	446	49.2	6.47	4.27	2	metabolic	cytosol	catalytic ac	Pf01513	65220	ENSG0000	NADK	1	Nicotinate	NAD+ met	Nicotinate	7								High	High	High	High	High	High	1	
FALSE	High	Master Prc	O76024	Wolframin	0	6.021	3	3	4	3	890	100.2	8.05	1.75	3	cellular ho	endoplasm	protein bir		7466	ENSG0000	WFS1	4	Protein pro		Post-transl	8								High	High	High	High	High	High	1	
FALSE	High	Master Prc	Q9Y2H5	Pleckstrin	0	4.638	2	2	2	2	1048	117.1	9.1	1.84	2				Pf00169, P	22874	ENSG0000	PLEKHA6	1			Synthesis c	5								High	High	High	High	High	High	1	
FALSE	Medium	Master Prc	Q9P2H3-1	Intraflagell	0.032	1.409	2	1	1	1	777	88	7.69	1.62	1	cell organi	cytoplasm;	protein bir	Pf00400	57560	ENSG0000	IFT80	3			Intraflagell	3								High	High	High	High	High	High	1	[Q74;
FALSE	High	Master Prc	Q147X3	N-alpha-ac	0.003	2.957	4	1	1	1	362	39.3	5.52	0	1	metabolic	cytoplasm;	catalytic ac	Pf00583, P	122830	ENSG0000	NAA30	14		Metapathv	Retrograde	5								High	High	High	High	High	High	1	
FALSE	High	Master Prc	O15270	Serine palr	0	13.659	5	2	2	2	562	62.9	7.78	6.31	2	metabolic	endoplasm	catalytic ac	Pf00155, P	9517	ENSG0000	SPTLC2	14	Sphingolip	Sphingolip	Sphingolip	8								High	High	High	High	High	High	1	
FALSE	Medium	Master Prc	Q96A73-1	Putative m	0.014	1.827	3	1	1	1	305	33.2	9.39	0	1	metabolic	cytoplasm	catalytic ac	Pf15302	57179	ENSG0000	KIAA1191	5				0								High	High	High	High	High	High	1	
FALSE	High	Master Prc	O15403	Monocarb	0.003	3.003	2	1	1	1	523	57.4	7.81	2.54	1	transport	membrane	transporte	Pf07690, P	9120	ENSG0000	SLC16A6	17				0								High	High	High	High	High	High	1	
FALSE	Medium	Master Prc	Q6PCB5-1	Round spe	0.015	1.777	2	1	1	1	846	94.8	8.78	2.21	1		nucleus			222194	ENSG0000	RSBN1L	7				0								High	High	High	High	High	High	1	
FALSE	Medium	Master Prc	Q9H799-1	Protein JBT	0.026	1.48	0	1	1	1	3197	361.5	6.99	1.62	1	cell organi	membrane	protein bir	Pf15392	65250	ENSG0000	C5orf42	5				0								High	High	High	High	High	High	1	
FALSE	Low	Master Prc	O43159	ribosomal	0.054	1.161	2	1	1	1	456	50.7	9.42	0	1	cell commu	cytosol;me	catalytic ac	Pf05148, P	23378	ENSG0000	RRP8	11			SIRT1 nega	4								High	High	High	High	High	High	1	
FALSE	High	Master Prc	O95376	E3 ubiquiti	0	4.679	3	1	1	1	493	57.8	5.63	2.8	1	cell growth	cytoplasm;	catalytic ac	Pf01485	10425	ENSG0000	ARIH2	3			Antigen pr	4								High	High	High	High	High	High	1	
FALSE	High	Master Prc	Q53GA4	pleckstrin	0.007	2.275	9	2	2	2	152	17.1	9.17	2.53	2	cell death;	cytoplasm;		Pf00169	7262	ENSG0000	PHLDA2	11; CHR_H				0								High	High	High	High	High	High	1	
FALSE	High	Master Prc	P45983-1	Mitogen-a	0	4.685	6	1	1	1	427	48.3	6.89	3.32	1	cell commu	cytoplasm;	catalytic ac	Pf00069, P	5599	ENSG0000	MAPK8	10	Protein pro	Osteoclast	FCERI med	150								High	High	High	High	High	High	1	
FALSE	High	Master Prc	Q3KQU3	MAP7 dom	0.002	3.721	2	1	1	1	841	92.8	10.11	1.6	1	cell organi	cytoplasm;	structural		55700	ENSG0000	MAP7D1	1				0								High	High	High	High	High	High	1	
FALSE	High	Master Prc	O00308	NEDD4-like	0.006	2.499	1	1	1	1	870	98.9	7.12	0	1	metabolic	cytoplasm;	catalytic ac	Pf00168, P	11060	ENSG0000	WWP2	16	Ubiquitin r		Regulation	6								High	High	High	High	High	High	1	
FALSE	High	Master Prc	Q96I25	Splicing fac	0.002	3.74	5	2	2	2	401	44.9	5.97	2.84	2	metabolic	nucleus;sp	protein bir	Pf00076, P	84991	ENSG0000	RBM17	10	Spliceosom	mRNA Pro	mRNA Spli	6								High	High	High	High	High	High	1	
FALSE	Medium	Master Prc	Q9Y5A7	NEDD8 ult	0.012	1.997	1	1	2	1	615	70.5	5.96	0	1	metabolic	cytosol;nu	protein bir	Pf00627	51667		NUB1	7			Neddylatic	3								High	High	High	High	High	High	1	
FALSE	Medium	Master Prc	Q13067	G antigen	0.024	1.522	8	1	1	1	118	12.9	4.25	0	1				Pf05831			GAGE3					0								High	High	High	High	High	High	1	
FALSE	High	Master Prc	Q8TAQ2	SWI/SNF c	0	14.194	3	4	5	1	1214	132.8	5.69	9.44	4	cell organi	nucleus	DNA bindi	Pf04433	6601	ENSG0000	SMARCC2	12			RUNX1 int	8								High	High	High	High	High	High	1	
FALSE	High	Master Prc	Q9H0G5	Nuclear sp	0.002	4.031	2	1	1	1	558	66.4	8.84	0	1	metabolic	nucleus	protein bir	Pf09745	84081	ENSG0000	NSRP1	17				0								High	High	High	High	High	High	1	
FALSE	High	Master Prc	P78536-1	Disintegrin	0	4.919	2	2	2	2	824	93	5.76	2.71	2	cell differe	cell surface	catalytic ac	Pf00200, P	6868	ENSG0000	ADAM17	2	Notch sign	Alzheimers	Collagen d	41								High	High	High	High	High	High	1	
FALSE	High	Master Prc	Q9NYZ1	Golgi appa	0.002	3.886	8	2	2	2	205	23.6	8.35	0	2	transport	membrane		Pf05832	51030	ENSG0000	FAM18B1;	17				0								High	High	High	High	High	High	1	
FALSE	High	Master Prc	P41223	Protein BU	0	10.073	15	2	3	2	144	17	8.82	4.43	2	cell organi	nucleus;sp	catalytic ac	Pf01125	8896	ENSG0000	BUD31	7	Spliceosom		mRNA Spli	5								High	High	High	High	High	High	1	
FALSE	High	Master Prc	P14649	Myosin ligl	0	28.302	19	3	15	1	208	22.8	5.73	46.52	3	cellular co	cytosol	catalytic ac	Pf13405, P	140465	ENSG0000	MYL6B	12	Vascular sr		Smooth M	4								High	High	High	High	High	High	1	
FALSE	High	Master Prc	O43432	Eukaryotic	0	7.708	2	3	4	1	1585	176.5	5.38	5.06	3	metabolic	cytosol	protein bir	Pf02020, P	8672	ENSG0000	EIF4G3	1	RNA transp	Translatior	ISG15 anti	8								High	High	High	High	High	High	1	
FALSE	High	Master Prc	P00374	dihydrofol	0	4.935	13	2	2	2	187	21.4	7.42	2.36	2	cell organi	cytoplasm;	catalytic ac	Pf00186	1719; 573	ENSG0000	DHFR; DHF	5; 18	Metabolic	Fluoropyri	Activation	23								High	High	High	High	High	High	1	
FALSE	High	Master Prc	Q8IXB1-1	DnaJ homo	0.001	4.295	3	2	2	2	793	91	7.18	2.74	2	cellular ho	endoplasm	catalytic ac	Pf00085, P	54431	ENSG0000	DNAJC10	2	Protein pro			1								High	High	High	High	High	High	1	
FALSE	Medium	Master Prc	Q53S58	transmeml	0.028	1.457	4	1	1	1	311	33.7	9.61	1.87	1		membrane			80775	ENSG0000	TMEM177	2				0								High	High	High	High	High	High	1	
FALSE	Medium	Master Prc	P48067-1	Sodium- ar	0.037	1.329	1	1	1	1	706	78.2	7.93	0	1	transport	membrane	transporte	Pf00209	6536	ENSG0000	SLC6A9	1		NRF2 path	Na+/Cl- de	5								High	High	High	High	High	High	1	[K645]
FALSE	High	Master Prc	O43665	Regulator	0	8.64	14	2	3	2	173	20.2	5.49	4.92	2	regulation	cytoplasm;	enzyme re	Pf00615	6001	ENSG0000	RGS10	10		Mesoderm	G alpha (i)	7								High	High	High	High	High	High	1	
FALSE	High	Master Prc	Q96HW7	integrator	0.003	3.189	2	1	1	1	963	108.1	6.44	2.78	1	metabolic	nucleus	protein bir	Pf01602, P	92105	ENSG0000	INTS4	11			RNA polym	3								High	High	High	High	High	High	1	
FALSE	High	Master Prc	P35610	sterol O-ac	0.003	2.86	2	1	1	1	550	64.7	8.94	0	1	cell differe	endoplasm	catalytic ac	Pf03062	6646	ENSG0000	SOAT1	1	Steroid bic	Statin Path	LDL cleara	6								High	High	High	High	High	High	1	
FALSE	Medium	Master Prc	P36969	Phospholip	0.013	1.918	7	1	1	1	197	22.2	8.37	2.48	1	cell organi	cytoplasm;	antioxidan	Pf00255	2879	ENSG0000	GPX4	19	Glutathion	Glutathion	Synthesis c	14								High	High	High	High	High	High	1	
FALSE	Low	Master Prc	P50281	Matrix me	0.069	1.05	1	1	1	1	582	65.9	7.77	0	1	cell differe	cytoplasm;	catalytic ac	Pf00045, P	4323	ENSG0000	MMP14	14	GnRH signa	Matrix Me	Collagen d	10								High	High	High	High	High	High	1	
FALSE	High	Master Prc	P51532-1	Transcripti	0	7.116	2	3	3	3	1647	184.5	7.88	2.98	3	cell organi	membrane	catalytic ac	Pf00176, P	6597	ENSG0000	SMARCA4	19		Preimplant	RUNX1 int	18								High	High	High	High	High	High	1	
FALSE	High	Master Prc	Q8IV38	Ankyrin re	0.003	2.84	2	1	1	1	441	49.3	6.25	0	1	regulation		metal ion l	Pf00023, P	57037	ENSG0000	ANKMY2	7				0								High	High	High	High	High	High	1	
FALSE	High	Master Prc	O60337	E3 ubiquiti	0.006	2.463	1	1	1	1	910	102.5	6.28	0	1	metabolic	endoplasm	catalytic ac	Pf12906	10299	ENSG0000	MARCH6	5	Protein pro		ER Quality	7								High	High	High	High	High	High	1	
FALSE	High	Master Prc	A5A3E0	POTE anky	0	49.276	9	8	75	1	1075	121.4	6.2	170.99	8		cytoplasm	protein bir	Pf00022, P	728378	ENSG0000	POTEF	2				0								High	High	High	High	High	High	1	[Q925]
FALSE	Medium	Master Prc	O00534	von Willeb	0.012	1.97	2	1	1	1	786	86.4	6.58	0	1				Pf00092, P	4013	ENSG0000	VWA5A	11				0								High	High	High	High	High	High	1	
FALSE	High	Master Prc	O95926	Pre-mRNA	0.001	4.472	7	1	2	1	243	28.7	8.4	5.89	1	cell organi	nucleus;sp	protein bir	Pf08231	25949	ENSG0000	SYF2	1	Spliceosom		mRNA Spli	5								High	High	High	High	High	High	1	
FALSE	High	Master Prc	Q96HH9	GRAM don	0	6.264	4	1	1	1	432	47.8	7.9	2.79	1			protein bir	Pf02893	65983	ENSG0000	GRAMD3;	5				0								High	High	High	High	High	High	1	
FALSE	High	Master Prc	P23497	Nuclear au	0	21.27	4	4	12	1	879	100.4	8.22	18.35	4	cell organi	cytoplasm;	DNA bindi	Pf00505, P	6672	ENSG0000	SP100	2	Viral carcir		SUMOylati	11								High	High	High	High	High	High	1	
FALSE	High	Master Prc	P36551	Oxygen-de	0	5.849	7	3	3	3	454	50.1	8.25	3.33	3	metabolic	cytosol;me	catalytic ac	Pf01218	1371	ENSG0000	CPOX	3	Porphyrin	Heme Bios	Heme bios	6								High	High	High	High	High	High	1	
FALSE	Medium	Master Prc	Q6UW78	Ubiquinol-	0.014	1.814	15	1	1	1	93	10.1	9.41	0	1	cell organi	membrane		Pf15141	790955	ENSG0000	C11orf83;	11				0								High	High	High	High	High	High	1	
FALSE	High	Master Prc	P20585	DNA mism	0.005	2.588	1	1	1	1	1137	127.3	8.02	0	1	cell organi	membrane	catalytic ac	Pf00488, P	4437	ENSG0000	MSH3	5	Platinum d		Defective I	11								High	High	High	High	High	High	1	
FALSE	High	Master Prc	P53801	pituitary tu	0.002	3.704	10	1	1	1	180	20.3	8.79	1.79	1	regulation	cytoplasm;	protein bir		754	ENSG0000	PTTG1IP	21				0								High	High	High	High	High	High	1	
FALSE	Low	Master Prc	A0A1B0GU	Small integ	0.069	1.046	9	1	1	1	152	16.5	4.56	0	1		membrane			11080627	ENSG0000	SMIM28	6				0								High	High	High	High	High	High	1	
FALSE	High	Master Prc	P19525	interferon-	0	13.8	7	4	6	4	551	62.1	8.4	9.99	4	cell commu	cytoplasm;	catalytic ac	Pf00035, P	5610	ENSG0000	EIF2AK2	2	Hepatitis C	Translatior	Inhibition	20								High	High	High	High	High	High	1	
FALSE	Low	Master Prc	Q7L311	Armadillo	0.071	1.037	2	1	1	1	632	65.6	8.53	1.89	1		membrane	protein bir	Pf00514, P	9823	ENSG0000	ARMCX2	X				0								High	High	High	High	High	High	1	[R54;
FALSE	High	Master Prc	Q13490-1	Baculovira	0	6.048	4	3	3	3	618	69.9	6.7	0	3	cell death;	chromosor	catalytic ac	Pf00619, P	329	ENSG0000	BIRC2	11	NF-kappa I	TNF relate	Ub-specific	48								High	High	High	High	High	High	1	
FALSE	High	Master Prc	Q9NPH0-1	Lysophosp	0.005	2.548	2	1	1	1	428	48.8	6.47	0	1	metabolic	cytoplasm;	catalytic ac	Pf00328	51205		ACP6	1			Synthesis c	5								High	High	High	High	High	High	1	
FALSE	High	Master Prc	Q9NX74	tRNA-dihy	0.002	3.547	3	1	1	1	493	55	7.11	0	1	metabolic	cytoplasm;	catalytic ac	Pf00035, P	54920	ENSG0000	DUS2; DUS	16			tRNA mod	3								High	High	High	High	High	High	1	
FALSE	High	Master Prc	Q9BUR5-1	MICOS con	0.003	3.304	5	1	1	1	198	22.3	9.13	2.47	1	cell organi	cytosol;en	protein bir	Pf09769	79135	ENSG0000	APOO	X			Cristae for	3								High	High	High	High	High	High	1	
FALSE	High	Master Prc	O75391	Sperm-ass	0.002	3.282	10	1	1	1	227	26	7.91	2.69	1		nucleus		Pf01424	9552	ENSG0000	SPAG7	17				0								Not Found	Not Found	Not Found	Not Found	Not Found	Not Found	1	

FALSE	High	Master Prc	P17480-1	Nucleolar t	0.002	3.606	1	1	1	1	764	89.4	5.81	1.8	1	metabolic	nucleus	DNA bindi	Pf00505, P	7343	ENSG0000	UBTF	17		Ectoderm	RNA Polym	12								High	High	High	High	High	High	1	
FALSE	High	Master Prc	Q9UPY5	Cystine/glu	0.004	2.76	1	1	1	1	501	55.4	9.19	0	1	cell differe	cell surface	protein bir	Pf00324, P	23657	ENSG0000	SLC7A11	4		NRF2 path	Basigin int	9								High	High	High	High	High	High	1	
FALSE	Low	Master Prc	P42702-1	Leukemia i	0.051	1.186	1	1	1	1	1097	123.7	5.72	1.72	1	regulation	extracellul	protein bir	Pf00041	3977	ENSG0000	LIFR	5	Jak-STAT si	Physiologi	IL-6-type c	17								High	High	High	High	High	High	1	
FALSE	High	Master Prc	O15013	rho guanin	0.002	3.526	1	1	1	1	1369	151.5	5.68	2.84	1	cell organi	cytosol	protein bir	Pf00621	9639	ENSG0000	ARHGEF10	8; CHR_HS			G alpha (1:	11								High	High	High	High	High	High	1	
FALSE	Low	Master Prc	O94763-1	Unconvent	0.05	1.196	2	1	1	1	535	59.8	5.05	0	1	metabolic	cytoplasm;	enzyme re	Pf02996, P	8725	ENSG0000	URI1	19				0								High	High	High	High	High	High	1	
FALSE	High	Master Prc	Q96IZ0	PRKC apop	0	7.939	4	1	1	1	340	36.5	5.41	3.04	1	cell death;	cytoplasm;	protein bir	Pf01544, P	5074	ENSG0000	PAWR	12				0								High	High	High	High	High	High	1	
FALSE	High	Master Prc	Q9GZM8-1	Nuclear dis	0.006	2.393	11	2	3	2	345	38.4	5.24	0	2	cell differe	chromosor	catalytic a	Pf04880, P	81565	ENSG0000	NDEL1	17			Amplificati	16								High	High	High	High	High	High	1	[R333]
FALSE	Medium	Master Prc	Q96EI5	Transcripti	0.026	1.474	4	1	1	1	215	24.6	5.2	0	1	metabolic	nucleus	protein bir	Pf04538	79921	ENSG0000	TCEAL4	X				0								High	High	High	High	High	High	1	
FALSE	High	Master Prc	Q8IYI6	Exocyst co	0.003	3.468	2	2	2	2	725	81.7	5.49	0	2	cell organi	cytoplasm;	protein bir	Pf00169, P	149371	ENSG0000	EXOC8	1		RalA down	Translocat	11								High	High	High	High	High	High	1	
FALSE	Medium	Master Prc	Q13287	N-myc-inte	0.031	1.421	3	1	1	1	307	35	5.34	0	1	defense re	cytoplasm;	protein bir	Pf07292, P	9111	ENSG0000	NMI	2		IL-2 Signali		1								High	High	High	High	High	High	1	
FALSE	Medium	Master Prc	O95073-1	Fibrinogen	0.015	1.736	5	1	1	1	299	34.7	7.12	0	1	metabolic	nucleus	catalytic a	Pf13873	10086141	ENSG0000	FSBP; RAD	8				0								High	High	High	High	High	High	1	
FALSE	High	Master Prc	Q86TM6	E3 ubiquiti	0.005	2.563	3	2	2	2	617	67.6	6.95	1.74	2	metabolic	endoplasm	catalytic a	Pf00097, P	84447	ENSG0000	SYVN1	11	Protein prc		Hh mutant	18								High	High	High	High	High	High	1	
FALSE	High	Master Prc	Q01658	Protein Dr:	0	6.244	9	2	3	2	176	19.4	4.75	6.17	2	cell organi	nucleus	DNA bindi	Pf00808	1810	ENSG0000	DR1	1			HATs acety	3								High	High	High	High	High	High	1	
FALSE	High	Master Prc	P07305	Histone H1	0.009	2.105	7	1	1	1	194	20.9	10.84	2.65	1	cell organi	chromosor	DNA bindi	Pf00538	3005	ENSG0000	H1F0	22			Formation	10								High	High	High	High	High	High	1	
FALSE	High	Master Prc	Q6P4E1	Protein CA	0	4.686	6	2	2	2	433	48.8	5.82	2.74	2		Golgi;mem		Pf04156	113201		CASC4	15				0								High	High	High	High	High	High	1	
FALSE	High	Master Prc	Q96K21	Abscission,	0	6.312	7	3	3	2	471	51.5	5.73	1.85	3	cell divisio	cytoplasm;	metal ion l	Pf01363	84936	ENSG0000	ZFYVE19	15				0								High	High	High	High	High	High	1	
FALSE	High	Master Prc	P33121	Long-chain	0	6.133	5	2	2	2	698	77.9	7.15	4.49	2	metabolic	endoplasm	catalytic a	Pf00501	2180	ENSG0000	ACSL1	4	Adipocytol	Fatty Acid	Synthesis c	21								High	High	High	High	High	High	1	
FALSE	Medium	Master Prc	P0C7V8	DDB1- and	0.036	1.345	1	1	1	1	631	71.1	4.73	0	1			protein bir	Pf00400	347442	ENSG0000	DCAF8L2	X				0								High	High	High	High	High	High	1	
FALSE	High	Master Prc	P84243	histone H3	0	20.189	31	6	18	1	136	15.3	11.27	24.26	6	cell differe	chromosor	DNA bindi	Pf00125, P	3020; 440	ENSG0000	H3F3A; H3	1; 2; 17	Transcripti	Gastric Ca	SIRT1 nega	54								High	High	High	High	High	High	1	[K80]
FALSE	High	Master Prc	P53803	DNA-direct	0	5.852	43	2	2	2	58	7	9.06	0	2	metabolic	cytosol;nu	catalytic a	Pf03604	5440	ENSG0000	POLR2K	8	RNA polym	Eukaryotic	Signaling b	98								High	High	High	High	High	High	1	
FALSE	Medium	Master Prc	Q9Y376	Calcium-bi	0.015	1.751	2	1	2	1	341	39.8	6.89	3.53	1	cell organi	cytoplasm;	catalytic a	Pf08569	51719	ENSG0000	CAB39	2	AMPK sign	AMP-activ	Energy dep	11								High	High	High	High	High	High	1	
FALSE	High	Master Prc	Q7L1S5	carbohydra	0.008	2.19	2	1	1	1	443	52	9.38	2.42	1	metabolic	extracellul	catalytic a	Pf03567	83539	ENSG0000	CHST9	18	Metabolic	Metapathw		2								High	High	High	High	High	High	1	
FALSE	High	Master Prc	Q9UJX4	Anaphase-	0.003	2.885	2	1	1	1	755	85	6.87	2.15	1	cell divisio	cytosol;nu	protein bir	Pf12862	51433	ENSG0000	ANAPC5	12	Progesterc		APC/C:Cdc	37								High	High	High	High	High	High	1	
FALSE	High	Master Prc	O43760	Synaptogy	0.004	2.767	4	1	1	1	224	24.8	4.94	0	1	cell organi	membrane	protein bir	Pf01284	9144	ENSG0000	SYNGR2	17				0								High	High	High	High	High	High	1	
FALSE	High	Master Prc	Q9NVS9	Pyridoxine	0	13.211	17	3	3	3	261	30	7.06	10.05	3	metabolic	cytosol	catalytic a	Pf01243, P	55163	ENSG0000	PNPO	17	Vitamin B6	Selenium M	Vitamins B	7								High	High	High	High	High	High	1	
FALSE	High	Master Prc	Q15126	phosphom	0.001	4.398	6	1	1	1	192	22	5.73	2.17	1	metabolic	cytoplasm;	catalytic a	Pf04275	10654	ENSG0000	PMVK	1	Terpenoid	Cholestero	Cholestero	10								High	High	High	High	High	High	1	
FALSE	Medium	Master Prc	O15375	Monocarb	0.036	1.348	2	1	1	1	505	55	8.22	2.06	1	transport	membrane	transporte	Pf07690	9121	ENSG0000	SLC16A5	17				0								High	High	High	High	High	High	1	
FALSE	High	Master Prc	O43688	Phospholip	0	6.975	7	2	3	2	288	32.6	8.35	3.73	2	metabolic	membrane	catalytic a	Pf01569	8612	ENSG0000	PPAP2C; P	19	Glyceroph	Triacylglyc	Sphingolip	14								High	High	High	High	High	High	1	
FALSE	High	Master Prc	Q5HYI8	Rab-like pr	0.003	3.028	5	1	1	1	236	26.4	7.11	1.79	1	regulation		nucleotide	Pf00071, P	285282	ENSG0000	RABL3	3				0								High	High	High	High	High	High	1	
FALSE	High	Master Prc	P18858	DNA ligase	0	5.855	3	3	3	3	919	101.7	5.62	2	3	cell divisio	mitochond	catalytic a	Pf01068, P	3978	ENSG0000	LIG1	19	DNA replic	Mismatch	Mismatch	37								High	High	High	High	High	High	1	
FALSE	High	Master Prc	O00186	syntaxin-bi	0	10.02	9	4	4	4	592	67.7	7.8	7.67	4	cell organi	cytoplasm;	protein bir	Pf00995	6814	ENSG0000	STXBP3	1		Insulin Sign	Translocat	8								High	High	High	High	High	High	1	
FALSE	High	Master Prc	Q96C57	Uncharact	0	12.176	10	2	3	2	262	28.2	9.42	6.52	2			protein bir		64897	ENSG0000	C12orf43	12				0								High	High	High	High	High	High	1	
FALSE	High	Master Prc	Q9NPE3	H/ACA ribo	0.002	3.52	25	1	1	1	64	7.7	9.99	2.57	1	cell organi	nucleus	protein bir	Pf04135	55505	ENSG0000	NOP10	15	Ribosome		rRNA mod	5								High	High	High	High	High	High	1	
FALSE	Medium	Master Prc	Q02556	Interferon	0.036	1.338	2	1	2	1	426	48.3	6.77	1.83	1	cell differe	cytoplasm;	DNA bindi	Pf00605, P	3394	ENSG0000	IRF8	16	Pertussis	Developme	Interferon	8								High	High	High	High	High	High	1	
FALSE	Medium	Master Prc	Q9Y6J9	TAF6-like F	0.015	1.74	1	1	1	1	622	67.8	8.97	0	1	cell organi	nucleus	catalytic a	Pf02969, P	10629	ENSG0000	TAF6L	11	Basal trans		HATs acety	5								High	High	High	High	High	High	1	
FALSE	High	Master Prc	P34931	Heat shock	0	170.028	29	17	94	1	641	70.3	6.02	233.92	17	cell organi	cytosol;mi	nucleotide	Pf00012, P	3305	ENSG0000	HSPA1L	6; CHR_HS	Influenza A	Parkin-Ubi	Attenuatio	20								High	High	High	High	High	High	1	
FALSE	High	Master Prc	Q06546	GA-binding	0.002	3.789	3	1	1	1	454	51.3	4.97	2.75	1	cell differe	nucleus	DNA bindi	Pf00178, P	2551	ENSG0000	GABPA	21		Energy Me	Transcripti	8								High	High	High	High	High	High	1	
FALSE	High	Master Prc	Q8WTS6	Histone-lys	0.003	2.929	2	1	1	1	366	40.7	4.63	1.64	1	cell organi	chromosor	catalytic a	Pf00856	80854	ENSG0000	SETD7	4	Lysine deg	Histone M	PKMTs me	6								High	High	High	High	High	High	1	
FALSE	High	Master Prc	P49366-1	deoxyhypu	0.002	3.702	6	2	2	2	369	40.9	5.36	0	2	cell organi	cytoplasm;	catalytic a	Pf01916	1725	ENSG0000	DHPS	19			Hypusine s	4								High	High	High	High	High	High	1	
FALSE	Medium	Master Prc	P51784	Ubiquitin c	0.012	1.969	1	1	1	1	963	109.7	5.45	1.75	1	metabolic	chromosor	catalytic a	Pf00443, P	8237	ENSG0000	USP11	X			Associatio	7								High	High	High	High	High	High	1	
FALSE	High	Master Prc	Q8N4V1	Membrane	0	6.907	18	1	1	1	131	14.7	9.16	2.78	1	transport	endoplasm	transporte	Pf10270	93380	ENSG0000	MMGT1	X			Miscellane	2								High	High	High	High	High	High	1	
FALSE	High	Master Prc	O00178	GTP-bindir	0.003	2.849	2	1	1	1	669	72.4	8.34	2.25	1	metabolic	cytoplasm;	catalytic a	Pf00009, P	9567	ENSG0000	GTPBP1	22				0								High	High	High	High	High	High	1	
FALSE	Low	Master Prc	P06127	T-cell surfa	0.053	1.171	4	1	1	1	495	54.5	8.21	2.02	1	cell prolife	membrane	protein bir	Pf00530	921	ENSG0000	CD5	11	Hematopo			1								High	High	High	High	High	High	1	[Q205]
FALSE	High	Master Prc	Q9UJ68-1	Mitochond	0.01	2.09	4	1	1	1	235	26.1	8.09	0	1	metabolic	cytoplasm;	catalytic a	Pf01625	4482	ENSG0000	MSRA	8		Sulindac M	Protein rep	3								High	High	High	High	High	High	1	
FALSE	High	Master Prc	Q8TD55	pleckstrin l	0.001	4.37	5	1	1	1	490	53.3	5.43	2.99	1	transport	extracellul		Pf00169, P	80301	ENSG0000	PLEKHO2	15	Cytokine-c		Neutrophil	4								High	High	High	High	High	High	1	
FALSE	Medium	Master Prc	A6NF01	Putative nu	0.037	1.331	1	1	1	1	834	83	9.95	0	1	transport	nucleus	structural	Pf15229	10028854		POM121B	7				0								High	High	High	High	High	High	1	
FALSE	High	Master Prc	Q7LGC8	Carbohydr	0.003	2.948	4	1	1	1	479	54.7	8.59	2.32	1	metabolic	Golgi;mem	catalytic a	Pf00685	9469	ENSG0000	CHST3	10	Glycosami	Metapathw	Defective (	11								High	High	High	High	High	High	1	
FALSE	Low	Master Prc	Q5VST9	Obscurin [(	0.069	1.051	0	1	1	1	7968	867.9	5.99	1.62	1	cell differe	cytoplasm;	catalytic a	Pf00041, P	84033	ENSG0000	OBSCN	1			G alpha (1:	11								High	High	High	High	High	High	1	[Q5542];
FALSE	Medium	Master Prc	Q9BVG9	phosphatic	0.029	1.441	5	1	1	1	487	56.2	6.25	0	1	metabolic	endoplasm	catalytic a	Pf03034	81490	ENSG0000	PTDSS2	11	Glyceroph	Kennedy p	Synthesis c	8								High	High	High	High	High	High	1	
FALSE	High	Master Prc	Q8NCN4	E3 ubiquiti	0.001	4.272	2	1	1	1	708	77.1	9.1	0	1	metabolic	nucleus	catalytic a	Pf00097, P	254225	ENSG0000	RNF169	11				0								High	High	High	High	High	High	1	
FALSE	High	Master Prc	P10619	lysosomal	0	10.223	7	2	2	2	480	54.4	6.61	5.7	2	metabolic	endoplasm	catalytic a	Pf00450	5476	ENSG0000	CTSA	20	Lysosome;		Sialic acid	17								High	High	High	High	High	High	1	
FALSE	High	Master Prc	Q8IZ69	tRNA (urac	0	8.683	6	2	2	2	625	68.7	7.94	0	2	metabolic		catalytic a	Pf00076, P	27037	ENSG0000	TRMT2A	22				0								High	High	High	High	High	High	1	
FALSE	High	Master Prc	Q6ZSZ5	Rho guanir	0	7.728	2	2	2	2	1173	130.7	7.08	3.12	2	cell organi	cytoplasm;		Pf00169, P	23370	ENSG0000	ARHGEF18	19			G alpha (1:	14								High	High	High	High	High	High	1	
FALSE	High	Master Prc	Q5VV41-1	rho guanin	0.003	2.942	2	1	1	1	709	80.1	7.36	2.77	1	cellular co	cytoplasm;	protein bir	Pf00169, P	27237	ENSG0000	ARHGEF16	1			G alpha (1:	11								High	High	High	High	High	High	1	
FALSE	Low	Master Prc	Q6ZU69	Protein FA	0.062	1.108	1	1	1	1	1335	148	8.29	0	1		membrane		Pf14650, P	259308	ENSG0000	FAM205A	9				0								High	High	High	High	High	High	1	[Q560;
FALSE	High	Master Prc	Q9UIG0	Tyrosine-p	0	15.822	3	3	3	3	1483	170.8	8.48	5.84	3	cell organi	chromosor	catalytic a	Pf00439, P	9031	ENSG0000	BAZ1B	7			Recruitme	8								High	High	High	High	High	High	1	
FALSE	High	Master Prc	O00115	Deoxyribo	0.002	4.034	3	1	1	1	360	39.6	8.05	1.7	1	cell death;	vacuole	catalytic a	Pf03265	1777	ENSG0000	DNASE2	19	Lysosome		Lysosome '	6								High	High	High	High	High	High	1	
FALSE	Medium	Master Prc	Q8TD19	Serine/thre	0.039	1.284	1	1	1	1	979	107.1	5.74	2.24	1	cell divisio	cytoplasm;	catalytic a	Pf00069, P	91754	ENSG0000	NEK9	14			Activation	7								High	High	High	High	High	High	1	[Q524;
FALSE	High	Master Prc	P22830-1	Ferrochela	0.004	2.808	3	1	1	1	423	47.8	8.73	1.86	1	metabolic	membrane	catalytic a	Pf00762	2235	ENSG0000	FECH	18	Metabolic	Heme Bios	Heme bios	6								High	High	High	High	High	High	1	
FALSE	Low	Master Prc	O95396	Adenylyltr	0.068	1.056	5	1	1	1	460	49.6	6.21	0	1	metabolic	cytoplasm;	catalytic a	Pf00581, P	27304	ENSG0000	MOCS3	20	Sulfur rela		Molybdenu	5								High	High	High	High	High	High	1	[R198;
FALSE	Medium	Master Prc	Q96PV6-2	Isoform 2 (	0.012	1.957	1	1	1	1	800	88.1	9.11	0	1			protein bir	Pf03399	114823	ENSG0000	LENG8	19; CHR_H				0								High	High	High	High	High	High	1	
FALSE	Medium	Master Prc	Q9BQI3-1	Eukaryotic	0.032	1.409	2	1	1	1	630	71.1	5.99	0	1	cell differe	cytoplasm	catalytic a	Pf00069, P	27102	ENSG0000	EIF2AK1	7	Influenza A	Translatior		7								High	High	High	High	High	High	1	
FALSE	High	Master Prc	Q92828	Coronin-2A	0.002	3.556	3	2	2	1	525	59.7	8.05	1.7	2	cell organi	cytoskelet	protein bir	Pf00400, P	7464	ENSG0000	CORO2A	9				0								High	High	High	High	High	High	1	
FALSE	High	Master Prc	Q09019	Dystrophia	0.005	2.576	3	1	4	1	674	70.4	7.24	0	1	metabolic	nucleus	protein bir	Pf00400	1762	ENSG0000	DMWD	19				0								High	High	High	High	High	High	1	[Q664]
FALSE	High	Master Prc	P51809	vesicle-ass	0	8.326	22	3	3	3	220	24.9	8.6	6.37	3	cell organi	cell surface	protein bir	Pf00957, P	6845	ENSG0000	VAMP7	X|Y; X	SNARE inte		Cargo reco	13								High	High	High	High	High	High	1	
FALSE	High	Master Prc	Q9Y2T7	Y-box-bind	0	55.984	19	6	21	2	364	38.5	10.8	43.44	6	metabolic	cytoplasm;	DNA bindi	Pf00313	51087	ENSG0000	YBX2	17				0								High	High	High	High	High	High	1	
FALSE	Low	Master Prc	Q53R41	FAST kinas	0.063	1.099	2	1	1	1	847	97.3	7.74	0	1	metabolic	mitochond	catalytic a	Pf06743, P	79675	ENSG0000	FASTKD1	2				0								High	High	High	High	High	High	1	[Q701]
FALSE	Medium	Master Prc	P52657	transcripti	0.02	1.609	6	1	1	1	109	12.4	6.62	1.97	1	cell organi	nucleus	protein bir	Pf02268, P	2958	ENSG0000	GTF2A2	15	Basal trans	Eukaryotic	RNA polym	19								High	High	High	High	High	High	1	
FALSE	High	Master Prc	Q12873-1	Chromodo	0	6.023	1	2	2	1	2000	226.5	7.3	3.7	2	cell organi	cytoplasm;	catalytic a	Pf00176, P	1107	ENSG0000	CHD3	17			SUMOylati	25								High	High	High	High	High	High	1	
FALSE	High	Master Prc	Q9Y2H6	Fibronectir	0.002	3.706	1	1	1	1	1198	131.8	6.71	0	1		cytosol;Go	protein bir	Pf00041	22862	ENSG0000	FNDC3A	13				0								High	High	High	High	High	High	1	
FALSE	High	Master Prc	O43670-1	BUB3-inter	0.003	3.124	3	1	1	1	478	50.7	9.1	3.11	1	cell divisio	chromosor	DNA bindi		7756	ENSG0000	ZNF207	17				0								High	High	High	High	High	High	1	
FALSE	High	Master Prc	P0CG39	POTE anky	0	36.807	6	6	43	1	1038	117.3	5.97	103.17	6			protein bir	Pf00022, P	653781	ENSG0000	POTEJ	2				0								High	High	High	High	High	High	1	
FALSE	Medium	Master Prc	Q86UP3-1	Zinc finger	0.017	1.679	0	1	1	1	3567	393.5	6.37	0	1	metabolic	nucleus	DNA bindi	Pf00046, P	79776		ZFHX4	8		Mesoderm		2								High	High	High	High	High	High	1	
FALSE	High	Master Prc	Q6PID6	Tetratricop	0.002	3.832	4	1	1	1	262	29.4	5.44	2.47	1			protein bir	Pf07719, P	23548	ENSG0000	TTC33	5				0								High	High	High	High	High	High	1	
FALSE	High	Master Prc	Q9BY43	Charged m	0	5.858	6	1	1	1	222	25.1	4.7	1.86	1	cell commu	cytoplasm;	protein bir	Pf03357	29082	ENSG0000	CHMP4A	14	Endocytosi		Budding ar	12								High	High	High	High	High	High	1	
FALSE	High	Master Prc	Q96B36	Proline-ricl	0	5.772	9	2	2	2	256	27.4	4.75	2.47	2	cell organi	cytoplasm;	protein bir		84335	ENSG0000	AKT1S1	19	mTOR sign	Target Of I	HSF1-depe	22								High	High	High	High	High	High	1	
FALSE	Low	Master Prc	P01023	alpha-2-ma	0.071	1.036	1	1	1	1	1474	163.2	6.46	0	1	cell differe	cytosol;ext	enzyme re	Pf00207, P	2	ENSG0000	A2M	12	Compleme	Compleme	Platelet de	17								High	High	High	High	High	High	1	
FALSE	High	Master Prc	Q8IUD2-1	ELKS/Rab6	0	8.92	4	4	4	4	1116	128	5.97	2.52	4	developme	cytoplasm;	protein bir	Pf01496, P	23085	ENSG0000	ERC1	12; CHR_H	NF-kappa I			1								High	High	High	High	High	High	1	
FALSE	High	Master Prc	Q9UPN4	centrosom	0	18.928	5	5	6	5	1083	122.1	8.69	13.62	5	cell differe	cytoplasm;	catalytic a	Pf01576, P	22994	ENSG0000	AZI1; CEP1	17			Regulation	16								High	High	High	High	High	High	1	

FALSE	High	Master Prc	O95394	phosphoac	0	7.904	8	4	4	4	542	59.8	6.25	1.82	4	metabolic	cytosol	catalytic a	Pf00408, P	5238	ENSG0000	PGM3	6	Amino sug	Glucuronic	Synthesis c	9								High	High	High	High	High	High	1	
FALSE	High	Master Prc	P32970	CD70 antig	0.003	2.84	6	1	1	1	193	21.1	8.53	1.72	1	cell comm	membrane	protein bir	Pf00229	970	ENSG0000	CD70	19	Cytokine-c		TNFs bind	5								High	High	High	High	High	High	1	
FALSE	High	Master Prc	Q8WY22	BRI3-bindi	0	5.726	10	2	2	2	251	27.8	9.44	1.7	2		membrane		Pf14965	140707	ENSG0000	BRI3BP	12				0								High	High	High	High	High	High	1	
FALSE	High	Master Prc	Q4KMQ2-1	Anoctamin	0.002	3.722	3	2	2	2	910	106.1	7.77	2.53	2	cell organi	cell surface	protein bir	Pf04547	196527	ENSG0000	ANO6	12			Neutrophil	6								High	High	High	High	High	High	1	
FALSE	High	Master Prc	Q9HB90	ras-related	0.002	3.917	3	1	1	1	399	44.2	5.1	2.55	1	cell comm	cytoplasm;	catalytic a	Pf00025, P	64121	ENSG0000	RRAGC	1	mTOR sign	Target Of I	Energy dep	17								High	High	High	High	High	High	1	
FALSE	High	Master Prc	P61978-2	Isoform 2 c	0	242.785	65	28	139	1	464	51	5.33	363.05	28	metabolic	cytoplasm;	DNA bindi	Pf00013, P	3190	ENSG0000	HNRNPK	9		mRNA Pro		1								High	High	High	High	High	High	1	
FALSE	Medium	Master Prc	P18850	Cyclic AMF	0.018	1.661	2	1	1	1	670	74.5	8.22	0	1	metabolic	cytosol;en	DNA bindi	Pf00170, P	22926	ENSG0000	ATF6	1	Protein prc	Sterol Reg	ATF4 activ	12								High	High	High	High	High	High	1	
FALSE	High	Master Prc	O15239	NADH deh	0.001	4.469	19	1	1	1	70	8.1	8.84	3.73	1	cell organi	cytosol;me	catalytic a		4694	ENSG0000	NDUFA1	X	Metabolic	Electron Tr	Complex I	12								High	High	High	High	High	High	1	
FALSE	Medium	Master Prc	Q8IWB1	Inositol 1,4	0.032	1.397	1	1	1	1	547	62	5.88	0	1		membrane		Pf03281	85450	ENSG0000	ITPRIP	10				0								High	High	High	High	High	High	1	
FALSE	High	Master Prc	Q86XA9	HEAT repe	0.008	2.199	1	1	1	1	2040	221.9	6.58	2.52	1					25938		HEATR5A	14				0								High	High	High	High	High	High	1	
FALSE	High	Master Prc	P48029-1	Sodium- ar	0	4.836	2	1	1	1	635	70.5	6.42	1.97	1	metabolic	membrane	transporte	Pf00209	6535	ENSG0000	SLC6A8	X		NRF2 path	Creatine m	5								High	High	High	High	High	High	1	
FALSE	Medium	Master Prc	P01008	Antithroml	0.036	1.344	2	1	1	1	464	52.6	6.71	0	1	coagulatio	extracellul	enzyme re	Pf00079	462	ENSG0000	SERPINC1	1	Compleme	Compleme	Post-transl	10								High	High	High	High	High	High	1	
FALSE	High	Master Prc	O14965	Aurora kin	0	6.171	5	1	1	1	403	45.8	9.39	4.39	1	cell divisio	cytoplasm;	catalytic a	Pf00069, P	6790		AURKA	20	Oocyte me	Gastric Ca	FBXL7 dow	27								High	High	High	High	High	High	1	
FALSE	High	Master Prc	O75717	WD repeat	0	12.296	5	4	5	4	1129	125.9	5.62	4.31	4		cytoplasm;	DNA bindi	Pf00400, P	11169	ENSG0000	WDHD1	14		Mesoderm		1								High	High	High	High	High	High	1	
FALSE	High	Master Prc	Q13308-1	Inactive ty	0	11.169	4	2	2	2	1070	118.3	7.09	8.8	2	cell differe	membrane	catalytic a	Pf00047, P	5754	ENSG0000	PTK7	6				0								High	High	High	High	High	High	1	
FALSE	High	Master Prc	Q8WU90	Zinc finger	0	9.691	9	4	4	4	426	48.6	5.31	1.68	4	regulation	cytoplasm;	metal ion I	Pf00642	55854	ENSG0000	ZC3H15	2				0								High	High	High	High	High	High	1	
FALSE	Medium	Master Prc	Q96MX6	WD repeat	0.028	1.444	7	1	1	1	357	39.7	8.09	0	1	cell death		protein bir		116143	ENSG0000	WDR92	2				0								High	High	High	High	High	High	1	
FALSE	Low	Master Prc	Q7Z478	ATP-depen	0.072	1.025	1	1	1	1	1369	155.1	8.09	0	1	metabolic	cytoplasm;	catalytic a	Pf00270, P	54505	ENSG0000	DHX29	5				0								High	High	High	High	High	High	1	
FALSE	High	Master Prc	O60825-1	6-phospho	0.003	2.913	3	1	1	1	505	58.4	8.38	0	1	metabolic	cytoplasm;	catalytic a	Pf00300, P	5208	ENSG0000	PFKFB2	1	Fructose a	Focal Adhe	Glycolysis	8								High	High	High	High	High	High	1	
FALSE	Medium	Master Prc	Q15751	Probable E	0.012	1.991	0	1	1	1	4861	531.9	6.04	0	1	cell differe	cytoplasm;	catalytic a	Pf00400, P	8925	ENSG0000	HERC1	15	Ubiquitin r		Antigen pr	5								High	High	High	High	High	High	1	
FALSE	Medium	Master Prc	Q9Y5K3	Choline-ph	0.034	1.371	2	1	1	1	369	41.9	6.4	0	1	metabolic	cytoplasm;	catalytic a	Pf01467	9468	ENSG0000	PCYT1B	X	Glyceroph	Kennedy p	Synthesis c	10								High	High	High	High	High	High	1	
FALSE	Medium	Master Prc	Q9Y6J6	Potassium	0.034	1.369	13	1	1	1	123	14.5	5.83	0	1	regulation	cell surface	protein bir	Pf02060	9992	ENSG0000	KCNE2	21	Gastric aci		Phase 3 - r	5								High	High	High	High	High	High	1	[K120]
FALSE	Medium	Master Prc	P46976-1	Glycogenir	0.015	1.759	3	1	1	1	350	39.4	5.53	0	1	metabolic	cytosol;ext	catalytic a	Pf01501	2992	ENSG0000	GYG1	3	Starch and	Insulin Sig	Neutrophil	22								High	High	High	High	High	High	1	
FALSE	Medium	Master Prc	P04217	Alpha-1B-g	0.026	1.478	2	1	1	1	495	54.2	5.86	0	1	transport	extracellul	protein bir	Pf00047, P	1	ENSG0000	A1BG	19			Platelet de	7								High	High	High	High	High	High	1	
FALSE	High	Master Prc	Q96GC5-1	39S riboso	0.003	3.379	5	1	2	1	212	23.9	8.98	0	1	cell organi	membrane	protein bir	Pf00338	51642	ENSG0000	MRPL48	11			Mitochonc	6								High	High	High	High	High	High	1	
FALSE	High	Master Prc	Q96G28	cilia- and f	0.002	4.035	6	2	2	2	342	39.4	4.97	2.28	2		cytoplasm;	protein bir	Pf11527	112942	ENSG0000	CCDC104;	2				0								High	High	High	High	High	High	1	
FALSE	High	Master Prc	Q7L4I2-1	Arginine/se	0.002	4.073	2	1	1	1	434	50.5	11.33	2.04	1			protein bir	Pf15477	65117	ENSG0000	RSRC2	12				0								High	High	High	High	High	High	1	
FALSE	High	Master Prc	Q96GN5-1	cell divisio	0.003	3.32	4	2	2	2	454	52.2	5.74	0	2	cell divisio	cytoplasm;	protein bir	Pf10497	55536	ENSG0000	CDCA7L	7		Sudden Inf		1								High	High	High	High	High	High	1	
FALSE	High	Master Prc	Q5VWQ0	Round spe	0	5.45	2	1	1	1	802	90	8.6	2.71	1		nucleus			54665	ENSG0000	RSBN1	1				0								High	High	High	High	High	High	1	
FALSE	Medium	Master Prc	Q9HAQ2-1	Kinesin-like	0.034	1.371	2	1	1	1	790	89.9	7.17	2.57	1	cell organi	cytoplasm;	catalytic a	Pf00225	64147	ENSG0000	KIF9	3			COPI-depe	8								High	High	High	High	High	High	1	[S559(50);
FALSE	High	Master Prc	P11441	Ubiquitin-l	0.002	3.581	6	1	1	1	157	17.8	8.66	2.74	1	metabolic	cytoplasm;	catalytic a	Pf00240, P	8266	ENSG0000	UBL4A	X				0								High	High	High	High	High	High	1	
FALSE	High	Master Prc	Q92817	Envoplakin	0	6.427	1	2	2	2	2033	231.5	6.96	4.06	2	cell death;	cytoplasm;	protein bir	Pf00681, P	2125	ENSG0000	EVPL	17			Formation	3								High	High	High	High	High	High	1	
FALSE	High	Master Prc	Q06265-1	Exosome c	0.002	3.566	4	2	4	2	439	48.9	5.29	3.77	2	metabolic	chromosor	catalytic a	Pf01138, P	5393	ENSG0000	EXOSC9	4	RNA degra		Major path	15								High	High	High	High	High	High	1	
FALSE	Medium	Master Prc	Q9UIM3	FK506-binc	0.011	2.024	3	1	1	1	349	38.2	5.5	0	1	regulation	cytosol	protein bir	Pf00515, P	63943	ENSG0000	FKBPL	6; CHR_HS			The role of	5								High	High	High	High	High	High	1	
FALSE	High	Master Prc	P01137	Transformi	0.006	2.351	2	1	1	1	390	44.3	8.53	2.41	1	cell death;	cell surface	enzyme re	Pf00019, P	7040		TGFB1	19	Chronic my	MicroRNA	SMAD2/3 I	98								High	High	High	High	High	High	1	
FALSE	High	Master Prc	Q9P253	Vacuolar p	0.007	2.309	1	1	1	1	973	110.1	6.07	0	1	cell organi	endosome	catalytic a	Pf00637, P	57617	ENSG0000	VPS18	15				0								High	High	High	High	High	High	1	
FALSE	High	Master Prc	Q5VU43-2	Isoform 2 c	0.009	2.107	1	1	1	1	1132	128.4	5.11	0	1	cell organi	cytoplasm;	metal ion I	Pf02463, P	9659; 653		PDE4DIP; I	1				0								High	High	High	High	High	High	1	
FALSE	Low	Master Prc	Q8IWV8	E3 ubiquiti	0.067	1.069	1	1	1	1	1755	200.4	6.24	0	1	cell organi	chromosor	catalytic a	Pf02207, P	23304	ENSG0000	UBR2	6			Antigen pr	4								High	High	High	High	High	High	1	
FALSE	High	Master Prc	Q9NP72	Ras-relatec	0	6.766	20	3	3	3	206	23	5.24	0	3	cell organi	cytosol;Go	catalytic a	Pf00025, P	22931	ENSG0000	RAB18	10			COPI-indep	13								High	High	High	High	High	High	1	
FALSE	High	Master Prc	Q8WUX9	Charged m	0	7.949	5	1	1	1	453	50.9	5.35	5.71	1	cell organi	chromosor	protein bir	Pf03357	91782	ENSG0000	CHMP7	8	Endocytosi		Budding ar	12								High	High	High	High	High	High	1	
FALSE	High	Master Prc	O60830-1	Mitochonc	0.003	3.139	13	1	1	1	172	18.3	9.03	2.3	1	transport	membrane	protein bir	Pf02466	10245	ENSG0000	TIMM17B	X			Mitochonc	2								High	High	High	High	High	High	1	
FALSE	Medium	Master Prc	Q9UN70-2	Isoform 2 c	0.026	1.477	2	1	1	1	863	94.4	5.16	0	1	cell comm	membrane	metal ion I	Pf00028, P	5098	ENSG0000	PCDHGC3	5				0								High	High	High	High	High	High	1	[Q810;
FALSE	High	Master Prc	O75530	Polycomb	0.003	2.926	3	1	2	1	441	50.2	7.03	0	1	cell organi	chromosor	catalytic a	Pf00400	8726	ENSG0000	EED	11		Histone M	Regulation	23								High	High	High	High	High	High	1	
FALSE	High	Master Prc	Q8NHZ8	Anaphase-	0.004	2.694	14	1	1	1	85	9.8	6.81	2.54	1	cell divisio	cytosol;nu	protein bir	Pf10471	246184	ENSG0000	CDC26	9	Cell cycle;		APC/C:Cdc	37								High	High	High	High	High	High	1	
FALSE	High	Master Prc	Q9BV68-1	E3 ubiquiti	0	5.833	10	1	1	1	326	35.6	5.72	3.13	1	metabolic	cytoplasm;	catalytic a	Pf00097, P	55658		RNF126	19			Antigen pr	4								High	High	High	High	High	High	1	
FALSE	High	Master Prc	Q9UJU6-3	Isoform 3 c	0	26.719	21	7	8	1	439	49	5.05	20.7	7	cell organi	cytoplasm;	enzyme re	Pf00018, P	28988	ENSG0000	DBNL	7		T-Cell anti		1								High	High	High	High	High	High	1	
FALSE	High	Master Prc	Q9BRT9	DNA replic	0.003	3.112	6	1	1	1	223	26	4.98	0	1	cell prolife	cytoplasm;	catalytic a	Pf05916	84296	ENSG0000	GINS4	8			Unwinding	7								High	High	High	High	High	High	1	
FALSE	Medium	Master Prc	Q4LE39-1	AT-rich int	0.024	1.531	1	1	1	1	1312	147.7	5.12	0	1	cell organi	cytoplasm;	catalytic a	Pf00855, P	51742	ENSG0000	ARID4B	1		Pathways	HDACs dea	8								High	High	High	High	High	High	1	
FALSE	High	Master Prc	Q9HBH1	Peptide de	0.003	3.146	4	1	1	1	243	27	9.16	0	1	metabolic	mitochond	catalytic a	Pf01327	64146	ENSG0000	PDF	16				0								High	High	High	High	High	High	1	
FALSE	High	Master Prc	Q53HL2	Borealin [C	0.003	3.19	4	1	1	1	280	31.3	9.86	2.04	1	cell divisio	chromosor	protein bir	Pf10444, P	55143	ENSG0000	CDCA8	1			Amplificati	21								High	High	High	High	High	High	1	
FALSE	Low	Master Prc	O14508	Suppressor	0.058	1.13	4	1	1	1	198	22.2	8.69	0	1	metabolic	cytoplasm;	enzyme re	Pf00017, P	8835	ENSG0000	SOCS2	12	Prolactin s	Leptin sign	Interleukin	16								High	High	High	High	High	High	1	
FALSE	High	Master Prc	O75629	Protein CR	0	9.609	10	1	1	1	220	24.1	7.59	5.43	1	cell prolife	extracellul	protein bir	Pf13883	8804	ENSG0000	CREG1	1		Senescenc	Neutrophil	4								High	High	High	High	High	High	1	
FALSE	Low	Master Prc	O00471	Exocyst co	0.062	1.101	1	1	1	1	708	81.8	6.71	0	1	transport	cytoplasm;	protein bir	Pf03938, P	10640	ENSG0000	EXOC5	14			Translocat	10								High	High	High	High	High	High	1	
FALSE	Medium	Master Prc	A6NI28	Rho GTPas	0.033	1.384	1	1	1	1	874	98.5	7.99	0	1	regulation		enzyme re	Pf00018, P	143872	ENSG0000	ARHGAP42	11			Rho GTPas	3								High	High	High	High	High	High	1	
FALSE	High	Master Prc	Q9UBG0	C-type mai	0.006	2.537	1	1	1	1	1479	166.6	5.83	1.78	1	cell differe	membrane	protein bir	Pf00040, P	9902	ENSG0000	MRC2	17	Tuberculos		Cross-pres	7								High	High	High	High	High	High	1	
FALSE	Medium	Master Prc	Q8WUA2	Peptidyl-pr	0.015	1.763	3	1	1	1	492	57.2	5.92	0	1	metabolic	cytosol;nu	catalytic a	Pf00076, P	85313	ENSG0000	PPIL4	6			mRNA Spli	4								High	High	High	High	High	High	1	
FALSE	High	Master Prc	O43716	Glutamyl-t	0	5.581	14	2	2	2	136	15.1	5.05	2.64	2	cell organi	mitochond	catalytic a	Pf02686	283459	ENSG0000	GATC	12	Aminoacyl			2								High	High	High	High	High	High	1	
FALSE	High	Master Prc	Q9Y6I3	epsin-1 [O	0	5.057	4	1	2	1	576	60.3	4.83	5.83	1	cell organi	cytoplasm;	protein bir	Pf01417	29924	ENSG0000	EPN1	19	Endocytosi	VEGFA-VE	Cargo reco	11								High	High	High	High	High	High	1	
FALSE	High	Master Prc	Q8TB36	ganglioside	0	5.489	4	1	1	1	358	41.3	8.34	2	1	cell comm	cytoplasm;	catalytic a	Pf00043, P	54332	ENSG0000	GDAP1	8				0								High	High	High	High	High	High	1	
FALSE	High	Master Prc	Q8NEJ9	neuroguidi	0.003	2.98	3	1	1	1	315	35.9	9.57	0	1	metabolic	chromosor	protein bir	Pf04000	25983	ENSG0000	NGDN	14				0								High	High	High	High	High	High	1	
FALSE	High	Master Prc	Q05513-1	Protein kin	0.002	3.555	3	2	2	1	592	67.6	5.77	3.4	2	cell growth	cytoplasm;	catalytic a	Pf00069, P	5590	ENSG0000	PRKCZ	1	Insulin resi	Type II dial	VEGFR2 m	40								High	High	High	High	High	High	1	
FALSE	Low	Master Prc	P38570	Integrin al	0.059	1.124	1	1	1	1	1179	130.1	5.74	0	1	cell organi	membrane	metal ion I	Pf00092, P	3682	ENSG0000	ITGAE	17	Regulation	Integrin-m	Integrin ce	6								High	High	High	High	High	High	1	
FALSE	High	Master Prc	O00194	ras-related	0	8.909	13	3	3	2	218	24.6	5.52	1.73	3	regulation	Golgi;mem	catalytic a	Pf00009, P	5874	ENSG0000	RAB27B	18	Pancreatic	Deregulati	Platelet de	13								High	High	High	High	High	High	1	
FALSE	High	Master Prc	Q15746-1	Myosin ligl	0.006	2.412	1	1	1	1	1914	210.6	6.15	0	1	cell organi	cytoplasm;	catalytic a	Pf00041, P	4638	ENSG0000	MYLK	3	Focal adhe	MicroRNA	RHO GTPa	19								High	High	High	High	High	High	1	
FALSE	High	Master Prc	Q5T3I0	G patch do	0	10.254	7	3	4	3	446	50.4	9.63	7.99	3			protein bir	Pf01585	54865		GPATCH4	1				0								High	High	High	High	High	High	1	
FALSE	Medium	Master Prc	Q8N138	ORM1-like	0.026	1.481	7	1	1	1	153	17.5	9.64	0	1	cellular ho	endoplasm	protein bir	Pf04061	94103	ENSG0000	ORMDL3	17			Neutrophil	7								High	High	High	High	High	High	1	
FALSE	Medium	Master Prc	O94886	CSC1-like p	0.016	1.723	1	1	1	1	807	92.1	7.27	2.55	1	transport	membrane	transporte	Pf02714, P	9725	ENSG0000	TMEM63A	1			Neutrophil	3								High	High	High	High	High	High	1	
FALSE	High	Master Prc	Q9UPY3	endoribon	0.006	2.529	1	1	1	1	1922	218.5	5.68	0	1	cell organi	cytoplasm;	catalytic a	Pf00270, P	23405	ENSG0000	DICER1	14	MicroRNA	miRNA Bic	MicroRNA	9								High	High	High	High	High	High	1	
FALSE	High	Master Prc	Q9BVV7	Mitochonc	0.001	4.202	4	1	1	1	248	28.2	9.7	1.94	1	cell organi	membrane	protein bir	Pf08294, P	29090	ENSG0000	TIMM21	18			Mitochonc	2								High	High	High	High	High	High	1	
FALSE	High	Master Prc	Q5JTD0-1	Tight junct	0.002	3.814	2	1	1	1	557	61.8	5.97	1.75	1	cell organi	cytoplasm;	protein bir	Pf15453	93643	ENSG0000	TJAP1	6	Tight junct			1								High	High	High	High	High	High	1	
FALSE	High	Master Prc	Q9BQE5	apolipopro	0	4.866	3	1	2	1	337	37.1	6.74	1.92	1	defense re	cytoplasm;	protein bir	Pf05461	23780		APOL2	22				0								High	High	High	High	High	High	1	
FALSE	Low	Master Prc	Q9BWM5	Zinc finger	0.051	1.185	2	1	1	1	594	67.1	7.97	0	1	metabolic	nucleus	DNA bindi	Pf00096, P	55659	ENSG0000	ZNF416	19			Generic Tr	3								High	High	High	High	High	High	1	[Q309;
FALSE	High	Master Prc	Q8N201	integrator	0	4.592	1	1	1	1	2190	244.1	6.13	0	1	metabolic	membrane		Pf12432	26173	ENSG0000	INTS1	7			RNA polym	3								High	High	High	High	High	High	1	
FALSE	High	Master Prc	Q96N21-1	AP-4 comp	0.009	2.133	2	1	1	1	525	55.1	7.03	0	1		cytoplasm;	protein bir		146705	ENSG0000	ENTHD2; T	17				0								High	High	High	High	High	High	1	
FALSE	High	Master Prc	Q12841	Follistatin-	0	6.155	4	1	1	1	308	35	5.52	2.87	1	metabolic	extracellul	metal ion I	Pf00050, P	11167	ENSG0000	FSTL1	3			Post-transl	7								High	High	High	High	High	High	1	
FALSE	Medium	Master Prc	Q8N960	Centrosom	0.036	1.341	2	1	1	1	986	112.6	6.25	0	1	cell differe	cytoplasm;	catalytic a	Pf00769, P	153241	ENSG0000	CEP120	5				0								High	High	High	High	High	High	1	[R592]
FALSE	High	Master Prc	Q9UHI6	Probable A	0	9.86	5	3	3	3	824	92.2	6.95	3.63	3	cell organi	cytoplasm;	catalytic a	Pf00270, P	11218	ENSG0000	DDX20	1	RNA trans	mRNA Pro	snRNP Ass	5								High	High	High	High	High	High	1	
FALSE	High	Master Prc	P46939	Utrophin [	0	7.457	1	2	2	1	3433	394.2	5.33	5.25	2	cell organi	cytoplasm;	metal ion I	Pf00307, P	7402	ENSG0000	UTRN	6		Primary Fc		1								High	High	High	High	High	High	1	
FALSE	High	Master Prc	Q5J8M3-1	ER membr	0.002	3.756	9	1	1	1	183	20.1	8.62	1.96	1	cell death;	membrane		Pf06417	51234	ENSG0000	EMC4	15				0								High	High	High	High	High	High	1	

FALSE	High	Master Prc	Q14157-1	Isoform 2 (	0	119.203	28	18	33	1	983	103.9	6.98	88.63	18	regulation	cytoplasm	protein bir	Pf00627, P	9898	ENSG0000	UBAP2L	1				0	High	High	High	High	High	High	1	
FALSE	High	Master Prc	Q13472-1	DNA topoi	0.007	2.3	1	1	1	1	1001	112.3	8.34	1.64	1	metabolic	chromosor	catalytic a	Pf01131, P	7156	ENSG0000	TOP3A	17; CHR_H	Fanconi an		G2/M DNA	27	High	High	High	High	High	High	1	
FALSE	High	Master Prc	Q9ULC3	Ras-relatec	0.002	3.831	4	1	1	1	237	26.6	6.6	2.3	1	cell organi	cytoplasm;	catalytic a	Pf00009, P	51715	ENSG0000	RAB23	6			RAB geran	3	High	High	High	High	High	High	1	
FALSE	High	Master Prc	Q9HC35	Echinoderr	0	5.748	1	1	1	1	981	108.8	6.4	1.73	1		cytoplasm;	protein bir	Pf00400, P	27436	ENSG0000	EML4	2	Non-small			1	High	High	High	High	High	High	1	
FALSE	Medium	Master Prc	Q96RL7	Vacuolar p	0.038	1.316	0	1	1	1	3174	360	6.33	2.12	1	metabolic	cytosol	protein bir	Pf06650, P	23230	ENSG0000	VPS13A	9				0	High	High	High	High	High	High	1	[T631(99);
FALSE	High	Master Prc	P82979	SAP domai	0	8.323	16	3	3	3	210	23.7	6.42	5.17	3	metabolic	nucleus	DNA bindi	Pf02037	84324	ENSG0000	SARNP	12			Cleavage o	9	High	High	High	High	High	High	1	
FALSE	Medium	Master Prc	Q5T0N5-1	Formin-bir	0.012	1.954	1	1	1	1	605	70	6.64	1.65	1	cell organi	cytoplasm;	protein bir	Pf00018, P	54874	ENSG0000	FNBP1L	1			Clathrin-m	3	High	High	High	High	High	High	1	
FALSE	High	Master Prc	Q14790-1	Caspase-8	0.003	3.173	3	1	1	1	479	55.4	5.1	3.13	1	cell death;	cytoplasm;	catalytic a	Pf00656, P	841	ENSG0000	CASP8	2	p53 signali	Nucleotide	CLEC7A/in	78	High	High	High	High	High	High	1	
FALSE	High	Master Prc	Q96QU8	Exportin-6	0.003	3.016	1	1	1	1	1125	128.8	6.35	1.78	1	transport	cytoplasm;	protein bir	Pf03810, P	23214	ENSG0000	XPO6	16				0	High	High	High	High	High	High	1	
FALSE	High	Master Prc	O95229	ZW10 inte	0	5.587	4	1	1	1	277	31.3	5.15	0	1	cell divisio	chromosor	protein bir	Pf09731, P	11130	ENSG0000	ZWINT	10			Amplificati	16	High	High	High	High	High	High	1	
FALSE	High	Master Prc	Q15050	Ribosome	0	9.253	8	2	2	2	365	41.2	10.7	5.84	2	cell differe	chromosor	protein bir	Pf04939	23212	ENSG0000	RRS1	8				0	High	High	High	High	High	High	1	
FALSE	High	Master Prc	O75683	Surfeit locu	0.002	4.079	5	2	2	2	361	41.4	10.64	2.39	2		nucleus	DNA bindi	Pf04935	6838	ENSG0000	SURF6	9; CHR_HG				0	High	High	High	High	High	High	1	
FALSE	High	Master Prc	Q9BV79	Enoyl-[acyl	0	7.948	7	2	2	2	373	40.4	8.76	5.49	2	metabolic	cytoplasm;	catalytic a	Pf00107, P	51102	ENSG0000	MECR	1	Fatty acid	Fatty Acid	Beta oxida	10	High	High	High	High	High	High	1	
FALSE	High	Master Prc	O75828	Carbonyl re	0	12.316	10	2	4	1	277	30.8	6.18	13.64	2	metabolic	cytoplasm;	catalytic a	Pf00106, P	874	ENSG0000	CBR3	21	Metabolic	NRF2 path	Phase I - Fu	7	High	High	High	High	High	High	1	
FALSE	High	Master Prc	Q9BRS2	Serine/thre	0.003	3.243	2	1	1	1	568	65.5	6.19	2.48	1	metabolic	cytoplasm;	catalytic a	Pf01163, P	83732	ENSG0000	RIOK1	6	Ribosome		Major path	5	High	High	High	High	High	High	1	
FALSE	Medium	Master Prc	Q9NQA5	Transient r	0.034	1.365	1	1	1	1	729	82.5	6.46	0	1	cell organi	membrane	metal ion I	Pf00023, P	56302		TRPV5	7	Endocrine	Osteoclast	TRP chann	6	High	High	High	High	High	High	1	[S13(100)]
FALSE	Medium	Master Prc	O14782	Kinesin-like	0.022	1.579	1	1	1	1	793	89.4	8.22	0	1	cellular co	cytoplasm;	catalytic a	Pf00225	3797		KIF3C	2			COPI-depe	14	High	High	High	High	High	High	1	
FALSE	High	Master Prc	Q14728	Major facil	0.003	2.924	3	1	1	1	455	48.3	9.6	0	1	cell death;	membrane	transporte	Pf00083, P	10227	ENSG0000	MFSD10	4				0	High	High	High	High	High	High	1	
FALSE	High	Master Prc	O00400	Acetyl-coe	0.001	4.305	2	1	1	1	549	60.9	7.33	1.75	1	regulation	endoplasm	transporte	Pf13000	9197	ENSG0000	SLC33A1	3	Metabolic	Ganglio Sp	Transport	10	High	High	High	High	High	High	1	
FALSE	High	Master Prc	Q13427	peptidyl-pr	0	13.915	8	5	6	5	754	88.6	10.29	12.67	5	metabolic	cytosol;nu	catalytic a	Pf00160	9360	ENSG0000	PPIG	2				0	High	High	High	High	High	High	1	[S290(100
FALSE	High	Master Prc	Q14696	LRP chaper	0	5.091	9	2	3	2	234	26.1	7.78	5.02	2	metabolic	endoplasm	protein bir	Pf10185	23184	ENSG0000	MESDC2; M	15				0	High	High	High	High	High	High	1	
FALSE	High	Master Prc	Q14746	Conserved	0.002	4.083	1	1	1	1	738	83.2	6.62	2.13	1	cell organi	cytosol;Go	protein bir	Pf06148, P	22796	ENSG0000	COG2	1			COPI-medi	11	High	High	High	High	High	High	1	
FALSE	Medium	Master Prc	Q92968	Peroxisom	0.04	1.276	1	1	1	1	403	44.1	8.05	0	1	cellular co	membrane	protein bir	Pf00018, P	5194	ENSG0000	PEX13	2	Peroxisom			1	High	High	High	High	High	High	1	
FALSE	Medium	Master Prc	Q8WXI7	Mucin-16 [	0.034	1.372	0	1	1	1	14507	1518.2	5.26	0	1	metabolic	extracellul	protein bir	Pf01390, P	94025	ENSG0000	MUC16	19			Defective (	15	High	High	High	High	High	High	1	[T27(50);
FALSE	High	Master Prc	Q8WVJ2	NudC dom	0	5.041	11	1	1	1	157	17.7	5.07	4.18	1	metabolic	chromosor	protein bir	Pf04969	134492	ENSG0000	NUDCD2	5				0	High	High	High	High	High	High	1	
FALSE	High	Master Prc	Q8WUY1	Protein TH	0.003	3.214	6	1	2	1	208	23.9	9.55	3.67	1		extracellul		Pf13279	51337	ENSG0000	THEM6	8				0	High	High	High	High	High	High	1	
FALSE	High	Master Prc	P50583	Bis(5'-nucl	0.001	4.353	10	1	1	1	147	16.8	5.35	3.09	1	cell death;	mitochond	catalytic a	Pf00293	318	ENSG0000	NUDT2	9	Purine met		Detoxificat	5	High	High	High	High	High	High	1	
FALSE	High	Master Prc	Q3V6T2-1	Girdin [OS-	0	5.371	2	2	2	2	1871	215.9	6.21	2.76	2	cell organi	cytoplasm;	protein bir	Pf04111, P	55704	ENSG0000	CCDC88A	2		Mesoderm		1	High	High	High	High	High	High	1	
FALSE	High	Master Prc	Q14966-1	Zinc finger	0	8.655	1	2	2	2	1978	220.5	6.38	0	2	metabolic	cytoplasm;	DNA bindi	Pf00076, P	27332	ENSG0000	ZNF638	2			Transcripti	2	High	High	High	High	High	High	1	
FALSE	Low	Master Prc	Q13901	nuclear nu	0.051	1.182	4	1	1	1	141	16	9.03	2.26	1	cell death;	cytoplasm;	DNA bindi	Pf04000	10438	ENSG0000	C1D	2	RNA degra		Major path	5	High	High	High	High	High	High	1	
FALSE	Medium	Master Prc	P20338	Ras-relatec	0.014	1.843	5	1	1	1	218	24.4	6.07	1.95	1	metabolic	cytoplasm;	catalytic a	Pf00009, P	5867	ENSG0000	RAB4A	1	Endocytosi	VEGFA-VE(	Translocat	20	High	High	High	High	High	High	1	
FALSE	High	Master Prc	Q9Y512	sorting anc	0.006	2.37	3	1	1	1	469	51.9	6.9	0	1	cell organi	cytoplasm;	protein bir	Pf01103, P	25813	ENSG0000	SAMM50	22			Cristae for	5	High	High	High	High	High	High	1	
FALSE	High	Master Prc	Q8ND04	Protein SM	0.006	2.477	1	1	1	1	991	109.6	7.68	1.79	1	metabolic	cytosol	protein bir	Pf10220	55181	ENSG0000	SMG8	17			Nonsense	3	High	High	High	High	High	High	1	
FALSE	High	Master Prc	Q9UEU0	vesicle trar	0	8.59	14	2	2	2	232	26.7	9.04	6.63	2	cell organi	cytosol;en	protein bir	Pf03908, P	10490	ENSG0000	VTI1B	14	SNARE inte		Platelet de	5	High	High	High	High	High	High	1	
FALSE	Low	Master Prc	Q9NWB6	Arginine ar	0.059	1.127	2	1	1	1	273	33.2	10.35	1.67	1	metabolic	mitochond	protein bir	Pf15346	55082	ENSG0000	ARGLU1	13				0	High	High	High	High	High	High	1	
FALSE	Medium	Master Prc	Q969S8-1	Histone de	0.045	1.239	1	1	1	1	669	71.4	5.74	0	1	cell organi	cytoplasm;	catalytic a	Pf00850	83933	ENSG0000	HDAC10	22	Viral carcir	Neural Cre	NOTCH1 Ir	18	High	High	High	High	High	High	1	
FALSE	High	Master Prc	O96017-1	Serine/thre	0.002	4.183	6	2	2	2	543	60.9	5.91	0	2	cell death;	Golgi;nucle	catalytic a	Pf00069, P	11200	ENSG0000	CHEK2	22	Cell cycle;	miRNA Re	Chk1/Chk2	32	High	High	High	High	High	High	1	
FALSE	High	Master Prc	Q8NBJ4	Golgi mem	0	11.82	10	5	9	5	401	45.3	4.97	4.32	5	cell organi	Golgi;mem	protein bir		51280	ENSG0000	GOLM1	9			Post-transl	4	High	High	High	High	High	High	1	
FALSE	High	Master Prc	O95983-1	methyl-Cp(	0.007	2.247	5	1	1	1	291	32.8	5.34	0	1	cell organi	chromosor	catalytic a	Pf01429, P	53615	ENSG0000	MBD3	19			ERCC6 (CSI	20	High	High	High	High	High	High	1	
FALSE	High	Master Prc	Q96D53-1	Atypical ki	0	4.698	4	2	2	2	544	60	7.23	0	2	metabolic	cytoplasm;	catalytic a	Pf03109	79934	ENSG0000	ADCK4; CC	19				0	High	High	High	High	High	High	1	
FALSE	High	Master Prc	Q15334	Lethal(2) g	0.004	2.673	1	1	1	1	1064	115.3	6.29	0	1	cell organi	cytoplasm;	enzyme re	Pf08366	3996	ENSG0000	LLGL1	17; CHR_H	Tight junct			2	High	High	High	High	High	High	1	
FALSE	High	Master Prc	Q8NI27-1	THO comp	0	6.172	2	3	4	3	1593	182.7	8.44	2.54	3	cell differe	nucleus	protein bir	Pf11262, P	57187	ENSG0000	THOC2	X	Spliceosom		Cleavage o	11	High	High	High	High	High	High	1	
FALSE	High	Master Prc	Q9BPX5	actin-relat	0	5.351	12	1	1	1	153	16.9	6.6	5.31	1	cell organi	cytoplasm;	protein bir	Pf04699	81873	ENSG0000	ARPC5L	9	Endocytosi	Pathogenic		8	High	High	High	High	High	High	1	
FALSE	Medium	Master Prc	Q9NX01	thioredoxi	0.011	2.001	7	1	1	1	149	17	6	2.39	1	cell organi	cytosol;nu	protein bir	Pf02966	54957	ENSG0000	TXNL4B	16				0	High	High	High	High	High	High	1	
FALSE	High	Master Prc	Q92995	Ubiquitin c	0.009	2.168	1	1	1	1	863	97.3	5.53	0	1	cell differe	cytosol	catalytic a	Pf00443, P	8975	ENSG0000	USP13	3			Regulation	9	High	High	High	High	High	High	1	
FALSE	High	Master Prc	O60907	F-box-like/	0	8.965	5	2	3	1	577	62.5	6.55	6	2	cell organi	nucleus	DNA bindi	Pf00400, P	6907	ENSG0000	TBL1X	X	Wnt signal	Ectoderm	NOTCH1 Ir	37	High	High	High	High	High	High	1	
FALSE	High	Master Prc	O00244	Copper tra	0.004	2.666	12	1	2	1	68	7.4	7.24	0	1	cellular ho	cytosol	metal ion I	Pf00403	475	ENSG0000	ATOX1	5	Mineral ab	Copper ho	Ion influx/	9	High	High	High	High	High	High	1	
FALSE	High	Master Prc	O75410-1	Transformi	0.005	2.561	1	1	1	1	805	87.7	4.88	3.02	1	cell divisio	cytoplasm;	catalytic a	Pf01496, P	6867	ENSG0000	TACC1	8				0	High	High	High	High	High	High	1	
FALSE	High	Master Prc	P98066	Tumor nec	0.006	2.416	5	1	1	1	277	31.2	6.79	0	1	cell commu	extracellul	protein bir	Pf00193, P	7130	ENSG0000	TNFAIP6	2			Neutrophil	3	High	High	High	High	High	High	1	
FALSE	High	Master Prc	Q8NFH5	Nucleopori	0	14.202	13	4	5	4	326	34.8	9.09	6.07	4	cell organi	membrane	DNA bindi	Pf05172, P	129401	ENSG0000	NUP35	2	RNA trans		Rev-media	64	High	High	High	High	High	High	1	
FALSE	Low	Master Prc	Q5SY16	Polynucleo	0.068	1.058	1	1	1	1	702	79.3	9.13	0	1	metabolic	cytoskelet	catalytic a	Pf03205, P	79707	ENSG0000	NOL9	1			Major path	4	High	High	High	High	High	High	1	
FALSE	High	Master Prc	P49281-1	natural res	0.003	2.969	2	1	1	1	568	62.2	6.09	0	1	cell organi	cell surface	metal ion I	Pf01566	4891	ENSG0000	SLC11A2	12	Lysosome;	Iron metak	Metal ion	13	High	High	High	High	High	High	1	
FALSE	Low	Master Prc	Q9UQ90	Paraplegin	0.067	1.073	2	1	1	1	795	88.2	8.69	0	1	cell organi	membrane	catalytic a	Pf00004, P	6687	ENSG0000	SPG7	16			Processing	3	High	High	High	High	High	High	1	
FALSE	High	Master Prc	P50613	Cyclin-dep	0	5.215	6	1	1	1	346	39	8.47	3.84	1	cell divisio	cytoplasm;	catalytic a	Pf00069, P	1022	ENSG0000	CDK7	5; CHR_HS	Cell cycle;	MicroRNA	RNA polym	70	High	High	High	High	High	High	1	
FALSE	High	Master Prc	Q8IWV7-1	E3 ubiquiti	0.006	2.537	1	1	1	1	1749	200.1	6.01	2.13	1	metabolic	cytoplasm;	catalytic a	Pf02207, P	197131	ENSG0000	UBR1	15			Antigen pr	4	High	High	High	High	High	High	1	
FALSE	High	Master Prc	Q9P000-1	COMM dor	0.009	2.173	5	1	1	1	198	21.8	5.88	0	1	metabolic	cytosol;ext	protein bir	Pf07258	29099	ENSG0000	COMMD9	11			Neutrophil	6	High	High	High	High	High	High	1	
FALSE	High	Master Prc	Q58FF6	Putative he	0	49.11	13	8	53	1	505	58.2	4.73	142.98	8	metabolic	cytoplasm	nucleotide	Pf00183, P	664618		HSP90AB4	15				0	High	High	High	High	High	High	1	[K414]
FALSE	High	Master Prc	Q96T88	E3 ubiquiti	0.002	4.136	1	1	1	1	793	89.8	7.56	0	1	cell organi	nucleus	catalytic a	Pf00097, P	29128	ENSG0000	UHRF1	19		Pathways	DNA meth	4	High	High	High	High	High	High	1	
FALSE	High	Master Prc	Q6VN20	Ran-bindin	0.001	4.423	2	1	1	1	620	67.2	6.77	1.71	1		cytoplasm;	protein bir	Pf00622, P	57610	ENSG0000	RANBP10	16			MET activa	4	High	High	High	High	High	High	1	
FALSE	Medium	Master Prc	Q8NB16	Mixed line	0.036	1.356	5	1	1	1	471	54.4	8.82	0	1	cell death;	cytoplasm;	catalytic a	Pf00069, P	197259	ENSG0000	MLKL	16	TNF signali	DNA Dama	TRP chann	9	High	High	High	High	High	High	1	
FALSE	High	Master Prc	Q9H633-1	Ribonuclea	0	4.898	10	1	1	1	154	17.6	9.45	0	1	metabolic	nucleus	catalytic a	Pf04032	79897	ENSG0000	RPP21	6; CHR_HS	RNA trans		Major path	7	High	High	High	High	High	High	1	
FALSE	High	Master Prc	Q9UBV8	Peflin [OS=	0.003	3.047	4	1	1	1	284	30.4	6.54	0	1	cell organi	cytoplasm;	catalytic a	Pf00036, P	553115	ENSG0000	PEF1	1				0	High	High	High	High	High	High	1	
FALSE	Medium	Master Prc	A6NHY2	ankyrin rep	0.019	1.621	1	1	1	1	528	59.1	8.35	0	1	regulation		protein bir	Pf00023, P	728780	ENSG0000	ANKDD1B	5				0	High	High	High	High	High	High	1	
FALSE	High	Master Prc	Q8TC12	Retinol del	0	24.615	17	4	6	4	318	35.4	8.82	15.43	4	metabolic	endoplasm	catalytic a	Pf00106, P	51109	ENSG0000	RDH11	14	Metabolic		Signaling b	13	High	High	High	High	High	High	1	
FALSE	High	Master Prc	Q9BWW4	Single-stra	0	6.803	3	2	2	2	388	40.4	6.9	1.91	2	cell differe	nucleus	DNA bindi	Pf04503	23648	ENSG0000	SSBP3	1				0	High	High	High	High	High	High	1	
FALSE	High	Master Prc	Q9NUQ8	ATP-bindir	0	7.839	6	3	3	3	709	79.7	6.34	0	3	defense re	membrane	catalytic a	Pf00005, P	55324	ENSG0000	ABCF3	3				0	High	High	High	High	High	High	1	[R162];
FALSE	High	Master Prc	Q8N3R9-1	MAGUK p5	0	5.979	2	1	1	1	675	77.2	6.14	3.08	1	cell organi	cytoplasm;	protein bir	Pf00018, P	64398	ENSG0000	MPP5	14	Tight junct		Tight junct	6	High	High	High	High	High	High	1	
FALSE	High	Master Prc	P54098	DNA polyn	0.003	2.893	1	1	1	1	1239	139.5	6.9	0	1	cellular ho	mitochond	catalytic a	Pf00476	5428	ENSG0000	POLG	15	Metabolic	Nucleotide		2	High	High	High	High	High	High	1	
FALSE	High	Master Prc	Q8WWH5	probable t	0.003	3.198	3	1	2	1	349	37.2	8.25	1.62	1	metabolic	mitochond	catalytic a	Pf01509	142940	ENSG0000	TRUB1	10				0	High	High	High	High	High	High	1	
FALSE	High	Master Prc	Q6P2H3-1	Centrosom	0.003	3.159	2	1	1	1	762	85.6	6	0	1	cell organi	cytoplasm;	catalytic a	Pf00901, P	64793	ENSG0000	CEP85	1				0	High	High	High	High	High	High	1	
FALSE	High	Master Prc	Q9BVL2	Nucleopori	0.008	2.201	1	1	1	1	599	60.9	9.33	1.68	1	cell organi	membrane	protein bir		9818	ENSG0000	NUPL1; NU	13	RNA trans		Rev-media	64	High	High	High	High	High	High	1	
FALSE	High	Master Prc	O14545	TRAF-type	0	6.503	4	2	3	2	582	64.8	5.29	4.47	2	regulation		metal ion I		10906	ENSG0000	TRAFD1	12		Regulation		1	High	High	High	High	High	High	1	
FALSE	High	Master Prc	Q9H9F9	actin-relat	0.002	3.651	2	1	1	1	607	68.3	5.27	0	1	metabolic	cytoplasm;	protein bir	Pf00022, P	79913	ENSG0000	ACTR5	20			UCH prote	8	High	High	High	High	High	High	1	
FALSE	High	Master Prc	O43414-1	ERI1 exorit	0.006	2.364	4	1	1	1	337	37.2	8.07	2.32	1	metabolic		catalytic a	Pf00929	79033	ENSG0000	ERI3	1				0	High	High	High	High	High	High	1	
FALSE	High	Master Prc	Q9NVA1	Ubiquinol-	0.009	2.109	6	1	1	1	299	34.6	8.91	2.98	1	cell organi	membrane	protein bir	Pf03981	55245	ENSG0000	UQCC; UQ	20				0	High	High	High	High	High	High	1	
FALSE	High	Master Prc	O60563	Cyclin-T1 [	0	6.328	4	2	2	2	726	80.6	8.78	3.03	2	cell divisio	nucleus	catalytic a	Pf00134	904	ENSG0000	CCNT1	12	Transcripti	Initiation c	RNA polym	32	High	High	High	High	High	High	1	
FALSE	High	Master Prc	Q03111	Protein EN	0.005	2.559	2	1	1	1	559	62	8.59	0	1	metabolic	cytosol;nu	DNA bindi	Pf03366	4298	ENSG0000	MLLT1	19	Transcripti		Formation	6	High	High	High	High	High	High	1	
FALSE	Medium	Master Prc	Q9H467	CUE domai	0.028	1.448	3	1	1	1	287	32	4.81	0	1	regulation	cytoplasm;	protein bir	Pf02845	79004	ENSG0000	CUEDC2	10		Regulation		1	High	High	High	High	High	High	1	
FALSE	Medium	Master Prc	P42263-1	Glutamate	0.036	1.349	1	1	1	1	894	101.1	8.54	0	1	regulation	membrane	receptor a	Pf00060, P	2892	ENSG0000	GRIA3	X	Nicotine a	Brain-Deri	Activation	23	High	High	High	High	High	High	1	

FALSE	Medium	Master Prc	Q9NYP7-1	elongation	0.024	1.503	3	1	1	1	299	35.3	9.42	1.87	1	metabolic	endoplasm	catalytic a	Pf01151	60481	ENSG0000	ELOVL5	6	Fatty acid		Synthesis c	11								High	High	High	High	High	High	1	
FALSE	High	Master Prc	Q92922	SWI/SNF c	0	19.999	5	6	9	3	1105	122.8	5.76	11.12	6	cell organi	chromosor	DNA bindi	Pf04433	6599	ENSG0000	SMARCC1	3			RUNX1 int	8								High	High	High	High	High	High	1	
FALSE	High	Master Prc	Q96CN9	GRIP and c	0.001	4.264	1	1	1	1	775	87.8	5.45	0	1	metabolic	cytoplasm;	protein bir	Pf01465, P	79571	ENSG0000	GCC1	7			Retrograde	4								High	High	High	High	High	High	1	
FALSE	High	Master Prc	Q9NVZ3	Adaptin ea	0.003	3.133	4	1	1	1	263	28.3	8.38	2.21	1	transport	membrane		Pf07933	55707	ENSG0000	NECAP2	1			Cargo reco	4								High	High	High	High	High	High	1	
FALSE	High	Master Prc	P52732	Kinesin-like	0	9.852	3	3	3	3	1056	119.1	5.64	6.08	3	cell divisio	cytoplasm;	catalytic a	Pf00225, P	3832	ENSG0000	KIF11	10			COPI-depe	11								High	High	High	High	High	High	1	
FALSE	Medium	Master Prc	Q9UKU7	Isobutyryl-	0.016	1.714	2	1	1	1	415	45	7.85	1.79	1	metabolic	mitochond	catalytic a	Pf00441, P	27034	ENSG0000	ACAD8	11	Metabolic		Branched-	5								High	High	High	High	High	High	1	
FALSE	High	Master Prc	O95989	Diphospho	0	5.148	5	1	1	1	172	19.5	6.34	0	1	cell commu	cytoplasm;	catalytic a	Pf00293	11165	ENSG0000	NUDT3	6			Synthesis c	3								High	High	High	High	High	High	1	
FALSE	High	Master Prc	Q13033	Striatin-3 [	0	6.181	2	1	1	1	797	87.2	5.36	2.55	1	regulation	cytoplasm;	protein bir	Pf00400, P	29966	ENSG0000	STRN3	14				0								High	High	High	High	High	High	1	
FALSE	High	Master Prc	Q9P2W9	Syntaxin-1	0.003	2.976	6	1	1	1	335	38.7	5.49	2.3	1	cell organi	endoplasm	protein bir	Pf10496	53407	ENSG0000	STX18	4	Phagosom		COPI-depe	7								High	High	High	High	High	High	1	
FALSE	Medium	Master Prc	A2RRP1-1	neuroblast	0.026	1.468	0	1	1	1	2371	268.4	5.96	0	1	metabolic	cytoplasm;	protein bir	Pf08314, P	51594	ENSG0000	NBAS	2			COPI-depe	5								High	High	High	High	High	High	1	
FALSE	High	Master Prc	Q8WXG6-1	MAP kinas	0.003	3.11	1	1	1	1	1647	183.2	6.04	2.03	1	cell death;	cytoplasm;	enzyme re	Pf02141, P	8567	ENSG0000	MADD	11		Deregulati		2								High	High	High	High	High	High	1	
FALSE	High	Master Prc	Q8NHG8	E3 ubiquiti	0.003	2.86	14	1	1	1	242	24.1	7.09	2.39	1	metabolic	cytoplasm;	catalytic a	Pf00097, P	223082	ENSG0000	ZNRF2	7			Antigen pr	4								High	High	High	High	High	High	1	
FALSE	High	Master Prc	Q5VZK9	F-actin-unc	0.006	2.519	1	2	2	2	1371	151.5	7.85	1.75	2	cell organi	cytoplasm;	protein bir	Pf08542	55604	ENSG0000	LRRC16A;	6			Factors inv	2								High	High	High	High	High	High	1	
FALSE	High	Master Prc	Q9NZJ6	Ubiquinone	0.004	2.7	5	1	1	1	369	41	7.42	0	1	metabolic	membrane	catalytic a	Pf01209, P	51805	ENSG0000	COQ3	6	Metabolic		Ubiquinol	6								High	High	High	High	High	High	1	
FALSE	Medium	Master Prc	P17540	Creatine ki	0.037	1.324	3	1	1	1	419	47.5	8.19	0	1	metabolic	membrane	catalytic a	Pf00217, P	1160	ENSG0000	CKMT2	5	Metabolic		Creatine m	6								High	High	High	High	High	High	1	[K200];
FALSE	High	Master Prc	O43324-1	Eukaryotic	0.001	4.452	6	1	1	1	174	19.8	8.54	2.36	1	metabolic	cytoplasm;	catalytic a	Pf00043, P	9521	ENSG0000	EEF1E1	6			Cytosolic t	8								High	High	High	High	High	High	1	
FALSE	High	Master Prc	Q9P0M9	39S riboso	0.004	2.754	10	1	2	1	148	16.1	10.42	2.49	1	cell organi	membrane	protein bir	Pf01016	51264	ENSG0000	MRPL27	17	Ribosome		Mitochonc	7								High	High	High	High	High	High	1	
FALSE	High	Master Prc	Q9NR19-1	Acetyl-coe	0.002	3.601	1	1	1	1	701	78.5	6.46	0	1	cell organi	cytoplasm;	catalytic a	Pf00501, P	55902	ENSG0000	ACSS2	20	Carbon me	Fatty Acid	Transcripti	15								High	High	High	High	High	High	1	
FALSE	Medium	Master Prc	Q8IY37	Probable A	0.01	2.062	1	1	1	1	1157	129.5	8.1	0	1	metabolic	cytoplasm	catalytic a	Pf00270, P	57647	ENSG0000	DHX37	12			Major path	5								High	High	High	High	High	High	1	
FALSE	Medium	Master Prc	Q6UWH4-	protein FA	0.026	1.468	1	1	1	1	519	57.5	9.7	0	1		Golgi;mem		Pf15051	51313	ENSG0000	FAM198B	4				0								High	High	High	High	High	High	1	
FALSE	Medium	Master Prc	Q96BP3	Peptidylpr	0.012	1.991	1	1	1	1	646	73.5	7.15	0	1	metabolic	nucleus;sp	catalytic a	Pf00160, P	23398	ENSG0000	PPWD1	5			mRNA Spli	4								High	High	High	High	High	High	1	
FALSE	Low	Master Prc	O15037	protein KH	0.051	1.189	1	1	1	1	678	74.5	7.02	0	1			RNA bindi	Pf11977	23351	ENSG0000	KHNYN	14				0								High	High	High	High	High	High	1	[K23];
FALSE	High	Master Prc	Q9Y450-1	HBS1-like	0.002	3.688	3	2	2	2	684	75.4	6.61	1.68	2	metabolic	cytosol;me	catalytic a	Pf00009, P	10767	ENSG0000	HBS1L	6	Legionellos		mRNA dec	5								High	High	High	High	High	High	1	
FALSE	High	Master Prc	Q8WUX1-1	Sodium-co	0	5.216	4	1	1	1	472	51.4	8.21	3.11	1	transport	membrane	transporte	Pf01490	92745	ENSG0000	SLC38A5	X	GABAergic		Amino acic	5								High	High	High	High	High	High	1	
FALSE	High	Master Prc	Q9UHY1	Nuclear re	0	6.705	4	2	3	2	535	59.8	5.08	6.99	2	metabolic	cytoplasm;	catalytic a	Pf00069, P	29959	ENSG0000	NRBP1	2			Nuclear Re	4								High	High	High	High	High	High	1	
FALSE	Low	Master Prc	Q9BWT7	caspase re	0.054	1.163	1	1	1	1	1032	115.9	5.95	0	1	cell organi	cytoplasm	protein bir	Pf00619, P	29775	ENSG0000	CARD10	22	NF-kappa I			1								High	High	High	High	High	High	1	
FALSE	Medium	Master Prc	P68402-1	platelet-ac	0.024	1.499	3	1	1	1	229	25.6	5.92	0	1	metabolic	cytoplasm;	catalytic a	Pf00657, P	5049	ENSG0000	PAFAH1B2	11	Metabolic		COPI-indep	10								High	High	High	High	High	High	1	
FALSE	Low	Master Prc	Q9Y238	Deleted in	0.071	1.038	1	1	1	1	1755	195.6	6.33	0	1	regulation	cytoplasm;		Pf09321	9940	ENSG0000	DLEC1	3				0								High	High	High	High	High	High	1	[S116(98.
FALSE	High	Master Prc	O43715	TP53-regul	0	5.532	37	3	3	3	76	8.8	5.48	4.26	3	cell death;	cytoplasm;	protein bir	Pf05254	51499	ENSG0000	TRIAP1	12			TP53 Regu	6								High	High	High	High	High	High	1	
FALSE	Medium	Master Prc	Q12774-1	Rho guanir	0.044	1.254	1	1	1	1	1597	176.7	5.53	0	1	cell organi	cytoplasm;	nucleotide	Pf00018, P	7984	ENSG0000	ARHGEF5	7			G alpha (1:	11								High	High	High	High	High	High	1	
FALSE	High	Master Prc	Q96E22	Dehydrodc	0.001	4.422	5	1	1	1	293	33.2	8.47	2.81	1	cell differe	endoplasm	catalytic a	Pf01255	116150	ENSG0000	NUS1	6	Terpenoid		Synthesis c	7								High	High	High	High	High	High	1	
FALSE	Medium	Master Prc	Q92934	Bcl2-associ	0.039	1.296	10	1	1	1	168	18.4	7.15	0	1	cell death;	cytoplasm;	enzyme re	Pf10514	572	ENSG0000	BAD	11	cGMP-PKG	Leptin sign	AKT phosp	67								High	High	High	High	High	High	1	
FALSE	High	Master Prc	Q9UPT5	Exocyst co	0.005	2.56	1	1	2	1	735	83.3	6.79	0	1	regulation	cytoplasm;	protein bir	Pf03081	23265	ENSG0000	EXOC7	17	Insulin sigr	Angiopoiet	Translocat	12								High	High	High	High	High	High	1	
FALSE	High	Master Prc	Q8WTV0-1	Scavenger	0.003	2.953	2	1	1	1	552	60.8	8.24	2.27	1	cell organi	cell surface	protein bir	Pf01130	949	ENSG0000	SCARB1	12	Vitamin di	Selenium M		13								High	High	High	High	High	High	1	
FALSE	High	Master Prc	Q86U70	LIM domai	0.007	2.273	3	1	1	1	411	46.5	6.96	0	1	cell differe	nucleus	DNA bindi	Pf01803	8861	ENSG0000	LDB1	10	Transcripti		Regulation	10								High	High	High	High	High	High	1	
FALSE	Medium	Master Prc	Q9Y657	Spindlin-1	0.048	1.222	2	1	1	1	262	29.6	6.96	2.08	1	cell organi	cytosol;me	protein bir	Pf02513	10927	ENSG0000	SPIN1	9				0								High	High	High	High	High	High	1	
FALSE	High	Master Prc	Q9NP58	ATP-bindir	0	7.21	3	2	2	2	842	93.8	8.48	3.76	2	cellular ho	cytosol;en	catalytic a	Pf00005, P	10058	ENSG0000	ABCB6	2	ABC transp		Defective A	8								High	High	High	High	High	High	1	
FALSE	Medium	Master Prc	P60520	Gamma-ar	0.018	1.658	7	1	1	1	117	13.7	8.1	0	1	cell commu	cytoplasm;	protein bir	Pf02991, P	11345	ENSG0000	GABARAPL	16	Autophagy	Senescenc	TBC/RABG	11								High	High	High	High	High	High	1	
FALSE	High	Master Prc	P98170	E3 ubiquiti	0	5.637	3	1	1	1	497	56.6	6.65	1.96	1	cell death;	cytoplasm;	catalytic a	Pf00653, P	331	ENSG0000	XIAP	X	Focal adhe	Nucleotide	Regulation	39								High	High	High	High	High	High	1	
FALSE	High	Master Prc	P36543	V-type pro	0	12.322	6	1	2	1	226	26.1	8	6.76	1	cellular ho	cytoplasm;	catalytic a	Pf01991	529	ENSG0000	ATP6V1E1	22	mTOR sign		Transferrir	20								High	High	High	High	High	High	1	
FALSE	High	Master Prc	P49757	Protein nu	0.006	2.433	1	1	2	1	651	70.8	8.51	0	1	cell differe	cytoplasm;	protein bir	Pf00640, P	8650	ENSG0000	NUMB	14	Notch sign	Notch Sign	Hedgehog	15								High	High	High	High	High	High	1	
FALSE	High	Master Prc	Q9BYT8	Neurolysin	0.001	4.309	2	1	1	1	704	80.6	6.64	3.6	1	metabolic	cytoplasm;	catalytic a	Pf01432	57486	ENSG0000	NLN	5	Renin-angi		Peptide lig	6								High	High	High	High	High	High	1	
FALSE	High	Master Prc	Q9BSV6	tRNA-splic	0.003	3.077	5	1	1	1	310	33.6	8.43	1.79	1	metabolic	nucleus	catalytic a	Pf01974	79042	ENSG0000	TSEN34	19; CHR_H			tRNA proc	3								High	High	High	High	High	High	1	
FALSE	High	Master Prc	O15541	RING finge	0.003	3.33	3	1	1	1	343	38.8	5.69	1.9	1	metabolic	spliceosom	metal ion I	Pf00642, P	7737	ENSG0000	RNF113A	X				0								High	High	High	High	High	High	1	
FALSE	High	Master Prc	Q9Y6D0	selenoprot	0.005	2.595	10	1	1	1	94	10.6	10.86	0	1	cell differe	endoplasm	protein bir	Pf10961	58515	ENSG0000	SELK; SELE	3		Selenium M		2								High	High	High	High	High	High	1	
FALSE	Low	Master Prc	Q6NUQ1	RAD50-int	0.07	1.045	2	1	1	1	792	90.6	5.45	2.33	1	regulation	cytoplasm;	protein bir	Pf04437	60561	ENSG0000	RINT1	7			COPI-depe	5								High	High	High	High	High	High	1	[S105(100
FALSE	High	Master Prc	P42566-1	epidermal	0	8.908	4	3	4	3	896	98.6	4.64	0	3	cell organi	cytoplasm;	catalytic a	Pf00769, P	2060	ENSG0000	EPS15	1	Endocytosi	VEGFA-VE(	Cargo reco	17								High	High	High	High	High	High	1	
FALSE	High	Master Prc	Q96HP4	Oxidoredu	0	4.991	5	1	1	1	312	34.8	8.37	2.45	1	metabolic	mitochond	catalytic a	Pf00175, P	92106	ENSG0000	OXNAD1	3				0								High	High	High	High	High	High	1	
FALSE	High	Master Prc	Q96GK7	fumarylace	0	7.613	11	3	3	3	314	34.6	8.24	2.35	3	metabolic		catalytic a	Pf01557	51011	ENSG0000	FAHD2A	2				0								High	High	High	High	High	High	1	
FALSE	Medium	Master Prc	Q8N2I9	Serine/thre	0.012	1.94	2	1	1	1	435	49	7.93	0	1	metabolic	cytoplasm;	catalytic a	Pf00069, P	83931	ENSG0000	STK40	1				0								High	High	High	High	High	High	1	
FALSE	High	Master Prc	Q8WXD5	gem-assoc	0	5.433	14	2	2	2	167	18.8	5.12	3.89	2	cell organi	cytoplasm;	protein bir	Pf06372	79833	ENSG0000	GEMIN6	2	RNA transp		snRNP Ass	4								High	High	High	High	High	High	1	
FALSE	High	Master Prc	P03886	NADH-ubic	0	6.407	6	1	1	1	318	35.6	6.55	3.97	1	cell organi	membrane	catalytic a	Pf00146	4535	ENSG0000	ND1	MT	Parkinson's	Electron Tr	Complex I	10								High	High	High	High	High	High	1	
FALSE	High	Master Prc	O00257-1	E3 SUMO-p	0.003	3.074	2	1	1	1	560	61.3	9.36	2.95	1	cell organi	nucleus	catalytic a	Pf00385	8535	ENSG0000	CBX4	17			RUNX1 int	21								High	High	High	High	High	High	1	
FALSE	High	Master Prc	P50993	Sodium/pc	0	108.047	19	14	42	1	1020	112.2	5.66	93.53	14	cell commu	cytoplasm;	catalytic a	Pf00122, P	477	ENSG0000	ATP1A2	1	Aldosteror	Synaptic V	Ion homeo	24								High	High	High	High	High	High	1	[K411]
FALSE	Low	Master Prc	Q9ULK6	RING finge	0.051	1.18	1	1	1	1	438	48	5.27	0	1		membrane	metal ion I	Pf00097, P	57484	ENSG0000	RNF150	4				0								High	High	High	High	High	High	1	
FALSE	High	Master Prc	Q9UGN5-1	Poly [ADP-	0.002	3.928	1	1	1	1	583	66.2	8.88	2.54	1	metabolic	cytoplasm;	catalytic a	Pf00644, P	10038	ENSG0000	PARP2	14	Base excisi	NAD+ bios	Formation	16								High	High	High	High	High	High	1	
FALSE	High	Master Prc	Q8WUD6-	Cholinepho	0.002	3.657	3	1	1	1	406	45.1	6.92	1.96	1	metabolic	Golgi;mem	catalytic a	Pf01066	56994	ENSG0000	CHPT1	12	Glyceroph	Kennedy p	Synthesis c	11								High	High	High	High	High	High	1	
FALSE	Medium	Master Prc	Q969G3	SWI/SNF-r	0.048	1.223	3	1	1	1	411	46.6	4.88	0	1	cell differe	chromosor	catalytic a	Pf00505	6605	ENSG0000	SMARCE1	17		Androgen	RUNX1 int	10								High	High	High	High	High	High	1	
FALSE	Medium	Master Prc	Q9P2Y4	Zinc finger	0.013	1.903	4	1	1	1	722	76.8	9.39	0	1	developme	nucleus	DNA bindi	Pf00096, P	51222	ENSG0000	ZNF219	14		Ectoderm		1								High	High	High	High	High	High	1	[Q144;
FALSE	Medium	Master Prc	Q9BZI7-1	Regulator	0.03	1.431	2	1	1	1	483	57.7	9.48	0	1	metabolic	cytoplasm;	protein bir	Pf03467	65109	ENSG0000	UPF3B	X	RNA transp		mRNA 3'-e	19								High	High	High	High	High	High	1	
FALSE	High	Master Prc	Q15011-1	homocyste	0.002	3.912	3	1	1	1	391	43.7	5.25	0	1	cellular ho	endoplasm	protein bir	Pf00240	9709	ENSG0000	HERPUD1	16	Protein prc	VEGFA-VE(	ATF4 activ	7								High	High	High	High	High	High	1	
FALSE	High	Master Prc	P30047	GTP cycloh	0	10.019	40	2	2	2	84	9.7	6.54	6.38	2	cell organi	cytoplasm;	enzyme re	Pf06399	2644	ENSG0000	GCHFR	15			eNOS activ	4								High	High	High	High	High	High	1	
FALSE	High	Master Prc	Q9UK59	lariat debr	0	12.729	7	2	2	2	544	61.5	5.47	6.43	2	metabolic	nucleus	catalytic a	Pf00149, P	51163	ENSG0000	DBR1	3				0								High	High	High	High	High	High	1	
FALSE	Medium	Master Prc	P43155	Carnitine C	0.011	2.026	1	1	2	1	626	70.8	8.44	6.25	1	metabolic	endoplasm	catalytic a	Pf00755	1384	ENSG0000	CRAT	9	Peroxisom	Fatty Acid	Beta-oxida	7								High	High	High	High	High	High	1	
FALSE	High	Master Prc	P07196	Neurofilam	0	11.829	5	3	4	1	543	61.5	4.65	10.16	3	cell organi	cytoplasm;	catalytic a	Pf00038, P	4747	ENSG0000	NEFL	8	Amyotropl	Amyotropl	Ras activat	20								High	High	High	High	High	High	1	[K339]
FALSE	High	Master Prc	Q13424	Alpha-1-sy	0.008	2.202	2	1	1	1	505	53.9	6.8	0	1	regulation	cytoplasm;	protein bir	Pf00169, P	6640	ENSG0000	SNTA1	20		Sudden Inf		1								High	High	High	High	High	High	1	
FALSE	High	Master Prc	Q9NYP9	protein Mi	0.001	4.313	5	1	1	1	233	25.8	5.2	3.07	1	cell divisio	chromosor	metal ion I	Pf03226	54069	ENSG0000	MIS18A	21			Deposition	4								High	High	High	High	High	High	1	
FALSE	High	Master Prc	Q30201-1	Hereditary	0	6.71	3	1	2	1	348	40.1	6.6	5.43	1	cell commu	cytoskelet	protein bir	Pf00129, P	3077	ENSG0000	HFE	6		Hfe effect	Transferrir	4								High	High	High	High	High	High	1	
FALSE	High	Master Prc	Q8N8N7-1	Prostaglan	0.006	2.364	2	1	1	1	351	38.5	5.41	0	1	metabolic	cytoplasm	catalytic a	Pf00107	145482	ENSG0000	PTGR2	14			Synthesis c	5								High	High	High	High	High	High	1	
FALSE	Medium	Master Prc	Q658Y4	Protein FA	0.015	1.766	1	1	1	1	838	93.9	6.39	0	1				Pf14647, P	157769	ENSG0000	FAM91A1	8		Gastric car		1								High	High	High	High	High	High	1	
FALSE	Medium	Master Prc	Q6UWP8-2	Isoform 2 c	0.01	2.063	7	1	1	1	247	25.3	7.08	1.74	1					374897	ENSG0000	SBSN	19				0								High	High	High	High	High	High	1	[Q125;
FALSE	Medium	Master Prc	Q8NBP7-1	Proprotein	0.013	1.876	2	1	1	1	692	74.2	6.61	0	1	cell commu	cell surface	catalytic a	Pf00082, P	255738	ENSG0000	PCSK9	1		Proprotein	Post-transl	11								High	High	High	High	High	High	1	
FALSE	Medium	Master Prc	Q96GR2	Long-chain	0.011	2.017	2	1	2	1	724	81.2	6.02	4.44	1	metabolic	cytoplasm;	catalytic a	Pf00501	23205	ENSG0000	ACSBG1	15	Metabolic	PPAR signa	Synthesis c	12								High	High	High	High	High	High	1	[R144]
FALSE	High	Master Prc	Q9P2G1	Ankyrin re	0.006	2.377	1	1	1	1	1089	121.9	5.11	0	1	metabolic	cytoplasm	catalytic a	Pf00023, P	54467	ENSG0000	ANKIB1	7				0								High	High	High	High	High	High	1	
FALSE	High	Master Prc	Q9BZL1	ubiquitin-li	0.008	2.195	12	1	1	1	73	8.5	8.44	0	1	metabolic	cytoplasm;	protein bir	Pf00240	59286	ENSG0000	UBL5	19				0								High	High	High	High	High	High	1	
FALSE	Medium	Master Prc	Q6ZU80-1	Isoform 1 c	0.02	1.611	1	1	1	1	987	115.7	6.23	0	1	cellular co		catalytic a	Pf01576, P	145508		CEP128	14				0								High	High	High	High	High	High	1	
FALSE	High	Master Prc	Q5ST30-1	Valine--tRI	0.004	2.691	1	1	1	1	1063	118.4	7.02	0	1	metabolic	cytosol;mi	catalytic a	Pf00133, P	57176	ENSG0000	VARS2	6; CHR_HS	Aminoacyl		Mitochonc	5								High	High	High	High	High	High	1	
FALSE	Medium	Master Prc	P16278	Beta-galac	0.036	1.329	1	1	1	1	677	76	6.57	0	1	metabolic	cytoplasm;	catalytic a	Pf01301, P	2720		GLB1	3	Sphingolip	Ectoderm	Sialic acid	32								High	High	High	High	High	High	1	
FALSE	High	Master Prc	Q9NRN9	Methyltrar	0.003	3.222	4	1	1	1	209	23.7	6.68	0	1	metabolic		catalytic a	Pf01170, P	29081	ENSG0000	METTL5	2				0								High	High	High	High	High	High	1	

FALSE	High	Master Prc	Q6ZSR9	Uncharact	0	7.326	4	1	1	1	355	38	5.26	3.78	1				Pf15282								0	High	High	High	High	High	High	1	
FALSE	High	Master Prc	Q13541	Eukaryotic	0.009	2.141	10	1	1	1	118	12.6	5.48	2.14	1	metabolic	cytoplasm;	protein bir	Pf05456	1978	ENSG0000	EIF4EBP1	8	Longevity r	Translatior	mTORC1-n	38	High	High	High	High	High	High	1	
FALSE	High	Master Prc	P49746	Thrombosp	0	6.111	3	2	2	2	956	104.1	4.65	3.21	2		extracellul	metal ion I	Pf02412, P	7059	ENSG0000	THBS3	1	ECM-recep	Focal Adhe	Signaling b	11	High	High	High	High	High	High	1	
FALSE	Low	Master Prc	Q01973	Inactive ty	0.063	1.097	1	1	1	1	937	104.2	7.17	0	1	metabolic	cell surface	catalytic a	Pf00047, P	4919	ENSG0000	ROR1	1		Nuclear Re	WNT5A-de	8	High	High	High	High	High	High	1	
FALSE	High	Master Prc	Q8IWD4-1	Coiled-coil	0.004	2.804	4	1	1	1	279	30.5	5.07	0	1			protein bir		150275	ENSG0000	CCDC117	22				0	High	High	High	High	High	High	1	
FALSE	High	Master Prc	Q00266	S-adenosyl	0	22.44	12	5	6	1	395	43.6	6.29	17.04	5	cell organi	cytosol	catalytic a	Pf00438, P	4143	ENSG0000	MAT1A	10	Metabolic	Vitamin B1	Metabolisr	21	High	High	High	High	High	High	1	[R264]
FALSE	High	Master Prc	Q13751	Laminin su	0	5.367	2	2	2	2	1172	129.5	7.21	3.21	2	cell differe	extracellul	protein bir	Pf00053, P	3914	ENSG0000	LAMB3	1	Toxoplasm	Alpha 6 Be	Laminin in	25	High	High	High	High	High	High	1	
FALSE	High	Master Prc	P51948-1	CDK-activa	0	20.671	18	4	5	4	309	35.8	6.09	12.31	4	cell organi	cytosol;nu	catalytic a	Pf00097, P	4331	ENSG0000	MNAT1	14	Basal trans	Eukaryotic	NoRC nega	65	High	High	High	High	High	High	1	
FALSE	Low	Master Prc	Q6P4F7	rho GTPase	0.071	1.036	1	1	1	1	1023	113.8	9.07	0	1	regulation	cytosol	enzyme re	Pf00620	9824	ENSG0000	ARHGAP11	15; CHR_H			Rho GTPas	3	High	High	High	High	High	High	1	[Q618]
FALSE	High	Master Prc	Q6Y1H2	Very-long-	0	7.61	9	2	2	2	254	28.4	9.55	5.17	2	metabolic	endoplasm	catalytic a	Pf04387	201562	ENSG0000	PTPLB; HA	3	Fatty acid		Synthesis c	8	High	High	High	High	High	High	1	
FALSE	High	Master Prc	Q8TCY9	Up-regulat	0.002	3.49	1	1	1	1	931	104.9	6.42	3.37	1		cytoplasm;	nucleotide	Pf04312	55665	ENSG0000	URGCP	7				0	High	High	High	High	High	High	1	
FALSE	Low	Master Prc	Q9Y5J1	U3 small n	0.071	1.029	3	1	1	1	556	62	8.76	0	1	metabolic	membrane	protein bir		51096	ENSG0000	UTP18	17	Ribosome		Major path	6	High	High	High	High	High	High	1	
FALSE	High	Master Prc	O00233-1	26S protea	0	8.312	13	3	5	3	223	24.7	6.95	0	3	cell organi	cytoplasm;	protein bir	Pf04495, P	5715	ENSG0000	PSMD9	12		Proteasom	Regulation	150	High	High	High	High	High	High	1	
FALSE	High	Master Prc	Q99666	RANBP2-lil	0	50.961	9	16	19	2	1765	198.8	6.42	25.65	16	transport	cytoplasm	protein bir	Pf00515, P	729540; 8	ENSG0000	RGPD6; RC	2	RNA transp			1	High	High	High	High	High	High	1	[K409]
FALSE	High	Master Prc	Q9NTX5	Ethylmalor	0.001	4.211	4	1	1	1	307	33.7	8.21	2.24	1	metabolic	cytoplasm;	catalytic a	Pf00378	55862	ENSG0000	ECHDC1	6	Propanoat	Fatty Acid		2	High	High	High	High	High	High	1	
FALSE	High	Master Prc	P21127-1	Cyclin-dep	0	4.639	2	2	2	2	795	92.7	5.57	3.86	2	cell death;	cytoplasm;	catalytic a	Pf00069, P	984		CDK11B	1			Recruitme	6	High	High	High	High	High	High	1	
FALSE	Medium	Master Prc	Q9ULU4	protein kin	0.044	1.252	1	1	1	1	1186	131.6	7.2	0	1	metabolic	cytoplasm;	DNA bindi	Pf00439, P	23613	ENSG0000	ZMYND8	20				0	High	High	High	High	High	High	1	
FALSE	High	Master Prc	P49585	choline-ph	0	8.802	5	1	1	1	367	41.7	7.25	5.08	1	metabolic	cytoplasm;	catalytic a	Pf01467	5130	ENSG0000	PCYT1A	3	Metabolic	Kennedy p	Synthesis c	11	High	High	High	High	High	High	1	
FALSE	High	Master Prc	Q96A65	Exocyst co	0.002	3.912	1	1	1	1	974	110.4	6.49	0	1	cell comm	cytoplasm;	protein bir	Pf04048	60412	ENSG0000	EXOC4	7	Tight junct		Translocat	11	High	High	High	High	High	High	1	
FALSE	High	Master Prc	P46108-1	Adapter m	0.003	2.955	5	1	1	1	304	33.8	5.55	1.97	1	cell organi	cytoplasm;	protein bir	Pf00017, P	1398	ENSG0000	CRK	17	Neurotrop	Integrin-m	VEGFA-VE	63	High	High	High	High	High	High	1	
FALSE	High	Master Prc	Q9BRP1	Programm	0	12.109	7	2	2	2	358	39.4	4.86	4.73	2		cytoplasm;		Pf04194	84306	ENSG0000	PDCD2L	19				0	High	High	High	High	High	High	1	
FALSE	Low	Master Prc	Q9GZU1	Mucolipin-	0.053	1.174	1	1	1	1	580	65	7.24	0	1	cell organi	cytosol;en	protein bir	Pf00520, P	57192	ENSG0000	MCOLN1	19	Lysosome	Iron metab	TRP chann	8	High	High	High	High	High	High	1	
FALSE	Medium	Master Prc	P50336	protoporpl	0.034	1.374	1	1	1	1	477	50.7	8.16	2.61	1	metabolic	membrane	catalytic a	Pf01266, P	5498	ENSG0000	PPOX	1	Metabolic	Heme Bios	Heme bios	6	High	High	High	High	High	High	1	
FALSE	High	Master Prc	Q9NY61	Protein AA	0	6.339	6	2	2	2	560	63.1	4.94	5.67	2	cell divisio	cytoplasm;	protein bir	Pf08164, P	26574	ENSG0000	AATF	17; CHR_H			NRAGE sig	6	High	High	High	High	High	High	1	
FALSE	High	Master Prc	Q6NXE6-1	Armadillo	0	14.599	6	3	6	3	501	54.1	6.24	6.81	3	cell differe	cytosol	protein bir	Pf00514	93436	ENSG0000	ARMC6	19				0	High	High	High	High	High	High	1	
FALSE	High	Master Prc	Q5SW96	Low densit	0.002	4.087	6	1	1	1	308	33.9	6.7	2.71	1	cell organi	cytoplasm;	protein bir	Pf00640, P	26119	ENSG0000	LDLRAP1	1	Endocytosi		Cargo reco	10	High	High	High	High	High	High	1	
FALSE	High	Master Prc	Q9H7N4	Splicing fa	0	5.575	1	1	1	1	1312	139.2	9.25	0	1	metabolic	nucleus	protein bir		58506	ENSG0000	SCAF1	19				0	High	High	High	High	High	High	1	
FALSE	High	Master Prc	Q9BW60	Elongation	0	4.554	9	2	2	2	279	32.6	9.6	1.81	2	metabolic	endoplasm	catalytic a	Pf01151	64834	ENSG0000	ELOVL1	1	Fatty acid		Synthesis c	9	High	High	High	High	High	High	1	
FALSE	Medium	Master Prc	Q8TAD8	Smad nucl	0.026	1.476	2	1	1	1	396	45.8	9.99	0	1	metabolic	cytosol;nu	protein bir	Pf00498	79753	ENSG0000	SNIP1	1		TGF-beta S		1	High	High	High	High	High	High	1	
FALSE	High	Master Prc	Q12834	cell divisio	0.003	3.006	2	1	1	1	499	54.7	9.23	2.24	1	cell differe	cytoplasm;	enzyme re	Pf00400	991	ENSG0000	CDC20	1	Ubiquitin r	Cell Cycle	Inactivatio	46	High	High	High	High	High	High	1	
FALSE	High	Master Prc	Q9H0J9	Poly [ADP-	0.008	2.22	1	1	1	1	701	79	8.51	0	1		nucleus	catalytic a	Pf00644, P	64761	ENSG0000	PARP12	7				0	High	High	High	High	High	High	1	
FALSE	High	Master Prc	Q9NS91	E3 ubiquiti	0.002	3.292	2	1	1	1	495	56.2	7.58	1.82	1	metabolic	cytoplasm;	catalytic a	Pf00097, P	56852	ENSG0000	RAD18	3			E3 ubiquiti	7	High	High	High	High	High	High	1	
FALSE	High	Master Prc	P61964	WD repeat	0.002	3.609	4	1	1	1	334	36.6	8.27	2.06	1	cell organi	nucleus	catalytic a	Pf00400, P	11091	ENSG0000	WDR5	9			RUNX1 reg	16	High	High	High	High	High	High	1	
FALSE	High	Master Prc	P35813	Protein ph	0	10.219	9	2	2	2	382	42.4	5.36	5.21	2	metabolic	cytoplasm;	catalytic a	Pf00481, P	5494	ENSG0000	PPM1A	14	MAPK sign	TGF-beta S	Energy dep	13	High	High	High	High	High	High	1	
FALSE	High	Master Prc	Q9Y673	dolichyl-ph	0	6.645	6	1	1	1	324	36.9	9.28	1.64	1	metabolic	endoplasm	catalytic a	Pf00535, P	29880	ENSG0000	ALG5	13	Metabolic		Synthesis c	8	High	High	High	High	High	High	1	
FALSE	High	Master Prc	Q5ZPR3	CD276 ant	0	7.096	5	1	1	1	534	57.2	4.91	2.61	1	cell prolife	membrane	protein bir	Pf00047, P	80381	ENSG0000	CD276	15	Cell adhesi			1	High	High	High	High	High	High	1	
FALSE	High	Master Prc	Q14739	Lamin-B re	0.003	3.239	5	2	2	2	615	70.7	9.36	0	2	metabolic	membrane	catalytic a	Pf01222, P	3930	ENSG0000	LBR	1		Gastric car	Cholestero	6	High	High	High	High	High	High	1	
FALSE	High	Master Prc	Q9Y5J6	Mitochonc	0	7.606	16	1	1	1	103	11.6	7.43	4.65	1	transport	membrane	metal ion I	Pf02953	26515	ENSG0000	FXC1; TIMI	11			Mitochonc	2	High	High	High	High	High	High	1	
FALSE	High	Master Prc	Q92692-1	Nectin-2 [(	0.003	3.066	2	1	1	1	538	57.7	4.82	1.98	1	cell organi	cell surface	protein bir	Pf08205, P	5819	ENSG0000	PVRL2; NE	19	Herpes sim	Monoamir	Immunore	12	High	High	High	High	High	High	1	
FALSE	Medium	Master Prc	Q96RW7-1	Hemicenti	0.045	1.239	0	1	1	1	5635	613	6.49	2.03	1	cell divisio	cell surface	metal ion I	Pf00047, P	83872	ENSG0000	HMCN1	1				0	High	High	High	High	High	High	1	[K1815;
FALSE	High	Master Prc	Q6PKC3-1	Thioredoxi	0.003	3.359	2	1	1	1	985	110.5	6.55	2.91	1	cellular ho	endoplasm	catalytic a	Pf00038, P	51061	ENSG0000	TXNDC11	16				0	High	High	High	High	High	High	1	
FALSE	High	Master Prc	Q8WVK7-1	Spindle an	0.002	3.813	9	1	1	1	121	14.2	7.33	0	1	cell divisio	chromosor	protein bir		348235	ENSG0000	SKA2	17			Amplificati	16	High	High	High	High	High	High	1	
FALSE	Medium	Master Prc	Q9P0B6	Coiled-coil	0.013	1.916	8	1	2	1	97	11.5	9.5	0	1		membrane		Pf15188	154467	ENSG0000	CCDC167	6				0	High	High	High	High	High	High	1	
FALSE	High	Master Prc	Q99758	ATP-bindir	0.003	2.828	1	1	1	1	1704	191.2	7.62	1.95	1	metabolic	membrane	catalytic a	Pf00005, P	21	ENSG0000	ABCA3	16	ABC transp		Defective A	13	High	High	High	High	High	High	1	
FALSE	High	Master Prc	P17707-1	S-adenosyl	0.003	2.887	4	1	1	1	334	38.3	6	1.81	1	metabolic	cytosol	catalytic a	Pf01536	262	ENSG0000	AMD1	6	Cysteine a		Metabolisr	6	High	High	High	High	High	High	1	
FALSE	High	Master Prc	P08069	Insulin-like	0	5.125	1	2	3	2	1367	154.7	5.8	6.69	2	cell organi	membrane	catalytic a	Pf00041, P	3480	ENSG0000	IGF1R	15	HIF-1 signa	MicroRNA:	IRS-related	42	High	High	High	High	High	High	1	
FALSE	High	Master Prc	P46734-1	dual specif	0	13.947	9	3	7	1	347	39.3	7.43	17.86	3	defense re	cytoplasm;	catalytic a	Pf00069, P	5606	ENSG0000	MAP2K3	17	TNF signali	Regulation	Activated T	62	High	High	High	High	High	High	1	
FALSE	High	Master Prc	Q06587	E3 ubiquiti	0	14.498	10	2	2	2	406	42.4	5.62	4.92	2	cell organi	cytosol;nu	catalytic a	Pf00097, P	6015	ENSG0000	RING1	6; CHR_HS		Notch Sign	RUNX1 int	23	High	High	High	High	High	High	1	
FALSE	High	Master Prc	P09001	39S riboso	0	5.315	5	1	1	1	348	38.6	9.48	2.94	1	cell organi	membrane	catalytic a	Pf00297	11222	ENSG0000	MRPL3	3	Ribosome		Mitochonc	7	High	High	High	High	High	High	1	
FALSE	High	Master Prc	P12259	Coagulatio	0.006	2.343	0	1	1	1	2224	251.5	6.05	0	1	cell organi	endoplasm	metal ion I	Pf00754, P	2153	ENSG0000	F5	1	Compleme	Blood Clot	Platelet de	20	High	High	High	High	High	High	1	
FALSE	High	Master Prc	P57772	selenocyst	0	7.482	5	2	2	2	596	65.3	8.35	5.77	2	metabolic	cytoplasm;	catalytic a	Pf00009, P	60678	ENSG0000	EEFSEC	3		Selenium M	Selenocyst	5	High	High	High	High	High	High	1	[K38];
FALSE	Low	Master Prc	Q5SW79-1	Centrosom	0.065	1.08	1	1	1	1	1584	175.2	7.11	0	1		cytoplasm;	protein bir	Pf00498, P	9859	ENSG0000	CEP170	1; CHR_HS				0	Not Found	Not Found	Not Found	Not Found	Not Found	Not Found	1	
FALSE	High	Master Prc	Q15061	WD repeat	0	6.429	4	2	2	2	677	74.8	5.57	4.98	2	metabolic	nucleus	protein bir	Pf00400, P	23160	ENSG0000	WDR43	2	Ribosome		Major path	6	High	High	High	High	High	High	1	
FALSE	High	Master Prc	Q9HD26-1	Golgi-asso	0	5.134	5	2	2	2	462	50.5	5.92	2.87	2	cell organi	cytoplasm;	DNA bindi	Pf00595, P	57120	ENSG0000	GOPC	6			RHO GTPas	4	High	High	High	High	High	High	1	
FALSE	High	Master Prc	Q9NZR1	Tropomod	0.001	4.534	5	2	2	1	351	39.6	5.27	4.5	2	cell comm	cytoplasm;	protein bir	Pf03250	29767	ENSG0000	TMOD2	15			Striated M	2	High	High	High	High	High	High	1	
FALSE	High	Master Prc	Q9NZT2-1	opioid gro	0	12.819	5	3	3	3	677	73.3	4.84	5.44	3	regulation	cytoplasm;	protein bir	Pf04652, P	11054	ENSG0000	OGFR	20				0	High	High	High	High	High	High	1	
FALSE	High	Master Prc	Q96G21	U3 small n	0	4.713	5	2	3	2	291	33.7	9.47	2.03	2	metabolic	nucleus	protein bir	Pf04427	92856	ENSG0000	IMP4	2	Ribosome		Major path	6	High	High	High	High	High	High	1	
FALSE	High	Master Prc	Q8IXI1	Mitochonc	0.002	4.138	2	1	1	1	618	68.1	5.86	2.27	1	cell organi	cytosol;me	catalytic a	Pf00071, P	89941	ENSG0000	RHOT2	16			Rho GTPas	3	High	High	High	High	High	High	1	
FALSE	High	Master Prc	Q9HBM0-1	vezatin [O	0.003	3.389	3	2	2	2	779	88.6	5.2	0	2		cytosol;me	protein bir	Pf04290, P	55591	ENSG0000	VEZT	12				0	High	High	High	High	High	High	1	
FALSE	High	Master Prc	O75190	DnaJ homc	0.006	2.344	2	1	1	1	326	36.1	9.16	0	1	cell differe	cytoplasm;	DNA bindi	Pf00226	10049	ENSG0000	DNAJB6	7			Regulation	4	High	High	High	High	High	High	1	
FALSE	Medium	Master Prc	P19474	E3 ubiquiti	0.012	1.992	2	1	1	1	475	54.1	6.38	0	1	cell organi	cytoplasm;	catalytic a	Pf00097, P	6737	ENSG0000	TRIM21	11	Systemic lu		STING mec	12	High	High	High	High	High	High	1	
FALSE	Medium	Master Prc	O60344-1	Endothelin	0.016	1.711	1	1	1	1	883	99.7	5.1	0	1	cell comm	Golgi;mem	catalytic a	Pf01431, P	11059958	ENSG0000	EEF1AKMT	3			Peptide lig	5	High	High	High	High	High	High	1	
FALSE	High	Master Prc	P07948-1	Tyrosine-p	0	5.801	4	2	2	1	512	58.5	7.11	2.01	2	cell comm	cytoplasm;	catalytic a	Pf00017, P	4067	ENSG0000	LYN	8	NF-kappa I	IL-5 Signali	FCERI med	62	High	High	High	High	High	High	1	
FALSE	Medium	Master Prc	P78352	Disks large	0.016	1.705	1	1	1	1	724	80.4	5.88	0	1	cell comm	cytoplasm;	catalytic a	Pf00018, P	1742	ENSG0000	DLG4	17	Cocaine ac	BDNF-TrkE	NrCAM int	38	High	High	High	High	High	High	1	
FALSE	Medium	Master Prc	Q9NZU5	LIM and cy	0.015	1.778	4	1	1	1	365	40.8	7.93	0	1	metabolic	cytoplasm;	metal ion I	Pf00412, P	29995	ENSG0000	LMCD1	3			Surfactant	2	High	High	High	High	High	High	1	
FALSE	Medium	Master Prc	Q9H2Y7	Zinc finger	0.013	1.931	0	1	1	1	1883	208.8	7.15	0	1	regulation	cytosol;me	metal ion I	Pf00400	64397	ENSG0000	ZFP106; ZN	15				0	High	High	High	High	High	High	1	
FALSE	High	Master Prc	Q15059	bromodom	0.009	2.102	1	1	1	1	726	79.5	9.36	1.69	1	cell organi	nucleus	protein bir	Pf00439	8019	ENSG0000	BRD3	9				0	High	High	High	High	High	High	1	
FALSE	Medium	Master Prc	Q9NZN1	Interleukin	0.037	1.318	1	1	1	1	696	79.9	6.23	0	1	cell differe	cell surface	protein bir	Pf00047, P	11141	ENSG0000	IL1RAPL1	X			Receptor-t	8	High	High	High	High	High	High	1	
FALSE	High	Master Prc	Q6PJG6-1	BRCA1-ass	0	8.854	7	4	4	4	821	88.1	5.27	3.77	4	cell death;	cytoplasm;	protein bir		221927	ENSG0000	BRAT1	7				0	High	High	High	High	High	High	1	
FALSE	High	Master Prc	Q9NX08	COMM doi	0	6.226	7	1	2	1	183	21.1	5.43	3.02	1	metabolic	cytoplasm;	protein bir	Pf07258	54951	ENSG0000	COMMD8	4			Neddylatic	3	High	High	High	High	High	High	1	
FALSE	High	Master Prc	P15509	Granulocyt	0	4.665	3	1	1	1	400	46.2	7.75	0	1	cell differe	extracellul	catalytic a	Pf09240	1438	ENSG0000	CSF2RA	X|Y; X	Hematopo		Surfactant	29	High	High	High	High	High	High	1	
FALSE	Medium	Master Prc	Q9H6K5	proline-ricl	0.034	1.364	1	1	1	1	1346	132.7	10.48	0	1				Pf15363	80164	ENSG0000	FLJ22184;	19				0	High	High	High	High	High	High	1	
FALSE	Low	Master Prc	Q14191	Werner sy	0.057	1.133	2	1	1	1	1432	162.4	6.34	0	1	cell comm	cytoplasm;	catalytic a	Pf00270, P	7486	ENSG0000	WRN	8			G2/M DNA	27	High	High	High	High	High	High	1	
FALSE	High	Master Prc	Q9H8M5-1	Metal tran	0.002	3.556	1	1	1	1	875	96.6	6.38	0	1	transport	membrane	nucleotide	Pf00571, P	54805	ENSG0000	CNNM2	10				0	High	High	High	High	High	High	1	
FALSE	High	Master Prc	Q9Y2S6	Translatior	0.003	3.445	25	2	2	2	64	7.1	9.99	2.3	2					51372	ENSG0000	TMA7	3				0	High	High	High	High	High	High	1	
FALSE	High	Master Prc	O14657	Torsin-1B [	0	5.731	3	1	1	1	336	38	8.54	2.23	1	cell organi	cytoplasm;	catalytic a	Pf06309, P	27348	ENSG0000	TOR1B	9			Cargo reco	4	High	High	High	High	High	High	1	
FALSE	High	Master Prc	Q6VY07	Phosphofu	0	5.314	2	1	1	1	963	104.8	7.74	0	1	cell organi	cytosol;Go	protein bir	Pf10254	55690	ENSG0000	PACS1	11			Nef media	7	High	High	High	High	High	High	1	
FALSE	High	Master Prc	Q9Y6M4-1	Casein kina	0.008	2.208	2	1	1	1	447	51.4	9.22	2.05	1	metabolic	cytoplasm;	catalytic a	Pf00069, P	1456	ENSG0000	CSNK1G3	5	Hedgehog			1	High	High	High	High	High	High	1	
FALSE	High	Master Prc	Q9UP83-1	conserved	0.002	3.976	2	2	2	2	839	92.7	6.6	1.63	2	transport	cytoplasm;	protein bir	Pf05992, P	10466	ENSG0000	COG5	7			COPI-medi	11	High	High	High	High	High	High	1	

FALSE	Medium	Master Prc	Q9BVK8-1	transmeml	0.015	1.754	3	1	1	1	224	25.2	7.71	0	1	regulation	endoplasm	protein bir	Pf09767	10430	ENSG0000	TMEM147	19				0								High	High	High	High	High	High	1	
FALSE	High	Master Prc	Q14146	Unhealthy	0.002	3.512	1	1	1	1	1524	170.4	7.31	2.42	1		nucleus		Pf10441	9816	ENSG0000	URB2	1				0								High	High	High	High	High	High	1	
FALSE	Low	Master Prc	P22676	Calretinin	0.056	1.147	3	1	1	1	271	31.5	5.15	0	1	cellular ho	cytoplasm;	metal ion l	Pf00036, P	794	ENSG0000	CALB2	16; CHR_H				0								High	High	High	High	High	High	1	
FALSE	Medium	Master Prc	P55268	laminin sul	0.032	1.413	0	1	1	1	1798	195.9	6.52	0	1	cell growth	cytoplasm;	catalytic a	Pf00053, P	3913	ENSG0000	LAMB2	3	PI3K-Akt si	Alpha 6 Be	ECM prote	26								High	High	High	High	High	High	1	[Q1483;
FALSE	High	Master Prc	Q9Y5X2	Sorting ne	0	6.158	5	2	2	2	465	52.5	7.39	0	2	cell organi	cytosol;en	protein bir	Pf00787	29886	ENSG0000	SNX8	7				0								High	High	High	High	High	High	1	
FALSE	High	Master Prc	Q9Y3A6-1	Transmem	0.006	2.533	4	1	1	1	229	26	4.84	0	1	cell organi	endoplasm	protein bir	Pf01105	50999	ENSG0000	TMED5	1			WNT ligan	3								High	High	High	High	High	High	1	
FALSE	High	Master Prc	P43403-1	Tyrosine-p	0.009	2.112	2	1	1	1	619	69.8	7.69	2.16	1	cell differe	cytoplasm;	catalytic a	Pf00017, P	7535	ENSG0000	ZAP70	2	T cell recep	Prolactin S	Translocat	17								High	High	High	High	High	High	1	[K544;
FALSE	High	Master Prc	Q8N9T8	Protein KR	0.008	2.193	1	1	1	1	703	82.5	5.14	1.68	1	metabolic		RNA bindi	Pf05178, P	65095		KRI1	19				0								High	High	High	High	High	High	1	
FALSE	High	Master Prc	Q9NX00	Transmem	0.009	2.168	5	1	1	1	188	19.6	8.03	0	1		membrane			54958	ENSG0000	TMEM160	19				0								High	High	High	High	High	High	1	
FALSE	High	Master Prc	Q6AI12	Ankyrin rep	0.003	2.926	5	1	1	1	368	41.1	4.97	0	1			protein bir	Pf00023, P	91369	ENSG0000	ANKRD40	17				0								High	High	High	High	High	High	1	
FALSE	Medium	Master Prc	Q96C45	Serine/thre	0.014	1.818	1	1	1	1	1275	142.4	6.29	0	1	cell organi		catalytic a	Pf00069, P	54986	ENSG0000	ULK4	3				0								High	High	High	High	High	High	1	
FALSE	High	Master Prc	P07947	tyrosine-pr	0	8.088	5	3	4	1	543	60.8	6.74	6.56	3	cell differe	cytoplasm;	catalytic a	Pf00017, P	7525	ENSG0000	YES1	18	Tight junct	Interleukin	PECAM1 ir	35								High	High	High	High	High	High	1	
FALSE	Low	Master Prc	Q8NFX7-1	Syntaxin-b	0.071	1.036	4	1	1	1	210	23.5	9.04	0	1	regulation	cytoplasm;	protein bir	Pf00957, P	29091	ENSG0000	STXBP6	14				0								High	High	High	High	High	High	1	
FALSE	Medium	Master Prc	Q15113	Procollage	0.049	1.208	2	1	1	1	449	47.9	7.43	0	1	developme	extracellul	enzyme re	Pf00431, P	5118	ENSG0000	PCOLCE	7			Collagen b	5								High	High	High	High	High	High	1	
FALSE	High	Master Prc	Q9Y6N1	Cytochrom	0.003	3.045	4	1	2	1	276	31.4	9.06	5.08	1	cell organi	membrane	catalytic a	Pf04442	1353	ENSG0000	COX11	17	Oxidative p	Electron Tr	TP53 Regu	13								High	High	High	High	High	High	1	
FALSE	High	Master Prc	Q03468	DNA excisi	0.003	3.157	1	1	1	1	1493	168.3	8.09	2.84	1	metabolic	cytosol;Go	catalytic a	Pf00176, P	2074	ENSG0000	ERCC6	10	Nucleotide		Dual incisi	15								High	High	High	High	High	High	1	
FALSE	Medium	Master Prc	P78563-1	Double-str	0.014	1.856	1	1	1	1	741	80.7	9.01	0	1	cell commu	cytosol;nu	catalytic a	Pf00035, P	104	ENSG0000	ADARB1	21			C6 deamin	5								High	High	High	High	High	High	1	
FALSE	High	Master Prc	Q8NB90-1	Spermatog	0	11.014	4	2	3	1	893	97.8	5.66	4.69	2	cell differe	cytoplasm;	catalytic a	Pf00004, P	166378	ENSG0000	SPATA5	4	Ribosome			1								High	High	High	High	High	High	1	
FALSE	High	Master Prc	P45985	Dual specif	0.003	3.031	3	1	1	1	399	44.3	8.07	0	1	cell death;	cytoplasm;	catalytic a	Pf00069, P	6416	ENSG0000	MAP2K4	17	ErbB signa	Regulation	Uptake an	70								High	High	High	High	High	High	1	
FALSE	High	Master Prc	P23258	tubulin gar	0	26.173	12	2	2	1	451	51.1	6.14	13.08	2	cell organi	chromosor	catalytic a	Pf00091, P	7283	ENSG0000	TUBG1	17			Regulation	16								High	High	High	High	High	High	1	
FALSE	High	Master Prc	Q9H3H5-1	UDP-N-ace	0.009	2.098	2	1	3	1	408	46.1	8	4.05	1	cell organi	endoplasm	catalytic a	Pf00953	1798	ENSG0000	DPAGT1	11	N-Glycan b		Defective I	10								High	High	High	High	High	High	1	
FALSE	Medium	Master Prc	P16383-1	GC-rich sec	0.018	1.651	3	1	1	1	781	89.3	5.99	0	1	cell organi	cytosol;nu	DNA bindi	Pf07842	6936	ENSG0000	GCFC2	2			mRNA Spli	4								High	High	High	High	High	High	1	
FALSE	High	Master Prc	Q9NZL4-1	Hsp70-bind	0	9.864	12	3	3	3	362	39.4	5.21	6.2	3	metabolic		enzyme re		23640		HSPBP1	19	Protein pro			1								High	High	High	High	High	High	1	
FALSE	High	Master Prc	Q9NUG6	p53 and DI	0	6.848	13	1	2	1	133	15.5	6.06	7.6	1	metabolic	cytoplasm	protein bir	Pf01920	81572	ENSG0000	PDRG1	20				0								High	High	High	High	High	High	1	
FALSE	High	Master Prc	Q86WR7-1	Proline and	0	5.384	3	1	1	1	435	45.8	7.2	0	1			protein bir	Pf15385	254427	ENSG0000	C10orf47;	10				0								High	High	High	High	High	High	1	
FALSE	High	Master Prc	Q9NRK6	ATP-bindir	0	6.208	4	2	2	1	738	79.1	9.85	2.42	2	transport	membrane	catalytic a	Pf00005, P	23456	ENSG0000	ABCB10	1	ABC transp		Mitochonc	4								High	High	High	High	High	High	1	
FALSE	High	Master Prc	P35080-1	Profilin-2 [	0	14.593	27	4	7	3	140	15	6.99	17.51	4	cell organi	cytoplasm;	catalytic a	Pf00235	5217	ENSG0000	PFN2	3	Shigellosis	Associatio	RHO GTPa	12								High	High	High	High	High	High	1	
FALSE	High	Master Prc	Q9ULX3	RNA-bindi	0.004	2.758	4	1	1	1	412	46.6	7.18	0	1	metabolic	cytosol;nu	catalytic a	Pf08772, P	28987	ENSG0000	NOB1	16	Ribosome		Major path	5								High	High	High	High	High	High	1	
FALSE	High	Master Prc	Q9H425	Uncharact	0.003	2.858	3	1	1	1	327	36.3	5.72	0	1		cytoplasm;			84886	ENSG0000	C1orf198	1				0								High	High	High	High	High	High	1	
FALSE	Medium	Master Prc	P57081-1	tRNA (guai	0.011	2.038	2	1	1	1	412	45.5	7.11	0	1	metabolic	cytosol;nu	catalytic a		10785	ENSG0000	WDR4	21			tRNA mod	3								High	High	High	High	High	High	1	
FALSE	High	Master Prc	P26583	High mobil	0	15.574	17	4	7	2	209	24	7.81	10.88	4	cell organi	chromosor	DNA bindi	Pf00505, P	3148	ENSG0000	HMGB2	4		Retinoblas	Activation	7								High	High	High	High	High	High	1	
FALSE	High	Master Prc	Q16864-1	V-type pro	0	5.937	20	1	1	1	119	13.4	5.52	4.67	1	cellular ho	cytosol;me	catalytic a	Pf01990	9296	ENSG0000	ATP6V1F	7	Collecting		Transferrin	20								High	High	High	High	High	High	1	
FALSE	High	Master Prc	O75940	Survival of	0	4.552	14	2	2	2	238	26.7	7.24	0	2	cell death;	cytoplasm;	protein bir	Pf06003	10285	ENSG0000	SMNDC1	10	Spliceosom		mRNA Spli	5								High	High	High	High	High	High	1	
FALSE	Low	Master Prc	Q12830-1	Nucleosom	0.05	1.201	1	1	1	1	3046	338.1	6.54	0	1	cell organi	cytoplasm;	catalytic a	Pf00439, P	2186	ENSG0000	BPTF	17		Endoderm		1								High	High	High	High	High	High	1	[R113]
FALSE	High	Master Prc	Q9UDW1	cytochrom	0.001	4.288	27	1	1	1	63	7.3	9.47	3.56	1	cell organi	membrane	catalytic a	Pf05365	29796	ENSG0000	UQCR10	22	Cardiac mu	Electron Tr	Respirator	12								High	High	High	High	High	High	1	
FALSE	High	Master Prc	Q9H6Z4	Ran-bindin	0.002	3.766	2	1	2	1	567	60.2	4.78	1.66	1	cell organi	cytoplasm;	enzyme re	Pf00638	8498	ENSG0000	RANBP3	19	HTLV-I infe			1								High	High	High	High	High	High	1	
FALSE	Medium	Master Prc	O43286	Beta-1,4-g	0.016	1.691	2	1	1	1	388	45.1	8.15	0	1	metabolic	Golgi;mem	catalytic a	Pf02709, P	9334	ENSG0000	B4GALT5	20	Mucin type		Keratan su	14								High	High	High	High	High	High	1	
FALSE	High	Master Prc	Q9BTU6	Phosphatic	0	14.57	9	3	4	3	479	54	8.29	6.81	3	cell organi	cytosol;en	catalytic a	Pf00454	55361	ENSG0000	PI4K2A	10	Metabolic	Wnt Signal	Synthesis c	11								High	High	High	High	High	High	1	
FALSE	High	Master Prc	Q9BRX5	DNA replic	0	7.276	7	1	1	1	216	24.5	5.34	4.15	1	metabolic	nucleus		Pf05916	64785	ENSG0000	GINS3	16			Unwinding	7								High	High	High	High	High	High	1	
FALSE	High	Master Prc	Q9ULR0-1	Isoform 2 c	0	8.777	8	2	2	2	331	37.5	5.78	2.45	2	cell organi	endosome	catalytic a	Pf06246	10053459	ENSG0000	ISY1-RAB4	3	Spliceosom			1								High	High	High	High	High	High	1	
FALSE	Medium	Master Prc	Q9Y6M0-1	Testisin [O	0.028	1.445	4	1	1	1	314	34.9	7.62	1.97	1	metabolic	cytoplasm;	catalytic a	Pf00089	10942	ENSG0000	PRSS21	16; CHR_H			Post-transl	3								High	High	High	High	High	High	1	
FALSE	Medium	Master Prc	P78316	Nucleolar p	0.015	1.75	1	1	1	1	857	97.6	7.58	0	1	metabolic	membrane	protein bir	Pf04147	8602	ENSG0000	NOP14	4			Major path	5								High	High	High	High	High	High	1	
FALSE	High	Master Prc	Q8N6H7	ADP-ribosy	0.002	3.278	2	1	1	1	521	56.7	7.99	0	1	regulation	cytoplasm;	enzyme re	Pf01412	84364	ENSG0000	ARFGAP2	11	Endocytosi		COPI-depe	12								High	High	High	High	High	High	1	
FALSE	Medium	Master Prc	Q8N6R0-5	Methyltrar	0.011	2.006	2	1	1	1	699	78.7	6.73	0	1	metabolic		catalytic a	Pf01564, P	51603	ENSG0000	METTL13	1				0								High	High	High	High	High	High	1	
FALSE	High	Master Prc	O95239-1	Chromosor	0	9.291	3	3	3	3	1232	139.8	6.27	3.3	3	cell organi	chromosor	catalytic a	Pf00225, P	24137	ENSG0000	KIF4A	X		Retinoblas	COPI-depe	16								High	High	High	High	High	High	1	
FALSE	High	Master Prc	Q9UBF8	Phosphatic	0	5.888	2	1	1	1	816	91.3	6.25	3.96	1	metabolic	cytoplasm;	catalytic a	Pf00454	5298	ENSG0000	PI4KB	1	Phosphatic		Synthesis c	8								High	High	High	High	High	High	1	
FALSE	High	Master Prc	Q96H20-1	Vacuolar-s	0.003	2.844	5	1	1	1	258	28.8	6.65	2.05	1	cell commu	cytoplasm;	protein bir	Pf04157	11267	ENSG0000	SNF8	17	Endocytosi		Endosoma	4								High	High	High	High	High	High	1	
FALSE	High	Master Prc	Q92990	Glomulin [	0.002	4.176	2	1	1	1	594	68.2	5.33	2.55	1	cell differe	cytoplasm	enzyme re	Pf08568	11146	ENSG0000	GLMN	1			Antigen pr	4								High	High	High	High	High	High	1	
FALSE	High	Master Prc	Q13144	Translatior	0.003	3.26	2	1	1	1	721	80.3	5.08	2.83	1	cell differe	cytoplasm;	protein bir	Pf00132, P	8893	ENSG0000	EIF2B5	3	RNA transp	Translatior	Recycling c	8								High	High	High	High	High	High	1	
FALSE	High	Master Prc	O00488	Zinc finger	0.007	2.242	10	1	1	1	134	15.2	9.82	1.76	1	metabolic	cytosol;nu	DNA bindi	Pf12171	51042	ENSG0000	ZNF593	1				0								High	High	High	High	High	High	1	
FALSE	High	Master Prc	Q9H1A4	anaphase-p	0	6.26	1	2	2	2	1944	216.4	6.3	3.85	2	cell divisio	cytosol;nu		Pf01851, P	64682	ENSG0000	ANAPC1	2	Progesterc		APC/C:Cdc	37								High	High	High	High	High	High	1	
FALSE	High	Master Prc	Q13636	ras-related	0.005	2.556	8	1	1	1	194	21.6	7.06	0	1	cell organi	cytosol;en	catalytic a	Pf00009, P	11031		RAB31	18	Endocytosi		Neutrophil	11								High	High	High	High	High	High	1	
FALSE	Medium	Master Prc	Q9NVU0	DNA-direct	0.018	1.635	2	1	1	1	708	79.8	6.47	1.61	1	defense re	cytosol;nu	catalytic a	Pf04801	55718; 10	ENSG0000	POLR3E; L	16; CHR_H	Epstein-Ba	Eukaryotic	RNA Polym	18								High	High	High	High	High	High	1	
FALSE	High	Master Prc	O60826	Coiled-coil	0	6.14	5	2	2	2	627	70.7	6.74	2.95	2	cellular ho	cytosol;en	protein bir	Pf05667	28952	ENSG0000	CCDC22	X			Neddylatic	3								High	High	High	High	High	High	1	
FALSE	High	Master Prc	Q14697-2	Isoform 2 c	0	190.278	37	33	96	1	966	109.4	6.24	235.99	33	metabolic	Golgi;mem	catalytic a	Pf01055, P	23193	ENSG0000	GANAB	11				0								High	High	High	High	High	High	1	
FALSE	Medium	Master Prc	Q9NQS7	Inner centr	0.012	1.979	1	1	1	1	918	105.4	9.44	0	1	cell divisio	chromosor	protein bir	Pf03941, P	3619	ENSG0000	INCENP	11			Amplificati	21								High	High	High	High	High	High	1	
FALSE	High	Master Prc	Q01968-1	Inositol po	0	5.23	1	1	1	1	901	104.1	6.55	1.75	1	cell organi	cytoplasm;	catalytic a	Pf00620, P	4952	ENSG0000	OCRL	X	Metabolic		Synthesis c	21								High	High	High	High	High	High	1	
FALSE	High	Master Prc	Q9H9J4-1	Ubiquitin c	0.008	2.206	1	1	1	1	1324	145.3	8.63	0	1	cell differe		catalytic a	Pf00443, P	84132		USP42	7			Ub-specific	4								High	High	High	High	High	High	1	
FALSE	High	Master Prc	O60504-1	Vinexin [O	0.003	3.361	2	1	2	1	671	75.3	9.45	2.26	1	cell organi	cytoplasm;	protein bir	Pf00018, P	10174	ENSG0000	SORBS3	8			Smooth M	2								High	High	High	High	High	High	1	
FALSE	High	Master Prc	P50148	Guanine nu	0	7.507	7	2	3	1	359	42.1	5.68	4.69	2	coagulatio	cytoplasm;	catalytic a	Pf00025, P	2776	ENSG0000	GNAQ	9	cGMP-PKG	Human Th	Thrombin	64								High	High	High	High	High	High	1	
FALSE	Low	Master Prc	Q8WV92	MIT domai	0.053	1.175	2	1	1	1	249	29.3	8	0	1	cell divisio	endosome	protein bir	Pf04212	129531	ENSG0000	MITD1	2				0								High	High	High	High	High	High	1	
FALSE	High	Master Prc	Q8NFZ8	Cell adhesi	0.003	3.052	3	1	1	1	388	42.8	6.3	1.94	1		membrane	protein bir	Pf00047, P	199731	ENSG0000	CADM4	19				0								High	High	High	High	High	High	1	
FALSE	Medium	Master Prc	Q9Y6Y0	influenza v	0.011	2.027	2	1	1	1	642	71.7	5.53	0	1	metabolic	cytoplasm;	protein bir	Pf00651, P	10625	ENSG0000	IVNS1ABP	1	Influenza A			1								High	High	High	High	High	High	1	
FALSE	Low	Master Prc	P05496	ATP syntha	0.067	1.072	5	1	1	1	136	14.3	9.74	0	1	cell organi	membrane	catalytic a	Pf00137	516	ENSG0000	ATP5G1	17	Metabolic	Electron Tr	Cristae for	16								High	High	High	High	High	High	1	
FALSE	High	Master Prc	Q5NDL2	EGF domai	0.001	4.541	3	1	2	1	527	62	7.05	6.52	1	metabolic	endoplasm	catalytic a	Pf04577	285203	ENSG0000	EOGT	3	Other type			1								High	High	High	High	High	High	1	
FALSE	High	Master Prc	Q66K14-1	TBC1 dom	0	5.632	1	1	1	1	1250	140.4	5.25	3.01	1	regulation	membrane	enzyme re	Pf00566, P	23061	ENSG0000	TBC1D9B	5; CHR_HG				0								High	High	High	High	High	High	1	
FALSE	High	Master Prc	O15269	serine paln	0	5.436	5	2	2	2	473	52.7	6.01	4.73	2	metabolic	endoplasm	catalytic a	Pf00155	10558	ENSG0000	SPTLC1	9	Sphingolip	Sphingolip	Sphingolip	8								High	High	High	High	High	High	1	
FALSE	High	Master Prc	Q9NPA8-1	Transcripti	0	7.339	26	2	2	2	101	11.5	9.33	4.76	2	cell organi	mitochond		Pf10163	56943	ENSG0000	ENY2	8			HATs acety	3								High	High	High	High	High	High	1	
FALSE	High	Master Prc	Q6UX53	Methyltrar	0.002	3.75	8	2	2	2	244	27.8	8.38	2.44	2	metabolic		catalytic a	Pf01209, P	196410	ENSG0000	METTL7B	12				0								High	High	High	High	High	High	1	
FALSE	High	Master Prc	Q99700-1	Ataxin-2 [C	0	7.535	2	2	2	1	1313	140.2	9.57	4.46	2	cell organi	cytoplasm;	protein bir	Pf06741, P	6311	ENSG0000	ATXN2	12		Parkinsons		2								High	High	High	High	High	High	1	
FALSE	Low	Master Prc	P49768-1	Presenilin-	0.053	1.172	2	1	1	1	467	52.6	5.31	0	1	cell death;	cell surface	catalytic a	Pf01080	5663	ENSG0000	PSEN1	14	Notch sign	Alzheimers	Activated I	40								High	High	High	High	High	High	1	
FALSE	High	Master Prc	Q8IYU8	calcium up	0	5.845	4	1	1	1	434	49.6	9.09	4.52	1	cellular ho	membrane	metal ion l	Pf13833	221154	ENSG0000	EFHA1; MI	13			Processing	3								High	High	High	High	High	High	1	
FALSE	High	Master Prc	Q15154-1	Pericentric	0.003	3.341	1	1	1	1	2024	228.4	5.02	3.69	1	cell organi	cytoplasm;	protein bir		5108		PCM1	8			Regulation	16								High	High	High	High	High	High	1	
FALSE	High	Master Prc	Q8IZP0	abl interac	0	6.73	2	1	2	1	508	55	7.06	6	1	cell organi	cytoplasm;	enzyme re	Pf00018, P	10006	ENSG0000	ABI1	10		EGF/EGFR	Regulation	12								High	High	High	High	High	High	1	
FALSE	High	Master Prc	P68400	Casein kina	0	16.787	13	3	5	3	391	45.1	7.74	7.85	3	cell commu	chromosor	catalytic a	Pf00069, P	1457	ENSG0000	CSNK2A1	20	Epstein-Ba	TNF alpha	WNT medi	46								High	High	High	High	High	High	1	
FALSE	High	Master Prc	Q9Y4P8-1	WD repeat	0	8.074	7	2	2	2	454	49.4	6.46	3.64	2	cell commu	cytosol;me	protein bir		26100	ENSG0000	WIPI2	7			Macroautc	2								High	High	High	High	High	High	1	
FALSE	High	Master Prc	P46087	Probable 2	0	85.498	25	17	30	1	812	89.2	9.23	62.4	17	cell organi	nucleus	catalytic a	Pf01189, P	4839	ENSG0000	NOP2	12		Imatinib ai	TFAP2A ac	10								High	High	High	High	High	High	1	
FALSE	High	Master Prc	Q96S59-1	Ran-bindin	0	6.061	2	1	1	1	729	77.8	6.79	4.55	1	cell organi	cytoplasm;	protein bir	Pf00622, P	10048	ENSG0000	RANBP9	6		Brain-Deri	L1CAM int	15								High	High	High	High	High	High	1	
FALSE	High	Master Prc	Q9NUI1	Peroxisom	0.003	2.91	7	2	2	2	292	30.8	9.22	2.08	2	metabolic	membrane	catalytic a	Pf00106, P	26063	ENSG0000	DECR2	16; CHR_H	Peroxisom		Beta-oxida	6								High	High	High	High	High	High	1	

FALSE	High	Master Prc	Q86SR1	Polypeptid	0.009	2.114	1	1	1	1	603	68.9	8.59	2.49	1	metabolic	Golgi;mem	catalytic a	Pf00535, P	55568	ENSG0000	GALNT10	5	Metabolic		O-linked gl	6								High	High	High	High	High	High	1	
FALSE	High	Master Prc	Q92575	UBX doma	0	9.071	9	3	4	3	508	56.7	6.38	3.76	3	metabolic	cytosol;en	protein bir	Pf00789	23190	ENSG0000	UBXN4	2				0								High	High	High	High	High	High	1	
FALSE	High	Master Prc	Q9P2B2	prostaglan	0.003	3.165	1	1	1	1	879	98.5	6.61	1.72	1	cell organi	cell surface	protein bir	Pf07679	5738	ENSG0000	PTGFRN	1				0								High	High	High	High	High	High	1	
FALSE	High	Master Prc	Q9UDT6-1	CAP-Gly dc	0	7.613	4	4	4	1	1046	115.8	6.73	6.33	4	metabolic	cytoplasm;	catalytic a	Pf01302, P	7461	ENSG0000	CLIP2	7				0								High	High	High	High	High	High	1	
FALSE	High	Master Prc	Q9NUM4	Transmem	0	7.933	8	2	2	2	274	31.1	6.99	4.38	2	cell organi	endosome	protein bir	Pf07092	54664	ENSG0000	TMEM106	7				0								High	High	High	High	High	High	1	
FALSE	Medium	Master Prc	Q6NW34	Nucleolus	0.048	1.217	2	1	1	1	567	64.5	9.74	0	1	developme	nucleus		Pf14780	25871	ENSG0000	C3orf17; N	3				0								High	High	High	High	High	High	1	
FALSE	High	Master Prc	Q96EY1-1	DnaJ homc	0.002	3.548	3	1	2	1	480	52.5	9.26	2.31	1	cell death;	cytoplasm;	enzyme re	Pf00226, P	9093	ENSG0000	DNAJA3	16; CHR_H	Viral carcir			1								High	High	High	High	High	High	1	
FALSE	Medium	Master Prc	P36402	Transcripti	0.048	1.218	2	1	1	1	384	41.6	6.81	1.88	1	cell differe	nucleus	DNA bindi	Pf00505, P	6932		TCF7	5	Wnt signal	Arrhythmc	RUNX3 reg	33								High	High	High	High	High	High	1	
FALSE	High	Master Prc	Q8N9E0	Protein FA	0.009	2.112	3	1	1	1	248	28.9	10.1	1.76	1			protein bir		286499	ENSG0000	FAM133A	X				0								High	High	High	High	High	High	1	
FALSE	High	Master Prc	Q1ED39	Lysine-rich	0.002	3.943	4	1	1	1	458	51.6	9.86	3.19	1		nucleus	RNA bindi	Pf15477	400506	ENSG0000	C16orf88;	16				0								High	High	High	High	High	High	1	
FALSE	High	Master Prc	Q9Y324	rRNA-proc	0	5.471	6	1	2	1	198	23.4	9.7	2.48	1	metabolic	nucleus	RNA bindi	Pf04900	51077	ENSG0000	FCF1	14	Ribosome		Major path	6								High	High	High	High	High	High	1	
FALSE	Low	Master Prc	P98196	Probable p	0.075	1.015	1	1	1	1	1134	129.7	6.6	0	1	cell organi	endoplasm	catalytic a	Pf00122, P	23250	ENSG0000	ATP11A	13		Lung fibro	Neutrophil	7								High	High	High	Not Found	High	High	1	
FALSE	High	Master Prc	Q9BZQ8	Protein Nil	0	6.206	3	2	2	2	928	103.1	4.78	3.66	2	regulation	cytoplasm;	protein bir		116496	ENSG0000	FAM129A	1				0								High	High	High	High	High	High	1	
FALSE	High	Master Prc	P82664	28S riboso	0.003	2.908	6	1	1	1	201	23	8	0	1	cell organi	membrane		Pf00338	55173	ENSG0000	MRPS10	6	Ribosome		Mitochonc	7								High	High	High	High	High	High	1	
FALSE	Medium	Master Prc	Q86WI3-6	Isoform 6	0.018	1.662	1	1	1	1	1551	170.3	6.61	0	1	defense re	cytoplasm;	DNA bindi	Pf05729, P	84166		NLRC5	16				0								High	High	High	High	High	High	1	[R1533]
FALSE	Medium	Master Prc	Q08378	Golgin sub	0.028	1.449	1	1	1	1	1498	167.3	5.44	0	1	metabolic	cytoplasm;	catalytic a	Pf00038, P	2802	ENSG0000	GOLGA3	12				0								High	High	High	High	High	High	1	
FALSE	High	Master Prc	Q96SY0	Integrator	0.005	2.546	6	1	1	1	518	57.4	5.12	0	1	metabolic	nucleus		Pf13519	81556	ENSG0000	VWA9; INT	15			RNA polym	3								High	High	High	High	High	High	1	
FALSE	Medium	Master Prc	O60245-1	Protocadh	0.023	1.555	1	1	1	1	1069	116	5.19	0	1	transport	membrane	metal ion l	Pf00028, P	5099	ENSG0000	PCDH7	4			Platelet de	4								High	High	High	High	High	High	1	
FALSE	High	Master Prc	Q9NQ55-1	suppressor	0	14.411	8	4	6	4	473	53.2	10.13	8.86	4	cell organi	nucleus	RNA bindi	Pf04427	56342; 69	ENSG0000	PPAN; PPA	19				0								High	High	High	High	High	High	1	
FALSE	High	Master Prc	P51553-1	isocitrate c	0	14.682	13	3	3	3	393	42.8	8.5	5.02	3	metabolic	mitochond	catalytic a	Pf00180	3421	ENSG0000	IDH3G	X	2-Oxocarb	TCA Cycle	Mitochonc	12								High	High	High	High	High	High	1	
FALSE	High	Master Prc	Q9UK58	Cyclin-L1 [	0.003	2.855	5	1	1	1	526	59.6	10.7	2.1	1	metabolic	nucleus	enzyme re	Pf00134	57018	ENSG0000	CCNL1	3				0								High	High	High	High	High	High	1	
FALSE	High	Master Prc	P82909	28S riboso	0.002	3.915	13	1	1	1	103	11.5	9.99	2.93	1	cell organi	membrane	structural		92259	ENSG0000	MRPS36	5; CHR_HS			Mitochonc	6								High	High	High	High	High	High	1	
FALSE	Medium	Master Prc	P39210	Protein Mp	0.013	1.864	4	1	1	1	176	19.7	9.47	0	1	cell organi	membrane		Pf04117	4358	ENSG0000	MPV17	2	Peroxisom			1								High	High	High	High	High	High	1	
FALSE	Medium	Master Prc	Q86X02	Cerebellar	0.015	1.757	2	1	1	1	465	53	6.01	1.87	1			protein bir		30850	ENSG0000	CDR2L	17				0								High	High	High	High	High	High	1	
FALSE	High	Master Prc	Q15819	Ubiquitin-	0	16.144	46	5	7	2	145	16.4	8.09	16.96	5	cell prolife	cytoplasm;	catalytic a	Pf00179	7336	ENSG0000	UBE2V2	8			G2/M DNA	23								High	High	High	High	High	High	1	
FALSE	High	Master Prc	Q9NS93	Transmem	0.001	4.365	3	1	1	1	570	64.1	7.01	2.34	1	regulation	membrane		Pf13886	51768	ENSG0000	TM7SF3	12				0								High	High	High	High	High	High	1	
FALSE	High	Master Prc	P18887	DNA repai	0	6.412	5	3	4	3	633	69.4	6.39	2.31	3	metabolic	nucleus	catalytic a	Pf00533, P	7515		XRCC1	19	Base excisi		Resolution	14								High	High	High	High	High	High	1	
FALSE	Medium	Master Prc	Q9Y3D0	Mitotic spi	0.015	1.792	4	1	2	1	163	17.7	5.19	0	1	cell organi	cytoplasm;	protein bir	Pf01883	51647	ENSG0000	FAM96B	16			Cytosolic i	2								High	High	High	High	High	High	1	
FALSE	Low	Master Prc	Q8N608-1	Inactive di	0.05	1.202	1	1	1	1	796	90.8	6.57	0	1	cell organi	membrane	catalytic a	Pf00326, P	57628	ENSG0000	DPP10	2				0								High	High	High	High	High	High	1	
FALSE	Medium	Master Prc	A8MXV4	nucleoside	0.044	1.247	3	1	1	1	375	42.2	7.64	2.35	1	metabolic	mitochond	catalytic a		390916	ENSG0000	NUDT19	19	Peroxisom		Peroxisom	5								High	High	High	High	High	High	1	
FALSE	High	Master Prc	Q15651-1	High mobil	0	6.385	13	3	3	3	99	10.7	9.66	4.47	3	cell organi	chromosor	DNA bindi		9324	ENSG0000	HMGN3	6				0								High	High	High	High	High	High	1	
FALSE	Medium	Master Prc	Q9UKL6	Phosphatic	0.042	1.27	8	1	1	1	214	24.8	5.78	0	1	metabolic	cytoplasm;	protein bir	Pf01852	58488	ENSG0000	PCTP	17			Synthesis c	7								High	High	High	High	High	High	1	[R136;
FALSE	High	Master Prc	Q92600	CCR4-NOT	0.002	4.113	8	2	3	2	299	33.6	8.03	4.16	2	metabolic	cytoplasm;	protein bir	Pf04078	9125	ENSG0000	RQCD1; CN	2	RNA degra		Activation	13								High	High	High	High	High	High	1	
FALSE	Low	Master Prc	Q6JBY9	CapZ-inter	0.071	1.035	2	1	1	1	416	44.5	5.4	1.63	1	response t		protein bir	Pf05177, P	92241	ENSG0000	RCSD1	1				0								High	High	High	High	High	High	1	
FALSE	High	Master Prc	Q5T440	Putative tr	0	5.728	4	1	1	1	356	38.1	9.83	4.56	1	cell organi	mitochond	catalytic a	Pf01571, P	200205	ENSG0000	IBA57	1				0								High	High	High	High	High	High	1	
FALSE	Medium	Master Prc	Q9UJW0-1	Dynactin s	0.044	1.258	2	1	1	1	460	52.3	7.34	0	1	transport	chromosor	protein bir	Pf05502	51164	ENSG0000	DCTN4	5	Vasopressi		COPI-indep	19								High	High	High	High	High	High	1	
FALSE	Low	Master Prc	Q8WY21-3	Isoform 3	0.057	1.137	1	1	1	1	1179	131.2	7.11	0	1	regulation	membrane	protein bir	Pf00801	114815		SORCS1	10				0								High	High	High	High	High	High	1	
FALSE	High	Master Prc	Q8NG11-1	Tetraspani	0.003	3.416	4	1	1	1	270	30.7	6.84	2.06	1	cell organi	membrane	protein bir	Pf00335	81619	ENSG0000	TSPAN14	10			Neutrophil	3								High	High	High	High	High	High	1	
FALSE	Low	Master Prc	P47929	galectin-7	0.067	1.069	12	1	1	1	136	15.1	7.62	0	1	cell death	cytoplasm;		Pf00337	3963; 653	ENSG0000	LGALS7; LC	19; CHR_H				0								High	High	High	High	High	High	1	[S94(100)]
FALSE	High	Master Prc	P35222	Catenin be	0	5.399	2	2	2	1	781	85.4	5.86	3.17	2	cell commu	cytoplasm;	DNA bindi	Pf00514	1499	ENSG0000	CTNNB1	3	Endometri	Androgen	Beta-caten	107								High	High	High	High	High	High	1	
FALSE	Medium	Master Prc	Q02040-1	A-kinase a	0.031	1.43	1	1	1	1	695	80.7	9.73	0	1	metabolic	cytosol;nu	protein bir	Pf00076	8227	ENSG0000	AKAP17A	X|Y; X				0								High	High	High	High	High	High	1	
FALSE	Medium	Master Prc	Q8IWE4	DCN1-like	0.018	1.646	5	1	1	1	304	34.3	5.12	0	1	metabolic	membrane	protein bir	Pf03556	123879	ENSG0000	DCUN1D3	16			Neddylatic	3								High	High	High	High	High	High	1	
FALSE	High	Master Prc	Q9H446	rwd domai	0	10.275	11	2	2	2	243	27.9	4.2	7.04	2	metabolic	cytoplasm	protein bir	Pf05773	51389	ENSG0000	RWDD1	6				0								High	High	High	High	High	High	1	
FALSE	High	Master Prc	Q14344	Guanine n	0	20.148	9	3	5	2	377	44	8	10.26	3	cell differe	cytoplasm;	catalytic a	Pf00025, P	10672	ENSG0000	GNA13	17	Vascular sr	Human Th	Thrombin	28								High	High	High	High	High	High	1	
FALSE	Medium	Master Prc	P09758	Tumor-ass	0.024	1.532	4	1	1	1	323	35.7	8.87	0	1	cell prolife	cytosol;me	protein bir	Pf00086	4070	ENSG0000	TACSTD2	1				0								High	High	High	High	High	High	1	
FALSE	High	Master Prc	P05161	Ubiquitin-l	0.003	3.217	10	2	2	2	165	17.9	7.44	2.43	2	defense re	cytoplasm;	protein bir	Pf00240	9636	ENSG0000	ISG15	1	RIG-I-like r	RIG-I-like F	Interferon	21								High	High	High	High	High	High	1	
FALSE	High	Master Prc	Q3KQZ1-1	Solute carr	0.001	4.457	4	1	1	1	300	32.4	9.11	2.3	1	transport	membrane	transporte	Pf00153	399512	ENSG0000	SLC25A35	17				0								High	High	High	High	High	High	1	
FALSE	High	Master Prc	O96013-1	serine/thre	0.003	3.324	2	1	1	1	591	64	9.73	0	1	cell death;	cytoplasm;	catalytic a	Pf00069, P	10298	ENSG0000	PAK4	19	Ras signali	Integrin-m	Activation	16								High	High	High	High	High	High	1	
FALSE	High	Master Prc	Q8N490	Probable h	0.003	3.442	2	1	1	1	385	42.8	9.09	0	1	metabolic	cytoplasm;	catalytic a	Pf00753, P	25953	ENSG0000	PNKD	2				0								High	High	High	High	High	High	1	
FALSE	High	Master Prc	O14531	dihydropyr	0.001	4.488	3	1	1	1	572	61.8	7.09	2.24	1	cell death;	cytoplasm;	catalytic a	Pf01979, P	10570	ENSG0000	DPYSL4	10			CRMPs in S	4								High	High	High	High	High	High	1	
FALSE	High	Master Prc	O94888	UBX doma	0.001	4.236	2	1	1	1	489	54.8	5.16	1.86	1	metabolic	cytosol;nu	protein bir	Pf00789, P	26043	ENSG0000	UBXN7	3			Neddylatic	3								High	High	High	High	High	High	1	
FALSE	High	Master Prc	P54278	Mismatch	0	4.646	3	2	2	2	862	95.7	6.86	0	2	metabolic	cytoskelet	catalytic a	Pf01119, P	5395; 107	ENSG0000	PMS2; LOC	7	Mismatch		Mismatch	15								High	High	High	High	High	High	1	
FALSE	Low	Master Prc	Q53GD3-1	choline tra	0.051	1.194	1	1	2	1	710	79.2	8.59	0	1	metabolic	membrane	transporte	Pf04515	80736	ENSG0000	SLC44A4	6; CHR_HS	Choline me		Synthesis c	9								High	High	High	High	High	High	1	
FALSE	High	Master Prc	P49840	Glycogen s	0.003	3.48	2	1	1	1	483	50.9	8.75	1.6	1	cellular co	cytoplasm;	catalytic a	Pf00069, P	2931	ENSG0000	GSK3A	19	Dopamine	IL-5 Signali	AKT phosp	25								High	High	High	High	High	High	1	
FALSE	High	Master Prc	P22570	NADPH:ad	0.003	3.466	3	1	1	1	491	53.8	8.44	2.39	1	metabolic	mitochond	catalytic a	Pf13450	2232		FDXR	17			Endogenou	15								High	High	High	High	High	High	1	
FALSE	High	Master Prc	P82914	28S riboso	0.003	2.998	4	1	2	1	257	29.8	10.48	4.05	1	cell organi	membrane	RNA bindi	Pf00312	64960	ENSG0000	MRPS15	1	Ribosome		Mitochonc	7								High	High	High	High	High	High	1	
FALSE	High	Master Prc	Q9Y4K0	Lysyl oxida	0	9.228	2	1	1	1	774	86.7	6.38	5.59	1	cell organi	chromosor	catalytic a	Pf00530, P	4017	ENSG0000	LOXL2	8		Canonical	Elastic fibr	6								High	High	High	High	High	High	1	
FALSE	High	Master Prc	O60264	SWI/SNF-r	0	6.826	3	3	3	3	1052	121.8	8.09	0	3	cell organi	chromosor	catalytic a	Pf00176, P	8467	ENSG0000	SMARCA5	4			Recruitme	14								High	High	High	High	High	High	1	
FALSE	High	Master Prc	Q9H0H5	Rac GTPas	0.002	4.191	5	2	2	2	632	71	8.88	0	2	cell differe	cytoplasm;	catalytic a	Pf00130, P	29127	ENSG0000	RACGAP1	12		Signal Trar	COPI-depe	16								High	High	High	High	High	High	1	
FALSE	High	Master Prc	Q9H7E9-1	UPF0488 p	0.005	2.561	5	1	1	1	229	25	9.95	1.92	1			protein bir	Pf15393	65265	ENSG0000	C8orf33	8				0								High	High	High	High	High	High	1	
FALSE	High	Master Prc	P16035	Metallopro	0.003	3.105	7	1	1	1	220	24.4	7.49	1.85	1	cell organi	cell surface	enzyme re	Pf00965	7077	ENSG0000	TIMP2	17		Matrix Me	Activation	9								High	High	High	High	High	High	1	
FALSE	Medium	Master Prc	Q9BTC8	Metastasis	0.016	1.711	1	1	1	1	594	67.5	8.57	0	1	cell organi	cytoplasm;	catalytic a	Pf00320, P	57504	ENSG0000	MTA3	2		Preimplant	ERCC6 (CSI	16								High	High	High	High	High	High	1	
FALSE	High	Master Prc	Q9BTC0	Death-indu	0	9.842	2	5	5	5	2240	243.7	7.88	4.37	5	cell death;	cytoplasm;	metal ion l	Pf00628, P	11083	ENSG0000	DIDO1	20				0								High	High	High	High	High	High	1	
FALSE	Medium	Master Prc	Q06547	GA-binding	0.042	1.265	2	1	1	1	395	42.5	4.86	1.68	1	cell organi	nucleus	DNA bindi	Pf00023, P	2553	ENSG0000	GABPB1	15		Myometria	Transcripti	6								High	High	High	High	High	High	1	
FALSE	High	Master Prc	Q96FZ2	embryonic	0	19.335	19	5	5	5	354	40.5	8.15	9.25	5	metabolic		catalytic a	Pf02586	56941	ENSG0000	C3orf37; H	3				0								High	High	High	High	High	High	1	
FALSE	Medium	Master Prc	Q8IXM3	39S riboso	0.013	1.913	7	1	1	1	137	15.4	9.57	0	1	cell death;	membrane	protein bir	Pf09809	64975	ENSG0000	MRPL41	9			Mitochonc	6								High	High	High	High	High	High	1	
FALSE	High	Master Prc	Q5R3I4	tetratricop	0.002	3.872	2	1	1	1	469	52.8	5.99	1.69	1			protein bir		55020	ENSG0000	TTC38	22				0								High	High	High	High	High	High	1	
FALSE	High	Master Prc	Q92508	Piezo-type	0.007	2.302	0	1	1	1	2521	286.6	7.47	2.24	1	regulation	endoplasm	transporte	Pf12166	9780	ENSG0000	PIEZO1	16				0								High	High	High	High	High	High	1	
FALSE	High	Master Prc	Q14558	Phosphorib	0	7.497	9	3	3	2	356	39.4	7.2	4.63	3	metabolic		catalytic a	Pf13793, P	5635		PRPSAP1	17				0								High	High	High	High	High	High	1	
FALSE	High	Master Prc	Q96EL2	28S riboso	0.004	2.724	7	1	1	1	167	19	9.38	0	1	cell organi	membrane	RNA bindi	Pf14955	64951	ENSG0000	MRPS24	7			Mitochonc	6								High	High	High	High	High	High	1	
FALSE	High	Master Prc	Q99543-1	dnaJ homc	0	15.053	8	5	6	5	621	72	8.7	5.24	5	cell organi	cytoplasm;	DNA bindi	Pf00226, P	27000	ENSG0000	DNAJC2	7			Regulation	4								High	High	High	High	High	High	1	
FALSE	High	Master Prc	Q9UDX5	Mitochonc	0.004	2.777	7	1	1	1	166	18	9.31	1.66	1	cell death;	membrane		Pf10558	51537	ENSG0000	MTFP1	22				0								High	High	High	High	High	High	1	
FALSE	High	Master Prc	P52298-1	Nuclear ca	0.002	4.144	10	2	2	2	156	18	8.21	2.09	2	metabolic	cytoplasm;	protein bir	Pf00076, P	22916	ENSG0000	NCBP2	3	Spliceosom	mRNA Pro	Signaling b	61								High	High	High	High	High	High	1	
FALSE	Medium	Master Prc	Q05519	serine/argi	0.031	1.421	4	1	1	1	484	53.5	10.52	0	1	metabolic	nucleus	protein bir	Pf00076, P	9295	ENSG0000	SRSF11	1			Cleavage o	11								High	High	High	High	High	High	1	
FALSE	Medium	Master Prc	Q9Y5R2	Matrix me	0.042	1.272	2	1	1	1	645	73.2	9.23	0	1	cell differe	extracellul	catalytic a	Pf00045, P	10893	ENSG0000	MMP24	20		Matrix Me	Activation	4								High	High	High	High	High	High	1	[R62]
FALSE	Medium	Master Prc	Q9HD42-1	Charged m	0.015	1.76	4	1	1	1	196	21.7	8.06	1.73	1	cell commu	chromosor	catalytic a	Pf03357	5119	ENSG0000	CHMP1A	16	Endocytosi			1								High	High	High	High	High	High	1	
FALSE	High	Master Prc	Q9UJX2-1	cell divisio	0.006	2.537	2	1	1	1	597	68.8	7.02	0	1	cell divisio	cytosol	catalytic a	Pf00515, P	8697	ENSG0000	CDC23	5	Progesterc		APC/C:Cdc	37								High	High	High	High	High	High	1	
FALSE	Medium	Master Prc	Q8N2M8	CLK4-assoc	0.039	1.285	1	1	1	1	674	77.1	10.45	0	1	metabolic	nucleus	protein bir	Pf09750	11129		CLASRP	19		mRNA Pro		1								High	High	High	High	High	High	1	
FALSE	High	Master Prc	Q9H6X2	Anthrax to	0.001	4.514	2	1	2	1	564	62.7	7.61	2.24	1	cell organi	cell surface	metal ion l	Pf00092, P	84168	ENSG0000	ANTXR1	2	NOD-like r		Uptake an	5								High	High	High	High	High	High	1	

FALSE	Medium	Master Prc	Q9HAF1	Chromatin	0.01	2.064	4	1	1	1	191	21.6	9.32	0	1	cell organi:	chromosor	protein bir	Pf09340, P	64769	ENSG0000	MEAF6	1			HATs acety	9								High	High	High	High	High	High	1	
FALSE	High	Master Prc	P25325	3-mercapt	0	10.736	12	3	4	3	297	33.2	6.6	1.94	3	metabolic	cytoplasm;	catalytic a	Pf00581	4357	ENSG0000	MPST	22	Sulfur met	Trans-sulfu		6								High	High	High	High	High	High	1	
FALSE	High	Master Prc	Q7Z7E8-1	Ubiquitin-c	0.003	2.975	4	2	2	2	422	46.1	5.1	0	2	metabolic	cytoplasm;	catalytic a		55585	ENSG0000	UBE2Q1	1	Ubiquitin r		Antigen pr	5								High	High	High	High	High	High	1	
FALSE	High	Master Prc	Q9BRG1	Vacuolar p	0.002	3.962	6	1	1	1	176	20.7	6.34	0	1	cell commu	cytoplasm;	protein bir	Pf05871	84313	ENSG0000	VPS25	17	Endocytosi		Endosoma	4								High	High	High	High	High	High	1	
FALSE	High	Master Prc	Q6P161	39S riboso	0.003	3.207	7	1	1	1	138	15.8	9.6	1.65	1	cell organi:	membrane	RNA bindi	Pf08561	116541	ENSG0000	MRPL54	19			Mitochonc	6								High	High	High	High	High	High	1	
FALSE	High	Master Prc	Q96FV2	Secernin-2	0.008	2.215	2	1	1	1	425	46.6	5.67	0	1	metabolic		catalytic a	Pf03577	90507	ENSG0000	SCRN2	17				0								High	High	High	High	High	High	1	
FALSE	High	Master Prc	Q9UDY4	DnaJ homc	0.006	2.44	3	1	1	1	337	37.8	8.5	0	1	metabolic	cytoplasm;	enzyme re	Pf00226, P	11080	ENSG0000	DNAJB4	1				0								High	High	High	High	High	High	1	
FALSE	High	Master Prc	O95466-2	Isoform 2 c	0	15.632	6	5	5	5	1104	122.3	5.83	6.99	5	cell organi:	cytosol;me	protein bir	Pf02181, P	752		FMNL1	17				0								High	High	High	High	High	High	1	
FALSE	Medium	Master Prc	Q99959	Plakophilir	0.036	1.345	1	1	1	1	881	97.4	9.33	0	1	cell commu	cytoskelet	protein bir		5318	ENSG0000	PKP2	12	Arrhythmc	Arrhythmc	Formation	5								High	High	High	High	High	High	1	
FALSE	Medium	Master Prc	P58557	Endoribon	0.011	2.032	7	1	1	1	167	19.3	7.55	1.8	1	metabolic	mitochond	catalytic a	Pf02130	54059	ENSG0000	YBEY	21				0								High	High	High	High	High	High	1	
FALSE	Low	Master Prc	Q13772-1	Nuclear re	0.067	1.067	1	1	1	1	614	69.7	6.01	0	1	cellular ho	mitochond	protein bir	Pf12489	8031	ENSG0000	NCOA4	10	Pathways i	Androgen		4								High	High	High	High	High	High	1	
FALSE	Medium	Master Prc	Q9Y216	Myotubula	0.049	1.209	1	1	1	1	660	75.8	6.35	0	1	metabolic	cytoplasm;	catalytic a	Pf06602	9108	ENSG0000	MTMR7	8	Inositol ph		Synthesis c	10								High	High	High	High	High	High	1	
FALSE	High	Master Prc	Q9NQC1-1	E3 ubiquiti	0	4.786	2	1	1	1	790	87.4	5.26	3.06	1	cell organi:		catalytic a	Pf00628, P	23338	ENSG0000	PHF15; JAI	5			HATs acety	3								High	High	High	High	High	High	1	
FALSE	High	Master Prc	Q92685-1	Dol-P-Man	0	4.799	3	1	1	1	438	50.1	9.44	0	1	metabolic	endoplasm	catalytic a	Pf05208	10195	ENSG0000	ALG3	3	N-Glycan b		Defective A	10								High	High	High	High	High	High	1	
FALSE	Medium	Master Prc	Q9UJL9-1	Zinc finger	0.049	1.211	2	1	1	1	534	61.4	9.01	2.16	1	metabolic	nucleus	DNA bindi	Pf00096, P	65243	ENSG0000	ZFP69B; ZI	1			Generic Tr	3								High	High	High	High	High	High	1	[S140(100
FALSE	Medium	Master Prc	Q13686	Nucleic aci	0.012	1.996	2	1	1	1	389	43.8	7.08	0	1	cell differe	mitochond	catalytic a	Pf13532	8846	ENSG0000	ALKBH1	14				0								High	High	High	High	High	High	1	
FALSE	High	Master Prc	Q6GQQ9-1	OTU doma	0	7.557	3	1	1	1	843	92.5	6.71	0	1	metabolic	cytoplasm;	catalytic a	Pf01754, P	56957	ENSG0000	OTUD7B	1		TNF alpha	Regulation	11								High	High	High	High	High	High	1	
FALSE	High	Master Prc	Q13616	Cullin-1 [O	0	7.569	4	2	2	2	776	89.6	8	6.26	2	cell death;	cytosol	catalytic a	Pf00888, P	8454	ENSG0000	CUL1	7	TGF-beta s	TNF alpha	FBXL7 dow	104								High	High	High	High	High	High	1	
FALSE	High	Master Prc	Q96AT1	Uncharact	0.004	2.77	7	1	1	1	154	17.5	6.11	0	1			protein bir	Pf15377	57456	ENSG0000	KIAA1143	3; CHR_HG				0								High	High	High	High	High	High	1	
FALSE	High	Master Prc	Q15038-1	DAZ-associ	0.002	3.665	9	1	1	1	168	17.3	7.33	2.63	1		cytoplasm;	protein bir	Pf11029	9802	ENSG0000	DAZAP2	12		Exercise-in		1								High	High	High	High	High	High	1	
FALSE	High	Master Prc	P81605	Dermcidin	0.002	4.141	23	2	3	2	110	11.3	6.54	1.96	2	defense re	extracellul	catalytic a	Pf15291	117159	ENSG0000	DCD	12			Antimicrob	3								High	High	High	High	High	High	1	
FALSE	High	Master Prc	Q9H1P3-1	Oxysterol-l	0.003	2.901	2	1	1	1	480	55.2	6.35	2.05	1	metabolic	cytosol	protein bir	Pf01237	9885	ENSG0000	OSBPL2	20			Synthesis c	5								High	High	High	High	High	High	1	
FALSE	High	Master Prc	Q96B23-1	Uncharact	0.001	4.487	3	1	1	1	403	43.3	4.88	1.91	1	metabolic		catalytic a	Pf15303	147339		C18orf25	18				0								High	High	High	High	High	High	1	
FALSE	Medium	Master Prc	P30038	Delta-1-py	0.022	1.556	2	1	1	1	563	61.7	8.07	0	1	metabolic	mitochond	catalytic a	Pf00171	8659	ENSG0000	ALDH4A1	1	Alanine, as		Glyoxylate	8								High	High	High	High	High	High	1	
FALSE	High	Master Prc	Q9NRH3	tubulin gar	0	17.37	12	2	2	1	451	51.1	5.8	8.18	2	cell organi:	cytoplasm;	catalytic a	Pf00091, P	27175	ENSG0000	TUBG2	17			Recruitme	9								High	High	High	High	High	High	1	[Q429;
FALSE	High	Master Prc	Q05086	Ubiquitin-p	0.002	3.289	1	1	2	1	875	100.6	5.22	1.68	1	cellular coi	cytoplasm;	catalytic a	Pf00632	7337	ENSG0000	UBE3A	15	Ubiquitin r	Androgen	Antigen pr	9								High	High	High	High	High	High	1	
FALSE	Medium	Master Prc	Q969Q5	Ras-relatec	0.012	1.938	4	1	1	1	203	23.1	6.23	1.76	1	metabolic	cytoplasm;	catalytic a	Pf00025, P	53917	ENSG0000	RAB24	5			Neutrophil	6								High	High	High	High	High	High	1	
FALSE	High	Master Prc	Q9GZR2-1	RNA exonu	0	4.825	4	2	3	2	422	46.6	9.77	4.1	2	metabolic	nucleus	catalytic a	Pf00929	57109	ENSG0000	REXO4	9; CHR_HG				0								High	High	High	High	High	High	1	
FALSE	Low	Master Prc	Q9Y616	Interleukin	0.053	1.168	2	1	1	1	596	67.7	6.7	0	1	metabolic	cytoplasm;	catalytic a	Pf00069, P	11213	ENSG0000	IRAK3	12	Neurotrop	Regulation	Interleukin	15								High	High	High	High	High	High	1	[S275(100
FALSE	High	Master Prc	Q9Y4X5	E3 ubiquiti	0.001	4.5	2	1	1	1	557	64.1	5.08	2.66	1	metabolic	cytoplasm;	catalytic a	Pf01485	25820	ENSG0000	ARIH1	15			ISG15 anti	5								High	High	High	High	High	High	1	
FALSE	High	Master Prc	Q9BR61	Acyl-CoA-b	0.002	3.694	10	2	2	2	282	31.1	5.11	2.22	2	metabolic	cytoplasm;	protein bir	Pf00023, P	84320	ENSG0000	ACBD6	1			Mitochonc	4								High	High	High	High	High	High	1	
FALSE	High	Master Prc	Q5VSL9-1	striatin-int	0.002	3.523	2	1	1	1	837	95.5	6.29	2.68	1	cell organi:	cytoplasm;	protein bir	Pf07923, P	85369	ENSG0000	STRIP1	1				0								High	High	High	High	High	High	1	
FALSE	High	Master Prc	Q9GZN8	UPF0687 p	0.006	2.331	8	1	1	1	174	19.3	6.84	2.57	1				Pf15006	54976	ENSG0000	C20orf27	20				0								High	High	High	High	High	High	1	
FALSE	High	Master Prc	Q58FF7	Putative he	0	234.665	31	32	227	1	597	68.3	4.79	471.66	32	metabolic	cytoplasm	nucleotide	Pf00183, P	3327		HSP90AB3	4				0								High	High	High	High	High	High	1	
FALSE	High	Master Prc	Q6NW29	RWD doma	0.004	2.7	5	1	1	1	188	21.2	5.31	0	1			protein bir	Pf05773	201965	ENSG0000	RWDD4	4				0								High	High	High	High	High	High	1	
FALSE	High	Master Prc	Q8NBM4-1	Ubiquitin-a	0.003	3.151	8	2	2	2	344	38.9	9.01	0	2		endoplasm	catalytic a	Pf00627	337867	ENSG0000	UBAC2	13				0								High	High	High	High	High	High	1	
FALSE	High	Master Prc	Q9BVW5	TIMELESS-i	0.002	3.949	3	1	1	1	301	34.5	4.82	0	1	cell divisio	cytoplasm;	DNA bindi	Pf07962	54962	ENSG0000	TIPIN	15			Processing	5								High	High	High	High	High	High	1	
FALSE	High	Master Prc	Q92791	Endoplasm	0	4.667	3	1	1	1	437	50.3	4.77	2.11	1	cell organi:	chromosor	protein bir		10609	ENSG0000	LEPREL4; P	17				0								High	High	High	High	High	High	1	
FALSE	High	Master Prc	Q96IX5	Up-regulat	0.002	3.957	17	1	2	1	58	6.5	9.76	0	1		membrane		Pf14960	84833	ENSG0000	USMG5	10				0								High	High	High	High	High	High	1	
FALSE	Medium	Master Prc	Q8N3V7-1	synaptopo	0.017	1.674	1	1	1	1	929	99.4	8.72	0	1	regulation	cytoplasm;	protein bir		11346	ENSG0000	SYNPO	5		Primary Fc		1								High	High	High	High	High	High	1	
FALSE	High	Master Prc	Q99417	C-Myc-binc	0	5.73	30	2	2	2	103	12	5.91	3.62	2	metabolic	cytoplasm;	protein bir		26292	ENSG0000	MYCBP	1		Pathways A		1								High	High	High	High	High	High	1	
FALSE	Low	Master Prc	Q96P50	Arf-GAP w	0.071	1.037	2	1	1	1	834	92.4	5.99	0	1	regulation		enzyme re	Pf00023, P	116983	ENSG0000	ACAP3	1	Endocytosi			1								High	High	High	High	High	High	1	[Q105;
FALSE	Medium	Master Prc	Q86WA6	valacyclovi	0.015	1.727	3	1	1	1	291	32.5	9.14	0	1	metabolic	membrane	catalytic a	Pf00561, P	670	ENSG0000	BPHL	6			Phase I - Fu	3								High	High	High	High	High	High	1	
FALSE	High	Master Prc	Q9Y4H2	Insulin rec	0.002	4.029	1	1	1	1	1338	137.2	8.65	2.08	1	cell prolife	cytoplasm;	catalytic a	Pf00169, P	8660	ENSG0000	IRS2	13	Adipocytol	Prolactin S	PI3K Casca	61								High	High	High	High	High	High	1	
FALSE	Low	Master Prc	Q9Y5W8	Sorting ne	0.067	1.071	1	1	1	1	968	112.1	6.58	0	1	regulation	endosome		Pf00615, P	23161		SNX13	7				0								High	High	High	High	High	High	1	[T897(100
FALSE	High	Master Prc	Q9BVG4	Protein PB	0.002	4.07	6	1	1	1	233	26	4.79	4.17	1		cytoplasm		Pf04669	51260	ENSG0000	CXorf26; P	X				0								High	High	High	High	High	High	1	
FALSE	High	Master Prc	Q9C0H2-1	Protein tw	0.006	2.436	3	1	1	1	523	57.5	5.39	1.87	1	transport	membrane	transporte	Pf04906	80727	ENSG0000	TTYH3	7			Stimuli-ser	3								High	High	High	High	High	High	1	
FALSE	Medium	Master Prc	Q96F63	Coiled-coil	0.022	1.559	3	1	1	1	343	38.9	4.59	0	1				Pf09747	90324	ENSG0000	CCDC97	19				0								High	High	High	High	High	High	1	
FALSE	Low	Master Prc	Q9Y4E6-1	WD repeat	0.066	1.075	1	1	1	1	1490	163.7	6.92	0	1	cell differe	cytoplasm	protein bir	Pf00400	23335	ENSG0000	WDR7	18				0								High	High	High	High	High	High	1	[R1407]
FALSE	Medium	Master Prc	Q7Z333	Probable h	0.036	1.333	0	1	1	1	2677	302.7	7.17	0	1	cell differe	chromosor	catalytic a	Pf00580, P	23064	ENSG0000	SETX	9		Interactom		1								High	High	High	High	High	High	1	
FALSE	High	Master Prc	Q8IX03	Protein KIE	0.001	4.543	2	2	2	1	1113	125.2	5.85	1.97	2	cellular coi	cytoplasm;	protein bir	Pf00397	23286	ENSG0000	WWC1	5	Hippo sign		Signaling b	4								High	High	High	High	High	High	1	
FALSE	High	Master Prc	Q13867	bleomycin	0	4.753	4	2	2	2	455	52.5	6.27	1.63	2	metabolic	cytoplasm;	catalytic a	Pf03051	642	ENSG0000	BLMH	17			Antigen pr	4								High	High	High	High	High	High	1	
FALSE	High	Master Prc	Q9Y6B6	GTP-bindir	0	18.98	27	4	6	2	198	22.4	6.11	10.08	4	cell organi:	cytosol;en	catalytic a	Pf00025, P	51128	ENSG0000	SAR1B	5	Protein prc	Sterol Reg	Regulation	25								High	High	High	High	High	High	1	
FALSE	Medium	Master Prc	P30260	cell divisio	0.01	2.076	1	1	2	1	824	91.8	7.02	0	1	cell prolife	cytoplasm;	protein bir	Pf00515, P	996	ENSG0000	CDC27	17	Oocyte me		APC/C:Cdc	37								High	High	High	High	High	High	1	
FALSE	High	Master Prc	P83111-1	Serine beta	0.001	4.428	2	1	2	1	547	60.7	8.53	4.59	1	metabolic	cytosol;mi	catalytic a	Pf00144	114294	ENSG0000	LACTB	15				0								High	High	High	High	High	High	1	
FALSE	High	Master Prc	Q92506	estradiol 1	0	5.288	8	2	3	2	261	27	6.54	0	2	cell organi:	membrane	catalytic a	Pf00106, P	7923	ENSG0000	HSD17B8	6; CHR_HS	Steroid ho		Fatty acyl-	6								High	High	High	High	High	High	1	
FALSE	Medium	Master Prc	Q8N1I0	Dedicator	0.048	1.222	0	1	1	1	1966	225.1	7.65	0	1	cellular coi	cytoplasm;	enzyme re	Pf00018, P	9732	ENSG0000	DOCK4	7	Rap1 signa		Factors inv	3								High	High	High	High	High	High	1	[Q705]
FALSE	High	Master Prc	P49916-1	DNA ligase	0	5.684	2	2	3	2	1009	112.8	9.01	2.02	2	cell divisio	mitochond	catalytic a	Pf00645, P	3980	ENSG0000	LIG3	17	Base excisi		Resolution	14								High	High	High	High	High	High	1	
FALSE	Medium	Master Prc	Q8N3C0	Activating	0.016	1.688	0	1	1	1	2202	251.3	7.09	0	1	cell prolife	cytosol;me	catalytic a	Pf00270, P	10973	ENSG0000	ASCC3	6		Mesoderm	ALKBH3 m	5								High	High	High	High	High	High	1	
FALSE	Medium	Master Prc	P62306	Small nucl	0.032	1.396	9	1	1	1	86	9.7	4.67	0	1	cell organi:	cytoplasm;	protein bir	Pf01423	6636	ENSG0000	SNRPF	12	Spliceosom	mRNA Pro	SLBP indep	16								High	High	High	High	High	High	1	
FALSE	High	Master Prc	Q9H0U6	39S riboso	0.001	4.429	9	1	1	1	180	20.6	9.54	0	1	cell organi:	membrane	RNA bindi	Pf00861	29074	ENSG0000	MRPL18	6	Ribosome		Mitochonc	7								High	High	High	High	High	High	1	
FALSE	High	Master Prc	Q969T9	WW doma	0.003	3.061	4	1	1	1	261	28.1	5.91	0	1	regulation	cytoplasm;	DNA bindi	Pf02893, P	23558	ENSG0000	WBP2	17				0								High	High	High	High	High	High	1	
FALSE	Medium	Master Prc	Q9NQ84-1	G-protein c	0.012	1.955	2	1	1	1	441	48.2	8.43	2.5	1	regulation	membrane	receptor a	Pf00003	55890		GPRC5C	17		GPCRs, Cla		1								High	High	High	High	High	High	1	
FALSE	High	Master Prc	Q14353-1	guanidinoa	0.01	2.092	3	1	2	1	236	26.3	6.14	2.31	1	metabolic	cytoplasm;	catalytic a	Pf13659	2593	ENSG0000	GAMT	19	Arginine ai	Mecp2 anc	Creatine m	9								High	High	High	High	High	High	1	
FALSE	Medium	Master Prc	Q8NCE0-1	tRNA-splic	0.013	1.875	2	1	1	1	465	53.2	7.85	0	1	metabolic	cytosol;nu	catalytic a	Pf01974, P	80746	ENSG0000	TSEN2	3			tRNA proc	3								High	High	High	High	High	High	1	
FALSE	High	Master Prc	Q9UK61	Protein TA	0	4.917	1	2	2	2	1670	188.9	5.8	0	2	metabolic	chromosor	protein bir	Pf12509	23272		FAM208A	3				0								High	High	High	High	High	High	1	
FALSE	Low	Master Prc	P26378-1	ELAV-like p	0.056	1.144	6	1	1	1	380	41.7	9.39	2.28	1	metabolic		RNA bindi	Pf00076, P	1996	ENSG0000	ELAVL4	1				0								High	High	High	High	High	High	1	
FALSE	High	Master Prc	P49674	Casein kina	0	6.525	3	1	1	1	416	47.3	9.66	3.03	1	metabolic	cytoplasm;	catalytic a	Pf00069, P	1454; 102	ENSG0000	CSNK1E; L	22	FoxO signa	Melatonin	WNT medi	35								High	High	High	High	High	High	1	
FALSE	High	Master Prc	P09417	dihydropte	0.005	2.598	7	1	1	1	244	25.8	7.37	2.48	1	metabolic	cytoplasm;	catalytic a		5860	ENSG0000	QDPR	4	Metabolic		Phenylalar	6								High	High	High	High	High	High	1	
FALSE	Medium	Master Prc	Q8ND83-1	SLAIN mot	0.015	1.783	1	1	1	1	568	60.6	7.64	0	1		cytoplasm;	protein bir	Pf15301	122060	ENSG0000	SLAIN1	13				0								High	High	High	High	High	High	1	
FALSE	High	Master Prc	P84157-1	Matrix-ren	0.003	3.061	5	1	2	1	204	21.5	4.26	1.67	1		membrane			439921	ENSG0000	MXRA7	17				0								High	High	High	High	High	High	1	
FALSE	Medium	Master Prc	Q9BRJ2	39S riboso	0.026	1.471	3	1	1	1	306	35.3	9.03	0	1	cell organi:	membrane	protein bir	Pf04280	84311	ENSG0000	MRPL45	17; CHR_H			Mitochonc	6								High	High	High	High	High	High	1	
FALSE	Medium	Master Prc	Q9NZW5	MAGUK p5	0.013	1.887	2	1	1	1	540	61.1	6.18	0	1	cell organi:	membrane	protein bir	Pf00018, P	51678	ENSG0000	MPP6	7				0								High	High	High	High	High	High	1	
FALSE	High	Master Prc	O75044	SLIT-ROBO	0	7.35	2	2	2	2	1071	120.8	6.7	3.99	2	cell organi:	cytoplasm;	enzyme re	Pf00018, P	23380		SRGAP2	1	Axon guida		RHO GTPa:	10								High	High	High	High	High	High	1	
FALSE	Medium	Master Prc	Q9BSR8	protein YIF	0.016	1.69	4	1	2	1	244	27.1	4.65	0	1		endoplasm	protein bir		84272	ENSG0000	YIPF4	2				0								High	High	High	High	High	High	1	
FALSE	Medium	Master Prc	Q96MG2	Junctional	0.023	1.537	3	1	1	1	331	36.3	9.38	0	1	regulation	endoplasm		Pf15312	126306	ENSG0000	JSRP1	19				0								High	High	High	High	High	High	1	
FALSE	High	Master Prc	Q9NQZ2	Something	0.002	3.79	4	2	2	2	479	54.5	5.62	0	2	cell organi:	nucleus	protein bir	Pf04000, P	57050	ENSG0000	UTP3	4			Major path	5								High	High	High	High	High	High	1	
FALSE	High	Master Prc	Q9H8H2	Probable A	0	7.058	3	1	1	1	851	94	9.99	4.37	1	metabolic	cytoplasm;	catalytic a	Pf00270, P	64794	ENSG0000	DDX31	9				0								High	High	High	High	High	High	1	
FALSE	High	Master Prc	Q9Y2V7-1	Conserved	0	4.805	4	3	3	3	657	73.2	5.76	0	3	metabolic	Golgi;mem	protein bir	Pf06419	57511	ENSG0000	COG6	13			COPI-medi	11								High	High	High	High	High	High	1	

FALSE	Medium	Master Prc	Q9NYJ8-1	TGF-beta-a	0.011	2	2	1	1	1	693	76.4	8.54	0	1	metabolic	cytoplasm;	metal ion l	Pf00641, P	23118	ENSG0000	TAB2	6	Epstein-Ba	Regulation	IRAK2 mec	67								High	High	High	High	High	High	1	
FALSE	High	Master Prc	P40189-1	Interleukin	0.002	4.115	1	1	1	1	918	103.5	5.95	1.93	1	metabolic	extracellul	protein bir	Pf00041, P	3572	ENSG0000	IL6ST	5	Signaling p	Physiologic	IL-6-type c	26								High	High	High	High	High	High	1	
FALSE	Low	Master Prc	Q8TCT1	Phosphoet	0.063	1.094	3	1	1	1	267	29.7	7.71	0	1	metabolic	cytosol	catalytic ac	Pf00702, P	162466	ENSG0000	PHOSPHO1	17	Glycerophc		Synthesis c	8								High	High	High	High	High	High	1	[Q233]
FALSE	High	Master Prc	Q96NU1	Sterile alph	0	5.207	2	1	2	1	681	72.7	7.59	5.01	1		nucleus	protein bir	Pf07647	148398	ENSG0000	SAMD11	1				0								High	High	High	High	High	High	1	
FALSE	High	Master Prc	P62487	DNA-direct	0.002	3.528	5	1	2	1	172	19.3	5.54	1.88	1	cell death;	nucleus	catalytic ac	Pf00575, P	5436	ENSG0000	POLR2G	11	Pyrimidine	Eukaryotic	Signaling b	75								High	High	High	High	High	High	1	
FALSE	High	Master Prc	Q15643	Thyroid rec	0	6.469	1	1	1	1	1979	227.4	5.26	3.51	1	cell differe	cytoplasm;	protein bir	Pf04111, P	9321	ENSG0000	TRIP11	14			Intra-Golgi	7								High	High	High	High	High	High	1	
FALSE	High	Master Prc	Q9UNK0	Syntaxin-8	0	5.016	6	1	1	1	236	26.9	4.98	2.9	1	cell organi:	cytosol;enc	protein bir	Pf05739	9482	ENSG0000	STX8	17	SNARE inte			1								High	High	High	High	High	High	1	
FALSE	High	Master Prc	Q9UL15-1	BAG family	0.002	3.927	3	1	1	1	447	51.2	6.05	4.48	1	cell death;	cytosol;me	enzyme re	Pf02179	9529	ENSG0000	BAG5	14			Regulation	4								High	High	High	High	High	High	1	
FALSE	High	Master Prc	P49427	ubiquitin-c	0.003	3.061	5	1	1	1	236	26.7	4.54	0	1	cell divisio	cytoplasm;	catalytic ac	Pf00179, P	997	ENSG0000	CDC34	19	Ubiquitin r		Synthesis c	17								High	High	High	High	High	High	1	
FALSE	High	Master Prc	Q9NPA0	ER membr	0	6.56	10	1	1	1	242	26.5	9.25	3.25	1		membrane		Pf09430	56851	ENSG0000	EMC7	15				0								High	High	High	High	High	High	1	
FALSE	High	Master Prc	P31321	cAMP-depe	0.002	3.5	5	2	2	1	381	43	5.71	2.34	2	coagulatio	cytosol;me	enzyme re	Pf00027, P	5575	ENSG0000	PRKAR1B	7	Insulin sigr	miRs in Mu	Signaling b	32								High	High	High	High	High	High	1	
FALSE	High	Master Prc	Q8IX01-1	SURP and (	0	7.612	3	3	3	3	1082	120.1	7.28	1.64	3	metabolic	nucleus	RNA bindir	Pf01585, P	10147	ENSG0000	SUGP2	19		mRNA Pro		1								High	High	High	High	High	High	1	
FALSE	Medium	Master Prc	Q86Y39-1	NADH deh	0.012	1.958	8	1	1	1	141	14.8	8.72	0	1	cell organi:	membrane	catalytic ac		126328	ENSG0000	NDUFA11	19	Alzheimer'	Oxidative p	Complex I	12								High	High	High	High	High	High	1	
FALSE	High	Master Prc	Q8NBN7	Retinol del	0.003	2.87	9	2	2	2	331	35.9	8.1	1.88	2	metabolic	membrane	catalytic ac	Pf00106, P	112724	ENSG0000	RDH13	19; CHR_H			RA biosynt	4								High	High	High	High	High	High	1	
FALSE	Medium	Master Prc	P98172	ephrin-B1	0.016	1.703	5	1	1	1	346	38	8.94	0	1	cell commu	cytoplasm;	protein bir	Pf00812	1947	ENSG0000	EFNB1	X	Axon guida		EPH-ephrir	7								High	High	High	High	High	High	1	
FALSE	High	Master Prc	Q9H4I3-1	traB doma	0	6.255	6	2	2	2	376	42.3	8	4.05	2				Pf01963	80305	ENSG0000	TRABD	22				0								High	High	High	High	High	High	1	
FALSE	High	Master Prc	Q14139	ubiquitin c	0.001	4.515	2	2	2	2	1066	122.5	5.24	2.66	2	metabolic	cytoplasm;	catalytic ac	Pf04564, P	9354	ENSG0000	UBE4A	11	Ubiquitin r		Antigen pr	5								High	High	High	High	High	High	1	
FALSE	High	Master Prc	Q96PU8	protein qu	0	7.732	9	2	2	2	341	37.6	8.56	7	2	cell differe	cytoplasm;	protein bir	Pf00013, P	9444	ENSG0000	QKI	6		Exercise-in	Signaling b	5								High	High	High	High	High	High	1	
FALSE	Medium	Master Prc	Q8NCX0	Coiled-coil	0.019	1.621	2	1	1	1	1101	128.7	7.02	0	1	regulation		catalytic ac	Pf01576, P	284992	ENSG0000	CCDC150	2				0								High	High	High	High	High	High	1	[R1033]
FALSE	High	Master Prc	Q8TF01	Arginine/se	0.003	3.055	1	1	1	1	805	92.5	10.02	2.25	1		cytosol;nu	RNA bindir		25957	ENSG0000	PNISR	6				0								High	High	High	High	High	High	1	
FALSE	High	Master Prc	Q9H479	fructosami	0	7.945	6	2	2	2	309	35.1	7.55	3.59	2	cell differe	cytosol	catalytic ac	Pf01636, P	64122	ENSG0000	FN3K	17			Gamma ca	3								High	High	High	High	High	High	1	
FALSE	High	Master Prc	Q92520	Protein FA	0	8.427	9	2	2	2	227	24.7	8.29	5.29	2	cell differe	cytoplasm;	protein bir		10447; 51	ENSG0000	FAM3C; W	7		Wnt Signa	Platelet de	7								High	High	High	High	High	High	1	
FALSE	High	Master Prc	Q9UHY7	Enolase-ph	0.003	2.857	5	2	2	2	261	28.9	4.78	1.68	2	metabolic	cytoplasm;	catalytic ac	Pf13419	58478	ENSG0000	ENOPH1	4	Cysteine ar		Methionin	7								High	High	High	High	High	High	1	
FALSE	High	Master Prc	Q2TAL8	Glutamine	0	10.798	3	2	2	2	776	86.4	5.87	2.88	2	cell organi:	cytoplasm	DNA bindir	Pf00619, P	54870	ENSG0000	QRICH1	3				0								High	High	High	High	High	High	1	
FALSE	High	Master Prc	Q9Y4F1	FERM, Rho	0	6.548	1	1	2	1	1045	118.6	8.15	3.83	1	cell organi:	cytoplasm;	protein bir	Pf00169, P	10160	ENSG0000	FARP1	13				0								High	High	High	High	High	High	1	
FALSE	High	Master Prc	P35573-1	glycogen d	0	10.118	2	2	2	2	1532	174.7	6.76	8.69	2	metabolic	cytoplasm;	catalytic ac	Pf06202, P	178	ENSG0000	AGL	1	Starch and	Glycogen M	Neutrophil	10								High	High	High	High	High	High	1	
FALSE	Medium	Master Prc	Q4G0X9	Coiled-coil	0.048	1.222	1	1	1	1	1142	130	5.29	0	1	cell organi:	cytoplasm		Pf08647	55036	ENSG0000	CCDC40	17				0								High	High	High	High	High	High	1	
FALSE	Medium	Master Prc	Q9H267	Vacuolar p	0.047	1.228	3	1	1	1	617	70.5	6.71	2.15	1	cell organi:	cytoplasm;	protein bir	Pf00995	26276	ENSG0000	VPS33B	15				0								High	High	High	High	High	High	1	[K258];
FALSE	High	Master Prc	Q8TCD1	UPF0729 p	0.001	4.208	13	1	1	1	76	8.7	9.13	2.09	1	regulation		signal tran	Pf14975	497661	ENSG0000	C18orf32	18				0								High	High	High	High	High	High	1	
FALSE	High	Master Prc	Q96T58	Msx2-inter	0.002	4.037	0	2	2	1	3664	402	7.64	2.48	2	metabolic	nucleus	DNA bindir	Pf00076, P	23013	ENSG0000	SPEN	1		Notch Sign		1								High	High	High	High	High	High	1	
FALSE	Medium	Master Prc	Q8NEF9	serum resp	0.012	1.959	2	1	1	1	429	48.6	9.58	0	1	metabolic	cytoplasm;	RNA bindir	Pf09073	153443	ENSG0000	SRFBP1	5				0								High	High	High	High	High	High	1	
FALSE	High	Master Prc	Q9H330	Transmem	0.009	2.181	1	1	1	1	911	100.9	8.87	0	1		membrane		Pf06679	23731		TMEM245	9				0								High	High	High	High	High	High	1	
FALSE	Medium	Master Prc	Q96C01	Protein FA	0.024	1.521	5	1	1	1	138	15.6	7.61	0	1		cytoplasm		Pf05811	84908	ENSG0000	FAM136A	2				0								High	High	High	High	High	High	1	
FALSE	High	Master Prc	Q9H5Q4	Dimethylac	0	9.66	9	3	3	3	396	45.3	9.19	9.95	3	cell organi:	mitochond	catalytic ac	Pf00398	64216	ENSG0000	TFB2M	1		Energy Me	Mitochonc	8								High	High	High	High	High	High	1	
FALSE	Low	Master Prc	Q7L1V2	Vacuolar fu	0.068	1.063	1	1	1	1	547	59.2	6.29	0	1	metabolic	cytoplasm	protein bir	Pf03164	22879	ENSG0000	MON1B	16			RAB GEFs e	4								High	High	High	High	High	High	1	
FALSE	High	Master Prc	Q99871-1	HAUS augr	0.003	3.324	4	1	1	1	368	40.8	4.83	2.79	1	cell divisio	cytoplasm;	protein bir		55559	ENSG0000	HAUS7	X			Regulation	16								High	High	High	High	High	High	1	
FALSE	Low	Master Prc	Q9Y2G8	DnaJ homc	0.067	1.072	2	1	1	1	782	90.5	7.12	0	1	cellular ho	cytosol;me		Pf00085, P	23341	ENSG0000	DNAJC16	1				0								High	High	High	High	High	High	1	[K741];
FALSE	High	Master Prc	Q12824-1	SWI/SNF-re	0.003	3.461	3	1	3	1	385	44.1	6.23	7.79	1	cell differe	chromosor	DNA bindir	Pf04855	6598	ENSG0000	SMARCB1	22; CHR_H			RUNX1 int	8								High	High	High	High	High	High	1	
FALSE	Medium	Master Prc	Q8NFV4	Protein Ab	0.022	1.588	3	1	1	1	315	34.7	9.48	0	1		mitochond	catalytic ac	Pf00561, P	83451	ENSG0000	ABHD11	7				0								High	High	High	High	High	High	1	
FALSE	High	Master Prc	O00358	Forkhead b	0.006	2.341	4	1	1	1	373	38.1	9.58	0	1	cell differe	nucleus	DNA bindir	Pf00250	2304	ENSG0000	FOXE1	9				0								High	High	High	High	High	High	1	
FALSE	High	Master Prc	Q2T9J0	Peroxisom	0	6.653	4	1	1	1	566	59.3	6.2	3.9	1	cell organi:	membrane	catalytic ac	Pf13365	219743	ENSG0000	TYSND1	10				0								High	High	High	High	High	High	1	
FALSE	High	Master Prc	Q8NI60-1	Atypical ki	0.006	2.457	2	1	1	1	647	71.9	6.99	0	1	metabolic	membrane	catalytic ac	Pf03109	56997	ENSG0000	ADCK3; CC	1				0								High	High	High	High	High	High	1	
FALSE	High	Master Prc	O00399	Dynactin s	0.002	3.643	9	1	1	1	190	20.7	6.32	2.28	1	transport	chromosor			10671	ENSG0000	DCTN6	8	Vasopressi		COPI-indep	18								High	High	High	High	High	High	1	
FALSE	High	Master Prc	O75592-1	E3 ubiquiti	0	7.598	0	1	1	1	4640	509.8	7.03	1.8	1	metabolic	membrane	catalytic ac	Pf00415, P	23077	ENSG0000	MYCBP2	13		Integrated		1								High	High	High	High	High	High	1	
FALSE	High	Master Prc	Q53F19	Nuclear ca	0.006	2.528	2	1	1	1	620	70.5	5.73	0	1	defense re	cytoplasm;	catalytic ac	Pf08675, P	55421	ENSG0000	C17orf85;	17				0								High	High	High	High	High	High	1	
FALSE	Medium	Master Prc	P40337	Von Hippel	0.015	1.793	3	1	1	1	213	24.1	4.73	2.35	1	cell organi:	cytoplasm;	catalytic ac	Pf01847	7428	ENSG0000	VHL	3	Renal cell c	Amplificati	Oxygen-de	17								High	High	High	High	High	High	1	
FALSE	Medium	Master Prc	Q8ND56	protein LSI	0.028	1.459	2	1	1	1	463	50.5	9.52	0	1	cell organi:	cytoplasm;	DNA bindir	Pf09532, P	26065	ENSG0000	LSM14A	19; CHR_H				0								High	High	High	High	High	High	1	
FALSE	Medium	Master Prc	Q13426-1	DNA repair	0.032	1.406	2	1	1	1	336	38.3	4.98	0	1	metabolic	chromosor	catalytic ac	Pf06632	7518	ENSG0000	XRCC4	5	Non-homo	Non-homo	2-LTR circle	17								High	High	High	High	High	High	1	
FALSE	High	Master Prc	Q9NQ29	putative RI	0	24.361	11	4	13	1	371	43.7	9.92	34.32	4	cell organi:	nucleus;sp	protein bir	Pf03194	55692	ENSG0000	LUC7L	16				0								High	High	High	High	High	High	1	
FALSE	Medium	Master Prc	Q8WVS4	WD repeat	0.022	1.568	1	1	1	1	1066	122.5	7.31	1.82	1	cell organi:		catalytic ac		55112	ENSG0000	WDR60	7			Intraflagell	3								High	High	High	High	High	High	1	
FALSE	High	Master Prc	Q8NI35	InaD-like p	0.009	2.109	0	1	1	1	1801	196.2	4.94	0	1	cell organi:	cytoplasm;	protein bir	Pf00595, P	10207	ENSG0000	INADL; PA	1	Tight junct		Tight junct	6								High	High	High	High	High	High	1	
FALSE	High	Master Prc	Q5BJF2	sigma intra	0.005	2.606	6	1	1	1	176	20.8	9.38	2.45	1	regulation	cytosol;enc	protein bir	Pf10914	27346	ENSG0000	TMEM97	17				0								High	High	High	High	High	High	1	
FALSE	High	Master Prc	Q68D10-1	Protein SP	0.006	2.431	2	1	1	1	685	75.6	9.79	0	1	cell organi:	nucleus	DNA bindir	Pf08243	144108	ENSG0000	SPTY2D1	11				0								High	High	High	High	High	High	1	
FALSE	High	Master Prc	O60216	Double-str	0.006	2.33	3	2	2	2	631	71.6	4.65	1.76	2	cell death;	chromosor	protein bir	Pf04824, P	5885	ENSG0000	RAD21	8	Cell cycle		Meiotic sy	21								High	High	High	High	High	High	1	
FALSE	Medium	Master Prc	P45973	chromobox	0.019	1.626	4	1	1	1	191	22.2	5.86	0	1	coagulatio	chromosor	protein bir	Pf00385, P	23468	ENSG0000	CBX5	12			SUMOylati	11								High	High	High	High	High	High	1	
FALSE	High	Master Prc	Q9BVJ7	Dual specif	0.01	2.086	5	1	1	1	150	16.6	8.21	0	1	metabolic	cytoplasm;	catalytic ac	Pf00102, P	54935	ENSG0000	DUSP23	1				0								High	High	High	High	High	High	1	
FALSE	High	Master Prc	P42696	RNA-bindir	0	20.159	17	7	10	7	430	48.5	10.11	10.35	7		nucleus	RNA bindir	Pf00076, P	23029	ENSG0000	RBM34	1				0								High	High	High	High	High	High	1	
FALSE	High	Master Prc	P55854	Small ubiq	0	18.461	26	3	8	1	103	11.6	5.49	16.11	3	metabolic	chromosor	protein bir	Pf00240, P	6612	ENSG0000	SUMO3	21	RNA transp	Exercise-in	SUMOylati	18								High	High	High	High	High	High	1	
FALSE	Medium	Master Prc	Q8N4Q1	Mitochonc	0.049	1.207	6	1	1	1	142	16	4.31	0	1	metabolic	mitochond	catalytic ac	Pf06747	131474	ENSG0000	CHCHD4	3			Mitochonc	2								High	High	High	High	High	High	1	
FALSE	High	Master Prc	Q9BXX3	Ankyrin rep	0.009	2.1	1	1	1	1	1397	158.7	6.48	0	1	regulation	nucleus	DNA bindir	Pf00023, P	91074	ENSG0000	ANKRD30A	10				0								High	High	High	High	High	High	1	
FALSE	Medium	Master Prc	Q9H7D7	WD repeat	0.02	1.603	3	1	1	1	661	72.1	6.16	0	1		cytoplasm;	protein bir	Pf00400, P	80232	ENSG0000	WDR26	1				0								High	High	High	High	High	High	1	
FALSE	High	Master Prc	Q5QNW6-	Histone H2	0	116.397	75	15	106	0	126	13.9	10.32	271.88	15	cell organi:	chromosor	DNA bindir	Pf00125, P	440689	ENSG0000	HIST2H2BF	1	Alcoholism		HDACs dea	11								Not Found	Not Found	Not Found	Not Found	Not Found	Not Found	1	
FALSE	High	Master Prc	Q93034	Cullin-5 [O	0.002	4.152	2	1	1	1	780	90.9	7.94	0	1	cell prolife	cytosol;nu	catalytic ac	Pf00888, P	8065	ENSG0000	CUL5	11	Ubiquitin r	Apoptosis-	Downregu	19								High	High	High	High	High	High	1	
FALSE	Medium	Master Prc	Q8N5G0	Small integ	0.016	1.694	12	1	1	1	67	7.7	9.86	0	1	cell organi:	membrane	protein bir	Pf15061	389203	ENSG0000	C4orf52; S	4				0								High	High	High	High	High	High	1	
FALSE	High	Master Prc	O14777	Kinetochor	0.002	3.663	3	1	1	1	642	73.9	5.6	2.82	1	cell divisio	chromosor	protein bir	Pf01496, P	10403	ENSG0000	NDC80	18			Amplificati	16								High	High	High	High	High	High	1	
FALSE	High	Master Prc	P41236	Protein ph	0.003	2.892	4	1	2	1	205	23	4.74	3.29	1	metabolic	cytosol	enzyme re	Pf04979	5504	ENSG0000	PPP1R2	3				0								High	High	High	High	High	High	1	
FALSE	High	Master Prc	P02533	Keratin, ty	0	50.795	13	7	29	1	472	51.5	5.16	72.13	7	cell death;	cytoplasm;	protein bir	Pf00038, P	3861	ENSG0000	KRT14	17		Corticotro	Formation	7								High	High	High	High	High	High	1	
FALSE	High	Master Prc	P49914	5-formylte	0.003	3.244	4	1	2	1	203	23.2	7.88	0	1	metabolic	cytoplasm;	catalytic ac	Pf01812	10588	ENSG0000	MTHFS	15	One carbo	Folate Met	Metabolisr	8								High	High	High	High	High	High	1	
FALSE	High	Master Prc	P41214	Eukaryotic	0.001	4.194	5	2	2	2	584	64.7	7.65	0	2	cell organi:	cytoplasm;	protein bir	Pf01253, P	1939	ENSG0000	EIF2D	1				0								High	High	High	High	High	High	1	
FALSE	Medium	Master Prc	O75817	Ribonuclea	0.016	1.723	7	1	1	1	140	15.6	8.94	2.07	1	metabolic	cytoplasm;	catalytic ac	Pf01918, P	10248	ENSG0000	POP7	7	Ribosome		tRNA proc	5								High	High	High	High	High	High	1	
FALSE	High	Master Prc	Q99808	Equilibrati	0.003	3.204	2	1	1	1	456	50.2	8.29	2.58	1	cellular ho	membrane	transporte	Pf01733	2030	ENSG0000	SLC29A1	6	Alcoholism	Fluoropyri	Transport c	6								High	High	High	High	High	High	1	
FALSE	Medium	Master Prc	Q96AA3	Protein RF	0.019	1.63	2	1	1	1	541	60.3	8.85	0	1	cell organi:	membrane	transporte	Pf04506	91869	ENSG0000	RFT1	3			Defective I	8								High	High	High	High	High	High	1	
FALSE	High	Master Prc	Q5T8P6	RNA-bindir	0	8.762	4	3	3	3	1007	113.5	9.16	7.84	3	metabolic		metal ion l	Pf00076, P	64062	ENSG0000	RBM26	13				0								High	High	High	High	High	High	1	
FALSE	High	Master Prc	Q14674-1	Separin [O	0.003	2.981	1	1	1	1	2120	233	7.55	1.69	1	cell death;	cytoplasm;	catalytic ac	Pf03568	9700	ENSG0000	ESPL1	12	Cell cycle;	Cell Cycle	Separation	9								High	High	High	High	High	High	1	
FALSE	Low	Master Prc	O94830	Phospholip	0.069	1.052	2	1	1	1	711	81	5.39	2.25	1	cell organi:	cytoplasm;	catalytic ac	Pf00536, P	23259	ENSG0000	DDHD2	8			Synthesis c	5								High	High	High	High	High	High	1	
FALSE	Medium	Master Prc	Q9Y2R4	Probable A	0.023	1.549	1	1	1	1	599	67.5	9.67	0	1	metabolic	cytoplasm;	catalytic ac	Pf00176, P	11056	ENSG0000	DDX52	17; CHR_H			Major path	5								High	High	High	High	High	High	1	
FALSE	Low	Master Prc	Q504Q3	PAN2-PAN	0.059	1.125	1	1	1	1	1202	135.3	5.99	0	1	metabolic	cytoplasm;	catalytic ac	Pf00443, P	9924	ENSG0000	PAN2	12	RNA degra	Ectoderm I	Deadenyla	5								High	High	High	High	High	High	1	
FALSE	Low	Master Prc	Q6KC79	Nipped-B-l	0.051	1.191	0	1	2	1	2804	315.9	7.91	3.49	1	cell differe	chromosor	DNA bindir	Pf03154, P	25836	ENSG0000	NIPBL	5			Cohesin Lo	5								High	High	High	High	High	High	1	[K2347]
FALSE	Medium	Master Prc	P35716	Transcripti	0.032	1.409	2	1	1	1	441	46.7	5.05	0	1	cell differe	cytoplasm;	DNA bindir	Pf00505, P	6664	ENSG0000	SOX11	2		Preimplant		1								High	High	High	High	High	High	1	

FALSE	Medium	Master Prc	Q5C9Z4	nucleolar M	0.01	2.074	2	1	1	1	860	96.2	8.1	0	1	cell differe	nucleus	protein bir	Pf02847, P	64434	ENSG0000	NOM1	7				0								High	High	High	High	High	High	1	
FALSE	Medium	Master Prc	O43292-1	Glycosylph	0.014	1.847	1	1	1	1	621	67.6	8.06	0	1	cell organi	endoplasm	catalytic a	Pf04114	8733	ENSG0000	GPAA1	8	Metabolic		Attachmer	6								High	High	High	High	High	High	1	
FALSE	Medium	Master Prc	Q3YEC7-1	Rab-like pr	0.013	1.859	2	1	1	1	729	79.5	5.22	2.17	1	regulation	cytoplasm;	catalytic a	Pf00025, P	55684	ENSG0000	RABL6	9				0								High	High	High	High	High	High	1	
FALSE	Medium	Master Prc	Q6PF18-1	MORN rep	0.034	1.371	8	1	1	1	240	27.6	8.44	0	1		nucleus	protein bir		283385	ENSG0000	MORN3	12				0								High	High	High	High	High	High	1	[K86]
FALSE	High	Master Prc	Q969G6	Riboflavin	0.003	3.055	6	1	1	1	155	17.6	8.13	2.69	1	cell death;	cytoplasm;	catalytic a	Pf01687	55312	ENSG0000	RFK	9	Metabolic	Selenium M	Vitamin B2	9								High	High	High	High	High	High	1	
FALSE	High	Master Prc	O14841	5-oxoproli	0	5.202	1	2	2	2	1288	137.4	6.58	1.62	2	metabolic	cytosol	catalytic a	Pf01968, P	26873	ENSG0000	OPLAH	8	Glutathion	Glutathion	Defective (	11								High	High	High	High	High	High	1	
FALSE	High	Master Prc	Q9P265	Disco-inter	0	5.207	1	2	2	2	1576	171.4	8.09	2.1	2	metabolic	cytoplasm;	catalytic a	Pf00501, P	57609	ENSG0000	DIP2B	12				0								High	High	High	High	High	High	1	
FALSE	Medium	Master Prc	P53350	Serine/thre	0.011	2.036	2	1	1	1	603	68.2	8.91	0	1	cell divisio	chromosor	catalytic a	Pf00069, P	5347	ENSG0000	PLK1	16	FoxO signa	Regulation	Regulation	53								High	High	High	High	High	High	1	
FALSE	High	Master Prc	Q07954	prolow-der	0.001	4.444	1	2	2	2	4544	504.3	5.39	1.78	2	cell prolife	cytoplasm;	metal ion l	Pf00057, P	4035	ENSG0000	LRP1	12	Alzheimer'	Alzheimers	Signaling b	15								High	High	High	High	High	High	1	
FALSE	High	Master Prc	P36268	Inactive glu	0	7.184	4	2	2	2	569	61.7	7.58	1.64	2	metabolic	cytoplasm;	catalytic a	Pf01019	728441		GGT2	22				0								High	High	High	High	High	High	1	
FALSE	High	Master Prc	P35520-1	Cystathion	0.006	2.496	2	1	1	1	551	60.5	6.65	0	1	cell commu	cytoplasm;	catalytic a	Pf00291, P	875; 1027:	ENSG0000	CBS; LOC10	21	Glycine, se	Selenium M	Metabolisr	18								High	High	High	High	High	High	1	
FALSE	Medium	Master Prc	Q15041	ADP-ribosy	0.038	1.308	3	1	1	1	203	23.3	9.32	0	1	cell death;	cytoplasm;	protein bir	Pf02453	23204	ENSG0000	ARL6IP1	16				0								High	High	High	High	High	High	1	
FALSE	Medium	Master Prc	Q9NYH9	U3 small n	0.025	1.494	1	1	1	1	597	70.1	7.28	0	1	metabolic	nucleus	protein bir	Pf08640	55813	ENSG0000	UTP6	17	Ribosome		Major path	6								High	High	High	High	High	High	1	
FALSE	High	Master Prc	Q9P215	Pogo trans	0.005	2.572	1	1	1	1	609	69.4	5.14	2.34	1	developme	nucleus	DNA bindi	Pf01352, P	57645	ENSG0000	POGK	1				0								High	High	High	High	High	High	1	
FALSE	Low	Master Prc	Q8N2H4	protein SYS	0.06	1.119	6	1	1	1	156	17.6	7.72	0	1	cell organi	cytosol;Go		Pf09801	90196	ENSG0000	SYS1	20			Retrograde	4								High	High	High	High	High	High	1	
FALSE	Medium	Master Prc	P30281-1	G1/S-speci	0.036	1.344	8	1	1	1	292	32.5	7.06	0	1	cell divisio	cytoplasm;	catalytic a	Pf00134, P	896	ENSG0000	CCND3	6	Focal adhe	miRNA Reg	Transcripti	37								High	High	High	High	High	High	1	[Q276]
FALSE	High	Master Prc	Q7Z3T8	Zinc finger	0.006	2.48	1	1	1	1	1539	168.8	4.82	1.94	1	cell organi	cytoplasm;	metal ion l	Pf01363, P	9765	ENSG0000	ZFYVE16	5	Endocytosi	TGF-beta S	Signaling b	6								High	High	High	High	High	High	1	
FALSE	Medium	Master Prc	A6NHR9-1	structural	0.038	1.304	0	1	1	1	2005	226.2	7.3	3.38	1	cell organi	chromosor	catalytic a	Pf06470, P	23347	ENSG0000	SMCHD1	18				0								High	High	High	High	High	High	1	
FALSE	High	Master Prc	Q9H9Q2	COP9 signa	0.002	4.133	10	1	1	1	264	29.6	6.15	0	1	cell organi	cytoplasm;	protein bir	Pf01399	64708	ENSG0000	COPS7B	2			Cargo reco	13								High	High	High	High	High	High	1	
FALSE	High	Master Prc	Q9H0P0-1	Isoform 1	0.001	4.471	7	2	2	2	297	33.9	5.86	2.44	2	defense re	cytoplasm;	catalytic a	Pf05822, P	51251	ENSG0000	NT5C3; NT	7				0								High	High	High	High	High	High	1	
FALSE	Medium	Master Prc	Q9UGR2	Zinc finger	0.014	1.82	1	1	1	1	993	111.5	7.17	0	1	metabolic	nucleus	metal ion l	Pf00515, P	23264		ZC3H7B	22				0								High	High	High	High	High	High	1	
FALSE	High	Master Prc	O96007	molybdopt	0.001	4.333	5	1	1	1	188	20.9	5.44	0	1	metabolic	cytoplasm;	catalytic a	Pf02391	4338	ENSG0000	MOCS2	5	Sulfur rela		Molybdenu	7								High	High	High	High	High	High	1	
FALSE	Low	Master Prc	P41440	Folate trar	0.074	1.016	1	1	1	1	591	64.8	8.95	0	1	metabolic	membrane	transporte	Pf01770, P	6573	ENSG0000	SLC19A1	21	Antifolate	Folate Met	Metabolisr	7								High	High	High	High	High	High	1	
FALSE	High	Master Prc	O76031	ATP-depen	0	4.549	7	3	3	3	633	69.2	7.58	0	3	metabolic	cytosol;me	catalytic a	Pf00004, P	10845	ENSG0000	CLPX	15				0								High	High	High	High	High	High	1	
FALSE	High	Master Prc	P46100	transcripti	0	10.528	1	2	2	2	2492	282.4	6.58	4.17	2	cell organi	chromosor	catalytic a	Pf00176, P	546		ATRX	X		Pathways /		1								High	High	High	High	High	High	1	
FALSE	Medium	Master Prc	Q9BW19	Kinesin-like	0.023	1.539	1	1	1	1	673	73.7	8.98	0	1	cell divisio	cytoplasm;	catalytic a	Pf00225	3833	ENSG0000	KIFC1	6; CHR_HS			COPI-depe	8								High	High	High	High	High	High	1	
FALSE	High	Master Prc	Q92541	RNA polym	0.002	3.511	2	1	1	1	710	80.3	8.15	1.75	1	cell organi	nucleus	DNA bindi	Pf03126	23168	ENSG0000	RTF1	15		Endoderm	E3 ubiquiti	10								High	High	High	High	High	High	1	
FALSE	High	Master Prc	O43772	mitochond	0	11.417	15	3	3	3	301	32.9	9.41	6.09	3	transport	cytosol;me	transporte	Pf00153	788	ENSG0000	SLC25A20	3		Fatty Acid	Import of p	6								High	High	High	High	High	High	1	
FALSE	High	Master Prc	O43156	TELO2-inte	0	6.623	2	1	1	1	1089	122	5.97	2.01	1	regulation	cytoplasm	protein bir		9675	ENSG0000	TTI1	20	mTOR sign			1								High	High	High	High	High	High	1	
FALSE	High	Master Prc	Q63HN8-4	Isoform 2	0	12.236	0	1	2	1	5256	596.1	6.43	10	1	cell organi	cytoplasm;	catalytic a	Pf00097, P	57674		RNF213	17				0								High	High	High	High	High	High	1	
FALSE	High	Master Prc	Q96PU5	E3 ubiquiti	0	5.234	2	2	2	1	975	111.9	5.82	0	2	cell differe	cytoplasm;	catalytic a	Pf00168, P	23327	ENSG0000	NEDD4L	18	Ubiquitin r	TGF-beta S	Budding ar	27								High	High	High	High	High	High	1	
FALSE	High	Master Prc	Q13112	Chromatin	0.003	3.357	2	1	1	1	559	61.5	7.5	2.9	1	cell organi	cytoplasm;	protein bir	Pf00400, P	8208	ENSG0000	CHAF1B	21				0								High	High	High	High	High	High	1	
FALSE	High	Master Prc	Q9UN37	Vacuolar p	0	8.148	8	3	3	3	437	48.9	7.8	3.04	3	cell commu	cytoplasm;	catalytic a	Pf00004, P	27183	ENSG0000	VPS4A	16	Endocytosi		Budding ar	10								High	High	High	High	High	High	1	
FALSE	High	Master Prc	Q9UQN3-1	Charged m	0	4.567	8	2	3	1	213	23.9	8.76	3.43	2	cell commu	cytoplasm;	protein bir	Pf03357	25978	ENSG0000	CHMP2B	3	Endocytosi		Budding ar	12								High	High	High	High	High	High	1	
FALSE	High	Master Prc	P60891	ribose-pho	0	55.411	35	9	15	3	318	34.8	6.98	43.44	9	metabolic	cytosol	catalytic a	Pf00156, P	5631	ENSG0000	PRPS1	X	Biosynthes	Nucleotide	5-Phospho	10								High	High	High	High	High	High	1	
FALSE	High	Master Prc	O15061-1	Synemin [(	0	6.66	1	2	3	2	1565	172.7	5.16	1.78	2	cell organi	cytoplasm;	protein bir	Pf00038	23336		SYNM	15				0								High	High	High	High	High	High	1	
FALSE	High	Master Prc	Q14157-5	Isoform 5	0	120.387	25	18	33	1	1104	116.6	7.02	88.63	18	regulation	cytoplasm	protein bir	Pf00627, P	9898		UBAP2L	1				0								High	High	High	High	High	High	1	
FALSE	High	Master Prc	P50851-1	Lipopolysa	0	12.549	2	3	3	3	2863	318.9	5.6	8.59	3		endoplasm	protein bir	Pf02138, P	987	ENSG0000	LRBA	4				0								High	High	High	High	High	High	1	
FALSE	Medium	Master Prc	Q9HC36	rRNA meth	0.015	1.767	2	1	1	1	420	47	8.73	0	1	metabolic	mitochond	catalytic a	Pf00588, P	55178	ENSG0000	RNMTL1; N	17			rRNA mod	4								High	High	High	High	High	High	1	
FALSE	High	Master Prc	Q15392	Delta(24)-s	0	6.503	4	3	5	3	516	60.1	8.16	0	3	cell death;	cytoplasm;	catalytic a	Pf01565	1718	ENSG0000	DHCR24	1	Steroid bic	Tryptopha	Cholestero	9								High	High	High	High	High	High	1	
FALSE	High	Master Prc	P26358-1	DNA (cytos	0	4.668	1	1	1	1	1616	183.1	7.75	2.93	1	cell organi	nucleus	catalytic a	Pf00145, P	1786	ENSG0000	DNMT1	19	MicroRNA:	Trans-sulfu	PRC2 meth	14								High	High	High	High	High	High	1	
FALSE	High	Master Prc	Q9Y281-1	Cofilin-2 [(	0	49.807	48	8	43	3	166	18.7	7.88	85.7	8	cell organi	cytoplasm;	protein bir	Pf00241	1073	ENSG0000	CFL2	14	Axon guida	Leptin sign		8								High	High	High	High	High	High	1	
FALSE	High	Master Prc	Q9H974-1	Queuine tR	0	4.672	5	2	2	2	415	46.7	6.81	4.16	2	metabolic	cytoplasm;	catalytic a	Pf01702	79691	ENSG0000	QTRTD1; C	3			tRNA mod	3								High	High	High	High	High	High	1	
FALSE	Medium	Master Prc	Q8NFC6	Biorientati	0.034	1.375	0	1	1	1	3051	330.3	5.08	0	1	metabolic	chromosor		Pf05205	259282	ENSG0000	BOD1L1	4				0								High	High	High	High	High	High	1	
FALSE	Medium	Master Prc	Q13438-1	Protein os-	0.015	1.788	2	1	1	1	667	75.5	4.87	2.71	1	metabolic	endoplasm	protein bir	Pf07915	10956	ENSG0000	OS9	12	Protein prc		Hh mutant	19								High	High	High	High	High	High	1	
FALSE	Medium	Master Prc	Q9H4L7	SWI/SNF-re	0.011	2.009	2	1	1	1	1026	117.3	5.55	2.25	1	cell organi	chromosor	catalytic a	Pf00176, P	56916	ENSG0000	SMARCAD	4	Signaling p			1								High	High	High	High	High	High	1	
FALSE	Low	Master Prc	P14635	G2/mitotic	0.071	1.028	2	1	1	1	433	48.3	7.47	0	1	cell divisio	cytoplasm;	catalytic a	Pf00134, P	891	ENSG0000	CCNB1	5	Cell cycle;	AMP-activ	APC/C:Cdc	56								High	High	High	High	High	High	1	
FALSE	High	Master Prc	Q6GMV2	SET and M	0.009	2.133	3	1	1	1	418	47.3	5.05	2.74	1	metabolic		catalytic a	Pf00856	10322	ENSG0000	SMYD5	2		Histone M		1								High	High	High	High	High	High	1	
FALSE	Medium	Master Prc	Q5T8D3-1	Acyl-CoA-b	0.024	1.504	2	1	1	1	534	60.1	5.33	1.75	1	cell commu	membrane		Pf00887	91452	ENSG0000	ACBD5	10			Peroxisom	4								High	High	High	High	High	High	1	
FALSE	High	Master Prc	Q15629	Translocati	0.003	3.352	3	1	1	1	374	43	9.63	3.43	1	cell organi	endoplasm	protein bir	Pf03798, P	23471	ENSG0000	TRAM1	8	Protein prc	TLR4 Signa	SRP-depen	5								High	High	High	High	High	High	1	
FALSE	High	Master Prc	Q9BQS8	fyve and cc	0	5.769	1	1	1	1	1478	166.9	4.92	2.6	1	cellular coi	endosome	metal ion l	Pf01363, P	79443	ENSG0000	FYCO1	3				0								High	High	High	High	High	High	1	
FALSE	High	Master Prc	O75787	Renin rece	0.002	4.099	6	1	1	1	350	39	6.1	2.35	1	cellular coi	membrane	protein bir	Pf07850	10159	ENSG0000	ATP6AP2	X	Renin-angi	ACE Inhibit	Metabolisr	9								High	High	High	High	High	High	1	
FALSE	High	Master Prc	Q08117	amino-terr	0.003	3.057	6	1	2	1	197	22	6.37	1.98	1	cell commu	nucleus	protein bir	Pf03920	166	ENSG0000	AES	19		Androgen	Repression	6								High	High	High	High	High	High	1	
FALSE	High	Master Prc	Q13206	probable A	0	6.273	1	1	2	1	875	100.8	8.63	3.28	1	metabolic	cytoplasm	catalytic a	Pf00270, P	1662	ENSG0000	DDX10	11				0								High	High	High	High	High	High	1	
FALSE	Medium	Master Prc	O14926-1	Fascin-2 [C	0.032	1.408	2	1	1	1	492	55	7.84	0	1	cell organi	cytoplasm;	protein bir	Pf00167, P	25794	ENSG0000	FSCN2	17				0								High	High	High	High	High	High	1	
FALSE	High	Master Prc	P22681	E3 ubiquiti	0	5.584	3	2	2	2	906	99.6	6.54	3.77	2	cell death;	cytoplasm;	catalytic a	Pf00097, P	867	ENSG0000	CBL	11	Proteoglyc	RANKL/RA	Spry regula	72								High	High	High	High	High	High	1	
FALSE	High	Master Prc	Q5U5X0	Complex II	0	7.089	31	2	2	2	104	11.9	9.66	2.75	2	cell organi	membrane	protein bir	Pf05347, P	90624	ENSG0000	LYRM7	5				0								High	High	High	High	High	High	1	
FALSE	High	Master Prc	Q96SQ9	Cytochrom	0.003	3.057	3	1	1	1	504	55.8	8.62	0	1	metabolic	endoplasm	catalytic a	Pf00067	29785	ENSG0000	CYP2S1	19	Retinol me	Oxidation	CYP2E1 rea	12								High	High	High	High	High	High	1	
FALSE	High	Master Prc	Q9GZT8-1	NIF3-like p	0	11.734	12	4	4	4	377	41.9	6.65	6.02	4	cell differe	cytoplasm;	protein bir	Pf01784	60491	ENSG0000	NIF3L1	2				0								High	High	High	High	High	High	1	
FALSE	High	Master Prc	Q01433-1	AMP deam	0	9.973	4	3	3	3	879	100.6	6.93	3.99	3	metabolic	cytosol	catalytic a	Pf00962	271	ENSG0000	AMPD2	1	Purine met		Purine salv	6								High	High	High	High	High	High	1	
FALSE	Medium	Master Prc	Q13488	V-type pro	0.011	2.017	1	1	1	1	830	92.9	7.12	0	1	cell commu	membrane	catalytic a	Pf01496	10312	ENSG0000	TCIRG1	11	Metabolic	TYROBP Ca	Transferrir	23								High	High	High	High	High	High	1	
FALSE	Medium	Master Prc	Q9Y3A3	MOB-like p	0.017	1.666	4	1	1	1	225	26	5.78	1.72	1	transport	cytoplasm;	metal ion l	Pf03637	25843	ENSG0000	MOB4	2				0								High	High	High	High	High	High	1	
FALSE	High	Master Prc	Q9H900-1	Protein zw	0	6.054	3	2	2	2	591	67.2	6.27	4.64	2	cell divisio	chromosor	protein bir	Pf09817	55055	ENSG0000	ZWILCH	15			Amplificati	16								High	High	High	High	High	High	1	
FALSE	High	Master Prc	Q6Y288	Beta-1,3-g	0.004	2.693	3	1	1	1	498	56.5	7.5	2.62	1	metabolic	endoplasm	catalytic a	Pf02434	145173	ENSG0000	B3GALTL; I	13	Other type		O-glycosyla	9								High	High	High	High	High	High	1	
FALSE	High	Master Prc	Q9UBC2-1	Epidermal	0	10.743	5	3	3	3	864	94.2	5.11	5.43	3	cell differe	cytoplasm;	catalytic a	Pf00038, P	58513	ENSG0000	EPS15L1	19	Endocytosi	EGF/EGFR	Cargo reco	10								High	High	High	High	High	High	1	
FALSE	High	Master Prc	P41240	tyrosine-pr	0.003	3.084	3	1	1	1	450	50.7	7.06	2.01	1	cell differe	cytoplasm;	catalytic a	Pf00017, P	1445	ENSG0000	CSK	15	Epithelial c	Integrin-m	PD-1 signa	36								High	High	High	High	High	High	1	
FALSE	High	Master Prc	Q13509	tubulin bet	0	165.744	41	17	106	2	450	50.4	4.93	217.7	17	cell organi	cytoplasm;	catalytic a	Pf00091, P	10381	ENSG0000	TUBB3	16	Phagosom	Pathogeni	COPI-indep	66								High	High	High	High	High	High	1	[R156]
FALSE	Low	Master Prc	Q6NUM9-	all-trans-re	0.055	1.151	2	1	1	1	610	66.8	8.28	0	1	metabolic	endoplasm	catalytic a	Pf13450	54884	ENSG0000	RETSAT	2	Retinol me	Vitamin A	Signaling b	9								High	High	High	High	High	High	1	
FALSE	High	Master Prc	P36639	7,8-dihydro	0.003	2.842	6	1	1	1	197	22.5	5.27	0	1	cell commu	cytoplasm;	catalytic a	Pf00293	4521		NUDT1	7			Phosphate	5								High	High	High	High	High	High	1	
FALSE	High	Master Prc	Q96MH2	Protein HE	0	5.042	7	2	3	1	286	32.4	6.55	1.65	2	metabolic	cytoplasm;	enzyme re	Pf04111, P	124790	ENSG0000	HEXIM2	17				0								High	High	High	High	High	High	1	
FALSE	High	Master Prc	P62256	ubiquitin-c	0.003	2.843	5	1	1	1	183	20.6	4.67	0	1	metabolic	cytoplasm;	catalytic a	Pf00179	7328	ENSG0000	UBE2H	7	Ubiquitin r		Synthesis c	9								High	High	High	High	High	High	1	
FALSE	High	Master Prc	Q92747	Actin-relat	0	7.834	11	2	2	1	370	41.5	8.18	5.61	2	cell organi	cytoplasm;	protein bir	Pf00400	10552	ENSG0000	ARPC1A	7	Regulation	Pathogeni	EPHB-med	23								High	High	High	High	High	High	1	
FALSE	Low	Master Prc	Q6ZNG1	zinc finger	0.051	1.18	2	1	1	1	722	83.1	9.25	0	1	metabolic	nucleus	DNA bindi	Pf13465	162966	ENSG0000	ZNF600	19			Generic Tra	3								High	High	High	High	High	High	1	[Q344];
FALSE	Low	Master Prc	Q8WUD4	Coiled-coil	0.05	1.197	4	1	1	1	166	19.2	7.34	0	1	metabolic	cytoplasm	catalytic a	Pf08315	151903	ENSG0000	CCDC12	3	Spliceosom			1								High	High	High	High	High	High	1	
FALSE	High	Master Prc	Q6P087	Mitochond	0.008	2.193	5	1	1	1	351	38.4	10.32	3.16	1	metabolic	mitochond	catalytic a	Pf00849	285367	ENSG0000	RPUSD3	3				0								High	High	High	High	High	High	1	
FALSE	High	Master Prc	P42785-1	lysosomal	0	5.91	4	1	1	1	496	55.8	7.21	4.82	1	defense re	membrane	catalytic a	Pf00561, P	5547	ENSG0000	PRCP	11	Protein dig		Intrinsic Pa	8								High	High	High	High	High	High	1	
FALSE	High	Master Prc	Q9NWT6	Hypoxia-in	0.002	3.538	4	1	1	1	349	40.3	5.57	2.03	1	metabolic	cytoplasm;	catalytic a	Pf02373, P	55662	ENSG0000	HIF1AN	10		miR-148a/	Oxygen-de	7								High	High	High	High	High	High	1	
FALSE	High	Master Prc	Q9Y3P9	Rab GTPas	0	5.334	2	2	2	2	1069	121.7	5.25	4.59	2	regulation	cytoplasm;	enzyme re	Pf00566, P	23637	ENSG0000	RABGAP1	9			TBC/RABG	4								High	High	High	High	High	High	1	
FALSE	Medium	Master Prc	Q8IXU6-1	Solute carr	0.012	1.939	3	1	1	1	374	41.2	6.21	1.65	1	transport	membrane		Pf06027, P	54733	ENSG0000	SLC35F2	11				0								High	High	High	High	High	High	1	

FALSE	High	Master Prc	Q8IZ73	RNA pseuc	0	9.964	7	2	2	2	545	61.3	7.17	3.44	2	metabolic		catalytic a	Pf00849	27079	ENSG0000	RPUSD2	15				0								High	High	High	High	High	High	1	
FALSE	High	Master Prc	Q8N0T1	Uncharact	0.003	3.296	20	2	2	2	100	11.4	10.46	1.71	2	metabolic		catalytic a	Pf02384	401466	ENSG0000	C8orf59	8				0								High	High	High	High	High	High	1	
FALSE	High	Master Prc	Q9H0R6-1	Glutamyl-t	0.004	2.704	2	1	1	1	528	57.4	5.68	1.83	1	cell organi	mitochond	catalytic a	Pf01425	55278	ENSG0000	QRSL1	6	Aminoacyl			2								High	High	High	High	High	High	1	
FALSE	High	Master Prc	Q5M775-1	Cytospin-B	0.003	3.363	1	1	2	1	1068	118.5	6.7	6.21	1	cell organi	cytoskelet	catalytic a	Pf00307, P	92521	ENSG0000	SPECC1	17				0								High	High	High	High	High	High	1	
FALSE	High	Master Prc	Q9H8G2-1	Caspase ac	0	4.709	3	1	1	1	361	38.3	4.72	2.73	1	cell death			Pf15335	79886	ENSG0000	CAAP1	9				0								High	High	High	High	High	High	1	
FALSE	Low	Master Prc	P29083	General tra	0.057	1.138	2	1	1	1	439	49.4	4.82	1.75	1	metabolic	cytosol;nu	DNA bindi	Pf02002, P	2960	ENSG0000	GTF2E1	3	Epstein-Ba	Eukaryotic	RNA polym	20								High	High	High	High	High	High	1	
FALSE	High	Master Prc	Q96T23	remodelin	0.003	3.441	1	1	1	1	1441	163.7	5.01	2.4	1	cell organi	nucleus	catalytic a	Pf00628, P	51773	ENSG0000	RSF1	11			Deposition	4								High	High	High	High	High	High	1	
FALSE	Medium	Master Prc	Q8NCM8-	Cytoplasm	0.016	1.688	0	1	1	1	4307	492.3	6.54	1.62	1	cell differe	cytoplasm;	catalytic a	Pf00769, P	79659	ENSG0000	DYNC2H1	11	Salmonella		Hedgehog	9								High	High	High	High	High	High	1	
FALSE	High	Master Prc	Q99470	Stromal ce	0	8.095	15	2	2	2	211	23	7.33	5.23	2	metabolic	extracellul	catalytic a	Pf02815	6388	ENSG0000	SDF2	17				0								High	High	High	High	High	High	1	
FALSE	High	Master Prc	O14662-1	Syntaxin-1	0.001	4.273	7	2	2	2	325	37	6.11	3.38	2	cell organi	cytoplasm;	protein bir	Pf00804, P	8675	ENSG0000	STX16	20	SNARE inte	Ectoderm	Intra-Golgi	7								High	High	High	High	High	High	1	
FALSE	High	Master Prc	P12931	Proto-onco	0	6.246	4	2	4	1	536	59.8	7.42	4.39	2	cell commu	cytoplasm;	catalytic a	Pf00017, P	6714	ENSG0000	SRC	20	Proteoglyc	Androgen	MET activa	175								High	High	High	High	High	High	1	
FALSE	High	Master Prc	Q96L92	Sorting ne	0.002	3.607	2	1	2	1	541	61.2	6.49	5.8	1	regulation	cytoplasm;	protein bir	Pf00595, P	81609	ENSG0000	SNX27	1				0								High	High	High	High	High	High	1	
FALSE	High	Master Prc	O15514	DNA-direct	0	5.111	14	1	1	1	142	16.3	4.79	4.65	1	cell organi	cytosol;nu	catalytic a	Pf03874	5433	ENSG0000	POLR2D	2	Huntingto		Signaling b	74								High	High	High	High	High	High	1	
FALSE	High	Master Prc	Q9C0B5	Palmitoylt	0.002	4.13	2	1	1	1	715	77.5	9.01	2.12	1	metabolic	cytoplasm;	catalytic a	Pf01529	25921	ENSG0000	ZDHHC5	11				0								High	High	High	High	High	High	1	
FALSE	High	Master Prc	Q8N128	Protein FA	0	11.668	23	3	4	3	213	23.7	4.45	7.42	3				Pf14774	283635	ENSG0000	FAM177A	14				0								High	High	High	High	High	High	1	
FALSE	Low	Master Prc	Q5TCZ1	SH3 and P	0.069	1.048	1	1	1	1	1133	125.2	8.9	0	1	cell organi	cytoplasm;	enzyme re	Pf00018, P	9644	ENSG0000	SH3PXD2A	10			Invadopod	2								High	High	High	High	High	High	1	[T938(50);
FALSE	High	Master Prc	Q86YA3-1	Protein ZG	0.004	2.637	1	1	1	1	2104	236.5	6.16	2.9	1		membrane	metal ion I	Pf06839, P	55345	ENSG0000	C4orf21; Z	4				0								High	High	High	High	High	High	1	
FALSE	High	Master Prc	Q8IUC8-1	Polypeptid	0.002	3.636	3	2	4	2	556	64	6.83	3.49	2	metabolic	Golgi;mem	catalytic a	Pf00535, P	114805	ENSG0000	GALNT13	2	Metabolic		O-linked gl	6	4.103		142.29	707.6	543.2	156.56	115.53	High	High	High	High	High	High	1	[R447]
FALSE	High	Master Prc	P78562	Phosphate	0.006	2.527	1	1	2	1	749	86.4	8.76	4.87	1	cell commu	endoplasm	catalytic a	Pf01431, P	5251	ENSG0000	PHEX	X				0	3.701	0.000628739	32.14	2698.3	729	13.86	38.59	High	High	High	High	High	High	1	
FALSE	High	Master Prc	P02765	Alpha-2-HS	0	15.721	5	3	10	3	367	39.3	5.72	19.01	3	defense re	extracellul	enzyme re	Pf00031	197		AHSG	3			Platelet de	11	2.51	0.020534441	10.69	4077	1926.3	10.58	20.22	High	High	High	High	High	High	1	
FALSE	Low	Master Prc	Q9ULE4	Protein FA	0.068	1.062	1	1	1	1	1060	121	6.18	0	1	metabolic		catalytic a	Pf01576, P	27146	ENSG0000	FAM184B	4				0	2.242	0.57356595	20.11	15.9	7.4	65.74	26.61	High	High	High	High	High	High	1	[Q632;
FALSE	High	Master Prc	P68431	Histone H3	0	51.464	58	9	36	4	136	15.4	11.12	51.68	9	cell organi	chromosor	catalytic a	Pf00125, P	8968; 835	ENSG0000	HIST1H3F;	6	Transcripti	Histone M	SIRT1 nega	64	2.207	0.088903983	33.95	9646.2	3518.1	27.55	35.18	High	High	High	High	High	High	1	[K28; K38;
FALSE	High	Master Prc	P69905	Hemoglobi	0	16.648	23	4	7	4	142	15.2	8.68	11.3	4	cell organi	cytosol;ext	antioxidan	Pf00042	3040; 303	ENSG0000	HBA2; HBA	16	Malaria; A	Selenium M	Scavenging	13	2.035	0.028121377	6.81	465.1	175.9	17.03	33.62	High	High	High	High	High	High	1	
FALSE	High	Master Prc	Q8WZ42	Titin [OS=H	0.002	3.533	0	2	4	2	34350	3813.7	6.35	9.75	2	cell organi	chromosor	catalytic a	Pf00041, P	7273	ENSG0000	TTN	2	Dilated car	Striated M	Platelet de	9	2.029	0.120572979	6.1	2031	1001.1	5	45.22	High	High	High	High	High	High	1	
FALSE	High	Master Prc	Q99878	Histone H2	0	68.167	41	7	55	2	128	13.9	10.89	146.01	7	regulation	chromosor	DNA bindi	Pf00125, P	8331; 833	ENSG0000	HIST1H2AJ	6	Alcoholism		SIRT1 nega	66	2.001	0.280962503	80.06	7137.4	3647.1	35.7	42.41	High	High	High	High	High	High	1	
FALSE	High	Master Prc	P43487	Ran-specifi	0	76.237	43	7	22	2	201	23.3	5.29	66.83	7	cell organi	cytoplasm;	enzyme re	Pf00638	5902	ENSG0000	RANBP1	22	Viral carcir		Rev-media	10	1.902	0.141028255	58.97	2111.8	1030.6	15.72	37.97	High	High	High	High	High	High	1	
FALSE	High	Master Prc	Q9Y2R0	Cytochrom	0	13.578	27	3	6	3	106	11.7	9.6	15.11	3	cell organi	membrane	protein bir	Pf09813	28958	ENSG0000	COA3	17				0	1.85	0.087307127	23.77	1746	964.2	34.88	17.37	High	High	High	High	High	High	1	
FALSE	High	Master Prc	P16104	Histone H2	0	47.084	43	7	27	2	143	15.1	10.74	48.92	7	cell organi	chromosor	DNA bindi	Pf00125, P	3014	ENSG0000	H2AFX	11	Alcoholism	miRNA Re	SIRT1 nega	75	1.847	0.139971542	26.97	2162.9	1121.9	30.2	27.76	High	High	High	High	High	High	1	
FALSE	High	Master Prc	Q9NYV4-1	Cyclin-dep	0.001	4.386	1	1	1	1	1490	164.1	9.44	2.85	1	metabolic	chromosor	catalytic a	Pf00069, P	51755	ENSG0000	CDK12	17			TP53 Regu	5	1.762	0.21149203	59.98	662.4	375.9	29.48	23.6	High	High	High	High	High	High	1	
FALSE	High	Master Prc	Q5SNV9	Uncharact	0.008	2.2	1	1	1	1	1468	162.3	10.7	2.56	1					284498	ENSG0000	C1orf167	1				0	1.748	0.463180124	119.22	204.4	122.9	142.76	70.09	High	High	High	High	High	High	1	
FALSE	Medium	Master Prc	O75151	lysine-spec	0.019	1.621	1	1	2	1	1096	120.7	9.17	0	1	cell organi	chromosor	catalytic a	Pf00628, P	5253	ENSG0000	PHF2	9			HDMs dem	3	1.703	0.169063068	28.71	555.2	338.2	13.23	29.34	High	High	High	High	High	High	1	
FALSE	High	Master Prc	Q15269	Periodic tr	0	10.27	5	4	4	4	919	102.4	6.15	8.06	4	cell organi	nucleus	protein bir	Pf00400, P	5822; 102	ENSG0000	PWP2; LOC	21	Ribosome		Major path	6	1.701	0.359935239	76.68	1291.3	789	68.8	21.13	High	High	High	High	High	High	1	
FALSE	High	Protein	P21912	dehydrog	0	15.052	19	5	6	5	280	31.6	8.76	8.75	5	process;tr	e;mitocho	activity;m	Pf13183,	6390	0117118.	SDHB	1	s disease;	Transport	y electron	16	1.681	0.011555477	10.52	3555.7	2114.8	5.83	19.85	High	High	High	High	High	High	1	
FALSE	High	Master Prc	P23142-4	Isoform C	0	12.238	6	3	3	3	683	74.4	5.24	9.62	3	cell organi	extracellul	enzyme re	Pf01821, P	2192	ENSG0000	FBLN1	22				0	1.68	0.039630745	9.66	1268.4	901	14.46	10.98	High	High	High	High	High	High	1	
FALSE	High	Master Prc	P53367	Arfaptin-1	0	7.329	7	2	3	2	373	41.7	6.7	5.59	2	regulation	cytoplasm;	protein bir	Pf03114, P	27236	ENSG0000	ARFIP1	4				0	1.667	0.567488442	133.51	46.7	28	146.55	118.84	High	High	High	High	High	High	1	
FALSE	High	Master Prc	Q96A26	Protein FA	0.002	3.821	5	1	2	1	154	17.3	9.77	2.61	1	cell death;	cytosol;me	protein bir	Pf06388	26355	ENSG0000	FAM162A	3				0	1.662	0.019367779	15.12	2160.9	1250.4	9.09	4.2	High	High	High	High	High	High	1	
FALSE	High	Master Prc	Q5VTU8	ATP syntha	0	6.532	29	2	4	2	51	5.8	10.14	8.64	2	metabolic	membrane	catalytic a	Pf04627	432369	ENSG0000	ATP5EP2	13				0	1.616	0.054044039	0.49	1217.5	828.1	9.61	19.55	High	High	High	High	High	High	1	
FALSE	High	Master Prc	P10412	Histone H1	0	126.984	47	18	96	5	219	21.9	11.03	150.21	18	cell organi	chromosor	DNA bindi	Pf00538	3008	ENSG0000	HIST1H1E	6		Pathways	Formation	11	1.615	0.064634053	18.95	13026.1	8067.1	11.24	14.75	High	High	High	High	High	High	1	[K34]
FALSE	High	Master Prc	Q14166	Tubulin--ty	0	16.776	10	5	7	5	644	74.4	5.53	22.94	5	metabolic		catalytic a	Pf03133	23170	ENSG0000	TTLL12	22			Carboxyte	3	1.615	0.372508617	78.74	886.5	548.8	67.04	12.5	High	High	High	High	High	High	1	
FALSE	High	Master Prc	Q8NBT2	kinetochor	0	7.105	19	2	2	2	197	22.5	4.7	7.88	2	cell divisio	chromosor	protein bir	Pf08286	147841		SPC24	19			Amplificati	16	1.58	0.061770526	2.55	547.6	402.2	13.24	9.39	High	High	High	High	High	High	1	
FALSE	High	Master Prc	P16403	Histone H1	0	113.468	48	17	78	5	213	21.4	10.93	124.3	17	cell organi	chromosor	DNA bindi	Pf00538	3006	ENSG0000	HIST1H1C	6			Formation	10	1.576	0.047627559	21.75	7463.4	4741.6	4.95	18.76	High	High	High	High	High	High	1	[K34]
FALSE	Medium	Master Prc	A8MVM7	Putative u	0.026	1.484	1	1	1	1	634	73.4	9.79	0	1				Pf15090								0	1.576	0.056340173	8.26	147.4	106.8	8.19	15.49	High	High	High	High	High	High	1	
FALSE	High	Master Prc	P18084	Integrin be	0.002	3.834	3	2	2	2	799	88	6.06	2.09	2	cell differe	cell surface	protein bir	Pf00362, P	3693	ENSG0000	ITGB5	3	Focal adhe	Integrin-m	ECM prote	29	1.568	0.267231459	27.06	291	199.3	26.48	12.4	High	High	High	High	High	High	1	
FALSE	High	Master Prc	Q9BRX2	Protein pel	0	12.018	8	2	3	2	385	43.3	6.34	9.88	2	cell divisio	cytoplasm;	catalytic a	Pf03463, P	53918	ENSG0000	PELO	5	mRNA surv	Focal Adhe		3	1.541	0.146479292	8.98	1132.1	706.6	26.32	6.25	High	High	High	High	High	High	1	
FALSE	High	Master Prc	Q8N1W1	Rho guanir	0	7.71	1	1	1	1	1705	191.8	6.04	2.3	1	cell differe	cytoplasm;	metal ion I	Pf00130, P	64283	ENSG0000	ARHGEF28	5			EPHB-med	4	1.538	0.394580021	72.08	155.7	110	64.72	25.21	High	High	High	High	High	High	1	
FALSE	High	Master Prc	P16401	Histone H1	0	95.785	39	14	71	9	226	22.6	10.92	105.51	14	cell organi	chromosor	DNA bindi	Pf00538	3009	ENSG0000	HIST1H1B	6			Formation	10	1.529	0.117195118	8.63	11589.4	7371.4	21.38	16.22	High	High	High	High	High	High	1	
FALSE	High	Master Prc	Q5VST6-1	Protein AB	0.003	2.953	6	2	2	2	288	32.2	6.29	2.29	2	metabolic	extracellul	catalytic a	Pf00326, P	51104	ENSG0000	ABHD17B;	9				0	1.524	0.197740268	24.4	692.6	454.5	8.39	24.37	High	High	High	High	High	High	1	
FALSE	Medium	Master Prc	Q5JWR5	protein do	0.014	1.831	0	1	2	1	2465	277.2	6.29	2.18	1	transport	cytosol;en		Pf04118	23033	ENSG0000	DOPEY1	6				0	1.502	0.515431293	49.95	2119.8	1328.8	5.99	54.83	High	High	High	High	High	High	1	
FALSE	High	Master Prc	Q12962	transcripti	0	10.459	11	1	1	1	218	21.7	6.57	6.6	1	cell death;	cytoplasm;	catalytic a	Pf03540	6881	ENSG0000	TAF10	11	Basal trans		Ub-specific	28	1.493	0.40785963	75.02	345.6	250.9	75.63	15.82	High	High	High	High	High	High	1	
FALSE	High	Master Prc	Q99880	Histone H2	0	107.912	75	14	103	1	126	13.9	10.32	264.66	14	cell organi	chromosor	DNA bindi	Pf00125, P	8340	ENSG0000	HIST1H2BL	6	Viral carcir		SIRT1 nega	77	1.489	0.177624119	37.13	897.7	574.3	13.61	31.89	High	High	High	High	High	High	1	
FALSE	High	Master Prc	P33552	cyclin-depe	0	5.218	20	1	1	1	79	9.9	8.46	5.26	1	cell divisio		enzyme re	Pf01111	1164	ENSG0000	CKS2	9	Small cell l			2	1.488	0.126425476	10.23	501.6	373.6	10.89	15.47	High	High	High	High	High	High	1	
FALSE	High	Master Prc	Q9HCN8	Stromal ce	0.002	3.549	8	2	2	2	221	23.6	7.03	3.03	2	metabolic	endoplasm	catalytic a	Pf02815	23753	ENSG0000	SDF2L1	22				0	1.485	0.00988727	13.68	634.1	427	5.96	16.56	High	High	High	High	High	High	1	
FALSE	High	Master Prc	P16402	Histone H1	0	114.12	49	17	70	6	221	22.3	11.02	109.87	17	cell organi	chromosor	DNA bindi	Pf00538	3007	ENSG0000	HIST1H1D	6			Formation	10	1.467	0.12851042	21.6	4632	3147.4	17.81	15.62	High	High	High	High	High	High	1	[K35]
FALSE	High	Master Prc	P56181-2	Isoform 2	0	8.829	9	3	3	3	473	51	9.17	5.92	3	cell organi	membrane	catalytic a		4731	ENSG0000	NDUFV3	21		Electron Tr		2	1.467	0.307143545	51.86	448.1	322	40.32	18.41	High	High	High	High	High	High	1	
FALSE	High	Master Prc	P17275	transcripti	0.002	3.908	9	2	2	1	347	35.9	9.22	1.91	2	cell differe	chromosor	DNA bindi	Pf00170, P	3726	ENSG0000	JUNB	19	Osteoclast	Corticotro	Interleukin	21	1.463	0.25212264	33.41	31.5	25.3	33.65	10.11	High	High	High	High	High	High	1	
FALSE	Medium	Master Prc	P10588	Nuclear re	0.039	1.281	3	1	2	1	404	43	7.78	4.23	1	metabolic	nucleus	DNA bindi	Pf00104, P	2063	ENSG0000	NR2F6	19		Nuclear Re	Nuclear Re	6	1.463	0.306108716	32.16	1645.4	1328.7	28.17	15.56	High	High	High	High	High	High	1	[R128;
FALSE	High	Master Prc	P30508	HLA class I	0	59.988	39	10	22	5	366	40.9	6.3	53.07	10	regulation	cell surface	protein bir	Pf00129, P	3107		HLA-C	6		Proteasom	Interferon	14	1.462	0.314416721	42.06	2324.5	2065.1	37.88	15.55	High	High	High	High	High	High	1	
FALSE	High	Master Prc	Q9NX14-1	NADH deh	0	8.19	7	1	4	1	153	17.3	5.22	7.36	1	cell organi	membrane	protein bir	Pf10183	54539	ENSG0000	NDUFB11	X	Alzheimer'		Complex I	11	1.455	0.07334813	8.61	3493.6	2102.2	13.18	15.5	High	High	High	High	High	High	1	
FALSE	High	Master Prc	P06748-2	Isoform 2	0	218.223	52	18	169	1	265	29.4	4.61	449.35	18	cell growth	cytoplasm;	DNA bindi	Pf03066	4869	ENSG0000	NPM1	5				0	1.451	0.530734378	34.41	249.8	175.4	16.53	40.83	High	High	High	High	High	High	1	
FALSE	Low	Master Prc	P02788	Lactotrans	0.05	1.198	1	1	1	1	710	78.1	8.12	2.07	1	cellular ho	cell surface	catalytic a	Pf00405	4057	ENSG0000	LTF	3			Neutrophil	12	1.447	0.010621434	7.5	586.8	405.5	2.05	5.89	High	High	High	High	High	High	1	
FALSE	High	Master Prc	Q9Y4W6	AFG3-like	0	20.175	9	7	8	7	797	88.5	8.66	13.76	7	cell organi	membrane	catalytic a	Pf00004, P	10939	ENSG0000	AFG3L2	18			Processing	3	1.445	0.205464012	11.73	593.3	461	12.32	18.22	High	High	High	High	High	High	1	
FALSE	High	Master Prc	P48163	NADP-dep	0.003	3.042	2	1	1	1	572	64.1	6.13	2.28	1	cell organi	cytoplasm;	catalytic a	Pf00390, P	4199	ENSG0000	ME1	6	PPAR signa	NRF2 path	PPARA act	10	1.443	0.378841741	60.62	202.9	144.9	38.89	28.82	High	High	High	High	High	High	1	
FALSE	High	Master Prc	Q8TB61	Adenosine	0	8.187	5	3	4	3	432	47.5	9.16	6.33	3	metabolic	Golgi;mem	signal tran	Pf08449	347734	ENSG0000	SLC35B2	6			Transport	11	1.442	0.40779735	63.76	347.4	277.4	82.2	28.21	High	High	High	High	High	High	1	
FALSE	High	Master Prc	O15085-1	Rho guanir	0	6.323	1	1	1	1	1522	167.6	5.5	3.92	1	cell organi	cytoplasm;	enzyme re	Pf00595, P	9826	ENSG0000	ARHGEF11	1	Vascular sr		G alpha (1	18	1.438	0.451705493	85.73	424.9	295.5	89.53	24.84	High	High	High	High	High	High	1	
FALSE	High	Master Prc	P02686-1	myelin bas	0	5.139	5	1	1	1	304	33.1	9.79	0	1	cell commu	membrane	protein bir	Pf01669	4155	ENSG0000	MBP	18		Neural Cre		6	1.437	0.086791817	9.36	874.2	608.2	2.06	20.11	High	High	High	High	High	High	1	
FALSE	High	Master Prc	A6NDG6	glycerol-3-	0	27.727	25	6	8	6	321	34	6.14	18.91	6	metabolic	cytosol	catalytic a	Pf00702, P	283871	ENSG0000	PGP	16	Glyoxylate		Glycolysis	7	1.435	0.360821119	9.33	1727.4	1358.4	32.56	9.68	High	High	High	High	High	High	1	
FALSE	High	Master Prc	Q86WR0	Coiled-coil	0.002	4.152	8	1	1	1	208	24.5	6.8	2.17	1			protein bir	Pf05670	55246	ENSG0000	CCDC25	8				0	1.434	0.694418613	108.42	149	110.2	76.15	59.07	High	High	High	High	High	High	1	
FALSE	High	Master Prc	P13073	Cytochrom	0	58.949	45	9	25	9	169	19.6	9.51	57.82	9	metabolic	membrane	catalytic a	Pf02936	1327	ENSG0000	COX4I1	16	Parkinson's	Electron Tr	TP53 Regu	17	1.433	0.0666722	4.81	7787.5	5435.3	17.36	7.37	High	High	High	High	High	High	1	
FALSE	High	Master Prc	P14314	Glucosidas	0	87.472	23	14	47	14	528	59.4	4.41	86.4	14	metabolic	endoplasm	metal ion I	Pf07915, P	5589	ENSG0000	PRKCSH	19	Protein pro		Advanced	11	1.432	0.00835537	10.62	11445.8	7990.1	4.45	13.29	High	High	High	High	High	High	1	
FALSE	High	Master Prc	P29372	DNA-3-me	0	9.889	10	1	1	1	298	32.8	9.57	5.41	1	metabolic	cytoplasm;	catalytic a	Pf02245	4350	ENSG0000	MPG	16	Base excisi		Recognitio	9	1.421	0.413262218	67.96	264.3	234.5	52.46	14.7	High	High	High	High	High	High	1	
FALSE	High	Master Prc	O95274	Ly6/PLAUR	0	12.045	11	3	4	3	346	35.9	7.75	5.12	3	metabolic	extracellul	protein bir	Pf00021	27076	ENSG0000	LYPD3	19			Post-transl	3	1.42	0.166255368	35.32	764.3	477.9	16.78	26.52	High	High	High	High	High	High	1	
FALSE	High	Master Prc	Q8NHP8	Putative pl	0.008	2.193	3	1	1	1	589	65.4	6.8	1.67	1	metabolic	organelle l	catalytic a	Pf04916	196463	ENSG0000	PLBD2	12				0	1.413	0.024179052	2.4	283	212.8	8.6	11.78	High	High	High	High	High	High	1	
FALSE	High	Master Prc	P50213-1	Isocitrate	0	43.975	36	13	21	13	366	39.6	6.92	35.99	13	metabolic	mitochond	catalytic a	Pf00180	3419	ENSG0000	IDH3A	15	2-Oxocarb	TCA Cycle	Citric acid	11	1.41	0.02156605	11.6	3795.6	2692.2	6.61	1.34	High	High	High	High	High	High	1	
FALSE	High	Master Prc	O75367-1	Core histor	0	29.178	26	6	7	6	372	39.6	9.79	17.58	6	cell organi	chromosor	DNA bindi	Pf00125, P	9555	ENSG0000	H2AFY	5	Systemic lu	H19 actior		3	1.409	0.084488957	23.99	2694.6	1913	8.7	10.63	High	High	High	High	High	High	1	
FALSE	High	Master Prc	P02768-1	Serum albu	0	17.273	6	3	11	3	609	69.3	6.28	24.28	3	cell commu	cytoplasm;	antioxidan	Pf00273	213	ENSG0000	ALB	4	Thyroid ho	Selenium M	Post-transl	32	1.409	0.089913618	12.7	3354.2	2476	18.39	4.73	High	High	High	High	High	High	1	
FALSE	High	Master Prc	Q00839-2	Isoform Sh	0	345.544	50	45	169	2	806	88.9	5.78	410.23	45	cell differe	cell surface	DNA bindi	Pf00622, P	3192	ENSG0000	HNRNPU	1		mRNA Pro		1	1.407	0.254250078	35.03	3506.7	2196.6	32.62	25.28	High	High	High	High	High	High	1	
FALSE	High	Master Prc	P17858-1	ATP-depen	0	35.571	16	8	12	5	780	85	7.5	27.67	8	cell organi	cytoplasm;	catalytic a	Pf00365	5211	ENSG0000	PFKL	21	Glycolysis ,	Photodyna	Glycolysis;	22	1.406	0.114807916	28.43	312.8	222.5	13.97	7.5	High	High	High	High	High	High	1	
FALSE	High	Master Prc	Q9Y6A5	Transformi	0	7.191	4	2	2	2	838	90.3	5.05	5.1	2	cell divisio	cytoplasm;	protein bir	Pf05010, P	10460	ENSG0000	TACC3	4	RNA trans			1	1.403	0.345328361	50.29	379.2	293.6	46.19	20.99	High	High	High	High	High	High	1	
FALSE	High	Master Prc	P27338	Amine oxic	0	11.235	6	3	5	3	520	58.7	7.5	13.47	3	metabolic	membrane	catalytic a	Pf00070, P	4129	ENSG0000	MAOB	X	Arginine a	Dopamine	Biogenic a	20	1.388	0.545163858	11.05	3042.2	2383.6	36.45	5.04	High	High	High	High	High	High	1	

FALSE	High	Master Pro	P20290-2	Isoform 2 (	0	119.099	67	12	49	1	162	17.7	7.5	132.42	12	metabolic	cytosol;nu	protein bir	Pf01849	689	ENSG0000	BTF3	5				0	1.385	0.333650044	1.37	1029.1	854.3	29.68	13.88	High	High	High	High	High	High	1	[K2]
FALSE	High	Master Pro	O76095-1	Protein JTE	0	9.137	9	1	2	1	146	16.3	8.28	5.46	1	cell death;	cytoplasm;	protein bir	Pf05439	10899	ENSG0000	JTB	1				0	1.384	0.109578199	24.3	282.7	204.6	17.98	0.27	High	High	High	High	High	High	1	
FALSE	High	Master Pro	P10606	Cytochrom	0	36.615	36	8	29	8	129	13.7	8.81	38.43	8	metabolic	membrane	catalytic a	Pf01215	1329	ENSG0000	COX5B	2	Alzheimer'	Electron Tr	TP53 Regu	17	1.382	0.495628222	4.9	10819.2	8120.4	24.08	8.74	High	High	High	High	High	High	1	
FALSE	High	Master Pro	Q5BJD5-1	Transmem	0.006	2.484	7	1	1	1	291	32.5	9.58	0	1		membrane		Pf09335	440026	ENSG0000	TMEM41B	11				0	1.382	0.594994837	20.65	61.8	44.7	3.35	97.15	High	High	High	High	High	High	1	
FALSE	High	Master Pro	Q9HD33-1	39S riboso	0	10.491	12	3	4	3	250	29.4	10.37	8.36	3	cell organi:	membrane	structural	Pf06984	57129	ENSG0000	MRPL47	3			Mitochonc	6	1.38	0.007422346	6.36	1722.4	1196.6	6.65	12.17	High	High	High	High	High	High	1	
FALSE	High	Master Pro	Q9Y6V7	Probable A	0.009	2.104	3	1	1	1	483	54.2	9.06	1.69	1	cell organi:	cytoplasm	catalytic a	Pf00270, P	54555	ENSG0000	DDX49	19			Major path	5	1.378	0.130363327	28.76	346.7	203.1	26.94	18.9	High	High	High	High	High	High	1	
FALSE	High	Master Pro	Q9BXS5-1	AP-1 comp	0	13.059	10	3	3	3	423	48.6	7.3	9.9	3	cell organi:	cytosol;Go	protein bir	Pf00928	8907	ENSG0000	AP1M1	19	Lysosome		Neutrophil	19	1.377	0.133160052	20.73	1018	761	10.06	14.75	High	High	High	High	High	High	1	
FALSE	High	Master Pro	Q9Y5Q8-1	general tra	0	11.096	8	3	4	3	519	59.5	6.9	2.55	3	cell differe	nucleus	DNA bindi	Pf09734	9328	ENSG0000	GTF3C5	9			RNA Polym	6	1.374	0.152867601	16.12	1021.3	743.1	16.98	18.13	High	High	High	High	High	High	1	
FALSE	High	Master Pro	O00622	Protein CY	0	8.624	9	3	4	3	381	42	8.21	9.46	3	cell commi	extracellul	nucleotide	Pf00007, P	3491	ENSG0000	CYR61	1		Oncostatir	Post-transl	6	1.374	0.33045209	38.99	1636.9	1201.3	16.08	22.26	High	High	High	High	High	High	1	
FALSE	High	Master Pro	O60287	nucleolar p	0	5.731	1	1	1	1	2271	254.2	6.47	2.28	1		nucleus	RNA bindi	Pf11707	9875	ENSG0000	URB1	21				0	1.372	0.22880731	4.51	291.4	294.3	33.26	25.05	High	High	High	High	High	High	1	
FALSE	High	Master Pro	Q92620	Pre-mRNA	0	6.689	2	2	2	2	1227	140.4	6.54	3.67	2	metabolic	cytoplasm;	catalytic a	Pf00270, P	9785	ENSG0000	DHX38	16	Spliceosom	mRNA Pro	Cleavage o	13	1.362	0.384859129	58.38	346.8	257.1	40.16	10.42	High	High	High	High	High	High	1	
FALSE	High	Master Pro	O00566	U3 small n	0	16.48	7	5	5	5	681	78.8	4.86	7.72	5	metabolic	chromosor	protein bir	Pf04006	10199	ENSG0000	MPHOSPH	2	Ribosome		Major path	6	1.362	0.454227873	4.62	733.8	539.5	16.88	12.41	High	High	High	High	High	High	1	
FALSE	High	Master Pro	Q8WUA8	Tsukushin	0	5.182	4	1	1	1	353	37.8	6.87	4.27	1	cell organi:	extracellul	protein bir	Pf12799, P	25987	ENSG0000	TSKU	11		Ectoderm		1	1.361	0.189551725	10.98	151	120.3	11.62	12.04	High	High	High	High	High	High	1	
FALSE	High	Master Pro	Q9BV86-1	N-terminal	0	12.604	17	3	3	3	223	25.4	5.52	7.07	3	cell organi:	cytoplasm;	catalytic a	Pf00891, P	28989	ENSG0000	NTMT1	9				0	1.359	0.081026857	23.1	293.4	214.8	2.49	22.11	High	High	High	High	High	High	1	
FALSE	High	Master Pro	Q8WXX5	dnaJ homo	0	7.82	15	2	2	2	260	29.9	5.73	4.89	2	regulation	cytoplasm;	protein bir	Pf00226	23234	ENSG0000	DNAJC9	10				0	1.359	0.411702812	60.61	219.6	167.3	63.62	18.46	High	High	High	High	High	High	1	
FALSE	High	Master Pro	Q96AY3	Peptidyl-pr	0	51.991	25	14	27	14	582	64.2	5.62	27.03	14	cell organi:	cytoplasm;	catalytic a	Pf00254	60681	ENSG0000	FKBP10	17				0	1.354	0.022031765	7.24	7682.7	5347.5	5.65	6.43	High	High	High	High	High	High	1	
FALSE	High	Master Pro	Q9Y639-1	Isoform 1 (	0	6.815	5	1	1	1	282	31.3	6.87	3.65	1	cellular ho	cell surface	protein bir	Pf00047, P	27020	ENSG0000	NPTN	15				0	1.347	0.155524325	14.52	190.6	147.7	8.31	14.07	High	High	High	High	High	High	1	
FALSE	High	Master Pro	Q6NUK1	Calcium-bi	0	25.626	13	7	13	7	477	53.3	6.33	9.34	7	regulation	membrane	metal ion l	Pf00036, P	29957	ENSG0000	SLC25A24	1; CHR_HS				0	1.347	0.246982633	32.93	2884	2563.5	24.58	13.31	High	High	High	High	High	High	1	
FALSE	High	Master Pro	O75208-1	Ubiquinon	0	11.052	17	3	3	3	318	35.5	5.94	5.79	3	metabolic	membrane	protein bir	Pf08511	57017	ENSG0000	COQ9	16			Ubiquinol	4	1.343	0.302592448	43.05	111.3	82.9	27	16.3	High	High	High	High	High	High	1	
FALSE	High	Master Pro	Q9NPE2-2	Neugrin [C	0	10.475	14	4	5	4	291	32.4	9.1	7.5	4	cell differe	extracellul	RNA bindi	Pf06413	51335	ENSG0000	NGRN	15				0	1.34	0.16857734	30.53	577.9	443.2	15.6	15.16	High	High	High	High	High	High	1	
FALSE	High	Master Pro	Q8TB72-1	pumilio ho	0	17.221	3	3	6	1	1066	114.1	7.08	15.32	3	cell organi:	cytoplasm;	protein bir	Pf00806	23369		PUM2	2			Neddylatic	3	1.339	0.479344956	74.1	250.3	194.8	62.64	18.94	High	High	High	High	High	High	1	
FALSE	High	Master Pro	O00560-1	Syntenin-1	0	8.448	10	2	3	2	298	32.4	7.53	11.8	2	cell commi	cytoplasm;	protein bir	Pf00595, P	6386	ENSG0000	SDCBP	8		Ectoderm	Ephrin sigr	10	1.337	0.07186507	15.94	1841.7	1377.1	8.76	6.96	High	High	High	High	High	High	1	
FALSE	High	Master Pro	Q6ZNB6-1	nf-x1-type	0	9.317	4	3	3	3	911	101.3	8.41	5.93	3	metabolic	membrane	DNA bindi		152518	ENSG0000	NFXL1	4				0	1.336	0.416898312	61.1	750.3	561.6	61.92	14.67	High	High	High	High	High	High	1	
FALSE	High	Master Pro	P06733-2	Isoform MI	0	415.27	82	26	277	1	341	36.9	6.28	683.61	26	metabolic	cell surface	catalytic a	Pf00113, P	2023		ENO1	1		Glycolysis		1	1.334	0.539956584	115.67	1292.8	969	127.84	87.97	High	High	High	High	High	High	1	[S277(100
FALSE	High	Master Pro	O43768	alpha-endo	0	18.07	42	5	10	4	121	13.4	7.24	15.24	5	cell divisio	cytoplasm	enzyme re	Pf04667	2029	ENSG0000	ENSA	1			MASTL Fac	5	1.333	0.300820861	13.4	348.7	268.7	9.58	16.46	High	High	High	High	High	High	1	
FALSE	High	Master Pro	P10696	Alkaline ph	0	195.87	55	25	69	8	532	57.3	6.34	169.87	25	metabolic	extracellul	catalytic a	Pf00245	251	ENSG0000	ALPPL2	2	Metabolic		Post-transl	6	1.333	0.694842324	67.13	4995.7	3749.1	33.39	26.54	High	High	High	High	High	High	1	
FALSE	High	Master Pro	Q9NX40	OCIA doma	0	24.164	18	5	8	5	245	27.6	7.49	12.55	5	regulation	endosome	protein bir	Pf07051	54940	ENSG0000	OCIAD1	4				0	1.33	0.041034524	12.11	769	585.5	10.07	14.49	High	High	High	High	High	High	1	
FALSE	Medium	Master Pro	Q9NR81-2	Isoform 2 (	0.047	1.227	2	1	1	1	558	63.7	6.92	2.08	1	regulation	cytosol	protein bir	Pf00621, P	50650	ENSG0000	ARHGEF3	3				0	1.33	0.440653387	18.27	108.5	97.6	23.87	16.2	High	High	High	High	High	High	1	[Q27]
FALSE	High	Master Pro	P63316	Troponin C	0	6.614	9	1	1	1	161	18.4	4.18	4.47	1	cellular co	cytosol	metal ion l	Pf00036, P	7134	ENSG0000	TNNC1	3	Cardiac mu	MFAP5-me	Striated M	9	1.329	0.416774093	28.39	850.3	756	20.8	22.22	High	High	High	High	High	High	1	
FALSE	High	Master Pro	Q9NX18	Succinate (	0	8.012	19	3	3	3	166	19.6	6.8	3.84	3	cell organi:	cytosol;mi	protein bir	Pf03937	54949	ENSG0000	SDHAF2	11				0	1.325	0.871555006	8.6	185.2	140.9	19.62	25.02	High	High	High	High	High	High	1	
FALSE	High	Master Pro	Q9BT25	HAUS augr	0	10.431	11	3	4	3	410	44.8	7.06	3.91	3	cell divisio	cytoplasm;	protein bir		93323	ENSG0000	HAUS8	19			Regulation	16	1.322	0.282447448	5.24	69.9	67.8	18.43	15.19	High	High	High	High	High	High	1	
FALSE	High	Master Pro	Q9HC07	Transmem	0	18.534	17	3	3	3	324	34.9	7.02	11.33	3	cellular ho	endosome		Pf01169	55858	ENSG0000	TMEM165	4				0	1.321	0.409094362	53.78	1210.4	1114.2	69.51	18.4	High	High	High	High	High	High	1	
FALSE	High	Master Pro	P07919	Cytochrom	0	19.647	38	3	10	3	91	10.7	4.44	22.44	3	metabolic	membrane	catalytic a	Pf02320	7388	ENSG0000	UQCRH	1	Metabolic	Electron Tr	Respirator	12	1.319	0.005894986	2.4	3680.5	2918.5	9.32	9.97	High	High	High	High	High	High	1	
FALSE	High	Master Pro	Q15056-2	Isoform Sh	0	39.096	61	11	22	11	228	25.2	8.09	25.86	11	cell organi:	cytosol;me	catalytic a	Pf00076, P	7458	ENSG0000	EIF4H	7		Translatior		1	1.317	0.074371568	2.32	2896.8	2199	3.43	15.61	High	High	High	High	High	High	1	
FALSE	High	Master Pro	Q8N806	Putative E3	0	10.455	8	3	3	3	425	48	4.81	6.87	3	metabolic		catalytic a	Pf02207	55148	ENSG0000	UBR7	14; CHR_H				0	1.317	0.398657535	54.96	369.4	280.4	56.45	13.01	High	High	High	High	High	High	1	
FALSE	Medium	Master Pro	A6NHN6	Nuclear po	0.029	1.434	2	1	1	1	443	51.2	10.49	0	1		extracellul		Pf06409	440348	ENSG0000	NPIPB15; N	16				0	1.316	0.061865382	15.41	287.7	201.2	13.6	6.32	High	High	High	High	High	High	1	
FALSE	High	Master Pro	Q93052	Lipoma-pre	0	14.202	10	4	4	4	612	65.7	7.37	4.27	4		cytoplasm;	metal ion l	Pf00412, P	4026	ENSG0000	LPP	3				0	1.315	0.250326689	31.4	84	63.9	17.26	7.82	High	High	High	High	High	High	1	
FALSE	High	Master Pro	P08047	Transcripti	0.003	3.353	1	1	1	1	785	80.6	7.34	1.75	1	metabolic	cytoplasm;	DNA bindi	Pf00096, P	6667	ENSG0000	SP1	12	Huntingtor	Androgen	RNA polym	47	1.311	0.098724863	3.68	250.6	187.3	20.43	6.33	High	High	High	High	High	High	1	
FALSE	High	Master Pro	P80217	interferon-	0	15.684	17	3	3	3	286	31.5	6.09	7.05	3	regulation	cytosol;nu	protein bir	Pf07292, P	3430	ENSG0000	IFI35	17			Interferon	4	1.309	0.450840998	59.57	457.2	367.4	39.89	17.13	High	High	High	High	High	High	1	
FALSE	High	Master Pro	Q8IUE6	Histone H2	0	41.103	42	6	26	2	130	14	10.89	42.09	6	regulation	chromosor	DNA bindi	Pf00125, P	317772	ENSG0000	HIST2H2AE	1	Alcoholism		HDACs dea	13	1.308	0.170668041	0.69	1095.9	835.8	17.69	25.65	High	High	High	High	High	High	1	[R89]
FALSE	High	Master Pro	Q13505-1	Metaxin-1	0	5.121	7	2	2	2	466	51.4	9.79	0	2	transport	membrane		Pf10568, P	4580		MTX1	1			Cristae for	5	1.308	0.221758226	15.71	539.1	482.4	15.85	14.29	High	High	High	High	High	High	1	
FALSE	High	Master Pro	O95881	Thioredoxi	0	12.189	22	3	4	3	172	19.2	5.4	11.49	3	cell differe	endoplasm	catalytic a	Pf00085, P	51060	ENSG0000	TXNDC12	1	Glutathion			1	1.306	0.64518329	65.17	455.9	310	56.03	8.53	High	High	High	High	High	High	1	
FALSE	High	Master Pro	P48634-1	Protein PR	0	36.356	6	9	12	7	2157	228.7	9.45	18.09	9	cell differe	cytoplasm;	protein bir	Pf07001	7916	ENSG0000	PRRC2A	6				0	1.305	0.188072652	11.83	333.4	259.7	5.63	15.46	High	High	High	High	High	High	1	
FALSE	High	Master Pro	Q6PID8-1	Kelch dom	0	6.895	5	1	1	1	442	49.1	9.38	2.6	1		cytoplasm;	protein bir	Pf01344, P	23008	ENSG0000	KLHDC10	7				0	1.305	0.395974942	6.89	477.9	377.6	20.09	5.38	High	High	High	High	High	High	1	
FALSE	Low	Master Pro	Q9Y2G0	protein EFI	0.057	1.136	1	1	1	1	817	92.4	6.7	2.62	1	cell organi:	cytoplasm;	protein bir		22979	ENSG0000	EFR3B	2				0	1.305	0.537302179	0.85	159.5	164.2	18.22	21.82	High	High	High	High	High	High	1	[R60]
FALSE	High	Master Pro	Q9UMY4-1	Sorting ne	0	15.741	28	5	5	4	172	19.7	7.87	9.95	5	cell organi:	endosome	protein bir	Pf00787	29934		SNX12	X	Endocytosi			1	1.304	0.144152434	24.4	208.2	159.7	12.23	5.84	High	High	High	High	High	High	1	
FALSE	High	Master Pro	O43815	striatin [OS	0	13.603	5	2	3	2	780	86.1	5.27	10.61	2	cell organi:	cytoplasm;	protein bir	Pf00400, P	6801	ENSG0000	STRN	2				0	1.303	0.187721848	29.4	364.5	309.5	17.78	11.75	High	High	High	High	High	High	1	
FALSE	High	Master Pro	Q8NEY8-3	Isoform 3 (	0	6.563	4	1	1	1	434	49.6	8.72	2.71	1	metabolic	chromosor	protein bir	Pf11488	51535	ENSG0000	PPHLN1	12				0	1.3	0.008620845	3.12	602.6	438.3	7.88	8.35	High	High	High	High	High	High	1	
FALSE	High	Master Pro	Q8WWC4	m-AAA pro	0	13.334	11	3	4	3	291	32.5	9.17	7.49	3	cell organi:	membrane	protein bir		79568	ENSG0000	C2orf47; M	2			Processing	3	1.3	0.040298869	3.52	670.9	503.9	5.49	13.42	High	High	High	High	High	High	1	
FALSE	High	Master Pro	P26006	Integrin alp	0	44.964	12	11	14	11	1051	116.5	6.77	27.05	11	cell differe	cell surface	metal ion l	Pf01839, P	3675	ENSG0000	ITGA3	17	Small cell l	Integrin-m	Integrin ce	26	1.3	0.073854218	19.4	696.1	509.5	4.16	13.07	High	High	High	High	High	High	1	
FALSE	High	Master Pro	Q92504	Zinc transp	0	5.147	7	3	3	3	469	50.1	6.87	4.29	3	cellular ho	endoplasm	protein bir	Pf02535	7922	ENSG0000	SLC39A7	6; CHR_HS		NRF2 path	Zinc influx	8	1.294	0.264167096	33.5	1223.1	1133.1	28.92	11.83	High	High	High	High	High	High	1	
FALSE	High	Master Pro	P14927	Cytochrom	0	25.455	39	5	9	5	111	13.5	8.78	23.36	5	cell organi:	membrane	catalytic a	Pf02271	7381	ENSG0000	UQCRB	8	Non-alcoh	Electron Tr	Respirator	12	1.293	0.265603202	12.49	2669.5	2100	21.03	2.6	High	High	High	High	High	High	1	
FALSE	High	Master Pro	P50914	60S riboso	0	37.171	41	10	23	10	215	23.4	10.93	38.47	10	cell organi:	cytosol;me	protein bir	Pf01929	9045	ENSG0000	RPL14	3	Ribosome	Cytoplasm	Peptide ch	34	1.292	0.256317333	27.38	9431.6	7257.4	5.7	20.19	High	High	High	High	High	High	1	
FALSE	High	Master Pro	P56377	AP-1 comp	0	5.182	10	1	1	1	157	18.6	5.47	4.35	1	regulation	cytosol;Go	protein bir	Pf01217	8905	ENSG0000	AP1S2	X	Lysosome		Nef media	17	1.29	0.014183612	7.67	866.5	629.8	4.39	8.32	High	High	High	High	High	High	1	
FALSE	High	Master Pro	Q6IAA8	ragulator c	0	28.184	30	3	5	3	161	17.7	5.15	20.9	3	cell organi:	endosome	protein bir	Pf15454	55004	ENSG0000	LAMTOR1	11	mTOR sign		Energy dep	19	1.289	0.615327611	92.28	987.8	723.9	53.83	15.28	High	High	High	High	High	High	1	
FALSE	High	Master Pro	P53611	Geranylger	0.002	4.112	6	2	2	2	331	36.9	5.03	2.14	2	metabolic	cytosol;me	catalytic a	Pf00432, P	5876	ENSG0000	RABGGTB	1			TP53 regul	9	1.287	0.353217746	43.98	1053.5	818.3	29.47	13.48	High	High	High	High	High	High	1	
FALSE	High	Master Pro	P15923	Transcripti	0	10.54	6	3	3	3	654	67.6	6.47	4.09	3	cell differe	cytoplasm;	DNA bindi	Pf00010	6929	ENSG0000	TCF3	19	Signaling p	Ectoderm	CDO in my	15	1.287	0.499066694	44.46	281.5	255.4	37.03	10.81	High	High	High	High	High	High	1	
FALSE	High	Master Pro	Q15796-1	Mothers ag	0	8.853	8	3	5	3	467	52.3	6.58	10.81	3	cell organi:	cytoplasm;	DNA bindi	Pf03165, P	4087	ENSG0000	SMAD2	18	Pancreatic	AGE/RAGE	Signaling b	55	1.285	0.216386743	19.53	1645	1280.1	8.35	12.65	High	High	High	High	High	High	1	
FALSE	High	Master Pro	O95232-1	Luc7-like p	0	9.803	6	3	4	3	432	51.4	9.79	6.25	3	cell organi:	nucleus;sp	DNA bindi	Pf03194	51747	ENSG0000	LUC7L3	17				0	1.284	0.087236857	19.79	932	725.6	2.32	12.17	High	High	High	High	High	High	1	
FALSE	High	Master Pro	P48735	Isocitrate (	0	9.149	6	2	3	2	452	50.9	8.69	8.67	2	cell organi:	cytosol;me	catalytic a	Pf00180	3418	ENSG0000	IDH2	15	Peroxisom	TCA Cycle	Transcripti	15	1.284	0.228390905	3.29	1446.2	1167.1	2.16	16.05	High	High	High	High	High	High	1	
FALSE	High	Master Pro	Q9Y2W6	tudor and	0	9.827	4	1	2	1	561	62	5.02	10.31	1	cell differe	cytoplasm;	RNA bindi	Pf00013, P	11022	ENSG0000	TDRKH	1			PIWI-intera	3	1.283	0.366747123	45.16	496.6	418.1	32.32	11.84	High	High	High	High	High	High	1	
FALSE	High	Master Pro	Q9Y570	Protein ph	0	28.092	20	6	8	6	386	42.3	5.97	18.94	6	metabolic		catalytic a	Pf00561, P	51400	ENSG0000	PPME1	11			Cyclin A/B1	5	1.283	0.450062448	56.83	1852.6	1551.3	36.55	9.27	High	High	High	High	High	High	1	
FALSE	High	Master Pro	Q86VU5	Catechol O	0	16.786	24	4	5	4	262	28.8	8.38	13.06	4	metabolic	membrane	catalytic a	Pf01596, P	118881	ENSG0000	COMTD1	10				0	1.282	0.019517171	5.33	1229.6	1046.2	9.17	11.69	High	High	High	High	High	High	1	
FALSE	High	Master Pro	P04183	Thymidine	0	21.881	12	2	3	2	234	25.5	8.51	11.53	2	cell organi:	cytoplasm;	catalytic a	Pf00265	7083	ENSG0000	TK1	17	Pyrimidine	Fluoropyri	Activation	14	1.281	0.406488466	51.25	700.2	609.1	42.21	9.76	High	High	High	High	High	High	1	
FALSE	High	Master Pro	Q68DH5	LMBR1 dor	0.001	4.356	2	1	1	1	695	81.1	7.5	1.89	1		membrane		Pf04791	92255	ENSG0000	LMBRD2	5				0	1.28	0.077703565	0.48	262.4	236.2	14.86	12.07	High	High	High	High	High	High	1	
FALSE	High	Master Pro	Q9UJ83	2-hydroxya	0.001	4.377	3	1	1	1	578	63.7	7.36	3.31	1	cell organi:	organelle l	catalytic a	Pf00205, P	26061	ENSG0000	HACL1	3	Peroxisom		Alpha-oxid	6	1.28	0.422257688	53.04	493.8	463.4	73.45	17.32	High	High	High	High	High	High	1	
FALSE	High	Master Pro	Q9NQH7-1	Probable X	0.003	3.024	2	2	4	1	507	57	6.83	0	2	metabolic	mitochond	catalytic a	Pf00557, P	63929	ENSG0000	XPNPEP3	22				0	1.277	0.791066777	11.57	551.7	456.4	18.7	19.58	High	High	High	High	High	High	1	
FALSE	Low	Master Pro	Q8TDW7	Protocadh	0.062	1.107	0	1	1	1	4589	505.2	4.87	1.93	1	developme	membrane	metal ion l	Pf00008, P	120114		FAT3	11				0	1.274	0.010763144	0.78	758.7	607.7	3.37	1.2	High	High	High	High	High	High	1	
FALSE	High	Master Pro	P11177	Pyruvate d	0	43.662	29	9	19	9	359	39.2	6.65	39.61	9	metabolic	mitochond	catalytic a	Pf02779, P	5162	ENSG0000	PDHB	3	Glucagon s	Glycolysis	Regulation	19	1.273	0.056637036	6.88	8931.8	7604.2	9.15	4.79	High	High	High	High	High	High	1	
FALSE	High	Master Pro	Q9UMY1-1	Nucleolar p	0	14.671	14	3	3	3	257	29.4	9.67	7.44	3		mitochond	RNA bindi	Pf08157	51406	ENSG0000	NOL7	6				0	1.273	0.131915904	20.08	764.8	606.4	9.04	11.27	High	High	High	High	High	High	1	
FALSE	High	Master Pro	Q6P158-1	putative A	0	6.575	2	1	1	1	1386	155.5	7.71	3.42	1	metabolic	mitochond	catalytic a	Pf00270, P	90957	ENSG0000	DHX57	2				0	1.273	0.515025006	61.62	187.6	166.1	39.05	15.99	High	High	High	High	High	High	1	
FALSE	High	Protein	Q96I99-1	-CoA	0	53.63	31	12	19	12	432	46.5	6.39	37.51	12	process	e;mitocho	activity;m	Pf01071,	8801	0172340.	SUCLG2	3	cycle (TCA	and	cycle (TCA	10	1.272	0.007369925	1.42	9788.3	7693.7	6.97	4.84	High	High	High	High	High	High	1	
FALSE	High	Master Pro	Q9Y2B0	Protein car	0	38.363	43	6	12	6	182	20.6	4.92	24	6	regulation	endoplasm	protein bir	Pf11938	10330	ENSG0000	CNPY2	12				0	1.272	0.095908581	9.35	5513.6	4068.4	11.88	8.07	High	High	High	High	High	High	1	
FALSE	High	Master Pro	P01111	GTPase NR	0	30.838	49	6	9	3	189	21.2	5.17	21.3	6	cellular co	Golgi;mem	catalytic a	Pf00009, P	4893	ENSG0000	NRAS	1	Longevity r	Signaling P	Regulation	179	1.269	0.037957432	12.12	694.8	572.8	4.45	7.13	High	High	High	High	High	High	1	
FALSE	High	Master Pro	P09874	Poly [ADP-	0	194.306	39	31	67	31	1014	113	8.88	158.78	31	cell death;	membrane	catalytic a	Pf00533, P	142	ENSG0000	PARP1	1	Apoptosis;	Corticotro	POLB-Depe	35	1.269	0.054802665	13	21720.5	17110.9	2.97	7.76	High	High	High	High	High	High	1	
FALSE	High	Master Pro	Q13425-1	Beta-2-syn	0	13.66	9	4	5	4	540	57.9	8.82	5.3	4		cytoplasm;	protein bir	Pf00169, P	6645	ENSG0000	SNTB2	16				0	1.269	0.278879234	34.68	1292.6	1002.2	12.31	20.41	High	High	High	High	High	High	1	
FALSE	High	Master Pro	Q9UBS4	DnaJ homo	0	15.56	11	3	4	3	358	40.5	6.18	8.02	3	metabolic	cytoplasm;	protein bir	Pf00226, P	51726	ENSG0000	DNAJB11	3	Protein pro	Photodyna	XBP1(S) ac	6	1.268	0.026932968	9.37	2235.8	1713.4	8.61	7.77	High	High	High	High	High	High	1	
FALSE	High	Master Pro	Q8NFJ5	Retinoic ac	0	18.391	15	3	5	3	357	40.2	8.15	18.29	3	regulation	membrane	protein bir	Pf00003	9052	ENSG0000	GPRC5A	12		GPCRs, Cla		1	1.268	0.12825058	1.2	1440.3	1153.2	4.32	14.03	High	High	High	High	High	High	1	
FALSE	High	Master Pro	Q04917	14-3-3 pro	0	68.328	50	13	40	8	246	28.2	4.84	62.29	13	cell organi:	cytoplasm;	catalytic a	Pf00244	7533	ENSG0000	YWHAH	22	Oocyte me	Cell Cycle;	Translocat	32	1.267	0.302345389	6.2	3498.3	2819.6	15.08	3.8	High	High	High	High	High	High	1	

FALSE	High	Master Prc	P09651-2	Isoform A1	0	268.742	86	31	111	1	320	34.2	9.23	302.05	31	cell comm	cytoplasm;	DNA bindi	Pf00076, P	3178	ENSG0000	HNRNPA1	12		mRNA Pro		1	1.266	0.048347735	11.55	695.8	522	3.37	11.49	High	High	High	High	High	High	1	[R31;
FALSE	High	Master Prc	P14209	CD99 antig	0.002	4.12	5	1	2	1	185	18.8	4.75	4.97	1	regulation	cytoplasm;	protein bir	Pf12301	4267	ENSG0000	CD99	X|Y; X	Leukocyte		Immunore	7	1.266	0.315921247	34.75	1261.7	839.3	41.56	66.03	High	High	High	High	High	High	1	
FALSE	High	Master Prc	Q9BPW8	Protein Nip	0	4.818	12	3	3	3	284	33.3	9.31	2.72	3		membrane	protein bir	Pf07978	8508	ENSG0000	NIPSNAP1	22				0	1.265	0.304905301	5.59	841.7	665.5	3.87	22.07	High	High	High	High	High	High	1	
FALSE	High	Master Prc	Q9BS26	Endoplasm	0	19.362	19	7	7	7	406	46.9	5.26	17.85	7	cellular ho	cell surface	catalytic a	Pf00085, P	23071	ENSG0000	ERP44	9			Neutrophil	3	1.264	0.006168475	1.38	2532.1	1918	3.51	3.55	High	High	High	High	High	High	1	
FALSE	High	Master Prc	P05026	Sodium/pc	0	57.002	25	6	16	6	303	35	8.53	46.8	6	cell comm	membrane	catalytic a	Pf00287	481	ENSG0000	ATP1B1	1	Thyroid ho	Calcium Re	Ion homeo	27	1.264	0.111493368	16.5	6137.3	4597.7	9.98	10.42	High	High	High	High	High	High	1	
FALSE	High	Master Prc	Q5VTR2	E3 ubiquiti	0	19.1	7	7	8	5	975	113.6	5.94	8.48	7	cell organi	nucleus	catalytic a	Pf00097, P	56254	ENSG0000	RNF20	9		ATM Signa	E3 ubiquiti	5	1.264	0.506938701	67.53	459.6	376.4	52.36	13.01	High	High	High	High	High	High	1	
FALSE	High	Master Prc	Q14657	EKC/KEOPS	0	5.447	16	1	2	1	143	14.8	8.63	0	1	metabolic	nucleus	protein bir	Pf09341	8270	ENSG0000	LAGE3	X			tRNA mod	3	1.263	0.268312547	25.34	32.8	23.4	26.03	18.61	High	High	High	High	High	High	1	
FALSE	High	Master Prc	P61254	60S riboso	0	33.623	46	10	19	2	145	17.2	10.55	44.09	10	cell organi	cytoplasm;	protein bir	Pf00467	6154	ENSG0000	RPL26	17	Ribosome	Cytoplasm	Peptide ch	34	1.263	0.285917517	9.29	2645.9	2349.9	14.48	9.08	High	High	High	High	High	High	1	
FALSE	High	Master Prc	P13051	uracil-DNA	0	13.07	8	1	4	1	313	34.6	9.32	14.96	1	metabolic	mitochond	catalytic a	Pf03167	7374	ENSG0000	UNG	12	Base excisi		Recognitio	10	1.263	0.489950391	59.73	64.6	51.2	38.88	16.61	High	High	High	High	High	High	1	
FALSE	High	Master Prc	Q9NS69	Mitochonc	0	8.706	25	2	3	2	142	15.5	4.34	7.77	2	cell comm	membrane	protein bir		56993	ENSG0000	TOMM22	22			Mitochonc	4	1.263	0.703708066	55.95	1315.6	1041.4	35.44	18.19	High	High	High	High	High	High	1	
FALSE	High	Master Prc	Q8NBS9-1	Thioredoxi	0	35.627	29	12	18	12	432	47.6	5.97	30.04	12	cellular ho	endoplasm	catalytic a	Pf00085, P	81567	ENSG0000	TXNDC5	6	Protein prc		Neutrophil	10	1.262	0.32601289	4.86	4835.3	3830.1	16.19	2.42	High	High	High	High	High	High	1	
FALSE	High	Master Prc	P08574	Cytochrom	0	17.319	16	5	10	5	325	35.4	9	25.27	5	metabolic	membrane	metal ion l	Pf02167	1537	ENSG0000	CYC1	8	Parkinson's		Respirator	13	1.261	0.056598113	10.97	3271	2481.1	10.91	7.39	High	High	High	High	High	High	1	
FALSE	High	Master Prc	Q9H9B4	Sideroflexi	0	68.356	46	9	23	8	322	35.6	9.07	59.92	9	cell differe	membrane	transporte	Pf03820	94081	ENSG0000	SFXN1	5				0	1.261	0.153453186	19.69	9066.4	6864	13.99	12.51	High	High	High	High	High	High	1	
FALSE	High	Master Prc	O00625	Pirin [OS=H	0	37.862	33	7	12	7	290	32.1	6.92	28.99	7	cell differe	cytoplasm;	catalytic a	Pf02678, P	8544	ENSG0000	PIR	X			Digestion	2	1.26	0.076207807	8.82	4390.1	3484.5	9.78	3.53	High	High	High	High	High	High	1	
FALSE	Medium	Master Prc	Q9NVP4-1	Double zin	0.029	1.433	2	1	1	1	752	82.1	8.06	0	1			metal ion l	Pf03174, P	55184	ENSG0000	DZANK1	20				0	1.26	0.128848204	17	729.9	577.4	8.09	12.12	High	High	High	High	High	High	1	[R690];
FALSE	High	Master Prc	Q9H9E3-1	Conserved	0	7.208	4	3	3	3	785	89	5.19	7.48	3	cell organi	endoplasm	protein bir	Pf08318	25839		COG4	16			COPI-medi	11	1.26	0.228784483	15.18	319.6	259.6	12.4	6.66	High	High	High	High	High	High	1	
FALSE	High	Master Prc	P28331-1	NADH-ubic	0	99.821	34	17	24	17	727	79.4	6.23	59.73	17	cell organi	membrane	catalytic a	Pf00111, P	4719	ENSG0000	NDUFS1	2; CHR_HS	Oxidative p	Electron Tr	Complex I	13	1.26	0.286956931	0.61	6820.2	5641.7	13.4	3.84	High	High	High	High	High	High	1	
FALSE	High	Master Prc	O75781	Paralemmi	0	6.523	7	3	3	3	387	42.1	5	3.61	3	cell organi	cytoplasm;	protein bir	Pf03285	5064	ENSG0000	PALM	19				0	1.259	0.333235716	4.05	645.4	552.2	9.4	11.4	High	High	High	High	High	High	1	
FALSE	High	Master Prc	Q96AQ8-1	mitochond	0	8.636	10	3	3	3	359	39.7	9.63	4.98	3	cellular ho	membrane	protein bir	Pf07798	63933	ENSG0000	CCDC90A;	6				0	1.258	0.166713919	11.78	2413.4	1925.1	5.48	11.08	High	High	High	High	High	High	1	
FALSE	High	Master Prc	Q8WVB6	Chromosor	0	7.686	2	1	2	1	975	107.3	7.21	4.04	1	metabolic	cytosol;me	catalytic a	Pf00004, P	63922	ENSG0000	CHTF18	16		Gastric car		1	1.258	0.405062351	33.19	791	709.5	32.78	8.78	High	High	High	High	High	High	1	
FALSE	High	Master Prc	Q14699	raftlin [OS=	0	19.432	11	3	3	3	578	63.1	5.67	10.43	3	cell organi	cytoplasm;	RNA bindi	Pf15250	23180	ENSG0000	RFTN1	3				0	1.258	0.491808201	61.93	21.8	17.3	33.84	16.62	High	High	High	High	High	High	1	
FALSE	High	Master Prc	P06576	ATP syntha	0	394.452	74	26	148	26	529	56.5	5.4	491.8	26	cell differe	cell surface	catalytic a	Pf00006, P	506	ENSG0000	ATP5B	12	Huntingtor	Electron Tr	Transcripti	17	1.256	0.367175758	19.01	47140.5	38033.4	17.4	6.78	High	High	High	High	High	High	1	
FALSE	High	Master Prc	Q5VTL8	Pre-mRNA	0	5.258	5	2	2	2	546	64.4	10.54	3	2	metabolic	nucleus;sp	RNA bindi	Pf03371	55119	ENSG0000	PRPF38B	1	Spliceosom			1	1.256	0.44444627	64.74	466.3	331	51.74	25.63	High	High	High	High	High	High	1	
FALSE	High	Master Prc	Q96DV4	39S riboso	0	11.286	11	4	6	4	380	44.6	7.53	10.71	4	cell organi	membrane		Pf01161	64978	ENSG0000	MRPL38	17			Mitochonc	6	1.255	0.221746262	25.68	1141.3	915.7	19.59	15.18	High	High	High	High	High	High	1	
FALSE	High	Master Prc	Q96HE7	ERO1-like p	0	25.81	22	8	9	8	468	54.4	5.68	16.9	8	cell death;	endoplasm	catalytic a	Pf04137	30001	ENSG0000	ERO1L; ER	14	Vibrio chol		Insulin pro	8	1.254	0.11774576	4.21	2766.6	2205.9	6.96	4.39	High	High	High	High	High	High	1	
FALSE	High	Master Prc	P35659-1	Protein DE	0	8.199	6	2	3	2	375	42.6	8.56	5.64	2	cell organi	nucleus	DNA bindi	Pf02037, P	7913	ENSG0000	DEK	6			Transcripti	7	1.253	0.264994074	31.37	96.6	77.1	15.39	14.49	High	High	High	High	High	High	1	
FALSE	High	Master Prc	O43148-1	mRNA cap	0	7.881	4	2	3	2	476	54.8	6.61	6.07	2	metabolic	nucleus	catalytic a	Pf03291	8731	ENSG0000	RNMT	18	mRNA surv	mRNA Pro	RNA Pol II	14	1.252	0.466985094	65.78	359.6	317	84.87	24.98	High	High	High	High	High	High	1	
FALSE	High	Master Prc	Q69YH5-1	cell divisio	0	5.75	2	1	2	1	1023	112.6	8.4	2.15	1	cell divisio	chromosor		Pf15276	157313	ENSG0000	CDCA2	8				0	1.252	0.472972635	52.1	372.7	334.3	32.83	18.24	High	High	High	High	High	High	1	
FALSE	High	Master Prc	P60033	CD81 antig	0	7.663	10	1	1	1	236	25.8	5.29	5.81	1	cell prolife	membrane	protein bir	Pf00335	975	ENSG0000	CD81	11	Hepatitis C	B Cell Rece	Regulation	10	1.252	0.636793288	2.91	180.8	152.2	18.91	8.55	High	High	High	High	High	High	1	
FALSE	High	Master Prc	P09669	Cytochrom	0	4.551	37	3	4	3	75	8.8	10.39	3.26	3	metabolic	membrane	catalytic a	Pf02937	1345	ENSG0000	COX6C	8	Non-alcoh	Electron Tr	TP53 Regu	17	1.252	0.820793249	5.57	457.5	374.2	12.27	20.93	High	High	High	High	High	High	1	
FALSE	High	Master Prc	Q9BRR6-1	ADP-deper	0	25.848	8	3	6	3	497	54.1	6.2	20.19	3	metabolic	endoplasm	catalytic a	Pf04587	83440		ADPGK	15	Carbon me		Glycolysis	7	1.251	0.159374092	16.73	1458	1244.9	19.06	7.37	High	High	High	High	High	High	1	
FALSE	High	Master Prc	O75947-1	ATP syntha	0	79.292	80	11	32	11	161	18.5	5.3	81.3	11	cell organi	cytosol;me	catalytic a	Pf05873	10476	ENSG0000	ATP5H	17	Oxidative p	Electron Tr	Cristae for	14	1.251	0.386623686	14	7765.7	6490.8	18.98	4.82	High	High	High	High	High	High	1	
FALSE	High	Master Prc	P27824	Calnexin [(	0	226.769	46	31	117	31	592	67.5	4.6	312.17	31	metabolic	cytoplasm;	metal ion l	Pf00262	821	ENSG0000	CANX	5; CHR_HG	HTLV-I infe		Assembly c	25	1.25	0.147896253	2.48	41926.3	35527.4	6.43	7.54	High	High	High	High	High	High	1	
FALSE	High	Master Prc	Q9UHV9	Prefoldin s	0	27.377	51	7	10	7	154	16.6	6.58	10.41	7	metabolic	cytoplasm;	protein bir	Pf01920	5202	ENSG0000	PFDN2	1			Prefoldin r	5	1.25	0.403072471	2.55	2148.4	1689.9	24.68	6.65	High	High	High	High	High	High	1	
FALSE	High	Master Prc	Q9NRR4-1	Ribonuclea	0.003	3.018	2	2	2	2	1374	159.2	7.87	0	2	defense re	membrane	catalytic a	Pf00035, P	29102	ENSG0000	DROSHA	5	Proteoglyc	miRNA Bic	MicroRNA	8	1.25	0.548620571	70.55	1012.6	904.9	58.85	28.02	High	High	High	High	High	High	1	[Q1096];
FALSE	High	Master Prc	P42694	Probable h	0.009	2.147	1	1	3	1	1942	218.8	7.42	5.02	1		membrane	catalytic a	Pf00580, P	9931	ENSG0000	HELZ	17				0	1.25	0.578359685	97.13	1069.8	1100	96.35	34.48	High	High	High	High	High	High	1	[Q1088]
FALSE	High	Master Prc	Q9Y679-1	Ancient ub	0	9.905	6	2	4	2	476	53	8.09	4.69	2	metabolic	endoplasm	enzyme re	Pf02845	550		AUP1	2				0	1.249	0.190599262	18.36	800.9	587.2	17.9	13.52	High	High	High	High	High	High	1	
FALSE	Medium	Master Prc	Q9BXT2	voltage-de	0.048	1.223	3	1	1	1	260	28.1	9.17	0	1	regulation	membrane	transporte	Pf13903	59285	ENSG0000	CACNG6	19	Cardiac mu	Arrhythmo	Phase 0 - r	12	1.246	0.013438827	2.67	1151.6	940.8	13.26	11.81	High	High	High	High	High	High	1	
FALSE	High	Master Prc	Q7Z3K3-1	Pogo trans	0	6.828	2	2	2	2	1410	155.2	7.4	5.85	2	cell divisio	chromosor	DNA bindi	Pf03184, P	23126	ENSG0000	POGZ	1				0	1.246	0.379582466	3.94	302	251	10.5	9.09	High	High	High	High	High	High	1	
FALSE	High	Master Prc	Q96N66-1	Lysophospl	0	25.564	16	6	10	6	472	52.7	8.97	17.69	6	metabolic	membrane	catalytic a	Pf03062	79143	ENSG0000	MBOAT7	19; CHR_H	Glyceropho		Acyl chain	6	1.245	0.093703706	10.07	2326.4	1621.1	9.55	14.17	High	High	High	High	High	High	1	
FALSE	High	Master Prc	Q15155	Nodal moc	0	26.854	7	7	8	1	1222	134.2	5.81	16.69	7		membrane		Pf09430, P	23420	ENSG0000	NOMO1	16; CHR_H				0	1.245	0.203807938	25.24	181.7	146	12.47	10.69	High	High	High	High	High	High	1	
FALSE	High	Master Prc	P16219	Short-chai	0	4.614	4	1	1	1	412	44.3	7.99	3.73	1	metabolic	mitochond	catalytic a	Pf00441, P	35	ENSG0000	ACADS	12	Carbon me	Fatty Acid	Beta oxida	15	1.245	0.879101916	7.53	303.9	244.5	19.45	16.77	High	High	High	High	High	High	1	
FALSE	High	Master Prc	P23434	Glycine cle	0	15.633	17	2	4	2	173	18.9	4.88	13.83	2	metabolic	mitochond	catalytic a	Pf01597	2653	ENSG0000	GCSH	16	Metabolic		Glycine de	7	1.244	0.172516028	20.31	1564.6	1257.8	10.56	5.88	High	High	High	High	High	High	1	
FALSE	Medium	Master Prc	Q8IZD9	Dedicator	0.032	1.398	0	1	1	1	2030	233	6.98	0	1	regulation	cytoplasm;	protein bir	Pf06920, P	1795	ENSG0000	DOCK3	3		Brain-Deri	Factors inv	3	1.243	0.104972617	15.03	64	54.7	5.73	17.31	High	High	High	High	High	High	1	
FALSE	High	Master Prc	Q5T1J5	Putative cc	0	16.463	9	1	3	1	151	15.5	9.89	8.49	1	cell organi	mitochond	DNA bindi		645345		CHCHD2P9	9				0	1.243	0.193237028	3.91	1207.3	881.8	27	6.22	High	High	High	High	High	High	1	
FALSE	High	Master Prc	Q9HDC9	Adipocyte	0	32.607	20	6	12	6	416	46.5	6.16	20.39	6	metabolic	cell surface	catalytic a	Pf03088, P	57136	ENSG0000	APMAP	20				0	1.241	0.033312631	5.98	2395.3	1959.9	5.74	1.02	High	High	High	High	High	High	1	
FALSE	High	Master Prc	Q9NZJ7	Mitochonc	0.002	3.631	3	1	2	1	389	41.5	9.32	0	1	cell death;	membrane	protein bir	Pf00153	23787	ENSG0000	MTCH1	6				0	1.241	0.317841746	2.3	980.6	890.8	10.65	10.3	High	High	High	High	High	High	1	
FALSE	High	Master Prc	P11047	Laminin su	0	8.838	2	2	5	2	1609	177.5	5.12	7.57	2	cell organi	extracellul	catalytic a	Pf00052, P	3915	ENSG0000	LAMC1	1	PI3K-Akt si	Alpha 6 Be	ECM prote	31	1.239	0.093526464	14.88	381.1	308.3	9.69	3.06	High	High	High	High	High	High	1	
FALSE	High	Master Prc	P58107	epiplakin [	0	103.509	21	17	30	13	5090	555.3	5.6	77.98	17	cell organi	cytoplasm;	protein bir	Pf00681	83481		EPPK1	8				0	1.238	0.202683243	3.22	1866.8	1512.1	11	2.46	High	High	High	High	High	High	1	
FALSE	High	Master Prc	O15481	Melanoma	0	4.786	5	2	2	1	346	38.9	9.25	0	2		cytoplasm	protein bir	Pf01454, P	4115	ENSG0000	MAGEB4	X				0	1.238	0.253291035	22.4	680.7	535.8	20.17	16.29	High	High	High	High	High	High	1	
FALSE	High	Master Prc	Q9H089	Large subu	0.004	2.662	2	1	1	1	658	75.2	6.38	2.81	1	transport	cytoplasm;	catalytic a	Pf01926, P	55341	ENSG0000	LSG1	3	Ribosome			1	1.237	0.03235032	4	863.4	726.7	3.14	5	High	High	High	High	High	High	1	
FALSE	High	Master Prc	P80303	Nucleobinc	0	22.372	13	4	7	4	420	50.2	5.12	19.86	4		cytoplasm;	DNA bindi	Pf13499	4925		NUCB2	11				0	1.236	0.039595916	1.94	4288.4	3430.4	9.5	3.51	High	High	High	High	High	High	1	
FALSE	High	Master Prc	Q96B70	Leukocyte	0.006	2.377	2	1	1	1	501	53.1	7.84	1.68	1			metal ion l	Pf00642, P	94059	ENSG0000	LENG9	19; CHR_H				0	1.236	0.312498147	31.2	630.4	510	28.67	9.77	High	High	High	High	High	High	1	
FALSE	High	Master Prc	Q03519-1	antigen pe	0	7.43	4	3	4	2	686	75.6	8.02	3.25	3	response t	endoplasm	catalytic a	Pf00005, P	6891	ENSG0000	TAP2	6; CHR_HS	Herpes sim		ER-Phagos	11	1.236	0.345622155	35.32	2447.7	2109.7	29.47	13.86	High	High	High	High	High	High	1	
FALSE	High	Master Prc	Q09028	Histone-bi	0	39.544	35	9	20	5	425	47.6	4.89	43.28	9	cell organi	cytosol;nu	catalytic a	Pf00400, P	5928	ENSG0000	RBBP4	1		Retinoblas	Regulation	50	1.235	0.211264464	21.42	2021.8	1733.4	20.46	6.86	High	High	High	High	High	High	1	[K264]
FALSE	High	Master Prc	P56545	c-terminal-	0	30.844	19	7	8	3	445	48.9	6.95	13.02	7	cell differe	cytosol;nu	catalytic a	Pf00389, P	1488	ENSG0000	CTBP2	10	Chronic my	Neural Cre	Repression	16	1.234	0.145118298	21.52	1418.9	1137.4	5.33	12.27	High	High	High	High	High	High	1	
FALSE	High	Master Prc	Q2TB90-1	Putative he	0	42.12	15	12	15	11	917	102.5	7.12	31.59	12	cellular ho	cytosol;mi	catalytic a	Pf00349, P	80201	ENSG0000	HKDC1	10	Carbon me			13	1.234	0.257452971	18.93	4254	3807.7	13.56	11.17	High	High	High	High	High	High	1	
FALSE	High	Master Prc	P82673-1	28S riboso	0	12.892	17	4	4	4	323	36.8	8.24	12.76	4	cell organi	membrane	RNA bindi	Pf10213	60488	ENSG0000	MRPS35	12			Mitochonc	6	1.234	0.320447869	37.22	434.9	343.7	27.18	1.94	High	High	High	High	High	High	1	
FALSE	High	Master Prc	Q9H6R4-1	Nucleolar p	0	23.67	6	5	6	5	1146	127.5	7.64	11.69	5	metabolic	chromosor	protein bir	Pf03813	65083	ENSG0000	NOL6	9	Ribosome		Major path	6	1.234	0.534661038	46.72	1051.4	880	24.72	15.9	High	High	High	High	High	High	1	
FALSE	High	Master Prc	Q9NXV2	BTB/POZ d	0	8.496	12	2	2	2	234	26.1	6.24	5.34	2	cell organi	cytoplasm;	protein bir	Pf02214	54442	ENSG0000	KCTD5	16				0	1.233	0.389821818	39.7	138.5	112.3	43.13	18.56	High	High	High	High	High	High	1	
FALSE	High	Master Prc	Q9H3R5	Centromer	0	5.468	7	1	1	1	247	28.5	5.29	4.37	1	cell organi	chromosor	protein bir	Pf05837	64946	ENSG0000	CENPH	5			Amplificati	19	1.232	0.398212975	42.22	452.5	374.1	22.72	11.57	High	High	High	High	High	High	1	
FALSE	High	Master Prc	P54709	sodium/po	0	42.66	47	8	14	8	279	31.5	8.35	39.28	8	cell organi	cytoplasm;	catalytic a	Pf00287	483	ENSG0000	ATP1B3	3	cAMP sign	Calcium Re	Ion homeo	27	1.231	0.057472611	11.07	7912.7	6722.8	6.91	4.67	High	High	High	High	High	High	1	
FALSE	High	Master Prc	Q14527	Helicase-lil	0	16.882	5	4	5	4	1009	113.9	8.6	14.35	4	cell organi	cytoplasm;	catalytic a	Pf00097, P	6596	ENSG0000	HLTF	3		Retinoblas	E3 ubiquiti	5	1.231	0.198379289	2.61	580.3	531.3	9.92	8.29	High	High	High	High	High	High	1	
FALSE	High	Master Prc	P20839	Inosine-5'-	0	13.356	10	4	7	2	514	55.4	6.9	5.36	4	cell prolife	cytoplasm;	catalytic a	Pf00478, P	3614		IMPDH1	7	Drug meta	Nucleotide	Neutrophil	11	1.231	0.421073068	4.23	554.8	469	8.35	11.52	High	High	High	High	High	High	1	
FALSE	High	Master Prc	P29084	transcripti	0	6.562	5	1	1	1	291	33	9.66	3.78	1	metabolic	cytosol;nu	DNA bindi	Pf02186	2961	ENSG0000	GTF2E2	8	Viral carcir	Eukaryotic	RNA polym	20	1.231	0.44192817	50.57	487.4	396.1	44.46	13.63	High	High	High	High	High	High	1	
FALSE	High	Master Prc	Q969H8	Myeloid-de	0	38.558	27	4	16	4	173	18.8	6.68	44.81	4	cell death;	extracellul	protein bir	Pf10572	56005	ENSG0000	C19orf10;	19			XBP1(S) ac	4	1.231	0.87333874	9.53	7147.5	5996.6	26.86	10.49	High	High	High	High	High	High	1	
FALSE	High	Master Prc	Q6P1L8	39S riboso	0	10.792	26	3	3	3	145	15.9	10.24	2.37	3	cell organi	membrane	RNA bindi	Pf00238	64928	ENSG0000	MRPL14	6	Ribosome		Mitochonc	7	1.23	0.010808431	1.82	2232.5	1793.3	10.07	8.75	High	High	High	High	High	High	1	
FALSE	Medium	Master Prc	Q9GZT6	Coiled-coil	0.012	1.987	3	1	2	1	254	29.5	7.55	2.39	1		membrane	protein bir	Pf07798	60492	ENSG0000	CCDC90B	11				0	1.23	0.200510579	17.75	574.4	485.6	3.17	14.97	High	High	High	High	High	High	1	[K124]
FALSE	High	Master Prc	Q8IZL8	Proline-, gl	0	16.071	4	3	4	3	1130	119.6	4.34	7.95	3	metabolic	cytoplasm;	protein bir	Pf08166, P	27043	ENSG0000	PELP1	17			Major path	8	1.23	0.297534399	28.43	651.8	518.8	9.8	16.5	High	High	High	High	High	High	1	
FALSE	High	Master Prc	Q7Z7K6-1	Centromer	0	8.223	10	2	2	2	275	29.9	9.73	5.42	2	cell divisio	chromosor	catalytic a	Pf04828	201161		CENPV	17				0	1.23	0.40788789	12.26	352.7	286.8	17.51	4.53	High	High	High	High	High	High	1	
FALSE	High	Master Prc	Q9BV73-1	Centrosom	0	8.042	1	2	2	2	2442	281	5.02	4.11	2	cell organi	cytoplasm;	protein bir	Pf01496, P	11190	ENSG0000	CEP250	20		Mesoderm	Regulation	17	1.228	0.451096338	55.43	444.1	361.6	64.69	16.94	High	High	High	High	High	High	1	
FALSE	High	Master Prc	Q9UIJ7-1	GTP:AMP p	0.002	3.696	5	1	2	1	227	25.6	9.16	4.62	1	coagulatio	mitochond	catalytic a	Pf00406, P	50808	ENSG0000	AK3	9	Purine met		Factors inv	3	1.227	0.004026085	0.36	1539.7	1269.9	7.72	8.74	High	High	High	High	High	High	1	
FALSE	High	Master Prc	P19367-1	Hexokinase	0	79.765	23	20	27	19	917	102.4	6.8	45.84	20	cellular ho	cytosol;me	catalytic a	Pf00349, P	3098	ENSG0000	HK1	10	Carbon me	Cori Cycle;	Glycolysis;	25	1.226	0.007380284	3.61	11365.2	9506.6	3.12	1.51	High	High	High	High	High	High	1	
FALSE	High	Master Prc	Q9Y3D9	28S riboso	0	6.666	13	2	2	2	190	21.8	8.9	5.07	2	cell organi	cytoskelet	protein bir	Pf10484, P	51649	ENSG0000	MRPS23	17			Mitochonc	6	1.226	0.028678062	5.55	57.6	47.2	12.18	13.27	High	High	High	High	High	High	1	
FALSE	High	Master Prc	O75175	CCR4-NOT	0	16.479	5	3	3	3	753	81.8	6.2	10.99	3	cell differe	cytoplasm;	protein bir	Pf04065, P	4849	ENSG0000	CNOT3	19; CHR_H	RNA degra		TP53 regul	10	1.226	0.032087218	3.7	453.1	369.5	4.43	13.74	High	High	High	High	High	High	1	
FALSE	High	Master Prc	Q9Y5J7	Mitochonc	0	21.608	48	4	8	4	89	10.4	7.21	20.78	4	cell organi	membrane	metal ion l	Pf02953	26520	ENSG0000	TIMM9	14			Mitochonc	2	1.226	0.054676492	5.15	2526.5	1991	10	6.85	High	High	High	High	High	High	1	
FALSE	High	Master Prc	Q14254	Flotillin-2 [	0	7.01	6	3	6	3	428	47	5.25	4.42	3	cell organi	cytoskelet	protein bir	Pf01145	2319	ENSG0000	FLOT2	17	Insulin sigr	Angiopoiet	Synaptic a	6	1.226	0.167167401	21.83	1071.5	873.9	9.12	6.98	High	High	High	High	High	High	1	
FALSE	High	Master Prc	Q8NBX0	saccharopi	0	76.704	35	9	18	9	429	47.1	9.14	54.1	9	metabolic	extracellul	catalytic a	Pf03435, P	51097	ENSG0000	SCCPDH	1			Platelet de	4	1.226	0.175454098	1.64	5898.5	5096.4	8.56	4.8	High	High	High	High	High	High	1	
FALSE	High	Master Prc	O96008	Mitochonc	0	44.455	34	7	10	7	361	37.9	7.25	30.24	7	cell comm	cytosol;me	protein bir	Pf01459	10452	ENSG0000	TOMM40	19	Amyotropl		Mitochonc	5	1.225	0.001399704	0.14	4197.4	3422	0.84	2.41	High	High	High	High	High	High	1	

FALSE	High	Master Prc	P13473-1	Lysosome-	0	4.909	4	2	2	2	410	44.9	5.63	0	2	cell commu	endosome	protein bir	Pf01299	3920	ENSG0000	LAMP2	X	Tuberculos	Senescenc	Platelet de	11	1.225	0.109807086	13.67	528.6	431.7	5.29	6.59	High	High	High	High	High	High	1	
FALSE	High	Master Prc	O00468	Agrin [OS=	0	22.991	4	4	5	4	2067	217.1	6.39	16.62	4	cell differe	cytosol;ext	metal ion l	Pf00050, P	375790		AGRN	1	ECM-recep	Primary Fc	Integrin ce	32	1.225	0.281539752	24.44	998.4	882	29.99	7.84	High	High	High	High	High	High	1	
FALSE	High	Master Prc	Q9NRL3	Striatin-4 [	0	10.347	4	1	1	1	753	80.5	5.4	6.2	1		cytoplasm;	protein bir	Pf00400, P	29888	ENSG0000	STRN4	19				0	1.225	0.515213167	67.37	677	610.1	61.72	12.96	High	High	High	High	High	High	1	
FALSE	High	Master Prc	Q9Y3B9	RRP15-like	0	23.445	18	5	11	5	282	31.5	5.52	19.98	5	metabolic			Pf07890	51018	ENSG0000	RRP15	1				0	1.224	0.29526736	21.55	1559.1	1369.2	18.89	4.7	High	High	High	High	High	High	1	
FALSE	High	Master Prc	Q86XZ4	Spermatog	0	22.653	12	5	6	5	545	59.5	8.9	15.27	5		cytoplasm;	protein bir	Pf07139	65244	ENSG0000	SPATS2	12				0	1.224	0.486635425	78.12	528.4	447.7	101.78	29.72	High	High	High	High	High	High	1	
FALSE	High	Master Prc	P14406	Cytochrom	0	16.912	30	3	4	3	83	9.4	9.76	10.01	3	metabolic	membrane	catalytic ac	Pf02238	1347		COX7A2	6	Alzheimer'	Electron Tr		7	1.223	0.038164733	6.25	1497.9	1224.7	11.17	5.65	High	High	High	High	High	High	1	
FALSE	High	Master Prc	P16435	NADPH--cy	0	68.275	23	14	31	14	677	76.6	5.58	52.29	14	metabolic	endoplasm	catalytic ac	Pf00175, P	5447		POR	7			Cytochrom	4	1.223	0.217762614	7.1	11873.6	10033.9	14.96	3.61	High	High	High	High	High	High	1	
FALSE	Medium	Master Prc	O14936	peripheral	0.048	1.219	1	1	1	1	926	105.1	6.43	1.62	1	metabolic	cytoplasm;	catalytic ac	Pf00018, P	8573	ENSG0000	CASK	X	Tight junct	Parkin-Ubi	Nephrin fa	13	1.222	0.30887399	32.87	303.4	248.3	16.45	11.64	High	High	High	High	High	High	1	
FALSE	High	Master Prc	P31150	Rab GDP d	0	49.195	24	9	18	4	447	50.6	5.14	53.14	9	metabolic	cytoplasm;	catalytic ac	Pf00996	2664	ENSG0000	GDI1	X		Integrated	RAB GEFs e	8	1.221	0.189726675	14.71	306.5	251	34.31	18.44	High	High	High	High	High	High	1	
FALSE	High	Master Prc	P31930	Cytochrom	0	102.171	36	11	34	10	480	52.6	6.37	109.14	11	metabolic	cytosol;me	catalytic ac	Pf00675, P	7384	ENSG0000	UQCRC1	3	Huntingtor	Electron Tr	Respirator	12	1.221	0.336162383	20.99	12526	10262.5	15.9	4.86	High	High	High	High	High	High	1	
FALSE	High	Master Prc	Q9UPN7	Serine/thre	0	5.171	3	1	1	1	881	96.7	4.55	0	1	cell organi:	cytoplasm;	protein bir	Pf03154, P	22870	ENSG0000	PPP6R1	19			COPII (Coa	8	1.221	0.888957898	3.77	269.1	247.1	17.81	25.93	High	High	High	High	High	High	1	
FALSE	High	Master Prc	O43493-1	Trans-Golg	0	10.798	11	5	5	5	480	51.1	5.73	2.6	5	cell organi:	cytosol;en	protein bir		10618		TGOLN2	2			Post-transl	13	1.219	0.159847245	1.16	1224.1	1012	22.94	1.11	High	High	High	High	High	High	1	
FALSE	High	Master Prc	Q8TBF4	Zinc finger	0.004	2.71	4	1	2	1	217	24.6	8.53	1.8	1	metabolic	nucleus;sp	metal ion l	Pf00076, P	85437	ENSG0000	ZCRB1	12			mRNA Spli	4	1.219	0.255557225	16	969.6	773.8	30.19	11.94	High	High	High	High	High	High	1	
FALSE	High	Master Prc	Q92734-1	Protein TF	0	41.605	34	9	11	9	400	43.4	5.1	19.33	9	cell organi:	cytoplasm;	protein bir	Pf00564, P	10342	ENSG0000	TFG	3	Thyroid ca		COPII (Coa	10	1.218	0.016427817	5.56	1239.2	1017.2	4.22	10.47	High	High	High	High	High	High	1	
FALSE	High	Master Prc	Q96EC8-1	Protein YIP	0	9.738	9	3	3	3	236	26.2	5.64	2.54	3		endoplasm	protein bir	Pf04893	286451	ENSG0000	YIPF6	X			Golgi Asso	5	1.218	0.044596018	0.76	236.9	195.8	4.79	1.34	High	High	High	High	High	High	1	
FALSE	High	Master Prc	Q13586	stromal int	0	7.051	4	2	2	2	685	77.4	6.67	4.62	2	cellular ho	cytoplasm;	metal ion l	Pf00430, P	6786	ENSG0000	STIM1	11	Calcium sig		Ion homeo	13	1.217	0.192380738	17.03	390.9	321.3	13.06	10.59	High	High	High	High	High	High	1	
FALSE	High	Master Prc	P62987	Ubiquitin-6	0	38.408	50	7	30	2	128	14.7	9.83	64.1	7	cell commu	cytoplasm;	protein bir	Pf00240, P	7311	ENSG0000	UBA52	19	Ribosome	DNA Replic	NOTCH1 Ir	361	1.217	0.396469027	22.09	1397.9	1148.8	20.13	2.64	High	High	High	High	High	High	1	
FALSE	High	Master Prc	Q1KMD3	heterogen	0	110.014	30	19	30	19	747	85.1	4.91	63.77	19		membrane	protein bir	Pf00622, P	221092	ENSG0000	HNRNPUL2	11				0	1.216	0.13216932	9.37	5810.8	4634.8	14.55	6.55	High	High	High	High	High	High	1	
FALSE	High	Master Prc	O95182	NADH deh	0	18.845	33	3	5	3	113	12.5	10.18	8.98	3	cell organi:	membrane	catalytic ac	Pf07347	4701	ENSG0000	NDUFA7	19	Parkinson's	Electron Tr	Complex I	13	1.216	0.336983374	15.82	1636	1345.6	11.43	8.29	High	High	High	High	High	High	1	
FALSE	High	Master Prc	Q5UCC4-1	ER membra	0	10.321	9	2	2	2	262	27.3	6.13	4.04	2		extracellul			284361	ENSG0000	EMC10	19				0	1.216	0.900895736	10.14	500.4	421.2	35.23	14.29	High	High	High	High	High	High	1	
FALSE	High	Master Prc	Q16822-1	Phosphoer	0	32.372	17	7	9	7	640	70.7	7.62	21.35	7	cellular ho	mitochond	catalytic ac	Pf00821	5106		PCK2	14	Pyruvate n	Adipogene	Gluconeog	21	1.215	0.2629629	16.76	1743.3	1435.1	48.94	26.7	High	High	High	High	High	High	1	
FALSE	High	Master Prc	P50552	Vasodilato	0	53.817	34	10	16	10	380	39.8	8.94	30.42	10	cell organi:	cytoplasm;	protein bir	Pf00568	7408	ENSG0000	VASP	19	Leukocyte	Integrin-m	Cell-extrac	18	1.215	0.277038955	13.71	3882.3	3194.7	8.67	8.29	High	High	High	High	High	High	1	
FALSE	High	Master Prc	P10809	60 kDa hea	0	682.116	82	53	471	53	573	61	5.87	1320.33	53	cell organi:	cell surface	catalytic ac	Pf00118	3329	ENSG0000	HSPD1	2	RNA degra	Apoptosis-	TFAP2A ac	13	1.215	0.489983504	20.7	131000.8	107817.1	13.66	11.46	High	High	High	High	High	High	1	[S67(99.2)]
FALSE	High	Master Prc	Q7Z6E9	E3 ubiquiti	0	6.897	2	2	2	2	1792	201.4	9.64	7.24	2	metabolic	chromosor	catalytic ac	Pf00097, P	5930	ENSG0000	RBBP6	16			Antigen pr	4	1.214	0.169457988	16.96	761.5	628.5	14.35	1.05	High	High	High	High	High	High	1	
FALSE	High	Master Prc	Q5JTZ9	alanine--tR	0	39.212	15	9	11	9	985	107.3	6.27	28.42	9	cell organi:	cytoplasm;	catalytic ac	Pf01411, P	57505	ENSG0000	AARS2	6	Aminoacyl		Mitochonc	5	1.214	0.391168678	38.3	4269.9	3723.9	22.21	14.3	High	High	High	High	High	High	1	
FALSE	High	Master Prc	Q9HD15	Steroid rec	0	7.528	13	2	2	2	236	25.7	7.03	3.11	2	cell death;	cytoplasm;	protein bir	Pf07304	10011	ENSG0000	SRA1	5				0	1.214	0.452493169	53.78	380.1	336	78.82	24.99	High	High	High	High	High	High	1	
FALSE	High	Master Prc	Q9GZP4	PITH doma	0.003	3.482	6	1	1	1	211	24.2	5.74	3.24	1	response t	nucleus		Pf06201	57095	ENSG0000	PITHD1	1				0	1.213	0.031592243	9	805.8	692	11.8	12.39	High	High	High	High	High	High	1	
FALSE	High	Master Prc	P23219	Prostaglan	0	16.36	13	6	8	6	599	68.6	7.23	15.73	6	defense re	cytoplasm;	antioxidan	Pf00008, P	5742	ENSG0000	PTGS1	9	Serotonerg	Selenium M	COX reacti	17	1.213	0.208955379	18.6	2660.4	2177.9	17.16	8.37	High	High	High	High	High	High	1	
FALSE	High	Master Prc	P55809	Succinyl-Co	0	205.374	52	24	91	24	520	56.1	7.46	256.83	24	metabolic	mitochond	catalytic ac	Pf01144	5019	ENSG0000	OXCT1	5	Synthesis a	Synthesis a	Utilization	8	1.213	0.414029092	21.21	31394.2	25871.3	12.45	10.71	High	High	High	High	High	High	1	
FALSE	High	Master Prc	Q96DX5	Ankyrin rep	0	10.192	13	2	2	2	294	31.8	6.98	5.06	2	metabolic	cytosol;mi	protein bir	Pf00023, P	140462	ENSG0000	ASB9	X			Antigen pr	7	1.213	0.476912221	50.34	463.4	456	41.45	12.18	High	High	High	High	High	High	1	
FALSE	High	Master Prc	A6NDU8	UPF0600 p	0	6.934	12	2	2	2	294	33.6	5.26	2.2	2		cytosol			285636	ENSG0000	C5orf51	5				0	1.213	0.919961512	0.88	27.2	25.1	18.99	20.59	High	High	High	High	High	High	1	
FALSE	High	Master Prc	O43678	NADH deh	0	15.75	46	4	9	4	99	10.9	9.57	15.82	4	cell organi:	membrane	catalytic ac	Pf05047	4695	ENSG0000	NDUFA2	5	Huntingtor	Electron Tr	Complex I	13	1.212	0.101419373	8.54	2862.3	2566.9	8.94	8.97	High	High	High	High	High	High	1	
FALSE	High	Master Prc	P42166	Lamina-ass	0	49.28	19	8	17	4	694	75.4	7.66	41.07	8	regulation	chromosor	DNA bindi	Pf03020, P	7112	ENSG0000	TMPO	12				0	1.212	0.1963158	21.64	2353.3	1941.8	12.95	5.84	High	High	High	High	High	High	1	[T74(100)]
FALSE	High	Master Prc	Q5JRA6-1	Transport	0	26.148	4	8	9	8	1907	213.6	4.84	15.53	8	cell organi:	endoplasm	protein bir	Pf02463, P	375056	ENSG0000	MIA3	1			Post-transl	10	1.211	0.176643117	13.73	719.9	643	7.63	10.33	High	High	High	High	High	High	1	
FALSE	High	Master Prc	Q13151	Heterogen	0	89.759	40	13	39	12	305	30.8	9.29	90.87	13	defense re	nucleus	protein bir	Pf00076, P	10949	ENSG0000	HNRNPA0	5			mRNA Spli	4	1.211	0.219082356	0.34	8562.3	7408.7	9.13	3.93	High	High	High	High	High	High	1	
FALSE	High	Master Prc	O60784	Target of M	0	7.786	4	1	1	1	492	53.8	4.7	4.97	1	transport	cytoplasm;	protein bir	Pf00790, P	10043	ENSG0000	TOM1	22			Neutrophil	3	1.211	0.551536087	41.07	148.6	160.2	37.79	14.74	High	High	High	High	High	High	1	
FALSE	High	Master Prc	Q3SXM5	Inactive hy	0	11.984	17	3	3	3	330	37	8.72	9.84	3		mitochond		Pf00106, P	83693	ENSG0000	HSDL1	16				0	1.21	0.457615545	47.94	388.6	321.3	49.44	25.02	High	High	High	High	High	High	1	
FALSE	High	Master Prc	Q9NV70-1	exocyst co	0.002	3.862	2	1	1	1	894	101.9	6.61	2.48	1	defense re	cytoplasm;	protein bir	Pf09763, P	55763	ENSG0000	EXOC1	4			Translocat	10	1.21	0.526078444	55.92	500.3	423.5	36.3	8	High	High	High	High	High	High	1	
FALSE	High	Master Prc	Q9BX68	Histidine tr	0	27.09	28	3	5	3	163	17.2	9.16	16.76	3	cell death;	mitochond	catalytic ac	Pf01230, P	84681	ENSG0000	HINT2	9				0	1.209	0.089620241	11.34	1754.1	1557.9	8.63	5.47	High	High	High	High	High	High	1	
FALSE	High	Master Prc	P14735	insulin-deg	0	29.794	8	8	9	8	1019	117.9	6.61	22.91	8	cell organi:	cell surface	catalytic ac	Pf00675, P	3416	ENSG0000	IDE	10	Alzheimer'	Alzheimers	Ub-specific	6	1.209	0.663647641	63.33	1775.3	1468.2	29.57	28.25	High	High	High	High	High	High	1	
FALSE	High	Master Prc	Q9P032	NADH deh	0	13.292	11	2	5	2	175	20.3	8.82	9.81	2	cell organi:	membrane	protein bir	Pf06784	29078	ENSG0000	NDUFAF4	6			Complex I	5	1.208	0.068012013	4.56	133.3	107.1	12.07	6.64	High	High	High	High	High	High	1	
FALSE	High	Master Prc	P22307-1	Non-specif	0	63.979	20	10	26	10	547	59	6.89	63.1	10	cell organi:	cytoplasm;	catalytic ac	Pf00108, P	6342	ENSG0000	SCP2	1	Metabolic	PPAR Alph	Beta-oxida	19	1.208	0.142507036	0.15	12797	11194.5	10.25	4.77	High	High	High	High	High	High	1	
FALSE	High	Master Prc	Q5JTV8	Torsin-1A-i	0	23.419	14	8	10	7	583	66.2	8.18	16.96	8	cell organi:	membrane	enzyme re	Pf05609	26092	ENSG0000	TOR1AIP1	1				0	1.207	0.006283295	4.9	3389.2	2754.3	10.79	14.98	High	High	High	High	High	High	1	
FALSE	High	Master Prc	Q5T5Y3	Calmodulin	0.006	2.463	1	2	2	2	1602	177.9	6.73	2.37	2	cell organi:	cytoplasm;	protein bir	Pf00307, P	157922	ENSG0000	CAMSAP1	9				0	1.207	0.331552382	33.6	554.7	459.5	18.62	4.52	High	High	High	High	High	High	1	
FALSE	High	Master Prc	P48449-1	lanosterol	0	9.484	6	3	3	3	732	83.3	6.61	6.88	3	metabolic	endoplasm	catalytic ac	Pf00432, P	4047	ENSG0000	LSS	21	Metabolic	Cholestero	Cholestero	10	1.207	0.365684947	12.69	243.1	201.5	19.5	1.16	High	High	High	High	High	High	1	
FALSE	High	Master Prc	Q9H6Y2	WD repeat	0	5.97	4	1	1	1	383	42	4.92	4.71	1	metabolic	cytoplasm;	protein bir	Pf00400	54853	ENSG0000	WDR55	5				0	1.207	0.744370685	3.07	386.8	330.3	24.58	2.17	High	High	High	High	High	High	1	
FALSE	High	Master Prc	Q9NX46	Poly(ADP-r	0	11.243	10	3	3	3	363	38.9	5.07	9.08	3	response t	cytoplasm;	catalytic ac	Pf03747	54936	ENSG0000	ADPRHL2	1			POLB-Depe	5	1.206	0.127176882	15.19	825	668.8	10.91	10.05	High	High	High	High	High	High	1	
FALSE	High	Master Prc	O60684	Importin su	0	47.765	18	8	17	5	536	60	4.98	49.99	8	regulation	cytoplasm;	protein bir	Pf00514, P	23633	ENSG0000	KPNA6	1				0	1.206	0.206815318	16.55	2464.5	2125.5	9.11	8.99	High	High	High	High	High	High	1	
FALSE	High	Master Prc	O15533-1	Tapasin [O	0	13.611	7	3	8	3	448	47.6	7.15	5.99	3	cell organi:	endoplasm	catalytic ac	Pf07654	6892	ENSG0000	TAPBP	6; CHR_HS	Antigen pr		ER-Phagos	7	1.205	0.100736464	10.83	2090	1666.7	9.2	7.3	High	High	High	High	High	High	1	
FALSE	High	Master Prc	Q92643	GPI-anchor	0	13.104	11	3	3	3	395	45.2	6.16	7.3	3	metabolic	endoplasm	catalytic ac	Pf01650	10026	ENSG0000	PIGK	1	Glycosylph		Attachmer	6	1.205	0.105947859	14.8	1009.6	837.6	5.89	7.32	High	High	High	High	High	High	1	
FALSE	High	Master Prc	Q96BR5	Cytochrom	0	16.786	20	4	7	4	231	25.7	6.02	16.85	4		mitochond	protein bir		65260	ENSG0000	SELRC1; CC	1				0	1.204	0.3388599	20.96	2410.4	1914	44.75	13.93	High	High	High	High	High	High	1	
FALSE	High	Master Prc	P48651	phosphatic	0	10.295	11	3	4	3	473	55.5	8.43	10.33	3	metabolic	endoplasm	catalytic ac	Pf03034	9791	ENSG0000	PTDSS1	8	Glycerophc	Kennedy p	Synthesis c	8	1.204	0.484052343	23.56	748.9	678.4	19.99	9.42	High	High	High	High	High	High	1	
FALSE	High	Master Prc	P07203	Glutathion	0	41.936	46	6	9	6	203	22.1	6.55	26.86	6	cell death;	cytoplasm;	antioxidan	Pf00255	2876	ENSG0000	GPX1	3	Arachidoni	Glutathion	Synthesis c	27	1.204	0.926354146	4.19	1103.1	928	22.88	16.42	High	High	High	High	High	High	1	
FALSE	High	Master Prc	Q6UW63	KDEL motif	0	8.316	4	1	1	1	502	58	7.71	5.62	1	metabolic	endoplasm	catalytic ac	Pf00630, P	79070	ENSG0000	KDELC1	13				0	1.203	0.48148314	52.65	230.2	210.5	48.96	13.69	High	High	High	High	High	High	1	
FALSE	High	Master Prc	P56385	ATP syntha	0	8.678	14	1	5	1	69	7.9	9.35	14.94	1	cell organi:	membrane	catalytic ac	Pf05680	521	ENSG0000	ATP5I	4	Metabolic	Electron Tr	Cristae for	11	1.202	0.027703624	7.43	6965.2	5561.8	8.57	9.8	High	High	High	High	High	High	1	
FALSE	High	Master Prc	P51397	Death-asso	0	10.105	24	3	5	3	102	11.2	9.32	8.02	3	cell commu		protein bir	Pf15228	1611	ENSG0000	DAP	5				0	1.202	0.245732955	5.78	2757.4	2326.5	4.23	9.61	High	High	High	High	High	High	1	
FALSE	High	Master Prc	Q8N8A6	ATP-depen	0	8.22	3	1	1	1	666	72.4	8.16	5.49	1	metabolic	cytoplasm;	catalytic ac	Pf00270, P	317781	ENSG0000	DDX51	12				0	1.202	0.375373594	36.8	145.5	121.1	29.46	9.86	High	High	High	High	High	High	1	
FALSE	High	Master Prc	Q99567	Nuclear po	0	32.714	10	6	9	6	741	83.5	5.69	15.08	6	cell organi:	cytosol;nu	protein bir	Pf02050, P	4927	ENSG0000	NUP88	17	RNA transp		Rev-media	64	1.2	0.261168242	3.56	1249.6	1147.1	5.84	11.58	High	High	High	High	High	High	1	
FALSE	High	Master Prc	P63165-1	Small ubiq	0	15.323	28	3	5	3	101	11.6	5.52	10.5	3	cell organi:	chromosor	catalytic ac	Pf00240, P	7341	ENSG0000	SUMO1	2	RNA transp	Androgen	G2/M DNA	43	1.198	0.021735292	2.81	3180	2821.4	7.99	7.83	High	High	High	High	High	High	1	
FALSE	Low	Master Prc	Q8WYH8-1	Inhibitor o	0.068	1.053	3	1	1	1	240	27.7	7.61	0	1	cell organi:	nucleus	metal ion l	Pf00628, P	84289	ENSG0000	ING5	2			HATs acety	9	1.198	0.39369056	37.37	517.4	432	27.07	15.11	High	High	High	High	High	High	1	
FALSE	Medium	Master Prc	P82912-1	28S riboso	0.013	1.859	7	1	1	1	194	20.6	10.81	0	1	cell organi:	membrane	RNA bindi	Pf00411	64963	ENSG0000	MRPS11	15	Ribosome		Mitochonc	7	1.198	0.400318182	28.51	138.5	130.2	18.19	14.66	High	High	High	High	High	High	1	
FALSE	High	Master Prc	Q01130	serine/argi	0	10.918	15	4	10	4	221	25.5	11.85	4.25	4	metabolic	cytosol;nu	protein bir	Pf00076, P	6427	ENSG0000	SRSF2	17	Spliceosom	mRNA Pro	Cleavage o	15	1.198	0.423742582	42.18	1321.2	1103.1	34.44	7.6	High	High	High	High	High	High	1	
FALSE	High	Master Prc	P62072	Mitochonc	0	15.318	44	3	5	3	90	10.3	6.29	20.32	3	cell organi:	membrane	metal ion l	Pf02953	26519	ENSG0000	TIMM10	11			Mitochonc	2	1.197	0.141909519	11.25	1299.3	1174.1	19.81	8.38	High	High	High	High	High	High	1	
FALSE	High	Master Prc	A0FGR8-2	Isoform 2 c	0	46.042	16	10	11	10	893	98.8	8.68	35.98	10	metabolic	membrane	metal ion l	Pf00168	57488	ENSG0000	ESYT2	7				0	1.197	0.2096711	18.97	3723	3318.1	11.05	8.91	High	High	High	High	High	High	1	
FALSE	High	Master Prc	Q15165-1	Isoform 1 c	0	22.542	14	3	11	3	354	39.4	5.5	35.47	3	metabolic	extracellul	catalytic ac	Pf01731, P	5445		PON2	7				0	1.197	0.737750208	13.25	1601.1	1338.1	20.23	8.42	High	High	High	High	High	High	1	
FALSE	High	Master Prc	P21796	voltage-de	0	197.349	67	17	77	17	283	30.8	8.54	221.65	17	cell commu	membrane	protein bir	Pf01459	7416	ENSG0000	VDAC1	5	Huntingtor		Mitochonc	16	1.196	0.02957129	6.27	29336.2	24078.3	4.27	4.75	High	High	High	High	High	High	1	[K236]
FALSE	High	Master Prc	Q8WUI4	histone de	0	6.099	5	3	3	3	952	102.9	7.58	6.3	3	cell differe	cytoplasm;	catalytic ac	Pf00850	51564	ENSG0000	HDAC7	12	Viral carcir	MicroRNA	NOTCH1 Ir	28	1.194	0.178665619	4.45	614.2	442	15.95	15.04	High	High	High	High	High	High	1	
FALSE	High	Master Prc	Q13740-1	CD166 ant	0	80.585	32	12	17	12	583	65.1	6.25	59.46	12	cell growth	extracellul	protein bir	Pf07679, P	214	ENSG0000	ALCAM	3	Cell adhesi		L1CAM int	4	1.194	0.34164909	6.39	3869.8	3322	9.5	6.68	High	High	High	High	High	High	1	
FALSE	High	Master Prc	Q8NC42	E3 ubiquiti	0	8.766	6	1	1	1	400	43.1	6.54	4.02	1	metabolic	membrane	catalytic ac	Pf00097, P	284996	ENSG0000	RNF149	2				0	1.194	0.388655129	32.77	361.6	312.8	57.77	5.79	High	High	High	High	High	High	1	
FALSE	High	Master Prc	Q03518	Antigen pe	0	24.119	7	3	4	3	808	87.2	8.02	16.6	3	defense re	endoplasm	catalytic ac	Pf00005, P	6890	ENSG0000	TAP1	6; CHR_HS	Herpes sim	Mecp2 and	ER-Phagos	13	1.194	0.406249744	34.76	856	711	67.4	17.14	High	High	High	High	High	High	1	
FALSE	High	Master Prc	P00167-1	Cytochrom	0	28.925	58	7	14	7	134	15.3	4.96	27.9	7	metabolic	cytoplasm;	catalytic ac	Pf00173	1528	ENSG0000	CYB5A	18		Oxidation	Vitamin C	5	1.193	0.104854475	8.36	4993	4186.9	13.13	1.67	High	High	High	High	High	High	1	
FALSE	High	Master Prc	Q96D15	Reticulocal	0	50.214	29	6	16	6	328	37.5	4.89	45.88	6		endoplasm	metal ion l	Pf13499	57333	ENSG0000	RCN3	19				0	1.193	0.172295568	3.09	5277.3	4422.7	25.54	6.79	High	High	High	High	High	High	1	
FALSE	High	Master Prc	O43653	prostate st	0	49.72	43	4	9	4	123	12.9	5.29	27.27	4	metabolic	extracellul	protein bir	Pf00021	8000		PSCA	8			Post-transl	3	1.193	0.444501824	30.04	1827.6	1620	6.38	20.14	High	High	High	High	High	High	1	
FALSE	High	Master Prc	P20674	Cytochrom	0	57.995	57	6	23	6	150	16.8	6.79	69.55	6	metabolic	membrane	catalytic ac	Pf02284	9377	ENSG0000	COX5A	15	Metabolic	Electron Tr	TP53 Regu	18	1.193	0.751995581	13.29	5588.8	4685.8	16.43	12.12	High	High	High	High	High	High	1	
FALSE	High	Master Prc	P49903-1	Selenide, w	0	21.656	15	3	4	3	392	42.9	5.97	14.76	3	metabolic	cytoplasm;	catalytic ac	Pf00586, P	22929	ENSG0000	SEPHS1	10	Selenocom			2	1.193	0.906882704	25.92	342.9	290.5	24.93	16.61	High	High	High	High	High	High	1	
FALSE	High	Master Prc	Q96RQ1	Endoplasm	0.002	4.145	3	1	1	1	377	42.5	6.77	3	1	transport	cytoplasm;	protein bir	Pf07970, P	51290	ENSG0000	ERGIC2	12				0	1.192	0.052390726	9.82	686.3	576.7	6.21	0.72	High	High	High	High	High	High	1	
FALSE	High	Master Prc	O43852	Calumenin	0	92.753	59	14	29	14	315	37.1	4.64	86.36	14	metabolic	endoplasm	metal ion l	Pf13499	813	ENSG0000	CALU	7		NOTCH1 re	Platelet de	9	1.192	0.07869057	11.95	9343.5	7841.1	4.74	5	High	High	High	High	High	High	1	[S44(100)]
FALSE	High	Master Prc	P11279	Lysosome-	0	36.175	10	4	14	4	417	44.9	8.75	30.22	4	cell death;	cell surface	protein bir	Pf01299	3916	ENSG0000	LAMP1	13	Phagosom	Senescenc	Neutrophil	7	1.192	0.441241053	7.65	1472.1	1262.2	12.75	6.12	High	High	High	High	High	High	1	
FALSE	High	Master Prc	O14828	Secretory c	0	15.643	5	1	4	1	347	38.3	7.64	18	1	transport	membrane	protein bir	Pf04144	10067	ENSG0000	SCAMP3	1; CHR_HS				0	1.191	0.346410901	5.5	1185.4	1012	14.88	1.21	High	High	High	High	High	High	1	

FALSE	High	Master Prc	O94776	Metastasis	0	33.57	15	10	11	10	668	75	9.66	21.41	10	cell organi	membrane	catalytic a	Pf00249, P	9219	ENSG0000	MTA2	11			ERCC6 (CSI	20	1.19	0.280974215	24.58	4239.2	3622.8	20.05	6.66	High	High	High	High	High	High	1	
FALSE	High	Master Prc	Q7L8L6	FAST kinas	0	6.145	1	1	2	1	764	86.5	8.13	4.61	1	cell organi	mitochond	catalytic a	Pf06743, P	60493	ENSG0000	FASTKD5	20				0	1.19	0.609700196	45.85	477.8	485.2	32.9	15.74	High	High	High	High	High	High	1	
FALSE	High	Master Prc	P40926	Malate del	0	232.562	66	19	105	19	338	35.5	8.68	266.39	19	metabolic	membrane	catalytic a	Pf00056, P	4191	ENSG0000	MDH2	7	Metabolic	Amino Aci	Gluconeog	16	1.189	0.042947945	7.43	40239.9	34798.2	6.72	4.11	High	High	High	High	High	High	1	
FALSE	Medium	Master Prc	O14524-1	Nuclear en	0.015	1.752	2	1	1	1	444	50.6	6.93	2.2	1		membrane		Pf10225	23306	ENSG0000	TMEM194	12				0	1.189	0.136219596	12.94	208.6	175.4	5.6	9.96	High	High	High	High	High	High	1	
FALSE	High	Master Prc	P52306-1	Rap1 GTPa	0	5.995	2	1	1	1	607	66.3	5.31	3.6	1	cell organi	cytosol;en	enzyme re	Pf00514	5910	ENSG0000	RAP1GDS1	4				0	1.188	0.609987004	65.63	747.6	565.2	49.83	43.35	High	High	High	High	High	High	1	
FALSE	High	Master Prc	O75439	mitochond	0	81.579	41	15	21	14	489	54.3	6.83	54.57	15	metabolic	membrane	catalytic a	Pf00675, P	9512	ENSG0000	PMPCB	7			Processing	5	1.187	0.028079303	1.04	6509.9	5516.3	4.93	1.54	High	High	High	High	High	High	1	
FALSE	High	Master Prc	P78381	UDP-galac	0	11.674	8	1	2	1	396	41.3	9.96	9.85	1	metabolic	endoplasm	protein bir	Pf04142	7355	ENSG0000	SLC35A2	X			Defective S	8	1.187	0.597271694	96	225.4	194.8	99.79	55.27	High	High	High	High	High	High	1	
FALSE	High	Master Prc	Q9BTX1	Nucleopori	0	12.165	4	2	4	2	674	76.3	9.09	12.27	2	cell organi	cytoplasm;	structural	Pf09531	55706	ENSG0000	NDC1; TM	1	RNA trans		Rev-media	64	1.186	0.265072021	17.9	136.8	126.4	27.83	5.26	High	High	High	High	High	High	1	
FALSE	High	Master Prc	Q8NEZ2	Vacuolar p	0	14.15	9	2	2	2	397	44.3	5.57	7.65	2	cell commu	cytosol;en	protein bir	Pf07200	137492	ENSG0000	VPS37A	8	Endocytosi		Budding ar	13	1.186	0.407256743	35.84	649.9	559.8	37.78	15.96	High	High	High	High	High	High	1	
FALSE	High	Master Prc	Q9UNH7-1	Sorting ne	0	19.319	14	5	5	5	406	46.6	6.16	6.25	5	cell organi	cytoplasm;	protein bir	Pf00787, P	58533		SNX6	14	Endocytosi			1	1.186	0.445397038	44.48	381.7	321.7	39.58	7.34	High	High	High	High	High	High	1	
FALSE	High	Master Prc	Q9BUR4	Telomeras	0	6.158	7	3	3	3	548	59.3	4.58	4.03	3	cell organi	cytoplasm;	protein bir	Pf00400	55135	ENSG0000	WRAP53	17			Associatio	9	1.186	0.82324302	8.37	838.4	669.3	7.46	43.86	High	High	High	High	High	High	1	
FALSE	High	Master Prc	P31937	3-hydroxyi	0	64.808	35	8	16	8	336	35.3	8.13	56.49	8	metabolic	mitochond	catalytic a	Pf02254, P	11112	ENSG0000	HIBADH	7	Valine, leu	Amino Aci	Branched-(	6	1.185	0.362711507	19.08	4115.3	3562.9	16.35	3.61	High	High	High	High	High	High	1	
FALSE	High	Master Prc	O75381-1	Peroxisom	0	8.464	6	2	4	2	377	41.2	4.94	7.42	2	cell organi	membrane	protein bir	Pf04695	5195	ENSG0000	PEX14	1	Peroxisom			1	1.185	0.413094208	37.23	878.9	837.4	36.03	10.72	High	High	High	High	High	High	1	
FALSE	High	Master Prc	Q9HCC0-1	Methylcrot	0	54.168	22	9	11	9	563	61.3	7.68	36.23	9	cell organi	cytosol;mi	catalytic a	Pf01039	64087	ENSG0000	MCCC2	5	Metabolic		Branched-(	13	1.184	0.201282518	12.82	4329.9	4004.6	23.55	7.31	High	High	High	High	High	High	1	
FALSE	High	Master Prc	Q9BW71-1	HIRA-inter	0	14.478	4	2	4	2	556	61.9	8.54	4.15	2	cell organi	nucleus	protein bir	Pf09649	8479	ENSG0000	HIRIP3	16				0	1.183	0.356038996	31.25	174.9	152.7	14.71	12.56	High	High	High	High	High	High	1	
FALSE	High	Master Prc	O95155	Ubiquitin c	0	20.347	8	8	11	8	1302	146.1	6.55	22.44	8	cell organi	cytoplasm;	catalytic a	Pf04564, P	10277	ENSG0000	UBE4B	1	Protein pro			2	1.183	0.476338362	48.18	837.5	707.9	40.51	11.66	High	High	High	High	High	High	1	[R654];
FALSE	Medium	Master Prc	Q14814-1	Myocyte-s	0.012	1.997	2	1	1	1	521	55.9	7.88	2.27	1	cell death;	cytoplasm;	DNA bindi	Pf00319, P	4209	ENSG0000	MEF2D	1	cGMP-PKG	Energy Me	Transcripti	16	1.182	0.166528318	7.15	477.5	391.4	10.1	12.43	High	High	High	High	High	High	1	
FALSE	High	Master Prc	Q9H788	SH2 domai	0	5.079	3	1	2	1	454	52.7	8.06	8.27	1	regulation	cytoplasm;	protein bir	Pf00017	63898	ENSG0000	SH2D4A	8				0	1.182	0.346017245	30.25	470.9	405.9	23.38	6.28	High	High	High	High	High	High	1	
FALSE	High	Master Prc	P42765	3-ketoacyl-	0	177.396	89	25	51	25	397	41.9	8.09	133.13	25	cell organi	membrane	catalytic a	Pf00108, P	10449	ENSG0000	ACAA2	18	Fatty acid	Fatty Acid	Mitochonc	10	1.182	0.628661412	26.75	16446.2	13911.3	17.74	11.47	High	High	High	High	High	High	1	
FALSE	High	Master Prc	Q16352	alpha-inter	0	45.887	27	12	16	10	499	55.4	5.4	32.06	12	cell differe	cytoskelet	structural	Pf00038, P	9118	ENSG0000	INA	10				0	1.181	0.437933629	18.68	2160.9	1988.8	12.76	14.97	High	High	High	High	High	High	1	
FALSE	High	Master Prc	O75607	Nucleoplas	0	9.095	16	2	2	2	178	19.3	4.63	3.71	2	metabolic	cytoskelet	protein bir	Pf02087, P	10360	ENSG0000	NPM3	10				0	1.18	0.255822915	4.98	228.3	195.5	6.72	5.85	High	High	High	High	High	High	1	
FALSE	High	Master Prc	P62745	Rho-relate	0	5.769	18	2	2	2	196	22.1	5.24	2.85	2	cell death;	cytosol;en	catalytic a	Pf00025, P	388	ENSG0000	RHOB	2		Androgen	G alpha (1	24	1.18	0.283683833	3.68	619.2	507.1	36.97	9.47	High	High	High	High	High	High	1	
FALSE	High	Master Prc	Q99798	Aconitate	0	90.659	31	15	26	15	780	85.4	7.61	69.5	15	metabolic	mitochond	catalytic a	Pf00330, P	50	ENSG0000	ACO2	22	Citrate cyc	Amino Aci	Mitochonc	14	1.18	0.382638178	27.78	4258.8	3256.9	45.61	19.86	High	High	High	High	High	High	1	
FALSE	High	Master Prc	P30101	Protein dis	0	271.594	72	36	124	36	505	56.7	6.35	327.68	36	cellular ho	cell surface	catalytic a	Pf00085, P	2923	ENSG0000	PDIA3	15	Antigen pr		ER-Phagos	13	1.179	0.137046007	5.25	42728.8	36236.4	6.92	2.2	High	High	High	High	High	High	1	
FALSE	High	Master Prc	P08648	Integrin al	0	15.142	3	2	4	2	1049	114.5	5.77	9.45	2	cell differe	cell surface	metal ion	Pf01839, P	3678	ENSG0000	ITGA5	12	Dilated ca	Integrin-m	RUNX2 reg	33	1.179	0.275852897	16.47	922.3	766	26.98	11.23	High	High	High	High	High	High	1	
FALSE	High	Master Prc	P06748	Nucleopho	0	243.63	55	23	206	6	294	32.6	4.78	513.44	23	cell growth	cytoplasm;	DNA bindi	Pf03066	4869	ENSG0000	NPM1	5			Nuclear im	18	1.178	0.141079068	17.77	11892.8	10407.1	5.33	8.85	High	High	High	High	High	High	1	
FALSE	High	Master Prc	Q92797-1	Symplekin	0	8.315	3	2	2	2	1274	141.1	6.13	3.51	2	metabolic	cytoplasm;	protein bir	Pf11935, P	8189	ENSG0000	SYMPK	19	Tight junct		mRNA 3'-e	16	1.178	0.361419141	30.19	366.9	311.5	26.1	9.21	High	High	High	High	High	High	1	
FALSE	High	Master Prc	O75348	V-type pro	0	16.06	21	3	5	3	118	13.7	8.79	12.6	3	cellular ho	cytosol;me	catalytic a	Pf03179	9550	ENSG0000	ATP6V1G1	9	Vibrio chol	Osteoclast	Transferrir	21	1.178	0.468139235	45.03	1067.9	970.7	40.28	8.71	High	High	High	High	High	High	1	
FALSE	High	Master Prc	P20962	Parathymc	0	16.079	28	4	6	4	102	11.5	4.16	9	4	metabolic	nucleus			5763	ENSG0000	PTMS	12				0	1.177	0.011368586	2.17	1377.1	1169.6	4.71	8.34	High	High	High	High	High	High	1	
FALSE	High	Master Prc	P13987	CD59 glyco	0	24.46	25	4	10	4	128	14.2	6.48	20.28	4	cell organi	cell surface	protein bir	Pf00021	966	ENSG0000	CD59	11	Compleme		Regulation	17	1.177	0.066409941	9.47	4635.2	3639.8	9.14	5.12	High	High	High	High	High	High	1	
FALSE	High	Master Prc	Q16629	serine/argi	0	46.799	33	8	21	8	238	27.4	11.82	38.72	8	metabolic	cytoplasm;	metal ion	Pf00076, P	6432	ENSG0000	SRSF7	2	Spliceosom	mRNA Pro	Cleavage o	15	1.177	0.16231505	0.71	6086.3	5910.7	8.57	7.75	High	High	High	High	High	High	1	
FALSE	High	Master Prc	Q9NZ63	Telomere	0	6.031	8	2	2	2	289	33.7	6.74	3.3	2		cytosol	protein bir	Pf07052	51759	ENSG0000	C9orf78	9				0	1.177	0.31728763	27.83	629	546	15.35	8.92	High	High	High	High	High	High	1	
FALSE	High	Master Prc	P28482	mitogen-a	0	13.447	9	2	3	2	360	41.4	6.98	5.7	2	cell commu	cytoplasm;	catalytic a	Pf00069, P	5594	ENSG0000	MAPK1	22	MicroRNA	IL-5 Signali	phospho-P	290	1.177	0.3500778	32.65	219.3	179.4	16.29	13.23	High	High	High	High	High	High	1	
FALSE	High	Master Prc	O15234	Protein CA	0	10.677	5	2	2	2	703	76.2	6.48	4.17	2	metabolic	cytoplasm;	protein bir	Pf09405	22794	ENSG0000	CASC3	17	RNA trans		mRNA 3'-e	19	1.176	0.425864793	37.99	393.7	329.6	57.05	19.03	High	High	High	High	High	High	1	
FALSE	High	Master Prc	Q8NF37	Lysophosp	0	22.828	12	5	13	5	534	59.1	6.02	29.12	5	metabolic	endoplasm	catalytic a	Pf01553, P	79888	ENSG0000	LPCAT1	5	Ether lipid		Synthesis c	14	1.175	0.091045611	0.04	1349.3	1171.7	6.21	1.28	High	High	High	High	High	High	1	
FALSE	High	Master Prc	Q9UPQ0-1	LIM and ca	0	5.754	3	3	3	3	1083	121.8	6.47	2.67	3	cell organi		metal ion	Pf00307, P	22998	ENSG0000	LIMCH1	4				0	1.175	0.307418539	27.09	645.7	549.4	10.97	11.12	High	High	High	High	High	High	1	
FALSE	High	Master Prc	P30084	Enoyl-CoA	0	150.1	62	17	56	17	290	31.4	8.07	159.61	17	metabolic	mitochond	catalytic a	Pf00378, P	1892	ENSG0000	ECHS1	10	Propanoat	Fatty Acid	Beta oxida	24	1.175	0.728084972	19.25	15349	13065.1	15.53	12.54	High	High	High	High	High	High	1	
FALSE	High	Master Prc	P84090	Enhancer c	0	53.989	52	6	30	6	104	12.3	5.92	89.11	6	metabolic		DNA bindi	Pf01133	2079	ENSG0000	ERH	14				0	1.174	0.080468826	7.14	13262.2	10472.7	9.66	9.62	High	High	High	High	High	High	1	
FALSE	High	Master Prc	Q16134	Electron tr	0	30.873	11	5	6	5	617	68.5	7.55	24.73	5	metabolic	membrane	catalytic a	Pf00890, P	2110	ENSG0000	ETFDH	4			Respirator	4	1.173	0.194579742	7.55	1734.2	1584.9	10.02	4.28	High	High	High	High	High	High	1	
FALSE	High	Master Prc	Q9Y2Z0-1	Protein SG	0	20.925	16	5	8	5	365	41	5.16	20.1	5	regulation	chromosor	protein bir	Pf00515, P	10910	ENSG0000	SUGT1	13	NOD-like r	Nucleotide	The NLRP3	7	1.173	0.360332644	29.88	3345.5	2937.2	25.63	6.88	High	High	High	High	High	High	1	
FALSE	High	Master Prc	Q86UE4	protein LYI	0	31.503	12	6	9	6	582	63.8	9.32	23.27	6	cell organi	cytoplasm;	protein bir		92140	ENSG0000	MTDH	8		Gastric car		1	1.173	0.374525931	29.36	1639.5	1397.6	28	17.07	High	High	High	High	High	High	1	
FALSE	High	Master Prc	P53597	Succinate--	0	52.275	22	6	18	6	346	36.2	8.79	48.9	6	metabolic	cytosol;me	catalytic a	Pf00549, P	8802	ENSG0000	SUCLG1	2	Propanoat	Amino Aci	Citric acid	10	1.172	0.063552711	7.09	3227.8	2645.1	8.25	3.08	High	High	High	High	High	High	1	
FALSE	High	Master Prc	P62263	40S riboso	0	62.532	73	11	29	11	151	16.3	10.05	64.2	11	cell differe	cytosol;me	protein bir	Pf00411	6208	ENSG0000	RPS14	5	Ribosome	Cytoplasm	Peptide ch	39	1.172	0.105785797	13.6	5971.1	5095.5	1.88	13.2	High	High	High	High	High	High	1	
FALSE	High	Master Prc	P15927-1	Replicatior	0	26.231	28	4	6	4	270	29.2	6.15	18.47	4	cell organi	chromosor	DNA bindi	Pf08784	6118	ENSG0000	RPA2	1	Mismatch	miRNA Re	Translesior	81	1.172	0.127189349	7.63	593.6	443.9	7.45	15.54	High	High	High	High	High	High	1	
FALSE	High	Master Prc	Q02978	Mitochonc	0	15.951	15	4	4	4	314	34	9.91	4.63	4	metabolic	membrane	RNA bindi	Pf00153	8402	ENSG0000	SLC25A11	17			Gluconeog	4	1.172	0.374869559	31.87	660.9	563.8	17.14	11.67	High	High	High	High	High	High	1	
FALSE	High	Master Prc	Q7LBC6-1	Lysine-spe	0	6.295	2	2	2	2	1761	191.5	7.18	6.36	2	cell organi	chromosor	antioxidan	Pf02373	51780	ENSG0000	KDM3B	5			HDMs den	3	1.172	0.929997287	37.56	18.3	16.1	11.6	29.93	High	High	High	High	High	High	1	
FALSE	High	Master Prc	Q9BXB5	Oxysterol-	0	9.552	2	1	1	1	764	83.9	8.31	2.21	1	metabolic	cytoplasm;	transporte	Pf00169, P	114884	ENSG0000	OSBPL10	3			Acyl chain	5	1.171	0.072035108	9.27	125.7	101	14.31	10.83	High	High	High	High	High	High	1	
FALSE	High	Master Prc	P14625	Endoplasm	0	335.099	59	55	174	53	803	92.4	4.84	325.37	55	cell organi	cytosol;en	metal ion	Pf00183, P	7184	ENSG0000	HSP90B1	12	Protein pr	Photodyna	Post-transl	26	1.171	0.15946622	4.56	50901	45189.6	8.32	4.63	High	High	High	High	High	High	1	[S650(98.
FALSE	High	Master Prc	P62633-1	Cellular nu	0	58.929	67	11	23	11	177	19.5	7.71	62.25	11	metabolic	cytoplasm;	DNA bindi	Pf00098	7555	ENSG0000	CNBP	3				0	1.171	0.161154488	8.22	12379.9	10962.4	18.17	3.63	High	High	High	High	High	High	1	
FALSE	High	Master Prc	P10909-1	Clusterin [	0	15.735	11	4	4	4	449	52.5	6.27	6.54	4	cell death;	cell surface	catalytic a	Pf01093	1191	ENSG0000	CLU	8	Compleme	Compleme	Platelet de	12	1.171	0.196727155	16.49	572.3	483.7	12.2	8.39	High	High	High	High	High	High	1	
FALSE	High	Master Prc	O00193	Small acidi	0	28.685	34	6	8	6	183	20.3	4.72	19.18	6				Pf15477	10944	ENSG0000	C11orf58	11				0	1.171	0.388541386	32.81	754.9	732.1	25.73	7.56	High	High	High	High	High	High	1	
FALSE	High	Master Prc	P09496-2	Isoform Nc	0	27.303	32	7	13	7	218	23.6	4.53	25.84	7	cell divisio	cytosol;me	protein bir	Pf01086	1211	ENSG0000	CLTA	9		Synaptic V		1	1.17	0.023968897	6.46	11998.8	10259.3	4.11	9.16	High	High	High	High	High	High	1	
FALSE	High	Master Prc	Q13444	Disintegrin	0	5.307	2	1	1	1	863	92.9	6.73	2.7	1	cell death;	cell surface	catalytic a	Pf00200, P	8751	ENSG0000	ADAM15	1			Degradatic	3	1.17	0.228306308	18.97	735.9	665.3	15.77	5.83	High	High	High	High	High	High	1	
FALSE	High	Master Prc	Q8N357	Solute carr	0.002	3.61	3	1	1	1	371	40.2	6.93	2.44	1	regulation	cytosol;me	protein bir	Pf00892, P	54978	ENSG0000	C2orf18; S	2				0	1.17	0.247475551	8.37	552.7	518.2	13.68	11.32	High	High	High	High	High	High	1	
FALSE	High	Master Prc	O00330-1	Pyruvate d	0	35.589	15	7	11	7	501	54.1	8.66	19.95	7	metabolic	mitochond	catalytic a	Pf00198, P	8050	ENSG0000	PDHX	11	Metabolic	Amino Aci	Regulation	13	1.17	0.276019292	14.86	2258.5	2042.6	28.69	6.47	High	High	High	High	High	High	1	
FALSE	High	Master Prc	Q96EA4	protein Spi	0	5.318	2	1	2	1	605	70.1	5.47	4.72	1	cell divisio	chromosor	protein bir		54908	ENSG0000	SPDL1	5			Amplificati	16	1.17	0.299342096	21.63	248.4	216.8	24.61	5.05	High	High	High	High	High	High	1	
FALSE	High	Master Prc	Q9H0V9-1	VIP36-like	0	10.425	7	1	1	1	348	39.7	8.38	5.7	1	metabolic	endoplasm	metal ion	Pf03388	81562	ENSG0000	LMAN2L	2			Cargo conc	9	1.17	0.50404645	15.34	167.6	143.3	5.38	14.51	High	High	High	High	High	High	1	
FALSE	High	Master Prc	P50402	Emerin [OS	0	28.436	33	6	15	6	254	29	5.5	29.32	6	cell differe	cytoplasm;	protein bir	Pf03020	2010	ENSG0000	EMD	X	Arrhythmc	Arrhythmc	Depolymer	15	1.169	0.001448874	1.64	2978.2	2575.3	3.74	4.47	High	High	High	High	High	High	1	
FALSE	High	Master Prc	Q9H8Y8-1	Golgi reass	0	23.625	17	6	12	5	452	47.1	4.82	20.13	6	cell organi	Golgi;mem	protein bir	Pf04495	26003	ENSG0000	GORASP2	2			Golgi Ciste	5	1.169	0.078928133	9.29	2181.5	1973.8	9.77	5.77	High	High	High	High	High	High	1	
FALSE	High	Master Prc	Q15084-1	Protein dis	0	94.762	31	12	28	12	440	48.1	5.08	88.4	12	cellular ho	cytosol;en	catalytic a	Pf00085, P	10130	ENSG0000	PDIA6	2	Protein pr	Photodyna	Post-transl	9	1.168	0.41242016	4.88	11880	10670.1	8.09	7.99	High	High	High	High	High	High	1	
FALSE	High	Master Prc	Q9Y5Y0	Feline leuk	0	5.427	2	1	1	1	555	59.8	5.97	2.88	1	cell differe	membrane	protein bir	Pf07690, P	28982	ENSG0000	FLVCR1	1			Iron uptak	2	1.168	0.448207372	39.19	382.5	361.4	33.83	9.46	High	High	High	High	High	High	1	
FALSE	High	Master Prc	Q9NVS2	39S riboso	0	7.217	12	2	5	2	196	22.2	10.33	5.09	2	cell organi	membrane	structural	Pf01084	55168	ENSG0000	MRPS18A	6	Ribosome		Mitochonc	7	1.167	0.015686783	3.07	460.7	400.7	7.93	5.92	High	High	High	High	High	High	1	
FALSE	High	Master Prc	P07237	Protein dis	0	267.433	71	36	134	36	508	57.1	4.87	328.06	36	cell organi	endoplasm	catalytic a	Pf00085, P	5034	ENSG0000	P4HB	17	Protein pr		Post-transl	23	1.166	0.00323386	0.24	48183.4	40794.8	3.09	2.42	High	High	High	High	High	High	1	
FALSE	High	Master Prc	P08174-1	Compleme	0	72.359	33	11	38	11	381	41.4	7.59	96.39	11	cellular ho	cell surface	protein bir	Pf00084	1604	ENSG0000	CD55	1	Compleme	Allograft R	Regulation	23	1.166	0.086315315	11.19	19298.7	16544.4	2.04	6.56	High	High	High	High	High	High	1	
FALSE	High	Master Prc	Q9UH62	Armadillo	0	7.443	8	2	2	2	379	42.5	8.37	7.74	2	regulation	cytoplasm;	protein bir	Pf04826, P	51566	ENSG0000	ARMCX3	X				0	1.166	0.27276573	23.95	622.7	534.1	8.23	10.38	High	High	High	High	High	High	1	
FALSE	High	Master Prc	O75251	NADH deh	0	11.724	10	2	5	2	213	23.5	9.99	11.07	2	cell organi	membrane	catalytic a	Pf01058	374291	ENSG0000	NDUFS7	19	Non-alcoh	Electron T	Complex I	13	1.166	0.516104625	3.03	2374.3	2262.7	11.45	8.48	High	High	High	High	High	High	1	
FALSE	High	Master Prc	Q9NX55-2	Huntingtin	0	12.344	40	4	6	4	129	14.7	4.93	14.25	4	regulation	cytoplasm;	protein bir		25764	ENSG0000	HYPK	15				0	1.166	0.591311899	24.06	485.9	424.3	25.58	1.17	High	High	High	High	High	High	1	
FALSE	High	Master Prc	Q16836-1	Hydroxyac	0	79.759	62	10	19	10	314	34.3	8.85	58.99	10	metabolic	cytoplasm;	catalytic a	Pf00070, P	3033	ENSG0000	HADH	4	Fatty acid	Fatty Acid	Beta oxida	23	1.166	0.856181907	30.4	3373.8	2892.7	23.46	13.82	High	High	High	High	High	High	1	
FALSE	High	Master Prc	P08729	Keratin, ty	0	150.437	45	28	105	21	469	51.4	5.48	185.4	28	cell death	cytoplasm;	protein bir	Pf00038, P	3855	ENSG0000	KRT7	12			Formation	3	1.165	0.246735213	7.97	20287.2	18214.6	11.14	2.67	High	High	High	High	High	High	1	[Y283(100
FALSE	High	Master Prc	P24752	Acetyl-CoA	0	195.386	71	26	60	26	427	45.2	8.85	184.66	26	cell organi	membrane	catalytic a	Pf00108, P	38	ENSG0000	ACAT1	11	Fatty acid	Fatty Acid	Synthesis c	23	1.165	0.284971542	9.07	22973.6	20021.3	10.01	3.45	High	High	High	High	High	High	1	
FALSE	High	Master Prc	P78362-1	SRSF prote	0	15.126	6	3	3	2	688	77.5	4.97	7.65	3	cell differe	cytoplasm;	catalytic a	Pf00069, P	6733	ENSG0000	SRPK2	7		mRNA Pro		1	1.164	0.522885293	47.99	391.7	408.6	41.2	11.65	High	High	High	High	High	High	1	
FALSE	High	Master Prc	Q9H2P0	Activity-de	0.003	3.016	1	1	1	1	1102	123.5	7.34	3.28	1	cell commu	cytoplasm;	DNA bindi	Pf00046, P	23394	ENSG0000	ADNP	20				0	1.164	0.629149476	51.75	78.7	67.6	23.71	14.51	High	High	High	High	High	High	1	
FALSE	Medium	Master Prc	P45954	Short/bran	0.024	1.496	2	1	1	1	432	47.5	6.99	0	1	metabolic	mitochond	catalytic a	Pf00441, P	36	ENSG0000	ACADSB	10	Fatty acid	Valproic ac	Branched-(	8	1.163	0.080298538	8.45	246.5	224.8	11.99	6.42	High	High	High	High	High	High	1	
FALSE	High	Master Prc	P62070	Ras-relate	0	7.704	9	2	3	2	204	23.4	6.01	4.94	2	cell differe	endoplasm	catalytic a	Pf00071, P	22800	ENSG0000	RRAS2	11	Regulation	Regulation		9	1.163	0.200860025	8.77	653	640.2	11.72	8.78	High	High	High	High	High	High	1	
FALSE	High	Master Prc	Q13423	NAD(P) tra	0	268.724	36	38	102	38	1086	113.8	8.09	255.02	38	metabolic	membrane	catalytic a	Pf01262, P	23530	ENSG0000	NNT	5	Nicotinate		Citric acid	6	1.163	0.303517863	9.56	37390.9	34108.8	10.67	5.31	High	High	High	High	High	High	1	
FALSE	High	Master Prc	Q86YP4	Transcripti	0	17.477	9	4	4	4	633	68	9.94	4.33	4	metabolic	nucleus	DNA bindi		54815	ENSG0000	GATAD2A	19			ERCC6 (CSI	20	1.163	0.43326048	40.74	1018.6	875.7	60.76	15.78	High	High	High	High	High	High	1	
FALSE	High	Master Prc	Q9UPQ9	Trinucleoti	0	9.838	1	1	1	1	1833	193.9	6.76	5.37	1	regulation	cytoplasm;	protein bir	Pf10427, P	23112	ENSG0000	TNRC6B	22			Post-trans	29	1.163	0.671117498	15.77	480	474.4	16.77	14.14	High	High	High	High	High	High	1	
FALSE	High	Master Prc	P43897	Elongation	0	32.541	31	8	13	8	325	35.4	8.38	29.83	8	metabolic	mitochond	protein bir	Pf00627, P	10102	ENSG0000	TSFM	12			Mitochonc	4	1.163	0.974740108	34.6	3042.1	2629.3	44.81	5.31	High	High	High	High	High	High	1	
FALSE	High	Master Prc	P16615	Sarcoplasn	0	122.742	31	27	44	27	1042	114.7	5.34	100.56	27	cell organi	endoplasm	catalytic a	Pf00122, P	488	ENSG0000	ATP2A2	12	Dilated ca	Alzheimer	Ion homeo	30	1.162	0.002375739	1.79	13058.4	11213.1	1.36	2.72	High	High	High	High	High	High	1	

FALSE	High	Master Prc	P48729-3	Isoform 3	0	12.887	14	3	6	1	325	37.5	9.48	18.83	3	cell divisio	cytosol;me	catalytic a	Pf00069, P	1452	ENSG0000	CSNK1A1	5		T-Cell Rece		4	1.162	0.461629863	37.01	187.7	161.6	18.99	19.52	High	High	High	High	High	High	1	
FALSE	Medium	Master Prc	O60313	Dynamin-li	0.031	1.427	1	1	1	1	960	111.6	7.87	0	1	cell death;	cytosol;me	catalytic a	Pf00350, P	4976	ENSG0000	OPA1	3			Regulation	3	1.162	0.475078141	18.04	575.2	555.5	14.54	14.23	High	High	High	High	High	High	1	
FALSE	High	Master Prc	Q10713	Mitochonc	0	50.616	27	10	16	10	525	58.2	6.92	40.71	10	metabolic	membrane	catalytic a	Pf00675, P	23203	ENSG0000	PMPCA	9			Processing	5	1.161	0.03885018	1.06	3226.6	2758.5	2.52	7.16	High	High	High	High	High	High	1	
FALSE	High	Master Prc	O95302	Peptidyl-pi	0	19.021	10	5	10	5	570	63	5.08	17.32	5	metabolic	cytoplasm;	catalytic a	Pf00254, P	11328	ENSG0000	FKBP9	7			Associatio	4	1.16	0.069373663	2.74	3739.5	3417.8	5.27	3.6	High	High	High	High	High	High	1	
FALSE	High	Master Prc	Q9NVU7	Protein SD	0	5.826	1	1	2	1	687	79.8	9.25	4.91	1	cell organi	nucleus		Pf05285, P	55153	ENSG0000	SDAD1	4				0	1.16	0.184495811	15.35	786.5	678	6.55	11.42	High	High	High	High	High	High	1	
FALSE	High	Master Prc	Q16543	Hsp90 co-c	0	49.103	36	11	15	11	378	44.4	5.25	31.27	11	cell divisio	cytoplasm;	catalytic a	Pf03234, P	11140	ENSG0000	CDC37	19	PI3K-Akt si	TNF alpha	Downregu	17	1.16	0.364580773	25.37	2422.3	2263.6	14.86	9.99	High	High	High	High	High	High	1	
FALSE	High	Master Prc	O15155	BET1 hom	0	11.537	25	2	4	2	118	13.3	9.06	18.03	2	cell organi	endoplasm	protein bir	Pf05739	10282	ENSG0000	BET1	7	SNARE inte		COPI-medi	10	1.159	0.108007812	2.14	2596.3	2197.7	9.75	3.02	High	High	High	High	High	High	1	
FALSE	High	Master Prc	P09110-1	3-ketoacyl	0	9.757	8	2	2	2	424	44.3	8.44	3.95	2	metabolic	extracellul	catalytic a	Pf00108, P	30	ENSG0000	ACAA1	3	Metabolic	PPAR Alph	Neutrophil	21	1.159	0.185599584	16.36	268.3	259.3	16.6	8.47	High	High	High	High	High	High	1	
FALSE	High	Master Prc	Q16778	Histone H2	0	108.838	75	15	93	3	126	13.9	10.32	246.61	15	cell organi	chromosor	DNA bindi	Pf00125, P	8349	ENSG0000	HIST2H2BE	1	Systemic lu		SIRT1 nega	75	1.159	0.273730141	23.34	4805.4	3988.7	28.33	13.54	High	High	High	High	High	High	1	
FALSE	High	Master Prc	P19013	Keratin, ty	0	22.171	9	5	21	1	534	57.3	6.61	36.48	5	cell death;	cell surface	protein bir	Pf00038, P	3851		KRT4	12			Formation	3	1.159	0.611263343	19.51	780.3	673.4	12.75	10.73	High	High	High	High	High	High	1	[Q174;
FALSE	High	Master Prc	P00414	Cytochrom	0	5.001	5	1	2	1	261	29.9	7.31	6.4	1	cell organi	membrane	catalytic a	Pf00510	4514	ENSG0000	COX3	MT	Non-alcoh	Electron Tı	TP53 Regu	17	1.157	0.142118138	9.03	1218.4	965.9	12.85	18.46	High	High	High	High	High	High	1	
FALSE	High	Master Prc	Q9BSE4	Homocyste	0.001	4.535	2	1	2	1	406	45.1	5.06	4.26	1	response t	membrane	protein bir	Pf00240, P	64224	ENSG0000	HERPUD2	7				0	1.157	0.257311233	20.52	1165.2	1041.4	13.06	7.62	High	High	High	High	High	High	1	
FALSE	High	Master Prc	P56199	Integrin al	0	21.449	5	5	7	5	1179	130.8	6.29	12.64	5	cell organi	cell surface	metal ion l	Pf00092, P	3672	ENSG0000	ITGA1	5	PI3K-Akt si	Integrin-m	CHL1 inter	27	1.157	0.652271323	6.25	1038.3	997.2	9.19	15.78	High	High	High	High	High	High	1	
FALSE	High	Master Prc	P11310-1	medium-ch	0	64.513	37	12	17	12	421	46.6	8.37	34.14	12	metabolic	membrane	catalytic a	Pf00441, P	34	ENSG0000	ACADM	1	PPAR signa	Fatty Acid	PPARA act	24	1.156	0.033815144	5.35	2816.9	2496.1	5.69	5.39	High	High	High	High	High	High	1	
FALSE	High	Master Prc	Q96ER9	Coiled-coil	0	12.151	8	2	3	2	411	45.8	8.19	7.49	2		membrane			79714	ENSG0000	CCDC51	3				0	1.156	0.264174948	5.53	784.4	672.3	2.63	11.17	High	High	High	High	High	High	1	
FALSE	High	Master Prc	Q9UGI8	Testin [OS	0	10.424	10	4	5	4	421	48	7.68	6.1	4	regulation	cytoplasm;	metal ion l	Pf00412, P	26136	ENSG0000	TES	7				0	1.156	0.369009055	27.14	769.5	665.5	20.16	19.88	High	High	High	High	High	High	1	
FALSE	High	Master Prc	P43304	Glycerol-3-	0	5.373	4	3	5	3	727	80.8	7.69	3.97	3	metabolic	membrane	catalytic a	Pf00036, P	2820	ENSG0000	GPD2	2	Glyceroph	Fatty Acid	Triglycerid	9	1.156	0.38985886	25.38	800.8	732.7	33.02	15.12	High	High	High	High	High	High	1	
FALSE	High	Master Prc	O95391	Pre-mRNA	0	20.105	11	6	8	6	586	68.3	7.14	9.78	6	cell organi	cytoplasm;	catalytic a	Pf11708	10569	ENSG0000	SLU7	5	Spliceoson		Cleavage o	12	1.156	0.485375652	37.91	300.6	286.6	24.37	12.1	High	High	High	High	High	High	1	
FALSE	High	Master Prc	O43674-1	NADH deh	0	9.736	15	2	2	2	189	21.7	9.63	8.17	2	cell organi	membrane	catalytic a	Pf09781	4711	ENSG0000	NDUFB5	3	Huntingtoı	Electron Tı	Complex I	13	1.156	0.77756523	19.55	1070.7	926.3	14.43	13.21	High	High	High	High	High	High	1	
FALSE	High	Master Prc	Q9UI42-1	Carboxype	0	16.626	5	1	3	1	421	47.3	6.7	14.1	1	cell organi	extracellul	catalytic a	Pf00246, P	51200	ENSG0000	CPA4	7				0	1.155	0.558853845	25.93	830.2	795.4	21.7	8.21	High	High	High	High	High	High	1	
FALSE	High	Master Prc	Q96G46	tRNA-dihy	0	4.687	3	1	1	1	650	72.5	8.05	4.05	1	metabolic		catalytic a	Pf01207	56931	ENSG0000	DUS3L	19				0	1.155	0.596346582	52.08	143.2	138.2	35.72	16.17	High	High	High	High	High	High	1	
FALSE	High	Master Prc	Q9Y5Y2	Cytosolic F	0	14.984	19	2	3	2	271	28.8	5.83	12.41	2	cell organi	cytoplasm;	metal ion l	Pf00142, P	10101	ENSG0000	NUBP2	16			Cytosolic ii	2	1.155	0.71815539	42.41	28.2	36.4	57.96	36.6	High	High	High	High	High	High	1	
FALSE	High	Master Prc	Q9Y2W1	Thyroid ho	0	102.986	23	20	45	20	955	108.6	10.15	96.79	20	metabolic	nucleus	DNA bindi	Pf15440	9967	ENSG0000	THRAP3	1		Interactom	Transcripti	7	1.154	0.059774675	6.82	18923.1	16393.4	9.82	4.74	High	High	High	High	High	High	1	
FALSE	High	Master Prc	P54725-1	UV excisio	0	14.618	13	3	6	2	363	39.6	4.58	10.99	3	metabolic	cytoplasm;	DNA bindi	Pf00240, P	5886	ENSG0000	RAD23A	19	Nucleotide		Josephin d	11	1.154	0.255127776	18.26	588.8	498.3	8.78	13.89	High	High	High	High	High	High	1	
FALSE	High	Master Prc	O15230	Laminin su	0	29.047	3	7	9	7	3695	399.5	7.02	16.17	7	cell differe	extracellul	catalytic a	Pf00052, P	3911	ENSG0000	LAMA5	20	Toxoplasm	Alpha 6 Be	ECM prote	27	1.154	0.367820838	28.56	2358.9	2255.9	24.84	6.7	High	High	High	High	High	High	1	
FALSE	High	Master Prc	Q8NFQ8	Torsin-1A-	0	9.216	6	3	3	2	470	51.2	4.96	6.2	3	cell organi	endoplasm	enzyme re	Pf05609	163590	ENSG0000	TOR1AIP2	1				0	1.154	0.43123173	32.08	642.6	492.8	21.75	22.66	High	High	High	High	High	High	1	
FALSE	High	Master Prc	Q9UM00-	Calcium lo	0	25.733	21	4	9	4	188	21.2	9.74	27.44	4	cellular ho	endoplasm	transporte	Pf01956	54499	ENSG0000	TMCO1	1				0	1.154	0.483998648	35.51	2537.7	2318.1	17.43	12.67	High	High	High	High	High	High	1	
FALSE	High	Master Prc	Q92905	COP9 signa	0	7.085	6	1	1	1	334	37.6	6.54	4.92	1	cell organi	chromosor	catalytic a	Pf01398, P	10987	ENSG0000	COPS5	8		TGF-beta S	Cargo reco	14	1.154	0.683149423	27.82	369.3	317.3	25.67	4.89	High	High	High	High	High	High	1	
FALSE	Low	Master Prc	Q8NGC2	Olfactory r	0.057	1.136	2	1	1	1	313	35.4	7.97	0	1	regulation	membrane	receptor a	Pf00001, P	26686		OR4E2	14			Olfactory S	4	1.154	0.758196393	28.53	84.6	73.3	12.24	20.05	High	High	High	High	High	High	1	
FALSE	High	Master Prc	P39687	Acidic leuc	0	42.35	29	8	17	6	249	28.6	4.09	37.53	8	cell organi	cytoplasm;	protein bir	Pf12799, P	8125	ENSG0000	ANP32A	15			HuR (ELAV	3	1.153	0.041936887	3.07	1512.3	1285.1	2.55	6.37	High	High	High	High	High	High	1	
FALSE	High	Master Prc	Q9BTL3	RNMT-acti	0	9.545	19	1	2	1	118	14.4	8.94	9.02	1	cell organi	nucleus	catalytic a	Pf15320	83640	ENSG0000	FAM103A1	15				0	1.153	0.065575055	3.65	111.5	94	13.24	8.84	High	High	High	High	High	High	1	
FALSE	High	Master Prc	Q96BN8	Ubiquitin t	0	24.865	24	5	5	5	352	40.2	5.47	12.79	5	defense re	cytoplasm;	catalytic a	Pf10275	90268	ENSG0000	FAM105B;	5			Regulation	8	1.153	0.158752307	11.54	1637.8	1538.9	10.62	4.69	High	High	High	High	High	High	1	
FALSE	High	Master Prc	O75380	NADH deh	0	31.079	48	4	8	4	124	13.7	8.28	29.53	4	cell organi	membrane	catalytic a	Pf10276	4726	ENSG0000	NDUFS6	5	Metabolic	Electron Tı	Complex I	13	1.153	0.458883992	3.12	1069	934.6	13.63	0.53	High	High	High	High	High	High	1	
FALSE	High	Master Prc	Q96EB1-1	Elongator	0.002	3.818	2	1	1	1	424	46.6	8.51	2.9	1	cell organi	cytoplasm;	catalytic a	Pf05625	26610	ENSG0000	ELP4	11		Mesoderm	HATs acety	4	1.153	0.480092937	41.56	462.2	477.7	42.46	12.84	High	High	High	High	High	High	1	
FALSE	Low	Master Prc	Q6UWP8-	Suprabasir	0.069	1.052	4	1	1	1	590	60.5	7.01	0	1		extracellul			374897	ENSG0000	SBSN	19				0	1.153	0.863093962	65.08	280.4	243.1	27.38	22.76	High	High	High	High	High	High	1	[K349]
FALSE	High	Master Prc	Q8NBJ7-1	Sulfatase-r	0	28.325	23	5	10	5	301	33.8	8	29.6	5	metabolic	endoplasm	metal ion l	Pf03781	25870		SUMF2	7			The activa	8	1.152	0.01088111	4.3	2163.9	1878.9	1.29	4.17	High	High	High	High	High	High	1	
FALSE	High	Master Prc	Q13232	Nucleoside	0	7.709	17	2	2	2	169	19	7.84	7.73	2	cell death;	cytosol;mi	catalytic a	Pf00334	4832	ENSG0000	NME3	16	Metabolic		Interconve	6	1.152	0.140927318	0.49	807.2	756.1	5.37	5.35	High	High	High	High	High	High	1	
FALSE	High	Master Prc	Q7L7L0	Histone H2	0	51.135	41	7	46	2	130	14.1	11.05	104.8	7	cell organi	chromosor	DNA bindi	Pf00125, P	92815	ENSG0000	HIST3H2A	1	Alcoholism		HDACs dea	13	1.152	0.569936789	39.36	3086.8	3095.1	23.79	15.51	High	High	High	High	High	High	1	
FALSE	High	Master Prc	Q86SF2	N-acetylga	0	5.518	5	3	3	3	657	75.3	7.11	5.08	3	metabolic	Golgi;mem	catalytic a	Pf00535, P	51809	ENSG0000	GALNT7	4	Mucin type		O-linked gl	6	1.151	0.102352559	6.07	715	639.8	11.45	5.16	High	High	High	High	High	High	1	
FALSE	High	Master Prc	Q96SU4	Oxysterol-l	0	9.296	3	1	2	1	736	83.1	6.18	9.9	1	metabolic	cytosol;en	transporte	Pf00169, P	114883	ENSG0000	OSBPL9	1			Synthesis c	5	1.151	0.221647091	13.03	773.7	721.2	14.8	6.08	High	High	High	High	High	High	1	
FALSE	High	Master Prc	P07954	fumarate h	0	153.479	54	21	78	20	510	54.6	8.76	196.8	21	metabolic	cytoplasm;	catalytic a	Pf00206, P	2271	ENSG0000	FH	1	Pathways i	TCA Cycle	Citric acid	13	1.151	0.323142828	11.31	18495.8	16899.6	9.43	6.55	High	High	High	High	High	High	1	
FALSE	High	Master Prc	Q9UNP9-1	Peptidyl-pi	0	39.44	22	6	10	6	301	33.4	5.6	21.68	6	metabolic	cytosol;ext	catalytic a	Pf00076, P	10450	ENSG0000	PPIE	1	Spliceoson		Dual incisi	14	1.151	0.379840144	26.19	3629.2	3120.2	30.01	16.1	High	High	High	High	High	High	1	[K218]
FALSE	High	Master Prc	Q6ICB0	Desumoyla	0	4.655	5	1	2	1	168	18.3	4.94	4.67	1	metabolic	cytoplasm;	catalytic a	Pf05903	27351	ENSG0000	DESI1	22				0	1.151	0.542204315	3.97	721	616.4	18.12	2.62	High	High	High	High	High	High	1	
FALSE	High	Master Prc	P00505	Aspartate	0	129.496	58	21	53	21	430	47.5	9.01	112.17	21	metabolic	cell surface	catalytic a	Pf00155	2806	ENSG0000	GOT2	16	Alanine, as	Alanine an	Gluconeog	22	1.151	0.54590699	13.15	13486.8	12389.1	18.15	3.4	High	High	High	High	High	High	1	
FALSE	High	Master Prc	P00387	NADH-cytc	0	64.651	34	9	19	9	301	34.2	7.59	56.01	9	metabolic	cytoplasm;	catalytic a	Pf00175, P	1727	ENSG0000	CYB5R3	22	Amino sug	Oxidation	Vitamin C	11	1.15	0.08164395	2.34	5970.2	5298.7	1.18	5.82	High	High	High	High	High	High	1	
FALSE	High	Master Prc	O95562	Vesicle tra	0.002	4.053	6	1	3	1	160	17.8	9.13	7.66	1	transport	membrane		Pf04178	375035	ENSG0000	SFT2D2	1		TYROBP Ca		1	1.15	0.08526775	5.38	1964.9	1618	5.95	6.07	High	High	High	High	High	High	1	
FALSE	High	Master Prc	Q8IY67	Ribonuclec	0	23.379	12	3	3	3	606	63.8	8.48	11.22	3	metabolic	cytoplasm;	protein bir	Pf00076, P	125950	ENSG0000	RAVER1	19				0	1.15	0.168621816	9.88	124.1	106	5.85	12.91	High	High	High	High	High	High	1	
FALSE	High	Master Prc	P26572	alpha-1,3-	0	9.366	6	2	3	2	445	50.8	9.16	6.67	2	metabolic	Golgi;mem	catalytic a	Pf03071	4245	ENSG0000	MGAT1	5	Metabolic		N-glycan tı	7	1.15	0.302909278	23.75	452.6	393.6	4.65	12.99	High	High	High	High	High	High	1	
FALSE	High	Master Prc	P35244	Replicatior	0	20.293	47	4	5	4	121	13.6	5.08	17.82	4	cell organi	nucleus	DNA bindi	Pf08661	6119	ENSG0000	RPA3	7	Mismatch	Retinoblas	Translesio	79	1.15	0.315592974	19.92	1655.1	1487.7	25.26	6.09	High	High	High	High	High	High	1	
FALSE	High	Master Prc	Q9NZ08-1	endoplasm	0	12.644	6	5	7	5	941	107.2	6.46	13.61	5	cell commı	cytoplasm;	catalytic a	Pf01433, P	51752	ENSG0000	ERAP1	5			Antigen Pr	4	1.15	0.32567139	16.18	128.7	101.8	30.37	13.43	High	High	High	High	High	High	1	
FALSE	High	Master Prc	Q9NVT9	armadillo r	0	13.101	15	3	3	3	282	31.3	5.74	5.73	3	transport	cytoplasm;	metal ion l	Pf00514	55156	ENSG0000	ARMC1	8				0	1.15	0.989699234	16.2	380.1	337.5	30.15	5.86	High	High	High	High	High	High	1	
FALSE	High	Master Prc	P04843	Dolichyl-di	0	166.06	55	27	61	27	607	68.5	6.38	165.34	27	metabolic	cytosol;en	catalytic a	Pf04597	6184	ENSG0000	RPN1	3	Metabolic	Proteasom	Asparagine	9	1.149	0.00410274	2.74	23542.6	20090.7	4.76	6.14	High	High	High	High	High	High	1	
FALSE	High	Master Prc	Q15293	Reticulocal	0	63.407	45	10	18	10	331	38.9	5	61.29	10	metabolic	endoplasm	metal ion l	Pf13499	5954	ENSG0000	RCN1	11			Post-transl	4	1.149	0.064969341	8.85	4917.5	4279.6	1.98	5.26	High	High	High	High	High	High	1	
FALSE	High	Master Prc	Q8IUR7-1	Armadillo	0.003	3.168	1	1	1	1	673	75.5	6.73	2.72	1	transport	extracellul	protein bir	Pf01602	25852	ENSG0000	ARMC8	3			Neutrophil	3	1.149	0.234478055	19.76	657.1	572	7.12	7.08	High	High	High	High	High	High	1	
FALSE	High	Master Prc	Q68CQ7-1	Glycosyltra	0	22.493	15	4	4	4	371	41.9	9.35	10.48	4	metabolic	Golgi;mem	catalytic a	Pf01501	55830	ENSG0000	GLT8D1	3				0	1.149	0.261597001	21.12	961	836.2	5.86	10.33	High	High	High	High	High	High	1	
FALSE	High	Master Prc	Q8N5N7	39S riboso	0.009	2.134	7	1	1	1	158	18.3	7.88	1.82	1	cell organi	membrane		Pf10501	54534	ENSG0000	MRPL50	9			Mitochonc	6	1.149	0.322518809	20.41	398.2	384.9	29.18	6.07	High	High	High	High	High	High	1	
FALSE	High	Master Prc	Q96QC0	Serine/thre	0	5.168	3	2	2	2	940	99	9.17	3.06	2	metabolic	chromosor	DNA bindi	Pf00642, P	5514	ENSG0000	PPP1R10	6; CHR_HS				0	1.149	0.492860656	28.45	85.7	74.6	11.2	12.02	High	High	High	High	High	High	1	
FALSE	Low	Master Prc	Q6ZN03	Putative ui	0.06	1.117	7	1	1	1	302	32.4	8.68	0	1					10012669		LINC00322	21				0	1.148	0.093747378	6.96	624.6	549.5	12.95	14.27	High	High	High	High	High	High	1	[Q151]
FALSE	High	Master Prc	Q13952-1	Nuclear tra	0	6.673	4	2	2	2	458	50.3	6.1	2.34	2	metabolic	nucleus	DNA bindi	Pf00125, P	4802	ENSG0000	NFYC	1	Antigen pr		ATF4 activ	15	1.148	0.181061626	5.52	661.4	560.9	11.98	7.87	High	High	High	High	High	High	1	
FALSE	High	Master Prc	Q5T200-1	Zinc finger	0	8.013	1	2	2	2	1668	196.5	9.42	1.85	2		nucleus	metal ion l	Pf00642	23091	ENSG0000	ZC3H13	13				0	1.148	0.476726005	44.81	119.3	111.3	48.02	5.69	High	High	High	High	High	High	1	
FALSE	High	Master Prc	P26440	Isovaleryl-	0	39.864	22	7	13	7	423	46.3	8.19	38.04	7	metabolic	mitochond	catalytic a	Pf00441, P	3712		IVD	15	Metabolic	Valproic ac	Branched-	6	1.148	0.86106111	10.65	7358.4	6655.4	27.39	12.62	High	High	High	High	High	High	1	
FALSE	High	Master Prc	P61006	Ras-relate	0	18.201	22	5	7	2	207	23.7	9.07	8.19	5	cell organi	cytoplasm;	catalytic a	Pf00025, P	4218	ENSG0000	RAB8A	19	Endocytosi	Focal Adhe	Translocat	23	1.147	0.114179133	4.71	1967.7	1607.5	5.91	9.93	High	High	High	High	High	High	1	
FALSE	High	Master Prc	Q13620-1	Isoform 2	0	20.379	8	5	7	4	895	102.2	7.94	13.19	5	cell organi	cytosol	catalytic a	Pf00888, P	8450	ENSG0000	CUL4B	X		Mesoderm		1	1.147	0.142663643	12.96	1092.7	952.4	7.92	2.81	High	High	High	High	High	High	1	
FALSE	High	Master Prc	Q9NTZ6	RNA-bindi	0	42.585	12	10	15	10	932	97.3	8.63	30.4	10		nucleus	protein bir	Pf00076, P	10137	ENSG0000	RBM12	20				0	1.147	0.193514868	11.78	3674.8	3203.8	4.93	5.73	High	High	High	High	High	High	1	
FALSE	High	Master Prc	P23634-1	Plasma me	0	14.455	3	3	6	2	1241	137.8	6.6	10.83	3	cellular co	membrane	catalytic a	Pf00122, P	493		ATP2B4	1	Pancreatic		Ion homeo	16	1.147	0.318920523	6.5	606.4	632.2	20.03	14.77	High	High	High	High	High	High	1	
FALSE	High	Master Prc	Q16698	2,4-dienoy	0	20.787	22	5	6	5	335	36	9.28	11.81	5	cell organi	cytosol;mi	catalytic a	Pf00106, P	1666	ENSG0000	DECR1	8		Fatty Acid	mitochond	7	1.146	0.007289959	2.06	314.5	268.6	3.77	2.65	High	High	High	High	High	High	1	
FALSE	High	Master Prc	P53041	Serine/thre	0	52.577	21	8	14	8	499	56.8	6.28	39.62	8	cell organi	cytoplasm;	catalytic a	Pf00149, P	5536	ENSG0000	PPP5C	19	MAPK sign	MAPK Sigr	Recruitme	16	1.146	0.180335335	4.19	2127.6	1857.3	11.25	3.02	High	High	High	High	High	High	1	
FALSE	High	Master Prc	Q9NUL7	Probable A	0.003	3.174	2	1	2	1	540	59.5	10.42	4.36	1	cell organi	cytosol;mi	catalytic a	Pf00270, P	55794	ENSG0000	DDX28	16				0	1.146	0.264394766	20.62	780.9	676.2	9.08	10.29	High	High	High	High	High	High	1	
FALSE	High	Master Prc	Q7Z7K0	COX assem	0.001	4.326	9	1	2	1	106	12.5	8.63	5.4	1		mitochond	metal ion l	Pf08583	152100	ENSG0000	CMC1	3				0	1.146	0.4082588	30.1	389.9	360.5	42.87	9.03	High	High	High	High	High	High	1	
FALSE	High	Master Prc	P46013	Proliferatic	0	18.088	3	4	5	4	3256	358.5	9.45	10.32	4	cell prolife	chromosor	DNA bindi	Pf00498, P	4288	ENSG0000	MKI67	10		Primary Fc		1	1.146	0.458013521	37.19	478.8	417.8	29.88	19.77	High	High	High	High	High	High	1	
FALSE	High	Master Prc	P63027	Vesicle-ass	0	32.792	34	3	4	1	116	12.7	8.13	15.73	3	cell organi	cytosol;me	protein bir	Pf00957	6844	ENSG0000	VAMP2	17	Salivary se	Synaptic V	Cargo reco	45	1.146	0.629890223	20.08	121.1	105.7	13.1	9.68	High	High	High	High	High	High	1	
FALSE	High	Master Prc	O75964	ATP syntha	0	27.689	60	7	14	7	103	11.4	9.64	27.11	7	cell organi	membrane	catalytic a	Pf04718	10632	ENSG0000	ATP5L	11	Metabolic	Electron Tı	Cristae for	11	1.146	0.861114516	1.63	2187.7	2004.1	17.31	4.9	High	High	High	High	High	High	1	
FALSE	High	Master Prc	Q99714-1	3-hydroxya	0	173.264	80	15	83	15	261	26.9	7.78	334.95	15	cell organi	cytoplasm;	catalytic a	Pf00106, P	3028	ENSG0000	HSD17B10	X	Metabolic	Alzheimer:	tRNA proc	15	1.146	0.966356826	20.1	20151.5	17588.6	21.48	12.84	High	High	High	High	High	High	1	
FALSE	High	Master Prc	P11498	pyruvate c	0	155.913	31	28	51	28	1178	129.6	6.84	129.63	28	metabolic	cytoplasm;	catalytic a	Pf00289, P	5091	ENSG0000	PC	11	Pyruvate n	Alanine an	Gluconeog	22	1.145	0.041355663	0.96	18888.3	16501.3	5.1	9.21	High	High	High	High	High	High	1	
FALSE	High	Master Prc	Q15233	Non-POU	0	127.034	43	23	69	20	471	54.2	8.95	132.03	23	defense re	membrane	DNA bindi	Pf00076, P	4841	ENSG0000	NONO	X		mRNA Pro		1	1.145	0.057268349	2.23	22421	18612.1	4.39	5.04	High	High	High	High	High	High	1	
FALSE	Medium	Master Prc	Q12974	Protein tyr	0.014	1.829	4	1	1	1	167	19.1	8.37	1.9	1	metabolic	cytoplasm;	catalytic a	Pf00102, P	8073	ENSG0000	PTP4A2	1			RAB geran	3	1.145	0.094633083	7.11	761.6	683.7	5.66	11.36	High	High	High	High	High	High	1	
FALSE	High	Master Prc	Q14203	Dynactin s	0	36.713	9	10	14	10	1278	141.6	5.81	26.49	10	cell divisio	chromosor	catalytic a	Pf00261, P	1639	ENSG0000	DCTN1	2	Vasopressi		COPI-indep	22	1.145	0.45250433	39.35	3406.1	2973.5	46.03	13.04	High	High	High	High	High	High	1	
FALSE	High	Master Prc	Q15067	Peroxisom	0	11.595	5	2	2	2	660	74.4	8.16	4.39	2	cell organi	cytoplasm;	catalytic a	Pf01756, P	51	ENSG0000	ACOX1	17	Peroxisom	Estrogen R	PPARA act	14	1.145	0.942792389	63.85	283.4	247.5	26.86	32.2	High	High	High	High	High	High	1	
FALSE	High	Master Prc	Q16540	39S riboso	0	9.916	35	4	5	4	153	17.8	9.69	4.32	4	cell organi	membrane	protein bir	Pf00276	6150	ENSG0000	MRPL23	11	Ribosome		Mitochonc	7	1.144	0.005882216	1.53	1159	1043.7	7.1	8.31	High	High	High	High	High	High	1	

FALSE	High	Master Prc	Q9UKX7	Nuclear pc	0	28.637	26	6	6	6	468	50.1	7.06	16.9	6	cell organi	membrane	protein bir	Pf00638, F	10762	ENSG0000	NUP50	22	RNA transp		Rev-media	64	1.144		0.010513968	3.21	1377.7	1204	1.12	3.79	High	High	High	High	High	High	1	
FALSE	Medium	Master Prc	O75368	SH3 domai	0.011	2.039	6	1	2	1	114	12.8	5.25	2.41	1	cellular ho	cytoplasm;	catalytic a	Pf00462, F	6451	ENSG0000	SH3BGRL	X				0	1.144		0.221077805	11.39	1251.7	1087.2	2.63	9.61	High	High	High	High	High	High	1	
FALSE	High	Master Prc	Q9BV20-1	methylthic	0	24.053	20	6	9	6	369	39.1	6.3	15.22	6	metabolic	cytoplasm;	catalytic a	Pf01008	84245	ENSG0000	MRI1	19	Cysteine a		Methionin	7	1.144		0.476248931	6.99	2102.8	1891.3	7.32	8.36	High	High	High	High	High	High	1	
FALSE	High	Master Prc	P0DMU9	Cancer/tes	0	5.189	14	2	2	2	189	21.1	9.55	4.63	2				Pf15300	10272363	ENSG0000	LOC10272	X				0	1.144		0.479371861	67.09	308.3	269.6	100.01	26	High	High	High	High	High	High	1	
FALSE	High	Master Prc	Q9Y6Y8	SEC23-inte	0	13.186	5	4	4	4	1000	111	5.54	8.02	4	cell organi	cytoplasm;	catalytic a	Pf00536, F	11196	ENSG0000	SEC23IP	10			COPII (Coa	8	1.144		0.575400976	13.76	55.7	53.2	13.43	9.96	High	High	High	High	High	High	1	
FALSE	High	Master Prc	Q6NUQ4	Transmem	0	9.655	5	3	4	3	689	77.1	9.14	7.12	3	cell death	cytosol;en		Pf10151	54867	ENSG0000	TMEM214	2				0	1.144		0.5989603	35.54	110.1	86.8	34.67	9.96	High	High	High	High	High	High	1	
FALSE	High	Master Prc	Q99747	Gamma-so	0	7.688	6	2	2	2	312	34.7	5.41	2.9	2	cell organi	membrane	protein bir	Pf14938	8774	ENSG0000	NAPG	18			COPI-depe	14	1.143		0.317982299	21.51	1001.8	771.3	8.8	21.77	High	High	High	High	High	High	1	
FALSE	High	Master Prc	P46937-1	Transcripti	0	83.022	51	12	20	12	504	54.4	5.17	75.18	12	cell organi	cytoplasm;	DNA bindi	Pf00397	10413	ENSG0000	YAP1	11	Hippo sign	Mesoderm	RUNX2 reg	26	1.143		0.365434927	24.15	3041.1	2719.7	32.52	4.45	High	High	High	High	High	High	1	
FALSE	High	Master Prc	P50895	Basal cell a	0	14.239	5	2	3	2	628	67.4	5.81	9.04	2	regulation	cell surface	protein bir	Pf00047, F	4059	ENSG0000	BCAM	19				0	1.142		0.164897132	0.78	416.5	396.5	9.46	7.05	High	High	High	High	High	High	1	
FALSE	High	Master Prc	Q9BUT9-1	MAPK regu	0	12.37	23	2	2	2	160	17.8	9.41	6.76	2		cytoplasm;	protein bir	Pf14799	84331	ENSG0000	FAM195A;	16				0	1.142		0.196153811	14.63	992.9	863.8	8.72	6.48	High	High	High	High	High	High	1	
FALSE	High	Master Prc	Q12849	G-rich sequ	0	25.285	14	5	7	5	480	53.1	6.19	17.01	5	metabolic	cytoplasm;	RNA bindi	Pf00076, F	2926	ENSG0000	GRSF1	4			Viral mRN/	6	1.142		0.387704203	21.47	521.2	488.4	57.57	8.1	High	High	High	High	High	High	1	
FALSE	High	Master Prc	Q9P015	39S riboso	0	7.911	9	2	3	2	296	33.4	10.01	7.14	2	cell organi	membrane	protein bir	Pf00828	29088	ENSG0000	MRPL15	8	Ribosome		Mitochonc	7	1.142		0.392877577	23.99	2602.8	2189	4.92	21.57	High	High	High	High	High	High	1	
FALSE	High	Master Prc	Q9UL25	Ras-relatec	0	46.623	33	8	13	8	225	24.3	7.94	35.42	8	cellular co	cytosol;en	catalytic a	Pf00025, F	23011	ENSG0000	RAB21	12			RAB GEFs	7	1.142		0.445730895	1.97	4684.7	4100.5	8.94	3.54	High	High	High	High	High	High	1	
FALSE	High	Master Prc	P47985	cytochrom	0	26.831	40	6	7	6	274	29.6	8.32	24.34	6	metabolic	membrane	catalytic a	Pf00355, F	7386	ENSG0000	UQCRFS1	19	Non-alcoh	Electron Tr	Respirator	12	1.141		0.028130735	4.16	712.8	643	5.77	3.64	High	High	High	High	High	High	1	
FALSE	High	Master Prc	Q96MU7-1	YTH domai	0	6.842	2	1	2	1	727	84.6	6.23	2.16	1	cell organi	membrane	protein bir	Pf04146	91746	ENSG0000	YTHDC1	4; CHR_HS				0	1.141		0.305111629	20.38	283.5	248.4	18.1	5.26	High	High	High	High	High	High	1	
FALSE	High	Master Prc	Q9Y6M9	NADH deh	0	14.645	21	3	8	3	179	21.8	8.38	25.8	3	cell organi	membrane	catalytic a	Pf05347, F	4715	ENSG0000	NDUFB9	8	Oxidative	Electron Tr	Complex I	13	1.141		0.408796928	27.53	1989.1	1746	64.07	10.44	High	High	High	High	High	High	1	
FALSE	High	Master Prc	P62857	40S riboso	0	20.761	52	4	10	4	69	7.8	10.7	10.52	4	cell organi	cytosol;rib	protein bir	Pf01200	6234	ENSG0000	RPS28	19	Ribosome	Cytoplasm	Peptide ch	38	1.141		0.696075754	57.69	684.6	599.8	21.66	25.81	High	High	High	High	High	High	1	
FALSE	High	Master Prc	Q15005	Signal pept	0	14.348	22	3	5	3	226	25	8.47	8.01	3	metabolic	endoplasm	catalytic a	Pf06703	9789	ENSG0000	SPCS2	11	Protein exp		Synthesis,	9	1.14		0.011858845	2.32	872.5	786.5	5.69	5.39	High	High	High	High	High	High	1	
FALSE	High	Master Prc	Q8NDI1	EH domain	0.001	4.474	1	2	3	1	1231	139.9	5.35	0	2	transport	cytoplasm;	protein bir	Pf00307, F	23301	ENSG0000	EHBP1	2				0	1.14		0.266034145	3.04	1129.1	1018.4	3.56	7.22	High	High	High	High	High	High	1	
FALSE	High	Master Prc	Q6P4A7-1	Sideroflexi	0.006	2.35	4	1	1	1	337	38	9.19	0	1	transport	membrane	transporte	Pf03820	119559	ENSG0000	SFXN4	10				0	1.14		0.33448386	22.73	265.1	215.8	13.21	17	High	High	High	High	High	High	1	
FALSE	High	Master Prc	Q92888-1	Rho guanir	0.007	2.235	1	1	1	1	912	102.4	5.66	3.02	1	cell prolife	cytoplasm;	enzyme re	Pf00621, F	9138	ENSG0000	ARHGEF1	19	Proteoglyc	G Protein S	G alpha (1.	20	1.14		0.616433299	26.51	199.9	175.4	7.42	16.36	High	High	High	High	High	High	1	
FALSE	High	Master Prc	O95197	Reticulon-3	0.003	3.137	1	1	1	1	1032	112.5	4.96	4.21	1	cell death;	endoplasm	protein bir	Pf02453, F	10313	ENSG0000	RTN3	11	Alzheimer'		Synaptic a	4	1.139		0.095932131	0.89	468.6	430.6	4.36	6.61	High	High	High	High	High	High	1	
FALSE	High	Master Prc	P84074	Neuron-sp	0	6.431	15	3	4	3	193	22.4	4.97	4.89	3	regulation	cytoplasm;	metal ion l	Pf00036, F	3208	ENSG0000	HPCA	1				0	1.139		0.131780002	2.85	54.1	45.9	13.34	5.93	High	High	High	High	High	High	1	
FALSE	High	Master Prc	Q9BW92	Threonine-	0	10.747	4	2	3	2	718	81	7.3	12.3	2	metabolic	cytoplasm;	catalytic a	Pf00587, F	80222	ENSG0000	TARS2	1	Aminoacyl		Mitochonc	5	1.139		0.213956923	13.23	933.2	825.9	7.09	9.02	High	High	High	High	High	High	1	
FALSE	High	Master Prc	O14732-1	inositol mc	0	13.831	7	2	4	2	288	31.3	6.61	9.28	2	metabolic	cytoplasm;	catalytic a	Pf00459	3613	ENSG0000	IMPA2	18	Inositol ph		Synthesis c	6	1.139		0.367458181	24.22	1503.1	1295.4	20.33	14.1	High	High	High	High	High	High	1	
FALSE	High	Master Prc	Q8NCH0	Carbohydr	0	7.246	6	2	2	2	376	43	9.48	3.95	2	metabolic	Golgi;mem	catalytic a	Pf03567	113189	ENSG0000	CHST14	15	Glycosami	Metapathw	Defective (	11	1.139		0.417459372	28.89	1161.5	1006.2	17.01	13.98	High	High	High	High	High	High	1	
FALSE	High	Master Prc	P13928	Annexin A8	0	66.385	36	12	26	1	327	36.9	5.78	64.73	12	cell organi	cytosol;me	metal ion l	Pf00191	653145; 7	ENSG0000	ANXA8; AN	10				0	1.139		0.517064524	40.42	754.4	673.2	28.96	17.05	High	High	High	High	High	High	1	
FALSE	High	Master Prc	P13995	Bifunctiona	0	47.119	42	9	13	9	350	37.9	8.73	35.38	9	metabolic	cytosol;mi	catalytic a	Pf00763, F	10797	ENSG0000	MTHFD2	2	Metabolic	Folate Met	Metabolisr	10	1.138		0.033767724	0.22	4164.3	3664.4	8.81	5.31	High	High	High	High	High	High	1	
FALSE	High	Master Prc	Q96EP5	DAZ-associ	0	38.947	21	5	13	5	407	43.4	8.56	36.58	5	cell differe	cytoplasm;	protein bir	Pf00076, F	26528	ENSG0000	DAZAP1	19	mRNA surv			1	1.138		0.042687576	3.52	2767.4	2492.9	6.44	7.15	High	High	High	High	High	High	1	
FALSE	High	Master Prc	O95071	E3 ubiquiti	0	20.001	3	5	5	5	2799	309.2	5.85	8.73	5	cell prolife	cytosol;me	catalytic a	Pf00632, F	51366	ENSG0000	UBR5	8	Ubiquitin r	Mesoderm		3	1.138		0.078493755	0.13	216.2	190.9	0.93	4.36	High	High	High	High	High	High	1	
FALSE	High	Master Prc	Q9H307	Pinin [OS=	0	41.365	18	10	13	10	717	81.6	7.14	33.32	10	metabolic	membrane	DNA bindi	Pf04696, F	5411		PNN	14	RNA transp			2	1.138		0.085374729	2.95	864.8	779.1	1.7	5.12	High	High	High	High	High	High	1	[R56]
FALSE	High	Master Prc	Q8TC07-1	TBC1 doma	0	6.054	1	1	2	1	691	79.4	5.67	4.03	1	regulation	cytoplasm;	enzyme re	Pf00566, F	64786	ENSG0000	TBC1D15	12			TBC/RABG	4	1.138		0.341863428	21.49	565.4	473.9	17.31	14.81	High	High	High	High	High	High	1	
FALSE	High	Master Prc	P05187	alkaline ph	0	229.369	58	26	83	9	535	57.9	6.29	208.04	26	metabolic	cell surface	catalytic a	Pf00245	250	ENSG0000	ALPP	2	Metabolic		Intra-Golgi	7	1.138		0.961203318	38.13	10939.4	9612.8	18.5	23.02	High	High	High	High	High	High	1	
FALSE	High	Master Prc	P18754-1	Regulator	0	12.272	12	4	5	4	421	44.9	7.52	6.88	4	cell divisio	chromosor	DNA bindi	Pf00415, F	1104	ENSG0000	RCC1	1			Rev-media	9	1.137		0.031922243	1.3	665	580.4	5.01	2.26	High	High	High	High	High	High	1	
FALSE	High	Master Prc	Q8IV08	Phospholip	0	11.307	10	3	4	3	490	54.7	6.47	5.18	3	metabolic	endoplasm	catalytic a	Pf00614, F	23646	ENSG0000	PLD3	19	Ether lipid		Synthesis c	12	1.137		0.102616291	4.35	753.3	662.2	2.02	8.72	High	High	High	High	High	High	1	
FALSE	High	Master Prc	Q9NRZ9	Lymphoid-	0.002	3.761	3	2	2	2	838	97	7.93	2.59	2	cell divisio	nucleus	catalytic a	Pf00176, F	3070	ENSG0000	HELLS	10		Apoptosis;		2	1.137		0.133148954	2.06	617.1	574.6	5.75	7.61	High	High	High	High	High	High	1	[K80]
FALSE	High	Master Prc	Q9H4L4	Sentrin-spe	0	5.318	3	1	2	1	574	65	8.56	4.09	1	metabolic	cytoplasm;	catalytic a	Pf02902	26168	ENSG0000	SENP3	17			Major path	4	1.137		0.433018223	13.3	167.4	147.3	20.34	5.13	High	High	High	High	High	High	1	
FALSE	High	Master Prc	Q5TC12-1	ATP syntha	0	5.843	7	2	3	2	328	36.4	7.96	6.94	2	cell organi	mitochond	protein bir	Pf06644	64756	ENSG0000	ATPAF1	1				0	1.137		0.592607885	3.87	608.8	574.3	9.05	8.16	High	High	High	High	High	High	1	
FALSE	High	Master Prc	O14949	Cytochrom	0	17.975	38	4	9	4	82	9.9	10.08	16.55	4	metabolic	membrane	catalytic a	Pf02939, F	27089	ENSG0000	UQCRQ	5	Non-alcoh	Electron Tr	Respirator	12	1.136		0.0741671	8.49	3340.1	3150.8	5.77	7.93	High	High	High	High	High	High	1	
FALSE	High	Master Prc	Q16204	coiled-coil	0	32.559	20	10	15	10	474	53.3	7.34	28.51	10	cell organi	cytoplasm;	protein bir	Pf04111, F	8030	ENSG0000	CCDC6	10	Pathways i	Retinoblas		4	1.136		0.255158932	18.25	1848.8	1657.7	11.03	8.28	High	High	High	High	High	High	1	
FALSE	High	Master Prc	Q9BRP8	Partner of	0	56.548	61	9	19	9	204	22.6	9.45	53.73	9	cell organi	cytoplasm;	protein bir	Pf09282	84305	ENSG0000	WIBG; PYM	12	RNA transp			2	1.136		0.279392236	17.09	4649.4	4072.5	13.54	9.28	High	High	High	High	High	High	1	
FALSE	High	Master Prc	P25705-1	ATP syntha	0	224.617	64	37	127	37	553	59.7	9.13	271.79	37	cell organi	membrane	catalytic a	Pf00006, F	498	ENSG0000	ATP5A1	18	Huntingtor	Electron Tr	Cristae for	16	1.136		0.384718264	9.41	32419.9	29070.6	7.52	6.63	High	High	High	High	High	High	1	
FALSE	High	Master Prc	Q9BQP7	mitochond	0	7.172	6	1	1	1	344	39.4	7.68	4.35	1	cell organi	mitochond	catalytic a		92667	ENSG0000	C20orf72;	20				0	1.135		0.062599409	1.82	315	297.5	10.39	8.31	High	High	High	High	High	High	1	
FALSE	High	Master Prc	Q96DB5	regulator c	0	10.677	12	3	3	3	314	35.8	8.5	5.63	3		cytoplasm;	protein bir		51115	ENSG0000	FAM82B; F	8				0	1.135		0.11692415	5.65	419.9	370.1	6.45	4.3	High	High	High	High	High	High	1	
FALSE	High	Master Prc	P37108	Signal reco	0	30.413	43	5	17	5	136	14.6	10.04	44.23	5	cell organi	cytoplasm;	protein bir	Pf02290	6727	ENSG0000	SRP14	15	Protein exp		Neutrophil	7	1.135		0.156996382	8.87	6189	5451.6	8.54	4.65	High	High	High	High	High	High	1	
FALSE	High	Master Prc	P84095	Rho-relate	0	52.532	42	7	20	6	191	21.3	8.12	64.45	7	cell organi	cytosol;me	catalytic a	Pf00025, F	391	ENSG0000	RHOG	11	Shigellosis	Brain-Deriv	G alpha (1.	23	1.135		0.223701596	10.33	2328	2026.5	17.11	7.49	High	High	High	High	High	High	1	
FALSE	High	Master Prc	Q9HCS7	Pre-mRNA	0	36.966	12	8	11	8	855	99.9	6.23	25.47	8	cell organi	membrane	protein bir	Pf13424	56949	ENSG0000	XAB2	19	Spliceosom		Dual incisi	11	1.135		0.343012303	17.54	2374.7	1992	30.09	10.74	High	High	High	High	High	High	1	
FALSE	High	Master Prc	Q9Y5L4	mitochond	0	5.202	12	1	1	1	95	10.5	8.18	4.52	1	cell organi	membrane	metal ion l	Pf02953	26517	ENSG0000	TIMM13	19			Mitochonc	2	1.135		0.350915428	25.54	639.4	550.3	12.01	7.99	High	High	High	High	High	High	1	
FALSE	High	Master Prc	Q9UKZ1	CCR4-NOT	0.007	2.294	2	1	1	1	510	55.2	6.4	0	1	cell prolife	cytoplasm;	protein bir	Pf10155	55571	ENSG0000	C2orf29; C	2			TP53 regul	9	1.135		0.369966692	25.4	487.8	429.7	9.8	13.74	High	High	High	High	High	High	1	
FALSE	High	Master Prc	Q8WW59	SPRY doma	0	6.988	14	3	3	3	207	23.1	6.93	4	3		mitochond	protein bir	Pf00622, F	283377		SPRYD4	12				0	1.135		0.419791626	28.49	1262.7	1152.1	20.5	13.12	High	High	High	High	High	High	1	
FALSE	High	Master Prc	P05204	Non-histor	0	42.48	54	8	20	8	90	9.4	9.99	30.72	8		chromosor	DNA bindi		3151	ENSG0000	HMGN2	1				0	1.134		0.268363909	7.53	8346.8	6989.9	15.39	19.38	High	High	High	High	High	High	1	
FALSE	High	Master Prc	O75347	Tubulin-sp	0	65.869	71	12	24	12	108	12.8	5.29	51.19	12	cell organi	cytoplasm;	protein bir	Pf02970	6902	ENSG0000	TBCA	5			Post-chape	3	1.134		0.393451806	13.7	9388.9	8064.3	1.83	14.53	High	High	High	High	High	High	1	
FALSE	High	Master Prc	Q96KG9	N-terminal	0	8.422	4	2	4	2	808	89.6	6.3	12.89	2	metabolic	cytoplasm;	catalytic a	Pf00069, F	57410	ENSG0000	SCYL1	11				0	1.134		0.465123605	35.72	1051.6	927.2	27.82	8.01	High	High	High	High	High	High	1	
FALSE	High	Master Prc	Q12931	heat shock	0	151.453	42	27	75	26	704	80.1	8.21	167.67	27	metabolic	membrane	nucleotide	Pf00183, F	10131	ENSG0000	TRAP1	16		TNF alpha	Respirator	6	1.133		0.047904499	5.31	20545	18811.3	8.6	5.79	High	High	High	High	High	High	1	
FALSE	High	Master Prc	P67812	Signal pept	0	5.721	14	3	4	3	179	20.6	9.48	4.54	3	metabolic	endoplasm	catalytic a	Pf00717	23478	ENSG0000	SEC11A	15	Protein exp		Synthesis,	9	1.133		0.189200724	14.26	135.9	121.8	11.37	9.88	High	High	High	High	High	High	1	
FALSE	High	Master Prc	Q9UHQ4	B-cell rece	0	4.731	4	1	2	1	241	28.3	9.54	3.82	1	cell death;	endoplasm		Pf04111, F	55973	ENSG0000	BCAP29	7; CHR_HG				0	1.133		0.338227274	20.02	716.3	655.7	29.38	2.95	High	High	High	High	High	High	1	
FALSE	High	Master Prc	P30049	ATP syntha	0	32.653	38	3	14	3	168	17.5	5.49	48.69	3	cell organi	membrane	catalytic a	Pf02823	513	ENSG0000	ATP5D	19	Oxidative	Electron Tr	Cristae for	14	1.133		0.430067507	9.13	5170.1	4747.6	2.61	13.39	High	High	High	High	High	High	1	
FALSE	High	Master Prc	Q9UNZ2-4	Isoform 2 c	0	29.106	24	7	12	1	339	37.3	5.11	15.69	7	cell organi	chromosor	enzyme re	Pf00789, F	55968	ENSG0000	NSFL1C	20				0	1.133		0.669901317	42.36	54.2	47.9	17.31	13.71	High	High	High	High	High	High	1	
FALSE	High	Master Prc	P15291	Beta-1,4-g	0	25.519	22	5	6	5	398	43.9	8.65	20.73	5	cell organi	cell surface	catalytic a	Pf02709, F	2683	ENSG0000	B4GALT1	9	Other type		Pre-NOTCH	34	1.132		0.050246027	2.66	1409.6	1199.4	5.04	2.34	High	High	High	High	High	High	1	
FALSE	High	Master Prc	Q9NQW6	Anillin [OS	0	21.308	7	5	5	5	1124	124.1	8.07	7.95	5	cell differe	cytoplasm;	protein bir	Pf00169, F	54443	ENSG0000	ANLN	7		Retinoblas		1	1.132		0.450938401	40.31	364.7	327.2	56.44	2.35	High	High	High	High	High	High	1	
FALSE	High	Master Prc	Q6UUV7-1	CREB-regu	0	12.061	6	2	2	2	619	66.9	6.84	6.91	2	cell organi	cytoplasm;		Pf12884, F	64784	ENSG0000	CRTC3	15	HTLV-I infe		Transcripti	5	1.132		0.50656468	39.58	472.6	417.5	29.7	16.9	High	High	High	High	High	High	1	
FALSE	High	Master Prc	P30042-1	ES1 protei	0	28.755	29	5	8	5	268	28.2	8.27	20.12	5		mitochond		Pf01965	8209; 102	ENSG0000	C21orf33;	21		Gastric Ca		1	1.131		0.197853703	13.66	3106.2	2745.6	3.67	14.23	High	High	High	High	High	High	1	
FALSE	High	Master Prc	O43464	Serine prot	0	10.81	8	2	5	2	458	48.8	10.07	8.64	2	cell death;	chromosor	catalytic a	Pf00089, F	27429	ENSG0000	HTRA2	2	Apoptosis	Parkinsons		5	1.131		0.270415966	8.41	59.3	60.4	13	8.59	High	High	High	High	High	High	1	
FALSE	High	Master Prc	O14979-1	Heterogen	0	95.17	26	15	47	13	420	46.4	9.57	100.93	15	metabolic	cytoplasm;	DNA bindi	Pf00076, F	9987	ENSG0000	HNRNPDL;	4			Gene and	5	1.131		0.337828112	23.36	15584.2	14098.9	15.08	2.57	High	High	High	High	High	High	1	
FALSE	High	Master Prc	O15446-1	DNA-direct	0	8.803	5	1	1	1	510	55	8.51	4.87	1	metabolic	chromosor	catalytic a	Pf08208	10849	ENSG0000	CD3EAP	19			RNA Polym	12	1.131		0.614897818	21.54	64.4	66.1	30.03	12.73	High	High	High	High	High	High	1	
FALSE	High	Master Prc	Q9UI10-2	Isoform 2 c	0	25.37	11	4	4	4	543	59.6	9.42	16.03	4	metabolic	cytoplasm;	protein bir	Pf01008	8890	ENSG0000	EIF2B4	2		Translatior		1	1.131		0.960374549	24.31	949.2	896.7	16.82	20.59	High	High	High	High	High	High	1	
FALSE	High	Master Prc	O43657	Tetraspani	0.001	4.482	8	2	3	2	245	27.5	8.1	2.11	2	regulation	membrane	protein bir	Pf00335	7105	ENSG0000	TSPAN6	X				0	1.13		0.000170013	1.12	658.3	582.4	5.99	7.03	High	High	High	High	High	High	1	
FALSE	High	Master Prc	Q6P4F2	Ferredoxin	0	4.575	6	1	1	1	183	19.5	5.38	2.62	1	metabolic	mitochond	metal ion l	Pf00111	112812		FDX1L	19			Endogenou	15	1.13		0.027441586	2.59	580	513.1	9.55	6.91	High	High	High	High	High	High	1	
FALSE	High	Master Prc	P27105	erythrocyt	0	36.045	26	6	17	6	288	31.7	7.88	37.14	6	cell organi	cytoplasm;	protein bir	Pf01145	2040	ENSG0000	STOM	9			Neutrophil	6	1.13		0.058855889	0.51	5810	5523.9	7.27	6.41	High	High	High	High	High	High	1	
FALSE	High	Master Prc	O94966	Ubiquitin c	0	6.186	5	4	4	4	1318	145.6	6.28	4.71	4	metabolic	cytosol;en	catalytic a	Pf00443, F	10869	ENSG0000	USP19	3			Ub-specific	4	1.13		0.065792721	8.4	202.5	171.1	5.1	7.57	High	High	High	High	High	High	1	
FALSE	High	Master Prc	Q9Y3B7-1	39S riboso	0	32.117	45	7	9	7	192	20.7	9.91	22.63	7	cell organi	membrane	protein bir	Pf00298, F	65003	ENSG0000	MRPL11	11	Ribosome		Mitochonc	7	1.13		0.239951034	9.83	2346.3	2369.4	11.92	7.74	High	High	High	High	High	High	1	
FALSE	High	Master Prc	P28838	cytosol am	0	56.299	29	11	18	11	519	56.1	7.93	36.5	11	metabolic	cytoplasm;	catalytic a	Pf00883, F	51056	ENSG0000	LAP3	4	Metabolic			3	1.13		0.486947423	0.28	3925.6	3686	9.29	4.06	High	High	High	High	High	High	1	
FALSE	High	Master Prc	P42025	Beta-centr	0	13.748	9	3	5	1	376	42.3	6.4	9.45	3	transport	cytoplasm;	nucleotide	Pf00022	10120	ENSG0000	ACTR1B	2			Neutrophil	5	1.129		0.006085029	1.28	637.9	568.4	1.52	2.87	High	High	High	High	High	High	1	
FALSE	High	Master Prc	Q9Y4L1	Hypoxia up	0	135.775	28	23	46	23	999	111.3	5.22	139.85	23	regulation	endoplasm	nucleotide	Pf00012	10525	ENSG0000	HYOU1	11; CHR_H	Protein pro		Scavenging	8	1.129		0.057707376	5.6	14562.4	13505.3	7.19	4.05	High	High	High	High	High	High	1	
FALSE	High	Master Prc	Q07817-1	bcl-2-like p	0	8.384	12	3	3	3	233	26	4.93	4.8	3	cell death;	cytoplasm;	protein bir	Pf00452, F	598	ENSG0000	BCL2L1	20	Jak-STAT s	Leptin sign	The NLRP1	42	1.129		0.239431696	16.36	513.7	454.8	7.72	4.26	High	High	High	High	High	High	1	
FALSE	High	Master Prc	P20020	Plasma me	0	27.92	5	4	8	3	1258	138.7	6.04	16.8	4	cell comm	membrane	catalytic a	Pf00122, F	490		ATP2B1	12	Salivary se	Calcium Re	Ion homeo	19	1.129		0.26368393	10.53	951.8	842.7	5.59	5.28	High	High	High	High	High	High	1	
FALSE	High	Master Prc	Q9H299	SH3 domai	0	7.49	27	3	10	3	93	10.4	4.93	7.36	3	cellular ho	cytoplasm;	catalytic a	Pf00462, F	83442	ENSG0000	SH3BGRL3	1				0	1.129		0.321174999	3.29	7300.9	6618.1	1.39	9.58	High	High	High	High	High	High	1	
FALSE	High	Master Prc	Q9Y3D3	28S riboso	0	6.863	28	3	3	3	137	15.3	9.5	5.04	3	cell organi	cytosol;me	protein bir	Pf00886	51021	ENSG0000	MRPS16	10	Ribosome		Mitochonc	7	1.129		0.399588044	26.2	33.9	30.5	18.65	9.97	High	High	High	High	High	High	1	
FALSE	High	Master Prc	Q9BZJ0	Crooked ne	0	10.541	4	3	3	3	848	100.4	8	7.37	3	cell organi	cytoplasm;	catalytic a	Pf02184, F	51340	ENSG0000	CRNKL1	20	Spliceosom		mRNA Spli	5	1.129		0.431931151	2.68	275.7	256.2	5.86	6.82	High	High	High	High	High	High	1	

FALSE	High	Master Prc	Q9BYN0	sulfiredoxi	0	26.37	39	4	4	4	137	14.3	8.19	16.35	4	metabolic	cytoplasm;	antioxidan	Pf02195	140809	ENSG0000	SRXN1	20		Photodyna		1	1.129	0.755807259	27.47	1096	996	12	42.25	High	High	High	High	High	High	1	[R16]
FALSE	High	Master Prc	Q14320	Protein FA	0	28.145	15	5	7	5	339	40.2	6.83	13.45	5		nucleus	RNA bindi	Pf04921	9130	ENSG0000	FAM50A	X				0	1.128	0.066785417	7.59	1286.4	1145.9	3.23	3.38	High	High	High	High	High	High	1	
FALSE	High	Master Prc	P31327-1	Carbamoyl	0	646.644	59	85	301	81	1500	164.8	6.74	761.59	85	cell differe	cytoplasm;	catalytic a	Pf00117, P	1373	ENSG0000	CPS1	2	Nitrogen n	Amino Aci	Urea cycle	12	1.128	0.384604039	21.3	101584.8	91422.2	11.35	6.44	High	High	High	High	High	High	1	
FALSE	High	Master Prc	Q9UNE7-1	E3 ubiquiti	0	17.496	19	6	9	6	303	34.8	5.87	19.27	6	metabolic	cytoplasm;	catalytic a	Pf00515, P	10273	ENSG0000	STUB1	16	Protein pro	Androgen	Regulation	25	1.128	0.698268752	1.57	2657	2465	22.34	10.77	High	High	High	High	High	High	1	
FALSE	High	Master Prc	P16298	serine/thre	0	4.831	6	2	2	2	524	59	5.91	2.08	2	cell differe	cytosol;me	catalytic a	Pf00149	5532	ENSG0000	PPP3CB	10	Natural kil	Physiologi	Signaling b	52	1.128	0.776866454	3.39	46.7	50.9	20.18	19.58	High	High	High	High	High	High	1	
FALSE	High	Master Prc	O43399	Tumor pro	0	45.228	62	9	20	9	206	22.2	5.36	42.98	9	regulation	cytoplasm	protein bir	Pf04201	7165	ENSG0000	TPD52L2	20				0	1.127	0.093050267	3.82	3846.6	3426.8	8.39	3.79	High	High	High	High	High	High	1	
FALSE	High	Master Prc	Q9NW64-1	Pre-mRNA	0	9.896	9	4	4	4	420	46.9	8.54	7.86	4	cell organi	cytoplasm;	metal ion l	Pf00076, P	55696	ENSG0000	RBM22	5	Spliceosom		mRNA Spli	5	1.127	0.159388202	9.28	930.3	820.2	9.42	4.78	High	High	High	High	High	High	1	
FALSE	High	Master Prc	Q13895	Bystin [OS	0	6.143	7	3	3	3	437	49.6	8.12	2.81	3	cell differe	cytoplasm;	protein bir	Pf05291	705	ENSG0000	BYSL	6			Major path	4	1.127	0.35059337	22.85	535.2	474.8	3.74	15.85	High	High	High	High	High	High	1	
FALSE	High	Master Prc	Q9NX20	39S riboso	0	6.624	8	2	2	2	251	28.4	10.13	2.7	2	cell organi	membrane	RNA bindi	Pf00252	54948	ENSG0000	MRPL16	11	Ribosome		Mitochonc	7	1.127	0.352533482	8.37	107.8	105.8	11.26	6.09	High	High	High	High	High	High	1	
FALSE	Medium	Master Prc	Q6I9Y2	THO comp	0.01	2.068	6	1	1	1	204	23.7	5.67	0	1	metabolic	cytoplasm;	protein bir	Pf05615	80145	ENSG0000	THOC7	3	RNA trans		Cleavage o	10	1.127	0.546875364	12.04	592.6	529.8	7.36	9.86	High	High	High	High	High	High	1	
FALSE	High	Master Prc	Q7Z2T5-1	TRMT1-like	0	21.535	6	3	4	3	733	81.7	7.88	16.02	3	metabolic		catalytic a	Pf02005, P	81627	ENSG0000	TRMT1L	1				0	1.127	0.68567728	4.46	1011.3	897.2	6.54	9.42	High	High	High	High	High	High	1	
FALSE	High	Master Prc	P30519	Heme oxyg	0	60.835	43	10	19	10	316	36	5.41	57.2	10	cellular ho	endoplasm	catalytic a	Pf01126	3163	ENSG0000	HMOX2	16; CHR_H	Mineral ab		Heme degr	10	1.126	0.197927378	11.1	2271.1	2179.6	17.23	7.3	High	High	High	High	High	High	1	
FALSE	High	Master Prc	Q562R1	Beta-actin-	0	118.41	21	10	137	2	376	42	5.59	344.88	10		cytoplasm;	nucleotide	Pf00022	345651	ENSG0000	ACTBL2	5				0	1.126	0.770936322	2.11	192.8	184.4	13.5	5.62	High	High	High	High	High	High	1	
FALSE	High	Master Prc	Q96EE3	nucleopori	0	8.295	5	2	3	2	360	39.6	8.09	7.09	2	cell commu	chromosor	protein bir	Pf00400	81929	ENSG0000	SEH1L	18	RNA trans		Rev-media	65	1.126	0.77399958	0.22	1239.2	1114.9	11.9	4	High	High	High	High	High	High	1	
FALSE	High	Master Prc	P23246-1	splicing fac	0	188.083	43	32	78	31	707	76.1	9.44	190.21	32	cell organi	chromosor	DNA bindi	Pf00076, P	6421	ENSG0000	SFPQ	1		mRNA Pro	PTK6 Regu	5	1.125	0.083545941	8.39	23962	21290.9	3.87	2.6	High	High	High	High	High	High	1	
FALSE	High	Master Prc	Q9NSE4	Isoleucine-	0	101.525	24	22	35	22	1012	113.7	7.2	89.09	22	metabolic	cytoplasm;	catalytic a	Pf00133, P	55699	ENSG0000	IARS2	1	Aminoacyl		Mitochonc	5	1.125	0.086175672	1.42	7064.8	6150.7	8.87	3.68	High	High	High	High	High	High	1	
FALSE	High	Master Prc	Q8N1F7-1	Nuclear po	0	71.151	27	20	38	20	819	93.4	5.72	61.06	20	cell organi	membrane	protein bir	Pf04097	9688	ENSG0000	NUP93	16	RNA trans		Rev-media	64	1.125	0.157189864	9.53	10709.8	9800.9	5.5	5.32	High	High	High	High	High	High	1	
FALSE	High	Master Prc	P14859-1	POU doma	0.003	3.027	2	1	1	1	743	76.4	6.81	2.67	1	metabolic	endoplasm	DNA bindi	Pf00046, P	5451	ENSG0000	POU2F1	1		Integrated	RNA Polym	12	1.125	0.272248704	17.4	496.6	446.1	12.53	0.68	High	High	High	High	High	High	1	
FALSE	High	Master Prc	O95833	Chloride in	0	4.599	9	2	3	2	236	26.6	6.43	2.9	2	metabolic	cytoplasm;	catalytic a	Pf13409, P	9022	ENSG0000	CLIC3	9				0	1.125	0.29904764	13.45	896.8	847.9	9.2	6.73	High	High	High	High	High	High	1	
FALSE	High	Master Prc	Q9NWM8	Peptidyl-pr	0	7.145	12	2	2	2	211	24.2	6.07	6.07	2	metabolic	cytoplasm;	catalytic a	Pf00254	55033	ENSG0000	FKBP14	7			XBP1(S) ac	4	1.125	0.91775448	14.45	457.9	443.2	16.83	17.44	High	High	High	High	High	High	1	
FALSE	High	Master Prc	P55081	microfibril	0	14.651	7	3	4	2	439	51.9	4.98	10.68	3	cell organi	extracellul	protein bir	Pf06991	4236	ENSG0000	MFAP1	15		Canonical	Molecules	4	1.124	0.257350279	17.23	340.2	280.1	4.77	14.17	High	High	High	High	High	High	1	
FALSE	High	Master Prc	P08195	4F2 cell-su	0	165.174	40	24	63	24	630	68	5.01	173.89	24	cell growth	cell surface	catalytic a	Pf00128	6520	ENSG0000	SLC3A2	11	mTOR sign		Tryptopha	13	1.124	0.301164449	5.55	21624.5	19321.8	7.61	2.92	High	High	High	High	High	High	1	
FALSE	High	Master Prc	Q14847	LIM and SH	0	94.056	47	15	49	15	261	29.7	7.05	96.74	15	regulation	cytoplasm;	metal ion l	Pf00018, P	3927	ENSG0000	LASP1	17				0	1.124	0.30216528	11.84	21831.2	19416.5	4.5	7.12	High	High	High	High	High	High	1	
FALSE	High	Master Prc	P02753	Retinol-bir	0	8.308	11	1	1	1	201	23	6.07	5.03	1	metabolic	cytosol;ext	protein bir	Pf00061, P	5950	ENSG0000	RBP4	10		Sterol Reg	Signaling b	15	1.124	0.367911507	21.62	263.1	234	32.35	0.75	High	High	High	High	High	High	1	
FALSE	High	Master Prc	Q9NR46	Endophilin	0	42.224	30	10	14	10	395	43.9	5.99	30.37	10		cytoplasm;	protein bir	Pf03114, P	56904	ENSG0000	SH3GLB2	9	Endocytosi			1	1.124	0.373635287	24.82	1530.9	1274.9	14.25	7.24	High	High	High	High	High	High	1	
FALSE	High	Master Prc	Q12846	Syntaxin-4	0	9.739	8	2	2	2	297	34.2	6.28	5.22	2	cell organi	cell surface	enzyme re	Pf00804, P	6810	ENSG0000	STX4	16	SNARE inte	Insulin Sig	Translocat	20	1.124	0.486062085	40.3	262.1	262.1	47.43	10.43	High	High	High	High	High	High	1	
FALSE	High	Master Prc	O75970	multiple PI	0	6.614	1	1	1	1	2070	221.5	5.06	4.26	1		cytoplasm;	protein bir	Pf00595, P	8777	ENSG0000	MPDZ	9	Tight junct			1	1.124	0.870810148	2.78	238.7	243.3	17.71	13.32	High	High	High	High	High	High	1	
FALSE	High	Master Prc	P08621-1	U1 small n	0	64.985	26	12	30	12	437	51.5	9.94	61.06	12	metabolic	cytoplasm;	protein bir	Pf00076, P	6625	ENSG0000	SNRNP70	19	Spliceosom	mRNA Pro	mRNA Spli	6	1.123	0.026695124	0.33	10422.3	9624.1	3.21	4.22	High	High	High	High	High	High	1	
FALSE	High	Master Prc	P19387	DNA-direct	0	7.421	13	2	2	2	275	31.4	4.92	3.29	2	metabolic	cytoplasm;	catalytic a	Pf01000, P	5432	ENSG0000	POLR2C	16	Metabolic	Eukaryotic	Signaling b	75	1.123	0.070417583	5.14	392.1	320	13.27	12.34	High	High	High	High	High	High	1	
FALSE	High	Master Prc	P23786	Carnitine O	0	41.403	24	11	15	11	658	73.7	8.18	28.28	11	metabolic	membrane	catalytic a	Pf00755	1376	ENSG0000	CPT2	1	Fatty acid	Fatty Acid	Import of p	13	1.123	0.152860291	4.48	4342.3	3269.1	15.37	15.26	High	High	High	High	High	High	1	
FALSE	High	Master Prc	Q9Y2S7	Polymeras	0	35.177	30	8	9	8	368	42	8.63	23.78	8	cell organi	mitochond	DNA bindi	Pf04379, P	26073	ENSG0000	POLDIP2	17				0	1.123	0.219388871	10.88	518.1	442.1	10.01	8.65	High	High	High	High	High	High	1	
FALSE	High	Master Prc	Q9Y3E0	vesicle tra	0.004	2.774	9	1	1	1	138	15.4	10.36	0	1	regulation	endoplasm	signal tran	Pf04178	51026	ENSG0000	GOLT1B	12				0	1.123	0.386511475	24.3	530.2	472	14.64	11.99	High	High	High	High	High	High	1	
FALSE	Medium	Master Prc	Q96JM3	Chromoso	0.015	1.735	1	1	1	1	812	89	8.44	1.79	1	cell organi	chromosor	metal ion l	Pf00096	283489	ENSG0000	CHAMP1	13				0	1.123	0.457114868	65.31	573.9	511.2	125.58	54.82	High	High	High	High	High	High	1	
FALSE	High	Master Prc	Q9H814	Phosphory	0	11.501	7	2	3	2	394	44.4	5.4	12.53	2	metabolic	cytoplasm;	protein bir	Pf10258	51808	ENSG0000	PHAX	5	RNA trans		snRNP Ass	7	1.123	0.491408374	33.83	878	793.2	23.54	10.06	High	High	High	High	High	High	1	
FALSE	High	Master Prc	Q9NR45	sialic acid	0	39.974	27	10	20	10	359	40.3	6.74	38.36	10	metabolic	cytoplasm;	catalytic a	Pf03102, P	54187	ENSG0000	NANS	9	Metabolic		Sialic acid	8	1.122	0.277197061	9.67	5485.4	5065.9	6.54	6.03	High	High	High	High	High	High	1	
FALSE	High	Master Prc	P00403	Cytochrom	0	34.604	31	7	14	7	227	25.5	4.82	36.44	7	metabolic	membrane	catalytic a	Pf00116, P	4513	ENSG0000	COX2	MT	Alzheimer'	Electron Tr	TP53 Regu	19	1.122	0.79091007	18.24	3698.1	3295.8	13.33	10.94	High	High	High	High	High	High	1	
FALSE	Low	Master Prc	Q9Y2Q0	Phospholip	0.069	1.046	1	1	1	1	1164	131.3	6.84	2.59	1	cell organi	endoplasm	catalytic a	Pf00122, P	10396	ENSG0000	ATP8A1	4		Peptide GF	Neutrophil	7	1.122	0.818490379	16.33	616.1	647.4	20.76	16.49	High	High	High	High	High	High	1	[Y588(100
FALSE	High	Master Prc	P27694	Replicatior	0	48.867	20	8	13	8	616	68.1	7.21	45.33	8	cell organi	nucleus	DNA bindi	Pf01336, P	6117	ENSG0000	RPA1	17	DNA replic	Homologo	Translesior	87	1.121	0.022755724	2.23	4860.2	4330.5	1.7	3.65	High	High	High	High	High	High	1	
FALSE	High	Master Prc	Q8IVS2	Malonyl-Co	0.002	4.076	4	1	1	1	390	42.9	8.72	3.44	1	metabolic	mitochond	catalytic a	Pf00698	27349	ENSG0000	MCAT	22	Metabolic		Mitochonc	7	1.121	0.325788407	19.71	313.2	292.9	11.08	8.89	High	High	High	High	High	High	1	
FALSE	High	Master Prc	Q01167	Forkhead b	0	5.719	4	1	1	1	660	69	9.54	3.31	1	cell differe	nucleus	DNA bindi	Pf00250, P	3607	ENSG0000	FOXK2	17			UCH prote	4	1.121	0.411233288	26.41	379.1	340.3	29.77	8.49	High	High	High	High	High	High	1	
FALSE	High	Master Prc	Q96EY4	translation	0.001	4.238	10	1	1	1	203	23.8	9.26	3.81	1		nucleus;rib	protein bir	Pf11176	55319	ENSG0000	TMA16	4				0	1.121	0.452887715	33.39	167.3	149.2	38.06	21.82	High	High	High	High	High	High	1	
FALSE	High	Master Prc	P07738	Bisphosph	0	5.932	7	1	1	1	259	30	6.54	2.8	1	metabolic	cytosol	catalytic a	Pf00300	669	ENSG0000	BPGM	7	Metabolic		Glycolysis	7	1.121	0.471210565	45.43	269.4	262.7	75.16	19.09	High	High	High	High	High	High	1	
FALSE	High	Master Prc	Q14692	ribosome b	0	8.067	2	2	2	2	1282	145.7	6.44	5.01	2	cell organi	nucleus	catalytic a	Pf04950, P	9790	ENSG0000	BMS1	10	Ribosome		Major path	6	1.121	0.7192322	38.15	251.6	231.5	29.51	3.81	High	High	High	High	High	High	1	
FALSE	High	Master Prc	P00367	Glutamate	0	162.881	48	24	61	24	558	61.4	7.8	164.97	24	cell organi	cytoplasm;	catalytic a	Pf00208, P	2746	ENSG0000	GLUD1	10	Arginine bi	Amino Aci	Transcripti	15	1.12	0.018131275	1.71	19305.3	17232.5	5.67	3.66	High	High	High	High	High	High	1	
FALSE	High	Master Prc	Q15418-1	Ribosomal	0	33.697	16	8	10	2	735	82.7	7.83	24.7	8	cell death;	cytoplasm;	catalytic a	Pf00069, P	6195	ENSG0000	RPS6KA1	1; CHR_HG	Insulin resi	IL-5 Signali	CREB phos	68	1.12	0.060432971	2.43	542.1	491.6	1.19	3.79	High	High	High	High	High	High	1	[Q132]
FALSE	High	Master Prc	Q92522	Histone H1	0	19.601	17	4	13	4	213	22.5	10.76	16.78	4	cell organi	chromosor	DNA bindi	Pf00538	8971	ENSG0000	H1FX	3				0	1.12	0.080154915	0.2	1629.5	1350.2	7	4.57	High	High	High	High	High	High	1	
FALSE	High	Master Prc	O75844	caax preny	0.004	2.641	4	2	2	2	475	54.8	7.49	1.62	2	cell organi	endoplasm	catalytic a	Pf01435	10269	ENSG0000	ZMPSTE24	1	Terpenoid	Adipogene		2	1.12	0.098160777	7.03	161.3	146.2	5.29	5.79	High	High	High	High	High	High	1	
FALSE	High	Master Prc	P52815	39S riboso	0	45.741	40	5	20	5	198	21.3	8.87	46.08	5	cell organi	membrane	protein bir	Pf00542	6182	ENSG0000	MRPL12	17	Ribosome		Mitochonc	7	1.12	0.165671419	12.46	6681	6095.8	10.33	2.5	High	High	High	High	High	High	1	
FALSE	High	Master Prc	Q9NXW2-1	DnaJ homo	0	13.394	13	4	4	4	375	41.8	8.53	5.74	4	cell organi	endoplasm	protein bir	Pf00226, P	54788		DNAJB12	10	Protein pro			1	1.12	0.379773042	23.56	1275.2	1192.1	14.52	6.81	High	High	High	High	High	High	1	
FALSE	High	Master Prc	O75915	PRA1 fami	0	24.397	23	4	9	4	188	21.6	9.77	31.38	4	regulation	cytoplasm;	protein bir	Pf02990, P	10550	ENSG0000	ARL6IP5	3			Glutamate	4	1.12	0.383083604	2.24	3539	3212.2	2.73	7.73	High	High	High	High	High	High	1	
FALSE	High	Master Prc	P51159-1	Ras-relatec	0	7.72	9	2	2	1	221	24.9	5.22	2.39	2	cell differe	cytosol;en	catalytic a	Pf00009, P	5873	ENSG0000	RAB27A	15		Deregulati	Insulin pro	13	1.12	0.517928361	4.47	966.9	889.3	4.56	9.18	High	High	High	High	High	High	1	
FALSE	High	Master Prc	P61026	ras-related	0	22.202	35	7	9	5	200	22.5	8.38	12.75	7	cell differe	cytosol;en	catalytic a	Pf00025, P	10890	ENSG0000	RAB10	2	Endocytosi	Focal Adhe	Translocat	14	1.12	0.893552877	42.59	656.7	586.4	43.33	13.78	High	High	High	High	High	High	1	
FALSE	High	Master Prc	Q13733	sodium/po	0	49.853	10	9	29	1	1029	114.1	6.64	52.57	9	cellular co	membrane	catalytic a	Pf00122, P	480	ENSG0000	ATP1A4	1	Thyroid ho	Calcium Re	Ion homeo	24	1.119	0.021399551	3.35	2315.3	2035.4	4.54	2.41	High	High	High	High	High	High	1	[Q233;
FALSE	High	Master Prc	P13674-1	prolyl 4-hy	0	58.366	22	10	15	10	534	61	6.01	38.98	10	cell organi	endoplasm	catalytic a	Pf03171, P	5033	ENSG0000	P4HA1	10	Metabolic		Collagen b	5	1.119	0.069510757	3.29	4470.8	3997	7.62	1.92	High	High	High	High	High	High	1	
FALSE	High	Master Prc	Q14126	Desmoglei	0	90.409	23	17	26	17	1118	122.2	5.24	65.1	17	cell death;	cell surface	metal ion l	Pf00028, P	1829	ENSG0000	DSG2	18	Arrhythmo	Arrhythmo	Apoptotic	10	1.119	0.122125419	2.4	5600.2	4885.3	9.7	1.42	High	High	High	High	High	High	1	
FALSE	High	Master Prc	Q9H0U4	ras-related	0	81.704	68	12	30	5	201	22.2	5.73	95.22	12	cell organi	cytoplasm;	catalytic a	Pf00025, P	81876	ENSG0000	RAB1B	11	Legionello		COPI-depe	21	1.119	0.165676608	3.19	6592.1	5769	7.4	1.72	High	High	High	High	High	High	1	
FALSE	High	Master Prc	Q15907	Ras-relatec	0	43.211	45	10	19	10	218	24.5	5.94	35.72	10	metabolic	cytoplasm;	catalytic a	Pf00025, P	9230	ENSG0000	RAB11B	19	AMPK sign	Focal Adhe	TBC/RABG	11	1.119	0.211520516	14.34	8173.8	7307.6	12.12	2.31	High	High	High	High	High	High	1	
FALSE	High	Master Prc	O15126-1	Secretory	0.003	3.391	4	1	1	1	338	37.9	7.42	2.27	1	transport	endosome	protein bir	Pf04144	9522	ENSG0000	SCAMP1	5			Neutrophil	3	1.119	0.283045444	4.65	380.9	346.2	10.44	5.79	High	High	High	High	High	High	1	
FALSE	High	Master Prc	Q08380	Galectin-3-	0	44.321	23	11	14	11	585	65.3	5.27	27.19	11	defense re	extracellul	protein bir	Pf00530, P	3959	ENSG0000	LGALS3BP	17			Platelet de	4	1.119	0.356186944	18.1	2763.7	2719.1	31.8	6.42	High	High	High	High	High	High	1	
FALSE	High	Master Prc	P51149	ras-related	0	79.2	64	11	29	11	207	23.5	6.7	74.53	11	cell organi	cytoplasm;	catalytic a	Pf00025, P	7879	ENSG0000	RAB7A	3	Amoebiasi		TBC/RABG	18	1.119	0.538818861	9.2	10726.9	9583.1	11.07	3.49	High	High	High	High	High	High	1	
FALSE	High	Master Prc	P61020	Ras-relatec	0	39.904	43	7	12	5	215	23.7	8.13	35.43	7	cell organi	endosome	catalytic a	Pf00009, P	5869	ENSG0000	RAB5B	12	Vasopressi		TBC/RABG	18	1.119	0.623276137	24.06	3696.9	3305	18.73	3.95	High	High	High	High	High	High	1	
FALSE	High	Master Prc	O43264	Centromer	0	4.759	4	2	2	2	779	88.8	6.27	2.16	2	cell divisio	chromosor	DNA bindi	Pf06248	9183	ENSG0000	ZW10	11			COPI-depe	21	1.119	0.851017522	10	60.7	54.2	21.71	11.51	High	High	High	High	High	High	1	
FALSE	High	Master Prc	P20645	Cation-dep	0	12.247	12	4	10	4	277	31	5.83	11.54	4	cell organi	endosome	protein bir	Pf02157	4074	ENSG0000	M6PR	12	Phagosom		Cargo reco	11	1.118	0.016292039	2.44	5008	4480.8	1.56	4.59	High	High	High	High	High	High	1	
FALSE	High	Master Prc	L0R6Q1	SLC35A4 u	0	11.744	40	3	4	3	103	11.1	8.1	9.76	3	regulation	Golgi;mem	transporte	Pf06553, P	113829	ENSG0000	SLC35A4	5				0	1.118	0.174617618	10.22	3492.6	3217.7	4.18	12.57	High	High	High	High	High	High	1	
FALSE	High	Master Prc	P13667	Protein dis	0	208.354	56	33	73	33	645	72.9	5.07	162.67	33	cellular ho	cell surface	catalytic a	Pf00085, P	9601	ENSG0000	PDIA4	7	Protein pro			3	1.118	0.312937142	14.23	21761.1	19463.5	10.73	1.54	High	High	High	High	High	High	1	
FALSE	High	Master Prc	Q8IWA0	WD repeat	0	10.557	5	3	3	3	830	94.4	5.96	8.67	3	metabolic	nucleus	protein bir	Pf00400	84128	ENSG0000	WDR75	2	Ribosome		Major path	6	1.118	0.4098898	25.61	270.6	242	15.35	8.07	High	High	High	High	High	High	1	
FALSE	High	Master Prc	Q7Z6K5	Arpin [OS=	0	5.763	5	1	1	1	226	24.9	5.83	1.71	1	regulation		protein bir	Pf10574	348110	ENSG0000	C15orf38;	15				0	1.118	0.475582651	1.6	899	893	7.55	7.89	High	High	High	High	High	High	1	
FALSE	High	Master Prc	P49821	NADH deh	0	30.694	16	6	8	6	464	50.8	8.21	27.48	6	cell organi	cytosol;me	catalytic a	Pf01512, P	4723	ENSG0000	NDUFV1	11	Non-alcoh	Electron Tr	Complex I	13	1.118	0.867337596	21.47	670.2	599.4	10.9	15.57	High	High	High	High	High	High	1	
FALSE	High	Master Prc	A1L0T0	Acetolacta	0	32.494	16	7	7	7	632	67.8	8.15	23.84	7		membrane	catalytic a	Pf00205, P	10994	ENSG0000	ILVBL	19				0	1.117	0.052426386	2.73	1075.2	994.8	4.49	2.31	High	High	High	High	High	High	1	
FALSE	High	Master Prc	A6NJ78	Probable n	0	5.508	4	1	1	1	407	46.1	8.62	3.65	1	metabolic		catalytic a	Pf01795, P	196074	ENSG0000	METTL15	11				0	1.117	0.161921263	9.07	330.9	296.1	13.26	4.07	High	High	High	High	High	High	1	
FALSE	High	Master Prc	P30536	translocato	0.002	3.615	5	1	2	1	169	18.8	9.36	5.47	1	cell death;	membrane	protein bir	Pf03073	706	ENSG0000	TSPO	22	HTLV-I infe		Pregnenolo	7	1.117	0.187982172	8.24	1224.8	1096.2	21.91	11.35	High	High	High	High	High	High	1	
FALSE	High	Master Prc	Q99757	Thioredoxi	0	12.131	13	1	1	1	166	18.4	8.29	6.02	1	cell commu	mitochond	catalytic a	Pf00085, P	25828	ENSG0000	TXN2	22	NOD-like r	Oxidative S	Detoxificat	5	1.117	0.692949759	63.53	425.9	381.3	35.89	17	High	High	High	High	High	High	1	
FALSE	High	Master Prc	Q6L8Q7-1	2',5'-phosp	0	19.413	8	4	4	4	609	67.3	6.57	14.34	4	metabolic	mitochond	catalytic a	Pf03372	201626	ENSG0000	PDE12	3				0	1.117	0.831981025	4.79	765.8	685.5	13.81	4.4	High	High	High	High	High	High	1	
FALSE	High	Master Prc	Q5JRX3	Presequen	0	73.491	21	16	25	16	1037	117.3	6.92	51.28	16	metabolic	mitochond	catalytic a	Pf00675, P	10531	ENSG0000	PITRM1	10				0	1.116	0.073168129	1.26	5653.5	5109.7	10.31	5.29	High	High	High	High	High	High	1	
FALSE	High	Master Prc	P42167	Lamina-ass	0	48.421	28	7	11	3	454	50.6	9.38	37.67	7	regulation	chromosor	DNA bindi	Pf03020, P	7112	ENSG0000	TMPO	12			Depolymer	11	1.116	0.098257769	4.97	766.3	686.5	6.21	1.65	High	High	High	High	High	High	1	[T74(100)
FALSE	High	Master Prc	Q15599	Na(+)/H(+)	0	8.47	9	3	4	3	337	37.4	7.93	6.63	3	cell organi	membrane	protein bir	Pf00595, P	9351	ENSG0000	SLC9A3R2	16	Aldosteror			1	1.116	0.159707531	8.18	66.9	59.9	2.66	5.09	High	High	High	High	High	High	1	
FALSE	High	Master Prc	O95831-1	Apoptosis-	0	72.496	28	11	21	11	613	66.9	8.95	47.32	11	cell death;	cytoplasm;	catalytic a	Pf00070, P	9131	ENSG0000	AIFM1	X	Apoptosis	Apoptosis		4	1.116	0.289891793	4.55	5637.1	5428.5	7.27	5.15	High	High	High	High	High	High	1	
FALSE	High	Master Prc	Q9NSK0-1	Kinesin ligh	0	8.123	5	2	2	1	619	68.6	6.18	5.94	2		cytoplasm;	catalytic a	Pf00515, P	89953	ENSG0000	KLC4	6	Salmonella		COPI-depe	16	1.116	0.419747465	25.14	301.8	270.5	9.27	13.24	High	High	High	High	High	High	1	
FALSE	High	Master Prc	P18074-1	Tfiih basal	0.004	2.668	1	1	1	1	760	86.9	7.15	2.39	1	cell death;	cytoplasm;	catalytic a	Pf06733, P	2068	ENSG0000	ERCC2	19	Basal trans	Fluoropyri	Cytosolic ir	54	1.116	0.438117359	29.25	1164.6	1071.1	33.11	8.24	High	High	High	High	High	High	1	

FALSE	High	Master Prc	Q86W56	Poly(ADP-r	0	5.466	2	1	1	1	976	111	6.43	4.97	1	metabolic	cytoplasm;	catalytic a	Pf05028	8505; 670	ENSG0000	PARG; BPH	10; 6			POLB-Depe	5	1.116	0.709091717	2.13	431.3	417.3	11.6	5.3	High	High	High	High	High	High	1	
FALSE	High	Master Prc	Q14671-3	Isoform 3 (	0	39.221	8	7	10	5	1188	126.6	6.84	27.19	7	cell differe	cytosol;nu	protein bir	Pf00806	9698	ENSG0000	PUM1	1				0	1.115	0.238121748	2	572.1	518.5	5.72	2.12	High	High	High	High	High	High	1	
FALSE	High	Master Prc	Q3MHD2	Protein LSI	0.004	2.655	4	1	2	1	195	21.7	7.74	4.76	1				Pf09793	124801; 6!	ENSG0000	LSM12; LSI	17; 8				0	1.115	0.283713314	13.54	345.7	310.1	9.89	12.8	High	High	High	High	High	High	1	
FALSE	High	Master Prc	P50750	Cyclin-depe	0	4.598	7	2	2	2	372	42.8	8.79	2.39	2	cell prolife	chromosor	catalytic a	Pf00069, P	1025	ENSG0000	CDK9	9	Transcripti	MicroRNA	RNA polym	35	1.115	0.378192393	21.78	134.9	120.9	21.56	8.7	High	High	High	High	High	High	1	
FALSE	High	Master Prc	Q96I36	Cytochrom	0.006	2.349	12	1	1	1	57	6.6	9.55	1.73	1	cell organi:	membrane		Pf14880	84987	ENSG0000	COX14	12			TP53 Regu	9	1.115	0.547598963	8.15	1345	1149.7	3.85	16.43	High	High	High	High	High	High	1	
FALSE	High	Master Prc	P78310-1	Coxsackiev	0	11.232	7	2	3	2	365	40	7.56	2.29	2	cell commu	cytoplasm;	protein bir	Pf07679, P	1525	ENSG0000	CXADR	21	Viral myoc		Immunore	6	1.115	0.778726208	16.35	667.5	633.3	24.55	4.79	High	High	High	High	High	High	1	
FALSE	High	Master Prc	Q9BQQ3-1	Golgi reass	0	16.455	8	2	2	1	440	46.5	4.5	8.91	2	cell organi:	Golgi;mem	protein bir	Pf04495, P	64689	ENSG0000	GORASP1	3			COPII (Coa	14	1.115	0.820300244	33.72	229.7	206	23.65	8.11	High	High	High	High	High	High	1	
FALSE	High	Master Prc	Q9HD34	LYR motif-	0	4.839	15	1	2	1	91	10.8	10.73	8.07	1	metabolic	mitochond		Pf05347, P	57128	ENSG0000	LYRM4	6			Mitochonc	2	1.114	0.060398077	7.12	1224.4	1095.3	1.62	6.61	High	High	High	High	High	High	1	
FALSE	High	Master Prc	P16070	CD44 antig	0	47.821	13	9	26	9	742	81.5	5.33	47.58	9	cell organi:	cell surface	protein bir	Pf00193	960	ENSG0000	CD44	11	ECM-recep	Hepatitis C	Integrin ce	25	1.114	0.134092143	9.18	3159.7	2835.2	11.06	4.55	High	High	High	High	High	High	1	
FALSE	High	Master Prc	Q15007	Pre-mRNA-	0	7.68	7	2	2	2	396	44.2	5.19	6.84	2	metabolic	membrane	protein bir		9589	ENSG0000	WTAP	6			Processing	2	1.114	0.332763738	9.4	1022.3	975.1	3.87	9.88	High	High	High	High	High	High	1	
FALSE	High	Master Prc	Q96A35	39S riboso	0	8.634	16	3	4	3	216	24.9	9.29	1.7	3	cell organi:	membrane	structural	Pf00467	79590	ENSG0000	MRPL24	1	Ribosome		Mitochonc	7	1.114	0.474960706	32.58	292.3	279.8	30.78	7.33	High	High	High	High	High	High	1	
FALSE	High	Master Prc	P04062	glucosylcer	0	19.11	11	4	7	4	536	59.7	7.61	18.76	4	cell commu	membrane	catalytic a	Pf02055	2629	ENSG0000	GBA	1	Other glyc:		Glycosphin	12	1.113	0.036450278	5.31	3126.3	2735.3	5.56	9.17	High	High	High	High	High	High	1	
FALSE	High	Master Prc	Q03252	Lamin-B2 [	0	43.251	21	15	21	10	620	69.9	5.59	30.18	15		membrane	catalytic a	Pf00038, P	84823	ENSG0000	LMNB2	19	Apoptosis	Gastric car		3	1.113	0.187667217	6.46	4420.1	4080.8	5.86	4.3	High	High	High	High	High	High	1	
FALSE	High	Master Prc	P48509	CD151 ant	0.005	2.551	3	1	3	1	253	28.3	7.47	1.93	1	cell organi:	cytosol;me	protein bir	Pf00335	977	ENSG0000	CD151	11		Primary Fc	Assembly c	7	1.113	0.237513332	6.59	150.6	115.4	14.25	13.54	High	High	High	High	High	High	1	
FALSE	High	Master Prc	P04792	Heat shock	0	142.331	72	16	80	16	205	22.8	6.4	207.92	16	cellular co	cytoplasm;	enzyme re	Pf00011	3315	ENSG0000	HSPB1	7	Amoebiasi	Apoptosis-	AUF1 (hnR	19	1.113	0.333291448	7.41	23507.7	22524.8	7.39	6.18	High	High	High	High	High	High	1	
FALSE	High	Master Prc	Q96CV9-1	Optineurin	0	5.782	5	2	2	2	577	65.9	5.24	4.2	2	cell commu	cytoplasm;	metal ion I	Pf11577	10133		OPTN	10			TBC/RABG	9	1.113	0.381084855	21.98	358.2	321.7	18.56	8.74	High	High	High	High	High	High	1	
FALSE	High	Master Prc	Q9P2T1-1	GMP reduc	0	11.176	13	2	2	2	348	37.9	7.23	6.5	2	metabolic	cytosol	catalytic a	Pf00478, P	51292	ENSG0000	GMPR2	14	Purine met		Purine salv	5	1.113	0.488476112	44.01	416.8	374.6	55.59	11.63	High	High	High	High	High	High	1	
FALSE	High	Master Prc	P60903	Protein S10	0	34.938	41	5	13	5	97	11.2	7.37	43.43	5	cell organi:	endoplasm	metal ion I	Pf01023	6281	ENSG0000	S100A10	1		Prostaglan	Dissolution	3	1.113	0.633899524	7.88	5832.9	5566.3	6.77	10.09	High	High	High	High	High	High	1	
FALSE	High	Master Prc	P13807	glycogen [s	0	15.802	8	3	4	3	737	83.7	6.18	17.14	3	metabolic	cytoplasm;	catalytic a	Pf00534, P	2997	ENSG0000	GYS1	19	AMPK sign	AMP-activ	Glycogen s	24	1.113	0.734314371	4.4	400.3	370.3	5.41	10.17	High	High	High	High	High	High	1	
FALSE	High	Master Prc	Q9NV31	U3 small n	0.003	3.125	8	1	1	1	184	21.8	9.5	2.08	1	metabolic	nucleus	protein bir	Pf01479	55272	ENSG0000	IMP3	15	Ribosome		Major path	6	1.112	0.059418211	2.83	358.1	322	3.09	7.52	High	High	High	High	High	High	1	
FALSE	High	Master Prc	Q6P1N0	Coiled-coil	0	17.283	7	4	6	4	951	104	8.09	12.89	4	metabolic	cytoplasm;	DNA bindi	Pf00168	54862	ENSG0000	CC2D1A	19		Sudden Inf		1	1.112	0.53695579	43.87	1378.8	1398.8	43.8	10.26	High	High	High	High	High	High	1	
FALSE	High	Master Prc	P31040	Succinate (	0	139.008	41	20	60	20	664	72.6	7.39	144.44	20	metabolic	membrane	catalytic a	Pf00890, P	6389	ENSG0000	SDHA	5	Alzheimer'	Electron Tr	Respirator	18	1.112	0.625838315	15.95	14686.8	13265.9	14.25	4.3	High	High	High	High	High	High	1	
FALSE	High	Master Prc	Q8IVD9	NudC dom	0	9.293	12	2	2	2	361	40.8	5.25	8.49	2	cell organi:	cytoplasm	protein bir	Pf04969, P	23386	ENSG0000	NUDCD3	7				0	1.112	0.826105353	3.73	398.8	355.5	22.9	5.5	High	High	High	High	High	High	1	
FALSE	High	Master Prc	P14923	Junction pl	0	41.878	18	12	17	11	745	81.7	6.14	24.75	12	cell death;	cytoplasm;	protein bir	Pf00514	3728	ENSG0000	JUP	17	Transcripti	Arrhythmo	Adherens j	22	1.111	0.13087767	9.13	1451.7	1306.5	4.79	3.41	High	High	High	High	High	High	1	
FALSE	High	Master Prc	P29692-3	Isoform 3 (	0	96.779	51	11	39	1	257	28.5	4.92	104.7	11	metabolic	cytoplasm;	DNA bindi	Pf00736, P	1936	ENSG0000	EEF1D	8; CHR_HS			Translation	1	1.111	0.280059962	13.09	157.6	141.8	9.6	11.3	High	High	High	High	High	High	1	[S109(100
FALSE	High	Master Prc	Q96I59	probable a	0	6.202	3	1	2	1	477	54.1	7.24	3.53	1	metabolic	cytoplasm;	catalytic a	Pf00152, P	79731	ENSG0000	NARS2	11	Aminoacyl	Ectoderm I	Mitochonc	6	1.111	0.445445059	4.29	649.9	603.4	9.61	2.18	High	High	High	High	High	High	1	
FALSE	High	Master Prc	O14618	copper cha	0	16.826	16	4	7	4	274	29	5.58	11.77	4	metabolic	cytoplasm;	antioxidan	Pf00080, P	9973	ENSG0000	CCS	11	Amyotropl	Amyotropl	Detoxificat	6	1.111	0.453718142	28.59	1725.6	1560.5	21.63	3.93	High	High	High	High	High	High	1	
FALSE	High	Master Prc	Q15149-1	plectin [OS	0	733.158	38	162	279	157	4684	531.5	5.96	605.54	162	cell organi:	cytoplasm;	protein bir	Pf00307, P	5339	ENSG0000	PLEC	8			Assembly c	11	1.111	0.59321553	10.16	68911.9	62053.3	10.39	4.62	High	High	High	High	High	High	1	
FALSE	High	Master Prc	Q8NFH3	Nucleopori	0	12.224	7	2	2	2	380	42.1	5.63	6.74	2	cell divisio	chromosor	protein bir	Pf00400	348995	ENSG0000	NUP43	6	RNA trans		Rev-media	77	1.111	0.807775983	22.68	433.4	390.2	37.8	9.87	High	High	High	High	High	High	1	
FALSE	High	Master Prc	O95757	Heat shock	0	67.23	23	16	25	10	839	94.5	5.88	52.31	16	metabolic	cytoplasm;	nucleotide	Pf00012	22824	ENSG0000	HSPA4L	4	Protein prc		Regulation	5	1.11	0.085454102	3.03	1019.4	857.6	3.99	7.44	High	High	High	High	High	High	1	
FALSE	High	Master Prc	P08133-1	annexin A6	0	86.081	33	19	24	19	673	75.8	5.6	58.34	19	cell organi:	cytoplasm;	metal ion I	Pf00191	309	ENSG0000	ANXA6	5		Calcium Re	Smooth M	4	1.11	0.247744947	4.45	6666.6	6004.6	24.77	5.2	High	High	High	High	High	High	1	
FALSE	High	Master Prc	O14745	Na(+)/H(+)	0	63.224	50	14	24	14	358	38.8	5.77	37.29	14	cell differe	cytoplasm;	protein bir	Pf00595, P	9368	ENSG0000	SLC9A3R1	17				0	1.11	0.278221218	7.58	5201.8	4732.3	3.85	6.5	High	High	High	High	High	High	1	
FALSE	High	Master Prc	P19623	spermidine	0	29.348	24	6	14	6	302	33.8	5.49	26	6	metabolic	cytosol	catalytic a	Pf01564	6723	ENSG0000	SRM	1	Metabolic	Amino Aci	Metabolisr	11	1.11	0.349761022	2.37	4984.3	4494.4	1.79	7.24	High	High	High	High	High	High	1	
FALSE	High	Master Prc	P22695	Cytochrom	0	111.011	42	16	39	16	453	48.4	8.63	112.64	16	metabolic	membrane	catalytic a	Pf00675, P	7385	ENSG0000	UQCRC2	16; CHR_H	Huntingtor	Electron Tr	Respirator	12	1.11	0.578722531	0.11	16272.6	15724.6	7.45	5.63	High	High	High	High	High	High	1	
FALSE	High	Master Prc	Q9BU61-1	NADH deh	0	17.819	24	3	5	3	184	20.3	8.22	20.64	3	cell organi:	membrane	protein bir	Pf04430	25915	ENSG0000	NDUFAF3	3			Complex I	5	1.11	0.812161968	35.73	797.8	718.9	11.75	17.99	High	High	High	High	High	High	1	
FALSE	High	Master Prc	P52789	Hexokinase	0.001	4.273	4	3	3	3	917	102.3	6.05	0	3	cell organi:	cytosol;me	catalytic a	Pf00349, P	3099	ENSG0000	HK2	2	Galactose	Glycolysis	Glycolysis	18	1.109	0.162299146	6.39	756.9	681.3	7.49	4.44	High	High	High	High	High	High	1	
FALSE	High	Master Prc	O43823	A-kinase ai	0	23.526	10	5	7	5	692	76.1	5.15	19.94	5	cell organi:	chromosor	DNA bindi	Pf04988	10270	ENSG0000	AKAP8	19		G Protein S		1	1.109	0.179380182	10.95	4182.2	3770.2	7.95	2.19	High	High	High	High	High	High	1	
FALSE	High	Master Prc	Q16850-1	lanosterol	0	26.908	16	6	11	6	503	56.8	8.53	18.06	6	metabolic	endoplasm	catalytic a	Pf00067	1595		CYP51A1	7	Steroid bic	Cholestero	Cholestero	16	1.109	0.283371932	13.51	1327.2	1182.1	15.09	5.12	High	High	High	High	High	High	1	
FALSE	High	Master Prc	Q11206	CMP-N-ace	0.002	3.856	3	1	1	1	333	38	9.41	3.41	1	metabolic	extracellul	catalytic a	Pf00777	6484	ENSG0000	ST3GAL4	11	Glycosphir		Sialic acid	23	1.109	0.539324291	40.68	265.5	269.4	38.18	9.17	High	High	High	High	High	High	1	
FALSE	High	Master Prc	O00458	Interferon-	0.002	3.542	5	1	1	1	451	50.2	7.18	0	1	cell differe	cytoplasm;		Pf04836, P	3475	ENSG0000	IFRD1	7		Hypertropl		1	1.108	0.015375357	0.78	62.7	57.4	8.82	7.83	High	High	High	High	High	High	1	
FALSE	High	Master Prc	Q9H857-2	Isoform 2 (	0	25.792	10	4	4	4	557	64.1	7.74	12.57	4	metabolic		catalytic a	Pf05761	64943	ENSG0000	NT5DC2	3				0	1.108	0.040795657	2.9	1180.8	1068.3	5.72	2.12	High	High	High	High	High	High	1	
FALSE	High	Master Prc	P34897-1	Serine hyd	0	170.836	49	23	64	22	504	56	8.53	162.82	23	cell organi:	cytoplasm;	catalytic a	Pf00464	6472	ENSG0000	SHMT2	12	Biosynthes	Vitamin B1	Metabolisr	16	1.108	0.117180618	7.7	15837.5	14264.4	7.1	5.41	High	High	High	High	High	High	1	
FALSE	High	Master Prc	Q9H3R2	Mucin-13 [	0	30.347	13	4	6	4	512	54.6	5.07	18.07	4	metabolic	cytosol;ext	protein bir	Pf01390	56667	ENSG0000	MUC13	3			Defective (	15	1.108	0.253021107	14.35	740.6	646.5	14.44	3.62	High	High	High	High	High	High	1	
FALSE	High	Master Prc	Q9NXG2	THUMP dc	0	11.952	7	2	5	2	353	39.3	7.88	6.9	2	metabolic		protein bir	Pf02926	55623	ENSG0000	THUMPD1	16			rRNA mod	4	1.108	0.984816964	16.82	425.4	435.5	15.78	18.85	High	High	High	High	High	High	1	
FALSE	High	Master Prc	Q14197	Peptidyl-tF	0	19.156	30	5	5	5	206	23.6	10.07	11.71	5	cell organi:	membrane	catalytic a	Pf00472	3396	ENSG0000	ICT1; MRP	17			Mitochonc	6	1.107	0.019958348	3.16	2478.5	2295.5	11.76	11.79	High	High	High	High	High	High	1	
FALSE	High	Protein	P51649-1	semialdeh	0	43.522	22	9	13	9	535	57.2	8.28	29.56	9	organizati	drion;orga	activity;nu	Pf00171	7915	0112294.	ALDH5A1	6	pathways;		on of	8	1.107	0.020897288	2.07	3645.2	3293.9	4.9	8.54	High	High	High	High	High	High	1	
FALSE	High	Master Prc	O43615	Mitochonc	0	42.475	31	13	17	13	452	51.3	8.32	31.34	13	transport	membrane	nucleotide	Pf04280, P	10469	ENSG0000	TIMM44	19			Mitochonc	2	1.107	0.068636805	2.2	4488.3	4294.6	9.08	7.44	High	High	High	High	High	High	1	
FALSE	High	Master Prc	Q4G0N4	NAD kinase	0	15.732	14	6	8	6	442	49.4	8.18	8.45	6	metabolic	mitochond	catalytic a	Pf00781, P	133686	ENSG0000	NADK2; N/	5	Nicotinate		Nicotinate	6	1.107	0.223303216	9.87	154.5	132.8	11.56	4.72	High	High	High	High	High	High	1	
FALSE	High	Master Prc	P56182	Ribosomal	0	12.971	7	3	5	3	461	52.8	9.33	10.07	3	metabolic	nucleus	RNA bindi	Pf05997	8568	ENSG0000	RRP1	21			Major path	4	1.107	0.362848218	16.8	274.1	247.5	29.45	5.74	High	High	High	High	High	High	1	
FALSE	High	Master Prc	P31350-1	ribonucleo	0	20.14	16	5	5	5	389	44.8	5.38	14.49	5	metabolic	cytoplasm;	catalytic a	Pf00268	6241	ENSG0000	RRM2	2	Pyrimidine	Fluoropyri	Activation	22	1.107	0.43266413	25.25	1934.2	1875.8	22.1	8.8	High	High	High	High	High	High	1	
FALSE	High	Master Prc	Q9Y296	Trafficking	0.003	3.346	11	1	1	1	219	24.3	6.21	2.07	1	cell organi:	cytosol;en	protein bir	Pf04099, P	51399	ENSG0000	TRAPPC4	11; CHR_H			COPII (Coa	13	1.107	0.468193286	40.6	252.3	240.9	69.46	17.47	High	High	High	High	High	High	1	
FALSE	Medium	Master Prc	Q32P44-2	Isoform 2 (	0.022	1.574	2	1	2	1	917	97.9	8.97	4.82	1		cytoplasm;	protein bir	Pf00400, P	256364	ENSG0000	EML3	11				0	1.106	0.195276298	5.33	321	282.9	5.31	11.27	High	High	High	High	High	High	1	
FALSE	High	Master Prc	Q13247	Serine/argi	0	35.702	25	10	18	6	344	39.6	11.43	30.34	10	cell organi:	nucleus	protein bir	Pf00076, P	6431	ENSG0000	SRSF6	20	Spliceosom	mRNA Pro	Cleavage o	15	1.106	0.304008298	1.06	2749.4	2732.4	6.82	5.65	High	High	High	High	High	High	1	
FALSE	High	Master Prc	Q7L5Y9-1	Macrophag	0	5.328	8	3	3	3	396	45.3	8.69	2.7	3	cell divisio	cytoplasm;	catalytic a	Pf10607	10296	ENSG0000	MAEA	4				0	1.106	0.385156599	22.41	115.5	100	20.11	6.52	High	High	High	High	High	High	1	
FALSE	High	Master Prc	Q08752	peptidyl-pr	0	25.596	18	6	12	4	370	40.7	7.21	17.08	6	cell death;	cytoplasm;	catalytic a	Pf00160, P	5481	ENSG0000	PPID	4				0	1.106	0.396687344	21.84	2065.8	1867.2	9.06	9.52	High	High	High	High	High	High	1	
FALSE	High	Master Prc	Q53H82	Endoribonu	0	16.258	15	3	3	3	288	32.8	6.8	9.24	3	metabolic	mitochond	catalytic a	Pf00753	51110	ENSG0000	LACTB2	8				0	1.106	0.415699437	23.17	521.1	447.7	39	13.82	High	High	High	High	High	High	1	
FALSE	High	Master Prc	Q9BXW7	Haloacid d	0	18.342	11	3	3	3	423	46.3	8.13	12.84	3	metabolic	mitochond		Pf13344	27440	ENSG0000	CECR5; HD	22				0	1.106	0.485721641	41.77	1062.2	1094.6	58.68	8.56	High	High	High	High	High	High	1	
FALSE	High	Master Prc	P07996	thrombosp	0.002	3.921	2	2	2	2	1170	129.3	4.94	0	2	cell organi:	cell surface	metal ion I	Pf00090, P	7057	ENSG0000	THBS1	15	ECM-recep	Bladder Ca	Platelet de	44	1.106	0.546462402	78.72	522.6	509.6	95.46	27.38	High	High	High	High	High	High	1	
FALSE	High	Master Prc	Q7L5Y1-1	Mitochonc	0	9.058	4	1	1	1	443	49.8	6.48	5.29	1	metabolic	mitochond	catalytic a	Pf01188, P	55556	ENSG0000	ENOSF1	18	Fructose a			1	1.106	0.592766913	43.01	356.6	322.5	29.45	14.6	High	High	High	High	High	High	1	
FALSE	High	Master Prc	P09525	annexin A4	0	43.794	38	10	15	10	319	35.9	6.13	26.07	10	cell differe	cell surface	enzyme re	Pf00191	307		ANXA4	2		Prostaglan		1	1.106	0.674579565	6.1	802.7	847.6	30.35	16.23	High	High	High	High	High	High	1	
FALSE	Medium	Master Prc	Q5JVS0	Intracellula	0.043	1.259	3	1	1	1	413	45.8	7.46	0	1	metabolic	cytoplasm;	protein bir	Pf04774	22927	ENSG0000	HABP4	9			Platelet de	4	1.106	0.854859317	0.04	121.8	113.1	15.81	3.42	High	High	High	High	High	High	1	[R70]
FALSE	High	Master Prc	Q9P0W2	SWI/SNF-r	0	6.132	7	2	2	2	317	35.8	9.35	4.37	2	cell differe	chromosor	catalytic a	Pf00505, P	10362	ENSG0000	HMG20B	19			HDACs dea	5	1.106	0.867285083	21.03	103	115.7	22.24	19.04	High	High	High	High	High	High	1	
FALSE	Medium	Master Prc	A5YM69	Rho guanir	0.024	1.517	5	1	1	1	484	53.3	4.31	0	1				Pf15441	445328	ENSG0000	ARHGEF35	7			G alpha (1:	11	1.105	0.145370127	9.82	319.7	275.9	9.23	3.77	High	High	High	High	High	High	1	
FALSE	High	Master Prc	Q9NUJ1	Mycophen	0	17.777	16	4	5	4	306	33.9	8.57	12.27	4	metabolic	cytosol;mi	catalytic a	Pf00326, P	55347	ENSG0000	ABHD10	3			Glucuronic	4	1.105	0.389253985	1.05	1160.2	1050.1	12.47	4.98	High	High	High	High	High	High	1	
FALSE	High	Master Prc	P02749	Beta-2-gly	0	5.837	6	1	1	1	345	38.3	7.97	3.32	1	metabolic	cell surface	enzyme re	Pf00084, P	350	ENSG0000	APOH	17			Platelet de	4	1.104	0.305838482	8.38	308.4	316.9	11.9	8.38	High	High	High	High	High	High	1	[K324]
FALSE	High	Master Prc	Q9BRA2	Thioredoxi	0	19.964	30	3	8	3	123	13.9	5.52	14.09	3	metabolic	cytoplasm;	antioxidan	Pf06110, P	84817	ENSG0000	TXNDC17	17				0	1.104	0.416857326	23.43	1842.5	1647.7	32.62	11.57	High	High	High	High	High	High	1	
FALSE	High	Master Prc	Q9NX62	Inositol mc	0.002	3.939	4	2	2	2	359	38.7	6.86	2.36	2	metabolic	cytosol;Go	catalytic a	Pf00459	54928	ENSG0000	IMPAD1	8	Inositol ph		Cytosolic s	8	1.104	0.454069067	30.78	641.3	659	47.57	11.26	High	High	High	High	High	High	1	
FALSE	High	Master Prc	P48739	Phosphatic	0	22.37	24	4	5	3	271	31.5	6.87	14.5	4	cell organi:	cytoplasm;	transporte	Pf02121	23760	ENSG0000	PITPNB	22			PI and PC t	6	1.104	0.514827261	40.66	1092.5	989.6	39.49	10.03	High	High	High	High	High	High	1	
FALSE	High	Master Prc	Q969V3	Nicalin [OS	0	26.613	12	4	7	4	563	62.9	6.89	22.26	4	metabolic	endoplasm	protein bir	Pf04389, P	56926	ENSG0000	NCLN	19				0	1.104	0.658730253	18.02	1466.1	1365.9	14.28	6.05	High	High	High	High	High	High	1	
FALSE	High	Master Prc	Q04323-1	UBX doma	0	17.958	16	4	6	4	297	33.3	5.25	7.26	4	metabolic	cytoplasm;	protein bir	Pf00627, P	51035	ENSG0000	UBXN1	11			N-glycan tı	4	1.104	0.689451177	5.1	1193.1	1080.7	4.16	9.51	High	High	High	High	High	High	1	
FALSE	High	Master Prc	P42126	Enoyl-CoA	0	31.875	21	5	9	5	302	32.8	8.54	14.36	5	metabolic	membrane	catalytic a	Pf00378	1632	ENSG0000	ECI1	16	Fatty acid	Fatty Acid	mitochond	8	1.104	0.708854906	0.24	1254.3	1218.5	10.1	19.71	High	High	High	High	High	High	1	
FALSE	High	Master Prc	P60660-2	Isoform Sn	0	97.804	81	11	44	9	151	17	4.55	126.16	11	cellular co	cytosol;me	catalytic a	Pf00036, P	4637	ENSG0000	MYL6	12		Focal Adhe		1	1.103	0.037579614	2.27	4642.9	4208.5	1.16	5.48	High	High	High	High	High	High	1	
FALSE	High	Master Prc	Q13443	disintegrin	0	12.096	5	2	5	2	819	90.5	7.52	19.92	2	cell differe	cell surface	catalytic a	Pf00200, P	8754	ENSG0000	ADAM9	8		Copper ho	Collagen d	4	1.103	0.121467212	8.08	247.9	224.7	8.67	2.79	High	High	High	High	High	High	1	
FALSE	High	Master Prc	Q9P0L0	vesicle-ass	0	48.262	41	10	27	9	249	27.9	8.62	59.19	10	cell death;	cytoskelet	protein bir	Pf00635	9218	ENSG0000	VAPA	18	Tight junct	Exercise-in	Neutrophil	9	1.103	0.164972218	7.87	6100	5529.5	8.2	2.29	High	High	High	High	High	High	1	
FALSE	High	Master Prc	O14656	Torsin-1A [	0	5.515	4	1	2	1	332	37.8	6.99	5.86	1	cell organi:	cytoplasm;	catalytic a	Pf06309	1861	ENSG0000	TOR1A	9			Cargo reco	4	1.103	0.342231752	16.57	590.6	555.7	20.68	4.57	High	High	High	High	High	High	1	
FALSE	High	Master Prc	Q12888-1	TP53-bindi	0	27.246	4	5	5	5	1972	213.4	4.7	12.81	5	cell organi:	chromosor	DNA bindi	Pf09038, P	7158	ENSG0000	TP53BP1	15	NOD-like r	ATM Signa	G2/M DNA	20	1.103	0.43211664	26.96	687.4	623	25.51	11.94	High	High	High	High	High	High	1	
FALSE	High	Master Prc	Q6UW68	Transmem	0.003	3.061	9	1	1	1	189	21.2	8.62	0	1		membrane		Pf13664	374882	ENSG0000	TMEM205	19				0	1.103	0.651405283	0.75	398.9	390.3	7.11	6.75	High	High	High	High	High	High	1	
FALSE	High	Master Prc	O00116	Alkyldihyd	0	28.34	12	5	6	5	658	72.9	7.34	18.71	5	metabolic	membrane	catalytic a	Pf01565, P	8540	ENSG0000	AGPS	2	Ether lipid	Triacylglyc	Plasmaloge	7	1.102	0.030721322	2.79	2088.7	1894.5	1.5	1.83	High	High	High	High	High	High	1	
FALSE	High	Master Prc	Q8IX12	Cell divisio	0	38.361	8	8	13	8	1150	132.7	5.76	23.29	8	cell death;	cytoplasm	DNA bindi	Pf02037, P	55749	ENSG0000	CCAR1	10			mRNA Spli	4	1.102	0.06789517	4.84	2587.1	2347.2	1.84	7.67	High	High	High	High	High	High	1	

FALSE	High	Master Prc	P26885	Peptidyl-pı	0	14.49	19	2	4	2	142	15.6	9.13	10.43	2	metabolic	endoplasm	catalytic a	Pf00254	2286	ENSG0000	FKBP2	11				0	1.102		0.139168773	8.96	2066.1	1874.6	2.18	4.75	High	High	High	High	High	High	1	
FALSE	High	Master Prc	P43034	Platelet-ac	0	13.449	9	3	5	3	410	46.6	7.37	19.19	3	cell commı	chromosor	catalytic a	Pf00400, P	5048	ENSG0000	PAFAH1B1	17	Ether lipid	Wnt Signal	COPI-indep	37	1.102		0.22388281	10.81	2190.1	1956.8	7.69	6.42	High	High	High	High	High	High	1	
FALSE	High	Master Prc	Q14980-1	nuclear mi	0	109.812	14	25	29	25	2115	238.1	5.78	84.14	25	cell differe	chromosor	protein bir	Pf04111, P	4926	ENSG0000	NUMA1	11		Ectoderm	Mitotic Prc	7	1.102		0.393172828	20.61	8684.5	8047.4	25.63	6.74	High	High	High	High	High	High	1	
FALSE	High	Master Prc	Q96AE4-2	Isoform 2 (	0	177.517	49	28	74	2	653	68.6	7.31	134.95	28	metabolic	nucleus	DNA bindi	Pf00013, P	8880	ENSG0000	FUBP1	1				0	1.102		0.406631627	21.48	214.1	221.2	37.24	8.91	High	High	High	High	High	High	1	
FALSE	High	Master Prc	Q9NWZ5-1	Uridine-cyt	0.002	3.641	2	1	1	1	548	61.1	7.36	3.8	1	metabolic	cytoplasm;	catalytic a	Pf00485, P	54963	ENSG0000	UCKL1	20	Metabolic		Pyrimidine	7	1.102		0.419819872	23.52	447.6	406.3	17.06	7.11	High	High	High	High	High	High	1	
FALSE	High	Master Prc	Q9H6U8	Alpha-1,2-	0.002	4.098	2	1	1	1	611	69.8	8.68	3.52	1	metabolic	endoplasm	catalytic a	Pf03901	79796	ENSG0000	ALG9	11	N-Glycan b		Biosynthes	10	1.102		0.88322171	27.24	274.9	330.4	40.33	18.57	High	High	High	High	High	High	1	
FALSE	High	Master Prc	P01892	HLA class I	0	131.685	34	12	41	3	365	40.9	6.99	117.88	12	cell organi	cell surface	protein bir	Pf00129, P	3105; 100!	ENSG0000	HLA-A; LO(	6; CHR_HS		Proteasom	Interferon	25	1.101		0.129946718	7.28	2407.2	2134.7	1.82	9.08	High	High	High	High	High	High	1	
FALSE	High	Master Prc	P11166	Solute carr	0	16.327	10	3	4	3	492	54	8.72	16.14	3	cell commı	cytoplasm;	protein bir	Pf00083, P	6513	ENSG0000	SLC2A1	1	Pathways i	Cori Cycle;	Lactose syı	33	1.101		0.313095554	15.32	764.5	694.5	26.28	10.38	High	High	High	High	High	High	1	
FALSE	High	Master Prc	Q13057	Bifunction	0	52.327	23	9	14	9	564	62.3	6.99	41.15	9	metabolic	cytoplasm;	catalytic a	Pf01121, P	80347	ENSG0000	COASY	17	Metabolic		Coenzyme	7	1.101		0.357443371	8.9	3116.9	2829.9	43.83	0.52	High	High	High	High	High	High	1	
FALSE	High	Master Prc	O15400	Syntaxin-7	0	16.585	20	4	5	4	261	29.8	5.55	9.16	4	cell organi	endosome	protein bir	Pf05739, P	8417	ENSG0000	STX7	6	SNARE inte			2	1.101		0.447185317	25.82	452.5	406.4	16.62	3.68	High	High	High	High	High	High	1	
FALSE	High	Master Prc	Q32MZ4-2	Isoform 2 (	0	35.316	11	6	8	6	784	86.4	4.65	26.43	6	metabolic	cytoplasm;	DNA bindi	Pf09738	9208	ENSG0000	LRRFIP1	2				0	1.101		0.489662617	38.91	2204.1	2002.6	51.1	16.98	High	High	High	High	High	High	1	
FALSE	High	Master Prc	Q9BRQ6	MICOS con	0	9.759	7	1	2	1	235	26.4	8.85	9.13	1	cell organi	cytosol;me	protein bir	Pf05300	84303	ENSG0000	CHCHD6	3			Cristae for	3	1.101		0.545439489	36.75	251.8	228.8	21.93	15.08	High	High	High	High	High	High	1	
FALSE	High	Master Prc	Q9BRX8-1	Redox-regı	0	31.366	28	5	11	5	229	25.7	8.84	23.92	5	metabolic	cytoplasm;	antioxidan	Pf13911	84293	ENSG0000	FAM213A	10				0	1.101		0.911365255	12.01	1402.4	1273.7	12	9.25	High	High	High	High	High	High	1	
FALSE	High	Master Prc	P51114-1	Fragile X m	0	28.978	12	5	10	3	621	69.7	6.15	30.29	5	cell death;	cytoplasm;	protein bir	Pf00013, P	8087	ENSG0000	FXR1	3	RNA trans		Signaling b	5	1.1		0.211569327	6.95	1927.7	1752.6	1.06	6.41	High	High	High	High	High	High	1	
FALSE	High	Master Prc	A4D1E9-1	GTP-bindir	0.001	4.286	4	2	2	2	387	42.9	9.03	3.93	2	metabolic	chromosor	catalytic a	Pf00009, P	85865	ENSG0000	GTPBP10	7				0	1.1		0.283740223	1.54	279.9	271.1	9.6	5.93	High	High	High	High	High	High	1	
FALSE	High	Master Prc	A5PLL7	transmeml	0.007	2.307	6	2	2	2	270	31.1	6.83	2	2	metabolic	cytoplasm;	catalytic a	Pf10520	387521; 3	ENSG0000	TMEM189	20				0	1.1		0.339986624	17.95	259.9	231.7	1.41	15.12	High	High	High	High	High	High	1	
FALSE	High	Master Prc	O00139	Kinesin-like	0	17.611	4	3	5	2	706	79.9	6.68	8.52	3	cell differe	cytoplasm;	catalytic a	Pf00225	3796	ENSG0000	KIF2A	5			COPI-depe	27	1.1		0.354839761	14.17	1412.5	1283.9	34.2	3.35	High	High	High	High	High	High	1	
FALSE	High	Master Prc	P48047	ATP syntha	0	47.204	56	9	18	9	213	23.3	9.96	45.34	9	cell organi	membrane	catalytic a	Pf00213	539	ENSG0000	ATP5O	21	Oxidative	Electron Tı	Cristae for	14	1.1		0.39096052	6.04	3341.9	3132.1	2.12	8.69	High	High	High	High	High	High	1	
FALSE	High	Master Prc	P31153	S-adenosyl	0	74.201	34	14	33	10	395	43.6	6.48	84.52	14	cell organi	cytosol	catalytic a	Pf00438, P	4144	ENSG0000	MAT2A	2	Cysteine ai	Trans-sulfu	Methylatic	10	1.1		0.490887708	16.58	16459	14967.8	5.68	10.12	High	High	High	High	High	High	1	
FALSE	High	Master Prc	P62873	Guanine nı	0	105.523	52	11	30	7	340	37.4	6	106.55	11	cell death;	cytosol;me	catalytic a	Pf00400	2782	ENSG0000	GNB1	1	Dopamine	Nicotine A	G alpha (z)	84	1.1		0.679814108	23.07	5301	4819.2	16.62	5.93	High	High	High	High	High	High	1	
FALSE	High	Master Prc	A3KMH1	von Willeb	0.001	4.193	0	1	2	1	1905	214.7	7.4	7.37	1		mitochond	catalytic a	Pf00092, P	23078	ENSG0000	VWA8	13				0	1.099		0.010067778	0.91	401.7	365.4	4.02	5.82	High	High	High	High	High	High	1	
FALSE	High	Master Prc	P27797	Calreticulir	0	241.664	62	24	101	24	417	48.1	4.44	301.23	24	cell differe	cell surface	DNA bindi	Pf00262	811	ENSG0000	CALR	19	Antigen pr	Androgen	Scavenging	32	1.099		0.018978121	2.14	36738.9	33443.1	1.73	4.03	High	High	High	High	High	High	1	[Y109(100
FALSE	High	Master Prc	Q9BTW9	Tubulin-sp	0	26.359	7	6	6	6	1192	132.5	6.19	13.57	6	cell organi	cytoplasm;	enzyme re	Pf12612	6904	ENSG0000	TBCD	17			Post-chape	3	1.099		0.036687604	1.81	741.4	687.2	2.26	2.82	High	High	High	High	High	High	1	
FALSE	High	Master Prc	P62241	40S riboso	0	49.004	39	8	27	8	208	24.2	10.32	79.43	8	cell organi	cytoplasm;	protein bir	Pf01201	6202	ENSG0000	RPS8	1	Ribosome	Cytoplasm	Peptide ch	38	1.099		0.153840536	9.49	11654.7	10590.4	2.37	6.08	High	High	High	High	High	High	1	
FALSE	High	Master Prc	Q00839	Heterogen	0	350.09	52	49	172	6	825	90.5	6	403.53	49	cell differe	cell surface	DNA bindi	Pf00622, P	3192	ENSG0000	HNRNPU	1	Spliceoson	mRNA Pro	mRNA Spli	6	1.099		0.184558897	1.69	5214.7	4370.4	13.69	5.98	High	High	High	High	High	High	1	
FALSE	High	Master Prc	Q8N490-2	Isoform 2 (	0	6.117	12	1	2	1	142	15.4	10.58	6.21	1	metabolic	membrane	catalytic a		25953	ENSG0000	PNKD	2				0	1.099		0.199318808	6.13	890.2	737.1	9.93	8	High	High	High	High	High	High	1	
FALSE	High	Master Prc	Q9NQR4	Omega-am	0	41.122	35	9	12	9	276	30.6	7.21	23.49	9	metabolic	cytoplasm;	catalytic a	Pf00795	56954	ENSG0000	NIT2	3	Alanine, as		Neutrophil	4	1.099		0.199702795	4.34	2983.5	2766.5	3.4	5.84	High	High	High	High	High	High	1	
FALSE	High	Master Prc	Q14137	Ribosome	0	51.051	25	11	12	11	746	83.6	6.19	32.11	11	cell organi	nucleus	protein bir	Pf00400, P	23246	ENSG0000	BOP1	8			Major path	4	1.099		0.209250095	3.19	1660.8	1511.3	1.67	8.24	High	High	High	High	High	High	1	
FALSE	High	Master Prc	P49790	Nuclear pc	0	55.226	10	11	16	11	1475	153.8	8.73	39.4	11	cell organi	cytosol;me	DNA bindi	Pf00641, P	9972	ENSG0000	NUP153	6	RNA trans	TGF-beta S	Rev-media	65	1.099		0.245769294	5.05	2689.6	2449.3	4.55	3.16	High	High	High	High	High	High	1	
FALSE	High	Master Prc	Q8TB03	Uncharact	0	8.024	4	1	2	1	319	36.6	6.3	3.08	1				Pf15112	159013	ENSG0000	CXorf38	X				0	1.099		0.526176967	12.59	243.8	236	13.68	3.6	High	High	High	High	High	High	1	
FALSE	High	Master Prc	P62328	Thymosin	0	97.455	57	11	64	8	44	5.1	5.06	157.51	11	cell differe	cytoplasm;	catalytic a	Pf01290	7114	ENSG0000	TMSB4X	X	Regulation	TarBasePa	Platelet de	7	1.099		0.575312003	32.11	11692	11872.1	21.04	12.85	High	High	High	High	High	High	1	
FALSE	High	Master Prc	Q7Z7F7	39S riboso	0	8.35	19	2	2	2	128	15.1	11.15	7.32	2	cell organi	membrane	structural	Pf09776	128308	ENSG0000	MRPL55	1			Mitochonc	6	1.098		0.001302564	0.69	711.1	643.8	1.01	0.81	High	High	High	High	High	High	1	
FALSE	High	Master Prc	Q9H9P8-1	L-2-hydrox	0	17.659	10	4	5	4	463	50.3	8.15	13.04	4	metabolic	membrane	catalytic a	Pf01266	79944	ENSG0000	L2HGDH	14	Butanoate		Interconve	5	1.098		0.462028336	26.65	1610.8	1693.5	29.48	8.96	High	High	High	High	High	High	1	
FALSE	High	Master Prc	Q15031	Probable L	0	15.534	6	4	4	4	903	101.9	8.22	10.63	4	cell organi	mitochond	catalytic a	Pf00133, P	23395	ENSG0000	LARS2	3	Aminoacyl	Amino Aci	Mitochonc	6	1.098		0.480523352	1.65	763.2	719.2	6.5	3.55	High	High	High	High	High	High	1	
FALSE	High	Master Prc	P31942	Heterogen	0	137.57	64	13	44	12	346	36.9	6.87	118.89	13	cell differe	nucleus;sp	protein bir	Pf00076, P	3189	ENSG0000	HNRNPH3	10				0	1.098		0.531095131	25.34	8332.3	7474.2	17.87	1.52	High	High	High	High	High	High	1	
FALSE	High	Master Prc	Q9NRX2	39S riboso	0	11.831	21	3	6	3	175	20	10.11	11.41	3	cell organi	membrane	protein bir	Pf01196	63875	ENSG0000	MRPL17	11	Ribosome		Mitochonc	7	1.098		0.732747697	44.85	909.5	930.6	25.19	17.87	High	High	High	High	High	High	1	
FALSE	High	Master Prc	O00401	Neural Wis	0	6.538	3	1	1	1	505	54.8	7.93	4.98	1	cell divisio	cytoplasm;	catalytic a	Pf00568, P	8976; 142(	ENSG0000	WASL; ASE	7	Regulation	Pathogeni	Nephrin fa	37	1.098		0.931024647	13.7	333.1	395.9	23.96	15.12	High	High	High	High	High	High	1	
FALSE	High	Master Prc	Q16718	NADH deh	0	32.124	53	6	11	6	116	13.5	5.99	32.01	6	cell organi	membrane	catalytic a	Pf04716	4698	ENSG0000	NDUFA5	7	Oxidative	Electron Tı	Complex I	13	1.097		0.008522912	0.2	3116.9	2812.2	9.8	9.36	High	High	High	High	High	High	1	
FALSE	High	Master Prc	O43504	Ragulator	0	24.344	33	2	4	2	91	9.6	4.87	12.71	2	cell organi	cytoplasm;	protein bir		10542	ENSG0000	LAMTOR5	1	Hepatitis E		Energy dep	17	1.097		0.046672335	3.17	1626.4	1487.4	3.96	2.14	High	High	High	High	High	High	1	
FALSE	High	Master Prc	P51812	Ribosomal	0	39.321	17	9	11	3	740	83.7	6.89	24.66	9	cell death;	cytoplasm;	catalytic a	Pf00069, P	6197	ENSG0000	RPS6KA3	X	Neurotrop	Brain-Deri	CREB phos	62	1.097		0.158257842	4.48	267	237.4	7.01	4.59	High	High	High	High	High	High	1	[Q138]
FALSE	High	Master Prc	Q08AM6	Protein VA	0.002	4.18	2	1	1	1	782	87.9	6.13	3.1	1	metabolic	endoplasm	protein bir	Pf11916, P	55697	ENSG0000	VAC14	16	Viral carcir		Synthesis c	9	1.097		0.353926333	1.38	355.9	339.8	6.37	3.02	High	High	High	High	High	High	1	
FALSE	High	Master Prc	P16949-1	Stathmin [	0	95.669	75	15	53	15	149	17.3	5.97	123.99	15	cell differe	cytoplasm;	protein bir	Pf00836, P	3925	ENSG0000	STMN1	1	MicroRNA	Regulation		7	1.097		0.465753984	0.27	24508.2	22731.9	2.36	6.38	High	High	High	High	High	High	1	
FALSE	High	Master Prc	Q15388	Mitochonc	0	4.978	9	1	3	1	145	16.3	8.6	7.23	1	cell commı	membrane	protein bir	Pf02064	9804	ENSG0000	TOMM20	1			Ub-specific	7	1.097		0.468962173	31.04	910.1	846.9	38.8	10.44	High	High	High	High	High	High	1	
FALSE	High	Master Prc	P13804-1	Electron tr	0	156.318	67	18	53	18	333	35.1	8.38	138.07	18	metabolic	mitochond	catalytic a	Pf00766, P	2108	ENSG0000	ETFA	15			Respirator	4	1.097		0.739372414	12.32	14327.7	13061.4	12.87	4.45	High	High	High	High	High	High	1	
FALSE	High	Master Prc	Q9NRG0	Chromatin	0.003	2.983	11	1	1	1	131	14.7	5.1	2.55	1	cell organi	nucleus	catalytic a	Pf00808	54108	ENSG0000	CHRAC1	8				0	1.097		0.854110516	8.15	391.3	360.9	27.8	5.33	High	High	High	High	High	High	1	
FALSE	High	Master Prc	Q9Y6K9	NF-kappa-l	0	9.413	7	3	3	3	419	48.2	5.71	6.44	3	cell death;	cytoplasm;	metal ion l	Pf04111, P	8517	ENSG0000	IKBKG	X	Chagas dis	Nucleotide	IRAK1 recr	129	1.096		0.099856371	4.56	582.5	508.9	5.83	7.47	High	High	High	High	High	High	1	
FALSE	Low	Master Prc	O95857	Tetraspani	0.056	1.147	3	1	2	1	204	22.1	7.93	1.72	1	regulation	membrane		Pf00335	27075	ENSG0000	TSPAN13	7				0	1.096		0.253149561	12.94	523	477.2	5.91	6.34	High	High	High	High	High	High	1	
FALSE	High	Master Prc	P61978-3	Isoform 3 (	0	248.041	63	28	141	1	440	48.5	5.54	368.48	28	metabolic	cytoplasm;	DNA bindi	Pf00013, P	3190		HNRNPK	9		mRNA Pro		1	1.096		0.293180862	14.37	2069.5	1868.2	7.3	5.14	High	High	High	High	High	High	1	
FALSE	High	Master Prc	P05783	Keratin, ty	0	332.012	71	34	149	34	430	48	5.45	421.4	34	cell death;	cytoplasm;	protein bir	Pf00038	3875	ENSG0000	KRT18	12	Pathogeni	Pathogeni	Formation	5	1.096		0.594557941	17.49	54105.3	49348.8	5.74	10.61	High	High	High	High	High	High	1	
FALSE	High	Master Prc	P51148	Ras-relatec	0	65.092	54	8	20	6	216	23.5	8.41	63.75	8	regulation	endosome	catalytic a	Pf00009, P	5878	ENSG0000	RAB5C	17	Vasopressi		TBC/RABG	21	1.096		0.884825976	41.7	3255.6	2971.6	34.69	15.31	High	High	High	High	High	High	1	
FALSE	High	Master Prc	Q9BRQ0	Pygopus h	0	5.77	5	1	1	1	406	41.2	7.28	4.47	1	cell organi	nucleus	enzyme re	Pf00628	90780	ENSG0000	PYGO2	1			Formation	5	1.095		0.172270411	3.95	208.4	181	6.69	7.28	High	High	High	High	High	High	1	
FALSE	High	Master Prc	Q92945	Far upstrea	0	181.695	49	29	69	24	711	73.1	7.3	165.75	29	metabolic	cytoplasm;	DNA bindi	Pf00013, P	8570	ENSG0000	KHSRP	19		Preimplant	ATF4 activ	8	1.095		0.261927578	7.06	14520.1	13261.3	15.11	3.9	High	High	High	High	High	High	1	[Q60]
FALSE	High	Master Prc	Q16775-1	Hydroxyac	0	12.929	5	1	2	1	308	33.8	8.12	7.75	1	metabolic	cytoplasm;	catalytic a	Pf00753	3029	ENSG0000	HAGH	16	Pyruvate n			1	1.095		0.416529943	21.36	695.7	633.4	18.01	9.67	High	High	High	High	High	High	1	
FALSE	High	Master Prc	Q7Z5L9-2	Isoform 2 (	0	45.57	22	8	13	6	571	59.4	8.6	35.46	8	metabolic	cytoplasm;	metal ion l	Pf11261	359948	ENSG0000	IRF2BP2	1				0	1.094		0.008430916	0.29	1491.7	1365.7	5.51	4.46	High	High	High	High	High	High	1	
FALSE	High	Master Prc	P63220	40S riboso	0	38.721	51	5	18	5	83	9.1	8.5	34.21	5	cell organi	cytosol;rib	protein bir	Pf01249	6227	ENSG0000	RPS21	20	Ribosome	Cytoplasm	Peptide ch	38	1.094		0.012747823	0.89	8185.6	7527.4	6.26	5.42	High	High	High	High	High	High	1	
FALSE	Medium	Master Prc	Q5JTY5	COBW don	0.012	1.957	3	1	1	1	395	44	4.87	2.26	1			nucleotide	Pf02492, P	445571; 2	ENSG0000	CBWD3; C	9				0	1.094		0.149046223	4.06	308.2	283.1	3.8	1.85	High	High	High	High	High	High	1	
FALSE	High	Master Prc	P51610-1	Host cell fa	0	42.265	7	11	12	11	2035	208.6	7.46	27.75	11	cell organi	cytoplasm;	catalytic a	Pf01344, P	3054	ENSG0000	HCFC1	X	Herpes sim	Mitochonc	Transcripti	12	1.094		0.277845276	8.84	1638.3	1497.6	13.16	7.04	High	High	High	High	High	High	1	
FALSE	High	Master Prc	Q8NC60	Nitric oxid	0.003	3.164	2	1	1	1	698	78.4	8.66	2.69	1	cell death;	membrane	catalytic a	Pf01926, P	84273	ENSG0000	NOA1	4				0	1.094		0.323474657	11.73	264.3	259.6	27.42	7.79	High	High	High	High	High	High	1	
FALSE	High	Master Prc	Q10567	AP-1 comp	0	58.358	16	12	22	5	949	104.6	5.06	44.88	12	regulation	cytosol;Go	protein bir	Pf01602, P	162		AP1B1	22	Lysosome		Nef media	17	1.094		0.354010451	17.52	1715.1	1578	7.97	5.63	High	High	High	High	High	High	1	
FALSE	Medium	Master Prc	Q9C093	Sperm flag	0.043	1.262	0	1	1	1	1822	209.7	5.54	0	1			protein bir	Pf00406, P	79925	ENSG0000	SPEF2	5				0	1.093		0.078090088	5.18	1748.2	1591.6	0.35	5.25	High	High	High	High	High	High	1	
FALSE	High	Master Prc	O60763-1	General ve	0	45.3	7	5	15	5	962	107.8	4.91	37.42	5	cell organi	cytoplasm;	protein bir	Pf02403, P	8615	ENSG0000	USO1	4			COPII (Coa	14	1.093		0.182746251	10.69	3324.9	2989	6.11	2.99	High	High	High	High	High	High	1	
FALSE	High	Master Prc	P05166-1	propionyl-	0	10.634	7	3	4	3	539	58.2	7.64	9.01	3	metabolic	cytosol;mi	catalytic a	Pf01039	5096	ENSG0000	PCCB	3	Carbon me		Defective I	18	1.093		0.216727654	3.21	600.8	549.6	1.36	7.59	High	High	High	High	High	High	1	
FALSE	High	Master Prc	Q96M27-1	protein PR	0	29.619	13	4	7	4	445	46.7	5.83	19.96	4		Golgi	protein bir	Pf01931	133619	ENSG0000	PRRC1	5				0	1.093		0.225893787	10.84	3894.1	3539.7	6.52	5.51	High	High	High	High	High	High	1	
FALSE	High	Master Prc	Q9BT78	COP9 signa	0	30.25	25	8	9	8	406	46.2	5.83	18.49	8	cell organi	cytoplasm;	catalytic a	Pf01399	51138	ENSG0000	COPS4	4			Cargo reco	13	1.093		0.241542509	3.23	883.6	818.6	6.77	1.19	High	High	High	High	High	High	1	
FALSE	High	Master Prc	Q9NYL9	tropomodu	0	28.414	22	6	9	5	352	39.6	5.19	21.96	6	cell organi	cytoplasm;	protein bir	Pf03250	29766	ENSG0000	TMOD3	15			Striated M	2	1.093		0.345455767	13.67	3020.7	2752.9	19.55	7.96	High	High	High	High	High	High	1	
FALSE	High	Master Prc	Q9UBM7	7-dehydro	0	8.035	6	3	4	3	475	54.5	8.7	2.15	3	cell differe	cytosol;en	catalytic a	Pf01222	1717	ENSG0000	DHCR7	11	Steroid bic	Vitamin D	Cholestero	12	1.093		0.360414605	15.63	2589	2170.5	16.26	12.89	High	High	High	High	High	High	1	
FALSE	High	Master Prc	P31431-1	syndecan-4	0	5.574	11	2	4	2	198	21.6	4.5	3.83	2	cell organi	cell surface	protein bir	Pf01034	6385	ENSG0000	SDC4	20	ECM-recep		Defective I	30	1.093		0.391806594	15.51	1019.7	851.9	47.96	10.57	High	High	High	High	High	High	1	
FALSE	High	Master Prc	P33897	ATP-bindir	0.003	2.944	2	1	1	1	745	82.9	8.95	2.35	1	cell organi	cytoplasm;	catalytic a	Pf00005, P	215	ENSG0000	ABCD1	X	ABC transp		Defective	17	1.093		0.429509003	21.9	232.1	236.2	18.84	6.16	High	High	High	High	High	High	1	
FALSE	High	Master Prc	Q8WVC0-1	RNA polym	0	10.683	3	1	1	1	666	75.4	4.51	5.14	1	cell organi	nucleus	protein bir	Pf04004, P	123169	ENSG0000	LEO1	15		Endoderm	E3 ubiquiti	14	1.093		0.49863352	30.17	193.8	200.1	30.61	7.56	High	High	High	High	High	High	1	
FALSE	High	Master Prc	Q9NRX4	14 kDa pho	0	12.154	24	3	4	3	125	13.8	6.07	11.46	3	metabolic	cytoplasm;	catalytic a	Pf05005	29085	ENSG0000	PHPT1	9				0	1.093		0.499141255	24.66	2132.9	1940.2	6.17	14.59	High	High	High	High	High	High	1	
FALSE	High	Master Prc	Q7RTV0	PHD finger	0.003	3.176	15	2	2	2	110	12.4	8.41	0	2	metabolic	nucleus;sp	DNA bindi	Pf03660	84844	ENSG0000	PHF5A	22	Spliceoson	mRNA Pro	mRNA Spli	6	1.093		0.576831152	43.54	297.2	272.1	32.48	18.08	High	High	High	High	High	High	1	
FALSE	High	Master Prc	Q02539	Histone H1	0	44.841	20	6	25	1	215	21.8	10.99	37.15	6	cell organi	cell surface	DNA bindi	Pf00538	3024	ENSG0000	HIST1H1A	6			Formation	10	1.093		0.605981246	14.11	648	606.7	13.59	2.61	High	High	High	High	High	High	1	
FALSE	High	Master Prc	P12694	2-oxoisova	0	5.786	4	1	1	1	445	50.4	8.27	4	1	metabolic	mitochond	catalytic a	Pf00456, P	593	ENSG0000	BCKDHA	19	Valine, leu		Branched-	7	1.093		0.704817099	43.81	166.8	152.6	19.01	15.03	High	High	High	High	High	High	1	
FALSE	High	Master Prc	Q8TBB5	kelch dom	0	15.917	12	4	4	4	520	57.9	5.72	7.95	4			protein bir	Pf01344, P	54758	ENSG0000	KLHDC4	16				0	1.093		0.769976821	61.86	819.7	751.5	37.44	30.05	High	High	High	High	High	High	1	
FALSE	High	Master Prc	Q6ZRP7	Sulfhydryl	0	25.458	9	4	7	4	698	77.5	7.72	27.15	4	cellular ho	extracellul	catalytic a	Pf00085, P	169714	ENSG0000	QSOX2	9				0	1.093		0.878423307	8.26	1714	1621.9	13.54	11.87	High	High	High	High	High	High	1	
FALSE	High	Master Prc	P21964-1	Catechol O	0	14.739	18	3	4	3	271	30	5.47	12.34	3	cell commı	cytoplasm;	catalytic a	Pf01596, P	1312	ENSG0000	COMT	22	Tyrosine m	Dopamine	Enzymatic	19	1.093		0.966109797	12.76	470.6	434.6	10.42	11.76	High	High	High	High	High	High	1	
FALSE	High	Master Prc	P38159-1	RNA-bindi	0	141.063	59	26	106	9	391	42.3	10.05	179.62	26	cell differe	membrane	DNA bindi	Pf00076, P	27316	ENSG0000	RBMX	X	Spliceoson	mRNA Pro	mRNA Spli	6	1.092		0.011379228	1.99	12876.1	11859.3	3.32	3.24	High	High	High	High	High	High	1	
FALSE	High	Master Prc	Q96RP9	Elongation	0	57.045	23	13	21	13	751	83.4	7.01	42.13	13	metabolic	mitochond	catalytic a	Pf00009, P	85476	ENSG0000	GFM1	3			Mitochonc	4	1.092		0.062786504	5.32	5927.3	5635.6	6.69	4.82	High	High	High	High	High	High	1	
FALSE	High	Master Prc	Q16851	UTP--glucc	0	33.718	21	8	10	8	508	56.9	8.15	24.11	8	metabolic	cytoplasm;	catalytic a	Pf01704	7360	ENSG0000	UGP2	2	Pentose ar	Exercise-in	Formation	16	1.092		0.120337968	0.2	2060.6	2028.3	4.85	4.39	High	High	High	High	High	High	1	

FALSE	High	Master Prc	Q8TED1	Probable g	0	12.012	16	3	5	3	209	23.9	9.35	12.58	3	metabolic	membrane	antioxidan	Pf00255, P	493869	ENSG0000	GPX8	5	Thyroid ho		Detoxificat	6	1.092	0.169914909	9.3	463.3	420.1	0.59	8.53	High	High	High	High	High	High	1	
FALSE	High	Master Prc	O60568	Procollage	0	44.709	22	12	16	12	738	84.7	6.05	37.19	12	cell organi	endoplasm	catalytic a	Pf03171	8985	ENSG0000	PLOD3	7	Other type		Collagen b	5	1.092	0.277085227	8.89	3275	2895.9	2.47	8.61	High	High	High	High	High	High	1	
FALSE	High	Master Prc	Q8WYA6	Beta-caten	0	31.174	14	9	11	9	563	65.1	5.05	19.7	9	cell death;	cytoplasm;	protein bir	Pf08216	56259	ENSG0000	CTNNBL1	20	Spliceosom		mRNA Spli	5	1.092	0.394996548	19.19	2035.2	1933.7	18.61	7.29	High	High	High	High	High	High	1	
FALSE	High	Master Prc	Q9HD45	Transmem	0.003	3.343	2	1	1	1	589	67.8	7.21	1.92	1		membrane		Pf02990	56889	ENSG0000	TM9SF3	10				0	1.092	0.428807747	22.22	673.4	595.6	21.63	13.62	High	High	High	High	High	High	1	
FALSE	High	Master Prc	Q9BV40	vesicle-ass	0	5.012	8	1	2	1	100	11.4	7.34	4.98	1	cell organi	cytoplasm;	protein bir	Pf00957	8673	ENSG0000	VAMP8	2	SNARE inte		Cargo reco	17	1.092	0.432579459	3.22	1176.8	1148.1	7.84	3.83	High	High	High	High	High	High	1	
FALSE	High	Master Prc	Q07960	rho GTPase	0	22.371	17	5	9	5	439	50.4	6.29	21.28	5	regulation	cytoplasm;	enzyme re	Pf00620, P	392	ENSG0000	ARHGAP1	11			Rho GTPas	3	1.092	0.487746847	4.79	4828.9	4424	2.2	8.22	High	High	High	High	High	High	1	
FALSE	High	Master Prc	P54886	delta-1-pyı	0	181.652	44	25	61	25	795	87.2	7.12	159.6	25	metabolic	cytoplasm;	catalytic a	Pf00171, P	5832	ENSG0000	ALDH18A1	10	Metabolic	Amino Aci	Amino acic	8	1.092	0.669746878	5.41	18257	17520.9	6.73	7.39	High	High	High	High	High	High	1	
FALSE	High	Master Prc	Q9NZN4	EH domain	0	15.698	10	5	8	4	543	61.1	6.46	15.28	5	cell organi	cytoplasm;	catalytic a	Pf00350, P	30846	ENSG0000	EHD2	19	Endocytosi	Insulin Sigı	Factors inv	4	1.092	0.986358352	0.92	1913.3	1867.5	11.41	6.41	High	High	High	High	High	High	1	
FALSE	High	Master Prc	Q04941-1	proteolipic	0	5.378	9	1	4	1	152	16.7	7.24	14.22	1	regulation	endoplasm	protein bir	Pf01284	5355	ENSG0000	PLP2	X				0	1.091	0.150479644	4.82	1874.7	1675.4	4	9.27	High	High	High	High	High	High	1	
FALSE	High	Master Prc	O95168-1	NADH deh	0	10.996	36	4	5	3	129	15.2	9.85	0	4	cell organi	membrane	catalytic a	Pf07225	4710	ENSG0000	NDUFB4	3	Oxidative p	Electron Tı	Complex I	13	1.091	0.156999555	6.21	236	225.7	7.89	16.07	High	High	High	High	High	High	1	
FALSE	High	Master Prc	P09622	Dihydrolip	0	107.396	40	14	45	14	509	54.1	7.85	119.85	14	cellular ho	mitochond	catalytic a	Pf00070, P	1738	ENSG0000	DLD	7	Metabolic	Fatty Acid	Glycine de	29	1.091	0.164889044	7.16	16885.1	15928.5	5.32	2.5	High	High	High	High	High	High	1	
FALSE	High	Master Prc	Q08945	FACT comp	0	58.913	19	13	22	13	709	81	6.87	51.85	13	metabolic	chromosor	DNA bindi	Pf00505, P	6749	ENSG0000	SSRP1	11			HIV elonga	24	1.091	0.397501766	3.74	6778.6	6383.4	2.23	6.98	High	High	High	High	High	High	1	
FALSE	High	Master Prc	Q9Y4C2-1	TRPM8 cha	0.007	2.239	1	1	1	1	921	102.1	6.54	2.28	1	regulation	membrane	protein bir	Pf13402	9747	ENSG0000	FAM115A;	7				0	1.091	0.403189646	20.03	612.5	561.2	17.48	7.94	High	High	High	High	High	High	1	
FALSE	High	Master Prc	P82932	28S riboso	0	12.525	44	4	5	4	125	14.2	9.26	6.78	4	cell organi	membrane	protein bir	Pf01250	64968	ENSG0000	MRPS6	21	Ribosome		Mitochonc	7	1.091	0.407752718	19.23	961	925.7	35.59	9.43	High	High	High	High	High	High	1	
FALSE	High	Master Prc	Q6UVK1	Chondroiti	0	37.488	5	8	11	8	2322	250.4	5.47	22.94	8	cell differe	cell surface	protein bir	Pf00054, P	1464	ENSG0000	CSPG4	15		Spinal Cor	A tetrasacc	19	1.091	0.462431606	26.86	1098.9	1007	28.54	8.53	High	High	High	High	High	High	1	
FALSE	Medium	Master Prc	Q9NXG0-1	Centlein [C	0.02	1.612	1	1	2	1	1405	161.5	8.15	4.98	1	cell organi	cytoplasm;	protein bir	Pf02463, P	54875		CNTLN	9				0	1.091	0.579005653	28.65	601.6	588.2	4.05	23.28	High	High	High	High	High	High	1	[K1049;
FALSE	High	Master Prc	Q15125	3-beta-hyc	0	12.501	5	1	3	1	230	26.3	7.9	9.8	1	metabolic	endoplasm	catalytic a	Pf05241	10682	ENSG0000	EBP	X	Metabolic		Cholestero	8	1.091	0.849630786	0.78	703	656.4	9.01	17.65	High	High	High	High	High	High	1	
FALSE	High	Master Prc	P01891	HLA class I	0	117.398	36	11	38	2	365	40.9	6.7	104.9	11	regulation	cell surface	protein bir	Pf00129, P	3105		HLA-A	6		Proteasom	Interferon	14	1.09	0.399528745	22.06	235.1	212.9	27.58	11.23	High	High	High	High	High	High	1	
FALSE	High	Master Prc	O15027-1	Protein tra	0	41.564	6	9	16	9	2179	233.4	5.63	32.09	9	cell organi	cytosol;en	protein bir	Pf12931, P	9919		SEC16A	9			COPII (Coa	8	1.09	0.465866703	23.26	995.4	913.2	12.77	7.96	High	High	High	High	High	High	1	
FALSE	High	Master Prc	P55789	FAD-linked	0	9.977	18	2	2	2	205	23.4	7.62	4.38	2	metabolic	cytoplasm;	catalytic a	Pf04777	2671	ENSG0000	GFER	16			Mitochonc	2	1.09	0.497940898	29.67	420	399.8	28.21	9.48	High	High	High	High	High	High	1	
FALSE	High	Master Prc	Q99729-3	Isoform 3 c	0	131.442	54	19	68	17	285	30.6	7.91	195.79	19	cell organi	cytoplasm;	DNA bindi	Pf00076, P	3182	ENSG0000	HNRNPAB	5		Preimplant		2	1.09	0.629921726	3.87	17330.7	16503.3	6.89	5.29	High	High	High	High	High	High	1	[R275;
FALSE	High	Master Prc	P17813	Endoglin [(	0	31.263	11	6	10	6	658	70.5	6.61	25.66	6	cell differe	cell surface	catalytic a	Pf00100	2022	ENSG0000	ENG	9		Hypothesi:		2	1.09	0.9547117	5.78	2006.7	1886.7	9.15	8.61	High	High	High	High	High	High	1	
FALSE	High	Master Prc	Q02218	2-oxogluta	0	171.861	31	26	44	26	1023	115.9	6.86	125.15	26	cellular coı	cytosol;me	catalytic a	Pf00676, P	4967	ENSG0000	OGDH	7	Tryptophaı	TCA Cycle	Lysine cata	17	1.089	0.287797457	14.2	11835.7	10724.7	5	7	High	High	High	High	High	High	1	
FALSE	High	Master Prc	Q9NRR5-1	Ubiquilin-4	0	16.642	9	4	8	2	601	63.8	5.22	15.16	4	metabolic	cytoplasm;	protein bir	Pf00240, P	56893	ENSG0000	UBQLN4	1	Protein prc			1	1.089	0.30156471	0.48	428.5	396.1	6.84	1.4	High	High	High	High	High	High	1	
FALSE	High	Master Prc	Q96T37-1	Putative RI	0	41.759	14	8	10	8	977	107.1	10.08	23.28	8	metabolic	membrane	protein bir	Pf00076, P	64783	ENSG0000	RBM15	1				0	1.089	0.443302481	24.85	1581.2	1452.3	32.26	9.31	High	High	High	High	High	High	1	
FALSE	High	Master Prc	Q9H6S0	Probable A	0	9.418	2	2	2	2	1430	160.1	8.4	2.58	2	metabolic	cytoplasm;	catalytic a	Pf00271, P	64848	ENSG0000	YTHDC2	5				0	1.089	0.462205236	32.36	164.4	151	49.19	8.21	High	High	High	High	High	High	1	
FALSE	High	Master Prc	Q9BXS6	Nucleolar a	0.003	3.443	2	1	1	1	441	49.4	9.91	3.33	1	cell divisio	chromosor	DNA bindi		51203	ENSG0000	NUSAP1	15				0	1.089	0.480739719	28.81	865.4	794.6	28.38	8.75	High	High	High	High	High	High	1	
FALSE	High	Master Prc	Q8IU81	Interferon	0	16.305	11	5	5	5	584	61.6	8.18	9.23	5	metabolic	nucleus	catalytic a	Pf00097, P	26145	ENSG0000	IRF2BP1	19				0	1.089	0.551743078	41.76	313.1	287.5	39.06	13.38	High	High	High	High	High	High	1	
FALSE	High	Master Prc	Q9UJ70	N-acetyl-D	0	11.12	11	3	3	3	344	37.4	6.24	4.21	3	metabolic	cytosol	catalytic a	Pf01869	55577	ENSG0000	NAGK	2	Metabolic		Synthesis c	8	1.089	0.553853194	42.14	366.4	339.7	40.73	13.13	High	High	High	High	High	High	1	
FALSE	High	Master Prc	O95571	Persulfide	0	31.882	46	6	9	6	254	27.9	6.83	30.22	6	metabolic	cytoplasm;	catalytic a	Pf00753, P	23474	ENSG0000	ETHE1	19	Sulfur met		Sulfide oxi	6	1.088	0.024181867	0.23	1167.1	1074.7	4.13	2.12	High	High	High	High	High	High	1	
FALSE	High	Master Prc	Q96AJ9-1	Isoform 1 c	0.006	2.352	11	1	1	1	203	23.4	6.15	2.21	1	cell organi	cytosol;en	protein bir	Pf05008, P	143187	ENSG0000	VTI1A	10				0	1.088	0.070010868	0.71	239.6	221.2	6.93	12.29	High	High	High	High	High	High	1	
FALSE	High	Master Prc	P36776	Lon protea	0	52.412	18	13	18	13	959	106.4	6.39	30.37	13	cell organi	cytosol;me	catalytic a	Pf00004, P	9361	ENSG0000	LONP1	19				0	1.088	0.071490928	0.58	2888.1	2653.4	4.97	8.06	High	High	High	High	High	High	1	
FALSE	High	Master Prc	Q9NYU2-1	UDP-gluco	0	67.337	12	16	22	16	1555	177.1	5.63	49.57	16	metabolic	endoplasm	catalytic a	Pf06427	56886	ENSG0000	UGGT1	2	Protein prc		ER Quality	7	1.088	0.113428869	6.15	3508.7	3357.6	6.5	2.65	High	High	High	High	High	High	1	
FALSE	High	Master Prc	Q14116	Interleukin	0	13.649	13	3	6	3	193	22.3	4.67	5.87	3	cell commı	cytosol;ext	protein bir	Pf00340	3606	ENSG0000	IL18	11	Influenza A	Nucleotide	Interleukin	25	1.088	0.233064464	11.25	920.4	806.4	5.18	7.86	High	High	High	High	High	High	1	
FALSE	High	Master Prc	Q9GZT3-1	SRA stem-l	0	40.632	67	6	19	6	109	12.3	10.24	54.5	6	cell organi	cytoplasm;	protein bir	Pf00076, P	81892	ENSG0000	SLIRP	14				0	1.088	0.245371137	1.96	9182.5	8836.3	4.32	3.71	High	High	High	High	High	High	1	
FALSE	High	Master Prc	Q9H488-1	GDP-fucos	0.003	3.251	3	1	1	1	388	43.9	8.53	3.23	1	metabolic	endoplasm	catalytic a	Pf10250	23509	ENSG0000	POFUT1	20	Other type		Pre-NOTCH	5	1.088	0.366380073	14.47	501.9	466.7	25.1	6.94	High	High	High	High	High	High	1	
FALSE	High	Master Prc	Q9NQC3-2	Isoform 2 c	0	48.57	21	6	17	6	373	40.3	4.78	49.47	6	cell death;	endoplasm	protein bir	Pf02453	57142	ENSG0000	RTN4	2		Spinal Cor		1	1.088	0.567349654	10.87	6394.7	5891.3	2.1	10.49	High	High	High	High	High	High	1	
FALSE	High	Master Prc	P07686	Beta-hexos	0	13.601	8	4	4	4	556	63.1	6.76	8.27	4	cell organi	extracellul	catalytic a	Pf00728, P	3074		HEXB	5	Metabolic		Neutrophil	22	1.088	0.613903623	19.67	1012.8	1018.5	8.43	12.95	High	High	High	High	High	High	1	
FALSE	High	Master Prc	Q8N3E9	1-phospha	0	5.116	3	2	2	2	789	89.2	6.98	1.85	2	metabolic	cytoplasm;	catalytic a	Pf00168, P	113026	ENSG0000	PLCD3	17	Inositol ph	GPR40 PA	Synthesis c	10	1.088	0.676923654	3.41	1727.9	1709.6	8.89	5.73	High	High	High	High	High	High	1	
FALSE	High	Master Prc	P04179	Superoxide	0	44.476	28	4	12	4	222	24.7	8.25	38.18	4	cell organi	mitochond	antioxidan	Pf00081, P	6648		SOD2	6	Longevity	Selenium N	Gene and	26	1.088	0.751000971	1.49	4383.4	4128.7	7.32	16.2	High	High	High	High	High	High	1	
FALSE	High	Master Prc	P10599-1	thioredoxi	0	58.691	66	8	43	8	105	11.7	4.92	96.17	8	cell commı	cytoplasm;	antioxidan	Pf00085, P	7295	ENSG0000	TXN	9	NOD-like r	Selenium N	Interconve	25	1.088	0.801439922	1.46	18923.4	17230.5	31.59	7.11	High	High	High	High	High	High	1	
FALSE	High	Master Prc	Q9BWU0	kanadaptir	0	9.364	5	3	3	3	796	88.8	5.19	6.63	3		cytoplasm;	protein bir	Pf00035, P	22950	ENSG0000	SLC4A1AP	2				0	1.088	0.874991715	8.72	1066.6	980.5	2.6	18.43	High	High	High	High	High	High	1	
FALSE	High	Master Prc	Q6WKZ4-4	Rab11 fam	0	11.782	3	3	3	3	1283	137.1	5.43	3.16	3	regulation	cytoplasm;	protein bir	Pf00168, P	80223	ENSG0000	RAB11FIP1	8	Endocytosi			1	1.087	0.278658092	13.71	506.7	460.9	4.34	6.74	High	High	High	High	High	High	1	
FALSE	High	Master Prc	O60547-1	GDP-mann	0	8.952	7	2	3	2	372	41.9	7.31	9.78	2	metabolic	cytoplasm;	catalytic a	Pf01370, P	2762	ENSG0000	GMDS	6	Fructose a		GDP-fucos	9	1.087	0.452755965	19.01	757.4	642	12	24.73	High	High	High	High	High	High	1	
FALSE	High	Master Prc	P11021	78 kDa glu	0	386.467	59	45	214	42	654	72.3	5.16	508.99	45	cell commı	cell surface	catalytic a	Pf00012, P	3309	ENSG0000	HSPA5	9	Protein exp	Parkin-Ubi	Platelet de	27	1.086	0.050044947	1.41	71878	67568.8	3.75	2.06	High	High	High	High	High	High	1	
FALSE	High	Master Prc	P35658-1	Nuclear pc	0	74.762	8	12	21	12	2090	213.5	7.47	49.52	12	cell organi	cytosol;nu	protein bir		8021	ENSG0000	NUP214	9	RNA transp	TGF-beta S	Rev-media	68	1.086	0.379547912	14.93	4522.6	4130.5	27.23	7.37	High	High	High	High	High	High	1	
FALSE	High	Master Prc	O43252	bifunctiona	0	16.584	7	3	4	2	624	70.8	6.86	13.06	3	metabolic	cytosol	catalytic a	Pf01583, P	9061	ENSG0000	PAPSS1	4	Sulfur met	Sulfation E	Transport a	19	1.086	0.549876306	46.04	739.9	715.1	49.83	9.99	High	High	High	High	High	High	1	
FALSE	High	Master Prc	Q99575	Ribonuclea	0	16.914	5	4	5	4	1024	114.6	9.22	13.03	4	metabolic	Golgi;mito	catalytic a	Pf06978, P	10940	ENSG0000	POP1	8	Ribosome		tRNA proc	5	1.086	0.714827182	57.3	921.3	959.3	43.62	10.34	High	High	High	High	High	High	1	
FALSE	High	Master Prc	Q8IWE2	Protein No	0	8.492	6	2	3	2	563	60.7	4.68	5.23	2		cytoplasm;	protein bir	Pf05334	92689	ENSG0000	FAM114A1	4				0	1.086	0.841826168	15.46	635.7	597.2	20.83	11.08	High	High	High	High	High	High	1	
FALSE	High	Master Prc	Q92804-1	TATA-bind	0	93.845	43	16	49	14	592	61.8	8.02	106.61	16	regulation	cytoplasm;	DNA bindi	Pf00076, P	8148	ENSG0000	TAF15	17; CHR_H	Transcripti			2	1.085	0.132538891	3.56	5524.2	4887.1	5.48	12	High	High	High	High	High	High	1	[R206;
FALSE	High	Master Prc	P20810-6	Isoform 6 c	0	87.454	31	21	34	21	791	84.9	5.36	69.74	21	regulation	cytosol;en	enzyme re	Pf00748	831	ENSG0000	CAST	5				0	1.085	0.233803652	11.56	6498.7	5986.9	2.4	5.88	High	High	High	High	High	High	1	
FALSE	High	Master Prc	Q9P013	spliceosom	0	35.392	27	5	8	5	229	26.6	5.71	23.05	5	metabolic	mitochond	protein bir	Pf04889	51503	ENSG0000	CWC15	11	Spliceosom		mRNA Spli	5	1.085	0.302186466	8.62	1801.5	1659.8	15.97	5.72	High	High	High	High	High	High	1	
FALSE	High	Master Prc	P62753	40S RIBOS	0	101.5	49	15	52	15	249	28.7	10.84	148.32	15	cell death;	cytoplasm;	protein bir	Pf01092	6194	ENSG0000	RPS6	9	Ribosome;	IL-5 Signali	Peptide ch	62	1.085	0.37515865	6.52	17674.9	16771.6	6.34	3.45	High	High	High	High	High	High	1	
FALSE	High	Master Prc	P32929	Cystathion	0	20.171	18	6	6	6	405	44.5	6.7	11.29	6	cell organi	cytoplasm;	catalytic a	Pf00155, P	1491	ENSG0000	CTH	1	Biosynthes	Selenium N	Metabolisı	19	1.085	0.469722659	1.7	2151	2038.2	8.24	2.21	High	High	High	High	High	High	1	
FALSE	High	Master Prc	P19388	DNA-direct	0	15.283	15	3	7	3	210	24.5	5.95	14.82	3	metabolic	cytosol;nu	catalytic a	Pf01191, P	5434	ENSG0000	POLR2E	19	Purine met	Eukaryotic	Signaling b	98	1.085	0.47576339	25.75	611.9	582.4	24.36	5.21	High	High	High	High	High	High	1	
FALSE	High	Master Prc	Q05682-1	Caldesmor	0	37.432	10	6	8	6	793	93.2	5.66	24.9	6	cellular coı	cytoplasm;	protein bir	Pf02029	800	ENSG0000	CALD1	7	Vascular sr	Myometria	Smooth M	4	1.085	0.504772227	25.16	2317.1	2136.1	15.08	7.56	High	High	High	High	High	High	1	
FALSE	High	Master Prc	P53985	Monocarb	0	35.62	12	5	9	5	500	53.9	8.66	21.74	5	cell organi	membrane	protein bir	Pf00083, P	6566	ENSG0000	SLC16A1	1; CHR_HC		Photodyna	Proton-cou	16	1.085	0.577708779	3.34	2443.1	2322	3.51	8.66	High	High	High	High	High	High	1	[K494]
FALSE	High	Master Prc	Q9Y6I9	Testis-expr	0	7.245	5	1	1	1	313	34.2	4.86	4.57	1	transport	extracellul	protein bir	Pf06445	51368	ENSG0000	TEX264	3			Platelet de	4	1.085	0.604108355	12.39	204.5	196.5	10.07	5.55	High	High	High	High	High	High	1	
FALSE	High	Master Prc	P29590	Protein PM	0	14.626	4	3	4	3	882	97.5	6.21	5.47	3	cell death;	cytoplasm;	DNA bindi	Pf00097, P	5371	ENSG0000	PML	15	Acute mye	miRNA Reg	Interferon	33	1.085	0.683979603	9.7	1618.2	1580.9	13.13	3.51	High	High	High	High	High	High	1	
FALSE	High	Master Prc	Q9UMX0-1	Ubiquilin-1	0	35.793	19	6	12	3	589	62.5	5.11	37.38	6	cell commı	cytoplasm;	protein bir	Pf00240, P	29979	ENSG0000	UBQLN1	9	Protein prc		Cargo reco	5	1.085	0.729162109	40.11	1171.9	1079.6	20.68	8.17	High	High	High	High	High	High	1	
FALSE	High	Master Prc	Q5SSJ5-1	Heterochrc	0	17.93	10	5	7	5	553	61.2	9.67	16.45	5	cell organi	chromosor	DNA bindi	Pf00538	50809	ENSG0000	HP1BP3	1				0	1.084	0.172044431	4.08	1731.2	1634.7	10.2	6.21	High	High	High	High	High	High	1	
FALSE	High	Master Prc	O95777	U6 snRNA-	0	18.624	58	4	10	4	96	10.4	4.48	20.87	4	metabolic	nucleus;sp	protein bir	Pf01423	51691	ENSG0000	NAA38; LS	7	Spliceosom		mRNA Spli	6	1.084	0.226332548	4.15	2617	2413.4	0.39	6.54	High	High	High	High	High	High	1	
FALSE	High	Master Prc	P29317	Ephrin typ	0	31.036	10	7	9	7	976	108.2	6.23	22.68	7	cell death;	cell surface	catalytic a	Pf00041, P	1969	ENSG0000	EPHA2	1	Ras signali	NRF2 path	EPH-ephrir	11	1.084	0.311248736	12.31	3200.7	2888.8	9.62	8.31	High	High	High	High	High	High	1	
FALSE	High	Master Prc	Q5T280	putative m	0	22.677	24	6	7	6	376	42	7.43	11.43	6	cell divisio	chromosor	catalytic a	Pf02598	51490	ENSG0000	C9orf114;	9				0	1.084	0.396480174	14.35	76.6	65.5	12.18	5.89	High	High	High	High	High	High	1	
FALSE	High	Master Prc	Q04721	Neurogeni	0	5.596	1	1	1	1	2471	265.2	5.14	2.61	1	cell death;	cell surface	metal ion l	Pf00008, P	4853	ENSG0000	NOTCH2	1	Breast can	Neural Cre	NOTCH2 A	29	1.084	0.401034864	17.84	79	72.8	30.94	4.9	High	High	High	High	High	High	1	
FALSE	High	Master Prc	Q14165	Malectin [(	0	18.4	9	2	6	2	292	32.2	5.41	12.19	2	metabolic	endoplasm	protein bir	Pf11721	9761	ENSG0000	MLEC	12			Neutrophil	7	1.084	0.574718751	33.9	849.9	733.8	24.27	4.54	High	High	High	High	High	High	1	
FALSE	High	Master Prc	Q99962	Endophilin	0	14.251	12	3	5	2	352	39.9	5.45	15.37	3	cell growth	cytoplasm;	protein bir	Pf00018, P	6456	ENSG0000	SH3GL2	9	Endocytosi	EGF/EGFR	Cargo reco	30	1.084	0.599592209	5.74	300.6	360.2	17.25	31.95	High	High	High	High	High	High	1	
FALSE	High	Master Prc	Q9BV38	WD repeat	0	9.727	6	2	2	2	432	47.4	6.7	6.67	2	developme	cytoplasm;	protein bir	Pf00400, P	57418	ENSG0000	WDR18	19			Major path	4	1.084	0.823094775	19.29	335.6	331.6	24	13.35	High	High	High	High	High	High	1	
FALSE	High	Master Prc	P24539	ATP syntha	0	42.647	32	7	18	7	256	28.9	9.36	41.62	7	cell organi	membrane	catalytic a	Pf05405	515	ENSG0000	ATP5F1	1	Metabolic	Electron Tı	Cristae for	14	1.083	0.101709867	5.23	6802.5	6141.9	2.63	4.13	High	High	High	High	High	High	1	
FALSE	High	Master Prc	P49006	MARCKS-re	0	33.16	19	3	11	3	195	19.5	4.67	31.52	3	regulation	cytoplasm;	protein bir		65108	ENSG0000	MARCKSL1	1	Leishmania			2	1.083	0.380833933	6.86	2317.6	2293.8	11.17	5.66	High	High	High	High	High	High	1	
FALSE	High	Master Prc	Q13868	Exosome c	0	22.894	20	5	6	5	293	32.8	7.5	12.84	5	metabolic	cytoplasm;	catalytic a	Pf14382	23404	ENSG0000	EXOSC2	9	RNA degra		Major path	15	1.083	0.623792749	37.3	1656.1	1528.7	23.37	6.36	High	High	High	High	High	High	1	
FALSE	High	Master Prc	Q13526	peptidyl-pı	0	8.489	18	2	4	2	163	18.2	8.82	8.39	2	cell differe	cytoplasm;	catalytic a	Pf00397, P	5300	ENSG0000	PIN1	19	RIG-I-like r	RIG-I-like F	ISG15 anti	18	1.083	0.700947062	19.98	1222.6	1139.4	15.49	3.65	High	High	High	High	High	High	1	
FALSE	High	Master Prc	O76071	Probable c	0	18.22	17	4	6	4	339	37.8	4.97	20.19	4	cell organi		protein bir	Pf00400, P	9391	ENSG0000	CIAO1	2			Cytosolic ir	2	1.083	0.927776754	4.81	1322.3	1317.5	11.51	6.43	High	High	High	High	High	High	1	
FALSE	High	Master Prc	P61769	Beta-2-mic	0	33.313	49	6	15	6	119	13.7	6.52	51.64	6	cell differe	cell surface	protein bir	Pf07654	567	ENSG0000	B2M	15; CHR_H	Antigen pr		DAP12 sigı	25	1.082	0.005426455	0.62	8211.8	7562	5.9	5.71	High	High	High	High	High	High	1	
FALSE	High	Master Prc	P62841	40S riboso	0	74.441	50	5	25	5	145	17	10.39	64.33	5	cell differe	cytosol;me	DNA bindi	Pf00203	6209	ENSG0000	RPS15	19	Ribosome	Cytoplasm	Peptide ch	38	1.082	0.028661363	1.06	5011.6	4562.7	2.2	3.39	High	High	High	High	High	High	1	[Q32]
FALSE	High	Master Prc	Q9Y6K0	Choline/et	0.003	3.226	3	1	1	1	416	46.5	8.21	2.84	1	metabolic	endoplasm	catalytic a	Pf01066	10390	ENSG0000	CEPT1	1	Ether lipid	Kennedy p	Synthesis c	11	1.082	0.177826387	7.54	505.4	467.1	2.01	4.3	High	High	High	High	High	High	1	
FALSE	High	Master Prc	Q9NYK5-1	39S riboso	0	22.529	14	7	13	7	338	38.7	7.65	35.81	7	cell organi	membrane	nucleotide	Pf02824	54148	ENSG0000	MRPL39	21			Mitochonc	6	1.082	0.200377505	7.09	5345.6	4620.4	7.65	7.25	High	High	High	High	High	High	1	
FALSE	High	Master Prc	Q9NVM9	integrator	0	5.541	3	2	2	2	706	80.2	6.7	5.83	2	cell divisio	cytoplasm;	protein bir	Pf10221	55726	ENSG0000	ASUN; INT	12			RNA polym	3	1.082	0.30246209	8.14	330.6	305.5	12.17	10.23	High	High	High	High	High	High	1	
FALSE	High	Master Prc	Q8IWS0-1	PHD finger	0	18.908	15	4	4	4	365	41.3	8.68	10.62	4	metabolic	chromosor	DNA bindi	Pf13771, P	84295	ENSG0000	PHF6	X		Mesoderm		1	1.082	0.326031812	5.72	1598.1	1442.1	25.65	4.9	High	High	High	High	High	High	1	

FALSE	High	Master Prc	Q9H4K7	Mitochonc	0	7.182	5	2	3	2	406	43.9	9.45	4.24	2	metabolic	membrane	catalytic a	Pf00025, P	26164	ENSG0000	GTPBP5; M	20				0	1.082	0.524699683	24.15	1009.5	933.2	9.56	7.85	High	High	High	High	High	High	1	
FALSE	High	Master Prc	P30040-1	Endoplasm	0	86.356	53	14	42	14	261	29	7.31	90.79	14	metabolic	cell surface	catalytic a	Pf07749, P	10961	ENSG0000	ERP29	12	Protein prc			1	1.082	0.568462363	18.33	11963.5	11055.6	12.35	2.78	High	High	High	High	High	High	1	
FALSE	High	Master Prc	P32004	Neural cell	0	68.627	15	16	23	16	1257	139.9	6.24	45.28	16	cell differe	cell surface	protein bir	Pf00041, P	3897	ENSG0000	L1CAM	X	Cell adhesi		Basigin int	11	1.081	0.13976242	2.54	7220	6677.4	5.88	1.62	High	High	High	High	High	High	1	
FALSE	High	Master Prc	P98088	Mucin-5AC	0	10.99	1	4	4	4	5654	585.2	7.02	6.26	4	metabolic	cytoplasm;	protein bir	Pf00094, P	4586	ENSG0000	MUC5AC	11; CHR_H			Defective (	15	1.081	0.306660037	9.57	267	247	23.21	6.14	High	High	High	High	High	High	1	[Q4691;
FALSE	High	Master Prc	P00390	Glutathion	0	24.439	13	4	7	4	522	56.2	8.5	12.94	4	cellular ho	cytoplasm;	antioxidan	Pf00070, P	2936	ENSG0000	GSR	8	Glutathion	Glutathion	Detoxificat	13	1.081	0.479690747	28.01	1283.1	1246.4	34.86	8.6	High	High	High	High	High	High	1	
FALSE	High	Master Prc	Q5VT52	Regulation	0	12.441	4	3	4	3	1461	155.9	7.42	7.25	3	metabolic			Pf04818	23248	ENSG0000	RPRD2	1			RNA polym	3	1.081	0.495808269	40.5	935.2	935.2	60.63	9.43	High	High	High	High	High	High	1	
FALSE	High	Master Prc	O60318	Germinal-c	0.004	2.793	1	1	1	1	1980	218.3	6.39	3.38	1	transport	cytoplasm;	catalytic a	Pf03399, P	8888	ENSG0000	MCM3AP	21				0	1.081	0.949066538	6.76	333.3	340.7	17.23	6.52	High	High	High	High	High	High	1	
FALSE	High	Master Prc	Q9Y305-1	Acyl-coenz	0	24.639	16	7	15	7	439	49.9	8.6	35.25	7	metabolic	mitochond	catalytic a	Pf03061	23597	ENSG0000	ACOT9	X			Mitochonc	4	1.081	0.971256255	13.33	4981.3	4744.3	22	1.92	High	High	High	High	High	High	1	
FALSE	High	Master Prc	P06730	Eukaryotic	0	8.341	10	2	4	2	217	25.1	6.15	8.48	2	defense re	cytoplasm;	protein bir	Pf01652	1977	ENSG0000	EIF4E	4	HIF-1 signa	Translatior	ISG15 anti	45	1.08	0.056426775	3.5	718.2	680.9	4.49	7.22	High	High	High	High	High	High	1	
FALSE	High	Master Prc	O60831	PRA1 fami	0	13.542	16	2	3	2	178	19.2	9.19	7.58	2	transport	endosome		Pf03208	11230	ENSG0000	PRAF2	X				0	1.08	0.579153542	9.33	2118.1	1970.7	5.17	6.66	High	High	High	High	High	High	1	
FALSE	High	Master Prc	P63172	Dynein ligh	0	6.319	16	1	1	1	113	12.4	5.08	6.12	1	cell divisio	cytoplasm;	catalytic a	Pf03645	6993	ENSG0000	DYNLT1	6			Neutrophil	3	1.08	0.715833522	42.66	107	98	23.48	7.76	High	High	High	High	High	High	1	
FALSE	High	Master Prc	Q04724	Transducin	0	27.025	7	3	4	1	770	83.1	7.24	11.7	3	developme	cytosol;nu	protein bir	Pf00400, P	7088	ENSG0000	TLE1	9		Notch Sign	NOTCH1 Ir	11	1.08	0.872209052	25.26	430.6	398.7	28.74	8.24	High	High	High	High	High	High	1	
FALSE	High	Master Prc	Q99638	cell cycle c	0	5.924	6	1	1	1	391	42.5	5.66	3.01	1	metabolic	cytoplasm;	catalytic a	Pf04139	5883	ENSG0000	RAD9A	11		Androgen	G2/M DNA	26	1.08	0.940377755	24.39	123.3	119.2	22.44	11.78	High	High	High	High	High	High	1	
FALSE	High	Master Prc	Q96IR7	4-hydroxyp	0	16.292	11	3	4	3	371	39.4	7.03	13.71	3	metabolic		catalytic a	Pf12681	84842	ENSG0000	HPDL	1				0	1.079	0.035367946	2.02	2727.1	2516.4	2.88	5.37	High	High	High	High	High	High	1	
FALSE	High	Master Prc	P62140	Serine/thre	0	76.16	53	14	27	4	327	37.2	6.19	91.2	14	cell divisio	cytoplasm;	catalytic a	Pf00149	5500	ENSG0000	PPP1CB	2	Insulin sigr	Common F	Triglycerid	43	1.079	0.337822024	6.43	2127	2082.3	5.76	5.76	High	High	High	High	High	High	1	
FALSE	High	Master Prc	P13984	General tra	0	4.839	13	2	2	2	249	28.4	9.23	2.81	2	metabolic	cytoskelet	catalytic a	Pf02270	2963	ENSG0000	GTF2F2	13	Basal trans	Eukaryotic	Signaling b	56	1.079	0.358892973	15.27	530.8	496.7	2.35	15.5	High	High	High	High	High	High	1	
FALSE	High	Master Prc	Q15631-1	Translin [O	0	29.58	33	8	12	8	228	26.2	6.44	31.18	8	metabolic	cytoplasm;	catalytic a	Pf01997	7247	ENSG0000	TSN	2			Small inter	3	1.079	0.47067636	2.07	5340.5	5270.7	5.31	5.13	High	High	High	High	High	High	1	
FALSE	High	Master Prc	Q00169	Phosphatic	0	9.289	16	3	4	2	270	31.8	6.55	4.2	3	cell organi	cytoplasm;	transporte	Pf02121	5306	ENSG0000	PITPNA	17			Gene and		9	1.079	0.555172261	50.5	168.2	171.1	59.67	15.98	High	High	High	High	High	High	1	
FALSE	High	Master Prc	P12004	proliferatir	0	101.689	50	11	39	11	261	28.8	4.69	116.79	11	cell differe	nucleus	catalytic a	Pf00705, P	5111	ENSG0000	PCNA	20	HTLV-I infe	Cell Cycle;	Translesior	80	1.079	0.604705403	1.05	13122.4	12077.4	9.13	0.87	High	High	High	High	High	High	1	
FALSE	High	Master Prc	Q15526	Surfeit locu	0	10.139	9	1	1	1	300	33.3	9.6	5.18	1	cell organi	membrane	catalytic a	Pf02104	6834	ENSG0000	SURF1	9; CHR_HG		Electron T	TP53 Regu	11	1.079	0.975866396	26	146.8	136	17.36	10.67	High	High	High	High	High	High	1	
FALSE	High	Master Prc	P09493-3	Isoform 3 (	0	59.065	38	16	32	3	284	32.9	4.78	49.22	16	cell organi	cytoplasm;	protein bir	Pf00261, P	7168	ENSG0000	TPM1	15		Striated M		1	1.078	0.09159197	1.26	2415.9	2256.4	1.79	4.54	High	High	High	High	High	High	1	
FALSE	High	Master Prc	Q13310-3	Isoform 3 (	0	133.138	30	22	54	11	660	72.3	9.35	148.45	22	coagulatio	cytoplasm;	protein bir	Pf00076, P	8761	ENSG0000	PABPC4	1				0	1.078	0.210507686	9.09	3947.7	3533.8	2.34	9.15	High	High	High	High	High	High	1	
FALSE	High	Master Prc	Q8WTT2	Nucleolar (	0	16.355	7	4	6	4	800	92.5	9.17	16.23	4	cell differe	mitochond	RNA bindi	Pf03914, P	64318	ENSG0000	NOC3L	10				0	1.078	0.237961348	6.38	1395.3	1252.9	11.01	2.69	High	High	High	High	High	High	1	
FALSE	High	Master Prc	O14802	DNA-direct	0	8.817	4	3	3	3	1390	155.5	8.48	5.97	3	defense re	cytosol;me	catalytic a	Pf00623, P	11128	ENSG0000	POLR3A	10	Purine met		RNA Polym	18	1.078	0.296629794	7.58	307.3	285.1	26.17	10.22	High	High	High	High	High	High	1	
FALSE	High	Master Prc	Q08170	Serine/argi	0	26.787	15	8	12	4	494	56.6	11.52	17.97	8	metabolic	nucleus	protein bir	Pf00076, P	6429	ENSG0000	SRSF4	1	Spliceosom	mRNA Pro	Cleavage o	14	1.078	0.369136139	10.21	1546.4	1367.4	32.1	9.04	High	High	High	High	High	High	1	
FALSE	High	Master Prc	Q92974	Rho guanir	0	10.821	4	4	6	4	986	111.5	7.27	6.76	4	cell divisio	cytoplasm;	metal ion l	Pf00130, P	9181	ENSG0000	ARHGEF2	1	Pathogeni	Pathogeni	G alpha (1:	13	1.078	0.40582686	16.93	1125.9	1003.3	4.34	12.48	High	High	High	High	High	High	1	
FALSE	High	Master Prc	Q8IXT5	RNA-bindir	0	4.904	2	2	2	2	1001	118	6.81	2.22	2			protein bir	Pf00076, P	389677	ENSG0000	RBM12B	8				0	1.078	0.530609728	38.22	608.4	657.3	47.49	9.83	High	High	High	High	High	High	1	
FALSE	High	Master Prc	Q9Y4E1	WASH com	0	16.882	4	3	3	3	1318	144.6	4.79	8.68	3	regulation	cytosol;en	protein bir	Pf15255	253725		FAM21C; \	10	Endocytosi			1	1.078	0.570655979	39.88	776.4	793.2	39.92	8.04	High	High	High	High	High	High	1	
FALSE	High	Master Prc	Q92879	CUGBP Ela	0	46.189	11	5	11	5	486	52	8.46	32.89	5	cell organi	cytoplasm;	protein bir	Pf00076, P	10658	ENSG0000	CELF1	11		Adipogene		2	1.078	0.643962249	21.97	4654.2	4317.8	15.96	1.98	High	High	High	High	High	High	1	
FALSE	High	Master Prc	Q96H79	Zinc finger	0	17.579	14	3	3	3	300	32.9	8.13	10.41	3		cytosol	metal ion l		92092	ENSG0000	ZC3HAV1L	7				0	1.078	0.71841058	25.06	1685.9	1337.3	15.17	27.51	High	High	High	High	High	High	1	
FALSE	High	Master Prc	P49406	39S riboso	0	15.081	20	4	4	4	292	33.5	9.5	8.25	4	cell organi	membrane	structural	Pf01245	9801	ENSG0000	MRPL19	2	Ribosome	Cytoplasm	Mitochonc	8	1.078	0.790402254	6.09	286.2	264.2	5.01	13.39	High	High	High	High	High	High	1	
FALSE	High	Master Prc	P47914	60S riboso	0	37.403	30	6	19	6	159	17.7	11.66	39.77	6	cell organi	cytosol;me	protein bir	Pf01779	6159	ENSG0000	RPL29	3	Ribosome	Cytoplasm	Peptide ch	34	1.077	0.16217416	5.42	6448.9	5974	3.06	11.13	High	High	High	High	High	High	1	
FALSE	High	Master Prc	Q9Y265	RuvB-like 1	0	78.962	35	11	22	11	456	50.2	6.42	76	11	cell divisio	cytoplasm;	catalytic a	Pf00004, P	8607	ENSG0000	RUVBL1	3	Wnt signal	Gastric Ca	Ub-specifi	25	1.077	0.199305713	2.69	8400.9	8001.8	4.09	1.93	High	High	High	High	High	High	1	
FALSE	High	Master Prc	P51116	Fragile X m	0	30.91	12	7	10	5	673	74.2	6.23	27.01	7	regulation	cytoplasm;	protein bir	Pf00013, P	9513	ENSG0000	FXR2	17	RNA trans			1	1.077	0.23915268	7.57	2052.6	1906.2	15.2	4.81	High	High	High	High	High	High	1	
FALSE	High	Master Prc	Q6PIU2	Neutral ch	0	7.371	6	2	3	2	408	45.8	7.23	10.85	2	metabolic	endoplasm	catalytic a	Pf00135, P	57552		NCEH1	3	Bile secret		LDL cleara	5	1.077	0.253186644	8.87	575.7	523.1	10.67	1.52	High	High	High	High	High	High	1	
FALSE	High	Master Prc	O43395	U4/U6 sma	0	21.863	12	8	10	8	683	77.5	9.5	17.34	8	cell organi	cytosol;nu	protein bir	Pf01480, P	9129	ENSG0000	PRPF3	1	Spliceosom	mRNA Pro	mRNA Spli	6	1.077	0.476334717	26.68	2397.2	2226.1	27.57	6.04	High	High	High	High	High	High	1	
FALSE	High	Master Prc	Q92597	Protein ND	0	27.729	14	3	4	3	394	42.8	5.82	12.67	3	cell organi	cytoplasm;	protein bir	Pf00561, P	10397	ENSG0000	NDRG1	8		VEGFA-VE	TP53 regul	7	1.077	0.510525998	10.75	959.6	1028.4	14.37	10.13	High	High	High	High	High	High	1	
FALSE	High	Master Prc	Q8N3U4-1	Cohesin su	0	20.086	5	4	5	4	1231	141.2	5.43	12.28	4	cell divisio	chromosor	protein bir	Pf00670, P	10735	ENSG0000	STAG2	X	Cell cycle		Meiotic sy	21	1.077	0.515497112	45.57	406.8	377.7	61.3	16.59	High	High	High	High	High	High	1	
FALSE	High	Master Prc	O15504	nucleopori	0.003	3.2	3	1	1	1	423	44.8	9.19	2.24	1	cell organi	cytosol;me	metal ion l	Pf00642	11097	ENSG0000	NUPL2	7	RNA trans		Rev-media	64	1.077	0.526406101	20.95	155.9	144.8	3.91	10.6	High	High	High	High	High	High	1	
FALSE	High	Master Prc	Q9Y6U3	ADSEVERIN	0	54.342	20	10	14	10	715	80.4	5.71	39.18	10	cell organi	cytoplasm;	metal ion l	Pf00626	85477	ENSG0000	SCIN	7	Viral carcir	Endochonc		4	1.077	0.586805978	16.44	4286.4	4403	16.16	6.26	High	High	High	High	High	High	1	
FALSE	High	Master Prc	Q99622	Protein C1(	0	8.918	10	1	2	1	126	13.2	5.14	4.95	1	cell organi	cytoplasm		Pf14974	113246	ENSG0000	C12orf57	12				0	1.077	0.906187542	11.27	456.6	440.8	14.95	7.03	High	High	High	High	High	High	1	
FALSE	High	Master Prc	Q9NRW7	Vacuolar p	0	7.375	2	1	1	1	570	65	8.24	4.59	1	coagulatio	endosome	protein bir	Pf00995	11311	ENSG0000	VPS45	1	Endocytosi		Intra-Golgi	7	1.077	0.934470801	53.56	901.6	663.3	47.83	14.37	High	High	High	High	High	High	1	
FALSE	High	Master Prc	Q8WVV9	Heterogen	0	44.078	28	11	15	10	542	60	7.72	28.72	11	metabolic	membrane	protein bir	Pf00076, P	92906	ENSG0000	HNRNPLL;	2				0	1.076	0.048845344	1.58	1447.8	1331.7	6.9	5.12	High	High	High	High	High	High	1	
FALSE	High	Master Prc	Q15428	splicing fac	0	39.265	17	7	13	7	464	49.2	9.64	37.93	7	cell organi	nucleus;sp	metal ion l	Pf12874	8175	ENSG0000	SF3A2	19	Spliceosom	mRNA Pro	mRNA Spli	6	1.076	0.414145036	15.59	6047.3	5057.5	14.12	24.98	High	High	High	High	High	High	1	
FALSE	High	Master Prc	Q9BTT6	Leucine-ric	0	15.918	7	3	3	2	524	59.2	5.02	9.28	3	regulation	cytoplasm;	protein bir	Pf12799, P	55227	ENSG0000	LRRC1	6				0	1.076	0.500488502	22.34	469.3	466.7	16.22	5.61	High	High	High	High	High	High	1	
FALSE	High	Master Prc	P61923	Coatomer	0	28.204	27	4	8	4	177	20.2	4.81	20.78	4	transport	cytoplasm;		Pf01217	22818	ENSG0000	COPZ1	12			COPI-depe	11	1.076	0.552470863	25.56	1694.3	1527.8	13.46	10.81	High	High	High	High	High	High	1	
FALSE	High	Master Prc	Q12907	Vesicular ir	0	45.58	25	7	12	7	356	40.2	6.95	32.59	7	regulation	cell surface	metal ion l	Pf03388	10960	ENSG0000	LMAN2	5	Protein prc		Cargo conc	10	1.076	0.739872614	16.1	2160.6	2003.6	17.26	0.38	High	High	High	High	High	High	1	
FALSE	High	Master Prc	Q96AG4	Leucine-ric	0	91.905	57	18	38	17	307	34.9	9.57	86.89	18		endoplasm	protein bir	Pf12799, P	55379	ENSG0000	LRRC59	17				0	1.075	0.03700697	0.95	14676.6	13658	5.2	2.84	High	High	High	High	High	High	1	
FALSE	High	Master Prc	Q96JP5-1	E3 ubiquiti	0	5.181	4	1	1	1	570	63.4	7.36	0	1	metabolic	nucleus	catalytic a	Pf13465	80829	ENSG0000	ZFP91	11				0	1.075	0.041739683	2.7	307.3	295.3	9.61	10	High	High	High	High	High	High	1	
FALSE	High	Master Prc	Q10589	bone marr	0	7.63	14	2	4	2	180	19.8	5.6	6.37	2	cell commu	cell surface	enzyme re		684	ENSG0000	BST2	19			Interferon	6	1.075	0.067699328	4.35	1647.1	1572	4.24	2.43	High	High	High	High	High	High	1	
FALSE	High	Master Prc	Q9NVA2-1	Septin-11		0	20.103	13	4	5	2	429	49.4	6.81	9.31	4	cell divisio	cytoplasm;	nucleotide	Pf00735	55752	ENSG0000	SEPT11	4	Bacterial ir			1	1.075	0.249706976	2.27	200	182.8	14.74	2.15	High	High	High	High	High	High	1	
FALSE	High	Master Prc	Q9UHG3	prenylcyst	0	39.173	21	8	8	8	505	56.6	6.18	24.82	8	metabolic	membrane	catalytic a	Pf01593, P	51449	ENSG0000	PCYOX1	2	Terpenoid			1	1.075	0.367601305	14.91	2766.4	2573.7	8.95	5.48	High	High	High	High	High	High	1	
FALSE	High	Master Prc	Q13685	angio-asso	0	12.448	11	2	2	2	434	46.7	4.42	7.4	2	cell differe	cell surface	protein bir	Pf00400, P	14	ENSG0000	AAMP	2		Nucleotide		1	1.075	0.382764494	0.37	285.8	275.5	2.58	4.66	High	High	High	High	High	High	1	
FALSE	High	Master Prc	Q9UKY7	Protein CD	0	61.528	55	6	12	6	258	27.3	6.4	39.91	6	cell prolife	cytoplasm		Pf15359	55573	ENSG0000	CDV3	3				0	1.075	0.383526193	15.86	3835.9	3461.2	18.51	3.66	High	High	High	High	High	High	1	
FALSE	High	Master Prc	P57740	Nuclear pc	0	40.621	11	9	13	9	925	106.3	5.43	24.54	9	cell organi	chromosor	protein bir	Pf04121	57122	ENSG0000	NUP107	12	RNA trans	Gastric Ca	Rev-media	78	1.075	0.47898155	22.77	2571.7	2392.3	17.6	9.16	High	High	High	High	High	High	1	
FALSE	High	Master Prc	Q86Y56	Dynein ass	0	16.481	5	4	4	4	855	93.5	6.42	11.03	4	cell organi	cytoplasm	protein bir		54919	ENSG0000	HEATR2; D	7				0	1.075	0.491206684	24.97	1642.6	1528.5	22.24	6.71	High	High	High	High	High	High	1	
FALSE	High	Master Prc	P17568	NADH deh	0.004	2.631	7	1	3	1	137	16.4	8.92	3.84	1	cell organi	membrane	catalytic a	Pf05676	4713	ENSG0000	NDUFB7	19	Parkinson's	Electron T	Complex I	13	1.075	0.493984709	14.42	577.1	573	19.93	18.97	High	High	High	High	High	High	1	
FALSE	High	Master Prc	Q8NBL1	Protein O-	0.003	3.386	4	1	1	1	392	46.2	8.72	3.42	1	developme	endoplasm	catalytic a	Pf05686	56983	ENSG0000	POGLUT1	3	Other type		Pre-NOTCH	5	1.075	0.514009009	7.79	280.9	265.4	8.3	4.55	High	High	High	High	High	High	1	
FALSE	High	Master Prc	P29144	Tripeptidyl	0	29.37	7	7	11	7	1249	138.3	6.32	14.02	7	metabolic	cytoplasm;	catalytic a	Pf00082, P	7174	ENSG0000	TPP2	13			Antigen pr	4	1.075	0.554089672	5.11	1237.8	1200.5	5.37	5.64	High	High	High	High	High	High	1	
FALSE	High	Master Prc	O60925	prefoldin s	0	10.04	23	3	4	3	122	14.2	6.81	9.63	3	cell organi	cytoplasm	protein bir	Pf01920	5201	ENSG0000	PFDN1	5			Prefoldin r	5	1.075	0.567560575	1.18	4009.2	3698.9	13.09	5.45	High	High	High	High	High	High	1	
FALSE	High	Master Prc	O00411	DNA-direct	0	6.864	4	2	2	2	1230	138.5	8.98	3.58	2	cell organi	mitochond	catalytic a	Pf00940, P	5442	ENSG0000	POLRMT	19		Mitochonc	Mitochonc	7	1.075	0.963176771	20.59	147.9	142.4	14.27	11.32	High	High	High	High	High	High	1	
FALSE	High	Master Prc	Q04695	Keratin, ty	0	148.705	47	19	75	12	432	48.1	5.02	188.12	19	cell death;	cytoplasm;	protein bir	Pf00038, P	3872	ENSG0000	KRT17	17			Formation	3	1.074	0.393787764	3.49	19820.4	18290.8	7.33	0.66	High	High	High	High	High	High	1	
FALSE	High	Master Prc	P62879	Guanine nu	0	86.941	34	9	27	4	340	37.3	6	98.9	9	metabolic	cytoplasm;	catalytic a	Pf00400	2783	ENSG0000	GNB2	7	Morphine	Corticotro	G alpha (z)	74	1.074	0.510553444	22.12	3382.6	3118.2	11.47	5.09	High	High	High	High	High	High	1	
FALSE	High	Master Prc	Q9BUL9	Ribonuclea	0	8.505	14	2	3	2	199	20.6	9.61	7.07	2	metabolic	nucleus	catalytic a	Pf01918	54913	ENSG0000	RPP25	15	RNA trans		Major path	8	1.074	0.524914978	32.21	604	562.5	33.41	12.92	High	High	High	High	High	High	1	
FALSE	High	Master Prc	Q9HA77	probable c	0	34.269	19	9	16	9	564	62.2	8.34	32.93	9	metabolic	cytoplasm;	catalytic a	Pf00133, P	79587	ENSG0000	CARS2	13	Aminoacyl		Mitochonc	5	1.074	0.531663166	11.48	4002.3	3815.3	9.32	3	High	High	High	High	High	High	1	
FALSE	High	Master Prc	Q9NPL8	Complex I	0	8.641	12	3	3	3	285	32.2	8.5	7.22	3	cell organi	membrane	protein bir	Pf02466	51300	ENSG0000	TIMMDC1	3			Complex I	5	1.074	0.607934148	5.87	297	287.4	7.45	4.01	High	High	High	High	High	High	1	
FALSE	High	Master Prc	Q7Z2K6	Endoplasm	0.002	3.666	2	1	2	1	904	100.2	7.52	2.25	1	metabolic	endoplasm	catalytic a	Pf04389	79956	ENSG0000	ERMP1	9				0	1.074	0.706788303	5.88	1379.6	1451.8	16.81	10.38	High	High	High	High	High	High	1	
FALSE	High	Master Prc	O94855	Protein tra	0	7.088	3	2	2	2	1032	112.9	7.25	5.37	2	cell organi	cytoplasm;	metal ion l	Pf00626, P	9871	ENSG0000	SEC24D	4	Protein prc	Sterol Reg	Regulation	20	1.074	0.881422052	6.65	575.1	535.7	3.53	10.25	High	High	High	High	High	High	1	
FALSE	High	Master Prc	Q14011	Cold-induc	0	11.199	17	2	3	2	172	18.6	9.51	12.58	2	cell organi	cytoplasm;	protein bir	Pf00076, P	1153	ENSG0000	CIRBP	19				0	1.074	0.954366625	94.1	817.4	761.4	44.83	27.56	High	High	High	High	High	High	1	
FALSE	High	Master Prc	Q96C86	M7GpppX	0	19.084	20	5	8	5	337	38.6	6.38	15.14	5	metabolic	cytoplasm;	catalytic a	Pf05652, P	28960	ENSG0000	DCPS	11	RNA degra		mRNA dec	4	1.073	0.25067785	8.17	2464.6	2188	4.93	8.79	High	High	High	High	High	High	1	
FALSE	High	Master Prc	O15031	Plexin-B2 [	0	35.612	5	6	8	6	1838	205	6.24	27.01	6	cell prolife	cell surface	protein bir	Pf01403, P	23654	ENSG0000	PLXNB2	22	Axon guida			1	1.073	0.370871855	13.83	1775.3	1709.9	16.3	5.05	High	High	High	High	High	High	1	
FALSE	High	Master Prc	Q9BTE6-1	Alanyl-tRN	0	7.06	6	2	2	2	412	45.5	6.42	3.72	2	metabolic	cytoplasm;	catalytic a	Pf01411, P	80755; 10(	ENSG0000	AARSD1; P	17				0	1.073	0.548507547	25.28	1115.9	1109.3	18.24	7.38	High	High	High	High	High	High	1	
FALSE	Medium	Master Prc	Q8IYB5	Stromal m	0.024	1.523	2	1	1	1	467	50.4	8.75	1.63	1	regulation	cytoplasm;	enzyme re	Pf01412	60682	ENSG0000	SMAP1	6	Endocytosi			1	1.073	0.887859999	2.52	477.5	437.7	18.39	1.96	High	High	High	High	High	High	1	
FALSE	High	Master Prc	P04040	catalase [C	0	58.391	24	12	14	12	527	59.7	7.39	32.65	12	cell differe	cytosol;en	antioxidan	Pf00199, P	847	ENSG0000	CAT	11	Longevity r	Selenium M	Neutrophil	20	1.073	0.892988183	4.55	2689.7	2565.8	8.45	9.46	High	High	High	High	High	High	1	
FALSE	High	Master Prc	P07339	Cathepsin	0	132.786	39	13	39	13	412	44.5	6.54	125.72	13	metabolic	extracellul	catalytic a	Pf00026, P	1509	ENSG0000	CTSD	11	Tuberculos	Prolactin S	Collagen d	16	1.073	0.945086141	32.96	10983	10240.3	22.25	8.92	High	High	High	High	High	High	1	
FALSE	High	Master Prc	Q07955-1	Serine/argi	0	90.71	60	16	79	15	248	27.7	10.36	161.49	16	cell differe	cytoplasm;	protein bir	Pf00076, P	6426	ENSG0000	SRSF1	17	Herpes sim	mRNA Pro	Cleavage o	15	1.072	0.174368672	6.39	21015.6	19777.3	7.16	4.32	High	High	High	High	High	High	1	[S199(100
FALSE	High	Master Prc	Q9BWJ5	Splicing fa	0	24.283	69	4	6	4	86	10.1	6.35	20.63	4	metabolic	nucleus;sp	RNA bindi	Pf07189	83443	ENSG0000	SF3B5	6	Spliceosom	mRNA Pro	mRNA Spli	7	1.072	0.292597404	7.24	1952	1781.8	15.29	1.91	High	High	High	High	High	High	1	
FALSE	High	Master Prc	Q96I24-1	Far upstre	0	78.869	37	18	29	14	572	61.6	8.38	46.3	18	metabolic	cytoplasm;	DNA bindi	Pf00013, P	8939	ENSG0000	FUBP3	9				0	1.072	0.76627217	3.46	6136.1	5834.4	8.06	3.23	High	High	High	High	High	High	1	
FALSE	High	Master Prc	Q14CX7-1	N-alpha-ac	0.002	4.151	2	2	2	2	972	112.2	6.64	2.61	2	metabolic	cytoplasm;	catalytic a	Pf09797	80018	ENSG0000	NAA25	12				0	1.072	0.86259295	8.5	36.9	36.4	14.05	9.2	High	High	High	High	High	High	1	

FALSE	High	Master Prc	P36542-1	ATP syntha	0	62.529	37	11	33	11	298	33	9.22	51.43	11	cell organi	membrane	catalytic a	Pf00231	509	ENSG0000	ATP5C1	10	Huntingto	Electron Ti	Cristae for	13	1.072		0.963544739	10.14	10410.1	10036.9	17.8	1.98	High	High	High	High	High	High	1	
FALSE	High	Master Prc	O94874-1	E3 UFM1-p	0	15.299	10	7	9	7	794	89.5	6.79	13.6	7	cell differe	cytoplasm;	catalytic a	Pf09743	23376	ENSG0000	UFL1	6			Antigen pr	4	1.071		0.038765397	1.09	2344.4	2204.7	4.3	2.15	High	High	High	High	High	High	1	
FALSE	High	Master Prc	Q9H4A6	Golgi phos	0	30.413	23	6	7	6	298	33.8	6.44	23.94	6	cell organi	cytosol;en	protein bir	Pf05719	64083	ENSG0000	GOLPH3	5	Transcripti			1	1.071		0.139664771	2.92	3143.6	2993.5	8.11	2.79	High	High	High	High	High	High	1	
FALSE	High	Master Prc	P05556-1	Integrin be	0	115.766	27	18	47	18	798	88.4	5.39	132.34	18	cell differe	cell surface	enzyme re	Pf00362, P	3688	ENSG0000	ITGB1	10	Arrhythmc	Integrin-m	Integrin ce	74	1.071		0.155914848	6.29	19419.5	18014.9	1.42	5.34	High	High	High	High	High	High	1	
FALSE	High	Master Prc	P15529-2	Isoform B c	0	42.207	18	9	20	9	399	44.2	6.9	43.35	9	defense re	cell surface	catalytic a	Pf00084	4179	ENSG0000	CD46	1		Compleme		1	1.071		0.229795741	0.28	7306.5	6961.5	1.75	4.67	High	High	High	High	High	High	1	
FALSE	High	Master Prc	Q9BT09	Protein car	0	4.732	6	2	2	2	278	30.7	5.49	4.83	2	defense re	endoplasm	protein bir	Pf11938	10695	ENSG0000	CNPY3	6			Trafficking	4	1.071		0.420009664	17.58	626.8	585.3	39.17	12.54	High	High	High	High	High	High	1	
FALSE	High	Master Prc	Q6UN15-1	Pre-mRNA	0	10.056	7	3	3	3	594	66.5	5.59	5.9	3	metabolic	nucleus	protein bir	Pf05182	81608	ENSG0000	FIP1L1	4	mRNA surv		mRNA 3'-e	15	1.071		0.469835818	55.8	195.3	182.4	111.21	30.32	High	High	High	High	High	High	1	
FALSE	High	Master Prc	Q68CZ2-1	Tensin-3 [C	0	40.832	7	6	8	6	1445	155.2	6.81	26.56	6	cellular coi	cytosol	protein bir	Pf00017, P	64759	ENSG0000	TNS3	7			MET intera	5	1.071		0.528279968	23.54	2168.3	1935.7	13.01	14.47	High	High	High	High	High	High	1	
FALSE	High	Master Prc	Q15654-1	thyroid rec	0	30.978	18	5	7	5	476	50.3	7.37	24.19	5	cell organi	cytoplasm;	metal ion l	Pf00412	7205	ENSG0000	TRIP6	7	NOD-like r			1	1.071		0.637856161	17.84	1341.1	1251.7	21.05	5.43	High	High	High	High	High	High	1	
FALSE	High	Master Prc	Q14318-2	Isoform 2 c	0	14.025	10	4	5	4	413	44.6	4.84	8.9	4	cell death;	cytoplasm;	catalytic a	Pf00254, P	23770	ENSG0000	FKBP8	19		Senescenc		1	1.071		0.728445886	7.81	418.1	390.4	4.22	7.58	High	High	High	High	High	High	1	
FALSE	High	Master Prc	Q9H2H9	Sodium-co	0	7.971	6	3	4	3	487	54	7.02	5.15	3	transport	membrane	protein bir	Pf01490	81539	ENSG0000	SLC38A1	12	GABAergic	Synaptic V	Amino acic	11	1.07		0.06087674	3.17	377.3	349.5	10.02	7.86	High	High	High	High	High	High	1	
FALSE	High	Master Prc	Q13421	mesothelir	0	12.529	9	5	6	5	630	68.9	6.38	8.95	5	metabolic	cell surface	protein bir	Pf06060	10232	ENSG0000	MSLN	16			Post-transl	5	1.07		0.064573416	3.93	838.5	791.8	4.72	3.94	High	High	High	High	High	High	1	
FALSE	High	Master Prc	Q13283	Ras GTPase	0	61.753	32	9	18	9	466	52.1	5.52	61.9	9	metabolic	cytoplasm;	catalytic a	Pf00076, P	10146	ENSG0000	G3BP1	5				0	1.07		0.088346638	3.21	4775.7	4662.1	3.13	4.02	High	High	High	High	High	High	1	
FALSE	High	Master Prc	P51659-1	peroxisom	0	113.656	32	18	28	18	736	79.6	8.84	85.75	18	cell differe	membrane	catalytic a	Pf00106, P	3295	ENSG0000	HSD17B4	5	Peroxisom	Steroid Bic	Beta-oxida	16	1.07		0.195164069	6.89	7928.3	7864.9	12.03	8.05	High	High	High	High	High	High	1	
FALSE	High	Master Prc	Q9BTV4	Transmem	0	18.541	14	5	9	5	400	44.8	8.13	18.31	5	cell organi	endoplasm	protein bir	Pf07787	79188	ENSG0000	TMEM43	3				0	1.07		0.211864586	1.16	1195.3	1117	8.75	4.58	High	High	High	High	High	High	1	
FALSE	High	Master Prc	Q7KZ85	transcripti	0	42.199	8	11	13	11	1726	198.9	4.91	30.39	11	cell organi	nucleus	DNA bindi	Pf00017, P	6830	ENSG0000	SUPT6H	17			Formation	5	1.07		0.27894746	5.33	2326.5	2215.2	18.53	3.49	High	High	High	High	High	High	1	
FALSE	High	Master Prc	Q92947-1	Glutaryl-Cc	0	17.408	9	2	3	2	438	48.1	8.06	15.68	2	metabolic	mitochond	catalytic a	Pf00441, P	2639	ENSG0000	GCDH	19	Lysine deg	Fatty Acid	Lysine cata	10	1.07		0.328236682	9.04	1236.1	1230.5	20.6	5.29	High	High	High	High	High	High	1	
FALSE	High	Master Prc	Q4V328	GRIP1-asso	0	5.799	2	2	2	2	841	95.9	5.11	4.3	2	transport	cytosol;en	protein bir		56850		GRIPAP1	X				0	1.07		0.483484762	18.88	858.2	770	8.59	10.58	High	High	High	High	High	High	1	
FALSE	High	Master Prc	P15170-3	Isoform 3 c	0	59.295	25	14	20	14	637	68.7	5.33	49.15	14	cell organi	cytosol	catalytic a	Pf00009, P	2935	ENSG0000	GSPT1	16				0	1.07		0.519305276	24.59	7644.4	7462.5	20.8	6.18	High	High	High	High	High	High	1	
FALSE	High	Master Prc	O15213	WD repeat	0	6.163	2	1	1	1	610	68	9.67	3.32	1	metabolic	nucleus	protein bir	Pf00400, P	9277	ENSG0000	WDR46	6			Major path	5	1.07		0.768477032	30.64	277.8	265.4	15.61	10.08	High	High	High	High	High	High	1	
FALSE	High	Master Prc	P12270	Nucleoprot	0	292.356	32	62	94	62	2363	267.1	5.02	226.58	62	cell divisio	chromosor	catalytic a	Pf00038, P	7175	ENSG0000	TPR	1	RNA trans		Rev-media	69	1.069		0.036550045	0.97	25602.1	24105.2	4.57	2.86	High	High	High	High	High	High	1	
FALSE	High	Master Prc	P62834	ras-related	0	63.548	61	10	24	3	184	21	6.67	69.34	10	cell organi	cytoplasm;	catalytic a	Pf00009, P	5906	ENSG0000	RAP1A	1	Renal cell c	Integrin-m	Signaling b	67	1.069		0.28896404	0.13	1460.6	1378.1	6.5	2.94	High	High	High	High	High	High	1	
FALSE	High	Master Prc	Q04206	Transcripti	0	21.81	9	4	6	4	551	60.2	5.68	14.75	4	defense re	cytoplasm;	DNA bindi	Pf00554	5970	ENSG0000	RELA	11	Epstein-Ba	Androgen	CLEC7A/in	153	1.069		0.445595042	16.82	1048.9	981.5	6.27	5.82	High	High	High	High	High	High	1	
FALSE	High	Master Prc	O95721	Synaptoso	0	10.549	12	2	2	2	258	29	5.81	6.73	2	cell organi	cytoplasm;	protein bir	Pf00835, P	9342	ENSG0000	SNAP29	22	SNARE inte		Intra-Golgi	8	1.069		0.494228433	37.79	47.7	44.6	57.66	13.37	High	High	High	High	High	High	1	
FALSE	High	Master Prc	O95372	acyl-protei	0	24.111	31	5	6	5	231	24.7	7.23	19.15	5	cellular coi	cytoplasm;	catalytic a	Pf00561, P	11313	ENSG0000	LYPLA2	1	Glyceroph		L1CAM int	4	1.069		0.536931188	23.48	704.6	648.7	12.13	12.62	High	High	High	High	High	High	1	
FALSE	High	Master Prc	Q9H3N1	Thioredoxi	0	21.915	19	5	6	5	280	31.8	4.98	10.55	5	cellular ho	endoplasm	catalytic a	Pf00085, P	81542	ENSG0000	TMX1	14				0	1.069		0.555405368	15.97	2268.9	2225.2	12.69	2.96	High	High	High	High	High	High	1	
FALSE	High	Master Prc	P78406	mRNA exp	0	78.589	38	10	19	10	368	40.9	7.83	65.09	10	cell divisio	cytoplasm;	protein bir	Pf00400	8480	ENSG0000	RAE1	20	RNA trans		Rev-media	65	1.069		0.897820512	2.26	4135.5	3960.1	9.5	2.43	High	High	High	High	High	High	1	
FALSE	High	Master Prc	Q96A49	Synapse-as	0	21.59	17	4	5	4	352	39.9	4.53	15.63	4		cytosol;Go	protein bir	Pf03909	94056	ENSG0000	SYAP1	X				0	1.068		0.073610847	3.65	1704.4	1581.6	1.53	2.63	High	High	High	High	High	High	1	
FALSE	High	Master Prc	Q9NRP2	COX assem	0.001	4.236	20	2	6	2	79	9.5	7.87	10.2	2		mitochond		Pf08583	56942	ENSG0000	CMC2	16			Mitochonc	2	1.068		0.077904022	1.51	1657.7	1466.8	4.22	4.54	High	High	High	High	High	High	1	
FALSE	High	Master Prc	Q9Y4Y9	U6 snRNA-	0	9.01	32	2	3	2	91	9.9	4.54	8.18	2	metabolic	cytosol;nu	protein bir	Pf01423	23658	ENSG0000	LSM5	7	RNA degra		mRNA Spli	8	1.068		0.095843928	2.19	3346.3	3134.6	2.97	0.19	High	High	High	High	High	High	1	
FALSE	High	Master Prc	Q86X29	Lipolysis-st	0	10.943	6	3	3	3	649	71.4	7.97	3.34	3	regulation	membrane			51599	ENSG0000	LSR	19			VLDL clear	5	1.068		0.237165726	5.36	501.9	436.7	5.81	9.32	High	High	High	High	High	High	1	
FALSE	High	Master Prc	Q13838-1	spliceosom	0	104.378	45	16	44	7	428	49	5.67	110.49	16	cell organi	cytoplasm;	catalytic a	Pf00270, P	7919	ENSG0000	DDX39B	6; CHR_HS	mRNA surv		Cleavage o	13	1.068		0.288878439	11.18	5577.5	4807.7	9.15	6.19	High	High	High	High	High	High	1	
FALSE	High	Master Prc	P49792	E3 SUMO-p	0	209.055	24	53	76	37	3224	358	6.2	144.85	53	cell organi	cytoplasm;	catalytic a	Pf00160, P	5903	ENSG0000	RANBP2	2	RNA trans		Rev-media	77	1.068		0.292620668	10.24	9044.2	8470.2	6.42	4.47	High	High	High	High	High	High	1	
FALSE	High	Master Prc	P20700	Lamin-B1 [	0	108.579	48	29	47	24	586	66.4	5.16	80.5	29	regulation	membrane	catalytic a	Pf00038, P	4001	ENSG0000	LMNB1	5	Apoptosis	Fas Ligand	Gene and p	34	1.068		0.30056477	11.33	6242.2	6050.8	11.92	4.26	High	High	High	High	High	High	1	
FALSE	High	Master Prc	O60341	Lysine-spe	0	43.887	19	10	11	10	852	92.8	6.52	29.6	10	cell organi	nucleus	catalytic a	Pf01593, P	23028	ENSG0000	KDM1A	1		Androgen	Activated I	16	1.068		0.320390009	9.01	1764.1	1613.4	1.48	7.57	High	High	High	High	High	High	1	
FALSE	High	Master Prc	Q07065	Cytoskelet	0	125.067	43	24	44	23	602	66	5.92	111.57	24	metabolic	cytoplasm;	RNA bindi	Pf13166	10970	ENSG0000	CKAP4	12	Protein pro		Post-transl	9	1.068		0.406213872	15.17	13188.8	13015	12.85	6.13	High	High	High	High	High	High	1	
FALSE	Medium	Master Prc	P51157-1	Ras-relatec	0.026	1.474	5	1	1	1	221	24.8	5.97	1.73	1	regulation	cytoplasm;	catalytic a	Pf00025, P	9364	ENSG0000	RAB28	4				0	1.068		0.491198226	15.22	464	430.5	1.67	12.56	High	High	High	High	High	High	1	
FALSE	High	Master Prc	P21283	V-type pro	0.001	4.533	4	2	2	2	382	43.9	7.46	1.89	2	cellular ho	cytoplasm;	catalytic a	Pf03223	528	ENSG0000	ATP6V1C1	8	Epithelial c		Transferrir	20	1.068		0.597394468	26.36	432.7	438.2	17.83	8.24	High	High	High	High	High	High	1	
FALSE	High	Master Prc	P82663	28S riboso	0	10.582	26	3	4	3	173	20.1	8.82	7.95	3	cell organi	membrane	structural	Pf05047	64432	ENSG0000	MRPS25	3			Mitochonc	6	1.068		0.73488487	4.23	343.8	330.5	4.62	6.06	High	High	High	High	High	High	1	
FALSE	Medium	Master Prc	Q86WZ6	Zinc finger	0.016	1.696	3	1	1	1	799	92	8.84	2.89	1	metabolic	nucleus	DNA bindi	Pf00096, P	7770	ENSG0000	ZNF227	19			Generic Tr	3	1.068		0.907292474	61.64	70.1	65.7	27.49	17.67	High	High	High	High	High	High	1	[Q20];
FALSE	High	Master Prc	Q96EY7-1	pentatrico	0	20.213	11	4	4	4	689	78.5	6.42	14.82	4	cell organi	cytosol;me	protein bir	Pf13041, P	55037	ENSG0000	PTCD3	2			Mitochonc	6	1.068		0.999590776	89.08	165.5	155	40.36	31.76	High	High	High	High	High	High	1	
FALSE	High	Master Prc	P38117	electron tr	0	71.935	65	14	31	14	255	27.8	8.1	64.75	14	metabolic	cytosol;mi	protein bir	Pf01012	2109	ENSG0000	ETFB	19			Protein me	7	1.067		0.002295337	0.3	13756.5	12916.7	0.91	1.53	High	High	High	High	High	High	1	[Q146;
FALSE	High	Master Prc	P54920	alpha-solul	0	24.284	28	6	7	6	295	33.2	5.36	14.73	6	cell commi	cytosol;me	protein bir	Pf14938	8775	ENSG0000	NAPA	19	Synaptic ve	Synaptic V	COPI-depe	19	1.067		0.030869608	0.6	1622.4	1461.3	4.75	5.04	High	High	High	High	High	High	1	
FALSE	High	Master Prc	P52597	Heterogen	0	101.928	42	12	46	10	415	45.6	5.58	169.27	12	metabolic	cytosol;me	protein bir	Pf00076, P	3185	ENSG0000	HNRNPF	10		Mecp2 anc	Gene and p	15	1.067		0.075355516	1.9	11221.1	10259	3.6	2.55	High	High	High	High	High	High	1	[Y306(100
FALSE	High	Master Prc	Q12797	Aspartyl/A	0	27.479	8	4	7	4	758	85.8	5.01	24.22	4	metabolic	cytoplasm;	catalytic a	Pf05118, P	444	ENSG0000	ASPH	8			Ion homeo	6	1.067		0.108715957	2.62	1505	1418.5	8.83	4.22	High	High	High	High	High	High	1	
FALSE	High	Master Prc	O43818	U3 small n	0	14.073	11	4	6	4	475	51.8	7.85	6.43	4	metabolic	nucleus	protein bir	Pf00400	9136	ENSG0000	RRP9	3			Major path	5	1.067		0.259084529	7.17	190.2	178.2	3.8	8.91	High	High	High	High	High	High	1	
FALSE	High	Master Prc	P50995	annexin A1	0	48.444	26	12	20	12	505	54.4	7.65	31.15	12	cell divisio	cytoplasm;	metal ion l	Pf00191	311	ENSG0000	ANXA11	10				0	1.067		0.336278254	12.93	3853.9	3349.1	13.26	5.79	High	High	High	High	High	High	1	
FALSE	High	Master Prc	P23229-1	integrin alp	0.003	3.357	1	1	1	1	1130	126.5	6.61	2.65	1	cell commi	cell surface	metal ion l	Pf01839, P	3655	ENSG0000	ITGA6	2	Toxoplasm	Integrin-m	Integrin ce	31	1.067		0.459397597	16.66	314.8	260.8	7.34	15.49	High	High	High	High	High	High	1	
FALSE	High	Master Prc	O60361	putative nu	0	94.492	66	8	61	1	137	15.5	8.57	176.32	8	metabolic	cytosol;nu	catalytic a	Pf00334	283458		NME2P1	12			Interconve	3	1.067		0.573313926	112.19	377.6	354.1	130.63	67.84	High	High	High	High	High	High	1	
FALSE	High	Master Prc	Q8TAP9	M-phase-s	0	15.938	19	2	2	2	179	19.1	10.23	11.83	2	cell divisio	cytoplasm;	protein bir	Pf15502	136647	ENSG0000	MPLKIP	7				0	1.067		0.573944798	29.61	154.7	129	11.85	61.71	High	High	High	High	High	High	1	[Q113;
FALSE	Low	Master Prc	Q8N9L1-1	Zinc finger	0.053	1.175	4	1	2	1	334	36.5	9.52	4.68	1		nucleus	DNA bindi	Pf13465	84107	ENSG0000	ZIC4	3				0	1.067		0.640639903	65.55	110.6	103.7	58.26	25.72	High	High	High	High	High	High	1	[K262]
FALSE	High	Master Prc	O75396	Vesicle-tra	0	63.789	35	7	25	7	215	24.6	6.92	51.96	7	cell organi	endoplasm	protein bir	Pf00957, P	9554		SEC22B	1	Phagosom		COPI-depe	20	1.067		0.706378078	10.47	7257	6799.6	16.61	3.83	High	High	High	High	High	High	1	
FALSE	Medium	Master Prc	Q5W111	SPRY doma	0.016	1.719	4	1	1	1	196	21.7	6.7	0	1			protein bir	Pf00622	57213	ENSG0000	SPRYD7	13				0	1.067		0.818714866	47.07	58.9	59.4	38.74	19.34	High	High	High	High	High	High	1	
FALSE	High	Master Prc	Q99439	Calponin-2	0	22.02	17	3	7	2	309	33.7	7.33	15.23	3	cell organi	cytoskelet	protein bir	Pf00307, P	1265	ENSG0000	CNN2	19		Myometria	Gene and p	8	1.067		0.861320876	15.66	461.3	470	18.83	11.21	High	High	High	High	High	High	1	
FALSE	High	Master Prc	P98175-1	RNA-bindir	0	56.405	13	9	12	9	930	103.5	5.97	28.24	9	metabolic	nucleus	metal ion l	Pf00076, P	8241	ENSG0000	RBM10	X				0	1.067		0.963265292	2.95	1413.4	1324.2	7.99	4.57	High	High	High	High	High	High	1	
FALSE	High	Master Prc	Q9NZZ3	Charged m	0	19.017	19	4	6	4	219	24.6	4.83	12.52	4	cell organi	cytoplasm;	protein bir	Pf03357	51510	ENSG0000	CHMP5	9	Endocytosi		Budding ar	10	1.066		0.194935924	3.94	489.1	458.8	13.47	5.54	High	High	High	High	High	High	1	
FALSE	High	Master Prc	Q9Y333	U6 snRNA-	0	7.526	20	1	1	1	95	10.8	6.52	5.81	1	metabolic	cytosol;nu	protein bir	Pf01423	57819	ENSG0000	LSM2	6; CHR_HS	Spliceosom	mRNA Pro	mRNA Spli	10	1.066		0.210831642	0.37	792.7	743.7	14.76	4.01	High	High	High	High	High	High	1	
FALSE	High	Master Prc	Q5EG05	Caspase re	0	21.473	20	3	6	2	197	22.6	8.44	13.08	3	regulation		enzyme re	Pf00619	114769	ENSG0000	CARD16	11	NOD-like r			1	1.066		0.304104654	4.99	38.2	25.6	23.05	28.72	High	High	High	High	High	High	1	
FALSE	High	Master Prc	Q9H0B6	Kinesin ligh	0	16.493	9	5	8	3	622	68.9	7.15	12.31	5	cellular coi	cytoplasm;	catalytic a	Pf00515, P	64837	ENSG0000	KLC2	11	Salmonella		COPI-depe	16	1.066		0.565971589	34.6	77.1	74.6	34.01	2.31	High	High	High	High	High	High	1	
FALSE	High	Master Prc	Q02818	Nucleobinc	0	34.665	19	9	15	9	461	53.8	5.25	33.71	9	metabolic	cytoplasm;	DNA bindi		4924	ENSG0000	NUCB1	19			Post-transl	4	1.066		0.599842412	13	3266.8	3084.4	9.6	2.83	High	High	High	High	High	High	1	
FALSE	High	Master Prc	P56134	ATP syntha	0	9.416	26	2	4	2	94	10.9	9.67	8.47	2	cell organi	membrane	catalytic a	Pf10206	9551	ENSG0000	ATP5J2	7	Oxidative p	Electron Ti	Cristae for	11	1.066		0.647527683	7.61	1119.1	1049.9	26.74	8.87	High	High	High	High	High	High	1	
FALSE	High	Master Prc	O95390	Growth/dif	0.006	2.441	1	1	3	1	407	45.1	7.93	1.83	1	regulation	extracellul	protein bir	Pf00019, P	10220	ENSG0000	GDF11	12				0	1.066		0.714305303	32.49	1764.6	1654.8	4.62	22.36	High	High	High	High	High	High	1	
FALSE	High	Master Prc	Q9BZX2-1	Uridine-cyt	0	17.919	16	3	4	3	261	29.3	6.7	15.78	3	metabolic	cytosol	catalytic a	Pf00485, P	7371	ENSG0000	UCK2	1	Metabolic	Fluoropyri	Pyrimidine	8	1.066		0.847259291	6.61	1914.6	1831.7	10.23	2.91	High	High	High	High	High	High	1	
FALSE	High	Master Prc	P55036-1	26S protea	0	25.916	19	5	6	5	377	40.7	4.79	10.77	5	cell organi	cytoplasm;	catalytic a	Pf02809, P	5710	ENSG0000	PSMD4	1	Epstein-Ba	Proteasom	Regulation	152	1.066		0.894040701	12.78	481.6	451.8	13.15	9.23	High	High	High	High	High	High	1	
FALSE	High	Master Prc	P35914	Hydroxyme	0	11.039	9	2	2	2	325	34.3	8.54	6.72	2	cell organi	membrane	catalytic a	Pf00682	3155	ENSG0000	HMGCL	1	Metabolic	Synthesis a	Synthesis c	11	1.066		0.984187528	37.9	267.4	250.7	23.51	11.46	High	High	High	High	High	High	1	
FALSE	High	Master Prc	O14975	very long-c	0	13.389	6	3	3	3	620	70.3	8.51	10.99	3	metabolic	endoplasm	catalytic a	Pf00501, P	11001	ENSG0000	SLC27A2	15	PPAR signa	PPAR signa	Neutrophil	17	1.065		0.103071633	1.36	571.2	536.5	3.62	6.39	High	High	High	High	High	High	1	
FALSE	High	Master Prc	P61586	Transformi	0	63.129	48	8	24	8	193	21.8	6.1	64.78	8	cell differe	cytoplasm;	catalytic a	Pf00025, P	387	ENSG0000	RHOA	3	Phospholip	Androgen	Rho GTPas	111	1.065		0.502107876	19.72	8246.9	7746.1	11.94	6.02	High	High	High	High	High	High	1	
FALSE	High	Master Prc	P38646	Stress-70 p	0	511.597	69	50	207	49	679	73.6	6.16	562.62	50	cell differe	cytoplasm;	nucleotide	Pf00012, P	3313	ENSG0000	HSPA9	5	Tuberculos	Parkin-Ubi	Gene and p	17	1.065		0.667855429	30.51	58848	55270.1	13.47	9.46	High	High	High	High	High	High	1	
FALSE	High	Master Prc	Q96RN5	Mediator c	0.003	3.136	2	2	2	2	788	86.7	9.42	4.53	2	metabolic	cytoplasm;	protein bir	Pf09606	51586	ENSG0000	MED15	22		Sterol Reg	Transcripti	11	1.065		0.78245216	40.19	575.7	540.5	13.95	22.29	High	High	High	High	High	High	1	
FALSE	High	Master Prc	Q96AE4	Far upstrea	0	176.668	54	28	72	2	644	67.5	7.61	138.21	28	metabolic	nucleus	DNA bindi	Pf00013, P	8880	ENSG0000	FUBP1	1				0	1.065		0.829274709	40.46	461.4	417.1	25.95	6.06	High	High	High	High	High	High	1	
FALSE	High	Master Prc	Q13011	Delta(3,5)-	0	116.884	49	11	35	11	328	35.8	8	110.59	11	metabolic	membrane	catalytic a	Pf00378	1891	ENSG0000	ECH1	19; CHR_H	Peroxisom	Fatty Acid		2	1.065		0.918023939	7.42	7872.6	7529	26.46	15.28	High	High	High	High	High	High	1	
FALSE	High	Master Prc	P35613	Basigin [OS	0	93.957	26	10	39	10	385	42.2	5.66	97.32	10	cell organi	membrane	protein bir	Pf00047, P	682	ENSG0000	BSG	19		Matrix Me	Integrin ce	15	1.064		0.01641235	1.07	9834	9305.6	3.37	2.19	High	High	High	High	High	High	1	
FALSE	High	Master Prc	Q969S9	Ribosome-	0.002	3.288	2	1	1	1	779	86.5	6.51	4.4	1	cell organi	mitochond	catalytic a	Pf00009, P	84340	ENSG0000	GFM2	5			Mitochonc	4	1.064		0.154074327	1.92	572.6	475.4	8.96	9.6	High	High	High	High	High	High	1	
FALSE	High	Master Prc	O75436-1	Vacuolar p	0	33.48	26	6	11	6	327	38.1	6.57	28.12	6	regulation	cytoplasm;	protein bir	Pf03643	9559	ENSG0000	VPS26A	10	Endocytosi		WNT ligan	4	1.064		0.168400858	3.46	2512.9	2320	5.21	3.1	High	High	High	High	High	High	1	
FALSE	High	Master Prc	Q9BRZ2-1	E3 ubiquiti	0	8.843	3	1	1	1	755	81.4	7.74	3.24	1	defense re	cytoplasm;	catalytic a	Pf00097, P	81844	ENSG0000	TRIM56	7			Regulation	4	1.064		0.315165128	9.24	96.3	90.5	4.55	10.24	High	High	High	High	High	High	1	
FALSE	High	Master Prc	O43143	Pre-mRNA	0	80.354	21	14	23	14	795	90.9	7.46	66.49	14	metabolic	cytoplasm;	catalytic a	Pf00270, P	1665	ENSG0000	DHX15	4	Spliceosom	mRNA Pro	mRNA Spli	6	1.064		0.409636711	15.08	9392.2	8825.9	18.77	4.63	High	High	High	High	High	High	1	
FALSE	High	Master Prc	Q8IWR0	Zinc finger	0	13.726	4	2	2	2	971	110.5	7.3	4.61	2	metabolic	nucleus	metal ion l	Pf00642, P	29066	ENSG0000	ZC3H7A	16				0	1.064		0.619085872	1.91	152.5	159.6	15.54	14.96	High	High	High	High	High	High	1	
FALSE	High	Master Prc	Q13451-1	Peptidyl-pr	0	21.308	18	8	8	7	457	51.2	5.9	12.26	8	metabolic	cytoplasm;	catalytic a	Pf00254, P	2289	ENSG0000	FKBP5	6	Estrogen s	Farnesoid	HSP90 cha	6	1.064		0.676113425	9.03	2207.8	2074.7	9.18	1.95	High	High	High	High	High	High	1	
FALSE	High	Master Prc	Q5SRE5	Nucleopori	0	40.737	8	10	11	10	1749	195.9	6.73	25.41	10	cell organi	membrane	structural	Pf10487, P	23511	ENSG0000	NUP188	9	RNA trans		Rev-media	64	1.064		0.757523102	9.64	918.3	869.5	5.13	7.46	High	High	High	High	High	High	1	

FALSE	High	Master Prc	P11233	Ras-relatec	0	25.791	33	7	13	4	206	23.6	7.11	20.3	7	cell divisio	cell surface	catalytic a	Pf00025, P	5898	ENSG0000	RALA	7	Rap1 signa	RalA down	Translocat	22	1.064		0.762175809	2.17	2421.9	2308.7	7.1	2.19	High	High	High	High	High	High	1	
FALSE	High	Master Prc	P08559	Pyruvate d	0	54.935	41	18	31	18	390	43.3	8.06	44.74	18	metabolic	mitochond	catalytic a	Pf00676	5160	ENSG0000	PDHA1	X	Carbon me	TCA Cycle	Regulation	22	1.064		0.80963084	0.41	10093.2	9717.8	10.56	5.09	High	High	High	High	High	High	1	
FALSE	High	Master Prc	Q9P2R7	Succinate--	0	54.036	30	12	21	12	463	50.3	7.42	45.33	12	metabolic	mitochond	catalytic a	Pf00549, P	8803	ENSG0000	SUCLA2	13	Metabolic		Citric acid	8	1.063		0.063751276	1.77	9701.5	9239.5	6.55	4.6	High	High	High	High	High	High	1	
FALSE	High	Master Prc	O94826	Mitochonc	0	111.873	43	21	38	21	608	67.4	7.12	94	21	cell commu	membrane	protein bir	Pf00515, P	9868	ENSG0000	TOMM70A	3			Ub-specific	7	1.063		0.135096392	0.71	16490.4	15509.9	9.26	4.05	High	High	High	High	High	High	1	
FALSE	High	Master Prc	Q70J99-1	Protein un	0	20.818	8	5	5	5	1090	123.2	6.65	13.01	5	defense re	cytoplasm;	protein bir	Pf00168, P	201294	ENSG0000	UNC13D	17		Deregulati	Neutrophil	4	1.063		0.23455524	4.56	462.3	423.1	6.41	5.82	High	High	High	High	High	High	1	
FALSE	High	Master Prc	Q9UBE0-1	SUMO-acti	0	57.941	55	14	17	14	346	38.4	5.3	43.54	14	metabolic	cytoplasm;	catalytic a	Pf00899	10055	ENSG0000	SAE1	19	Ubiquitin r		SUMO is c	7	1.063		0.282946594	9.2	3770.6	3561.3	7.82	2.24	High	High	High	High	High	High	1	
FALSE	High	Master Prc	Q8TAE8	Growth ar	0	10.499	14	2	3	2	222	25.4	10.02	3.97	2	cell organi	mitochond	protein bir	Pf10147	90480	ENSG0000	GADD45GI	19			Mitochonc	6	1.063		0.342371191	12.04	1350.5	1276.2	5.92	3.23	High	High	High	High	High	High	1	
FALSE	High	Master Prc	Q9GZU8	Protein FA	0	10.932	10	3	3	3	254	28.9	5.45	2.84	3		nucleus	protein bir	Pf10187	80011	ENSG0000	FAM192A	16				0	1.063		0.441514124	16.85	482.4	453.8	8.58	11.31	High	High	High	High	High	High	1	
FALSE	High	Master Prc	Q15843	NEDD8 [OS	0	10.512	43	4	9	4	81	9.1	8.43	6.78	4	cellular ho	cytosol;nu	protein bir	Pf00240, P	4738	ENSG0000	NEDD8	14		EGF/EGFR	Cargo reco	16	1.063		0.57395125	29.42	2710.2	2587.7	24.69	5.53	High	High	High	High	High	High	1	
FALSE	High	Master Prc	Q9UI15	Transgelin-	0	10.315	18	3	5	2	199	22.5	7.33	3.64	3	regulation	nucleus	protein bir	Pf00307, P	29114	ENSG0000	TAGLN3	3				0	1.063		0.926473065	30.52	889.8	1029.5	29.88	16.08	High	High	High	High	High	High	1	
FALSE	High	Master Prc	P21281	V-type pro	0	62.02	34	10	15	10	511	56.5	5.81	58.85	10	cellular ho	cytoplasm;	catalytic a	Pf00006, P	526	ENSG0000	ATP6V1B2	8	Metabolic		Transferrir	20	1.063		0.944338542	2.57	2411	2403.1	5.78	8.72	High	High	High	High	High	High	1	
FALSE	High	Master Prc	Q9BZZ5-1	Isoform 1 (	0	12.673	9	3	4	3	438	49.5	6.46	7.14	3	cell death;	cytoplasm;	protein bir	Pf05918	8539		API5	11				0	1.063		0.966902064	13.8	271.4	257.1	12.28	6.65	High	High	High	High	High	High	1	
FALSE	High	Master Prc	P52294	Importin su	0	30.544	16	6	10	3	538	60.2	5.01	30.59	6	metabolic	cytoplasm;	protein bir	Pf00514, P	3836	ENSG0000	KPNA1	3	Influenza A		Integration	25	1.062		0.038183798	1.22	651.4	592.9	7.69	7.39	High	High	High	High	High	High	1	
FALSE	High	Master Prc	P00533-1	epidermal	0	128.756	25	22	36	22	1210	134.2	6.68	109.77	22	cell organi	cell surface	catalytic a	Pf00069, P	1956	ENSG0000	EGFR	7	Breast can	Androgen	EGFR inter	115	1.062		0.172477182	1.65	8881.2	8592.2	2.83	2.23	High	High	High	High	High	High	1	
FALSE	High	Master Prc	O75438	NADH deh	0	9.049	19	1	5	1	58	7	8.92	14.77	1	cell organi	membrane	catalytic a	Pf08040	4707	ENSG0000	NDUFB1	14	Alzheimer'	Electron Tr	Complex I	13	1.062		0.188608988	3.63	1610.2	1604.5	9.35	7.79	High	High	High	High	High	High	1	
FALSE	High	Master Prc	O15479	Melanoma	0.001	4.51	6	3	3	2	319	35.3	8.76	0	3			protein bir	Pf01454	4113	ENSG0000	MAGEB2	X				0	1.062		0.208156731	1.59	72.6	69.1	8.89	21.73	High	High	High	High	High	High	1	
FALSE	High	Master Prc	Q96G23	Ceramide s	0	12.755	12	3	5	3	380	44.8	8.98	5.18	3	metabolic	endoplasm	catalytic a	Pf03798	29956	ENSG0000	CERS2	1	Sphingolip	Sphingolip	Sphingolip	8	1.062		0.346855746	12.11	1628.2	1476.4	8.32	4.89	High	High	High	High	High	High	1	
FALSE	High	Master Prc	Q9NX24	H/ACA ribc	0	34.134	41	3	10	3	153	17.2	8.22	36.92	3	cell organi	nucleus	protein bir	Pf01248	55651	ENSG0000	NHP2	5	Ribosome		rRNA mod	10	1.062		0.374781102	12.44	1326	1253.8	25.1	10.89	High	High	High	High	High	High	1	
FALSE	High	Master Prc	Q9UBT2	SUMO-acti	0	44.947	22	12	15	12	640	71.2	5.29	31.72	12	metabolic	cytoplasm;	catalytic a	Pf00899, P	10054	ENSG0000	UBA2	19	Ubiquitin r		SUMO is c	7	1.062		0.37894619	11.69	5308.1	4968.3	13.89	8.74	High	High	High	High	High	High	1	
FALSE	High	Master Prc	P01116-2	Isoform 2B	0	27.003	52	6	7	3	188	21.4	8.12	20.74	6	cell commu	cytoplasm;	catalytic a	Pf00009, P	3845	ENSG0000	KRAS	12		IL-5 Signali		21	1.062		0.422925264	15.99	1453.9	1368.5	18.11	5.46	High	High	High	High	High	High	1	
FALSE	High	Master Prc	O15067	Phosphorib	0	54.591	12	11	15	11	1338	144.6	5.76	39.84	11	metabolic	cytoplasm;	catalytic a	Pf00586, P	5198	ENSG0000	PFAS	17	Purine met		Purine ribc	6	1.062		0.487995506	18.04	3812.4	3550	10.4	8.48	High	High	High	High	High	High	1	
FALSE	High	Master Prc	Q96P11-1	Probable 2	0	20.185	13	4	5	4	429	46.7	8.62	14.08	4	metabolic	nucleus	catalytic a	Pf01189	55695	ENSG0000	NSUN5	7				0	1.062		0.50097537	25.4	1327.5	1269.2	29.85	5.27	High	High	High	High	High	High	1	
FALSE	High	Master Prc	Q99447-1	ethanolam	0	9.395	7	2	2	2	389	43.8	6.92	0	2	metabolic	membrane	catalytic a	Pf01467	5833	ENSG0000	PCYT2	17	Metabolic	Kennedy p	Synthesis c	9	1.062		0.58833224	49.43	572.8	662.2	58.23	15.14	High	High	High	High	High	High	1	
FALSE	High	Master Prc	Q96HY6-1	DDRGK do	0	25.44	18	4	6	4	314	35.6	5.12	17.24	4	regulation	cytoplasm;	protein bir	Pf09756	65992	ENSG0000	DDRGK1	20				0	1.061		0.081122754	0.26	1896.6	1807.7	4.12	2.64	High	High	High	High	High	High	1	
FALSE	High	Master Prc	P31948	stress-indu	0	191.131	65	39	106	39	543	62.6	6.8	175.27	39	response t	cytoplasm;	protein bir	Pf00515, P	10963	ENSG0000	STIP1	11	Prion disea		HSP90 cha	4	1.061		0.418314168	4.18	27919.4	26808.6	6.81	1.6	High	High	High	High	High	High	1	
FALSE	High	Master Prc	Q8TBC4-1	NEDD8-act	0	36.812	17	5	8	5	463	51.8	5.45	23.24	5	metabolic	cytoplasm;	catalytic a	Pf00899, P	9039	ENSG0000	UBA3	3	Ubiquitin r		NIK-->nonc	15	1.061		0.515380276	24.36	2682.2	2527.8	23.44	9.34	High	High	High	High	High	High	1	
FALSE	High	Master Prc	Q9Y282-1	Endoplasm	0	10.808	7	3	3	3	383	43.2	6.06	2.21	3	transport	endoplasm		Pf07970, P	51614	ENSG0000	ERGIC3	20				0	1.061		0.68694813	25.3	809.5	763	8.32	9.82	High	High	High	High	High	High	1	
FALSE	High	Master Prc	Q96AC1	Fermitin fa	0	19.791	5	3	5	3	680	77.8	6.7	15.12	3	cell organi	cell surface	protein bir	Pf00169, P	10979	ENSG0000	FERMT2	14			Cell-extrac	3	1.061		0.940948028	13.95	1050.8	1003.2	12.29	6.12	High	High	High	High	High	High	1	
FALSE	High	Master Prc	Q9BSY4	Coiled-coil	0	12.279	28	2	2	2	110	12.4	6.73	5.9	2	cell organi	mitochond		Pf06747	84269	ENSG0000	CHCHD5	2			Mitochonc	2	1.061		0.989232044	1.52	251.2	252.7	6.32	6.57	High	High	High	High	High	High	1	
FALSE	High	Master Prc	O95456	Proteasom	0	8.498	11	3	4	3	288	32.8	7.17	9.99	3	cell organi	cytoplasm;	protein bir		8624	ENSG0000	PSMG1	21				0	1.06		0.094419621	4.63	1352.6	1286.9	2.91	0.92	High	High	High	High	High	High	1	
FALSE	High	Master Prc	P61421	V-type pro	0	23.658	17	5	6	5	351	40.3	5	18.12	5	cell organi	endosome	catalytic a	Pf01992	9114	ENSG0000	ATP6V0D1	16	Collecting		Transferrir	26	1.06		0.213166531	4.36	2838	2646.6	7.73	2.91	High	High	High	High	High	High	1	
FALSE	High	Master Prc	Q9UBK8-1	Methionin	0	19.16	7	4	5	4	725	80.4	6.49	12.75	4	metabolic	cytoplasm;	catalytic a	Pf00175, P	4552	ENSG0000	MTRR	5		Vitamin B1	Defective I	18	1.06		0.288990057	9.43	2043.7	1927.2	4.79	4.59	High	High	High	High	High	High	1	
FALSE	High	Master Prc	Q9NZB2-1	Constitutiv	0	37.647	9	7	7	7	1118	121.8	8.88	23.24	7		cytoplasm;	RNA bindir		23196	ENSG0000	FAM120A	9				0	1.06		0.477914066	22.28	2998.5	2879.3	29.32	4.82	High	High	High	High	High	High	1	
FALSE	High	Master Prc	P09493-5	Isoform 5 (	0	45.716	36	12	24	2	245	28.4	4.77	36.88	12	cell organi	cytoskeleto	protein bir	Pf00261, P	7168	ENSG0000	TPM1	15		Striated M		1	1.06		0.553313743	8.57	309.6	291.6	7.95	4.13	High	High	High	High	High	High	1	
FALSE	High	Master Prc	Q9P0V3-1	SH3 domai	0	5.763	2	1	1	1	963	107.4	7.71	4.53	1	regulation	cytoplasm;	enzyme re	Pf00018, P	23677	ENSG0000	SH3BP4	2				0	1.06		0.556910494	37.39	192.5	181.5	39.35	7.74	High	High	High	High	High	High	1	
FALSE	High	Master Prc	Q96GQ7	Probable A	0	63.054	25	18	26	18	796	89.8	9.28	51.57	18	metabolic	chromosor	catalytic a	Pf00270, P	55661	ENSG0000	DDX27	20				0	1.06		0.652632562	22.43	8931.1	8869.3	13.22	6.4	High	High	High	High	High	High	1	
FALSE	High	Master Prc	P55327	Tumor pro	0	41.154	31	6	9	6	224	24.3	4.83	23.14	6	cell differe	cytoplasm;	metal ion I	Pf04201	7163	ENSG0000	TPD52	8			Golgi Asso	5	1.06		0.80253939	14.31	2889.3	2615.9	15.34	6.83	High	High	High	High	High	High	1	
FALSE	High	Master Prc	P30566	adenylosuc	0	14.16	11	3	3	3	484	54.9	7.11	10.8	3	cell organi	cytosol;mi	catalytic a	Pf00206, P	158	ENSG0000	ADSL	22	Metabolic	Nucleotide	Purine ribc	8	1.059		0.142074713	6.56	393	388.2	6.35	9.54	High	High	High	High	High	High	1	
FALSE	High	Master Prc	Q92896	Golgi appa	0	9.422	4	3	4	3	1179	134.5	6.9	6.36	3	cellular co	Golgi;mem	protein bir	Pf00839	2734	ENSG0000	GLG1	16	Cell adhesi		Cell surfac	3	1.059		0.175252775	1.28	600.8	600	6.33	6.03	High	High	High	High	High	High	1	
FALSE	High	Master Prc	P07942	Laminin su	0	43.601	8	10	11	10	1786	197.9	4.94	27.95	10	cell differe	extracellul	catalytic a	Pf00015, P	3912	ENSG0000	LAMB1	7	Small cell I	Alpha 6 Be	ECM prote	28	1.059		0.188498361	2.75	2657.1	2544	9.66	3.86	High	High	High	High	High	High	1	
FALSE	High	Master Prc	Q14677-1	Clathrin in	0	49.11	12	6	16	6	625	68.2	6.42	64.11	6	cell organi	cytoplasm;	protein bir	Pf01417, P	9685	ENSG0000	CLINT1	5			Golgi Asso	5	1.059		0.273939151	8.92	3155.1	3060.2	2.2	5.66	High	High	High	High	High	High	1	
FALSE	High	Master Prc	Q9GZY8-1	Mitochonc	0	6.403	5	1	1	1	342	38.4	8.95	3.9	1	cell organi	membrane	protein bir	Pf05644	56947	ENSG0000	MFF	2				0	1.059		0.31629508	6.8	536.9	484.6	12.21	6.03	High	High	High	High	High	High	1	
FALSE	High	Master Prc	Q12765	Secernin-1	0	33.3	23	9	17	8	414	46.4	4.75	37.51	9	metabolic	cytoplasm;	catalytic a	Pf03577	9805	ENSG0000	SCRN1	7				0	1.059		0.349099007	8.24	4019.6	3716.6	17.01	4.71	High	High	High	High	High	High	1	
FALSE	High	Master Prc	O60427	Fatty acid (	0	22.354	11	4	4	4	444	51.9	8.87	14.15	4	cell commu	endoplasm	catalytic a	Pf00173, P	3992		FADS1	11	Biosynthes		PPARA act	10	1.059		0.635622425	11	906	841.9	12.15	1.65	High	High	High	High	High	High	1	
FALSE	High	Master Prc	Q9UKM9-1	RNA-bindi	0	59.132	40	12	33	12	306	32.4	9.17	66.51	12	metabolic	nucleus;sp	protein bir	Pf00076, P	22913	ENSG0000	RALY	20				0	1.059		0.701233037	3.41	11871.8	11578	8.29	2.11	High	High	High	High	High	High	1	[S135(100
FALSE	High	Master Prc	Q9BR76	Coronin-1E	0	12.082	11	3	6	3	489	54.2	5.88	12.68	3	cell organi	cytoplasm;	protein bir	Pf00400, P	57175	ENSG0000	CORO1B	11				0	1.059		0.784754941	9.75	2852.9	2706.2	7.54	5.12	High	High	High	High	High	High	1	
FALSE	High	Master Prc	Q5JSZ5-1	Protein PR	0	17.66	4	6	6	6	2229	242.8	8.34	9.53	6	cell differe		protein bir	Pf07001	84726	ENSG0000	PRRC2B	9				0	1.058		0.265527069	6.18	899.2	833.2	9.32	4.8	High	High	High	High	High	High	1	
FALSE	High	Master Prc	Q02750	Dual specif	0	30.593	19	5	8	5	393	43.4	6.62	23.88	5	cell differe	cytoplasm;	catalytic a	Pf00069, P	5604	ENSG0000	MAP2K1	15	Vascular sr	IL-5 Signali	MAP2K an	172	1.058		0.317023194	10.23	2433.1	2319.7	0.75	8.45	High	High	High	High	High	High	1	
FALSE	High	Master Prc	Q9NYY8	FAST kinas	0.003	2.926	2	1	1	1	710	81.4	8.05	3.33	1	cell organi	mitochond	catalytic a	Pf06743, P	22868	ENSG0000	FASTKD2	2				0	1.058		0.333565106	4.95	420.3	375.7	20.61	6.46	High	High	High	High	High	High	1	
FALSE	High	Master Prc	E9PRG8	Uncharact	0	5.678	11	1	1	1	122	13.8	11.69	1.81	1					10228841	ENSG0000	LOC10228	11				0	1.058		0.444149622	15.09	683.6	597.2	10.98	4.61	High	High	High	High	High	High	1	
FALSE	High	Master Prc	Q96CW1	AP-2 comp	0	31.305	21	6	8	6	435	49.6	9.54	22.29	6	cell organi	cytosol;me	protein bir	Pf00928	1173	ENSG0000	AP2M1	3	Huntingto	Synaptic V	Cargo reco	47	1.058		0.568983255	4.79	401.5	379.4	1.34	8.51	High	High	High	High	High	High	1	
FALSE	Medium	Master Prc	Q9H7E2-1	Tudor dom	0.015	1.775	4	1	1	1	651	73.1	9.23	2.47	1	cell organi	cytoplasm;	protein bir	Pf00567, P	81550	ENSG0000	TDRD3	13				0	1.058		0.582638156	27.49	212.8	201.1	13.19	15.07	High	High	High	High	High	High	1	[T149(99)
FALSE	High	Master Prc	P55769	NHP2-like	0	35.478	34	6	10	6	128	14.2	8.46	21.5	6	cell organi	nucleus;sp	protein bir	Pf01248	4809	ENSG0000	NHP2L1; S	22	Spliceosom	mRNA Pro	Major path	12	1.058		0.590393041	1.74	3205.1	2993.1	8.43	2.1	High	High	High	High	High	High	1	
FALSE	High	Master Prc	Q5VIR6-1	Vacuolar p	0	9.685	6	3	3	3	699	79.6	6.02	6.58	3	transport	cytosol;en	protein bir	Pf04100, P	55275	ENSG0000	VPS53	17; CHR_H			Retrograde	4	1.058		0.785654932	3.7	1073.7	1024.9	12.05	5.87	High	High	High	High	High	High	1	
FALSE	High	Master Prc	Q9NXH9-1	tRNA (guai	0	26.384	11	5	5	5	659	72.2	7.64	11.42	5	metabolic	mitochond	catalytic a	Pf00642, P	55621	ENSG0000	TRMT1	19			tRNA modi	3	1.058		0.811727758	37.24	833.4	824.2	18.28	13.55	High	High	High	High	High	High	1	
FALSE	High	Master Prc	O43181	NADH deh	0	6.977	14	2	3	2	175	20.1	10.3	9.07	2	cell organi	membrane	catalytic a	Pf04800	4724	ENSG0000	NDUFS4	5	Parkinson's	Electron Tr	Complex I	13	1.058		0.883990441	12.29	501.4	485.4	7.33	9.2	High	High	High	High	High	High	1	
FALSE	High	Master Prc	Q9NR33	DNA polym	0	10.091	24	2	2	2	117	12.2	4.92	8.48	2	cell organi	nucleus	catalytic a	Pf00125, P	56655	ENSG0000	POLE4	2	Metabolic		Gap-filling	43	1.058		0.946281756	14.28	409.5	393.1	8.98	9.53	High	High	High	High	High	High	1	
FALSE	High	Master Prc	P36957	Dihydrolip	0	33.743	17	8	15	8	453	48.7	8.95	25.34	8	metabolic	membrane	catalytic a	Pf00198, P	1743	ENSG0000	DLST	14	Citrate cyc	TCA Cycle	Lysine cata	15	1.057		0.162997416	2.58	4389.8	4154.6	0.81	6.38	High	High	High	High	High	High	1	
FALSE	High	Master Prc	Q00403	Transcripti	0	6.71	13	2	2	2	316	34.8	8.35	5.77	2	cell organi	nucleus	DNA bindi	Pf00382, P	2959	ENSG0000	GTF2B	1	Viral carcir	Eukaryotic	RNA polym	20	1.057		0.191616155	4.64	331.7	313.8	5.01	12.67	High	High	High	High	High	High	1	
FALSE	High	Master Prc	Q9UHB6	LIM domai	0	145.172	36	23	43	23	759	85.2	6.84	112.5	23	cell organi	cytoplasm;	metal ion I	Pf00412	51474	ENSG0000	LIMA1	12				0	1.057		0.27589601	2.03	13879.4	13127.9	13.42	2.51	High	High	High	High	High	High	1	
FALSE	High	Master Prc	Q15836	Vesicle-ass	0	38.502	41	4	6	2	100	11.3	8.79	26.89	4	cell organi	cell surface	protein bir	Pf00957	9341	ENSG0000	VAMP3	1	Phagosom		Cargo reco	13	1.057		0.412528621	13.58	1268	1200.1	7.48	3.74	High	High	High	High	High	High	1	
FALSE	High	Master Prc	Q15014	Mortality f	0	5.921	8	2	3	2	288	32.3	9.72	6.47	2	cell organi	membrane	protein bir	Pf05712	9643	ENSG0000	MORF4L2	X			HATs acety	3	1.057		0.432230698	15.07	754.8	713.8	4.82	15.24	High	High	High	High	High	High	1	
FALSE	High	Master Prc	Q86WB0	Nuclear-in	0	25.512	13	5	5	5	502	55.2	5.62	11.01	5	cell divisio	membrane	metal ion I	Pf07967, P	51530	ENSG0000	ZC3HC1	7				0	1.057		0.467082247	15.84	1519.3	1518.2	13.63	3.6	High	High	High	High	High	High	1	
FALSE	High	Master Prc	P56192	Methionin	0	61.844	20	13	22	13	900	101.1	6.16	37.55	13	cell commu	cytoplasm;	catalytic a	Pf00043, P	4141	ENSG0000	MARS	12	Aminoacyl		Cytosolic t	10	1.057		0.77956575	11.67	7782	7945.5	13.6	4.58	High	High	High	High	High	High	1	
FALSE	High	Master Prc	P10515	Dihydrolip	0	64.687	24	12	20	12	647	69	7.84	50.59	12	metabolic	mitochond	catalytic a	Pf00198, P	1737	ENSG0000	DLAT	11	Metabolic	TCA Cycle	Regulation	17	1.057		0.81483531	11.73	9059.4	8638.3	16.44	7.27	High	High	High	High	High	High	1	
FALSE	High	Master Prc	Q13564-1	NEDD8-act	0	25.029	12	4	9	4	534	60.2	5.4	16.71	4	cell death;	cytoplasm;	catalytic a	Pf00899	8883	ENSG0000	NAE1	16	Alzheimer'	Alzheimers	Neddylatic	5	1.057		0.89439889	9.16	3669.9	3736.6	11.83	5.13	High	High	High	High	High	High	1	
FALSE	High	Master Prc	Q9UNX3	60S riboso	0	31.878	51	10	16	2	145	17.2	10.55	40.49	10	metabolic	ribosome	RNA bindir	Pf00467	51121	ENSG0000	RPL26L1	5	Ribosome		Peptide ch	33	1.057		0.958588717	19.07	1787.2	1819.2	17.96	6.53	High	High	High	High	High	High	1	[R26]
FALSE	High	Master Prc	P28072	Proteasom	0	45.272	47	7	18	7	239	25.3	4.92	29.57	7	metabolic	cytoplasm;	catalytic a	Pf00227	5694	ENSG0000	PSMB6	17	Proteasom	Proteasom	Regulation	150	1.057		0.98952041	9.18	1412.5	1315.1	17.56	1.77	High	High	High	High	High	High	1	
FALSE	High	Master Prc	P63241-1	Eukaryotic	0	108.721	72	12	59	12	154	16.8	5.24	129.68	12	cell death;	cytoplasm;	protein bir	Pf01287	1984	ENSG0000	EIF5A	17		Translatior	Hypusine s	6	1.056		0.023234541	1.81	20177.5	19346.4	6.09	7.78	High	High	High	High	High	High	1	
FALSE	High	Master Prc	P02786	Transferrir	0	212.205	44	30	77	30	760	84.8	6.61	171.53	30	cell differe	cell surface	protein bir	Pf02225, P	7037	ENSG0000	TFRC	3	Hematopo	Iron metab	Cargo reco	15	1.056		0.030175803	0.27	28970.4	27423.2	2.54	0.71	High	High	High	High	High	High	1	[Q431]
FALSE	High	Master Prc	Q00325-1	Phosphate	0	37.488	27	9	28	9	362	40.1	9.38	46.71	9	transport	membrane	protein bir	Pf00153	5250	ENSG0000	SLC25A3	12				0	1.056		0.170954494	5.15	11423.4	10816.3	9.51	5.67	High	High	High	High	High	High	1	
FALSE	High	Master Prc	Q9BQI0	allograft ir	0	6.318	14	2	2	1	150	17.1	7.2	0	2		cytoplasm;	metal ion I	Pf13499	83543	ENSG0000	AIF1L	9				0	1.056		0.479821606	23.07	428.2	408.4	33.54	4.89	High	High	High	High	High	High	1	
FALSE	High	Master Prc	Q8N3X1	Formin-bir	0	16.853	5	4	5	4	1017	110.2	4.74	6.94	4			protein bir	Pf00397	23360	ENSG0000	FNBP4	11				0	1.056		0.51817915	32.87	1459.8	1382	39.52	14.21	High	High	High	High	High	High	1	
FALSE	High	Master Prc	P43487-2	Isoform 2 (	0	69.219	43	6	18	1	200	23.2	5.29	60.1	6	cell organi	cytoplasm;	enzyme re	Pf00638	5902	ENSG0000	RANBP1	22				0	1.056		0.623079921	25.37	523	548.2	9.86	19.77	High	High	High	High	High	High	1	
FALSE	Medium	Master Prc	O43505	beta-1,4-gl	0.016	1.691	2	1	1	1	415	47.1	7.2	1.62	1	cellular co	Golgi;mem	catalytic a	Pf13896	11041	ENSG0000	B3GNT1; B	11	Glycosami		Keratan su	11	1.056		0.787209553	22.34	299	283.1	8.47	9.28	High	High	High	High	High	High	1	
FALSE	High	Master Prc	O75179-1	Ankyrin re	0	29.277	3	7	7	4	2603	274.1	6.52	15.22	7	defense re	chromosor	protein bir	Pf00013, P	26057	ENSG0000	ANKRD17	4				0	1.056		0.907753256	28.78	1112.6	1053.9	9.56	14.31	High	High	High	High	High	High	1	
FALSE	High	Master Prc	Q7Z2Z2	Elongation	0	5.391	3	2	2	2	1120	125.4	5.91	3.22	2	cell organi		catalytic a	Pf00009, P	79631	ENSG0000	EFTUD1; E	15	Ribosome			1	1.055		0.36614059	9.71	87.4	82.8	22.37	5.3	High	High	High	High	High	High	1	
FALSE	High	Master Prc	O14672	Disintegrin	0.001	4.398	4	2	2	2	748	84.1	7.77	3.23	2	cell commu	cell surface	catalytic a	Pf00200, P	102	ENSG0000	ADAM10	15	Epithelial (	Alzheimers	Collagen d	41	1.055		0.44353593	17.8	372.1	345.2	32.46	13.56	High	High	High	High	High	High	1	
FALSE	High	Master Prc	P54819	Adenylate	0	58.61	51	10	26	10	239	26.5	7.81	62.05	10	metabolic	membrane	catalytic a	Pf00406, P	204	ENSG0000	AK2	1	Metabolic		Interconve	6	1.055		0.508800696	7.94	7080	6637.7	2.65	6.66	High	High	High	High	High	High	1	

FALSE	High	Master Prc	Q15642	Cdc42-inte	0	27.958	15	6	6	6	601	68.3	5.73	11.01	6	cell commu	cytoplasm;	enzyme re	Pf00018, P	9322	ENSG0000	TRIP10	19	Insulin sigr	Angiopoiet	Rho GTPas	8	1.055	0.75020682	23.28	189.2	181.3	18.92	17	High	High	High	High	High	High	1	
FALSE	High	Master Prc	P42771-1	cyclin-depe	0	31.35	45	4	10	4	156	16.5	5.81	23.43	4	cell organi:	cytoplasm;	catalytic ac	Pf12796, P	1029	ENSG0000	CDKN2A	9	HTLV-I infe	TP53 Netw	Cyclin D as	32	1.055	0.750726979	6.22	1859	1872.6	7.27	5.31	High	High	High	High	High	High	1	
FALSE	High	Master Prc	Q8IYB8	ATP-depen	0	21.691	5	3	6	3	786	87.9	7.99	15	3	cell organi:	mitochond	catalytic ac	Pf00271, P	6832	ENSG0000	SUPV3L1	10				0	1.055	0.87691009	17.01	708.4	671.6	10.61	12.55	High	High	High	High	High	High	1	
FALSE	High	Master Prc	Q00059	Transcripti	0	18.451	28	7	10	7	246	29.1	9.72	12.08	7	cell organi:	cytosol;mi	DNA bindi	Pf00505, P	7019	ENSG0000	TFAM	10	Huntingtor	Energy Me	Mitochonc	10	1.054	0.01193592	0.15	1157.9	1113.4	5.86	5.61	High	High	High	High	High	High	1	
FALSE	High	Master Prc	Q99459	Cell divisio	0	94.612	27	14	26	14	802	92.2	8.18	95.39	14	cell differe	cytoplasm;	DNA bindi	Pf00249, P	988	ENSG0000	CDC5L	6	Spliceosom		mRNA Spli	5	1.054	0.203826788	2.03	6970.5	6613.8	4.65	4.48	High	High	High	High	High	High	1	
FALSE	High	Master Prc	O43920	NADH deh	0	17.972	47	4	4	4	106	12.5	9.14	13.91	4	cell organi:	membrane	catalytic ac	Pf10200	4725	ENSG0000	NDUFS5	1	Oxidative p	Electron Tr	Complex I	13	1.054	0.3325506	7.8	1529	1432.4	13.68	1.15	High	High	High	High	High	High	1	
FALSE	High	Master Prc	Q8N163-1	Cell cycle a	0	94.102	27	18	26	18	923	102.8	5.22	70.01	18	cell death;	cytoplasm;	enzyme re	Pf14443, P	57805	ENSG0000	CCAR2; KIA	8			Regulation	4	1.054	0.376893738	11.1	4289.3	4068.2	8.28	7.28	High	High	High	High	High	High	1	
FALSE	High	Master Prc	O95573	long-chain	0	124.538	34	17	33	15	720	80.4	8.38	97.44	17	cell organi:	endoplasm	catalytic ac	Pf00501	2181	ENSG0000	ACSL3	2	Metabolic	Fatty Acid	Synthesis c	16	1.054	0.467135307	17.38	8851.9	8581.4	19.67	4.43	High	High	High	High	High	High	1	
FALSE	High	Master Prc	O95340	bifunctiona	0	58.445	22	11	15	10	614	69.5	8.03	43.2	11	coagulatio	cytosol	catalytic ac	Pf01583, P	9060	ENSG0000	PAPSS2	10	Selenocom	RANKL/RA	Transport a	20	1.054	0.497406801	22.72	3139.9	2978.7	27.38	0.92	High	High	High	High	High	High	1	
FALSE	High	Master Prc	Q9C0C2	182 kDa ta	0	106.809	18	23	31	23	1729	181.7	4.86	67.24	23	cell organi:	chromosor	protein bir	Pf15327	85456	ENSG0000	TNKS1BP1	11			TP53 regul	9	1.054	0.571774923	30.6	4171.8	3968.1	29.11	8.45	High	High	High	High	High	High	1	
FALSE	High	Master Prc	O15397-1	Importin-8	0	9.334	4	3	4	2	1037	119.9	5.16	9.3	3	regulation	cytoplasm;	protein bir	Pf03810, P	10526	ENSG0000	IPO8	12			Transcripti	3	1.054	0.668364867	49.35	249.7	236.9	40.31	12.8	High	High	High	High	High	High	1	
FALSE	High	Master Prc	O15540	Fatty acid-	0.006	2.5	5	1	2	1	132	14.9	5.59	4.49	1	cell differe	cytoplasm;	transporte	Pf00061, P	2173	ENSG0000	FABP7	6	PPAR signa	PPAR signa	Triglycerid	6	1.054	0.864421675	4.93	492	472.7	9.27	6.07	High	High	High	High	High	High	1	
FALSE	High	Master Prc	Q9ULT8	E3 ubiquiti	0	21.884	3	6	7	6	2610	289.2	5.35	12.63	6	metabolic	cytoplasm	catalytic ac	Pf00023, P	25831		HECTD1	14			Antigen pr	4	1.054	0.888943777	27.77	876.8	896.5	22.3	5.68	High	High	High	High	High	High	1	
FALSE	High	Master Prc	Q5T653	39S riboso	0	7.1	5	1	1	1	305	33.3	11.3	1.62	1	cell organi:	membrane	catalytic ac	Pf00181, P	51069	ENSG0000	MRPL2	6	Ribosome		Mitochonc	7	1.054	0.996113322	70.39	381.3	357.7	46.05	8.78	High	High	High	High	High	High	1	
FALSE	High	Master Prc	Q9UBX3	Mitochonc	0	28.415	21	4	8	4	287	31.3	9.54	30.05	4	metabolic	membrane	protein bir	Pf00153	1468	ENSG0000	SLC25A10	17	Proximal tu		Gluconeog	13	1.053	0.079503007	3.31	3382.9	3106.6	5.09	4.3	High	High	High	High	High	High	1	
FALSE	High	Master Prc	Q9NWT8	Aurora kin	0.002	4.013	5	1	1	1	199	22.3	10.76	2.14	1	cell organi:	membrane	catalytic ac	Pf08213	54998	ENSG0000	AURKAIP1	1			Mitochonc	6	1.053	0.301105918	8.48	772.7	721.4	7.76	1.52	High	High	High	High	High	High	1	
FALSE	High	Master Prc	Q15276	Rab GTPas	0	26.174	10	6	6	6	862	99.2	5.01	14.04	6	cell death;	cytoplasm;	enzyme re	Pf01496, P	9135	ENSG0000	RABEP1	17	Endocytosi		TBC/RABG	5	1.053	0.315006706	2.33	324.7	289.2	18.22	4.44	High	High	High	High	High	High	1	
FALSE	High	Master Prc	Q9H936	Mitochonc	0	22.995	22	5	7	5	323	34.4	9.29	15.55	5	transport	membrane	transporte	Pf00153	79751	ENSG0000	SLC25A22	11			Organic an	4	1.053	0.334790631	2.61	978	920.4	0.89	4.63	High	High	High	High	High	High	1	
FALSE	Low	Master Prc	A2A288-1	Probable ri	0.071	1.037	3	1	1	1	527	58	8.68	0	1	metabolic	cytoplasm;	catalytic ac	Pf11977	340152	ENSG0000	ZC3H12D	6				0	1.053	0.499286694	22.41	825.3	806.4	28.22	7.12	High	High	High	High	High	High	1	[T43(50);
FALSE	High	Master Prc	P62979	Ubiquitin-4	0	72.075	64	9	39	4	156	18	9.64	119.63	9	cell commu	cytoplasm;	metal ion l	Pf00240, P	6233	ENSG0000	RPS27A	2	Ribosome	Cytoplasm	NOTCH1 Ir	364	1.053	0.511798381	3.19	2160.1	2146.6	2.78	5.88	High	High	High	High	High	High	1	
FALSE	High	Master Prc	O43819	Protein SC	0	8.59	14	3	4	3	266	29.8	8.85	5.94	3	cell organi:	membrane	antioxidan	Pf00578, P	9997	ENSG0000	SCO2	22	Central car	Copper ho	TP53 Regu	11	1.053	0.697893721	40.94	46.1	43.9	29.51	8.98	High	High	High	High	High	High	1	
FALSE	High	Master Prc	Q9NXV6	CDKN2A-ir	0	16.343	9	4	5	4	580	61.1	9.01	12.17	4	regulation	nucleus	protein bir	Pf11952	55602	ENSG0000	CDKN2AIP	4				0	1.053	0.736685874	20.15	407.1	386.4	1.1	15.63	High	High	High	High	High	High	1	
FALSE	High	Master Prc	P67870	Casein kina	0	20.349	30	3	6	3	215	24.9	5.55	20.1	3	cell commu	chromosor	catalytic ac	Pf01214	1460	ENSG0000	CSNK2B	6; CHR_HS	Adherens j		WNT medi	47	1.053	0.798374131	11.92	1001.8	920.4	12.29	4.55	High	High	High	High	High	High	1	
FALSE	High	Master Prc	Q96S52	GPI transa	0	15.909	8	3	3	3	555	61.6	6.49	10.85	3	metabolic	endoplasm	catalytic ac	Pf10510	94005	ENSG0000	PIGS	17	Metabolic		Attachmer	6	1.052	0.119635372	5.07	1446.5	1375.1	3.95	7.36	High	High	High	High	High	High	1	
FALSE	High	Master Prc	P80723	Brain acid	0	336.367	87	21	174	21	227	22.7	4.63	442.9	21	cell differe	cytoplasm;	DNA bindi		10409	ENSG0000	BASP1	5				0	1.052	0.326376573	5.96	48528.9	48378.3	7.65	3.96	High	High	High	High	High	High	1	
FALSE	Low	Master Prc	Q8IXQ3	Uncharacte	0.065	1.081	6	1	1	1	194	21.1	5.01	0	1				Pf15017	55071	ENSG0000	C9orf40	9				0	1.052	0.415825862	12.5	260.8	247.9	5.2	8.45	High	High	High	High	High	High	1	
FALSE	High	Master Prc	O75306-1	NADH deh	0	46.328	19	7	16	7	463	52.5	7.55	40.83	7	cell organi:	membrane	catalytic ac	Pf00346	4720	ENSG0000	NDUFS2	1	Oxidative p	Electron Tr	Complex I	13	1.052	0.440248744	16.57	6959.3	6850.6	39.27	8.12	High	High	High	High	High	High	1	
FALSE	High	Master Prc	Q6ZRV2	Protein FA	0.008	2.214	2	1	1	1	1179	127	6.98	1.8	1	cell organi:	cytoplasm;	protein bir	Pf07894, P	286077	ENSG0000	FAM83H	8; CHR_HS				0	1.052	0.605301138	36.01	981.4	1114.5	40.34	10.33	High	High	High	High	High	High	1	
FALSE	High	Master Prc	P27540	Aryl hydro	0	5.447	2	1	1	1	789	86.6	6.54	3.96	1	cell differe	cytoplasm;	DNA bindi	Pf00010, P	405	ENSG0000	ARNT	1	Chemical c	Angiogene	Aryl hydro	23	1.052	0.607709979	29.53	559.7	554	25.39	5.79	High	High	High	High	High	High	1	
FALSE	High	Master Prc	Q9H9T3	elongator	0.003	2.893	3	2	2	2	547	62.2	8.88	4.52	2	cell organi:	cytoplasm;	catalytic ac	Pf04055	55140	ENSG0000	ELP3	8			HATs acety	3	1.052	0.707813543	16.05	663.3	630.9	8.36	5.74	High	High	High	High	High	High	1	
FALSE	High	Master Prc	Q13557	Calcium/ca	0	12.458	9	2	2	1	499	56.3	7.25	8.11	2	cell differe	cytoplasm;	catalytic ac	Pf00069, P	817	ENSG0000	CAMK2D	4	HIF-1 signa	Physiologic	Ion homeo	64	1.052	0.794020146	28.94	34.2	32.5	22.04	16.22	High	High	High	High	High	High	1	
FALSE	High	Master Prc	Q9Y2Z4	Tyrosine--t	0	24.04	18	6	6	6	477	53.2	8.98	19.1	6	metabolic	cytoplasm;	catalytic ac	Pf00579	51067	ENSG0000	YARS2	12	Aminoacyl		Mitochonc	5	1.051	0.196623195	1.33	1931.1	1837.5	5.05	1.9	High	High	High	High	High	High	1	
FALSE	High	Master Prc	P30405	Peptidyl-pr	0	72.4	70	12	27	10	207	22	9.38	48.63	12	cell death;	membrane	catalytic ac	Pf00160	10105	ENSG0000	PPIF	10	Parkinson's			5	1.051	0.209677563	6.54	6879.2	6345.7	4.72	2.6	High	High	High	High	High	High	1	
FALSE	High	Master Prc	Q7Z3B4	Nucleopori	0	19.865	11	5	5	5	507	55.4	7.02	14.69	5	cell organi:	membrane	protein bir	Pf13874	53371	ENSG0000	NUP54	4	RNA transp		Rev-media	64	1.051	0.232399627	2.18	2016	1914.3	2.02	4.24	High	High	High	High	High	High	1	
FALSE	High	Master Prc	O95801	Tetratricop	0	5.21	5	2	2	2	387	44.7	5.6	5.2	2			protein bir	Pf13414, P	7268	ENSG0000	TTC4	1				0	1.051	0.416074551	12.94	712.9	632.7	11.61	4.49	High	High	High	High	High	High	1	
FALSE	High	Master Prc	Q8N9N8	probable R	0	10.118	18	2	3	2	165	19	5.21	11.55	2	metabolic	nucleus	protein bir	Pf01176	84285	ENSG0000	EIF1AD	11				0	1.051	0.458255555	14.46	1384.5	1317.9	10.01	6.36	High	High	High	High	High	High	1	
FALSE	High	Master Prc	Q8WVM8	Sec1 family	0	30.244	13	6	7	6	642	72.3	6.27	17.16	6	cell organi:	cytoplasm;	protein bir	Pf00995	23256	ENSG0000	SCFD1	14			COPII (Coa	8	1.051	0.485260591	4.59	1591.7	1524.3	0.75	5.59	High	High	High	High	High	High	1	
FALSE	Medium	Master Prc	P61962	DDB1- and	0.011	2.016	3	1	1	1	342	38.9	5.52	2.92	1	developme	cytoplasm;	protein bir	Pf00400	10238	ENSG0000	DCAF7	17			Associatior	6	1.051	0.511482581	19.1	730.6	675.3	13.09	12.78	High	High	High	High	High	High	1	
FALSE	High	Master Prc	P11182	Lipoamide	0	6.825	5	2	3	2	482	53.5	8.51	8.09	2	metabolic	mitochond	catalytic ac	Pf00198, P	1629		DBT	1	Valine, leu		Branched-c	7	1.051	0.52654804	21	771.8	734.5	16.09	8.49	High	High	High	High	High	High	1	
FALSE	High	Master Prc	Q9UJX3-1	Anaphase-	0	5.014	4	2	2	2	599	66.8	5.64	2.78	2	cell divisio	cytosol;nu	protein bir	Pf00515, P	51434	ENSG0000	ANAPC7	12	HTLV-I infe		APC/C:Cdc	37	1.051	0.589951783	2.6	49.5	59	15.55	19.67	High	High	High	High	High	High	1	
FALSE	High	Master Prc	P51571	translocon	0	33.51	31	4	10	4	173	19	6.15	23	4		endoplasm		Pf05404	6748	ENSG0000	SSR4	X	Protein prc		SRP-depen	4	1.051	0.66193163	32	3703.3	3276	24.64	4.72	High	High	High	High	High	High	1	
FALSE	High	Master Prc	Q9HB09-1	Bcl-2-like p	0	4.823	4	1	1	1	334	36.8	9.22	4.85	1	cell death;	membrane	protein bir		83596	ENSG0000	BCL2L12	19		Regulation		1	1.051	0.834536321	8.27	118	116.7	12.11	7.3	High	High	High	High	High	High	1	
FALSE	High	Master Prc	Q7L592	Protein arg	0.003	3.325	4	1	1	1	441	49.2	8.34	0	1	cell organi:	mitochond	catalytic ac	Pf02636	55471	ENSG0000	NDUFAF7	2			Complex I	5	1.051	0.967969882	25.24	479.9	456.6	4.66	16.95	High	High	High	High	High	High	1	
FALSE	High	Master Prc	Q7Z4V5	Hepatoma	0	21.797	12	7	9	5	671	74.3	7.49	18.89	7	regulation	nucleus	protein bir	Pf00855, P	84717	ENSG0000	HDGFRP2;	19				0	1.051	0.971419986	0.39	842.4	852.3	4.5	6.66	High	High	High	High	High	High	1	
FALSE	High	Master Prc	Q86SX6	Glutaredox	0	5.955	9	1	1	1	157	16.6	6.79	3.25	1	cellular ho	mitochond	catalytic ac	Pf00462	51218	ENSG0000	GLRX5	14			Mitochonc	2	1.051	0.996930599	57.49	290.2	276	30	13.31	High	High	High	High	High	High	1	
FALSE	High	Master Prc	P09012	U1 SMALL	0	23.248	16	4	9	3	282	31.3	9.83	20.25	4	metabolic	nucleus;sp	protein bir	Pf00076, P	6626	ENSG0000	SNRPA	19	Spliceosom	mRNA Pro	mRNA Spli	6	1.05	0.32308278	2.46	1768.3	1615.8	14.1	8.85	High	High	High	High	High	High	1	
FALSE	High	Master Prc	P49750-1	YLP motif-c	0	14.681	3	4	5	4	1951	219.8	6.57	12.52	4	metabolic	cytosol;nu	protein bir	Pf13207, P	56252		YLPM1	14				0	1.05	0.444244856	15.18	2217.5	2046.3	18.58	14.39	High	High	High	High	High	High	1	
FALSE	High	Master Prc	P06753-2	Isoform 2 c	0	182.569	79	35	109	25	248	29	4.78	219.46	35	cell organi:	cytoskelet	protein bir	Pf00261, P	7170	ENSG0000	TPM3	1		Striated M		1	1.05	0.518051816	6	29888.4	28285.2	6.63	3.22	High	High	High	High	High	High	1	
FALSE	High	Master Prc	Q14118	Dystroglyc	0	20.257	7	5	6	5	895	97.4	8.56	12.31	5	cell differe	cytoplasm;	metal ion l	Pf05454	1605	ENSG0000	DAG1	3	Hypertropl	Integrated	ECM prote	25	1.05	0.540332282	11.91	2715.6	2596.4	8.59	0.97	High	High	High	High	High	High	1	
FALSE	High	Master Prc	O00170	AH recepto	0	26.672	23	6	9	6	330	37.6	6.29	25.14	6	metabolic	cytoplasm;	protein bir	Pf00254	9049		AIP	11		Aryl Hydro	Gene and p	11	1.05	0.571021393	34.09	3007.2	3044.9	38.13	5.93	High	High	High	High	High	High	1	
FALSE	High	Master Prc	Q8N684-1	Cleavage a	0	39.72	27	10	17	10	471	52	8	24.66	10	cell organi:	membrane	protein bir	Pf00076, P	79869	ENSG0000	CPSF7	11	mRNA surv		Cleavage o	12	1.05	0.667660608	1.44	3095.4	2920.4	22.72	2.72	High	High	High	High	High	High	1	
FALSE	High	Master Prc	P27361-1	mitogen-ac	0	18.148	11	3	3	3	379	43.1	6.74	9.98	3	cell commu	cytoplasm;	catalytic ac	Pf00069, P	5595	ENSG0000	MAPK3	16	Fc epsilon	IL-5 Signali	ISG15 antiv	253	1.05	0.719786336	4.05	1073.2	1119.2	6.83	9.67	High	High	High	High	High	High	1	
FALSE	High	Master Prc	Q9BRK5-1	45 kDa calc	0	21.061	21	4	6	4	362	41.8	4.86	17.5	4	cell differe	cytoplasm;	metal ion l	Pf13499	51150	ENSG0000	SDF4	1				0	1.05	0.85024588	1.13	720.8	701.3	4.81	9.96	High	High	High	High	High	High	1	
FALSE	High	Master Prc	Q15435	Protein ph	0	21.006	14	3	3	3	360	41.5	4.91	11.52	3	regulation	chromosor	enzyme re	Pf12799, P	5510	ENSG0000	PPP1R7	2				0	1.049	0.213490678	0.89	204.5	192.2	5.77	3.86	High	High	High	High	High	High	1	
FALSE	High	Master Prc	Q02809	Procollage	0	10.361	7	5	5	5	727	83.5	6.95	10.82	5	metabolic	endoplasm	catalytic ac	Pf03171	5351	ENSG0000	PLOD1	1	Lysine deg		Collagen b	4	1.049	0.220226132	2.51	1251	1223.1	4.08	1.88	High	High	High	High	High	High	1	
FALSE	High	Master Prc	P55209	Nucleosom	0	75.951	31	10	18	8	391	45.3	4.46	53.88	10	cell organi:	membrane	protein bir	Pf00956	4673	ENSG0000	NAP1L1	12				0	1.049	0.226551351	0.51	2882	2824.2	3.24	1.85	High	High	High	High	High	High	1	
FALSE	High	Master Prc	Q15773	Myeloid le	0	4.866	5	1	2	1	248	28.1	6.9	5.4	1	defense re	cytoplasm;	protein bir	Pf10248	8079	ENSG0000	MLF2	12				0	1.049	0.327699465	5.19	744.4	709.6	12.99	3.96	High	High	High	High	High	High	1	
FALSE	High	Master Prc	Q96QG7	myotubula	0.002	3.843	3	1	1	1	549	63.4	6.39	3.36	1	metabolic	cytoplasm;	catalytic ac	Pf06602	66036	ENSG0000	MTMR9	8			Synthesis c	8	1.049	0.469289004	17.72	333.3	317.8	24.11	2.62	High	High	High	High	High	High	1	
FALSE	High	Master Prc	Q9NVX2	Notchless p	0	17.146	12	4	4	4	485	53.3	7.34	14.03	4	cell differe	nucleus;rib	protein bir	Pf00400, P	54475	ENSG0000	NLE1	17				0	1.049	0.484715266	23.84	257.5	261.2	41	6.69	High	High	High	High	High	High	1	
FALSE	High	Master Prc	Q14683	structural r	0	54.94	13	15	18	15	1233	143.1	7.64	28.7	15	cell divisio	chromosor	nucleotide	Pf02463, P	8243	ENSG0000	SMC1A	X	Oocyte me	miRNA Reg	Meiotic syr	30	1.049	0.563083782	24.29	4633.2	4418.6	14.31	13.4	High	High	High	High	High	High	1	
FALSE	High	Master Prc	Q15287-1	RNA-bindir	0	36.057	24	5	15	5	305	34.2	11.84	60.73	5	metabolic	cytoplasm;	protein bir	Pf00076, P	10921	ENSG0000	RNPS1	16	mRNA surv	mRNA Pro	mRNA 3'-e	20	1.049	0.77314708	3.78	2405.4	2421.1	5.58	11.69	High	High	High	High	High	High	1	
FALSE	High	Master Prc	P43307	Translocon	0	42.408	21	4	18	4	286	32.2	4.49	43.89	4	cell organi:	endoplasm	protein bir	Pf03896	6745	ENSG0000	SSR1	6	Protein prc		XBP1(S) ac	7	1.049	0.911310646	23.43	6474.7	6171.6	14.49	5.97	High	High	High	High	High	High	1	
FALSE	High	Master Prc	Q96Q06-1	Perilipin-4	0	8.807	2	3	4	3	1357	134.3	8.73	6.34	3		cytoplasm;		Pf03036	729359	ENSG0000	PLIN4	19	PPAR signa			1	1.049	0.97321531	20.1	455.9	459.2	15.42	7.86	High	High	High	High	High	High	1	
FALSE	High	Master Prc	O15118	Niemann-P	0	21.308	7	7	8	7	1278	142.1	5.36	12.75	7	cell organi:	endoplasm	protein bir	Pf00873, P	4864	ENSG0000	NPC1	18	Lysosome	Irinotecan	LDL cleara	6	1.049	0.977026583	21.94	332.6	338.8	12.42	14.43	High	High	High	High	High	High	1	
FALSE	High	Master Prc	Q5BKZ1	DBIRD com	0	25.856	13	8	12	8	582	65.6	5.15	16.96	8	metabolic	nucleus;sp	DNA bindi	Pf04988	284695	ENSG0000	ZNF326	1				0	1.049	0.991035756	0.71	1787.7	1781.5	5.61	4.25	High	High	High	High	High	High	1	
FALSE	High	Master Prc	Q9UMR5	Lysosomal	0	7.964	6	1	2	1	302	34.2	6.33	8.4	1	metabolic	organelle l	catalytic ac	Pf00561, P	9374	ENSG0000	PPT2	6	Metabolic		Fatty acyl-	8	1.048	0.086373588	0.18	741.7	707.9	6.75	5.04	High	High	High	High	High	High	1	
FALSE	High	Master Prc	Q15075	Early endo	0	34.779	6	7	9	7	1411	162.4	5.68	21.96	7	cell organi:	cytoplasm;	catalytic ac	Pf01363, P	8411	ENSG0000	EEA1	12	Phagosom	VEGFA-VEC	Toll Like Re	8	1.048	0.322555529	0.26	497.9	470.7	19.95	3.48	High	High	High	High	High	High	1	
FALSE	High	Master Prc	Q9H2W6	39S riboso	0	16.059	16	3	5	3	279	31.7	7.05	13.38	3	cell organi:	membrane	catalytic ac	Pf11788	26589	ENSG0000	MRPL46	15			Mitochonc	6	1.048	0.348827111	7.52	793.4	756.8	16.62	2.05	High	High	High	High	High	High	1	
FALSE	High	Master Prc	Q7Z4Q2	HEAT repe	0	9.463	4	2	2	2	680	74.5	5.11	3.38	2	transport		protein bir	Pf13513	55027	ENSG0000	HEATR3	16				0	1.048	0.560069763	65.36	78.8	80.1	83.43	19.25	High	High	High	High	High	High	1	
FALSE	High	Master Prc	Q6ZS17	RHO family	0	7.589	3	3	3	3	1223	132.2	6.28	6.34	3	cell commu	cytoplasm;	protein bir	Pf13646	79567	ENSG0000	FAM65A; F	16				0	1.048	0.620916351	4.61	779.4	747.9	1.54	7.76	High	High	High	High	High	High	1	
FALSE	High	Master Prc	Q9BQB6-1	vitamin K e	0	7.883	8	1	3	1	163	18.2	9.36	4.55	1	coagulatio	endoplasm	catalytic ac	Pf07884	79001	ENSG0000	VKORC1	16	Ubiquinon		Metabolisr	5	1.048	0.73561164	22.29	562.2	536.5	31.12	6.7	High	High	High	High	High	High	1	
FALSE	High	Master Prc	Q9UKF6	Cleavage a	0.003	2.908	1	1	1	1	684	77.4	5.6	1.67	1	metabolic	nucleus	catalytic ac	Pf00753, P	51692	ENSG0000	CPSF3	2	mRNA surv	mRNA Pro	mRNA 3'-e	16	1.048	0.738194619	0.74	741.3	715.6	33.32	21.07	High	High	High	High	High	High	1	
FALSE	High	Master Prc	Q9NWU5	39S riboso	0.005	2.585	4	1	1	1	206	23.6	9.94	0	1	cell organi:	membrane	RNA bindir	Pf00237	29093	ENSG0000	MRPL22	5	Ribosome		Mitochonc	7	1.048	0.742195283	22.08	542.3	548	23.03	7.77	High	High	High	High	High	High	1	
FALSE	High	Master Prc	P30876	DNA-direct	0	16.145	4	4	4	4	1174	133.8	6.87	6.66	4	metabolic	membrane	catalytic ac	Pf00562, P	5431	ENSG0000	POLR2B	4	RNA polym	Eukaryotic	Signaling b	75	1.048	0.744595886	44.15	271.9	269.9	30.71	13.84	High	High	High	High	High	High	1	
FALSE	High	Master Prc	Q8IW92	Beta-galac	0	6.178	3	1	1	1	636	72	7.62	4.5	1	metabolic	extracellul	catalytic ac	Pf01301, P	89944	ENSG0000	GLB1L2	11				0	1.048	0.749883805	31.48	143.4	142.2	25.31	17.61	High	High	High	High	High	High	1	
FALSE	High	Master Prc	P48681	Nestin [OS	0	67.707	13	18	25	18	1621	177.3	4.36	40.13	18	cell organi:	cytoplasm;	protein bir	Pf00038	10763	ENSG0000	NES	1				0	1.048	0.855670883	10.51	4570.6	4362	4.69	14	High	High	High	High	High	High	1	
FALSE	High	Master Prc	O14548	Cytochrom	0	16.27	27	3	5	3	114	12.6	9.42	11.27	3	metabolic	membrane	catalytic ac	Pf02238	9167	ENSG0000	COX7A2L	2	Oxidative p	Electron Tr	TP53 Regu	16	1.047	0.033229902	1.16	3230.8	3085.1	3.61	5.11	High	High	High	High	High	High	1	
FALSE	High	Master Prc	Q32P28-1	Prolyl 3-hy	0	42.083	14	8	10	8	736	83.3	5.14	23.24	8	metabolic	endoplasm	catalytic ac	Pf13640	64175	ENSG0000	LEPRE1; P3	1			Collagen b	3	1.047	0.092702175	2.67	5015.8	4669.3	1.83	3.71	High	High	High	High	High	High	1	
FALSE	High	Master Prc	P09497-2	Isoform Nc	0	29.791	29	8	17	8	211	23.2	4.69	22.27	8	cell organi:	cytosol;me	protein bir	Pf01086	1212	ENSG0000	CLTB	5				0	1.047	0.16272722	1.15	7091	6647.9	2.39	6.72	High	High	High	High	High	High	1	

FALSE	High	Master Prc	P41091	eukaryotic	0	57.561	31	11	18	11	472	51.1	8.4	41.86	11	metabolic	cytoplasm;	catalytic a	Pf00009, P	1968	ENSG0000	EIF2S3	X	RNA transp	Translation	Formation	15	1.047		0.190055178	4.86	4034.3	3854.2	2.85	1.1	High	High	High	High	High	High	1	
FALSE	High	Master Prc	Q13409-1	cytoplasmi	0	6.634	3	1	2	1	638	71.4	5.2	7.72	1	cell divisio	cytoplasm;	catalytic a	Pf00400, P	1781	ENSG0000	DYNC1I2	2	Phagosom		COPI-indep	48	1.047		0.449602957	15.19	870.2	766	13.29	11.25	High	High	High	High	High	High	1	
FALSE	High	Master Prc	O75554	WW doma	0	7.275	3	1	1	1	376	42.5	5.57	3.97	1	metabolic	nucleus;sp	metal ion l	Pf00397, P	11193	ENSG0000	WBP4	13			mRNA Spli	4	1.047		0.547689794	22.76	192.2	190	22.44	9.09	High	High	High	High	High	High	1	
FALSE	High	Master Prc	O75907	Diacylglyce	0.003	3.209	4	1	1	1	488	55.2	9.32	3.32	1	cell organi	endoplasm	catalytic a	Pf03062	8694	ENSG0000	DGAT1	8	Retinol me	Triacylglyc	Triglycerid	17	1.047		0.673739591	12.16	552.5	538.4	13.88	4.02	High	High	High	High	High	High	1	
FALSE	High	Master Prc	Q96LJ7	Dehydroge	0	13.994	12	2	2	2	313	33.9	7.83	9.06	2	metabolic	endoplasm	catalytic a	Pf00106, P	115817	ENSG0000	DHRS1	14				0	1.047		0.886088698	11.23	156.5	149.4	5.77	11.52	High	High	High	High	High	High	1	
FALSE	High	Master Prc	Q9Y3Y2	Chromatin	0	34.542	23	5	11	5	248	26.4	12.23	32.41	5	metabolic	nucleus	DNA bindi	Pf13865	26097	ENSG0000	CHTOP	1			Cleavage o	9	1.046		0.347563015	8.69	3927.3	3755.5	7.12	2.14	High	High	High	High	High	High	1	
FALSE	High	Master Prc	Q9Y520	Protein Prr	0	74.042	8	18	23	16	2896	316.7	9.13	53.97	18	cell differe	membrane	protein bir	Pf07001	23215		PRRC2C	1				0	1.046		0.446173178	13.29	3975.8	3896.8	15.38	2.45	High	High	High	High	High	High	1	
FALSE	High	Master Prc	O75475-1	PC4 and SF	0	31.257	15	7	12	6	530	60.1	9.13	27.43	7	cell organi	cytosol;nu	DNA bindi	Pf00855, P	11168	ENSG0000	PSIP1	9			2-LTR circl	13	1.046		0.462550154	13.8	1521.3	1546.2	15.33	4.03	High	High	High	High	High	High	1	
FALSE	Medium	Master Prc	P84101-1	Small EDRI	0.029	1.436	14	1	1	1	59	6.9	10.45	2.04	1		cytosol;nu			10169	ENSG0000	SERF2	15				0	1.046		0.516535073	34.97	457.8	437.7	52.13	8.66	High	High	High	High	High	High	1	
FALSE	High	Master Prc	P07910-2	Isoform C1	0	242.733	56	23	142	23	293	32.3	5.08	357.07	23	cell differe	cytoskelet	DNA bindi	Pf00076, P	3183	ENSG0000	HNRNPC	14		mRNA Pro		1	1.046		0.548994035	8.72	50974	48754.9	4.4	3.73	High	High	High	High	High	High	1	
FALSE	High	Master Prc	Q9GZR7	ATP-depen	0	24.012	10	6	8	6	859	96.3	9.06	18.79	6	metabolic	cytoplasm;	catalytic a	Pf00270, P	57062	ENSG0000	DDX24	14; CHR_H				0	1.046		0.568324222	28.39	1706.7	1766.5	32.28	11.83	High	High	High	High	High	High	1	
FALSE	High	Master Prc	O14776-1	Transcripti	0	21.125	8	7	8	7	1098	123.8	8.65	11.03	7	metabolic	nucleus	protein bir	Pf00397, P	10915	ENSG0000	TCERG1	5	Spliceosom			1	1.046		0.880567799	15.25	1809.7	1729.3	5.15	9.32	High	High	High	High	High	High	1	
FALSE	High	Master Prc	P61927	60S riboso	0	13.411	39	6	13	6	97	11.1	11.74	14.62	6	cell organi	cytosol;rib	metal ion l	Pf01907	6167	ENSG0000	RPL37	5	Ribosome	Cytoplasm	Peptide ch	34	1.045		0.286668719	5.17	6240.5	5971	3.34	6.99	High	High	High	High	High	High	1	
FALSE	Medium	Master Prc	Q13637	Ras-relate	0.024	1.511	6	1	1	1	225	25	6.54	0	1	cell organi	cytosol;en	catalytic a	Pf00025, P	10981	ENSG0000	RAB32	6			RAB GEFs e	7	1.045		0.357556825	3.36	295.7	282.8	22.22	5.03	High	High	High	High	High	High	1	
FALSE	High	Master Prc	Q13561-1	dynactin su	0	47.66	26	8	11	8	401	44.2	5.21	23.67	8	cell organi	chromosor	catalytic a	Pf04912	10540	ENSG0000	DCTN2	12	Huntingtor		COPI-indep	35	1.045		0.408308775	11.2	2642.7	2521.1	18.17	2.08	High	High	High	High	High	High	1	
FALSE	High	Master Prc	Q9BVK6	Transmem	0	32.415	17	4	12	4	235	27.3	8.02	28.81	4	cell organi	endoplasm	protein bir	Pf01105	54732	ENSG0000	TMED9	5			COPI-depe	11	1.045		0.472169029	9.16	1912.7	1853.3	5.03	3.18	High	High	High	High	High	High	1	
FALSE	High	Master Prc	Q9UK45	U6 snRNA-	0	8.728	33	2	5	2	103	11.6	5.27	4.82	2	metabolic	cytosol;nu	protein bir	Pf01423	51690	ENSG0000	LSM7	19	Spliceosom	mRNA Pro	mRNA Spli	9	1.045		0.489036395	14.97	1279	1178.3	10.21	5.7	High	High	High	High	High	High	1	
FALSE	High	Master Prc	Q9NU22	Midasin [C	0	8.878	1	3	3	3	5596	632.4	5.68	5.88	3	cell organi	cytoplasm;	catalytic a	Pf00004, P	23195	ENSG0000	MDN1	6	Ribosome			1	1.045		0.517325468	17.5	248.5	237.8	11.82	8.1	High	High	High	High	High	High	1	
FALSE	High	Master Prc	Q14247-1	Src substra	0	124.288	49	28	56	28	550	61.5	5.4	110.02	28	cell organi	cytoplasm;	protein bir	Pf00018, P	2017	ENSG0000	CTTN	11	Proteoglyc	Pathogeni	RHO GTPa:	14	1.045		0.643301951	14.27	8365.3	8007.4	5.68	5.6	High	High	High	High	High	High	1	[T401(98.
FALSE	High	Master Prc	P05787	Keratin, ty	0	432.298	76	44	273	37	483	53.7	5.59	593.32	44	cell death;	cytoplasm;	catalytic a	Pf00038, P	3856	ENSG0000	KRT8	12		Primary Fc	Formation	4	1.045		0.729177945	23.18	80708.4	77255.5	8.41	7.55	High	High	High	High	High	High	1	[R47]
FALSE	High	Master Prc	Q96AT9	ribulose-ph	0	11.922	12	2	2	2	228	24.9	5.58	9.64	2	metabolic	cytosol	catalytic a	Pf00218, P	6120	ENSG0000	RPE	2	Biosynthes	Pentose Pl	Pentose ph	9	1.045		0.739399193	22.78	224.4	214.8	11.2	5.49	High	High	High	High	High	High	1	
FALSE	High	Master Prc	P35237	serpin B6 [	0	66.584	42	13	25	13	376	42.6	5.27	64.55	13	regulation	cytoplasm;	enzyme re	Pf00079	5269	ENSG0000	SERPINB6	6	Amoebiasi	Ectoderm I	Dissolution	7	1.045		0.823267022	25.52	5296.6	5506.9	18.79	5.87	High	High	High	High	High	High	1	
FALSE	High	Master Prc	P63313	thymosin b	0	20.066	48	5	19	2	44	5	5.36	41.9	5	cell organi	cytoplasm;	protein bir		9168	ENSG0000	TMSB10	2				0	1.045		0.869460669	13.61	1983	1975.6	7.76	14.6	High	High	High	High	High	High	1	
FALSE	High	Master Prc	Q9Y3Z3	deoxynucle	0	10.246	6	3	3	3	626	72.2	7.14	9.3	3	cell organi	membrane	catalytic a	Pf00536, P	25939	ENSG0000	SAMHD1	20			Nucleobas	7	1.045		0.903851689	20.91	1091	1026.8	4.77	14.65	High	High	High	High	High	High	1	
FALSE	High	Master Prc	P12429	annexin A3	0	90.598	40	15	36	15	323	36.4	5.92	67.78	15	defense re	cytoplasm;	enzyme re	Pf00191	306	ENSG0000	ANXA3	4		Prostaglan		1	1.045		0.949248519	2.64	17796.2	17828.3	7.87	3.59	High	High	High	High	High	High	1	
FALSE	High	Master Prc	Q15424-1	Scaffold at	0	59.077	16	14	25	9	915	102.6	5.47	44.89	14	cell organi	nucleus	DNA bindi	Pf00076, P	6294	ENSG0000	SAFB	19		Structural		1	1.044		0.276324223	6.4	3701.8	3519.3	2.72	4.36	High	High	High	High	High	High	1	
FALSE	High	Master Prc	Q9GZS3	WD repeat	0	33.362	35	6	8	6	305	33.6	5.47	24.19	6	cell organi	cytoplasm;	protein bir	Pf00400, P	80349	ENSG0000	WDR61	15	RNA degra		mRNA dec	13	1.044		0.327491297	3.86	1410.3	1342.7	13.29	3.91	High	High	High	High	High	High	1	
FALSE	High	Master Prc	P51648	Fatty aldeh	0	30.659	21	8	13	8	485	54.8	7.88	25.37	8	metabolic	endoplasm	catalytic a	Pf00171	224	ENSG0000	ALDH3A2	17	Glycerolipi	Tryptopha	Sphingolip	18	1.044		0.351711247	4.65	1419	1358.7	4.42	0.32	High	High	High	High	High	High	1	
FALSE	High	Master Prc	Q02388-1	Collagen al	0	8.153	2	4	4	4	2944	295	6.27	0	4	cell differe	extracellul	enzyme re	Pf00014, P	1294	ENSG0000	COL7A1	3	Protein dig		Collagen d	20	1.044		0.414474846	10.74	70	66.9	16.62	9.47	High	High	High	High	High	High	1	[K1786];
FALSE	High	Master Prc	Q14566	DNA replic	0	70.992	22	16	24	16	821	92.8	5.41	51.08	16	metabolic	nucleus	catalytic a	Pf00493, P	4175	ENSG0000	MCM6	2	Cell cycle;	Cell Cycle;	M/G1 Trar	26	1.044		0.459188983	13.44	9186.6	8803.2	9.7	6.2	High	High	High	High	High	High	1	
FALSE	High	Master Prc	Q9NZD8	Maspardin	0.004	2.768	3	1	1	1	308	34.9	6.28	1.93	1	metabolic	cytoplasm;	catalytic a	Pf00561, P	51324	ENSG0000	SPG21	15	Endocytosi			1	1.044		0.48821899	15.43	571.3	559.2	15.87	4.79	High	High	High	High	High	High	1	
FALSE	High	Master Prc	O75143-1	Autophagy	0	7.758	6	2	3	2	517	56.5	5.12	6.55	2	cell commu	cytoplasm;	protein bir	Pf10033	9776	ENSG0000	ATG13	11	Autophagy	PI3K-AKT-r	Macroautc	6	1.044		0.524449627	39.56	539.2	516.2	57.72	18.2	High	High	High	High	High	High	1	
FALSE	High	Master Prc	Q12904-1	aminoacyl	0	41.571	30	7	12	7	312	34.3	8.43	35.39	7	cell commu	cell surface	protein bir	Pf01588	9255	ENSG0000	AIMP1	4			Cytosolic t	8	1.044		0.672080423	36.37	5778.9	6230.7	33.01	8.24	High	High	High	High	High	High	1	
FALSE	High	Master Prc	Q9Y3E5	Peptidyl-tF	0	14.138	16	2	2	2	179	19.2	8.73	9.57	2	cell death;	cytosol;me	catalytic a	Pf01981	51651	ENSG0000	PTRH2	17			Ub-specific	4	1.044		0.71238508	10.51	630.5	674	15.96	9.2	High	High	High	High	High	High	1	
FALSE	High	Master Prc	O43390-1	heterogen	0	175.629	43	27	90	22	633	70.9	8.13	204.65	27	metabolic	cytoplasm;	protein bir	Pf00076, P	10236	ENSG0000	HNRNPR	1; CHR_HS		mRNA Pro	mRNA Spli	5	1.044		0.915283071	5.33	19230.5	18970.8	5.59	5.13	High	High	High	High	High	High	1	
FALSE	High	Master Prc	Q9NVM6	DnaJ homo	0.003	3.13	7	2	2	2	304	34.7	8.53	4.09	2	metabolic	cytoplasm;	RNA bindi	Pf00076, P	55192	ENSG0000	DNAJC17	15				0	1.043		0.523635701	23.42	487.9	468	26.34	7.36	High	High	High	High	High	High	1	
FALSE	High	Master Prc	P51151	Ras-relate	0	43.55	50	6	10	6	201	22.8	5.47	33.95	6	regulation	cytosol;en	catalytic a	Pf00025, P	9367	ENSG0000	RAB9A	X	Measles		Retrograde	10	1.043		0.532624103	15.26	2289	2107.2	9.86	4.29	High	High	High	High	High	High	1	
FALSE	High	Master Prc	O14562	Ubiquitin d	0	21.881	20	4	8	4	309	33.4	5.77	28.52	4			protein bir	Pf00240, P	56061	ENSG0000	UBFD1	16				0	1.043		0.662235749	3.91	746.2	777	18.84	6.72	High	High	High	High	High	High	1	
FALSE	High	Master Prc	Q9H4G0-2	Isoform 2 c	0	31.769	11	7	13	7	779	87.6	5.92	25.43	7	cell organi	cytoskelet	protein bir	Pf00373, P	2036	ENSG0000	EPB41L1	20				0	1.043		0.795063513	9.81	4813.2	4788.7	7.48	4.97	High	High	High	High	High	High	1	
FALSE	High	Master Prc	Q9H7Z7	Prostaglan	0	50.698	33	10	18	10	377	41.9	9.16	45.94	10	cellular ho	cytoplasm;	catalytic a	Pf00043, P	80142	ENSG0000	PTGES2	9	Arachidoni	Eicosanoid	Synthesis c	11	1.042		0.012758795	1.06	4100.5	3899.2	4.75	5.02	High	High	High	High	High	High	1	
FALSE	High	Master Prc	Q5SWX8-1	Protein od	0	5.199	3	1	1	1	454	51.1	5.92	4.07	1	metabolic	membrane	catalytic a	Pf00848, P	54953	ENSG0000	C1orf27; C	1				0	1.042		0.223828396	1.35	470.8	435.3	5.15	11.5	High	High	High	High	High	High	1	
FALSE	High	Master Prc	Q13573	SNW doma	0	46.369	26	12	16	12	536	61.5	9.52	38.79	12	cell organi	nucleus;sp	catalytic a	Pf02731	22938	ENSG0000	SNW1	14	Viral carcir	TGF-beta S	NOTCH1 Ir	34	1.042		0.227652952	2.75	3791.2	3694.6	2.13	1.95	High	High	High	High	High	High	1	
FALSE	High	Master Prc	Q14376	UDP-gluco	0	8.115	9	2	2	2	348	38.3	6.73	4.83	2	metabolic	cytosol	catalytic a	Pf00106, P	2582	ENSG0000	GALE	1	Amino sug		Defective (	10	1.042		0.314544647	0.72	650.5	614.9	17.09	1.34	High	High	High	High	High	High	1	
FALSE	High	Master Prc	P55145	Mesencepl	0	20.157	20	3	4	3	182	20.7	8.69	13.24	3	cell differe	cytosol;en	protein bir	Pf10208	7873		MANF	3			Platelet de	4	1.042		0.343759273	1.52	1262.9	1200.2	6.52	3.41	High	High	High	High	High	High	1	
FALSE	High	Master Prc	Q9NUP9	Protein lin-	0	10.985	15	2	4	2	197	21.8	8.43	10.59	2	cell organi	cytoplasm;	protein bir	Pf00595, P	55327	ENSG0000	LIN7C	11			Dopamine	6	1.042		0.449918393	12.81	1107.8	1058.5	12.21	8.47	High	High	High	High	High	High	1	
FALSE	High	Master Prc	Q9UBW8	COP9 signa	0	11.127	15	3	3	3	275	30.3	8.22	7.65	3	cell organi	cytoplasm;	protein bir	Pf01399	50813	ENSG0000	COPS7A	12			Cargo reco	13	1.042		0.481616119	27.27	989.5	988.8	56.62	11.53	High	High	High	High	High	High	1	
FALSE	High	Master Prc	O00148	ATP-depen	0	88.335	40	14	39	5	427	49.1	5.68	97.82	14	metabolic	cytoplasm;	catalytic a	Pf00270, P	10212	ENSG0000	DDX39A	19			Cleavage o	9	1.042		0.51546978	17.11	2431.4	2364.6	18.17	0.83	High	High	High	High	High	High	1	
FALSE	High	Master Prc	Q96QR8	Transcripti	0	23.794	22	4	6	4	312	33.2	5.43	9.33	4	cell differe	nucleus	DNA bindi	Pf04845	5814	ENSG0000	PURB	7				0	1.042		0.642014227	47.49	624.3	598.9	43.16	14.23	High	High	High	High	High	High	1	
FALSE	High	Master Prc	Q9BQ70	Transcripti	0	9.064	4	2	2	2	676	76.6	6.35	5.73	2	metabolic	nucleus	DNA bindi	Pf04910	22980	ENSG0000	TCF25	16				0	1.042		0.917919529	3.24	189	186.1	4.97	3.91	High	High	High	High	High	High	1	
FALSE	High	Master Prc	P11142-1	Heat shock	0	542.644	76	48	342	31	646	70.9	5.52	894.86	48	cell commu	cytoplasm;	catalytic a	Pf00012, P	3312	ENSG0000	HSPA8	11	Protein pro	Parkin-Ubi	Protein me	52	1.042		0.986859372	2.13	81825.9	79155.6	7.82	1.35	High	High	High	High	High	High	1	[K557;
FALSE	High	Master Prc	P52788-1	Spermine s	0	71.086	41	11	23	11	366	41.2	5.02	55.09	11	metabolic	cytosol	catalytic a	Pf01564	6611	ENSG0000	SMS	X	Arginine ar	Amino Aci	Metabolisr	10	1.041		0.200923386	0.57	7437.5	7207.1	2.71	1.23	High	High	High	High	High	High	1	
FALSE	High	Master Prc	P11234	Ras-relate	0	13.485	20	6	8	3	206	23.4	6.62	9.33	6	cell commu	membrane	catalytic a	Pf00025, P	5899	ENSG0000	RALB	2	Ras signali	EGF/EGFR	p38MAPK	13	1.041		0.207116613	4.73	1781.6	1768.6	6.48	3.81	High	High	High	High	High	High	1	
FALSE	High	Master Prc	Q9NQP4	Prefoldin s	0	26.651	20	2	6	2	134	15.3	4.53	24.3	2	metabolic	cytoplasm;	protein bir	Pf01920	5203	ENSG0000	PFDN4	20			Prefoldin r	5	1.041		0.273167444	5.46	1210.4	1131.2	5.35	2.75	High	High	High	High	High	High	1	
FALSE	High	Master Prc	Q9Y6N5	Sulfide:qui	0	26.061	20	6	8	6	450	49.9	9.11	7.59	6	metabolic	membrane	catalytic a	Pf07992, P	58472	ENSG0000	SQRDL; SQ	15	Sulfur met		Sulfide oxi	6	1.041		0.323536466	0.48	121.3	107.2	17.43	5.31	High	High	High	High	High	High	1	
FALSE	Medium	Master Prc	Q9NX76	CKLF-like M	0.018	1.643	5	1	1	1	183	20.4	5.29	1.64	1	regulation	membrane	protein bir	Pf01284	54918	ENSG0000	CMTM6	3			Neutrophil	3	1.041		0.361795091	3.6	409.8	409.3	7.01	4.23	High	High	High	High	High	High	1	
FALSE	High	Master Prc	Q9P2E9-1	Ribosome-	0	322.102	57	60	133	60	1410	152.4	8.6	294.95	60	cell differe	endoplasm	receptor a	Pf03276, P	6238		RRBP1	20	Protein pro	Ectoderm I		2	1.041		0.403171001	9.28	28756	28751.7	7.78	2.89	High	High	High	High	High	High	1	
FALSE	High	Master Prc	P51511	Matrix me	0.003	2.999	2	1	1	1	669	75.8	7.46	1.96	1	cell differe	membrane	catalytic a	Pf00045, P	4324	ENSG0000	MMP15	16		Matrix Me	Collagen d	5	1.041		0.434608984	11.04	292.8	281.2	9.13	2.09	High	High	High	High	High	High	1	
FALSE	High	Master Prc	Q71UI9-1	Histone H2	0	30.139	26	3	16	2	128	13.5	10.58	31.03	3	regulation	chromosor	DNA bindi	Pf00125	94239	ENSG0000	H2AFV	7	Alcoholism		SIRT1 nega	57	1.041		0.478208737	16.11	5748.2	5523.9	16.75	7.95	High	High	High	High	High	High	1	
FALSE	High	Master Prc	Q9GZZ9-1	Ubiquitin-l	0	13.417	13	3	3	3	404	44.8	4.84	8.87	3	metabolic	cytoplasm;	catalytic a	Pf00899	79876	ENSG0000	UBA5	3			Antigen pr	4	1.041		0.597465671	7.87	1143.5	1098.4	5.72	12.48	High	High	High	High	High	High	1	
FALSE	High	Master Prc	Q9BZ67	FERM dom	0	7.448	4	1	1	1	464	51.2	6.23	3.86	1		cytoskelet	protein bir	Pf00373	83786	ENSG0000	FRMD8	11				0	1.041		0.615918744	35.41	425.2	408.4	33.23	6.02	High	High	High	High	High	High	1	
FALSE	High	Master Prc	P41229	Lysine-spe	0	5.062	1	1	1	1	1560	175.6	5.58	3.07	1	cell organi	cytosol;nu	catalytic a	Pf00628, P	8242	ENSG0000	KDM5C	X			HDMs den	3	1.041		0.622299429	8.73	210.4	231.7	13.27	17.93	High	High	High	High	High	High	1	
FALSE	High	Master Prc	O00461	Golgi integ	0	50.499	15	9	14	9	696	81.8	4.77	40	9	transport	endosome			27333	ENSG0000	GOLIM4	3			Intra-Golgi	4	1.041		0.745350193	23.92	3357.4	3144.9	15.35	2.55	High	High	High	High	High	High	1	
FALSE	High	Master Prc	P12236	ADP/ATP t	0	125.428	58	18	58	9	298	32.8	9.74	108.82	18	cell death;	membrane	protein bir	Pf00153	293	ENSG0000	SLC25A6	X|Y; X	cGMP-PKG	Electron Tr	Mitochonc	21	1.041		0.825232687	28.83	5358.6	4995.2	33.37	4.43	High	High	High	High	High	High	1	
FALSE	High	Master Prc	Q2TAA5	GDP-Man:	0.003	2.943	2	1	1	1	492	55.6	8.48	3.04	1	metabolic	endoplasm	catalytic a	Pf00534, P	440138	ENSG0000	ALG11	13	Metabolic		Biosynthes	10	1.041		0.835382601	8.51	442.6	432.5	7.43	9.06	High	High	High	High	High	High	1	
FALSE	High	Master Prc	Q92665	28S riboso	0	18.469	13	3	3	3	395	45.3	9.29	7.83	3	cell organi	membrane	protein bir	Pf15433	10240	ENSG0000	MRPS31	13			Mitochonc	6	1.041		0.931232044	59.57	63.6	60.3	30.83	13.8	High	High	High	High	High	High	1	
FALSE	High	Master Prc	Q9NRV9	Heme-binc	0	27.056	37	6	11	6	189	21.1	5.8	21.98	6	regulation	cytoplasm;		Pf04832	50865	ENSG0000	HEBP1	12			Formyl pep	8	1.041		0.99835466	2.61	937.9	912.2	7.6	1.84	High	High	High	High	High	High	1	
FALSE	High	Master Prc	Q99829	Copine-1 [(	0	54.895	27	12	22	12	537	59	5.83	34.19	12	cell differe	cytoplasm;	catalytic a	Pf00092, P	8904	ENSG0000	CPNE1	20			Neutrophil	7	1.04		0.255064629	3.9	7713.3	7470.1	3.74	0.56	High	High	High	High	High	High	1	
FALSE	High	Master Prc	O43707	Alpha-actir	0	428.235	66	50	197	37	911	104.8	5.44	555.09	50	cell organi	cytoplasm;	DNA bindi	Pf00307, P	81	ENSG0000	ACTN4	19; CHR_H	Amoebiasi	Arrhythmc	Platelet de	18	1.04		0.262937198	4.37	41836.6	40215.9	1.52	2.5	High	High	High	High	High	High	1	
FALSE	High	Master Prc	Q8IWZ3	Ankyrin re	0	18.678	2	4	4	1	2542	269.3	5.73	9.11	4	defense re	cytoplasm	protein bir	Pf00013, P	54882; 40	ENSG0000	ANKHD1; A	5				0	1.04		0.265108667	6.19	384.7	354.4	2.46	5.36	High	High	High	High	High	High	1	
FALSE	High	Master Prc	Q14573	Inositol 1,4	0	41.581	5	11	12	11	2671	303.9	6.48	23.3	11	cell organi	cytoplasm;	metal ion l	Pf00520, P	3710	ENSG0000	ITPR3	6	Taste trans	Alzheimers	Ion homeo	79	1.04		0.283122854	4.36	1301.7	1277.7	3.23	1.95	High	High	High	High	High	High	1	
FALSE	High	Master Prc	Q9Y2D5	A-kinase a	0.001	4.46	1	1	1	1	859	94.6	5.11	2.5	1					11217; 44	ENSG0000	AKAP2; PA	9		G Protein S		1	1.04		0.38547506	7.3	521.2	501.1	16.34	5.26	High	High	High	High	High	High	1	
FALSE	High	Master Prc	O95365	Zinc finger	0	5.1	4	1	1	1	584	61.4	5.19	3.18	1	cell differe	nucleus	DNA bindi	Pf00096, P	51341	ENSG0000	ZBTB7A	19				0	1.04		0.517242993	62.07	244.7	248.5	98.18	27.62	High	High	High	High	High	High	1	
FALSE	High	Master Prc	Q86X76	Deaminate	0	18.39	16	5	6	5	327	35.9	7.74	15.42	5	metabolic	cytoplasm;	catalytic a	Pf00795	4817	ENSG0000	NIT1	1				0	1.04		0.525612702	20.8	3185.5	3285.1	26.64	7.15	High	High	High	High	High	High	1	
FALSE	High	Master Prc	Q15691	Microtubu	0	43.407	32	7	14	7	268	30	5.14	42.73	7	cell divisio	cytoplasm;	protein bir	Pf00307, P	22919	ENSG0000	MAPRE1	20		Regulation	Regulation	30	1.04		0.538822736	23.83	4792.5	4609.9	25.86	9.71	High	High	High	High	High	High	1	
FALSE	High	Master Prc	Q9Y230	RuvB-like 2	0	59.415	29	12	22	12	463	51.1	5.64	46.75	12	cell organi	cytoplasm;	catalytic a	Pf00004, P	10856	ENSG0000	RUVBL2	19			HATs acety	8	1.04		0.540595068	15.72	7748.4	7451.8	9.98	5.85	High	High	High	High	High	High	1	
FALSE	High	Master Prc	O43709-1	Probable 1	0	17.213	17	4	6	4	281	31.9	8.73	13.09	4	cell organi	cytoplasm;	catalytic a	Pf01209, P	114049	ENSG0000	WBSCR22;	7			Major path	5	1.04		0.56973829	26.41	726	698.3	25.65	8.86	High	High	High	High	High	High	1	
FALSE	High	Master Prc	Q05048	Cleavage s	0	33.351	19	5	5	5	431	48.3	6.58	20.69	5	metabolic	nucleus	protein bir	Pf00400, P	1477	ENSG0000	CSTF1	20	mRNA surv	mRNA Pro	Cleavage o	13	1.04		0.6573764	39.14	1138.9	1036.4	32.92	4.98	High	High	High	High	High	High	1	
FALSE	High	Master Prc	Q9NSV4-3	Protein dia	0	10.93	5	4	4	4	1193	136.8	7.03	7.41	4	cell organi	cytoplasm;	protein bir	Pf02181, P	81624	ENSG0000	DIAPH3	13	Regulation	Regulation	RHO GTPa:	6	1.04		0.658540884	32.82	254.2	255.7	29.02	3.9	High	High	High	High	High	High	1	[K952];
FALSE	High	Master Prc	O75340	programm	0	30.083	32	6	17	6	191	21.9	5.4	48.02	6	cell death;	cytoplasm;	catalytic a	Pf00036, P	10016	ENSG0000	PDCD6	5				0	1.04		0.68139713	2.14	5124.6	4927.1	14.75	1.57	High	High	High	High	High	High	1	

FALSE	High	Master Prc	P61019-1	Ras-relatec	0	43.263	33	5	13	5	212	23.5	6.54	37.27	5	cell organi	cytosol;en	catalytic a	Pf00009, P	5862	ENSG0000	RAB2A	8	AMPK sign	Focal Adhe	Golgi Ciste	10	1.04		0.886546264	27.92	2865.2	2755.9	23.11	6.35	High	High	High	High	High	High	1	
FALSE	High	Master Prc	Q99614	tetratricop	0	40.101	29	8	18	8	292	33.5	4.84	34.17	8	metabolic	cytosol;me	protein bir	Pf00515, P	7265	ENSG0000	TTC1	5				0	1.04		0.903384056	10.51	3200.9	3239.1	13.98	4.28	High	High	High	High	High	High	1	
FALSE	High	Master Prc	Q03169	Tumor nec	0	11.429	6	4	4	4	654	72.6	6.46	4.87	4	cell differe		protein bir	Pf06046	7127	ENSG0000	TNFAIP2	14				0	1.04		0.971867221	4.05	1580.5	1580.5	5.49	5.5	High	High	High	High	High	High	1	
FALSE	High	Master Prc	P20340-2	Isoform 2 c	0	35.596	42	8	13	8	208	23.5	5.54	27.01	8	cellular coi	cytosol;Go	catalytic a	Pf00025, P	5870	ENSG0000	RAB6A	11				0	1.039		0.231408775	2.87	3417.1	3401	8.88	8.5	High	High	High	High	High	High	1	
FALSE	High	Master Prc	O95249-1	Golgi SNAF	0.003	2.942	4	1	1	1	250	28.6	9.42	1.69	1	cell organi	cytosol;Go	protein bir	Pf12352	9527	ENSG0000	GOSR1	17	SNARE inte		COPI-medi	11	1.039		0.363649587	8.41	761.8	733.5	13.05	6.83	High	High	High	High	High	High	1	
FALSE	High	Master Prc	Q9UKA9-1	Polypyrimi	0	9.908	10	3	3	3	531	57.5	8.66	6.98	3	cell organi	nucleus;sp	RNA bindi	Pf00076, P	58155	ENSG0000	PTBP2	1		TarBasePa		2	1.039		0.451029955	25.18	895.5	839.2	79.62	13.36	High	High	High	High	High	High	1	
FALSE	High	Master Prc	Q9BS40	Latexin [OS	0	12.261	17	3	4	3	222	25.7	5.78	9.57	3	defense re	cytoplasm	enzyme re	Pf06907	56925	ENSG0000	LXN	3				0	1.039		0.472559254	21.94	946.4	874.9	46.65	12.61	High	High	High	High	High	High	1	
FALSE	High	Master Prc	Q13347	Eukaryotic	0	87.974	56	12	24	12	325	36.5	5.64	67.96	12	cell organi	cytoplasm;	protein bir	Pf00400	8668	ENSG0000	EIF3I	1	RNA transp	Translatior	Formation	13	1.039		0.81594975	5.23	4752.8	4459.7	8.73	2.88	High	High	High	High	High	High	1	
FALSE	High	Master Prc	O15357	Phosphatic	0	16.292	3	3	4	3	1258	138.5	6.54	5.64	3	cell organi	cytoplasm;	catalytic a	Pf00017, P	3636	ENSG0000	INPPL1	11	Inositol ph	EGF/EGFR	Synthesis c	20	1.039		0.859448883	24.49	1185.2	1212.5	16.02	6.57	High	High	High	High	High	High	1	
FALSE	High	Master Prc	P36871-1	Phosphogl	0	42.02	26	11	15	11	562	61.4	6.76	27.28	11	metabolic	cytoplasm;	catalytic a	Pf00408, P	5236	ENSG0000	PGM1	1	Pentose ph	Ectoderm	Neutrophil	23	1.038		0.014204919	0.03	2839.1	2736.1	2.02	2.63	High	High	High	High	High	High	1	
FALSE	High	Master Prc	P14550	alcohol del	0	16.353	19	5	7	5	325	36.6	6.79	11.19	5	metabolic	cytosol;me	catalytic a	Pf00248	10327	ENSG0000	AKR1A1	1	Pentose ar	Benzo(a)py	Catabolism	12	1.038		0.412093023	8.98	545.9	523.2	22.31	6.6	High	High	High	High	High	High	1	
FALSE	Medium	Master Prc	P29373	Cellular ret	0.02	1.596	5	1	1	1	138	15.7	5.4	1.74	1	metabolic	cytoplasm;	protein bir	Pf00061, P	1382	ENSG0000	CRABP2	1		Vitamin A	Signaling b	4	1.038		0.455260215	11.98	517.3	400.9	16.03	21.18	High	High	High	High	High	High	1	
FALSE	High	Master Prc	P53365	Arfaptin-2	0	24.516	20	5	6	5	341	37.8	6.04	14.74	5	cell organi	cytoplasm;	nucleotide	Pf03114, P	23647	ENSG0000	ARFIP2	11			Retrograde	4	1.038		0.460117828	14.3	582.9	561.7	22.08	5.06	High	High	High	High	High	High	1	
FALSE	High	Master Prc	Q8WV41	Sorting nex	0	5.796	3	1	1	1	574	65.2	6.79	2.71	1	cell divisio	cytoplasm;	protein bir	Pf00018, P	257364	ENSG0000	SNX33	15				0	1.038		0.549935025	3.32	374.4	409.9	25.6	9.73	High	High	High	High	High	High	1	
FALSE	High	Master Prc	Q7KZN9	Cytochrom	0	12.753	10	3	4	3	410	46	9.82	13.47	3	cell organi	membrane	catalytic a	Pf02628	1355	ENSG0000	COX15	10	Oxidative	Electron Tr	Heme bios	7	1.038		0.554007518	20.54	608.8	673.2	25.81	8.25	High	High	High	High	High	High	1	
FALSE	High	Master Prc	Q9BQ48	39S riboso	0.001	4.472	11	1	2	1	92	10.2	12.25	5.05	1	cell organi	membrane	structural	Pf00468	64981	ENSG0000	MRPL34	19	Ribosome		Mitochonc	7	1.038		0.556516163	26.25	1096.4	1205.7	35.84	8.18	High	High	High	High	High	High	1	
FALSE	High	Master Prc	P15531	Nucleoside	0	99.08	70	11	53	1	152	17.1	6.19	135.17	11	cell differe	cytoplasm;	catalytic a	Pf00334	4830	ENSG0000	NME1	17	Metabolic		Interconve	6	1.038		0.676222554	11.91	698.5	682.7	3.11	20.8	High	High	High	High	High	High	1	
FALSE	High	Master Prc	Q13185	chromobox	0	72.03	44	9	34	8	183	20.8	5.33	84.4	9	cell organi	chromosor	protein bir	Pf00385, P	11335; 90	ENSG0000	CBX3; C15	7; 15		Exercise-in	RNA Polym	10	1.038		0.715285546	0.35	10635.5	10378.6	1.24	10.96	High	High	High	High	High	High	1	
FALSE	High	Master Prc	P49005	DNA polyn	0	7.598	6	2	2	2	469	51.3	5.58	2.77	2	cell organi	nucleus	catalytic a	Pf04042	5425	ENSG0000	POLD2	7	HTLV-I infe	Homologo	Mismatch	52	1.038		0.804450981	32.67	484	474.9	14.91	19.58	High	High	High	High	High	High	1	
FALSE	High	Master Prc	Q9H4M9	EH domain	0	39.553	14	7	15	6	534	60.6	6.83	47.6	7	cell organi	endosome	metal ion l	Pf00350, P	10938	ENSG0000	EHD1	11	Endocytosi	Insulin Sigr	Factors inv	4	1.038		0.874619488	1.78	1328.1	1290.6	2.63	3.82	High	High	High	High	High	High	1	
FALSE	High	Master Prc	O00161	Synaptosor	0	17.44	25	3	3	3	211	23.3	5.01	10.79	3	cell organi	cytoplasm;	catalytic a	Pf00835, P	8773	ENSG0000	SNAP23	15	Platelet ac	Insulin Sigr	Translocat	15	1.038		0.880645548	4.76	494.7	476.5	10.37	1.27	High	High	High	High	High	High	1	
FALSE	High	Master Prc	O15020	Spectrin be	0	27.933	4	7	7	4	2390	271.2	6.11	20.06	7	cell organi	cytoplasm;	protein bir	Pf00169, P	6712	ENSG0000	SPTBN2	11			Interactior	23	1.038		0.898353228	11.26	509.6	514.8	10.09	8.33	High	High	High	High	High	High	1	
FALSE	High	Master Prc	Q9P035	Very-long-	0	6.99	8	2	3	2	362	43.1	8.94	9.08	2	metabolic	cytosol;en	catalytic a	Pf04387, P	51495	ENSG0000	PTPLAD1;	15	Biosynthes		Synthesis c	8	1.037		0.052006309	1.76	1465.2	1399.2	2.14	3.49	High	High	High	High	High	High	1	
FALSE	High	Master Prc	Q12913-1	Receptor-t	0	5.046	2	1	1	1	1337	145.9	5.58	2.84	1	metabolic	cell surface	catalytic a	Pf00041, P	5795	ENSG0000	PTPRJ	11	Adherens j	VEGFA-VEC	Negative r	12	1.037		0.098305788	2.13	233.6	220.2	11.1	11.83	High	High	High	High	High	High	1	
FALSE	High	Master Prc	Q13217	DnaJ homc	0	24.594	14	5	9	5	504	57.5	6.15	23.14	5	defense re	cytoplasm;	enzyme re	Pf00226, P	5611	ENSG0000	DNAJC3	13	Influenza A	Photodyna	Post-transl	19	1.037		0.366669779	0.84	1356.6	1307.9	31.02	2.22	High	High	High	High	High	High	1	
FALSE	High	Master Prc	O60220	Mitochonc	0	10.552	23	2	4	2	97	11	5.16	3.67	2	transport	membrane	metal ion l	Pf02953	1678	ENSG0000	TIMM8A	X			Mitochonc	2	1.037		0.46244269	10.31	875.9	853.3	4.56	5.88	High	High	High	High	High	High	1	
FALSE	High	Master Prc	Q01469	Fatty acid-	0	44.55	63	8	12	8	135	15.2	7.01	35.19	8	metabolic	cytoplasm;	protein bir	Pf00061	2171	ENSG0000	FABP5	8	PPAR signa	PPAR signa	Triglycerid	12	1.037		0.528346206	20.07	3900.1	3790.7	24.23	3.1	High	High	High	High	High	High	1	
FALSE	High	Master Prc	O75122-3	Isoform 3 c	0	7.223	2	2	2	2	1515	165.8	8.37	4.32	2	cell divisio	chromosor	protein bir	Pf02985, P	23122		CLASP2	3				0	1.037		0.537893635	31.79	246.5	237.7	38.74	16.67	High	High	High	High	High	High	1	[Q112]
FALSE	High	Master Prc	P06703	protein S10	0	21.077	43	6	17	6	90	10.2	5.48	35.83	6	cell organi	cytoplasm;	metal ion l	Pf01023	6277	ENSG0000	S100A6	1		Gastric car		2	1.037		0.617617992	0.56	12498.5	12243.5	5.37	10.45	High	High	High	High	High	High	1	
FALSE	High	Master Prc	P23193	Transcripti	0	14.237	13	4	5	4	301	33.9	8.38	10.52	4	metabolic	nucleus	DNA bindi	Pf01096, P	6917	ENSG0000	TCEA1	8			HIV elonga	28	1.037		0.760072796	31.33	2710.8	2642.1	20.73	4.37	High	High	High	High	High	High	1	
FALSE	High	Master Prc	Q71RC2	La-related	0	35.704	15	7	13	7	724	80.5	6.61	40.79	7	cell organi	cytoplasm;	protein bir	Pf05383, P	113251	ENSG0000	LARP4	12				0	1.037		0.824641406	0.69	2193.1	2127.1	6.14	1.54	High	High	High	High	High	High	1	
FALSE	High	Master Prc	Q7Z4W1	L-xylulose	0	10.538	13	3	3	3	244	25.9	8.1	6.34	3	cell organi	membrane	catalytic a	Pf00106, P	51181	ENSG0000	DCXR	17	Metabolic		Catabolism	9	1.037		0.848068425	15.21	277.4	259.2	19.22	4.51	High	High	High	High	High	High	1	
FALSE	High	Master Prc	P55072	Transitiona	0	254.008	56	36	98	36	806	89.3	5.26	278.43	36	cell commu	cytoplasm;	catalytic a	Pf00004, P	7415	ENSG0000	VCP	9	Protein prc		Protein me	35	1.037		0.886016677	1.83	31568.2	31167.1	3.15	3.97	High	High	High	High	High	High	1	[T549(100
FALSE	High	Master Prc	P41134	DNA-bindi	0	5.575	14	2	2	2	155	16.1	6.99	2.62	2	cell differe	cytoplasm;	protein bir	Pf00010	3397	ENSG0000	ID1	20	TGF-beta s	Neural Cre	Oncogene	13	1.037		0.911649013	62.55	1430.5	1379.3	34.45	14.84	High	High	High	High	High	High	1	
FALSE	High	Master Prc	Q5VZM2	Ras-relatec	0	6.909	5	2	2	2	374	43.2	6.21	5.03	2	cell commu	cytoplasm;	catalytic a	Pf00025, P	10325	ENSG0000	RRAGB	X	mTOR sign	Target Of I	Energy dep	17	1.036		0.090892862	0.8	1663.1	1613.6	9.44	12.83	High	High	High	High	High	High	1	
FALSE	High	Master Prc	O15212	Prefoldin s	0	24.459	30	5	9	5	129	14.6	8.88	20.49	5	cell organi	cytoplasm	catalytic a	Pf01920, P	10471	ENSG0000	PFDN6	6; CHR_HS			Prefoldin r	5	1.036		0.235666927	4.33	2769.1	2658.9	2.9	2.47	High	High	High	High	High	High	1	
FALSE	High	Master Prc	Q9NTJ5	Phosphatic	0	15.88	7	4	5	4	587	66.9	7.12	11.1	4	metabolic	endoplasm	catalytic a	Pf02383	22908	ENSG0000	SACM1L	3			Synthesis c	6	1.036		0.291934727	2.14	913.9	847.6	10.79	3.03	High	High	High	High	High	High	1	
FALSE	High	Master Prc	Q6PJ69	Tripartite r	0	5.459	4	1	1	1	517	57.3	6.7	3.99	1	metabolic	cytosol;nu	metal ion l	Pf00097, P	201292	ENSG0000	TRIM65	17				0	1.036		0.349281718	1.76	193.8	176	16.21	7.56	High	High	High	High	High	High	1	
FALSE	High	Master Prc	Q15814	Tubulin-sp	0.003	3.301	4	1	1	1	346	39.2	5.71	4.04	1	cell organi	cytoplasm;	catalytic a	Pf07986	6903	ENSG0000	TBCC	6			Post-chape	3	1.036		0.629683988	11.93	333.1	313.7	4.91	26.26	High	High	High	High	High	High	1	
FALSE	High	Master Prc	P02795	metallothi	0	13.281	84	5	5	5	61	6	7.83	10.49	5	cellular ho	cytoplasm;	metal ion l		4502	ENSG0000	MT2A	16	Mineral ab	Copper ho	Metallothi	11	1.036		0.630899075	31.73	331.4	323.8	33.03	3.69	High	High	High	High	High	High	1	
FALSE	High	Master Prc	Q00653-1	Nuclear fa	0	38.629	11	8	11	7	900	96.7	6.25	23.75	8	cell differe	cytoplasm;	DNA bindi	Pf00023, P	4791	ENSG0000	NFKB2	10	MAPK sign	Regulation	IKBKG defi	66	1.036		0.723593319	25.32	1356.3	1309	14.54	6.52	High	High	High	High	High	High	1	
FALSE	High	Master Prc	Q7Z434	Mitochonc	0.003	3.33	3	1	1	1	540	56.5	5.52	1.97	1	cellular coi	membrane	protein bir		57506	ENSG0000	MAVS	20	Hepatitis E	RIG-I-like F	Ovarian tu	21	1.036		0.745751875	17.47	285.5	297.8	13.36	10.03	High	High	High	High	High	High	1	
FALSE	High	Master Prc	Q15637	Splicing fa	0	68.139	22	12	22	12	639	68.3	8.98	63.7	12	cell differe	nucleus;rib	metal ion l	Pf00013, P	7536	ENSG0000	SF1	11			mRNA Spli	4	1.036		0.858700868	5.53	3231.2	3102	0.94	8.62	High	High	High	High	High	High	1	[K15]
FALSE	High	Master Prc	P61158	actin-relat	0	65.246	34	12	20	12	418	47.3	5.88	46.22	12	cell divisio	cytoplasm;	nucleotide	Pf00022	10096	ENSG0000	ACTR3	2		Associatior	EPHB-med	16	1.036		0.932300232	4.6	4613.1	4475	9.43	1.7	High	High	High	High	High	High	1	
FALSE	High	Master Prc	P18846	Cyclic AMF	0.001	4.339	13	2	2	2	271	29.2	8.37	0	2	cell organi	nucleus	DNA bindi	Pf00170, P	466	ENSG0000	ATF1	12	Transcripti	Integrated	CREB phos	38	1.036		0.977518495	12.32	341.6	329.6	18.32	3.98	High	High	High	High	High	High	1	
FALSE	High	Master Prc	Q8TCS8	Polyribonu	0	67.328	26	15	18	15	783	85.9	7.77	50.93	15	cell organi	cytoplasm;	catalytic a	Pf00013, P	87178	ENSG0000	PNPT1	2	RNA degra			3	1.036		0.994108702	9.73	2824.6	2727.7	3.84	8.22	High	High	High	High	High	High	1	
FALSE	High	Master Prc	P82933	28S riboso	0	31.368	22	7	12	7	396	45.8	9.51	33.78	7	cell organi	membrane	protein bir	Pf00380	64965	ENSG0000	MRPS9	2	Ribosome		Mitochonc	7	1.035		0.307420095	0.43	4222.7	4078.3	17.38	3.55	High	High	High	High	High	High	1	
FALSE	High	Master Prc	O43488	aflatoxin B	0	31.741	18	4	5	4	359	39.6	7.17	21.01	4	metabolic	cytoplasm;	catalytic a	Pf00248	8574	ENSG0000	AKR7A2	1	Metabolisr	Aflatoxin E	Aflatoxin a	6	1.035		0.384821018	7.23	1636.8	1543.4	7.48	5.22	High	High	High	High	High	High	1	
FALSE	High	Master Prc	Q86V21-1	acetoacety	0.003	3.423	1	1	1	1	672	75.1	6.24	2.1	1	cell differe	cytoplasm;	catalytic a	Pf00501	65985	ENSG0000	AACS	12	Butanoate		Synthesis c	6	1.035		0.399372324	6.13	500.1	504.8	4.85	5.95	High	High	High	High	High	High	1	
FALSE	High	Master Prc	Q9NPJ3-1	Acyl-coenz	0	28.697	38	4	7	4	140	15	9.14	20.98	4	cell organi	cytoplasm;	catalytic a	Pf03061, P	55856	ENSG0000	ACOT13	6			Mitochonc	4	1.035		0.535495648	3.68	1217.6	1242.4	17.76	14.49	High	High	High	High	High	High	1	
FALSE	High	Master Prc	Q9NUL3	Double-str	0	4.608	2	1	2	1	570	62.6	9.61	3.67	1	transport	cytoplasm;	protein bir	Pf00035	27067		STAU2	8				0	1.035		0.657989898	34.02	395.7	382.4	27.49	6.89	High	High	High	High	High	High	1	
FALSE	High	Master Prc	Q9NW13-1	RNA-bindi	0	36.606	13	10	16	9	759	85.7	9.22	20.61	10	metabolic	nucleus;sp	RNA bindi	Pf00076, P	55131	ENSG0000	RBM28	7	Ribosome		Major path	5	1.035		0.712580601	20.87	1659.5	1760.6	14.8	7.24	High	High	High	High	High	High	1	
FALSE	High	Master Prc	O00469-1	procollage	0	11.691	5	3	3	3	737	84.6	6.71	6.89	3	metabolic	endoplasm	catalytic a	Pf03171	5352	ENSG0000	PLOD2	3	Lysine deg		Collagen b	4	1.035		0.850068493	6.81	776.5	849.6	7.52	11.89	High	High	High	High	High	High	1	
FALSE	High	Master Prc	P56537-1	eukaryotic	0	70.94	60	8	20	8	245	26.6	4.68	61.13	8	cell organi	cytoplasm;	protein bir	Pf01912	3692	ENSG0000	EIF6	20	Ribosome	Translatior		2	1.035		0.973574616	11.32	5235.8	4984.3	12.72	2.66	High	High	High	High	High	High	1	
FALSE	High	Master Prc	Q9H5N1	rab GTPase	0	5.879	4	2	3	2	569	63.5	4.78	1.84	2	regulation	cytoplasm;	enzyme re	Pf03528, P	79874	ENSG0000	RABEP2	16				0	1.034		0.492170481	17.69	363	344.3	28.7	9.41	High	High	High	High	High	High	1	
FALSE	High	Master Prc	P10586-1	Receptor-t	0	11.542	2	3	3	3	1907	212.7	6.3	5.79	3	cell organi	membrane	catalytic a	Pf00041, P	5792	ENSG0000	PTPRF	1	Adherens j	Brain-Deriv	Receptor-t	10	1.034		0.551609799	15.47	156.2	159.6	3.5	18.34	High	High	High	High	High	High	1	
FALSE	High	Master Prc	Q9NVI7-2	Isoform 2 c	0	47.735	28	13	26	13	586	66.2	9.25	52.99	13	cell growth	membrane	catalytic a	Pf00004, P	55210	ENSG0000	ATAD3A	1				0	1.034		0.586112009	18.54	6691.2	6468.4	14.14	6.77	High	High	High	High	High	High	1	
FALSE	High	Master Prc	P40939	Trifunctior	0	151.426	51	29	51	29	763	82.9	9.04	116.38	29	metabolic	membrane	catalytic a	Pf00378, P	3030	ENSG0000	HADHA	2	Carbon me	Fatty Acid	Beta oxida	31	1.034		0.678085344	3.57	10146	10037.7	2.85	4.25	High	High	High	High	High	High	1	[Q378];
FALSE	High	Master Prc	O14737	Programm	0	26.296	49	8	12	8	125	14.3	6.04	9.8	8	cell death;	cytoplasm;	DNA bindi	Pf01984	9141	ENSG0000	PDCD5	19				0	1.034		0.706153594	18.4	3188.4	2965.1	31.23	5.2	High	High	High	High	High	High	1	
FALSE	High	Master Prc	P25815	Protein S10	0	11.979	35	4	7	4	95	10.4	4.88	11.71	4	cellular coi	cytoplasm;	metal ion l	Pf01023	6286	ENSG0000	S100P	4		Gastric Car	Neutrophil	4	1.034		0.809792838	24.78	932.8	899.5	8.02	12.5	High	High	High	High	High	High	1	
FALSE	High	Master Prc	P35221	Catenin alp	0	60.578	18	12	18	9	906	100	6.29	50.16	12	cell organi	cytoplasm;	protein bir	Pf01044	1495	ENSG0000	CTNNA1	5	Adherens j	Arrhythmc	Adherens j	27	1.034		0.893556208	1.28	1710	1745.1	4.45	3.82	High	High	High	High	High	High	1	
FALSE	High	Master Prc	P30043	flavin redu	0	37.11	29	4	18	4	206	22.1	7.65	77.73	4	metabolic	cytoplasm;	catalytic a	Pf01073, P	645	ENSG0000	BLVRB	19	Porphyrin	NRF2 path	Heme degr	6	1.033		0.443967732	8.94	6222.5	6021.9	4.48	3.55	High	High	High	High	High	High	1	
FALSE	High	Master Prc	P14868	Aspartate-	0	17.611	14	7	11	7	501	57.1	6.55	17.74	7	cell organi	cytoplasm;	catalytic a	Pf00152, P	1615	ENSG0000	DARS	2	Aminoacyl	Alanine an	Cytosolic t	10	1.033		0.528632281	16.91	3872.3	3925.6	20.51	4.37	High	High	High	High	High	High	1	
FALSE	High	Master Prc	P47897	glutamine-	0	117.776	31	18	27	18	775	87.7	7.15	95.1	18	metabolic	cytoplasm;	catalytic a	Pf00749, P	5859	ENSG0000	QARS	3	Metabolic		Mitochonc	11	1.033		0.547941293	13.72	6964.5	6739.8	9.4	4.75	High	High	High	High	High	High	1	
FALSE	High	Master Prc	Q9H2P9-1	Diphthine	0	11.793	7	1	2	1	285	31.6	5.31	12.8	1	metabolic	cytosol	catalytic a	Pf00590	51611	ENSG0000	DPH5	1			Synthesis c	4	1.033		0.555811375	18.48	556.2	538.3	11.64	10.92	High	High	High	High	High	High	1	
FALSE	High	Master Prc	O96019-1	Actin-like p	0	9.443	7	2	2	2	429	47.4	5.6	4.45	2	cell organi	membrane	DNA bindi	Pf00022	86	ENSG0000	ACTL6A	3		Gastric Car	RUNX1 int	18	1.033		0.559549702	6.16	196.6	206.8	11.92	18.37	High	High	High	High	High	High	1	
FALSE	High	Master Prc	Q9HAV7	GrpE prote	0	27.569	38	9	12	9	217	24.3	8.12	26.91	9	metabolic	mitochond	enzyme re	Pf01025	80273	ENSG0000	GRPEL1	4			Mitochonc	2	1.033		0.578155509	15.04	4329.2	4192.9	8.69	4.81	High	High	High	High	High	High	1	
FALSE	High	Master Prc	Q5F1R6	DnaJ homc	0	23.595	13	5	6	5	531	62	5.47	14.52	5	metabolic	cytoplasm;	DNA bindi	Pf00226, P	134218	ENSG0000	DNAJC21	5				0	1.033		0.724346307	2.12	207.5	209.5	18.67	11.18	High	High	High	High	High	High	1	
FALSE	High	Master Prc	Q9NRG9-1	Aladin [OS	0	18.446	8	3	7	3	546	59.5	7.5	20.33	3	cell organi	cytosol;me	protein bir	Pf00400	8086	ENSG0000	AAAS	12	RNA transp		Rev-media	64	1.033		0.741461658	22.27	1796	1738.3	11.73	4.27	High	High	High	High	High	High	1	
FALSE	High	Master Prc	Q99442	translocati	0	22.369	16	6	10	6	399	45.8	7.12	23.12	6	cell organi	cytosol;en	receptor a	Pf03839	7095	ENSG0000	SEC62	3	Protein exp		XBP1(S) ac	6	1.033		0.92802056	14.59	4224.7	4373.6	13.58	4.14	High	High	High	High	High	High	1	
FALSE	High	Master Prc	Q13363-1	C-terminal	0	31.445	17	7	8	3	440	47.5	6.77	14.82	7	cell differe	cytoplasm;	catalytic a	Pf00389, P	1487	ENSG0000	CTBP1	4	Pathways i	Notch Sign	Repression	19	1.032		0.131193361	0.75	1591.8	1482.8	4.77	6.56	High	High	High	High	High	High	1	
FALSE	High	Master Prc	Q9H061-1	Transmem	0	12.808	24	4	5	4	195	21.5	9.26	8.27	4		membrane		Pf07114	84233	ENSG0000	TMEM126	11				0	1.032		0.283734376	4.4	710.8	647.1	3.96	6.82	High	High	High	High	High	High	1	
FALSE	High	Master Prc	P67809	Nuclease-s	0	168.938	54	15	61	10	324	35.9	9.88	170.24	15	metabolic	cytoplasm;	DNA bindi	Pf00313	4904	ENSG0000	YBX1	1		Brain-Deriv	mRNA Spli	9	1.032		0.348064259	2.47	8234.1	8341.1	8.53	6.85	High	High	High	High	High	High	1	
FALSE	High	Master Prc	Q06203	amidophos	0	27.731	12	5	6	5	517	57.4	6.76	18.94	5	cell organi	cytosol	catalytic a	Pf00156, P	5471	ENSG0000	PPAT	4	Purine met	Fluoropyri	Purine ribc	8	1.032		0.369599741	6.71	2058.9	1981.6	2.36	4.41	High	High	High	High	High	High	1	
FALSE	High	Master Prc	Q6KB66	Keratin, ty	0	18.656	10	4	7	3	452	50.5	5.67	17.87	4	cell death;	cytoplasm;	protein bir	Pf00038, P	144501	ENSG0000	KRT80	12			Formation	3	1.032		0.475767551	0.57	70.6	71.3	24.95	20.56	High	High	High	High	High	High	1	
FALSE	High	Master Prc	Q6PJT7-1	Zinc finger	0	9.329	3	2	5	2	736	82.8	7.31	14.13	2	regulation	cytoplasm;	metal ion l	Pf14608	79882	ENSG0000	ZC3H14	14				0	1.032		0.477177023	19.16	1565.6	1446.3	36.94	3.26	High	High	High	High	High	High	1	
FALSE	High	Master Prc	Q03701	CCAAT/enh	0	16.083	7	5	6	5	1054	120.9	5.94	10.99	5	metabolic	nucleus	DNA bindi	Pf03914	10153	ENSG0000	CEBPZ	2		Gastric car		1	1.032		0.519733388	7.24	1525	1457	2.19	4.73	High	High	High	High	High	High	1	
FALSE	High	Master Prc	Q9H2G2-1	STE20-like	0	25.74	7	8	8	8	1235	142.6	5.15	12	8	cell death;	cytoplasm	catalytic a	Pf00069, P	9748	ENSG0000	SLK	10	Oocyte me			1	1.032		0.592585238	19.06	1347.4	1431	20.1	5.88	High	High	High	High	High	High	1	
FALSE	High	Master Prc	Q15046	Lysine--tRN	0	81.92	37	20	36	20	597	68	6.35	68.16	20	metabolic	cytoplasm;	catalytic a	Pf00152, P	3735	ENSG0000	KARS	16	Aminoacyl		Mitochonc	10	1.032		0.628307769	8.55	15496.4	15014.1	0.34	7.14	High	High	High	High	High	High	1	

FALSE	High	Master Prc	Q14151	scaffold at	0	40.111	14	11	14	6	953	107.4	6.16	25.19	11	cell differe	cytoplasm;	DNA bindi	Pf00076, P	9667	ENSG0000	SAFB2	19				0	1.032	0.628524714	21.8	817.8	835.8	20.47	3.32	High	High	High	High	High	High	1	[K746;
FALSE	High	Master Prc	P06756	Integrin al	0.002	3.644	2	2	2	2	1048	116	5.68	2.36	2	cell differe	cell surface	metal ion I	Pf01839, P	3685	ENSG0000	ITGAV	2	Small cell l	Osteopont	Integrin ce	48	1.032	0.6779263	33.16	684.6	606.7	24.62	9.43	High	High	High	High	High	High	1	
FALSE	High	Master Prc	Q9NVG8	TBC1 dom:	0	4.547	4	1	1	1	400	46.5	5.24	3.09	1	regulation	cytoplasm;	enzyme re;	Pf00566	54662	ENSG0000	TBC1D13	9			TBC/RABG	4	1.032	0.681827068	24.8	315	323.5	21.37	3.41	High	High	High	High	High	High	1	
FALSE	High	Master Prc	Q8TBC3	SH3KBP1-b	0	7.917	4	2	2	1	707	76.3	8.28	4.12	2	cell organi	vacuole	protein bir	Pf02214	92799	ENSG0000	SHKBP1	19				0	1.032	0.716198618	34.42	717.2	720.4	27.24	6.72	High	High	High	High	High	High	1	
FALSE	High	Master Prc	Q99956	Dual specif	0	11.244	8	2	2	2	384	41.8	6.11	6.53	2	metabolic	cytoplasm;	catalytic a	Pf00102, P	1852	ENSG0000	DUSP9	X	MAPK sign		Negative r	12	1.032	0.781490986	25.97	433.7	420.1	8.64	11.74	High	High	High	High	High	High	1	
FALSE	High	Master Prc	Q9Y4P3	Transducin	0	9.131	5	2	2	2	447	49.8	9.44	2.65	2	cell commu	endoplasm	protein bir	Pf00400	26608	ENSG0000	TBL2	7				0	1.032	0.801246731	20.83	628.9	582.5	7.88	12.2	High	High	High	High	High	High	1	
FALSE	High	Master Prc	O43660-1	Pleiotropic	0	23.704	13	6	11	6	514	57.2	9.17	17.09	6	cell organi	membrane	catalytic a	Pf00400	5356	ENSG0000	PLRG1	4	Spliceosom		mRNA Spli	5	1.032	0.884303855	11.12	630	601.7	15.53	0.94	High	High	High	High	High	High	1	
FALSE	High	Master Prc	Q9Y2Q3-1	Glutathion	0	18.901	19	4	5	4	226	25.5	8.41	11.39	4	cell differe	membrane	antioxidan	Pf01323	373156	ENSG0000	GSTK1	7	Chemical c	Metapathv	Glutathion	10	1.032	0.906763837	15.87	2220.2	2150.8	13.55	4.55	High	High	High	High	High	High	1	
FALSE	High	Master Prc	P49419	Alpha-ami	0	76.409	33	14	24	14	539	58.5	7.99	73.39	14	metabolic	cytoplasm;	catalytic a	Pf00171	501	ENSG0000	ALDH7A1	5	beta-Alani	Amino Aci	Lysine cata	21	1.032	0.909089382	0.99	11150.5	11417.3	5.36	4.01	High	High	High	High	High	High	1	
FALSE	High	Master Prc	P52565-1	rho GDP-di	0	46.755	50	7	16	7	204	23.2	5.11	44.5	7	cellular co	cytoplasm;	enzyme re;	Pf02115	396	ENSG0000	ARHGDIA	17	Vasopressi		Signalling l	11	1.032	0.910223855	2.95	5245.6	5334.9	5.3	4.03	High	High	High	High	High	High	1	
FALSE	High	Master Prc	O75694	nuclear po	0	109.787	20	19	31	19	1391	155.1	6.16	81.94	19	cell organi	membrane	protein bir	Pf03177, P	9631	ENSG0000	NUP155	5	RNA trans		Rev-media	64	1.032	0.915490959	4.88	7935.9	7699.6	8.13	2.23	High	High	High	High	High	High	1	
FALSE	High	Master Prc	O43684	Mitotic che	0	49.978	44	9	17	9	328	37.1	6.84	36.58	9	cell divisio	chromosor	protein bir	Pf00400, P	9184	ENSG0000	BUB3	10	Cell cycle;	Cell Cycle	Inactivatio	29	1.032	0.976288107	12.17	1851.7	1798.4	4.84	7.85	High	High	High	High	High	High	1	
FALSE	High	Master Prc	Q14257	Reticulocal	0	17.479	20	5	5	5	317	36.9	4.4	11.89	5		endoplasm	metal ion I	Pf13499	5955	ENSG0000	RCN2	15				0	1.031	0.058126333	1.44	445	431.4	3.76	2.42	High	High	High	High	High	High	1	
FALSE	High	Master Prc	Q9NQ50	39S riboso	0	12.455	20	3	5	3	206	24.5	9.63	13.64	3	cell organi	membrane	protein bir	Pf09812	64976	ENSG0000	MRPL40	22			Mitochond	6	1.031	0.198289926	1.46	629.7	581.3	6.36	4.36	High	High	High	High	High	High	1	
FALSE	High	Master Prc	P49257	Protein ER	0	101.976	37	13	35	13	510	57.5	6.77	128.04	13	cell organi	endoplasm	metal ion I	Pf03388	3998	ENSG0000	LMAN1	18	Protein pro	Compleme	Cargo conc	11	1.031	0.204233188	0.12	12964.8	12473.2	3	3.06	High	High	High	High	High	High	1	
FALSE	High	Master Prc	P52948-1	nuclear po	0	98.783	17	25	33	25	1817	197.5	6.4	74.65	25	cell organi	chromosor	catalytic a	Pf04096, P	4928	ENSG0000	NUP98	11	Influenza A			2	1.031	0.361092331	5.15	9443.6	9162.3	8.66	2.63	High	High	High	High	High	High	1	
FALSE	High	Master Prc	O00567	Nucleolar p	0	94.362	33	13	26	13	594	66	9.19	89.62	13	metabolic	cytoplasm;	protein bir	Pf01798, P	10528	ENSG0000	NOP56	20	Ribosome		Major path	10	1.031	0.371430041	2.52	7851.1	7606.7	2.87	1.18	High	High	High	High	High	High	1	
FALSE	High	Master Prc	Q9NZ45	CDGSH iron	0	23.374	26	2	8	2	108	12.2	9.09	29.97	2	regulation	membrane	metal ion I	Pf09360	55847	ENSG0000	CISD1	10		Adipogene		1	1.031	0.415029144	7.3	3138.4	3045.3	26.23	5.08	High	High	High	High	High	High	1	
FALSE	High	Master Prc	P60866	40S riboso	0	33.012	30	5	26	5	119	13.4	9.94	73	5	cell organi	cytoplasm;	protein bir	Pf00338	6224	ENSG0000	RPS20	8	Ribosome	Cytoplasm	Peptide ch	38	1.031	0.434821199	8.65	9850.1	8947	4.99	12.04	High	High	High	High	High	High	1	
FALSE	High	Master Prc	Q96PM9	Zinc finger	0	5.085	8	2	2	2	386	40.4	9.86	4.4	2	cell death;	cytoplasm;	DNA bindi	Pf07535, P	25946	ENSG0000	ZNF385A	12			Regulation	16	1.031	0.55236997	24.66	718.7	697	28.2	8.14	High	High	High	High	High	High	1	
FALSE	High	Master Prc	Q8N556-1	actin filam	0	5.636	4	2	2	2	730	80.7	8.68	3.69	2	regulation	cytoplasm;	protein bir	Pf00169	60312	ENSG0000	AFAP1	4				0	1.031	0.595533945	3.68	445.4	450.5	4.85	4.73	High	High	High	High	High	High	1	
FALSE	High	Master Prc	Q96FS4	signal-indu	0	18.309	6	4	4	4	1042	112.1	6.6	10	4	cell commu	cytoplasm;	catalytic a	Pf00595, P	6494	ENSG0000	SIPA1	11	Rap1 signa	MAPK Cas	Rap1 signa	6	1.031	0.627776504	1.79	628.5	635.5	10.4	5.36	High	High	High	High	High	High	1	
FALSE	High	Master Prc	P18583-1	Protein SO	0	8.985	2	3	3	3	2426	263.7	5.64	6.32	3	cell organi	extracellul	DNA bindi	Pf00035, P	6651	ENSG0000	SON	21				0	1.031	0.649990792	43.42	192.9	187.2	40.18	15.91	High	High	High	High	High	High	1	
FALSE	High	Master Prc	Q15365	Poly(RC)-b	0	113.159	65	16	65	10	356	37.5	7.09	167.88	16	metabolic	cytoplasm;	DNA bindi	Pf00013, P	5093	ENSG0000	PCBP1	2	Spliceosom		mRNA Spli	5	1.031	0.839982705	4.1	9303.2	9200.7	8.83	2.72	High	High	High	High	High	High	1	
FALSE	High	Master Prc	Q9C0C9	(E3-indepe	0	8.311	2	2	2	2	1292	141.2	5.12	5.67	2	metabolic	cytoplasm;	catalytic a	Pf00179	63893	ENSG0000	UBE2O	17	Ubiquitin r		Antigen pr	5	1.031	0.853342698	58.85	330.3	381.4	44.94	13.32	High	High	High	High	High	High	1	
FALSE	High	Master Prc	Q96GA3	protein LTV	0	10.422	6	2	2	2	475	54.8	4.91	6.54	2	metabolic	cytosol;me	protein bir	Pf04180	84946	ENSG0000	LTV1	6			Major path	4	1.031	0.937690263	7.25	181.6	176.1	2.23	10.39	High	High	High	High	High	High	1	
FALSE	High	Master Prc	Q9UKL0	REST corep	0	6.242	7	2	2	2	485	53.3	7.03	4.7	2	cell organi	nucleus	catalytic a	Pf00249, P	23186	ENSG0000	RCOR1	14	Huntingtor		HDACs dea	11	1.031	0.940426185	10.72	255.5	257.1	9.83	5.03	High	High	High	High	High	High	1	
FALSE	High	Master Prc	Q9NWH9-	SAFB-like t	0	24.452	8	7	9	6	1034	117.1	7.87	14.88	7	cell death;	nucleus	DNA bindi	Pf00076, P	79811	ENSG0000	SLTM	15				0	1.03	0.012933045	0.56	2232	2159.4	4.97	5.49	High	High	High	High	High	High	1	
FALSE	High	Master Prc	Q16576-1	Histone-bi	0	65.197	38	11	29	7	425	47.8	5.05	72.31	11	cell organi	cytosol;nu	catalytic a	Pf00400, P	5931	ENSG0000	RBBP7	X		Retinoblas	RMTs meth	41	1.03	0.35911094	5.06	7087.1	6601.3	7.91	4.67	High	High	High	High	High	High	1	
FALSE	High	Master Prc	P09429	High mobil	0	72.298	38	8	28	4	215	24.9	5.74	79.24	8	cell differe	cell surface	catalytic a	Pf00505, P	3146	ENSG0000	HMGB1	13	Base excisi	Retinoblas	TRAF6 med	40	1.03	0.368174254	5.59	3603.2	3231.7	5.38	10.84	High	High	High	High	High	High	1	
FALSE	High	Master Prc	P50897	Palmitoyl-p	0	11.428	12	3	4	3	306	34.2	6.52	9.96	3	cell commu	cytosol;ext	catalytic a	Pf02089	5538	ENSG0000	PPT1	1	Lysosome;		Fatty acyl-	8	1.03	0.540929707	11.3	829.7	805.3	7.2	1.79	High	High	High	High	High	High	1	
FALSE	High	Master Prc	P29353	SHC-transf	0	6.22	7	1	1	1	583	62.8	6.44	3.73	1	cell organi	cytoplasm;	protein bir	Pf00017, P	6464	ENSG0000	SHC1	1	Breast can	IL-5 Signali	Tie2 Signal	101	1.03	0.685039674	54.97	209.7	235.2	54.58	18.82	High	High	High	High	High	High	1	
FALSE	High	Master Prc	Q14974	Importin su	0	115.31	27	21	45	21	876	97.1	4.78	113.38	21	cell organi	cytoplasm;	metal ion I	Pf02985, P	3837	ENSG0000	KPNB1	17	RNA trans	Sterol Reg	ISG15 anti	29	1.03	0.922489364	7.03	13016.7	12541.8	16.4	5.2	High	High	High	High	High	High	1	
FALSE	High	Master Prc	Q9Y697	Cysteine de	0	29.35	23	6	6	6	457	50.2	8.31	20.76	6	cell organi	cytoplasm;	catalytic a	Pf00266, P	9054	ENSG0000	NFS1	20	Thiamine r		Mitochond	5	1.03	0.95855148	40.03	1858.9	1804.5	18.18	13.29	High	High	High	High	High	High	1	
FALSE	High	Master Prc	P31943	Heterogen	0	147.191	46	14	57	6	449	49.2	6.3	196.45	14	metabolic	cytosol;me	protein bir	Pf00076, P	3187	ENSG0000	HNRNPH1	5; CHR_HG		Mecp2 and	mRNA Spli	11	1.029	0.137330215	0.52	8688.4	8469	3.1	6.5	High	High	High	High	High	High	1	[Y306(100
FALSE	High	Master Prc	P49189	4-trimethy	0	89.39	39	16	30	16	494	53.8	5.87	63.85	16	metabolic	cytoplasm;	catalytic a	Pf00171	223		ALDH9A1	1	Tryptopha	Tryptopha	Carnitine s	17	1.029	0.227566592	0.45	7984	7779.8	6.92	1.11	High	High	High	High	High	High	1	
FALSE	High	Master Prc	P09651-1	Heterogen	0	254.831	70	31	110	1	372	38.7	9.13	289.48	31	cell commu	cytoplasm;	DNA bindi	Pf00076, P	3178	ENSG0000	HNRNPA1	12	Spliceosom	mRNA Pro	mRNA Spli	11	1.029	0.498528766	12.55	60.6	57.8	16.35	8.01	High	High	High	High	High	High	1	[R31;
FALSE	High	Master Prc	P54577	Tyrosine--t	0	83.738	34	15	23	15	528	59.1	7.05	51.24	15	cell death;	cytoplasm;	catalytic a	Pf00579, P	8565	ENSG0000	YARS	1	Aminoacyl		Cytosolic t	5	1.029	0.551260282	11.99	4747.4	4613.7	6.92	5.31	High	High	High	High	High	High	1	
FALSE	High	Master Prc	P29401	Transketol	0	412.004	69	36	169	36	623	67.8	7.66	508.13	36	metabolic	cytosol	catalytic a	Pf00456, P	7086	ENSG0000	TKT	3	Pentose ph	Pentose Ph	Insulin effe	10	1.029	0.552547355	8.8	61173.7	58404.6	5.42	2.09	High	High	High	High	High	High	1	
FALSE	High	Master Prc	Q6YHK3-1	CD109 ant	0.005	2.558	1	1	1	1	1445	161.6	5.85	0	1	metabolic	cell surface	enzyme re;	Pf00207, P	135228	ENSG0000	CD109	6			Platelet de	7	1.029	0.684449459	26.04	602.7	613.1	20.68	9.07	High	High	High	High	High	High	1	
FALSE	High	Master Prc	P61077-1	Ubiquitin-c	0	6.249	7	1	3	1	147	16.7	7.8	7.47	1	cell death;	cytosol;en	catalytic a	Pf00179, P	7323	ENSG0000	UBE2D3	4	Ubiquitin r	Proteasom	IKK comple	37	1.029	0.80459918	12.82	1410.1	1260.7	12.04	5.04	High	High	High	High	High	High	1	
FALSE	High	Master Prc	P17174	Aspartate a	0	25.746	20	5	5	5	413	46.2	7.01	16.58	5	metabolic	cytoplasm;	catalytic a	Pf00155	2805	ENSG0000	GOT1	10	Tyrosine m	Alanine an	Gluconeog	24	1.029	0.865416128	0.81	374.8	362.4	8.23	4.81	High	High	High	High	High	High	1	
FALSE	High	Master Prc	P46778	60S riboso	0	23.869	35	8	16	8	160	18.6	10.49	30.14	8	cell organi	cytosol;me	protein bir	Pf01157	6144	ENSG0000	RPL21	13	Ribosome	Cytoplasm	Peptide ch	34	1.029	0.925428372	14.6	3405.9	3437.7	12.65	5.24	High	High	High	High	High	High	1	
FALSE	High	Master Prc	Q3LXA3	Triokinase/	0	49.584	22	10	12	10	575	58.9	7.49	35.39	10	defense re	cytosol;nu	catalytic a	Pf02733, P	26007	ENSG0000	DAK; TKFC	11	RIG-I-like r	RIG-I-like F	Fructose ca	13	1.028	0.283953345	4.41	3040.6	2958.9	5.73	2.82	High	High	High	High	High	High	1	
FALSE	High	Master Prc	Q7L9L4	mob kinase	0.002	4.15	5	1	1	1	216	25.1	6.73	2.99	1	regulation	cytoplasm;	enzyme re;	Pf03637	92597	ENSG0000	MOB1B	4	Hippo sign		Signaling b	4	1.028	0.305471592	2.42	646.5	625.9	2.6	7.17	High	High	High	High	High	High	1	
FALSE	High	Master Prc	O75390	citrate syn	0	68.483	29	13	36	13	466	51.7	8.32	69.18	13	metabolic	mitochond	catalytic a	Pf00285	1431	ENSG0000	CS	12	Glyoxylate	TCA Cycle	Mitochond	15	1.028	0.424069785	7.29	9288	9203.5	9.23	3.31	High	High	High	High	High	High	1	
FALSE	High	Master Prc	Q9BXP5	serrate RN	0	57.674	20	18	27	18	876	100.6	5.96	36.38	18	cell prolife	cytoplasm;	DNA bindi	Pf04959, P	51593	ENSG0000	SRRT	7			RNA polym	7	1.028	0.429087615	7.14	6621.4	6342	4.06	4.2	High	High	High	High	High	High	1	
FALSE	High	Master Prc	P43121	Cell surface	0	22.064	13	7	9	7	646	71.6	5.76	10.01	7	regulation	extracellul	protein bir	Pf00047, P	4162	ENSG0000	MCAM	11				0	1.028	0.436549419	8.25	2748.7	2674	12.08	2.13	High	High	High	High	High	High	1	
FALSE	High	Master Prc	Q14134-1	Tripartite r	0	50.665	20	12	18	12	588	65.8	7.15	36.39	12	metabolic	cytoplasm	metal ion I	Pf00643, P	23650	ENSG0000	TRIM29	11			Interferon	4	1.028	0.501610954	16.42	3701.1	3554.5	25.04	8.17	High	High	High	High	High	High	1	
FALSE	High	Master Prc	Q9BVC4	target of ra	0	5.127	7	2	2	2	326	35.9	5.86	4.8	2	cell organi	cytoplasm;	enzyme re;	Pf00400	64223	ENSG0000	MLST8	16	PI3K-Akt si	Regulation	HSF1-depe	43	1.028	0.606578218	19.86	257.5	263.5	35.73	11.58	High	High	High	High	High	High	1	
FALSE	High	Master Prc	Q9NP92	39S riboso	0	20.984	13	4	6	4	439	50.3	7.97	19.03	4	cell death;	membrane	RNA bindi	Pf07147	10884	ENSG0000	MRPS30	5			Mitochond	6	1.028	0.617305683	2.58	1001.7	1021.7	12.97	4.2	High	High	High	High	High	High	1	
FALSE	High	Master Prc	Q14244	Ensconsin	0	24.31	11	7	9	7	749	84	9.61	17.69	7	cell organi	cytoplasm;	protein bir		9053	ENSG0000	MAP7	6				0	1.028	0.632590372	22.42	177.8	172.9	19.69	2.56	High	High	High	High	High	High	1	
FALSE	High	Master Prc	P07737	profilin-1 [	0	121.223	74	12	54	12	140	15	8.27	138.02	12	cell organi	cytoplasm;	enzyme re;	Pf00235	5216	ENSG0000	PFN1	17	Regulation	Associatio	Platelet de	22	1.028	0.70275229	20.85	18553.9	15710.4	17.13	8.62	High	High	High	High	High	High	1	
FALSE	High	Master Prc	P04049	RAF proto-	0	5.252	4	1	1	1	648	73	9.2	3.49	1	cell death;	cytoplasm;	catalytic a	Pf00069, P	5894	ENSG0000	RAF1	3	Insulin sigr	IL-5 Signali	Signaling b	147	1.028	0.89014145	20.12	100	97.3	6.86	8.53	High	High	High	High	High	High	1	
FALSE	High	Master Prc	P19404	NADH deh	0	15.313	18	3	6	3	249	27.4	8.06	16.29	3	cell organi	membrane	catalytic a	Pf01257	4729	ENSG0000	NDUFV2	18	Alzheimer'	Electron Tr	Complex I	13	1.028	0.942969841	21.72	889.7	865.5	7.78	8.93	High	High	High	High	High	High	1	
FALSE	High	Master Prc	Q9UN86	Ras GTPas	0	27.899	21	8	19	8	482	54.1	5.55	50.22	8	cell organi	cytoplasm;	protein bir	Pf00076, P	9908	ENSG0000	G3BP2	4				0	1.027	0.305153585	2.57	7369.3	6729.3	5.56	8.49	High	High	High	High	High	High	1	
FALSE	High	Master Prc	Q9BQ04	RNA-bindi	0	34.935	25	9	13	9	359	40.1	6.74	31.52	9	metabolic	cytosol;nu	metal ion I	Pf00076, P	83759	ENSG0000	RBM4B	11				0	1.027	0.358138738	0.66	3897.5	3707.7	16.67	5.08	High	High	High	High	High	High	1	
FALSE	High	Master Prc	O00273-1	DNA fragm	0	36.471	18	5	9	5	331	36.5	4.79	25.56	5	cell death;	cytoplasm;	enzyme re;	Pf02017, P	1676	ENSG0000	DFFA	1	Apoptosis	Apoptosis;	Activation	8	1.027	0.38861028	5.99	2964.2	2864.8	0.58	5.92	High	High	High	High	High	High	1	
FALSE	High	Master Prc	P11717	Cation-ind	0	66.296	8	16	19	16	2491	274.2	5.94	31.08	16	cell organi	cell surface	catalytic a	Pf00040, P	3482	ENSG0000	IGF2R	6	Lysosome;		Cargo reco	14	1.027	0.510263184	9.84	3602.2	3388.9	3.75	8.55	High	High	High	High	High	High	1	
FALSE	High	Master Prc	Q13595-1	Transform	0	42.851	38	9	17	6	282	32.7	11.27	28.59	9	metabolic	nucleus	RNA bindi	Pf00076, P	29896	ENSG0000	TRA2A	7	Spliceosom			1	1.027	0.566023598	1.29	3225.9	3202.2	8.62	8.47	High	High	High	High	High	High	1	
FALSE	High	Master Prc	Q13404	Ubiquitin-c	0	25.446	60	8	13	5	147	16.5	7.93	24.91	8	cell differe	cytoplasm;	catalytic a	Pf00179	7335; 387!	ENSG0000	UBE2V1; T	20		IL-1 signali	IRAK1 recr	46	1.027	0.57631183	14.09	2308.1	2226.4	3.56	12.64	High	High	High	High	High	High	1	
FALSE	High	Master Prc	Q8WWI1	LIM domai	0	100.147	17	24	36	24	1683	192.6	8.09	77.94	24	metabolic	cytoplasm;	catalytic a	Pf00307, P	4008		LMO7	13	Adherens j		Antigen pr	8	1.027	0.633331399	16.51	7901.7	7888.5	12.19	6.2	High	High	High	High	High	High	1	
FALSE	High	Master Prc	Q8WZA0	Protein LZI	0	4.728	12	1	1	1	190	21.5	4.94	3.88	1	response t		protein bir	Pf06384	84328	ENSG0000	LZIC	1				0	1.027	0.68292237	35.79	181.2	182.2	34.1	16.98	High	High	High	High	High	High	1	
FALSE	High	Master Prc	Q9ULX6	A-kinase a	0	20.998	8	5	5	5	646	71.6	5.07	13.3	5	cell organi	chromosor	DNA bindi	Pf04988	26993		AKAP8L	19	Epstein-Ba			1	1.027	0.683023568	1.91	708	719.2	4.22	2.62	High	High	High	High	High	High	1	
FALSE	High	Master Prc	P21980	Protein-glu	0	20.69	8	4	5	4	687	77.3	5.22	15.97	4	cell organi	cytoplasm;	catalytic a	Pf00868, P	7052	ENSG0000	TGM2	20	Huntingtor			1	1.027	0.694245748	17.18	1813.3	1856.5	17.01	11.96	High	High	High	High	High	High	1	
FALSE	High	Master Prc	Q00577	Transcripti	0	8.837	7	1	1	1	322	34.9	6.44	3.24	1	cell differe	cytoplasm;	DNA bindi	Pf04845	5813	ENSG0000	PURA	5		Exercise-in		1	1.027	0.748960424	21.07	164	169.3	5.1	16.29	High	High	High	High	High	High	1	
FALSE	High	Master Prc	Q8IY81	pre-rRNA p	0	62.686	20	12	16	12	847	96.5	8.4	45.25	12	metabolic	nucleus	catalytic a	Pf01728, P	117246	ENSG0000	FTSJ3	17			Major path	4	1.027	0.831010177	21.5	4147	4049.9	15.42	9.31	High	High	High	High	High	High	1	
FALSE	High	Master Prc	P08865	40S riboso	0	85.381	47	10	50	10	295	32.8	4.87	135.97	10	cell organi	cytoplasm;	protein bir	Pf00318	3921	ENSG0000	RPSA	3	Ribosome	Cytoplasm	Peptide ch	38	1.027	0.863663895	14.28	14254.3	13609.9	17.11	1.87	High	High	High	High	High	High	1	
FALSE	High	Master Prc	Q70UQ0-4	Isoform 4 c	0	40.792	24	7	12	7	377	43.1	4.92	35.18	7	response t	endoplasm	protein bir	Pf10146, P	121457	ENSG0000	IKBIP	12				0	1.026	0.3529228	2.02	5935.2	5717.5	13.05	2.05	High	High	High	High	High	High	1	
FALSE	High	Master Prc	P30533	alpha-2-ma	0	26.931	17	4	4	4	357	41.4	8.78	14.38	4	regulation	cell surface	protein bir	Pf06400, P	4043	ENSG0000	LRPAP1	4				0	1.026	0.378673141	2.5	747.6	741.3	26.19	7.35	High	High	High	High	High	High	1	
FALSE	High	Master Prc	Q969X5	Endoplasm	0	42.436	22	5	18	5	290	32.6	7.06	40.92	5	transport	endoplasm	protein bir	Pf07970, P	57222	ENSG0000	ERGIC1	5				0	1.026	0.415695667	6.83	9019.8	8642.1	10.81	3.7	High	High	High	High	High	High	1	
FALSE	High	Master Prc	Q9BTE3-1	Mini-chror	0	42.666	18	9	13	9	642	72.9	5.87	33.81	9	cell divisio	cytosol;me	protein bir	Pf09739, P	79892	ENSG0000	MCMBP	10				0	1.026	0.47363837	2.61	4389.5	4412.4	3.27	1.83	High	High	High	High	High	High	1	
FALSE	High	Master Prc	P14866	Heterogen	0	182.148	47	22	73	20	589	64.1	8.22	178.33	22	metabolic	cytoplasm;	DNA bindi	Pf00076, P	3191	ENSG0000	HNRNPL	19; CHR_H		mRNA Pro	mRNA Spli	5	1.026	0.546832744	13.47	13645.1	12905.9	11.18	4.59	High	High	High	High	High	High	1	
FALSE	High	Master Prc	P09543-1	2',3'-cyclic-	0	35.209	18	5	7	5	421	47.5	9.07	21.07	5	cell commu	cytoplasm;	catalytic a	Pf01591, P	1267	ENSG0000	CNP	17		Glial Cell D		2	1.026	0.589099658	17.16	830.7	809.9	13.21	5.72	High	High	High	High	High	High	1	
FALSE	High	Master Prc	P49721	proteasom	0	13.484	20	3	7	3	201	22.8	7.02	18.57	3	metabolic	cytoplasm;	catalytic a	Pf00227	5690	ENSG0000	PSMB2	1	Proteasom	Proteasom	Regulation	150	1.026	0.606265225	35.82	1358	1467.5	43.1	12.05	High	High	High	High	High	High	1	
FALSE	High	Master Prc	O75600-1	2-amino-3-	0	7.561	5	1	1	1	419	45.3	8.05	5.55	1	metabolic	membrane	catalytic a	Pf00155, P	23464	ENSG0000	GCAT	22	Glycine, se		Threonine	4	1.026	0.631636745	9.9	269.8	262.9	13.83	9.99	High	High	High	High	High	High	1	
FALSE	Low	Master Prc	P13942-1	Collagen al	0.069	1.048	2	1	1	1	1736	171.7	6.21	2.6	1	cell organi	extracellul	metal ion I	Pf01391, P	1302	ENSG0000	COL11A2	6; CHR_HS	Protein dig	Neural Cre	Collagen d	17	1.026	0.706145529	19.37	216.8	201.6	4.9	15.93	High	High	High	High	High	High	1	[R130;
FALSE	High	Master Prc	O75489	NADH deh	0	50.711	43	9	14	9	264	30.2	7.5	39.75	9	cell organi	membrane	catalytic a	Pf00329	4722	ENSG0000	NDUFS3	11	Alzheimer'	Electron Tr	Complex I	13	1.026	0.716501201	2.16	2687.4	2583	1.13	4.55	High	High	High	High	High	High	1	
FALSE	High	Master Prc	O14579-1	coatomer s	0	31.23	31	8	10	8	308	34.5	5.12	16.4	8	transport	cytoplasm;	protein bir	Pf04733	11316	ENSG0000	COPE	19			COPI-depe	11	1.026	0.878194142	4.86	1662.5	1620.9	9.8	0.58	High	High	High	High	High	High	1	

FALSE	High	Master Prc	Q9NVC6	mediator c	0	7.713	5	2	2	2	651	72.8	7.44	4.69	2	metabolic	membrane	protein bir	Pf10156	9440	ENSG0000	MED17	11	Thyroid ho		Transcripti	10	1.026	0.905658437	9.6	640.5	624.4	9.67	2.56	High	High	High	High	High	High	1	
FALSE	High	Master Prc	Q9H7M9	V-type imr	0	6.559	11	2	3	2	311	33.9	6.95	5.98	2		membrane	protein bir		64115	ENSG0000	C10orf54;	10				0	1.026	0.934764796	9.26	1686.7	1716.8	5.06	11.58	High	High	High	High	High	High	1	
FALSE	High	Master Prc	O60502	protein O-(	0	47.342	14	9	12	9	916	102.8	4.91	33.76	9	metabolic	cytoplasm;	catalytic a	Pf07555	10724	ENSG0000	MGEA5	10	Insulin resi			1	1.026	0.970214053	6.28	1260.5	1314	6.76	5.15	High	High	High	High	High	High	1	
FALSE	High	Master Prc	Q13619-1	cullin-4A [(	0	28.762	12	8	9	7	759	87.6	8.13	22.22	8	cell organi:		catalytic a	Pf00888, P	8451	ENSG0000	CUL4A	13	Nucleotide		Formation	17	1.025	0.278917428	0.83	1658.9	1462.1	6.85	9.51	High	High	High	High	High	High	1	
FALSE	High	Master Prc	Q04446	1,4-alpha-g	0	25.179	11	7	8	7	702	80.4	6.32	15.32	7	metabolic	cytosol	catalytic a	Pf00128, P	2632	ENSG0000	GBE1	3	Metabolic	Glycogen M	Glycogen s	12	1.025	0.282520246	2.86	1462	1470.8	2.82	3.92	High	High	High	High	High	High	1	
FALSE	High	Master Prc	Q6RW13	Type-1 ang	0	9.725	14	1	1	1	159	17.4	6.14	5.27	1	regulation	cytosol;en	protein bir	Pf06396	57085	ENSG0000	AGTRAP	1			Signaling b	4	1.025	0.390269683	5.84	136.1	132	3.95	1.25	High	High	High	High	High	High	1	
FALSE	High	Master Prc	Q99755	Phosphatic	0	8.521	6	2	2	2	562	62.6	8.21	4.87	2	cell differe	cytoplasm;	catalytic a	Pf01504	8394	ENSG0000	PIP5K1A	1	Metabolic	B Cell Rece	Synthesis c	20	1.025	0.45351789	14.64	767.3	748.9	45.53	1.89	High	High	High	High	High	High	1	[Q368];
FALSE	High	Master Prc	P99999	cytochrom	0	49.35	63	9	18	9	105	11.7	9.57	34.68	9	cell death;	cytosol;me	catalytic a	Pf00034, P	54205	ENSG0000	CYCS	7	Hepatitis E	miRNA Reg	Transcripti	54	1.025	0.511389868	0.1	2727.1	2755.5	15.68	6.12	High	High	High	High	High	High	1	
FALSE	High	Master Prc	Q9Y3A5	Ribosome	0	16.075	14	3	8	3	250	28.7	8.75	10.9	3	cell organi:	cytoplasm;	protein bir	Pf01172, P	51119	ENSG0000	SBDS	7	Ribosome			1	1.025	0.551901532	1.76	573.3	639.1	12.08	11.21	High	High	High	High	High	High	1	
FALSE	High	Master Prc	Q9NNW7-	thioredoxi	0	12.381	6	3	4	2	524	56.5	7.5	8.15	3	cellular ho	cytosol;mi	antioxidan	Pf00070, P	10587	ENSG0000	TXNRD2	22	Pyrimidine	Selenium M	Detoxificat	8	1.025	0.630043126	2.91	311.7	310.7	13.88	10.8	High	High	High	High	High	High	1	
FALSE	High	Master Prc	O60664-1	Perilipin-3	0	164.074	69	22	49	22	434	47	5.44	150.63	22	transport	cytoplasm;	protein bir	Pf03036	10226	ENSG0000	PLIN3	19			Triglycerid	8	1.025	0.631247176	10.85	16189.6	15853.8	4.56	4.67	High	High	High	High	High	High	1	
FALSE	High	Master Prc	Q9UKM7	endoplasm	0.003	3.474	2	1	1	1	699	79.5	7.72	2.37	1	metabolic	endoplasm	catalytic a	Pf01532	11253	ENSG0000	MAN1B1	9	Metabolic		ER Quality	13	1.025	0.645871451	28.77	344	380.9	27.82	20.11	High	High	High	High	High	High	1	
FALSE	High	Master Prc	Q9NZ53-1	Podocalyxi	0	5.623	2	1	4	1	605	65	4.34	6.71	1	metabolic	membrane	protein bir	Pf06365	50512	ENSG0000	PODXL2	3			Cytosolic s	4	1.025	0.690835127	39.24	164.7	163.5	21.8	24.42	High	High	High	High	High	High	1	
FALSE	Medium	Master Prc	Q9H0K6	Pseudouric	0.037	1.326	1	1	1	1	701	80.6	7.56	0	1	metabolic		catalytic a	Pf01142	83448	ENSG0000	PUS7L	12				0	1.025	0.955040829	28.5	545.1	544.1	15.07	11.07	High	High	High	High	High	High	1	
FALSE	High	Master Prc	Q07021	Compleme	0	59.678	36	8	17	8	282	31.3	4.84	56.8	8	cell death;	cell surface	protein bir	Pf02330	708	ENSG0000	C1QBP	17	Herpes sim	Mesoderm	Intrinsic Pa	5	1.025	0.973571473	45.29	5147.6	5024.3	17.64	15.41	High	High	High	High	High	High	1	
FALSE	High	Master Prc	Q5RKV6	Exosome c	0	16.329	28	5	5	5	272	28.2	6.28	9.93	5	metabolic	cytoplasm;	catalytic a	Pf01138	118460	ENSG0000	EXOSC6	16	RNA degra		Major path	15	1.025	0.979004286	14.9	614.7	599.6	6.83	6.87	High	High	High	High	High	High	1	
FALSE	High	Master Prc	P54652	Heat shock	0	149.277	26	18	106	2	639	70	5.74	245.86	18	cell differe	cell surface	nucleotide	Pf00012, P	3306	ENSG0000	HSPA2	14	Protein pro	Parkin-Ubi	Meiotic syn	24	1.024	0.305127756	1.39	63.9	60.8	3.61	5.75	High	High	High	High	High	High	1	[Y184(100
FALSE	High	Master Prc	Q9NVN8	Guanine nu	0	28.035	15	7	7	7	582	65.5	8.44	17.31	7	regulation	cytosol;me	catalytic a	Pf01926, P	54552	ENSG0000	GNL3L	X	Ribosome			1	1.024	0.411611962	5.57	1229.9	1201.1	16.91	7.12	High	High	High	High	High	High	1	
FALSE	High	Master Prc	Q9H3K6-1	bola-like p	0	13.693	49	3	5	3	86	10.1	6.52	14.48	3	cell organi:	cytoplasm;	protein bir	Pf01722	654483; 5!		BOLA2B; B	16			Gene and p	5	1.024	0.458138415	7.13	731.2	713.8	7.08	1.43	High	High	High	High	High	High	1	
FALSE	High	Master Prc	Q14232	Translation	0	12.539	20	4	5	4	305	33.7	7.33	10.21	4	metabolic	cytoplasm;	protein bir	Pf01008	1967	ENSG0000	EIF2B1	12	RNA transp	Translation	Recycling c	7	1.024	0.526319495	21.67	1502.5	1535.5	37.76	5.31	High	High	High	High	High	High	1	
FALSE	High	Master Prc	Q9BUJ2-1	Heterogen	0	47.077	15	11	16	11	856	95.7	6.92	33.81	11	metabolic	nucleus	protein bir	Pf00622, P	11100	ENSG0000	HNRNPUL1	19	Influenza A		mRNA Spli	5	1.024	0.609618347	3.5	3332	3255	4.69	8.01	High	High	High	High	High	High	1	
FALSE	High	Master Prc	P26368	Splicing fac	0	50.712	26	9	19	9	475	53.5	9.09	44.74	9	metabolic	nucleus;sp	protein bir	Pf00076, P	11338	ENSG0000	U2AF2	19	Spliceosom	mRNA Pro	Cleavage o	13	1.024	0.710016175	13.09	7156.1	6975.5	5.87	5.36	High	High	High	High	High	High	1	
FALSE	High	Master Prc	P20339	Ras-related	0	43.779	48	7	14	5	215	23.6	8.15	39.97	7	cell organi:	cytoplasm;	catalytic a	Pf00009, P	5868	ENSG0000	RAB5A	3	Amyotroph	Amyotroph	TBC/RABG	26	1.024	0.784636708	29.13	2160.7	2110.7	25	8.27	High	High	High	High	High	High	1	
FALSE	High	Master Prc	O00429	Dynamin-1	0	97.538	31	15	24	15	736	81.8	6.81	79.86	15	cell death;	cytoplasm;	catalytic a	Pf00350, P	10059	ENSG0000	DNM1L	12	TNF signali	Synaptic V	Apoptotic	6	1.024	0.8415373	0.33	2960.3	2890.5	7.5	4.43	High	High	High	High	High	High	1	
FALSE	High	Master Prc	Q10469	Alpha-1,6-	0	16.435	12	3	3	3	447	51.5	8.76	10.25	3	metabolic	Golgi;mem	catalytic a	Pf05060	4247	ENSG0000	MGAT2	14	N-Glycan b		Defective I	12	1.023	0.362834304	2.71	814.1	796	10.98	1.69	High	High	High	High	High	High	1	
FALSE	High	Master Prc	O00592	Podocalyxi	0	29.389	11	6	16	6	558	58.6	5.49	28.03	6	cellular co	cytoplasm;	protein bir	Pf05109, P	5420	ENSG0000	PODXL	7		Primary Fo		3	1.023	0.379825113	1.69	4250.2	4153.3	4.16	6.54	High	High	High	High	High	High	1	
FALSE	High	Master Prc	Q9Y6E0	Serine/thre	0	67.083	34	14	20	8	443	49.3	5.69	42.31	14	cell commu	cytoplasm;	catalytic a	Pf00069, P	8428	ENSG0000	STK24	13			Apoptotic	4	1.023	0.403902121	5.33	4954.2	4768.7	1	4.08	High	High	High	High	High	High	1	
FALSE	High	Master Prc	P21953	2-oxoisova	0.002	3.729	3	1	1	1	392	43.1	6.29	2.78	1	metabolic	mitochond	catalytic a	Pf02779, P	594	ENSG0000	BCKDHB	6	Propanoat		Branched-c	7	1.023	0.430201536	6.76	221.7	203.5	13.17	8.5	High	High	High	High	High	High	1	
FALSE	High	Master Prc	Q04726	Transducin	0	45.613	17	9	12	7	772	83.4	7.2	25.25	9	metabolic	nucleus	protein bir	Pf00400, P	7090	ENSG0000	TLE3	15		White fat c	Repression	8	1.023	0.47913413	46.85	478.5	455.3	103.5	25.69	High	High	High	High	High	High	1	
FALSE	High	Master Prc	Q14444-1	Caprin-1 [(	0	92.076	24	14	46	14	709	78.3	5.25	117.75	14	cell differe	cytoplasm;	protein bir	Pf12287	4076	ENSG0000	CAPRIN1	11				0	1.023	0.538860309	12.59	14040.6	13687.6	8.86	8.88	High	High	High	High	High	High	1	
FALSE	High	Master Prc	O75369-8	Isoform 8 c	0	592.965	43	89	202	83	2633	281.5	5.71	542.36	89	cell organi:	cytoplasm;	protein bir	Pf00307, P	2317	ENSG0000	FLNB	3		VEGFA-VEG		1	1.023	0.557952554	10.67	60705	59313.8	7.2	2.89	High	High	High	High	High	High	1	
FALSE	High	Master Prc	P46087-2	Isoform 2 c	0	89.444	25	17	32	1	808	88.9	9.23	68.64	17	cell organi:		catalytic a	Pf01189, P	4839	ENSG0000	NOP2	12		Imatinib ai		1	1.023	0.619959242	14.75	3506.8	3143.5	11.39	6.99	High	High	High	High	High	High	1	
FALSE	High	Master Prc	Q92769	Histone de	0	19.746	13	3	4	2	488	55.3	5.91	12.15	3	cell differe	chromosor	catalytic a	Pf00850	3066	ENSG0000	HDAC2	6	Thyroid ho	Cell Cycle;	NOTCH1 Ir	59	1.023	0.648405505	22.83	345.9	368	29.88	12.94	High	High	High	High	High	High	1	
FALSE	High	Master Prc	Q9H6T3	RNA polym	0	42.019	18	10	14	10	665	75.7	6.84	24.7	10			protein bir	Pf00515, P	79657	ENSG0000	RPAP3	12				0	1.023	0.661705871	12.74	7581.2	7563.2	8.34	3.16	High	High	High	High	High	High	1	
FALSE	High	Master Prc	Q12959	Disks large	0	6.966	3	2	3	2	904	100.4	5.76	9.26	2	cell commu	cytoplasm;	catalytic a	Pf00018, P	1739	ENSG0000	DLG1	3	T cell recep	Brain-Deri	NrCAM int	20	1.023	0.713973868	18.56	2048.9	2064.8	13.92	2.82	High	High	High	High	High	High	1	
FALSE	High	Master Prc	P30048	Thioredoxi	0	90.989	49	11	35	11	256	27.7	7.78	112.43	11	cell death;	cytoplasm;	antioxidan	Pf00578, P	10935	ENSG0000	PRDX3	10		Selenium M	Detoxificat	4	1.023	0.76017904	23.75	14590	14262.9	15.77	12.82	High	High	High	High	High	High	1	
FALSE	High	Master Prc	O75400	pre-mRNA	0	32.145	11	7	11	7	957	108.7	7.56	17.6	7	cell divisio	cytosol;me	protein bir	Pf00397, P	55660		PRPF40A	2	Spliceosom	mRNA Pro	mRNA Spli	6	1.023	0.853196366	3.38	1731.6	1821.9	5.95	4.5	High	High	High	High	High	High	1	
FALSE	High	Master Prc	Q14240-1	eukaryotic	0	75.653	29	11	60	2	407	46.4	5.48	166.18	11	metabolic	cytosol	catalytic a	Pf00270, P	1974	ENSG0000	EIF4A2	3	RNA transp	Translation	ISG15 anti	19	1.022	0.325313542	3.97	610	566.7	3.78	5.79	High	High	High	High	High	High	1	
FALSE	High	Master Prc	O94973	AP-2 comp	0	54.557	17	11	13	6	939	103.9	6.96	38.98	11	cell organi:	cytosol;me	protein bir	Pf01602, P	161	ENSG0000	AP2A2	11	Endocrine	Synaptic V	Cargo reco	43	1.022	0.558437245	9.42	1321.3	1266.9	6.14	5.26	High	High	High	High	High	High	1	
FALSE	High	Master Prc	Q9H444	Charged m	0	29.242	34	6	13	6	224	24.9	4.82	19.11	6	cell commu	cytoplasm;	protein bir	Pf03357	128866	ENSG0000	CHMP4B	20	Endocytosi		Budding ar	12	1.022	0.580328239	6.94	4258.7	4081.5	1.32	5.45	High	High	High	High	High	High	1	
FALSE	High	Master Prc	O95900	Mitochond	0	8.36	6	2	4	2	331	36.7	8.98	5.89	2	metabolic	cytosol;mi	catalytic a	Pf01509	26995	ENSG0000	TRUB2	9				0	1.022	0.587764887	37.75	185.1	181.1	45.86	14.56	High	High	High	High	High	High	1	
FALSE	High	Master Prc	Q96EY8	Cob(I)yrini	0	13.943	18	3	4	3	250	27.4	8.6	15.14	3	metabolic	mitochond	catalytic a	Pf01923	326625	ENSG0000	MMAB	12	Metabolic	Vitamin B1	Cobalamin	12	1.022	0.818920955	21.44	450.4	481.6	20.38	8.03	High	High	High	High	High	High	1	
FALSE	High	Master Prc	P53990-1	IST1 homo	0	16.109	11	3	4	3	364	39.7	5.35	14.57	3	cell divisio	chromosor	protein bir	Pf03398	9798		IST1	16	Endocytosi		Neutrophil	4	1.022	0.8275082	8.62	946.7	926	4.59	7.31	High	High	High	High	High	High	1	
FALSE	High	Master Prc	Q9BYX2	TBC1 doma	0	6.128	3	2	2	2	928	105.3	6.58	1.68	2	regulation	cytoplasm;	enzyme re	Pf00169, P	55357	ENSG0000	TBC1D2	9			TBC/RABG	4	1.022	0.976959624	16.88	444.2	449.5	10.21	7.11	High	High	High	High	High	High	1	
FALSE	High	Master Prc	Q8N6N3	UPF0690 p	0.005	2.564	5	1	2	1	182	20.6	5.21	0	1		nucleus	RNA bindir	Pf15559	148423	ENSG0000	C1orf52	1				0	1.021	0.197695362	1.96	978.8	936.6	2.43	2.4	High	High	High	High	High	High	1	
FALSE	High	Master Prc	Q9Y696	Chloride in	0	39.47	42	6	8	6	253	28.8	5.59	28.01	6	cell differe	cell surface	catalytic a	Pf00043, P	25932	ENSG0000	CLIC4	1				0	1.021	0.411827069	5.24	2512.6	2449.1	2.49	3.17	High	High	High	High	High	High	1	
FALSE	High	Master Prc	Q92878	DNA repair	0	41.849	10	10	10	10	1312	153.8	6.89	27.93	10	cell organi:	chromosor	catalytic a	Pf02463, P	10111	ENSG0000	RAD50	5	Homologo	miRNA Reg	G2/M DNA	42	1.021	0.422397833	6.06	479.4	467	50.99	0.44	High	High	High	High	High	High	1	
FALSE	High	Master Prc	Q14108-1	Lysosome i	0	6.333	4	1	2	1	478	54.3	5.14	8.56	1	cell organi:	membrane	protein bir	Pf01130	950	ENSG0000	SCARB2	4	Lysosome	Primary Fo	Cargo reco	6	1.021	0.507174773	47.52	208	242.2	88.98	16.47	High	High	High	High	High	High	1	
FALSE	High	Master Prc	Q5GLZ8-1	Probable E	0	24.028	7	5	5	5	1057	118.5	6.19	12.57	5	cell differe	cytoplasm;	catalytic a	Pf00415, P	26091	ENSG0000	HERC4	10	Ubiquitin r		Antigen pr	5	1.021	0.510655679	18.66	581.5	569.5	29.69	6.09	High	High	High	High	High	High	1	
FALSE	High	Master Prc	Q08043	Alpha-actir	0	41.015	7	6	20	1	901	103.2	5.52	35.32	6	cell organi:	cytosol	metal ion l	Pf00307, P	89	ENSG0000	ACTN3	11	Adherens j	Arrhythmo	Nephrin fa	15	1.021	0.556137845	11.77	1477.4	1256.6	8.74	19.01	High	High	High	High	High	High	1	[Q748;
FALSE	High	Master Prc	O00264	Membrane	0	19.257	27	5	11	5	195	21.7	4.7	28.07	5	transport	endoplasm	protein bir	Pf00173	10857	ENSG0000	PGRMC1	X			Neutrophil	3	1.021	0.854815163	17.66	4088.3	4003.8	3.71	16.66	High	High	High	High	High	High	1	
FALSE	High	Master Prc	P48960-1	CD97 antig	0	9.97	2	1	1	1	835	91.8	6.87	5.77	1	cell commu	cell surface	metal ion l	Pf00001, P	976	ENSG0000	CD97; ADG	19		GPCRs, Cla	Neutrophil	8	1.021	0.863347236	17.29	623.9	611.1	9.46	9.27	High	High	High	High	High	High	1	
FALSE	High	Master Prc	Q12789-2	General tra	0.002	3.772	1	1	1	1	2109	238.7	7.3	3.97	1	metabolic	membrane	DNA bindi	Pf04182	2975	ENSG0000	GTF3C1	16			RNA Polym	6	1.021	0.882370963	32.8	324.8	318	14.57	14.63	High	High	High	High	High	High	1	
FALSE	High	Master Prc	Q9BV44	THUMP do	0	13.921	13	4	4	4	507	57	6.37	6.18	4	metabolic	cytosol	catalytic a	Pf01170, P	25917	ENSG0000	THUMPD3	3				0	1.021	0.954756987	41.45	37	36.9	17.82	17.5	High	High	High	High	High	High	1	
FALSE	Medium	Master Prc	Q92673	Sortilin-rel	0.039	1.29	1	1	1	1	2214	248.3	5.55	3.14	1	metabolic	endoplasm	protein bir	Pf00041, P	6653	ENSG0000	SORL1	11			Amyloid fil	2	1.02	0.580162265	5.84	44.3	47.6	12.24	20.49	High	High	High	High	High	High	1	
FALSE	High	Master Prc	P63000-1	Ras-related	0	31.57	35	8	21	7	192	21.4	8.5	38.77	8	cell organi:	cytoplasm;	catalytic a	Pf00025, P	5879	ENSG0000	RAC1	7	Colorectal	Androgen	SEMA3A-P	160	1.02	0.607838718	16.16	489.6	443	14.75	16.77	High	High	High	High	High	High	1	
FALSE	High	Master Prc	O95336	6-phospho	0	27.338	28	5	7	5	258	27.5	6.05	12.47	5	metabolic	cytoplasm;	catalytic a	Pf01182	25796	ENSG0000	PGLS	19	Carbon me	Pentose Ph	Pentose ph	7	1.02	0.870608341	3.82	1561.4	1492.9	3.06	6.69	High	High	High	High	High	High	1	
FALSE	High	Master Prc	P62993	Growth fac	0	7.234	6	1	1	1	217	25.2	6.32	3.33	1	cell commu	cytoplasm;	catalytic a	Pf00017, P	2885	ENSG0000	GRB2	17	Osteoclast	IL-5 Signali	PI3K Casca	223	1.02	0.935865688	8.13	605.5	593.5	3.31	4.81	High	High	High	High	High	High	1	
FALSE	High	Master Prc	O75718	Cartilage-a	0	15.245	14	5	7	5	401	46.5	5.73	15.7	5	metabolic	endoplasm	protein bir		10491	ENSG0000	CRTAP	3			Collagen b	3	1.019	0.410249921	3.48	487.7	448.2	22.95	7.4	High	High	High	High	High	High	1	
FALSE	High	Master Prc	P48729-1	Casein kina	0	21.41	14	3	7	1	337	38.9	9.57	27.3	3	cell divisio	chromosor	catalytic a	Pf00069, P	1452	ENSG0000	CSNK1A1	5	Wnt signal	T-Cell Rece	S37 mutan	34	1.019	0.475987115	11.91	86.1	84.5	22.08	8.48	High	High	High	High	High	High	1	
FALSE	High	Master Prc	Q99733	Nucleosom	0	74.272	27	9	25	7	375	42.8	4.69	73.16	9	cell organi:	cytoplasm;	protein bir	Pf00956	4676	ENSG0000	NAP1L4	11; CHR_H				0	1.019	0.492646634	1.48	8815.9	8821.4	19.59	6.3	High	High	High	High	High	High	1	
FALSE	High	Master Prc	Q13601	KRR1 smal	0	7.616	6	2	3	2	381	43.6	9.77	6.73	2	metabolic	cytoplasm;	protein bir	Pf00013	11103	ENSG0000	KRR1	12			Major path	5	1.019	0.52186351	1.78	1087.6	1113.7	12.09	7.95	High	High	High	High	High	High	1	
FALSE	High	Master Prc	Q6ZYL4	general tra	0.002	3.771	15	1	1	1	71	8	4.65	2.12	1	cell organi:	nucleus	DNA bindi	Pf06331	404672	ENSG0000	GTF2H5	6	Basal trans		Gap-filling	50	1.019	0.52910615	41.88	311.6	339.5	71.08	13.67	High	High	High	High	High	High	1	
FALSE	High	Master Prc	Q7Z417	Nuclear fra	0	56.59	20	10	17	10	695	76.1	8.7	30.54	10		cytoplasm;	protein bir	Pf15293	57532	ENSG0000	NUFIP2	17				0	1.019	0.610658144	18.03	3276.8	3214.4	13.12	8.6	High	High	High	High	High	High	1	
FALSE	High	Master Prc	P62318	small nucle	0	25.774	32	3	6	3	126	13.9	10.32	24.44	3	cell organi:	cytoplasm;	protein bir	Pf01423	6634	ENSG0000	SNRPD3	22	Spliceosom	mRNA Pro	SLBP indep	17	1.019	0.741362081	14.46	1914.2	1951.9	14.61	7.67	High	High	High	High	High	High	1	
FALSE	High	Master Prc	Q12792-2	Twinfilin-1	0	31.511	28	8	13	6	350	40.3	6.96	26.76	8	cell organi:	cytoplasm;	catalytic a	Pf00241	5756	ENSG0000	TWF1	12				0	1.019	0.878950337	26.05	861.5	860.8	14.62	5.92	High	High	High	High	High	High	1	
FALSE	High	Master Prc	P13010	X-ray repai	0	213.676	42	26	62	26	732	82.7	5.81	167.06	26	cell differe	chromosor	catalytic a	Pf02735, P	7520	ENSG0000	XRCC5	2	Non-homo	Non-homo	2-LTR circl	18	1.019	0.890909576	9.52	19009.6	17908.4	11.26	3.03	High	High	High	High	High	High	1	
FALSE	High	Master Prc	P15924-1	Desmoplak	0	105.639	10	22	30	22	2871	331.6	6.81	60.63	22	cell death;	cytoplasm;	catalytic a	Pf00038, P	1832	ENSG0000	DSP	6	Arrhythmo	Arrhythmo	Apoptotic	14	1.019	0.8926194	23.09	4356.9	4275.5	12.89	3.23	High	High	High	High	High	High	1	
FALSE	High	Master Prc	P82094	TATA elem	0	29.851	7	7	9	7	1093	122.8	4.92	16.16	7	cell differe	cytoplasm;	DNA bindi	Pf12325, P	7110	ENSG0000	TMF1	3			Retrograde	4	1.019	0.946516487	17.86	708.6	695.6	6.37	8.1	High	High	High	High	High	High	1	
FALSE	High	Master Prc	Q9BQ61	Uncharact	0	7.674	8	1	2	1	176	18.4	9.44	3.74	1			protein bir		79002	ENSG0000	C19orf43;	19				0	1.019	0.974943118	10.71	785	778.6	3.66	7.05	High	High	High	High	High	High	1	
FALSE	High	Master Prc	O95202-1	mitochond	0	67.426	28	16	21	16	739	83.3	6.7	51.9	16	cell organi:	membrane	metal ion l	Pf07766, P	3954	ENSG0000	LETM1	4			Mitochonc	2	1.018	0.285302694	1	3723.9	3741.2	4.49	4.39	High	High	High	High	High	High	1	
FALSE	High	Master Prc	Q5QJE6	Deoxynucl	0	29.021	14	7	7	7	756	84.4	6.16	17.22	7	metabolic	nucleus	RNA bindir	Pf08698	30836	ENSG0000	DNTTIP2	1				0	1.018	0.402879027	2.82	1621.1	1527.7	19.96	4.84	High	High	High	High	High	High	1	
FALSE	High	Master Prc	Q10472	Polypeptid	0	13.236	7	3	3	3	559	64.2	7.72	5.02	3	metabolic	extracellul	catalytic a	Pf00535, P	2589	ENSG0000	GALNT1	18	Metabolic		COPI-indep	11	1.018	0.405516082	3.18	62.2	56.4	16.54	10.59	High	High	High	High	High	High	1	
FALSE	High	Master Prc	Q9UBI6	guanine nu	0	14.648	46	3	4	3	72	8	8.97	10.9	3	regulation	membrane	protein bir	Pf00631	55970	ENSG0000	GNG12	1	Circadian e	Myometria	G alpha (z)	76	1.018	0.438570616	5.99	1237.4	1200.1	11.68	4.55	High	High	High	High	High	High	1	
FALSE	High	Master Prc	Q05655	protein kin	0	9.937	4	2	2	2	676	77.5	7.75	6.8	2	cell death;	cytoplasm;	catalytic a	Pf00069, P	5580	ENSG0000	PRKCD	3	Type II dial	Type II dial	G alpha (z)	74	1.018	0.44336287	8.67	174	160.6	36	7.54	High	High	High	High	High	High	1	
FALSE	High	Master Prc	Q9BXR0	Queuine tR	0	10.586	9	3	4	3	403	44	7.23	6.41	3	metabolic	cytoplasm;	catalytic a	Pf01702	81890	ENSG0000	QTRT1	19			tRNA modi	3	1.018	0.462001964	9.18	799	764.2	20.68	2.74	High	High	High	High	High	High	1	
FALSE	High	Master Prc	Q9NQZ5	StAR-relate	0	17.946	15	4	4	4	370	43.1	8.95	7.52	4	metabolic	membrane		Pf01852	56910	ENSG0000	STARD7	2			Synthesis c	5	1.018	0.501866178	16.19	185.1	181.7	30.26	2.45	High	High	High	High	High	High	1	
FALSE	High	Master Prc	Q10570	Cleavage a	0	8.633	2	2	3	2	1443	160.8	6.4	6.46	2	metabolic	nucleus	protein bir	Pf03178, P	29894	ENSG0000	CPSF1	8	mRNA surv	mRNA Pro	mRNA 3'-e	18	1.018	0.516669097	38.83	335	329	66.88	21.95	High	High	High	High	High	High	1	
FALSE	High	Master Prc	P28799	Granulins [	0	9.468	4	3	5	3	593	63.5	6.83	4.23	3	regulation	endoplasm	protein bir	Pf00396	2896	ENSG0000	GRN	17		Integrated	Neutrophil	4	1.018	0.533096906	15.07	259.4	254.7	23.21	1.27	High	High	High	High	High	High	1	

FALSE	High	Master Prc	Q9BYD3	39S riboso	0	15.542	15	3	4	3	311	34.9	9.72	8.27	3	cell organi	membrane	protein bir	Pf00573	51073	ENSG0000	MRPL4	19	Ribosome		Mitochonc	7	1.018	0.538440513	0.2	906	1037.4	9.16	10.35	High	High	High	High	High	High	1	
FALSE	High	Master Prc	P07437	tubulin bet	0	404.89	83	29	233	5	444	49.6	4.89	644.41	29	cell divisio	cytoplasm;	catalytic a	Pf00091, P	203068	ENSG0000	TUBB	6; CHR_HS	Gap juncti	Pathogeni	Neutrophil	24	1.018	0.593524401	1.94	14788.6	14332.8	16.71	4.47	High	High	High	High	High	High	1	[T290(100
FALSE	High	Master Prc	P22102-1	trifunction	0	162.107	38	28	42	28	1010	107.7	6.7	134.32	28	metabolic	cytoplasm;	catalytic a	Pf00551, P	2618	ENSG0000	GART	21	Purine met	Folate Met	Purine ribc	10	1.018	0.68441447	1.13	8327.7	8234.5	7.55	1.01	High	High	High	High	High	High	1	
FALSE	High	Master Prc	Q96IJ6	Mannose-1	0	7.255	6	2	2	2	420	46.3	7.21	5.78	2	metabolic	cytoplasm	catalytic a	Pf00132, P	29926	ENSG0000	GMPPA	2	Fructose ai		Synthesis c	9	1.018	0.725294599	27.33	185.8	210.1	26.85	11.45	High	High	High	High	High	High	1	
FALSE	High	Master Prc	Q15024	Exosome c	0	10.73	12	3	3	3	291	31.8	5.19	6.33	3	metabolic	cytoplasm;	catalytic a	Pf01138, P	23016	ENSG0000	EXOSC7	3	RNA degra		Major patl	15	1.018	0.813260356	16.68	618	607.1	14.49	4.63	High	High	High	High	High	High	1	
FALSE	High	Master Prc	Q8TCT9-1	Minor hist	0	42.292	17	6	19	6	377	41.5	6.43	46.8	6	cell organi	cell surface	catalytic a	Pf04258, P	81502	ENSG0000	HM13	20				0	1.018	0.957391581	14.89	6289.1	6180.7	9.83	2.31	High	High	High	High	High	High	1	
FALSE	High	Master Prc	P12814-1	Alpha-acti	0	163.29	38	27	97	14	892	103	5.41	226.97	27	cell differe	cytoplasm;	metal ion l	Pf00036, P	87	ENSG0000	ACTN1	14	Regulation	Arrhythmc	Platelet de	24	1.017	0.131267345	1.29	7121.1	6943.2	3.32	2.72	High	High	High	High	High	High	1	[Q747]
FALSE	High	Master Prc	P49589-1	Cysteine--t	0	31.351	11	8	10	8	748	85.4	6.76	29.94	8	metabolic	cytoplasm;	catalytic a	Pf01406, P	833	ENSG0000	CARS	11; CHR_H	Aminoacyl		Cytosolic t	5	1.017	0.466014488	7.82	569.4	547.4	16	4.59	High	High	High	High	High	High	1	
FALSE	High	Master Prc	P46777	60S riboso	0	105.289	46	16	52	16	297	34.3	9.72	117.87	16	cell organi	cytoplasm;	protein bir	Pf00861, P	6125	ENSG0000	RPL5	1	Ribosome	Cytoplasm	Peptide ch	34	1.017	0.503659808	5.94	16832.2	15893.4	3.59	4.78	High	High	High	High	High	High	1	
FALSE	High	Master Prc	Q6Y7W6-1	GRB10-inte	0	35.424	7	7	8	7	1299	150	5.54	21.93	7	cell differe	cytoplasm;	protein bir	Pf02213	26058	ENSG0000	GIGYF2	2				0	1.017	0.534881402	36.3	707.1	695.2	57.07	15.11	High	High	High	High	High	High	1	
FALSE	High	Master Prc	P51452	dual specif	0	23.279	24	3	7	3	185	20.5	7.8	19.8	3	metabolic	cytosol;nu	catalytic a	Pf00102, P	1845	ENSG0000	DUSP3	17	MAPK sign		ERKs are ir	32	1.017	0.56709977	16.59	988	971.5	19.95	5.95	High	High	High	High	High	High	1	
FALSE	High	Master Prc	Q9BYN8	28S riboso	0	10.652	18	3	3	3	205	24.2	10.39	4.66	3	cell organi	membrane	RNA bindi	Pf14943	64949	ENSG0000	MRPS26	20			Mitochonc	6	1.017	0.573948654	23.56	188.5	190.7	29.2	20.11	High	High	High	High	High	High	1	
FALSE	High	Master Prc	Q96CS3	FAS-associ	0	32.059	19	6	9	6	445	52.6	5.62	26.63	6	cell organi	cytoplasm;	enzyme re	Pf00789, P	23197	ENSG0000	FAF2	5			Neutrophil	3	1.017	0.575318211	24.85	1056.6	1140.9	35.44	8.56	High	High	High	High	High	High	1	
FALSE	High	Master Prc	P50416	Carnitine C	0	48.687	17	12	18	12	773	88.3	8.65	37.16	12	cell differe	membrane	catalytic a	Pf00755	1374	ENSG0000	CPT1A	11	Insulin resi	AMP-activ	RORA activ	25	1.017	0.579269069	4.26	3752.8	3666.2	5.44	2.17	High	High	High	High	High	High	1	
FALSE	High	Master Prc	P50502	Hsc70-inte	0	73.131	30	12	31	12	369	41.3	5.27	66.46	12	cell organi	cytoplasm;	nucleotide	Pf00515, P	6767	ENSG0000	ST13	22			Regulation	4	1.017	0.603399819	4.3	16885.3	16267.6	16.68	1.84	High	High	High	High	High	High	1	
FALSE	High	Master Prc	P61326	Protein ma	0	26.234	47	6	9	6	146	17.2	6.11	20.25	6	metabolic	cytoplasm;	protein bir	Pf02792	4116	ENSG0000	MAGOH	1	RNA trans		mRNA 3'-e	20	1.017	0.646337889	8.47	1267.4	1321.3	5	8.42	High	High	High	High	High	High	1	
FALSE	High	Master Prc	O75792	Ribonuclea	0	12.932	12	2	3	2	299	33.4	5.25	5.3	2	metabolic	cytosol;nu	catalytic a	Pf01351	10535	ENSG0000	RNASEH2A	19	DNA replic			1	1.017	0.733408945	18.21	329.4	339.5	14.37	4.49	High	High	High	High	High	High	1	
FALSE	High	Master Prc	Q6PD62	RNA polym	0	20.154	5	5	6	5	1173	133.4	6.77	10.21	5	cell differe	nucleus	protein bir	Pf08424, P	9646	ENSG0000	CTR9	11		Endoderm	E3 ubiquiti	10	1.017	0.83493041	20.73	1377.3	1354	13.55	1.22	High	High	High	High	High	High	1	
FALSE	High	Master Prc	Q07812	Apoptosis	0	6.344	6	1	3	1	192	21.2	5.22	4.45	1	cell death;	cytoplasm;	protein bir	Pf00452	581	ENSG0000	BAX	19	Viral carcir	miRNA Re	TP53 Regu	49	1.017	0.923737721	11.49	757.5	731.6	8.57	2.06	High	High	High	High	High	High	1	
FALSE	High	Master Prc	P52434	DNA-direct	0	8.817	22	4	4	4	150	17.1	4.68	4.42	4	metabolic	cytosol;nu	catalytic a	Pf03870	5437	ENSG0000	POLR2H	3	Purine met	Eukaryotic	Signaling b	98	1.017	0.972434759	12.65	1354.8	1334	5.44	7.69	High	High	High	High	High	High	1	
FALSE	High	Master Prc	P22059	Oxysterol-l	0	31.33	12	6	8	6	807	89.4	7.3	17.41	6	metabolic	cytoplasm;	protein bir	Pf00169, P	5007	ENSG0000	OSBP	11			Synthesis c	7	1.016	0.37123808	3.29	992	963.9	2.19	2.36	High	High	High	High	High	High	1	
FALSE	High	Master Prc	Q9BUQ8	Probable A	0	42.072	16	11	13	11	820	95.5	9.55	13.37	11	cell organi	cytoplasm;	catalytic a	Pf00270, P	9416	ENSG0000	DDX23	12	Spliceosom		mRNA Spli	6	1.016	0.506605449	4.87	407.4	440.8	14.86	15.67	High	High	High	High	High	High	1	
FALSE	High	Master Prc	Q7Z5K2-1	Wings apa	0	9.933	2	2	2	2	1190	132.9	5.44	5.97	2	cell divisio	chromosor	protein bir	Pf07814	23063	ENSG0000	WAPAL; W	10			Cohesin Lo	12	1.016	0.566035582	17.7	927	912	19.11	8.85	High	High	High	High	High	High	1	
FALSE	High	Master Prc	Q8TF05	Serine/thre	0	5.553	2	2	2	2	950	106.9	4.77	3.74	2	metabolic		catalytic a		9989	ENSG0000	PPP4R1	18				0	1.016	0.566179677	3.31	257.1	286.4	7.56	12.69	High	High	High	High	High	High	1	
FALSE	High	Master Prc	O00291	Huntingtin	0	19.358	7	6	7	6	1037	116.1	5.3	9.76	6	cell death;	cytoplasm;	catalytic a	Pf00038, P	3092	ENSG0000	HIP1	7	Huntingtoi		Clathrin-m	4	1.016	0.572369582	2.48	992.2	1046.2	10.18	5.43	High	High	High	High	High	High	1	[Q51;
FALSE	High	Master Prc	O60888-1	Protein Cu	0	8.708	18	2	3	2	179	19.1	5.5	5.87	2	response t	membrane	metal ion l	Pf03091	51596	ENSG0000	CUTA	6; CHR_HS				0	1.016	0.583956202	5.87	874	860.4	4.8	9.13	High	High	High	High	High	High	1	
FALSE	High	Master Prc	Q75N03	E3 ubiquiti	0	5.799	4	1	1	1	491	54.5	8.29	3.54	1	metabolic	cytosol;nu	catalytic a	Pf00097, P	79872	ENSG0000	CBLL1	7			InlA-media	4	1.016	0.591565296	10.84	423.9	423.3	11.75	1.11	High	High	High	High	High	High	1	
FALSE	High	Master Prc	P38435-1	vitamin K-c	0.003	2.999	2	1	1	1	758	87.5	8.02	2.57	1	coagulatio	endoplasm	catalytic a	Pf05090	2677	ENSG0000	GGCX	2	Ubiquinon		Gamma-ca	6	1.016	0.59957749	1.29	618.5	635.1	11.46	4.83	High	High	High	High	High	High	1	
FALSE	High	Master Prc	O15371	Eukaryotic	0	27.594	14	8	10	8	548	63.9	6.05	24.94	8	cell organi	cytoplasm;	protein bir	Pf05091	8664	ENSG0000	EIF3D	22	RNA trans	Translatior	Formation	13	1.016	0.652773021	1.77	1865	1871.5	2.3	7.5	High	High	High	High	High	High	1	
FALSE	High	Master Prc	P30825	High affini	0	14.691	7	3	6	3	629	67.6	5.43	16.42	3	transport	membrane	protein bir	Pf00324, P	6541	ENSG0000	SLC7A1	13	MicroRNA:		Amino acic	5	1.016	0.653915797	15.8	709.3	701.9	15.88	10.17	High	High	High	High	High	High	1	
FALSE	High	Master Prc	Q5TFE4	5'-nucleoti	0	37.952	27	8	8	8	455	51.8	6.35	22.21	8	metabolic		catalytic a	Pf05761	221294	ENSG0000	NT5DC1	6				0	1.016	0.661869874	8.74	887.4	911.6	12.57	5.8	High	High	High	High	High	High	1	
FALSE	High	Master Prc	P45880	Voltage-de	0	101.283	56	14	33	14	294	31.5	7.56	88.71	14	regulation	membrane	nucleotide	Pf01459	7417	ENSG0000	VDAC2	10	Calcium si		Mitochonc	12	1.016	0.7106401	3.27	5839.3	5667.2	9.3	2.17	High	High	High	High	High	High	1	
FALSE	High	Master Prc	Q15397	Pumilio ho	0	19.209	13	5	5	5	648	73.5	9.64	11.13	5	regulation	chromosor	DNA bindi	Pf07369, P	9933	ENSG0000	KIAA0020;	9				0	1.016	0.80191625	4.19	815.8	802.7	0.45	6.86	High	High	High	High	High	High	1	
FALSE	High	Master Prc	Q14498-1	RNA-bindi	0	75.01	30	13	24	13	530	59.3	10.1	66.97	13	metabolic	cytoskelet	protein bir	Pf00076, P	9584	ENSG0000	RBM39	20		mRNA Pro		1	1.016	0.805808366	13.57	4645.9	4454.7	14.15	4.25	High	High	High	High	High	High	1	
FALSE	High	Master Prc	P36954	DNA-direct	0.009	2.178	18	1	1	1	125	14.5	5.14	0	1	metabolic	nucleus	catalytic a	Pf01096, P	5438	ENSG0000	POLR2I	19	Huntingtoi	Eukaryotic	Signaling b	75	1.016	0.806363188	14.83	29.9	29.4	13.55	3.44	High	High	High	High	High	High	1	
FALSE	High	Master Prc	P48059	LIM and se	0.003	3.473	4	1	1	1	325	37.2	8.05	2.2	1	cell organi	cytosol;me	metal ion l	Pf00412	3987	ENSG0000	LIMS1	2		Primary Fc	Regulation	5	1.016	0.822800957	29.09	313	335.2	20.78	10.07	High	High	High	High	High	High	1	
FALSE	High	Master Prc	P50395-1	Rab GDP d	0	110.242	60	20	33	15	445	50.6	6.47	97.98	20	metabolic	cytoplasm;	catalytic a	Pf00996	2665	ENSG0000	GDI2	10			Neutrophil	10	1.015	0.350631582	2.07	3754.8	3564.7	4.67	3.14	High	High	High	High	High	High	1	
FALSE	High	Master Prc	P27816-1	Microtubu	0	100.192	24	22	40	22	1152	120.9	5.43	78.11	22	cell divisio	cytoplasm;	protein bir	Pf00418	4134	ENSG0000	MAP4	3				0	1.015	0.393607211	1.2	7033.2	6768.5	15.77	6.64	High	High	High	High	High	High	1	
FALSE	High	Master Prc	Q9Y6C9	Mitochonc	0	35.86	23	4	7	4	303	33.3	7.97	27.96	4	regulation	membrane		Pf00153	23788	ENSG0000	MTCH2	11				0	1.015	0.451157081	4.23	1494	1390.3	4.25	5.53	High	High	High	High	High	High	1	
FALSE	High	Master Prc	O75880	Protein SC	0	7.353	12	2	3	2	301	33.8	8.88	8.65	2	cell organi	membrane	antioxidan	Pf00578, P	6341	ENSG0000	SCO1	17		Electron Tı	TP53 Regu	11	1.015	0.53219993	3.51	734.8	740.5	2.95	14.39	High	High	High	High	High	High	1	
FALSE	High	Master Prc	P07900	Heat shock	0	557.653	55	52	363	30	732	84.6	5.02	935.96	52	cell organi	cytoplasm;	catalytic a	Pf00183, P	3320	ENSG0000	HSP90AA1	14	Pathways i	Nucleotide	Sema3A P/	86	1.015	0.569617331	0.37	42909.4	42152.4	14.79	5.19	High	High	High	High	High	High	1	
FALSE	High	Master Prc	O43572	A-kinase a	0	5.221	2	1	1	1	662	73.8	6.39	2.69	1	coagulatio	cytoplasm;	catalytic a	Pf00615	11216	ENSG0000	AKAP10	17		G Protein !	Factors inv	3	1.015	0.601674612	31.97	76.8	75.7	30.93	14.5	High	High	High	High	High	High	1	
FALSE	Low	Master Prc	O75394	39S riboso	0.064	1.09	9	1	1	1	65	7.6	10.8	1.98	1	cell organi	membrane	structural	Pf00471, P	9553	ENSG0000	MRPL33	2	Ribosome		Mitochonc	7	1.015	0.63041846	7.78	1614.7	1548	2.57	5.51	High	High	High	High	High	High	1	
FALSE	High	Master Prc	Q16626	Male-enha	0.001	4.303	7	1	1	1	185	19.9	4.22	3.17	1	cell differe	cytoplasm	protein bir	Pf06910	4201	ENSG0000	MEA1	6				0	1.015	0.634070106	9.48	448.8	510.1	15.05	11.4	High	High	High	High	High	High	1	
FALSE	High	Master Prc	O43865	S-adenosyl	0	48.707	24	12	20	12	530	58.9	6.89	28.78	12	metabolic	cytoplasm;	catalytic a	Pf00670, P	10768	ENSG0000	AHCYL1	1	Metabolic	Trans-sulfu	Ion homeo	33	1.015	0.645531533	8.64	5309.2	5256.2	7.46	0.8	High	High	High	High	High	High	1	
FALSE	High	Master Prc	P51572	B-cell rece	0	66.583	48	14	34	14	246	28	8.44	66.76	14	cell death;	cytosol;en	protein bir	Pf05529, P	10134	ENSG0000	BCAP31	X	Protein prc		Apoptotic	9	1.015	0.670028781	7.22	16393.1	16152.7	1.31	6.37	High	High	High	High	High	High	1	
FALSE	High	Master Prc	O43837-1	isocitrate c	0	18.549	13	4	6	4	385	42.2	8.46	9.63	4	metabolic	mitochond	catalytic a	Pf00180	3420	ENSG0000	IDH3B	20	Citrate cyc	TCA Cycle	Citric acid	10	1.015	0.677112525	20.69	2001.1	2097.7	26.93	9.13	High	High	High	High	High	High	1	
FALSE	High	Master Prc	Q9HAV4	exportin-5	0	41.618	10	9	12	9	1204	136.2	5.8	20.91	9	metabolic	cytoplasm;	protein bir	Pf08389	57510	ENSG0000	XPO5	6	RNA trans	miRNA Bic	MicroRNA	6	1.014	0.347194572	2.88	2118.2	2025.7	4.01	2.49	High	High	High	High	High	High	1	
FALSE	High	Master Prc	Q6P1M0	Long-chain	0	17.361	11	5	6	5	643	72	8.47	15.26	5	metabolic	endoplasm	catalytic a	Pf00501, P	10999	ENSG0000	SLC27A4	9	Insulin resi	PPAR signa	Defective S	12	1.014	0.393357145	0.47	2030.6	2036.1	2.59	1.96	High	High	High	High	High	High	1	
FALSE	High	Master Prc	P42766	60S riboso	0	33.19	45	7	24	7	123	14.5	11.05	44.75	7	cell organi	cytosol;me	RNA bindi	Pf00831	11224	ENSG0000	RPL35	9	Ribosome	Cytoplasm	Peptide ch	34	1.014	0.403235267	3.39	9046.6	8921	2.59	6.18	High	High	High	High	High	High	1	
FALSE	High	Master Prc	O00165	HCLS1-asso	0	10.115	13	2	2	2	279	31.6	4.92	5.86	2	cell organi	cytoplasm;	protein bir		10456	ENSG0000	HAX1	1				0	1.014	0.492442603	4.82	696	709.1	6.14	3.68	High	High	High	High	High	High	1	
FALSE	High	Master Prc	Q02241-1	Kinesin-like	0	18.411	7	4	5	4	960	110	8.51	13.9	4	cell divisio	cytoplasm;	catalytic a	Pf00225, P	9493	ENSG0000	KIF23	15	MicroRNA:		COPI-depe	16	1.014	0.586590165	18.8	264.8	298.8	28.94	8.05	High	High	High	High	High	High	1	
FALSE	High	Master Prc	Q8IZ83	Aldehyde c	0	16.206	5	3	5	3	802	85.1	6.79	11.49	3	metabolic	membrane	catalytic a	Pf00171, P	126133	ENSG0000	ALDH16A1	19				0	1.014	0.617407465	19.25	1318.9	1300.6	16.4	10.1	High	High	High	High	High	High	1	
FALSE	High	Master Prc	P63104-1	14-3-3 pro	0	142.796	78	21	65	15	245	27.7	4.79	157.88	21	cell organi	cytoplasm;	catalytic a	Pf00244	7534	ENSG0000	YWHAZ	8	Oocyte me	Cell Cycle;	Chk1/Chk2	51	1.014	0.709873178	5.5	12981.1	12800.6	8.84	1.96	High	High	High	High	High	High	1	[Y149(100
FALSE	High	Master Prc	P05387	60S acidic	0	104.88	92	8	36	7	115	11.7	4.54	118.28	8	cell organi	cytosol;me	protein bir	Pf00428	6181	ENSG0000	RPLP2	11	Ribosome	Cytoplasm	Peptide ch	34	1.014	0.82404492	5.45	10946.5	10735.2	4.06	8.23	High	High	High	High	High	High	1	
FALSE	High	Master Prc	Q66PJ3-1	ADP-ribosy	0	17.026	15	3	3	3	421	44.9	10.93	14.34	3	metabolic	nucleus	protein bir	Pf10500	51329	ENSG0000	ARL6IP4	12				0	1.014	0.887865219	13.6	700	690	6.11	4.07	High	High	High	High	High	High	1	
FALSE	High	Master Prc	Q9BYD2	39S riboso	0	17.904	21	7	11	7	267	30.2	10.08	14.96	7	cell organi	membrane	protein bir	Pf01281	65005	ENSG0000	MRPL9	1	Ribosome		Mitochonc	7	1.013	0.416758637	3.31	5726.5	5567	7.72	3.16	High	High	High	High	High	High	1	
FALSE	High	Master Prc	O75533-1	splicing fac	0	198.175	29	31	59	31	1304	145.7	7.09	150.49	31	cell organi	nucleus;sp	DNA bindi	Pf02161, P	23451	ENSG0000	SF3B1	2	Spliceosom	mRNA Pro	B-WICH co	11	1.013	0.501507873	5.72	13196.4	13020.7	10.24	3.81	High	High	High	High	High	High	1	
FALSE	High	Master Prc	Q8IVT2	Mitotic int	0	46.246	20	10	16	10	679	75.3	6.83	37.91	10	cell divisio	cytoplasm;	protein bir	Pf15304	126353	ENSG0000	C19orf21;	19				0	1.013	0.545102615	16.73	2350.9	2303.4	25.89	0.42	High	High	High	High	High	High	1	
FALSE	High	Master Prc	Q9Y6W5	Wiskott-Al	0	6.376	5	2	2	2	498	54.3	5.53	4.57	2	cell organi	cytoplasm;	protein bir		10163	ENSG0000	WASF2	1	Bacterial ir	Regulation	Regulation	19	1.013	0.602229488	15.83	43.6	43.1	8.67	13.4	High	High	High	High	High	High	1	
FALSE	High	Master Prc	P30520	adenylosu	0	22.898	13	5	8	5	456	50.1	6.55	18.54	5	metabolic	cytoplasm;	catalytic a	Pf00709	159	ENSG0000	ADSS	1	Metabolic	Nucleotide	Purine ribc	8	1.013	0.608304709	13.45	862.1	898.1	12.08	13.74	High	High	High	High	High	High	1	
FALSE	High	Master Prc	O15305	Phosphom	0	7.761	9	2	2	2	246	28.1	6.77	4.83	2	metabolic	cytoplasm;	catalytic a	Pf03332, P	5373	ENSG0000	PMM2	16	Amino sug		Defective I	13	1.013	0.679857123	39.38	177.8	175.5	37.51	10.7	High	High	High	High	High	High	1	
FALSE	High	Master Prc	Q00688	peptidyl-pı	0	26.908	28	5	7	5	224	25.2	9.28	22.4	5	metabolic	cytoplasm;	catalytic a	Pf00254	2287	ENSG0000	FKBP3	14				0	1.013	0.707735113	12.5	1445.1	1426.2	7.73	3.5	High	High	High	High	High	High	1	
FALSE	High	Master Prc	Q15020	Squamous	0	58.004	16	10	11	10	963	109.9	5.57	32.18	10	cell organi	cytoplasm;	protein bir	Pf00076, P	9733	ENSG0000	SART3	12				0	1.013	0.724741752	2.43	3096.4	3056.9	8.37	1.9	High	High	High	High	High	High	1	
FALSE	High	Master Prc	P55084	Trifunctior	0	95.477	37	16	34	16	474	51.3	9.41	80.96	16	metabolic	endoplasm	catalytic a	Pf00108, P	3032	ENSG0000	HADHB	2	Fatty acid	Fatty Acid	Beta oxida	23	1.013	0.7821213	5.47	10028.3	10269.9	9.5	6.93	High	High	High	High	High	High	1	
FALSE	High	Master Prc	Q8TED0	U3 small n	0	15.04	8	3	3	3	518	58.4	9.11	10.95	3	metabolic	cytoplasm;	protein bir	Pf00400, P	84135	ENSG0000	UTP15	5	Ribosome		Major patl	6	1.013	0.81145983	17.52	336.4	332.2	7.53	9.98	High	High	High	High	High	High	1	
FALSE	High	Master Prc	P61970	nuclear tra	0	22.429	46	4	10	4	127	14.5	5.38	20.1	4	cell organi	cytoplasm;	protein bir	Pf02136	10204	ENSG0000	NUTF2	16				0	1.013	0.955902552	7.96	2713.7	2659.5	12.31	4.35	High	High	High	High	High	High	1	
FALSE	High	Master Prc	O75312	Zinc finger	0	22.398	20	7	10	7	459	50.9	4.73	16.49	7	cell death;	cytoplasm;	metal ion l	Pf03367	8882	ENSG0000	ZNF259; ZI	11				0	1.013	0.965816681	1.55	2782.3	2754.3	0.36	3.09	High	High	High	High	High	High	1	
FALSE	High	Master Prc	P30085-1	UMP-CMP	0	68.604	47	7	20	7	196	22.2	5.57	57.21	7	metabolic	cytoplasm;	catalytic a	Pf00406, P	51727		CMPK1	1	Metabolic		Interconve	5	1.012	0.364340285	2.1	6473.9	6282.3	3.68	2.12	High	High	High	High	High	High	1	
FALSE	High	Master Prc	Q00587-1	Cdc42 effe	0	11.716	7	1	1	1	391	40.3	7.15	6.3	1	cell organi	cytoplasm;	enzyme re	Pf00786	11135	ENSG0000	CDC42EP1	22				0	1.012	0.566593607	8.05	107.1	110.7	3.29	10.9	High	High	High	High	High	High	1	
FALSE	High	Master Prc	Q9NPD3	Exosome c	0	31.531	22	4	8	4	245	26.4	6.52	24.66	4	defense re	cytoplasm;	catalytic a	Pf01138, P	54512	ENSG0000	EXOSC4	8	RNA degra		Major patl	15	1.012	0.570365555	9.11	3616.8	3547.7	24.16	2.39	High	High	High	High	High	High	1	
FALSE	High	Master Prc	Q9NZE8	39S riboso	0.002	3.584	4	1	2	1	188	21.5	11.3	6.96	1	cell organi	membrane	structural	Pf01632	51318	ENSG0000	MRPL35	2	Ribosome		Mitochonc	7	1.012	0.589726007	5.14	763.3	805.7	11.25	5.55	High	High	High	High	High	High	1	
FALSE	High	Master Prc	Q8NFH4	Nucleopori	0	17.073	11	3	4	3	326	36.7	5.92	14.91	3	cell divisio	chromosor	protein bir	Pf00400	79023	ENSG0000	NUP37	12	RNA trans		Rev-media	77	1.012	0.666897931	16.37	606.8	633.3	17.04	7.31	High	High	High	High	High	High	1	
FALSE	High	Master Prc	Q9P206-2	Isoform 2 c	0	17.885	9	6	7	6	1094	113	10.02	4.64	6	cell differe				57648	ENSG0000	KIAA1522	1				0	1.012	0.774505684	10.67	177.8	184.1	8.73	5.71	High	High	High	High	High	High	1	
FALSE	High	Master Prc	Q96FJ0	AMSH-like	0	7.903	5	1	1	1	436	49.8	7.23	4.79	1	metabolic	cytosol;me	catalytic a	Pf01398, P	57559	ENSG0000	STAMBPL1	10		TGF-beta S	Metallopro	5	1.012	0.829091722	34.44	118.3	120.4	13.18	16.77	High	High	High	High	High	High	1	
FALSE	High	Master Prc	P40937-1	Replicatior	0	12.164	12	3	4	3	340	38.5	7.2	7.41	3	cell organi	nucleus	catalytic a	Pf00004, P	5985	ENSG0000	RFC5	12	DNA replic	Retinoblas	Translesior	57	1.012	0.846493692	1.91	847.5	826.7	3.28	6.1	High	High	High	High	High	High	1	
FALSE	High	Master Prc	Q8TD16	Protein bic	0	7.134	2	2	3	2	824	93.5	5.44	3.44	2	cell organi	cytoplasm;	protein bir	Pf03961, P	23299	ENSG0000	BICD2	9			COPI-indep	5	1.012	0.874951383	25.99	190.4	187.9	11.49	10.77	High	High	High	High	High	High	1	
FALSE	High	Master Prc	P82650	28S riboso	0	20.304	16	6	9	6	360	41.3	7.9	12.26	6	cell organi	membrane	structural	Pf10245	56945	ENSG0000	MRPS22	3			Mitochonc	6	1.012	0.889334782	3.13	5974.3	6029.7	3.44	5.85	High	High	High	High	High	High	1	
FALSE	High	Master Prc	P48730-1	Casein kina	0	6.373	11	2	2	2	415	47.3	9.74	3.18	2	cell organi	cytoplasm;	catalytic a	Pf00069, P	1453	ENSG0000	CSNK1D	17	Hippo sign	Integrated	Regulation	36	1.012	0.977236077	25.89	348.6	375.2	20.92	5.38	High	High	High	High	High	High	1	

FALSE	High	Master Prc	P62805	histone H4	0	80.038	65	13	47	13	103	11.4	11.36	95.77	13	cell organi	chromosor	DNA bindi	Pf00125, P	8359; 836	ENSG0000	HIST1H4A;	6; 1; 12	Viral carcir	Gastric Car	SIRT1 nega	82	1.011		0.381673169	1.14	17988.4	16530.1	12.51	19.44	High	High	High	High	High	High	1	
FALSE	High	Master Prc	O95299	NADH deh	0	36.006	19	5	11	5	355	40.7	8.48	31.5	5	cell organi	membrane	catalytic a	Pf01712, P	4705	ENSG0000	NDUFA10	2; CHR_HG	Oxidative p	Electron Tr	Complex I	13	1.011		0.411247567	1.8	3645.3	3595.4	24.68	1.25	High	High	High	High	High	High	1	
FALSE	High	Master Prc	P52735	Guanine nu	0	18.211	7	4	5	4	878	101.2	7.08	13.92	4	cell organi	cytoplasm;	metal ion l	Pf00017, P	7410	ENSG0000	VAV2	9	Regulation	Integrin-m	G alpha (1	53	1.011		0.478009974	9.33	414.4	460.2	22.87	27.62	High	High	High	High	High	High	1	
FALSE	High	Master Prc	Q12769	nuclear po	0	50.763	9	9	11	9	1436	162	5.5	30.18	9	cell organi	chromosor	protein bir	Pf11715	23279	ENSG0000	NUP160	11	RNA transp		Rev-media	77	1.011		0.549041641	1.96	2212.1	2320.5	7.6	5.34	High	High	High	High	High	High	1	
FALSE	High	Master Prc	Q9UPN3	Microtubu	0	13.468	1	3	3	3	7388	837.8	5.39	6.54	3	cell organi	cytoplasm;	catalytic a	Pf00036, P	23499	ENSG0000	MACF1	1		Mesoderm	Signaling b	5	1.011		0.575353879	6.39	569.1	593.8	10.68	7.89	High	High	High	High	High	High	1	[K7375;
FALSE	High	Master Prc	Q9Y3T9	Nucleolar c	0	33.194	11	8	12	8	749	84.9	5.62	30.23	8	cell death;	cytosol;nu	protein bir	Pf03715	26155	ENSG0000	NOC2L	1			Regulation	6	1.011		0.58471887	5.09	3148.1	3224.4	8.42	6.13	High	High	High	High	High	High	1	[S49(100)]
FALSE	High	Master Prc	O43294-1	Transformi	0.001	4.268	3	1	1	1	461	49.8	7.03	4.28	1	cell differe	cytoplasm;	metal ion l	Pf00412	7041	ENSG0000	TGFB1I1	16		Androgen		4	1.011		0.619278843	2.27	149.9	152	6.12	2.83	High	High	High	High	High	High	1	
FALSE	High	Master Prc	Q53RE8	ankyrin rep	0	9.93	11	1	1	1	183	19.6	6.89	5.57	1			protein bir	Pf00023, P	51239	ENSG0000	ANKRD39	2				0	1.011		0.6932741	8.14	302.3	281.6	7.43	3.83	High	High	High	High	High	High	1	
FALSE	High	Master Prc	Q9H0W9	Ester hydro	0	5.816	7	2	2	2	315	35.1	6.7	6.07	2		nucleus	catalytic a	Pf08925	28970	ENSG0000	C11orf54	11				0	1.011		0.744331793	8.2	1251.8	1295.4	12.82	8.05	High	High	High	High	High	High	1	
FALSE	High	Master Prc	Q7L273	BTB/POZ d	0	9.282	6	2	2	2	389	42.5	6.37	5.29	2	cell organi		protein bir	Pf00651, P	54793	ENSG0000	KCTD9	8				0	1.01		0.331158808	1.35	230.3	228.1	7.36	4.52	High	High	High	High	High	High	1	
FALSE	High	Master Prc	Q6NVY1	3-hydroxyi	0	6.766	5	2	2	2	386	43.5	8.19	3.05	2	metabolic	mitochond	catalytic a	Pf00378, P	26275	ENSG0000	HIBCH	2	Metabolic	Amino Aci	Branched-c	9	1.01		0.377682217	1.11	256.1	253.7	13.09	7.3	High	High	High	High	High	High	1	
FALSE	High	Master Prc	Q14997	Proteasom	0	12.94	3	3	4	3	1843	211.2	6.9	9.85	3	cell differe	cytoplasm;	enzyme re	Pf11919	23198	ENSG0000	PSME4	2	Proteasom		Regulation	141	1.01		0.481000439	8.66	662.3	656.1	18.9	6.42	High	High	High	High	High	High	1	
FALSE	High	Master Prc	Q9BW91-1	ADP-ribose	0.003	3.192	7	3	3	3	350	39.1	8.22	0	3	metabolic	membrane	catalytic a	Pf00293	53343	ENSG0000	NUDT9	4	Purine met		Phosphate	6	1.01		0.502668209	11.07	1132.8	1125.5	22.95	4.11	High	High	High	High	High	High	1	
FALSE	High	Master Prc	O94760-1	N(G),N(G)-	0	15.541	19	4	8	3	285	31.1	5.81	18.99	4	metabolic	cytosol;mi	catalytic a	Pf02274	23576	ENSG0000	DDAH1	1		Mesoderm	eNOS activ	5	1.01		0.525868661	19.23	517.2	512.2	34.91	5.87	High	High	High	High	High	High	1	
FALSE	High	Master Prc	P17096-1	High mobil	0	52.625	59	7	28	7	107	11.7	10.32	74.74	7	cell organi	chromosor	catalytic a		3159	ENSG0000	HMGA1	6		Adipogene	2-LTR circl	21	1.01		0.548802673	21.26	7495.7	7419.5	31.56	7.91	High	High	High	High	High	High	1	
FALSE	High	Master Prc	Q6IN85	Serine/thre	0	12.658	6	4	4	2	833	95.3	4.94	8.01	4		cytoplasm;		Pf04802	55671	ENSG0000	SMEK1; PP	14	Glucagon s	Integrated		2	1.01		0.63643111	9.52	487	435.5	9.62	6.93	High	High	High	High	High	High	1	
FALSE	High	Master Prc	Q7Z2W9	39S riboso	0	13.766	25	4	5	4	205	22.8	9.89	9.21	4	cell organi	membrane	RNA bindi	Pf00829	219927	ENSG0000	MRPL21	11	Ribosome		Mitochonc	7	1.01		0.655169036	17.5	424.2	420.2	11.74	8.93	High	High	High	High	High	High	1	
FALSE	High	Master Prc	Q6UXN9	WD repeat	0	7.788	6	1	1	1	313	35.1	7.69	3.93	1	cell organi	chromosor	catalytic a	Pf00400	80335	ENSG0000	WDR82	3	mRNA surv			1	1.01		0.772982732	50.4	324.8	321.7	34.76	15.23	High	High	High	High	High	High	1	
FALSE	High	Master Prc	P84085	ADP-ribosy	0	61.674	49	8	19	2	180	20.5	6.79	62.33	8	regulation	cytoplasm;	catalytic a	Pf00025, P	381	ENSG0000	ARF5	7	Endocytosi		COPI-depe	12	1.01		0.779987351	14.83	453.6	470.5	10.95	7.79	High	High	High	High	High	High	1	
FALSE	High	Master Prc	Q9UQB8	Brain-spec	0	22.14	15	6	6	6	552	60.8	8.9	16.91	6	cell differe	cytoplasm;	protein bir	Pf00018, P	10458	ENSG0000	BAIAP2	17	Regulation	Regulation	Regulation	14	1.01		0.813379986	2.9	879.5	913.7	4.13	3.41	High	High	High	High	High	High	1	
FALSE	High	Master Prc	P62191	26S protea	0	58.477	33	13	26	11	440	49.2	6.21	53.39	13	metabolic	cytoplasm;	catalytic a	Pf00004, P	5700	ENSG0000	PSMC1	14	Viral carcir	Proteasom	Regulation	155	1.01		0.967284514	19.69	7007.7	7092	10.93	4.64	High	High	High	High	High	High	1	
FALSE	High	Master Prc	Q8N983	39S riboso	0	11.172	17	3	3	3	215	23.4	8.65	6.75	3	cell organi	membrane	protein bir	Pf05047	84545	ENSG0000	MRPL43	10			Mitochonc	6	1.01		0.989448266	11.34	1425.4	1482.5	7.48	4.79	High	High	High	High	High	High	1	
FALSE	High	Master Prc	Q01082-1	Spectrin be	0	220.213	23	50	74	47	2364	274.4	5.57	156.85	50	cell organi	cytoplasm;	protein bir	Pf00169, P	6711	ENSG0000	SPTBN1	2		TGF-beta S	Nephrin fa	25	1.009		0.342012578	0.66	20322.4	19580.4	2.16	3.82	High	High	High	High	High	High	1	
FALSE	High	Master Prc	Q13724-1	Mannosyl-	0	36.141	13	8	10	8	837	91.9	8.9	29.64	8	metabolic	endoplasm	catalytic a	Pf03200	7841	ENSG0000	MOGS	2	N-Glycan b		Defective I	11	1.009		0.431574735	3.21	3677.8	3471.1	15.7	4.48	High	High	High	High	High	High	1	
FALSE	High	Master Prc	P09884	DNA polym	0	38.714	8	8	10	8	1462	165.8	5.85	23.49	8	cell organi	chromosor	catalytic a	Pf00136, P	5422	ENSG0000	POLA1	X	Purine met	Retinoblas	Activation	34	1.009		0.475269551	21.75	1911.1	1893.7	62.12	10.91	High	High	High	High	High	High	1	
FALSE	High	Master Prc	Q9H6F5	Coiled-coil	0	19.121	18	4	5	4	360	40.2	10.33	10.15	4		nucleus	protein bir		79080	ENSG0000	CCDC86	11				0	1.009		0.476731928	16.23	1401.4	1389.2	44.75	7.95	High	High	High	High	High	High	1	
FALSE	High	Master Prc	P67936	Tropomyo	0	130.449	77	27	65	15	248	28.5	4.69	111.63	27	cell differe	cytoplasm;	metal ion l	Pf00261, P	7171	ENSG0000	TPM4	19	Hypertropl	Striated M	Smooth M	8	1.009		0.549424783	2.94	19545.2	19594.7	11.6	0.94	High	High	High	High	High	High	1	
FALSE	High	Master Prc	P49736	DNA replic	0	135.465	33	23	38	23	904	101.8	5.52	110.9	23	cell death;	chromosor	catalytic a	Pf00493, P	4171	ENSG0000	MCM2	3	DNA replic	Cell Cycle;	M/G1 Trar	25	1.009		0.596472127	5.74	6548.4	6493.4	9.16	4.45	High	High	High	High	High	High	1	[Q321]
FALSE	High	Master Prc	Q6R327-1	Rapamycir	0	6.289	1	1	1	1	1708	192.1	7.47	5.62	1	cell organi	cytosol	enzyme re	Pf14663, P	253260	ENSG0000	RICTOR	5	mTOR sign	Target Of I	PIP3 activa	30	1.009		0.612591012	46.4	369.9	366.4	54.14	16.88	High	High	High	High	High	High	1	
FALSE	High	Master Prc	Q9P2X0-1	Dolichol-pl	0.002	4.162	11	1	2	1	92	10.1	5.94	2.96	1	metabolic	endoplasm	catalytic a	Pf08285	54344	ENSG0000	DPM3	1	N-Glycan b		Defective I	13	1.009		0.66012929	16.45	425.5	421.7	18.5	1.66	High	High	High	High	High	High	1	
FALSE	High	Master Prc	P62906	60S riboso	0	96.459	46	11	51	11	217	24.8	9.94	149.2	11	cell organi	cytosol;me	protein bir	Pf00687	4736	ENSG0000	RPL10A	6	Ribosome	Cytoplasm	Peptide ch	34	1.009		0.661482343	3.28	19369.5	19205.5	7.88	0.58	High	High	High	High	High	High	1	
FALSE	Medium	Master Prc	Q13572-1	Inositol-tet	0.013	1.905	3	1	1	1	414	45.6	6.16	2.11	1	coagulatio	cytosol;me	catalytic a	Pf05770	3705	ENSG0000	ITPK1	14; CHR_H	Phosphatic		Synthesis c	9	1.009		0.736546569	15.21	326.1	323.1	10.84	2.96	High	High	High	High	High	High	1	
FALSE	High	Master Prc	P52272	Heterogen	0	265.195	62	40	125	40	730	77.5	8.7	283.94	40	metabolic	membrane	protein bir	Pf00076, P	4670	ENSG0000	HNRNPM	19	Spliceosom	mRNA Pro	mRNA Spli	11	1.009		0.74399584	5.09	37545.9	36209.5	8.75	2.92	High	High	High	High	High	High	1	
FALSE	High	Master Prc	O60832-1	H/ACA ribo	0	89.472	33	15	29	15	514	57.6	9.42	72.81	15	cell organi	cytoplasm;	catalytic a	Pf01472, P	1736	ENSG0000	DKC1	X	Ribosome		rRNA mod	10	1.009		0.749882386	5.09	6117.2	6528.5	5.39	8.52	High	High	High	High	High	High	1	
FALSE	High	Master Prc	P63167	Dynein ligh	0	18.757	51	3	8	3	89	10.4	7.4	21.27	3	cell commu	chromosor	catalytic a	Pf01221	8655	ENSG0000	DYNLL1	12	Vasopressi		COPI-indep	55	1.009		0.768497797	14.19	1646.9	1631.9	3.52	9.8	High	High	High	High	High	High	1	
FALSE	High	Master Prc	Q8NBJ5	Procollage	0	30.083	15	9	13	9	622	71.6	7.31	19.12	9		endoplasm	catalytic a	Pf00535, P	79709	ENSG0000	COLGALT1	19	Lysine deg		Collagen b	5	1.009		0.911504256	13.72	4216.6	4177.4	6.61	5.09	High	High	High	High	High	High	1	
FALSE	High	Master Prc	P61247	40S riboso	0	120.807	63	21	78	21	264	29.9	9.73	174.51	21	cell differe	cytoplasm;	protein bir	Pf01015	6189	ENSG0000	RPS3A	4	Ribosome	Cytoplasm	Peptide ch	38	1.009		0.975439804	1.94	26525.4	25953.6	3.21	5.16	High	High	High	High	High	High	1	
FALSE	High	Master Prc	B5ME19	eukaryotic	0	73.267	22	21	29	21	914	105.4	5.64	62.47	21	metabolic	cytoplasm	protein bir	Pf01399, P	728689	ENSG0000	EIF3CL	16	RNA transp			1	1.008		0.651911238	24.05	4723.6	4822.6	26.03	6.93	High	High	High	High	High	High	1	
FALSE	High	Master Prc	Q9UK76	Jupiter mic	0	31.534	42	5	11	5	154	16	5.6	34.96	5		cytoplasm;			51155	ENSG0000	HN1; JPT1	17				0	1.008		0.679416911	1.69	4602	4566.8	0.35	1.28	High	High	High	High	High	High	1	
FALSE	High	Master Prc	P52292	Importin su	0	68.694	30	9	19	9	529	57.8	5.4	49.99	9	metabolic	cytoplasm;	protein bir	Pf00514, P	3838	ENSG0000	KPNA2	17	Influenza A		ISG15 anti	15	1.008		0.701014827	1.02	5666.2	5620.6	6.69	3.16	High	High	High	High	High	High	1	
FALSE	High	Master Prc	P14174	Macrophag	0	32.261	32	3	27	3	115	12.5	7.88	73	3	cell organi	cell surface	catalytic a	Pf01187	4282	ENSG0000	MIF	22; CHR_H	Phenylalar	Adipogene	Gene and p	13	1.008		0.733981983	33.24	2664.7	2644.2	29.62	8.74	High	High	High	High	High	High	1	
FALSE	High	Master Prc	O95865	N(G),N(G)-	0	26.582	18	4	7	3	285	29.6	6.01	17.25	4	metabolic	cytoplasm;	catalytic a	Pf02274	23564	ENSG0000	DDAH2	6; CHR_HS			eNOS activ	4	1.007		0.424692601	2.14	1425.3	1435.6	18.41	8.2	High	High	High	High	High	High	1	
FALSE	High	Master Prc	Q9NZQ3-1	NCK-intera	0.006	2.382	1	1	1	1	722	78.9	6.38	2.02	1	cell organi	cytosol;nu	protein bir	Pf00018, P	51517	ENSG0000	NCKIPSD	3			Regulation	8	1.007		0.46003567	0.32	779.1	783.9	15.45	1.05	High	High	High	High	High	High	1	
FALSE	High	Master Prc	P15408-1	Fos-relatec	0	10.098	6	1	2	1	326	35.2	7.49	4.88	1	cell death;	nucleus	DNA bindi		2355	ENSG0000	FOSL2	2	Osteoclast	Corticotro		2	1.007		0.488025923	7.42	309.7	323.8	30.65	15.91	High	High	High	High	High	High	1	
FALSE	High	Master Prc	Q13084	39S riboso	0	24.909	24	4	7	4	256	30.1	8.29	26.04	4	cell organi	cytosol;me	protein bir	Pf00830	10573	ENSG0000	MRPL28	16	Ribosome		Mitochonc	7	1.007		0.525782591	3.68	1639.5	1694.5	3.22	11.42	High	High	High	High	High	High	1	
FALSE	High	Master Prc	Q96EL3	39S riboso	0	16.189	38	3	6	3	112	12.1	8.76	22.05	3	cell organi	membrane	protein bir	Pf10780	116540	ENSG0000	MRPL53	2			Mitochonc	6	1.007		0.588637193	11.29	244.3	242.7	2.87	17.54	High	High	High	High	High	High	1	
FALSE	High	Master Prc	Q49A26-1	Putative ox	0	24.901	8	4	6	4	553	60.5	9.17	12.76	4	metabolic	chromosor	catalytic a	Pf00855, P	84656		GLYR1	16				0	1.007		0.676761736	6.3	1381.9	1372.9	4.17	1.65	High	High	High	High	High	High	1	
FALSE	High	Master Prc	Q96C19	EF-hand dc	0	13.721	13	4	4	4	240	26.7	5.2	6.76	4		membrane	metal ion l		79180	ENSG0000	EFHD2	1				0	1.007		0.70523888	12.47	618.4	613.9	3.91	11.09	High	High	High	High	High	High	1	
FALSE	High	Master Prc	Q6DKI1	60S riboso	0	24.852	17	3	6	3	246	28.6	10.51	22.09	3	metabolic	ribosome	RNA bindi	Pf00327	285855	ENSG0000	RPL7L1	6				0	1.007		0.725835998	1.73	1560.1	1549.4	6.57	2.69	High	High	High	High	High	High	1	
FALSE	High	Master Prc	Q9Y285	Phenylalar	0	12.201	8	3	4	3	508	57.5	7.8	13.2	3	cell organi	cytoplasm;	catalytic a	Pf00152, P	2193	ENSG0000	FARSA	19	Aminoacyl		Cytosolic t	5	1.007		0.732592426	0.36	2084.5	2157.6	6.09	5.52	High	High	High	High	High	High	1	
FALSE	High	Master Prc	Q9BZK7	F-box-like/	0	13.338	10	3	5	2	514	55.6	5.55	15.88	3	cell differe	nucleus	DNA bindi	Pf00400, P	79718	ENSG0000	TBL1XR1	3	Wnt signal		NOTCH1 Ir	36	1.007		0.79781908	25.14	1336.2	1327.1	17.46	6.57	High	High	High	High	High	High	1	
FALSE	High	Master Prc	P11387	DNA topoi	0	28.729	14	9	11	9	765	90.7	9.31	18.52	9	cell death;	chromosor	catalytic a	Pf01028, P	7150	ENSG0000	TOP1	20			SUMOylati	5	1.006		0.42762073	1.97	354.3	357.1	20.6	5.63	High	High	High	High	High	High	1	
FALSE	High	Master Prc	Q9UJU6-1	Drebrin-lik	0	27.642	22	7	8	1	430	48.2	5.05	21.15	7	cell organi	cytoplasm;	enzyme re	Pf00018, P	28988	ENSG0000	DBNL	7		T-Cell anti	Neutrophil	12	1.006		0.511907899	24.96	644.8	640.9	49.39	12.61	High	High	High	High	High	High	1	
FALSE	High	Master Prc	O60232	Sjoegren sy	0	25.311	24	3	6	3	199	21.5	5.24	25.96	3	cell divisio		protein bir	Pf06677	10534	ENSG0000	SSSCA1	11				0	1.006		0.636026319	2.09	712.3	707.9	4.9	9.03	High	High	High	High	High	High	1	
FALSE	High	Master Prc	Q9Y277	Voltage-de	0	35.189	29	7	9	6	283	30.6	8.66	18.83	7	cell commu	membrane	nucleotide	Pf01459	7419	ENSG0000	VDAC3	8	Parkinson's		Mitochonc	14	1.006		0.642490414	8.35	4029	4001	5.26	4.92	High	High	High	High	High	High	1	
FALSE	High	Master Prc	P40227-1	T-complex	0	162.589	55	24	67	24	531	58	6.68	185.14	24	metabolic	cytoplasm;	nucleotide	Pf00118, P	908	ENSG0000	CCT6A	7			Prefoldin r	9	1.006		0.646714929	4.63	23027.5	22893.7	3.09	1.25	High	High	High	High	High	High	1	
FALSE	High	Master Prc	P50991	T-complex	0	214.671	66	31	83	30	539	57.9	7.83	219.89	31	metabolic	cytoplasm;	nucleotide	Pf00118	10575	ENSG0000	CCT4	2			BBSome-m	13	1.006		0.657365534	3.19	22630	22090.2	2.38	1.38	High	High	High	High	High	High	1	[K21;
FALSE	High	Master Prc	Q8TEM1-1	Nuclear po	0	46.396	9	11	11	11	1887	205	6.81	29.6	11	cell organi	endoplasm	protein bir	Pf02368	23225	ENSG0000	NUP210	3	RNA transp		Rev-media	64	1.006		0.714249584	13.86	2251.5	2174.5	11.82	2.88	High	High	High	High	High	High	1	
FALSE	High	Master Prc	O60231	Putative pr	0	42.735	10	7	8	7	1041	119.2	6.8	20.47	7	metabolic	cytoplasm;	catalytic a	Pf00271, P	8449	ENSG0000	DHX16	6; CHR_HS	Spliceosom	mRNA Pro	mRNA Spli	6	1.006		0.733703247	24.58	1093.1	1086.6	21.73	3.18	High	High	High	High	High	High	1	
FALSE	High	Master Prc	P60709	Actin, cyto	0	478.258	76	29	612	11	375	41.7	5.48	1725.35	29	cell organi	cytoplasm;	DNA bindi	Pf00022	60	ENSG0000	ACTB	7	Dilated car	Arrhythmc	Formation	102	1.006		0.776780996	43.15	32159.1	32004.1	20.15	18.03	High	High	High	High	High	High	1	[T229(100
FALSE	High	Master Prc	Q9BVP2	Guanine nu	0	47.081	25	10	16	10	549	62	9.16	37.87	10	cell divisio	membrane	catalytic a	Pf01926, P	26354	ENSG0000	GNL3	3	Ribosome		Major path	5	1.006		0.804028665	7.88	3651	3690.2	1.36	5.91	High	High	High	High	High	High	1	
FALSE	High	Master Prc	Q14160	protein scr	0	29.101	5	5	6	4	1630	174.8	5.07	17.04	5	cell commu	cytoplasm;	catalytic a	Pf00595, P	23513		SCRIB	8	Viral carcir	Human Th	Asymmetri	8	1.006		0.808985696	12.09	903.1	897.5	27.86	14.04	High	High	High	High	High	High	1	
FALSE	High	Master Prc	Q9HC38-2	Isoform 2 c	0	44.153	43	10	14	10	298	33.2	5.6	31.72	10		mitochond	protein bir	Pf00903, P	51031	ENSG0000	GLOD4	17				0	1.006		0.82946841	18.59	2866.3	2857.6	11.49	5.84	High	High	High	High	High	High	1	
FALSE	High	Master Prc	Q15738	sterol-4-alp	0	32.857	32	10	14	10	373	41.9	8.06	23.98	10	metabolic	endoplasm	catalytic a	Pf00106, P	50814	ENSG0000	NSDHL	X	Steroid bic	Cholestero	Cholestero	7	1.006		0.983100258	14.11	3707.8	3659.2	11.05	0.57	High	High	High	High	High	High	1	
FALSE	High	Master Prc	Q9BZE4	Nucleolar G	0	31.88	13	8	13	8	634	73.9	9.5	34.98	8	cell differe	cytoplasm;	catalytic a	Pf00009, P	23560	ENSG0000	GTPBP4	10	Ribosome			1	1.005		0.495013081	6.26	2973.2	2957.2	12.67	4.24	High	High	High	High	High	High	1	
FALSE	High	Master Prc	O43776	Asparagine	0	54.968	25	10	13	10	548	62.9	6.25	44.43	10	metabolic	cytoplasm;	catalytic a	Pf00152, P	4677	ENSG0000	NARS	18	Aminoacyl	Photodyna	Cytosolic t	6	1.005		0.565795607	0.76	3626.9	3667.3	1.04	3.7	High	High	High	High	High	High	1	
FALSE	High	Master Prc	P04899-1	guanine nu	0	76.605	46	10	22	7	355	40.4	5.54	63.25	10	cell divisio	cytoplasm;	catalytic a	Pf00025, P	2771	ENSG0000	GNAI2	3	Adrenergic	Human Th	G alpha (z)	70	1.005		0.666945889	18.57	3117.8	3115.3	15.81	9.24	High	High	High	High	High	High	1	
FALSE	High	Master Prc	Q13177	Serine/thre	0	49.322	22	9	13	9	524	58	5.96	37.95	9	cell death;	cytoplasm;	catalytic a	Pf00069, P	5062	ENSG0000	PAK2	3	Renal cell c	Integrin-m	Sema3A PA	65	1.005		0.812390606	7.08	2462.8	2339.8	13.91	7.41	High	High	High	High	High	High	1	
FALSE	High	Master Prc	Q9P2E5-1	Chondroiti	0.001	4.203	2	2	2	2	772	85.9	7.83	1.62	2	metabolic	Golgi;mem	catalytic a	Pf02434, P	54480	ENSG0000	CHPF2	7	Glycosami		Chondroiti	7	1.005		0.852768818	13.96	1121.3	1083.8	13	4.32	High	High	High	High	High	High	1	
FALSE	High	Master Prc	Q6UWE0	E3 ubiquiti	0	13.854	6	3	3	3	723	83.5	5.94	8.64	3	cell organi	cytoplasm;	catalytic a	Pf00536, P	90678	ENSG0000	LRSAM1	9			Antigen pr	4	1.005		0.878431077	22.71	120.2	135	18.37	8.31	High	High	High	High	High	High	1	
FALSE	High	Master Prc	Q9UNS1	Protein tim	0	4.61	2	1	1	1	1208	138.6	5.4	4.29	1	cell divisio	nucleus	protein bir	Pf04821, P	8914	ENSG0000	TIMELESS	12			Processing	5	1.005		0.909761705	27.74	150.8	156.8	10.66	12.44	High	High	High	High	High	High	1	
FALSE	High	Master Prc	Q9UNL2	Translocon	0	19.407	12	2	7	2	185	21.1	9.61	15.62	2	cell organi	endoplasm		Pf07074	6747	ENSG0000	SSR3	3	Protein pro		SRP-depen	4	1.004		0.46194613	0.86	3450.5	3527.2	4.68	7.79	High	High	High	High	High	High	1	
FALSE	High	Master Prc	Q5T1M5	FK506-binc	0	8.779	4	3	3	3	1219	133.5	5.2	1.94	3	cell differe	cytoplasm;	catalytic a	Pf00038, P	23307	ENSG0000	FKBP15	9		TYROBP Ca		1	1.004		0.521644208	3.62	479.2	467.2	2.77	5.06	High	High	High	High	High	High	1	
FALSE	High	Master Prc	Q9UQ35	serine/argi	0	330.979	28	63	120	63	2752	299.4	12.06	265.46	63	metabolic	nucleus;sp	nucleotide		23524	ENSG0000	SRRM2	16			mRNA Spli	4	1.004		0.71411106	15.14	31371.1	31248.5	10.8	5.4	High	High	High	High	High	High	1	[S263(50);
FALSE	High	Master Prc	P82930	28S riboso	0	20.119	17	3	6	3	218	25.6	9.98	14.03	3	cell organi	membrane	structural		65993	ENSG0000	MRPS34	16			Mitochonc	6	1.004		0.743745504	8.41	1082.7	1102.4	1.49	8.79	High	High	High	High	High	High	1	
FALSE	High	Master Prc	Q9Y2X3	Nucleolar p	0	52.045	23	9	17	9	529	59.5	8.92	42.95	9	cell growth	cytosol;me	protein bir	Pf01798, P	51602	ENSG0000	NOP58	2	Ribosome		Major path	11	1.004		0.774951045	2.3	4251.3	4345	4.26	2.24	High	High	High	High	High	High	1	
FALSE	High	Master Prc	Q03154-1	Aminoacyl	0	7.959	10	2	2	2	408	45.9	6.18	7.55	2	metabolic	cytoplasm;	catalytic a	Pf01546, P	95	ENSG0000	ACY1	3	Arginine bi	Urea cycle	Defective A	12	1.004		0.804433732	35.06	271.3	270.1	13.08	15.62	High	High	High	High	High	High	1	
FALSE	High	Master Prc	Q5JPH6	Probable g	0	21.009	12	5	6	5	523	58.7	8.76	16.3	5	metabolic	cytoplasm;	catalytic a	Pf00749	124454	ENSG0000	EARS2	16	Aminoacyl		Mitochonc	7	1.004		0.817458585	3.49	2093.3	2170.4	3.88	3.1	High	High	High	High	High	High	1	
FALSE	High	Master Prc	Q96TA2-1	ATP-depen	0	26.207	10	5	7	5	773	86.4	8.76	21.73	5	cell organi	membrane	catalytic a	Pf00004, P	10730	ENSG0000	YME1L1	10			Processing	3	1.004		0.819896753	7.61	1539.3	1503.3	2.64	8.23	High	High	High	High	High	High	1	

FALSE	High	Master Prc	Q9UL63-1	muskelin [(	0	7.101	4	2	2	2	735	84.7	6.34	2.64	2	cell organi	cytoplasm;	protein bir	Pf00754, P	4289	ENSG0000	MKLN1	7				0	1.004	0.883501158	3.15	957.3	959.6	3.43	0.47	High	High	High	High	High	High	1	
FALSE	High	Master Prc	Q6YN16	Hydroxyste	0	33.172	17	6	11	6	418	45.4	7.99	21.78	6	metabolic	membrane	catalytic a	Pf00106, P	84263	ENSG0000	HSDL2	9				0	1.004	0.934919741	53.35	1803	1795.9	23.07	15.48	High	High	High	High	High	High	1	
FALSE	High	Master Prc	Q9NZM1	Myoferlin	0	380.281	43	75	131	75	2061	234.6	6.18	316.03	75	cell organi	membrane	protein bir	Pf00168, P	26509	ENSG0000	MYOF	10		Aryl Hydro		2	1.004	0.951361823	6.89	36905.5	36750.1	1.55	4.03	High	High	High	High	High	High	1	
FALSE	High	Master Prc	P09601	heme oxyg	0	8.632	8	1	1	1	288	32.8	8.25	5.61	1	cell commu	cytosol;end	catalytic a	Pf01126	3162	ENSG0000	HMOX1	22	MicroRNA	NRF2 path	Interleukin	19	1.003	0.467791769	7.65	213.6	237.2	24.37	20.29	High	High	High	High	High	High	1	
FALSE	High	Master Prc	P13929-1	Beta-enola	0	84.996	29	9	76	4	434	47	7.71	182.5	9	metabolic	cytoplasm;	catalytic a	Pf00113, P	2027	ENSG0000	ENO3	17	Metabolic	Glycolysis	Gluconeog	12	1.003	0.498507795	5.58	605.8	602.1	24.17	0.28	High	High	High	High	High	High	1	[Y44(100)]
FALSE	High	Master Prc	P62826	GTP-bindir	0	129.973	60	17	68	17	216	24.4	7.49	214.99	17	cell divisio	chromosor	catalytic a	Pf00025, P	5901	ENSG0000	RAN	12	Ribosome	Androgen	MicroRNA	30	1.003	0.508556702	1.39	22363.5	22906.9	3.3	6.69	High	High	High	High	High	High	1	
FALSE	High	Master Prc	P61956	small ubiq	0	27.832	29	3	12	1	95	10.9	5.5	27.66	3	metabolic	nucleus	catalytic a	Pf00240, P	6613	ENSG0000	SUMO2	17	RNA transp		SUMOylati	22	1.003	0.545457581	1.62	786	783.5	5.51	7.46	High	High	High	High	High	High	1	
FALSE	High	Master Prc	Q9Y676	28S riboso	0	22.08	23	4	10	4	258	29.4	9.38	31.09	4	cell organi	membrane	protein bir	Pf01084	28973	ENSG0000	MRPS18B	6; CHR_HS	Viral carcir		Mitochond	7	1.003	0.597421105	3.21	2981.7	2972.6	5.99	2.21	High	High	High	High	High	High	1	
FALSE	High	Master Prc	P42356	phosphatic	0	11.723	1	1	3	1	2102	236.7	7.06	8.67	1	cell organi	cytoplasm;	catalytic a	Pf00454, P	5297	ENSG0000	PI4KA	22	Metabolic	Ectoderm	Synthesis c	10	1.003	0.740242834	24.46	204.6	228.8	23.25	9.73	High	High	High	High	High	High	1	
FALSE	High	Master Prc	O60443	Non-syndr	0	35.108	17	6	7	6	496	54.5	5.17	15.32	6	cell death;	cytoplasm;		Pf04598	1687	ENSG0000	DFNA5; GS	7				0	1.003	0.762199622	27.94	940.8	937.8	18.79	9.74	High	High	High	High	High	High	1	
FALSE	High	Master Prc	Q8WWV3-	Reticulon-	0	4.91	5	1	1	1	396	43.6	9.11	4.4	1	metabolic	membrane	catalytic a	Pf00107, P	84816	ENSG0000	RTN4IP1	6				0	1.003	0.78193451	9.49	452.2	459.5	9.51	1.36	High	High	High	High	High	High	1	
FALSE	High	Master Prc	P21926	CD9 antige	0	39.66	15	2	7	2	228	25.4	7.15	39.62	2	cell organi	cell surface	protein bir	Pf00335	928	ENSG0000	CD9	12	Hematopo		Platelet de	12	1.003	0.807271841	54.5	877.9	880	29.54	17.67	High	High	High	High	High	High	1	
FALSE	High	Master Prc	Q02127	Dihydroord	0	6.604	6	1	2	1	395	42.8	9.67	9.93	1	metabolic	cytoplasm;	catalytic a	Pf01180	1723	ENSG0000	DHODH	16	Metabolic		Pyrimidine	6	1.003	0.820330321	23.86	424.6	423.5	11.11	9.03	High	High	High	High	High	High	1	
FALSE	High	Master Prc	P56211	cAMP-regu	0	14.744	17	2	6	1	112	12.3	9.09	13.35	2	cell divisio	cytoplasm	enzyme re	Pf04667	10776	ENSG0000	ARPP19	15			MASTL Fac	5	1.003	0.843763522	5.81	1691.7	1623.3	6.35	10.79	High	High	High	High	High	High	1	
FALSE	High	Master Prc	Q13148-1	TAR DNA-b	0	75.981	34	11	19	11	414	44.7	6.19	52.58	11	cell organi	cytoplasm;	DNA bindi	Pf00076, P	23435	ENSG0000	TARDBP	1				0	1.002	0.464686547	12.39	4298.8	4292.1	47.43	9.13	High	High	High	High	High	High	1	
FALSE	High	Master Prc	Q14789	Golgin sub	0	89.134	9	23	27	23	3259	375.8	5	56.26	23	cell organi	Golgi;mem	catalytic a	Pf01496, P	2804	ENSG0000	GOLGB1	3			COPI-medi	11	1.002	0.5245551	4.28	3174.3	3068.3	8.53	4.22	High	High	High	High	High	High	1	
FALSE	High	Master Prc	P49959	Double-str	0	11.757	6	4	6	4	708	80.5	5.9	14.97	4	cell organi	chromosor	catalytic a	Pf00149, P	4361	ENSG0000	MRE11A; I	11	Homologo	miRNA Re	G2/M DNA	47	1.002	0.591944791	6.63	2085	2081.1	3.55	7.25	High	High	High	High	High	High	1	
FALSE	High	Master Prc	Q9BQ52-1	Zinc phosp	0	19.331	7	5	6	5	826	92.2	7.9	11.27	5	cell organi	mitochond	catalytic a	Pf00753, P	60528	ENSG0000	ELAC2	17	RNA transp		tRNA proc	7	1.002	0.621080007	3.71	790.4	788.9	8.32	3.84	High	High	High	High	High	High	1	
FALSE	High	Master Prc	P61011-1	signal reco	0	21.73	10	5	7	5	504	55.7	8.75	13.84	5	cell organi	cytoplasm;	catalytic a	Pf00448, P	6729	ENSG0000	SRP54	14	Protein ex	mRNA Pro	SRP-depen	5	1.002	0.65793306	8.27	1919.1	2014.7	8.28	6.02	High	High	High	High	High	High	1	
FALSE	High	Master Prc	P43243	Matrin-3 [(	0	214.987	48	37	91	37	847	94.6	6.25	262.98	37	defense re	membrane	metal ion l	Pf00076, P	9782	ENSG0000	MATR3	5				0	1.002	0.699477083	6.3	19867.5	20151.1	2.7	5.28	High	High	High	High	High	High	1	
FALSE	High	Master Prc	Q9NVH1-1	DnaJ homo	0	13.23	6	2	2	2	559	63.2	8.4	5.72	2	cell organi	membrane	protein bir	Pf00226, P	55735	ENSG0000	DNAJC11	1			Cristae for	3	1.002	0.718573715	8.84	502.7	491.1	2.56	11.27	High	High	High	High	High	High	1	
FALSE	High	Master Prc	P36915-1	Guanine nu	0	10.36	7	4	5	4	607	68.6	5.8	11	4	regulation	nucleus	catalytic a	Pf01926, P	2794	ENSG0000	GNL1	6; CHR_HS				0	1.002	0.844024226	5.61	736.4	703.7	13.04	11.92	High	High	High	High	High	High	1	
FALSE	High	Master Prc	Q14678-1	KN motif a	0	21.319	5	4	5	4	1352	147.2	5.3	18.91	4	cell organi	cytoplasm;	protein bir	Pf00023, P	23189	ENSG0000	KANK1	9				0	1.002	0.9449033	24.87	905.3	903.5	9.15	11.57	High	High	High	High	High	High	1	
FALSE	High	Master Prc	P51665	26S protea	0	22.742	24	6	9	6	324	37	6.77	26.9	6	metabolic	cytosol;ext	catalytic a	Pf01398, P	5713	ENSG0000	PSMD7	16	Proteasom	Proteasom	Regulation	153	1.001	0.433597253	0.06	2147.4	2166.7	2.65	2.69	High	High	High	High	High	High	1	
FALSE	High	Master Prc	O75083	WD repeat	0	84.855	30	13	23	13	606	66.2	6.65	64.14	13	cell differe	cytoplasm;	protein bir	Pf00400, P	9948	ENSG0000	WDR1	4		Hypertropl	Platelet de	5	1.001	0.505502444	1.02	4087.9	4042	3.02	2.14	High	High	High	High	High	High	1	
FALSE	High	Master Prc	P61960	Ubiquitin-f	0.001	4.471	51	2	2	2	85	9.1	9.31	5.11	2	metabolic	cytoplasm;	protein bir	Pf03671	51569	ENSG0000	UFM1	13				0	1.001	0.511474522	14.63	243.7	231	35.08	7.93	High	High	High	High	High	High	1	
FALSE	High	Master Prc	P62304	small nucle	0	10.656	17	2	8	2	92	10.8	9.44	11.75	2	cell organi	cytoplasm;	protein bir	Pf01423	6635	ENSG0000	SNRPE	1	Spliceosom	mRNA Pro	SLBP indep	16	1.001	0.513110038	2.11	1467.3	1465.8	6.97	2.02	High	High	High	High	High	High	1	
FALSE	High	Master Prc	Q96HY7	Probable 2	0.006	2.431	1	1	1	1	919	103	6.93	0	1	cell differe	cytosol;mi	catalytic a	Pf00676, P	55526	ENSG0000	DHTKD1	10		Integrated	Glyoxylate	4	1.001	0.52211383	2.14	499.4	464.9	7.28	6.79	High	High	High	High	High	High	1	
FALSE	High	Master Prc	P11117	Lysosomal	0	10.927	8	3	3	3	423	48.3	6.74	9.01	3	cell organi	membrane	catalytic a	Pf00328	53	ENSG0000	ACP2	11	Lysosome			1	1.001	0.527278528	9.29	297	306.5	21.39	4.12	High	High	High	High	High	High	1	
FALSE	High	Master Prc	Q9UNZ5	Leydig cell	0.002	3.878	14	2	3	2	99	10.6	11.55	1.88	2		nucleus		Pf09495	28974	ENSG0000	C19orf53	19				0	1.001	0.579839342	11.35	1368.8	1366.9	10.62	10.08	High	High	High	High	High	High	1	
FALSE	High	Master Prc	Q9H3P7	Golgi resid	0	29.065	16	6	9	6	528	60.6	5.06	22.46	6	metabolic	Golgi;mem	protein bir	Pf00373, P	64746	ENSG0000	ACBD3	1			Golgi Asso	5	1.001	0.620782709	21.81	1852	1816.7	25.89	11.11	High	High	High	High	High	High	1	
FALSE	High	Master Prc	Q5JTJ3-1	Cytochrom	0	15.819	22	3	4	3	125	14.1	8.25	9.64	3	cell organi	membrane	catalytic a	Pf02297	388753	ENSG0000	COA6	1			Mitochond	2	1.001	0.64247436	14.42	363.4	340.3	14.36	6.79	High	High	High	High	High	High	1	
FALSE	High	Master Prc	Q9Y2R9	28S riboso	0	30.692	32	5	8	5	242	28.1	9.99	25.03	5	cell organi	membrane	RNA bindi	Pf00177	51081	ENSG0000	MRPS7	17	Ribosome		Mitochond	7	1.001	0.723518123	5.21	2972.7	2970.9	8.5	3.36	High	High	High	High	High	High	1	
FALSE	High	Master Prc	P42224-1	Signal tran	0	20.851	7	4	5	4	750	87.3	6.05	11.45	4	cell death;	cytoplasm;	DNA bindi	Pf00017, P	6772	ENSG0000	STAT1	2	Toxoplasm	IL-5 Signali	Regulation	81	1.001	0.74657351	4.09	566.8	542.3	2.68	4.32	High	High	High	High	High	High	1	
FALSE	High	Master Prc	Q96SB4-3	Isoform 1 c	0	24.371	9	6	7	5	826	92.4	7.71	22.3	6	cell organi	cytoplasm;	catalytic a	Pf00069, P	6732		SRPK1	6		mRNA Pro		1	1.001	0.810949234	22.58	2629	2626	15.66	6.2	High	High	High	High	High	High	1	
FALSE	High	Master Prc	O15145	Actin-relat	0	6.393	14	3	4	3	178	20.5	8.59	2.25	3	cell organi	cytoplasm;	protein bir	Pf04062	10094	ENSG0000	ARPC3	12	Salmonella	Pathogeni	EPHB-med	23	1.001	0.901123468	9.84	1958.6	1956.8	3.12	4.36	High	High	High	High	High	High	1	
FALSE	High	Master Prc	P06744	glucose-6-p	0	201.308	54	25	79	25	558	63.1	8.32	235.87	25	metabolic	cytoplasm;	catalytic a	Pf00342	2821	ENSG0000	GPI	19	Metabolic	Cori Cycle;	Glycolysis;	21	1	0.426542729	0.01	29505.8	29492.8	10.17	3.14	High	High	High	High	High	High	1	
FALSE	High	Master Prc	O14646	Chromodo	0	15.768	4	5	6	5	1710	196.6	7.14	12.35	5	cell organi	cytoplasm;	catalytic a	Pf00176, P	1105	ENSG0000	CHD1	5				0	1	0.442804155	4.04	1262.5	1262.6	33.01	5.78	High	High	High	High	High	High	1	
FALSE	High	Master Prc	O43237	Cytoplasm	0	10.746	11	4	4	4	492	54.1	6.38	2.23	4	cell organi	chromosor	catalytic a	Pf05783	1783	ENSG0000	DYNC1LI2	16	Vasopressi		COPI-indep	36	1	0.45133017	3.1	110.6	135.5	14.31	20.36	High	High	High	High	High	High	1	
FALSE	High	Master Prc	P37837	Transaldol	0	90.236	41	17	45	17	337	37.5	6.81	100.02	17	metabolic	cytoplasm;	catalytic a	Pf00923	6888	ENSG0000	TALDO1	11	Metabolic	Pentose Ph	Gene and	22	1	0.457117728	0.72	20221.6	20225.1	0.31	4.47	High	High	High	High	High	High	1	
FALSE	High	Master Prc	P98179	RNA-bindi	0	30.299	62	6	15	6	157	17.2	8.91	28.02	6	metabolic	cytoplasm;	protein bir	Pf00076, P	5935	ENSG0000	RBM3	X				0	1	0.539478174	1.4	2809.3	2793.8	2.62	4.4	High	High	High	High	High	High	1	
FALSE	High	Master Prc	P47756-2	Isoform 2 c	0	106.483	56	12	35	12	272	30.6	6	92	12	cell organi	cytoplasm;	protein bir	Pf01115	832	ENSG0000	CAPZB	1				0	1	0.562887937	13.6	10950.7	11487.6	23.58	2.81	High	High	High	High	High	High	1	
FALSE	High	Master Prc	Q9P2R3	Rabankyrir	0	21.005	5	4	5	4	1169	128.3	6.1	12.48	4	cell organi	cytoplasm;	metal ion l	Pf00023, P	51479	ENSG0000	ANKFY1	17				0	1	0.569682266	10.52	1241.4	1301	17.19	5.25	High	High	High	High	High	High	1	
FALSE	High	Master Prc	Q92805	Golgin sub	0	11.009	5	3	4	3	767	88.1	5.27	7.8	3	metabolic	cytosol;Go	catalytic a	Pf01465, P	2800	ENSG0000	GOLGA1	9			Retrograde	4	1	0.572719473	9.61	225.2	189.9	13.29	12.95	High	High	High	High	High	High	1	[Q243]
FALSE	High	Master Prc	Q8TF09	Dynein ligh	0.006	2.397	13	1	1	1	96	10.8	7.5	3.8	1	cellular co	cytoplasm;	catalytic a	Pf03259	83657	ENSG0000	DYNLRB2	16			Intraflagell	3	1	0.594501516	6.2	270.1	270.2	2.1	8.68	High	High	High	High	High	High	1	
FALSE	High	Master Prc	Q58EX7	Puratrophi	0.002	3.975	1	1	1	1	1191	130.7	5.64	3.61	1	regulation		protein bir	Pf00621, P	25894	ENSG0000	PLEKHG4	16				0	1	0.601224278	21.45	131.3	145.7	33.52	6.14	High	High	High	High	High	High	1	
FALSE	High	Master Prc	Q13614-1	Myotubula	0	15.036	6	3	6	3	643	73.3	7.4	12.88	3	cell organi	cytoplasm;	catalytic a	Pf02893, P	8898	ENSG0000	MTMR2	11	Metabolic		Synthesis c	11	1	0.617258245	43.74	1048.1	1047.6	52.65	8.75	High	High	High	High	High	High	1	
FALSE	High	Master Prc	Q96I51	RCC1-like (	0	22.334	19	5	7	5	464	50	8.4	22.64	5		mitochond	RNA bindi	Pf00415, P	81554		WBSCR16;	7				0	1	0.620845419	4.91	822.8	872	6.5	4.13	High	High	High	High	High	High	1	
FALSE	High	Master Prc	O60828	Polyglutam	0	16.581	10	2	3	2	265	30.5	6.33	8.47	2	cell divisio	cytoplasm;	DNA bindi	Pf00397	10084	ENSG0000	PQBP1	X	Spliceosom		mRNA Spli	5	1	0.69541213	29.47	284.5	288.1	28.77	11.84	High	High	High	High	High	High	1	
FALSE	High	Master Prc	P28340	DNA polyn	0	33.084	12	9	9	9	1107	123.6	7.03	24	9	cell organi	cytosol;me	catalytic a	Pf00136, P	5424	ENSG0000	POLD1	19	Nucleotide	Homologo	Cytosolic ir	56	1	0.750102366	34.62	2389.4	2444.5	24.53	8.77	High	High	High	High	High	High	1	
FALSE	High	Master Prc	Q9P0J0	NADH deh	0	18.028	24	3	6	3	144	16.7	8.43	11.26	3	cell death;	cytoplasm;	catalytic a	Pf06212	51079	ENSG0000	NDUFA13	19	Alzheimer'	Nucleotide	Complex I	13	1	0.80680602	8.65	706.2	700.7	9.43	8.05	High	High	High	High	High	High	1	
FALSE	High	Master Prc	Q9UII2-1	ATPase inh	0	24.065	31	5	16	5	106	12.2	9.35	15.26	5	cell differe	cell surface	catalytic a	Pf04568	93974	ENSG0000	ATPIF1	1		Electron Tr		1	1	0.820484706	11.07	3445.9	3444.4	5.33	4.68	High	High	High	High	High	High	1	
FALSE	High	Master Prc	O75027-1	ATP-bindir	0	26.833	13	8	9	8	752	82.6	9.33	15.02	8	cellular ho	membrane	catalytic a	Pf00005, P	22	ENSG0000	ABCB7	X	ABC transp		Cytosolic ir	6	1	0.849531093	3.45	1175.8	1149.7	4.92	7.35	High	High	High	High	High	High	1	
FALSE	High	Master Prc	Q09161	Nuclear ca	0	18.15	4	2	4	2	790	91.8	6.43	12.58	2	cell organi	cytoplasm;	protein bir	Pf02854, P	4686	ENSG0000	NCBP1	9	RNA transp	mRNA Pro	Signaling b	61	1	0.964928979	36.36	725.8	713.9	22.8	10.14	High	High	High	High	High	High	1	
FALSE	High	Master Prc	Q13136	Liprin-alph	0	13.115	3	3	3	3	1202	135.7	6.29	3.31	3	cell organi	cytoplasm;	catalytic a	Pf00536, P	8500	ENSG0000	PPFIA1	11			Norepinep	10	1	0.977877629	42.74	166.5	185.3	34.58	10.83	High	High	High	High	High	High	1	
FALSE	High	Master Prc	Q08379	Golgin sub	0	66.439	17	12	19	12	1002	113	5.02	34.02	12	cell divisio	cytoplasm;	catalytic a	Pf01576, P	2801	ENSG0000	GOLGA2	9			COPII (Coa	17	1	0.982633555	18.76	3235.4	3031.4	14.95	3.84	High	High	High	High	High	High	1	
FALSE	High	Master Prc	Q04637-8	Isoform 8 c	0	109.714	20	29	45	27	1600	175.5	5.33	50.83	29	cell organi	cytoplasm;	nucleotide	Pf00566, P	1981	ENSG0000	EIF4G1	3		Translation		1	0.999	0.463874668	0.62	7785.4	7680.3	3.23	1.99	High	High	High	High	High	High	1	
FALSE	High	Master Prc	P00558	phosphogl	0	305.705	85	35	157	35	417	44.6	8.1	413.13	35	cell differe	cytoplasm;	catalytic a	Pf00162	5230	ENSG0000	PGK1	X	Metabolic	Cori Cycle;	Gluconeog	13	0.999	0.477321102	0.93	58128.4	58385.8	0.58	2.57	High	High	High	High	High	High	1	
FALSE	High	Master Prc	Q27J81	Inverted fo	0	52.971	13	13	17	13	1249	135.5	5.38	40.62	13	cell organi	cytoplasm	protein bir	Pf02181, P	64423	ENSG0000	INF2	14		Primary Fo		1	0.999	0.536825811	3.95	2557.9	2465.3	5.99	4.52	High	High	High	High	High	High	1	
FALSE	High	Master Prc	P32322	Pyrroline-5	0	67.209	39	12	20	11	319	33.3	7.61	69.2	12	metabolic	mitochond	catalytic a	Pf01210, P	5831	ENSG0000	PYCR1	17	Metabolic	Amino Aci	Amino acid	8	0.999	0.694239417	8.82	3988.3	4066	5.67	6.21	High	High	High	High	High	High	1	
FALSE	High	Master Prc	Q13523	Serine/thre	0	19.131	5	4	4	4	1007	116.9	10.26	8.38	4	metabolic	chromosor	catalytic a	Pf00069, P	8899	ENSG0000	PRPF4B	6		mRNA Pro		1	0.999	0.754246375	25.62	273	274.9	20.99	10.96	High	High	High	High	High	High	1	
FALSE	High	Master Prc	Q86TS9-1	39S riboso	0	11.479	15	1	1	1	123	13.7	10.26	6.66	1	cell organi	membrane	structural		122704	ENSG0000	MRPL52	14			Mitochond	6	0.999	0.791898899	47.67	251	251.3	25.03	25.9	High	High	High	High	High	High	1	
FALSE	High	Master Prc	O14684	Prostaglan	0	6.866	16	3	7	3	152	17.1	9.5	12.64	3	defense re	cytoplasm;	catalytic a		9536	ENSG0000	PTGES	9	Metabolic	Eicosanoid	Synthesis c	8	0.999	0.819528694	6.11	4191.3	4114.2	2.15	4.73	High	High	High	High	High	High	1	
FALSE	High	Master Prc	O95602	DNA-direct	0	30.689	5	6	6	6	1720	194.7	7.03	19.73	6	metabolic	nucleus	catalytic a	Pf00623, P	25885	ENSG0000	POLR1A	2	RNA polym	Eukaryotic	RNA Polym	17	0.999	0.822780424	1.56	1771	1794.9	2.36	1.58	High	High	High	High	High	High	1	
FALSE	High	Master Prc	P62424	60S riboso	0	174.263	61	19	75	19	266	30	10.61	195.42	19	cell organi	cytoplasm;	protein bir	Pf01248	6130	ENSG0000	RPL7A	9; CHR_HG	Ribosome	Cytoplasm	Peptide ch	34	0.999	0.835139694	10.61	34104.4	34139.2	1.1	10.62	High	High	High	High	High	High	1	
FALSE	High	Master Prc	A0MZ66	Shootin-1	0	55.84	28	14	20	14	631	71.6	5.33	32.43	14	cell organi	cytoplasm;	protein bir		57698	ENSG0000	KIAA1598;	10			Recycling p	4	0.999	0.881193771	14.95	2859.3	2933	15.22	2.8	High	High	High	High	High	High	1	
FALSE	High	Master Prc	Q99816	tumor susc	0.001	4.248	3	1	1	1	390	43.9	6.46	3.44	1	cell commu	cytoplasm;	DNA bindi	Pf03148, P	7251	ENSG0000	TSG101	11	Endocytosi		Budding ar	13	0.999	0.936271982	4.57	285.3	285.6	2.96	5.93	High	High	High	High	High	High	1	
FALSE	High	Master Prc	Q15393-1	Splicing fac	0	150.973	32	31	57	31	1217	135.5	5.26	116.19	31	cell organi	nucleus;sp	protein bir	Pf03178, P	23450	ENSG0000	SF3B3	16	Spliceosom	mRNA Pro	mRNA Spli	7	0.998	0.407132721	0.14	12264.9	12432.4	2.59	5.46	High	High	High	High	High	High	1	
FALSE	High	Master Prc	P02545	Prelamin-A	0	142.172	38	23	68	22	664	74.1	7.02	204.12	23	cell organi	cytosol;me	protein bir	Pf00038, P	4000	ENSG0000	LMNA	1	Hypertropl	Arrhythmo	Signaling b	33	0.998	0.563552462	5.58	15800.5	15798.1	7.74	4.47	High	High	High	High	High	High	1	
FALSE	High	Master Prc	Q96GJ1	tRNA (urac	0.001	4.266	3	1	1	1	504	56.4	8.81	3.44	1	metabolic	mitochond	catalytic a	Pf05175, P	79979	ENSG0000	TRMT2B	X				0	0.998	0.584791444	17.85	185.4	201.5	10.79	20.27	High	High	High	High	High	High	1	
FALSE	High	Master Prc	P26447	Protein S1(	0	63.811	58	11	73	11	101	11.7	6.11	132.09	11	cell organi	extracellul	metal ion l	Pf01023, P	6275	ENSG0000	S100A4	1				0	0.998	0.6133775	7.16	19602	19636.6	10.04	1.05	High	High	High	High	High	High	1	
FALSE	High	Master Prc	Q86XP3-1	ATP-depen	0	49.995	14	8	10	8	938	102.9	7.02	30.85	8	metabolic	cytoplasm;	catalytic a	Pf00270, P	11325	ENSG0000	DDX42	17	Spliceosom		mRNA Spli	6	0.998	0.622931071	13.95	1349	1367.4	18.32	1.28	High	High	High	High	High	High	1	
FALSE	High	Master Prc	Q8TE77	Protein ph	0	6.676	6	2	2	2	659	73	5.3	4.99	2	cell organi	cytoplasm;	catalytic a	Pf00782, P	54961	ENSG0000	SSH3	11	Axon guida	Regulation		3	0.998	0.688521544	9.47	94.5	96.6	22.81	11.32	High	High	High	High	High	High	1	[S286(100
FALSE	High	Master Prc	P12268	inosine-5'-	0	175.281	50	20	43	18	514	55.8	6.9	121.25	20	cell organi	cytoplasm;	catalytic a	Pf00478, P	3615	ENSG0000	IMPDH2	3	Drug meta		Neutrophil	10	0.998	0.738708653	4.85	12684.6	12414.5	4.03	2.43	High	High	High	High	High	High	1	
FALSE	High	Master Prc	Q86W92-1	Liprin-beta	0	7.106	3	2	3	2	1011	114	5.55	9.24	2	cell organi	cytosol;me	protein bir	Pf00536, P	8496	ENSG0000	PPFIBP1	12			Receptor-t	3	0.998	0.83751728	18.16	1043.2	1045.1	10.96	6.6	High	High	High	High	High	High	1	
FALSE	High	Master Prc	Q9BU76-1	Multiple m	0	23.304	16	3	6	3	263	29.4	10.02	13.42	3	transport	extracellul	protein bir	Pf10159	79169	ENSG0000	C1orf35	1			Neutrophil	3	0.998	0.940204093	24.44	1786.8	1790.9	13.56	7.72	High	High	High	High	High	High	1	[S220(99.
FALSE	High	Master Prc	Q13748-1	tubulin alp	0	255.828	61	21	155	2	450	49.9	5.1	527.89	21	cell organi	cytoplasm;	catalytic a	Pf00091, P	113457; 7	ENSG0000	TUBA3D; T	2; 13	Phagosom	Pathogeni	COPI-indep	66	0.997	0.458986174	1.9	236.8	257.1	13.95	8.96	High	High	High	High	High	High	1	[T271(100
FALSE	High	Master Prc	Q4VC31	coiled-coil	0	13.171	35	4	6	4	144	16.6	7.81	9.05	4		mitochond		Pf09774	131076	ENSG0000	CCDC58	3				0	0.997	0.472358619	1.27	243.1	247.3	1.86	1.52	High	High	High	High	High	High	1	
FALSE	High	Master Prc	P47736	Rap1 GTPa	0	5.603	3	1	1	1	663	73.3	5.82	5.02	1	cellular co	cytosol;Go	catalytic a	Pf02145, P	5909	ENSG0000	RAP1GAP	1	Rap1 signa	Human Th	RET signali	8	0.997	0.480074476	19.66	342.6	356.4	57.49	5.37	High	High	High	High	High	High	1	

FALSE	High	Master Prc	Q9NY27	Serine/thre	0	14.647	11	4	4	4	417	46.9	4.54	10.72	4	metabolic	cytoplasm;	enzyme re	Pf09184	151987	ENSG0000	PPP4R2	3			Processing	5	0.997	0.4903699	3.68	768.4	771	9.88	1.28	High	High	High	High	High	High	1	
FALSE	High	Master Prc	O95870-1	Protein AB	0.001	4.415	2	1	1	1	558	63.2	8.13	4.04	1	metabolic	membrane	catalytic a	Pf00561, P	7920	ENSG0000	ABHD16A	6; CHR_HS				0	0.997	0.505039756	6.36	283.4	294.6	8.25	10.34	High	High	High	High	High	High	1	
FALSE	High	Master Prc	Q9BRP4	Proteasom	0	13.849	9	3	3	3	392	42.2	6.32	7.98	3		proteasom	protein bir	Pf00400	80227	ENSG0000	PAAF1	11				0	0.997	0.543802588	4.21	576.7	574.8	6.59	10.95	High	High	High	High	High	High	1	
FALSE	High	Master Prc	P25787	Proteasom	0	61.89	52	9	23	9	234	25.9	7.43	56.72	9	metabolic	cytoplasm;	catalytic a	Pf00227, P	5683	ENSG0000	PSMA2	7	Proteasom	Proteasom	Regulation	151	0.997	0.563299392	6.26	5867.4	5600.5	10.24	4	High	High	High	High	High	High	1	
FALSE	High	Master Prc	Q9Y4D7-1	plexin-D1 |	0	11.281	3	2	2	2	1925	211.9	7.15	8.27	2	cell organi	membrane	protein bir	Pf01403, P	23129	ENSG0000	PLXND1	3			Other sem	4	0.997	0.670102285	9.77	331.6	326.7	3.06	10.06	High	High	High	High	High	High	1	
FALSE	High	Master Prc	P30622-1	Isoform 2 (	0	57.838	13	16	18	13	1427	160.9	5.39	32.65	16	cell differe	chromosor	catalytic a	Pf01302, P	6249	ENSG0000	CLIP1	12		Regulation		1	0.997	0.679862751	49.94	1031.7	1046.6	39.51	10.1	High	High	High	High	High	High	1	
FALSE	High	Master Prc	Q99536	Synaptic ve	0	56.964	23	6	17	6	393	41.9	6.29	44.04	6	metabolic	cytoplasm;	catalytic a	Pf00107, P	10493	ENSG0000	VAT1	17			Neutrophil	3	0.997	0.803696272	9.91	5300.3	5314	5.25	4.05	High	High	High	High	High	High	1	
FALSE	High	Master Prc	Q9NVP2	Histone ch	0	10.155	17	2	2	2	202	22.4	4.56	8.48	2	cell differe	chromosor	protein bir	Pf04729	55723	ENSG0000	ASF1B	19				0	0.996	0.434531907	2.11	453.3	467.3	15.74	5.96	High	High	High	High	High	High	1	
FALSE	High	Master Prc	Q8N7H5	RNA polym	0	11.719	7	3	3	3	531	59.9	4.63	7.93	3	cell organi	cytoplasm;	protein bir	Pf03985	54623	ENSG0000	PAF1	19		Endoderm	E3 ubiquiti	10	0.996	0.462749607	2.84	141	153.7	10.84	4.93	High	High	High	High	High	High	1	
FALSE	High	Master Prc	Q14978-3	Isoform 3 (	0	193.017	46	28	88	1	700	73.7	9.47	212.49	28	cell organi	cytoplasm	nucleotide	Pf05022	9221	ENSG0000	NOLC1	10				0	0.996	0.50202858	17.73	382.4	433.3	23.39	28.45	High	High	High	High	High	High	1	
FALSE	High	Master Prc	O60678	protein arg	0	10.263	7	3	4	3	531	59.8	5.35	8.84	3	cell organi	cytoplasm;	catalytic a	Pf06325, P	10196		PRMT3	11			Protein me	6	0.996	0.603182314	11.33	492.5	532.5	26.3	11.24	High	High	High	High	High	High	1	
FALSE	High	Master Prc	Q7Z6Z7	E3 ubiquiti	0	253.95	14	46	73	46	4374	481.6	5.22	195.31	46	cell differe	cytoplasm;	catalytic a	Pf00632, P	10075	ENSG0000	HUWE1	X	Ubiquitin r		Neutrophil	7	0.996	0.641888546	18.28	19554.3	19640.4	19.53	5.54	High	High	High	High	High	High	1	
FALSE	High	Master Prc	P17706-1	Tyrosine-p	0	10.436	6	2	2	2	415	48.4	8.29	2.12	2	cell differe	cytoplasm;	catalytic a	Pf00102	5771	ENSG0000	PTPN2	18	Jak-STAT si		Negative r	5	0.996	0.684048247	3.52	517.4	531.9	7.06	2.6	High	High	High	High	High	High	1	
FALSE	High	Master Prc	P46459	Vesicle-fus	0	26.414	12	6	10	6	744	82.5	6.95	24.76	6	cell organi	cytoplasm;	catalytic a	Pf00004, P	4905	ENSG0000	NSF	17; CHR_H	GABAergic	Synaptic V	COPI-depe	25	0.996	0.786333292	16.01	2422.1	2415.8	6.69	9.77	High	High	High	High	High	High	1	
FALSE	High	Master Prc	P40616	ADP-ribosy	0	9.382	23	3	3	3	181	20.4	5.72	8.8	3	cell organi	cytoplasm;	catalytic a	Pf00025, P	400	ENSG0000	ARL1	12			Retrograde	4	0.996	0.795828431	19.9	678.6	718.3	10.31	11.42	High	High	High	High	High	High	1	
FALSE	High	Master Prc	Q9NX63	MICOS con	0	15.349	14	3	4	3	227	26.1	8.28	10.99	3	cell organi	cytoplasm;	protein bir	Pf05300, P	54927	ENSG0000	CHCHD3	7			Cristae for	5	0.996	0.881710878	42.85	2299.2	2308.2	24.29	7.52	High	High	High	High	High	High	1	
FALSE	High	Master Prc	P53004	Biliverdin r	0	33.786	26	7	10	7	296	33.4	6.44	27.87	7	metabolic	cytoplasm;	catalytic a	Pf01408, P	644	ENSG0000	BLVRA	7	Porphyrin		Heme degr	4	0.995	0.413166922	0.79	2151.2	2217.3	8.33	7.1	High	High	High	High	High	High	1	
FALSE	Low	Master Prc	Q9UKZ4	Teneurin-1	0.059	1.124	1	1	1	1	2725	304.8	6.42	0	1	cell organi	cytoplasm;	protein bir	Pf06484, P	10178	ENSG0000	TENM1	X				0	0.995	0.544291803	13.5	37.9	43.2	27.61	10.18	High	High	High	High	High	High	1	[Q2607]
FALSE	High	Master Prc	P84103	Serine/argi	0	43.825	47	8	22	8	164	19.3	11.65	31.08	8	metabolic	cytoplasm;	protein bir	Pf00076, P	6428	ENSG0000	SRSF3	6	Herpes sim	mRNA Pro	Cleavage o	14	0.995	0.658200546	5.64	1489.5	1472.1	1	5.96	High	High	High	High	High	High	1	
FALSE	High	Master Prc	Q96KR1	Zinc finger	0	32.713	9	7	10	7	1074	116.9	9.04	22.01	7	developme	chromosor	DNA bindi	Pf07528, P	51663	ENSG0000	ZFR	5		Exercise-in		1	0.995	0.663046417	8.45	1450.6	1441.6	9.84	1.84	High	High	High	High	High	High	1	
FALSE	High	Master Prc	P35241-1	radixin [OS	0	178.566	49	34	91	18	583	68.5	6.37	164.2	34	cell organi	cytoplasm;	protein bir	Pf00373, P	5962	ENSG0000	RDX	11	MicroRNA:	Regulation	Recycling p	8	0.995	0.689716655	8.93	6789.4	7112.2	11.84	2.45	High	High	High	High	High	High	1	
FALSE	High	Master Prc	P78371-1	T-complex	0	322.384	75	31	106	31	535	57.5	6.46	381.95	31	cell organi	cytoplasm;	nucleotide	Pf00118	10576	ENSG0000	CCT2	12			BBSome-m	16	0.995	0.747785788	11.52	31418.7	30632.3	9.87	4.26	High	High	High	High	High	High	1	
FALSE	High	Master Prc	Q9Y6A9	Signal pept	0	6.045	17	1	2	1	102	11.8	9.31	6.62	1	metabolic	endoplasm	catalytic a	Pf06645	28972	ENSG0000	SPCS1	3	Protein ex		Synthesis,	9	0.995	0.838881917	47.23	801.5	805.8	18.38	21.19	High	High	High	High	High	High	1	
FALSE	High	Master Prc	O95782	AP-2 comp	0	59.769	16	11	14	6	977	107.5	7.03	46.59	11	cell organi	cytosol;me	protein bir	Pf01602, P	160	ENSG0000	AP2A1	19	Endocytosi	Synaptic V	Cargo reco	48	0.995	0.851127788	23.04	1220.2	1192.8	16.49	6.72	High	High	High	High	High	High	1	
FALSE	High	Master Prc	P62312	U6 snRNA-	0	6.229	14	1	4	1	80	9.1	9.58	7.7	1	metabolic	cytoplasm;	protein bir	Pf01423	11157	ENSG0000	LSM6	4	RNA degra		mRNA Spli	8	0.995	0.967967776	26.25	900.8	1082.7	27.03	10.85	High	High	High	High	High	High	1	
FALSE	High	Master Prc	P62136-1	serine/thre	0	65.8	48	13	25	3	330	37.5	6.33	79.01	13	cell divisio	cytoplasm;	catalytic a	Pf00149	5499	ENSG0000	PPP1CA	11	Proteoglyc	Nicotine A	Signaling b	35	0.994	0.406576299	1.2	248.3	252.4	5.14	3.11	High	High	High	High	High	High	1	
FALSE	High	Master Prc	Q15404-1	Ras suppre	0	23.568	24	4	7	4	277	31.5	8.65	19.74	4	regulation	cytosol	protein bir	Pf08757	6251	ENSG0000	RSU1	10			Regulation	4	0.994	0.469747766	4.82	1306.1	1354.8	3.69	11.93	High	High	High	High	High	High	1	
FALSE	High	Master Prc	Q7Z6M1-1	Rab9 effec	0	13.686	8	2	2	2	372	40.5	6.25	6.12	2	transport	cytoplasm;	protein bir	Pf01344, P	10244	ENSG0000	RABEPK	9			Retrograde	4	0.994	0.496894434	19.61	270.4	272.1	46.86	7.9	High	High	High	High	High	High	1	
FALSE	High	Master Prc	Q8NCA5	protein FA	0	32.06	10	4	5	3	519	55.4	9.03	19.7	4	metabolic		catalytic a	Pf10239	25940		FAM98A	2				0	0.994	0.533278463	11.28	387.6	362.7	20.49	11.6	High	High	High	High	High	High	1	
FALSE	High	Master Prc	O00499-2	Isoform IIB	0	59.186	20	7	14	7	518	56.5	5.12	42.06	7	cell differe	cytoplasm;	protein bir	Pf00018, P	274	ENSG0000	BIN1	2		VEGFA-VE(		1	0.994	0.566316046	17.57	2791.1	2807.5	28.58	1.49	High	High	High	High	High	High	1	
FALSE	High	Master Prc	Q13769	THO comp	0	10.773	5	3	4	3	683	78.5	6.87	11.77	3	cell differe	cytoplasm;	protein bir	Pf09766	8563	ENSG0000	THOC5	22	RNA trans		Cleavage o	10	0.994	0.575243274	0.19	1785.8	1785.7	1.57	1.75	High	High	High	High	High	High	1	
FALSE	High	Master Prc	P07951-1	Tropomyos	0	63.92	38	14	31	1	284	32.8	4.7	55.8	14	cell organi	cytoplasm;	protein bir	Pf00261, P	7169	ENSG0000	TPM2	9	Cardiac mu	Striated M	Smooth M	8	0.994	0.580456113	9.77	93.4	96.9	5.85	11.96	High	High	High	High	High	High	1	
FALSE	High	Master Prc	Q13131	5'-AMP-act	0	11.815	10	4	4	4	559	64	8.12	7.82	4	cell commu	cytoplasm;	catalytic a	Pf00069, P	5562	ENSG0000	PRKAA1	5	Oxytocin s	AMP-activ	Energy dep	41	0.994	0.581704927	16.34	144.2	152.9	14.45	13.89	High	High	High	High	High	High	1	
FALSE	High	Master Prc	Q9HA64	Ketosamin	0	10.935	9	2	2	2	309	34.4	7.33	7.27	2	metabolic	cytosol	catalytic a	Pf01636, P	79672	ENSG0000	FN3KRP	17			Gamma ca	3	0.994	0.657203042	23.3	535.1	538.4	21.09	7.23	High	High	High	High	High	High	1	
FALSE	High	Master Prc	O14907	Tax1-bindi	0	21.133	33	3	3	3	124	13.7	8.48	10.58	3	regulation	cytoplasm;	protein bir	Pf00595, P	30851	ENSG0000	TAX1BP3	17			RHO GTPa:	4	0.994	0.69549938	28.73	718.4	718.4	30.14	10.88	High	High	High	High	High	High	1	
FALSE	High	Master Prc	P62820	Ras-relate	0	70.477	60	13	28	6	205	22.7	6.21	65.62	13	cell organi	cytoplasm;	catalytic a	Pf00009, P	5861	ENSG0000	RAB1A	2	Legionello		COPI-depe	21	0.994	0.780758201	7.18	2438.1	2461.1	3.42	3.06	High	High	High	High	High	High	1	
FALSE	High	Master Prc	P62913	60S riboso	0	45.34	43	9	27	9	178	20.2	9.6	47.25	9	cell organi	cytoplasm;	protein bir	Pf00281, P	6135	ENSG0000	RPL11	1	Ribosome	Cytoplasm	Peptide ch	34	0.994	0.976707852	23.74	7528.5	7573.9	8.71	8.73	High	High	High	High	High	High	1	
FALSE	High	Master Prc	P04083	annexin A1	0	193.652	69	24	105	24	346	38.7	7.02	305.75	24	cell differe	cell surface	catalytic a	Pf00191	301	ENSG0000	ANXA1	9		Integrated	Gastrin-CR	20	0.994	0.98710686	17.21	32606.8	32808.3	5.44	7.25	High	High	High	High	High	High	1	
FALSE	High	Master Prc	Q9UNI6	Dual specif	0	11.43	10	2	2	2	340	37.7	6.84	7.28	2	metabolic	cytoplasm;	catalytic a	Pf00782	11266	ENSG0000	DUSP12	1				0	0.993	0.425941466	2.4	830.4	836.6	17.15	1.88	High	High	High	High	High	High	1	
FALSE	High	Master Prc	O43681	ATPase ASI	0	24.128	16	4	5	4	348	38.8	4.91	17.79	4	transport	cytoplasm;	catalytic a	Pf02374	439	ENSG0000	ASNA1	19			XBP1(S) ac	4	0.993	0.449050947	3.23	1279.7	1324.2	8.39	4.9	High	High	High	High	High	High	1	
FALSE	High	Master Prc	P17980	26S protea	0	134.693	53	18	41	18	439	49.2	5.24	114.85	18	metabolic	cytoplasm;	catalytic a	Pf00004, P	5702	ENSG0000	PSMC3	11	Proteasom	Proteasom	Regulation	153	0.993	0.4513138	3.2	14700.8	14974.1	9.23	2.59	High	High	High	High	High	High	1	
FALSE	High	Master Prc	Q96PZ0	pseudouric	0	15.555	5	2	4	2	661	75	6.37	6.81	2	metabolic	nucleus	catalytic a	Pf01142	54517	ENSG0000	PUS7	7			tRNA modi	3	0.993	0.496092306	139.37	1159.2	1167.7	135.55	63.69	High	High	High	High	High	High	1	
FALSE	High	Master Prc	P35250-1	Replicatior	0	19.074	11	2	3	2	354	39.1	6.44	11.9	2	cell organi	nucleus	catalytic a	Pf00004, P	5982	ENSG0000	RFC2	7	Nucleotide	DNA Repli	Translesior	56	0.993	0.500420361	6.62	406.2	408.7	19.73	5.36	High	High	High	High	High	High	1	
FALSE	High	Master Prc	Q13439-1	Golgin sub	0	4.895	1	2	2	2	2230	261	5.39	3.4	2	cell organi	cytoplasm;	protein bir	Pf01465, P	2803	ENSG0000	GOLGA4	3			Retrograde	4	0.993	0.579196054	16.89	535.3	558.4	13.32	15.21	High	High	High	High	High	High	1	
FALSE	High	Master Prc	Q01518-1	adenylyl cy	0	164.125	51	21	45	21	475	51.9	8.06	127.4	21	cell organi	cytoplasm;	protein bir	Pf01213, P	10487	ENSG0000	CAP1	1		Angiopoiet	Platelet de	13	0.993	0.593321795	4.36	13984.8	14082.4	2.42	2.3	High	High	High	High	High	High	1	
FALSE	High	Master Prc	P56378-1	6.8 kDa mi	0	5.756	28	2	3	2	58	6.7	10.08	4.08	2		membrane		Pf08039	9556	ENSG0000	C14orf2	14				0	0.993	0.59619612	5.72	2252.6	2203.2	12.04	5.41	High	High	High	High	High	High	1	
FALSE	High	Master Prc	O75131	Copine-3 [(	0	53.477	23	8	11	8	537	60.1	5.85	36.4	8	metabolic	cytoplasm;	catalytic a	Pf00168, P	8895	ENSG0000	CPNE3	8			Neutrophil	7	0.993	0.650661673	3.26	2023.7	2012.8	5.36	1.61	High	High	High	High	High	High	1	
FALSE	High	Master Prc	Q9P0J7	E3 ubiquiti	0	28.923	15	3	5	3	381	41.9	5.66	21.02	3	transport	extracellul	catalytic a	Pf00569, P	56888	ENSG0000	KCMF1	2			Neutrophil	3	0.993	0.651056295	23.63	876.2	916.2	20.7	13.51	High	High	High	High	High	High	1	
FALSE	High	Master Prc	P60953	Cell divisio	0	72.197	42	7	37	6	191	21.2	6.55	100.52	7	cell commu	cytoplasm;	catalytic a	Pf00025, P	998	ENSG0000	CDC42	1	Viral carcir	Androgen	RHO GTPa:	102	0.993	0.65793675	2.65	12711.5	12806.8	8.23	2.58	High	High	High	High	High	High	1	
FALSE	High	Master Prc	A5YKK6	CCR4-NOT	0	44.158	5	8	9	8	2376	266.8	7.11	26.97	8	developme	cytoplasm;	catalytic a	Pf04054, P	23019	ENSG0000	CNOT1	16	RNA degra		TP53 regul	10	0.993	0.6615394	10.14	1515.3	1671.5	11.79	5.59	High	High	High	High	High	High	1	
FALSE	High	Master Prc	Q9UBQ0	Vacuolar p	0	30.567	23	4	10	4	182	20.5	6.79	34.83	4	regulation	cytoplasm;	catalytic a	Pf00149, P	51699	ENSG0000	VPS29	12	Endocytosi		WNT ligan	4	0.993	0.691982328	12.24	7163.4	7203.7	10.47	6.33	High	High	High	High	High	High	1	
FALSE	High	Master Prc	Q9H4H8	Protein FA	0.002	4.124	2	1	1	1	585	64.4	6.54	3.35	1	cell divisio	cytoplasm;	protein bir	Pf07894, P	81610	ENSG0000	FAM83D	20				0	0.993	0.702456667	10.86	254	302.3	17.85	11.45	High	High	High	High	High	High	1	
FALSE	High	Master Prc	O75152	Zinc finger	0	25.89	7	4	7	4	810	89.1	8.37	18.09	4	metabolic		metal ion l		9877	ENSG0000	ZC3H11A	1			Cleavage o	9	0.993	0.717849378	23.3	2449.8	2465.9	20.83	2.06	High	High	High	High	High	High	1	
FALSE	High	Master Prc	P13861	cAMP-depe	0	30.855	27	9	13	9	404	45.5	5.07	22.87	9	coagulatio	cytoplasm;	catalytic a	Pf00027, P	5576	ENSG0000	PRKAR2A	3	Insulin sigr	miRs in Mu	DARPP-32	37	0.993	0.986275844	9.55	2815.4	2939.2	7.37	2.15	High	High	High	High	High	High	1	
FALSE	High	Master Prc	P05362	Intercellula	0	35.902	14	6	11	6	532	57.8	7.99	21.73	6	cell commu	cell surface	protein bir	Pf03921, P	3383	ENSG0000	ICAM1	19	Malaria; C	Selenium M	Integrin ce	35	0.992	0.401052954	1.47	1291.1	1384.3	7.66	4.83	High	High	High	High	High	High	1	
FALSE	High	Master Prc	O95292	Vesicle-ass	0	39.488	36	8	14	7	243	27.2	7.3	34.87	8	cell organi	cytoplasm;	protein bir	Pf00635, P	9217	ENSG0000	VAPB	20			Sphingolip	4	0.992	0.476016738	2.58	1506	1551.2	2.77	3.13	High	High	High	High	High	High	1	
FALSE	High	Master Prc	P43686	26S protea	0	77.539	50	16	30	15	418	47.3	5.21	60.67	16	metabolic	cytoplasm;	catalytic a	Pf00004, P	5704	ENSG0000	PSMC4	19; CHR_H	Epstein-Ba	Proteasom	Regulation	152	0.992	0.50358998	5.51	11276.1	11578.7	7.99	3.57	High	High	High	High	High	High	1	
FALSE	High	Master Prc	Q13576-1	Ras GTPase	0	8.94	2	3	5	2	1575	180.5	5.64	1.71	3	cell organi	cell surface	enzyme re	Pf00307, P	10788	ENSG0000	IQGAP2	5	Regulation	G13 Signal	RHO GTPa:	9	0.992	0.525897857	1.24	464.8	472.5	2.49	10.85	High	High	High	High	High	High	1	[R139]
FALSE	High	Master Prc	P22033	Methylmal	0	26.073	12	7	7	7	750	83.1	6.93	13.57	7	metabolic	mitochond	catalytic a	Pf01642, P	4594	ENSG0000	MUT	6	Valine, leu	Vitamin B1	Cobalamin	21	0.992	0.538029873	3.33	4011.4	3965.1	1.64	4.19	High	High	High	High	High	High	1	
FALSE	High	Master Prc	Q9NVP1	ATP-depen	0	87.016	31	18	30	18	670	75.4	9.5	65.41	18	metabolic	chromosor	catalytic a	Pf00270, P	8886	ENSG0000	DDX18	2				0	0.992	0.647517123	12.31	6699.3	6752.9	14.54	4.12	High	High	High	High	High	High	1	
FALSE	High	Master Prc	O60437	periplakin	0	93.342	15	25	29	24	1756	204.6	5.6	60.51	25	cell death;	cytoplasm;	protein bir	Pf04111	5493	ENSG0000	PPL	16			Butyrophil	6	0.992	0.720007078	6.23	2681.8	2721.6	3.17	2.33	High	High	High	High	High	High	1	
FALSE	High	Master Prc	Q96F86	Enhancer c	0	13.212	10	4	5	4	508	56	7.11	13.71	4	cell organi	cytoplasm;	catalytic a	Pf03853, P	80153	ENSG0000	EDC3	15	RNA degra		mRNA dec	4	0.992	0.774093972	9.7	1740	1753.4	6.35	4.47	High	High	High	High	High	High	1	
FALSE	High	Master Prc	Q58FF8	Putative he	0	128.791	27	15	84	1	381	44.3	4.84	202.15	15	metabolic	cytoplasm	nucleotide	Pf00183, P	391634		HSP90AB2	4				0	0.992	0.785516874	18.05	64.2	64.7	4.52	11.1	High	High	High	High	High	High	1	
FALSE	High	Master Prc	Q9UBW7	Zinc finger	0.006	2.475	1	1	1	1	1377	154.8	6.34	2.16	1	cell organi	cytoplasm;	catalytic a	Pf06467, P	7750	ENSG0000	ZMYM2	13			Signaling b	6	0.992	0.839469119	22.68	522.2	526.6	12.44	7.32	High	High	High	High	High	High	1	
FALSE	High	Master Prc	P98082	Disabled h	0	29.4	9	4	5	4	770	82.4	5.53	16.22	4	cell death;	cytoplasm;	protein bir	Pf00640	1601	ENSG0000	DAB2	5	Endocytosi	Endoderm	Cargo reco	11	0.992	0.955778393	30.7	1174.4	1183.6	31.05	3.62	High	High	High	High	High	High	1	
FALSE	High	Master Prc	Q29RF7-1	Sister chro	0	13.924	3	3	3	3	1337	150.7	7.91	7.8	3	cell divisio	chromosor	protein bir		23244	ENSG0000	PDS5A	4			Cohesin Lo	12	0.991	0.449917531	1.54	284.8	267.1	29.76	5.68	High	High	High	High	High	High	1	
FALSE	High	Master Prc	P13489	Ribonuclea	0	83.51	43	14	24	14	461	49.9	4.82	65.32	14	metabolic	cytoplasm;	enzyme re		6050	ENSG0000	RNH1	11; CHR_H				0	0.991	0.475026124	4.31	6311.1	6367.7	25.56	2.02	High	High	High	High	High	High	1	
FALSE	High	Master Prc	Q96RS6	NudC dom	0	11.706	7	3	3	3	583	66.7	5.11	6.15	3		cytoplasm;	protein bir	Pf04969	84955	ENSG0000	NUDCD1	8				0	0.991	0.522786087	6.42	174.3	176.8	31.07	12.91	High	High	High	High	High	High	1	
FALSE	High	Master Prc	Q99961	Endophilin	0	28.885	17	5	7	4	368	41.5	5.43	21.06	5	cell organi	cytoplasm;	protein bir	Pf00018, P	6455	ENSG0000	SH3GL1	19	Endocytosi		Cargo reco	15	0.991	0.526728836	5.45	945.1	953.9	12.6	6.85	High	High	High	High	High	High	1	
FALSE	High	Master Prc	O75150	E3 ubiquiti	0	28.035	9	7	7	5	1001	113.6	6.23	12.84	7	cell organi	cytosol;me	catalytic a	Pf00097, P	9810		RNF40	16		ATM Signa	E3 ubiquiti	5	0.991	0.580485133	15.88	1980.2	1997.4	23.34	4.25	High	High	High	High	High	High	1	
FALSE	High	Master Prc	Q16555-1	Dihydropyr	0	229.082	65	25	87	25	572	62.3	6.38	287.37	25	cell differe	cytoplasm;	catalytic a	Pf01979, P	1808	ENSG0000	DPYSL2	8	Axon guida	Regulation	Recycling p	9	0.991	0.598431153	0.37	22017	22049.9	2.02	2.76	High	High	High	High	High	High	1	[T509(100
FALSE	High	Master Prc	Q13155	aminoacyl	0	15.783	9	3	7	3	320	35.3	8.22	17.16	3	cell death;	cytoplasm;	protein bir	Pf13410, P	7965	ENSG0000	AIMP2	7		Osteoclast	Cytosolic t	10	0.991	0.729457639	8.67	2377.3	2318.5	4.83	8.32	High	High	High	High	High	High	1	
FALSE	High	Master Prc	Q9Y224	UPF0568 p	0	40.381	31	7	13	7	244	28.1	6.65	36.9	7	metabolic	cytoplasm;	protein bir	Pf10036	51637	ENSG0000	C14orf166	14			tRNA proc	3	0.991	0.734845083	13.76	3285.1	3061.4	13.77	5.04	High	High	High	High	High	High	1	
FALSE	High	Master Prc	P29466-1	caspase-1 |	0	12.741	9	3	4	2	404	45.1	5.91	7.84	3	cell death;	cytoplasm;	catalytic a	Pf00619, P	834	ENSG0000	CASP1	11	NOD-like r	Nucleotide	The IPAF ir	31	0.991	0.741357675	7.03	266.6	256.2	11.01	6.71	High	High	High	High	High	High	1	
FALSE	High	Master Prc	Q9BTT0	Acidic leuc	0	46.938	17	4	15	4	268	30.7	3.85	50.78	4	cell organi	cytoplasm;	enzyme re	Pf12799, P	81611	ENSG0000	ANP32E	1				0	0.991	0.767095018	11.52	4005.8	3866.1	11.2	2.88	High	High	High	High	High	High	1	
FALSE	High	Master Prc	P18031	Tyrosine-p	0	14.802	11	4	6	4	435	49.9	6.27	19.79	4	cell organi	cytoplasm;	catalytic a	Pf00102, P	5770	ENSG0000	PTPN1	20	Insulin sigr	Leptin sign	Regulation	28	0.991	0.778327737	0.61	1358.9	1308.4	9.76	10.14	High	High	High	High	High	High	1	
FALSE	High	Master Prc	P35754	Glutaredox	0	28.3	58	4	5	4	106	11.8	8.09	25.65	4	cellular ho	cytoplasm;	catalytic a	Pf00462, P	2745	ENSG0000	GLRX	5		One Carbo	Interconve	4	0.991	0.779742706	18.7	1184.2	1209.3	5.54	11.36	High	High	High	High	High	High	1	
FALSE	High	Master Prc	Q9UKV3-1	Apoptotic (	0	68.156	11	14	29	14	1341	151.8	6.43	54.78	14	cell death;	cytosol;me	catalytic a	Pf02037, P	22985	ENSG0000	ACIN1	14	RNA trans		Apoptotic	7	0.991	0.784646468	4.19	6677.4	6586.9	4.64	6.51	High	High	High	High	High	High	1	
FALSE	High	Master Prc	Q93008-1	Isoform 2 (	0	40.402	5	10	13	10	2554	290.3	5.8	31.11	10	cell divisio	cytoplasm;	catalytic a	Pf00443, P	8239	ENSG0000	USP9X	X				0	0.991	0.976580304	7.39	1976.5	1966	8.54	14.75	High	High	High	High	High	High	1	

FALSE	High	Master Prc	P30626-1	Sorcin [OS	0	48.002	48	7	20	7	198	21.7	5.59	55.03	7	metabolic	cytoplasm;	catalytic a	Pf13405, P	6717	ENSG0000	SRI	7			Ion homeo	14	0.99	0.416138703	2.85	5508.1	5458.6	9.86	5.27	High	High	High	High	High	High	1	
FALSE	High	Master Prc	Q12802-1	A-kinase a	0	47.575	6	11	11	11	2813	307.4	5.24	16.91	11	cell differe	cytoplasm;	catalytic a	Pf00130, P	11214	ENSG0000	AKAP13	15		G Protein S	G alpha (1	12	0.99	0.540005714	6.36	2639.6	2694	7.41	2.37	High	High	High	High	High	High	1	
FALSE	High	Master Prc	P35527	Keratin, ty	0	86.682	25	8	15	7	623	62	5.24	48.94	8	cell death;	cytosol;me	structural	Pf00038, P	3857	ENSG0000	KRT9	17			Formation	3	0.99	0.540736731	6.06	3669.6	3664.7	18.02	9.23	High	High	High	High	High	High	1	
FALSE	High	Master Prc	A3KN83-1	protein str	0	12.393	2	3	3	3	1393	154.2	7.88	2.76	3	regulation			Pf09595, P	55206	ENSG0000	SBNO1	12				0	0.99	0.690213553	18.16	434.7	438.9	19	1.88	High	High	High	High	High	High	1	
FALSE	High	Master Prc	Q9NS87	Kinesin-like	0	6.628	1	1	1	1	1388	160.1	6	2.79	1	cell prolife	cytoplasm;	catalytic a	Pf00225, P	56992	ENSG0000	KIF15	3; CHR_HG		Gastric Ca	COPI-depe	12	0.99	0.842524675	41.64	101.5	112.7	28.55	8.46	High	High	High	High	High	High	1	
FALSE	High	Master Prc	P63010-1	AP-2 comp	0	95.813	30	21	35	14	937	104.5	5.38	69.39	21	cell organi:	cytosol;me	protein bir	Pf01602, P	163	ENSG0000	AP2B1	17	Synaptic ve	Synaptic V	Cargo reco	48	0.99	0.847325469	20.93	5947.7	6005.3	10.58	8.51	High	High	High	High	High	High	1	
FALSE	High	Master Prc	P11388-1	DNA topoi	0	10.545	5	6	6	6	1531	174.3	8.72	8.82	6	cell differe	chromosor	catalytic a	Pf00204, P	7153	ENSG0000	TOP2A	17	Platinum d	Gastric Ca	SUMOylati	14	0.99	0.858975427	0.84	448.5	455.7	1.46	3.93	High	High	High	High	High	High	1	[K1459]
FALSE	High	Master Prc	P07602-1	Prosaposin	0	115.714	37	16	63	16	524	58.1	5.17	152.26	16	cell differe	extracellul	catalytic a	Pf02199, P	5660	ENSG0000	PSAP	10	Lysosome		Platelet de	17	0.99	0.90841816	17.89	31416.1	30843.5	5.71	11.33	High	High	High	High	High	High	1	
FALSE	High	Master Prc	Q96JB2-1	Conserved	0	8.095	6	3	3	3	828	94	5.57	2.02	3	cell organi:	Golgi;mem	protein bir	Pf04136	83548	ENSG0000	COG3	13			COPI-medi	11	0.99	0.980813013	9.81	11.9	12.8	23.63	17.86	High	High	High	High	High	High	1	
FALSE	High	Master Prc	Q969U7	Proteasom	0	6.163	8	2	2	2	264	29.4	6.98	3.94	2	cell organi:	cytosol;nu	protein bir	Pf09754	56984	ENSG0000	PSMG2	18				0	0.989	0.298500591	0.63	757.9	763.2	3.59	7.17	High	High	High	High	High	High	1	
FALSE	High	Master Prc	Q9H0C8	Integrin-lir	0	17.433	11	4	6	4	392	42.9	7.09	10.64	4	metabolic	cytoplasm	catalytic a	Pf00481	80895	ENSG0000	ILKAP	2				0	0.989	0.349474785	1.84	817.2	883.2	8.26	9.72	High	High	High	High	High	High	1	
FALSE	High	Master Prc	P35232	Prohibitin	0	81.512	61	13	32	13	272	29.8	5.76	86.07	13	cell differe	cell surface	DNA bindi	Pf01145	5245	ENSG0000	PHB	17		Integrated	Paradoxica	19	0.989	0.385117896	2.43	8085.1	8350.7	2.98	2.8	High	High	High	High	High	High	1	
FALSE	High	Master Prc	Q6PGP7	Tetratricop	0	13.532	4	6	6	6	1564	175.4	7.53	5.71	6	metabolic	cytoplasm;	protein bir	Pf00515, P	9652	ENSG0000	TTC37	5	RNA degra		mRNA dec	4	0.989	0.414602866	3.04	1813.9	2012.6	7.7	6.43	High	High	High	High	High	High	1	
FALSE	High	Master Prc	O75182-1	paired am	0.003	3.273	1	1	1	1	1162	133	6.93	3.11	1	cell organi:	chromosor	catalytic a	Pf02671, P	23309	ENSG0000	SIN3B	19			NoRC nega	11	0.989	0.427454584	2.89	79.8	90.5	10.56	9.28	High	High	High	High	High	High	1	
FALSE	High	Master Prc	P29692	Elongation	0	100.4	48	11	42	1	281	31.1	5.01	115.23	11	metabolic	cytoplasm;	DNA bindi	Pf00736, P	1936	ENSG0000	EEF1D	8; CHR_HS	Herpes sim	Translatior	Eukaryotic	5	0.989	0.441732022	0.13	1461.6	1392.4	32.01	4.54	High	High	High	High	High	High	1	[S133(100
FALSE	High	Master Prc	O15127	Secretory c	0	11.567	8	2	5	2	329	36.6	6.1	7.82	2	transport	endosome	protein bir	Pf04144	10066	ENSG0000	SCAMP2	15		Serotonin		2	0.989	0.49811751	3.71	1162.7	1155.2	16.2	5.79	High	High	High	High	High	High	1	
FALSE	High	Master Prc	Q13126-1	S-methyl-5	0	19.707	18	3	3	3	283	31.2	7.18	13.49	3	metabolic	cytoplasm;	catalytic a	Pf01048	4507	ENSG0000	MTAP	9	Cysteine a		Gene and	12	0.989	0.606106572	28.4	1243.5	1256.9	35.32	3.43	High	High	High	High	High	High	1	
FALSE	High	Master Prc	O43290	U4/U6.U5	0	57.023	20	11	16	11	800	90.2	6.13	36.4	11	cell organi:	cytoplasm;	protein bir	Pf03343	9092	ENSG0000	SART1	11	Spliceosom		mRNA Spli	5	0.989	0.61414569	20.05	1950.7	2069.6	22.85	11.38	High	High	High	High	High	High	1	
FALSE	Medium	Master Prc	O95998-4	Isoform D	0.015	1.789	6	1	2	1	163	17.5	8.12	1.85	1	regulation	extracellul	protein bir	Pf05566	10068	ENSG0000	IL18BP	11				0	0.989	0.6780317	15.17	864.3	974	19.16	6.39	High	High	High	High	High	High	1	[Q162]
FALSE	High	Master Prc	Q9BUP3	Oxidoredu	0	36.488	44	11	14	11	242	27	8.38	21.8	11	cell death;	cytoplasm;	catalytic a	Pf01118, P	10553	ENSG0000	HTATIP2	11				0	0.989	0.696558703	9.3	3838.3	3898.8	5.17	3.55	High	High	High	High	High	High	1	
FALSE	High	Master Prc	Q14103-3	Isoform 3	0	144.729	61	22	67	2	306	32.8	8.16	150.64	22	metabolic	cytosol;nu	DNA bindi	Pf00076, P	3184	ENSG0000	HNRNPD	4		mRNA Pro	AUF1 (hnR	4	0.989	0.733655183	11.89	675	682.8	7.5	5.49	High	High	High	High	High	High	1	[Q268;
FALSE	High	Master Prc	Q16719	kynurenina	0	51.269	32	8	13	8	465	52.3	7.03	46.6	8	metabolic	cytoplasm;	catalytic a	Pf00266	8942	ENSG0000	KYNU	2	Tryptopha	Selenium M	Tryptopha	9	0.989	0.750433133	20.59	5502.8	5566.6	12.66	8.47	High	High	High	High	High	High	1	
FALSE	High	Master Prc	P08754	Guanine nu	0	58.102	37	8	16	5	354	40.5	5.69	46.36	8	cell divisio	cytoplasm;	catalytic a	Pf00025, P	2773	ENSG0000	GNAI3	1	Chagas dis	Human Th	G alpha (z)	65	0.989	0.815813463	4.66	1310.4	1232.6	7.09	7.05	High	High	High	High	High	High	1	
FALSE	High	Master Prc	P48556	26S protea	0	19.598	15	5	6	5	350	39.6	9.7	10.62	5	cell organi:	cytosol;nu	catalytic a	Pf03399, P	5714	ENSG0000	PSMD8	19	Proteasom	Proteasom	Regulation	152	0.989	0.830851662	7.62	2450.9	2479.3	9.05	0.69	High	High	High	High	High	High	1	
FALSE	High	Master Prc	O75794	Cell divisio	0	16.409	15	4	5	4	336	39.1	4.81	11.24	4	cell divisio	cytoplasm		Pf07065	8872	ENSG0000	CDC123	10				0	0.989	0.926567934	53.49	1247	1390.8	33.97	20.91	High	High	High	High	High	High	1	
FALSE	High	Master Prc	P05091	Aldehyde c	0	75.113	33	13	21	12	517	56.3	7.05	56.93	13	metabolic	mitochond	catalytic a	Pf00171	217	ENSG0000	ALDH2	12	Pyruvate n	Fatty Acid	Ethanol ox	24	0.989	0.976876055	31.89	3877.9	3922.1	15.94	8.07	High	High	High	High	High	High	1	
FALSE	High	Master Prc	P25789	Proteasom	0	88.028	58	13	34	13	261	29.5	7.72	80.37	13	metabolic	cytoplasm;	catalytic a	Pf00227, P	5685	ENSG0000	PSMA4	15	Proteasom	Proteasom	Regulation	151	0.988	0.352650679	1.2	7543.8	7758.9	6.4	1.6	High	High	High	High	High	High	1	
FALSE	High	Master Prc	Q12805	EGF-contai	0	19.589	11	3	3	3	493	54.6	5.07	10.45	3	metabolic	cell surface	catalytic a	Pf06247, P	2202	ENSG0000	EFEMP1	2			Molecules	3	0.988	0.429210256	4.35	212.4	215.1	29.19	1.89	High	High	High	High	High	High	1	
FALSE	High	Master Prc	P61966	AP-1 comp	0	12.408	10	1	2	1	158	18.7	5.73	7.43	1	regulation	cytosol;Go	transporte	Pf01217	1174	ENSG0000	AP1S1	7	Lysosome		Nef media	17	0.988	0.543473986	7.75	261.6	264.8	18.09	6	High	High	High	High	High	High	1	
FALSE	High	Master Prc	O43396	Thioredoxi	0	58.463	52	9	16	9	289	32.2	4.96	47.01	9	cellular ho	cytoplasm;	antioxidan	Pf00085, P	9352	ENSG0000	TXNL1	18				0	0.988	0.609939807	6.09	2976.2	3041.5	3.71	3.52	High	High	High	High	High	High	1	
FALSE	High	Master Prc	P61289-1	Proteasom	0	46.338	36	7	19	7	254	29.5	5.95	58.23	7	cell death;	cytoplasm;	enzyme re	Pf02251, P	10197	ENSG0000	PSME3	17	Hepatitis C	Proteasom	Regulation	152	0.988	0.666691593	2.61	2810.7	2804.8	3.79	3.72	High	High	High	High	High	High	1	
FALSE	High	Master Prc	Q5VV42	threonylca	0	12.354	9	4	4	4	579	65.1	7.46	8.03	4	metabolic	endoplasm	catalytic a	Pf00919, P	54901	ENSG0000	CDKAL1	6			tRNA mod	3	0.988	0.759268407	20.53	1406.5	1460.9	12.43	9.93	High	High	High	High	High	High	1	
FALSE	High	Master Prc	Q9BW27	nuclear po	0	13.606	10	5	5	5	656	75	5.55	7.78	5	cell organi:	chromosor	protein bir	Pf07575	79902	ENSG0000	NUP85	17	RNA trans	Integrated	Rev-media	78	0.988	0.812125257	15.5	920.6	1001.7	11.18	7.58	High	High	High	High	High	High	1	
FALSE	Medium	Master Prc	O15258	Protein RE	0.032	1.41	4	1	1	1	196	22.9	9.54	2.36	1	cell organi:	cell surface	protein bir	Pf03248	11079	ENSG0000	RER1	1				0	0.988	0.819575138	15.29	560.4	553.1	4.89	9.08	High	High	High	High	High	High	1	
FALSE	High	Master Prc	O75955	Flotillin-1 [	0	16.258	12	4	6	4	427	47.3	7.49	13.72	4	cell organi:	cytoskelet	protein bir	Pf01145	10211	ENSG0000	FLOT1	6; CHR_HS	Insulin sigr	Angiopoiet	Synaptic a	6	0.988	0.819605439	8.28	1610.1	1629.4	7.26	2.23	High	High	High	High	High	High	1	
FALSE	High	Master Prc	Q9Y3B2	Exosome c	0	11.368	17	2	3	2	195	21.4	8.24	3.89	2	metabolic	cytoplasm;	catalytic a	Pf10447, P	51013	ENSG0000	EXOSC1	10	RNA degra		Major path	15	0.988	0.878751557	16.42	381.1	385.6	6.47	6.07	High	High	High	High	High	High	1	
FALSE	High	Master Prc	O95208-1	epsin-2 [O	0	10.289	3	1	1	1	641	68.4	7.52	5.75	1	cell organi:	cytoplasm;	protein bir	Pf01417, P	22905	ENSG0000	EPN2	17	Endocytosi		Cargo reco	5	0.988	0.920141144	25.66	140.3	141.9	29.71	10.69	High	High	High	High	High	High	1	
FALSE	High	Master Prc	Q9UKK9	ADP-sugar	0	34.02	30	6	10	6	219	24.3	4.94	28.03	6	cell organi:	cytosol;nu	catalytic a	Pf00293	11164	ENSG0000	NUDT5	10	Purine met		Phosphate	6	0.987	0.27244768	1.6	1444.6	1447.4	4.52	2.12	High	High	High	High	High	High	1	
FALSE	High	Master Prc	Q9UBQ7	Glyoxylate	0	5.553	6	1	2	1	328	35.6	7.39	8.49	1	cell organi:	cytoplasm;	catalytic a	Pf00389, P	9380	ENSG0000	GRHPR	9	Pyruvate n		Glyoxylate	7	0.987	0.410960211	2.68	467.7	483.3	5.86	13.41	High	High	High	High	High	High	1	
FALSE	High	Master Prc	Q13492-1	Phosphatic	0	54.233	18	7	10	7	652	70.7	7.9	37.31	7	cell organi:	cell surface	protein bir	Pf01417, P	8301	ENSG0000	PICALM	11			Cargo reco	7	0.987	0.470984747	2.44	1759.8	1747	2.61	4.1	High	High	High	High	High	High	1	
FALSE	High	Master Prc	Q4G0J3	La-related	0	7.783	7	3	3	3	582	66.9	9.55	0	3	metabolic	cytosol;nu	protein bir	Pf00076, P	51574	ENSG0000	LARP7	4				0	0.987	0.585796044	64.34	291.3	345.5	68.41	12.85	High	High	High	High	High	High	1	
FALSE	High	Master Prc	P15374	Ubiquitin c	0	22.97	17	2	3	2	230	26.2	4.92	16.29	2	metabolic	cytoplasm;	catalytic a	Pf01088	7347	ENSG0000	UCHL3	13		Proteasom	Synthesis c	8	0.987	0.626954149	31.7	678.2	708.1	36.51	8.26	High	High	High	High	High	High	1	
FALSE	High	Master Prc	Q9H9A6	Leucine-ric	0	9.857	6	3	3	3	602	68.2	6.43	4.84	3	regulation	membrane	protein bir	Pf12799, P	55631	ENSG0000	LRRC40	1				0	0.987	0.671875189	9.98	331.6	355.4	16.41	4.1	High	High	High	High	High	High	1	
FALSE	High	Master Prc	Q96P48-1	Isoform 1 c	0	20.29	4	3	3	3	1210	136.2	6.29	10.94	3	cell organi:	cytosol;Go	enzyme re	Pf00169, P	116985	ENSG0000	ARAP1	11				0	0.987	0.678798534	33.13	1416.3	1434.8	25.64	17.13	High	High	High	High	High	High	1	
FALSE	High	Master Prc	O14964	Hepatocyt	0	16.842	7	5	6	5	777	86.1	6.16	11.92	5	cell commu	cytoplasm;	catalytic a	Pf00790, P	9146	ENSG0000	HGS	17	Phagosom	TGF-beta S	Cargo reco	27	0.987	0.811659365	7.74	2001.7	2027.8	2.83	3.36	High	High	High	High	High	High	1	
FALSE	High	Master Prc	Q92900	Regulator	0	64.909	15	13	15	13	1129	124.3	6.61	41.24	13	cell organi:	chromosor	catalytic a	Pf04851, P	5976	ENSG0000	UPF1	19	RNA trans		Nonsense	6	0.987	0.909404996	14.89	5130.3	5199.2	6.43	4.78	High	High	High	High	High	High	1	
FALSE	High	Master Prc	Q16512-1	serine/thre	0	11.156	4	2	2	1	942	103.9	6.37	5.41	2	cell death;	cytoplasm;	catalytic a	Pf00069, P	5585	ENSG0000	PKN1	19	Salmonella	Apoptosis-	Activated	9	0.987	0.986137458	31.41	35.5	42.1	25.67	10.55	High	High	High	High	High	High	1	
FALSE	High	Master Prc	P22314	Ubiquitin-l	0	371.999	55	46	126	46	1058	117.8	5.76	382.73	46	metabolic	cytoplasm;	catalytic a	Pf00899, P	7317	ENSG0000	UBA1	X	Ubiquitin r	Proteasom	Synthesis c	13	0.986	0.229269357	0.3	35661.3	35634.1	1.49	1.88	High	High	High	High	High	High	1	
FALSE	High	Master Prc	P62195-1	26S protea	0	76.496	41	12	28	10	406	45.6	7.55	71.44	12	metabolic	cytoplasm;	catalytic a	Pf00004, P	5705	ENSG0000	PSMC5	17	Epstein-Ba	Proteasom	Regulation	152	0.986	0.339409397	0.81	7826.2	8097.1	9.03	2.71	High	High	High	High	High	High	1	
FALSE	High	Master Prc	Q14562	ATP-depen	0	11.345	4	3	4	3	1220	139.2	8.32	5.52	3	metabolic	cytoplasm;	catalytic a	Pf00271, P	1659	ENSG0000	DHX8	17	Spliceosom	mRNA Pro		2	0.986	0.356736131	2	246.7	250.3	11.7	5.57	High	High	High	High	High	High	1	
FALSE	High	Master Prc	P07384	Calpain-1 c	0	32.485	12	7	10	7	714	81.8	5.67	23.99	7	cell death;	cytoplasm;	catalytic a	Pf00648, P	823	ENSG0000	CAPN1	11	Apoptosis;	Integrin-m	Degradatic	17	0.986	0.391894082	1.83	3013.1	3019.3	25.53	5.35	High	High	High	High	High	High	1	
FALSE	High	Master Prc	Q9NSI2-1	protein FA	0	5.683	9	2	3	2	230	25.4	11.08	7.9	2			protein bir	Pf15341	85395	ENSG0000	FAM207A	21				0	0.986	0.42481481	3.94	367.7	373	1.33	4.31	High	High	High	High	High	High	1	
FALSE	High	Master Prc	Q9UHR5-1	SAP30-bin	0	13.107	10	2	4	2	308	33.9	4.84	12.86	2	cell death;	cytoskelet	protein bir	Pf07818	29115	ENSG0000	SAP30BP	17			NoRC nega	4	0.986	0.437175193	4.5	473.5	516.7	6.2	9.05	High	High	High	High	High	High	1	
FALSE	High	Master Prc	Q9UP95	Solute carr	0	9.101	3	2	2	1	1085	120.6	6.44	4.65	2	cell commu	membrane	protein bir	Pf00324, P	6560	ENSG0000	SLC12A4	16			Cation-cou	4	0.986	0.461995221	14.51	48.5	58.5	23.61	33.57	High	High	High	High	High	High	1	
FALSE	High	Master Prc	O95361-1	Tripartite r	0	10.543	4	2	2	2	564	63.9	5.49	2.99	2	cell organi:	cytoplasm;	DNA bindi	Pf00622, P	10626	ENSG0000	TRIM16	17				0	0.986	0.466744386	9.48	455.1	481.6	22.15	10.28	High	High	High	High	High	High	1	
FALSE	High	Master Prc	Q15121	Astrocytic	0	16.125	28	3	4	3	130	15	5.02	8.06	3	cell death;	cytoplasm;	protein bir	Pf01335	8682	ENSG0000	PEA15	1		Apoptosis	RAF-indep	10	0.986	0.487575899	1.65	907.3	914.9	17.57	2.57	High	High	High	High	High	High	1	
FALSE	High	Master Prc	P55735-1	protein SE	0	33.444	38	8	14	8	322	35.5	5.48	27.86	8	cell organi:	chromosor	protein bir	Pf00400	6396	ENSG0000	SEC13	3	mTOR sign	Sterol Reg	COPII (Coa	33	0.986	0.573024459	2.69	2788.4	2711.2	10.42	3.59	High	High	High	High	High	High	1	
FALSE	High	Master Prc	O43809	Cleavage a	0	28.619	31	5	15	5	227	26.2	8.82	20.76	5	cell organi:	nucleus	catalytic a	Pf13869	11051	ENSG0000	NUDT21	16	mRNA surv	mRNA Pro	Cleavage o	13	0.986	0.664015197	20.23	4938.9	5133.7	14.82	11.44	High	High	High	High	High	High	1	
FALSE	High	Master Prc	Q96E11	Ribosome-	0	9.994	10	2	2	2	262	29.3	9.79	5.01	2	cell organi:	mitochond		Pf01765	92399	ENSG0000	MRRF	9			Mitochonc	4	0.986	0.666548933	31.69	390.9	419	28.53	16.66	High	High	High	High	High	High	1	
FALSE	High	Master Prc	P16152	Carbonyl r	0	29.678	23	5	14	4	277	30.4	8.32	37.59	5	cell differe	cytoplasm;	catalytic a	Pf00106, P	873; 5409	ENSG0000	CBR1; SETI	21	Chemical c	NRF2 path	Synthesis c	10	0.986	0.690026376	6.41	4788	4642.6	2.61	5.33	High	High	High	High	High	High	1	
FALSE	High	Master Prc	Q14694	Ubiquitin c	0	25.36	9	5	6	5	798	87.1	5.31	20.83	5	metabolic	cytoplasm;	catalytic a	Pf00443, P	9100	ENSG0000	USP10	16			Terminatic	8	0.986	0.829975065	1.37	1365	1328	5.04	4.13	High	High	High	High	High	High	1	
FALSE	High	Master Prc	Q6PK04	Coiled-coil	0	12.003	12	3	5	3	289	33.2	10.93	11.37	3		chromosor	protein bir		339230	ENSG0000	CCDC137	17				0	0.986	0.842015517	29.32	1147.1	1163.2	18.78	6.95	High	High	High	High	High	High	1	
FALSE	High	Master Prc	P25786-1	Proteasom	0	102.621	65	18	43	18	263	29.5	6.61	89.81	18	metabolic	cytoplasm;	catalytic a	Pf00227, P	5682	ENSG0000	PSMA1	11	Proteasom	Proteasom	Regulation	150	0.986	0.875649578	13.77	10363.6	10509.9	9.34	4.71	High	High	High	High	High	High	1	
FALSE	High	Master Prc	Q92552	28S riboso	0	29.652	19	8	9	8	414	47.6	6.18	20.92	8	cell organi:	cytoplasm;	protein bir	Pf10037, P	23107	ENSG0000	MRPS27	5			Mitochonc	6	0.986	0.895342419	11.72	3114.5	3161.7	5.81	4.01	High	High	High	High	High	High	1	
FALSE	High	Master Prc	P61160-1	Actin-relat	0	53.898	26	8	15	8	394	44.7	6.74	53.57	8	cell divisio	cytoplasm;	nucleotide	Pf00022	10097	ENSG0000	ACTR2	2		Associatio	EPHB-med	18	0.986	0.899875655	6	5435.5	5576.3	2.82	5.2	High	High	High	High	High	High	1	
FALSE	High	Master Prc	Q13242	serine/argi	0	45.842	44	11	18	10	221	25.5	8.65	34.5	11	cell organi:	nucleus	protein bir	Pf00076, P	8683	ENSG0000	SRSF9	12	Herpes sim	mRNA Pro	Cleavage o	14	0.986	0.901811298	5.74	2850.3	2889.7	1.99	5.01	High	High	High	High	High	High	1	
FALSE	High	Master Prc	Q9NY33	dipeptidyl	0	54.026	22	15	19	15	737	82.5	5.1	45.93	15	metabolic	cytoplasm;	catalytic a	Pf03571	10072	ENSG0000	DPP3	11				0	0.985	0.315818862	0.71	5192.6	5441.5	2.88	4.76	High	High	High	High	High	High	1	
FALSE	High	Master Prc	O75569-1	Interferon-	0	9.744	12	3	3	3	313	34.4	8.41	5.17	3	metabolic	cytoplasm;	enzyme re	Pf00035, P	8575	ENSG0000	PRKRA	2			MicroRNA	4	0.985	0.468173965	8.16	914	916.6	28.97	2.53	High	High	High	High	High	High	1	
FALSE	High	Master Prc	Q5JWF2-1	Guanine nu	0	70.142	16	11	18	10	1037	111	5.03	41.51	11	metabolic	cytosol;me	catalytic a	Pf00025, P	2778	ENSG0000	GNAS	20		Human Th	G alpha (z)	32	0.985	0.496903967	4.29	2265	2283.3	2.06	1.66	High	High	High	High	High	High	1	
FALSE	High	Master Prc	Q92530	proteasom	0	8.252	14	2	2	2	271	29.8	5.74	3.69	2	metabolic	cytoplasm;	enzyme re	Pf08577, P	9491	ENSG0000	PSMF1	20	Proteasom		Regulation	149	0.985	0.510179757	2.95	423.3	438.1	3.32	1.63	High	High	High	High	High	High	1	
FALSE	High	Master Prc	Q9Y3B3-1	Transmem	0	27.407	27	4	8	4	224	25.2	6.89	33.03	4	transport	endoplasm		Pf01105	51014; 10	ENSG0000	TMED7; TM	5		Regulation	COPI-depe	12	0.985	0.532132702	0.18	2222.7	2134	7.66	4.65	High	High	High	High	High	High	1	
FALSE	High	Master Prc	Q99805	Transmem	0	34.691	13	7	14	7	663	75.7	7.44	34.88	7	transport	endosome		Pf02990, P	9375	ENSG0000	TM9SF2	13				0	0.985	0.560456927	9.41	3559.4	3612.6	10.24	2.11	High	High	High	High	High	High	1	
FALSE	High	Master Prc	Q14C86-1	GTPase-act	0	30.366	5	6	6	6	1478	164.9	5.22	17.81	6	cell organi:	cytosol;en	enzyme re	Pf00616, P	26130	ENSG0000	GAPVD1	9			RAB GEFs	5	0.985	0.593866082	25.85	2902.6	2947.7	33.33	8.59	High	High	High	High	High	High	1	
FALSE	High	Master Prc	P49756-1	RNA-bindi	0	33.622	11	8	13	8	843	100.1	6.32	26.27	8	metabolic	cytoplasm;	protein bir	Pf00076, P	58517	ENSG0000	RBM25	14	Spliceosom			1	0.985	0.598514218	4.95	2799.5	2677.5	8.56	7.34	High	High	High	High	High	High	1	
FALSE	High	Master Prc	Q14315	Filamin-C [	0	138.871	10	22	47	13	2725	290.8	5.97	109.66	22	cell organi:	cytoplasm;	protein bir	Pf00307, P	2318	ENSG0000	FLNC	7	Proteoglyc		Cell-extrac	7	0.985	0.634353033	18.74	4343.3	4411.3	24.44	2.67	High	High	High	High	High	High	1	
FALSE	High	Master Prc	O60749	Sorting ne	0	24.782	13	6	9	4	519	58.4	5.12	16.32	6	cell organi:	cytoplasm;	protein bir	Pf00787, P	6643	ENSG0000	SNX2	5	Endocytosi		Golgi Asso	6	0.985	0.667114723	12.25	1934.6	1932.8	14.94	6.34	High	High	High	High	High	High	1	
FALSE	High	Master Prc	O43795	Unconvent	0	17.548	5	6	6	6	1136	131.9	9.38	7.27	6	cell organi:	cytoplasm;	catalytic a	Pf00063, P	4430	ENSG0000	MYO1B	2				0	0.985	0.681771217	32.18	818.7	830.8	27.81	7.63	High	High	High	High	High	High	1	[R626]
FALSE	High	Master Prc	Q9HAU5	Regulator	0	5.618	1	1	1	1	1272	147.7	5.69	3.68	1	metabolic	cytoplasm;	DNA bindi	Pf02854, P	26019	ENSG0000	UPF2	10	mRNA surv		Regulation	9	0.985	0.692807631	0.08	893.6	895.7	3.27	2.29	High	High	High	High	High	High	1	
FALSE	High	Master Prc	Q9C0J8	pre-mRNA	0	16.317	5	5	6	5	1336	145.8	9.17	11	5	metabolic	nucleus	protein bir	Pf00400	55339	ENSG0000	WDR33	2	mRNA surv		mRNA 3'-e	15	0.985	0.725914621	45.37	1178.6	1264.2	37.27	7.31	High	High	High	High	High	High	1	

FALSE	High	Master Prc	Q3ZCQ8	Mitochonc	0	40.836	21	7	12	7	353	39.6	8.37	23.96	7	cell organi:	membrane	catalytic ac	Pf03031	92609	ENSG0000	TIMM50	19			Mitochonc	2	0.985		0.782195605	4.01	1715.6	1778.3	6.73	3.2	High	High	High	High	High	High	1	
FALSE	High	Master Prc	Q96K17-1	transcripti	0	15.046	27	3	3	2	158	17.3	6.35	9.72	3			protein bir	Pf01849	91408	ENSG0000	BTF3L4	1				0	0.985		0.844742023	5.61	328.6	321.9	3.58	9.87	High	High	High	High	High	High	1	
FALSE	High	Master Prc	P39023	60S riboso	0	177.395	62	26	78	26	403	46.1	10.18	180.81	26	cell organi:	cytoplasm;	protein bir	Pf00297	6122	ENSG0000	RPL3	22	Ribosome	Cytoplasm	Peptide ch	34	0.985		0.845114843	7.96	26460.4	27001.8	0.43	6.6	High	High	High	High	High	High	1	
FALSE	High	Master Prc	Q15437	protein tra	0	60.888	17	9	14	7	767	86.4	6.89	47.33	9	transport	cytoplasm;	metal ion l	Pf00626, P	10483	ENSG0000	SEC23B	20	Protein prc	Sterol Regi		2	0.985		0.878662115	18.57	1517.2	1565.8	14.96	1.34	High	High	High	High	High	High	1	
FALSE	High	Master Prc	Q96T51	RUN and F	0	20.309	7	4	8	4	708	79.8	5.74	14.63	4	metabolic	cytoplasm;	metal ion l	Pf01363, P	80230	ENSG0000	RUFY1	5; CHR_HG	Endocytosi		Synthesis c	6	0.985		0.879560478	27.96	1517.8	1480.9	12.38	16.36	High	High	High	High	High	High	1	
FALSE	High	Master Prc	O94906-1	Pre-mRNA	0	93.542	29	23	34	23	941	106.9	8.25	72.48	23	cell organi:	membrane	protein bir	Pf06424, P	24148	ENSG0000	PRPF6	20	Spliceosom	mRNA Pro	mRNA Spli	7	0.985		0.986001896	13.12	7459.9	7573.1	7.88	3.05	High	High	High	High	High	High	1	
FALSE	High	Master Prc	P49411	elongation	0	157.948	59	22	63	22	452	49.5	7.61	150.47	22	metabolic	membrane	catalytic ac	Pf00009, P	7284		TUFM	16			Mitochonc	4	0.984		0.151214607	0.89	18828	18994.1	0.91	0.46	High	High	High	High	High	High	1	
FALSE	High	Master Prc	P08238	Heat shock	0	649.657	71	68	446	24	724	83.2	5.03	1169.1	68	cell organi:	cell surface	enzyme re	Pf00183, P	3326	ENSG0000	HSP90AB1	6	Prostate ca	NRF2 path	Sema3A P/	42	0.984		0.293796434	0.35	25587.7	25992.1	7.63	1.42	High	High	High	High	High	High	1	
FALSE	High	Master Prc	Q9UHQ9	NADH-cytc	0	11.845	10	2	2	2	305	34.1	9.38	3.14	2	metabolic	membrane	catalytic ac	Pf00175, P	51706	ENSG0000	CYB5R1	1	Amino sug	Oxidation	Platelet de	9	0.984		0.436667452	5.43	808.5	849.2	9.77	5.66	High	High	High	High	High	High	1	
FALSE	High	Master Prc	Q9NWU1-	3-oxoacyl-	0	13.717	9	2	2	2	459	48.8	7.66	8.69	2	metabolic	cytosol;mi	catalytic ac	Pf00108, P	54995	ENSG0000	OXSM	3	Metabolic			4	0.984		0.489672252	18.98	52.3	60.7	9.06	39.36	High	High	High	High	High	High	1	
FALSE	High	Master Prc	Q00765	Receptor e	0	16.119	15	4	8	4	189	21.5	8.1	8.82	4		endoplasm	protein bir	Pf03134	7905	ENSG0000	REEP5	5			Olfactory S	4	0.984		0.490308586	5.17	1299.8	1351.3	5.82	6.18	High	High	High	High	High	High	1	
FALSE	High	Master Prc	Q12788	Transducin	0	39.67	14	6	7	6	808	89	6.9	18.67	6	metabolic	nucleus	protein bir	Pf00400, P	10607	ENSG0000	TBL3	16	Ribosome		Major path	6	0.984		0.55122581	10.87	1088.5	1103.2	26.05	4.46	High	High	High	High	High	High	1	
FALSE	High	Master Prc	Q86V48-1	Leucine zip	0	23.603	6	4	5	4	1076	120.2	8.5	14.86	4	metabolic	membrane		Pf04111	7798	ENSG0000	LUZP1	1				0	0.984		0.551903409	40.14	670.5	681.2	53.66	13.08	High	High	High	High	High	High	1	
FALSE	High	Master Prc	Q8N1P7	beta/gamr	0	9.48	4	1	1	1	616	68.6	5.1	4.68	1				Pf00030, P	55057		AIM1L; CR	1				0	0.984		0.583815295	7.65	305.9	306.6	6.67	1.09	High	High	High	High	High	High	1	
FALSE	High	Master Prc	Q16181	Septin-7 [C	0	29.298	18	8	10	8	437	50.6	8.63	23.83	8	cell differe	chromosor	nucleotide	Pf00735, P	989		SEPT7	7			MAPK6/M.	3	0.984		0.587427043	5.14	3896.1	3960.4	11.8	3.88	High	High	High	High	High	High	1	
FALSE	High	Master Prc	O14880	Microsoma	0	26.343	16	2	3	2	152	16.5	9.38	15.73	2	metabolic	endoplasm	antioxidan	Pf01124	4259	ENSG0000	MGST3	1	Drug meta	NRF2 path	Glutathion	12	0.984		0.689544835	7.25	260.6	228.3	9.06	13.2	High	High	High	High	High	High	1	
FALSE	High	Master Prc	Q8TAT6-1	Nuclear pr	0	31.241	9	4	6	4	608	68.1	6.38	14.62	4	cell organi:	cytoplasm;	metal ion l	Pf05020, P	55666	ENSG0000	NPLOC4	17	Protein prc		Translesior	5	0.984		0.711728166	16.29	1465.2	1390.8	15.39	5.03	High	High	High	High	High	High	1	
FALSE	High	Master Prc	Q8N0V3-1	Putative ril	0	6.011	6	1	1	1	343	38.3	7.85	4.95	1	metabolic	mitochond	catalytic ac	Pf02033, P	79863	ENSG0000	RBFA	18				0	0.984		0.740954833	39.96	382.5	388.8	26.01	19	High	High	High	High	High	High	1	
FALSE	High	Master Prc	Q8WW12	PEST prote	0	59.591	53	8	17	8	178	18.9	7.49	44.86	8	metabolic	nucleus	protein bir	Pf15473	57092	ENSG0000	PCNP	3				0	0.984		0.762719499	20.45	5720.6	5816.3	17.96	3.38	High	High	High	High	High	High	1	
FALSE	High	Master Prc	O94903	Pyridoxal p	0.009	2.128	3	1	1	1	275	30.3	7.5	2.55	1		cytoplasm;		Pf01168	11212	ENSG0000	PROSC; PLI	8				0	0.984		0.836108611	18.15	397.2	403.5	13.16	1.3	High	High	High	High	High	High	1	
FALSE	High	Master Prc	Q9P2N5	RNA-bindi	0	6.535	2	2	2	2	1060	118.6	9.19	1.65	2	metabolic	cytoplasm;	metal ion l	Pf00076, P	54439	ENSG0000	RBM27	5				0	0.984		0.850867592	0.78	2186.4	2118.7	21.11	22.38	High	High	High	High	High	High	1	
FALSE	High	Master Prc	P30837	Aldehyde c	0	18.572	15	6	7	5	517	57.2	6.8	17.58	6	metabolic	mitochond	catalytic ac	Pf00171	219		ALDH1B1	9	Tryptopha		Ethanol ox	17	0.984		0.92205273	31.43	826.2	839.6	21.93	4.28	High	High	High	High	High	High	1	
FALSE	High	Master Prc	Q9UHD1	cysteine ar	0	78.117	48	12	24	12	332	37.5	7.87	71.94	12	metabolic		metal ion l	Pf04968, P	26973	ENSG0000	CHORDC1	11				0	0.984		0.926488378	14.07	7031.2	7147.4	7.02	3.73	High	High	High	High	High	High	1	
FALSE	High	Master Prc	Q6P2E9-1	Enhancer c	0	32.429	5	5	7	5	1401	151.6	5.86	25.06	5	metabolic	cytoplasm;	protein bir	Pf00400	23644	ENSG0000	EDC4	16	RNA degra		mRNA dec	4	0.984		0.965411418	12.94	1403.7	1470.1	12.25	3.28	High	High	High	High	High	High	1	
FALSE	High	Master Prc	Q9NUQ3-1	Gamma-ta	0	16.503	13	5	6	5	528	60.5	7.52	7.84	5	metabolic	cytoplasm;	protein bir	Pf09728	55787	ENSG0000	TXLNG	X				0	0.983		0.340473184	3.57	398.2	405.2	2.02	4.68	High	High	High	High	High	High	1	
FALSE	Medium	Master Prc	Q15047	Histone-lys	0.028	1.457	1	1	2	1	1291	143.1	6.02	1.96	1	cell organi:	chromosor	catalytic ac	Pf00856, P	9869	ENSG0000	SETDB1	1	Lysine deg	Histone M	PKMTs me	6	0.983		0.504950508	22.19	714.4	724.4	3.67	46.01	High	High	High	High	High	High	1	
FALSE	High	Master Prc	P17535	Transcripti	0	17.284	12	3	4	2	347	35.2	7.37	14.5	3	metabolic	chromosor	DNA bindi	Pf00170, P	3727	ENSG0000	JUND	19	MAPK sign	Corticotro		10	0.983		0.509375766	2.54	200.6	199.1	14.29	7.45	High	High	High	High	High	High	1	
FALSE	High	Master Prc	P06733-1	alpha-enol	0	605.086	81	40	410	14	434	47.1	7.39	1039.59	40	metabolic	cell surface	catalytic ac	Pf00113, P	2023	ENSG0000	ENO1	1	Biosynthes	Glycolysis	Gluconeog	12	0.983		0.567317991	7.72	36285	36925.3	4.57	3.09	High	High	High	High	High	High	1	[Y44(100);
FALSE	High	Master Prc	P49459-1	Ubiquitin-c	0	8.082	18	2	2	2	152	17.3	5.15	5.14	2	cell organi:	chromosor	catalytic ac	Pf00179, P	7319	ENSG0000	UBE2A	X	Ubiquitin r		Synthesis c	10	0.983		0.571246778	9.22	134.6	134	20.79	5.09	High	High	High	High	High	High	1	
FALSE	High	Master Prc	Q86UP2-1	Kinectin [C	0	78.895	13	16	22	16	1357	156.2	5.64	55.06	16	cellular cor	endoplasm	protein bir		3895	ENSG0000	KTN1	14			Post-transl	8	0.983		0.574736311	15.3	5137.1	5223.7	28.07	4.48	High	High	High	High	High	High	1	
FALSE	High	Master Prc	Q9H9L3	Interferon-	0.002	3.77	4	1	1	1	353	39.1	9.94	2.89	1	metabolic	nucleus	catalytic ac	Pf00929	81875	ENSG0000	ISG20L2	1			Major path	4	0.983		0.815745932	20.18	225.7	229.6	9.07	22.71	High	High	High	High	High	High	1	
FALSE	High	Master Prc	Q9UJX5	Anaphase-	0	5.99	2	1	1	1	808	92.1	5.53	4.34	1	cell divisio	cytosol;nu	catalytic ac	Pf12894, P	29945	ENSG0000	ANAPC4	4	HTLV-I infe		APC/C:Cdc	37	0.983		0.823103989	37.3	443.1	451	28.14	8.94	High	High	High	High	High	High	1	
FALSE	High	Master Prc	P24928	DNA-direct	0	27.798	3	3	4	3	1970	217	7.37	17.6	3	metabolic	cytoplasm;	catalytic ac	Pf00623, P	5430		POLR2A	17	Metabolic	Eukaryotic	Signaling b	77	0.983		0.848872788	1.74	730.1	705.5	3.92	4.43	High	High	High	High	High	High	1	
FALSE	High	Master Prc	Q9Y2R5	28S riboso	0	35.102	52	5	6	5	130	14.5	9.85	23.99	5	cell organi:	membrane	RNA bindi	Pf00366	51373	ENSG0000	MRPS17	7	Ribosome		Mitochonc	7	0.983		0.873980675	8	1615.9	1655.6	2.43	5.46	High	High	High	High	High	High	1	
FALSE	High	Master Prc	O60884	DnaJ homc	0	45.983	31	9	11	9	412	45.7	6.48	25.48	9	metabolic	cytosol;me	enzyme re	Pf00226, P	10294	ENSG0000	DNAJA2	16	Protein prc		HSP90 cha	4	0.983		0.88415284	11.73	3304.8	3362.8	8.95	3.23	High	High	High	High	High	High	1	
FALSE	Low	Master Prc	O95490-1	Adhesion (	0.062	1.108	1	1	1	1	1459	163.2	6.43	0	1	regulation	membrane	receptor a	Pf00002, P	23266	ENSG0000	LPHN2; AD	1		GPCRs, Otl		2	0.982		0.427285166	5.44	1160.3	1181.8	2.78	12.73	High	High	High	High	High	High	1	[R743]
FALSE	High	Master Prc	Q99653	Calcineurir	0.002	3.728	8	1	2	1	195	22.4	5.1	4.04	1	cell organi:	cytoplasm;	enzyme re	Pf00036, P	11261	ENSG0000	CHP1	15		Alzheimers	Hyalurona	7	0.982		0.461343974	7.09	614.7	626	9.56	4.74	High	High	High	High	High	High	1	
FALSE	High	Master Prc	Q12893	Transmem	0	8.133	5	1	1	1	351	38.2	8.16	3.54	1	metabolic	Golgi;mem	catalytic ac	Pf08551	11070	ENSG0000	TMEM115	3			COPI-medi	8	0.982		0.464039765	12.66	375.8	399.1	21.65	22.63	High	High	High	High	High	High	1	
FALSE	High	Master Prc	P54136-1	arginine--t	0	134.514	39	21	42	21	660	75.3	6.68	119.02	21	metabolic	cytoplasm;	catalytic ac	Pf00750, P	5917	ENSG0000	RARS	5	Aminoacyl	Amino Acid	Cytosolic t	10	0.982		0.507379032	6.79	10646	10842.5	2.4	4.46	High	High	High	High	High	High	1	
FALSE	High	Master Prc	Q15029	116 kDa U	0	206.416	40	31	72	30	972	109.4	5	213.5	31	cell organi:	cytosol;me	catalytic ac	Pf00009, P	9343	ENSG0000	EFTUD2	17	Spliceosom	mRNA Pro	mRNA Spli	7	0.982		0.539422584	6.84	17408.8	17865.7	2.41	3.7	High	High	High	High	High	High	1	
FALSE	High	Master Prc	Q9UNF0	Protein kin	0	25.321	17	7	7	7	486	55.7	5.2	17.24	7	cell organi:	cytoplasm;	protein bir	Pf00018, P	11252	ENSG0000	PACSIN2	22			Clathrin-m	3	0.982		0.684641589	21.16	1715.1	1696.3	24.05	2.44	High	High	High	High	High	High	1	
FALSE	High	Master Prc	Q00534	Cyclin-dep	0	67.874	43	10	23	10	326	36.9	6.46	60.05	10	cell differe	cytoplasm;	catalytic ac	Pf00069, P	1021	ENSG0000	CDK6	7	Glioma; M	miRNA Re	Regulation	40	0.982		0.733827677	12.78	6160.8	6274.6	7.69	2.45	High	High	High	High	High	High	1	
FALSE	High	Master Prc	O95817	BAG family	0	63.81	33	14	18	14	575	61.6	6.95	49.91	14	cell death;	cytoplasm;	enzyme re	Pf00397, P	9531	ENSG0000	BAG3	10			Regulation	4	0.982		0.799124804	59.69	1666.8	1698	32.8	17.87	High	High	High	High	High	High	1	
FALSE	High	Master Prc	Q9P086	Mediator c	0	10.974	34	2	2	2	117	13.1	5.96	3.55	2	metabolic	nucleus	catalytic ac	Pf10280	400569	ENSG0000	MED11	17			Transcripti	6	0.982		0.817020312	33.17	387.9	391.4	23.81	9.43	High	High	High	High	High	High	1	
FALSE	High	Master Prc	P33527	Multidrug	0	10.213	3	4	5	4	1531	171.5	7.11	4.48	4	cell organi:	membrane	catalytic ac	Pf00005, P	4363	ENSG0000	ABCC1	16; CHR_H	Vitamin di	Irinotecan	Cobalamin	16	0.982		0.908153893	21.73	105.3	105.5	11.88	7.37	High	High	High	High	High	High	1	
FALSE	High	Master Prc	P55039	Developme	0	26.61	16	5	7	4	364	40.7	8.88	16.64	5	regulation	cytoplasm;	nucleotide	Pf01926, P	1819	ENSG0000	DRG2	17				0	0.982		0.908332953	18.9	2087.2	2259.1	12.61	4.96	High	High	High	High	High	High	1	
FALSE	High	Master Prc	P09972	Fructose-b	0	131.4	46	16	55	10	364	39.4	6.87	166.59	16	cell differe	cytoskelet	catalytic ac	Pf00274	230	ENSG0000	ALDOC	17	Glycolysis ,	Glycolysis a	Gluconeog	15	0.982		0.957047274	5.2	8096.9	7652.5	4.45	7	High	High	High	High	High	High	1	
FALSE	High	Master Prc	Q9H773	dCTP pyro	0	13.843	34	3	3	3	170	18.7	5.03	7.73	3	cell commi	cytoplasm;	catalytic ac		79077	ENSG0000	DCTPP1	16	Metabolic		Interconve	5	0.981		0.311223924	0.99	568.9	590.1	1.44	7.49	High	High	High	High	High	High	1	
FALSE	High	Master Prc	O75521	Enoyl-CoA	0	26.103	21	6	7	6	394	43.6	9	20.51	6	metabolic	membrane	catalytic ac	Pf00378, P	10455	ENSG0000	ECI2	6	Peroxisom			2	0.981		0.399406723	4.29	1676.6	1836.4	11.36	5.89	High	High	High	High	High	High	1	
FALSE	High	Master Prc	A6NED2	RCC1 dom	0	5.362	5	1	1	1	376	40.1	5.27	5.48	1	cell organi:	chromosor		Pf00415, P	91433	ENSG0000	RCCD1	15				0	0.981		0.44210259	8.68	250.8	291.8	19.42	17.23	High	High	High	High	High	High	1	
FALSE	High	Master Prc	Q15019	septin-2 [C	0	65.379	41	10	15	10	361	41.5	6.6	49.75	10	cell differe	chromosor	nucleotide	Pf00071, P	4735	ENSG0000	SEPT2	2	Bacterial ir		Anchoring	4	0.981		0.487460152	0.4	6436.1	6028.9	15.66	8.16	High	High	High	High	High	High	1	
FALSE	High	Master Prc	Q16630	Cleavage a	0	72.226	22	9	21	9	551	59.2	7.15	61.22	9	cell organi:	membrane	protein bir	Pf00076, P	11052	ENSG0000	CPSF6	12	mRNA surv		Signaling b	7	0.981		0.546007862	5.04	7157.7	7298.8	2.64	1.29	High	High	High	High	High	High	1	
FALSE	High	Master Prc	Q9BXY0	Protein MA	0	6.384	5	1	2	1	300	35.3	5.38	10.88	1	metabolic	nucleus	RNA bindi	Pf01778, P	84549	ENSG0000	MAK16	8				0	0.981		0.642361718	2.63	482.5	491.9	11.2	1.45	High	High	High	High	High	High	1	
FALSE	High	Master Prc	Q96G74-1	OTU doma	0	29.137	12	4	6	4	571	60.6	6.54	31.09	4	metabolic	cytosol	catalytic ac	Pf02338	55593	ENSG0000	OTUD5	X	RIG-I-like r	Regulation	Ovarian tu	12	0.981		0.713556043	23.04	1510.6	1539.1	24.58	0.84	High	High	High	High	High	High	1	
FALSE	High	Master Prc	Q8WWM7	ataxin-2-lil	0	41.914	14	13	22	12	1075	113.3	8.59	50.46	13	cell organi:	cytoplasm;	protein bir	Pf06741, P	11273	ENSG0000	ATXN2L	16				0	0.981		0.725252216	19.3	5899.2	6354	23.22	3.18	High	High	High	High	High	High	1	
FALSE	High	Master Prc	Q8NFF5	FAD syntha	0	24.068	13	4	4	4	587	65.2	6.93	17.33	4	metabolic	cytoplasm;	catalytic ac	Pf00994, P	80308	ENSG0000	FLAD1	1	Riboflavin	Selenium M	Vitamin B2	8	0.981		0.729843055	7.87	676.6	642.5	8.13	8.61	High	High	High	High	High	High	1	
FALSE	High	Master Prc	Q86Y79	Probable p	0	5.45	5	1	3	1	214	22.9	10.56	10.53	1		mitochond	catalytic ac	Pf01195	138428	ENSG0000	PTRH1	9				0	0.981		0.921459923	10.68	907.9	911.7	6.58	6.01	High	High	High	High	High	High	1	
FALSE	High	Master Prc	P78347	General tra	0	60.753	22	18	26	18	998	112.3	6.39	35.99	18	cellular ho	cytoplasm;	DNA bindi	Pf02946	2969	ENSG0000	GTF2I	7	Basal trans	B Cell Rece		4	0.981		0.922743442	7.49	6957.7	6492.7	7.95	5.63	High	High	High	High	High	High	1	
FALSE	Medium	Master Prc	P15954	Cytochrom	0.024	1.531	14	1	1	1	63	7.2	10.27	2.46	1	metabolic	membrane	catalytic ac	Pf02935	1350	ENSG0000	COX7C	5	Metabolic	Electron Tr	TP53 Regu	17	0.981		0.977922877	20.04	627.7	662	7.18	13.86	High	High	High	High	High	High	1	
FALSE	High	Master Prc	Q8NEM2	SHC SH2 dc	0	14.612	8	4	4	4	672	75.6	4.75	8.02	4	regulation	cytoplasm;	protein bir	Pf05048, P	79801	ENSG0000	SHCBP1	16				0	0.981		0.989132081	16.51	535.2	474.8	17.69	11.27	High	High	High	High	High	High	1	
FALSE	High	Master Prc	O43747-1	AP-1 comp	0	14.868	4	3	5	3	822	91.3	6.8	7.72	3	cell organi:	cytoplasm;	protein bir	Pf01602, P	164	ENSG0000	AP1G1	16	Lysosome		Nef media	17	0.98		0.31970776	1.78	1675.5	1705.1	13.29	4.87	High	High	High	High	High	High	1	
FALSE	High	Master Prc	O15355	Protein ph	0	61.814	28	11	22	11	546	59.2	4.36	52.96	11	metabolic	cytoplasm;	catalytic ac	Pf00481	5496	ENSG0000	PPM1G	2		mRNA Pro		1	0.98		0.360013121	3.96	7773.5	7848.9	6.02	0.68	High	High	High	High	High	High	1	
FALSE	High	Master Prc	Q8NI36	WD repeat	0	26.12	6	4	6	4	951	105.3	7.53	20.42	4	metabolic	nucleus	protein bir	Pf00400, P	134430	ENSG0000	WDR36	5	Ribosome		Major path	6	0.98		0.557605866	3.9	1834.1	1843.3	2.46	2.55	High	High	High	High	High	High	1	
FALSE	High	Master Prc	P27695	DNA-(apur	0	56.29	43	11	16	11	318	35.5	8.12	35.98	11	cell organi:	cytoplasm;	catalytic ac	Pf03372, P	328	ENSG0000	APEX1	14	Base excisi	Human Th	POLB-Depe	12	0.98		0.574354306	7.83	4101	4019.2	6.79	2.73	High	High	High	High	High	High	1	
FALSE	High	Master Prc	Q96BP2	Coiled-coil	0	4.558	9	1	1	1	118	13.5	10.21	2.22	1	cell organi:	cytosol;me	protein bir	Pf06747	118487	ENSG0000	CHCHD1	10			Mitochonc	6	0.98		0.595801133	23.42	632.5	645.3	31.01	7.9	High	High	High	High	High	High	1	
FALSE	High	Master Prc	P06493	Cyclin-dep	0	60.078	51	11	18	10	297	34.1	8.4	46.66	11	cell death;	cytoplasm;	catalytic ac	Pf00069, P	983	ENSG0000	CDK1	10	Viral carcir	miRNA Re	Regulation	95	0.98		0.716104533	5.96	1661.9	1653.9	3.23	10.1	High	High	High	High	High	High	1	
FALSE	High	Master Prc	Q8NCG7	Sn1-specifi	0	19.735	6	3	3	3	672	73.7	6.55	12.81	3	cell differe	membrane	catalytic ac	Pf01764	221955	ENSG0000	DAGLB	7	Retrograde		Arachidon:	11	0.98		0.721583303	27.11	930.1	957.1	16.87	12.39	High	High	High	High	High	High	1	
FALSE	High	Master Prc	Q13435	Splicing fa	0	153.625	34	31	69	31	895	100.2	5.67	151.53	31	metabolic	nucleus;sp	protein bir	Pf02037, P	10992	ENSG0000	SF3B2	11	Spliceosom	mRNA Pro	mRNA Spli	7	0.98		0.792242255	1.52	17729.4	17967.5	4.26	2.02	High	High	High	High	High	High	1	
FALSE	High	Master Prc	Q9UK22	F-box only	0	38.333	29	6	9	6	296	33.3	4.37	32.05	6	metabolic	cytoplasm;	catalytic ac	Pf04300	26232	ENSG0000	FBXO2	1	Ubiquitin r		Antigen pr	9	0.98		0.892242266	10.12	3517.1	3437.9	9.94	3.87	High	High	High	High	High	High	1	
FALSE	High	Master Prc	O60841	Eukaryotic	0	45.386	9	8	13	8	1220	138.7	5.49	36.04	8	metabolic	cytoplasm;	catalytic ac	Pf00009, P	9669	ENSG0000	EIF5B	2	RNA trans	Translatior	GTP hydro	7	0.98		0.907883568	34.2	3052.9	3219.2	22.52	5.38	High	High	High	High	High	High	1	
FALSE	High	Master Prc	Q9BYG3	MKI67 FHA	0	18.458	19	5	5	5	293	34.2	9.88	12.75	5	cell organi:	chromosor	protein bir	Pf00076, P	84365	ENSG0000	MKI67IP; N	2				0	0.979		0.330262765	1.11	1133	1176.8	15.73	6.4	High	High	High	High	High	High	1	
FALSE	High	Master Prc	Q9NW82	WD repeat	0	32.372	14	7	8	7	654	73.2	6.33	16.26	7			protein bir	Pf00400	55100	ENSG0000	WDR70	5				0	0.979		0.382483754	3.82	1913.5	2055.5	9.96	3.62	High	High	High	High	High	High	1	
FALSE	High	Master Prc	Q9BUI4	DNA-direct	0	7.131	5	2	2	2	534	60.6	7.31	2.24	2	defense re	cytosol;nu	catalytic ac	Pf04929, P	10623	ENSG0000	POLR3C; LI	1	Metabolic		RNA Polym	18	0.979		0.387737686	1	22.7	24.3	36.73	3.38	High	High	High	High	High	High	1	
FALSE	High	Master Prc	Q9P003	Protein cor	0	16.812	14	1	4	1	139	16.1	6.65	21.18	1	transport	endoplasm	protein bir	Pf03311	29097	ENSG0000	CNIH4	1				0	0.979		0.415772864	5.48	955.7	980.5	3.12	2.82	High	High	High	High	High	High	1	
FALSE	High	Master Prc	Q6P6C2-1	Isoform 1 c	0	13.963	9	2	3	2	467	52.2	8.51	5.57	2	cell differe	cytosol;Go	catalytic ac	Pf13532	54890		ALKBH5	17				0	0.979		0.466882312	10.72	207.4	230.5	20.49	9.9	High	High	High	High	High	High	1	
FALSE	High	Master Prc	Q9C0C7-1	Activating	0	9.559	2	1	1	1	1298	142.4	7.18	4.61	1	cell commi	cytoplasm;	protein bir	Pf00400	55626	ENSG0000	AMBRA1	11		Nanoparti	Macroautc	4	0.979		0.504190728	5.19	69.4	69.4	26.73	3.27	High	High	High	High	High	High	1	
FALSE	High	Master Prc	Q969M3	Protein YIF	0	5.027	8	2	2	2	257	28	4.36	4.15	2	regulation	endoplasm	catalytic ac	Pf01694	81555	ENSG0000	YIPF5	5				0	0.979		0.529213229	0.77	658.8	633	8.15	9.96	High	High	High	High	High	High	1	
FALSE	High	Master Prc	Q7Z2W4	zinc finger	0	34.394	14	8	9	8	902	101.4	8.4	28.22	8	defense re	cytoplasm;	catalytic ac	Pf00644	56829	ENSG0000	ZC3HAV1	7			Signaling b	4	0.979		0.578808778	22.67	1746.3	1784	36.25	4.15	High	High	High	High	High	High	1	
FALSE	High	Master Prc	Q8NEZ5-1	F-box only	0	8.145	12	3	3	3	403	44.5	7.03	4.46	3	cell commi	cytoplasm;	catalytic ac	Pf00646, P	26263	ENSG0000	FBXO22	15			Antigen pr	7	0.979		0.603423129	40.39	1030.3	1168.4	52.56	7.11	High	High	High	High	High	High	1	

FALSE	High	Master Prc	Q8WUW1	Protein BR	0	4.643	25	2	2	2	75	8.7	5.45	3.14	2	cell organi	cytoplasm;	protein bir		55845	ENSG0000	BRK1	3	Regulation	Regulation	Regulation	13	0.979		0.612726485	10.58	1525.5	1557.8	15.71	7.41	High	High	High	High	High	High	1	
FALSE	High	Master Prc	Q92989-1	Polyribonu	0	6.548	5	2	2	2	425	47.6	6.62	5.36	2	cell organi	cytosol;nu	catalytic a	Pf03205, P	10978	ENSG0000	CLP1	11	mRNA surv	mRNA Pro	Cleavage o	15	0.979		0.684124027	24.96	775.1	792	12.52	15.91	High	High	High	High	High	High	1	
FALSE	High	Master Prc	P13798	Acylamino	0	34.015	14	7	10	7	732	81.2	5.48	26.85	7	cell organi	cytoplasm;	catalytic a	Pf00326, P	327	ENSG0000	APEH	3			Eukaryotic	6	0.979		0.781756995	3.03	2658.2	2771.2	12.24	8.62	High	High	High	High	High	High	1	
FALSE	High	Master Prc	O00505	Importin su	0	8.054	7	3	4	1	521	57.8	4.94	12.39	3	cell organi	cytoplasm;	protein bir	Pf00514, P	3839	ENSG0000	KPNA3	13			NS1 Media	10	0.979		0.8380377	4.91	335.2	328.5	5.75	4.51	High	High	High	High	High	High	1	
FALSE	High	Master Prc	P50990	T-complex	0	233.157	64	37	90	37	548	59.6	5.6	198.88	37	cell organi	cytoplasm;	catalytic a	Pf00118	10694	ENSG0000	CCT8	21			BBSome-m	16	0.978		0.23233904	2.67	27388.6	28268.9	3.61	1.11	High	High	High	High	High	High	1	
FALSE	High	Master Prc	Q9Y580	RNA-bindi	0	15.957	19	3	3	3	266	30.5	9.57	7.84	3	regulation	nucleus	protein bir	Pf00076, P	10179	ENSG0000	RBM7	11				0	0.978		0.384094304	5.14	477.3	484	2.18	4.07	High	High	High	High	High	High	1	
FALSE	High	Master Prc	P49023	Paxillin [OS	0	23.772	15	5	5	5	591	64.5	6.19	13.73	5	cell organi	cytoplasm;	DNA bindi	Pf00412, P	5829	ENSG0000	PXN	12	Leukocyte	Integrin-m	Localizatio	33	0.978		0.396832121	4.69	1100.7	1237.1	11.66	8.52	High	High	High	High	High	High	1	
FALSE	High	Master Prc	Q04760-1	lactoylglut	0	25.915	39	8	9	8	184	20.8	5.31	17.97	8	cell differe	cytoplasm;	catalytic a	Pf00903, P	2739	ENSG0000	GLO1	6	Pyruvate n		Pyruvate n	5	0.978		0.405106166	5.46	3359.8	3436.2	11.64	2.89	High	High	High	High	High	High	1	
FALSE	High	Master Prc	O94979	Protein tra	0	97.363	19	18	28	18	1220	132.9	6.89	79.13	18	cell organi	cytoplasm;	protein bir	Pf03154, P	22872	ENSG0000	SEC31A	4	Protein pro	Sterol Reg	Antigen Pr	18	0.978		0.619401176	11.63	8529.7	8314.6	10.15	5.43	High	High	High	High	High	High	1	
FALSE	High	Master Prc	Q86VM9	Zinc finger	0	24.89	9	7	10	7	953	106.3	8.32	15.8	7		nucleus	metal ion l	Pf00642	124245	ENSG0000	ZC3H18	16				0	0.978		0.63488812	6.12	2964.1	3009.5	11.36	5.64	High	High	High	High	High	High	1	[T611(100
FALSE	High	Master Prc	Q13630	GDP-L-fucc	0	7.258	7	2	2	2	321	35.9	6.6	4.8	2	metabolic	cytoplasm;	catalytic a	Pf01073, P	7264	ENSG0000	TSTA3	8; CHR_HS	Fructose a		GDP-fucos	9	0.978		0.687133736	19.07	1304	1336.2	25.73	11.23	High	High	High	High	High	High	1	
FALSE	High	Master Prc	Q9Y2A7-1	Nck-associ	0	13.995	4	4	4	4	1128	128.7	6.62	10.56	4	cell death;	cytosol;me	protein bir	Pf09735	10787	ENSG0000	NCKAP1	2	Regulation	Exercise-in	Regulation	14	0.978		0.712691628	2.78	537	547.6	3.54	5.3	High	High	High	High	High	High	1	
FALSE	High	Master Prc	Q15942	Zyxin [OS=	0	45.563	29	11	15	11	572	61.2	6.67	24.81	11	cell commu	cytoplasm;	metal ion l	Pf00412	7791	ENSG0000	ZYX	7	Focal adhe	Integrin-m		3	0.978		0.754352627	22.37	967	1025.2	25.31	3.02	High	High	High	High	High	High	1	
FALSE	High	Master Prc	P08243-1	Asparagine	0	9.657	6	3	3	3	561	64.3	6.86	5.77	3	cell commu	cytosol	catalytic a	Pf00310, P	440	ENSG0000	ASNS	7	Alanine, as	Photodyna	Amino acid	11	0.978		0.754618308	3.09	1425.4	1458	1.92	6.25	High	High	High	High	High	High	1	
FALSE	High	Master Prc	O75874	Isocitrate d	0	65.492	36	14	26	14	414	46.6	7.01	51.49	14	metabolic	cytoplasm;	catalytic a	Pf00180	3417	ENSG0000	IDH1	2	Biosynthes	Glutathion	Neutrophil	21	0.978		0.793879813	22.94	5449.5	5629.4	19.43	7.2	High	High	High	High	High	High	1	
FALSE	High	Master Prc	Q14181	DNA polym	0	18.328	13	4	5	4	598	65.9	5.24	13.12	4	cell organi	cytosol;nu	catalytic a	Pf04042, P	23649	ENSG0000	POLA2	11	Purine met	G1 to S cel	Activation	31	0.977		0.3942968	5.41	976.7	1114.9	9.42	8.49	High	High	High	High	High	High	1	
FALSE	High	Master Prc	Q9NPQ8	Synembryn	0	20.496	11	3	3	3	531	59.7	5.33	11.93	3	cell organi	cytoplasm;	enzyme re	Pf10165	60626	ENSG0000	RIC8A	11				0	0.977		0.394430941	4.38	403.3	423.4	6.15	11.14	High	High	High	High	High	High	1	
FALSE	Medium	Master Prc	Q9BVA0	Katanin p8	0.028	1.462	1	1	1	1	655	72.3	7.56	1.85	1	cell divisio	cytoplasm;	catalytic a	Pf00400, P	10300	ENSG0000	KATNB1	16				0	0.977		0.481823174	7.46	1557	1807.7	13.37	8.92	High	High	High	High	High	High	1	
FALSE	High	Master Prc	Q9Y312	Protein AA	0	8.939	9	2	2	2	384	43.4	5.96	8.35	2	cell organi	spliceosom		Pf05282	25980	ENSG0000	AAR2	20				0	0.977		0.551420013	4.33	153.5	161.8	4.42	7.02	High	High	High	High	High	High	1	
FALSE	High	Master Prc	Q8WZ82	Esterase O	0	16.464	22	2	3	2	227	24.4	6.89	15.42	2	response t	cytoplasm;	catalytic a	Pf03959	124641	ENSG0000	OVCA2	17				0	0.977		0.578989409	19.4	412	427.6	12.7	13.45	High	High	High	High	High	High	1	
FALSE	High	Master Prc	Q13395	Probable n	0.003	2.886	1	1	1	1	1621	181.6	7.05	1.69	1	metabolic	nucleus	catalytic a	Pf00588	6894	ENSG0000	TARBP1	1				0	0.977		0.655287249	45.91	317.9	350.5	46.7	7.79	High	High	High	High	High	High	1	
FALSE	High	Master Prc	Q14141-1	Septin-6 [C	0	14.678	7	3	3	2	434	49.7	6.67	7.32	3	cell differe	chromosor	nucleotide	Pf00735	23157	ENSG0000	SEPT6	X	Bacterial ir			1	0.977		0.700047861	11.53	1016.4	1014.4	9.11	11.78	High	High	High	High	High	High	1	
FALSE	High	Master Prc	Q8N5M9	Protein jag	0	9.09	7	1	3	1	183	21.1	9.73	12.35	1	cell differe	endoplasm	protein bir	Pf07086	84522	ENSG0000	JAGN1	3				0	0.977		0.781054383	1.67	1655.2	1580.1	4.1	6.8	High	High	High	High	High	High	1	
FALSE	High	Master Prc	P33240	cleavage st	0	28.768	24	8	10	4	577	60.9	6.83	18.29	8	metabolic	nucleus	protein bir	Pf00076, P	1478	ENSG0000	CSTF2	X	mRNA surv	mRNA Pro	Cleavage o	15	0.977		0.888686705	10.43	177.8	181.9	7.54	1.37	High	High	High	High	High	High	1	
FALSE	High	Master Prc	Q8IVM0	Coiled-coil	0.001	4.207	4	1	1	1	306	35.8	6.65	3.08	1		cytoplasm;	protein bir	Pf15295	152137	ENSG0000	CCDC50	3				0	0.977		0.921662134	3.39	320.7	316.3	6.59	5.53	High	High	High	High	High	High	1	
FALSE	High	Master Prc	Q969S3	Zinc finger	0	23.911	12	5	7	5	477	54.2	6.15	15.57	5	regulation	cytoplasm;	metal ion l	Pf12171, P	90441	ENSG0000	ZNF622	5				0	0.976		0.391952319	5.03	799.5	921.4	10.39	9.85	High	High	High	High	High	High	1	
FALSE	High	Master Prc	Q15785	Mitochond	0	5.032	5	2	3	1	309	34.5	8.98	7.1	2	transport	cytoplasm;	protein bir	Pf00515, P	10953	ENSG0000	TOMM34	20				0	0.976		0.391991859	4.41	180.6	190.1	2.05	15.23	High	High	High	High	High	High	1	
FALSE	High	Master Prc	Q15021	condensin	0	17.352	5	5	6	5	1401	157.1	6.61	17.92	5	cell divisio	chromosor	protein bir	Pf12717, P	9918	ENSG0000	NCAPD2	12			Condensat	5	0.976		0.395468814	5.1	920.7	968.5	9.69	5.34	High	High	High	High	High	High	1	
FALSE	High	Master Prc	Q53LP3	ankyrin rep	0	15.247	10	4	5	4	525	55.6	7.03	12.36	4			protein bir	Pf00023, P	65124	ENSG0000	SOWAHC	2				0	0.976		0.463212514	7.85	1193	1270.1	20.41	9.39	High	High	High	High	High	High	1	
FALSE	High	Master Prc	Q8NE86	Calcium ur	0	22.598	19	5	6	5	351	39.8	8.65	17.19	5	cell organi	membrane	catalytic a	Pf04678	90550	ENSG0000	MCU	10	NOD-like r		Processing	5	0.976		0.695530846	2.08	1209	1231	6.1	3.17	High	High	High	High	High	High	1	
FALSE	High	Master Prc	P61224-1	Ras-related	0	71.43	61	10	26	3	184	20.8	5.78	67.84	10	cell organi	cytoplasm;	catalytic a	Pf00009, P	5908	ENSG0000	RAP1B	12	Pancreatic	Integrin-m	Gene and p	55	0.976		0.721079045	0.26	1007.4	1023.2	3.74	3.62	High	High	High	High	High	High	1	
FALSE	High	Master Prc	P13693	Translatior	0	64.125	38	8	24	8	172	19.6	4.93	60.75	8	cell differe	cytoplasm;	metal ion l	Pf00838	7178	ENSG0000	TPT1	13				0	0.976		0.740458653	25.42	7951.8	8146.3	15.26	9.03	High	High	High	High	High	High	1	
FALSE	High	Master Prc	P0DMM9	Sulfotransf	0	24.346	27	5	7	5	295	34.2	6.01	24.13	5	metabolic	cytoplasm;	catalytic a	Pf00685, P	445329; 6	ENSG0000	SULT1A4; l	16	Chemical c	Sulfation B	Cytosolic s	11	0.975		0.383549463	4.94	1685.3	1813.8	9.13	6.41	High	High	High	High	High	High	1	
FALSE	High	Master Prc	Q9H2U1	ATP-depen	0	23.879	7	6	6	6	1008	114.7	7.68	11.75	6	metabolic	chromosor	catalytic a	Pf00270, P	170506	ENSG0000	DHX36	3	RNA degra		TRAF6 med	12	0.975		0.419326042	6.6	699.7	685.7	10.09	4.85	High	High	High	High	High	High	1	
FALSE	High	Master Prc	P09104	Gamma-er	0	93.432	31	9	71	5	434	47.2	5.03	176.98	9	metabolic	cytoplasm;	catalytic a	Pf00113, P	2026	ENSG0000	ENO2	12	Biosynthes	Glycolysis	Gluconeog	12	0.975		0.552842748	13.9	2725.3	2698.9	16.88	3.46	High	High	High	High	High	High	1	[Y44(100)
FALSE	High	Master Prc	Q9BSJ8	Extended s	0	112.609	24	18	32	18	1104	122.8	5.83	109.17	18	metabolic	endoplasm	metal ion l	Pf00168	23344	ENSG0000	ESYT1	12			Glycosphin	4	0.975		0.718622733	5.08	6704.4	6956.4	6.51	1.57	High	High	High	High	High	High	1	
FALSE	High	Master Prc	Q9UGP8	Translocati	0	30.728	9	6	11	6	760	87.9	5.31	24.8	6	cell organi	cytosol;en	nucleotide	Pf00183, P	11231	ENSG0000	SEC63	6	Protein exp		XBP1(S) ac	6	0.975		0.76629748	5.38	3269.8	3145.3	14.65	8.16	High	High	High	High	High	High	1	
FALSE	High	Master Prc	P55795	heterogen	0	103.995	34	11	41	4	449	49.2	6.3	114.68	11	metabolic	cytosol;me	protein bir	Pf00076, P	3188	ENSG0000	HNRNPH2	X		mRNA Pro	mRNA Spli	5	0.975		0.788878681	9.64	2857	3121.7	7.12	13.89	High	High	High	High	High	High	1	[R316]
FALSE	High	Master Prc	Q96QD8	sodium-co	0	15.696	10	3	3	3	506	56	8	9.95	3	cell commu	membrane	protein bir	Pf01490	54407	ENSG0000	SLC38A2	12	Glutamate	Endochond	Amino acid	12	0.975		0.845550676	35.1	128.1	130.7	18.28	16.19	High	High	High	High	High	High	1	
FALSE	High	Master Prc	Q17RY6-1	Lymphocyt	0	8.574	19	3	5	3	165	18.7	7.43	7.65	3	cellular co	cell surface			54742	ENSG0000	LY6K	8			Post-transl	3	0.975		0.856399762	15.14	1258.1	1265.1	14.24	8.48	High	High	High	High	High	High	1	
FALSE	High	Master Prc	Q9H8H0	nucleolar p	0	6.298	3	2	2	2	719	81.1	6.07	5.91	2	metabolic	nucleus	protein bir	Pf08168	25926	ENSG0000	NOL11	17			Major path	5	0.975		0.863327018	9.1	436.6	421.1	7.85	3.97	High	High	High	High	High	High	1	
FALSE	High	Master Prc	Q8IY17-1	Neuropath	0	34.235	10	9	9	9	1366	149.9	7.81	28.43	9	metabolic	endoplasm	catalytic a	Pf00027, P	10908		PNPLA6	19	Glycerophc		Glycerophc	6	0.975		0.878703994	1.83	2189.4	2123.3	4.2	4.19	High	High	High	High	High	High	1	
FALSE	High	Master Prc	Q13823	Nucleolar (	0	7.801	3	2	3	2	731	83.6	9.25	9.77	2		membrane	catalytic a	Pf01926, P	29889	ENSG0000	GNL2	1	Ribosome			1	0.975		0.95577005	6.35	953.6	964.1	5.35	3.01	High	High	High	High	High	High	1	
FALSE	High	Master Prc	O00425	Insulin-like	0	51.164	21	11	18	8	579	63.7	8.87	42.45	11	metabolic	cytoplasm;	protein bir	Pf00013, P	10643	ENSG0000	IGF2BP3	7			Insulin-like	2	0.974		0.105697065	1.74	2424	2474.2	1.02	0.93	High	High	High	High	High	High	1	
FALSE	High	Master Prc	P19838	Nuclear fa	0	15.544	4	2	2	2	968	105.3	5.4	7.38	2	cell death;	cytoplasm;	DNA bindi	Pf00023, P	4790	ENSG0000	NFKB1	4	TNF signali	Osteopont	CLEC7A/in	178	0.974		0.37090461	1.31	273.7	327.6	18.24	13.49	High	High	High	High	High	High	1	
FALSE	High	Master Prc	P23381	Tryptophar	0	15.464	9	3	4	3	471	53.1	6.23	11.69	3	metabolic	cytoplasm;	catalytic a	Pf00458, P	7453	ENSG0000	WARS	14	Aminoacyl	Photodyna	Cytosolic t	8	0.974		0.41563813	6.93	1117.7	1178.6	14.38	5.41	High	High	High	High	High	High	1	
FALSE	High	Master Prc	Q16763	ubiquitin-c	0	13.879	19	2	2	2	222	23.8	8.38	4.97	2	cell divisio	cytoplasm;	catalytic a	Pf00179	27338	ENSG0000	UBE2S	19	Ubiquitin r		Synthesis c	9	0.974		0.568203464	1.05	375.4	348	9.1	10	High	High	High	High	High	High	1	
FALSE	High	Master Prc	Q9HCL2	Glycerol-3-	0.005	2.596	2	1	1	1	828	93.7	7.74	3.29	1	defense re	membrane	catalytic a	Pf01553	57678	ENSG0000	GPAM	10	Glycerophc	Sterol Regi	Synthesis c	21	0.974		0.596455436	10.6	900.1	923.9	7.55	0.43	High	High	High	High	High	High	1	
FALSE	High	Master Prc	Q9HCG8	Pre-mRNA	0.003	3.157	1	1	1	1	908	105.4	7.03	3.19	1	metabolic	cytosol;nu	protein bir	Pf02847, P	57703	ENSG0000	CWC22	2			mRNA Spli	4	0.974		0.604176442	14	359.4	366.7	23.26	8.4	High	High	High	High	High	High	1	
FALSE	High	Master Prc	P29966	Myristoyla	0	62.269	33	7	16	7	332	31.5	4.45	35.7	7	cell organi	cytoplasm;	protein bir		4082	ENSG0000	MARCKS	6	MicroRNA	Brain-Deri	Acetylchol	7	0.974		0.707569438	7.14	3289.3	3378.4	20.28	6.83	High	High	High	High	High	High	1	
FALSE	High	Master Prc	O00151	PDZ and LI	0	47.528	28	7	11	7	329	36	7.02	34.9	7	regulation	cytoplasm;	metal ion l	Pf00412, P	9124	ENSG0000	PDLIM1	10				0	0.974		0.729499741	12.98	3350.2	3635.5	13.45	5.76	High	High	High	High	High	High	1	
FALSE	High	Master Prc	Q9UJZ1	Stomatin-l	0	68.936	53	11	19	11	356	38.5	7.39	67.45	11	cell organi	cytoplasm;	protein bir	Pf01145	30968	ENSG0000	STOML2	9			Processing	3	0.974		0.742627958	26.83	3016.4	3095.9	5.99	16.3	High	High	High	High	High	High	1	
FALSE	High	Master Prc	P63208	s-phase kir	0	31.266	34	7	14	7	163	18.6	4.54	31.57	7	cell organi	cytoplasm;	catalytic a	Pf01466, P	6500	ENSG0000	SKP1	5	Oocyte me	TNF alpha	FBXL7 dow	105	0.974		0.779560338	22.27	2918.8	3233	18.5	5.23	High	High	High	High	High	High	1	
FALSE	High	Master Prc	Q12905	Interleukin	0	191.298	73	20	59	20	390	43	5.26	183.77	20	metabolic	cytoplasm;	catalytic a	Pf07528	3608	ENSG0000	ILF2	1		B Cell Rece	Neutrophil	4	0.974		0.816040263	1.29	19813.2	19640.4	2.06	3.86	High	High	High	High	High	High	1	
FALSE	High	Master Prc	Q15004-1	PCNA-asso	0	8.088	16	1	2	1	111	12	9.83	6.72	1	cell organi	cytoplasm;	protein bir		9768	ENSG0000	KIAA0101;	15			Terminatic	4	0.974		0.823287027	0.19	1676.3	1571.6	12.56	10.99	High	High	High	High	High	High	1	
FALSE	High	Master Prc	P05413	Fatty acid-	0	46.399	58	8	20	8	133	14.8	6.8	49.88	8	metabolic	cytoplasm;	protein bir	Pf00061, P	2170	ENSG0000	FABP3	1	PPAR signa	PPAR signa	Triglycerid	6	0.974		0.960873169	20.03	9073.2	9316.3	8.81	7.34	High	High	High	High	High	High	1	
FALSE	High	Master Prc	Q9H0A0	RNA cytidi	0	123.213	33	22	30	22	1025	115.7	8.27	93.26	22	metabolic	membrane	catalytic a	Pf00583, P	55226	ENSG0000	NAT10	11	Ribosome	Metapathw	rRNA mod	6	0.973		0.140022248	2.46	6631.7	7007.9	2.74	3.98	High	High	High	High	High	High	1	
FALSE	High	Master Prc	Q96C23	aldose 1-ep	0	30.483	23	5	9	5	342	37.7	6.65	26.12	5	metabolic	cytoplasm	catalytic a	Pf01263	130589	ENSG0000	GALM	2	Metabolic			3	0.973		0.17790264	3.02	943.2	1000.9	7.84	6.56	High	High	High	High	High	High	1	
FALSE	High	Master Prc	P26639-1	Threonine-	0	155.815	42	30	64	30	723	83.4	6.67	126.05	30	metabolic	cytoplasm;	catalytic a	Pf00587, P	6897	ENSG0000	TARS	5	Aminoacyl		Cytosolic t	5	0.973		0.320838484	5.24	21136.3	21942.4	2.32	2.76	High	High	High	High	High	High	1	
FALSE	High	Master Prc	P18669	Phosphogl	0	126.545	69	14	49	14	254	28.8	7.18	147.04	14	metabolic	cytoplasm;	catalytic a	Pf00300	5223; 643	ENSG0000	PGAM1; L	10; 12	Biosynthes	Cori Cycle;	Gluconeog	17	0.973		0.322163325	0.93	16620.2	17651.3	13.85	2.38	High	High	High	High	High	High	1	
FALSE	High	Master Prc	Q14192	Four and a	0	13.736	21	4	4	4	279	32.2	7.55	5.5	4	cell differe	cytoplasm;	metal ion l	Pf00412	2274	ENSG0000	FHL2	2	Osteoclast	Androgen	PPARA act	9	0.973		0.462200469	9.18	539.2	592	10.91	6.77	High	High	High	High	High	High	1	
FALSE	High	Master Prc	P15311	Ezrin [OS=l	0	192.997	58	39	100	27	586	69.4	6.27	203.03	39	cell differe	cytoplasm;	protein bir	Pf00373, P	7430	ENSG0000	EZR	6	MicroRNA	Pathogeni	Netrin-1 si	15	0.973		0.532208426	9.41	14392.8	14843.3	5.97	1.73	High	High	High	High	High	High	1	
FALSE	High	Master Prc	P15559-1	NAD(P)H d	0	43.595	31	9	17	9	274	30.8	8.88	44.94	9	cell commu	cytoplasm;	antioxidan	Pf02525, P	1728	ENSG0000	NQO1	16	Ubiquinon	Dopamine	Regulation	16	0.973		0.561268998	11.05	5280.4	5428	7.34	2.52	High	High	High	High	High	High	1	
FALSE	High	Master Prc	P42704	Leucine-ric	0	414.549	56	66	149	66	1394	157.8	6.13	363.9	66	cellular co	chromosor	catalytic a	Pf12854, P	10128	ENSG0000	LRPPRC	2			TP53 Regu	9	0.973		0.56938416	10.42	36613.6	37889.4	6.56	2.22	High	High	High	High	High	High	1	
FALSE	High	Master Prc	P06396	Gelsolin [C	0	18.929	8	5	5	5	782	85.6	6.28	8.05	5	cell death;	cytoplasm;	metal ion l	Pf00626	2934	ENSG0000	GSN	9	Viral carcir	Regulation	Neutrophil	15	0.973		0.602433907	20.38	840	863.4	32.27	5.75	High	High	High	High	High	High	1	
FALSE	High	Master Prc	Q96LD4	Tripartite r	0	28.85	13	7	9	7	638	69.5	6.44	24.25	7		cytoplasm;	metal ion l	Pf00097, P	91107	ENSG0000	TRIM47	17				0	0.973		0.62397751	9.16	2725.2	2743.4	15.09	9.01	High	High	High	High	High	High	1	
FALSE	High	Master Prc	O95478	Ribosome	0	12.518	21	4	4	4	260	30	10.27	10.85	4	metabolic	nucleus	RNA bindir	Pf01201	10412	ENSG0000	NSA2	5				0	0.973		0.661699447	16.16	1003.5	1031.5	21.02	5.72	High	High	High	High	High	High	1	
FALSE	High	Master Prc	Q8WWY3	U4/U6 sma	0	36.245	20	8	14	8	499	55.4	5.78	33.81	8	cell organi	nucleus;sp	protein bir	Pf01798, P	26121	ENSG0000	PRPF31	19; CHR_H	Spliceosom		mRNA Spli	5	0.973		0.701623693	17.96	2667.4	2741.2	20.86	6.81	High	High	High	High	High	High	1	
FALSE	High	Master Prc	P31689-1	DnaJ homo	0	64.661	24	8	19	8	397	44.8	7.08	68.71	8	cellular co	cytoplasm;	enzyme re	Pf00226, P	3301	ENSG0000	DNAJA1	9	Protein pro	Exercise-in	HSP90 cha	5	0.973		0.81851178	1.97	11416.6	11613.5	1.63	8.85	High	High	High	High	High	High	1	
FALSE	High	Master Prc	Q9NYF8-3	Isoform 3 o	0	162.609	33	30	73	1	869	100.2	9.95	157.66	30	cell death;	cytoplasm;	DNA bindi	Pf15440	9774	ENSG0000	BCLAF1	6		Interactom		1	0.973		0.855447638	13.09	695.5	725.4	11.86	14.7	High	High	High	High	High	High	1	
FALSE	High	Master Prc	O00231	26S protea	0	60.306	29	11	22	11	422	47.4	6.48	53.17	11	cell differe	cytoplasm;	catalytic a	Pf01399	5717	ENSG0000	PSMD11	17	Epstein-Ba	Proteasom	Regulation	153	0.972		0.47330372	8.77	8807.5	8891.7	6.87	3.67	High	High	High	High	High	High	1	
FALSE	High	Master Prc	Q8TDD1	ATP-depen	0	20.912	8	5	5	5	881	98.5	10.02	8.98	5	metabolic	cytoplasm;	catalytic a	Pf00270, P	79039	ENSG0000	DDX54	12				0	0.972		0.546675781	37.96	903.2	929.6	58.44	10.88	High	High	High	High	High	High	1	
FALSE	High	Master Prc	Q96Q11-1	CCA tRNA	0	13.379	6	2	2	2	434	50.1	8.1	8.27	2	cell organi	mitochond	catalytic a	Pf01743, P	51095	ENSG0000	TRNT1	3	RNA transp		tRNA proc	5	0.972		0.560296488	23.15	289.2	297.6	29.69	9.36	High	High	High	High	High	High	1	
FALSE	High	Master Prc	Q9H9Y6-1	DNA-direct	0.003	3.104	1	1	2	1	1135	128.1	7.83	5.35	1	cell organi	cytosol;nu	catalytic a	Pf00562, P	84172	ENSG0000	POLR1B	2	Metabolic	Eukaryotic	RNA Polym	17	0.972		0.579727175	40.95	1382	1427	54.66	9.05	High	High	High	High	High	High	1	
FALSE	High	Master Prc	P00441	Superoxide	0	84.595	68	10	36	10	154	15.9	6.13	91.11	10	cell organi	cytoplasm;	antioxidan	Pf00080	6647	ENSG0000	SOD1	21	Amyotroph	Selenium M	Platelet de	27	0.972		0.583115437	7.45	10879.7	11111	1.91	4.58	High	High	High	High	High	High	1	
FALSE	High	Master Prc	Q9UNX4	WD repeat	0	11.842	5	3	3	3	943	106	6.64	7.94	3	metabolic	membrane	protein bir	Pf00400, P	10885	ENSG0000	WDR3	1	Ribosome		Major path	6	0.972		0.6594501	13.19	574.2	599.7	9.35	1.23	High	High	High	High	High	High	1	
FALSE	High	Master Prc	Q6WCQ1	Myosin ph	0	7.805	2	1	2	1	1025	116.5	6.21	4.13	1		cytoplasm;	protein bir	Pf00169	23164	ENSG0000	MPRIP	17			Signaling b	4	0.972		0.778688494	37.53	352.8	347	29.28	20.66	High	High	High	High	High	High	1	
FALSE	High	Master Prc	Q9UBB4-1	Ataxin-10	0	43.371	17	6	12	6	475	53.5	5.25	25.39	6	cell organi	cytoplasm;	protein bir	Pf09759	25814	ENSG0000	ATXN10	22				0	0.972		0.782598135	8.35	1679.8	1728.7	10.4	3.74	High	High	High	High	High	High	1	
FALSE	High	Master Prc	P28065	Proteasom	0	6.218	10	1	1	1	219	23.3	5.03	5.77	1	metabolic	cytoplasm;	catalytic a	Pf00227	5698	ENSG0000	PSMB9	6; CHR_HS	Proteasom	Proteasom	Regulation	151	0.972		0.79985418	21.16	433.4	385.1	17.78	11.94	High	High	High	High	High	High	1	

FALSE	High	Master Prc	Q9GZP8-1	Immortaliz	0	23.381	43	4	10	4	106	10.9	9.73	22.28	4		cytosol;me	DNA bindi		64073	ENSG0000	C19orf33	19				0	0.972		0.800350625	28.94	5737.5	5893.4	5.07	17.77	High	High	High	High	High	High	1	
FALSE	High	Master Prc	Q01780	exosome c	0	20.434	7	6	7	6	885	100.8	8.46	11.55	6	metabolic	cytoplasm;	catalytic ac	Pf00570, F	5394	ENSG0000	EXOSC10	1	RNA degra		Major path	5	0.972		0.862317712	13.04	869.1	893.9	12.38	4.35	High	High	High	High	High	High	1	
FALSE	High	Master Prc	Q14152	Eukaryotic	0	152.808	30	41	76	41	1382	166.5	6.79	142.81	41	cell organi	cytoplasm;	protein bir	Pf01399	8661	ENSG0000	EIF3A	10	RNA transp	Translation	Formation	13	0.972		0.930969455	2.15	18085.7	18258.6	4.56	2.86	High	High	High	High	High	High	1	
FALSE	High	Master Prc	Q9BUF5	Tubulin be	0	117.222	53	18	67	7	446	49.8	4.88	156.3	18	cell organi	cytoplasm;	catalytic ac	Pf00091, F	84617	ENSG0000	TUBB6	18	Gap junctic	Pathogenic	COPI-indep	65	0.971		0.051512635	1.02	2929.6	3010.8	5.21	3.66	High	High	High	High	High	High	1	
FALSE	High	Master Prc	P49590	probable h	0	35.164	22	7	8	4	506	56.9	8.24	19.23	7	cell organi	cytoplasm;	catalytic ac	Pf00587, F	23438	ENSG0000	HARS2	5	Aminoacyl		Mitochonc	5	0.971		0.266619819	2.73	1310.4	1324.8	3.21	8.77	High	High	High	High	High	High	1	
FALSE	High	Master Prc	P11413-1	Glucose-6-	0	154.021	58	25	70	25	515	59.2	6.84	177.87	25	metabolic	cytoplasm;	catalytic ac	Pf00479, F	2539	ENSG0000	G6PD	X	Carbon me	Glutathion	TP53 Regu	19	0.971		0.284257726	3.23	24502.4	26517.4	7.06	4.88	High	High	High	High	High	High	1	
FALSE	High	Master Prc	O43765	Small gluta	0	23.637	21	6	8	6	313	34	4.87	14.75	6	metabolic	cytoplasm;	protein bir	Pf00515, F	6449	ENSG0000	SGTA	19				0	0.971		0.388728043	6.18	359.2	369.9	11.54	2.24	High	High	High	High	High	High	1	
FALSE	High	Master Prc	Q969E8	pre-rRNA-p	0	10.209	20	2	4	2	191	20.9	4.39	3.15	2	metabolic	nucleus	protein bir	Pf10273	90121	ENSG0000	TSR2	X				0	0.971		0.470635019	12.46	809.9	833.9	29.34	0.81	High	High	High	High	High	High	1	
FALSE	High	Master Prc	Q9NX58	Cell growtl	0	9.914	7	3	5	3	379	43.6	9.54	10.67	3		nucleus	metal ion l	Pf08790	55646		LYAR	4				0	0.971		0.504406457	4.14	2248.9	2268.5	26.27	8.35	High	High	High	High	High	High	1	
FALSE	High	Master Prc	Q7L2E3-1	Putative A'	0	40.864	9	9	12	9	1194	133.9	8.78	26.19	9	cell organi	cytoplasm;	catalytic ac	Pf00270, F	22907	ENSG0000	DHX30	3				0	0.971		0.516390089	9.2	1693	1742.8	2.11	4.11	High	High	High	High	High	High	1	
FALSE	High	Master Prc	Q9Y314	nitric oxide	0	16.075	13	3	4	3	301	33.2	8.82	13.07	3	developme	cytoplasm;	catalytic ac	Pf04564, F	51070	ENSG0000	NOSIP	19			NOSIP mec	4	0.971		0.522910593	10.4	1267.6	1317.8	9.18	0.52	High	High	High	High	High	High	1	
FALSE	High	Master Prc	Q9Y6G9	Cytoplasm	0	11.597	10	3	6	3	523	56.5	6.42	10.69	3	cell divisio	chromosor	catalytic ac	Pf00005, F	51143	ENSG0000	DYNC1LI1	3	Salmonella		COPI-indep	38	0.971		0.56451306	28.58	490.8	580.1	20.2	24.42	High	High	High	High	High	High	1	
FALSE	High	Master Prc	P54105	Methyloso	0	29.434	27	5	11	5	237	26.2	4.11	29.18	5	cell organi	cytoplasm;	protein bir	Pf03517	1207	ENSG0000	CLNS1A	11	RNA transp		snRNP Ass	4	0.971		0.566359997	5.17	3188.5	3283.1	20.31	2.86	High	High	High	High	High	High	1	
FALSE	High	Master Prc	Q9H0U3	Magnesiur	0	4.556	5	2	2	2	335	38	9.63	2.31	2	metabolic	endoplasm	catalytic ac	Pf04756	84061	ENSG0000	MAGT1	X			Asparagine	8	0.971		0.59729033	11.51	152.9	159.1	3.58	5.44	High	High	High	High	High	High	1	
FALSE	High	Master Prc	Q96D46	60S riboso	0	23.182	14	5	6	5	503	57.6	7.14	16.05	5	regulation	cytoplasm;	protein bir	Pf04981	51068	ENSG0000	NMD3	3	Ribosome			2	0.971		0.607725372	6.48	2295.4	2289.8	13.71	9.41	High	High	High	High	High	High	1	
FALSE	High	Master Prc	P40692-1	DNA mism	0	6.584	3	2	2	2	756	84.5	5.72	3.48	2	cell organi	chromosor	catalytic ac	Pf01119, F	4292	ENSG0000	MLH1	3	Fanconi ar	Ovarian In	Mismatch	25	0.971		0.610417761	28.32	393.6	405.5	20.38	16.33	High	High	High	High	High	High	1	
FALSE	High	Master Prc	P61758	Prefoldin s	0	31.921	42	9	20	9	197	22.6	7.11	37.38	9	cell organi	cytoplasm;	protein bir	Pf02996	7411		VBP1	X			Prefoldin r	5	0.971		0.611376408	25.78	4152	4277.7	35.3	11.03	High	High	High	High	High	High	1	
FALSE	High	Master Prc	P25205	DNA replic	0	113.502	34	24	45	24	808	90.9	5.77	90.93	24	metabolic	cytoplasm;	catalytic ac	Pf00493, F	4172	ENSG0000	MCM3	6	DNA replic	Cell Cycle;	M/G1 Trar	26	0.971		0.987371894	1.25	8865.5	9047.1	2.36	3.73	High	High	High	High	High	High	1	
FALSE	High	Master Prc	P12081	Histidine--'	0	53.214	25	11	14	8	509	57.4	5.88	32.27	11	cell organi	cytoplasm;	catalytic ac	Pf00458, F	3035	ENSG0000	HARS	5	Aminoacyl	Allograft R	Cytosolic t	6	0.97		0.058834274	1.23	2988.2	3071.1	9.12	10.19	High	High	High	High	High	High	1	
FALSE	High	Master Prc	P33993-1	DNA replic	0	102.009	29	16	34	16	719	81.3	6.46	89.13	16	cell prolife	chromosor	catalytic ac	Pf00493, F	4176	ENSG0000	MCM7	7	Cell cycle;	miRNA Reg	M/G1 Trar	27	0.97		0.158995042	1.62	6695	6870.3	4.45	1.72	High	High	High	High	High	High	1	
FALSE	High	Master Prc	P53992	Protein tra	0	76.348	19	15	28	15	1094	118.2	7.06	78.72	15	cell organi	cytoplasm;	metal ion l	Pf00626, F	9632	ENSG0000	SEC24C	10	Protein prc	Sterol Reg	Regulation	20	0.97		0.231750481	4.04	4287.1	4343.8	4.9	2.56	High	High	High	High	High	High	1	
FALSE	High	Master Prc	Q9UKS6	Protein kin	0	46.928	29	10	18	10	424	48.5	6.18	46.98	10	cell organi	cytoplasm;	enzyme re	Pf00018, F	29763	ENSG0000	PACSIN3	11			Clathrin-m	3	0.97		0.234003574	2.57	4405.8	4587.1	4.1	1.57	High	High	High	High	High	High	1	
FALSE	High	Master Prc	Q9UNF1	Melanoma	0	128.714	44	28	53	28	606	64.9	9.32	120.97	28	transport	cytosol;ext	protein bir	Pf01454	10916	ENSG0000	MAGED2	X			Platelet de	4	0.97		0.342439647	5.13	10205.9	11036.7	7.15	3.48	High	High	High	High	High	High	1	
FALSE	High	Master Prc	O95379	Tumor nec	0	7.484	10	1	1	1	198	23	7.93	4.93	1	cell death;	cytoplasm;	protein bir	Pf05527	25816	ENSG0000	TNFAIP8	5			PI Metabo	4	0.97		0.584544135	46.4	58.8	60.6	27.89	41.62	High	High	High	High	High	High	1	
FALSE	High	Master Prc	O94925-3	Isoform 3 c	0	78.468	34	13	22	13	598	65.4	7.88	64.22	13	cell comm	cytosol;mi	catalytic ac	Pf04960	2744	ENSG0000	GLS	2		Amino Aci		1	0.97		0.608832622	15.05	3447.1	3553.8	8.41	5	High	High	High	High	High	High	1	
FALSE	High	Master Prc	Q96AX1	Vacuolar p	0.003	3.438	3	2	3	2	596	67.6	6.96	4.97	2	cell differe	endosome	protein bir	Pf00995	65082	ENSG0000	VPS33A	12				0	0.97		0.648076833	18.13	534.5	551.1	9.96	6.46	High	High	High	High	High	High	1	
FALSE	High	Master Prc	Q9UIA9	exportin-7	0	18.217	6	5	6	5	1087	123.8	6.32	12.53	5	transport	cytoplasm;	protein bir	Pf03810	23039	ENSG0000	XPO7	8				0	0.97		0.678633817	17.45	827.3	883.8	10.79	6.42	High	High	High	High	High	High	1	
FALSE	High	Master Prc	P78346-1	ribonuclea	0	8.117	15	3	4	3	268	29.3	8.91	4.94	3	metabolic	nucleus	catalytic ac	Pf01876	10556	ENSG0000	RPP30	10	RNA transp		Major path	8	0.97		0.770136287	10.54	998.4	1029.4	13.05	3.71	High	High	High	High	High	High	1	
FALSE	High	Master Prc	Q9P258	Protein RC	0	44.219	26	12	17	12	522	56	8.78	33.38	12	cell organi	chromosor	protein bir	Pf00415, F	55920	ENSG0000	RCC2	1; CHR_HC			Amplificati	16	0.97		0.775862596	6.22	2386.5	2459.6	4.9	1.99	High	High	High	High	High	High	1	
FALSE	High	Master Prc	Q96G03	Phosphogl	0	21.807	14	8	9	8	612	68.2	6.73	15.77	8	metabolic	cytoplasm;	catalytic ac	Pf00408, F	55276	ENSG0000	PGM2	4	Glycolysis,	Glucuronic	Neutrophil	18	0.97		0.939044133	1.63	1125	1107.2	5.11	9.45	High	High	High	High	High	High	1	
FALSE	High	Master Prc	P68371	Tubulin be	0	328.506	72	26	212	2	445	49.8	4.89	556.52	26	cell organi	cytoplasm;	catalytic ac	Pf00091, F	10383	ENSG0000	TUBB4B	9	Phagosom	Pathogenic	COPI-indep	74	0.969		0.181506192	2.61	3563	3613.7	5.11	7.71	High	High	High	High	High	High	1	[R241]
FALSE	High	Master Prc	P07741-1	Adenine pl	0	58.039	79	9	17	9	180	19.6	6.02	61.17	9	metabolic	cytoplasm;	catalytic ac	Pf00156	353	ENSG0000	APRT	16	Metabolic		Neutrophil	9	0.969		0.313095961	2.38	3691.3	3809.2	5.37	6.47	High	High	High	High	High	High	1	
FALSE	High	Master Prc	Q13263	Transcripti	0	137.384	42	23	50	23	835	88.5	5.77	91.04	23	cell organi	chromosor	catalytic ac	Pf00628, F	10155	ENSG0000	TRIM28	19		Corticotroj	Generic Tr	6	0.969		0.360309381	6.82	14567.8	15348.3	2.55	7.47	High	High	High	High	High	High	1	
FALSE	High	Master Prc	Q86V81	THO comp	0	54.608	41	8	15	8	257	26.9	11.15	32.67	8	cell organi	cytoplasm;	protein bir	Pf00076, F	10189		ALYREF	17	Herpes sim		mRNA 3'-e	19	0.969		0.403512614	7.46	5227.2	5519	10.75	8.12	High	High	High	High	High	High	1	[R38;
FALSE	High	Master Prc	Q14554	Protein dis	0	7.28	9	3	3	3	519	59.6	7.91	7.07	3	cellular ho	endoplasm	catalytic ac	Pf00085, F	10954	ENSG0000	PDIA5	3			XBP1(S) ac	4	0.969		0.498193556	12.6	719	742	7.7	8.77	High	High	High	High	High	High	1	
FALSE	High	Master Prc	P20073-1	Annexin A'	0	63.481	29	13	32	13	488	52.7	5.68	63.67	13	cell organi	cytosol;me	metal ion l	Pf00191	310	ENSG0000	ANXA7	10				0	0.969		0.585106022	2.78	10753.6	11094.8	1.15	5.5	High	High	High	High	High	High	1	
FALSE	High	Master Prc	Q9NZI8	Insulin-like	0	52.715	25	13	18	10	577	63.4	9.2	27.37	13	cell prolife	cytoplasm;	protein bir	Pf00013, F	10642	ENSG0000	IGF2BP1	17	MicroRNA:	Brain-Deri	MAPK6/M	8	0.969		0.588878454	21.42	3150.6	3202.1	24.46	5.43	High	High	High	High	High	High	1	
FALSE	High	Master Prc	Q86U38	Nucleolar p	0	9.663	5	2	2	2	636	69.4	7.28	4.88	2			RNA bindi		161424	ENSG0000	NOP9	14				0	0.969		0.615221806	23.55	106.6	109.9	20.06	9.67	High	High	High	High	High	High	1	
FALSE	High	Master Prc	Q96CT7	Coiled-coil	0	13.423	17	4	4	4	223	25.8	9.54	6.42	4	cell divisio	cytoplasm;	RNA bindi	Pf06244	115098	ENSG0000	CCDC124	19				0	0.969		0.676727212	4.98	2663.8	2749.8	10.73	5.19	High	High	High	High	High	High	1	
FALSE	High	Master Prc	Q8N6T3-1	ADP-ribosy	0	10.95	10	3	3	3	406	44.6	5.66	11.12	3	cell organi	cytoplasm;	catalytic ac	Pf01412	55738	ENSG0000	ARFGAP1	20	Endocytosi		COPI-depe	16	0.969		0.814493813	16.46	1998.3	2061.4	5.69	7.43	High	High	High	High	High	High	1	
FALSE	High	Master Prc	P08758	annexin A!	0	132.473	80	28	83	28	320	35.9	5.05	146.43	28	coagulatio	cytoplasm;	enzyme re	Pf00191	308	ENSG0000	ANXA5	4		Apoptosis-	Platelet de	7	0.969		0.840836401	3.26	14317.6	14212.6	5.17	5.68	High	High	High	High	High	High	1	
FALSE	High	Master Prc	Q7L014	probable A	0	95.214	29	25	35	25	1031	117.3	9.29	73.05	25	metabolic	cytoplasm;	catalytic ac	Pf00270, F	9879	ENSG0000	DDX46	5	Spliceosom		mRNA Spli	5	0.969		0.8915649	4.65	9264.1	9308.1	6.43	4.43	High	High	High	High	High	High	1	
FALSE	High	Master Prc	Q15436	protein tra	0	78.14	25	13	23	11	765	86.1	7.08	67.66	13	cell organi	cytoplasm;	metal ion l	Pf00626, F	10484	ENSG0000	SEC23A	14	Protein prc	Sterol Reg	Regulation	20	0.968		0.013919736	0.3	2997	3098	0.83	0.16	High	High	High	High	High	High	1	
FALSE	High	Master Prc	Q96C36	Pyrroline-!	0	33.421	19	5	15	4	320	33.6	7.77	41.65	5	cell organi	cytoplasm;	catalytic ac	Pf01210, F	29920	ENSG0000	PYCR2	1	Arginine ar		Amino acid	6	0.968		0.358831427	4.14	2830.6	3006	6.59	3.62	High	High	High	High	High	High	1	
FALSE	High	Master Prc	Q96A33-1	Coiled-coil	0	25.282	12	4	4	4	483	55.8	4.87	12.11	4	cell differe	endoplasm	metal ion l	Pf07946	57003	ENSG0000	CCDC47	17				0	0.968		0.387936356	7.03	1024.2	1047.9	12.95	0.8	High	High	High	High	High	High	1	
FALSE	High	Master Prc	Q53GS9	U4/U6.U5	0	28.113	11	4	8	4	565	65.3	8.91	28.99	4	cell divisio	nucleus;sp	catalytic ac	Pf00443, F	10713	ENSG0000	USP39	2	Spliceosom		mRNA Spli	5	0.968		0.488769601	12.98	1558.5	1609.9	15.46	4.2	High	High	High	High	High	High	1	
FALSE	Medium	Master Prc	Q15560	transcripti	0.024	1.52	8	1	2	1	299	33.6	9.13	2.03	1	metabolic	nucleus	DNA bindi	Pf01096, F	6919	ENSG0000	TCEA2	20				0	0.968		0.722319221	51.26	298.4	308.4	24.75	29.72	High	High	High	High	High	High	1	[Q255]
FALSE	High	Master Prc	P22087	rRNA 2'-O-	0	67.154	51	12	30	12	321	33.8	10.18	75.64	12	cell differe	chromosor	catalytic ac	Pf01135, F	2091	ENSG0000	FBL	19; CHR_H	Ribosome		Major path	6	0.968		0.836633172	8.73	8989.6	9288.8	12.6	1.38	High	High	High	High	High	High	1	
FALSE	High	Master Prc	O43172-1	U4/U6 sma	0	34.264	20	7	10	7	522	58.4	7.42	36.42	7	metabolic	nucleus;sp	protein bir	Pf00400, F	9128	ENSG0000	PRPF4	9	Spliceosom	mRNA Pro	mRNA Spli	6	0.968		0.873295326	11.64	3289.9	3168.4	9.86	4.66	High	High	High	High	High	High	1	
FALSE	High	Master Prc	P51692	Signal tran	0	43.741	9	6	12	2	787	89.8	6.05	32.37	6	cell differe	cytoplasm;	protein bir	Pf00017, F	6777	ENSG0000	STAT5B	17	Th1 and Th	IL-5 Signali	Interleukin	53	0.968		0.930762727	7.51	2110.3	1849.5	25.87	35.45	High	High	High	High	High	High	1	
FALSE	High	Master Prc	O75146	Huntingtin	0	14.24	6	4	4	4	1068	119.3	6.67	8.65	4	cell death;	cytoplasm;	protein bir	Pf01417, F	9026	ENSG0000	HIP1R	12			Golgi Asso	6	0.967		0.289245037	3.4	179.2	196.8	6.65	4.65	High	High	High	High	High	High	1	
FALSE	High	Master Prc	P40925	Malate del	0	62.204	49	15	29	15	334	36.4	7.36	45.35	15	metabolic	cytoplasm;	catalytic ac	Pf00056, F	4190	ENSG0000	MDH1	2	Metabolic	Sterol Reg	Gluconeog	15	0.967		0.304496744	4.14	9000.9	9436	11.61	3.55	High	High	High	High	High	High	1	
FALSE	High	Master Prc	Q15645	Pachytene	0	25.683	14	6	10	6	432	48.5	6.09	17.9	6	cell differe	nucleus	catalytic ac	Pf00004, F	9319	ENSG0000	TRIP13	5				0	0.967		0.313281527	4.78	904.3	928.4	14.41	20.72	High	High	High	High	High	High	1	
FALSE	High	Master Prc	Q9BYD1	39S riboso	0	18.625	27	4	7	4	178	20.7	9.16	22.62	4	cell organi	membrane	protein bir	Pf00572	28998	ENSG0000	MRPL13	8	Ribosome		Mitochonc	7	0.967		0.313563787	1.66	2026.9	2218.6	8.18	7.51	High	High	High	High	High	High	1	
FALSE	High	Master Prc	Q9BQ95	Evolutiona	0	15.587	8	3	3	3	431	49.1	6.29	8.89	3	cell organi	cytoplasm;	catalytic ac	Pf06239, F	51295	ENSG0000	ECSIT	19	MAPK sign	IL-1 signali	MyD88:Ma	25	0.967		0.324725475	3.11	606.9	650.3	3.22	10.58	High	High	High	High	High	High	1	
FALSE	High	Master Prc	Q9Y3F4	Serine-thre	0	81.544	52	14	22	14	350	38.4	5.12	62.32	14	cell organi	cytoplasm;	protein bir	Pf00400	11171	ENSG0000	STRAP	12	RNA transp	TGF-beta S	Downregu	7	0.967		0.514462385	0.05	10307.9	10495.9	4.77	2.84	High	High	High	High	High	High	1	
FALSE	High	Master Prc	O60936-3	Isoform 3 c	0	6.121	10	1	1	1	270	29.3	4.3	2.86	1	cell death;	cytoplasm;en	enzyme re	Pf00619	8996	ENSG0000	NOL3	16				0	0.967		0.548708859	12.37	141.6	141.2	30.63	9.5	High	High	High	High	High	High	1	
FALSE	High	Master Prc	P46019	Phosphory	0.006	2.38	1	1	1	1	1235	138.3	6.44	0	1	metabolic	cytosol;me	catalytic ac	Pf00723	5256	ENSG0000	PHKA2	X	Calcium sig	Type II diat	Glycogen b	9	0.967		0.581062789	3.06	224.7	225.4	2.63	22.26	High	High	High	High	High	High	1	
FALSE	High	Master Prc	P23588	eukaryotic	0	129.382	40	23	68	23	611	69.1	5.73	148.74	23	cell organi	cytosol	catalytic ac	Pf00076, F	1975	ENSG0000	EIF4B	12	RNA transp	Translation	mTORC1-n	22	0.967		0.581106389	12.69	17558.4	18163	4.59	4.37	High	High	High	High	High	High	1	
FALSE	High	Master Prc	O15460-1	Prolyl 4-hy	0	26.412	12	4	4	4	535	60.9	5.71	11.9	4	metabolic	cytosol;en	catalytic ac	Pf03171, F	8974	ENSG0000	P4HA2	5	Metabolic	Amino Aci	Collagen b	6	0.967		0.592426149	16.14	1111.5	1157.2	5.14	9.2	High	High	High	High	High	High	1	
FALSE	Medium	Master Prc	Q9H6E5	Speckle tar	0.014	1.851	2	1	1	1	874	93.8	6.16	2.79	1	metabolic	cytosol;nu	catalytic ac	Pf00076, F	64852	ENSG0000	TUT1	11				0	0.967		0.673010737	27.5	366.4	378.8	10.33	15.86	High	High	High	High	High	High	1	
FALSE	High	Master Prc	Q6RFH5-1	WD repeat	0	18.445	12	3	3	3	385	42.4	8.32	6.88	3		nucleus	protein bir	Pf00400	54663	ENSG0000	WDR74	11				0	0.967		0.814781665	40.86	811.9	977.8	35.6	10.84	High	High	High	High	High	High	1	
FALSE	High	Master Prc	Q9H845	Acyl-CoA d	0	80.439	37	17	27	17	621	68.7	7.96	75.21	17	cell organi	membrane	catalytic ac	Pf00441, F	28976	ENSG0000	ACAD9	3			Complex I	5	0.967		0.856305618	1.05	6655.9	6443.9	4.36	6.64	High	High	High	High	High	High	1	
FALSE	High	Master Prc	P21399	Cytoplasm	0	52.338	17	11	13	11	889	98.3	6.68	29.42	11	cellular ho	cytoplasm;	catalytic ac	Pf00330, F	48	ENSG0000	ACO1	9	Citrate cyc	Iron metab	Iron uptak	10	0.967		0.867636496	12.86	591.7	611.7	6.06	5.53	High	High	High	High	High	High	1	
FALSE	High	Master Prc	Q92616	eIF-2-alpha	0	168.191	21	42	56	42	2671	292.6	7.47	123.03	42	cell comm	cytoplasm;	enzyme re	Pf12074, F	10985		GCN1L1; G	12				0	0.967		0.90277481	12.48	11210.9	11588.2	9.08	6.22	High	High	High	High	High	High	1	
FALSE	Low	Master Prc	Q9UBP6	tRNA (guai	0.051	1.184	5	1	1	1	276	31.5	7.64	2.09	1	metabolic	cytosol;nu	catalytic ac	Pf02390, F	4234	ENSG0000	METTL1	12			tRNA mod	3	0.967		0.951394948	14.49	71.4	70.9	11.07	3.87	High	High	High	High	High	High	1	
FALSE	High	Master Prc	Q13428-1	Treacle prc	0	82.067	15	19	30	19	1488	152	9.04	45.53	19	cell organi	cytoplasm;	protein bir	Pf03546	6949	ENSG0000	TCOF1	5	Ribosome			1	0.967		0.954682789	25.3	6957.4	7194.1	16.34	4.8	High	High	High	High	High	High	1	
FALSE	High	Master Prc	Q9Y2Q9	28S riboso	0	12.168	13	1	3	1	187	20.8	9.1	8.24	1	cell organi	membrane	RNA bindi	Pf00575, F	28957	ENSG0000	MRPS28	8			Mitochonc	6	0.967		0.961704554	62.6	343.1	355	22.41	24.04	High	High	High	High	High	High	1	
FALSE	High	Master Prc	Q9UPT8	Zinc finger	0	9.95	3	3	4	3	1303	140.2	6.27	5.07	3			metal ion l	Pf00642	23211	ENSG0000	ZC3H4	19				0	0.966		0.402082718	7.33	348.6	369.1	7.79	17.4	High	High	High	High	High	High	1	
FALSE	High	Master Prc	P05023	Sodium/pc	0	194.327	37	29	82	16	1023	112.8	5.49	185.03	29	cell comm	endoplasm	catalytic ac	Pf00122, F	476	ENSG0000	ATP1A1	1	Gastric aci	Preimplan	Ion homeo	24	0.966		0.506919795	11.33	5552.4	5727.4	7.14	2.71	High	High	High	High	High	High	1	
FALSE	High	Master Prc	Q9H0S4	Probable A	0	29.809	23	8	10	8	455	50.6	9.1	27.88	8	cell death;	cytoplasm;	catalytic ac	Pf00270, F	51202	ENSG0000	DDX47	12			Major path	5	0.966		0.577750892	0.52	2642.2	2686.7	13.07	4.73	High	High	High	High	High	High	1	
FALSE	High	Master Prc	O15143	Actin-relat	0	36.716	15	3	6	2	372	40.9	8.35	19.6	3	cell organi	cytoplasm;	protein bir	Pf00400	10095	ENSG0000	ARPC1B	7	Fc gamma	Pathogenic	Regulation	21	0.966		0.821216097	7.3	2272.9	2237	10.96	4.15	High	High	High	High	High	High	1	
FALSE	High	Master Prc	O43242	26S protea	0	83.939	35	17	27	17	534	60.9	8.44	61.56	17	metabolic	cytosol;ext	catalytic ac	Pf01399, F	5709	ENSG0000	PSMD3	17	Proteasom	Proteasom	Regulation	153	0.965		0.172733575	1.53	6218.7	6714.1	6.67	3.8	High	High	High	High	High	High	1	
FALSE	High	Master Prc	Q15369-1	elongin-C [	0	28.562	53	5	12	5	112	12.5	4.78	31.88	5	metabolic	cytosol;nu	catalytic ac	Pf03931	6921	ENSG0000	TCEB1; ELC	8	Pathways i	Dual hijack	Oxygen-de	48	0.965		0.2305536	5.06	4337	4560.1	0.88	3.89	High	High	High	High	High	High	1	
FALSE	High	Master Prc	Q9NR30-1	Nucleolar l	0	270.476	46	40	108	39	783	87.3	9.28	275.07	40	cell differe	cytoplasm;	catalytic ac	Pf00270, F	9188	ENSG0000	DDX21	10			Major path	8	0.965		0.300819658	5.33	37082.4	38430.5	3	1.05	High	High	High	High	High	High	1	
FALSE	High	Master Prc	P62917	60S riboso	0	75.617	52	14	37	13	257	28	11.03	88.85	14	cell organi	cytoplasm;	RNA bindi	Pf00181, F	6132	ENSG0000	RPL8	8	Ribosome	Cytoplasm	Peptide ch	34	0.965		0.300982554	5.93	12696.7	13608	2.12	5.77	High	High	High	High	High	High	1	
FALSE	High	Master Prc	O00267	Transcripti	0	41.554	12	10	13	10	1087	120.9	5.06	30.78	10	cell organi	nucleus	DNA bindi	Pf00467, F	6829	ENSG0000	SUPT5H	19		Initiation c	RNA polym	32	0.965		0.457709952	13.76	1726.5	1800.6	23.1	9.16	High	High	High	High	High	High	1	
FALSE	High	Master Prc	P53701	Cytochrom	0	15.684	24	5	6	5	268	30.6	6.68	14.5	5	cell organi	membrane	catalytic ac	Pf01265	3052	ENSG0000	HCCS	X	Porphyrin			1	0.965		0.48155472	2.29	1266.9	1333.6	44.62	6.15	High	High	High	High	High	High	1	
FALSE	High	Master Prc	Q9C075	Keratin, ty	0	8.003	5	2	5	1	422	48.1	6.54	9.24	2	cell death	cytosol	structural	Pf00038	25984	ENSG0000	KRT23	17; CHR_H			Formation	3	0.965		0.490133519	13.12	599.1	620.8	6.26	8.86	High	High	High	High	High	High	1	[R234;

FALSE	High	Master Prc	P09382	Galectin-1	0	65.417	81	10	33	10	135	14.7	5.5	80.63	10	cell death;	cell surface	protein bir	Pf00337	3956	ENSG0000	LGALS1	22			Post-transl	4	0.965		0.526015018	16.64	10202.4	10708	15.21	7.89	High	High	High	High	High	High	1	
FALSE	High	Master Prc	Q16531	DNA dama	0	151.814	37	34	48	34	1140	126.9	5.26	117.48	34	cell organi	cytoplasm;	DNA bindi	Pf03178, P	1642	ENSG0000	DDB1	11	Viral carcir		Formation	19	0.965		0.563500261	9.64	12649.1	13963.4	9.86	4.83	High	High	High	High	High	High	1	
FALSE	High	Master Prc	P0CG38	POTE anky	0	44.16	7	7	58	1	1075	121.2	6.21	134.11	7			protein bir	Pf00022, P	653269	ENSG0000	POTEI	2				0	0.965		0.598682675	47.34	290.9	318.7	42.53	31.47	High	High	High	High	High	High	1	
FALSE	High	Master Prc	Q9BTD8-1	RNA-bindi	0	15.459	13	3	3	3	480	50.4	9.63	11.24	3	regulation	cytoplasm;	protein bir	Pf00076, P	79171	ENSG0000	RBM42	19				0	0.965		0.681770147	26.78	563.8	568.4	19.61	9.55	High	High	High	High	High	High	1	
FALSE	High	Master Prc	Q5JTH9-1	RRP12-like	0	42.254	9	8	11	8	1297	143.6	8.75	34.55	8		membrane	RNA bindi	Pf08161	23223	ENSG0000	RRP12	10				0	0.965		0.716595151	13.17	3667.3	3849.1	9.78	0.97	High	High	High	High	High	High	1	
FALSE	High	Master Prc	O75116	Rho-associ	0	16.523	6	7	8	7	1388	160.8	6.02	4.36	7	cell organi	cytoplasm;	catalytic a	Pf00069, P	9475	ENSG0000	ROCK2	2	Regulation	Androgen	EPHA-med	47	0.965		0.736551701	23.01	638.2	661.2	22.38	9.87	High	High	High	High	High	High	1	
FALSE	High	Master Prc	Q9NP81-1	Serine--tRI	0	43.001	29	10	10	10	518	58.2	8.13	27.38	10	cell organi	cytoplasm;	catalytic a	Pf00587, P	54938	ENSG0000	SARS2	19; CHR_H	Aminoacyl	Selenium I	Mitochonc	6	0.965		0.775943917	6.52	1360.8	1376.8	1.79	7.01	High	High	High	High	High	High	1	
FALSE	High	Master Prc	Q9H6S3	epidermal	0	47.625	21	10	13	10	715	80.6	6.84	31.77	10	regulation	cytoplasm;	protein bir	Pf00018, P	64787	ENSG0000	EPS8L2	11				0	0.965		0.80137704	34.17	1290.7	1337.8	24.99	12.04	High	High	High	High	High	High	1	
FALSE	High	Master Prc	Q3BBV0	Neuroblast	0.009	2.118	1	1	1	1	1214	139.3	4.78	1.79	1	metabolic	cytoplasm	catalytic a	Pf06758	55672		NBPF1	1				0	0.965		0.830020117	20.8	488	532.1	21.6	7.82	High	High	High	High	High	High	1	
FALSE	High	Master Prc	Q9H910-1	Jupiter mic	0	42.599	57	7	13	7	190	20.1	9.26	23.81	7		cytoplasm;			90861	ENSG0000	HN1L; JPT2	16				0	0.965		0.968317588	0.89	2196.1	2166.1	4.49	4.24	High	High	High	High	High	High	1	
FALSE	High	Master Prc	O43592	Exportin-T	0	17.629	7	5	6	5	962	109.9	5.39	9.59	5	transport	cytoplasm;	protein bir	Pf08389	11260	ENSG0000	XPOT	12	RNA trans		tRNA proc	4	0.964		0.147901697	1.38	241.8	251	6.99	3.13	High	High	High	High	High	High	1	
FALSE	High	Master Prc	O75608	Acyl-protei	0.002	3.798	7	2	3	2	230	24.7	6.77	5.36	2	metabolic	cytoplasm;	catalytic a	Pf00561, P	10434	ENSG0000	LYPLA1	8	Choline me		eNOS activ	6	0.964		0.243838649	1.92	685.6	716.4	10.66	4.28	High	High	High	High	High	High	1	
FALSE	High	Master Prc	O43447	peptidyl-pr	0	33.894	52	8	17	6	177	19.2	8.07	28.47	8	cell organi	cytoplasm;	catalytic a	Pf00160	10465	ENSG0000	PPIH	1	Spliceosom		mRNA Spli	5	0.964		0.245567498	2.84	1206.4	1260.5	1.68	6.05	High	High	High	High	High	High	1	
FALSE	High	Master Prc	Q8NDH3	Probable a	0.005	2.564	3	1	1	1	523	55.8	6.87	0	1	metabolic	cytoplasm;	catalytic a	Pf00883	79716	ENSG0000	NPEPL1	20				0	0.964		0.281542246	6.03	381	379.8	2.31	5.45	High	High	High	High	High	High	1	
FALSE	High	Master Prc	Q8NE01	Metal tran	0	12.284	6	3	3	3	707	76.1	6.09	8	3	transport	membrane	protein bir	Pf00571	26505	ENSG0000	CNNM3	2				0	0.964		0.282956892	6.32	601.3	627.6	3.43	3.63	High	High	High	High	High	High	1	
FALSE	High	Master Prc	P14678-1	Small nucl	0	67.589	23	6	30	6	240	24.6	11.19	91.37	6	cell organi	cytoplasm;	protein bir	Pf01423	6628	ENSG0000	SNRPB	20	Spliceosom	mRNA Pro	SLBP indep	17	0.964		0.405740226	9.52	7968.2	8942.2	4.79	12.38	High	High	High	High	High	High	1	
FALSE	High	Master Prc	Q99848	Probable r	0	55.965	37	10	20	10	306	34.8	10.1	50.51	10	metabolic	nucleus	protein bir	Pf05890	10969	ENSG0000	EBNA1BP2	1			Major patl	4	0.964		0.440633388	10.29	7480.8	7577.4	8.55	3.35	High	High	High	High	High	High	1	
FALSE	High	Master Prc	O60306	Intron-binc	0	51.079	11	11	13	11	1485	171.2	6.37	33.07	11	metabolic	membrane	catalytic a	Pf04851, P	9716	ENSG0000	AQR	15	Spliceosom		Dual incisi	11	0.964		0.456294452	3.97	4031.1	4173.6	3.72	0.99	High	High	High	High	High	High	1	
FALSE	High	Master Prc	P30044-1	Peroxiredo	0	122.876	69	14	50	14	214	22.1	8.7	139.06	14	cell death;	cytoplasm;	antioxidan	Pf00578, P	25824	ENSG0000	PRDX5	11	Peroxisom	Selenium I	Detoxificat	5	0.964		0.468212217	12.28	10294.9	10677.5	11.69	4.8	High	High	High	High	High	High	1	
FALSE	High	Master Prc	O43598-1	2'-deoxynu	0	14.339	44	4	5	4	174	19.1	5.05	6.37	4	cell differe	cytoplasm;	catalytic a	Pf05014	10591	ENSG0000	C6orf108;	6			Purine cat	4	0.964		0.515478246	20.35	77.9	100.2	14.69	24.82	High	High	High	High	High	High	1	
FALSE	High	Master Prc	P26232	Catenin alp	0	16.103	6	5	8	2	953	105.2	5.71	15.63	5	cell differe	cytoplasm;	protein bir	Pf01044	1496	ENSG0000	CTNNA2	2	Bacterial ir	Arrhythmc	CDO in my	13	0.964		0.524602008	124.83	678	703.2	115.29	27.81	High	High	High	High	High	High	1	
FALSE	High	Master Prc	Q9NZL9	Methionin	0	74.058	40	12	21	12	334	37.5	7.36	68.26	12	metabolic	cytosol;mi	catalytic a	Pf00106, P	27430	ENSG0000	MAT2B	5	Metabolic	Trans-sulfu	Ub-specific	15	0.964		0.592027399	10.02	9682.6	10045.9	5.16	2.76	High	High	High	High	High	High	1	
FALSE	High	Master Prc	Q6Q0C0-1	E3 ubiquiti	0	8.881	3	1	1	1	670	74.6	7.15	6.58	1	cell death;	membrane	catalytic a	Pf00097, P	84231	ENSG0000	TRAF7	16			Antigen pr	4	0.964		0.597744065	10.12	85.2	88.4	20.2	10.92	High	High	High	High	High	High	1	
FALSE	High	Master Prc	Q96J01	THO comp	0.001	4.385	5	2	2	2	351	38.7	6.09	4.61	2	metabolic	nucleus	protein bir	Pf00400, P	84321	ENSG0000	THOC3	5	RNA trans		Cleavage o	11	0.964		0.672376651	29.62	704.5	719	32.49	13.42	High	High	High	High	High	High	1	
FALSE	High	Master Prc	O14773	Tripeptidyl	0	30.388	17	6	8	6	563	61.2	6.48	17.37	6	cell differe	mitochond	catalytic a	Pf09286	1200	ENSG0000	TPP1	11	Lysosome		XBP1(S) ac	5	0.964		0.706653186	23.8	1318.6	1367.9	18.51	3.07	High	High	High	High	High	High	1	
FALSE	High	Master Prc	Q969Z0-1	Protein TB	0	23.053	10	6	7	6	631	70.7	7.42	17.95	6	metabolic	mitochond	catalytic a	Pf06743, P	9238	ENSG0000	TBRG4	7				0	0.964		0.873842393	6.71	1823.3	1938.7	13.6	4.66	High	High	High	High	High	High	1	
FALSE	High	Master Prc	P16144-3	Isoform Be	0	24.351	5	5	5	5	1805	200.6	6.3	9.76	5	cell commi	cell surface	protein bir	Pf00041, P	3691	ENSG0000	ITGB4	17		Integrin-m		8	0.964		0.997848824	7.51	373.9	396	15.31	5.4	High	High	High	High	High	High	1	
FALSE	High	Master Prc	P26640	Valine--tRI	0	138.247	28	26	42	26	1264	140.4	7.59	101.07	26	metabolic	cytoplasm;	catalytic a	Pf00043, P	7407; 571	ENSG0000	VARS; VAR	6; CHR_HS	Aminoacyl	Amino Aci	Cytosolic t	6	0.963		0.141688962	1.67	9776.1	10340.3	2.85	2	High	High	High	High	High	High	1	
FALSE	High	Master Prc	Q9UNM6	26S protea	0	43.88	25	9	12	9	376	42.9	5.81	32.05	9	cell organi	cytosol;ext	catalytic a	Pf01399	5719	ENSG0000	PSMD13	11	Epstein-Ba	Proteasom	Regulation	153	0.963		0.486750454	3.22	3198.9	3217.2	4.99	5.9	High	High	High	High	High	High	1	
FALSE	High	Master Prc	O15160	DNA-direct	0	19.329	18	4	6	4	346	39.2	5.5	19.61	4	metabolic	cytosol;nu	catalytic a	Pf01000, P	9533	ENSG0000	POLR1C	6	RNA polym		RNA Polym	29	0.963		0.549581282	0.95	881.5	912.7	19.22	3.4	High	High	High	High	High	High	1	
FALSE	High	Master Prc	Q96KA5-1	cleft lip an	0	11.415	5	2	3	2	538	62.2	8.56	10.23	2	cell death	membrane		Pf05602	81037	ENSG0000	CLPTM1L	5; CHR_HS				0	0.963		0.550879254	13.15	576.6	598.6	5.56	4.48	High	High	High	High	High	High	1	
FALSE	High	Master Prc	O15235	28S riboso	0	6.486	18	1	2	1	138	15.2	10.29	8.61	1	cell organi	membrane	protein bir	Pf00164	6183	ENSG0000	MRPS12	19; CHR_H	Ribosome		Mitochonc	7	0.963		0.654360464	26.98	183.5	172.2	31.49	18.69	High	High	High	High	High	High	1	
FALSE	High	Master Prc	P22626	heterogen	0	278.425	77	34	180	32	353	37.4	8.95	407.22	34	metabolic	cytoplasm;	DNA bindi	Pf00076, P	3181	ENSG0000	HNRNPA2I	7		mRNA Pro	Gene and	10	0.963		0.815160634	30.13	41543.4	43155.3	9.26	12.34	High	High	High	High	High	High	1	[R213;
FALSE	High	Master Prc	P51858	hepatoma-	0	126.367	61	15	52	14	240	26.8	4.73	128.69	15	cell prolife	cytoplasm;	DNA bindi	Pf00855	3068	ENSG0000	HDGF	1			XBP1(S) ac	4	0.962		0.039792886	1.56	12605	13238.2	4.78	3.82	High	High	High	High	High	High	1	[S165(100
FALSE	High	Master Prc	P55265-1	Double-str	0	107.513	23	24	45	24	1226	136	8.65	91.05	24	cell differe	cytoplasm;	catalytic a	Pf00035, P	103	ENSG0000	ADAR	1	Influenza A		C6 deamin	12	0.962		0.050761731	2.07	11652.5	11950.1	5.21	5.53	High	High	High	High	High	High	1	
FALSE	High	Master Prc	P83881	60S riboso	0	12.767	25	4	5	4	106	12.4	10.58	11.6	4	cell organi	cytoplasm;	protein bir	Pf00935	6173	ENSG0000	RPL36A	X	Ribosome	Cytoplasm	Peptide ch	34	0.962		0.355672456	7.26	2404.2	2572.7	13.42	7.21	High	High	High	High	High	High	1	
FALSE	High	Master Prc	P55957-1	BH3-intera	0	10.683	14	2	3	2	195	22	5.44	10.01	2	cell death;	cytoplasm;	protein bir	Pf06393	637	ENSG0000	BID	22	p53 signali	miRNA Re	Activation,	38	0.962		0.386068189	7.81	334.2	385.8	13.3	11.72	High	High	High	High	High	High	1	
FALSE	High	Master Prc	Q8IYS2-1	Uncharact	0	22.031	9	3	3	3	634	69.1	8.19	10.6	3		membrane		Pf10222	90231	ENSG0000	KIAA2013	1				0	0.962		0.538292439	26.33	337.9	372.5	21.32	19.86	High	High	High	High	High	High	1	
FALSE	High	Master Prc	Q9UMS4	Pre-mRNA	0	116.601	38	14	30	14	504	55.1	6.61	90.04	14	cell organi	cytoplasm;	catalytic a	Pf00400, P	27339	ENSG0000	PRPF19	11	Spliceosom		Dual incisi	12	0.962		0.577117433	12.14	7834.2	8143.2	2.04	6.76	High	High	High	High	High	High	1	
FALSE	High	Master Prc	P12955	xaa-Pro dip	0	13.096	9	3	3	3	493	54.5	6	6.24	3	metabolic		catalytic a	Pf00557, P	5184	ENSG0000	PEPD	19				0	0.962		0.851986649	17.49	336.4	356.7	19.88	1.57	High	High	High	High	High	High	1	
FALSE	High	Master Prc	Q68E01	Integrator	0	18.799	5	3	4	3	1043	118	5.8	12.33	3	metabolic	cytoplasm;	protein bir	Pf10189	65123		INTS3	1			RNA polym	3	0.962		0.973697491	34.92	1151.1	1196.4	32.4	7.26	High	High	High	High	High	High	1	
FALSE	High	Master Prc	Q10471	Polypeptid	0	79.85	30	16	23	16	571	64.7	8.35	35.24	16	metabolic	extracellul	catalytic a	Pf00535, P	2590	ENSG0000	GALNT2	1	Metabolic	Globo Sphi	COPI-indep	12	0.962		0.980153205	4.28	3587	3728.3	4.31	5.26	High	High	High	High	High	High	1	
FALSE	High	Master Prc	P62829	60S riboso	0	63.911	65	8	39	8	140	14.9	10.51	74.96	8	cell organi	cytoplasm;	protein bir	Pf00238	9349	ENSG0000	RPL23	17	Ribosome	Cytoplasm	Peptide ch	34	0.961		0.268728901	6.14	16520.3	17179.6	10.34	8.32	High	High	High	High	High	High	1	
FALSE	High	Master Prc	Q92890-1	Isoform Lo	0	14.214	12	4	5	4	343	38.7	6.34	10.89	4	metabolic	cytosol;en	catalytic a	Pf03152	7353		UFD1L; UF	22				0	0.961		0.353193818	6.4	1164	1196.5	20.85	4.41	High	High	High	High	High	High	1	
FALSE	High	Master Prc	P31939	bifunctiona	0	100.808	53	22	29	22	592	64.6	6.71	74.48	22	metabolic	cytosol;me	catalytic a	Pf01808, P	471	ENSG0000	ATIC	2	Metabolic	One Carbo	Purine ribc	9	0.961		0.447378189	11.73	5722.2	5954.4	8.36	5.47	High	High	High	High	High	High	1	
FALSE	High	Master Prc	Q16186	Proteasom	0	27.614	18	7	12	7	407	42.1	5.07	14.12	7	cell organi	cytoplasm;	enzyme re	Pf04683	11047	ENSG0000	ADRM1	20			UCH prote	5	0.961		0.453857968	9.54	2859.2	2975.5	4.73	2.07	High	High	High	High	High	High	1	
FALSE	High	Master Prc	P10768	S-formylglu	0	50.039	38	7	13	7	282	31.4	7.02	41.19	7	metabolic	cytoplasm;	catalytic a	Pf00326, P	2098	ENSG0000	ESD	13	Carbon me	Phase I bic	Glutathion	6	0.961		0.475842491	14.62	2623.4	2777.7	6.8	12.8	High	High	High	High	High	High	1	
FALSE	High	Master Prc	P25398	40S riboso	0	64.451	58	8	27	8	132	14.5	7.21	93.81	8	cell organi	cytoplasm;	RNA bindi	Pf01248	6206	ENSG0000	RPS12	6	Ribosome	Cytoplasm	Peptide ch	38	0.961		0.631434607	1.59	10481	10713	3.43	2.02	High	High	High	High	High	High	1	
FALSE	High	Master Prc	Q9HCE1	Putative he	0	14.86	7	6	6	6	1003	113.6	8.82	11.07	6	metabolic	cytoplasm;	catalytic a	Pf13086, P	4343	ENSG0000	MOV10	1			RUNX1 reg	27	0.961		0.656580668	2	1284.9	1337.6	12.68	3.51	High	High	High	High	High	High	1	
FALSE	High	Master Prc	Q14669-1	E3 ubiquiti	0	8.863	2	2	2	2	1992	220.3	8.48	5.29	2	metabolic	cytoplasm;	catalytic a	Pf00632, P	9320	ENSG0000	TRIP12	2	Ubiquitin r		Antigen pr	5	0.961		0.682062314	4.54	536.6	541.8	11.23	7.15	High	High	High	High	High	High	1	
FALSE	High	Master Prc	Q16644	MAP kinas	0	7.459	7	3	4	3	382	43	7.28	2.03	3	metabolic	cytoplasm;	catalytic a	Pf00069, P	7867	ENSG0000	MAPKAPK3	3	MAPK sign	Fas Ligand	p38MAPK	40	0.961		0.739748613	24.19	1613.5	1791.9	19.24	5.05	High	High	High	High	High	High	1	
FALSE	High	Master Prc	Q9BYD6	39S riboso	0	25.548	16	3	4	3	325	36.9	8.78	20.49	3	cell organi	membrane	protein bir	Pf00687, P	65008	ENSG0000	MRPL1	4	Ribosome		Mitochonc	7	0.961		0.840394597	75.99	224.1	233.1	43.79	35.04	High	High	High	High	High	High	1	
FALSE	High	Master Prc	Q7Z4H8	KDEL motif	0	15.585	9	4	6	4	507	58.5	8.24	16.03	4	metabolic	endoplasm	catalytic a	Pf00630, P	143888	ENSG0000	KDELC2	11				0	0.961		0.987023677	20	437	470.5	23.4	7.4	High	High	High	High	High	High	1	
FALSE	High	Master Prc	Q9NPD8	Ubiquitin-c	0	16.166	19	4	4	4	197	22.5	7.99	9.11	4	metabolic	cytoplasm;	catalytic a	Pf00179, P	29089	ENSG0000	UBE2T	1	Fanconi an	Gastric car	Synthesis c	8	0.96		0.145806017	3.88	2442.7	2543.6	6.12	2.57	High	High	High	High	High	High	1	
FALSE	High	Master Prc	P17931	Galectin-3	0	22.78	21	5	11	5	250	26.1	8.56	35.61	5	cell differe	cytoplasm;	protein bir	Pf00337	3958	ENSG0000	LGALS3	14		AGE/RAGE	RUNX2 reg	13	0.96		0.187845785	0.75	3182.7	3328.5	1.27	3.77	High	High	High	High	High	High	1	
FALSE	High	Master Prc	Q8TEQ6	gem-assoc	0	33.057	7	8	8	8	1508	168.5	6.62	15.47	8	cell organi	cytoplasm;	protein bir	Pf00400	25929	ENSG0000	GEMIN5	5	RNA trans		snRNP Ass	4	0.96		0.25433871	2.12	2384.5	2483.9	6.32	3.23	High	High	High	High	High	High	1	
FALSE	High	Master Prc	P39656	Dolichyl-di	0	54.992	27	10	18	10	456	50.8	6.55	38.47	10	metabolic	endoplasm	catalytic a	Pf03345	1650	ENSG0000	DDOST	1	N-Glycan b	AGE/RAGE	Advanced	13	0.96		0.339529995	5.12	3884.6	4048.3	14.9	2.78	High	High	High	High	High	High	1	
FALSE	High	Master Prc	Q9ULV4	coronin-1C	0	50.823	22	10	14	10	474	53.2	7.08	37.84	10	cell organi	cytoplasm;	protein bir	Pf00400, P	23603	ENSG0000	CORO1C	12				0	0.96		0.40335929	8.35	4687.3	4846.8	12.72	7.63	High	High	High	High	High	High	1	
FALSE	High	Master Prc	P25788-1	Proteasom	0	57.659	39	12	25	12	255	28.4	5.33	60.43	12	metabolic	cytoplasm;	catalytic a	Pf00227, P	5684	ENSG0000	PSMA3	14	Proteasom	Proteasom	Regulation	150	0.96		0.433861906	6.95	7248.1	7102.5	6.77	3.82	High	High	High	High	High	High	1	
FALSE	High	Master Prc	P48637	glutathion	0.001	4.286	3	1	1	1	474	52.4	5.92	3.82	1	metabolic	cytosol	catalytic a	Pf03199, P	2937	ENSG0000	GSS	20	Glutathion	Glutathion	Glutathion	17	0.96		0.617213606	2.76	318.7	332	16.35	3.27	High	High	High	High	High	High	1	
FALSE	High	Master Prc	P17812	CTP syntha	0	121.514	43	23	37	22	591	66.6	6.46	99.24	23	cell prolife	cytosol;me	catalytic a	Pf00117, P	1503	ENSG0000	CTPS1	1	Metabolic		Interconve	5	0.96		0.621587062	13.18	11522.6	11999.7	6.78	3.2	High	High	High	High	High	High	1	
FALSE	High	Master Prc	Q9NR31	GTP-bindir	0	62.961	53	8	16	6	198	22.4	6.68	47.39	8	cell organi	endoplasm	catalytic a	Pf00025, P	56681	ENSG0000	SAR1A	10	Legionello	Sterol Reg		3	0.96		0.647566219	18.47	4188.5	4361.3	28.59	6.12	High	High	High	High	High	High	1	
FALSE	High	Master Prc	Q9Y618-1	Nuclear re	0	12.58	2	4	4	4	2525	274.6	7.59	7.65	4	metabolic	membrane	DNA bindi	Pf00249, P	9612		NCOR2	12	Epstein-Ba	Androgen	NOTCH1 Ir	33	0.96		0.653417471	13.22	600.7	592.7	19.92	12.63	High	High	High	High	High	High	1	
FALSE	Medium	Master Prc	Q56NI9	N-acetyltra	0.047	1.229	3	1	1	1	601	68.3	9.39	0	1	cell differe	chromosor	catalytic a	Pf13878, P	157570	ENSG0000	ESCO2	8			Establishm	4	0.96		0.693220942	8.04	273	283.5	3.35	5.89	High	High	High	High	High	High	1	
FALSE	High	Master Prc	P48507	Glutamate	0	17.371	19	4	4	4	274	30.7	6.02	7.25	4	cell organi	cytosol	catalytic a	Pf00248	2730	ENSG0000	GCLM	1	Cysteine a	Glutathion	Glutathion	16	0.96		0.706094799	1.98	537.7	560.1	7.19	12.53	High	High	High	High	High	High	1	
FALSE	High	Master Prc	Q6P996-1	Pyridoxal-c	0	84.499	23	13	18	13	788	86.7	5.38	71.76	13	metabolic	Golgi	catalytic a	Pf00282, P	23042; 10	ENSG0000	PDXDC1; L	16; CHR_H				0	0.959		0.187628065	5.3	5501.4	5739.1	2.33	5.93	High	High	High	High	High	High	1	
FALSE	High	Master Prc	Q9BRQ8-1	Apoptosis-	0	9.465	8	3	6	3	373	40.5	9.11	6.69	3	cell death;	cytoplasm;	catalytic a	Pf00070, P	84883	ENSG0000	AIFM2	10		Apoptosis	TP53 Regu	7	0.959		0.224820622	2.39	329.4	356.8	6.72	2.72	High	High	High	High	High	High	1	
FALSE	High	Master Prc	P27348	14-3-3 pro	0	120.954	72	19	59	13	245	27.7	4.78	135.11	19	cell organi	cytoplasm;	protein bir	Pf00244	10971	ENSG0000	YWHAQ	2	Cell cycle;	Cell Cycle;	Translocat	37	0.959		0.236519309	5.85	6412.2	6758.8	3.37	3.05	High	High	High	High	High	High	1	
FALSE	High	Master Prc	Q15043	Zinc transp	0	7.936	5	2	3	2	492	54.2	5.33	3.22	2	cellular ho	cytoplasm;	transporte	Pf02535	23516	ENSG0000	SLC39A14	8		NRF2 path	Zinc influx	8	0.959		0.250365697	4.12	307.1	320.3	4.2	3.69	High	High	High	High	High	High	1	
FALSE	High	Master Prc	Q16881-1	Thioredoxi	0	161.554	45	20	51	19	649	70.9	7.39	155.91	20	cell prolife	cytoplasm;	antioxidan	Pf00070, P	7296	ENSG0000	TXNRD1	12	Pyrimidine	Selenium I	Metabolisr	28	0.959		0.289269603	7.22	15155.1	15075.4	7.73	4.11	High	High	High	High	High	High	1	
FALSE	High	Master Prc	Q9UL46	proteasom	0	41.041	43	9	17	9	239	27.4	5.73	43.44	9	metabolic	cytoplasm;	enzyme re	Pf02251, P	5721	ENSG0000	PSME2	14	Antigen pr	Proteasom	Regulation	153	0.959		0.338875939	3.94	3437.8	3830.2	10.57	11.19	High	High	High	High	High	High	1	
FALSE	High	Master Prc	P42229	Signal tran	0	44.352	9	6	13	2	794	90.6	6.39	44.27	6	metabolic	cytoplasm;	catalytic a	Pf00017, P	6776	ENSG0000	STAT5A	17	Prolactin s	IL-5 Signali	Interleukin	54	0.959		0.397784185	10.05	701.7	752.6	9.87	6.29	High	High	High	High	High	High	1	
FALSE	High	Master Prc	O15511-1	Actin-relat	0	5.291	8	1	1	1	151	16.3	5.67	3.46	1	cell organi	cytoplasm;	protein bir	Pf04699	10092	ENSG0000	ARPC5	1	Salmonella	Pathogeni	EPHB-med	25	0.959		0.439411532	12.08	269.5	286.5	9.98	7.71	High	High	High	High	High	High	1	
FALSE	High	Master Prc	Q96KB5	Lymphokin	0	14.975	10	4	6	4	322	36.1	5.12	11.51	4	metabolic	nucleus	catalytic a	Pf00069, P	55872	ENSG0000	PBK	8		4-hydroxyt		2	0.959		0.559881825	15.79	3498.1	3647.1	7.13	5.72	High	High	High	High	High	High	1	
FALSE	High	Master Prc	P61604	10 kDa hea	0	55.41	75	10	47	10	102	10.9	8.92	65.66	10	cell differe	cytoplasm;	metal ion l	Pf00166	3336	ENSG0000	HSPE1	2				0	0.959		0.734313139	34.18	19371.4	22399.4	11.38	20.95	High	High	High	High	High	High	1	
FALSE	High	Master Prc	Q6FI81-1	Anamorsin	0	13.161	15	4	7	4	312	33.6	5.62	17.05	4	cell death;	cytoplasm;	catalytic a	Pf05093, P	57019	ENSG0000	CIAPIN1	16			Cytosolic ir	2	0.959		0.746243806	27.03	688.4	717.9	27.05	5.29	High	High	High	High	High	High	1	
FALSE	High	Master Prc	P85037-1	Forkhead b	0	11.113	4	2	2	2	733	75.4	9.32	5	2	cell differe	nucleus	DNA bindi	Pf00250, P	221937	ENSG0000	FOXK1	7			UCH prote	4	0.959		0.774690357	27.99	102.6	104.7	12.23	11.16	High	High	High	High	High	High	1	
FALSE	High	Master Prc	O00410	Importin-5	0	252.046	37	33	82	31	1097	123.6	4.94	231.87	33	regulation	cytoplasm;	enzyme re	Pf02985, P	3843	ENSG0000	IPO5	13			vRNP Asse	6	0.958		0.106699444	2.18	19066	19973.9	3.98	2.31	High	High	High	High	High	High	1	
FALSE	High	Master Prc	Q15042-1	Rab3 GTPa	0	8.355	3	2	3	2	981	110.5	5.55	6.16	2	cell organi	cytoplasm;	enzyme re	Pf13890	22930	ENSG0000	RAB3GAP1	2			COPI-indep	7	0.958		0.126074807	3.32	993.4	1017.9	1.54	4.33	High	High	High	High	High	High	1	

FALSE	High	Master Prc	O60488-1	Long-chain	0	45.847	19	9	12	7	711	79.1	8.38	36.04	9	cell differe	cytoplasm;	catalytic ac	Pf00501	2182	ENSG0000	ACSL4	X	Peroxisom	Fatty Acid	Synthesis c	16	0.958		0.234391454	4.73	1810.9	1890.3	1.26	5.07	High	High	High	High	High	High	1	
FALSE	High	Master Prc	Q8WUM4	Programm	0	122.729	36	22	35	22	868	96	6.52	75.5	22	cell death;	cytoplasm;	protein bir	Pf03097, P	10015	ENSG0000	PDCD6IP	3	Endocytosi		Budding ar	9	0.958		0.276738013	4.24	5522.7	5766.1	1.06	3.04	High	High	High	High	High	High	1	
FALSE	Medium	Master Prc	P49642	DNA prima	0.024	1.521	2	1	1	1	420	49.9	8.21	2.29	1	cell organi	membrane	catalytic ac	Pf01896	5557	ENSG0000	PRIM1	12	Metabolic	Retinoblas	Activation	32	0.958		0.434024242	11.24	657.3	686.4	8	2.19	High	High	High	High	High	High	1	
FALSE	High	Master Prc	P51570	galactokin	0	21.485	21	6	6	6	392	42.2	6.46	15.52	6	metabolic	cytoplasm;	catalytic ac	Pf08544, P	2584	ENSG0000	GALK1	17	Metabolic		Galactose	10	0.958		0.444336832	11.66	658.4	691	10.59	2.01	High	High	High	High	High	High	1	
FALSE	High	Master Prc	P11940-1	Polyadenyl	0	203.942	50	33	102	22	636	70.6	9.5	199.74	33	metabolic	cytoplasm;	protein bir	Pf00076, P	26986	ENSG0000	PABPC1	8	mRNA surv	Translatior	AUF1 (hnR	24	0.958		0.478616063	12.44	16114.1	16815.9	5.18	4.43	High	High	High	High	High	High	1	[Q488;
FALSE	High	Master Prc	Q9NVJ2	ADP-ribosy	0	19.783	24	4	5	4	186	21.5	8.43	7.36	4	cell divisio	cytoplasm;	catalytic ac	Pf00025, P	55207	ENSG0000	ARL8B	3				0	0.958		0.638775973	46.58	681.3	804.2	54.92	9.65	High	High	High	High	High	High	1	
FALSE	High	Master Prc	Q12874	splicing fac	0	79.644	26	10	18	10	501	58.8	5.38	58.31	10	cell organi	nucleus;sp	metal ion l	Pf11931, P	10946	ENSG0000	SF3A3	1	Spliceosom	Exercise-in	mRNA Spli	7	0.958		0.692441446	2.86	3906.1	3968	14.52	1.86	High	High	High	High	High	High	1	
FALSE	High	Master Prc	O43427	Acidic fibro	0.002	3.608	4	1	1	1	364	41.9	6.48	0	1	regulation	membrane	protein bir	Pf05427	9158	ENSG0000	FIBP	11				0	0.958		0.844412296	4.11	490.4	507.3	10.13	2.81	High	High	High	High	High	High	1	
FALSE	High	Master Prc	P54727	UV excisio	0	63.457	26	9	23	8	409	43.1	4.84	66.31	9	cell organi	cytoplasm;	DNA bindi	Pf00240, P	5887	ENSG0000	RAD23B	9	Nucleotide		Josephin d	13	0.958		0.900642115	16.43	3564.1	3720.2	17.34	1.39	High	High	High	High	High	High	1	
FALSE	High	Master Prc	Q96FV9-1	THO comp	0	14.128	6	4	5	4	657	75.6	4.98	11.23	4	cell death;	cytoplasm;	DNA bindi	Pf00531, P	9984	ENSG0000	THOC1	18	Spliceosom		Cleavage o	11	0.958		0.971216644	40.31	1681.4	1754.4	21.94	9.4	High	High	High	High	High	High	1	
FALSE	High	Master Prc	P62333	26S protea	0	128.137	47	16	35	16	389	44.1	7.49	110.05	16	cell organi	cytoplasm;	catalytic ac	Pf00004, P	5706	ENSG0000	PSMC6	14	Epstein-Ba	Proteasom	Regulation	152	0.957		0.069998628	2.29	14535.7	14817	3.07	1.98	High	High	High	High	High	High	1	
FALSE	High	Master Prc	P49915	GMP synth	0	164.002	54	29	61	29	693	76.7	6.87	177.69	29	metabolic	cytoplasm;	catalytic ac	Pf00117, P	8833	ENSG0000	GMPS	3	Metabolic		Purine ribo	7	0.957		0.165501114	1.68	13454.4	13842.9	1.95	3.84	High	High	High	High	High	High	1	
FALSE	High	Master Prc	Q01844	RNA-bindi	0	75.157	15	7	23	7	656	68.4	9.33	76.71	7	metabolic	cytoplasm;	metal ion l	Pf00076, P	2130	ENSG0000	EWSR1	22	Transcripti			1	0.957		0.265499649	3.75	8406	9079.8	3.97	6.74	High	High	High	High	High	High	1	
FALSE	High	Master Prc	Q9UHX1-1	poly(U)-bir	0	69.101	28	12	19	12	559	59.8	5.29	61.83	12	cell death;	nucleus	catalytic ac	Pf00076, P	22827	ENSG0000	PUF60	8; CHR_HS	Spliceosom		mRNA Spli	5	0.957		0.365897986	8.59	8566.9	8948.3	11.59	2.8	High	High	High	High	High	High	1	
FALSE	High	Master Prc	P08397-1	porphobili	0	22.55	14	3	4	3	361	39.3	7.18	17.2	3	metabolic	cytoplasm;	catalytic ac	Pf01379, P	3145	ENSG0000	HMBS	11; CHR_H	Metabolic	Heme Bios	Heme bios	6	0.957		0.58828505	30.84	104.3	109	23.29	16.44	High	High	High	High	High	High	1	
FALSE	High	Master Prc	Q06830	peroxiredo	0	246.775	82	20	165	16	199	22.1	8.13	369.04	20	cell prolife	cytoplasm;	antioxidan	Pf00578, P	5052	ENSG0000	PRDX1	1	Peroxisom	Androgen	TP53 Regu	15	0.957		0.635566479	4.03	41411.7	39795.5	6.46	5.1	High	High	High	High	High	High	1	
FALSE	High	Master Prc	P46379-1	Large proli	0	44.109	10	8	15	8	1132	119.3	5.6	47.87	8	cell death;	cytoplasm;	protein bir	Pf00240, P	7917	ENSG0000	BAG6	6; CHR_HS				0	0.957		0.636715398	28.4	1575.9	1645.9	39.84	4.66	High	High	High	High	High	High	1	
FALSE	High	Master Prc	P43246-1	DNA mism	0	111.458	26	18	34	18	934	104.7	5.77	89.1	18	cell differe	membrane	catalytic ac	Pf00488, P	4436	ENSG0000	MSH2	2	Colorectal	Rac1/Pak1	Defective I	20	0.957		0.826546096	2.5	6177.7	6229.5	4.88	3.48	High	High	High	High	High	High	1	
FALSE	High	Master Prc	Q14839	Chromodo	0	20.454	4	4	4	3	1912	217.9	5.86	12.63	4	cell organi	cytoplasm;	catalytic ac	Pf00176, P	1108	ENSG0000	CHD4	12	Viral carcir		ERCC6 (CSI	21	0.957		0.921918792	39.83	725.6	759.6	27.11	5.74	High	High	High	High	High	High	1	
FALSE	High	Master Prc	Q02790	Peptidyl-pr	0	128.024	64	23	44	22	459	51.8	5.43	102.2	23	cell organi	cytoplasm;	catalytic ac	Pf00254, P	2288	ENSG0000	FKBP4	12	Estrogen si	Androgen	Attenuatio	8	0.956		0.091173601	3.34	14830.5	15594.1	2.92	0.64	High	High	High	High	High	High	1	
FALSE	High	Master Prc	P55786	puromycin	0	103.585	31	20	36	20	919	103.2	5.72	97.64	20	metabolic	cytoplasm;	catalytic ac	Pf01433, P	9520	ENSG0000	NPEPPS	17			Antigen pr	4	0.956		0.160117764	5.05	8480.3	8616.6	1.68	3.83	High	High	High	High	High	High	1	
FALSE	High	Master Prc	O95298-1	NADH deh	0.001	4.42	7	1	3	1	119	14.2	8.98	2.14	1	cell organi	cytoplasm;	catalytic ac	Pf06374	4718; 100	ENSG0000	NDUFC2; N	11	Alzheimer'	Electron Tr	Neutrophil	16	0.956		0.166177614	5.2	642.5	671.9	5.04	1.19	High	High	High	High	High	High	1	
FALSE	High	Master Prc	Q9UDY8	Mucosa-as	0	6.499	3	2	2	2	824	92.2	5.73	4.06	2	cell differe	cytoplasm;	catalytic ac	Pf00047, P	10892	ENSG0000	MALT1	18	NF-kappa I	B Cell Rece	CLEC7A/in	20	0.956		0.28312258	1.44	92.2	103.9	12.51	5.82	High	High	High	High	High	High	1	
FALSE	High	Master Prc	Q92598	Heat shock	0	204.681	44	31	61	27	858	96.8	5.39	171.97	31	metabolic	cytoplasm;	enzyme re	Pf00012	10808	ENSG0000	HSPH1	13	Protein pro		Scavenging	8	0.956		0.312945697	6.58	17821.5	18466.2	10.68	2.5	High	High	High	High	High	High	1	
FALSE	High	Master Prc	Q8N543	prolyl 3-hy	0	10.591	7	4	4	4	542	63.2	5.11	4.65	4	cell organi	cytoplasm;	catalytic ac	Pf10637, P	55239	ENSG0000	OGFOD1	16				0	0.956		0.322376011	7.11	833.8	874.2	6.11	0.83	High	High	High	High	High	High	1	
FALSE	High	Master Prc	P30046	D-dopachr	0.003	3.476	7	1	2	1	118	12.7	7.3	0	1	metabolic	cytoplasm	catalytic ac	Pf01187	1652	ENSG0000	DDT	22; CHR_H				0	0.956		0.356502444	2.78	111.7	128.4	31.22	7.77	High	High	High	High	High	High	1	
FALSE	High	Master Prc	P34896-1	Serine hyd	0	51.863	28	8	10	7	483	53	7.71	37.48	8	cell organi	cytoplasm;	catalytic ac	Pf00464	6470	ENSG0000	SHMT1	17	Glyoxylate	Folate Met	Metabolisr	18	0.956		0.35897439	9.29	652.5	683.2	8.21	4.19	High	High	High	High	High	High	1	
FALSE	High	Master Prc	O95373	Importin-7	0	85.277	19	16	26	15	1038	119.4	4.82	67.21	16	defense re	cytoplasm;	enzyme re	Pf03810, P	10527	ENSG0000	IPO7	11				0	0.956		0.614466341	21.82	6071	6098.6	15.28	8.83	High	High	High	High	High	High	1	
FALSE	High	Master Prc	Q9BY89-1	Uncharact	0	5.017	1	1	2	1	1806	196.6	8.47	6.23	1				Pf15327	85379	ENSG0000	KIAA1671	22				0	0.956		0.62673633	38.53	299.7	313.6	48.42	3.21	High	High	High	High	High	High	1	
FALSE	High	Master Prc	Q08257	Quinone o	0	69.45	48	12	20	12	329	35.2	8.44	58.54	12	cell organi	cytoplasm;	catalytic ac	Pf00107, P	1429	ENSG0000	CRYZ	1				0	0.956		0.645069251	6.1	4219.3	4158.8	6.04	4.7	High	High	High	High	High	High	1	
FALSE	High	Master Prc	P35052	Glypican-1	0.002	4.189	2	1	1	1	558	61.6	7.3	2.73	1	cell differe	cytosol;en	metal ion l	Pf01153	2817	ENSG0000	GPC1	2	Proteoglyc	VEGFA-VE(	Defective I	29	0.956		0.64781114	15.62	907.6	877.9	19.25	11.72	High	High	High	High	High	High	1	
FALSE	High	Master Prc	Q2TAY7	WD40 repe	0	29.524	17	6	12	6	513	57.5	7.18	41.48	6	cell organi	cytoplasm;	protein bir	Pf00400	55234	ENSG0000	SMU1	9				0	0.956		0.701812685	4.22	4724.5	4944.3	14.08	3.63	High	High	High	High	High	High	1	
FALSE	High	Master Prc	Q9P2J5	Leucine--tR	0	94.107	19	18	26	18	1176	134.4	7.3	72.38	18	cell commu	cytoplasm;	catalytic ac	Pf00133, P	51520	ENSG0000	LARS	5	Aminoacyl		Cytosolic t	9	0.956		0.793605108	13.08	4934.1	5161.3	16.16	5.48	High	High	High	High	High	High	1	
FALSE	High	Master Prc	Q96IU4	Protein AB	0	7.552	11	2	2	2	210	22.3	6.4	3.05	2	metabolic	cytoplasm;	catalytic ac	Pf00561, P	84836	ENSG0000	ABHD14B	3			Cytosolic s	4	0.956		0.865625086	63.63	491.8	572.7	45.45	15.39	High	High	High	High	High	High	1	
FALSE	High	Master Prc	P30740	Leukocyte	0	31.653	21	7	13	7	379	42.7	6.28	27.23	7	regulation	cytoplasm;	enzyme re	Pf00079	1992	ENSG0000	SERPINB1	6	Amoebiasi		Neutrophil	4	0.956		0.914556758	8.6	1652.1	1651.5	3.68	10.07	High	High	High	High	High	High	1	
FALSE	High	Master Prc	Q9BQG0	Myb-bindi	0	128.38	25	26	35	26	1328	148.8	9.28	91.9	26	cell commu	cytoplasm;	catalytic ac	Pf04931, P	10514	ENSG0000	MYBBP1A	17		Energy Me	B-WICH co	5	0.955		0.025435491	1.84	8553.7	8826.4	3.84	3.39	High	High	High	High	High	High	1	
FALSE	High	Master Prc	P07814	Bifunctiona	0	340.821	41	54	106	53	1512	170.5	7.33	308.99	54	cell organi	cytoplasm;	catalytic ac	Pf00458, P	2058	ENSG0000	EPRS	1	Aminoacyl	Parkinsons	tRNA modi	16	0.955		0.121362673	0.95	29787.9	31702.7	3.4	1.67	High	High	High	High	High	High	1	
FALSE	High	Master Prc	Q9UHA3	Probable ri	0	16.964	21	3	4	3	163	19.6	9.98	9.26	3	cell organi	nucleus;rib	protein bir	Pf01246	51187	ENSG0000	RSL24D1	15	Ribosome			1	0.955		0.124352036	2.14	759.8	829.1	6.27	5.28	High	High	High	High	High	High	1	
FALSE	High	Master Prc	Q9NZ01-1	Very-long-	0	23.648	20	6	11	6	308	36	9.45	31.27	6	metabolic	cytoplasm;	catalytic ac	Pf02544	9524	ENSG0000	TECR	19	Fatty acid		Synthesis c	8	0.955		0.135100809	4.21	2693.1	2819.9	2.09	5.43	High	High	High	High	High	High	1	
FALSE	High	Master Prc	P46063	ATP-depen	0	25.573	10	5	6	5	649	73.4	7.88	14.26	5	metabolic	chromosor	catalytic ac	Pf00270, P	5965	ENSG0000	RECQL	12				0	0.955		0.181017633	5.4	554.9	581.3	2.09	5.82	High	High	High	High	High	High	1	
FALSE	High	Master Prc	Q6UB35-1	Monofunct	0	24.577	7	6	6	5	978	105.7	8.06	12.67	6	metabolic	membrane	catalytic ac	Pf00763, P	25902	ENSG0000	MTHFD1L	6	Metabolic	One Carbo	Metabolisr	8	0.955		0.19871534	5.91	1785.5	1794.5	3.42	6.13	High	High	High	High	High	High	1	
FALSE	High	Master Prc	Q8WW01	tRNA-splic	0	20.827	33	3	4	3	171	18.6	4.58	9.83	3	metabolic	nucleus	catalytic ac	Pf09631	116461	ENSG0000	TSEN15	1			tRNA proc	3	0.955		0.234745419	2.41	998.3	1147.3	7.54	6.37	High	High	High	High	High	High	1	
FALSE	High	Master Prc	Q9NY93	Probable A	0	24.296	16	6	8	6	547	61.6	9.26	17.12	6	metabolic	cytoplasm;	catalytic ac	Pf00270, P	54606	ENSG0000	DDX56	7				0	0.955		0.2737563	7.16	1492.1	1562	1	7.11	High	High	High	High	High	High	1	
FALSE	High	Master Prc	O94964-2	Isoform 2 c	0	12.29	3	3	3	3	1661	183.7	6.52	5.1	3	regulation	Golgi		Pf09730, P	140710	ENSG0000	SOGA1	20				0	0.955		0.298867876	7.65	221	224	9.47	2.48	High	High	High	High	High	High	1	
FALSE	High	Master Prc	Q9BT22	chitobiosyl	0	6.174	6	2	2	2	464	52.5	7.23	4.45	2	metabolic	endoplasm	catalytic ac	Pf00534, P	56052	ENSG0000	ALG1	16	N-Glycan b		Defective A	10	0.955		0.463503858	16.85	300.7	314.8	19.45	11.81	High	High	High	High	High	High	1	
FALSE	High	Master Prc	Q12972-1	Nuclear inh	0	16.61	11	2	2	2	351	38.5	7.37	10.23	2	cell prolife	cytoplasm;	catalytic ac	Pf00498	5511	ENSG0000	PPP1R8	1				0	0.955		0.552088355	18.45	826.1	864.8	44.79	5.19	High	High	High	High	High	High	1	
FALSE	High	Master Prc	Q9NRP0-1	oligosacch	0.004	2.679	8	1	1	1	149	16.8	9.13	4.77	1	metabolic	endoplasm	catalytic ac	Pf04756	58505	ENSG0000	OSTC	4				0	0.955		0.6482046	33.99	519.6	543.9	20.95	15.65	High	High	High	High	High	High	1	
FALSE	High	Master Prc	Q9UPN9-2	Isoform Be	0	9.346	3	3	3	3	1110	120.5	6.67	4.41	3	metabolic	nucleus	catalytic ac	Pf00439, P	51592	ENSG0000	TRIM33	1		Ectoderm I		1	0.955		0.741149801	13.19	432.7	452.9	21.29	1.67	High	High	High	High	High	High	1	
FALSE	High	Master Prc	Q6IBS0	Twinfilin-2	0	24.713	23	7	9	5	349	39.5	6.84	14.31	7	cell organi	cytoplasm;	nucleotide	Pf00241	11344	ENSG0000	TWF2	3				0	0.955		0.745932993	12.12	871	900.7	18.36	6.41	High	High	High	High	High	High	1	
FALSE	High	Master Prc	Q8N8S7	Protein ena	0	25.81	12	7	10	7	591	66.5	6.93	9.27	7	cellular co	cytoplasm;	protein bir	Pf00568, P	55740	ENSG0000	ENAH	1	Regulation	Regulation	Generatior	10	0.955		0.871293373	11.34	2052.8	2133.1	6.62	6.67	High	High	High	High	High	High	1	
FALSE	High	Master Prc	P40938	replication	0	21.775	19	5	6	5	356	40.5	8.34	18.87	5	cell organi	nucleus	catalytic ac	Pf03215, P	5983	ENSG0000	RFC3	13	Nucleotide	Gastric car	Translesior	58	0.955		0.913276023	7.75	473.5	491.9	11.64	3.61	High	High	High	High	High	High	1	
FALSE	High	Master Prc	Q15363	Transmem	0	13.666	17	3	4	3	201	22.7	5.17	11	3	cell organi	endoplasm	protein bir	Pf01105	10959	ENSG0000	TMED2	12			COPI-depe	17	0.955		0.917336389	14.31	1161.2	1206.3	9	6.39	High	High	High	High	High	High	1	
FALSE	High	Master Prc	P13797	Plastin-3 [(	0	162.972	50	23	55	23	630	70.8	5.6	108.43	23	cell organi	cytoplasm;	metal ion l	Pf00036, P	5358	ENSG0000	PLS3	X				0	0.954		0.107110247	2.97	11135.5	11435.9	5.6	2.97	High	High	High	High	High	High	1	
FALSE	High	Master Prc	Q86UK7-1	E3 ubiquiti	0	12.403	5	3	4	3	904	98.6	8.4	7.46	3	metabolic		catalytic ac		90850		ZNF598	16				0	0.954		0.188633292	5.11	1174.6	1234.9	3.6	1.72	High	High	High	High	High	High	1	
FALSE	High	Master Prc	Q13123	Protein Re	0	14.968	8	5	7	5	557	65.6	6.64	14.65	5	metabolic	chromosor	protein bir	Pf07807, P	3550	ENSG0000	IK	5				0	0.954		0.203042763	3.5	1170.7	1198.5	7.13	12.39	High	High	High	High	High	High	1	
FALSE	High	Master Prc	Q15185	Prostaglan	0	14.014	13	2	5	2	160	18.7	4.54	14.44	2	cell organi	cytoplasm;	catalytic ac	Pf04969	10728	ENSG0000	PTGES3	12	Metabolic	Aryl Hydro	Attenuatio	18	0.954		0.241163745	5.46	1992.4	2090.5	4.37	3.15	High	High	High	High	High	High	1	
FALSE	High	Master Prc	P62937	peptidyl-pr	0	198.936	87	16	162	14	165	18	7.81	394.27	16	cell organi	cytoplasm;	catalytic ac	Pf00160	5478	ENSG0000	PPIA	7		Prolactin S	Budding ar	34	0.954		0.319113954	6.81	39541.5	39431.1	5.41	3.34	High	High	High	High	High	High	1	
FALSE	High	Master Prc	P61981	14-3-3 pro	0	122.006	74	18	78	11	247	28.3	4.89	157.59	18	cell organi	cytoplasm;	enzyme re	Pf00244	7532	ENSG0000	YWHAG	7	Epstein-Ba	Cell Cycle;	Chk1/Chk2	48	0.954		0.467974629	12.03	20950.4	21833.9	2.05	6.91	High	High	High	High	High	High	1	
FALSE	High	Master Prc	O95081	Arf-GAP do	0	19.664	12	4	4	4	481	48.9	9.11	6.81	4	regulation	membrane	enzyme re	Pf01412	3268	ENSG0000	AGFG2	7				0	0.954		0.814421343	37.43	167.7	175.8	11.55	18.04	High	High	High	High	High	High	1	
FALSE	High	Master Prc	Q15758-1	Neutral am	0	50.265	27	10	16	10	541	56.6	5.48	38.29	10	transport	membrane	protein bir	Pf00375	6510	ENSG0000	SLC1A5	19	Central car	TYROBP Ca	Amino acic	7	0.954		0.953379753	14.8	4436	5113.2	23.12	11.44	High	High	High	High	High	High	1	
FALSE	High	Master Prc	Q96P70	Importin-9	0	53.981	14	10	12	10	1041	115.9	4.81	42.27	10	transport	cytoplasm;	protein bir	Pf03810	55705	ENSG0000	IPO9	1				0	0.953		0.109877519	2.27	3538.6	3794.6	6.04	4.67	High	High	High	High	High	High	1	
FALSE	High	Master Prc	Q13501-1	sequestoso	0	110.706	58	15	31	15	440	47.7	5.22	92.56	15	cell commu	cytoplasm;	catalytic ac	Pf00564, P	8878	ENSG0000	SQSTM1	5	Osteoclast	Regulation	Interleukin	24	0.953		0.175626511	1.13	10402.9	11497.2	3.8	7.09	High	High	High	High	High	High	1	[T269(100
FALSE	High	Master Prc	Q9UI26	Importin-1	0	13.201	6	4	5	4	975	112.5	5.25	11.55	4	transport	cytoplasm;	protein bir	Pf03810	51194	ENSG0000	IPO11	5				0	0.953		0.231447917	0.69	2203.7	2301.8	13.5	1.48	High	High	High	High	High	High	1	
FALSE	High	Master Prc	O60566	Mitotic che	0.001	4.366	2	2	2	2	1050	119.5	5.27	1.88	2	cell death;	chromosor	catalytic ac	Pf08311	701	ENSG0000	BUB1B	15	HTLV-I infe	Cell Cycle	Inactivatio	29	0.953		0.440454115	15.82	209.7	220.1	35.31	7.95	High	High	High	High	High	High	1	
FALSE	High	Master Prc	Q15003	Condensin	0	27.06	10	5	5	5	741	82.5	5.06	15.19	5	cell divisio	chromosor	enzyme re	Pf05786	23397	ENSG0000	NCAPH	2			Condensat	5	0.953		0.562301462	12.83	435.5	457.1	35.57	9.03	High	High	High	High	High	High	1	
FALSE	High	Master Prc	P27635	60S riboso	0	43.714	38	10	27	10	214	24.6	10.08	56.2	10	cell organi	cytosol;en	protein bir	Pf00252	6134		RPL10	X	Ribosome	Cytoplasm	Peptide ch	34	0.953		0.899732118	2.11	13502	13575.8	3.38	7.38	High	High	High	High	High	High	1	
FALSE	High	Master Prc	P23528	Cofilin-1 [C	0	219.611	86	21	120	16	166	18.5	8.09	303.76	21	cell organi	cytoplasm;	protein bir	Pf00241	1072	ENSG0000	CFL1	11	Regulation	Brain-Deri	Platelet de	27	0.952		0.104443169	1.4	29789	31300.9	7.98	3.31	High	High	High	High	High	High	1	
FALSE	High	Master Prc	P06454-1	Prothymos	0	21.956	24	4	9	4	111	12.2	3.78	23.23	4	cell organi	cytosol;nu	protein bir		5757	ENSG0000	PTMA	2	Epstein-Ba			1	0.952		0.263350519	6.27	2291.4	2405.9	1.76	9.36	High	High	High	High	High	High	1	
FALSE	High	Master Prc	Q9P287	BRCA2 and	0	34.871	26	5	7	5	314	36	4.61	21.44	5	cell differe	cytoplasm;	enzyme re	Pf13862	56647	ENSG0000	BCCIP	10				0	0.952		0.305684201	7.36	2668.9	2663.9	14.51	4.43	High	High	High	High	High	High	1	
FALSE	High	Master Prc	Q9P289	serine/thre	0	37.425	25	10	11	4	416	46.5	5.29	22.24	10	cell commu	cytoplasm;	catalytic ac	Pf00069, P	51765	ENSG0000	MST4; STK	X			Apoptotic	4	0.952		0.30853825	3.25	820.7	874.2	14.34	5.77	High	High	High	High	High	High	1	
FALSE	High	Master Prc	Q9Y678	Coatomer	0	153.451	42	27	47	27	874	97.7	5.47	113.44	27	cellular co	cytoplasm;	protein bir	Pf01602, P	22820	ENSG0000	COPG1	3			COPI-depe	11	0.952		0.409505122	12.28	7965.6	7792.2	15.51	4.38	High	High	High	High	High	High	1	
FALSE	High	Master Prc	O60613-1	selenoprot	0	5.138	7	1	2	1	162	17.8	5.03	7.7	1	metabolic	endoplasm		Pf08806	9403		SEP15; SEL	1		Selenium M		1	0.952		0.567460986	20.43	136.9	140.3	4.56	14.8	High	High	High	High	High	High	1	
FALSE	High	Master Prc	Q16795	NADH deh	0	19.726	16	5	8	5	377	42.5	9.8	18.55	5	cell organi	membrane	catalytic ac	Pf01073, P	4704	ENSG0000	NDUFA9	12	Oxidative p	Electron Tr	Complex I	13	0.952		0.731131898	3.23	1272.8	1286.4	2.31	6.9	High	High	High	High	High	High	1	
FALSE	High	Master Prc	Q01105	Protein SET	0	83.792	45	12	40	12	290	33.5	4.32	101.69	12	cell organi	cytoplasm;	DNA bindi	Pf00956	6418	ENSG0000	SET	9		Histone M	HuR (ELAV	9	0.952		0.845068237	2.24	13048.4	12840.1	6.88	4.01	High	High	High	High	High	High	1	
FALSE	High	Master Prc	Q96CP2	FLYWCH fa	0	11.038	28	3	5	3	140	14.6	8.46	6.34	3			RNA bindi	Pf15423	114984	ENSG0000	FLYWCH2	16				0	0.952		0.879203624	29.3	947.6	1056.9	20.68	4.22	High	High	High	High	High	High	1	
FALSE	High	Master Prc	Q01650	large neutr	0	49.53	13	6	13	6	507	55	7.72	38.67	6	cell differe	cytoplasm;	protein bir	Pf00324, P	8140	ENSG0000	SLC7A5	16	Central car	Aryl Hydro	Tryptopha	14	0.951		0.169087146	0.16	3550.9	3734.4	6.71	3.88	High	High	High	High	High	High	1	
FALSE	High	Master Prc	P28288	ATP-bindin	0	35.331	16	9	12	9	659	75.4	9.36	24.89	9	cell organi	cytosol;me	catalytic ac	Pf00005, P	5825	ENSG0000	ABCD3	1	ABC transp	Nuclear Re	ABC transp	6	0.951		0.236674295	3.22	804.3	846	5.37	1.31	High	High	High	High	High	High	1	
FALSE	High	Master Prc	P24666-1	Low molec	0	18.348	29	3	4	3	158	18	6.74	15.4	3	metabolic	cytoplasm;	catalytic ac	Pf01451	52	ENSG0000	ACP1	2	Thiamine r	VEGFA-VE(		4	0.951		0.27269303	0.27	1882.8	1965.7	4.81	2.36	High	High	High	High	High	High	1	
FALSE	High	Master Prc	Q99497	protein/nu	0	135.705	81	16	51	16	189	19.9	6.79	128.4	16	cell commu	chromosor	antioxidan	Pf01965, P	11315	ENSG0000	PARK7	1	Parkinson's	Androgen		4	0.951		0.295460982	4.11	14202.3	15519.8	12.89	4.9	High	High	High	High	High	High	1	

FALSE	High	Master Prc	O75477	erlin-1 [OS	0	27.951	22	6	6	3	346	38.9	7.87	17.73	6	metabolic	endoplasm	protein bir	Pf01145	10613		ERLIN1	10			ABC-family	6	0.951	0.339327129	5.36	282.5	320.3	8.27	14.83	High	High	High	High	High	High	1	
FALSE	High	Master Prc	Q8IWX8	Calcium hc	0	33.642	11	6	9	6	916	103.6	9.04	22.52	6	cellular ho	cytoplasm;	protein bir	Pf01585, P	10523	ENSG0000	CHERP	19	Spliceosom		mRNA Spli	5	0.951	0.455059513	15.86	933.2	983.5	17.37	7.36	High	High	High	High	High	High	1	
FALSE	High	Master Prc	Q9BUE6	Iron-sulfur	0	7.969	19	2	2	2	129	14.2	9.07	2.69	2	cell organi:	mitochond	metal ion l	Pf01521	81689	ENSG0000	ISCA1	9			Mitochonc	2	0.951	0.595113836	17.43	626.5	652	10.67	1.73	High	High	High	High	High	High	1	
FALSE	High	Master Prc	P35270	Sepiapterii	0	16.005	19	4	5	4	261	28	8.05	13.43	4	metabolic	cytoplasm;	catalytic a	Pf00106	6697	ENSG0000	SPR	2	Folate bios		eNOS activ	6	0.951	0.699375068	26.99	253.9	266.4	12.56	9.61	High	High	High	High	High	High	1	
FALSE	High	Master Prc	O15173	Membrane	0	6.663	9	1	1	1	223	23.8	4.88	5.86	1	regulation	membrane	protein bir	Pf00173	10424	ENSG0000	PGRMC2	4				0	0.951	0.823828411	8.36	91.3	90.9	4.79	9.05	High	High	High	High	High	High	1	
FALSE	High	Master Prc	P23919	thymidylat	0	19.499	29	7	9	7	212	23.8	8.27	8.38	7	cell differe	cytoplasm;	catalytic a	Pf00406, P	1841	ENSG0000	DTYMK	2	Pyrimidine		Interconve	5	0.951	0.858889274	2.6	1318.1	1338.3	8.32	14.69	High	High	High	High	High	High	1	
FALSE	High	Master Prc	Q8TDN6	Ribosome	0	28.662	30	10	16	10	353	41.4	9.92	30.63	10	cell organi:	nucleus	RNA bindi	Pf04427	55299	ENSG0000	BRIX1	5		Gastric car		1	0.951	0.885884015	17.07	2366.5	2489.4	10.41	6.11	High	High	High	High	High	High	1	
FALSE	High	Master Prc	Q16891	MICOS con	0	41.904	15	10	14	10	758	83.6	6.48	34.62	10	cell organi:	membrane	protein bir	Pf09731	10989	ENSG0000	IMMT	2			Cristae for	3	0.951	0.998185905	4.28	2781.3	2831.9	8.3	4.33	High	High	High	High	High	High	1	
FALSE	High	Master Prc	Q9HB07	UPF0160 p	0	43.471	28	8	12	8	376	42.4	6.67	31.22	8	response t	mitochond		Pf03690	60314	ENSG0000	C12orf10	12				0	0.95	0.15660261	2.21	4304.9	4370.8	2.26	3.61	High	High	High	High	High	High	1	
FALSE	High	Master Prc	Q9C0B1-1	alpha-keto	0	9.842	7	2	2	2	505	58.2	5.22	5.18	2	metabolic	nucleus	catalytic a	Pf12933, P	79068	ENSG0000	FTO	16		FTO Obesit	Reversal o	4	0.95	0.229330769	6.47	105.1	110.6	11.02	6.17	High	High	High	High	High	High	1	
FALSE	High	Master Prc	O95861-1	3'(2'),5'-bis	0	9.053	8	1	1	1	308	33.4	5.69	3.48	1	metabolic	cytosol	catalytic a	Pf00459	10380	ENSG0000	BPNT1	1	Metabolic		Cytosolic s	6	0.95	0.550221698	8.11	44.1	45.4	3.5	10.61	High	High	High	High	High	High	1	
FALSE	High	Master Prc	Q96GX5-1	Serine/thre	0.001	4.259	2	2	2	1	879	97.3	5.99	0	2	cell divisio	cytoplasm;	catalytic a	Pf00069, P	84930	ENSG0000	MASTL	10			MASTL Fac	5	0.95	0.61170361	16.12	224.7	244.5	21.62	11.11	High	High	High	High	High	High	1	
FALSE	High	Master Prc	Q8IYB3	Serine/argi	0	74.698	18	15	21	15	904	102.3	11.84	40.58	15	metabolic	cytosol;nu	DNA bindi	Pf01480	10250	ENSG0000	SRRM1	1	mRNA surv	mRNA Pro	Cleavage o	14	0.95	0.617665909	5.63	6097.9	6131.4	2.9	7.22	High	High	High	High	High	High	1	[T220(100
FALSE	High	Master Prc	Q9BY77	Polymeras	0	12.703	7	2	2	2	421	46.1	9.99	4.85	2	metabolic	cytoplasm;	protein bir	Pf00076, P	84271	ENSG0000	POLDIP3	22			Cleavage o	9	0.95	0.626042726	6.79	269.5	281.6	18.14	11.25	High	High	High	High	High	High	1	
FALSE	High	Master Prc	P35580	Myosin-10	0	92.505	12	18	25	9	1976	228.9	5.54	64.64	18	cell organi:	cytoplasm;	catalytic a	Pf00063, P	4628	ENSG0000	MYH10	17	Salmonella	Regulation	RHO GTPa:	18	0.95	0.694347357	13.47	1767.3	1855.7	24.31	3.19	High	High	High	High	High	High	1	
FALSE	High	Master Prc	Q8NCF5-1	NFATC2-in	0	7.446	4	1	2	1	419	45.8	6.6	9.98	1	metabolic	cytoplasm;	protein bir	Pf11976	84901	ENSG0000	NFATC2IP	16				0	0.95	0.69966973	17.49	328.1	329.5	9.57	6.95	High	High	High	High	High	High	1	
FALSE	High	Master Prc	P47813	Eukaryotic	0	64.435	55	9	28	9	144	16.5	5.24	65.59	9	metabolic	cytosol	protein bir	Pf01176	1964; 107	ENSG0000	EIF1AX; LO	X; 1	RNA trans	Translatio	Formation	13	0.949	0.101192287	3.13	13262.4	13981.4	7.17	3.29	High	High	High	High	High	High	1	
FALSE	High	Master Prc	P10644	cAMP-dep	0	17.818	17	5	5	4	381	43	5.35	13.62	5	cell organi:	cytoplasm;	catalytic a	Pf00027, P	5573	ENSG0000	PRKAR1A	17	Insulin sigr	miRs in Mu	Signaling b	33	0.949	0.489597575	23.36	567.1	613	23.15	16.78	High	High	High	High	High	High	1	
FALSE	High	Master Prc	Q15427	Splicing fa	0	17.358	9	2	4	2	424	44.4	8.56	14.99	2	metabolic	nucleus;sp	protein bir	Pf00076, P	10262	ENSG0000	SF3B4	1	Spliceosom	mRNA Pro	mRNA Spli	7	0.949	0.570440958	5.24	655.2	641.1	4.89	8.76	High	High	High	High	High	High	1	
FALSE	High	Master Prc	P46926	glucosamir	0	25.884	24	4	5	4	289	32.6	6.92	12.96	4	metabolic	cytoplasm;	catalytic a	Pf01182	10007	ENSG0000	GNPDA1	5	Amino sug		Glycolysis	6	0.949	0.667747732	19.78	1522.4	1831.9	19.73	10.76	High	High	High	High	High	High	1	
FALSE	High	Master Prc	P54619-1	5'-AMP-ac	0	13.453	12	3	4	3	331	37.6	6.92	7.3	3	cell commu	cytosol;me	catalytic a	Pf00478, P	5571	ENSG0000	PRKAG1	12	Adipocytol	AMP-activ	Translocat	36	0.949	0.680665222	27.87	741.1	827.9	43.58	7.54	High	High	High	High	High	High	1	
FALSE	High	Master Prc	Q13613-1	Myotubula	0.003	3.186	2	1	2	1	665	74.6	7.14	5.97	1	metabolic	cytoplasm;	catalytic a	Pf02893, P	8776	ENSG0000	MTMR1	X	Metabolic		Synthesis c	8	0.949	0.869965175	4.23	379.2	394.9	4.68	10.01	High	High	High	High	High	High	1	
FALSE	High	Master Prc	Q07157	Tight junct	0	34.067	5	7	8	7	1748	195.3	6.7	20.42	7	cell commu	cytoplasm;	protein bir	Pf00018, P	7082	ENSG0000	TJP1	15	Adherens j		c-src medi	23	0.948	0.080362926	1.48	1146.8	1210.3	3.12	0.66	High	High	High	High	High	High	1	
FALSE	High	Master Prc	Q15054	DNA polym	0	8.555	5	2	2	2	466	51.4	9.35	5.86	2	cell organi:	cytoplasm;	catalytic a	Pf08597, P	10714	ENSG0000	POLD3	11	Base excisi	Homologo	Mismatch	53	0.948	0.20733172	3.86	1451.5	1571	1.81	7.65	High	High	High	High	High	High	1	
FALSE	High	Master Prc	Q9Y3U8	60S riboso	0	24.507	30	5	12	5	105	12.2	11.59	26.04	5	cell organi:	cytoplasm;	RNA bindi	Pf01158	25873	ENSG0000	RPL36	19	Ribosome	Cytoplasm	Peptide ch	34	0.948	0.387225722	12.23	6079	5990	15.71	5.88	High	High	High	High	High	High	1	
FALSE	High	Master Prc	P48147	prolyl endc	0	53.763	23	13	17	13	710	80.6	5.86	44.21	13	metabolic	cytoplasm;	catalytic a	Pf00326, P	5550	ENSG0000	PREP	6	Renin-angi			1	0.948	0.395877237	1.34	3030.8	3163.4	1.07	4.04	High	High	High	High	High	High	1	
FALSE	High	Master Prc	Q9UKV8	Protein arg	0	15.576	5	3	3	3	859	97.1	9.19	12.35	3	cell organi:	cytoplasm;	catalytic a	Pf02170, P	27161	ENSG0000	AGO2; EIF	8			Post-trans	26	0.948	0.402693708	5.45	997.9	1053.1	1.07	5.17	High	High	High	High	High	High	1	
FALSE	High	Master Prc	O60518	ran-bindin	0	14.282	6	5	8	3	1105	124.6	5.01	10.47	5	transport	cytoplasm;	protein bir	Pf12755, P	26953	ENSG0000	RANBP6	9				0	0.948	0.459769032	19.33	145.6	162	19.9	15.2	High	High	High	High	High	High	1	
FALSE	High	Master Prc	P25685	dnaJ homo	0	30.375	25	8	11	8	340	38	8.63	20.71	8	cell commu	cytoplasm;	catalytic a	Pf00226, P	3337	ENSG0000	DNAJB1	19	Influenza A	NRF2 path	MAPK6/M	13	0.948	0.65302278	16.66	1926.9	2031.8	6.98	5.91	High	High	High	High	High	High	1	
FALSE	High	Master Prc	P19532	Transcripti	0	5.308	4	3	3	3	575	61.5	5.58	2.28	3	metabolic	cytosol;nu	DNA bindi	Pf00010	7030	ENSG0000	TFE3	X	Transcripti	TGF-beta F		2	0.948	0.726901828	22.96	1469.6	1549.6	21.75	13.83	High	High	High	High	High	High	1	
FALSE	High	Master Prc	Q9Y320	Thioredoxi	0	14.926	21	4	4	4	296	34	8.69	8.12	4	cellular ho	membrane		Pf00085	51075	ENSG0000	TMX2	11				0	0.948	0.871949682	18.55	469	508	19.59	4.1	High	High	High	High	High	High	1	
FALSE	High	Master Prc	Q08211	Atp-depen	0	299.525	46	50	116	50	1270	140.9	6.84	311.12	50	cell death;	cytoplasm;	catalytic a	Pf00035, P	1660	ENSG0000	DHX9	1		mRNA Pro	TRAF6 me	34	0.948	0.889350727	0.65	23857.3	23833.1	5.18	4.91	High	High	High	High	High	High	1	[T138(99.
FALSE	High	Master Prc	Q9P2I0	Cleavage a	0	10.943	6	4	5	4	782	88.4	5.11	6.6	4	metabolic	membrane	protein bir	Pf00753, P	53981	ENSG0000	CPSF2	14	mRNA surv	mRNA Pro	mRNA 3'-e	16	0.947	0.115540265	0.29	794.7	921	8.73	8.39	High	High	High	High	High	High	1	
FALSE	High	Master Prc	Q96SZ5	2-aminoet	0.002	3.561	9	1	1	1	270	29.7	6.04	2.2	1	metabolic	cytosol;mi	catalytic a	Pf07847	84890	ENSG0000	ADO	10	Taurine an		Degradatic	6	0.947	0.22237296	0.38	235.4	268.6	15.38	7.4	High	High	High	High	High	High	1	
FALSE	High	Master Prc	O43847-1	Nardilysin	0	39.984	9	8	12	8	1150	131.5	5	34.37	8	cell organi:	cell surface	catalytic a	Pf00675, P	4898		NRD1; NRI	1				0	0.947	0.259807642	6.04	4156.4	4387.3	3.53	2.12	High	High	High	High	High	High	1	
FALSE	High	Master Prc	Q9BXJ9	N-alpha-ac	0	51.102	18	12	20	12	866	101.2	7.42	31.4	12	cell differe	cytoplasm;	catalytic a	Pf12569, P	80155	ENSG0000	NAA15	4				0	0.947	0.289154678	3.75	5658.8	5976.1	1.94	3	High	High	High	High	High	High	1	
FALSE	High	Master Prc	P35080-2	Isoform IIb	0	14.812	30	4	7	3	140	15.1	6.1	17.11	4	cell organi:	cytoplasm;	catalytic a	Pf00235	5217	ENSG0000	PFN2	3	Shigellosis,	Associatio		5	0.947	0.334368178	2.2	700.2	788.8	29.87	6.72	High	High	High	High	High	High	1	
FALSE	High	Master Prc	O15347	High mobil	0	11.322	10	2	6	2	200	23	8.37	8.1	2	defense re	chromosor	DNA bindi	Pf00505, P	3149	ENSG0000	HMGB3	X				0	0.947	0.5356216	13.32	1447.1	1492.4	42.11	15.19	High	High	High	High	High	High	1	
FALSE	High	Master Prc	P04424	argininosu	0	4.584	3	1	1	1	464	51.6	6.48	3.26	1	metabolic	cytoplasm;	catalytic a	Pf00206, P	435	ENSG0000	ASL	7	Alanine, as	Alanine an	Urea cycle	10	0.947	0.668128662	0.44	468	494.3	8.2	1.8	High	High	High	High	High	High	1	
FALSE	High	Master Prc	Q8N5I2	arrestin do	0.009	2.103	3	1	1	1	433	46	7.02	2.62	1	metabolic	membrane	protein bir	Pf00339, P	92714	ENSG0000	ARRDC1	9				0	0.947	0.67406003	14.06	400	422.1	25.91	4.84	High	High	High	High	High	High	1	
FALSE	High	Master Prc	O60716-1	Catenin de	0	81.657	24	17	22	17	968	108.1	6.23	62.85	17	cell organi:	cytoplasm;	protein bir	Pf00514	1500	ENSG0000	CTNND1	11	Rap1 signa	VEGFA-VE(	VEGFR2 m	18	0.947	0.777632203	5.55	5156.1	5446.4	9.88	0.39	High	High	High	High	High	High	1	
FALSE	High	Master Prc	P35637-1	RNA-bindi	0	150.644	28	14	59	12	526	53.4	9.36	182.92	14	metabolic	cytoplasm;	DNA bindi	Pf00076, P	2521	ENSG0000	FUS	16	Transcripti	mRNA Pro	mRNA Spli	6	0.947	0.814507262	3.86	11752.1	12374.1	9.4	0.69	High	High	High	High	High	High	1	[S462(50);
FALSE	High	Master Prc	Q8WUF5	RelA-assoc	0	8.057	3	2	2	2	828	89	6.81	2.7	2	cell death;	cytoplasm;	protein bir	Pf00018, P	10848	ENSG0000	PPP1R13L	19			Regulation	6	0.947	0.89236227	16.97	279.4	308.2	23.11	3.35	High	High	High	High	High	High	1	
FALSE	High	Master Prc	Q9Y5X3	sorting ne	0	17.78	11	4	5	4	404	46.8	6.76	11.96	4	cell organi:	cytoplasm;	protein bir	Pf00787, P	27131	ENSG0000	SNX5	20	Endocytosi		Golgi Asso	6	0.947	0.995621114	2.37	520.9	550.2	5.21	5.77	High	High	High	High	High	High	1	
FALSE	High	Master Prc	Q92917	G patch do	0	43.571	27	8	12	8	476	52.2	6.15	33.71	8	metabolic	nucleus	protein bir	Pf00467, P	27238	ENSG0000	GPKOW	X			mRNA Spli	4	0.946	0.259753613	7.96	1760	1908.4	8.03	1.65	High	High	High	High	High	High	1	
FALSE	High	Master Prc	P63151	Serine/thre	0	42.697	15	5	13	5	447	51.7	6.2	39.4	5	cell organi:	cytosol	catalytic a		5520	ENSG0000	PPP2R2A	8	Adrenergic	Wnt Signal	Cyclin D as	28	0.946	0.266390258	4.08	2602.8	2692.7	16.88	3.36	High	High	High	High	High	High	1	
FALSE	High	Master Prc	P57737	Coronin-7	0	5.941	4	2	2	2	925	100.5	5.8	3	2	cell organi:	cytoplasm;	protein bir	Pf00400, P	79585	ENSG0000	CORO7	16; CHR_H				0	0.946	0.493800889	15.77	148.1	156.6	6.9	3.92	High	High	High	High	High	High	1	
FALSE	High	Master Prc	P57678	Gem-assoc	0	14.177	4	4	4	4	1058	120	6.04	8.19	4	cell organi:	cytoplasm;	protein bir		50628	ENSG0000	GEMIN4	17	RNA trans		snRNP Ass	4	0.946	0.563495374	21.7	109.4	115.7	9.35	8.95	High	High	High	High	High	High	1	
FALSE	High	Master Prc	P63272	Transcripti	0.002	4.057	14	1	1	1	117	13.2	8.06	2.73	1	cell organi:	nucleus	metal ion l	Pf06093	6827	ENSG0000	SUPT4H1	17		Initiation c	HIV elonga	27	0.946	0.72714478	26.26	612	646.8	8.5	11.24	High	High	High	High	High	High	1	
FALSE	High	Master Prc	P61201-1	COP9 signa	0	38.148	17	8	17	8	443	51.6	5.53	45.91	8	cell differe	cytoplasm;	protein bir	Pf01399, P	9318	ENSG0000	COPS2	15			Cargo reco	13	0.945	0.058502648	1.81	8418.3	9024.9	3.12	2.11	High	High	High	High	High	High	1	
FALSE	High	Master Prc	Q7L2H7	Eukaryotic	0	38.104	18	7	10	7	374	42.5	5.63	22.26	7	metabolic	cytoplasm;	protein bir	Pf01399	10480	ENSG0000	EIF3M	11			Formation	11	0.945	0.227925196	7.28	3405.1	3426	10.76	5.29	High	High	High	High	High	High	1	
FALSE	High	Master Prc	Q9H490	Phosphatic	0	4.837	4	1	1	1	435	50	7.72	4.3	1	metabolic	endoplasm	catalytic a	Pf06728	128869	ENSG0000	PIGU	20	Metabolic		Attachmer	6	0.945	0.370686621	11.2	128	138.5	1.5	16.92	High	High	High	High	High	High	1	
FALSE	High	Master Prc	Q14978-1	nucleolar a	0	197.423	46	28	88	1	699	73.6	9.47	207.57	28	cell organi:	cytoplasm;	nucleotide	Pf05022	9221	ENSG0000	NOLC1	10				0	0.945	0.407374117	13.9	155.5	216.3	33.65	31.46	High	High	High	High	High	High	1	
FALSE	High	Master Prc	Q15417	Calponin-3	0	52.325	28	7	17	6	329	36.4	6.05	46.06	7	cell differe	cytoskelet	protein bir	Pf00307, P	1266	ENSG0000	CNN3	1				0	0.945	0.423318138	13.69	2190.9	2250.9	10.31	1.81	High	High	High	High	High	High	1	
FALSE	High	Master Prc	Q8N183	NADH deh	0	25.528	38	6	7	6	169	19.8	8.97	20.09	6	cell organi:	membrane	catalytic a	Pf05071	91942	ENSG0000	NDUFAF2	5			Complex I	5	0.945	0.509700141	2.55	955.2	997.6	41.19	3.38	High	High	High	High	High	High	1	
FALSE	High	Master Prc	Q96PK6-1	RNA-bindi	0	93.054	38	19	36	19	669	69.4	9.67	71.61	19	cell organi:	cytoplasm;	protein bir	Pf00076, P	10432; 10	ENSG0000	RBM14; RI	11			RUNX2 reg	5	0.945	0.926309801	2.35	8080.3	8682	12.13	3.44	High	High	High	High	High	High	1	
FALSE	High	Master Prc	Q7L5D6	Golgi to EF	0	5.888	6	2	2	2	327	36.5	5.41	3.78	2	regulation	cytoplasm;	protein bir	Pf04190	51608	ENSG0000	GET4	7				0	0.945	0.99815825	12.34	473.6	501.1	10.07	7.04	High	High	High	High	High	High	1	
FALSE	High	Master Prc	O00203-1	AP-3 comp	0	22.977	7	6	6	6	1094	121.2	6.04	11.09	6	cell organi:	Golgi;mem	protein bir	Pf01602, P	8546	ENSG0000	AP3B1	5	Lysosome		Signaling b	10	0.944	0.127637018	2.74	627.6	675.7	1.28	5.37	High	High	High	High	High	High	1	
FALSE	High	Master Prc	P11532-1	Dystrophin	0	8.324	1	3	3	2	3685	426.5	5.88	5.56	3	cell organi:	cell surface	metal ion l	Pf00307, P	1756		DMD	X	Arrhythmc	Arrhythmc	Striated M	11	0.944	0.229051532	1.75	677.9	726.5	6.8	4.01	High	High	High	High	High	High	1	
FALSE	High	Master Prc	Q16222-1	UDP-N-ace	0	11.361	8	4	4	4	522	58.7	6.33	7.21	4	metabolic	cytoplasm;	catalytic a	Pf01704	6675	ENSG0000	UAP1	1	Amino sug		Synthesis c	8	0.944	0.275168841	9	1208.5	1239.3	11.57	3.49	High	High	High	High	High	High	1	
FALSE	High	Master Prc	Q9UHD8-1	Septin-9 [C	0	73.372	36	15	25	15	586	65.4	8.97	49.24	15	cell divisio	cytoplasm;	catalytic a	Pf00735, P	10801	ENSG0000	SEPT9	17	Bacterial ir			1	0.944	0.286570702	5.89	5361.7	5681.2	4.37	1.49	High	High	High	High	High	High	1	
FALSE	High	Master Prc	P54578	Ubiquitin c	0	107.847	42	14	22	14	494	56	5.3	76.71	14	cell commu	cell surface	catalytic a	Pf00443, P	9097	ENSG0000	USP14	18			Ub-specific	4	0.944	0.315677009	8.49	4017.4	4664	7.19	11.16	High	High	High	High	High	High	1	
FALSE	High	Master Prc	P04181-1	Ornithine a	0	78.646	48	13	23	13	439	48.5	7.03	61.25	13	cell organi:	cytoplasm;	catalytic a	Pf00202	4942	ENSG0000	OAT	10	Arginine ai	Amino Aci	Amino acic	7	0.944	0.371932149	11.32	9303.4	9925	7.97	10.09	High	High	High	High	High	High	1	
FALSE	High	Master Prc	Q86YZ3	Hornerin [	0	14.287	5	3	4	3	2850	282.2	10.04	11.68	3	cell organi:	cytoplasm;	metal ion l		388697	ENSG0000	HRNR	1			Neutrophil	3	0.944	0.627512147	6.43	1341	1251.9	30.64	16.71	High	High	High	High	High	High	1	
FALSE	High	Master Prc	Q9BSE5	Agmatinas	0	6.811	9	1	2	1	352	37.6	7.59	4.94	1	metabolic	mitochond	catalytic a	Pf00491	79814	ENSG0000	AGMAT	1	Arginine ai		Agmatine l	6	0.944	0.952190783	35.18	341.2	361.5	10.21	20.68	High	High	High	High	High	High	1	
FALSE	High	Master Prc	Q7Z4S6-1	Kinesin-like	0	14.408	3	3	3	3	1674	187.1	6.42	6.8	3	cell organi:	cytoplasm;	catalytic a	Pf00038, P	55605	ENSG0000	KIF21A	12			COPI-depe	8	0.944	0.993322619	51.8	84.5	91.1	28.79	15.93	High	High	High	High	High	High	1	
FALSE	High	Master Prc	P26038	Moesin [O:	0	282.315	71	49	132	35	577	67.8	6.4	260.82	49	cell differe	cell surface	protein bir	Pf00373, P	4478	ENSG0000	MSN	X	Measles; P	Glial Cell D	Gene and	16	0.943	0.016858074	0.95	24127.8	25414.1	0.42	1.59	High	High	High	High	High	High	1	
FALSE	High	Master Prc	P48643	T-complex	0	257.493	74	35	115	34	541	59.6	5.66	333.46	35	metabolic	cytoplasm;	nucleotide	Pf00118	22948	ENSG0000	CCT5	5			BBSome-m	13	0.943	0.127368346	5.04	38760.9	40944.4	4.82	3.4	High	High	High	High	High	High	1	
FALSE	High	Master Prc	P06737-1	Glycogen p	0	30.203	9	6	9	5	847	97.1	7.17	24.56	6	cell death;	cytoplasm;	catalytic a	Pf00343	5836	ENSG0000	PYGL	14	Metabolic	TNF alpha	Neutrophil	14	0.943	0.191702245	1.81	1780	1887.9	0.67	3.15	High	High	High	High	High	High	1	
FALSE	High	Master Prc	P22234	multifunct	0	134.905	48	16	41	16	425	47	7.23	109.88	16	metabolic	cytoplasm;	catalytic a	Pf00731, P	10606	ENSG0000	PAICS	4	Metabolic		Purine ribc	6	0.943	0.218669618	3.2	11627	12299.8	3.92	1.75	High	High	High	High	High	High	1	
FALSE	High	Master Prc	O00743	Serine/thre	0	6.681	5	1	1	1	305	35.1	5.69	4.79	1	cell organi:	cytoplasm;	catalytic a	Pf00149	5537	ENSG0000	PPP6C	9			COPII (Coa	8	0.943	0.31087336	4.15	438.4	430.3	11.02	8.98	High	High	High	High	High	High	1	
FALSE	High	Master Prc	O94992	Protein HE	0	28.381	18	5	12	4	359	40.6	4.89	27.69	5	defense re	cytoplasm;	enzyme re	Pf15313	10614	ENSG0000	HEXIM1	17		Initiation c		1	0.943	0.341301405	11.72	1842.7	1923.9	11.75	6.98	High	High	High	High	High	High	1	
FALSE	High	Master Prc	P84098	60S riboso	0	51.427	41	11	37	11	196	23.5	11.47	79.02	11	cell organi:	cytosol;me	protein bir	Pf01280	6143	ENSG0000	RPL19	17	Ribosome	Cytoplasm	Peptide ch	34	0.943	0.379720658	1.39	8855.4	9158.3	1.48	4.83	High	High	High	High	High	High	1	
FALSE	High	Master Prc	P62995	transforme	0	46.498	38	11	21	8	288	33.6	11.25	49.71	11	cell organi:	membrane	protein bir	Pf00076, P	6434	ENSG0000	TRA2B	3	Spliceosom	mRNA Pro	mRNA Spli	6	0.943	0.434184841	9.38	2780.7	2902	5.81	3.27	High	High	High	High	High	High	1	
FALSE	High	Master Prc	O60493-1	sorting ne	0	14.736	19	4	6	3	162	18.8	8.66	15.62	4	cell organi:	cytoplasm;	protein bir	Pf00787	8724	ENSG0000	SNX3	6	Endocytosi		WNT ligan	8	0.943	0.494759993	3.24	535.6	551.6	8.34	3.77	High	High	High	High	High	High	1	
FALSE	High	Master Prc	Q13442	28 kDa hea	0	39	36	7	17	7	181	20.6	8.87	45.74	7	cell prolife	extracellul	RNA bindi	Pf10252	11333	ENSG0000	PDAP1	7			Neutrophil	3	0.943	0.566336225	22.04	6603.5	7071.4	19.7	1.81	High	High	High	High	High	High	1	
FALSE	High	Master Prc	Q9BQ69	O-acetyl-A	0	7.352	4	1	1	1	325	35.5	9.51	4.19	1	metabolic	membrane	catalytic a	Pf00820, P	28992	ENSG0000	MACROD1	11				0	0.943	0.587351474	16.26	421.8	485.1	46.28	6.3	High	High	High	High	High	High	1	
FALSE	High	Master Prc	Q8TEX9-1	Importin-4	0	36.17	10	7	11	7	1081	118.6	4.96	33.96	7	cell organi:	cytoplasm;	protein bir	Pf01602, P	79711	ENSG0000	IPO4	14				0	0.943	0.589358277	7.36	2573.8	2727	6.71	2.34	High	High	High	High	High	High	1	
FALSE	High	Master Prc	Q9NUQ6	SPATS2-lik	0	6.079	3	1	1	1	558	61.7	9.64	4.8	1		cytoplasm;	protein bir	Pf07139	26010	ENSG0000	SPATS2L	2				0	0.943	0.669121644	35.89	249.7	267.9	44.94	6.5	High	High	High	High	High	High	1	

FALSE	High	Master Prc	Q9NRF9	DNA polyn	0	9.235	18	3	3	3	147	16.8	4.74	2.67	3	cell organi	nucleus	catalytic a	Pf00808	54107	ENSG0000	POLE3	9	Purine met		Gap-filling	43	0.943	0.725496912	15.33	53	56.3	7.43	7.19	High	High	High	High	High	High	1	
FALSE	High	Master Prc	Q9UNS2	COP9 signa	0	35.793	22	6	8	6	423	47.8	6.65	24.88	6	cell organi	cytoplasm;	protein bir	Pf01399	8533	ENSG0000	COPS3	17			Cargo reco	13	0.943	0.851913573	1.74	1334	1374.7	14.76	4.22	High	High	High	High	High	High	1	
FALSE	High	Master Prc	Q9Y490	Talin-1 [OS	0	295.816	30	50	78	47	2541	269.6	6.07	213.94	50	cell organi	cell surface	protein bir	Pf00373, P	7094	ENSG0000	TLN1	9	Rap1 signa	Integrin-m	SEMA3A-P	43	0.942	0.048374845	1.11	13001.7	13374.2	3.65	3.42	High	High	High	High	High	High	1	
FALSE	High	Master Prc	Q6NZY4	Zinc finger	0	11.484	6	3	4	3	707	78.5	4.87	9.23	3	metabolic	nucleus;sp	metal ion l	Pf00098, P	55596	ENSG0000	ZCCHC8	12				0	0.942	0.109451339	4.05	674	729.9	1.23	3.62	High	High	High	High	High	High	1	
FALSE	High	Master Prc	Q12894	Interferon-	0.003	2.875	3	1	1	1	506	54.8	7.94	3.34	1		nucleus	protein bir	Pf04836, P	7866	ENSG0000	IFRD2	3				0	0.942	0.181340942	1.51	248.4	278.9	6.45	4.89	High	High	High	High	High	High	1	
FALSE	High	Master Prc	P18621	60S riboso	0	99.348	57	12	47	11	184	21.4	10.17	96.23	12	cell organi	cytosol;nu	protein bir	Pf00237	6139	ENSG0000	RPL17	18	Ribosome	Cytoplasm	Peptide ch	34	0.942	0.187248976	4.13	14103	14702.6	6.17	5.55	High	High	High	High	High	High	1	
FALSE	High	Master Prc	O43175	D-3-phospl	0	164.462	51	21	65	21	533	56.6	6.71	149.23	21	metabolic	cytosol	catalytic a	Pf00389, P	26227	ENSG0000	PHGDH	1	Glycine, se	Trans-sulfu	Serine bios	9	0.942	0.243136595	7.23	17289	18356.9	8	1.36	High	High	High	High	High	High	1	
FALSE	High	Master Prc	P05141	ADP/ATP t	0	116.829	50	19	62	8	298	32.8	9.69	107.77	19	regulation	membrane	protein bir	Pf00153	292	ENSG0000	SLC25A5	X	HTLV-I infe	Electron Tr	Vpr-media	15	0.942	0.24432548	2.49	5154.9	5469.8	9.75	4.64	High	High	High	High	High	High	1	
FALSE	Medium	Master Prc	O60725	protein-s-i	0.012	1.939	3	1	1	1	284	31.9	7.96	1.91	1	cell organi	endoplasm	catalytic a	Pf04140, P	23463	ENSG0000	ICMT	1	Terpenoid		Gamma ca	4	0.942	0.921533913	24.1	505.6	544.6	21.46	1.34	High	High	High	High	High	High	1	
FALSE	High	Master Prc	O15294	UDP-N-ace	0	25.929	7	5	6	5	1046	116.9	6.7	18.85	5	cell death;	cytoplasm;	catalytic a	Pf00515, P	8473	ENSG0000	OGT	X	Other type	Ectoderm	HATs acety	10	0.941	0.062460596	0.59	1875.2	2016	1.89	1.98	High	High	High	High	High	High	1	
FALSE	High	Master Prc	P56945	Breast can	0	31.504	13	6	7	6	870	93.3	5.67	20.08	6	cell divisio	cytoplasm;	protein bir	Pf00018, P	9564	ENSG0000	BCAR1	16	Focal adhe	Integrin-m	p130Cas lii	29	0.941	0.073930245	3.51	1104.8	1219.5	5.76	7.54	High	High	High	High	High	High	1	
FALSE	High	Master Prc	P0DMV8	heat shock	0	417.339	73	44	242	23	641	70	5.66	643.81	44	cell organi	cytoplasm;	catalytic a	Pf00012, P	3304; 330	ENSG0000	HSPA1B; H	6; CHR_HS	Longevity	Apoptosis	AUF1 (hnR	38	0.941	0.107537728	4.29	36993	38224.8	1.67	3.59	High	High	High	High	High	High	1	[R469]
FALSE	High	Master Prc	Q9H2M9	Rab3 GTPa	0	8.014	2	2	2	2	1393	155.9	5.62	5.74	2	regulation	cytoplasm;	enzyme re	Pf14655, P	25782	ENSG0000	RAB3GAP2	1			COPI-indep	7	0.941	0.201543886	7.4	423.5	431.8	2.4	6.63	High	High	High	High	High	High	1	
FALSE	High	Master Prc	P53999	Activated	0	151.962	65	13	63	13	127	14.4	9.6	229.65	13	cell organi	nucleus	catalytic a	Pf02229	10923	ENSG0000	SUB1	5				0	0.941	0.301813001	10.15	30734.2	30514	18.28	10.34	High	High	High	High	High	High	1	
FALSE	High	Master Prc	Q9UGV2	protein NC	0	19.556	18	4	4	4	375	41.4	5.31	13.1	4	cell differe	cytoplasm		Pf00561, P	57446	ENSG0000	NDRG3	20				0	0.941	0.537668586	7.85	402.1	419.5	43.37	2.08	High	High	High	High	High	High	1	
FALSE	High	Master Prc	Q9UKK3	Poly [ADP-	0	11.023	1	2	2	2	1724	192.5	5.66	5.12	2	cell death;	cytoplasm;	catalytic a	Pf00092, P	143	ENSG0000	PARP4	13	Apoptosis;	NAD+ bios	Nicotinam	8	0.941	0.602453745	78.16	399	424.2	76.05	20.79	High	High	High	High	High	High	1	
FALSE	Low	Master Prc	O60293	Zinc finger	0.054	1.164	1	1	1	1	1989	226.2	8.13	0	1	metabolic	nucleus	metal ion l	Pf08317, P	196441	ENSG0000	ZFC3H1	12				0	0.941	0.632252436	25.69	167.4	177.9	39.17	12.76	High	High	High	High	High	High	1	
FALSE	High	Master Prc	Q53H96	Pyrroline-5	0	6.051	7	1	1	1	274	28.6	7.72	4.95	1	metabolic	cytoplasm;	catalytic a	Pf03807, P	65263	ENSG0000	PYCRL; PY(	8; CHR_HS	Biosynthes	Urea cycle	Amino acic	7	0.941	0.76322369	24.53	152.9	162.8	7.91	13.45	High	High	High	High	High	High	1	
FALSE	High	Master Prc	P78318	Immunoglo	0	6.908	8	2	2	2	339	39.2	5.38	5.77	2	regulation	cytoplasm;	enzyme re	Pf04177	3476	ENSG0000	IGBP1	X				0	0.941	0.768498497	4.29	624.3	663.7	20.81	2.9	High	High	High	High	High	High	1	
FALSE	High	Master Prc	Q00796	Sorbitol de	0	24.13	22	6	6	6	357	38.3	7.97	13.19	6	cellular co	cytosol;me	catalytic a	Pf00107, P	6652	ENSG0000	SORD	15	Pentose ar	Polyol Patl	Catabolism	9	0.941	0.826709609	8.16	852.7	906.1	15.48	4.18	High	High	High	High	High	High	1	
FALSE	High	Master Prc	Q9H3U1	Protein un	0	44.55	11	9	12	8	944	103	6.07	29.67	9	cell differe	cytoplasm;	protein bir	Pf00515, P	55898	ENSG0000	UNC45A	15				0	0.94	0.015511831	1.37	1046.5	1113.2	1.03	2.21	High	High	High	High	High	High	1	
FALSE	High	Master Prc	Q13045-1	protein flig	0	32.506	7	9	16	9	1269	144.7	6.05	25.27	9	cell organi	cytoplasm;	protein bir	Pf00626, P	2314	ENSG0000	FLII	17; CHR_H				0	0.94	0.170855745	6.44	2301.7	2417.5	3.7	4	High	High	High	High	High	High	1	
FALSE	High	Master Prc	P40763	Signal tran	0	90.242	31	15	21	15	770	88	6.3	50.23	15	cell differe	cytoplasm;	DNA bindi	Pf00017, P	6774	ENSG0000	STAT3	17	Jak-STAT si	IL-5 Signali	Interleukin	106	0.94	0.185426579	4.01	3930.6	4068.3	13.51	4.36	High	High	High	High	High	High	1	
FALSE	High	Master Prc	O95400	CD2 antige	0	29.497	23	6	7	6	341	37.6	4.61	17.34	6	cell organi	cytoplasm;	protein bir	Pf02213	10421	ENSG0000	CD2BP2	16		mRNA Pro	mRNA Spli	5	0.94	0.217311498	2.15	2353.9	2756.1	9.21	6.88	High	High	High	High	High	High	1	
FALSE	High	Master Prc	O75821	Eukaryotic	0	30.766	21	7	13	7	320	35.6	6.13	41.93	7	metabolic	cytoplasm;	protein bir	Pf00076, P	8666	ENSG0000	EIF3G	19	RNA trans	Translatior	Formation	13	0.94	0.293023371	0.31	3673.4	4378.8	18.61	10.66	High	High	High	High	High	High	1	
FALSE	High	Master Prc	P52701	DNA mism	0	110.432	22	22	26	22	1360	152.7	6.9	67.24	22	cell organi	chromosor	catalytic a	Pf00488, P	2956	ENSG0000	MSH6	2	Colorectal	Signaling F	Defective I	14	0.94	0.300638552	10.74	6726.6	6891.3	3.85	7.25	High	High	High	High	High	High	1	
FALSE	High	Master Prc	Q9BZG1	Ras-relate	0	15.052	5	1	3	1	259	29	7.88	8.72	1	cell organi	cytoplasm;	catalytic a	Pf00025, P	83871	ENSG0000	RAB34	17			RAB geran	3	0.94	0.343302867	12.39	258.6	263.9	9.04	4.16	High	High	High	High	High	High	1	
FALSE	High	Master Prc	P33992	DNA replic	0	72.745	27	16	20	16	734	82.2	8.37	42.58	16	cell divisio	membrane	catalytic a	Pf00493, P	4174	ENSG0000	MCM5	22	Cell cycle;	Cell Cycle;	M/G1 Trar	25	0.94	0.360658616	8.76	5877.3	5904.4	5.96	7.68	High	High	High	High	High	High	1	
FALSE	High	Master Prc	Q96CN7	Isochorism	0.001	4.286	5	1	1	1	298	32.2	7.39	4.41	1	metabolic	cytoplasm	catalytic a	Pf00857	51015	ENSG0000	ISOC1	5				0	0.94	0.418521697	15.72	559.1	594.9	11.96	5.72	High	High	High	High	High	High	1	
FALSE	High	Master Prc	P62899	60S riboso	0	31.889	44	9	26	9	125	14.5	10.54	29.77	9	cell organi	cytosol;me	protein bir	Pf01198	6160	ENSG0000	RPL31	2	Ribosome	Cytoplasm	Peptide ch	34	0.94	0.473179998	4.41	7216.6	7810.2	1.06	7.56	High	High	High	High	High	High	1	
FALSE	High	Master Prc	Q9NUQ2	1-acyl-sn-g	0	7.761	6	1	1	1	364	42	9.1	4.68	1	cell differe	endoplasm	catalytic a	Pf01553	55326	ENSG0000	AGPAT5	8	Glycerolipi	Triacylglyc	Synthesis c	10	0.94	0.605054401	5.17	92.2	99.1	30.43	0.64	High	High	High	High	High	High	1	
FALSE	High	Master Prc	P49755	Transmem	0	34.04	31	7	11	7	219	25	7.44	26.91	7	cell organi	endoplasm	protein bir	Pf01105	10972	ENSG0000	TMED10	14		mRNA Pro	COPI-depe	14	0.94	0.605575582	25.03	3763.6	3848.4	18.59	4.91	High	High	High	High	High	High	1	
FALSE	High	Master Prc	P26599	Polypyrimi	0	199.942	62	21	63	21	531	57.2	9.17	222.91	21	metabolic	membrane	protein bir	Pf00076, P	5725	ENSG0000	PTBP1	19		mir-124 pr	mRNA Spli	11	0.94	0.683369892	7.05	12136.3	12911.4	4.31	6.04	High	High	High	High	High	High	1	
FALSE	High	Master Prc	Q6PKG0-1	La-related	0	43.733	10	9	16	9	1096	123.4	8.82	39.45	9	cell prolife	cytoplasm;	protein bir	Pf05383	23367		LARP1	5				0	0.94	0.955093229	16.06	3988.2	4243.1	12.88	6.58	High	High	High	High	High	High	1	
FALSE	High	Master Prc	Q16643	drebrin [O	0	67.143	21	10	20	10	649	71.4	4.45	59.79	10	cell commi	cytoplasm;	protein bir	Pf00241	1627	ENSG0000	DBN1	5				0	0.94	0.960468635	11.53	2571.3	2736.7	11.04	7.52	High	High	High	High	High	High	1	
FALSE	High	Master Prc	P62851	40S riboso	0	44.222	60	12	37	12	125	13.7	10.11	62.6	12	cell organi	cytosol;nu	catalytic a	Pf03297	6230	ENSG0000	RPS25	11; CHR_H	Ribosome	Cytoplasm	Peptide ch	38	0.939	0.152388157	3.3	12394.2	13197.3	4.3	2.83	High	High	High	High	High	High	1	
FALSE	High	Master Prc	P62266	40S riboso	0	59.302	54	9	29	9	143	15.8	10.49	84.03	9	cell organi	cytosol;me	protein bir	Pf00164	6228	ENSG0000	RPS23	5	Ribosome	Cytoplasm	Peptide ch	38	0.939	0.18454539	3.42	12558.8	13534.1	3.69	5.37	High	High	High	High	High	High	1	
FALSE	High	Master Prc	Q9UNQ2	Probable d	0	11.098	9	2	2	2	313	35.2	9.99	4.4	2	metabolic	cytosol;nu	catalytic a	Pf00398, P	27292	ENSG0000	DIMT1	5			rRNA mod	4	0.939	0.28124311	6.02	730.7	795.5	3.96	15.79	High	High	High	High	High	High	1	
FALSE	High	Master Prc	Q9H223	EH domain	0.004	2.765	2	1	1	1	541	61.1	6.76	2.74	1	cell organi	endoplasm	metal ion l	Pf00350, P	30844	ENSG0000	EHD4	15	Endocytosi			1	0.939	0.306729244	7.38	1041.4	1193.6	4.32	15.14	High	High	High	High	High	High	1	
FALSE	High	Master Prc	O15372	Eukaryotic	0	83.462	38	12	33	12	352	39.9	6.54	84.19	12	cell organi	cytoplasm;	protein bir	Pf01398	8667	ENSG0000	EIF3H	8	Measles; R	Translatior	Formation	14	0.939	0.333062655	6.08	7120.8	7630.4	6.6	0.39	High	High	High	High	High	High	1	
FALSE	High	Master Prc	P21333	Filamin-A [	0	709.777	47	93	292	83	2647	280.6	6.06	843.06	93	cell organi	cytoplasm;	protein bir	Pf00307, P	2316	ENSG0000	FLNA	X	MAPK sign	Androgen	Platelet de	22	0.939	0.595507106	10.61	75916.9	80836.7	7.4	3.28	High	High	High	High	High	High	1	
FALSE	High	Master Prc	O14818-1	Proteasom	0	86.45	50	11	25	11	248	27.9	8.46	68.61	11	metabolic	cytoplasm;	catalytic a	Pf00227, P	5688	ENSG0000	PSMA7	20	Proteasom	Proteasom	Regulation	150	0.939	0.602936712	10.59	9488.6	10109	7.05	3.8	High	High	High	High	High	High	1	
FALSE	High	Master Prc	P33176	Kinesin-1 h	0	88.6	22	17	23	17	963	109.6	6.51	53.24	17	cell differe	cytoplasm;	catalytic a	Pf00225, P	3799	ENSG0000	KIF5B	10	Dopamine	Insulin Sigi	COPI-depe	21	0.939	0.919879585	19.38	6072.6	6469.1	15.49	6.51	High	High	High	High	High	High	1	
FALSE	High	Master Prc	P46779	60S riboso	0	54.496	39	9	36	9	137	15.7	12.02	86.23	9	cell organi	cytosol;me	protein bir	Pf01778	6158	ENSG0000	RPL28	19	Ribosome	Cytoplasm	Peptide ch	34	0.939	0.956325151	7.49	11819.6	12495.4	7.2	7.75	High	High	High	High	High	High	1	
FALSE	High	Master Prc	O14980	Exportin-1	0	124.269	26	21	37	21	1071	123.3	6.06	108.26	21	cell organi	chromosor	protein bir	Pf03810, P	7514	ENSG0000	XPO1	2	Epstein-Ba	eIF5A regu	Rev-media	49	0.938	0.012490906	0.33	9501	10072.3	2.45	3.12	High	High	High	High	High	High	1	
FALSE	High	Master Prc	Q9Y263	Phospholip	0	22.138	8	4	5	4	795	87.1	6.37	12.79	4	cell commi	cytoplasm;	enzyme re	Pf00400, P	9373	ENSG0000	PLAA	9	Protein prc			1	0.938	0.045116469	2.69	2104.7	2276.9	1.94	1.64	High	High	High	High	High	High	1	
FALSE	High	Master Prc	Q9NWS0-1	PIH1 doma	0	18.007	20	3	3	3	290	32.3	5.14	13.26	3	cell differe	cytoplasm;	DNA bindi	Pf08190	55011	ENSG0000	PIH1D1	19				0	0.938	0.05510743	2.03	506.9	547.7	4.23	5.93	High	High	High	High	High	High	1	
FALSE	High	Master Prc	Q15070-1	Mitochonc	0	13.186	7	3	4	3	435	48.5	9.45	8.98	3	cell organi	membrane	protein bir	Pf02096	5018		OXA1L	14	Protein ex			1	0.938	0.288426103	0.55	2658.8	2643.3	4.22	4.43	High	High	High	High	High	High	1	
FALSE	High	Master Prc	P24534	Elongation	0	78.699	53	11	36	10	225	24.7	4.67	96.49	11	metabolic	cytoplasm;	protein bir	Pf00736, P	1933	ENSG0000	EEF1B2	2; CHR_HS		Translatior	Eukaryotic	4	0.938	0.293767658	11.47	18846.3	18566.2	9.97	6.18	High	High	High	High	High	High	1	
FALSE	High	Master Prc	Q9NUQ9	protein FA	0	23.79	22	5	8	5	324	36.7	6.06	22.85	5	regulation	extracellul	protein bir	Pf07159	51571	ENSG0000	FAM49B	8			Platelet de	4	0.938	0.653231965	7	1143.1	1202.6	10.11	1.19	High	High	High	High	High	High	1	
FALSE	High	Master Prc	Q7L5N1	COP9 signa	0	13.802	20	5	5	5	327	36.1	5.73	10.2	5	cell organi	cytoplasm;	protein bir	Pf01398, P	10980	ENSG0000	COPS6	7		Nucleotide	Cargo reco	14	0.938	0.792822798	41.76	355.1	410.6	39.35	12.92	High	High	High	High	High	High	1	
FALSE	High	Master Prc	P61221	ATP-bindir	0	44.931	22	11	20	11	599	67.3	8.34	40.68	11	cell organi	cytoplasm;	catalytic a	Pf00005, P	6059	ENSG0000	ABCE1	4			Interferon	4	0.938	0.929049933	23.59	6021.8	6421.9	17.88	5.77	High	High	High	High	High	High	1	
FALSE	High	Master Prc	P34932	Heat shock	0	314.867	60	42	114	38	840	94.3	5.19	304.41	42	cell organi	cytoplasm;	nucleotide	Pf00012, P	3308	ENSG0000	HSPA4	5	Antigen pr	Parkin-Ubi	Regulation	6	0.937	0.059655758	1	31217.3	32228.1	3.76	2.84	High	High	High	High	High	High	1	
FALSE	High	Master Prc	Q15366-3	Isoform 3 c	0	89.122	51	14	68	8	362	38.2	6.79	143.04	14	defense re	cytoplasm;	DNA bindi	Pf00013, P	5094	ENSG0000	PCBP2	12		mRNA Pro		1	0.937	0.062691436	2.87	6533.4	6989.8	2.46	0.58	High	High	High	High	High	High	1	[Q108]
FALSE	High	Master Prc	Q5VW32	BRO1 dom	0	32.384	14	3	5	3	411	46.4	7.65	22.15	3		membrane	protein bir	Pf03097	148362	ENSG0000	BROX	1				0	0.937	0.178593693	1.05	868.5	887.2	3.31	2.85	High	High	High	High	High	High	1	
FALSE	High	Master Prc	Q9H0W8	protein SM	0.002	3.857	3	1	1	1	520	57.6	7.01	3.81	1	metabolic	cytosol	protein bir		56006	ENSG0000	SMG9	19			Nonsense	3	0.937	0.275087275	6.93	351.8	375.5	4.71	9.07	High	High	High	High	High	High	1	
FALSE	High	Master Prc	Q13885	Tubulin be	0	223.694	54	22	151	2	445	49.9	4.89	361.92	22	cell organi	cytoplasm;	catalytic a	Pf00091, P	7280	ENSG0000	TUBB2A	6	Gap juncti	Pathogeni	COPI-indep	65	0.937	0.277161949	5.77	577.5	630	8.64	8.07	High	High	High	High	High	High	1	[R241]
FALSE	High	Master Prc	Q99570	Phosphoin	0	13.327	4	4	4	4	1358	153	7.17	12.08	4	cell commi	cytoskelet	catalytic a	Pf00069, P	30849	ENSG0000	PIK3R4	3	Autophagy	Focal Adhe	PI3K Casca	35	0.937	0.509329099	25.2	480	512.6	20.6	11.05	High	High	High	High	High	High	1	
FALSE	High	Master Prc	Q9H078-2	Isoform 2 c	0	83.46	25	12	19	12	677	75.4	9.01	70.41	12	response t	mitochond	catalytic a	Pf00004, P	81570	ENSG0000	CLPB	11				0	0.937	0.619221923	6.55	6594.7	7164.2	9.74	1.15	High	High	High	High	High	High	1	
FALSE	High	Master Prc	P47712	Cytosolic p	0	15.785	7	4	4	4	749	85.2	5.38	5.94	4	metabolic	cytoplasm;	catalytic a	Pf00168, P	5321	ENSG0000	PLA2G4A	1	Fc epsilon	AGE/RAGE	COPI-indep	60	0.937	0.648480577	4.2	957.1	1021.7	6.89	2.09	High	High	High	High	High	High	1	
FALSE	High	Master Prc	Q5VT66-1	Mitochonc	0	14.41	15	4	4	4	337	37.5	8.88	11.79	4	metabolic	membrane	catalytic a	Pf03473, P	64757	ENSG0000	MARC1	1			Phase I - Fu	3	0.936	0.101707239	5.09	854.9	938.3	5.78	5.19	High	High	High	High	High	High	1	
FALSE	High	Master Prc	P15880	40S riboso	0	125.289	47	13	69	13	293	31.3	10.24	171.26	13	cell organi	cytosol;me	protein bir	Pf00333, P	6187	ENSG0000	RPS2	16	Ribosome	Cytoplasm	Peptide ch	44	0.936	0.294389863	11.38	29440.1	31452.4	4.86	3.86	High	High	High	High	High	High	1	
FALSE	High	Master Prc	Q3ZAQ7	Vacuolar A	0	5.277	12	1	7	1	101	11.3	7.24	14.5	1	cell organi	endoplasm		Pf09446	203547	ENSG0000	VMA21	X				0	0.936	0.391822155	14.14	1140	1217.9	19.03	8.04	High	High	High	High	High	High	1	
FALSE	High	Master Prc	Q14432	cGMP-inhi	0	14.285	5	3	4	3	1141	124.9	6	14.15	3	metabolic	cytosol;me	catalytic a	Pf00233	5139	ENSG0000	PDE3A	12	Renin secr		G alpha (s)	13	0.936	0.549449169	28.66	255.3	274.4	23.57	14.77	High	High	High	High	High	High	1	
FALSE	High	Master Prc	Q96DG6	Carboxyme	0	25.124	24	6	9	6	245	28	7.18	25.98	6	metabolic	cytoplasm;	catalytic a	Pf00326, P	134147	ENSG0000	CMBL	5	Metabolic		Phase I - Fu	4	0.936	0.650435409	2.09	3730.3	3907.5	6.03	2.59	High	High	High	High	High	High	1	
FALSE	High	Master Prc	P00492	Hypoxanth	0	22.153	25	5	12	5	218	24.6	6.68	13.25	5	cell differe	cytoplasm;	catalytic a	Pf00156, P	3251	ENSG0000	HPRT1	X	Drug meta	Mesoderm	Purine salv	9	0.936	0.754961288	0.5	234.8	245.1	9.01	9.34	High	High	High	High	High	High	1	
FALSE	High	Master Prc	O95793	double-stra	0	7.589	4	2	2	2	577	63.1	9.44	4.81	2	regulation	cytoplasm;	protein bir	Pf00035, P	6780	ENSG0000	STAU1	20				0	0.936	0.895167168	13.79	632.2	669.8	9.43	8.12	High	High	High	High	High	High	1	
FALSE	High	Master Prc	Q96RT1	Erbin [OS=	0	8.863	3	3	3	3	1412	158.2	5.5	5.15	3	cell growth	cytoplasm;	protein bir	Pf00595, P	55914	ENSG0000	ERBB2IP; E	5	NOD-like r	Nucleotide	Downregu	8	0.936	0.93129621	19.13	241.4	236.2	19.69	13.61	High	High	High	High	High	High	1	
FALSE	High	Master Prc	P67775	Serine/thre	0	55.715	37	9	12	7	309	35.6	5.54	40.03	9	cell death;	chromosor	catalytic a	Pf00149	5515	ENSG0000	PPP2CA	5	Tight junct	TNF alpha	Glycolysis;	145	0.935	0.150727819	0.34	2819.3	3016.2	1.98	5.43	High	High	High	High	High	High	1	
FALSE	High	Master Prc	Q92841-1	Isoform 2 c	0	176.37	39	26	69	17	650	72.3	8.59	148.9	26	cell differe	cytoplasm;	catalytic a	Pf00270, P	10521		DDX17	22				0	0.935	0.174172921	1.47	7410.2	7870.5	3.99	1.37	High	High	High	High	High	High	1	
FALSE	High	Master Prc	P11802	Cyclin-dep	0	13.611	18	4	7	4	303	33.7	7.01	13.77	4	cell divisio	chromosor	catalytic a	Pf00069, P	1019	ENSG0000	CDK4	12	Glioma; No	miRNA Reg	Transcripti	67	0.935	0.202118487	6.83	967.2	1027.9	3.44	2.85	High	High	High	High	High	High	1	
FALSE	Low	Master Prc	P31274	homeobox	0.054	1.157	7	1	1	1	260	29.2	9.04	0	1	developme	nucleus	DNA bindi	Pf00046, P	3225	ENSG0000	HOXC9	12		Differentia		1	0.935	0.206917297	1.08	59.6	66.7	9.73	4.77	High	High	High	High	High	High	1	[Q155]
FALSE	High	Master Prc	P30419	glycylpepti	0	59.637	24	9	18	9	496	56.8	7.8	40.8	9	metabolic	cytoplasm;	catalytic a	Pf01233, P	4836	ENSG0000	NMT1	17			Signaling b	14	0.935	0.240690615	9.79	2922.6	3153.2	6.66	0.68	High	High	High	High	High	High	1	
FALSE	High	Master Prc	P49588	Alanine--tR	0	186.856	38	31	63	31	968	106.7	5.53	154.04	31	metabolic	cytoplasm;	catalytic a	Pf01411, P	16	ENSG0000	AARS	16	Aminoacyl		Cytosolic t	5	0.935	0.303157072	6.8	23104.2	24436.6	3.35	4.49	High	High	High	High	High	High	1	
FALSE	High	Master Prc	Q86UU1-1	pleckstrin	0	7.885	2	3	3	3	1377	151.1	8.63	3.79	3	metabolic		protein bir	Pf00169, P	23187	ENSG0000	PHLDB1	11				0	0.935	0.319168413	11.42	660.1	706	5.64	7.15	High	High	High	High	High	High	1	
FALSE	High	Master Prc	O15427	Monocarb	0	22.185	10	4	8	4	465	49.4	7.96	12.24	4	cellular co	membrane	protein bir	Pf07690	9123	ENSG0000	SLC16A3	17	Central car		Proton-cou	12	0.935	0.3246555	11.92	2129	2183.6	6.82	4.41	High	High	High	High	High	High	1	
FALSE	High	Master Prc	P12956	X-ray repai	0	156.544	41	27	70	27	609	69.8	6.64	141.64	27	cell organi	chromosor	catalytic a	Pf02037, P	2547	ENSG0000	XRCC6	22	Non-homo	Non-homo	2-LTR circl	18	0.935	0.397638165	7.15	23621.1	23875.8	6.23	4.83	High	High	High	High	High	High	1	
FALSE	High	Master Prc	P55263-1	Adenosine	0	37.62	28	7	10	7	362	40.5	6.7	33.09	7	metabolic	cytoplasm;	catalytic a	Pf00294	132	ENSG0000	ADK	10	Purine met		Purine salv	6	0.935	0.513577326	19.01	2865.8	3183.3	11.95	4.15	High	High	High	High	High	High	1	
FALSE	High	Master Prc	Q86YR5-1	G-protein-	0	7.3	6	2	2	2	675	74.5	6.54	7	2	cell differe	cytoplasm;	enzyme re	Pf00515, P	26086		GPSM1	9	Cocaine ac		G alpha (i)	5	0.935	0.542269665	18.99	466.7	474.4	5.42	11.64	High	High	High	High	High	High	1	

FALSE	High	Master Prc	Q00341-1	Vigilin [OS	0	121.959	28	29	47	29	1268	141.4	6.87	93.17	29	metabolic	cytoplasm;	protein bir	Pf00013, P	3069		HDLBP	2			HDL cleara	4	0.935		0.567304003	1.18	9054	9518.6	6.73	1.57	High	High	High	High	High	High	1	
FALSE	Low	Master Prc	Q96S66	chloride ch	0.063	1.098	2	1	1	1	551	62	5.55	1.76	1	transport	endoplasm	transporte	Pf05934	23155	ENSG0000	CLCC1	1				0	0.935		0.616962064	11.98	591.5	637.5	9.5	4.38	High	High	High	High	High	High	1	
FALSE	High	Master Prc	Q9P031	Thyroid tra	0.003	3.386	5	1	1	1	241	28.7	9.88	3.11	1	metabolic	nucleus	protein bir		29080	ENSG0000	CCDC59	12			Surfactant	6	0.935		0.745914238	0.88	681.6	722.7	13.39	5.48	High	High	High	High	High	High	1	
FALSE	High	Master Prc	P49591	Serine--tRI	0	34.868	22	10	11	10	514	58.7	6.43	20.37	10	metabolic	cytoplasm;	catalytic a	Pf00587, P	6301	ENSG0000	SARS	1	Aminoacyl		Selenocyst	9	0.935		0.810219399	17.08	5139.1	5273.4	12.52	9.27	High	High	High	High	High	High	1	
FALSE	High	Master Prc	Q08J23	tRNA (Cyto	0	180.057	43	29	54	29	767	86.4	6.77	130.1	29	cell divisio	cytoplasm;	catalytic a	Pf01189, P	54888	ENSG0000	NSUN2	5			tRNA mod	3	0.935		0.864552847	2.34	15231.9	15615.7	8.27	3.59	High	High	High	High	High	High	1	
FALSE	High	Master Prc	Q9H501	ESF1 homc	0	15.426	6	5	5	5	851	98.7	5.11	6.28	5	metabolic	nucleus	RNA bindi	Pf08159	51575	ENSG0000	ESF1	20				0	0.935		0.904826744	38.75	56.4	62.9	30.63	13.04	High	High	High	High	High	High	1	
FALSE	High	Master Prc	Q96HC4	PDZ and LI	0	30.364	17	6	8	6	596	63.9	8.21	27.51	6	cell growth	cytoplasm;	metal ion I	Pf00412, P	10611	ENSG0000	PDLIM5	4			Neurexins	3	0.935		0.944477743	19.49	1231.6	1235.8	15.28	13.84	High	High	High	High	High	High	1	
FALSE	High	Master Prc	P04075	fructose-bi	0	334.226	85	31	217	25	364	39.4	8.09	634.7	31	cell organi:	cytoplasm;	catalytic a	Pf00274	226	ENSG0000	ALDOA	16	Glycolysis ,	Glycolysis .	Platelet de	21	0.934		0.129094909	0.59	56668	62412.6	6.2	2.48	High	High	High	High	High	High	1	
FALSE	High	Master Prc	P05067-1	Amyloid-be	0	12.627	4	3	3	3	770	86.9	4.82	2.48	3	cell comm	cell surface	DNA bindi	Pf00014, P	351	ENSG0000	APP	21	Serotonerg	Alzheimers	Post-transl	69	0.934		0.129676982	6.64	337.5	382.6	10.49	6.84	High	High	High	High	High	High	1	
FALSE	High	Master Prc	Q16706	Alpha-mar	0	12.276	2	2	4	2	1144	131.1	7.58	8.93	2	cell organi:	Golgi;mem	catalytic a	Pf01074, P	4124	ENSG0000	MAN2A1	5	N-Glycan b		Intra-Golgi	12	0.934		0.394798578	14.32	1213.2	1425.2	16.57	8.9	High	High	High	High	High	High	1	
FALSE	High	Master Prc	Q93062-1	RNA-bindi	0	8.718	18	2	2	2	196	21.8	8.07	6.96	2	metabolic	cytoplasm;	protein bir	Pf00076, P	11030	ENSG0000	RBPMS	8		Exercise-in		1	0.934		0.45744637	0.17	138.7	148.5	6.89	1.02	High	High	High	High	High	High	1	
FALSE	High	Master Prc	Q9UHA4	ragulator c	0	9.5	23	1	1	1	124	13.6	7.34	6.31	1	metabolic	endosome	enzyme re	Pf08923	8649	ENSG0000	LAMTOR3	4	MAPK sign	MAPK Sigr	Energy dep	28	0.934		0.520488906	23.27	306	327.6	11.11	10.1	High	High	High	High	High	High	1	
FALSE	High	Master Prc	Q13309-1	S-phase kir	0	10.294	11	2	2	2	424	47.7	7.11	7.91	2	cell comm	cytoplasm;	catalytic a	Pf00646, P	6502	ENSG0000	SKP2	5	Small cell l	Cell Cycle;	Regulation	39	0.934		0.665259911	14.21	109.2	116.9	2.67	11.48	High	High	High	High	High	High	1	
FALSE	High	Master Prc	Q9Y6M5	zinc transp	0	11.994	9	4	5	4	507	55.3	6.48	8.54	4	cellular ho	cytoplasm;	protein bir	Pf01545	7779	ENSG0000	SLC30A1	1	Mineral ab	Zinc home	Zinc efflux	8	0.934		0.861914201	7.78	427	457	15.51	4.2	High	High	High	High	High	High	1	
FALSE	High	Master Prc	Q15286	Ras-related	0	24.236	22	5	13	4	201	23	8.29	28.26	5	cell organi:	cytosol;en	catalytic a	Pf00006, P	11021	ENSG0000	RAB35	12	Endocytosi		TBC/RABG	9	0.933		0.114564949	2.47	4087	4454.5	5.76	2.54	High	High	High	High	High	High	1	
FALSE	High	Master Prc	O75879	Glutamyl-t	0	16.366	7	2	2	2	557	61.8	8.63	6.24	2	cell organi:	mitochond	catalytic a	Pf02637, P	5188	ENSG0000	PET112; G.	4	Metabolic			2	0.933		0.28264506	6.41	70.7	75.8	8.92	2.36	High	High	High	High	High	High	1	
FALSE	High	Master Prc	Q8NAV1-1	Pre-mRNA	0	8.172	7	2	2	2	312	37.5	9.96	2.69	2	cell organi:	membrane	protein bir	Pf03371	84950	ENSG0000	PRPF38A	1	Spliceosom	Mecp2 and	mRNA Spli	6	0.933		0.305469563	11.79	1150	1247.2	4.74	6.35	High	High	High	High	High	High	1	
FALSE	High	Master Prc	O95159	Zinc finger	0.001	4.249	3	1	1	1	310	34.1	8.07	3.17	1	regulation	Golgi;mem	DNA bindi		7542	ENSG0000	ZFPL1	11				0	0.933		0.35198118	12.5	504.8	540.9	26.87	5.39	High	High	High	High	High	High	1	
FALSE	High	Master Prc	P50454	Serpin H1		0	86.937	38	11	29	11	418	46.4	8.69	76.13	11	cell organi:	cytoplasm;	enzyme re	Pf00079	871	ENSG0000	SERPINH1	11		Endochonc	Collagen b	4	0.933		0.376110204	11.29	6833.6	7286.9	4.56	6.04	High	High	High	High	High	High	1	
FALSE	High	Master Prc	Q9BQE4	selenoprot	0	12.426	20	2	2	2	189	21.2	9.7	2.09	2	cellular ho	cytoplasm;	antioxidan	Pf06936	55829	ENSG0000	VIMP; SELE	15	Protein pro	Selenium M	E3 ubiquiti	7	0.933		0.407190928	4.47	1277.2	1368.9	10	2.89	High	High	High	High	High	High	1	
FALSE	High	Master Prc	Q9H875	PRKR-inter	0.003	3.302	5	1	1	1	184	21	9.79	2.53	1	metabolic	nucleus	enzyme re	Pf06658	79706	ENSG0000	PRKRIP1	7				0	0.933		0.47467105	0.27	606.1	598.9	6.71	8.89	High	High	High	High	High	High	1	
FALSE	High	Master Prc	P46199	Translation	0	5.314	2	1	1	1	727	81.3	7.15	2.3	1	cell organi:	mitochond	catalytic a	Pf00009, P	4528	ENSG0000	MTIF2	2			Mitochonc	4	0.933		0.620597851	1.95	564.8	566.3	4.36	6.92	High	High	High	High	High	High	1	
FALSE	High	Master Prc	Q5H9R7-1	Serine/thre	0	13.015	5	4	4	4	873	97.6	4.6	6.13	4	cell organi:	cytoplasm;	protein bir	Pf04499	55291	ENSG0000	PPP6R3	11			COPII (Coa	8	0.933		0.708304368	0	74	75.9	13.25	7.7	High	High	High	High	High	High	1	
FALSE	High	Master Prc	P08670	Vimentin [	0	330.729	82	46	210	42	466	53.6	5.12	497.27	46	cell differe	cytoplasm;	protein bir	Pf00038, P	7431	ENSG0000	VIM	10	MicroRNA:	Allograft R	Interleukin	20	0.933		0.746657982	17.57	52803.7	56611.2	10.69	5.99	High	High	High	High	High	High	1	[Q274;
FALSE	High	Master Prc	Q69YN2-1	CWF19-like	0	18.798	11	4	4	4	538	60.6	7.24	9.95	4			catalytic a	Pf04676, P	55280	ENSG0000	CWF19L1	10				0	0.933		0.760093238	10.97	617.8	662.2	21.67	4.75	High	High	High	High	High	High	1	
FALSE	High	Master Prc	Q15018	BRISC com	0	15.96	10	3	3	3	415	46.9	6.21	9.24	3	cell divisio	cytoplasm;	protein bir	Pf00261	23172	ENSG0000	FAM175B;	10			Metallopro	4	0.933		0.935830131	50.97	596.4	639.3	30.59	16.48	High	High	High	High	High	High	1	
FALSE	High	Master Prc	P62314	Small nucle	0	8.722	17	1	2	1	119	13.3	11.56	8.09	1	cell organi:	cytoplasm;	protein bir	Pf01423	6632	ENSG0000	SNRPD1	18	Spliceosom	mRNA Pro	snRNP Ass	10	0.932		0.051177321	0.66	262.1	271.8	2.45	3.18	High	High	High	High	High	High	1	
FALSE	High	Master Prc	O60701	UDP-gluco	0	136.99	52	20	44	20	494	55	7.12	129.19	20	metabolic	cytosol;nu	catalytic a	Pf00984, P	7358	ENSG0000	UGDH	4	Ascorbate	Glucuronic	Formation	10	0.932		0.055096237	3.07	12779.4	13503.6	1.01	3.29	High	High	High	High	High	High	1	
FALSE	High	Master Prc	P61513	60S riboso	0	30.007	47	5	20	5	92	10.3	10.43	49.16	5	cell organi:	cytosol;nu	metal ion I	Pf01780	6168	ENSG0000	RPL37A	2	Ribosome	Cytoplasm	Peptide ch	34	0.932		0.058820106	1.88	12059.4	13232	2.63	2.09	High	High	High	High	High	High	1	
FALSE	High	Master Prc	P17987	T-complex	0	175.394	53	23	55	23	556	60.3	6.11	147.6	23	cell organi:	cytoplasm;	nucleotide	Pf00118	6950	ENSG0000	TCP1	6			BBSome-m	18	0.932		0.090051449	2.39	13456.7	14898.8	3.99	2.79	High	High	High	High	High	High	1	
FALSE	High	Master Prc	P38606	V-type pro	0	101.527	40	17	28	17	617	68.3	5.52	90.76	17	cellular ho	cytosol;me	catalytic a	Pf00006, P	523	ENSG0000	ATP6V1A	3	Synaptic ve		Transferrin	20	0.932		0.111963138	3.56	7215.7	7852.9	3.99	2.35	High	High	High	High	High	High	1	
FALSE	High	Master Prc	Q99471-1	prefoldin s	0	38.526	59	9	14	9	154	17.3	6.33	31.91	9	metabolic	cytoplasm;	protein bir	Pf02996	5204	ENSG0000	PFDN5	12			Prefoldin r	5	0.932		0.172829998	3.62	3151.2	3379.8	2.67	6.28	High	High	High	High	High	High	1	
FALSE	High	Master Prc	Q9H832	Ubiquitin-c	0	20.205	8	3	9	3	354	38.2	5.62	20.22	3	cell death;	cytoplasm;	catalytic a	Pf00179, P	65264	ENSG0000	UBE2Z	17	Ubiquitin r		Synthesis c	9	0.932		0.427434266	18.43	815.4	899.9	25.33	11.46	High	High	High	High	High	High	1	
FALSE	High	Master Prc	Q9UH65	Switch-ass	0	23.767	12	7	10	7	585	69	5.87	13.49	7	cell organi:	cytoplasm;	DNA bindi	Pf00169, P	23075	ENSG0000	SWAP70	11				0	0.932		0.432807477	9.66	3355.6	3600.1	1.45	7.9	High	High	High	High	High	High	1	
FALSE	High	Master Prc	Q9NWU2	Glucose-in	0	9.801	10	1	2	1	228	26.7	4.97	9.25	1		nucleus	protein bir	Pf08513, P	54994	ENSG0000	GID8	20				0	0.932		0.568341408	46.84	349.7	451.5	34.05	31.81	High	High	High	High	High	High	1	
FALSE	High	Master Prc	Q9Y383	Putative Rl	0	49.479	20	8	24	5	392	46.5	10.01	67.29	8	cell organi:	nucleus;sp	protein bir	Pf03194	51631	ENSG0000	LUC7L2	7				0	0.932		0.687698415	4.97	3730.3	4053.2	13.01	3.11	High	High	High	High	High	High	1	
FALSE	High	Master Prc	O75165	DnaJ homc	0	8.117	1	2	2	2	2243	254.3	6.74	4.87	2	cell differe	cytosol;en	protein bir	Pf00226, P	23317	ENSG0000	DNAJC13	3			Neutrophil	3	0.932		0.894097057	18.28	200.2	214.9	17.39	9.9	High	High	High	High	High	High	1	
FALSE	High	Master Prc	Q5T4S7-1	e3 ubiquiti	0	108.872	6	20	24	20	5183	573.5	6.04	58.7	20	metabolic	cytoplasm;	catalytic a	Pf02207, P	23352	ENSG0000	UBR4	1	Viral carcir		Neutrophil	7	0.932		0.92637052	10.81	5846.2	6572	17.17	2.73	High	High	High	High	High	High	1	
FALSE	High	Master Prc	P17655-1	Calpain-2 c	0	91.127	29	15	24	15	700	79.9	4.98	59.41	15	cell organi:	chromosor	catalytic a	Pf00648, P	824	ENSG0000	CAPN2	1	Focal adhe	Integrin-m	Degradatic	12	0.931		0.072764542	3.29	5340.1	6039.7	3.25	4.24	High	High	High	High	High	High	1	
FALSE	High	Master Prc	O60506-3	Isoform 3 c	0	187.258	44	25	74	20	562	62.6	7.56	191.45	25	cell differe	endoplasm	protein bir	Pf00076, P	10492	ENSG0000	SYNCRIP	6				0	0.931		0.097297986	5.03	15756.8	16605.1	3.86	7.49	High	High	High	High	High	High	1	
FALSE	High	Master Prc	P62277	40S riboso	0	52.218	46	9	28	9	151	17.2	10.54	48.7	9	cell organi:	cytosol;me	protein bir	Pf00312, P	6207	ENSG0000	RPS13	11	Ribosome	Cytoplasm	Peptide ch	38	0.931		0.133023429	1.49	9723.1	10446.6	2.86	4.87	High	High	High	High	High	High	1	
FALSE	High	Master Prc	Q8NCW5	NAD(P)H-h	0	32.031	26	7	10	7	288	31.7	7.66	23.32	7	cell organi:	cytosol;ext	catalytic a	Pf03853	128240	ENSG0000	APOA1BP;	1			Nicotinami	5	0.931		0.345666642	10.56	2549.7	2752	17.51	10.05	High	High	High	High	High	High	1	
FALSE	High	Master Prc	Q96NB3	zinc finger	0.003	3.49	5	2	2	2	372	42	5.31	0	2	cell divisio	chromosor	metal ion I	Pf12171, P	91603	ENSG0000	ZNF830	17			Dual incisi	6	0.931		0.388052651	14.01	217.3	255.5	18.71	10.55	High	High	High	High	High	High	1	
FALSE	High	Master Prc	Q13200	26S protea	0	154.656	37	27	47	27	908	100.1	5.2	131.14	27	metabolic	cytoplasm;	catalytic a	Pf01851	5708	ENSG0000	PSMD2	3	Epstein-Ba	Proteasom	Regulation	154	0.931		0.480840019	7.09	10905	11409.5	8.23	1.78	High	High	High	High	High	High	1	
FALSE	High	Master Prc	P19075	Tetraspani	0	6.747	10	2	2	2	237	26	5.6	5.32	2	regulation	cell surface	protein bir	Pf00335	7103	ENSG0000	TSPAN8	12		Extracellul		1	0.931		0.518832111	26.3	323.8	355.9	13.24	13.9	High	High	High	High	High	High	1	
FALSE	High	Master Prc	Q9NTJ3-1	Structural	0	28.04	7	6	6	6	1288	147.1	6.79	16.47	6	cell divisio	chromosor	nucleotide	Pf02463, P	10051	ENSG0000	SMC4	3			Condensat	7	0.931		0.692894356	33.92	729.2	783.5	40.12	12.82	High	High	High	High	High	High	1	
FALSE	High	Master Prc	O75506	Heat shock	0	6.111	25	1	1	1	76	8.5	4.36	6.14	1	cell differe	cytoskelet	protein bir	Pf06825	3281	ENSG0000	HSBP1	16			Attenuatio	6	0.931		0.792636146	66.59	101.7	115.5	33.33	34.11	High	High	High	High	High	High	1	
FALSE	High	Master Prc	P15121	aldose red	0	143.329	59	16	69	16	316	35.8	6.98	178.02	16	metabolic	cytoplasm;	catalytic a	Pf00248	231	ENSG0000	AKR1B1	7	Galactose	Polyol Patl	Pregnenol	15	0.93		0.029477066	0.71	23200.7	24740.3	0.96	1.83	High	High	High	High	High	High	1	
FALSE	High	Master Prc	P31947-1	14-3-3 pro	0	81.268	49	12	35	7	248	27.8	4.74	59.43	12	cell differe	cytoplasm;	enzyme re	Pf00244	2810	ENSG0000	SFN	1	Cell cycle;	miRNA Reg	Translocat	31	0.93		0.101948996	2.63	2121.7	2322.8	8.53	3.56	High	High	High	High	High	High	1	
FALSE	High	Master Prc	Q8N1G4	Leucine-ric	0	36.361	23	8	10	8	583	63.4	8.28	17.81	8	metabolic		catalytic a	Pf03483, P	57470	ENSG0000	LRRC47	1				0	0.93		0.110285853	0.3	1861.6	2084	7.08	2.6	High	High	High	High	High	High	1	
FALSE	High	Master Prc	O00487	26S protea	0	59.968	38	7	16	7	310	34.6	6.52	51.9	7	metabolic	cytoplasm;	catalytic a	Pf01398, P	10213	ENSG0000	PSMD14	2	Proteasom	Parkin-Ubi	Regulation	153	0.93		0.115839955	6.32	5057.4	5483.7	0.99	4.24	High	High	High	High	High	High	1	
FALSE	High	Master Prc	Q14690	Protein RR	0	30.92	4	7	9	7	1871	208.6	8.87	22.79	7	metabolic	cytosol;nu	protein bir	Pf00575, P	22984	ENSG0000	PDCD11	10			Major path	5	0.93		0.159167525	2.07	1620.7	1743.4	3.34	1.12	High	High	High	High	High	High	1	
FALSE	High	Master Prc	Q86W42	THO comp	0	18.449	13	3	4	3	341	37.5	7.43	14.48	3	cell death;	nucleus	protein bir	Pf00400	79228	ENSG0000	THOC6	16	RNA transp		Cleavage o	10	0.93		0.168484009	1.09	793.1	852	1.21	3.01	High	High	High	High	High	High	1	
FALSE	High	Master Prc	P08708	40S riboso	0	72.444	62	7	31	7	135	15.5	9.85	106.09	7	cell organi:	cytosol;me	RNA bindi	Pf00833	6218	ENSG0000	RPS17; RP:	15; CHR_H	Ribosome	Cytoplasm	Peptide ch	38	0.93		0.205717079	5.53	10330.4	10693.3	17.94	6.38	High	High	High	High	High	High	1	
FALSE	High	Master Prc	Q8N766-1	ER membra	0	25.428	8	5	6	5	993	111.7	7.66	18.47	5	metabolic	endoplasm		Pf07774, P	23065	ENSG0000	EMC1	1				0	0.93		0.331356606	8.43	1195.5	1222.3	9.68	29.63	High	High	High	High	High	High	1	
FALSE	High	Master Prc	Q12906-1	Interleukin	0	157.31	34	25	56	25	894	95.3	8.76	152.22	25	defense re	cytoplasm;	DNA bindi	Pf00035, P	3609	ENSG0000	ILF3	19				0	0.93		0.388349496	15.87	18261.7	19627.3	8.61	2.47	High	High	High	High	High	High	1	
FALSE	High	Master Prc	P41208	centrin-2 [	0	8.627	8	1	2	1	172	19.7	5	4.35	1	cell divisio	cytoplasm;	metal ion I	Pf00036, P	1069	ENSG0000	CETN2	X	Nucleotide		Regulation	27	0.93		0.562268206	32.06	435.8	480.5	11.2	20.11	High	High	High	High	High	High	1	
FALSE	High	Master Prc	Q8WX93-1	palladin [C	0	82.671	18	19	29	19	1383	150.5	7.09	61.64	19	cell organi:	cytoplasm;	protein bir	Pf00047, P	23022		PALLD	4				0	0.93		0.716038567	0.3	4709.1	4924	4.66	4.82	High	High	High	High	High	High	1	
FALSE	High	Master Prc	P61764	Syntaxin-b	0	18.435	5	2	3	2	594	67.5	6.96	11.52	2	cell comm	cytoplasm;	protein bir	Pf00995	6812	ENSG0000	STXBP1	9	Synaptic ve	Synaptic V	Glutamate	18	0.93		0.751498046	41.91	403.1	397.1	22.49	16.98	High	High	High	High	High	High	1	
FALSE	High	Master Prc	Q5RI15-1	Cytochrom	0	5.805	10	1	2	1	118	13.3	8.76	5.73	1	cell organi:	membrane		Pf12597	116228	ENSG0000	COX20	1			TP53 Regu	9	0.93		0.761044433	6.99	527.7	566.3	17.97	6.23	High	High	High	High	High	High	1	
FALSE	High	Master Prc	O96000	NADH deh	0	24.705	27	4	7	4	172	20.8	8.48	20.5	4	cell organi:	membrane	catalytic a	Pf10249	4716	ENSG0000	NDUFB10	16	Metabolic	Electron Tr	Complex I	13	0.93		0.76394123	0.33	1170.4	1149.9	9.98	11.67	High	High	High	High	High	High	1	
FALSE	High	Master Prc	Q9BUL8	programm	0	5.141	6	1	1	1	212	24.7	8.19	4.54	1	cell death;	cytoplasm;	protein bir	Pf06840	11235	ENSG0000	PDCD10	3				0	0.93		0.788150804	1.8	458.5	451.1	14.02	10.7	High	High	High	High	High	High	1	
FALSE	High	Master Prc	Q6ZVX7	F-box only	0	18.349	20	3	4	3	275	30.8	6.62	16.79	3	regulation	cytoplasm		Pf04300	342897	ENSG0000	NCCRP1	19				0	0.93		0.80564369	9.01	1000.2	1075	9.37	5.18	High	High	High	High	High	High	1	
FALSE	High	Master Prc	O00571	ATP-depen	0	95.913	36	24	38	24	662	73.2	7.18	71.54	24	cell death;	cytoplasm;	catalytic a	Pf00270, P	1654	ENSG0000	DDX3X	X	Hepatitis B	RIG-I-like F	Neutrophil	7	0.93		0.826067534	7.15	7390.9	7948.4	5.87	7.87	High	High	High	High	High	High	1	
FALSE	High	Master Prc	Q9Y262	eukaryotic	0	59.925	26	14	26	14	564	66.7	6.34	62.83	14	metabolic	cytoplasm;	protein bir	Pf10255	51386	ENSG0000	EIF3L	22			Formation	11	0.929		0.149773281	2.75	6713.1	7165.5	1.34	3.29	High	High	High	High	High	High	1	
FALSE	High	Master Prc	P00568	Adenylate	0	72.077	56	9	25	9	194	21.6	8.63	50.39	9	metabolic	cytoplasm;	catalytic a	Pf00406, P	203	ENSG0000	AK1	9	Purine met		Interconve	6	0.929		0.197024754	9.06	5809.2	6253.6	4.3	3.05	High	High	High	High	High	High	1	
FALSE	High	Master Prc	P12277	Creatine ki	0	53.363	20	5	11	5	381	42.6	5.59	42.83	5	cellular ho	cytoplasm;	catalytic a	Pf00217, P	1152	ENSG0000	CKB	14	Arginine ar	Urea cycle	Creatine m	7	0.929		0.221494382	1.9	2288.9	2604.2	12.16	5.55	High	High	High	High	High	High	1	
FALSE	High	Master Prc	Q9UNN5-1	fas-associa	0	12.303	6	4	5	4	650	73.9	4.88	10.03	4	cell death;	cytoplasm;	enzyme re	Pf00789, P	11124	ENSG0000	FAF1	1		Retinoblas		3	0.929		0.667513645	2.85	1450.9	1424.1	6.36	8	High	High	High	High	High	High	1	
FALSE	High	Master Prc	Q15813	Tubulin-sp	0	20.919	12	4	4	4	527	59.3	6.76	13.41	4	cell organi:	cytoplasm;	protein bir	Pf01302, P	6905	ENSG0000	TBCE	1; CHR_HS			Post-chape	3	0.929		0.735138625	9.49	281.5	298.3	24.43	4.94	High	High	High	High	High	High	1	
FALSE	High	Master Prc	P49407-1	Beta-arrest	0	5.248	5	2	4	2	418	47	6.2	5.84	2	cell death;	chromosor	catalytic a	Pf00339, P	408	ENSG0000	ARRB1	11	Morphine	Corticotro	Cargo reco	52	0.929		0.927433556	8.78	751.2	734.2	10.37	13.75	High	High	High	High	High	High	1	
FALSE	High	Master Prc	Q6DD88	Atlastin-3	0	60.634	26	11	15	11	541	60.5	5.66	46.89	11	cell organi:	endoplasm	catalytic a	Pf02263	25923	ENSG0000	ATL3	11				0	0.928		0.196710794	0.71	2666.7	2786	3.92	6.56	High	High	High	High	High	High	1	
FALSE	High	Master Prc	P40123	Adenylyl cy	0	4.668	2	1	2	1	477	52.8	6.37	4.87	1	cell organi:	cytoskelet	protein bir	Pf01213, P	10486	ENSG0000	CAP2	6		Ectoderm		Role of AB	6	0.928		0.260019065	3.93	399.9	430.8	10.51	8.59	High	High	High	High	High	High	1	
FALSE	High	Master Prc	Q5T6V5	queuosine	0	11.319	10	3	3	3	341	39	5.88	8.09	3	metabolic			Pf10343	84267	ENSG0000	C9orf64	9				0	0.928		0.285383801	6.49	738.1	794.9	8.52	11.07	High	High	High	High	High	High	1	
FALSE	High	Master Prc	Q9BUK6	Protein mi	0	16.301	11	3	3	3	570	61.8	6.11	4.82	3	cell organi:	cytoplasm;	catalytic a	Pf00091, P	55154	ENSG0000	MSTO1	1				0	0.928		0.338986338	10.53	643.9	715.5	8.69	17.05	High	High	High	High	High	High	1	
FALSE	High	Master Prc	P55060-1	Exportin-2	0	118.768	24	18	35	18	971	110.3	5.77	97.84	18	cell death;	cytoplasm;	protein bir	Pf03378, P	1434	ENSG0000	CSE1L	20				0	0.928		0.718568604	15.36	7254.3	7819.9	10.76	5.14	High	High	High	High	High	High	1	
FALSE	High	Master Prc	Q9NVE7	pantothen	0	7.021	3	2	3	2	773	85.9	6.28	8.73	2	metabolic	cytoplasm	catalytic a	Pf01937, P	55229	ENSG0000	PANK4	1; CHR_HS	Pantothen		Coenzyme	7	0.928		0.768690197	14.15	299.8	319	24.48	7.29	High	High	High	High	High	High	1	
FALSE	High	Master Prc	Q16658	Fascin [OS	0	141.033	55	24	81	24	493	54.5	7.24	163.67	24	cell organi:	cytoplasm;	protein bir	Pf06268	6624	ENSG0000	FSCN1	7	MicroRNA:		Interleukin	5	0.927		0.103749254	1.02	23792.1	26052.6	1.67	5.11	High	High	High	High	High	High	1	[S489(99.
FALSE	High	Master Prc	P50238	Cysteine-ri	0	14.283	62	4	9	4	77	8.5	8.75	14.5	4	cell prolife	cytoplasm	DNA bindi	Pf00412	1396	ENSG0000	CRIP1	14				0	0.927		0.22061687	10.25	1480.4	1633.9	6.21	6.17	High	High	High	High	High	High	1	[R68]
FALSE	High	Master Prc	Q6P1J9	Parafibrom	0	14.6	7	4	7	4	531	60.5	9.61	8.84	4	cell organi:	cytosol;nu	protein bir	Pf05179	79577	ENSG0000	CDC73	1		Endoderm	Hedgehog	16	0.927		0.425884588	5.3	1861.8	2008.5	1.68	6.59	High	High	High	High	High	High	1	

FALSE	High	Master Prc	Q9NSD9	Phenylalar	0	18.968	10	6	8	6	589	66.1	6.84	12.75	6	cell organi:	cytoplasm;	catalytic ac	Pf03483, P	10056	ENSG0000	FARSB	2	Aminoacyl	Amino Aci	Cytosolic t	6	0.927		0.705544246	39.19	1421.5	1533	17.69	14.59	High	High	High	High	High	High	1	
FALSE	High	Master Prc	P61313-1	60S riboso	0	65.517	50	11	28	11	204	24.1	11.62	53.14	11	cell organi:	cytosol;me	protein bir	Pf00827	6138	ENSG0000	RPL15	3	Ribosome	Cytoplasm	Peptide ch	34	0.927		0.912333629	1.66	8781.3	9412	5.08	7.6	High	High	High	High	High	High	1	
FALSE	High	Master Prc	Q06787	synaptic fu	0	21.653	11	6	8	5	632	71.1	7.42	29.88	6	metabolic	chromosor	protein bir	Pf00013, P	2332	ENSG0000	FMR1	X	RNA transp	Serotonin		2	0.927		0.948756472	11.21	1993	2150.1	19.16	1.3	High	High	High	High	High	High	1	
FALSE	High	Master Prc	O00221	NF-kappa-I	0	4.794	6	1	1	1	500	52.8	6.68	4.41	1	regulation	cytoplasm;	protein bir	Pf00023, P	4794	ENSG0000	NFKBIE	6	B cell recep	TNF alpha	Activation	14	0.926		0.009700853	1.04	63.1	67.1	4.77	4.83	High	High	High	High	High	High	1	
FALSE	High	Master Prc	O00483	Cytochrom	0	10.623	46	4	9	4	81	9.4	9.38	10.01	4	metabolic	membrane	catalytic ac	Pf06522	4697	ENSG0000	NDUFA4	7	Parkinson's	Electron Tr	TP53 Regu	17	0.926		0.015876867	1.83	3277.7	3612.1	9.06	9.07	High	High	High	High	High	High	1	
FALSE	High	Master Prc	O75153	Clustered r	0	43.827	11	10	13	10	1309	146.6	6.13	32.45	10	cell organi:	cytoplasm	protein bir	Pf12807, P	23277	ENSG0000	CLUH; KIAA	17		Translation		1	0.926		0.136478175	7.08	4640.5	4956.8	1.49	4.35	High	High	High	High	High	High	1	
FALSE	High	Master Prc	P62861	40S riboso	0	10.724	31	2	8	2	59	6.6	12.15	8.42	2	cell organi:	cytosol;rib	RNA bindi	Pf04758	2197		FAU	11		Cytoplasm	Peptide ch	37	0.926		0.170557844	8.55	846.9	915.7	3.77	3.89	High	High	High	High	High	High	1	
FALSE	High	Master Prc	O95140	Mitofusin-	0	6.975	3	2	3	2	757	86.3	6.98	3.16	2	cell commu	cytoskeleto	catalytic ac	Pf00350, P	9927	ENSG0000	MFN2	1	NOD-like r		Factors inv	5	0.926		0.263589698	8.17	435	485.6	14.16	5.32	High	High	High	High	High	High	1	
FALSE	High	Master Prc	Q96KP1	exocyst co	0	6.945	4	2	3	2	924	104	6.9	7.11	2	cell organi:	cytosol;me	protein bir	Pf01833, P	55770	ENSG0000	EXOC2	6	Ras signali	RalA down	Translocat	12	0.926		0.517300965	18.32	315	340.3	4.27	9.93	High	High	High	High	High	High	1	[Q66;
FALSE	High	Master Prc	P49773	Histidine tr	0	54.729	79	8	20	8	126	13.8	6.95	48.73	8	cell death;	cytoplasm;	catalytic ac	Pf01230, P	3094	ENSG0000	HINT1	5				0	0.926		0.662058696	9.16	4944.4	5430.1	32.73	0.98	High	High	High	High	High	High	1	
FALSE	High	Master Prc	Q99661-1	Kinesin-like	0	18.069	7	4	4	3	725	81.3	7.83	3.48	4	cell divisio	chromosor	catalytic ac	Pf00225	11004	ENSG0000	KIF2C	1		Regulation	COPI-depe	28	0.926		0.759134525	8	126.2	136.3	13.18	14.62	High	High	High	High	High	High	1	
FALSE	High	Master Prc	P17066	Heat shock	0	85.763	21	12	66	1	643	71	6.14	135.69	12	cell organi:	cytoplasm;	catalytic ac	Pf00012, P	3310	ENSG0000	HSPA6	1	Estrogen si	Parkin-Ubi	Neutrophil	20	0.926		0.802018115	72.11	65.5	68.1	46.31	40.04	High	High	High	High	High	High	1	[Q378;
FALSE	High	Master Prc	P11172	uridine 5'-r	0	24.906	17	6	6	6	480	52.2	7.24	15.53	6	metabolic	cytoplasm;	catalytic ac	Pf00156, P	7372	ENSG0000	UMPS	3	Pyrimidine	Fluoropyri	Pyrimidine	8	0.925		0.028391724	2.35	1155.4	1249	10.72	8.18	High	High	High	High	High	High	1	
FALSE	High	Master Prc	P08237	ATP-depen	0	96.69	33	17	27	14	780	85.1	7.99	65.71	17	cell organi:	cytoplasm;	catalytic ac	Pf00365	5213	ENSG0000	PFKM	12	Central car	Insulin Sig	Glycolysis	16	0.925		0.148689739	1.19	5521.8	6001.2	3.38	6.23	High	High	High	High	High	High	1	
FALSE	High	Master Prc	O95363	phenylalar	0	10.716	8	3	3	3	451	52.3	7.46	9.19	3	metabolic	cytoplasm;	catalytic ac	Pf01409, P	10667	ENSG0000	FARS2	6	Aminoacyl		Mitochond	5	0.925		0.338909403	12.57	484.2	523.2	16.73	5.86	High	High	High	High	High	High	1	
FALSE	High	Master Prc	Q9Y5A9-1	YTH domai	0	37.994	13	5	7	4	579	62.3	8.79	20.29	5	cell organi:	cytoplasm;	protein bir	Pf04146	51441	ENSG0000	YTHDF2	1				0	0.925		0.868956991	0.41	1031.5	1072.9	7.02	5.27	High	High	High	High	High	High	1	
FALSE	High	Master Prc	Q13362	Serine/thre	0	9.585	8	4	4	4	524	61	6.87	4.61	4	metabolic	chromosor	enzyme re	Pf01603	5527	ENSG0000	PPP2R5C	14	PI3K-Akt si	Focal Adhe	Beta-caten	74	0.925		0.968042879	42.49	588.1	635.9	23.8	12.79	High	High	High	High	High	High	1	
FALSE	High	Master Prc	P30050-1	60S riboso	0	59.985	71	12	29	12	165	17.8	9.42	66.86	12	cell organi:	cytosol;me	protein bir	Pf00298, P	6136	ENSG0000	RPL12	9	Ribosome	Cytoplasm	Peptide ch	34	0.924		0.008619992	0.72	6427.4	6942.2	5.02	3.28	High	High	High	High	High	High	1	
FALSE	High	Master Prc	Q92499	ATP-depen	0	83.078	24	17	31	17	740	82.4	7.23	61.12	17	cell organi:	cytoplasm;	catalytic ac	Pf00270, P	1653	ENSG0000	DDX1	2		DDX1 as a	tRNA proc	5	0.924		0.165456466	1.7	7754.1	8699.2	10.31	2.95	High	High	High	High	High	High	1	
FALSE	High	Master Prc	A0A0U1RF	negative re	0.005	2.554	12	1	2	1	68	7	9.42	4.89	1	metabolic	cytoplasm	protein bir		550643	ENSG0000	LOC55064:	X				0	0.924		0.461307705	21.66	431.2	470	3.96	12.24	High	High	High	High	High	High	1	
FALSE	High	Master Prc	Q96AB3	isochorism	0	20.429	34	3	3	3	205	22.3	7.77	9.59	3	metabolic	cytoplasm;	catalytic ac	Pf00857	79763	ENSG0000	ISOC2	19				0	0.924		0.578528908	38.36	342.8	370.8	24.88	18.3	High	High	High	High	High	High	1	
FALSE	High	Master Prc	P62258-1	14-3-3 pro	0	185.021	69	23	108	20	255	29.2	4.74	275.99	23	cell organi:	cytoplasm;	protein bir	Pf00244	7531	ENSG0000	YWHAE	17; CHR_H	Cell cycle;	Cell Cycle;	Regulation	65	0.924		0.649932811	5.13	31793.6	34221	10.15	2.98	High	High	High	High	High	High	1	
FALSE	High	Master Prc	O15381-1	Nuclear va	0	20.746	4	2	3	2	856	95	6.48	9.26	2	metabolic	membrane	catalytic ac	Pf00004, P	4931	ENSG0000	NVL	1	Ribosome			1	0.924		0.762624893	41.34	355.4	384.8	16.25	16.98	High	High	High	High	High	High	1	
FALSE	High	Master Prc	P18859	ATP syntha	0	46.922	56	6	18	6	108	12.6	9.52	53.67	6	cell organi:	membrane	catalytic ac	Pf05511	522	ENSG0000	ATP5J	21	Huntingtor	Electron Tr	Cristae for	14	0.924		0.804783901	7.15	3569.3	3861.1	5.79	8.28	High	High	High	High	High	High	1	
FALSE	High	Master Prc	Q8IWJ2	GRIP and c	0	9.85	3	3	3	3	1684	195.8	5.14	4.54	3	cell organi:	cytoplasm;	protein bir	Pf00038, P	9648	ENSG0000	GCC2	2		Deregulati	Retrograde	5	0.924		0.920819917	0.13	91.8	85	9.49	10.58	High	High	High	High	High	High	1	
FALSE	High	Master Prc	O75534	cold shock	0	117.867	34	29	52	29	798	88.8	6.25	97.14	29	metabolic	cytoplasm;	DNA bindi	Pf00313, P	7812	ENSG0000	CSDE1	1				0	0.923		0.048795239	4.35	9900.2	10588.4	3.38	3.83	High	High	High	High	High	High	1	
FALSE	High	Master Prc	O75643-1	U5 small n	0	347.713	36	59	102	59	2136	244.4	6.06	265.46	59	cell differe	membrane	catalytic ac	Pf00270, P	23020	ENSG0000	SNRNP200	2	Spliceosom		mRNA Spli	6	0.923		0.053744089	0.45	22452.9	24518.3	3.25	5.8	High	High	High	High	High	High	1	
FALSE	High	Master Prc	Q9NR56-1	Muscleblin	0	21.515	16	5	8	5	388	41.8	8.9	19.36	5	cell differe	cytoplasm;	metal ion l		4154	ENSG0000	MBNL1	3		Adipogene		1	0.923		0.061117799	4.32	2289.6	2448.5	5.1	3.43	High	High	High	High	High	High	1	
FALSE	High	Master Prc	Q8WUM0	Nuclear po	0	32.895	6	4	5	4	1156	128.9	5.1	24.05	4	cell differe	chromosor	protein bir	Pf03177, P	55746	ENSG0000	NUP133	1	RNA transp		Rev-media	77	0.923		0.09151602	0.88	802.1	825.3	4.3	5.53	High	High	High	High	High	High	1	
FALSE	High	Master Prc	Q5JSH3	WD repeat	0	10.189	4	3	3	3	913	101.3	5.45	5.49	3		cytoplasm;	protein bir	Pf00400	54521	ENSG0000	WDR44	X		Ectoderm		1	0.923		0.224792443	2.46	550.9	683.5	11.08	10.85	High	High	High	High	High	High	1	
FALSE	Medium	Master Prc	P46020-1	Phosphory	0.011	2.038	1	1	1	1	1223	137.2	6.19	2.61	1	metabolic	cytosol;me	catalytic ac	Pf00723	5255	ENSG0000	PHKA1	X	Insulin sigr	Glycogen M	Glycogen b	8	0.923		0.258334081	9.04	107.3	116.3	5.36	3.11	High	High	High	High	High	High	1	
FALSE	High	Master Prc	P31949	protein S10	0	52.369	56	6	34	6	105	11.7	7.12	63.51	6	regulation	cytoplasm;	metal ion l	Pf00036, P	6282	ENSG0000	S100A11	1			Neutrophil	3	0.923		0.258342911	7.23	8397.9	9016.8	26.81	6.33	High	High	High	High	High	High	1	
FALSE	High	Master Prc	Q8NE71-1	ATP-bindir	0	88.636	20	14	26	14	845	95.9	6.8	58.42	14	defense re	cytoplasm;	catalytic ac	Pf00005, P	23	ENSG0000	ABCF1	6; CHR_HS			ABC-family	2	0.923		0.566573272	13.85	7167.5	7768.7	9.5	3.88	High	High	High	High	High	High	1	
FALSE	High	Master Prc	Q6NZI2-1	caveolae-a	0	19.128	12	4	6	4	390	43.5	5.6	14.72	4	metabolic	cytoplasm;	protein bir	Pf15237	284119	ENSG0000	PTRF; CAVI	17			RNA Polym	3	0.923		0.667936115	27.1	953.4	1019	12.36	9.05	High	High	High	High	High	High	1	
FALSE	High	Master Prc	P27708	CAD protei	0	172.691	19	32	43	28	2225	242.8	6.46	112.66	32	metabolic	cytoplasm;	catalytic ac	Pf00117, P	790	ENSG0000	CAD	2	Metabolic	Endothelin	Pyrimidine	9	0.922		0.007513926	0.95	9671.4	10423.8	1.03	0.87	High	High	High	High	High	High	1	
FALSE	High	Master Prc	Q6P2Q9	Pre-mRNA-	0	219.729	25	47	66	47	2335	273.4	8.84	161.32	47	cell organi:	membrane	catalytic ac	Pf01398, P	10594	ENSG0000	PRPF8	17; CHR_H	Spliceosom	mRNA Pro	mRNA Spli	7	0.922		0.078585636	1.5	16488.1	17494.8	5.57	4.4	High	High	High	High	High	High	1	
FALSE	High	Master Prc	P61106	Ras-related	0	51.151	50	9	15	9	215	23.9	6.21	43.58	9	cell organi:	cytosol;en	catalytic ac	Pf00009, P	51552	ENSG0000	RAB14	9	AMPK sign	Focal Adhe	Translocat	18	0.922		0.421267219	18.11	6727.4	7295.3	8.84	3.78	High	High	High	High	High	High	1	
FALSE	High	Master Prc	P19784	casein kina	0	19.928	17	4	4	4	350	41.2	8.56	15.07	4	cell commu	chromosor	catalytic ac	Pf00069, P	1459	ENSG0000	CSNK2A2	16	Measles; R	Allograft R	WNT medi	45	0.922		0.442455331	21.43	890.4	902	18.89	6.53	High	High	High	High	High	High	1	
FALSE	High	Master Prc	Q9BSH4	Translation	0	10.214	9	2	3	2	297	32.5	8.13	11.08	2	regulation	mitochond	protein bir	Pf01709	51204	ENSG0000	TACO1	17			TP53 Regu	9	0.922		0.459836458	24.62	1159.1	1257.7	17.97	12.03	High	High	High	High	High	High	1	
FALSE	High	Master Prc	O76003	glutaredox	0	46.461	38	11	14	11	335	37.4	5.39	26.71	11	cell organi:	cytoplasm;	catalytic ac	Pf00085, P	10539	ENSG0000	GLRX3	10			Iron uptake	2	0.922		0.697628637	10.26	4697.7	5093.1	11.5	2.18	High	High	High	High	High	High	1	
FALSE	High	Master Prc	O75531	Barrier-to-	0	30.086	64	7	14	7	89	10.1	6.09	40.69	7	cell organi:	chromosor	DNA bindi	Pf02961	8815	ENSG0000	BANF1	11			2-LTR circl	23	0.922		0.731111166	0.85	2966.9	2912	17.36	8.21	High	High	High	High	High	High	1	
FALSE	High	Master Prc	P46940	Ras GTPase	0	343.147	32	49	103	48	1657	189.1	6.48	274.64	49	cell growth	cytoplasm;	enzyme re	Pf00307, P	8826	ENSG0000	IQGAP1	15	Proteoglyc	VEGFA-VEC	Signaling b	35	0.921		0.063090416	5.02	26581.5	28787.9	0.17	3.55	High	High	High	High	High	High	1	
FALSE	High	Master Prc	Q13555	Calcium/ca	0	10.711	8	3	3	2	558	62.6	7.83	8.63	3	cell differe	cytoplasm;	catalytic ac	Pf00069, P	818		CAMK2G	10	Adrenergic	Energy Me	Ion homeo	61	0.921		0.072883815	2.64	168.9	184.8	6.03	3.11	High	High	High	High	High	High	1	
FALSE	High	Master Prc	P60228	Eukaryotic	0	42.338	23	8	14	8	445	52.2	6.04	39.89	8	metabolic	chromosor	protein bir	Pf01399, P	3646	ENSG0000	EIF3E	8	RNA transp	Translation	Formation	14	0.921		0.100365116	2.13	5420.2	5802.7	1.91	3.47	High	High	High	High	High	High	1	
FALSE	High	Master Prc	P14324	Farnesyl py	0	44.858	16	6	19	6	419	48.2	6.15	34.01	6	metabolic	cytoplasm;	catalytic ac	Pf00348	2224	ENSG0000	FDPS	1	Terpenoid	Cholestero	Cholestero	12	0.921		0.15342195	7.18	4180.5	4451.8	2.54	6.57	High	High	High	High	High	High	1	
FALSE	High	Master Prc	P24941-1	cyclin-depe	0	24.618	26	7	10	6	298	33.9	8.68	22.51	7	cell divisio	chromosor	catalytic ac	Pf00069, P	1017	ENSG0000	CDK2	12	Small cell l	miRNA Reg	p53-Deper	88	0.921		0.199058402	10.79	3223.4	3526.5	1.3	6.44	High	High	High	High	High	High	1	
FALSE	High	Master Prc	P28066-1	Proteasom	0	127.141	63	12	46	12	241	26.4	4.79	138.44	12	metabolic	cytoplasm;	catalytic ac	Pf00227, P	5686	ENSG0000	PSMA5	1	Proteasom	Proteasom	Regulation	151	0.921		0.301185925	9.16	11232	12194.4	4.23	4.55	High	High	High	High	High	High	1	
FALSE	High	Master Prc	Q96DI7	U5 small n	0	52.853	44	10	13	10	357	39.3	8.1	26.79	10	cell organi:	cytosol;nu	protein bir	Pf00400	9410	ENSG0000	SNRNP40	1	Spliceosom	mRNA Pro	mRNA Spli	7	0.921		0.315359073	11.57	1314.3	1408.1	23.64	5.63	High	High	High	High	High	High	1	
FALSE	High	Master Prc	Q5VYK3	Proteasom	0	38.986	5	7	8	7	1845	204.2	7.12	24.36	7	cell organi:	cytoplasm;	protein bir	Pf12755, P	23392		KIAA0368	9				0	0.921		0.343042489	14.94	628.8	733.9	13.18	11.66	High	High	High	High	High	High	1	
FALSE	High	Master Prc	P20290-1	transcripti	0	111.006	63	13	47	2	206	22.2	9.38	118.05	13	metabolic	cytoplasm;	protein bir	Pf01849	689	ENSG0000	BTF3	5				0	0.921		0.368111503	12.08	709.4	679.9	13.91	10.61	High	High	High	High	High	High	1	
FALSE	High	Master Prc	P51398-1	28S riboso	0	34.676	19	6	9	6	398	45.5	8.88	31.5	6	cell death;	membrane	nucleotide	Pf10236	7818	ENSG0000	DAP3	1			Mitochond	6	0.921		0.426651872	3.31	3480.5	3727.3	4.41	4.19	High	High	High	High	High	High	1	
FALSE	High	Master Prc	Q9UQE7	Structural	0	57.935	13	13	18	13	1217	141.5	7.18	38.38	13	cell divisio	chromosor	catalytic ac	Pf01496, P	9126	ENSG0000	SMC3	10	Oocyte me	Retinoblas	Meiotic sy	24	0.921		0.510021071	6.21	3721.4	4039.7	3.84	6.42	High	High	High	High	High	High	1	
FALSE	High	Master Prc	Q9UHB9	Signal reco	0	65.546	26	12	18	12	627	70.7	8.56	61.2	12	cell organi:	cytoplasm;	protein bir		6730	ENSG0000	SRP68	17	Protein exp		SRP-depen	4	0.921		0.748699454	6.06	3017.4	3255.4	11.7	1.15	High	High	High	High	High	High	1	
FALSE	High	Master Prc	P63279	SUMO-con	0	27.866	36	3	8	3	158	18	8.66	34.09	3	cell divisio	cytoplasm;	catalytic ac	Pf00179, P	7329	ENSG0000	UBE2I	16	Ubiquitin r	Androgen	SUMOylati	36	0.921		0.758157461	0.98	3366.7	3623.8	6.94	4.08	High	High	High	High	High	High	1	
FALSE	High	Master Prc	Q8N0X7	Spartin [OS	0	37.949	14	8	11	8	666	72.8	5.91	25.15	8	cell divisio	cytoplasm;	protein bir	Pf04212, P	23111	ENSG0000	SPG20; SPA	13	Endocytosi			1	0.921		0.980914483	12.91	3627.9	3880.5	18.79	2.63	High	High	High	High	High	High	1	
FALSE	High	Master Prc	Q9NQW7-	xaa-Pro an	0	63.224	25	10	13	10	623	69.9	5.67	47.47	10	metabolic	cytoplasm;	catalytic ac	Pf00557, P	7511		XPNPEP1	10				0	0.92		0.061722511	4.98	6012.2	6559.2	7.86	3.89	High	High	High	High	High	High	1	
FALSE	High	Master Prc	P62316	Small nucle	0	60.031	53	8	32	8	118	13.5	9.91	67.34	8	cell organi:	cytoplasm;	protein bir	Pf01423	6633	ENSG0000	SNRPD2	19	Spliceosom	mRNA Pro	snRNP Ass	9	0.92		0.13688314	7.39	11086.7	12417	6.53	6.12	High	High	High	High	High	High	1	
FALSE	High	Master Prc	P11216	Glycogen p	0	14.555	4	2	3	1	843	96.6	6.86	9.99	2	metabolic	cytoplasm;	catalytic ac	Pf00343	5834	ENSG0000	PYGB	20	Starch and	Glycogen M	Neutrophil	13	0.92		0.224928286	3.03	239.3	260	5.44	0.6	High	High	High	High	High	High	1	
FALSE	High	Master Prc	P37802	Transgelin-	0	119.289	70	14	45	13	199	22.4	8.25	101.74	14	cell differe	cytosol;ext	protein bir	Pf00307, P	8407	ENSG0000	TAGLN2	1			Platelet de	4	0.92		0.283030439	10.85	12946.3	14073.3	24.33	5.41	High	High	High	High	High	High	1	
FALSE	High	Master Prc	Q9Y4W2	Ribosomal	0	19.302	11	6	6	6	734	83	4.73	7.94	6	cell organi:	cytoplasm;	RNA bindi	Pf04031	81887	ENSG0000	LAS1L	X			Major path	4	0.92		0.295747884	14.31	1253.9	1370.4	9.1	7.01	High	High	High	High	High	High	1	
FALSE	High	Master Prc	P39019	40S riboso	0	79.152	70	15	61	15	145	16.1	10.32	96	15	cell differe	cytoplasm;	protein bir	Pf01090	6223	ENSG0000	RPS19	19	Ribosome	Cytoplasm	Peptide ch	38	0.919		0.084030327	2.06	19576.4	21290.5	2.34	7.47	High	High	High	High	High	High	1	[T55(98.7)
FALSE	High	Master Prc	Q16762	thiosulfate	0	14.269	14	2	3	2	297	33.4	7.25	12.87	2	cell differe	membrane	catalytic ac	Pf00581	7263	ENSG0000	TST	22	Sulfur met		Sulfide oxi	9	0.919		0.138021525	6.78	188.5	222.8	18.01	16.72	High	High	High	High	High	High	1	
FALSE	High	Master Prc	P08240-1	signal reco	0	50.83	19	8	15	8	638	69.8	8.95	30.95	8	cell organi:	endoplasm	catalytic ac	Pf00448, P	6734	ENSG0000	SRPR; SRPI	11	Protein exp		XBP1(S) ac	7	0.919		0.281546083	7.73	3948.5	4347.4	20.59	4.53	High	High	High	High	High	High	1	
FALSE	High	Master Prc	Q53EL6-1	Programm	0	19.116	13	5	10	5	469	51.7	5.21	13.5	5	cell death;	cytoplasm;	protein bir	Pf02847	27250	ENSG0000	PDCD4	10	Proteoglyc	Interferon	Gene and p	8	0.919		0.474237436	13.04	1012.7	1037.8	12	3.72	High	High	High	High	High	High	1	
FALSE	High	Master Prc	Q16513	Serine/thre	0	18.668	6	5	6	4	984	112	6.3	9.98	5	cell death;	cytoplasm;	catalytic ac	Pf00069, P	5586	ENSG0000	PKN2	1	Salmonella		RHO GTPa:	6	0.919		0.498013392	8.59	690.9	741.4	3.6	7.97	High	High	High	High	High	High	1	
FALSE	High	Master Prc	Q9UK53-1	inhibitor o	0	7.838	6	2	2	2	422	46.7	9.06	5.45	2	regulation	nucleus	metal ion l	Pf00628, P	3621		ING1	13		Senescenc		1	0.919		0.88692236	18.74	20.5	19.8	22.73	10	High	High	High	High	High	High	1	
FALSE	High	Master Prc	P41252	isoleucine-	0	35.416	8	9	11	9	1262	144.4	6.15	16.19	9	cell differe	cytoplasm;	catalytic ac	Pf00133, P	3376	ENSG0000	IARS	9	Aminoacyl	Amino Aci	Cytosolic t	10	0.918		0.047629963	3.98	1853.5	1987.5	4.47	1.16	High	High	High	High	High	High	1	
FALSE	High	Master Prc	P60842	Eukaryotic	0	190.758	59	22	111	13	406	46.1	5.48	327.74	22	metabolic	cytoplasm;	catalytic ac	Pf00270, P	1973	ENSG0000	EIF4A1	17	RNA transp	Translation	ISG15 anti	20	0.918		0.224649265	12.19	15909.8	17330.4	4.55	3.91	High	High	High	High	High	High	1	
FALSE	High	Master Prc	Q9BWM7	Sideroflexi	0	18.795	25	6	9	5	321	35.5	9.1	13.6	6	transport	membrane	transporte	Pf03820	81855	ENSG0000	SFXN3	10				0	0.918		0.227082837	2.25	1293	1409	0.9	5.36	High	High	High	High	High	High	1	
FALSE	High	Master Prc	O00422	Histone de	0	29.619	43	7	15	7	153	17.6	9.35	37.18	7	cell organi:	cytoplasm;	catalytic ac	Pf06487	10284	ENSG0000	SAP18	13	mRNA surv	Hedgehog	HDACs dea	10	0.918		0.43844626	0.48	5077.9	5188.9	4.59	5.62	High	High	High	High	High	High	1	
FALSE	High	Master Prc	Q9UG63	ATP-bindir	0	37.673	12	6	11	6	623	71.2	7.37	31.88	6	transport	membrane	catalytic ac	Pf00005, P	10061	ENSG0000	ABCF2	7				0	0.918		0.549319494	6.84	2840.1	3053.6	8.84	2.46	High	High	High	High	High	High	1	
FALSE	High	Master Prc	P29218-1	Inositol mo	0	13.604	12	2	2	2	277	30.2	5.26	7.16	2	metabolic	cytoplasm;	catalytic ac	Pf00459	3612	ENSG0000	IMPA1	8	Phosphatic	Integrated	Synthesis c	7	0.917		0.079095907	2.01	293.8	320	3.2	5.1	High	High	High	High	High	High	1	
FALSE	High	Master Prc	Q6NZ67	Mitotic-spi	0.001	4.489	9	1	1	1	158	16.2	10.15	3.82	1		cytoplasm;	protein bir	Pf12926	80097	ENSG0000	MZT2B	2			Recruitme	9	0.917		0.152144473	1.27	341.7	372.6	2.87	7.45	High	High	High	High	High	High	1	
FALSE	High	Master Prc	P50570-1	Dynamin-2	0	18.387	6	4	6	4	870	98	7.44	12.69	4	cell organi:	cytoplasm;	catalytic ac	Pf00169, P	1785	ENSG0000	DNM2	19	Fc gamma	Synaptic V	Formation	37	0.917		0.175540021	10.85	2550.3	2694.9	7.98	2.06	High	High	High	High	High	High	1	
FALSE	High	Master Prc	P37268	squalene s	0	8.982	3	1	3	1	417	48.1	6.54	12.03	1	metabolic	endoplasm	catalytic ac	Pf00494	2222	ENSG0000	FDFT1	8	Metabolic	Cholestero	Cholestero	13	0.917		0.195603965	4.23	702.1	709.5	4.6	6.95	High	High	High	High	High	High	1	
FALSE	High	Master Prc	Q96QK1	Vacuolar p	0	85.73	25	17	25	17	796	91.6	5.49	75.92	17	cell organi:	cytoplasm;	protein bir	Pf03635	55737	ENSG0000	VPS35	16	Endocytosi		WNT ligan	4	0.917		0.312598505	12.08	4624	4917.2	6.11	5.57	High	High	High	High	High	High	1	
FALSE	High	Master Prc	P20618	proteasom	0	49.367	44	9	19	9	241	26.5	8.13	41.08	9	cell organi:	cytoplasm;	catalytic ac	Pf00227	5689	ENSG0000	PSMB1	6	Proteasom	Proteasom	Regulation	151	0.917		0.406778822	21.1	2606	2842.9	21.06	10.56	High	High	High	High	High	High	1	
FALSE	High	Master Prc	P56937-1	3-keto-ster	0	7.812	8	2	2	2	341	38.2	8.1	6.06	2	metabolic	endoplasm	catalytic ac	Pf00106, P	51478	ENSG0000	HSD17B7	1	Ovarian ste	Steroid Bic	Cholestero	9	0.917		0.442426137	6.9	791.2	862.8	3.98	6.12	High	High	High	High	High	High	1	
FALSE	High	Master Prc	Q9Y316-1	Protein ME	0	13.795	11	2	2	2	297	33.7	7.14	6.07	2	regulation	cytosol;nu	protein bir	Pf01875	51072	ENSG0000	MEMO1	2			ERBB2 Reg	4	0.917		0.50334411	31.39	242	262.4	29.66	13.17	High	High	High	High	High	High	1	

FALSE	High	Master Prc	P23368-1	NAD-deper	0	40.837	17	6	9	6	584	65.4	7.61	18.43	6	metabolic	mitochond	catalytic a	Pf00390, P	4200	ENSG0000	ME2	18	Pyruvate n			2	0.917	0.595597634	41.63	256.3	279.4	14.25	25.29	High	High	High	High	High	High	1	
FALSE	High	Master Prc	O00541-1	Pescadillo	0	35.746	12	7	15	7	588	68	7.33	30.6	7	cell organi	chromosor	protein bir	Pf00533, P	23481	ENSG0000	PES1	22			Major path	4	0.917	0.66326341	8.83	4356.5	4899.5	14.42	2.09	High	High	High	High	High	High	1	
FALSE	High	Master Prc	P04264	Keratin, ty	0	135.172	39	20	53	17	644	66	8.12	151.58	20	cell death;	cytoskelet	catalytic a	Pf00038, P	3848	ENSG0000	KRT1	12		Corticotro	Formation	7	0.917	0.775680047	7.06	13840.5	14594.2	28.74	8.74	High	High	High	High	High	High	1	
FALSE	High	Master Prc	P61081	NEDD8-cor	0	45.395	60	10	18	10	183	20.9	7.69	40.07	10	metabolic	cytoplasm;	catalytic a	Pf00179	9040	ENSG0000	UBE2M	19	Ubiquitin r		TGF-beta r	19	0.917	0.787491253	2.6	7054	7669.9	10.49	2.23	High	High	High	High	High	High	1	
FALSE	High	Master Prc	P78344-1	Eukaryotic	0	67.929	21	15	18	15	907	102.3	7.14	46.06	15	cell death;	cytosol;me	protein bir	Pf02020, P	1982	ENSG0000	EIF4G2	11	RNA trans	Exercise-in	ISG15 anti	8	0.916	0.027414238	2.26	2346.4	2562.6	2.64	4.72	High	High	High	High	High	High	1	
FALSE	High	Master Prc	P22392-2	Isoform 3	0	159.949	83	18	93	3	267	30.1	8.92	263.54	18	cell organi	cytoplasm;	catalytic a	Pf00334	654364; 4	ENSG0000	NME1-NM	17	Purine me	Nucleotide		4	0.916	0.028895432	3.46	3273.9	3488.7	4.17	5.38	High	High	High	High	High	High	1	
FALSE	High	Master Prc	P07858	Cathepsin	0	35.702	18	5	10	5	339	37.8	6.3	31.88	5	cell differe	extracellul	catalytic a	Pf00112, P	1508	ENSG0000	CTSB	8	Antigen pr		Collagen d	17	0.916	0.144299637	3.76	1655.3	1844.7	3.2	6.99	High	High	High	High	High	High	1	
FALSE	High	Master Prc	P51003	Poly(A) pol	0	8.432	3	2	2	2	745	82.8	7.37	0	2	metabolic	cytoplasm;	catalytic a	Pf01909, P	10914	ENSG0000	PAPOLA	14	mRNA surv	mRNA Pro	Cleavage o	13	0.916	0.162706636	7.14	1175.7	1250.7	9.77	2.83	High	High	High	High	High	High	1	
FALSE	High	Master Prc	P49902	Cytosolic p	0	18.281	11	5	5	5	561	64.9	6.14	11.32	5	metabolic	cytoplasm;	catalytic a	Pf05761	22978	ENSG0000	NT5C2	10	Metabolic		Abacavir m	10	0.916	0.173933777	7.45	1411.4	1541.6	1.75	7.6	High	High	High	High	High	High	1	
FALSE	High	Master Prc	Q9Y2W2	WW doma	0	33.272	15	7	11	7	641	70	8.38	16.73	7	metabolic	cytoplasm;	DNA bindi	Pf09429	51729	ENSG0000	WBP11	12	Spliceosom		mRNA Spli	5	0.916	0.189734089	1.4	1564.4	1697.5	4.07	1.45	High	High	High	High	High	High	1	
FALSE	High	Master Prc	P38919	Eukaryotic	0	108.22	49	18	54	15	411	46.8	6.73	117.25	18	metabolic	cytoplasm;	catalytic a	Pf00270, P	9775	ENSG0000	EIF4A3	17	mRNA surv		ISG15 anti	27	0.916	0.244348684	7.34	8834.1	10292.5	10.15	8.75	High	High	High	High	High	High	1	
FALSE	High	Master Prc	Q9Y2P8	RNA 3'-ter	0	7.253	4	1	1	1	373	40.8	9.26	3.64	1	metabolic	nucleus	catalytic a	Pf01137, P	10171	ENSG0000	RCL1	9	Ribosome		Major path	6	0.916	0.445041643	23.53	157.7	176.4	12.31	11.09	High	High	High	High	High	High	1	
FALSE	High	Master Prc	Q96FX7	tRNA (ade	0	9.751	11	2	3	2	289	31.4	7.36	12.45	2	metabolic	nucleus	catalytic a	Pf08704, P	115708	ENSG0000	TRMT61A	14			tRNA mod	3	0.916	0.468591838	17.48	899.4	981.5	10.42	3.58	High	High	High	High	High	High	1	
FALSE	High	Master Prc	P17844	probable A	0	151.992	40	31	75	22	614	69.1	8.92	152.04	31	cell differe	cytoplasm;	catalytic a	Pf00270, P	1655	ENSG0000	DDX5	17	Proteoglyc		mRNA Spli	7	0.916	0.717539147	2.44	18016.1	19672.6	6.4	5.37	High	High	High	High	High	High	1	
FALSE	High	Master Prc	P52209	6-phospho	0	141.746	51	18	39	18	483	53.1	7.23	137.1	18	metabolic	cytoplasm;	catalytic a	Pf00393, P	5226	ENSG0000	PGD	1	Carbon me	Pentose P	Pentose ph	9	0.915	0.023510528	2.77	11625.7	12680	1.83	0.69	High	High	High	High	High	High	1	
FALSE	High	Master Prc	P83731	60S riboso	0	57.577	41	9	30	9	157	17.8	11.25	71.67	9	cell organi	cytoplasm;	protein bir	Pf01246	6152	ENSG0000	RPL24	3	Ribosome	Cytoplasm	Peptide ch	34	0.915	0.155970389	2.6	17751.4	19069.8	22.73	8.25	High	High	High	High	High	High	1	
FALSE	High	Master Prc	Q9GZL7	ribosome b	0	41.061	21	6	11	6	423	47.7	5.9	31.16	6	cell prolife	nucleus	protein bir	Pf00400, P	55759	ENSG0000	WDR12	2			Major path	4	0.915	0.196640487	8.78	4137.2	4783.8	8.15	6.82	High	High	High	High	High	High	1	
FALSE	High	Master Prc	Q13228	Selenium-b	0	12.489	7	2	2	2	472	52.4	6.37	6.32	2	transport	cytoplasm;	protein bir	Pf05694, P	8991	ENSG0000	SELENBP1	1		Selenium M		2	0.915	0.280532454	11.97	202.9	208.7	3.79	9.32	High	High	High	High	High	High	1	
FALSE	High	Master Prc	Q9NPJ6-1	mediator c	0	16.644	11	2	2	2	270	29.7	5.1	8.18	2	metabolic	membrane	protein bir	Pf10018	29079	ENSG0000	MED4	13	Thyroid ho		Transcripti	10	0.915	0.3416896	0.59	721.9	779.2	9.13	3.8	High	High	High	High	High	High	1	
FALSE	High	Master Prc	O00764-1	Pyridoxal k	0	24.82	20	4	7	4	312	35.1	6.13	22	4	cell prolife	cytoplasm;	catalytic a	Pf00294, P	8566	ENSG0000	PDXK	21	Metabolic		Vitamins B	9	0.915	0.437400993	25.09	481.7	530.8	18.29	16.46	High	High	High	High	High	High	1	
FALSE	High	Master Prc	Q99627-1	COP9 signa	0	8.822	15	2	2	2	209	23.2	5.38	3.92	2	cell organi	cytoplasm;	protein bir	Pf10075	10920	ENSG0000	COPS8	2			Cargo reco	13	0.915	0.693180922	60.26	181.5	198.4	52.92	20.79	High	High	High	High	High	High	1	
FALSE	High	Master Prc	P49662	Caspase-4	0	7.896	5	1	1	1	377	43.2	6	4.87	1	cell death;	cytoplasm;	catalytic a	Pf00619, P	837	ENSG0000	CASP4	11	NOD-like r	Apoptosis	NOD1/2 Si	7	0.915	0.86705087	15.57	239.1	261.3	10.39	10.55	High	High	High	High	High	High	1	
FALSE	High	Master Prc	P30153	serine/thre	0	100.911	35	19	37	12	589	65.3	5.11	85.09	19	cell death;	chromosor	catalytic a	Pf02985, P	5518	ENSG0000	PPP2R1A	19	Hippo sign	Focal Adhe	Glycolysis;	150	0.915	0.914747925	1	5833.5	6386.4	17.43	1.01	High	High	High	High	High	High	1	
FALSE	High	Master Prc	Q15370	Elongin-B [	0	20.596	46	4	6	4	118	13.1	4.88	14.49	4	cell organi	cytosol;nu	protein bir	Pf00240	6923	ENSG0000	TCEB2; EL(	16	Ubiquitin r	Dual hijack	Oxygen-de	45	0.914	0.014440013	1.13	1176.2	1276.8	3.69	4.6	High	High	High	High	High	High	1	
FALSE	High	Master Prc	P78527	DNA-deper	0	306.376	20	69	99	69	4128	468.8	7.12	235.43	69	cell death;	cytosol;me	catalytic a	Pf00454, P	5591	ENSG0000	PRKDC	8	Non-homo	miRNA Re	Nonhomol	22	0.914	0.034934578	1.45	17761.7	19248.7	1.75	4.73	High	High	High	High	High	High	1	
FALSE	High	Master Prc	P40306	Proteasom	0	8.272	10	2	2	2	273	28.9	7.81	9.36	2	metabolic	cytoplasm;	catalytic a	Pf00227, P	5699	ENSG0000	PSMB10	16	Proteasom	Proteasom	Regulation	150	0.914	0.16847946	8.03	410.8	431.6	11.05	3.62	High	High	High	High	High	High	1	
FALSE	High	Master Prc	Q9HBL8	NmrA-like	0	8.003	6	1	1	1	299	33.3	7.52	5.19	1	metabolic	cytoplasm;	protein bir	Pf00106, P	57407	ENSG0000	NMRAL1	16; CHR_H			Urea cycle	4	0.914	0.168655513	5.56	569.6	616.8	3.29	3.52	High	High	High	High	High	High	1	
FALSE	High	Master Prc	O95433	activator o	0	38.655	21	6	15	6	338	38.3	5.53	30.84	6	regulation	cytoplasm;	enzyme re	Pf08327, P	10598	ENSG0000	AHSA1	14		T-Cell anti		1	0.914	0.192019945	3.02	5287.7	5960.4	10.97	5.11	High	High	High	High	High	High	1	
FALSE	High	Master Prc	Q8NBF2-1	NHL repea	0	6.96	3	1	1	1	726	79.4	5.55	5.91	1	cellular ho	extracellul	antioxidan	Pf00578, P	374354	ENSG0000	NHLRC2	10			Platelet de	4	0.914	0.216002027	10.65	110.7	126	6.92	6.78	High	High	High	High	High	High	1	
FALSE	High	Master Prc	Q6DKJ4	nucleoredc	0	33.667	17	5	6	5	435	48.4	4.97	20.88	5	cell differe	cytoplasm;	antioxidan	Pf00085, P	64359	ENSG0000	NXN	17				0	0.914	0.221907835	4.33	2099.6	2179.9	8.58	4.75	High	High	High	High	High	High	1	
FALSE	High	Master Prc	P04818	thymidylat	0	42.969	38	9	13	9	313	35.7	7.01	34.36	9	metabolic	cytoplasm;	catalytic a	Pf00303	7298	ENSG0000	TYMS	18	One carbo	Fluoropyri	Activation	18	0.914	0.278965357	14.42	2483.3	2849.9	12.38	3.1	High	High	High	High	High	High	1	
FALSE	High	Master Prc	P41743	Protein kin	0	25.487	14	5	6	4	596	68.2	5.85	19.08	5	cell organi	cytoplasm;	catalytic a	Pf00069, P	5584	ENSG0000	PRKCI	3	Insulin sigr	miRs in Mu	Tight junct	25	0.914	0.45918567	30.18	415.1	514.3	25.06	21.22	High	High	High	High	High	High	1	
FALSE	High	Master Prc	P51991-1	Heterogen	0	109.771	46	18	41	16	378	39.6	9.01	98.94	18	metabolic	cytoplasm;	protein bir	Pf00076, P	220988	ENSG0000	HNRNPA3	2	Spliceosom		mRNA Spli	5	0.914	0.548287924	32.65	7533.2	8246	15.08	14.3	High	High	High	High	High	High	1	[R52]
FALSE	High	Master Prc	Q15459	splicing fac	0	71.353	24	14	25	14	793	88.8	5.22	44.38	14	cell organi	nucleus;sp	protein bir	Pf00240, P	10291	ENSG0000	SF3A1	22	Spliceosom	mRNA Pro	mRNA Spli	6	0.914	0.910695933	8.95	4878.4	5473	17.47	2.5	High	High	High	High	High	High	1	
FALSE	High	Master Prc	P53582	Methionin	0	23.323	18	5	5	5	386	43.2	7.17	11.57	5	metabolic	cytoplasm;	catalytic a	Pf00557	23173	ENSG0000	METAP1	4			Signaling b	5	0.914	0.969849919	11.55	977.7	1070.1	17.83	4.67	High	High	High	High	High	High	1	
FALSE	High	Master Prc	Q7KZF4	staphyloco	0	150.589	42	34	63	34	910	101.9	7.17	116.6	34	cell differe	cytoplasm;	catalytic a	Pf00565, P	27044	ENSG0000	SND1	7	Epstein-Ba		Signaling b	6	0.913	0.086952351	7.07	20830.3	22823.7	1.6	3.52	High	High	High	High	High	High	1	
FALSE	High	Master Prc	P61163	Alpha-cent	0	35.019	24	6	10	4	376	42.6	6.64	27.27	6	regulation	cytoplasm;	nucleotide	Pf00022	10121	ENSG0000	ACTR1A	10			COPI-indep	33	0.913	0.087401456	5.02	982.6	1112.6	7.19	3.29	High	High	High	High	High	High	1	
FALSE	High	Master Prc	Q9Y3I0	tRNA-splic	0	42.304	26	10	17	10	505	55.2	7.23	27.25	10	metabolic	cytoplasm;	catalytic a	Pf01139	51493	ENSG0000	C22orf28;	22			tRNA proc	3	0.913	0.163242989	10.48	1165.5	1271.4	6.32	5.61	High	High	High	High	High	High	1	
FALSE	Medium	Master Prc	P60602-1	Reactive o	0.026	1.484	10	1	2	1	79	8.2	9.33	0	1	defense re	membrane		Pf10247	140823	ENSG0000	ROMO1	20				0	0.913	0.85591164	2.01	1382.2	1498.8	15.66	5.01	High	High	High	High	High	High	1	
FALSE	High	Master Prc	Q8NBI5	Solute carr	0	16.603	13	6	11	6	491	54.5	8.53	4.76	6	transport	membrane			29015	ENSG0000	SLC43A3	11				0	0.913	0.876317589	9.65	1305.2	1430.4	15.62	2.22	High	High	High	High	High	High	1	
FALSE	High	Master Prc	P36578	60S riboso	0	148	59	28	80	27	427	47.7	11.06	166.59	28	cell organi	cytoplasm;	protein bir	Pf00573, P	6124	ENSG0000	RPL4	15	Ribosome	Cytoplasm	Peptide ch	34	0.912	0.008968379	2.13	32403.8	35516.6	3.5	1.72	High	High	High	High	High	High	1	
FALSE	High	Master Prc	P42285	Superkiller	0	83.832	25	19	21	19	1042	117.7	6.52	58.43	19	metabolic	nucleus;sp	catalytic a	Pf00270, P	23517	ENSG0000	SKIV2L2	5	RNA degra		Major path	8	0.912	0.060206011	3.26	5646.4	6327.3	4.08	6.8	High	High	High	High	High	High	1	
FALSE	Low	Master Prc	Q6XZF7	Dynamin-b	0.054	1.162	1	1	1	1	1577	177.2	5.39	0	1	regulation	cytoplasm;	protein bir	Pf00018, P	23268	ENSG0000	DNMBP	10				0	0.912	0.064281573	4.46	83.8	95.4	3.29	6.09	High	High	High	High	High	High	1	[K971;
FALSE	High	Master Prc	Q2NL82	Pre-rRNA-p	0	41.44	14	9	13	9	804	91.8	7.42	28.5	9	cell organi	cytosol;nu	catalytic a	Pf04950, P	55720	ENSG0000	TSR1	17			Major path	4	0.912	0.258733171	10.52	2589.2	2714.7	6.91	6.23	High	High	High	High	High	High	1	
FALSE	High	Master Prc	Q09666-1	Neuroblast	0	807.447	51	144	265	144	5890	628.7	6.15	696.66	144	cell organi	cytoplasm;	protein bir		79026	ENSG0000	AHNAK	11				0	0.912	0.643871795	14.31	77617.7	80675.8	11.42	8.66	High	High	High	High	High	High	1	
FALSE	High	Master Prc	O60610-1	Protein dia	0	66.301	16	16	18	16	1272	141.3	5.41	42.73	16	cell organi	cytoplasm;	catalytic a	Pf01576, P	1729	ENSG0000	DIAPH1	5	Focal adhe	Regulation	RHO GTPa	19	0.911	0.011134632	1.47	3829.4	4199.2	1.24	0.51	High	High	High	High	High	High	1	
FALSE	High	Master Prc	P04632	Calpain sm	0	97.745	60	9	21	9	268	28.3	5.2	73.87	9	cell death;	cytoplasm;	catalytic a	Pf13833	826	ENSG0000	CAPNS1	19		Integrin-m	Degradatio	9	0.911	0.349751466	18.31	5038.9	5533.9	12.17	3.88	High	High	High	High	High	High	1	
FALSE	High	Master Prc	Q13510	Acid ceram	0	8.969	10	4	4	4	395	44.6	7.62	5.98	4	metabolic	extracellul	catalytic a	Pf02275, P	427	ENSG0000	ASAH1	8	Lysosome;	Sphingolip	Glycosphin	13	0.911	0.469892171	28.32	366.4	398.8	29.83	8.15	High	High	High	High	High	High	1	
FALSE	High	Master Prc	Q9Y5B9	FACT comp	0	55.949	13	15	22	15	1047	119.8	5.66	38.91	15	cell organi	chromosor	protein bir	Pf00557, P	11198	ENSG0000	SUPT16H	14			HIV elonga	24	0.911	0.664842796	8.22	3508.7	3799.7	32.02	6	High	High	High	High	High	High	1	
FALSE	High	Master Prc	P48506	glutamate-	0.003	3.467	2	1	1	1	637	72.7	6.09	1.73	1	cell organi	cytosol	catalytic a	Pf03074	2729	ENSG0000	GCLC	6	Glutathion	Glutathion	Defective (	21	0.911	0.943881737	26.95	714.3	845.1	31.78	4.6	High	High	High	High	High	High	1	
FALSE	High	Master Prc	P05455	Lupus La p	0	71.58	45	16	28	16	408	46.8	7.12	67.99	16	metabolic	cytoplasm;	protein bir	Pf00076, P	6741	ENSG0000	SSB	2	Systemic lu		RNA Polym	5	0.91	0.06674873	0.6	8985.1	9910.9	2.28	5.1	High	High	High	High	High	High	1	
FALSE	High	Master Prc	P13639	Elongation	0	413.268	71	49	263	48	858	95.3	6.83	757.97	49	cell differe	cytoplasm;	catalytic a	Pf00009, P	1938	ENSG0000	EEF2	19	Oxytocin s	Translation	Peptide ch	20	0.91	0.083759645	3.08	76712.7	84010.8	4.27	0.23	High	High	High	High	High	High	1	
FALSE	High	Master Prc	Q9Y3C1	nucleolar p	0	12.52	18	2	3	2	178	21.2	9.94	11.08	2		nucleus	RNA bindi	Pf09420	51491	ENSG0000	NOP16	5				0	0.91	0.201650661	7.33	706.9	830.2	11.98	6.97	High	High	High	High	High	High	1	
FALSE	High	Master Prc	Q14764	major vaul	0	43.794	15	10	12	10	893	99.3	5.48	19.36	10	regulation	cytoplasm;	protein bir	Pf01145, P	9961	ENSG0000	MVP	16			Neutrophil	3	0.91	0.43497677	23.89	1741.8	1913.2	8.34	15.75	High	High	High	High	High	High	1	
FALSE	High	Master Prc	Q9HB71	Calcyclin-b	0	62.841	54	11	18	11	228	26.2	8.25	57.66	11	cell differe	cytoplasm;	protein bir	Pf04969, P	27101	ENSG0000	CACYBP	1	Wnt signal	Gastric car		2	0.91	0.496619514	26.6	7431.4	8162.7	14.78	6.57	High	High	High	High	High	High	1	
FALSE	High	Master Prc	O95816	BAG family	0	49.56	42	10	20	10	211	23.8	6.7	39.39	10	metabolic	cytosol	enzyme re	Pf02179	9532	ENSG0000	BAG2	6	Protein pro		Regulation	5	0.91	0.499270139	15.06	5773	6345.1	6.37	7.73	High	High	High	High	High	High	1	
FALSE	High	Master Prc	Q9H000	Probable E	0.002	3.651	3	1	1	1	416	46.9	7.61	3.16	1	metabolic		catalytic a	Pf00097, P	23609	ENSG0000	MKRN2	3				0	0.91	0.617207985	38.83	430.7	473.2	59.49	11.33	High	High	High	High	High	High	1	
FALSE	High	Master Prc	Q96L12	Calreticulir	0.003	3.116	8	1	1	1	384	45	6.65	4.13	1	cell differe	endoplasm	metal ion l	Pf00262	125972	ENSG0000	CALR3	19				0	0.91	0.75197806	77.2	114.5	125.8	61.22	31.71	High	High	High	High	High	High	1	
FALSE	High	Master Prc	P35998	26S protea	0	116.025	56	22	37	22	433	48.6	5.95	77.09	22	cell differe	cytoplasm;	catalytic a	Pf00004, P	5701	ENSG0000	PSMC2	7	Epstein-Ba	Proteasom	Regulation	153	0.91	0.853292406	1.05	7941.9	8586.9	10	3.9	High	High	High	High	High	High	1	
FALSE	High	Master Prc	Q99832	T-complex	0	241.89	67	33	78	33	543	59.3	7.65	194.07	33	metabolic	cytoplasm;	nucleotide	Pf00118	10574	ENSG0000	CCT7	2			Prefoldin r	9	0.909	0.018220401	0.52	14595	16393.6	3.61	2.09	High	High	High	High	High	High	1	
FALSE	High	Master Prc	O76021	Ribosomal	0	101.614	40	24	45	24	490	54.9	10.13	87.01	24	cell differe	membrane	protein bir	Pf06030, P	26156	ENSG0000	RSL1D1	16		Senescenc		1	0.909	0.021919997	2.98	10795.1	11966.8	3.56	3.28	High	High	High	High	High	High	1	
FALSE	High	Master Prc	P18124	60S riboso	0	82.19	47	15	45	15	248	29.2	10.65	73.41	15	cell organi	cytoplasm;	DNA bindi	Pf00327, P	6129	ENSG0000	RPL7	8	Ribosome	Cytoplasm	Peptide ch	34	0.909	0.027700717	1.01	10784.4	11787.7	7.35	3.72	High	High	High	High	High	High	1	
FALSE	High	Master Prc	O43583	density-reg	0	7.668	15	2	2	2	198	22.1	5.3	6.8	2	cell organi	ribosome	protein bir	Pf01253	8562	ENSG0000	DENR	12				0	0.909	0.03982142	4.54	400.7	441	1.97	1.85	High	High	High	High	High	High	1	
FALSE	High	Master Prc	P49327	Fatty acid	0	449	38	75	158	75	2511	273.3	6.44	369.7	75	cell differe	cytoplasm;	catalytic a	Pf00106, P	2194	ENSG0000	FASN	17	Metabolic	AMP-activ	ChREBP ac	23	0.909	0.091571291	3.23	41109.2	44967.9	8.2	2.59	High	High	High	High	High	High	1	
FALSE	High	Master Prc	P61024	cyclin-depe	0	5.485	20	1	1	1	79	9.7	8.94	4.7	1	cell divisio		catalytic a	Pf01111	1163	ENSG0000	CKS1B	1	Pathways i		Cyclin D as	12	0.909	0.152067859	7.79	197.7	225	8.48	6.13	High	High	High	High	High	High	1	
FALSE	High	Master Prc	Q9P2B4	CTTNBP2 N	0	14.836	5	2	3	2	639	70.1	8.06	6.2	2	metabolic	cytoplasm;	catalytic a	Pf01576, P	55917	ENSG0000	CTTNBP2N	1				0	0.909	0.614421034	87.27	398.5	451.2	83.87	18.37	High	High	High	High	High	High	1	
FALSE	High	Master Prc	Q9H8M2-1	Isoform 5	0.001	4.336	1	1	2	1	501	55.6	5.34	5.48	1	cell organi		protein bir	Pf00439, P	65980		BRD9	5				0	0.909	0.885561145	18.74	1896.9	1957.1	24.22	11.83	High	High	High	High	High	High	1	
FALSE	High	Master Prc	Q14204	Cytoplasm	0	231.375	16	57	77	57	4646	532.1	6.4	182.31	57	cell divisio	cytoplasm;	catalytic a	Pf00769, P	1778	ENSG0000	DYNC1H1	14	Salmonella		COPI-indep	50	0.909	0.890751419	12.09	16867.8	18548.5	22.17	4.33	High	High	High	High	High	High	1	
FALSE	High	Master Prc	P53618	Coatomer	0	114.792	29	20	32	20	953	107.1	6.05	102.28	20	transport	cytoplasm;	protein bir	Pf01602, P	1315	ENSG0000	COPB1	11			COPI-depe	14	0.908	0.001334715	0.61	12976.4	14148.8	4.09	3.62	High	High	High	High	High	High	1	
FALSE	High	Master Prc	Q7L0Y3	Mitochonc	0	50.829	31	10	13	10	403	47.3	9.36	35.53	10	cell organi	mitochond	catalytic a	Pf01746	54931	ENSG0000	TRMT10C	3			tRNA proc	6	0.908	0.266689846	9.81	3069.1	3379	8.04	1.14	High	High	High	High	High	High	1	
FALSE	High	Master Prc	P00966	Argininosu	0	185.992	57	27	79	27	412	46.5	8.02	179.39	27	metabolic	cytoplasm;	catalytic a	Pf00764, P	445	ENSG0000	ASS1	9	Alanine, as	Alanine an	Urea cycle	11	0.907	0.01173267	2.46	22958.6	25693.3	1.7	2.97	High	High	High	High	High	High	1	
FALSE	High	Master Prc	Q9NQT4	Exosome c	0	16.48	14	2	2	2	235	25.2	7.59	11.45	2	defense re	cytoplasm;	catalytic a	Pf01138, P	56915	ENSG0000	EXOSC5	19	RNA degra		Major path	15	0.907	0.020499635	4.07	917.5	982.7	6.24	4.46	High	High	High	High	High	High	1	
FALSE	High	Master Prc	O00767	acyl-CoA d	0	18.504	7	3	7	3	359	41.5	9	18.17	3	metabolic	endoplasm	catalytic a	Pf00487	6319	ENSG0000	SCD	10	Biosynthes	Sterol Reg	Fatty acyl-	18	0.907	0.03942313	2.9	2940.6	3144.6	10.01	11.72	High	High	High	High	High	High	1	
FALSE	High	Master Prc	Q8N3D4	EH domain	0	41.193	8	8	9	7	1523	161.8	4.83	27.66	8		endosome	protein bir	Pf00307, P	254102	ENSG0000	EHBP1L1	11				0	0.907	0.114701216	8.12	924	1021.9	0.62	5.89	High	High	High	High	High	High	1	
FALSE	High	Master Prc	Q96EK5	KIF1-bindir	0	21.365	13	6	8	6	621	71.8	5.49	12.2	6	cell differe	cytoplasm;	protein bir	Pf12309	26128	ENSG0000	KIAA1279;	10				0	0.907	0.135263962	2.29	837.7	1006.7	9.59	5.82	High	High	High	High	High	High	1	
FALSE	High	Master Prc	Q9Y2V2	Calcium-re	0	15.569	31	4	8	4	147	15.9	8.21	10	4	regulation	cytoplasm;	DNA bindi	Pf00313	23589	ENSG0000	CARHSP1	16				0	0.907	0.146142485	8.3	5657.2	6235.1	5.26	3.54	High	High	High	High	High	High	1	
FALSE	High	Master Prc	O00159-1	Unconvent	0	77.693	18	15	22	15	1063	121.6	9.41	49.03	15	cell organi	cytoplasm;	catalytic a	Pf00063, P	4641	ENSG0000	MYO1C	17		Insulin Sig	Translocat	8	0.907	0.187756577	4.59	6779.8	7650.6	8.6	2.22	High	High	High	High	High	High	1	
FALSE	High	Master Prc	Q9NTK5-1	obg-like AT	0	62.12	33	10	20	10	396	44.7	7.81	51.8	10	metabolic	cytoplasm;	catalytic a	Pf01926, P	29789	ENSG0000	OLA1	2			Platelet de	4	0.907	0.306092118	14.21	3064.8	3664.1	15.2	11.73	High	High	High	High	High	High	1	
FALSE	High	Master Prc	Q8WXF1	Paraspeckl	0	40.574	22	9	13	7	523	58.7	6.67	15.33	9	defense re	cytoplasm;	DNA bindi	Pf00076, P	55269	ENSG0000	PSPC1	13				0	0.907	0.66654075	22.84	219.8	207.3	11.8	19.05	High	High	High	High	High	High	1	

FALSE	High	Master Prc	Q9Y5M8	signal reco	0	57.496	37	9	20	9	271	29.7	9.04	48.24	9	regulation	cytoplasm;	nucleotide	Pf00009, P	58477	ENSG0000	SRPRB	3	Protein ex		XBP1(S) ac	7	0.906		0.025316635	2.99	4697.5	5262.9	2.35	1.48	High	High	High	High	High	High	1	
FALSE	High	Master Prc	P08579	U2 small n	0	20.786	16	4	10	3	225	25.5	9.72	19.47	4	metabolic	nucleus;sp	protein bir	Pf00076, P	6629	ENSG0000	SNRPB2	20	Spliceosom	mRNA Pro	mRNA Spli	6	0.906		0.108072079	1.11	1269.2	1414.2	1.87	8.56	High	High	High	High	High	High	1	
FALSE	High	Master Prc	Q15477	Helicase SI	0	14.631	4	4	4	4	1246	137.7	6.06	7.81	4	metabolic	cytoplasm;	catalytic a	Pf00270, P	6499	ENSG0000	SKIV2L	6; CHR_HS	RNA degra		mRNA dec	8	0.906		0.122816304	1.42	1324.5	1516.1	12.61	3.95	High	High	High	High	High	High	1	
FALSE	High	Master Prc	O00232-1	26s protea	0	70.477	31	11	17	11	456	52.9	7.65	56.88	11	metabolic	cytoplasm;	catalytic a	Pf01399	5718	ENSG0000	PSMD12	17	Epstein-Ba	Proteasom	Regulation	153	0.906		0.139614748	10.05	5784.2	6106.8	9.99	3.44	High	High	High	High	High	High	1	
FALSE	High	Master Prc	Q9NY12-1	H/ACA ribo	0	19.577	32	5	7	5	217	22.3	10.92	12.08	5	cell organi	nucleus	protein bir	Pf04410	54433	ENSG0000	GAR1	4	Ribosome		rRNA mod	5	0.906		0.149385463	0.54	1833.1	2000.6	1.29	5.2	High	High	High	High	High	High	1	
FALSE	High	Master Prc	P56556	NADH deh	0	5.67	12	2	3	2	154	17.9	10.14	6.93	2	cell organi	membrane	catalytic a	Pf05347, P	4700	ENSG0000	NDUFA6	22; CHR_H	Metabolic	Electron Tr	Complex I	13	0.906		0.293463233	17.02	1015.8	1147.3	4.99	9.32	High	High	High	High	High	High	1	
FALSE	High	Master Prc	P19338	Nucleolin [	0	370.229	54	59	198	59	710	76.6	4.7	403.77	59	regulation	cytoplasm;	DNA bindi	Pf00076, P	4691	ENSG0000	NCL	2	Pathogenic	Pathogenic	Major path	7	0.906		0.446762056	5.64	71671.6	75455.5	7.17	5.05	High	High	High	High	High	High	1	[S563(100
FALSE	High	Master Prc	Q6P587-1	Acylpyruva	0	6.016	17	2	2	2	224	24.8	7.39	4	2	metabolic	cytoplasm;	catalytic a	Pf01557	81889	ENSG0000	FAHD1	16	Tyrosine m			2	0.906		0.611235136	34.27	199.2	219.8	14.13	12.64	High	High	High	High	High	High	1	
FALSE	High	Master Prc	Q13617-1	Cullin-2 [O	0	13.7	9	6	7	6	745	86.9	6.92	6.75	6	metabolic	cytosol	catalytic a	Pf00888, P	8453	ENSG0000	CUL2	10	Renal cell		Oxygen-de	20	0.906		0.72487696	18.58	1187.7	1375.8	23.77	5.88	High	High	High	High	High	High	1	
FALSE	High	Master Prc	Q99623	Prohibitin-	0	65.537	37	12	32	12	299	33.3	9.83	70.59	12	cell organi	cell surface	protein bir	Pf01145	11331	ENSG0000	PHB2	12			Processing	3	0.905		0.026127178	2.9	9511.2	10868.6	4.47	4.64	High	High	High	High	High	High	1	
FALSE	High	Master Prc	Q9Y2T3	guanine de	0	77.435	41	12	21	12	454	51	5.68	53.1	12	metabolic	cytosol	catalytic a	Pf01979, P	9615	ENSG0000	GDA	9	Purine met		Purine cat	6	0.905		0.08123649	4.12	3439.2	3829.4	7.5	1.05	High	High	High	High	High	High	1	
FALSE	High	Master Prc	Q9NQ88	Fructose-2	0	5.682	6	1	1	1	270	30	7.69	2.48	1	cell death;	cytoplasm;	catalytic a	Pf00300	57103	ENSG0000	C12orf5; T	12	Central car		TP53 Regu	7	0.905		0.117157696	4.22	151.8	176.3	8.31	3.16	High	High	High	High	High	High	1	
FALSE	High	Master Prc	Q15102	Platelet-ac	0	19.824	29	5	7	5	231	25.7	6.84	13.71	5	metabolic	cytoplasm;	catalytic a	Pf00657, P	5050	ENSG0000	PAFAH1B3	19	Metabolic		COPI-indep	7	0.905		0.129299209	3.18	1054.5	1182.8	7.69	3.19	High	High	High	High	High	High	1	
FALSE	High	Master Prc	P78417	Glutathion	0	43.623	39	10	22	10	241	27.5	6.6	29.28	10	cellular ho	cytoplasm;	antioxidan	Pf00043, P	9446	ENSG0000	GSTO1	10	Glutathion	Myometria	Gene and	20	0.905		0.148424647	10.4	5826.6	6441.2	3.13	4.72	High	High	High	High	High	High	1	
FALSE	High	Master Prc	Q13418	Integrin-lir	0	8.92	8	3	3	3	452	51.4	8.07	6.82	3	cell organi	cytoplasm;	catalytic a	Pf00023, P	3611	ENSG0000	ILK	11	Endometri	Integrin-m	Localizatio	15	0.905		0.332303383	13.72	783.5	825.8	5.53	8.92	High	High	High	High	High	High	1	
FALSE	High	Master Prc	Q9H2J4	phosducin-	0	15.006	14	3	4	3	239	27.6	4.84	5.42	3	cell death;	cytoplasm;	catalytic a	Pf02114	79031	ENSG0000	PDCL3	2				0	0.905		0.388928011	22.74	484.5	576.1	17.51	16.97	High	High	High	High	High	High	1	
FALSE	High	Master Prc	Q13596	Sorting ne:	0	39.934	16	7	13	5	522	59	5.15	26.83	7	cell organi	cytoplasm;	protein bir	Pf00787, P	6642	ENSG0000	SNX1	15	Endocytosi			1	0.905		0.463750599	25.24	1384.5	1424	17.56	5.09	High	High	High	High	High	High	1	
FALSE	High	Master Prc	P04114	apolipopro	0.004	2.683	0	1	1	1	4563	515.3	7.05	2.18	1	cell organi	cytoplasm;	protein bir	Pf01347, P	338		APOB	2	Vitamin di	Selenium M	Post-transl	46	0.905		0.509556131	25.89	231.8	261.4	8.29	13.22	High	High	High	High	High	High	1	
FALSE	High	Master Prc	O43491-1	band 4.1-li	0	39.09	11	9	10	9	1005	112.5	5.44	23.01	9	cell divisio	cytoplasm;	protein bir	Pf00373, P	2037	ENSG0000	EPB41L2	6	Tight junct		Neurexins	4	0.905		0.673198918	5.8	2279.2	2460.2	12.61	1.78	High	High	High	High	High	High	1	
FALSE	High	Master Prc	Q8NBZ7-1	UDP-glucu	0	7.559	6	1	2	1	420	47.5	8.94	6.63	1	cell organi	cytoplasm;	catalytic a	Pf01073, P	80146	ENSG0000	UXS1	2	Amino sug			2	0.905		0.818517763	72.39	30	27.4	69.88	21.31	High	High	High	High	High	High	1	
FALSE	High	Master Prc	O95169	NADH deh	0	14.774	30	4	4	4	186	21.8	6.8	10.05	4	cell organi	endoplasm	catalytic a	Pf05821	4714	ENSG0000	NDUFB8	10	Metabolic	Electron Tr	Mitochonc	15	0.905		0.925769083	0.21	559	592.9	3.6	13.44	High	High	High	High	High	High	1	
FALSE	High	Master Prc	Q06210-1	glutamine-	0	115.608	32	16	33	16	699	78.8	7.11	105.52	16	metabolic	cytosol	catalytic a	Pf00310, P	2673	ENSG0000	GFPT1	2	Amino sug		XBP1(S) ac	17	0.904		0.053657284	2.39	6802.6	7592.8	8.15	3.42	High	High	High	High	High	High	1	
FALSE	High	Master Prc	P04406-1	glyceraldel	0	489.219	84	30	391	30	335	36	8.46	1150.36	30	cell death;	cytoplasm;	catalytic a	Pf00044, P	2597	ENSG0000	GAPDH	12	Glycolysis ,	Cori Cycle;	Gluconeog	15	0.904		0.117524628	0.49	119900.5	134791.1	12.25	2.48	High	High	High	High	High	High	1	[S125(100
FALSE	High	Master Prc	Q14684	Ribosomal	0	21.867	8	5	8	5	758	84.4	9.76	12.61	5	metabolic	cytosol;nu	protein bir	Pf05997	23076	ENSG0000	RRP1B	21				0	0.904		0.245310651	15.24	630	696.7	8.61	2.27	High	High	High	High	High	High	1	
FALSE	High	Master Prc	Q9Y3D6	mitochond	0	13.239	22	3	11	3	152	16.9	8.79	21.84	3	cell death;	endoplasm	protein bir	Pf14852, P	51024	ENSG0000	FIS1	7				0	0.904		0.270212051	6.58	3361.6	4047	10.42	16.76	High	High	High	High	High	High	1	
FALSE	High	Master Prc	P78330	phosphose	0	7.634	12	2	2	2	225	25	5.69	4.12	2	metabolic	cytoplasm;	catalytic a	Pf00702, P	5723	ENSG0000	PSPH	7	Carbon me	Trans-sulfu	Serine bios	9	0.904		0.555652586	7.28	836	872.2	8.33	7.29	High	High	High	High	High	High	1	
FALSE	High	Master Prc	P61353	60S riboso	0	37.717	55	8	25	8	136	15.8	10.56	42.41	8	cell organi	cytosol;me	RNA bindi	Pf00467, P	6155	ENSG0000	RPL27	17	Ribosome	Cytoplasm	Peptide ch	34	0.903		0.020882019	1.66	15073.5	17270.1	10.08	10.25	High	High	High	High	High	High	1	
FALSE	High	Master Prc	P68366	Tubulin alp	0	223.338	53	21	139	4	448	49.9	5.06	475.12	21	cell organi	cytoplasm;	catalytic a	Pf00091, P	7277	ENSG0000	TUBA4A	2	Gap juncti	Pathogenic	COPI-indep	76	0.903		0.251318614	13.75	3102.1	3409.9	13.86	2.15	High	High	High	High	High	High	1	[T271(100
FALSE	High	Master Prc	Q9Y4E8	ubiquitin c	0	17.942	5	4	4	4	981	112.3	5.22	11.26	4	cell organi	cytoplasm;	catalytic a	Pf00443, P	9958	ENSG0000	USP15	12			UCH prote	10	0.903		0.289565845	7.25	1095.3	1222.3	10.95	1.85	High	High	High	High	High	High	1	
FALSE	High	Master Prc	P09234	U1 small n	0	29.146	30	4	8	4	159	17.4	9.67	29.44	4	cell organi	nucleus;sp	metal ion l	Pf06220	6631	ENSG0000	SNRPC	6	Spliceosom		mRNA Spli	5	0.903		0.569597046	32.6	2106.9	2052.6	19.5	11.79	High	High	High	High	High	High	1	
FALSE	Low	Master Prc	Q8NFM4	adenylate	0.057	1.136	1	1	1	1	1077	119.7	7.47	2.4	1	metabolic	cytoplasm;	catalytic a	Pf00211, P	196883	ENSG0000	ADCY4	14	GABAergic	Myometria	Adenylate	78	0.903		0.589734664	45.5	133	141.1	67.04	11.46	High	High	High	High	High	High	1	
FALSE	Low	Master Prc	Q9NVR2	Integrator	0.057	1.136	1	1	1	1	710	82.2	7.44	0	1	metabolic	nucleus	protein bir		55174	ENSG0000	INTS10	8			RNA polym	3	0.903		0.894182177	24.52	92.2	102.8	22.13	7.84	High	High	High	High	High	High	1	
FALSE	High	Master Prc	Q14697-1	Neutral alp	0	200.716	38	33	100	1	944	106.8	6.14	257.85	33	metabolic	endoplasm	catalytic a	Pf01055, P	23193	ENSG0000	GANAB	11	N-Glycan b		Calnexin/c	8	0.902		0.039522859	3.55	1462.2	1577.7	4.59	2.27	High	High	High	High	High	High	1	
FALSE	High	Master Prc	P40429	60S riboso	0	61.622	50	14	42	14	203	23.6	10.93	60.98	14	cell organi	cytoplasm;	RNA bindi	Pf00572	23521	ENSG0000	RPL13A	19	Ribosome	Cytoplasm	Peptide ch	34	0.902		0.040760631	2.02	16708.5	18529.4	4.01	7.34	High	High	High	High	High	High	1	
FALSE	High	Master Prc	P09488	Glutathion	0	25.538	34	7	9	7	218	25.7	6.7	16.83	7	metabolic	cytoplasm;	catalytic a	Pf00043, P	2944	ENSG0000	GSTM1	1	Platinum d	Glutathion	Glutathion	15	0.902		0.055234041	2.38	3303.9	3844	6.5	3.47	High	High	High	High	High	High	1	
FALSE	High	Master Prc	A0AV96	RNA-bindi	0	11.956	7	3	3	3	593	64.1	7.68	5.63	3	cell differe	nucleus	RNA bindi	Pf00076, P	54502	ENSG0000	RBM47	4		TYROBP Ca		1	0.902		0.074417697	7.75	482.4	530	4.81	4.44	High	High	High	High	High	High	1	
FALSE	High	Master Prc	Q969X6	U3 small n	0	5.314	2	1	1	1	686	76.8	8.85	4.55	1	metabolic	chromosor	protein bir	Pf00400, P	84916	ENSG0000	CIRH1A; U	16	Ribosome		Major path	6	0.902		0.088518306	2.21	76.5	84.7	13.52	17.38	High	High	High	High	High	High	1	
FALSE	High	Master Prc	Q9H1B7	interferon	0	15.244	4	3	5	1	796	82.6	8.24	7.71	3	regulation	nucleus	metal ion l	Pf11261	64207	ENSG0000	IRF2BPL	14				0	0.902		0.092906861	8.42	126.3	151.2	20.48	15.33	High	High	High	High	High	High	1	
FALSE	High	Master Prc	P16989-2	Isoform 2 c	0	92.827	45	10	27	5	303	31.9	9.66	74.28	10	metabolic	cytoplasm;	DNA bindi	Pf00313	8531	ENSG0000	CSDA; YBX	12		RalA down		2	0.902		0.093535026	8	1749.8	2064.4	15	12.59	High	High	High	High	High	High	1	
FALSE	High	Master Prc	P35606	Coatomer	0	131.982	28	20	40	20	906	102.4	5.27	130.75	20	transport	cytoplasm;	protein bir	Pf00400, P	9276	ENSG0000	COPB2	3			COPI-depe	11	0.902		0.221499825	13.56	13384.2	15018.9	7.77	6.14	High	High	High	High	High	High	1	
FALSE	High	Master Prc	O75446	Histone de	0.002	3.954	5	1	1	1	220	23.3	9.17	2.36	1	cell differe	nucleus	catalytic a	Pf13866, P	8819	ENSG0000	SAP30	4		Retinoblas	HDACs dea	9	0.902		0.356696307	8.22	37.2	40.9	6.66	4.1	High	High	High	High	High	High	1	
FALSE	High	Master Prc	P55010	Eukaryotic	0	37.24	21	8	15	8	431	49.2	5.58	34.88	8	cell organi	cytoplasm;	catalytic a	Pf01873, P	1983	ENSG0000	EIF5	14	RNA trans	Translation	GTP hydro	8	0.901		0.035353132	1.71	7185.5	7885	4.57	7.49	High	High	High	High	High	High	1	
FALSE	High	Master Prc	P60981-1	Destrin [OS	0	50.578	59	10	36	9	165	18.5	7.85	63.16	10	cell organi	cytoplasm;	protein bir	Pf00241	11034	ENSG0000	DSTN	20		Androgen		1	0.901		0.120530894	3.87	4045.1	4380.7	12.34	2.83	High	High	High	High	High	High	1	
FALSE	High	Master Prc	Q9NRF8	CTP syntha	0	16.91	10	5	5	4	586	65.6	6.9	7.49	5	metabolic	cytosol	catalytic a	Pf00117, P	56474	ENSG0000	CTPS2	X	Pyrimidine		Interconve	5	0.901		0.178257574	9.29	748.9	800.7	16.54	3.84	High	High	High	High	High	High	1	
FALSE	High	Master Prc	O60762	Dolichol-pl	0	14.912	23	5	7	5	260	29.6	9.57	7.69	5	metabolic	endoplasm	catalytic a	Pf00535, P	8813	ENSG0000	DPM1	20	N-Glycan b		Defective I	13	0.901		0.233911068	1.51	904.6	1003.7	22.59	5.48	High	High	High	High	High	High	1	
FALSE	High	Master Prc	O95347-1	structural i	0	22.189	5	6	7	6	1197	135.6	8.43	15.38	6	cell divisio	chromosor	catalytic a	Pf01576, P	10592	ENSG0000	SMC2	9		Retinoblas	Condensat	8	0.901		0.650125712	30.38	1644.5	1825.5	49.23	13.76	High	High	High	High	High	High	1	
FALSE	High	Master Prc	P26641	elongation	0	182.084	55	25	84	25	437	50.1	6.67	208.84	25	metabolic	cytoplasm;	catalytic a	Pf00043, P	1937	ENSG0000	EEF1G	11	Legionello:	Translation	Eukaryotic	5	0.9		0.021558502	1.38	19707.1	22094.2	5.25	2.59	High	High	High	High	High	High	1	
FALSE	High	Master Prc	P35269	General tra	0	21.409	13	5	6	5	517	58.2	7.49	14.81	5	metabolic	nucleus	catalytic a	Pf05793	2962	ENSG0000	GTF2F1	19	Basal trans		Signaling b	55	0.9		0.028501977	3.34	1407.2	1564	10.11	5.89	High	High	High	High	High	High	1	
FALSE	High	Master Prc	P35268	60S riboso	0	37.348	51	5	15	5	128	14.8	9.19	25.71	5	cell organi	cytoplasm;	protein bir	Pf01776	6146	ENSG0000	RPL22	1	Ribosome	Cytoplasm	Peptide ch	34	0.9		0.037859688	1.03	4008.4	4421.7	7.79	10.29	High	High	High	High	High	High	1	
FALSE	High	Master Prc	Q9BZF1-1	Oxysterol-l	0	21.072	6	3	3	3	889	101.1	6.96	7.25	3	cell differe	cytosol;en	transporte	Pf00169, P	114882	ENSG0000	OSBPL8	12			Acyl chain	5	0.9		0.280484167	1.27	373.7	399.6	3.33	5.53	High	High	High	High	High	High	1	
FALSE	High	Master Prc	O60524	nuclear ex	0	8.629	3	3	3	3	1076	122.9	6.35	3.22	3	transport	nucleus		Pf05670, P	9147	ENSG0000	NEMF	14				0	0.9		0.371215077	21.71	1143.8	1274.2	18.65	11.95	High	High	High	High	High	High	1	
FALSE	High	Master Prc	Q5VT79	annexin A8	0	74.606	40	12	26	1	327	36.9	5.78	67.27	12			metal ion l	Pf00191	728113	ENSG0000	ANXA8L1	10				0	0.9		0.666949182	55.2	454	504.6	29.2	23.95	High	High	High	High	High	High	1	
FALSE	High	Master Prc	P04004	Vitronectir	0	10.514	5	2	2	2	478	54.3	5.8	3.13	2	cell differe	cytoplasm;	protein bir	Pf00045, P	7448	ENSG0000	VTN	17	ECM-recep	Primary Fc	ECM prote	21	0.9		0.851327983	22.74	1173.8	1304.8	13.98	12.34	High	High	High	High	High	High	1	
FALSE	High	Master Prc	Q8IWZ8-1	SURP and	0	5.476	4	2	2	2	645	72.4	7.61	4.06	2	metabolic	nucleus;sp	protein bir	Pf01585, P	57794	ENSG0000	SUGP1	19		mRNA Pro	mRNA Spli	5	0.899		0.015143893	2.2	376	407.9	2.78	3.86	High	High	High	High	High	High	1	
FALSE	High	Master Prc	P62701	40S riboso	0	121.994	61	22	53	22	263	29.6	10.15	113.86	22	cell organi	cytoplasm;	protein bir	Pf00900, P	6191	ENSG0000	RPS4X	X	Ribosome	Cytoplasm	Peptide ch	38	0.899		0.147414137	2.46	25705.8	28581.1	2.06	7.82	High	High	High	High	High	High	1	
FALSE	High	Master Prc	P62158	Calmodulir	0	57.692	33	7	35	7	149	16.8	4.22	84.27	7	metabolic	cytoplasm;	catalytic a	Pf00036, P	808; 805;	ENSG0000	CALM3; CA	19; 2; 14	Dopamine	Physiologic	Smooth M	150	0.899		0.307007127	19.57	9358.8	11016.2	11.45	13.16	High	High	High	High	High	High	1	
FALSE	High	Master Prc	Q13765-1	Nascent pc	0	72.797	31	6	26	6	215	23.4	4.56	77.11	6	metabolic	cytoplasm;	DNA bindi	Pf01849	4666	ENSG0000	NACA	12				0	0.899		0.416403529	26.86	7428.1	7806.7	29.75	11.93	High	High	High	High	High	High	1	
FALSE	High	Master Prc	O14974	Protein ph	0	39.054	10	9	11	9	1030	115.2	5.4	23.73	9	cell organi	chromosor	enzyme re	Pf00023, P	4659	ENSG0000	PPP1R12A	12	Vascular sr	Focal Adhe	RHO GTPa	22	0.899		0.676471561	7.09	639.5	725.6	38.05	2.11	High	High	High	High	High	High	1	
FALSE	High	Master Prc	Q9H2U2	Inorganic p	0	34.017	31	8	13	8	334	37.9	7.39	31.39	8	metabolic	cytoplasm;	catalytic a	Pf00719	27068	ENSG0000	PPA2	4	Oxidative p		Mitochonc	7	0.898		0.148552873	3.06	2238.7	2492.2	8.36	4.92	High	High	High	High	High	High	1	
FALSE	High	Master Prc	P61086	Ubiquitin-c	0	12.557	17	2	6	2	200	22.4	5.44	20.28	2	cell organi	cytoplasm;	catalytic a	Pf00179, P	3093	ENSG0000	UBE2K	4	Ubiquitin r		Synthesis c	12	0.898		0.240741615	10.25	1734.8	2060.5	6.58	14.11	High	High	High	High	High	High	1	
FALSE	High	Master Prc	O00629	Importin su	0	29.98	17	5	7	3	521	57.9	4.96	27.87	5	regulation	cytoplasm;	protein bir	Pf00514, P	3840	ENSG0000	KPNA4	3		Leptin sign	NS1 Media	11	0.898		0.253037486	11.1	471.5	549.2	15.98	7.89	High	High	High	High	High	High	1	
FALSE	High	Master Prc	P46977	Dolichyl-di	0	29.81	9	7	12	7	705	80.5	8.07	25.38	7	metabolic	endoplasm	catalytic a	Pf02516	3703	ENSG0000	STT3A	11	Protein pr		Asparagine	6	0.898		0.564792211	0.33	3370.7	3463.2	7.96	5.95	High	High	High	High	High	High	1	
FALSE	High	Master Prc	P11171-1	protein 4.1	0.001	4.478	2	1	1	1	864	97	5.58	2.26	1	cell divisio	cytoplasm;	protein bir	Pf00373, P	2035	ENSG0000	EPB41	1	Tight junct		Neurexins	4	0.898		0.843665188	10.53	753.1	802	15.13	5.75	High	High	High	High	High	High	1	
FALSE	High	Master Prc	P51965-1	Ubiquitin-c	0	20.19	12	2	4	2	193	21.4	8.53	6.16	2	cell organi	cytosol;nu	catalytic a	Pf00179	7324	ENSG0000	UBE2E1	3	Ubiquitin r	Photodyna	Inactivatio	43	0.898		0.918078331	27.15	583.7	650	30.58	3.51	High	High	High	High	High	High	1	
FALSE	High	Master Prc	Q01813	ATP-depen	0	113.367	36	23	40	20	784	85.5	7.55	89.74	23	metabolic	cytoplasm;	catalytic a	Pf00365	5214	ENSG0000	PFKP	10	Galactose	Cori Cycle;	Glycolysis	17	0.897		0.005924795	1	8607	9413.8	4.13	4.11	High	High	High	High	High	High	1	
FALSE	High	Master Prc	Q99436	Proteasom	0	62.25	49	11	25	11	277	29.9	7.68	62.1	11	metabolic	cytoplasm;	catalytic a	Pf00227, P	5695	ENSG0000	PSMB7	9	Proteasom	Proteasom	Regulation	151	0.897		0.021821521	2.16	8258.2	9420.8	4.66	6.33	High	High	High	High	High	High	1	
FALSE	High	Master Prc	P07195	L-lactate d	0	203.261	57	18	95	17	334	36.6	6.05	233.31	18	metabolic	cytoplasm;	catalytic a	Pf00056, P	3945	ENSG0000	LDHB	12	Pyruvate n	Glycolysis	Pyruvate n	11	0.897		0.053328223	6.02	30972.1	34988.5	4.85	0.85	High	High	High	High	High	High	1	
FALSE	High	Master Prc	P41227-1	N-alpha-ac	0	38.955	20	5	12	5	235	26.4	5.64	33.95	5	cell organi	cytoplasm;	catalytic a	Pf00583, P	8260	ENSG0000	NAA10	X				0	0.897		0.065226122	3.04	2687	2969	9.36	7.87	High	High	High	High	High	High	1	
FALSE	High	Master Prc	Q9Y6M1	Insulin-like	0	14.496	7	4	4	3	599	66.1	8.46	10.38	4	regulation	cytoplasm;	protein bir	Pf00013, P	10644	ENSG0000	IGF2BP2	3			Insulin-like	2	0.897		0.148555537	7.58	581.4	647.8	2.62	9.33	High	High	High	High	High	High	1	
FALSE	High	Master Prc	Q9NP79	Vacuolar p	0	30.848	21	4	7	4	307	33.9	6.29	25.42	4	cell commi	cytoplasm;	protein bir	Pf04652	51534	ENSG0000	VTA1	6	Endocytosi		Budding ar	10	0.897		0.178970736	13.58	2511	2735.4	7.28	3.38	High	High	High	High	High	High	1	
FALSE	High	Master Prc	P53396-1	ATP-citrate	0	210.549	35	32	60	32	1101	120.8	7.33	159.14	32	metabolic	cytoplasm;	catalytic a	Pf00285, P	47	ENSG0000	ACLY	17	Metabolic	Sterol Reg	ChREBP ac	15	0.897		0.209852303	12.72	18247.9	20348.7	6.73	5.79	High	High	High	High	High	High	1	
FALSE	High	Master Prc	Q7Z5G4-1	Golgin sub	0.003	2.968	11	1	1	1	137	15.8	7.05	3.92	1	cell organi	extracellul	catalytic a	Pf10256	51125	ENSG0000	GOLGA7	8			Neutrophil	3	0.897		0.215671805	14.28	382.2	418.4	2.06	9.46	High	High	High	High	High	High	1	
FALSE	High	Master Prc	Q9BQ39	ATP-depen	0	50.558	11	6	15	5	737	82.5	9.17	46.52	6	metabolic	cytoplasm;	catalytic a	Pf00270, P	79009	ENSG0000	DDX50	10				0	0.897		0.278445636	11.4	1601.8	1966.9	10.11	16.78	High	High	High	High	High	High	1	
FALSE	High	Master Prc	Q14241	Elongin-A		0	7.423	2	1	1	1	798	89.9	9.57	2.98	1	metabolic	nucleus	DNA bindi	Pf06881, P	6924	ENSG0000	TCEB3; EL(	1		VEGFA-VE(	HIV elonga	23	0.897		0.530484	25.49	61.2	68.2	14.21	5.75	High	High	High	High	High	High	1	
FALSE	Low	Master Prc	Q9BZF9	Uveal auto	0.064	1.087	0	1	1	1	1416	162.4	7.03	0	1	metabolic	cytoplasm;	protein bir	Pf00023, P	55075	ENSG0000	UACA	15				0	0.897		0.646430541	7.5	1128.4	1289.8	15.76	4.3	High	High	High	High	High	High	1	
FALSE	High	Master Prc	P23921	Ribonuclec	0	188.526	42	25	47	25	792	90	7.15	162.2	25	metabolic	cytoplasm;	catalytic a	Pf00317, P	6240	ENSG0000	RRM1	11	Glutathion	Fluoropyrii	Interconve	10	0.896		0.006914966	1.31	14881.1	16850.6	2.56	3.48	High	High	High	High	High	High	1	
FALSE	High	Master Prc	Q13813	Spectrin al	0	244.781	24	44	71	44	2472	284.4	5.35	168.57	44	cell organi	cytoplasm;	metal ion l	Pf00018, P	6709	ENSG0000	SPTAN1	9	Tight junct	Fas Ligand	Interactior	33	0.896		0.114842506	10.27	18822.4	21770	4.32	6.56	High	High	High	High	High	High	1	
FALSE	Medium	Master Prc	P48995-1	Short trans	0.016	1.697	1	1	1	1	793	91.2	8.05	0	1	cellular ho	membrane	protein bir	Pf00520, P	7220	ENSG0000	TRPC1	3	Axon guid	Cell-type D	Ion homeo	21	0.896		0.161507103	8.84	485.4	541.5	20.59	6.77	High	High	High	High	High	High	1	
FALSE	High	Master Prc	Q9Y5L0-1	Isoform 1 c	0	10.218	3	3	3	3	957	107.9	5.71	7.85	3	transport	cytoplasm;	protein bir	Pf08389	23534		TNPO3	7				0	0.896		0.544648501	6.87	744.3	788.3	13.23	3.75	High	High	High	High	High	High	1	

FALSE	High	Master Prc	P21291	Cysteine ar	0	97.343	67	9	29	9	193	20.6	8.57	105.9	9		nucleus	metal ion l	Pf00412	1465	ENSG0000	CSRP1	1		Mecp2 anc	MTF1 activ	4	0.896	0.99138503	8.55	10484.9	11710.7	25.46	9.82	High	High	High	High	High	High	1
FALSE	High	Master Prc	P10155-1	60 kDa SS-	0	16.757	9	4	5	4	538	60.6	8.03	7.44	4	cell organi	cytoplasm;	metal ion l	Pf05731, P	6738	ENSG0000	TROVE2	1	Systemic lu			1	0.895	0.022865705	3.4	280.8	325.9	7.38	6.51	High	High	High	High	High	High	1
FALSE	High	Master Prc	P49321	Nuclear au	0	189.936	45	28	56	28	788	85.2	4.3	150.25	28	cell organi	cytoplasm;	protein bir	Pf00515, P	4678	ENSG0000	NASP	1				0	0.895	0.02999053	3.83	11271	12596.6	6.08	2.82	High	High	High	High	High	High	1
FALSE	High	Master Prc	O14929	histone ac	0	21.167	18	5	7	5	419	49.5	5.69	14.69	5	cell organi	cytoplasm;	catalytic a	Pf10394	8520		HAT1	2	Alcoholism		HATs acety	4	0.895	0.032406411	0.05	1146.8	1335	2.99	4.4	High	High	High	High	High	High	1
FALSE	High	Master Prc	P05198	Eukaryotic	0	61.209	42	11	23	11	315	36.1	5.08	50.11	11	cell commu	cytoplasm;	protein bir	Pf00575, P	1965	ENSG0000	EIF2S1	14	Herpes sim	Translatior	Formation	25	0.895	0.121982893	7.68	5983.4	6539.4	1.68	6.05	High	High	High	High	High	High	1
FALSE	High	Master Prc	Q15008	26S protea	0	35.16	23	7	13	7	389	45.5	5.62	42.07	7	metabolic	cytosol;ext	catalytic a	Pf01399, P	9861	ENSG0000	PSMD6	3	Epstein-Ba	Proteasom	Regulation	153	0.895	0.169122783	11.62	4310.4	5041.9	6.76	7.37	High	High	High	High	High	High	1
FALSE	High	Master Prc	P07355	Annexin A2	0	294.477	83	38	161	38	339	38.6	7.75	394.61	38	cell organi	cell surface	enzyme re	Pf00191	302	ENSG0000	ANXA2	15		Prostaglan	Dissolution	12	0.895	0.264519036	18.3	55761.2	57348.8	12.12	7.81	High	High	High	High	High	High	1
FALSE	High	Master Prc	Q2M2I8-1	AP2-associ	0	9.076	4	2	2	2	961	103.8	6.6	4.05	2	cell organi	cytoplasm;	catalytic a	Pf00069, P	22848	ENSG0000	AAK1	2			Cargo reco	4	0.895	0.275442354	2.45	270.4	294.3	7.21	11.72	High	High	High	High	High	High	1
FALSE	High	Master Prc	P30484	HLA class I	0	26.336	20	6	17	2	362	40.4	6.62	24.61	6	regulation	cell surface	protein bir	Pf00129, P	3106		HLA-B	6		Proteasom	Interferon	15	0.895	0.313444546	5.19	137.7	190.5	25.14	26.85	High	High	High	High	High	High	1
FALSE	High	Master Prc	P62847-1	40S riboso	0	36.827	31	5	17	5	133	15.4	10.78	55.88	5	cell organi	cytosol;me	protein bir	Pf01282	6229	ENSG0000	RPS24	10	Ribosome	Cytoplasm	Peptide ch	38	0.895	0.593570341	9.58	6433.9	7189.3	8.1	7.68	High	High	High	High	High	High	1
FALSE	High	Master Prc	Q9NQ92	Coordinato	0	4.672	7	1	3	1	184	20.1	4.18	2.64	1	cell organi	cytosol;me	protein bir	Pf15340	55352	ENSG0000	COPRS	17			RMTs metl	3	0.895	0.894728374	49.21	611.7	683.2	42.62	3.54	High	High	High	High	High	High	1
FALSE	High	Master Prc	P09960-1	leukotriene	0	68.848	25	12	20	12	611	69.2	6.18	54.76	12	metabolic	cytoplasm;	catalytic a	Pf01433, P	4048	ENSG0000	LTA4H	12	Metabolic	Eicosanoid	Synthesis c	11	0.894	0.028804927	4.74	6244.6	6926.4	2.13	2.57	High	High	High	High	High	High	1
FALSE	High	Master Prc	P62888	60S riboso	0	87.388	67	7	50	7	115	12.8	9.63	122.8	7	cell organi	cytosol;me	protein bir	Pf01248	6156	ENSG0000	RPL30	8	Ribosome	Selenium M	Peptide ch	35	0.894	0.054842452	5.25	15415.9	17245.9	1.02	5.24	High	High	High	High	High	High	1
FALSE	High	Master Prc	Q6IN84-1	rRNA meth	0	8.95	9	3	4	3	353	38.6	7.94	8.4	3	metabolic	mitochond	catalytic a	Pf00588, P	79922	ENSG0000	MRM1	17; CHR_H			rRNA mod	4	0.894	0.282131814	16.03	381.6	426.9	7.22	11.78	High	High	High	High	High	High	1
FALSE	High	Master Prc	P49841-1	Glycogen s	0	15.225	9	2	3	2	420	46.7	8.78	8.16	2	cell differe	cytoplasm;	catalytic a	Pf00069, P	2932	ENSG0000	GSK3B	3	Dopamine	IL-5 Signali	Regulation	124	0.894	0.392711734	24.16	634.6	710.2	11.81	10.32	High	High	High	High	High	High	1
FALSE	High	Master Prc	Q14914	Prostaglan	0	9.54	7	1	1	1	329	35.8	8.29	6.41	1	metabolic	cytoplasm	catalytic a	Pf00107	22949	ENSG0000	PTGR1	9		NRF2 path	Synthesis c	7	0.894	0.551068341	26.54	474	536.5	0.67	21.45	High	High	High	High	High	High	1
FALSE	High	Master Prc	Q9BVI4	Nucleolar c	0	6.771	4	1	1	1	516	58.4	7.49	4.74	1	metabolic	membrane	protein bir	Pf03914, P	79050	ENSG0000	NOC4L	12			Major path	5	0.894	0.68088412	56.13	48.4	54.1	29.12	23.26	High	High	High	High	High	High	1
FALSE	High	Master Prc	P09661	U2 small n	0	47.761	38	9	17	9	255	28.4	8.62	30.49	9	metabolic	nucleus;sp	protein bir	Pf12799, P	6627	ENSG0000	SNRPA1	15	Spliceosom	mRNA Pro	Gene and p	11	0.893	0.014369005	1.57	3178	3507.2	5.32	6.63	High	High	High	High	High	High	1
FALSE	High	Master Prc	Q9H9J2	39S riboso	0	16.022	13	3	4	3	332	37.5	8.4	15.04	3	cell organi	membrane	catalytic a	Pf00636	65080	ENSG0000	MRPL44	2			Mitochonc	6	0.893	0.070606808	6.96	1176.9	1249.1	4.29	6.79	High	High	High	High	High	High	1
FALSE	High	Master Prc	P32119	Peroxiredo	0	63.507	51	11	37	10	198	21.9	5.97	80.08	11	cell commu	cytoplasm;	antioxidan	Pf00578, P	7001	ENSG0000	PRDX2	19		Selenium M	TP53 Regu	12	0.893	0.073414428	2.36	9503.8	11150.5	5.06	4.24	High	High	High	High	High	High	1
FALSE	High	Master Prc	Q9Y3B8-1	Oligoribon	0	42.906	46	9	11	9	237	26.8	6.87	25.19	9	metabolic	cytoplasm;	catalytic a	Pf00929	25996	ENSG0000	REXO2	11	Ribosome			1	0.893	0.075031077	1.64	4558.2	4949.4	2.4	3.65	High	High	High	High	High	High	1
FALSE	High	Master Prc	P49770	Translatior	0	8.531	11	3	3	3	351	39	6.16	5.76	3	metabolic	cytoplasm;	nucleotide	Pf01008	8892	ENSG0000	EIF2B2	14	RNA transp	Translatior	Recycling c	8	0.893	0.125507066	7.66	214	228.6	6.07	10.99	High	High	High	High	High	High	1
FALSE	Medium	Master Prc	P61803	Dolichyl-di	0.034	1.374	9	1	1	1	113	12.5	7.08	0	1	cell death;	endoplasm	catalytic a	Pf02109	1603	ENSG0000	DAD1	14	Protein pro		Asparagine	6	0.893	0.143872784	4.61	161	180.3	3.32	8.49	High	High	High	High	High	High	1
FALSE	High	Master Prc	Q9Y5B0	RNA polym	0	7.287	3	3	4	3	961	104.3	5.27	4.94	3	cell divisio	cytoplasm;	catalytic a	Pf00533, P	9150	ENSG0000	CTDP1	18			HIV elonga	25	0.893	0.933666341	5.05	578.9	597.9	13.45	8.83	High	High	High	High	High	High	1
FALSE	High	Master Prc	Q9UHR4	Brain-spec	0	14.874	10	4	4	4	511	56.8	8.68	7.01	4	cell organi	cytoplasm;	protein bir	Pf00018, P	55971	ENSG0000	BAIAP2L1	7				0	0.892	0.058882701	1.33	907.7	1017.7	5.65	9.07	High	High	High	High	High	High	1
FALSE	High	Master Prc	P62750	60S riboso	0	65.561	54	11	46	11	156	17.7	10.45	91.51	11	cell organi	cytoplasm;	protein bir	Pf00276, P	6147	ENSG0000	RPL23A	17	Ribosome	Cytoplasm	Peptide ch	34	0.892	0.095803911	2	12235.8	13426.6	4.98	1.29	High	High	High	High	High	High	1
FALSE	High	Master Prc	P52943	Cysteine-ri	0	24.509	25	2	3	2	208	22.5	8.72	15.38	2	regulation		metal ion l	Pf00412	1397	ENSG0000	CRIP2	14				0	0.892	0.11447801	9.1	759.6	806.9	4.01	9.42	High	High	High	High	High	High	1
FALSE	High	Master Prc	Q92979	Ribosomal	0	18.666	18	3	3	3	244	26.7	9.17	8.85	3	metabolic	cytoplasm;	catalytic a	Pf03587	10436	ENSG0000	EMG1	12	Ribosome		Major path	6	0.892	0.246486268	14.29	756.4	909.6	11.55	10.13	High	High	High	High	High	High	1
FALSE	High	Master Prc	P62280	40S riboso	0	48.295	53	10	28	10	158	18.4	10.3	64.51	10	cell differe	cytoplasm;	protein bir	Pf00366	6205	ENSG0000	RPS11	19	Ribosome	Cytoplasm	Peptide ch	38	0.892	0.324439067	13.24	12213.8	13700.2	1.91	10.49	High	High	High	High	High	High	1
FALSE	High	Master Prc	Q9H7B2	Ribosome	0	7.676	6	2	3	2	306	35.6	9.99	4.26	2	cell organi	nucleus	RNA bindir	Pf04427	84154	ENSG0000	RPF2	6				0	0.892	0.482041719	11.9	176.9	198.3	10.88	4.73	High	High	High	High	High	High	1
FALSE	High	Master Prc	P47755	F-actin-cap	0	38.499	40	8	12	6	286	32.9	5.85	26.19	8	cell organi	cytoskeleto	protein bir	Pf01267	830	ENSG0000	CAPZA2	7	Endocytosi		COPI-indep	22	0.892	0.505988609	17.01	699	768	7.82	12.79	High	High	High	High	High	High	1
FALSE	High	Master Prc	Q16740	ATP-depen	0	25.456	27	4	5	4	277	30.2	8.09	22.54	4	cell organi	mitochond	catalytic a	Pf00574	8192	ENSG0000	CLPP	19				0	0.892	0.567207942	36.3	2209.9	2460.2	17.65	10.88	High	High	High	High	High	High	1
FALSE	High	Master Prc	P62244	40S riboso	0	29.676	61	9	12	9	130	14.8	10.13	21.14	9	cell organi	cytoplasm;	protein bir	Pf00410	6210	ENSG0000	RPS15A	16	Ribosome	Cytoplasm	Peptide ch	38	0.891	0.019713999	1.59	5950.8	6608.3	15.5	10.5	High	High	High	High	High	High	1
FALSE	High	Master Prc	Q9BY44-1	Eukaryotic	0	37.661	26	11	16	11	585	64.9	8.87	44.03	11	cell organi	cytoplasm	protein bir	Pf08662	83939	ENSG0000	EIF2A	3		Photodyna		2	0.891	0.045840124	5.05	3802.1	4154.5	5.86	5.77	High	High	High	High	High	High	1
FALSE	High	Master Prc	P83916	Chromobo	0	27.55	30	4	7	3	185	21.4	4.93	13.06	4	regulation	chromosor	protein bir	Pf00385, P	10951	ENSG0000	CBX1	17				0	0.891	0.071728653	7.91	22.8	24.2	17.17	15.11	High	High	High	High	High	High	1
FALSE	High	Master Prc	O00762	Ubiquitin-c	0	14.896	23	3	5	3	179	19.6	7.37	10.26	3	cell divisio	cytoplasm;	catalytic a	Pf00179, P	11065	ENSG0000	UBE2C	20	Ubiquitin r	Gastric Car	Inactivatio	39	0.891	0.103072313	8.13	2231.7	2436.2	4.67	4.02	High	High	High	High	High	High	1
FALSE	High	Master Prc	P49458	Signal reco	0	20.798	40	4	9	4	86	10.1	7.97	19.48	4	cell organi	cytoplasm;	protein bir	Pf05486	6726; 653:	ENSG0000	SRP9; SRP!	1; 10	Protein ex		SRP-depen	4	0.891	0.281753346	19.51	4864.3	4873.8	17.83	7.96	High	High	High	High	High	High	1
FALSE	High	Master Prc	Q9NR12-1	PDZ and LI	0	10.178	7	3	6	3	457	49.8	8.41	11.54	3	cell differe	cytoplasm;	metal ion l	Pf00412, P	9260	ENSG0000	PDLIM7	5		EBV LMP1	RET signali	4	0.891	0.957789027	26.86	1734.4	1946.9	25.85	5.41	High	High	High	High	High	High	1
FALSE	High	Master Prc	Q96FQ6	Protein S1(	0	16.291	52	4	5	4	103	11.8	6.79	14.21	4	response t	cytoplasm;	metal ion l	Pf01023, P	140576	ENSG0000	S100A16	1				0	0.891	0.970956445	21.46	1185.6	1384.2	32.97	6.02	High	High	High	High	High	High	1
FALSE	High	Master Prc	P28074-1	proteasom	0	73.049	48	11	24	11	263	28.5	6.92	56.15	11	metabolic	cytoplasm;	catalytic a	Pf00227	5693	ENSG0000	PSMB5	14	Proteasom	Proteasom	Regulation	150	0.89	0.021268579	1.74	8701.9	9775.4	6.68	2.72	High	High	High	High	High	High	1
FALSE	High	Master Prc	Q05209-1	Tyrosine-p	0	19.896	9	5	5	5	780	88.1	5.62	11.73	5	metabolic	cytoplasm;	catalytic a	Pf00102, P	5782	ENSG0000	PTPN12	7		EGF/EGFR	Downregu	9	0.89	0.052760921	1.15	204.3	229.5	5.61	1.53	High	High	High	High	High	High	1
FALSE	High	Master Prc	Q15257-2	Isoform 1 c	0	8.097	8	3	6	3	323	36.8	6.29	7.21	3	cell organi	cytoplasm;	catalytic a	Pf03095	5524	ENSG0000	PPP2R4; P	9		Interleukin		5	0.89	0.131073661	4.97	2153.4	2335.8	9.8	8.47	High	High	High	High	High	High	1
FALSE	High	Master Prc	Q15717	ELAV-like p	0	41.67	32	8	16	8	326	36.1	9.17	40.56	8	developme	cytoplasm;	protein bir	Pf00076, P	1994	ENSG0000	ELAVL1	19	AMPK sign	AMP-activ	HuR (ELAV	11	0.89	0.138925888	6.6	6879.8	7734	8.9	3.37	High	High	High	High	High	High	1
FALSE	High	Master Prc	Q5JVF3	PCI domair	0	10.055	7	3	3	3	399	46	8.53	2.89	3	cell organi		DNA bindir	Pf01399	55795	ENSG0000	PCID2	13				0	0.89	0.360195458	22.11	642.3	794.4	17.91	6.28	High	High	High	High	High	High	1
FALSE	High	Master Prc	Q13671	ras and Ra	0	11.838	4	2	2	2	783	84	8.02	6.05	2	regulation	cytoplasm;	enzyme re	Pf00788, P	9610	ENSG0000	RIN1	11	Ras signali	EGF/EGFR	RAB GEFs e	6	0.889	0.004677563	1.43	304.5	339.1	6.39	6.61	High	High	High	High	High	High	1
FALSE	High	Master Prc	O00299	chloride in	0	113.636	65	11	38	11	241	26.9	5.17	135.1	11	metabolic	cytoplasm;	catalytic a	Pf00043, P	1192	ENSG0000	CLIC1	6; CHR_HS				0	0.889	0.16073648	4.33	19614.6	22075.1	12.32	3.88	High	High	High	High	High	High	1
FALSE	High	Master Prc	Q9P0V9	septin-10 [	0	23.909	12	4	5	2	454	52.6	6.8	13.38	4	cell divisio	cytoplasm;	nucleotide	Pf00735	151011	ENSG0000	SEPT10	2				0	0.889	0.388209829	12.27	1474	1600.9	6.98	8.72	High	High	High	High	High	High	1
FALSE	High	Master Prc	P41567	eukaryotic	0	17.71	22	2	3	2	113	12.7	7.44	13.6	2	metabolic	cytoplasm;	protein bir	Pf01253	10209	ENSG0000	EIF1	17	RNA transp	Translatior		2	0.889	0.884873262	41.38	543.2	489.2	28.05	17.49	High	High	High	High	High	High	1
FALSE	High	Master Prc	P63173	60s riboso	0	30.505	61	6	15	6	70	8.2	10.1	28.72	6	cell organi	cytosol;rib	RNA bindir	Pf01781	6169	ENSG0000	RPL38	17	Ribosome	Mesoderm	Peptide ch	35	0.888	0.008083547	3.44	6476.3	7125.2	12.51	10.23	High	High	High	High	High	High	1
FALSE	High	Master Prc	Q86VP6-1	cullin-asso	0	74.054	17	18	25	18	1230	136.3	5.78	48.47	18	cell differe	cytoplasm;	protein bir	Pf08623, P	55832	ENSG0000	CAND1	12		Endoderm	Neutrophil	9	0.888	0.125159194	6.67	4156.3	4698.2	2.63	5.61	High	High	High	High	High	High	1
FALSE	High	Master Prc	P36404	ADP-ribosy	0	8.637	11	2	3	2	184	20.9	6.34	8.07	2	cell organi	cytoplasm;	catalytic a	Pf00025, P	402	ENSG0000	ARL2	11			Post-chape	6	0.888	0.169890733	5	683.1	787.6	14.97	3.84	High	High	High	High	High	High	1
FALSE	High	Master Prc	O43149	Zinc finger	0	4.875	1	1	1	1	2961	330.9	5.95	3.54	1			metal ion l	Pf00569, P	23140	ENSG0000	ZZEF1	17				0	0.888	0.268375528	10.72	196.1	245.3	21.17	9.92	High	High	High	High	High	High	1
FALSE	High	Master Prc	Q15036-1	Sorting ne	0	5.85	6	2	2	2	470	52.9	7.46	4.38	2	metabolic	cytoplasm;	protein bir	Pf00787	9784	ENSG0000	SNX17	2				0	0.888	0.964003403	8.69	254	231.7	16.53	19.57	High	High	High	High	High	High	1
FALSE	High	Master Prc	Q9Y2L1-1	exosome c	0	27.235	8	7	8	7	958	108.9	7.14	6.78	7	metabolic	cytoplasm;	catalytic a	Pf00773, P	22894	ENSG0000	DIS3	13	RNA degra		Major path	15	0.887	0.042701452	0.46	231.7	265.2	9.08	4.67	High	High	High	High	High	High	1
FALSE	High	Master Prc	Q96KP4	cytosolic n	0	43.767	30	9	12	9	475	52.8	5.97	41.4	9	metabolic	cytoplasm;	catalytic a	Pf01546, P	55748	ENSG0000	CNDP2	18	Metabolic		Glutathion	9	0.887	0.106902684	5.17	3819.6	4337.2	2.35	5.74	High	High	High	High	High	High	1
FALSE	High	Master Prc	P28070	Proteasom	0	23.132	23	5	13	5	264	29.2	5.97	21	5	metabolic	cytoplasm;	catalytic a	Pf00227	5692	ENSG0000	PSMB4	1	Proteasom	Proteasom	Regulation	150	0.887	0.28465481	10.51	2474.3	2817	30.9	5.95	High	High	High	High	High	High	1
FALSE	High	Master Prc	Q9Y221-1	60S riboso	0	24.55	30	3	4	3	180	20.4	8.51	15.65	3	cell organi	cytosol;nu	protein bir	Pf03657	51388	ENSG0000	NIP7	16			Major path	4	0.887	0.292961838	20.45	1961.3	2255	9.15	9.53	High	High	High	High	High	High	1
FALSE	High	Master Prc	Q9Y6K5	2'-5'-oligoa	0	23.857	8	5	7	5	1087	121.1	8.4	20.74	5	defense re	cytoplasm;	catalytic a	Pf01909, P	4940	ENSG0000	OAS3	12	Influenza A		Interferon	10	0.886	0.060851345	4.76	1271.2	1452	5.42	2.81	High	High	High	High	High	High	1
FALSE	High	Master Prc	O43617-1	Trafficking	0.003	3.21	6	1	1	1	180	20.3	4.96	1.74	1	cell organi	cytosol;en	protein bir	Pf04051	27095	ENSG0000	TRAPPC3	1			RAB GEFs e	10	0.886	0.59206098	11.85	403.4	455.5	12.65	4.39	High	High	High	High	High	High	1
FALSE	High	Master Prc	Q9Y266	nuclear mi	0	74.42	47	15	37	15	331	38.2	5.38	93.38	15	cell divisio	cytoplasm;	protein bir	Pf04969, P	10726	ENSG0000	NUDC	1			Amplificati	17	0.886	0.828707362	3.9	11209.5	12458.4	26.01	3.08	High	High	High	High	High	High	1
FALSE	High	Master Prc	Q9H2D1	Mitochonc	0	15.842	13	2	2	2	315	35.4	9.45	6.7	2	metabolic	membrane	transporte	Pf00153	81034	ENSG0000	SLC25A32	8			Metabolism	4	0.885	0.033903056	2.48	31.5	35.4	12.3	6.24	High	High	High	High	High	High	1
FALSE	High	Master Prc	P52907	F-actin-cap	0	70.093	44	9	20	7	286	32.9	5.69	61.19	9	cell organi	cytoplasm;	protein bir	Pf01267	829	ENSG0000	CAPZA1	1	Endocytosi		COPI-indep	26	0.885	0.268533537	6.85	2537.3	2754	8.97	12.32	High	High	High	High	High	High	1
FALSE	High	Master Prc	P08962	CD63 antig	0	5.067	8	2	9	2	238	25.6	7.81	12.62	2	cell differe	cell surface	protein bir	Pf00335	967	ENSG0000	CD63	12	Lysosome;		Platelet de	9	0.885	0.953340019	20.7	1724	2117.8	33.51	4.77	High	High	High	High	High	High	1
FALSE	High	Master Prc	P53621-1	coatomer s	0	248.34	42	41	67	41	1224	138.3	7.66	194.21	41	regulation	cytoplasm;	protein bir	Pf00400, P	1314	ENSG0000	COPA	1			COPI-depe	11	0.884	0.036171437	4.89	15794.7	17562.2	6.94	5.01	High	High	High	High	High	High	1
FALSE	High	Master Prc	P35249	replication	0	11.328	13	4	4	4	363	39.7	8.02	7.09	4	cell organi	nucleus	catalytic a	Pf00004, P	5984	ENSG0000	RFC4	3	Nucleotide	Gastric car	Translesion	58	0.883	0.009814376	0.27	468.8	531.5	6.12	3.67	High	High	High	High	High	High	1
FALSE	High	Master Prc	Q96FW1	Ubiquitin t	0	33.598	26	6	10	6	271	31.3	4.94	30.68	6	metabolic	cytoplasm;	catalytic a	Pf02338, P	55611	ENSG0000	OTUB1	11			Ovarian tu	5	0.883	0.062387228	5.7	1762.9	2083.2	4.37	8.17	High	High	High	High	High	High	1
FALSE	High	Master Prc	Q07666	KH domair	0	48.445	27	13	26	13	443	48.2	8.66	47.55	13	cell organi	cytoplasm;	DNA bindir	Pf00013, P	10657	ENSG0000	KHDRBS1	1		Leptin sign	PTK6 Regu	5	0.883	0.094589919	1.12	4814.8	5214.5	3.92	6.71	High	High	High	High	High	High	1
FALSE	High	Master Prc	P35609-2	Isoform 2 c	0	41.126	9	8	17	2	894	103.9	5.52	26.48	8	cell organi	cytoskeleto	metal ion l	Pf00307, P	88	ENSG0000	ACTN2	1		Arrhythmo		2	0.883	0.2309492	7.09	53.2	63.1	15.29	6.26	High	High	High	High	High	High	1
FALSE	High	Master Prc	Q15648	Mediator c	0	5.406	2	2	2	2	1581	168.4	8.73	0	2	cell differe	chromosor	catalytic a	Pf10744	5469	ENSG0000	MED1	17	Endocrine	Energy Me	Nuclear Re	26	0.883	0.287329994	2.52	1034.5	1171.9	19.08	11.39	High	High	High	High	High	High	1
FALSE	High	Master Prc	Q6K0P9-1	Pyrin and H	0.001	4.513	2	1	1	1	492	55	9.8	3.46	1	regulation	nucleus	protein bir	Pf02758, P	149628	ENSG0000	PYHIN1	1				0	0.883	0.900224838	62.93	567.8	642.8	47.1	10.07	High	High	High	High	High	High	1
FALSE	High	Master Prc	Q9Y276	Mitochonc	0.001	4.44	2	1	2	1	419	47.5	8.5	5	1	cell organi	membrane	catalytic a	Pf00004, P	617	ENSG0000	BCS1L	2			Mitochonc	2	0.882	0.018302507	1.92	559.8	643.3	6.25	3.51	High	High	High	High	High	High	1
FALSE	High	Master Prc	Q00610-1	Clathrin he	0	436.432	49	67	160	67	1675	191.5	5.69	440.56	67	cell differe	cytoplasm;	protein bir	Pf00637, P	1213	ENSG0000	CLTC	17	Huntingtor	Synaptic V	Cargo reco	50	0.882	0.10558891	9.15	52948.3	55000.9	9.39	5.75	High	High	High	High	High	High	1
FALSE	High	Master Prc	P53680	AP-2 comp	0.002	4.084	11	2	4	2	142	17	6.18	4.09	2	cell organi	cytosol;me	protein bir	Pf01217	1175	ENSG0000	AP2S1	19	Huntingtor	Synaptic V	Cargo reco	48	0.882	0.445838086	32.76	728.3	771.5	34.95	8.45	High	High	High	High	High	High	1
FALSE	High	Master Prc	O00214-1	Galectin-8	0.003	3.453	3	1	1	1	317	35.8	8.25	0	1	metabolic	cytoplasm;	protein bir	Pf00337	3964	ENSG0000	LGALS8	1				0	0.882	0.843315057	20.65	839.9	952.2	35.26	4.88	High	High	High	High	High	High	1
FALSE	High	Master Prc	Q6ZMR3	L-lactate d	0	4.884	5	2	4	1	332	36.5	6.99	1.93	2	metabolic	cytoplasm	catalytic a	Pf00056, P	160287	ENSG0000	LDHAL6A	11	Cysteine ar		Pyruvate n	10	0.881	0.075834144	2.09	466.4	546.4	10.87	14.89	High	High	High	High	High	High	1
FALSE	High	Master Prc	Q9GZV5	WW doma	0	6.282	4	1	1	1	400	44.1	5.82	2.33	1	cell differe	cytoplasm;	protein bir	Pf00397	25937	ENSG0000	WWTR1	3	Hippo sign	Adipogene	RUNX2 reg	25	0.881	0.152406699	13.84	896.2	934.4	15.39	6.86	High	High	High	High	High	High	1
FALSE	High	Master Prc	Q7L2J0-1	7SK snRNA	0	33.407	13	7	17	7	689	74.3	9.57	42.4	7	metabolic		catalytic a	Pf00891, P	56257	ENSG0000	MEPCE	7				0	0.881	0.201308246	15.52	3705	4235.1	11.34	2.38	High	High	High	High	High	High	1
FALSE	High	Master Prc	P06132	Uroporphy	0	16.262	15	4	5	4	367	40.8	6.14	7.72	4	metabolic	cytoplasm;	catalytic a	Pf01208	7389	ENSG0000	UROD	1	Metabolic	Heme Bios	Heme bios	6	0.881	0.239454958	7.67	537.9	613.3	19.78	8.51	High	High	High	High	High	High	1
FALSE	High	Master Prc	P26373-1	60S riboso	0	83.162	41	14	42	14	211	24.2	11.65	86.54	14	cell organi	cytosol;me	protein bir	Pf01294	6137	ENSG0000	RPL13	16	Ribosome	Cytoplasm	Peptide ch	34	0.881	0.3908666	9.3	11784.5	12878	12.49	2.23	High	High	High	High	High	High	1

FALSE	High	Master Pro	P04844-1	Dolichyl-di	0	101.079	38	13	21	13	631	69.2	5.69	81.01	13	metabolic	endoplasm	catalytic a	Pf05817	6185	ENSG0000	RPN2	20	Protein pro	Proteasom	Asparagine	9	0.881		0.446492142	31.36	5108.9	5695.2	18.04	9.41	High	High	High	High	High	High	1	
FALSE	High	Master Pro	Q8N129	Protein car	0	5.098	8	1	1	1	248	28.3	4.64	4.35	1	regulation	extracellul	protein bi	Pf11938	245812	ENSG0000	CNPY4	7				0	0.881		0.503583029	62.1	19.8	25.8	70.28	44.2	High	High	High	High	High	High	1	
FALSE	High	Master Pro	O75937	DnaJ homo	0	30.173	23	6	10	6	253	29.8	9.06	20.51	6	metabolic	cytosol;nu	protein bi	Pf00226	22826	ENSG0000	DNAJC8	1		mRNA Pro	mRNA Spli	5	0.88		0.1621498	13.69	3158.6	3587.7	9.55	2.24	High	High	High	High	High	High	1	
FALSE	High	Master Pro	Q96QE5	Transcripti	0	6.794	5	1	1	1	360	41.7	9.32	4.69	1	cell organi	mitochond	catalytic a	Pf12836	79736	ENSG0000	TEFM	17				0	0.88		0.224797593	13.79	88.3	100.3	18.18	1.48	High	High	High	High	High	High	1	
FALSE	High	Master Pro	Q96EB6	NAD-deper	0	18.435	9	6	7	6	747	81.6	4.67	11.2	6	cell commu	chromosor	catalytic a	Pf02146	23411	ENSG0000	SIRT1	10	MicroRNA	Androgen	SIRT1 nega	26	0.88		0.330563803	18.08	1073.5	1646.5	30.2	24.47	High	High	High	High	High	High	1	
FALSE	High	Master Pro	Q96K76	ubiquitin c	0	19.769	5	5	5	5	1375	157.2	5.08	10.02	5	metabolic	cytoplasm	catalytic a	Pf00443, P	55031	ENSG0000	USP47	11			Ub-specific	4	0.88		0.881026495	28.1	263.8	299.8	22.34	10.74	High	High	High	High	High	High	1	
FALSE	High	Master Pro	Q99873	protein arg	0	57.133	40	12	17	12	361	41.5	5.43	55.88	12	cell organi	cytoplasm;	catalytic a	Pf05175, P	3276		PRMT1	19	FoxO signa	Energy Me	TP53 Regu	17	0.88		0.90681456	4.65	5563.3	5951.9	18.65	5.36	High	High	High	High	High	High	1	
FALSE	High	Master Pro	P46783	40S riboso	0	50.487	53	12	47	11	165	18.9	10.15	71.54	12	cell organi	cytoplasm;	protein bi	Pf03501	6204	ENSG0000	RPS10	6	Ribosome	Cytoplasm	Peptide ch	38	0.879		0.023464425	1.52	15171.8	17440.5	3.96	6.22	High	High	High	High	High	High	1	
FALSE	High	Master Pro	Q14008	Cytoskelet	0	8.54	1	2	4	2	2032	225.4	7.8	7.58	2	cell divisio	chromosor	protein bi	Pf12348	9793	ENSG0000	CKAP5	11			Regulation	28	0.879		0.037754293	6.21	1338	1585.2	12.03	8.94	High	High	High	High	High	High	1	
FALSE	High	Master Pro	P60900	Proteasom	0	88.587	60	14	45	14	246	27.4	6.76	105.17	14	metabolic	cytoplasm;	catalytic a	Pf00227, P	5687	ENSG0000	PSMA6	14	Proteasom	Proteasom	Regulation	150	0.879		0.130004718	10.9	11682.7	13294.9	8.52	2.14	High	High	High	High	High	High	1	
FALSE	Medium	Master Pro	Q9H477	ribokinase	0.015	1.754	2	1	1	1	322	34.1	5.05	0	1	metabolic	cytoplasm;	catalytic a	Pf00294, P	64080	ENSG0000	RBKS	2	Pentose ph		Pentose ph	4	0.879		0.195608084	9.91	69.8	83.5	9.97	9.35	High	High	High	High	High	High	1	
FALSE	High	Master Pro	Q01081	Splicing fa	0	32.64	31	6	14	6	240	27.9	8.81	37.43	6	metabolic	nucleus;sp	metal ion b	Pf00076, P	7307; 102	ENSG0000	U2AF1; LO	21	Shigellosis	mRNA Pro	Cleavage o	14	0.879		0.501517434	7.73	3041.1	3155.6	8.75	10.31	High	High	High	High	High	High	1	[K39]
FALSE	High	Master Pro	P45974-1	Ubiquitin c	0	17.322	7	6	7	6	858	95.7	5.03	11.98	6	metabolic	cytosol;va	catalytic a	Pf00443, P	8078	ENSG0000	USP5	12			Synthesis c	6	0.878		0.018128712	3.7	2879.3	3282.6	5.02	5.28	High	High	High	High	High	High	1	
FALSE	High	Master Pro	Q05639	Elongation	0	216.024	54	24	134	12	463	50.4	9.03	325.4	24	metabolic	cytoplasm;	catalytic a	Pf00009, P	1917	ENSG0000	EEF1A2	20	Legionellos	Translation	Eukaryotic	6	0.878		0.064742723	4.31	11879.3	13533.5	2.58	4.64	High	High	High	High	High	High	1	[K165;
FALSE	High	Master Pro	P52888	thimet olig	0	31.174	14	8	11	8	689	78.8	6.05	21.74	8	metabolic	cytoplasm;	catalytic a	Pf01432	7064	ENSG0000	THOP1	19	Renin-angi		Antigen pr	6	0.878		0.140605809	12.04	1618.1	1884.7	2.1	8.04	High	High	High	High	High	High	1	
FALSE	High	Master Pro	Q96HS1-1	Serine/thre	0	7.515	7	2	4	2	289	32	8.68	3.9	2	cell commu	membrane	catalytic a	Pf00300	192111	ENSG0000	PGAM5	12	TNF signali			1	0.878		0.147424334	7.54	2253.5	2565.3	4.68	3.56	High	High	High	High	High	High	1	
FALSE	High	Master Pro	Q15833-1	Syntaxin-b	0	13.127	6	3	3	3	593	66.4	6.55	9.35	3	regulation	cytosol;ext	protein bi	Pf00995	6813	ENSG0000	STXBP2	19		Insulin Sig	Platelet de	9	0.878		0.157593743	1.97	900.8	1006	7.86	1.87	High	High	High	High	High	High	1	
FALSE	High	Master Pro	Q8IYS1	Peptidase	0.006	2.446	2	1	1	1	436	47.7	5.85	2.46	1	metabolic		catalytic a	Pf01546, P	135293	ENSG0000	PM20D2	6				0	0.878		0.160455091	12.21	327.1	373.6	3.92	6.68	High	High	High	High	High	High	1	
FALSE	High	Master Pro	Q9NY26	Zinc transp	0	4.991	4	1	1	1	324	34.2	5.92	2.04	1	transport	endoplasm	protein bi	Pf02535	27173	ENSG0000	SLC39A1	1		NRF2 path	Zinc influx	9	0.878		0.289537667	5.56	368.8	435.6	24.15	16.78	High	High	High	High	High	High	1	
FALSE	High	Master Pro	P50579	methionin	0	36.007	19	7	13	7	478	52.9	5.82	39.59	7	metabolic	cytoplasm;	catalytic a	Pf00557	10988	ENSG0000	METAP2	12			Signaling b	5	0.878		0.379427303	25.4	1680.9	1914.8	12.21	5.92	High	High	High	High	High	High	1	
FALSE	High	Master Pro	Q96ST2	protein IW	0	21.332	7	5	5	5	819	91.9	4.69	10.49	5	metabolic	nucleus	DNA bindi	Pf05297, P	55677	ENSG0000	IWS1	2			Formation	5	0.877		0.009135963	0.51	690	796.4	8.07	8.87	High	High	High	High	High	High	1	
FALSE	High	Master Pro	Q9UJA5-1	tRNA (ade	0	12.132	9	3	3	3	497	55.8	7.55	8.93	3	metabolic	nucleus	RNA bindi	Pf04189	51605	ENSG0000	TRMT6	20			tRNA modi	3	0.877		0.19894515	16.94	102.3	110.6	13.67	3.11	High	High	High	High	High	High	1	
FALSE	High	Master Pro	Q92621	Nuclear po	0	56.476	8	15	18	15	2012	227.8	6.19	29.63	15	cell organi	membrane	protein bi	Pf11894	23165	ENSG0000	NUP205	7	RNA trans		Rev-media	64	0.877		0.524461588	10.17	1442	1645.1	27.71	12.55	High	High	High	High	High	High	1	
FALSE	High	Master Pro	Q8WWX9	Selenoprot	0	10.505	19	2	2	2	145	16.2	5.54	6.78	2	metabolic	cytoplasm;	protein bi	Pf08806	140606	ENSG0000	SELM; SELI	22		Selenium M		2	0.877		0.560820081	52.9	299.7	358	28.32	27.77	High	High	High	High	High	High	1	
FALSE	High	Master Pro	O96005-1	Cleft lip an	0	5.659	3	2	2	2	669	76	6.3	1.97	2	cell differe	membrane	protein bi	Pf05602	1209	ENSG0000	CLPTM1	19				0	0.877		0.692476931	34.32	724.6	902.1	34.22	9.5	High	High	High	High	High	High	1	
FALSE	High	Master Pro	Q99460	26S protea	0	54.793	21	13	17	13	953	105.8	5.39	35.59	13	metabolic	cytosol;ext	catalytic a	Pf01851, P	5707	ENSG0000	PSMD1	2	Epstein-Ba	Proteasom	Regulation	153	0.876		0.071959369	7.97	2472.4	2821.4	2.99	8.71	High	High	High	High	High	High	1	
FALSE	High	Master Pro	P62854	40S riboso	0	27.452	37	4	10	4	115	13	11	16.03	4	cell organi	cytosol;me	protein bi	Pf01283	6231; 101	ENSG0000	RPS26; LO	12; 20; 4	Ribosome	Cytoplasm	Peptide ch	38	0.876		0.339428233	11.54	2784.2	3180.6	7.18	42.45	High	High	High	High	High	High	1	
FALSE	High	Master Pro	Q9UGP4	LIM domai	0	6.465	4	1	1	1	676	72.1	6.65	3.63	1	cell organi	cytoplasm;	metal ion b	Pf00412	8994	ENSG0000	LIMD1	3	Hippo sign		Oxygen-de	7	0.876		0.386340071	20.14	159.6	182.3	11.56	5.38	High	High	High	High	High	High	1	
FALSE	High	Master Pro	O75746	Calcium-bi	0	19.879	6	3	6	1	678	74.7	8.38	16.26	3	metabolic	membrane	metal ion b	Pf00153, P	8604	ENSG0000	SLC25A12	2			Gluconeog	6	0.876		0.42699645	29.77	233.3	266.5	15.54	5.54	High	High	High	High	High	High	1	
FALSE	High	Master Pro	Q9BUH6	Protein PA	0	22.93	30	4	4	4	204	21.6	5.48	14.77	4	metabolic	nucleus	protein bi	Pf15384	286257	ENSG0000	C9orf142;	9				0	0.876		0.622066379	25.11	445.5	508.6	57.06	8.92	High	High	High	High	High	High	1	
FALSE	High	Master Pro	Q9NUU7	ATP-depen	0	38.682	23	8	8	8	478	53.9	6.58	23.44	8	metabolic	cytoplasm;	catalytic a	Pf00270, P	55308	ENSG0000	DDX19A	16				0	0.876		0.814087677	15.36	1091.5	1218.5	20.08	4.51	High	High	High	High	High	High	1	
FALSE	High	Master Pro	Q9ULW3	activator o	0.003	3.273	10	1	1	1	272	31.1	9.88	2.58	1	cell differe	nucleus	DNA bindi	Pf14259	29777	ENSG0000	ABT1	6				0	0.876		0.933811354	18.76	296.4	282.7	29.49	11.22	High	High	High	High	High	High	1	
FALSE	High	Master Pro	P49368-1	T-complex	0	197.659	58	33	91	33	545	60.5	6.49	219.99	33	cell organi	cytoplasm;	nucleotide	Pf00118	7203	ENSG0000	CCT3	1			BBSome-m	13	0.875		0.109696819	1.95	30105.3	33824.7	5.08	1.89	High	High	High	High	High	High	1	
FALSE	High	Master Pro	P32969	60S riboso	0	40.484	52	9	23	9	192	21.9	9.95	39.96	9	cell organi	cytosol;me	protein bi	Pf00347	6133	ENSG0000	RPL9	4	Ribosome	Cytoplasm	Peptide ch	34	0.875		0.186021672	15.96	5804.9	6632.7	8.88	2.66	High	High	High	High	High	High	1	
FALSE	High	Master Pro	P49748-1	Very long-	0	162.981	45	24	58	24	655	70.3	8.75	120.55	24	cell differe	cytosol;me	catalytic a	Pf00441, P	37	ENSG0000	ACADVL	17	Metabolic	Fatty Acid	Beta oxida	15	0.875		0.250784313	18.77	15259.8	17444.4	5.3	11.01	High	High	High	High	High	High	1	
FALSE	High	Master Pro	O15042	U2 snRNP-	0	18.965	4	3	4	3	1029	118.2	8.47	14.53	3	metabolic	nucleus	protein bi	Pf00076, P	23350	ENSG0000	U2SURP	3	Spliceosom		mRNA Spli	5	0.874		0.286544175	21.22	1013.9	1140.9	19.55	9.65	High	High	High	High	High	High	1	
FALSE	High	Master Pro	Q9BY32-1	Inosine tri	0	5.981	12	2	2	2	194	21.4	5.66	2.11	2	cell organi	cytoplasm;	catalytic a	Pf01725	3704	ENSG0000	ITPA	20	Drug meta		Purine cat	7	0.874		0.534125856	58.84	14	16	65.91	28.97	High	High	High	High	High	High	1	
FALSE	High	Master Pro	P30086	phosphatic	0	97.538	79	11	33	11	187	21	7.53	106.17	11	metabolic	cytoplasm;	enzyme re	Pf01161	5037	ENSG0000	PEBP1	12		EGF/EGFR	Paradoxica	19	0.873		0.047404226	2.15	7040.4	8075.8	9.38	4.12	High	High	High	High	High	High	1	
FALSE	High	Master Pro	Q9Y295	developme	0	35.178	19	5	11	4	367	40.5	8.9	22.21	5	developme	cytoplasm;	nucleotide	Pf00009, P	4733	ENSG0000	DRG1	22				0	0.873		0.101933205	4.47	2735	3156.1	9.31	5.54	High	High	High	High	High	High	1	
FALSE	High	Master Pro	Q02252-2	Isoform 2	0	8.875	7	4	5	4	522	56.2	8.37	4.79	4	cell differe	mitochond	catalytic a	Pf00171	4329	ENSG0000	ALDH6A1	14				0	0.873		0.135986662	9.82	800.6	1034.1	13	6.87	High	High	High	High	High	High	1	
FALSE	Low	Master Pro	Q9Y3C4	EKC/KEOP	0.065	1.083	10	1	1	1	175	19.6	6.79	0	1	metabolic	cytoplasm;	protein bi	Pf08617	51002	ENSG0000	TPRKB	2			tRNA modi	3	0.873		0.973324428	64.44	40.5	35.7	40.87	19.88	High	High	High	High	High	High	1	
FALSE	High	Master Pro	Q9BWD1	Acetyl-CoA	0	55.563	34	8	13	8	397	41.3	6.92	41.14	8	metabolic	cytoplasm;	catalytic a	Pf00108, P	39	ENSG0000	ACAT2	6	Metabolic		Cholestero	18	0.872		0.230073974	1.93	2533.6	2906.2	1.84	10.77	High	High	High	High	High	High	1	
FALSE	High	Master Pro	Q8TCJ2	Dolichyl-di	0	23.575	7	4	6	4	826	93.6	8.91	17.48	4	metabolic	endoplasm	catalytic a	Pf02516, P	201595	ENSG0000	STT3B	3	N-Glycan b			3	0.872		0.331930735	1.67	1909	2187.1	9.34	1.67	High	High	High	High	High	High	1	
FALSE	High	Master Pro	Q13907	Isopenteny	0.004	2.693	5	1	1	1	227	26.3	6.34	2.51	1	metabolic	cytosol	catalytic a	Pf00293	3422		IDI1	10	Terpenoid	Target Of I	Cholestero	12	0.872		0.39700835	31.86	581.6	704.2	22.53	19.45	High	High	High	High	High	High	1	
FALSE	High	Master Pro	Q9BY42	protein RT	0	35.347	21	4	6	4	306	33.9	8.59	21.25	4	metabolic	nucleus		Pf04641	51507	ENSG0000	RTFDC1	20				0	0.872		0.624008169	5.91	1714	1813.9	47.81	4.74	High	High	High	High	High	High	1	
FALSE	High	Master Pro	P55884	Eukaryotic	0	108.565	32	19	39	19	814	92.4	5	109.94	19	cell organi	cytoplasm;	protein bi	Pf00076, P	8662	ENSG0000	EIF3B	7	RNA trans	Translation	Formation	13	0.871		0.035620405	0.44	13940.7	16115.2	3.29	2.52	High	High	High	High	High	High	1	
FALSE	High	Master Pro	Q02543	60S riboso	0	42.581	37	8	16	8	176	20.7	10.71	36.89	8	cell organi	cytosol;me	protein bi	Pf01775	6142	ENSG0000	RPL18A	19	Ribosome	Cytoplasm	Peptide ch	34	0.871		0.038634156	2.88	2943.2	3404.1	0.84	4.75	High	High	High	High	High	High	1	
FALSE	High	Master Pro	O75352	Mannose-P	0	5.511	10	2	2	2	247	26.6	8.94	7.1	2	metabolic	membrane	protein bi	Pf04193	9526	ENSG0000	MPDU1	17			Defective I	8	0.871		0.118648868	9.2	1136.9	1386.1	5.78	12.38	High	High	High	High	High	High	1	
FALSE	High	Master Pro	Q9NZM5	ribosome b	0	11.395	7	3	4	3	478	54.4	10.32	7.98	3	cell organi	cytosol;nu	protein bi	Pf07767	29997	ENSG0000	GLTSCR2; I	19	Herpes sim			1	0.871		0.257894135	6.59	228.4	245.4	8.17	5.59	High	High	High	High	High	High	1	
FALSE	High	Master Pro	Q9P016	Thymocyte	0.006	2.393	7	2	2	2	225	25.7	9.25	2.25	2		nucleus		Pf01878	29087	ENSG0000	THYN1	11				0	0.87		0.416360414	17.5	616.3	708.8	14.12	4.41	High	High	High	High	High	High	1	
FALSE	High	Master Pro	P19174-1	1-phospha	0	8.166	2	2	3	2	1290	148.4	6.05	5.87	2	cellular co	cytoplasm;	catalytic a	Pf00017, P	5335	ENSG0000	PLCG1	20	Neurotrop	Angiogene	EGFR inter	128	0.87		0.430638618	35.31	193.5	236.2	14.85	19.23	High	High	High	High	High	High	1	
FALSE	Medium	Master Pro	Q6NXT1-1	Ankyrin re	0.015	1.738	2	1	1	1	300	32.5	6.28	2.72	1	regulation	cytoplasm;	enzyme re	Pf00023, P	129138	ENSG0000	ANKRD54	22				0	0.869		0.085294059	6.26	105.1	118	4.07	4.13	High	High	High	High	High	High	1	
FALSE	High	Master Pro	Q16401	26S protea	0	41.545	17	7	11	7	504	56.2	5.48	35.21	7	cell organi	cytosol;pr	protein bi	Pf10508	5711	ENSG0000	PSMD5	9		Proteasom	Regulation	150	0.869		0.104647271	0.77	2137.8	2319.3	3.91	4.92	High	High	High	High	High	High	1	
FALSE	High	Master Pro	Q9UJY1	Heat shock	0	5.946	10	2	3	2	196	21.6	5.12	2.16	2	regulation	cytoplasm;	catalytic a	Pf00011	26353	ENSG0000	HSPB8	12			HSF1-depe	4	0.869		0.360913232	17.41	1105.8	1272.1	13.77	2.24	High	High	High	High	High	High	1	
FALSE	High	Master Pro	O14561	Acyl carrie	0	29.193	24	5	9	5	156	17.4	4.93	34.45	5	cell organi	cytosol;me	catalytic a	Pf00550	4706	ENSG0000	NDUFAB1	16	Oxidative p	Electron Tr	Mitochonc	18	0.869		0.480759144	43.89	2267.8	2781.6	22.19	25.64	High	High	High	High	High	High	1	
FALSE	High	Master Pro	O00154	cytosolic a	0	55.775	36	11	20	11	380	41.8	8.54	52.5	11	metabolic	cytoplasm;	catalytic a	Pf03061	11332	ENSG0000	ACOT7	1	Fatty acid		Mitochonc	6	0.868		0.063755869	7.39	5895.7	6468.2	10.34	5.22	High	High	High	High	High	High	1	
FALSE	High	Master Pro	Q13951	Core-bindi	0	18.102	14	2	4	2	182	21.5	6.6	14.48	2	cell differe	membrane	DNA bindi	Pf02312	865	ENSG0000	CBFB	16		Dual hijack	RUNX3 reg	30	0.868		0.120737038	10.58	381.5	469.8	10.96	4.26	High	High	High	High	High	High	1	
FALSE	High	Master Pro	P53794	sodium/my	0	6.939	3	2	3	2	718	79.6	7.27	1.93	2	metabolic	membrane	transporte	Pf00474	6526		SLC5A3	21		NRF2 path	Inositol tra	5	0.868		0.167518912	6.09	169.2	194.8	3.94	17.86	High	High	High	High	High	High	1	
FALSE	Medium	Master Pro	Q6P1N9-1	Putative de	0.023	1.554	2	1	1	1	297	33.6	6.96	0	1	metabolic	nucleus	catalytic a	Pf01026	83940	ENSG0000	TATDN1	8				0	0.868		0.190827268	15.99	64.6	76.3	10.69	10.34	High	High	High	High	High	High	1	
FALSE	High	Master Pro	Q86U42-1	polyadenyl	0	13.384	9	3	8	3	306	32.7	5.06	19.82	3	metabolic	cytoplasm;	protein bi	Pf00076, P	8106	ENSG0000	PABPN1	14	mRNA surv	mRNA Pro	Inhibition o	20	0.868		0.322016723	6.28	3024.2	3518.9	0.72	12.19	High	High	High	High	High	High	1	
FALSE	High	Master Pro	O14908-1	PDZ domai	0	27.194	17	5	7	5	333	36	6.28	14.86	5	cell commu	cytoplasm;	protein bi	Pf00595	10755	ENSG0000	GIPC1	19		VEGFA-VEC		1	0.868		0.781728085	12.42	1604.8	1849.2	30.82	10.19	High	High	High	High	High	High	1	
FALSE	High	Master Pro	Q9BRJ6	Uncharacte	0	14.186	28	4	6	4	194	22.1	9.64	11.45	4			protein bi	Pf10180	84310	ENSG0000	C7orf50	7				0	0.867		0.261305751	22.3	1697.3	1958.1	23.07	7.38	High	High	High	High	High	High	1	
FALSE	High	Master Pro	P39748	Flap endor	0	63.927	39	11	21	11	380	42.6	8.62	53.17	11	cell organi	membrane	catalytic a	Pf00752, P	2237	ENSG0000	FEN1	11	DNA replic		POLB-Depe	32	0.866		0.033253471	6.76	4267.6	4955.9	3.75	3.32	High	High	High	High	High	High	1	
FALSE	High	Master Pro	Q9C005	Protein dp	0	8.834	31	2	3	2	99	11.2	4.88	2.5	2	cell organi	Golgi;nucle	catalytic a	Pf05186	84661	ENSG0000	DPY30	2			PKMTs me	8	0.866		0.074098051	5.94	62.2	69	11.37	2.69	High	High	High	High	High	High	1	
FALSE	High	Master Pro	Q9H0D6-1	5'-3' exorib	0	22.918	10	6	9	6	950	108.5	7.47	14.7	6	cell differe	membrane	catalytic a	Pf03159	22803	ENSG0000	XRN2	20	RNA degra	mRNA Pro	Major path	11	0.866		0.095681462	4.85	1454.8	1656.1	1.3	7.07	High	High	High	High	High	High	1	
FALSE	High	Master Pro	P82675	28S riboso	0	23.356	18	6	8	6	430	48	9.92	16.92	6	cell organi	membrane	RNA bindi	Pf00333, P	64969	ENSG0000	MRPS5	2	Ribosome		Mitochonc	7	0.866		0.109458828	2.32	1832.6	2106.8	13.92	18.4	High	High	High	High	High	High	1	
FALSE	High	Master Pro	Q8NC51-1	Plasminog	0	115.125	44	18	45	18	408	44.9	8.65	101.31	18	regulation	cytoplasm;	protein bi	Pf04774	26135	ENSG0000	SERBP1	1		Apoptosis-		1	0.866		0.369323747	9.68	19132.4	21257.7	10.49	4.98	High	High	High	High	High	High	1	
FALSE	High	Master Pro	Q9NP77	RNA polym	0	8.566	13	2	3	2	194	22.6	5.33	7.89	2	metabolic	cytoplasm;	catalytic a	Pf04722	29101	ENSG0000	SSU72	1	mRNA surv		RNA polym	4	0.866		0.549282429	11.3	1169	1361.4	70.87	1.17	High	High	High	High	High	High	1	
FALSE	High	Master Pro	Q13085-1	Acetyl-CoA	0	97.801	12	20	26	20	2346	265.4	6.37	57.59	20	cell organi	cytoplasm;	catalytic a	Pf00289, P	31	ENSG0000	ACACA	17; CHR_H	AMPK sign	AMP-activ	Fatty acyl-	35	0.865		0.007241804	3.78	2789.3	3224.7	8.27	5.25	High	High	High	High	High	High	1	
FALSE	High	Protein	P42345	eonine-	0	14.199	2	4	4	4	2549	288.7	7.17	9.53	4	communic	;cytosol;e	activity;D	Pf02259,	2475	0198793.	MTOR	1	hormone	activated	dependen	92	0.865		0.012597403	1.93	1205.3	1394.4	2.91	5.05	High	High	High	High	High	High	1	
FALSE	High	Master Pro	P58546	Myotrophi	0	15.143	14	1	4	1	118	12.9	5.52	16.14	1	cell differe	cytoplasm;	DNA bindi	Pf00023, P	767558; 1	ENSG0000	LUZP6; MT	7				0	0.865		0.324090458	26.26	1041	1349.7	17.87	17.13	High	High	High	High	High	High	1	
FALSE	High	Master Pro	Q9NVI1-1	Isoform 1	0	4.852	2	3	4	3	1268	142.5	6.76	0	3	metabolic	cytosol;me	DNA bindi	Pf14674, P	55215	ENSG0000	FANCI	15		Gastric car		1	0.865		0.55794362	24.13	463.4	536	72.51	14.32	High	High	High	High	High	High	1	
FALSE	High	Master Pro	Q9H3K2	Growth ho	0	7.267	6	2	2	2	345	37.2	9.94	5.15	2	cell death	membrane	protein bi		27069	ENSG0000	GHITM	10				0	0.864		0.018365357	0.84	454.5	526.2	3.62	6.52	High	High	High	High	High	High	1	
FALSE	High	Master Pro	P49207	60S riboso	0	37.661	44	8	34	8	117	13.3	11.47	49.57	8	cell organi	cytosol;mi	protein bi	Pf01199	6164	ENSG0000	RPL34	4	Ribosome	Cytoplasm	Peptide ch	34	0.864		0.048055388	9.26	12068.9	13092.8	13.9	8.67	High	High	High	High	High	High	1	
FALSE	High	Master Pro	P33991	DNA replic	0	139.007	42	24	42	24	863	96.5	6.74	109.63	24	metabolic	membrane	catalytic a	Pf00493, P	4173	ENSG0000	MCM4	8	Cell cycle;	Cell Cycle;	M/G1 Trar	27	0.863		0.125401907	7.88	7074.6	8136	4.37	7.3	High	High	High	High	High	High	1	
FALSE	High	Master Pro	Q9Y5K5-1	Ubiquitin c	0	20.242	19	5	6	5	329	37.6	5.33	14.45	5	metabolic	cytoplasm;	catalytic a	Pf01088	51377	ENSG0000	UCHL5	1		TGF-beta S	UCH prote	10	0.863		0.273413291	21.79	1287.7	1491.5	10.34	9.75	High	High	High	High	High	High	1	
FALSE	High	Master Pro	Q9H967	WD repeat	0	12.468	5	2	2	2	626	69.7	9.25	7.35	2	regulation	nucleus	DNA bindi	Pf00400	79968	ENSG0000	WDR76	15				0	0.863		0.930003387	21.48	447.2	517.9	22.31	10.75	High	High	High	High	High	High	1	
FALSE	High	Master Pro	Q96PE3-1	Type I inos	0	7.519	2	1	1	1	977	109.9	6.96	4.94	1	metabolic	cytoplasm;	catalytic a	Pf00168	3631	ENSG0000	INPP4A	2	Inositol ph	Insulin Sig	Synthesis o	12	0.862		0.019800612	3.71	54.8	63.6	3.01	1.15	High	High	High	High	High	High	1	
FALSE	High	Master Pro	Q8WXA9-2	Isoform 2	0	11.893	6	3	3	3	624	71.6	10.2	8.72	3	metabolic	spliceosom	protein bi	Pf00076, P	140890	ENSG0000	SREK1	5		mRNA Pro		1	0.862		0.053488055	8.97	1483.4	1720.8	4.2	2.3	High	High	High	High	High	High	1	

FALSE	High	Master Prc	Q7Z3U7-1	Protein MC	0.003	2.945	1	1	1	1	1717	190.2	6.06	2.85	1	transport	cytosol	protein bir	Pf09324, P	23041	ENSG0000	MON2	12				0	0.862		0.079356469	9.39	343.3	403.5	4.44	5.03	High	High	High	High	High	High	1	
FALSE	High	Master Prc	P23526-1	Adenosylh	0	95.566	32	12	40	12	432	47.7	6.34	108.49	12	defense re	cytoplasm;	catalytic a	Pf00670, P	191	ENSG0000	AHCY	20	Cysteine ar	Folate Met	Metabolisr	18	0.861		0.015791353	2.69	12393.4	14445.6	3.43	1.7	High	High	High	High	High	High	1	
FALSE	High	Master Prc	Q96TA1	Niban-like	0	74.906	27	13	21	13	746	84.1	6.19	65.79	13	cell differe	cytoplasm;	protein bir		64855	ENSG0000	FAM129B	9				0	0.86		0.010752559	2.27	4638.9	5418.6	2.86	1.36	High	High	High	High	High	High	1	
FALSE	High	Master Prc	P62875	DNA-direct	0.003	3.099	22	2	3	2	67	7.6	7.77	2.34	2	metabolic	cytosol;nu	catalytic a	Pf01194	5441	ENSG0000	POLR2L	11	Metabolic		Signaling b	97	0.86		0.015913562	1.15	2249.3	2495.9	4.01	4.24	High	High	High	High	High	High	1	
FALSE	Medium	Master Prc	Q8TDB6-1	E3 ubiquiti	0.042	1.271	1	1	1	1	740	83.5	8.06	0	1	cell organi	cytoplasm;	catalytic a	Pf00097, P	151636	ENSG0000	DTX3L	3	Notch sign	Notch Sign	Antigen pr	6	0.86		0.224856678	6	72.6	83	10.74	1	High	High	High	High	High	High	1	
FALSE	High	Master Prc	Q93050-1	Isoform 2 c	0	7.447	3	2	2	2	831	95.7	6.65	4.64	2	cell organi	cytosol;Go	catalytic a	Pf01496	535	ENSG0000	ATP6V0A1	17				0	0.859		0.122104629	9.14	610.5	782.2	15.51	6.39	High	High	High	High	High	High	1	
FALSE	High	Master Prc	Q9H2H8-1	peptidyl-pr	0	6.243	7	1	2	1	161	18.1	6.79	1.84	1	metabolic	spliceosom	catalytic a	Pf00160	53938	ENSG0000	PPIL3	2			mRNA Spli	4	0.859		0.123171563	9.24	601.5	696.4	13.06	2.39	High	High	High	High	High	High	1	
FALSE	High	Master Prc	P60510	Serine/thre	0	33.538	23	5	6	3	307	35.1	5.06	21.12	5	metabolic	cytoplasm;	catalytic a	Pf00149	5531	ENSG0000	PPP4C	16	Glucagon s		Processing	6	0.859		0.256557697	21.04	1243.2	1470.1	14.78	10.15	High	High	High	High	High	High	1	
FALSE	High	Master Prc	Q9H0L4	cleavage st	0	19.744	16	6	8	2	616	64.4	7.25	9.59	6	metabolic	nucleus	protein bir	Pf00076, P	23283	ENSG0000	CSTF2T	10	mRNA surv	mRNA Pro	Cleavage o	13	0.859		0.673277886	36.96	410	477.4	20.08	12.6	High	High	High	High	High	High	1	
FALSE	High	Master Prc	Q96GM8	Target of E	0	12.978	6	2	2	2	510	56.5	7.18	6.24	2	metabolic	cytoplasm;	catalytic a	Pf00642, P	114034	ENSG0000	TOE1	1				0	0.859		0.700277958	12.24	356.9	415.2	44.3	5.59	High	High	High	High	High	High	1	
FALSE	High	Master Prc	Q96CW5	Gamma-tu	0	9.427	3	2	2	2	907	103.5	8.12	6.31	2	cell organi	cytoplasm;	protein bir	Pf04130	10426	ENSG0000	TUBGCP3	13			Recruitmer	9	0.858		0.058061127	3.88	163.1	210.3	7.86	10.35	High	High	High	High	High	High	1	
FALSE	High	Master Prc	P46060	Ran GTPas	0	93.366	30	12	23	12	587	63.5	4.68	65.67	12	cell organi	chromosor	enzyme re	Pf07834, P	5905	ENSG0000	RANGAP1	22	RNA transp		Rev-media	30	0.858		0.262491509	21.03	5984.1	6954.8	19.47	8.26	High	High	High	High	High	High	1	
FALSE	High	Master Prc	Q96AQ6	Pre-B-cell l	0	23.83	10	6	7	6	731	80.6	5.33	13.73	6	cell differe	cytoplasm;	protein bir		57326	ENSG0000	PBXIP1	1				0	0.858		0.422434239	34.45	1205	1329.8	20.66	8.62	High	High	High	High	High	High	1	
FALSE	High	Master Prc	P36405	ADP-ribosy	0	6.382	14	2	2	2	182	20.4	7.24	4.36	2	cell divisio	cytoplasm;	catalytic a	Pf00009, P	403	ENSG0000	ARL3	10			Trafficking	4	0.858		0.546736633	43.2	23.1	26.9	24.24	10.21	High	High	High	High	High	High	1	
FALSE	High	Master Prc	P48444	Coatomer	0	59.237	30	14	23	14	511	57.2	6.21	44.37	14	response t	cytoplasm;	RNA bindir	Pf00928	372	ENSG0000	ARCN1	11		miR-517 re	COPI-depe	12	0.857		0.056945318	1.67	8630.1	9700.2	3.12	6.17	High	High	High	High	High	High	1	
FALSE	High	Master Prc	P20042	Eukaryotic	0	24.646	18	5	6	5	333	38.4	5.8	20.45	5	cell prolife	cytoplasm;	metal ion l	Pf01873, P	8894	ENSG0000	EIF2S2	20	RNA transp	Translatior	Formation	16	0.857		0.771432646	14.81	1211.8	1381.5	19.89	8.3	High	High	High	High	High	High	1	
FALSE	Medium	Master Prc	O95714	E3 ubiquiti	0.017	1.672	0	1	1	1	4834	526.9	6.28	2.16	1	metabolic	cytoplasm;	catalytic a	Pf00173, P	8924	ENSG0000	HERC2	15	Ubiquitin r		G2/M DNA	22	0.856		0.014334919	3.81	508.2	587.2	4.52	3.05	High	High	High	High	High	High	1	
FALSE	High	Master Prc	Q14019	coactosin-l	0	51.66	63	14	32	14	142	15.9	5.67	52.9	14	defense re	cytoplasm;	protein bir	Pf00241, P	23406	ENSG0000	COTL1	16			Neutrophil	3	0.856		0.026791727	4.85	7260.7	8669.8	3.81	2.27	High	High	High	High	High	High	1	
FALSE	High	Master Prc	Q14258	E3 ubiquiti	0	51.905	25	12	16	12	630	70.9	8.09	41.55	12	defense re	cytoplasm;	catalytic a	Pf00097, P	7706	ENSG0000	TRIM25	17	NF-kappa I	RIG-I-like F	Terminatic	25	0.856		0.116108831	9.44	3583.1	4187.9	4.27	4.91	High	High	High	High	High	High	1	
FALSE	High	Master Prc	Q7Z739	YTH domai	0	18.738	6	3	4	2	585	63.8	9.04	5.84	3	regulation	cytoplasm;	protein bir	Pf04146	253943	ENSG0000	YTHDF3	8				0	0.856		0.205041738	16.28	306	394.8	17.18	7.28	High	High	High	High	High	High	1	
FALSE	High	Master Prc	Q9UJS0	Calcium-bi	0	41.898	17	7	11	5	675	74.1	8.62	32.36	7	metabolic	membrane	metal ion l	Pf00153, P	10165	ENSG0000	SLC25A13	7			Gluconeog	6	0.856		0.301517674	18.19	2572.5	3005.3	7.58	8.8	High	High	High	High	High	High	1	
FALSE	High	Master Prc	P62495	Eukaryotic	0	51.901	29	8	10	8	437	49	5.71	36.11	8	cell organi	cytoplasm;	protein bir	Pf03463, P	2107	ENSG0000	ETF1	5	mRNA surv	Translatior	Regulation	13	0.856		0.371217252	4.43	4376.3	5047.9	6.14	7.89	High	High	High	High	High	High	1	
FALSE	High	Master Prc	Q6NYC1	Bifunctiona	0	5.605	3	1	1	1	403	46.4	8.69	5	1	cell differe	cytosol;me	catalytic a	Pf02373, P	23210	ENSG0000	JMJD6	17			HDMs der	3	0.856		0.523674164	55.06	403	470.7	41.45	26.37	High	High	High	High	High	High	1	
FALSE	High	Master Prc	Q99685-1	monoglyce	0	22.809	19	4	6	4	303	33.2	6.99	18.96	4	defense re	cytoplasm;	catalytic a	Pf00561, P	11343	ENSG0000	MGLL	3	Metabolic		Triglycerid	20	0.855		0.226702483	14.17	2254.9	2643	13.77	11.5	High	High	High	High	High	High	1	
FALSE	High	Master Prc	Q9UDY2	Tight junct	0	41.846	11	11	13	11	1190	133.9	7.4	18.55	11	metabolic	cytosol;me	catalytic a	Pf00595, P	9414	ENSG0000	TJP2	9	Vibrio chol		Apoptotic	9	0.854		0.109515871	2.66	2056	2381.9	7.9	2.63	High	High	High	High	High	High	1	[Q480]
FALSE	High	Master Prc	P11586	C-1-tetrahy	0	146.953	39	32	59	31	935	101.5	7.3	118.04	32	metabolic	cytoplasm;	catalytic a	Pf00763, P	4522		MTHFD1	14	Metabolic	Folate Met	Metabolisr	9	0.854		0.112998945	0.29	9767.8	10928.1	7.24	3.3	High	High	High	High	High	High	1	
FALSE	High	Master Prc	Q16527	Cysteine ar	0	7.644	16	2	3	2	193	20.9	8.62	3.98	2	cell differe	nucleus	metal ion l	Pf00412	1466	ENSG0000	CSRP2	12		Mesoderm		2	0.854		0.199082411	19.15	320.7	379.7	16.23	1.16	High	High	High	High	High	High	1	
FALSE	High	Master Prc	P52564-1	Dual specif	0	32.215	19	6	14	4	334	37.5	7.39	33.45	6	cell death;	cytoplasm;	catalytic a	Pf00069, P	5608	ENSG0000	MAP2K6	17	GnRH sign	Regulation	PI5P Regul	81	0.854		0.780561282	13.48	2582.7	3023.5	22.88	3.19	High	High	High	High	High	High	1	
FALSE	High	Master Prc	P61088	ubiquitin-c	0	29.251	45	8	12	8	152	17.1	6.57	16.12	8	cell organi	cytoplasm;	catalytic a	Pf00179, P	7334	ENSG0000	UBE2N	12	Ubiquitin r	IL-1 signali	IRAK1 recr	69	0.853		0.069387553	9.89	2462.4	2812.7	8.53	2.05	High	High	High	High	High	High	1	
FALSE	High	Master Prc	P42695	condensin-	0	14.03	3	4	4	4	1498	168.8	7.5	9.87	4	cell divisio	membrane	protein bir	Pf12717, P	23310	ENSG0000	NCAPD3	11			Condensat	5	0.853		0.217444733	21.69	1080.9	1266.6	13.92	1.07	High	High	High	High	High	High	1	
FALSE	High	Master Prc	Q01581	hydroxyme	0	77.793	31	12	23	12	520	57.3	5.41	58.55	12	metabolic	cytoplasm;	catalytic a	Pf01154, P	3157	ENSG0000	HMGCS1	5	Butanoate	Cholestero	Cholestero	17	0.853		0.541162695	13.84	4905.8	5569	19.79	10.92	High	High	High	High	High	High	1	
FALSE	High	Master Prc	P26196	Probable A	0	79.986	37	14	23	14	483	54.4	8.66	57.11	14	cell organi	cytoplasm;	catalytic a	Pf00270, P	1656	ENSG0000	DDX6	11	RNA degra		mRNA dec	4	0.852		0.016790023	4.37	5272.3	6343.5	2.3	4.66	High	High	High	High	High	High	1	
FALSE	High	Master Prc	P14618	Pyruvate k	0	432.977	77	46	239	46	531	57.9	7.84	625.68	46	cell death;	cytoplasm;	catalytic a	Pf00224, P	5315	ENSG0000	PKM	15	Carbon me	Photodyna	Glycolysis;	20	0.852		0.020117179	5.02	59471.4	67562	4.88	2.23	High	High	High	High	High	High	1	
FALSE	High	Master Prc	P10620	Microsoma	0.002	3.721	8	1	1	1	155	17.6	9.39	3.13	1	cell differe	endoplasm	antioxidan	Pf01124	4257	ENSG0000	MGST1	12	Chemical c	Aryl Hydro	Glutathion	16	0.852		0.273283807	25.38	455.6	521.8	15.34	10.31	High	High	High	High	High	High	1	
FALSE	High	Master Prc	O43633	charged m	0	14.24	15	4	6	4	222	25.1	5.97	7.48	4	cell commu	chromosor	protein bir	Pf03357	27243	ENSG0000	CHMP2A	19	Endocytosi		Budding ar	15	0.852		0.401441919	32.01	515.6	605.5	18.03	4.77	High	High	High	High	High	High	1	
FALSE	High	Master Prc	Q92973-1	transportir	0	51.791	17	14	23	14	898	102.3	4.98	44.15	14	cellular co	cytoplasm;	protein bir	Pf03810, P	3842	ENSG0000	TNPO1	5			Tristetrapr	6	0.85		0.007018031	2.84	6890	8109.3	0.5	2.55	High	High	High	High	High	High	1	
FALSE	High	Master Prc	Q02878	60S riboso	0	82.635	56	17	52	17	288	32.7	10.58	101.11	17	cell organi	cytosol;me	DNA bindir	Pf01159, P	6128	ENSG0000	RPL6	12	Ribosome	Cytoplasm	Peptide ch	34	0.85		0.040598635	7.45	20174.4	22056.9	9.34	6.45	High	High	High	High	High	High	1	
FALSE	High	Master Prc	Q9UBU9-1	nuclear RN	0	18.445	11	6	7	6	619	70.1	8.51	11.28	6	transport	cytoplasm;	protein bir	Pf02136, P	10482	ENSG0000	NXF1	11	Ribosome	mRNA Pro	Transport	14	0.85		0.191269323	10.33	277.2	348.9	7.04	14.74	High	High	High	High	High	High	1	
FALSE	High	Master Prc	P60174-1	Isoform 2 c	0	212.697	91	21	83	21	249	26.7	6.9	248.92	21	developme	cytosol;nu	catalytic a	Pf00121	7167	ENSG0000	TPI1	12		Fatty Acid		3	0.85		0.202137536	0.32	28540.1	32402	9.2	2.28	High	High	High	High	High	High	1	[T46(50);
FALSE	High	Master Prc	O00303	Eukaryotic	0	39.32	31	9	16	9	357	37.5	5.45	29.89	9	metabolic	cytoplasm;	catalytic a	Pf01398, P	8665	ENSG0000	EIF3F	11; CHR_H	RNA transp	Translatior	Formation	13	0.849		0.137704731	16.77	3982.9	4466.4	10.2	8.59	High	High	High	High	High	High	1	
FALSE	High	Master Prc	Q96T76-1	MMS19 nu	0	8.562	3	2	2	2	1030	113.2	6.35	7.72	2	cell organi	cytoplasm;	protein bir	Pf12460, P	64210	ENSG0000	MMS19	10			Cytosolic ir	2	0.849		0.982597128	5.32	32.7	34	9.51	28.11	High	High	High	High	High	High	1	
FALSE	High	Master Prc	Q9BQE3	Tubulin alp	0	232.001	63	23	157	5	449	49.9	5.1	441.62	23	cell divisio	cytoplasm;	catalytic a	Pf00091, P	84790	ENSG0000	TUBA1C	12	Apoptosis;	Pathogeni	COPI-indep	66	0.848		0.010399627	0.88	8170.5	9284.6	6.56	6.98	High	High	High	High	High	High	1	[T271(100
FALSE	High	Master Prc	Q9H4A4	aminopept	0	67.335	33	15	24	15	650	72.5	5.74	60.65	15	metabolic	extracellul	catalytic a	Pf01433, P	6051	ENSG0000	RNPEP	1				0	0.848		0.013987587	2.6	4570.8	5141.2	4.27	3.07	High	High	High	High	High	High	1	
FALSE	High	Master Prc	Q99584	Protein S10	0	14.472	32	3	6	3	98	11.5	6.16	15.93	3	regulation	cytoplasm;	metal ion l	Pf01023	6284	ENSG0000	S100A13	1				0	0.848		0.064812889	5.68	3859.1	4471.5	4.55	9.28	High	High	High	High	High	High	1	
FALSE	High	Master Prc	Q9H694-1	Protein bic	0	18.791	6	3	4	3	974	104.8	8.54	11.85	3	developme	cytoplasm	protein bir	Pf00013, P	80114	ENSG0000	BICC1	10				0	0.848		0.649221472	1.46	1296.6	1543.4	55.41	13.93	High	High	High	High	High	High	1	
FALSE	High	Master Prc	Q9BSC4-1	Nucleolar p	0	7.042	7	3	3	3	688	80.3	8.46	6.33	3		nucleus	protein bir	Pf08159	79954	ENSG0000	NOL10	2				0	0.847		0.04086405	3.97	666.6	736.7	7.06	4.7	High	High	High	High	High	High	1	
FALSE	High	Master Prc	O75223-1	gamma-glu	0	46.94	63	9	16	9	188	21	5.14	43.5	9	cell organi	cytosol	catalytic a	Pf06094, P	79017	ENSG0000	GGCT	7	Glutathion		Glutathion	6	0.847		0.166897423	6.22	3430.8	3952.9	11.1	2.17	High	High	High	High	High	High	1	
FALSE	High	Master Prc	Q14691	DNA replic	0	8.912	7	1	2	1	196	23	7.39	7.51	1	cell prolife	cytoplasm;	catalytic a	Pf05916	9837	ENSG0000	GINS1	20			Unwinding	7	0.846		0.133465046	15.49	241.7	296.7	3.33	12.4	High	High	High	High	High	High	1	
FALSE	High	Master Prc	Q99598	Translin-as	0	31.855	24	6	8	6	290	33.1	6.55	22.17	6	cell differe	cytoplasm;	DNA bindir	Pf01997	7257	ENSG0000	TSNAX	1			Small inter	3	0.845		0.009780127	4.45	1487.4	1705.2	4.49	2.41	High	High	High	High	High	High	1	
FALSE	High	Master Prc	Q9UNZ2	NSFL1 cofa	0	34.5	24	7	12	1	370	40.5	5.1	17.91	7	cell organi	chromosor	enzyme re	Pf00789, P	55968	ENSG0000	NSFL1C	20	Protein pro			1	0.845		0.058525428	1.67	197.1	233.9	6.09	11.19	High	High	High	High	High	High	1	
FALSE	High	Master Prc	Q12996-1	Cleavage s	0	4.821	2	2	2	2	717	82.9	8.12	1.76	2	metabolic	nucleus	protein bir	Pf05843	1479	ENSG0000	CSTF3	11	mRNA surv	mRNA Pro	Cleavage o	13	0.845		0.511488167	134.69	386.8	457.8	133.21	34.29	High	High	High	High	High	High	1	
FALSE	High	Master Prc	P05386	60S acidic	0	39.283	67	3	9	2	114	11.5	4.32	43.93	3	cell organi	cytosol;rib	enzyme re	Pf00428	6176	ENSG0000	RPLP1	15	Ribosome	Cytoplasm	Peptide ch	34	0.843		0.126322107	13.57	2336.8	2772.5	8.72	4.95	High	High	High	High	High	High	1	
FALSE	Medium	Master Prc	Q8NAB2	Kelch repe	0.022	1.58	1	1	2	1	608	69.4	5.39	0	1	metabolic		catalytic a	Pf00651, P	143879		KBTBD3	11				0	0.843		0.380933667	38.92	352.4	523.5	14.35	41.72	High	High	High	High	High	High	1	
FALSE	High	Master Prc	Q9Y6E2	Basic leuci	0	28.67	16	7	12	7	419	48.1	6.68	21.37	7	cell differe	cytoplasm;	protein bir	Pf02020	28969	ENSG0000	BZW2	7				0	0.842		0.028425271	3.26	2302.9	2780.5	6.71	3.73	High	High	High	High	High	High	1	
FALSE	High	Master Prc	O75935	Dynactin s	0	11.397	15	2	2	2	186	21.1	5.47	7.88	2	cell divisio	chromosor	protein bir	Pf07426	11258	ENSG0000	DCTN3	9			COPI-indep	33	0.842		0.047046829	3.72	829	1099.3	12.17	8.25	High	High	High	High	High	High	1	
FALSE	Low	Master Prc	Q9NVM4	Protein arg	0.051	1.19	2	1	1	1	692	78.4	5.57	0	1	cell differe	cytoplasm;	catalytic a	Pf06325	54496	ENSG0000	PRMT7	16			RMTs metl	3	0.842		0.480618072	17.57	39.4	46.9	7.54	18.92	High	High	High	High	High	High	1	[R53;
FALSE	High	Master Prc	P10109	Adrenodox	0	8.196	14	3	3	3	184	19.4	5.83	2.35	3	metabolic	mitochond	metal ion l	Pf00111	2230	ENSG0000	FDX1	11			Endogenou	15	0.842		0.536977524	18.49	360.3	428.2	85.4	3.22	High	High	High	High	High	High	1	
FALSE	High	Master Prc	Q9NRN7	L-aminoad	0	10.026	11	3	3	3	309	35.8	6.8	10.74	3	metabolic	cytoplasm;	catalytic a	Pf01648	60496	ENSG0000	AASDHPPT	11	Pantothen		Vitamin B5	5	0.842		0.561164734	2.61	895.3	1031.9	8.85	10.11	High	High	High	High	High	High	1	
FALSE	High	Master Prc	P18206	Vinculin [C	0	281.376	43	49	128	49	1134	123.7	5.66	259.64	49	cell growth	cytoplasm;	protein bir	Pf01044	7414	ENSG0000	VCL	10	Bacterial ir	Integrin-m	Signaling b	37	0.841		0.035019641	1.5	38442.2	46612.6	8.45	2.11	High	High	High	High	High	High	1	
FALSE	High	Master Prc	Q9UKN8	General tra	0	7.827	4	3	3	3	822	91.9	6.65	9.61	3	cell organi	mitochond	catalytic a	Pf12657, P	9329	ENSG0000	GTF3C4	9			RNA Polym	6	0.841		0.25694336	25.09	60.9	74.1	16.3	11.87	High	High	High	High	High	High	1	
FALSE	High	Master Prc	P35908	Keratin, ty	0	83.5	23	13	41	8	639	65.4	8	91.69	13	cell death;	cytoskelet	protein bir	Pf00038, P	3849	ENSG0000	KRT2	12			Formation	3	0.841		0.338636584	15.67	3415.5	3848.8	15.8	12.23	High	High	High	High	High	High	1	
FALSE	High	Master Prc	Q9Y399	28S riboso	0	8.633	13	3	4	3	296	33.2	9.26	6.16	3	cell organi	membrane	structural	Pf00318	51116	ENSG0000	MRPS2	9	Ribosome		Mitochonc	7	0.841		0.846211168	14.05	446.9	531.2	34.2	6.65	High	High	High	High	High	High	1	
FALSE	High	Master Prc	O75663	TIP41-like	0	19.905	19	4	6	4	272	31.4	5.91	13.78	4	regulation	cytoplasm	protein bir	Pf04176	261726	ENSG0000	TIPRL	1				0	0.84		0.070713447	7.09	1387.4	1674.8	13.93	8.32	High	High	High	High	High	High	1	
FALSE	High	Master Prc	P46776	60S riboso	0	35.134	38	6	23	6	148	16.6	11	41.98	6	cell organi	cytosol;en	protein bir	Pf00828	6157	ENSG0000	RPL27A	11	Ribosome	Cytoplasm	Peptide ch	34	0.84		0.087902872	2.11	3804.5	4598.8	1.72	10.96	High	High	High	High	High	High	1	
FALSE	High	Master Prc	O75323-1	Protein Nip	0	11.345	16	3	4	3	286	33.7	9.36	8.21	3	metabolic	cytoplasm;	protein bir	Pf07978	2631	ENSG0000	GBAS; NIPS	7				0	0.84		0.272058332	27.9	1112.5	1236.7	15.33	7.43	High	High	High	High	High	High	1	
FALSE	Low	Master Prc	Q8IW35-1	Centrosom	0.068	1.064	1	1	1	1	865	96.9	5.02	0	1	cell organi	cytoplasm;	protein bir	Pf12799, P	79598	ENSG0000	CEP97	3			Anchoring	3	0.84		0.447128328	36.55	77.1	91.9	30.19	3.89	High	High	High	High	High	High	1	
FALSE	High	Master Prc	Q06323-1	Proteasom	0	57.429	59	12	25	12	249	28.7	6.02	58.31	12	metabolic	cytoplasm;	enzyme re	Pf02251, P	5720	ENSG0000	PSME1	14	Antigen pr	Proteasom	Regulation	151	0.839		0.015209311	4.34	8346.5	10224.9	3.97	1.81	High	High	High	High	High	High	1	
FALSE	High	Master Prc	Q4J6C6	prolyl endo	0	5.412	2	1	2	1	727	83.9	6.38	8.8	1	metabolic	cytoplasm;	catalytic a	Pf00326, P	9581	ENSG0000	PREPL	2				0	0.839		0.148075233	1.49	130.9	171.2	10.94	13.01	High	High	High	High	High	High	1	
FALSE	High	Master Prc	Q9Y3B4	Splicing fac	0	35.944	28	3	7	3	125	14.6	9.38	20.61	3	metabolic	nucleus;sp	protein bir	Pf00076, P	51639	ENSG0000	SF3B14; SF	2	Spliceosom		mRNA Spli	6	0.839		0.301349513	27.48	2495.3	3162.6	24.41	15.51	High	High	High	High	High	High	1	
FALSE	High	Master Prc	Q71UM5	40S riboso	0	19.282	38	3	11	1	84	9.5	9.45	23.88	3	cell organi	nucleus;rib	enzyme re	Pf01667	51065	ENSG0000	RPS27L	15	Ribosome		Peptide ch	37	0.839		0.917735656	0.43	473.7	521	27.22	6.63	High	High	High	High	High	High	1	
FALSE	High	Master Prc	P00338-1	L-lactate d	0	207.032	77	24	112	23	332	36.7	8.27	282.1	24	metabolic	cytoplasm;	catalytic a	Pf00056, P	3939	ENSG0000	LDHA	11	Metabolic	Cori Cycle;	Pyruvate n	16	0.836		0.005038027	3.12	30964.1	36678.2	4.05	3.75	High	High	High	High	High	High	1	
FALSE	High	Master Prc	P57088	Transmem	0	8.127	7	2	4	2	247	28	9.7	11.33	2	cell organi	endoplasm	protein bir	Pf03661	55161	ENSG0000	TMEM33	4				0	0.838		0.007607759	0.19	1268.3	1513.5	5.14	6.62	High	High	High	High	High	High	1	
FALSE	High	Master Prc	P33316-3	Deoxyuridi	0	36.186	33	7	18	7	252	26.5	9.36	36.27	7	metabolic	mitochond	catalytic a	Pf00692	1854	ENSG0000	DUT	15	Metabolic			2	0.838		0.054440608	2.89	2585.1	3392.1	7.28	6.68	High	High	High	High	High	High	1	
FALSE	High	Master Prc	Q93009	Ubiquitin c	0	34.013	11	10	14	10	1102	128.2	5.55	19.29	10	cell organi	chromosor	catalytic a	Pf00443, P	7874	ENSG0000	USP7	16	FoxO signa	Regulation	Synthesis c	29	0.837		0.007595853	0.38	1038.9	1241.3	4.06	1.33	High	High	High	High	High	High	1	
FALSE	High	Master Prc	Q9Y617-1	phosphose	0	43.197	27	9	18	8	370	40.4	7.66	37.36	9	metabolic	cytoplasm;	catalytic a	Pf00266	29968	ENSG0000	PSAT1	9	Glycine, se	Trans-sulfu	Serine bios	10	0.837		0.103443722	3.97	5365.5	6484.4	17.6	3.08	High	High	High	High	High	High	1	
FALSE	High	Master Prc	Q99615-1	DnaJ homo	0	61.88	27	12	21	12	494	56.4	6.96	56.06	12	metabolic	cytoplasm;	enzyme re	Pf00226, P	7266	ENSG0000	DNAJC7	17		Constitutiv	Regulation	5	0.837		0.216402103	8.84	4272.9	4303.9	9.95	13.72	High	High	High	High	High	High	1	
FALSE	High	Master Prc	Q5JPE7	NODAL mo	0	32.079	7	7	8	1	1267	139.4	5.76	22.71	7	regulation	endoplasm	protein bir	Pf09430, P	283820	ENSG0000	NOMO2	16				0	0.837		0.553103712	47.52	276.1	317.1	23.92	11.71	High	High	High	High	High	High	1	
FALSE	High	Master Prc	P62310	U6 snRNA-	0	7.2	12	1	2	1	102	11.8	4.7	2.72	1	cell organi	cytosol;nu	protein bir	Pf01423	27258	ENSG0000	LSM3	3	RNA degra		mRNA Spli	8	0.836		0.067662523	10.77	824.6	981.5	9.16	0.83	High	High	High	High	High	High	1	
FALSE	High	Master Prc	Q9NT62	ubiquitin-li	0	20.311	13	5	5	5	314	35.8	4.74	9.18	5	cell commu	cytoplasm;	catalytic a	Pf03986, P	64422	ENSG0000	ATG3	3	Autophagy	Nanoparti	Macroautc	5	0.836		0.115078905	13.01	1392.9	1686.5	6.3	8.32	High	High	High	High	High	High	1	

FALSE	High	Master Prc	Q53H12	Acylglycer	0	13.613	13	4	5	4	422	47.1	8.09	7.43	4	cell organi	cytosol;me	catalytic a	Pf00781	55750	ENSG0000	AGK	7	Glycerolipi		Signaling b	10	0.836		0.122546648	5.61	425.8	555.2	8.04	13.33	High	High	High	High	High	High	1	
FALSE	High	Master Prc	Q9BQ67	Glutamate	0	17.992	9	3	6	3	446	49.4	4.92	14.19	3	cell organi	chromosor	DNA bindi	Pf00400, P	83743	ENSG0000	GRWD1	19				0	0.836		0.254638118	10.78	1491.3	1766.7	11.25	3.65	High	High	High	High	High	High	1	
FALSE	High	Master Prc	Q9BX40	protein LSI	0	20.838	15	2	2	2	385	42	9.69	14.03	2	developme		RNA bindi	Pf09532, P	149986	ENSG0000	LSM14B	20				0	0.836		0.297609116	11.73	373.8	387.2	13.71	14.74	High	High	High	High	High	High	1	
FALSE	High	Master Prc	P11766	alcohol del	0	24.347	14	6	8	6	374	39.7	7.49	18.65	6	metabolic	cytoplasm;	catalytic a	Pf00107, P	128	ENSG0000	ADH5	4	Metabolisr	Folate-Alc	Ethanol ox	15	0.835		0.00990969	4.32	206.3	244.4	1.41	2.41	High	High	High	High	High	High	1	
FALSE	High	Master Prc	Q96S55-1	ATPase WI	0	26.454	9	4	6	4	665	72.1	6.1	15.17	4	metabolic	membrane	catalytic a	Pf00004, P	56897	ENSG0000	WRNIP1	6				0	0.835		0.230065108	23.68	848.8	1018	20.4	12.61	High	High	High	High	High	High	1	
FALSE	High	Master Prc	Q9H4A3	Serine/thre	0	6.569	1	2	2	2	2382	250.6	6.34	4.21	2	metabolic	cytoplasm;	catalytic a	Pf00069, P	65125	ENSG0000	WNK1	12			Stimuli-ser	3	0.835		0.33101191	32.34	310.1	361	22.6	6.45	High	High	High	High	High	High	1	
FALSE	High	Master Prc	Q7Z460-4	Isoform 4 (	0	32.702	7	7	8	7	1477	163	8.53	5.66	7	cell divisio	chromosor	protein bir	Pf02985, P	23332	ENSG0000	CLASP1	2		Regulation		1	0.835		0.739204847	5.78	2271.1	2568.6	44.11	3.6	High	High	High	High	High	High	1	
FALSE	High	Master Prc	O60271	C-jun-amir	0	10.046	4	3	3	3	1321	146.1	5.15	2.68	3	cell differe	cytoplasm;	protein bir	Pf09744	9043	ENSG0000	SPAG9	17			CDO in my	3	0.834		0.016098348	5.57	318.6	388.3	6.41	2.26	High	High	High	High	High	High	1	
FALSE	High	Master Prc	Q9Y6D9	mitotic spi	0	19.805	9	5	5	5	718	83	5.92	10.05	5	cell divisio	chromosor	protein bir	Pf05557	8379	ENSG0000	MAD1L1	7	Progesterc	Cell Cycle	Amplificati	20	0.834		0.0164638	4.33	909.4	1090.3	3.2	6.45	High	High	High	High	High	High	1	
FALSE	High	Master Prc	P61619	Protein tra	0	18.483	13	6	9	6	476	52.2	8.06	17.54	6	cell growth	cytosol;en	protein bir	Pf00344, P	29927	ENSG0000	SEC61A1	3	Vibrio chol		ER-Phagos	15	0.834		0.149834894	7	269.5	361.2	12.78	12.3	High	High	High	High	High	High	1	
FALSE	High	Master Prc	Q9NTM9	Copper hoi	0.003	3.118	4	1	1	1	273	29.3	8.18	2.22	1	cell organi	cytoplasm;	metal ion I	Pf03932	51076	ENSG0000	CUTC	10			Ion transp	3	0.834		0.413707786	16.42	882.6	1019	22.6	5.74	High	High	High	High	High	High	1	
FALSE	High	Master Prc	P15328	Folate rece	0	58.803	37	9	33	9	257	29.8	7.97	92.8	9	cell commu	cell surface	receptor a	Pf03024	2348	ENSG0000	FOLR1	11	Antifolate	Folate Met	COPI-medi	13	0.833		0.245527256	5	11285.4	12810.6	4.36	10.61	High	High	High	High	High	High	1	
FALSE	High	Master Prc	P40121	Macrophag	0	66.737	34	9	19	9	348	38.5	6.19	49.57	9	cell organi	cytoplasm;	protein bir	Pf00626	822	ENSG0000	CAPG	2		TYROBP Ca		1	0.833		0.343992498	17.79	2753.6	3484.1	19.34	3.69	High	High	High	High	High	High	1	
FALSE	High	Master Prc	Q86SQ4	Adhesion (	0.002	4.019	2	1	1	1	1221	136.6	7.87	1.66	1	cell differe	membrane	protein bir	Pf00002, P	57211	ENSG0000	GPR126; A	6				0	0.833		0.352248152	34	15.9	19.5	5.14	31.47	High	High	High	High	High	High	1	
FALSE	High	Master Prc	P43490	nicotinami	0	13.881	10	3	3	3	491	55.5	7.15	12.55	3	cell commu	cytoplasm;	catalytic a	Pf04095	10135	ENSG0000	NAMPT	7	Nicotinate	Adipogene	BMAL1:CL(	14	0.833		0.458840002	50.38	245.9	328.9	20.2	29.01	High	High	High	High	High	High	1	
FALSE	High	Master Prc	P55211-1	caspase-9	0	11.037	9	2	3	2	416	46.3	6.05	5.51	2	cell death;	cytoplasm;	catalytic a	Pf00619, P	842	ENSG0000	CASP9	1	Parkinson'	Nucleotide	AKT phosp	68	0.833		0.497119888	9.01	450.2	517.3	19.46	2.9	High	High	High	High	High	High	1	
FALSE	High	Master Prc	O14653-1	Golgi SNAI	0	13.18	22	3	3	3	212	24.8	8.06	5.61	3	cell organi	cytosol;en	protein bir	Pf12352	9570	ENSG0000	GOSR2	17	SNARE inte		Intra-Golgi	16	0.832		0.361621896	8.36	40.1	47.8	6.76	17.37	High	High	High	High	High	High	1	
FALSE	High	Master Prc	P37198	nuclear po	0	9.907	6	3	3	3	522	53.2	5.31	8.31	3	cell death;	cytoplasm;	protein bir	Pf05064	23636	ENSG0000	NUP62	19	RNA trans		Rev-media	64	0.832		0.386265113	38.94	374.3	431.3	25.98	14.84	High	High	High	High	High	High	1	
FALSE	High	Master Prc	Q96JP2	Unconvent	0.002	4	1	1	1	1	1530	167	8.41	3.25	1		cytoplasm;	catalytic a	Pf00063, P	80022		LOC10013	17				0	0.832		0.389088083	40.08	274.9	348.9	13.26	20.77	High	High	High	High	High	High	1	
FALSE	High	Master Prc	P31946	14-3-3 pro	0	111.212	58	15	60	6	246	28.1	4.83	120.32	15	cell organi	cytoplasm;	protein bir	Pf00244	7529	ENSG0000	YWHAB	20	Hippo sign	Cell Cycle;	MAP2K an	68	0.831		0.138441114	1.69	3747.2	4333.7	3.71	9.16	High	High	High	High	High	High	1	
FALSE	High	Master Prc	Q9UID3-1	Vacuolar p	0	9.218	3	1	1	1	782	86	6.47	5.02	1	cell organi	cytosol;en	protein bir	Pf04124, P	738	ENSG0000	VPS51	11			Retrograde	4	0.831		0.50423883	53.24	254.6	321.5	21.76	26.45	High	High	High	High	High	High	1	
FALSE	High	Master Prc	A0AVT1-1	Ubiquitin-l	0	19.544	8	8	9	8	1052	117.9	6.14	10.02	8	metabolic	cytoplasm;	catalytic a	Pf00899, P	55236	ENSG0000	UBA6	4	Ubiquitin r		Synthesis (	9	0.831		0.75491019	3.33	1546.8	1888.5	44.49	2.97	High	High	High	High	High	High	1	
FALSE	High	Master Prc	P62910	60S riboso	0	64.331	52	11	38	11	135	15.9	11.33	97	11	cell organi	cytosol;me	RNA bindi	Pf01655	6161	ENSG0000	RPL32	3	Ribosome	Cytoplasm	Peptide ch	34	0.83		0.033162928	3.95	12664	14236.1	8.17	5.6	High	High	High	High	High	High	1	
FALSE	High	Master Prc	Q9P275	Ubiquitin (	0	16.716	4	3	5	3	1121	122.6	9.67	10.77	3	metabolic	nucleus	catalytic a	Pf00443, P	57602		USP36	17				0	0.83		0.060573681	6.15	357.4	446.4	13.88	8.99	High	High	High	High	High	High	1	
FALSE	High	Master Prc	O60869-1	Endothelia	0	11.88	27	4	7	4	148	16.4	9.95	12.58	4	cell differe	cytoplasm;	catalytic a	Pf01381, P	8721	ENSG0000	EDF1	9				0	0.83		0.673300504	6.89	1805.4	2164	13.59	10.79	High	High	High	High	High	High	1	
FALSE	High	Master Prc	Q9GZZ1-1	N-alpha-ac	0	45.413	53	10	13	10	169	19.4	8.81	28.92	10	cell organi	cytoplasm;	catalytic a	Pf00583, P	80218	ENSG0000	NAA50	3		Metapathv		1	0.83		0.890373695	20.28	1280.5	1444	34.51	5.93	High	High	High	High	High	High	1	
FALSE	High	Master Prc	Q07866-1	Kinesin ligl	0	8.946	5	3	4	1	573	65.3	6.2	8.99	3	cell organi	cytoplasm;	catalytic a	Pf08317, P	3831	ENSG0000	KLC1	14	Salmonella		COPI-depe	16	0.829		0.070264592	12.61	174.8	227	9.68	5.15	High	High	High	High	High	High	1	
FALSE	High	Master Prc	Q9Y606	tRNA pseu	0	8.64	7	3	3	3	427	47.4	8.41	3.56	3	cell organi	mitochond	catalytic a	Pf01416	80324	ENSG0000	PUS1	12			tRNA modi	3	0.829		0.132067583	7.4	1104.5	1398.8	14.21	4.47	High	High	High	High	High	High	1	
FALSE	High	Master Prc	O76094	Signal reco	0	43.497	16	8	11	8	671	74.6	9.26	31.84	8	cell organi	cytoplasm;	protein bir	Pf08492, P	6731	ENSG0000	SRP72	4	Protein ex		SRP-depen	4	0.829		0.176868671	0.41	2888.3	3510	1.82	22.99	High	High	High	High	High	High	1	
FALSE	High	Master Prc	P10301	Ras-relatec	0	11.979	13	2	2	2	218	23.5	6.93	5.54	2	cell differe	membrane	catalytic a	Pf00071, P	6237	ENSG0000	RRAS	19	Proteoglyc	G Protein S	Sema4D m	28	0.829		0.438644774	30.17	438.7	513.3	15.78	11.47	High	High	High	High	High	High	1	
FALSE	Low	Master Prc	P35125-1	Ubiquitin (	0.05	1.197	1	1	1	1	1406	158.6	7.71	2.03	1	metabolic	cytoplasm;	catalytic a	Pf00443, P	9098	ENSG0000	USP6	17				0	0.828		0.386302574	40.66	713.8	880.7	19.08	20.46	High	High	High	High	High	High	1	
FALSE	High	Master Prc	P49720	proteasom	0	22.576	24	4	9	4	205	22.9	6.55	19.01	4	metabolic	cytoplasm;	catalytic a	Pf00227	5691	ENSG0000	PSMB3	17; CHR_H	Proteasom	Proteasom	Regulation	150	0.827		0.134486788	13.08	2895.8	3502.5	18.8	1.21	High	High	High	High	High	High	1	
FALSE	High	Master Prc	Q9NUL5	Repressor	0	5.23	6	1	1	1	291	33.1	7.25	3.08	1	defense re	cytoplasm;	protein bir	Pf15135	55337	ENSG0000	C19orf66	19				0	0.827		0.387991964	39.18	238.9	270.1	21.74	12.08	High	High	High	High	High	High	1	
FALSE	High	Master Prc	Q9Y5X1	Sorting ne	0	17.825	10	3	3	3	595	66.6	5.58	10.35	3	cell divisio	cytoplasm;	protein bir	Pf00018, P	51429	ENSG0000	SNX9	6			Golgi Asso	6	0.827		0.414660083	47.97	149	204.1	34.07	28.67	High	High	High	High	High	High	1	
FALSE	High	Master Prc	Q9NRP4	Succinate (	0	6.349	25	2	2	2	125	14.6	9.07	7.4	2	cell organi	mitochond		Pf05347, P	57001	ENSG0000	ACN9; SDH	7				0	0.827		0.595500351	19.39	94	103.5	6.77	29.84	High	High	High	High	High	High	1	
FALSE	High	Master Prc	O75691	Small subu	0	24.461	3	6	8	6	2785	318.2	7.39	16.1	6	metabolic	cytoplasm;	protein bir	Pf07539	27340	ENSG0000	UTP20	12			Major path	5	0.826		0.053933416	7.73	1515.7	1840.6	11.48	9.48	High	High	High	High	High	High	1	
FALSE	High	Master Prc	P62249	40S riboso	0	51.504	72	13	31	13	146	16.4	10.21	52.05	13	cell organi	cytosol;me	protein bir	Pf00380	6217	ENSG0000	RPS16	19	Ribosome	Cytoplasm	Peptide ch	38	0.826		0.06101115	11.26	11018.8	13344.9	6.23	2.36	High	High	High	High	High	High	1	
FALSE	High	Master Prc	Q9H3S7	Tyrosine-p	0	21.641	4	6	7	6	1636	178.9	6.92	10.3	6	cell organi	cytoplasm;	catalytic a	Pf00102, P	25930	ENSG0000	PTPN23	3				0	0.825		0.169947034	5.63	1177	1401.8	8.61	3.74	High	High	High	High	High	High	1	
FALSE	High	Master Prc	P26374	Rab protei	0.006	2.421	1	1	1	1	656	74	4.93	2.42	1	metabolic	cytoplasm;	catalytic a	Pf00996, P	1122	ENSG0000	CHML	1			RAB GEFs (	7	0.824		0.018025644	6.17	479.2	581.4	15.97	8.85	High	High	High	High	High	High	1	
FALSE	High	Master Prc	Q13162	Peroxiredo	0	98.548	62	12	48	9	271	30.5	6.29	134.66	12	cell organi	cytoplasm;	antioxidan	Pf00578, P	10549	ENSG0000	PRDX4	X		Selenium M	Neutrophil	4	0.824		0.098333312	9.02	8683.3	10989	5.32	9.64	High	High	High	High	High	High	1	
FALSE	High	Master Prc	Q9NRW3	DNA dC->d	0	7.534	7	1	1	1	190	22.8	7.59	2.67	1	defense re	cytoplasm;	catalytic a	Pf05240, P	27350	ENSG0000	APOBEC3C	22			Formation	4	0.824		0.261557573	1.54	125.8	139	6.04	10.8	High	High	High	High	High	High	1	
FALSE	High	Master Prc	Q9H583	HEAT repe	0	21.155	4	6	6	6	2144	242.2	6.54	10.16	6	metabolic	membrane	protein bir	Pf08146, P	55127	ENSG0000	HEATR1	1	Ribosome		Major path	6	0.824		0.488617766	6.94	638.3	770.5	19.18	3.69	High	High	High	High	High	High	1	
FALSE	High	Master Prc	O60256-1	Phosphorit	0	22.928	22	6	7	5	369	40.9	7.44	13.59	6	metabolic		catalytic a	Pf13793, P	5636	ENSG0000	PRPSAP2	17				0	0.823		0.013662282	1.72	1097.8	1387.1	5	3.78	High	High	High	High	High	High	1	
FALSE	High	Master Prc	P42677	40S riboso	0	23.008	38	3	13	1	84	9.5	9.45	28.46	3	cell organi	cytosol;nu	DNA bindi	Pf01667	6232	ENSG0000	RPS27	1	Ribosome	Cytoplasm	Peptide ch	54	0.823		0.931710737	9.9	1668.2	1964.8	28.76	12.7	High	High	High	High	High	High	1	
FALSE	High	Master Prc	O14530	Thioredoxi	0	11.114	14	3	4	3	226	26.5	5.88	2.9	3	cellular ho	cytoplasm;	catalytic a	Pf00085	10190	ENSG0000	TXNDC9	2				0	0.822		0.062411378	10.14	548.2	667.2	3.07	6.17	High	High	High	High	High	High	1	
FALSE	High	Master Prc	O75822	Eukaryotic	0	36.164	32	7	11	7	258	29	4.83	23.93	7	cell organi	cytoplasm;	protein bir	Pf08597	8669	ENSG0000	EIF3J	15	RNA trans	Translatior	Formation	13	0.822		0.091752356	11.82	2378	2651	9.55	6.28	High	High	High	High	High	High	1	
FALSE	High	Master Prc	Q9UMX5	Neudesin [	0	5.135	14	2	3	2	172	18.8	5.69	5.98	2	regulation	extracellul	metal ion I	Pf00173	29937	ENSG0000	NENF	1				0	0.822		0.238919584	18.38	636.6	800	9.39	16.55	High	High	High	High	High	High	1	
FALSE	High	Master Prc	Q7L1Q6-1	Basic leuci	0	33.211	17	9	20	9	419	48	5.92	37.2	9	metabolic	cytoplasm;	protein bir	Pf02020	9689	ENSG0000	BZW1	2				0	0.821		0.008061346	2.98	7086.2	8629.3	6.71	2.6	High	High	High	High	High	High	1	
FALSE	High	Master Prc	Q13618	Cullin-3 [O	0	5.414	3	3	4	3	768	88.9	8.48	7.7	3	cell death;	cytoplasm;	catalytic a	Pf00888, P	8452	ENSG0000	CUL3	2	Ubiquitin r		Hedgehog	22	0.821		0.180157013	18.15	1030.1	1303	14.08	4.3	High	High	High	High	High	High	1	
FALSE	High	Master Prc	P23396-1	40S riboso	0	129.167	77	18	77	18	243	26.7	9.66	140.05	18	cell death;	cytoplasm;	catalytic a	Pf00189, P	6188	ENSG0000	RPS3	11	Ribosome	Cytoplasm	Peptide ch	38	0.82		0.001842065	1.15	17758.8	21207.5	3.03	2.7	High	High	High	High	High	High	1	
FALSE	High	Master Prc	P23284	peptidyl-pı	0	90.537	54	14	47	13	216	23.7	9.41	98.5	14	metabolic	endoplasm	catalytic a	Pf00160	5479	ENSG0000	PPIB	15		Prolactin S	Collagen b	4	0.819		0.117610108	3.51	12228.1	14356.5	4.22	7.19	High	High	High	High	High	High	1	
FALSE	High	Master Prc	Q7L576-1	cytoplasmi	0	18.643	5	5	5	5	1253	145.1	6.9	7.08	5	cell differe	cytoplasm;	protein bir	Pf05994, P	23191	ENSG0000	CYFIP1	15	RNA trans	Brain-Deriv	Regulation	16	0.819		0.33564815	37.21	490.6	512.8	30.47	15.18	High	High	High	High	High	High	1	[R826]
FALSE	High	Master Prc	P41250	Glycine--tF	0	123.221	42	24	43	24	739	83.1	7.03	123.52	24	metabolic	cytoplasm;	catalytic a	Pf00458, P	2617	ENSG0000	GARS	7	Aminoacyl		Mitochond	6	0.818		0.057340564	7.41	12855.9	15629.8	3.46	5.14	High	High	High	High	High	High	1	
FALSE	High	Master Prc	O15498	Synaptobre	0	14.803	24	4	5	4	198	22.4	6.92	11.52	4	cell organi	cytoplasm;	catalytic a	Pf00957, P	10652	ENSG0000	YKT6	7	SNARE inte		COPI-medi	12	0.818		0.123218858	13.28	916.9	1183.1	12.57	8.51	High	High	High	High	High	High	1	
FALSE	High	Master Prc	Q15181	Inorganic p	0	184.456	79	21	52	20	289	32.6	5.86	169.82	21	metabolic	cytoplasm;	catalytic a	Pf00719	5464	ENSG0000	PPA1	10	Oxidative p		Cytosolic t	7	0.818		0.165492665	20.6	17761.9	23792.3	10	13.67	High	High	High	High	High	High	1	
FALSE	High	Master Prc	P09211	Glutathion	0	149.522	64	12	45	12	210	23.3	5.64	169.19	12	cell commu	cytoplasm;	antioxidan	Pf00043, P	2950	ENSG0000	GSTP1	11	Glutathion	NRF2 path	Glutathion	22	0.818		0.285351739	32.52	13987.9	17116.2	15.61	13.76	High	High	High	High	High	High	1	
FALSE	High	Master Prc	Q7Z406-1	myosin-14	0	38.221	6	8	11	3	1995	227.7	5.6	32.43	8	cell organi	cytoplasm;	catalytic a	Pf00063, P	79784	ENSG0000	MYH14	19	Salmonella		RHO GTPa:	17	0.818		0.483073813	20.28	288.2	352.4	29.19	6.51	High	High	High	High	High	High	1	
FALSE	High	Master Prc	P40261	Nicotinami	0	6.245	10	3	4	3	264	29.6	5.74	7.09	3	metabolic	cytoplasm;	catalytic a	Pf01234, P	4837	ENSG0000	NNMT	11	Nicotinate	Metapathv	Metabolisr	15	0.817		0.006508089	0.05	1205.3	1481.9	3.78	0.81	High	High	High	High	High	High	1	
FALSE	High	Master Prc	Q9Y508-1	E3 ubiquiti	0	15.844	18	3	5	3	228	25.7	7.25	12.82	3	cell differe	cytoplasm;	catalytic a	Pf00097, P	55905	ENSG0000	RNF114	20			Antigen pr	4	0.817		0.015453107	1.4	1303	1500.3	6.39	5.08	High	High	High	High	High	High	1	
FALSE	High	Master Prc	Q15382	GTP-bindir	0	12.36	16	3	4	3	184	20.5	5.92	9.02	3	regulation	cytoplasm;	catalytic a	Pf00071, P	6009	ENSG0000	RHEB	7	Phospholip	Target Of I	Energy dep	30	0.817		0.140838252	3.63	1201.5	1598.1	20.72	8.29	High	High	High	High	High	High	1	
FALSE	High	Master Prc	O15144	Actin-relat	0	24.248	32	7	10	7	300	34.3	7.36	21.28	7	cell organi	cytoplasm;	protein bir	Pf04045	10109	ENSG0000	ARPC2	2	Bacterial ir	Pathogenic	EPHB-med	23	0.817		0.88468185	19.12	2576.9	3153	30.88	6.13	High	High	High	High	High	High	1	
FALSE	High	Master Prc	Q92538	Golgi-speci	0	53.481	6	7	11	7	1859	206.3	5.73	30.91	7	cell organi	cytoplasm;	protein bir	Pf01369, P	8729	ENSG0000	GBF1	10	Endocytosi		Clathrin de	18	0.815		0.041799527	4	913.9	1138.9	6.69	3	High	High	High	High	High	High	1	
FALSE	High	Master Prc	P46782	40S riboso	0	48.619	46	9	21	9	204	22.9	9.72	30.52	9	cell organi	cytosol;me	protein bir	Pf00177	6193	ENSG0000	RPS5	19	Ribosome	Cytoplasm	Peptide ch	38	0.815		0.054931785	11.83	4796.1	5414.5	14.67	6.87	High	High	High	High	High	High	1	
FALSE	High	Master Prc	Q99426	tubulin-fol	0	13.826	26	6	8	6	244	27.3	5.15	9.23	6	cell differe	cytoplasm;	protein bir	Pf01302, P	1155	ENSG0000	TBCB	19			Post-chape	3	0.815		0.201445035	16.37	2833.2	3475.1	11.22	4.84	High	High	High	High	High	High	1	
FALSE	High	Master Prc	Q8WX92	Negative e	0	34.13	13	5	7	5	580	65.7	6.13	20.56	5	metabolic	cytoplasm;	protein bir	Pf06209	25920		COBRA1; N	9		Initiation c	HIV elonga	26	0.814		0.01737157	4.1	660.5	833.9	5.11	7.66	High	High	High	High	High	High	1	
FALSE	High	Master Prc	Q86TI2	Dipeptidyl	0.004	2.719	2	1	1	1	863	98.2	6.46	2.1	1	metabolic	cytoplasm;	catalytic a	Pf00326, P	91039	ENSG0000	DPP9	19		Lung fibro:		1	0.814		0.044104699	3	172.9	216.7	7.55	11.36	High	High	High	High	High	High	1	
FALSE	High	Master Prc	P63244	Receptor o	0	105.524	67	15	46	15	317	35.1	7.69	152.02	15	cell death;	cytoplasm;	enzyme re	Pf00400	10399	ENSG0000	GNB2L1; R	5	Measles	Androgen	Regulation	11	0.814		0.045190053	10.89	14074.6	16658.3	7.77	3.56	High	High	High	High	High	High	1	
FALSE	Medium	Master Prc	O95263-1	High affini	0.026	1.468	1	1	1	1	885	98.9	6.83	2.06	1	metabolic	cytosol	catalytic a	Pf00072, P	8622	ENSG0000	PDE8B	5	Purine me	G Protein S	G alpha (s)	7	0.814		0.883088356	3.8	325.8	410.7	41.78	3.89	High	High	High	High	High	High	1	
FALSE	High	Master Prc	O94905-1	Erlin-2 [OS	0	22.509	18	5	5	2	339	37.8	5.62	14.01	5	metabolic	cytosol;en	catalytic a	Pf01145	11160	ENSG0000	ERLIN2	8			ABC-family	11	0.813		0.340344269	39.33	119.9	153.3	8.97	21.08	High	High	High	High	High	High	1	
FALSE	High	Master Prc	P05388	60S acidic	0	121.111	52	14	51	14	317	34.3	5.97	127.04	14	cell organi	cytoplasm;	protein bir	Pf00428, P	6175	ENSG0000	RPLP0	12	Ribosome	Cytoplasm	Peptide ch	39	0.812		0.073279557	8.43	13357.9	16706.9	5.16	7.37	High	High	High	High	High	High	1	
FALSE	High	Master Prc	Q8NBN3-1	Transmem	0.004	2.654	2	1	1	1	555	63.4	6.74	2.58	1	transport	cytosol;Go		Pf06814	25963	ENSG0000	TMEM87A	15				0	0.812		0.418966625	14.66	602.7	707.2	9.38	15.4	High	High	High	High	High	High	1	
FALSE	High	Master Prc	O95218-2	Isoform 2 (	0	22.722	19	7	12	7	320	36.3	9.76	20.38	7	metabolic	nucleus	metal ion I	Pf00641	9406	ENSG0000	ZRANB2	1				0	0.81		0.142156185	15.97	813.6	1003.8	4.59	9.39	High	High	High	High	High	High	1	
FALSE	High	Master Prc	Q13405	39S riboso	0	12.696	32	5	7	5	166	19.2	9.45	9.58	5	cell organi	membrane	protein bir	Pf05046	740	ENSG0000	MRPL49	11			Mitochond	6	0.81		0.169769729	21.77	1509.8	1864	9.54	9.85	High	High	High	High	High	High	1	
FALSE	High	Master Prc	Q53GQ0	Very-long-(	0	12.899	9	3	4	3	312	34.3	9.32	12.01	3	cell organi	endoplasm	catalytic a	Pf00106, P	51144	ENSG0000	HSD17B12	11	Fatty acid (		Androgen	13	0.81		0.222914758	21.87	340.1	420.1	13.57	15.07	High	High	High	High	High	High	1	
FALSE	High	Master Prc	Q9UI30	Multifunct	0	29.931	42	4	8	4	125	14.2	5.26	23.14	4	cell organi	cytoplasm;	catalytic a	Pf03966	51504	ENSG0000	TRMT112	11			tRNA modi	13	0.81		0.243846104	12.97	2411.5	2788	10.54	15.2	High	High	High	High	High	High	1	
FALSE	High	Master Prc	P14854	Cytochrom	0	28.606	67	7	18	7	86	10.2	7.05	37.5	7	metabolic	membrane	catalytic a	Pf02297	1340	ENSG0000	COX6B1	19	Non-alcoh	Electron Tr	TP53 Regu	17	0.809		0.446603634	49.93	7376.7	9116.7	23.81	19.75	High	High	High	High	High	High	1	
FALSE	High	Master Prc	P49643-1	DNA prima	0	17.494	10	5	5	5	509	58.8	7.91	6.44	5	cell organi		catalytic a	Pf04104	5558	ENSG0000	PRIM2	6	Pyrimidine	G1 to S cel	Activation	31	0.808		0.279019489	5.54	1292.3	1399.4	10.24	14.53	High	High	High	High	High	High	1	
FALSE	High	Master Prc	Q9UBU8-1	Mortality f	0.003	3.395	3	1	1	1	362	41.4	9.28	2.53	1	cell organi	nucleus	protein bir	Pf05712, P	10933	ENSG0000	MORF4L1	15		Pathways /	HATs acety	4	0.808		0.880226447	24.3	361.9	447.8	54.78	7.48	High	High	High	High	High	High	1	
FALSE	High	Master Prc	Q92882	osteoclast-	0	6.366	14	2	2	2	214	23.8	5.68	5.68	2	regulation	cytoplasm;	protein bir	Pf00018, P	26578	ENSG0000	OSTF1	9			Neutrophil	3	0.807		0.20619325	18.22	133.5	146.5	7.57	16.37	High	High	High	High	High	High	1	

FALSE	Medium	Master Prc	Q16342-1	Programm	0.011	2.019	3	1	1	1	344	38.6	5.38	1.66	1	cell death;	cytoplasm;	DNA bindi	Pf01753, P	5134	ENSG0000	PDCD2	6				0	0.806	0.040371787	12.94	860	1067.7	18.28	8.63	High	High	High	High	High	High	1	
FALSE	High	Master Prc	Q12965	unconvent	0	75.927	16	13	20	13	1108	127	8.92	56.38	13	cell differe	cytoplasm;	catalytic a	Pf00018, P	4643	ENSG0000	MYO1E	15		Primary Fc		1	0.806	0.155375488	1.88	4513.9	5601	6.63	5.34	High	High	High	High	High	High	1	
FALSE	High	Master Prc	Q9H8Y5	Ankyrin re	0	22.348	8	4	5	4	726	80.9	8.41	17.48	4	metabolic	cytoplasm;	catalytic a	Pf00023, P	55139	ENSG0000	ANKZF1	2				0	0.806	0.165906294	16.44	340.1	422	8.56	7.12	High	High	High	High	High	High	1	
FALSE	High	Master Prc	O95352	Ubiquitin-l	0	19.912	8	4	4	4	703	77.9	6.24	11.02	4	cell commu	cytoplasm;	catalytic a	Pf00899	10533	ENSG0000	ATG7	3	Autophagy	Nanoparti	Signaling b	15	0.806	0.997022955	0.29	95.2	118.3	36.14	3.55	High	High	High	High	High	High	1	
FALSE	High	Master Prc	P59998	Actin-relat	0	8.705	17	3	5	3	168	19.7	8.43	6.62	3	cell organi	cytoplasm;	protein bir	Pf05856	10093	ENSG0000	ARPC4	3	Shigellosis	Pathogeni	EPHB-med	23	0.805	0.016594978	3.25	1276	1611.2	3.96	2.45	High	High	High	High	High	High	1	
FALSE	High	Master Prc	P21266	glutathion	0	70.253	50	12	24	12	225	26.5	5.54	65.88	12	metabolic	cytoplasm;	catalytic a	Pf00043, P	2947	ENSG0000	GSTM3	1	Drug meta	NRF2 path	Glutathion	12	0.805	0.0509477	0.12	5974	7238.7	6.93	1.51	High	High	High	High	High	High	1	
FALSE	High	Master Prc	O43719	HIV Tat-sp	0	17.644	9	7	7	7	755	85.8	4.4	11.7	7	metabolic	nucleus;sp	RNA bindi	Pf00076, P	27336	ENSG0000	HTATSF1	X				0	0.805	0.774198691	2.87	2010.4	2344.8	46.77	5.89	High	High	High	High	High	High	1	
FALSE	High	Master Prc	P32320	cytidine de	0	27.286	45	5	9	5	146	16.2	6.92	31.7	5	cell organi	cytosol;ext	catalytic a	Pf00383, P	978	ENSG0000	CDA	1	Pyrimidine	Fluoropyri	Pyrimidine	11	0.804	0.167700521	22.93	2517.1	3248.5	11.12	11.88	High	High	High	High	High	High	1	
FALSE	High	Master Prc	Q8N3Z3-1	GTP-bindir	0.002	3.898	5	1	1	1	284	32.1	9.42	2.31	1		membrane	catalytic a	Pf00009, P	29083	ENSG0000	GTPBP8	3				0	0.804	0.24166406	20.15	144.1	179.4	15.67	21.01	High	High	High	High	High	High	1	
FALSE	Medium	Master Prc	O14925	Mitochonc	0.026	1.481	4	1	2	1	209	21.9	8.6	3.94	1	transport	membrane	protein bir	Pf02466	10028793	ENSG0000	TIMM23	10			Mitochonc	2	0.804	0.258452996	16.17	1030.1	1282	45.91	12.89	High	High	High	High	High	High	1	
FALSE	High	Master Prc	Q9BVT8	transmeml	0	9.066	9	2	4	2	246	26.2	5.72	4.02	2	metabolic	cytoplasm;	protein bir	Pf00240, P	83590	ENSG0000	TMUB1	7				0	0.804	0.394893594	2.07	1630.1	1727	10.87	20.12	High	High	High	High	High	High	1	
FALSE	High	Master Prc	Q07020	60S riboso	0	37.675	42	7	14	7	188	21.6	11.72	27.9	7	cell organi	cytoplasm;	RNA bindi	Pf00828	6141	ENSG0000	RPL18	19	Ribosome	Cytoplasm	Peptide ch	34	0.802	0.072669139	10.59	4852.2	6036.2	4.31	6.62	High	High	High	High	High	High	1	
FALSE	High	Master Prc	P53609	Geranylger	0.004	2.723	3	1	1	1	377	42.3	6.83	2.1	1	metabolic		catalytic a	Pf00432, P	5229	ENSG0000	PGGT1B	5				0	0.801	0.189704493	7.29	485.1	563.5	6.92	11.1	High	High	High	High	High	High	1	
FALSE	High	Master Prc	O95747	Serine/thre	0	14.15	8	4	7	4	527	58	6.43	13.34	4	metabolic	cytoplasm;	catalytic a	Pf00069, P	9943	ENSG0000	OXSR1	3				0	0.801	0.439313214	25.54	1793.6	2237.8	17.97	10.04	High	High	High	High	High	High	1	
FALSE	High	Master Prc	O14744	Protein arg	0	23.115	14	7	10	7	637	72.6	6.29	12.71	7	cell organi	cytoplasm;	catalytic a	Pf02475, P	10419	ENSG0000	PRMT5	14	RNA trans		snRNP Ass	13	0.799	0.104404727	9.42	2578.3	3154.6	22.08	4.81	High	High	High	High	High	High	1	
FALSE	High	Master Prc	Q9Y5P6	Mannose-1	0	14.349	15	3	4	3	360	39.8	6.61	12.88	3	metabolic	cytoplasm;	catalytic a	Pf00132, P	29925	ENSG0000	GMPPB	3	Metabolic		Synthesis c	9	0.799	0.191673403	27.47	624.6	748.8	15.59	10.79	High	High	High	High	High	High	1	
FALSE	High	Master Prc	Q9NWT1	p21-activa	0	18.722	10	4	5	4	392	43.9	8.91	17.61	4	cell prolife	nucleus	protein bir	Pf00400	55003	ENSG0000	PAK1IP1	6				0	0.798	0.010153923	3.65	2461.2	3090.3	1.16	4.34	High	High	High	High	High	High	1	
FALSE	High	Master Prc	Q9BQC3	2-(3-aminc	0	4.727	7	2	2	2	489	52.1	5.53	1.83	2	metabolic	cytosol	catalytic a	Pf01866	1802	ENSG0000	DPH2	1			Synthesis c	4	0.796	0.042554528	9.53	41.6	49.1	12.56	14.47	High	High	High	High	High	High	1	
FALSE	High	Master Prc	Q9UKD2	mRNA turr	0	33.24	32	8	9	8	239	27.5	8.29	15.13	8	cell organi	cytoplasm;	protein bir	Pf00466	51154	ENSG0000	MRTO4	1				0	0.796	0.048944268	12.7	1303.3	1695.4	6.45	8.35	High	High	High	High	High	High	1	
FALSE	High	Master Prc	O95470	sphingosin	0	17.918	8	3	4	3	568	63.5	9.16	11.07	3	cell death;	endoplasm	catalytic a	Pf00155, P	8879	ENSG0000	SGPL1	10	Metabolic	Kennedy p	Sphingolip	8	0.796	0.620489259	8.18	446.8	564.2	75.1	17.79	High	High	High	High	High	High	1	
FALSE	High	Master Prc	Q92688	Acidic leuc	0	46.746	29	7	19	6	251	28.8	4.06	36.39	7	cell organi	cytoplasm;	protein bir	Pf12799, P	10541	ENSG0000	ANP32B	9				0	0.795	0.150155283	2.19	4240.9	4765.5	9.49	8.04	High	High	High	High	High	High	1	
FALSE	High	Master Prc	Q9Y3C6	Peptidyl-pr	0	7.578	17	2	2	2	166	18.2	7.99	6.93	2	metabolic	spliceosom	catalytic a	Pf00160	51645	ENSG0000	PPIL1	6	Spliceosom		mRNA Spli	5	0.794	0.105854	14.43	304.4	474.3	17.51	20.19	High	High	High	High	High	High	1	
FALSE	High	Master Prc	Q15800	Methylster	0	5.535	5	1	1	1	293	35.2	7.23	4.21	1	metabolic	endoplasm	catalytic a	Pf04116	6307	ENSG0000	MSMO1	4	Metabolic	Cholestero	Cholestero	7	0.794	0.167143133	8.48	60.7	72	13.22	3.88	High	High	High	High	High	High	1	
FALSE	High	Master Prc	Q13098	COP9 signa	0	12.091	7	3	4	3	491	55.5	6.74	8.42	3	cell organi	cytoplasm;	enzyme re	Pf01399, P	2873	ENSG0000	GPS1	17			Cargo reco	13	0.793	0.013363064	2.51	552.5	696.7	5.97	8.7	High	High	High	High	High	High	1	
FALSE	High	Master Prc	P68036	Ubiquitin-c	0	34.572	46	5	8	5	154	17.9	8.51	25.38	5	cell prolife	cytoplasm;	catalytic a	Pf00179	7332	ENSG0000	UBE2L3	22	Ubiquitin r	Parkin-Ubi	Synthesis c	13	0.793	0.017584515	0.04	2503.9	3079.5	3.34	5.81	High	High	High	High	High	High	1	
FALSE	High	Master Prc	P18077	60S riboso	0	51.537	53	8	33	8	110	12.5	11.06	73.8	8	cell organi	cytosol;me	protein bir	Pf01247	6165	ENSG0000	RPL35A	3	Ribosome	Cytoplasm	Peptide ch	34	0.793	0.023037188	4.49	12175.5	15029.5	1.63	5.63	High	High	High	High	High	High	1	
FALSE	High	Master Prc	Q9UHD9	Ubiquilin-2	0	24.363	7	3	7	2	624	65.7	5.22	16.17	3	metabolic	cytoplasm;	protein bir	Pf00240, P	29978	ENSG0000	UBQLN2	X	Protein pro		Cargo reco	5	0.793	0.114073387	16.17	861.1	970.7	15.45	18.92	High	High	High	High	High	High	1	
FALSE	High	Master Prc	P00491	purine nuc	0	43.26	29	6	12	6	289	32.1	6.95	37.8	6	metabolic	cytoplasm;	catalytic a	Pf01048	4860	ENSG0000	PNP	14	Metabolic		Neutrophil	13	0.792	0.113175413	19.3	2064.9	2605.6	12.01	5.87	High	High	High	High	High	High	1	
FALSE	High	Master Prc	Q6XQN6-1	nicotinate	0	24.155	13	5	5	5	538	57.5	5.68	11.55	5	metabolic	cytoplasm;	catalytic a	Pf04095	93100	ENSG0000	NAPRT1; N	8; CHR_HS	Metabolic	NAD+ bios	Neutrophil	11	0.791	0.039399158	5.98	2325.7	2756.8	3.98	7.71	High	High	High	High	High	High	1	
FALSE	Medium	Master Prc	Q9H5X1-1	MIP18 fam	0.024	1.527	7	1	1	1	160	18.3	4.88	0	1		cytoplasm;	metal ion l		84191	ENSG0000	FAM96A	15				0	0.791	0.116201213	9.25	397.8	538.2	9.87	12.35	High	High	High	High	High	High	1	
FALSE	High	Master Prc	Q9BVJ6-1	U3 small n	0	10.832	5	3	3	3	771	87.9	7.87	8.12	3	metabolic	cytosol;nu	protein bir	Pf04615	10813	ENSG0000	UTP14A	X	Ribosome		Major path	6	0.79	0.104284083	11.23	358.7	498.2	13.17	10.34	High	High	High	High	High	High	1	
FALSE	High	Master Prc	Q9ULC4-1	Malignant	0	30.651	27	3	5	3	181	20.5	8.82	20.97	3	cell organi	cytoplasm;	protein bir	Pf01472	28985	ENSG0000	MCTS1	X				0	0.79	0.225430411	14.88	1185.9	1218	17.5	16.25	High	High	High	High	High	High	1	
FALSE	High	Master Prc	P63096-1	Guanine nu	0	36.165	15	4	9	1	354	40.3	5.97	23.81	4	cell divisio	cytoplasm;	catalytic a	Pf00025, P	2770	ENSG0000	GNAI1	7	Morphine	Nicotine A	G alpha (z)	74	0.789	0.159387005	2.2	508.5	544.1	11.33	11.03	High	High	High	High	High	High	1	
FALSE	High	Master Prc	Q9UBQ5	Eukaryotic	0	39.626	40	7	9	7	218	25	4.93	21.03	7	metabolic	cytoplasm;	protein bir	Pf03399, P	27335	ENSG0000	EIF3K	19; CHR_H			Formation	11	0.788	0.016983641	7.26	1419.4	1811.7	2.98	3.94	High	High	High	High	High	High	1	
FALSE	High	Master Prc	Q9H9C1-1	Spermatog	0.002	3.595	3	1	1	1	493	57	7.4	2.31	1	cell differe	cytoplasm;	protein bir	Pf09787	63894	ENSG0000	VIPAS39	14				0	0.787	0.153429958	21.95	44.4	57.5	1.78	15.36	High	High	High	High	High	High	1	
FALSE	High	Master Prc	Q16637	Survival m	0	9.751	11	2	2	2	294	31.8	6.55	2.96	2	cell organi	cytoplasm;	protein bir	Pf06003	6606; 660	ENSG0000	SMN1; SM	5; CHR_HS	RNA trans		snRNP Ass	4	0.786	0.059249447	1.51	377.9	438.6	8.32	11.87	High	High	High	High	High	High	1	
FALSE	High	Master Prc	Q96R06	Sperm-ass	0.006	2.504	1	1	1	1	1193	134.3	5	2.06	1	cell divisio	chromosor	protein bir		10615	ENSG0000	SPAG5	17				0	0.785	0.176685818	24.55	95.3	127.4	14.11	12.59	High	High	High	High	High	High	1	
FALSE	High	Master Prc	P13645	Keratin, ty	0	70.192	22	10	21	9	584	58.8	5.21	64.63	10	cell death;	cytoplasm;	structural	Pf00038, P	3858	ENSG0000	KRT10	17	Staphylocc		Formation	4	0.785	0.549479997	2.74	5049.7	5726.1	19.06	9.66	High	High	High	High	High	High	1	
FALSE	High	Master Prc	Q8N1G2	Cap-specifi	0	7.927	2	1	1	1	835	95.3	7.05	3.03	1	metabolic	cytosol;nu	catalytic a	Pf01585, P	23070	ENSG0000	CMTR1; FT	6				0	0.783	0.677830327	22.37	237.7	303.6	42.66	10.79	High	High	High	High	High	High	1	
FALSE	High	Master Prc	Q9HCD5	Nuclear re	0	19.094	13	5	9	5	579	65.5	9.6	14.22	5	metabolic	cytoskelet	protein bir		57727	ENSG0000	NCOA5	20				0	0.782	0.014176096	5.31	2590.6	3293.3	8.17	7.37	High	High	High	High	High	High	1	
FALSE	High	Master Prc	P49247	ribose-5-pl	0.004	2.631	5	1	1	1	311	33.2	8.54	2.35	1	metabolic	cytosol	catalytic a	Pf00455, P	22934	ENSG0000	LOC10106	2	Biosynthes	Pentose Pl	RPIA defici	14	0.781	0.211756011	24.41	225.6	302.3	20.79	13.62	High	High	High	High	High	High	1	
FALSE	High	Master Prc	O43169	Cytochrom	0	26.701	40	4	13	4	146	16.3	4.97	37.64	4	metabolic	membrane	metal ion l	Pf00173	80777	ENSG0000	CYB5B	16		Oxidation	Phase I - Fu	4	0.781	0.374971842	49.15	3574.5	4577.9	29.57	25.94	High	High	High	High	High	High	1	
FALSE	High	Master Prc	Q99720	sigma non-	0.002	4.028	8	2	2	2	223	25.1	5.96	1.72	2	cell organi	endoplasm	protein bir	Pf04622	10280	ENSG0000	SIGMAR1	9				0	0.78	0.338646211	41.07	43	55.1	29.24	16.5	High	High	High	High	High	High	1	
FALSE	High	Master Prc	P61204	ADP-ribosy	0	100.826	63	11	27	5	181	20.6	7.43	76.95	11	metabolic	cytoplasm;	catalytic a	Pf00025, P	377	ENSG0000	ARF3	12	Endocytosi		Synthesis c	17	0.778	0.263547134	4.71	3011.7	3700.5	5.86	19.95	High	High	High	High	High	High	1	
FALSE	Medium	Master Prc	Q06124	tyrosine-pr	0.01	2.074	1	1	1	1	597	68.4	7.3	0	1	cell differe	cytoplasm;	catalytic a	Pf00017, P	5781	ENSG0000	PTPN11	12	Chronic my	IL-5 Signali	PI3K Casca	134	0.777	0.048071704	5.49	603.3	771.7	1.36	6.99	High	High	High	High	High	High	1	
FALSE	High	Master Prc	P62269	40S riboso	0	54.134	54	12	28	12	152	17.7	10.99	50.02	12	cell organi	cytoplasm;	protein bir	Pf00416	6222	ENSG0000	RPS18	6; CHR_HS	Ribosome	Cytoplasm	Peptide ch	38	0.777	0.074876481	6.04	8414.6	10824.7	22.48	1.43	High	High	High	High	High	High	1	
FALSE	High	Master Prc	P42330	Aldo-keto r	0	65.566	51	14	23	14	323	36.8	7.94	60.82	14	cell commu	cytoplasm;	catalytic a	Pf00248	8644	ENSG0000	AKR1C3	10	Arachidoni	Benzo(a)py	Signaling b	26	0.777	0.077013174	8.2	7657	9371.2	6.84	6.65	High	High	High	High	High	High	1	
FALSE	High	Master Prc	Q04837	Single-stra	0	22.055	36	4	6	4	148	17.2	9.6	20.32	4	cell organi	mitochond	DNA bindi	Pf00436	6742	ENSG0000	SSBP1	7	Mismatch		Transcripti	6	0.777	0.153105291	15.13	2680.6	3450	13.46	13.18	High	High	High	High	High	High	1	
FALSE	High	Master Prc	Q9UBR2	Cathepsin Z	0	14.185	13	3	4	3	303	33.8	7.11	9.86	3	cell organi	cell surface	catalytic a	Pf00112	1522	ENSG0000	CTSZ	20	Lysosome;		Metabolisn	19	0.777	0.362628644	48.46	288	329.3	25.27	19	High	High	High	High	High	High	1	
FALSE	High	Master Prc	Q9H074	Polyadenyl	0	12.202	8	4	5	4	479	53.5	4.81	4.02	4	metabolic	cytoplasm;	protein bir	Pf02854	10605	ENSG0000	PAIP1	5	RNA trans	Translatior	Deadenyla	5	0.776	0.03393525	7.58	2121.6	2607.3	4.82	5.75	High	High	High	High	High	High	1	
FALSE	High	Master Prc	Q0VDF9	Heat shock	0	22.406	13	4	4	4	509	54.8	5.59	13.17	4	cell organi	cytoplasm;	nucleotide	Pf00012, P	51182	ENSG0000	HSPA14	10		Parkin-Ubi	Regulation	5	0.776	0.071524142	2.07	757.8	1074.8	13.25	7.57	High	High	High	High	High	High	1	
FALSE	Low	Master Prc	Q4G0N8	Sodium/hy	0.065	1.079	2	1	1	1	1177	135.1	7.14	0	1	cell differe	membrane	transporte	Pf00027, P	285335	ENSG0000	SLC9C1	3			Stimuli-ser	3	0.774	0.226935208	22.62	155.9	181.7	9.46	25.6	High	High	High	High	High	High	1	[K846;
FALSE	High	Master Prc	Q9NYF8-1	Bcl-2-asso	0	153.99	32	30	71	1	920	106.1	9.98	149.47	30	cell death;	cytoplasm;	DNA bindi	Pf15440	9774	ENSG0000	BCLAF1	6		Interactom		1	0.773	0.461917544	59.69	1050.7	1348.4	35.01	21.13	High	High	High	High	High	High	1	
FALSE	High	Master Prc	O95486-1	Protein tra	0	9.521	3	2	2	2	1093	119.7	7.66	6.86	2	cell organi	cytoplasm;	metal ion l	Pf00626, P	10802	ENSG0000	SEC24A	5	Protein pro	Sterol Reg	Regulation	20	0.77	0.293816494	42.7	86	89.5	50.97	22.49	High	High	High	High	High	High	1	
FALSE	High	Master Prc	P35579-1	Myosin-9 [	0	320.804	35	65	104	55	1960	226.4	5.6	250.27	65	cell differe	cytoplasm;	catalytic a	Pf00063, P	4627	ENSG0000	MYH9	22	Salmonella	Primary Fc	RHO GTPa	23	0.767	0.25537837	0.34	23403.6	28506.3	12.38	7	High	High	High	High	High	High	1	
FALSE	High	Master Prc	P53384	Cytosolic F	0	14.186	14	3	3	3	320	34.5	5.33	7.32	3	cell growth	cytoplasm;	metal ion l	Pf01656, P	4682	ENSG0000	NUBP1	16			Cytosolic ir	2	0.766	0.188855471	22.22	405.8	696.7	24.24	24.08	High	High	High	High	High	High	1	
FALSE	High	Master Prc	Q86UU0	B-cell CLL/	0	7.975	2	2	3	2	1499	157	8.63	4.65	2	cell differe	nucleus	protein bir	Pf11502	283149	ENSG0000	BCL9L	11			Formation	5	0.764	0.569133568	14.96	691.8	906.1	91.76	3.96	High	High	High	High	High	High	1	
FALSE	High	Master Prc	Q9H9A5	CCR4-NOT	0	6.26	3	2	3	2	744	82.3	7.78	2.95	2	metabolic	cytoplasm;	protein bir	Pf10046	25904	ENSG0000	CNOT10	3	RNA degra		TP53 regul	10	0.762	0.167415131	29.31	407.2	480.9	25.55	12.19	High	High	High	High	High	High	1	
FALSE	High	Master Prc	Q13625-1	Apoptosis-	0	6.332	2	2	2	2	1128	125.5	6.07	2.65	2	cell death;	cytoplasm;	protein bir	Pf00018, P	7159		TP53BP2	1	Hippo sign		Regulation	16	0.762	0.182323483	8.81	208.2	289.6	24.54	34.72	High	High	High	High	High	High	1	
FALSE	High	Master Prc	Q8N5M4	Tetratricop	0	8.16	30	4	4	4	171	20	8.92	4.67	4			protein bir	Pf13414, P	283237	ENSG0000	TTC9C	11				0	0.759	0.14201637	25.97	252.3	353.7	17.19	16.38	High	High	High	High	High	High	1	
FALSE	High	Master Prc	Q7Z6G8	Ankyrin re	0.002	3.58	1	1	1	1	1248	138	6.37	2.94	1		cytoplasm;	protein bir	Pf00023, P	56899	ENSG0000	ANKS1B	12		Ectoderm		1	0.759	0.577508395	15	112.1	126.2	90.82	17.97	High	High	High	High	High	High	1	
FALSE	High	Master Prc	Q6PI48	Aspartate-	0	8.858	8	5	7	5	645	73.5	8.02	5.88	5	metabolic	cytoplasm;	catalytic a	Pf00152, P	55157	ENSG0000	DARS2	1	Aminoacyl		Mitochonc	5	0.758	0.013928129	1.48	378.4	494.5	2.48	6.93	High	High	High	High	High	High	1	
FALSE	High	Master Prc	Q5MIZ7-1	Serine/thre	0	12.417	4	3	3	1	849	97.4	4.96	9.47	3	metabolic	cytoplasm;		Pf04802	57223	ENSG0000	SMEK2; PP	2	Glucagon s	Integrated		2	0.758	0.276739198	43.74	105.2	139.5	23.97	21.88	High	High	High	High	High	High	1	
FALSE	High	Master Prc	Q9UQ80	proliferatic	0	198.266	68	25	89	25	394	43.8	6.55	219.94	25	cell prolife	cytoplasm;	DNA bindi	Pf00557	5036	ENSG0000	PA2G4	12			Neutrophil	3	0.757	0.009706808	0.89	26897	35742.9	3.07	5.76	High	High	High	High	High	High	1	
FALSE	High	Master Prc	Q12933	TNF recept	0	4.698	4	2	2	2	501	55.8	7.53	4.31	2	cell death;	cytoplasm;	catalytic a	Pf00097, P	7186	ENSG0000	TRAF2	9	NOD-like r	RANKL/RA	Ub-specific	53	0.756	0.005641616	0.81	467	617.1	0.32	3.16	High	High	High	High	High	High	1	
FALSE	High	Master Prc	Q96B26	Exosome c	0	11.634	11	3	3	3	276	30	5.3	4.47	3	metabolic	cytoplasm;	catalytic a	Pf01138, P	11340	ENSG0000	EXOSC8	13	RNA degra		Major path	15	0.754	0.70552487	5.44	148.9	171.3	65.63	11.85	High	High	High	High	High	High	1	
FALSE	High	Master Prc	P62877	E3 ubiquiti	0	9.191	13	2	4	2	108	12.3	6.96	7.27	2	cell organi	cytoplasm;	catalytic a	Pf00097, P	9978	ENSG0000	RBX1	22	Wnt signal	Dual hijack	FBXL7 dow	87	0.752	0.037077121	7.32	1267.6	1537.8	10.46	10.58	High	High	High	High	High	High	1	
FALSE	Medium	Master Prc	Q9NXE4-1	Sphingomy	0.013	1.879	1	1	1	1	827	93.3	7.97	1.84	1	metabolic	endoplasm	catalytic a	Pf14724	55627		SMPD4	2	Sphingolip		Glycosphin	6	0.752	0.259563334	7.77	595.7	717.2	16.78	7.79	High	High	High	High	High	High	1	
FALSE	High	Master Prc	P68104	Elongation	0	274.705	63	32	193	20	462	50.1	9.01	470.93	32	metabolic	cytoplasm;	catalytic a	Pf00009, P	1915	ENSG0000	EEF1A1	6	RNA trans	Translatior	Peptide ch	16	0.748	0.006725275	3.11	28167.2	37645.4	4.19	6.75	High	High	High	High	High	High	1	[Q164;
FALSE	High	Master Prc	Q9Y388	RNA-bindir	0.002	3.969	4	1	1	1	322	37.3	9.83	2.4	1	metabolic	spliceosom	catalytic a	Pf00076, P	51634	ENSG0000	RBMX2	X				0	0.746	0.604472506	28.52	331.3	444.2	30.67	11.62	High	High	High	High	High	High	1	
FALSE	Medium	Master Prc	Q8IW41	MAP kinas	0.013	1.863	2	1	1	1	473	54.2	7.78	0	1	metabolic	cytoplasm;	catalytic a	Pf00069, P	8550	ENSG0000	MAPKAPK!	12	MAPK sign	MAPK Sigr	MAPK6/M	17	0.743	0.112618704	1.45	458.7	604.1	8.82	5.61	High	High	High	High	High	High	1	
FALSE	High	Master Prc	P62081	40S riboso	0	68.267	63	13	34	13	194	22.1	10.1	85.17	13	cell organi	cytoplasm;	protein bir	Pf01251	6201	ENSG0000	RPS7	2	Ribosome	Cytoplasm	Peptide ch	39	0.742	0.052105377	10.34	9079.8	11242.2	9.21	6.9	High	High	High	High	High	High	1	
FALSE	High	Master Prc	P68133	Actin, alph	0	217.892	46	18	311	3	377	42	5.39	712.73	18	cell organi	cytoplasm;	nucleotide	Pf00022	58	ENSG0000	ACTA1	1		Myometria	Striated M	6	0.742	0.250793799	10.62	1021.1	1236.5	13.81	12.23	High	High	High	High	High	High	1	[K63;
FALSE	High	Master Prc	Q9BSD7	Cancer-rel	0	9.949	26	3	3	3	190	20.7	9.54	6.33	3	metabolic	membrane	catalytic a	Pf00004, P	84284	ENSG0000	NTPCR	1	Metabolic			3	0.741	0.458943176	53.37	29.3	28.4	23.9	40.91	High	High	High	High	High	High	1	
FALSE	High	Master Prc	Q9HAV0	Guanine nu	0	33.037	19	6	12	2	340	37.5	6	32.15	6	metabolic	cytosol;me	protein bir	Pf00400	59345	ENSG0000	GNB4	3	Pathways i	Myometria	G alpha (z)	64	0.737	0.071430381	20.45	403.4	546.7	15.33	13.89	High	High	High	High	High	High	1	
FALSE	High	Master Prc	Q9H1E3	Nuclear ub	0	33.094	20	6	10	6	243	27.3	5.08	19.38	6	cell organi	chromosor	DNA bindi		64710	ENSG0000	NUCKS1	1				0	0.737	0.141518495	9.07	2184.1	2452.5	10.89	17.61	High	High	High	High	High	High	1	
FALSE	High	Master Prc	P18085	ADP-ribosy	0	61.227	56	9	23	4	180	20.5	7.14	63.91	9	cellular co	cytosol;Go	catalytic a	Pf00025, P	378	ENSG0000	ARF4	3			COPI-depe	15	0.735	0.147252351	23.35	2709.7	3688.4	6.78	24.2	High	High	High	High	High	High	1	
FALSE	High	Master Prc	Q9NXR7-1	Isoform 1 c	0.003	3.421	3	1	1	1	415	46.9	6.14	0	1	cell death;	cytoplasm;	protein bir	Pf06113	9577	ENSG0000	BRE; BABA	2				0	0.731	0.072228215	10.18	207.5	308	9.95	13.56	High	High	High	High	High	High	1	
FALSE	High	Master Prc	Q4KWH8-	1-phospha	0.003	3.452	0	1	2	1	1693	189.1	7.74	0	1	metabolic	cytoplasm;	catalytic a	Pf00036, P	23007	ENSG0000	PLCH1	3	Metabolic	Mesoderm	Synthesis c	7	0.725	0.21089746	38.59	277.6	382.9	12.95	17.39	High	High	High	High	High	High	1	

FALSE	High	Master Prc	P46781	40S riboso	0	56.222	53	15	36	15	194	22.6	10.65	59.62	15	cell organi	cytoplasm;	protein bir	Pf00163, P	6203	ENSG0000	RPS9	19; CHR_H	Ribosome	Cytoplasm	Peptide ch	39	0.724	0.000989031	2.43	13985.7	19309.8	6.57	3.25	High	High	High	High	High	High	1	
FALSE	High	Master Prc	Q13547	histone de	0	20.195	13	4	5	3	482	55.1	5.48	8.62	4	cell differe	chromosor	catalytic a	Pf00850	3065	ENSG0000	HDAC1	1	Huntingtor	Androgen	NOTCH1 Ir	95	0.724	0.702800163	90.46	18.6	25.8	41.89	31.87	High	High	High	High	High	High	1	
FALSE	High	Master Prc	Q8IY57-1	YY1-associ	0	5.544	14	1	1	1	180	19.9	9.72	3.16	1	metabolic	cytosol;nu	metal ion l	Pf00641	10138	ENSG0000	YAF2	12			RUNX1 int	6	0.719	0.022590875	2.2	185.4	257.7	9.88	14.33	High	High	High	High	High	High	1	
FALSE	High	Master Prc	P30041	Peroxiredo	0	132.57	64	16	59	16	224	25	6.38	129.79	16	cellular ho	cytoplasm;	antioxidan	Pf00578, P	9588	ENSG0000	PRDX6	1	Metabolic	NRF2 path	Neutrophil	8	0.719	0.044470299	8.99	10976.2	15918.2	4.73	11.12	High	High	High	High	High	High	1	
FALSE	High	Master Prc	O75832	26S protea	0	22.42	25	4	4	4	226	24.4	6.1	12.94	4	cell death;	cytoplasm;	protein bir	Pf00023, P	5716	ENSG0000	PSMD10	X		Proteasom	Regulation	150	0.718	0.009017521	1.46	2467.3	3435.6	18.4	9.13	High	High	High	High	High	High	1	
FALSE	High	Master Prc	O14950	Myosin reg	0	21.905	40	5	7	5	172	19.8	4.84	16.6	5	regulation	cytosol	metal ion l	Pf00036, P	103910; 1(	ENSG0000	MYL12B; N	18	Regulation	Human Th	RHO GTPa:	22	0.718	0.036024574	7.54	2437.5	3252.5	7.09	13.31	High	High	High	High	High	High	1	
FALSE	High	Master Prc	P15941	mucin-1 [C	0	5.785	2	2	2	2	1255	122	7.47	2.12	2	metabolic	cytoplasm;	DNA bindi	Pf01390, P	4582		MUC1	1			Defective (	18	0.718	0.069193485	12.03	255.9	408.3	9.27	16.93	High	High	High	High	High	High	1	
FALSE	High	Master Prc	Q52LJ0	Protein Fai	0	19.194	8	2	6	1	330	37.2	6.29	17.24	2	metabolic	cytoplasm;	catalytic a	Pf10239	283742	ENSG0000	FAM98B	15			tRNA proc	3	0.716	0.010828214	1.23	934.5	1295	12.29	13.86	High	High	High	High	High	High	1	
FALSE	High	Master Prc	O43813	LanC-like p	0	6.043	7	3	3	3	399	45.3	7.75	6.05	3	regulation	cytoplasm;	metal ion l	Pf05147	10314	ENSG0000	LANCL1	2				0	0.713	0.005597657	4.46	720	979.8	16.04	7.87	High	High	High	High	High	High	1	
FALSE	High	Master Prc	O43663-1	Protein reg	0	4.673	2	1	2	1	620	71.6	6.68	6.97	1	cell divisio	cytoplasm;	protein bir	Pf03999	9055	ENSG0000	PRC1	15			RHO GTPa:	4	0.711	0.177687888	37.02	115.2	178.2	16.62	20.84	High	High	High	High	High	High	1	
FALSE	High	Master Prc	P53007	Tricarboxy	0	12.157	11	3	3	3	311	34	9.89	6.83	3	metabolic	membrane	transporte	Pf00153	6576	ENSG0000	SLC25A1	22			Gluconeog	7	0.707	0.068609315	1.14	596.5	805	4.03	9.57	High	High	High	High	High	High	1	
FALSE	High	Master Prc	P11908	ribose-pho	0	46.448	25	7	14	1	318	34.7	6.61	36.57	7	metabolic		catalytic a	Pf00156, P	5634	ENSG0000	PRPS2	X	Metabolic	Nucleotide	5-Phospho	10	0.703	0.004590656	4.8	2581.7	3596.9	13.11	5.71	High	High	High	High	High	High	1	
FALSE	High	Master Prc	Q9H3P2-1	Negative e	0	10.168	6	3	5	3	528	57.2	9.03	10.38	3	developme	cytosol;nu	protein bir		7469	ENSG0000	NELFA; WH	4		Initiation c	HIV elonga	26	0.699	0.126940335	17	316.2	455.2	3.58	16.76	High	High	High	High	High	High	1	
FALSE	High	Master Prc	Q92667	A-kinase ai	0	10.699	4	2	3	2	903	97.3	4.94	6.81	2	coagulatio	cytosol;me	protein bir	Pf00013, P	8165	ENSG0000	AKAP1	17		G Protein S	Mitochonc	5	0.695	0.143490398	32.54	42	67.7	35.38	6.63	High	High	High	High	High	High	1	
FALSE	High	Master Prc	P60468	protein tra	0	8.29	29	3	4	3	96	10	11.56	8.34	3	metabolic	cytosol;en	protein bir	Pf03911	10952	ENSG0000	SEC61B	9	Phagosom		ER-Phagos	15	0.692	0.136408793	4.61	944.4	1303.1	14.58	6.71	High	High	High	High	High	High	1	
FALSE	High	Master Prc	Q9BVC6	Transmem	0	11.394	9	2	4	2	243	26.2	10.48	3.76	2	regulation	endoplasm	transporte	Pf14965	79073	ENSG0000	TMEM109	11				0	0.692	0.680381157	109.52	1571.6	2271.7	104.16	16.78	High	High	High	High	High	High	1	
FALSE	High	Master Prc	P40222	alpha-taxil	0	24.258	22	7	7	7	546	61.9	6.52	13.97	7	cell prolife	cytoplasm;	protein bir	Pf09728	200081	ENSG0000	TXLNA	1			Other inte	4	0.69	0.155791888	4.89	1608.8	1929.1	12.67	16.56	High	High	High	High	High	High	1	
FALSE	High	Master Prc	O14617-1	AP-3 comp	0	13.338	3	2	2	2	1153	130.1	8.48	8.46	2	cell organi	cytoplasm;	transporte	Pf01602, P	8943	ENSG0000	AP3D1	19	Lysosome			1	0.689	0.274990339	56.9	46.6	65.3	21.98	26.85	High	High	High	High	High	High	1	
FALSE	High	Master Prc	P61009	Signal pept	0	8.154	11	2	3	2	180	20.3	8.62	3.39	2	metabolic	endoplasm	catalytic a	Pf04573	60559	ENSG0000	SPCS3	4	Protein ex		Synthesis,	9	0.686	0.059019056	12.44	423	540	12.57	9.89	High	High	High	High	High	High	1	
FALSE	High	Master Prc	Q9BPX3	Condensin	0	15.367	5	4	5	4	1015	114.3	5.59	9.61	4	cell divisio	chromosor	protein bir	Pf12719, P	64151	ENSG0000	NCAPG	4			Condensat	5	0.682	0.146804451	32.6	179.9	225.7	19.34	16.06	High	High	High	High	High	High	1	
FALSE	High	Master Prc	P51153	Ras-relatec	0	14.158	22	4	5	3	203	22.8	9.19	7.18	4	cell organi	cytosol;en	catalytic a	Pf00025, P	5872	ENSG0000	RAB13	1	Tight junct		Translocat	9	0.679	0.025944188	9.48	856.1	1260.5	1.77	10.61	High	High	High	High	High	High	1	
FALSE	High	Master Prc	O15121	Sphingolip	0	7.68	11	2	2	2	323	37.8	7.46	4.79	2	metabolic	endoplasm	catalytic a	Pf00487, P	8560	ENSG0000	DEGS1	1	Metabolic		Neutrophil	10	0.676	0.340875979	60.03	10.3	15.2	26.48	31.34	High	High	High	High	High	High	1	
FALSE	High	Master Prc	Q9Y5S9	RNA-bindi	0	22.37	27	3	8	3	174	19.9	5.72	25.33	3	metabolic	cytoplasm;	protein bir	Pf00076, P	9939	ENSG0000	RBM8A; L(	1	mRNA surv		mRNA 3'-e	20	0.675	0.110611164	29.48	2523.3	4242.7	10.5	20.41	High	High	High	High	High	High	1	
FALSE	High	Master Prc	Q96DH6-1	RNA-bindi	0.003	3.142	7	2	2	2	328	35.2	8.48	4.46	2		cytoplasm	protein bir	Pf00076, P	124540	ENSG0000	MSI2	17	mRNA surv			1	0.675	0.202936199	8.82	687.2	851.3	15.09	17.61	High	High	High	High	High	High	1	
FALSE	Low	Master Prc	Q9BZH6	WD repeat	0.05	1.195	1	1	1	1	1224	136.6	6.92	1.65	1		cytoplasm;	protein bir		55717	ENSG0000	WDR11	10				0	0.672	0.655733115	4.35	206.9	290.9	44.71	7.56	High	High	High	High	High	High	1	
FALSE	High	Master Prc	O75815-1	Breast can	0.002	3.642	1	1	1	1	825	92.5	7.96	2.08	1	regulation		protein bir	Pf00017, P	8412	ENSG0000	BCAR3	1				0	0.67	0.045682902	9.71	756.8	1115.8	7.79	6.74	High	High	High	High	High	High	1	
FALSE	High	Master Prc	Q86X55	Histone-ar	0	23.8	11	5	7	5	608	65.8	6.73	11.02	5	cell organi	cytoplasm;	catalytic a	Pf02475, P	10498	ENSG0000	CARM1	19	Endocrine	Androgen	Transcripti	29	0.669	0.027846538	10.37	731.8	1006	11.91	13.39	High	High	High	High	High	High	1	
FALSE	High	Master Prc	P62308	Small nucle	0	12.247	36	3	6	3	76	8.5	8.88	9.79	3	cell organi	cytoplasm;	protein bir	Pf01423, P	6637	ENSG0000	SNRPG	2	Spliceosom	mRNA Pro	SLBP indep	16	0.656	0.135107816	11.07	1815.3	2975.1	8.33	25.42	High	High	High	High	High	High	1	
FALSE	High	Master Prc	P07099	epoxide hy	0	16.074	8	4	5	4	455	52.9	7.25	7.71	4	metabolic	endoplasm	catalytic a	Pf00561, P	2052	ENSG0000	EPHX1	1	Metabolisr	Photodyna	Phase I - Fu	11	0.646	0.4006979	20.93	603.5	872.1	25	18.64	High	High	High	High	High	High	1	
FALSE	High	Master Prc	Q13610	Periodic tr	0	11.933	5	2	3	2	501	55.8	4.77	9.31	2	cell organi	Golgi;nucle	protein bir	Pf00400	11137	ENSG0000	PWP1	12				0	0.644	0.109444187	26.17	547.2	995.4	23.85	19.53	High	High	High	High	High	High	1	
FALSE	High	Master Prc	O15260-1	surfeit locu	0	9.997	9	2	3	2	269	30.4	7.78	4.25	2	cell organi	endoplasm	protein bir	Pf02077	6836	ENSG0000	SURF4	9; CHR_HG			COPI-depe	8	0.616	0.167330504	50.66	916.5	1327.3	37.43	21.9	High	High	High	High	High	High	1	
FALSE	High	Master Prc	P18615	Negative e	0	17.994	19	5	6	5	380	43.2	9.33	5.6	5	metabolic	membrane	protein bir	Pf00076, P	7936	ENSG0000	NELFE; RD	6; CHR_HS		Initiation c	HIV elonga	26	0.614	0.193877817	42.22	369.6	742.6	26.02	47.65	High	High	High	High	High	High	1	
FALSE	High	Master Prc	P62273-1	40S riboso	0	5.664	29	3	4	3	56	6.7	10.13	6.25	3	cell organi	cytosol;rib	metal ion l	Pf00253	6235	ENSG0000	RPS29	14	Ribosome	Cytoplasm	Peptide ch	38	0.606	0.963230354	41.4	2822.8	4188.4	74.44	22.89	High	High	High	High	High	High	1	
FALSE	High	Master Prc	O75494-1	Serine/argi	0	16.301	11	3	4	3	262	31.3	11.27	9.91	3	cell organi	cytoplasm;	protein bir	Pf00076, P	10772	ENSG0000	SRSF10; LC	1	Spliceosom	mRNA Pro	mRNA Spli	6	0.559	0.212984574	66.67	504.9	1138.9	35.6	40.11	High	High	High	High	High	High	1	
FALSE	High	Master Prc	P61457	Pterin-4-al	0	15.224	38	3	5	3	104	12	6.8	10.54	3	cell organi	cytoplasm;	catalytic a	Pf01329	5092	ENSG0000	PCBD1	10			Phenylalar	4	0.529	0.308403229	70.51	1115	1780.8	12.55	77.1	High	High	High	High	High	High	1	

Table S2. Gene Ontology (GO) information, including the biological process, cellular component and molecular function, of differentially expressed proteins (DEPs) with |FC| ≥1.2 identified in A549 cells exposed to ZQX-1 (0.5 μM) under irradiation at 420 nm for 20 min following 4.0 h of incubation at 37 °C in the dark. * NA: not available.

No.	Accession	Gene symbol	Protein name	FC (LZ vs.DZ)	Biological Process	Cellular Component	Molecular Function
1	Q86X55	CARM1	Histone-arginine methyltransferase CARM1	-1.49	cell organization and biogenesis; metabolic process; regulation of biological process; response to stimulus	cytoplasm; cytosol; nucleus	catalytic activity; DNA binding; protein binding
2	O75815-1	BCAR3	Breast cancer anti-estrogen resistance protein 3	-1.49	regulation of biological process; response to stimulus	N.A.*	protein binding
3	P51153	RAB13	Ras-related protein Rab-13	-1.47	cell organization and biogenesis; metabolic process; regulation of biological process; response to stimulus;	cytosol; Golgi; endosome; membrane	catalytic activity; nucleotide binding; protein binding
4	P11908	PRPS2	ribose-phosphate pyrophosphokinase 2	-1.42	metabolic process	N.A.*	catalytic activity; metal ion binding; nucleotide binding; protein binding
5	O43813	LANCL1	Lan C-like protein 1	-1.40	regulation of biological process; response to stimulus	cytoplasm; membrane	metal ion binding; protein binding; receptor activity; signal transducer activity
6	Q52LJ0	FAM98B	Protein Fam 98b	-1.40	metabolic process; regulation of biological process	cytoplasm; nucleus	catalytic activity; protein binding; RNA binding
7	O75832	PSMD10	26S proteasome non-ATPase regulatory subunit 10	-1.39	cell death; cell organization and biogenesis; metabolic process; regulation of biological process; response to stimulus; transport	cytoplasm; cytoskeleton; cytosol; nucleus;	protein binding

						proteasome	
8	O14950	MYL12B; MYL12A	Myosin regulatory light chain 12B	-1.39	regulation of biological process; response to stimulus	cytosol	metal ion binding; protein binding
9	Q8IY57-1	YAF2	YY1-associated factor 2	-1.39	metabolic process; regulation of biological process	cytosol; nucleus	metal ion binding; protein binding
10	P30041	PRDX6	Peroxiredoxin-6	-1.39	cellular homeostasis; metabolic process; regulation of biological process; response to stimulus; transport	cytoplasm; cytosol; extracellular; membrane; nucleus; organelle lumen; vacuole	antioxidant activity; catalytic activity; protein binding
11	P46781	RPS9	40S ribosomal protein S9	-1.38	cell organization and biogenesis; metabolic process; regulation of biological process; transport	cytoplasm; cytosol; membrane; nucleus; ribosome	protein binding; RNA binding; structural molecule activity; translation regulator activity
12	P68104	EEF1A1	Elongation factor 1-alpha 1	-1.34	metabolic process; regulation of biological process; response to stimulus; transport	cytoplasm; cytosol; membrane; nucleus; organelle lumen	catalytic activity; nucleotide binding; protein binding; RNA binding
13	P62877	RBX1	E3 ubiquitin-protein ligase RBX1	-1.33	cell organization and biogenesis; metabolic process; regulation of biological process; response to stimulus	cytoplasm; cytosol; nucleus	catalytic activity; metal ion binding; protein binding

14	Q12933	TRAF2	TNF receptor-associated factor 2	-1.32	cell death; cell organization and biogenesis; metabolic process; response to stimulus	cytoplasm; cytosol; membrane	catalytic activity; metal ion binding; protein binding; signal transducer activity
15	Q9UQ80	PA2G4	proliferation-associated protein 2G4	-1.32	cell proliferation; metabolic process; regulation of biological process; transport	cytoplasm; extracellular; membrane; nucleus; organelle lumen	DNA binding; protein binding; RNA binding
16	Q6PI48	DARS2	Aspartate-tRNA ligase, mitochondrial	-1.32	metabolic process	cytoplasm; mitochondrion; nucleus; organelle lumen	catalytic activity; nucleotide binding; protein binding; RNA binding
17	Q9H074	PAIP1	Polyadenylate-binding protein-interacting protein 1	-1.29	metabolic process; regulation of biological process	cytoplasm; cytosol	protein binding; RNA binding; translation regulator activity
18	Q06124	PTPN11	tyrosine-protein phosphatase non-receptor type 11	-1.29	cell differentiation; cell organization and biogenesis; cellular component movement; metabolic process; regulation of biological process; response to stimulus	cytoplasm; cytosol; mitochondrion; nucleus	catalytic activity; protein binding
19	Q9HCD5	NCOA5	Nuclear receptor coactivator 5	-1.28	metabolic process; regulation of biological process	cytoskeleton; nucleus	protein binding; RNA binding

20	Q9UBQ5	EIF3K	Eukaryotic translation initiation factor 3 subunit K	-1.27	metabolic process; regulation of biological process	cytoplasm; cytosol; membrane; nucleus	protein binding; RNA binding
21	Q6XQN6-1	NAPRT1; NAPRT	nicotinate phosphoribosyl transferase	-1.26	metabolic process; response to stimulus; transport	cytoplasm; cytosol; extracellular; organelle lumen	catalytic activity; protein binding
22	Q13098	GPS1	COP9 signalosome complex subunit 1	-1.26	cell organization and biogenesis; metabolic process; regulation of biological process; response to stimulus	cytoplasm; cytosol; nucleus	enzyme regulator activity; protein binding
23	P68036	UBE2L3	Ubiquitin-conjugating enzyme E2 L3	-1.26	cell proliferation; metabolic process; regulation of biological process; response to stimulus	cytoplasm; cytosol; nucleus	catalytic activity; enzyme regulator activity; nucleotide binding; protein binding; RNA binding
24	P18077	RPL35A	60S ribosomal protein L35a	-1.26	cell organization and biogenesis; metabolic process; transport	cytosol; membrane; ribosome	protein binding; RNA binding; structural molecule activity
25	Q9BQC3	DPH2	2-(3-amino-3-carboxypropyl)histidine synthase subunit 2	-1.26	metabolic process	cytosol	catalytic activity; protein binding
26	Q9UKD2	MRTO4	mRNA turnover protein 4 homolog	-1.26	cell organization and biogenesis; metabolic process	cytoplasm; nucleus	protein binding; RNA binding

27	Q9NWT1	PAK1IP1	p21-activated protein kinase-interacting protein 1	-1.25	cell proliferation; regulation of biological process	nucleus	protein binding
28	P59998	ARPC4	Actin-related protein 2/3 complex subunit 4	-1.24	cell organization and biogenesis; regulation of biological process; response to stimulus	cytoplasm; cytoskeleton; cytosol	protein binding; structural molecule activity
29	Q16342-1	PDCD2	Programmed cell death protein 2	-1.24	cell death; metabolic process; regulation of biological process	cytoplasm; nucleus	DNA binding; metal ion binding; protein binding
30	Q8WX92	COBRA1; NELFB	Negative elongation factor B	-1.23	metabolic process; regulation of biological process	cytoplasm; nucleus	protein binding; RNA binding
31	Q86TI2	DPP9	Dipeptidyl peptidase 9	-1.23	metabolic process	cytoplasm; cytosol; nucleus	catalytic activity; protein binding
32	P63244	GNB2L1; RACK1	Receptor of activated protein C kinase 1	-1.23	cell death; cellular homeostasis; development; metabolic process; regulation of biological process; response to stimulus	cytoplasm; mitochondrion; nucleus; ribosome	enzyme regulator activity; protein binding; RNA binding;
33	Q92538	GBF1	Golgi-specific brefeldin A-resistance guanine nucleotide exchange factor 1	-1.23	cell organization and biogenesis; cellular component movement; metabolic process; response to stimulus; transport	cytoplasm; cytosol; Golgi; mitochondrion; organelle lumen	protein binding
34	P40261	NNMT	Nicotinamide N-methyl transferase	-1.22	metabolic process; response to stimulus	cytoplasm; cytosol	catalytic activity

No.	Accession	Gene symbol	Protein name	FC (LZ vs. DZ)	Biological Process	Cellular Component	Molecular Function
35	Q9Y508-1	RNF114	E3 ubiquitin-protein ligase RNF114	-1.22	cell differentiation; development; metabolic process; regulation of biological process	cytoplasm; cytosol; membrane; nucleus	catalytic activity; metal ion binding; protein binding
36	P23396-1	RPS3	40S ribosomal protein S3	-1.22	cell death; cell division; cell organization and biogenesis; metabolic process; regulation of biological process; response to stimulus; transport	endoplasmic reticulum; mitochondrion; nucleus; ribosome	catalytic activity; DNA binding; protein binding; RNA binding;
37	Q7L1Q6-1	BZW1	Basic leucine zipper and W2 domain-containing protein 1	-1.22	metabolic process; regulation of biological process	cytoplasm; membrane	protein binding; RNA binding
38	O60256-1	PRPSAP2	Phosphoribosyl pyrophosphate synthase-associated protein 2	-1.22	metabolic process; regulation of biological process	N.A.*	catalytic activity; metal ion binding; protein binding
39	P26374	CHML	Rab proteins geranyl transferase component A 2	-1.21	metabolic process; regulation of biological process; response to stimulus; transport	cytoplasm; cytosol	catalytic activity; protein binding
40	P62910	RPL32	60S ribosomal protein L32	-1.20	cell organization and biogenesis; metabolic process; response to stimulus; transport	cytosol; membrane; ribosome	RNA binding; structural molecule activity
41	P00338-1	LDHA	L-lactate dehydrogenase A chain	-1.20	metabolic process; regulation of biological process	cytoplasm; nucleus	protein binding; RNA binding

1	P56385	ATP5ME	ATP synthase subunit e, mitochondrial	1.20	cell organization and biogenesis; metabolic process; transport	membrane; mitochondrion	catalytic activity; transporter activity
2	P63165-1	SUMO1	Small ubiquitin-related modifier 1	1.20	cell organization and biogenesis; metabolic process; regulation of biological process; response to stimulus	chromosome; cytoplasm; membrane; nucleus	catalytic activity; protein binding; RNA binding
3	Q5JTV8	TOR1AIP1	Torsin-1A-interacting protein 1	1.21	cell organization and biogenesis; regulation of biological process	membrane; nucleus	enzyme regulator activity; protein binding
4	Q9GZP4	PITHD1	PITH domain-containing protein 1	1.21	response to stimulus	nucleus	
5	Q92734-1	TFG	Protein TFG	1.22	cell organization and biogenesis; regulation of biological process; response to stimulus; transport	cytoplasm; endoplasmic reticulum;	protein binding; signal transducer activity
6	Q96EC8-1	YIPF6	Protein YIPF6	1.22	N.A.*	endoplasmic reticulum; Golgi;	protein binding
7	P14406	COX7A2	Cytochrome c oxidase subunit 7A2, mitochondrial	1.22	metabolic process; transport	membrane; mitochondrion	catalytic activity; transporter activity
8	O96008	TOMM40	Mitochondrial import receptor subunit TOM40 homolog	1.23	cell communication; metabolic process; response to stimulus; transport	cytosol; membrane; mitochondrion	protein binding; transporter activity
9	P19367-1	HK1	Hexokinase-1	1.23	cellular homeostasis; metabolic process; regulation of biological process	cytosol; membrane; mitochondrion	catalytic activity; nucleotide binding; protein binding

10	Q9Y3D9	MRPS23	28S ribosomal protein S23, mitochondrial	1.23	cell organization and biogenesis; metabolic process	cytoskeleton; mitochondrion; ribosome	protein binding; RNA binding;
11	O75175	CNOT3	CCR4-NOT transcription complex subunit 3	1.23	cell differentiation; development; metabolic process; regulation of biological process; response to stimulus	cytoplasm; cytosol; nucleus	protein binding
12	Q9UIJ7-1	AK3	GTP: AMP phosphotransferase AK3, mitochondrial	1.23	coagulation; metabolic process	mitochondrion; organelle lumen	catalytic activity; nucleotide binding; protein binding
13	Q6P1L8	MRPL14	39S ribosomal protein L14, mitochondrial	1.23	cell organization and biogenesis; metabolic process	membrane; mitochondrion; ribosome	RNA binding; structural molecule activity
14	P80303	NUCB2	Nucleobindin-2	1.24	N.A.*	endoplasmic reticulum; nucleus	DNA binding; metal ion binding; protein binding
15	Q9H089	LSG1	Large subunit GTPase 1 homolog	1.24	transport	cytoplasm; endoplasmic reticulum; nucleus	catalytic activity; nucleotide binding
16	Q9HDC9	APMAP	Adipocyte plasma membrane-associated protein	1.24	metabolic process	cell surface; endoplasmic reticulum;	catalytic activity
17	Q9BXT2	CACNG6	voltage-dependent calcium channel gamma-6 subunit	1.25	regulation of biological process; transport	membrane	transporter activity

18	Q9BS26	ERP44	Endoplasmic reticulum resident protein 44	1.26	cellular homeostasis; metabolic process; regulation of biological process; response to stimulus; transport	endoplasmic reticulum; organelle lumen	catalytic activity; metal ion binding; protein binding
19	P09651-2	HNRNPA1	Isoform A1-A of Heterogeneous nuclear ribonucleoprotein A1	1.27	cell communication; metabolic process; regulation of biological process; response to stimulus; transport	cytoplasm; nucleus; spliceosomal complex	DNA binding; protein binding; RNA binding
20	P09874	PARP1	Poly [ADP-ribose] polymerase 1	1.27	cell death; cell differentiation; metabolic process; regulation of biological process; response to stimulus	membrane; mitochondrion; nucleus	catalytic activity; DNA binding; metal ion binding; nucleotide binding; protein binding; RNA binding
21	Q9UBS4	DNAJB11	DNAJ homolog subfamily B member 11	1.27	metabolic process; regulation of biological process	endoplasmic reticulum; nucleus; organelle lumen	protein binding
22	P01111	NRAS	GTPase NRas	1.27	cellular component movement; metabolic process; regulation of biological process; response to stimulus; transport	Golgi; membrane	catalytic activity; nucleotide binding; protein binding
23	Q8TDW7	FAT3	Protocadherin Fat 3	1.27	development	membrane	metal ion binding; protein binding

24	Q86VU5	COMTD1	Catechol O-methyl transferase domain-containing protein 1	1.28	metabolic process	membrane; mitochondrion	catalytic activity; protein binding
25	P56377	AP1S2	AP-1 complex subunit sigma-2	1.29	regulation of biological process; transport	cytosol; Golgi; membrane	protein binding; transporter activity
26	Q8NEY8-3	PPHLN1	Isoform 3 of Periphilin-1	1.30	metabolic process; regulation of biological process	chromosome; cytosol; Golgi	protein binding; RNA binding
27	Q8WWC4	C2orf47; MAIP1	m-AAA protease-interacting protein 1, mitochondrial	1.30	cell organization and biogenesis; cellular homeostasis; transport	membrane; mitochondrion; organelle lumen	protein binding
28	P07919	UQCRH	Cytochrome b-c1 complex subunit 6, mitochondrial	1.32	metabolic process; transport	membrane; mitochondrion	catalytic activity; protein binding; transporter activity
29	Q9NX40	OCIAD1	OCIA domain-containing protein 1	1.33	regulation of biological process	endosome; membrane; mitochondrion	protein binding
30	Q96AY3	FKBP10	Peptidyl-prolyl cis-trans isomerase FKBP10	1.35	cell organization and biogenesis; metabolic process	cytoplasm; endoplasmic reticulum; organelle lumen	catalytic activity; metal ion binding
31	Q9HD33-1	MRPL47	39S ribosomal protein L47, mitochondrial	1.38	cell organization and biogenesis; metabolic process	membrane; mitochondrion; ribosome	structural molecule activity

32	P50213-1	IDH3A	Isocitrate dehydrogenase [8] subunit alpha, mitochondrial	1.41	metabolic process	mitochondrion; nucleus; organelle lumen	catalytic activity; metal ion binding; nucleotide binding
33	Q8NHP8	PLBD2	Putative phospholipase B-like 2	1.41	metabolic process	organelle lumen; vacuole	catalytic activity
34	P14314	PRKCSH	Glucosidase 2 subunit beta	1.43	metabolic process; regulation of biological process; response to stimulus	endoplasmic reticulum; organelle lumen	metal ion binding; protein binding
35	P02788	LTF	Lactotransferrin	1.45	cellular homeostasis; defense response; metabolic process; regulation of biological process; response to stimulus; transport	cell surface; cytoplasm; extracellular; nucleus; organelle lumen	catalytic activity; DNA binding; enzyme regulator activity; metal ion binding; protein binding
36	Q9HCN8	SDF2L1	Stromal cell-derived factor 2-like protein 1	1.49	metabolic process; regulation of biological process; response to stimulus	endoplasmic reticulum; membrane; organelle lumen	catalytic activity; protein binding
37	P16403	HIST1H1C	Histone H1.2	1.58	cell organization and biogenesis; metabolic process; regulation of biological process	chromosome; nucleus	DNA binding; protein binding; RNA binding
38	Q96A26	FAM162A	Protein FAM162A	1.66	cell death; metabolic process; regulation of biological process; response to stimulus	cytosol; membrane; mitochondrion	protein binding

39	P23142-4	FBLN1	Isoform C of Fibulin-1	1.68	cell organization and biogenesis; metabolic process; regulation of biological process; response to stimulus	extracellular	enzyme regulator activity; metal ion binding; protein binding; structural molecule activity
40	P21912	SDHB	Succinate dehydrogenase [ubiquinone] iron-sulfur subunit, mitochondrial	1.68	metabolic process; transport	membrane; mitochondrion	catalytic activity; metal ion binding; protein binding
41	P69905	HBA2; HBA1	Hemoglobin subunit alpha	2.04	cell organization and biogenesis; metabolic process; regulation of biological process; response to stimulus; transport	cytosol; extracellular; membrane; organelle lumen	antioxidant activity; catalytic activity; metal ion binding; protein binding; transporter activity
42	P02765	AHSG	Alpha-2-HS-glycoprotein	2.51	defense response; metabolic process; regulation of biological process; response to stimulus; transport	extracellular; organelle lumen; Golgi;	enzyme regulator activity
43	P78562	PHEX	Phosphate-regulating neutral endopeptidase PHEX	3.70	cell communication; metabolic process; response to stimulus	endoplasmic reticulum; Golgi; membrane	catalytic activity; metal ion binding

Table S3. Bioinformatics information and KEGG/Wiki/Reactome pathways of DNA damage repair proteins and DNA damage response proteins with a fold change (FC) ≤ -1.20 or of ≥ 1.20 identified in A549 cells exposed to ZQX-1 (0.5 μM) under irradiation at 420 nm. * NA: not available.

Accession	Gene Symbol	Protein name	FC (LZ vs. DZ)	KEGG pathways	Wiki pathways	Reactome pathways
P62877	RBX1	E3 ubiquitin-protein ligase	-1.33	nucleotide excision repair; HIF-1 signaling pathway; Ubiquitin mediated proteolysis; cell cycle; TGF-beta signaling pathway	TGF-beta signaling pathway;	DNA damage recognition in GG-NER; formation of incision complex in GG-NER; dual incision in GG-NER; formation of TC-NER pre-incision complex; Dual incision in TC-NER; Gap-filling DNA repair synthesis and ligation in TC-NER; recognition of DNA damage by PCNA-containing replication complex; NOTCH1 intracellular domain regulates transcription; Oxygen-dependent proline hydroxylation of Hypoxia-inducible factor Alpha; constitutive signaling by NOTCH1 HD+PEST domain mutants; Ubiquitination & Proteasome degradation; Neddylation;
Q13098	GPS1	COP9 signalosome complex subunit 1	-1.26	NA*	NA*	DNA Damage Recognition in GG-NER; formation of TC-NER Pre-Incision Complex; Neddylation; Cargo recognition for clathrin-mediated endocytosis
P63165-1	SUMO1	Small ubiquitin-related modifier 1	1.20	RNA transport	TP53 Network; TGF-beta Signaling Pathway;	SUMOylation of DNA damage response and repair proteins; Recruitment and ATM-mediated phosphorylation of repair and signaling proteins at DNA double strand breaks; Processing of DNA double-strand break ends G2/M DNA damage checkpoint; SUMOylation of DNA replication proteins; Nonhomologous End-Joining (NHEJ); Formation of Incision Complex in GG-NER;
P01111	NRAS	GTPase NRas	1.27	Apoptosis; Non-small cell lung cancer; Ras signaling pathway; PI3K-Akt signaling pathway;	DNA Damage Response (only ATM dependent);	Regulation of RAS by GAPs; SHC-mediated cascade:FGFR1/3/4; Signaling by high-kinase activity BRAF mutants; VEGFR2 mediated cell proliferation; RAF activation; Negative regulation of MAPK pathway; CERI mediated MAPK activation; Downstream signal transduction; RAS signaling downstream of NF1 loss-of-function variants; RAS GTPase and MAP kinases;
P09874	PARP1	Poly [ADP-ribose] polymerase 1	1.27	Apoptosis; Base excision repair; NF-kappa B signaling pathway;	NAD metabolism; NAD+ biosynthetic pathways; Nanoparticle triggered regulated necrosis;	POLB-Dependent Long Patch Base Excision Repair; Dual Incision in GG-NER; Formation of Incision Complex in GG-NER; Downregulation of SMAD2/3:SMAD4 transcriptional activity; DNA Damage Recognition in GG-NER; HDR through MMEJ (alt-NHEJ); SUMOylation of DNA damage response and repair proteins

Table S4. Proteins associated with photodynamic therapy-induced signaling pathways.

Accession	Gene Symbol	Protein name	FC (LZ vs. DZ)	Biological process	Cellular component	Molecular function	KEGG pathways	Wiki pathways
**P00338-1**	LDHA	L-lactate dehydrogenase A chain	-1.20	metabolic process; regulation of biological process	cytoplasm; nucleus	protein binding; RNA binding	HIF-1 signaling pathway; metabolic pathways; pyruvate metabolism; glycolysis / gluconeogenesis; central carbon metabolism in cancer; cysteine and methionine metabolism; glucagon signaling pathway;	photodynamic therapy-induced HIF-1 survival signaling; glycolysis and gluconeogenesis; Cori cycle; amino acid metabolism;
**P19367-1**	HK1	Hexokinase-1	1.23	cellular homeostasis; metabolic process; regulation of biological process	cytosol; membrane; mitochondrion	catalytic activity; nucleotide binding; protein binding	HIF-1 signaling pathway; carbon metabolism; glycolysis / gluconeogenesis; fructose and mannose metabolism; galactose metabolism; amino sugar and nucleotide sugar metabolism; central carbon metabolism in cancer;	photodynamic therapy-induced HIF-1 survival signaling; Cori cycle; glycolysis and gluconeogenesis; glucuronidation
**Q9UBS4**	DNAJB 11	DNA J homolog subfamily B member 11	1.27	metabolic process; regulation of biological process	cytoplasm; endoplasmic reticulum; nucleus; organelle lumen	protein binding	protein processing in endoplasmic reticulum	photodynamic therapy-induced unfolded protein response

Table S5. The enriched protein-protein (PPI) networks of the DEPs with |FC| ⩾ 1.2 identified in A549 cells exposed to ZQX-1 under irradiation. The protein names in bold represent upregulation (↑) or downregulation (↓).

No.	Molecules in the network	Top associated diseases and functions of Network
1	ACTN1, ADAM17, ARHGEF5, BCR, CXCL8, DDX10, DSG1, DSP, **EEF1A1** ↓, EGF, EGFR, **FKBP10** ↑, **GPS1** ↓, GRB2, HBB, HK2, HSPA8, HSPD1, IGF1R, JUP, MVP, NFKB1, NFKBIA, PKM, PRKCD, **PTPN11** ↓, RAD50, RELA, **RPS3** ↓, S100A9, **SDHB** ↑, SHC1, STK11, **TRAF2** ↓, UBA52	Cell Death and Survival, Cellular Development, Organismal Injury and Abnormalities
2	TP53BP1, **UBE2L3** ↓	Hereditary Disorder, Organismal Injury and Abnormalities
3	CDKN1A, **CNOT3** ↑	Cell Morphology, Connective Tissue Development and Function
4	**AP1S2** ↑, SMARCA4	Cancer, Cell Cycle
5	**ATP5ME** ↑, SREBF1	Dermatological Diseases and Conditions, Developmental Disorder
6	Let-7, **NRAS** ↑, RHOB	Cancer, Hematological disease, immunological disease
7	BMI1, FOXM1, GLI1, **RACK1** ↓, SMO, PTCH1	Cancer, Organismal Injury and Abnormalities
